# A Comprehensive Pan-cancer Analysis of the Biological Immunomodulatory Function and Clinical Value of *CD27*

**DOI:** 10.7150/jca.85446

**Published:** 2024-01-01

**Authors:** Yongfeng Wang, Ling Guan, Yanzong Zhao, Yanling Yang, Yitong Wang, Shengjiao Feng, Anqi Zou, Yawei Li, Botao Zhou, Dongzhi Zhang, Weiqi Che, Fangyu Liu

**Affiliations:** 1 The First Clinical Medical College of Lanzhou University, Lanzhou, Gansu, 730000, China.; 2 General Surgery Clinical Medical Center, Gansu Provincial Hospital, Lanzhou, Gansu, 730000, China.; 3 Key Laboratory of Molecular Diagnostics and Precision Medicine for Surgical Oncology in Gansu Province, Gansu Provincial Hospital, Gansu 730000, China.; 4 NHC Key Laboratory of Diagnosis and Therapy of Gastrointestinal Tumor, Gansu Provincial Hospital, Lanzhou, 730000, China.; 5 School of Stomatology, Lanzhou University, Lanzhou, Gansu, 730000, China.; 6 College of Health Science and Engineering, University of Shanghai for Science and Technology, Shanghai 200093, China.

**Keywords:** *CD27*, pan-cancer, immune, prognosis.

## Abstract

**Background:** *CD27* is an immunological checkpoint gene, plays a critical function inInhibition or activation of cancer immunity. The *CD27*/*CD27L* axis is its pathway of action. Therefore, our goal was to examine the predictive role of *CD27* in the clinical prognosis of 33 cancer types and its functions in cancer progression, as well as explore the link between pan-cancer *CD27* gene expression and immune infiltration.

**Methods:** By comprehensive use of datasets and methods from TCGA, cBioPortal, GTEx, HPA, KM-plotter, Spearman, CellMinerTM, R packages and RT-qPCR, we delved deeper into the potential impact of the *CD27* on cancer development. These include expression differences, immune infiltration, matrix infiltration, gene mutations, DNA methylation, signaling pathways, TMB, MSI, and prognosis. Also, we explored* CD27* interactions with different drugs.

**Results:** The results showed that, mutated *CD27* was highly expressed in most cancers. The *CD27* showed strong diagnostic value in 4 cancers and marked a positive prognosis for CESC, intracervical adenocarcinoma, HNSC, and endometrial cancer, and a poor prognosis for UVM. In addition, *CD27* affects multiple immune and inflammatory signaling pathways and is positively correlated with immune cell infiltration, T cell differentiation, macrophage M1 polarization, stromal infiltration, and drug sensitivity. DNA methylation is involved in *CD27* expression in cancer.

**Conclusion:** *CD27,* which is mutated in cancers and appears widely highly expressed and altered tumor immune invasion and stromal invasion by affecting multiple immune-related and inflammation signaling pathways, plays a significant role in CESC, HNSC, UCEC and UVM, and may be used as a therapeutic target for related cancers.

## Introduction

Cancer has had a significant negative impact on worldwide public health, and its morbidity and death rate have quickly jeopardized human health, and most of the treatments that are currently available have a poor level of efficacy 1-3. Studies of cancer can assist us find distinctive aspects of malignant tumors, as well as provide brand new ideas for the treatment of human cancers 4. Nowadays, the adhibition of biomarkers in cancer has aroused lots of attention, this suggests that the study of new biological markers of cancer is necessary 5-7. In order to do this, pan-cancer analysis can be adopted to locate useful prognostic biomarkers. Establishing more efficient molecular targets for cancer therapy and discovering new diagnostic and predictive markers 8-11.

The *CD27*, as known as *TNFRSF7*^12^. It is essentially a type-I transmembrane glycoprotein that binds to its ligand *CD27L* (*CD70*) and initiates a series of signal transduction pathways to regulate cellular function 13, 14. *CD27* is widely expressed in immune cells 15, and it performs a lot of biological functions. What is most noteworthy is that as a co-stimulated T, B cell receptors initiate functional immune responses and promote the proliferation and differentiation of T cells 16.

The autoimmune system is considered to be the most important means of fighting tumors, directly killing and removing tumor cells. Tumor-specific immunity relies on CD8^+^T cell-mediated cellular immunity 17-19. The activation of T cell is a complex biological process that relies on the first signal provided by the TCR with the MHCI and the second signal provided by the T cell co-receptor 20, 21. Deficiency of stimulatory T cell co-receptors or activation of inhibitory co-receptors is common in the TME and is one of the pathways by which tumors initiate immune escape 22, 23. For example, activation of the* PD-1/PD-L1* axis inhibits tumor-specific immunity and causes adverse outcomes 24.

**Figure 1:**
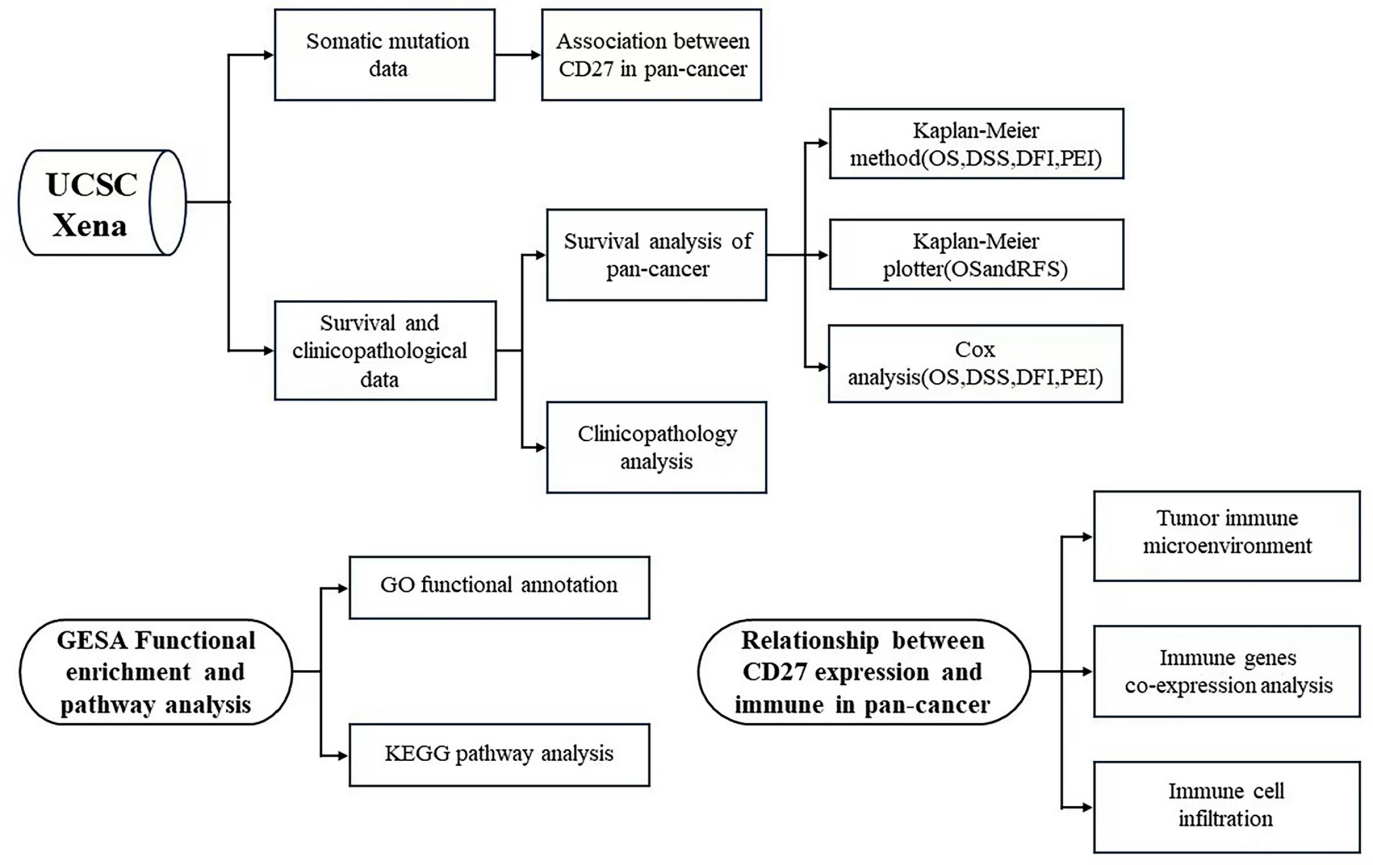
The flow chart of the study design and analysis.

As a T cell co-receptor that has not been fully studied, *CD27* has a great possibility to play a regulatory and therapeutic role in tumor progression. In this work, we studied the prognostic relevance of the expression of *CD27* in 33 different cancer types. Further, we investigated if there was a correlation between *CD27's* expression, TMB, MSI, and the levels of immune infiltration. We also analyzed the co-expression of *CD27* and the correct route with other immune-related genes. Based on these findings, it appears that *CD27* may influence cancer patients' prognoses by interacting with invading immune cells. We used a flow chart to show the design and analysis of this study in Figure 1.

## Methods

### Sample Information

The expression data of *CD27*, survival data and clinicopathological of the 33 cancers were obtained from the TCGA database. We included normal samples and cancer tissue samples from the TCGA and GTEx databases to compare their *CD27* expression levels. Besides, the HPA was utilized to present the human's protein expression models in both normal and tumor tissues 25.

### Expression Profiling of *CD27*

*CD27* is multi-expressed in lymphocytes and APCs and *CD27L* binds to *CD27* expressed on T cells to promote T cell signal transduction. The TCGA and GTEx databases and Perl software were used to analyze *CD27* expression in 33 cancers.

### Analyses of *CD27* Expression Levels in Human Cancer for Prognosis and Clinicopathological Association

To further investigate the link between *CD27* expression and clinical outcome, we gathered the survival data from the TCGA database. All the important metrics were calculated. And we evaluated the survival analysis using the Kaplan-Meier method and log-rank test.

For the human cancer dichotomy, the cut-off value was determined by using the median *CD27* expression level as the basis. This help classify patients into high-risk and low-risk categories. Besides, we conducted a COX analysis in order to evaluate the relationship between *CD27* expression and the prognosis of pan-cancer.

We used KM-plotter to investigate the link between *CD27* expression and the survival of pan-cancer.

### Indicators of gene mutation and DNA Methyltransferase Analysis

TMB is an indicator of cancer gene mutations and is associated with the effectiveness of immune checkpoint therapy. TMB can be assessed by MSI and MMR 26, 27. The TCGA database was used to determine the mutation levels of 5 MMR genes (*EPCAM*, *MLH1*, *MSH6*, *MSH2*, and *PMS2*). DNMTs also have a significant impact on how chromatin structure and gene expression are altered. This study employed Spearman's Pearson analysis to evaluate the connection between *CD27* expression and 5 MMR genes, as well as the 4 methyltransferases (*DNMT3B*, *DNMT3A*, *DNMT2*, and *DNMT1*) by using the R-packages “reshape2” and “RColorBrewer”.

### Correlations Between *CD27* Expression and Immune

The TIMER and CIBERSORT databases were used to obtain immune invasion data in 33 cancers, the scores of 6 kinds of TIICs (CD4^+^T cells, CD8^+^T cells, macrophages, B cells, dendritic cells, and neutrophils) in 33 cancers were obtained. Additionally, using Spearman correlation analyses, we assessed the associations between *CD27* expression and TIICs, immunological checkpoint marker expression levels as well as immune/stromal scores. An estimation algorithm in R-package “estimation” and “limma” was applied to assess the matrix score and immune score of stromal cells and immune cells (*P* < 0.001 as a cut-off value). And the co-expression analysis of *CD27* with other immune-related genes was conducted to further investigate the relations between *CD27* expression and immune.

### Pathway Analysis of *CD27*

Specifically, the study used gene sets downloaded from the GSEA website. GO and KEGG were implemented for *CD27* annotation by R-package"org.Hs.eg.db", "clusterProfiler", and "enrichplot".

### Drug Sensitivity of *CD27*

To analyze *CD27* chemosensitivity in tumors, we applied CallMiner-TM to download NCI-60 compound activity data and RNA-seq expression files. Some drugs which have been approved by FDA were chosen for analysis.

### Cell Culture

Human hepatocyte line L-O2, human hepatoma cell line HUH-7 HepG2 and SMMC-7721; Human colonic epithelial cell line NCM460, human colorectal cancer cell line SW480, HCT116 and RKO; Human renal proximal convoluted tubular epithelial cell line HK-2, human renal cancer cell line Caki-2; Human gastric mucosal cell line GES-1, human gastric cancer cell line AGS, MKN-45 and MGC-803. These cell lines were from our research group and preserved in Gansu Provincial People's Hospital Central Laboratory. They were all incubated in RPMI-1640 supplemented with 10 % FBS (Hy clone) plus 1% antibiotics (100 U/mL penicillin and 100 µg/mL streptomycin) (MA0110, MEILUNE, China), and maintained at 37℃ in a 5% CO_2_ incubator (HF90-HT, Heal Force, China).

### RNA isolation and Quantitative Real-time Polymerase Chain Reaction (qRT-PCR)

Follow the reagent provider's instructions for total RNA extraction, cDNA transcription, and RT-qPCR using the RNA Generic Kit, RNA Reverse Transcription Kit. *CD27* primers were designed by website Primer, and were synthesized by Bioengineering (Shanghai) Co. The primer sequences are as follows, forward: (5'-3'): TGCAGAGCCTTGTCGTTACAG, reverse (5'-3'): GCTCCGGTTTTCGGTAATCCT.

### Statistical analysis

Standard tests include the student t-test, the Kluscal-Walli's test, the Wilcoxon rank sum test, and the chi-square test. The Wilcox test could be used to examine the gene expression data. In survival analysis, univariate Cox regression analysis was used to get the HRs and *P* values. Using Spearman correlation analysis to assess the relationship between *CD27* expression and immune scores. The R package is used for all calculations. *P* <0.05 was judged significant.

## Results

### mRNA and protein expression of *CD27* in pan-cancer

**Figure 2:**
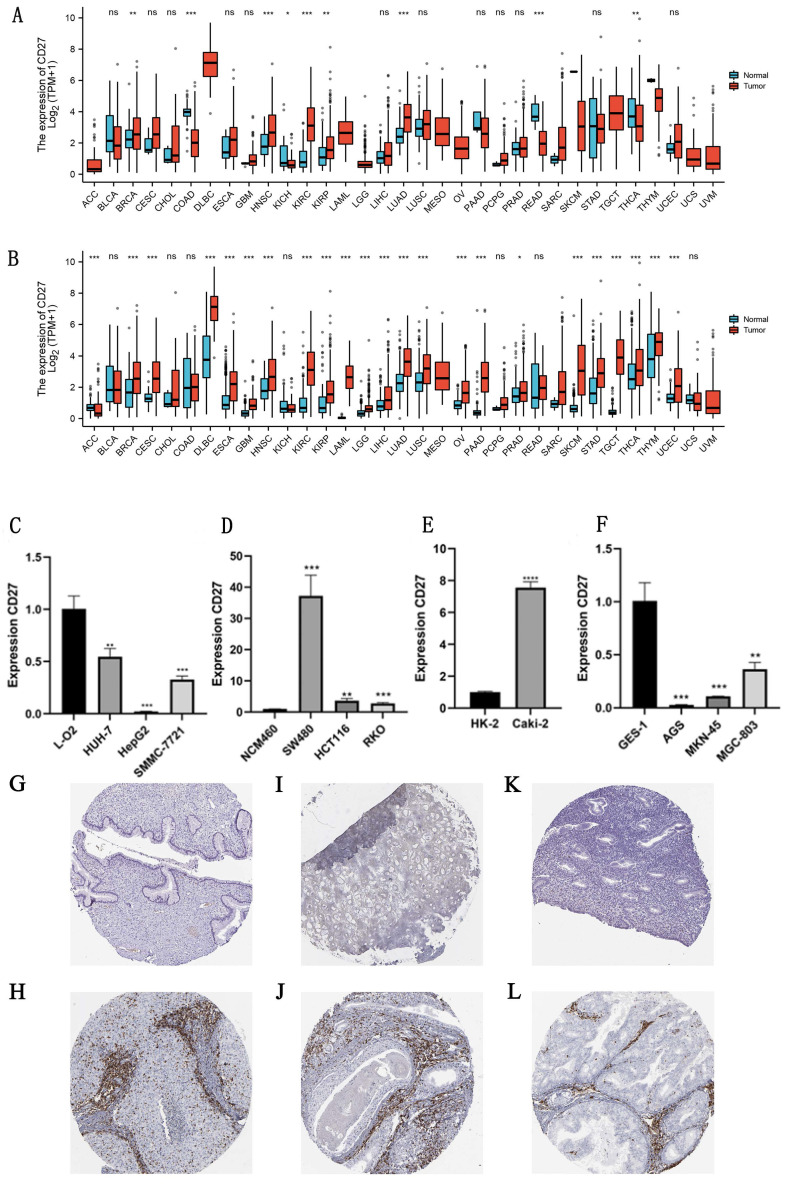
** mRNA and protein expression of *CD27.*** (A) *CD27* expression data from TCGA. (B*) CD27* expression data from TCGA and GTEx. (C) mRNA expression of *CD27* in liver cell lines. (D) mRNA expression of *CD27* in colon cell lines. (E) mRNA expression of *CD27* in kidney cell lines. (F) mRNA expression of *CD27* in gastric cell lines. (G) Immunohistochemical of normal cervix uterus. (H) Immunohistochemical of CESC. (I) Immunohistochemical of normal nasopharynx epithelial tissue. (J) Immunohistochemical of HNSC. (K) Immunohistochemical of normal endometrium. (L) Immunohistochemical of UCEC. *P<0.05, ** *P*<0.01, and *** *P*<0.001.

The TCGA and GTEx databases were used to analyze differences of *CD27* expression in 33 cancers, the data from these two databases formed a mutually validated comparison. And the absolute expression levels between normal and cancer tissues in TCGA, and TCGA combined with GTEx were displayed separately in Table S1 and Table S2. And the detailed information of the sample was summarized in Table S3. The results showed that *CD27* have a significant high expression profile in the vast majority of cancer samples. Such as GBM, HNSC, KIRC, KIRP, BRCA, CESC, DLBC, ESCA, PRAD, SKCM, STAD, TGCT, THYM, LAML, LGG, LIHC, LUSC, LUAD, OV, PAAD, and UCEC. THCA was excluded due to inconsistent expression in both databases. Uniquely, *CD27* was significantly low expressed in ACC (Figure 2A, 2B).

Furthermore, we experimentally verified the difference in the mRNA expression of *CD27* in some cancer cell lines and normal cell lines. Which showed that *CD27* expression was significantly reduced in human liver cancer cell line (HUH-7, HepG2, and SMMC-7721) compared to human normal liver cells line (L-O2) (Figure 2C). Compared with normal colon epithelial cells (NCM460), *CD27* was highly expressed in three colon cancer cells lines, SW480, HCT116 and RKO (Figure 2D). And the expression of *CD27* in the human renal clear cell cancer cell line (Caki-2) was significantly increased compared with the Human Kidney-2 cell line (HK-2) (Figure 2E). Compared with normal human stomach epithelial cells, *CD27* was highly expressed in three stomach cancer cells lines, AGS, MGC-803, and MKN-45 (Figure 2F).

The results of immunohistochemistry showed that *CD27* was highly expressed in most cancers compared to normal tissues, which was consistent with mRNA expression profiles in TCGA and GTEx databases. Such as CESC, HNSC and UCEC (Figure 2 G-L). In addition, in the mRNA expression profile, we found a significantly higher expression of *CD27* in DLBC that is different from other cancers. This suggests that *CD27* may serve as a marker for DLBC.

### Multifaceted Prognostic Value of *CD27*

**Figure 3:**
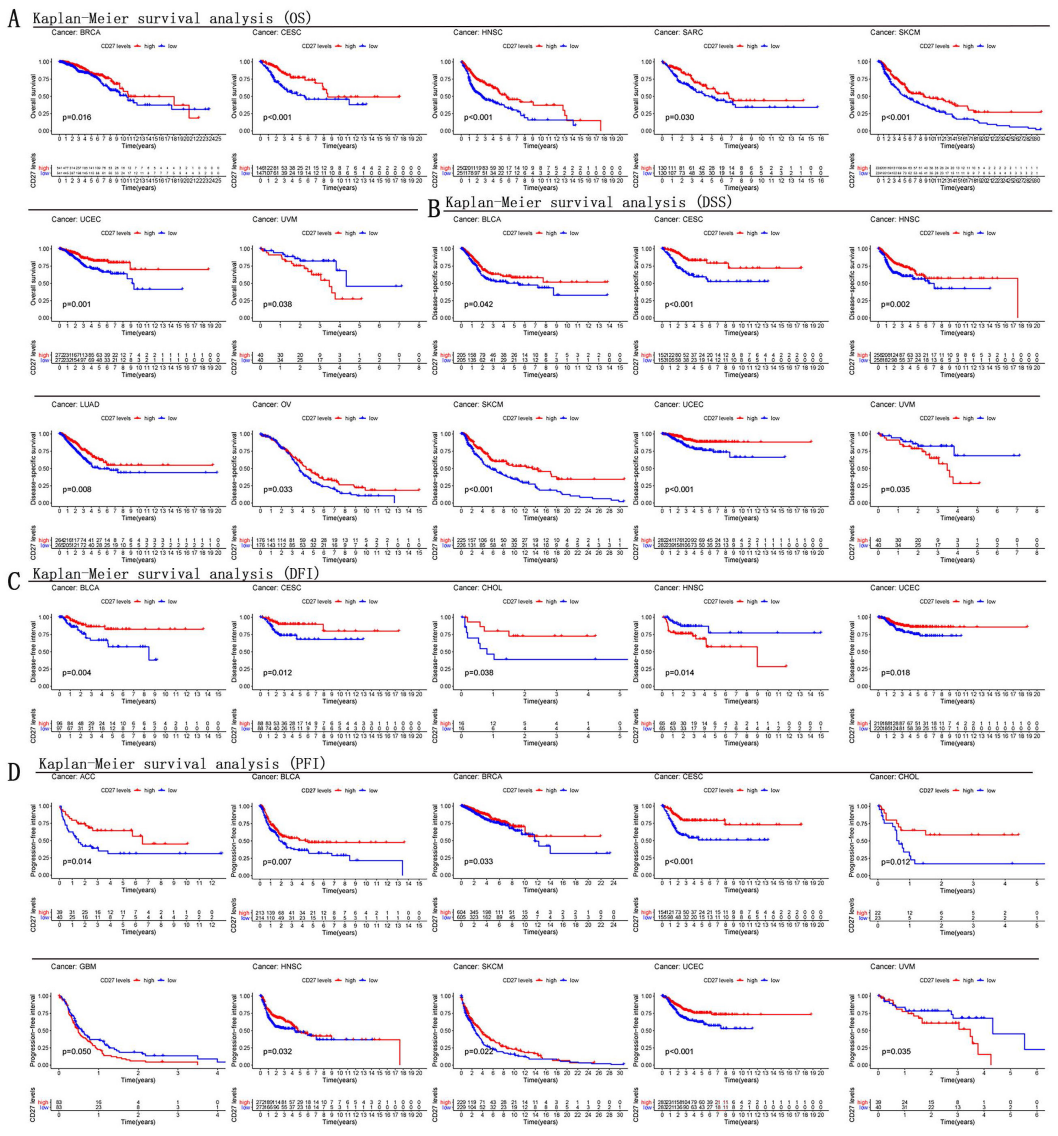
** Kaplan-Meier survival curves comparison of high and low expression of *CD27* gene for different cancer types.** (A) OS of BRCA, CESC, HNSC, SARC, SKCM, UCEC, UVM. (B) DSS of BLCA, CESC, HNSC, LUAD, OV, SKCM, UCEC, UVM. (C) DFI of BLCA, CESC, CHOL, HNSC, UCEC. (D) PFI of ACC, BLCA, BRCA, CESC, CHOL, GBM, HNSC, SKCM, UCEC, UVM.

The predictive significance of *CD27* for pan-cancer was investigated utilizing several databases. Notably, *CD27* expression was linked with prognosis in a several of Kaplan-Meier cumulative curves for cancers in the TCGA database. *CD27* was found to play a aggressive role against the prognosis of eleven distinct cancers, which included CESC, HNSC, SKCM, BLCA, CHOL, OV and ACC. Patients with increased *CD27* expression fared better than those with low *CD27* expression in this study. Contrarily, *CD27* played a detrimental role in other cancers, including UVM and GBM, in which patients whose *CD27* expression was high had a lower chance of survival than those whose *CD27* mRNA levels were low (Figure 3A-D).

**Figure 4:**
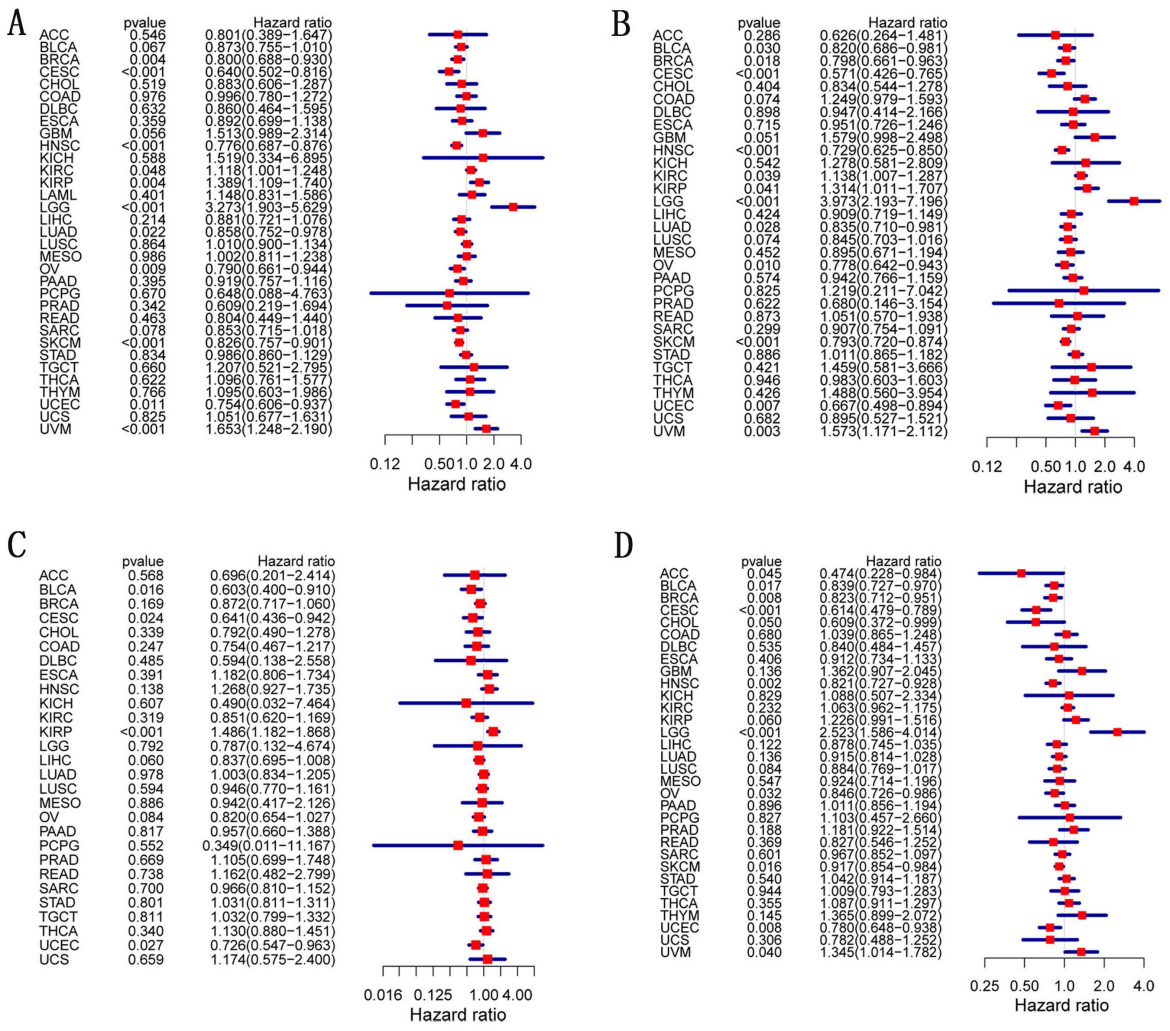
** Correlation of *CD27* mRNA expression with survival in TCGA.** (A) OS. (B) DSS. (C) DFI. (D) PFI.

We analyzed OS, DSS, DFI, and PFI in pan-cancer to determine the roles *CD27* played in the prognosis, furtherly. Using the cox proportional hazards model, the results showed that *CD27* expression levels were linked with OS in BRCA, CESC, HNSC, OV, SKCM, UCEC, KIRP, LGG, and UVM. Further, *CD27* was a high-risk gene in LGG, KIRP, and UVM (Figure 4A). The analysis of DSS revealed associations between low *CD27* expression and poor prognosis in BLCA, BRCA, CESC, LUAD, HNSC, OV, SKCM, and UCEC. however, in patients with KIRC, KIRP, LGG and LIHC, *CD27* expression displayed the inverse connection that one would expect with prognosis (Figure 4B). In addition, an examination of the DFI data (Figure 4C) uncovered links between low *CD27* expression and a poor prognosis in patients who had BLCA and CESC. However, in patients with KIRP, *CD27* expression displayed the inverse connection that one would expect with prognosis (Figure 4C). As for PFI and *CD27* expression, findings from forest plots demonstrated relationships between high expression and poor PFI in LGG and UVM (Figure 4D).

### Correlation of *CD27* Expression and Clinical pathology

**Figure 5:**
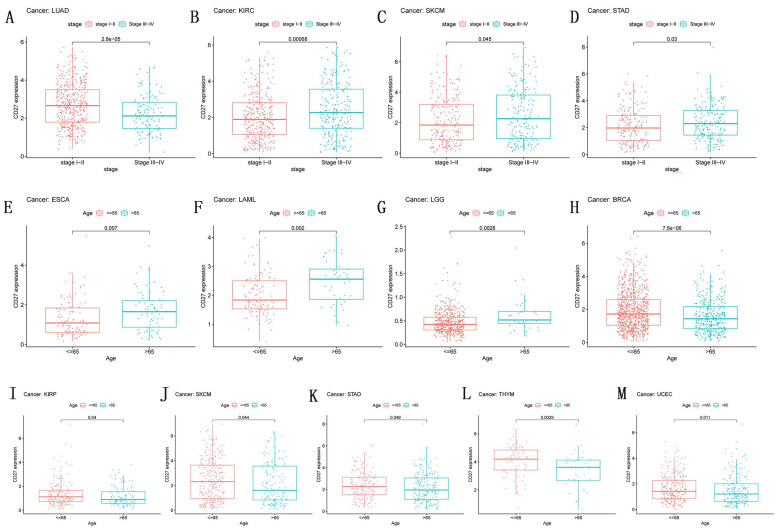
** Correlation of *CD27* Expression and Clinical pathology.** *CD27* expression related with the stage in LUAD (A), KIRC (B), SKCM (C), and STAD (D).*CD27* expression associated with age in ESCA (E), LAML (F), LGG (G), BRCA (H), KIRP (I), SKCM (J), STAD (K), THYM (L) and UCEC (M).

**Figure 6:**
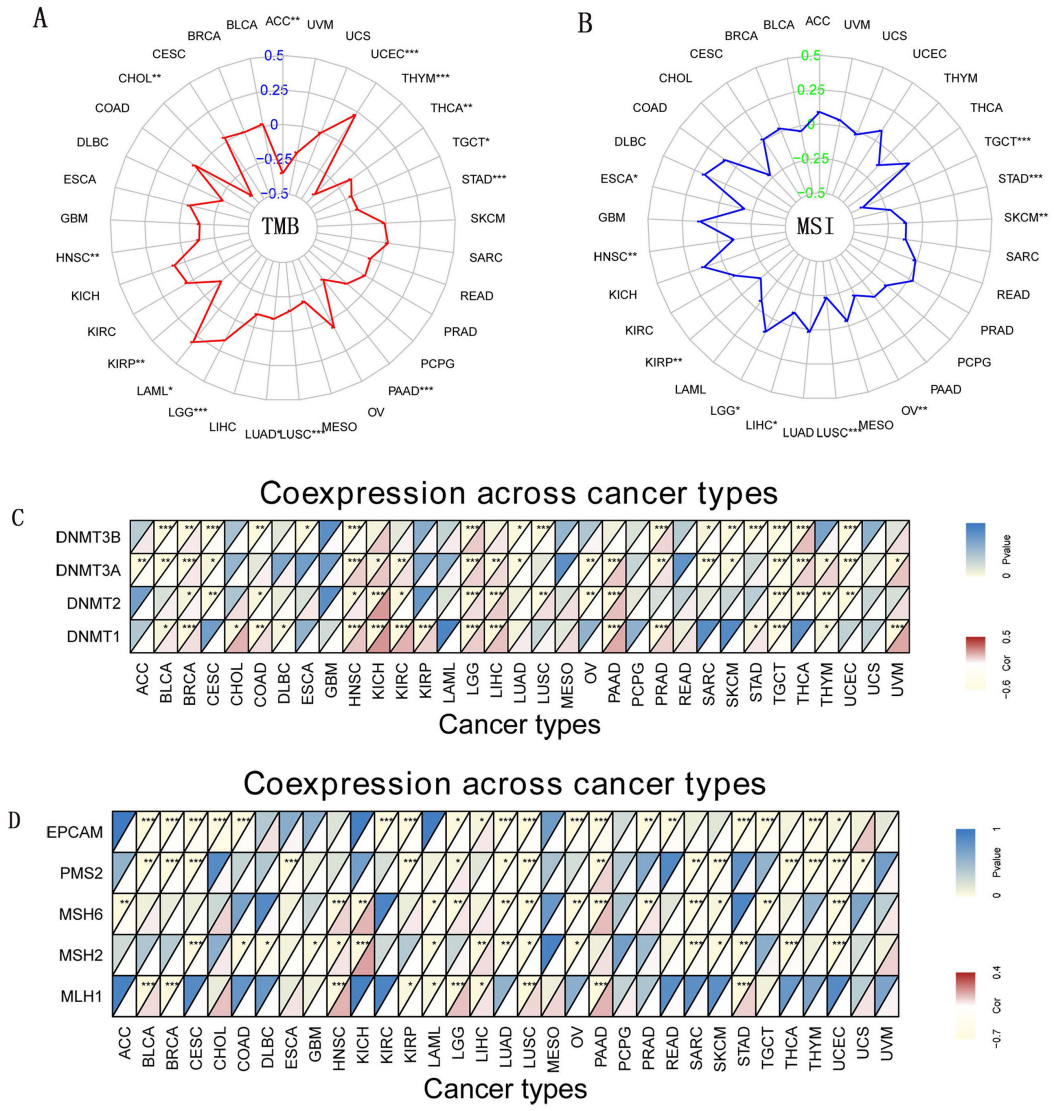
** Indicators of gene mutation and DNA Methyltransferase.** (A) The radar chart of the association between TMB and *CD27* gene expression. (B) The radar chart of the relationship between MSI and *CD27* gene expression (C) DNA methyltransferase. (D) Immune genes. *P < 0.05, **P < 0.01, and ***P < 0.001.

Numerous clinicopathological features of malignancies have been connected to *CD27* expression (Figure 5). Next, we looked at how age affected the expression of *CD27* in individuals with each type of tumor. We discovered that people under the age of 65 with BRCA, KIRP, SKCM, STAD, THYM, and UCEC (Figure 6H-M) had higher expression levels, while patients after the age of 65 had higher *CD27* expression levels with ESCA, LAML, and LGG (Figure 5E-G). Moreover, *CD27* was strongly expressed in LUAD patients at stages I and II, but in later stages, its expression was attenuated. Expression of *CD27* was greatest in patients with stages III and IV of KIRC, SKCM, and STAD (Figure 5B-D), and least in those with stages I and II.

### Indicators of gene mutation and DNA Methyltransferase with *CD27* Expression

TMB and MSI are newly discovered biomarkers linked to immune checkpoint inhibitor sensitivity 26, 27. Therefore, it is necessary to look at how TMB or MSI and *CD27* expression are related to various cancer types. Our data showed TMB was favorably linked with *CD27* expression in three different cancer types, including LAML, LGG, and UCEC. In contrast, in eleven additional cancer types, *CD27* expression was inversely related to TMB, which included ACC, CHOL, HNSC, KIRP, LUSC, LUAD, PAAD, STAD, TGCT, THCA, and THYM (Figure 6A). We further examined the possibility of an association between *CD27* expression and MSI and we found that MSI was substantially linked with *CD27* expression in 10 different kinds of cancer. *CD27's* expression was correlated favorably with MSI in two subtypes of LGG cancer. *CD27* expression was positively correlated with MSI in two cancers, LGG, but was negatively correlated with MSI in the other nine cancers, including ESCA, HNSC, KIRP, LUSC, LIHC, OV, SKCM, STAD, and TGCT (Figure 6B). At least one MMR-related gene was shown to be linked with *CD27* expression in 27 of the 33 cancer types studied (Figure 6C). In most malignant tumors, *CD27* expression was associated with *MLH1* expression (Figure 6D).

### Correlation of TME and *CD27* Expression

**Figure 7:**
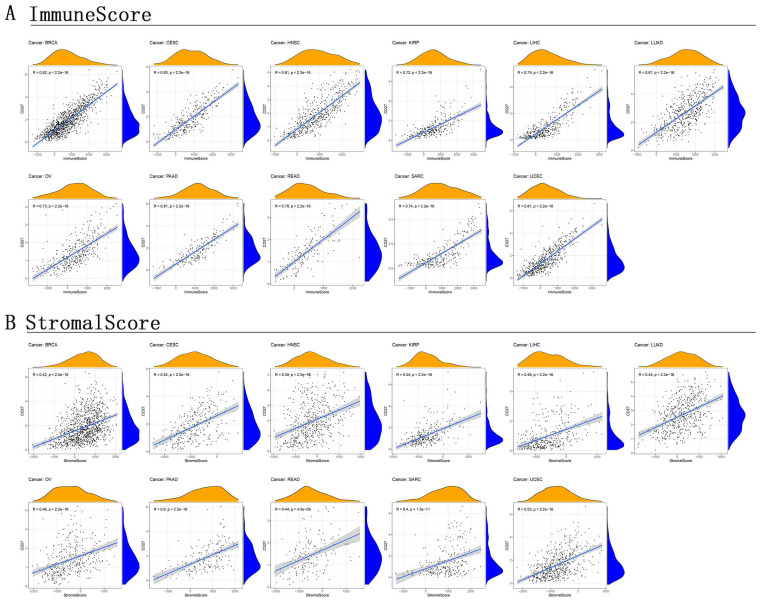
** Correlation of CD27 expression with stromal score and immune score.** (A) Immune score. (B) Stromal score.

TME is a microenvironment that contains not only tumor cells, immune cells, stromal cells, and other types are all present in TME 28, 29. It is crucial in promoting cancer cell heterogeneity, which raises treatment resistance and promotes the spread of cancer cells. Further investigation into the link between TME and *CD27* expression in various cancer types would be extremely appropriate given that our findings have verified the predictive significance of *CD27* in pan-cancer. This evaluation would be particularly important for BRCA, CSEC, KIRP, LUAD, LIHC, OV, PAAD, READ, HNSC, SARC, and UCEC. This allowed us to generate the stromal score and the immune score. In these cancers, *CD27* expression was positively correlated with immune (Figure 7A) and stromal (Figure 7B) scores, and this correlation is statistically significant (Figure 7).

### Immune correlations

**Figure 8:**
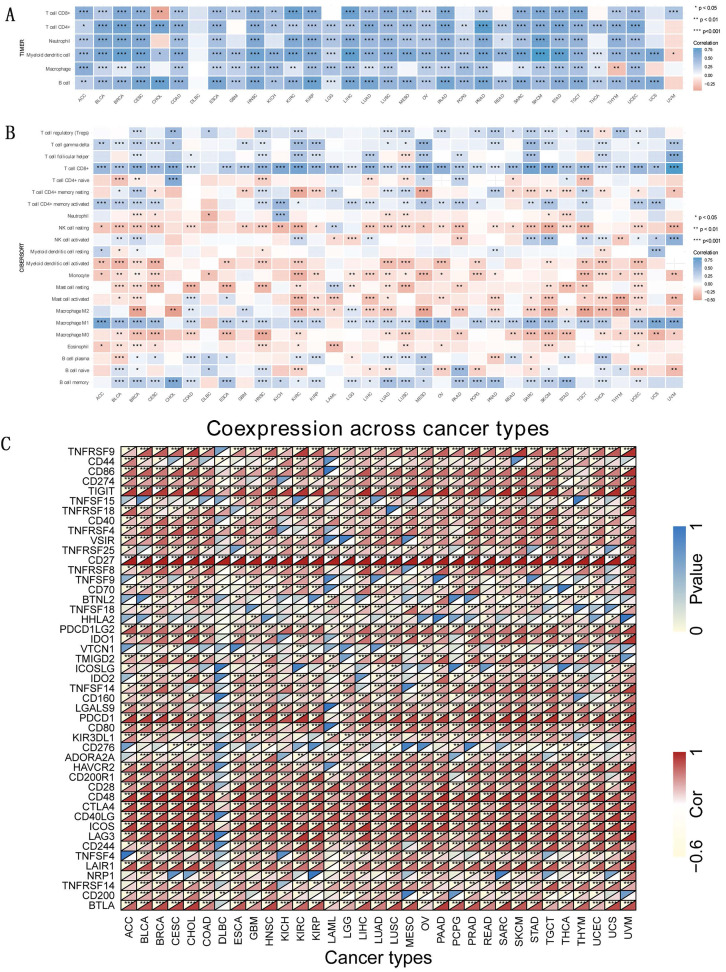
** Immune correlation.** TIICs analysis and correlation analysis of *CD27* expression with immune checkpoints in tumors. (A) *CD27* expression and immune-associated cell infiltration in the TIMER database. (B) *CD27* expression and immune-associated cell infiltration using the CIBERSORT algorithm. (C) The heatmap of the association between *CD27* and immunosuppressive genes in TCGA.

TIICs and their quantity correlated with *CD27* expression. As the parameter option for graphing, we went with the “log2” transformation of the expression data (Figure 8A). There are 22 classes of immune infiltrating cells (Figure 8B). To gain a deeper understanding of the underlying mechanism behind the immune suppression of *CD27* signaling, we investigated the association between *CD27* expression and a variety of immunological checkpoint markers in 33 different cancer types (Figure 8C). CD8^+^T cells have been shown to be positively correlated with *CD27* expression in most tumors. In addition, the high expression of *CD27* also had a significant effect on macrophage polarization, mainly in inhibiting macrophage M2 polarization and promoting its M1 polarization. This promotes the presentation of tumor-associated antigens and immune activation. This suggests that the upregulation of *CD27* can promote tumor-specific cellular immunity. The positive association between *CD27* and *TIGIT*, *CD48* in the majority of cancers suggested a complete co-expressing landscape, and our data demonstrated that *CD27* expression was strongly connected with various immunological checkpoints in varied immunocytes and unique T cells.

### Drug Sensitivity Analysis of *CD27*

**Figure 9:**
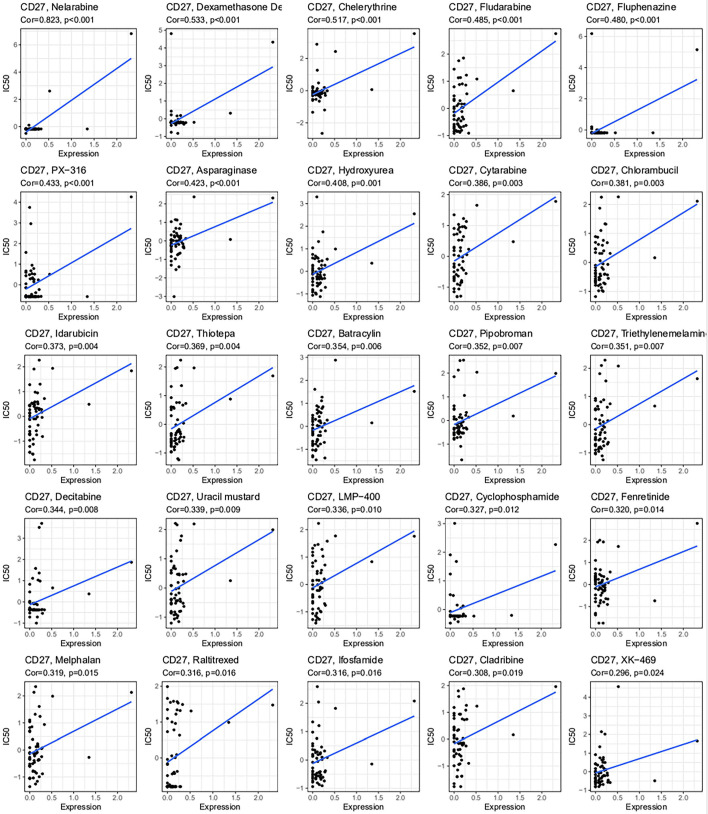
** Drug sensitivity analysis of *CD27*.** *CD27* expression was associated with the sensitivity of nelarabine, dexamethasone, chelerythrine, fludarabine, fluphenazine, PX-316, asparaginase, hydroxyurea, cytarabine, chlorambucil, idarubicin, thiotepa, batracylin, pipobroman, triethylenemelamine, decitabine, uracil mustard, LMP-400, cyclophosphamide, fenretinide, melphalan, raltitrexed, ifosfamide, cladribine, XK-469.

Using the CellMiner-TM database, we looked at the link between *CD27* expression and drug sensitivity (Figure 9). Notably, the drug sensitivity was positively linked with *CD27* expression.

### Functional Analysis by Gene Set Enrichment Analysis

**Figure 10:**
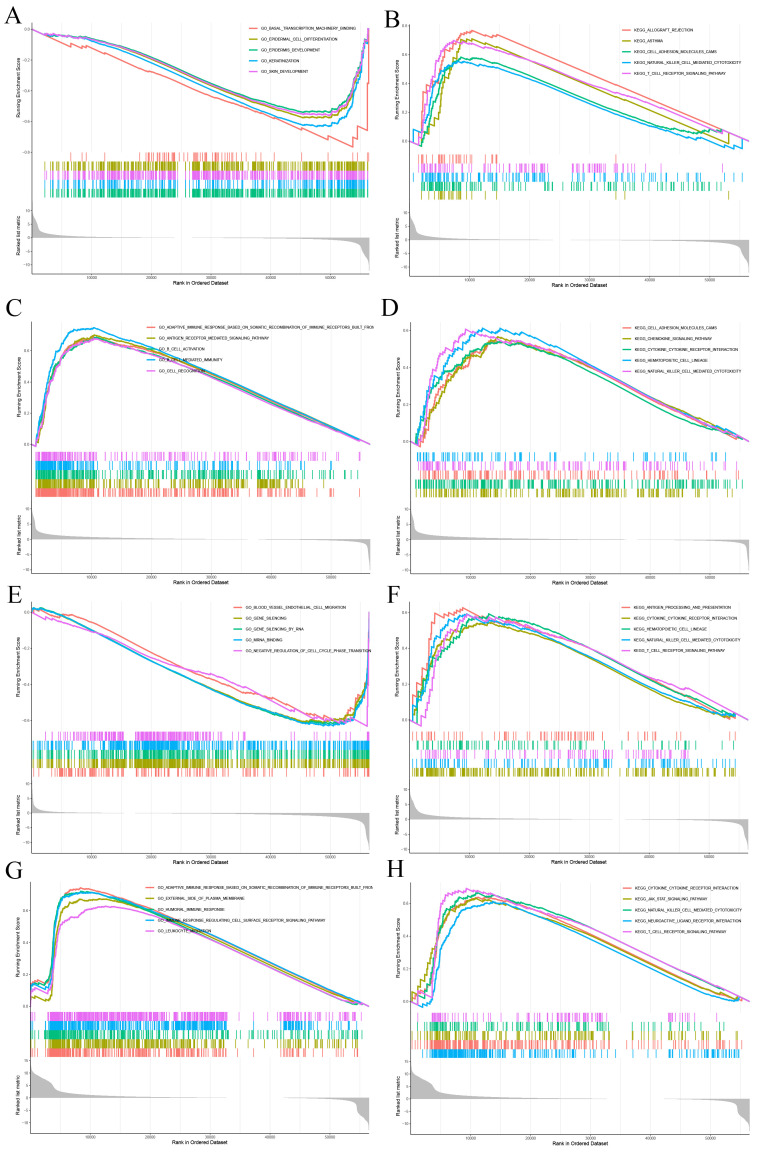
** GSEA for samples with high *CD27* expression and low expression.** (A, C, E and G) GO functional annotation of *CD27* in CESC, HNSC, UCEC and UVM. (B, D, F and H) KEGG pathway analysis of *CD27* in CESC, HNSC, UCEC and UVM.

GSEA was applied to study the primary biological function of *CD27* in CESC, HNSC, UCEC and UVM. In CESC, high expression of *CD27* corresponds to low levels of basal transcription, epithelial development, and epithelial differentiation (Figure 10A). This corresponds to elevated levels of cell adhesion, NK cytotoxicity, and TCR signaling pathways (Figure 10B). In HNSC, highly expressed *CD27* corresponds to elevated levels of adaptive immune response pathways based on somatic recombination, antigen receptor-mediated signaling pathways, B cell activation, chemokines, and NK cytotoxicity (Figure 10C-D). In UCEC, high expression of* CD27* corresponds to lower vascular endothelial migration, gene silencing, and cell cycle regulation (Figure 10E). However, it corresponds to a higher TCR pathway and NK cytotoxicity (Figure 10F). In UVM, high expression of* CD27* corresponds to higher humoral immunity, NK cytotoxicity, JAK-STAT inflammatory signaling pathway, and TCR signaling pathway (Figure 10G-H).

### Relationship between *CD27* expression and prognosis of SKCM and UVM

**Figure 11:**
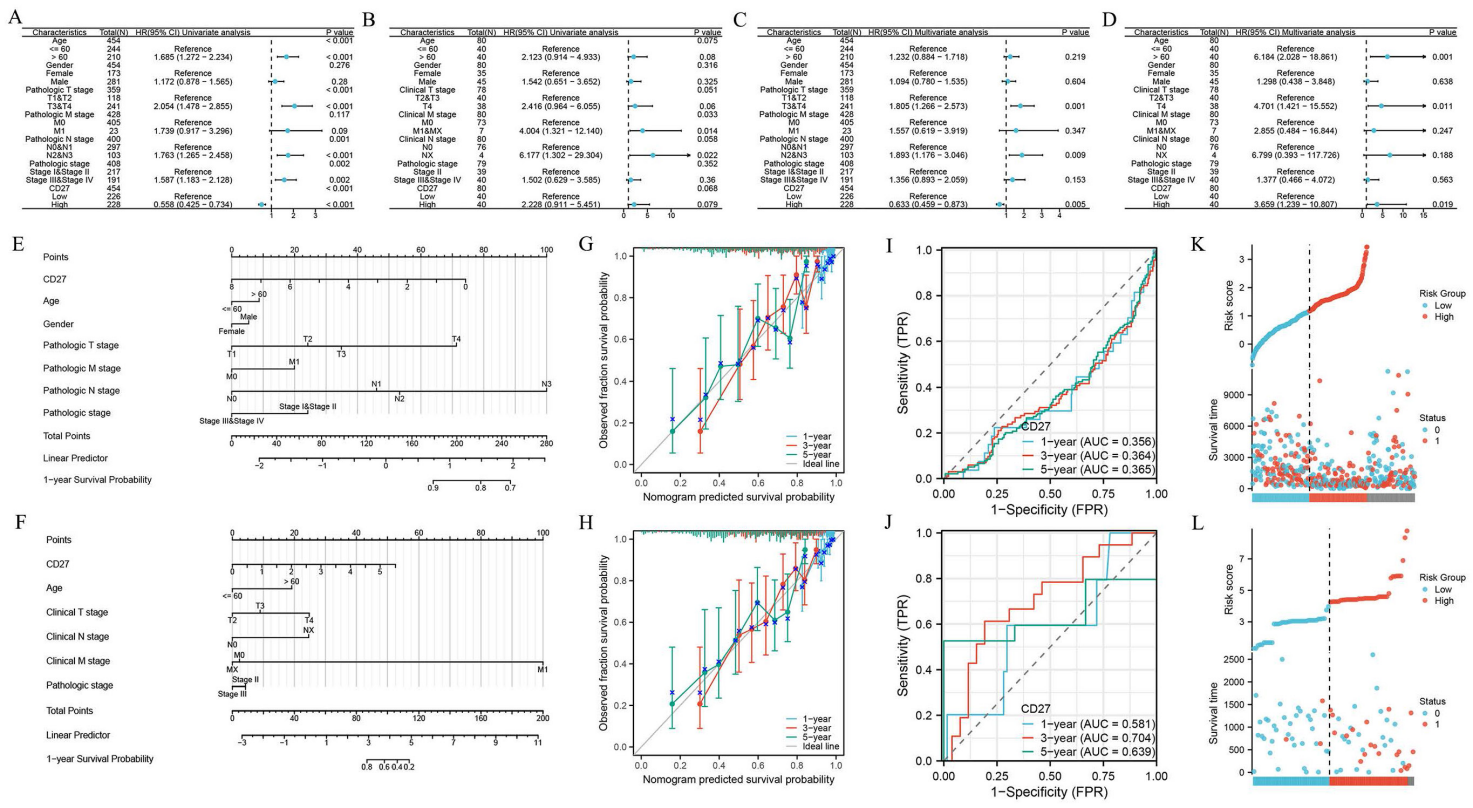
Univariate Cox regression analysis of *CD27* and other clinicopathological variables in SKCM (A), UVM (B). Multivariate Cox analysis of *CD27* and other clinicopathological variables in SKCM (C), UVM (D). Nomogram for 1-year, 3-year and 5-year OS of SKCM (E), UVM (F). Time-dependent ROC curves and AUC values for 1-year, 3-year and 5-year OS prediction of SKCM (G) and UVM (H) patients. Calibration plots for 1-year, 3-year and 5-year OS prediction of SKCM (I) and UVM (J). *CD27* expression, risk score and survival time distribution of SKCM (K) and UVM (L) patients.

By Cox regression analyses, we analyzed the predictors (the parameters include age, gender, clinical stage, pathologic stage, *CD27* expression level and so on). The univariate analysis showed that the factors we included were significantly associated with the OS of SKCM (Figure 11A) and UVM patients (Figure 11B). And these risk factors were further included in multivariate Cox regression (Figure 11C-D). Results showed that *CD27* was an independent prognostic factor of SKCM and UVM patients. The clinical features of SKCM (Figure 11E) and UVM (Figure 11F) were integrated into the nomogram mode. We developed time-dependent ROC curves and calibration plots to predicate the probability of 1-year, 3-year, and 5-year OS rate. The AUCs of 1-year, 3-year, and 5-year were 0.356, 0.364, and 0.365 of SKCM patients respectively (Figure 11I). And for UVM patients, the statistics are 0.581, 0.704, 0.639 respectively (Figure 11J). The predicted probability of calibration plots of SKCM (Figure 11G). and UVM (Figure 11H) was consistent with the results observed. We also analysis the correlation between risk score, survival time, and *CD27* expression of SKCM (Figure 11K) and UVM (Figure 11L) patients.

## Discussion

Cellular immunity against tumors is mainly achieved by initiating the activation of CD8^+^T cells. Specific receptors that TCR present on the surface of T cells recognize tumor antigens presented on the surface of tumor cells or APC, which require the assistance of MHC I 20, 21. In addition, the second necessary pathway to activate T cells is co-stimulation of receptors 30, which can result in cellular immune tolerance when in the state of deficiency 31, 32. Common T cell co-stimulation pathways such as *CD28*/*CD28L* axis, *PD-1*/*PD-L1* axis, etc. These co-stimulatory pathways, called immune checkpoints, can be divided into stimulatory and inhibitory 24, 33. For example, the *PD-1*/*PD-L1* axis is a common inhibitory immune checkpoint pathway. *PD-1* or *PD-L1* monoclonal antibodies have been used clinically to block inhibitory immune checkpoints to modulate the tumor immune environment 34, 35. Our research focuses on the *CD27*, which belongs to the tumor necrosis factor receptor family and regulates TCR signaling as a T cell co-receptor expressed on lymphocytes 12. And *CD70* is the only ligand for *CD27*^36^. The *CD27*/*CD70* axis is an immune checkpoint pathway that has not been fully studied and there is evidence that the *CD27* plays an important role as a stimulatory immune checkpoint in tumor progression and cellular immunity. In a B16 melanoma model, targeting the *CD27* with an agonistic antibody resulted in the reduction of growth in lung metastases and subcutaneous tumors 37. And in preclinical models of several cancers such as LAML and GBM, *CD27* targeting antibodies showed great efficacy 38-41. Therefore, the effects of the *CD27* in cancer were comprehensively evaluated by pan-cancer analysis, including regulation of immune invasion, TME, signal transduction, and prognosis. This helps us screen for high-value cancers targeted by the* CD27* and provide constructive advice on their treatment.

First, we evaluated the* CD27* expression differences in 33 cancers and came to meaningful conclusions. *CD27* showed significantly high expression in 21 cancers, the high expression and good prognosis of *CD27* in these cancers prove the theory that *CD27* acts as a co-stimulatory immune checkpoint in tumor cell immunity. Moreover, our study found that *CD27* expression exhibits consistency, which is manifested in common high expression 14. Therefore, we can infer that the *CD27* is initiated in tumor progression and have a positive inhibitory effect on the development of tumors. There are exceptions, the high expression of *CD27* in UVM always corresponds to a poor prognosis. We also did not detect significant *CD27* changes in UVM, suggesting that the *CD27* may be inhibited by certain pathways in UVM and alter its co-stimulatory immune checkpoint role. These show that the *CD27* has a dual role in the progression of certain cancers 42. The study of Ortiz-Cuaran et al. demonstrated that *CD27L* is highly expressed in non-small cell lung cancer and predicts reduced infiltration of CD8^+^T cells as well as immune depletion 43, 44. At the same time, CAR-T therapy targeting *CD27L* has also become a new role in cancer treatment 45, 46. A plausible explanation for this is that the *CD27*/*CD27L* axis activates the expression of other inhibitory immune checkpoints in long-term co-stimulation and ultimately depletes cellular immunity.

To further explore the role of the* CD27* in regulating cancer progression, we analyzed its effect on TIICs. Tumor infiltration of immune cells plays an important regulatory role in tumor progression. APC, mainly DC cells and macrophages. APC recognize tumor-specific receptors and stimulate T cell differentiation and proliferation 47-49. Our study found that the *CD27* is highly correlated with macrophage infiltration and differentiation, which is mainly manifested in the high expression of the axis enhancing the infiltration of M1-polarized macrophages and reducing the infiltration of M2-polarized macrophages. M1 polarization of macrophages significantly inhibits tumor growth, while M2 polarization is often accompanied by immune escape 50-52. This can reflect the positive effect of the *CD27* on tumor antigen presentation. CD8^+^T cells, as effector cells, are directly involved in the killing of tumor cells. Our study confirms that activation of the *CD27* is positively correlated with CD8^+^T cells in almost all cancers. However, *CD27* showed a negatively correlated co-expression landscape with NK cells. In UVM, we found that the expression of *CD27* initiates the NK cell activation pathway, which is inconsistent with the landscape in other cancers. This indicates that there may be other pathways in UVM that affect the effect of NK cells 53, resulting in abnormal landscape of TIICs, and corresponding to the poor prognosis reflected by the *CD27*.

Tumor-specific immunity is not only related to tumor infiltration of immune cells, but also to stromal cells. Stromal cells contain a variety of components and constitute tumor heterogeneity and even immunosuppression 54, 55. We use immunological scoring to gauge the quantity of invading CD3^+^/CD45RO^+^, CD3^+^/CD8^+^, or CD8^+^/CD45RO^+^ lymphocytes at the tumor's center and borders. A higher Immune Score or Stromal Score represents that the TME has more immune or matrix components. Our findings showed a substantial positive connection between *CD27* expression and stromal and immunological scores in these malignancies, showing that the quantity of stromal or immune cells rises concurrently with an increase in *CD27* expression levels. Additionally, we can see that immunological scores in the six cancers have a favorable connection with *CD27* expression. Additionally, the relationship between immunological check-point markers and* CD27* expression suggests that *CD27* has a function in controlling tumor immunology in malignancies, particularly in BRCA, PRAD, LUAD, BLCA, and OV. These data collectively point to a potential connection between abnormal *CD27* expression and immune infiltration of tumor cells.

*CD27* regulates multiple signaling pathways to influence cancer progression. We found that *CD27* was inversely correlated with multiple tumor-associated signaling pathways in four cancers, CESC, HNSC, UCEC and UVM, which were highly associated with the *CD27*. These include epidermal structure and differentiation, gene silencing, and cell cycle regulation. These pathways are associated with proto-oncogene activation, tumor heterogeneity, and abnormal cell cycle regulation 56-58. Therefore, it can be inferred that *CD27* can directly inhibit tumor progression by downregulating the relevant pathway. In addition, *CD27* synergistically upregulated several immune-related signaling pathways in four cancers, including antigen receptor-mediated signaling pathways, TCR signaling pathways, and chemokine signaling pathways. On the other hand, this proves that the *CD27* activates CD8^+^T cell-mediated cellular immunity and improves cancer prognosis 59, 60. In particular, *CD27* upregulates the JAK-STAT inflammatory pathway in UVM, activating NK cell-mediated cytotoxic pathway 61. This is related to previous findings that up-regulation to NK cell infiltration in UVM and may lead to UVM-specific adverse prognosis.

The *CD27* is expressed in cancer, which is related to different mutation patterns. The DNA damage repair mechanism known as MMRs is made up of several heterodimers 62. The accumulation of DNA replication mistakes caused by the functional loss of important genes in this pathway increases somatic mutation rates, MSI, and cancer 63, 64. Through correlation analysis, we discovered in this study that *CD27* expression was strongly correlated with the mutation levels of 5 MMR genes in human pan-cancer. Changes in DNA methylation status also play a role in the growth of cancer. According to research, cancer frequently exhibits hypermethylation of the gene promoter. Additionally, we found a significant link between *CD27* expression and four methylation-related genes, particularly in the cases of BRCA, HNSC, KICH, LIHC, LGG, PAAD, and TGCT. However, this evidence does not prove whether differential expression of *CD27* is related to genetic mutations in MMR. MMR mutations in cancer cells may be the direct cause of *CD27* expression and function abnormalities.

Patients with different forms of cancer who were treated with immune checkpoint inhibitors had a higher chance of surviving if they had TMB 65. In three cancers, including LAML, LGG, and UCEC,* CD27* expression was linked favorably with TMB. TMB was negatively correlated with *CD27* expression in eleven different cancers. Moreover, *CD27* expression was negatively correlated with MSI. And in most tumors the correlation between *CD27* expression and immune checkpoint biomarkers was incredibly significant, especially in BRCA, COAD, HNSC, KIRC, KICH, LIHC, PAAD, TGCT, and LUSC. We found that *CD27* expression had a positive correlation with the amount of *MLH1* genes in most malignancies. Besides, the results showed the prognostic impact of *CD27* across different cancers.

However, there are still caveats to our study that need to be addressed, despite the extensive investigation of pan-cancer using numerous databases. As a first step, a large amount of microarray and sequencing data was gathered through the examination of cancer tissue material. Additional research, including utilizing single-cell RNA sequencing data, is needed to resolve this issue. Second, *in vivo* and *in vitro* investigations were not included due to the study's emphasis on bioinformatic analysis of *CD27* expression and patient survival using multiple databases. To further understand *CD27* function in various cancers, more research is needed into its mechanism at the cellular and molecular levels. More mechanistic insights may be gleaned from prospective studies of *CD27* expression and immune cell infiltration in different cancer populations in the future.

## Conclusion

The *CD27* mutated in cancer and appears widely highly expressed and altered tumor immune invasion and stromal invasion by affecting multiple immune-related and inflammation signaling pathways, and upregulated macrophage M1 polarization and CD8^+^T cell activation and predicted a positive prognosis of cancer. The *CD27* plays a significant role in CESC, HNSC, UCEC and UVM, and may be used as a therapeutic target for related cancers.

## Supplementary Material

Supplementary tables.

## Abbreviations

K-M: The Kaplan-Meier
HPA: Human Protein Atlas
MSI: microsatellite instability
TMB: tumor mutational burden
DNMTs: DNA methyltransferases
MMR: the associated genes of mismatch repair
MSI: microsatellite instability
TIME: tumor immune microenvironment
ICIs: immune checkpoint inhibitors
TCGA: the Cancer Genome Atlas
CCLE: the Cancer Cell Line Encyclopedia
TCR: T cell reporter
MHCI: major histocompatibility complex class-I
TME: tumor microenvironment
APCs: antigen-presenting cells
GTEx: the Genotype-Tissue Expression
TIICs: tumor-infiltrating immune cells
GSEA: the Gene Set Enrichment Analysis
GO: Gene Ontology
KEGG: Kyoto Encyclopedia of Genes and Genomes
FDA: Food and Durg Administration
HNSC: head and neck squamous cell carcinoma
STAD: stomach adenocarcinoma
ESCA: esophageal carcinoma
COAD: colon adenocarcinoma
KIRC: kidney renal clear cell carcinoma
UCEC: uterine corpus endometrial carcinoma
KIRP: kidney renal papillary cell carcinoma
PAAD: pancreatic adenocarcinoma
LUSC: lung squamous cell carcinoma
LUAD: lung adenocarcinoma
LIHC: liver hepatocellular carcinoma
PRAD: prostate adenocarcinoma
BRCA: breast invasive carcinoma
DLBC: lymphoid neoplasm diffuse large B cell lymphoma
THCA: thyroid carcinoma
CESC: cervical squamous cell carcinoma
TGCT: testicular germ cell tumors
SKCM: skin cutaneous melanoma
READ: rectum adenocarcinoma
OV: ovarian serous cystadenocarcinoma
ACC: adrenocortical carcinoma
UCS: uterine carcinosarcoma
CLL: chronic lymphocytic leukemia
SARC: sarcoma
LGG: brain lower grade glioma
THYM: thymoma
BLCA: bladder urothelial carcinoma
DSS: disease-specific survival
PFI: progression-free interval
GBM: glioblastoma multiforme
LAML: acute myeloid leukemia
CHOL: cholangiocarcinoma
PCPG: pheochromocytoma and paraganglioma
TGCT: uveal melanoma
ECM: extracellular matrix
