# Supplementary material for: A Comprehensive Pan-cancer Analysis of the Biological Immunomodulatory Function and Clinical Value of CD27
Source: J Cancer. 2024 Jan 1;15(2):508–25. doi: 10.7150/jca.85446 (PMC10758032; doi:10.7150/jca.85446)
Supplement: Supplementary file 1 — Supplementary tables. [file jcav15p0508s1.zip › Supplementary materials/Table S1.pdf]

Expression levels of *CD27* comparing tumor and normal tissues from TCGA database.

Group1	Group2	Number	Minimum	Maximum	Median	IQR	Mean	SD	SE
ACC	Tumor	77	0	3.484	0.322	0.755	0.713	0.835	0.095
BLCA	Normal	19	0.475	5.989	2.134	2.309	2.596	1.575	0.361
BLCA	Tumor	407	0.084	7.029	1.828	2.061	2.054	1.343	0.067
BRCA	Normal	113	0.151	5.775	2.22	1.122	2.244	0.927	0.087
BRCA	Tumor	1099	0.084	7.213	2.546	1.998	2.664	1.371	0.041
CESC	Normal	3	1.269	2.987	1.551	0.859	1.936	0.922	0.532
CESC	Tumor	306	0.151	6.438	2.548	1.953	2.653	1.285	0.073
CHOL	Normal	9	0.696	1.824	0.918	0.867	1.17	0.456	0.152
CHOL	Tumor	36	0.124	8.037	1.198	2.311	2.045	1.765	0.294
COAD	Normal	41	1.47	5.327	3.981	0.448	3.94	0.718	0.112
COAD	Tumor	290	0.124	5.865	2.013	1.704	2.06	1.144	0.067
DLBC	Tumor	47	3.884	9.696	7.124	1.538	6.986	1.182	0.172
ESCA	Normal	13	0.333	2.864	1.395	1.366	1.561	0.849	0.236
ESCA	Tumor	182	0.275	6.672	2.204	1.854	2.214	1.24	0.092
GBM	Normal	5	0.475	0.774	0.687	0.035	0.669	0.114	0.051
GBM	Tumor	166	0	3.699	0.82	0.662	0.956	0.603	0.047
HNSC	Normal	44	0.475	3.695	1.763	1.284	1.918	0.935	0.141
HNSC	Tumor	520	0.057	7.061	2.664	1.998	2.792	1.411	0.062
KICH	Normal	25	0.202	4.456	0.714	1.295	1.2	1.129	0.226
KICH	Tumor	66	0.084	2.563	0.566	0.478	0.723	0.541	0.067
KIRC	Normal	72	0.111	3.281	0.766	1.01	1.052	0.819	0.097
KIRC	Tumor	531	0.098	6.869	3.108	2.108	3.219	1.486	0.065
KIRP	Normal	32	0.214	4.23	1.077	1.127	1.338	1.127	0.199
KIRP	Tumor	289	0.164	8.121	1.546	1.387	1.847	1.202	0.071
LAML	Tumor	173	0.782	4.969	2.644	1.429	2.667	0.944	0.072
LGG	Tumor	523	0.057	4.983	0.595	0.377	0.698	0.46	0.02
LIHC	Normal	50	0.029	3.146	1.025	0.737	1.207	0.746	0.106
LIHC	Tumor	371	0.057	6.329	1.17	1.442	1.487	1.195	0.062
LUAD	Normal	59	1.208	5.34	2.398	0.939	2.598	0.833	0.108
LUAD	Tumor	515	0.138	6.576	3.635	1.758	3.592	1.199	0.053
LUSC	Normal	50	0.687	6.176	2.914	1.078	3.071	1.097	0.155
LUSC	Tumor	498	0.275	7.076	3.201	1.848	3.186	1.276	0.057
MESO	Tumor	87	0.872	6.746	2.573	1.769	2.762	1.277	0.137
OV	Tumor	427	0	4.659	1.637	1.382	1.75	0.975	0.047
PAAD	Normal	4	2.667	6.893	2.946	1.145	3.863	2.025	1.012
PAAD	Tumor	179	0.164	6.93	2.585	1.891	2.652	1.306	0.098
PCPG	Normal	3	0.516	0.824	0.566	0.154	0.635	0.165	0.095
PCPG	Tumor	182	0.043	4.524	0.876	0.754	1.052	0.718	0.053
PRAD	Normal	52	0.176	4.629	1.606	0.782	1.676	0.85	0.118
PRAD	Tumor	496	0.138	5.967	1.639	1.253	1.811	0.962	0.043
READ	Normal	10	2.834	5.066	3.677	1.084	3.851	0.729	0.231
READ	Tumor	93	0.164	4.667	1.952	1.513	2.035	1.029	0.107
SARC	Normal	2	0.506	1.356	0.931	0.425	0.931	0.601	0.425
SARC	Tumor	262	0	7.724	1.698	2.097	2.187	1.658	0.102
SKCM	Normal	1	6.556	6.556	6.556	0	6.556		
SKCM	Tumor	469	0.138	7.642	3.051	3.156	3.155	1.871	0.086
STAD	Normal	36	0.124	6.233	3.071	3.792	2.976	1.982	0.33
STAD	Tumor	414	0.202	8.783	2.897	1.834	2.92	1.309	0.064
TGCT	Tumor	154	0.31	6.878	3.9	2.176	3.803	1.387	0.112
THCA	Normal	59	1.275	6.82	3.696	1.814	3.9	1.315	0.171
THCA	Tumor	512	0.189	9.937	3.066	2.307	3.295	1.579	0.07
THYM	Normal	2	5.852	6.146	5.999	0.147	5.999	0.207	0.147
THYM	Tumor	119	1.189	7.026	4.881	1.435	4.702	1.193	0.109
UCEC	Normal	23	0.799	3.126	1.585	0.605	1.719	0.517	0.108
UCEC	Tumor	181	0	6.783	2.074	2.143	2.161	1.321	0.098
UCS	Tumor	57	0.138	4.871	0.941	1.116	1.218	1.007	0.133
UVM	Tumor	79	0	5.628	0.678	1.446	1.326	1.441	0.162
