# Supplementary material for: A Comprehensive Pan-cancer Analysis of the Biological Immunomodulatory Function and Clinical Value of CD27
Source: J Cancer. 2024 Jan 1;15(2):508–25. doi: 10.7150/jca.85446 (PMC10758032; doi:10.7150/jca.85446)
Supplement: Supplementary file 1 — Supplementary tables. [file jcav15p0508s1.zip › Supplementary materials/Table S2.pdf]

Expression levels of *CD27* comparing tumor and normal tissues from TCGA and GTEx database.

Group1	Group2	Number	Minimum	Maximum	Median	IQR	Mean	SD	SE
ACC	Normal	128	0	2.064	0.683	0.374	0.727	0.36	0.032
ACC	Tumor	77	0	3.484	0.322	0.755	0.713	0.835	0.095
BLCA	Normal	28	0.475	5.989	1.823	2.271	2.28	1.499	0.283
BLCA	Tumor	407	0.084	7.029	1.828	2.061	2.054	1.343	0.067
BRCA	Normal	292	0.057	5.775	1.664	1.777	1.684	1.068	0.062
BRCA	Tumor	1099	0.084	7.213	2.546	1.998	2.664	1.371	0.041
CESC	Normal	13	0.411	2.987	1.269	0.483	1.448	0.693	0.192
CESC	Tumor	306	0.151	6.438	2.548	1.953	2.653	1.285	0.073
CHOL	Normal	9	0.696	1.824	0.918	0.867	1.17	0.456	0.152
CHOL	Tumor	36	0.124	8.037	1.198	2.311	2.045	1.765	0.294
COAD	Normal	349	0	5.463	1.956	3.144	2.223	1.599	0.086
COAD	Tumor	290	0.124	5.865	2.013	1.704	2.06	1.144	0.067
DLBC	Normal	444	0.263	8.089	3.758	2.64	4.035	1.873	0.089
DLBC	Tumor	47	3.884	9.696	7.124	1.538	6.986	1.182	0.172
ESCA	Normal	666	0	4.577	0.868	0.851	1.147	0.808	0.031
ESCA	Tumor	182	0.275	6.672	2.204	1.854	2.214	1.24	0.092
GBM	Normal	1157	0	2.39	0.322	0.332	0.355	0.248	0.007
GBM	Tumor	166	0	3.699	0.82	0.662	0.956	0.603	0.047
HNSC	Normal	44	0.475	3.695	1.763	1.284	1.918	0.935	0.141
HNSC	Tumor	520	0.057	7.061	2.664	1.998	2.792	1.411	0.062
KICH	Normal	53	0	5.5	0.623	0.697	1.104	1.288	0.177
KICH	Tumor	66	0.084	2.563	0.566	0.478	0.723	0.541	0.067
KIRC	Normal	100	0	5.5	0.678	0.891	1.043	1.019	0.102
KIRC	Tumor	531	0.098	6.869	3.108	2.108	3.219	1.486	0.065
KIRP	Normal	60	0	5.5	0.669	0.999	1.189	1.276	0.165
KIRP	Tumor	289	0.164	8.121	1.546	1.387	1.847	1.202	0.071
LAML	Normal	70	0	0.299	0.043	0.084	0.052	0.06	0.007
LAML	Tumor	173	0.782	4.969	2.644	1.429	2.667	0.944	0.072
LGG	Normal	1152	0	2.39	0.31	0.332	0.353	0.248	0.007
LGG	Tumor	523	0.057	4.983	0.595	0.377	0.698	0.46	0.02
LIHC	Normal	160	0.029	3.146	0.774	0.631	0.924	0.584	0.046
LIHC	Tumor	371	0.057	6.329	1.17	1.442	1.487	1.195	0.062
LUAD	Normal	347	0	5.564	2.26	1.084	2.356	0.859	0.046
LUAD	Tumor	515	0.138	6.576	3.635	1.758	3.592	1.199	0.053
LUSC	Normal	338	0	6.176	2.307	1.17	2.419	0.935	0.051
LUSC	Tumor	498	0.275	7.076	3.201	1.848	3.186	1.276	0.057
MESO	Tumor	87	0.872	6.746	2.573	1.769	2.762	1.277	0.137
OV	Normal	88	0.214	2.163	0.836	0.5	0.914	0.425	0.045
OV	Tumor	427	0	4.659	1.637	1.382	1.75	0.975	0.047
PAAD	Normal	171	0	6.893	0.356	0.269	0.475	0.659	0.05
PAAD	Tumor	179	0.164	6.93	2.585	1.891	2.652	1.306	0.098
PCPG	Normal	3	0.516	0.824	0.566	0.154	0.635	0.165	0.095
PCPG	Tumor	182	0.043	4.524	0.876	0.754	1.052	0.718	0.053
PRAD	Normal	152	0.176	4.807	1.414	0.926	1.587	0.858	0.07
PRAD	Tumor	496	0.138	5.967	1.639	1.253	1.811	0.962	0.043
READ	Normal	318	0	5.463	1.33	2.827	2.052	1.559	0.087
READ	Tumor	93	0.164	4.667	1.952	1.513	2.035	1.029	0.107
SARC	Normal	2	0.506	1.356	0.931	0.425	0.931	0.601	0.425
SARC	Tumor	262	0	7.724	1.698	2.097	2.187	1.658	0.102
SKCM	Normal	813	0.057	6.556	0.604	0.407	0.667	0.389	0.014
SKCM	Tumor	469	0.138	7.642	3.051	3.156	3.155	1.871	0.086
STAD	Normal	210	0	6.233	1.604	1.625	1.947	1.314	0.091
STAD	Tumor	414	0.202	8.783	2.897	1.834	2.92	1.309	0.064
TGCT	Normal	165	0.057	1.971	0.379	0.251	0.456	0.32	0.025
TGCT	Tumor	154	0.31	6.878	3.9	2.176	3.803	1.387	0.112
THCA	Normal	338	0	6.82	2.516	1.593	2.8	1.266	0.069
THCA	Tumor	512	0.189	9.937	3.066	2.307	3.295	1.579	0.07
THYM	Normal	446	0.263	8.089	3.798	2.747	4.044	1.873	0.089
THYM	Tumor	119	1.189	7.026	4.881	1.435	4.702	1.193	0.109
UCEC	Normal	101	0.465	3.126	1.275	0.541	1.316	0.469	0.047
UCEC	Tumor	181	0	6.783	2.074	2.143	2.161	1.321	0.098
UCS	Normal	78	0.465	2.23	1.167	0.547	1.198	0.382	0.043
UCS	Tumor	57	0.138	4.871	0.941	1.116	1.218	1.007	0.133
UVM	Tumor	79	0	5.628	0.678	1.446	1.326	1.441	0.162
