# Supplementary material for: A Comprehensive Pan-cancer Analysis of the Biological Immunomodulatory Function and Clinical Value of CD27
Source: J Cancer. 2024 Jan 1;15(2):508–25. doi: 10.7150/jca.85446 (PMC10758032; doi:10.7150/jca.85446)
Supplement: Supplementary file 1 — Supplementary tables. [file jcav15p0508s1.zip › Supplementary materials/Table S3.pdf]

Clinical annotation and Pathological featuresrn of the individual patient in TCGA database.

sample	_PATIENT	cancer typ	age at ini	gender	race	ajcc path	clinical st	histologic	histologic	initial pat	menopau	birth days	vital statu	tumor sta	last conta	death day	cause of	new tum	new tum	new tum	new tum	treatment	margin st	residual t	OS	OS.time	DSS	DSS.time	DFI	DFI.time	PFI	PFI.time
TCGA-OR	TCGA-OR	ACC	58	MALE	WHITE	Stage II		Adrenocortical carcir		2000		-21496	Dead	WITH TUMOR		1355		Distant M	Peritoneal Surfaces		754	Complete Remission/Response			1	1355	1	1355	1	754	1	754
TCGA-OR	TCGA-OR	ACC	44	FEMALE	WHITE	Stage IV		Adrenocortical carcir		2004		-16090	Dead	WITH TUMOR		1677		Distant M	Soft Tissue		289	Progressive Disease			1	1677	1	1677			1	289
TCGA-OR	TCGA-OR	ACC	23	FEMALE	WHITE	Stage III		Adrenocortical carcir		2008		-8624	Alive	WITH TUN	2091			Distant M	Lung		53	Complete Remission/Response			0	2091	0	2091	1	53	1	53
TCGA-OR	TCGA-OR	ACC	23	FEMALE	WHITE	Stage IV		Adrenocortical carcir		2000		-8451	Dead	WITH TUMOR		423		Locoregic	Peritoneal Surfaces		126	Progressive Disease			1	423	1	423			1	126
TCGA-OR	TCGA-OR	ACC	30	MALE	WHITE	Stage III		Adrenocortical carcir		2000		-11171	Dead	WITH TUMOR		365		Locoregic	Other, sp	vena cava	50	Progressive Disease			1	365	1	365			1	50
TCGA-OR	TCGA-OR	ACC	29	FEMALE	BLACK OI	Stage II		Adrenocortical carcir		2006		-10839	Alive	TUMOR F	2703							Complete Remission/Response			0	2703	0	2703	0	2703	0	2703
TCGA-OR	TCGA-OR	ACC	30	FEMALE	WHITE	Stage III		Adrenocortical carcir		2009		-11279	Dead	WITH TUMOR		490		Locoregic	Retroperitoneal lymp		162	Progressive Disease			1	490	1	490			1	162
TCGA-OR	TCGA-OR	ACC	66	MALE	WHITE	Stage III		Adrenocortical carcir		2011		-24266	Dead	WITH TUMOR		579		Locoregic	Peritoneal Surfaces		530	Complete Remission/Response			1	579	1	579	1	530	1	530
TCGA-OR	TCGA-OR	ACC	22	FEMALE	WHITE	Stage II		Adrenocortical carcir		2010		-8175	Alive	WITH TUN	1352			Distant M	Lung		414	Complete Remission/Response			0	1352	0	1352	1	414	1	414
TCGA-OR	TCGA-OR	ACC	53	FEMALE	WHITE	Stage IV		Adrenocortical carcir		2004		-19436	Dead	WITH TUMOR		922									1	922	1	922			1	922
TCGA-OR	TCGA-OR	ACC	52	MALE	WHITE	Stage IV		Adrenocortical carcir		2000		-19172	Dead			551						[Unknown]			1	551		551			0	551
TCGA-OR	TCGA-OR	ACC	37	MALE	WHITE			Adrenocortical carcir		2001		-13862	Dead			1750									1	1750		1750			0	1750
TCGA-OR	TCGA-OR	ACC	57	FEMALE	WHITE	Stage II		Adrenocortical Carci		2005		-20989	Alive	TUMOR F	3038							Complete Remission/Response			0	3038	0	3038	0	3038	0	3038
TCGA-OR	TCGA-OR	ACC	17	FEMALE	WHITE	Stage I		Adrenocortical carcir		2005		-6455	Dead	WITH TUMOR		2105		Locoregional Recurrence			477	[Unknown]			1	2105	1	2105			1	477
TCGA-OR	TCGA-OR	ACC	69	FEMALE	WHITE	Stage II		Adrenocortical carcir		2007		-25502	Alive	TUMOR F	2015							Complete Remission/Response			0	2015	0	2015	0	2015	0	2015
TCGA-OR	TCGA-OR	ACC	61	MALE	WHITE	Stage IV		Adrenocortical carcir		2008		-22390	Dead	WITH TUMOR		541									1	541	1	541			1	541
TCGA-OR	TCGA-OR	ACC	32	FEMALE	WHITE	Stage II		Adrenocortical Carci		2008		-11994	Alive	TUMOR F	2110							Complete Remission/Response			0	2110	0	2110	0	2110	0	2110
TCGA-OR	TCGA-OR	ACC	22	MALE	WHITE	Stage I		Adrenocortical carcir		2008		-8177	Alive	TUMOR F	2331							Complete Remission/Response			0	2331	0	2331	0	2331	0	2331
TCGA-OR	TCGA-OR	ACC	65	MALE	WHITE	Stage IV		Adrenocortical carcir		2009		-24082	Dead	WITH TUMOR		490						Progressive Disease			1	490	1	490			1	490
TCGA-OR	TCGA-OR	ACC	49	MALE	WHITE	Stage IV		Adrenocortical carcir		2009		-18214	Alive	TUMOR F	1497							Partial Remission/Response			0	1497	0	1497			0	1497
TCGA-OR	TCGA-OR	ACC	36	FEMALE	WHITE	Stage I		Adrenocortical carcir		2009		-13155	Alive	TUMOR F	2056							Complete Remission/Response			0	2056	0	2056	0	2056	0	2056
TCGA-OR	TCGA-OR	ACC	25	FEMALE	WHITE	Stage IV		Adrenocortical carcir		2011		-9359	Dead	WITH TUMOR		562		Distant M	Liver		72	Progressive Disease			1	562	1	562			1	72
TCGA-OR	TCGA-OR	ACC	26	FEMALE	WHITE	Stage I		Adrenocortical carcir		2005		-9547	Alive	TUMOR F	1194							Complete Remission/Response			0	1194	0	1194	0	1194	0	1194
TCGA-OR	TCGA-OR	ACC	40	MALE	WHITE	Stage II		Adrenocortical carcir		2012		-14667	Alive	TUMOR F	464							Complete Remission/Response			0	464	0	464	0	464	0	464
TCGA-OR	TCGA-OR	ACC	26	FEMALE	WHITE	Stage II		Adrenocortical Carci		2006		-9667	Alive	TUMOR F	1103							Complete Remission/Response			0	1103	0	1103	0	1103	0	1103
TCGA-OR	TCGA-OR	ACC	45	MALE	WHITE	Stage I		Adrenocortical carcir		2002		-16604	Alive	TUMOR F	3688							Complete Remission/Response			0	3688	0	3688	0	3688	0	3688
TCGA-OR	TCGA-OR	ACC	65	FEMALE	WHITE	Stage II		Adrenocortical carcir		2011		-24017	Alive	WITH TUN	383			Distant M	Lung		166				0	383	0	383			1	166
TCGA-OR	TCGA-OR	ACC	65	FEMALE	WHITE	Stage II		Adrenocortical carcir		2011		-23794	Alive	WITH TUN	907							Stable Disease			0	907	0	907			0	907
TCGA-OR	TCGA-OR	ACC	58	FEMALE	WHITE	Stage III		Adrenocortical carcir		2010		-21292	Dead	WITH TUMOR		289		Distant M	Other, sp	Ovarian	268	Progressive Disease			1	289	1	289			1	268
TCGA-OR	TCGA-OR	ACC	55	MALE	WHITE	Stage II		Adrenocortical carcir		2008		-20319	Alive	TUMOR F	2023			New Prim	Lymph Node(s)		953	Complete Remission/Response			0	2023	0	2023	0	2023	1	953
TCGA-OR	TCGA-OR	ACC	47	MALE	WHITE	Stage II		Adrenocortical carcir		2008		-17513	Alive	TUMOR F	2202							Complete Remission/Response			0	2202	0	2202	0	2202	0	2202
TCGA-OR	TCGA-OR	ACC	50	MALE	WHITE	Stage III		Adrenocortical carcir		2009		-18402	Alive	TUMOR F	950										0	950	0	950			0	950
TCGA-OR	TCGA-OR	ACC	68	FEMALE	WHITE	Stage IV		Adrenocortical carcir		2011		-25190	Dead	WITH TUMOR		552		Distant M	Liver		75	Progressive Disease			1	552	1	552			1	75
TCGA-OR	TCGA-OR	ACC	60	MALE	WHITE	Stage II		Adrenocortical carcir		2011		-22218	Alive	WITH TUN	822			Distant M	Liver		247	Progressive Disease			0	822	0	822			1	247
TCGA-OR	TCGA-OR	ACC	69	FEMALE	WHITE	Stage II		Adrenocortical carcir		2009		-25400	Alive	WITH TUN	1029			Distant M	Lung		659	Progressive Disease			0	1029	0	1029			1	659
TCGA-OR	TCGA-OR	ACC	48	MALE	WHITE	Stage II		Adrenocortical carcir		2006		-17596	Alive	TUMOR F	3289			Locoregic	Peritoneal Surfaces		826	Complete Remission/Response			0	3289	0	3289	1	826	1	826
TCGA-OR	TCGA-OR	ACC	32	FEMALE	WHITE	Stage III		Adrenocortical carcir		2010		-11970	Dead	WITH TUMOR		994		Distant M	Lung		97	Progressive Disease			1	994	1	994			1	97
TCGA-OR	TCGA-OR	ACC	53	MALE	WHITE	Stage II		Adrenocortical Carci		2005		-19375	Alive	TUMOR F	3465							Complete Remission/Response			0	3465	0	3465	0	3465	0	3465
TCGA-OR	TCGA-OR	ACC	64	FEMALE	WHITE	Stage III		Adrenocortical carcir		2011		-23481	Alive	TUMOR F	1082							Complete Remission/Response			0	1082	0	1082	0	1082	0	1082
TCGA-OR	TCGA-OR	ACC	59	FEMALE	WHITE	Stage III		Adrenocortical carcir		2012		-21907	Dead	WITH TUMOR		498		Distant M	Lung		108	Progressive Disease			1	498	1	498			1	108
TCGA-OR	TCGA-OR	ACC	56	FEMALE	WHITE	Stage II		Adrenocortical carcir		2010		-20657	Alive	WITH TUN	1493			Distant M	Liver		391	Complete Remission/Response			0	1493	0	1493	1	391	1	391
TCGA-OR	TCGA-OR	ACC	39	MALE	WHITE	Stage II		Adrenocortical carcir		2012		-14435	Alive	TUMOR F	749							Complete Remission/Response			0	749	0	749	0	749	0	749
TCGA-OR	TCGA-OR	ACC	61	FEMALE	WHITE	Stage II		Adrenocortical carcir		2002		-22596	Dead	WITH TUMOR		344		Locoregic	Peritoneal Surfaces		236				1	344	1	344			1	236
TCGA-OR	TCGA-OR	ACC	61	FEMALE	WHITE	Stage II		Adrenocortical carcir		2011		-22550	Alive	WITH TUN	741			Distant M	Soft Tissue		214	Progressive Disease			0	741	0	741			1	214
TCGA-OR	TCGA-OR	ACC	39	FEMALE	WHITE	Stage IV		Adrenocortical carcir		2009		-14606	Alive	WITH TUN	1414			Distant M	Lung		541	Progressive Disease			0	1414	0	1414			1	541
TCGA-OR	TCGA-OR	ACC	45	FEMALE	WHITE	Stage II		Adrenocortical carcir		2006		-16702	Alive	TUMOR F	2777			Distant M	Liver		1250	Complete Remission/Response			0	2777	0	2777	1	1250	1	1250
TCGA-OR	TCGA-OR	ACC	20	FEMALE	WHITE	Stage II		Adrenocortical carcir		2006		-7459	Alive	TUMOR F	2677							Complete Remission/Response			0	2677	0	2677	0	2677	0	2677
TCGA-OR	TCGA-OR	ACC	72	MALE	WHITE	Stage II		Adrenocortical carcir		2006		-26404	Alive	TUMOR F	2549							Complete Remission/Response			0	2549	0	2549	0	2549	0	2549
TCGA-OR	TCGA-OR	ACC	44	FEMALE	WHITE	Stage I		Adrenocortical carcir		2006		-16217	Alive	TUMOR F	2895			Distant M	Other, sp	Pancreas	2099	Complete Remission/Response			0	2895	0	2895	1	2099	1	2099
TCGA-OR	TCGA-OR	ACC	37	FEMALE	WHITE	Stage II		Adrenocortical carcir		2000		-13559	Alive	TUMOR F	4673							Complete Remission/Response			0	4673	0	4673	0	4673	0	4673
TCGA-OR	TCGA-OR	ACC	17	FEMALE	WHITE	Stage III		Adrenocortical carcir		2003		-6250	Alive	TUMOR F	3878			Distant M	Lung		1116	Complete Remission/Response			0	3878	0	3878	1	1116	1	1116
TCGA-OR	TCGA-OR	ACC	55	FEMALE	WHITE	Stage III		Adrenocortical carcir		2009		-20328	Alive	TUMOR F	2076							Complete Remission/Response			0	2076	0	2076	0	2076	0	2076
TCGA-OR	TCGA-OR	ACC	25	FEMALE	WHITE	Stage III		Adrenocortical carcir		2010		-9298	Alive	WITH TUN	1364			Distant M	Bone		62	Progressive Disease			0	1364	0	1364			1	62
TCGA-OR	TCGA-OR	ACC	23	FEMALE	WHITE	Stage IV		Adrenocortical carcir		2010		-8745	Dead	WITH TUMOR		391		Locoregic	Peritoneal Surfaces		178	Progressive Disease			1	391	1	391			1	178
TCGA-OR	TCGA-OR	ACC	42	MALE	WHITE	Stage II		Adrenocortical carcir		2012		-15594	Dead	WITH TUMOR		125		Locoregic	Other, sp	abdomen	36	Progressive Disease			1	125	1	125			1	36
TCGA-OR	TCGA-OR	ACC	37	FEMALE	WHITE	Stage II		Adrenocortical carcir		2000		-13840	Alive	WITH TUN	1781			Distant M	Bone		685	Complete Remission/Response			0	1781	0	1781	1	685	1	685
TCGA-OR	TCGA-OR	ACC	83	FEMALE	WHITE	Stage II		Adrenocortical carcir		2001		-30535	Dead	TUMOR FREE		1852						Complete Remission/Response			1	1852	0	1852	0	1852	0	1852
TCGA-OR	TCGA-OR	ACC	67	FEMALE	WHITE	Stage I		Adrenocortical carcir		2000		-24763	Alive	TUMOR F	4628							Complete Remission/Response			0	4628	0	4628	0	4628	0	4628
TCGA-OR	TCGA-OR	ACC	48	FEMALE	WHITE	Stage III		Adrenocortical carcir		2011		-17819	Alive	TUMOR F	967							Complete Remission/Response			0	967	0	967	0	967	0	967
TCGA-OR	TCGA-OR	ACC	77	FEMALE	WHITE	Stage I		Adrenocortical carcir		2010		-28270	Alive	TUMOR F	1317							Complete Remission/Response			0	1317	0	1317	0	1317	0	1317
TCGA-OR	TCGA-OR	ACC	60	MALE	WHITE	Stage II		Adrenocortical carcir		2011		-22057	Alive	TUMOR F	861							Complete Remission/Response			0	861	0	861	0	861	0	861
TCGA-OR	TCGA-OR	ACC	36	FEMALE	WHITE	Stage II		Adrenocortical carcir		2011		-13472	Alive	TUMOR F	885							Complete Remission/Response			0	885	0	885	0	885	0	885
TCGA-OR	TCGA-OR	ACC	53	FEMALE	WHITE	Stage II		Adrenocortical carcir		2011		-19526	Alive	TUMOR F	871							Complete Remission/Response			0	871	0	871	0	871	0	871
TCGA-OR	TCGA-OR	ACC	52	FEMALE	WHITE	Stage II		Adrenocortical carcir		2012		-19242	Alive	TUMOR F	719							Complete Remission/Response			0	719	0	719	0	719	0	719
TCGA-OR	TCGA-OR	ACC	59	MALE	WHITE	Stage IV		Adrenocortical carcir		2006		-21565	Dead	WITH TUMOR		1204		Distant M	Liver		611	Progressive Disease			1	1204	1	1204			1	611
TCGA-OR	TCGA-OR	ACC	71	FEMALE	WHITE	Stage IV		Adrenocortical carcir		2011		-26084	Dead	WITH TUMOR		159		Distant M	Lung		50	Progressive Disease			1	159	1	159			1	50
TCGA-OR	TCGA-OR	ACC	52	MALE	WHITE	Stage III		Adrenocortical carcir		1998		-19111	Dead	WITH TUMOR		1197		Distant M	Lung		444	[Unknown]			1	1197	1	1197			1	444
TCGA-OR	TCGA-OR	ACC	14	MALE	WHITE	Stage II		Adrenocortical carcir		1997		-5383	Dead	WITH TUMOR		662		Locoregic	Peritoneal Surfaces		265	[Unknown]			1	662	1	662			1	265
TCGA-OR	TCGA-OR	ACC	74	FEMALE	WHITE	Stage III		Adrenocortical carcir		2002		-27179	Dead	TUMOR FREE		445						Complete Remission/Response			1	445	0	445	0	445	0	445
TCGA-OR	TCGA-OR	ACC	46	MALE	WHITE	Stage III		Adrenocortical carcir		2008		-16875	Alive	WITH TUN	1589			Distant M	Lung		1393	Complete Remission/Response			0	1589	0	1589	1	1393	1	1393
TCGA-OR	TCGA-OR	ACC	36	FEMALE	WHITE	Stage II		Adrenocortical carcir		2005		-13182	Dead	WITH TUMOR		2385						Progressive Disease			1	2385	1	2385			1	2385
TCGA-OR	TCGA-OR	ACC	42	FEMALE	WHITE	Stage IV		Adrenocortical carcir		1998		-15536	Dead	WITH TUMOR		436		Distant M	Bone		61	Progressive Disease			1	436	1	436			1	61
TCGA-OR	TCGA-OR	ACC	54	FEMALE	WHITE	Stage IV		Adrenocortical carcir		2005		-20069	Dead	WITH TUMOR		1105		Distant M	Lung		98	Progressive Disease			1	1105	1	1105			1	98
TCGA-OR	TCGA-OR	ACC	62	MALE	[Not Evalu	Stage II		Adrenocortical Carci		2007		-22919	Alive	TUMOR F	2740							Complete Remission/Response			0	2740	0	2740	0	2740	0	2740
TCGA-OR	TCGA-OR	ACC	75	FEMALE	[Not Evalu	Stage II		Adrenocortical carcir		2007		-27607	Dead	WITH TUMOR		1613		Distant M	Liver		213	Complete Remission/Response			1	1613	1	1613	1	213	1	213
TCGA-OR	TCGA-OR	ACC	23	MALE	[Not Evalu	Stage II		Adrenocortical carcir		2008		-8735	Alive	TUMOR F	1858							Complete Remission/Response			0	1858	0	1858	0	1858	0	1858
TCGA-OR	TCGA-OR	ACC	31	FEMALE	[Not Evalu	Stage II		Adrenocortical carcir		2008		-11414	Alive	TUMOR F	2342							Complete Remission/Response			0	2342	0	2342	0	2342	0	2342
TCGA-OR	TCGA-OR	ACC	61	FEMALE	[Not Evalu	Stage II		Adrenocortical carcir		2008		-22370	Dead	WITH TUMOR		2405		Distant M	Lung		153	Complete Remission/Response			1	2405	1	2405	1	153	1	153
TCGA-OR	TCGA-OR	ACC	37	FEMALE	[Not Evalu	Stage II		Adrenocortical carcir		2009		-13574	Alive	TUMOR F	1857							Complete Remission/Response			0	1857	0	1857	0	1857	0	1857
TCGA-OR	TCGA-OR	ACC	30	FEMALE	[Not Evalu	Stage II		Adrenocortical carcir		2011		-11261	Alive	TUMOR F	853							Complete Remission/Response			0	853	0	853	0	853	0	853
TCGA-OR	TCGA-OR	ACC	34	FEMALE	[Not Evalu	Stage II		Adrenocortical carcir		2011		-12663	Alive	TUMOR F	1096							Complete Remission/Response			0	1096	0	1096	0	1096	0	1096
TCGA-OR	TCGA-OR	ACC	57	MALE	[Not Evalu	Stage III		Adrenocortical carcir		2012		-21032	Alive	TUMOR F	549							Complete Remission/Response			0	549	0	549	0	549	0	549
TCGA-OU	TCGA-OU	ACC	53	FEMALE	WHITE	Stage IV		Adrenocortical carcir		2011		-19492	Alive	WITH TUN	1171			Distant M	Lung		351	Stable Disease			0	1171	0	1171			1	351
TCGA-P6-	TCGA-P6-	ACC	55	FEMALE	WHITE	Stage III		Adrenocortical carcir		2010		-20106	Dead	WITH TUMOR		207		Distant M	Lung		143	[Unknown]			1	207	1	207			1	143
TCGA-P6-	TCGA-P6-	ACC	45	FEMALE	WHITE	Stage IV		Adrenocortical carcir		2011		-16659	Dead	WITH TUMOR		383		Distant M	Lung		11	Complete Remission/Response			1	383	1	383			1	11
TCGA-P6-	TCGA-P6-	ACC	59	FEMALE	WHITE	Stage IV		Adrenocortical carcir		2010		-21627	Dead	WITH TUMOR		0									1	0	1	0			1	0
TCGA-PA	TCGA-PA	ACC	51	MALE	WHITE	Stage II		Adrenocortical carcir		2012		-18926	Alive	TUMOR F	756							Complete Remission/Response			0	756	0	756	0	756	0	756
TCGA-PK	TCGA-PK	ACC	42	MALE	WHITE	Stage II		Adrenocortical carcir		2004		-15373	Alive	TUMOR F	3623							Complete Remission/Response			0	3623	0	3623	0	3623	0	3623
TCGA-PK	TCGA-PK	ACC	27	FEMALE	ASIAN	Stage II		Adrenocortical carcir		2012		-10173	Alive	TUMOR F	616							Complete Remission/Response			0	616	0	616	0	616	0	616
TCGA-PK	TCGA-PK	ACC	63	MALE	[Unknown	Stage I		Adrenocortical carcir		2011		-23345	Alive	TUMOR F	1201							Complete Remission/Response			0	1201	0	1201	0	1201	0	1201
TCGA-PK	TCGA-PK	ACC	63	MALE	[Unknown]			Adrenocortical carcir		2003		-23293	Alive	WITH TUN	1293			Distant M	Lung		213				0	1293	0	1293			1	213
TCGA-PK	TCGA-PK	ACC	44	FEMALE	ASIAN	Stage III		Adrenocortical carcir		2011		-16182	Alive	WITH TUN	679			Distant M	Lung		131	Complete Remission/Response			0	679	0	679	1	131	1	131
TCGA-2F-	TCGA-2F-	BLCA	63	MALE	WHITE	Stage IV		Muscle in	High Grac	2008		-23323	Dead	WITH TUMOR		734		Distant M	Liver		651	Progressive Disease			1	734	1	734			1	651
TCGA-2F-	TCGA-2F-	BLCA	66	MALE	WHITE	Stage IV		Muscle in	High Grac	2011		-24428	Dead	WITH TUMOR		364		Distant M	Liver		343	Progressive Disease			1	364	1	364			1	343
TCGA-2F-	TCGA-2F-	BLCA	69	MALE	WHITE	Stage III		Muscle in	High Grac	2007		-25259	Alive	TUMOR F	2886							Complete Remission/Response			0	2886	0	2886	0	2886	0	2886
TCGA-2F-	TCGA-2F-	BLCA	59	FEMALE	[Not Evalu	Stage III		Muscle in	High Grac	2000		-21848	Dead	WITH TUMOR		3183		Distant M	Lung		3086	Complete Remission/Response			1	3183	1	3183	1	3086	1	3086
TCGA-2F-	TCGA-2F-	BLCA	83	MALE	WHITE	Stage II		Muscle in	High Grac	2006		-30520	Alive	TUMOR F	3314							Complete Remission/Response			0	3314	0	3314	0	3314	0	3314
TCGA-2F-	TCGA-2F-	BLCA	67	FEMALE	WHITE	Stage III		Muscle in	High Grac	2013		-24703	Dead	WITH TUMOR		254		Distant M	Bone		85	Stable Disease			1	254	1	254			1	85
TCGA-4Z-	TCGA-4Z-	BLCA	65	MALE	WHITE	Stage III		Muscle in	High Grac	2013		-23817	Alive	TUMOR F	495							Complete Remission/Response			0	495	0	495	0	495	0	495
TCGA-4Z-	TCGA-4Z-	BLCA	65	MALE	WHITE	Stage III		Muscle in	High Grac	2010		-24090	Dead	TUMOR FREE		1367						Complete Remission/Response			1	1367	0	1367	0	1367	0	1367
TCGA-4Z-	TCGA-4Z-	BLCA	64	MALE	BLACK OI	Stage II		Muscle in	High Grac	2013		-23645	Alive	TUMOR F	512							Complete Remission/Response			0	512	0	512	0	512	0	512
TCGA-4Z-	TCGA-4Z-	BLCA	79	MALE	WHITE	Stage III		Muscle in	High Grac	2012		-28981	Dead	TUMOR FREE		510									1	510	0	510			0	510
TCGA-4Z-	TCGA-4Z-	BLCA	73	MALE	BLACK OI	Stage IV		Muscle in	High Grac	2010		-26781	Dead	WITH TUMOR		522		Locoregic	Lymph Node Only		353	Partial Remission/Response			1	522	1	522			1	353
TCGA-4Z-	TCGA-4Z-	BLCA	66	MALE	BLACK OI	Stage III		Muscle in	High Grac	2010		-24111	Dead	TUMOR FREE		1064									1	1064	0	1064			0	1064
TCGA-4Z-	TCGA-4Z-	BLCA	55	MALE	WHITE	Stage II		Muscle in	High Grac	2012		-20424	Alive	WITH TUN	840			Locoregic	Other, sp	Rectum	760	Complete Remission/Response			0	840	0	840	1	760	1	760
TCGA-4Z-	TCGA-4Z-	BLCA	60	MALE	WHITE	Stage II		Muscle in	High Grac	2010		-22056	Alive	TUMOR F	1522							Complete Remission/Response			0	1522	0	1522	0	1522	0	1522
TCGA-4Z-	TCGA-4Z-	BLCA	73	MALE	WHITE	Stage II		Muscle in	High Grac	2012		-26873	Dead	TUMOR FREE		19									1	19	0	19			0	19
TCGA-4Z-	TCGA-4Z-	BLCA	80	MALE	WHITE	Stage II		Muscle in	High Grac	2010		-29457	Dead	TUMOR FREE		1270						Complete Remission/Response			1	1270	0	1270	0	1270	0	1270
TCGA-4Z-	TCGA-4Z-	BLCA	59	MALE	BLACK OI	Stage IV		Muscle in	High Grac	2010		-21893	Dead	WITH TUMOR		1556		Distant M	Bone		1150	Complete Remission/Response			1	1556	1	1556			1	1150
TCGA-4Z-	TCGA-4Z-	BLCA	52	MALE	WHITE	Stage II		Muscle in	High Grac	2009		-19280	Alive	TUMOR F	2024							Complete Remission/Response			0	2024	0	2024	0	2024	0	2024
TCGA-4Z-	TCGA-4Z-	BLCA	61	MALE	WHITE	Stage IV		Muscle in	High Grac	2008		-22397	Alive	WITH TUN	460							Progressive Disease			0	460	0	460			0	460
TCGA-4Z-	TCGA-4Z-	BLCA	66	MALE	BLACK OI	Stage IV		Muscle in	High Grac	2009		-24465	Dead	WITH TUMOR		311		Locoregic	Other, sp	Pelvis	136	Complete Remission/Response			1	311	1	311			1	136
TCGA-4Z-	TCGA-4Z-	BLCA	72	MALE	BLACK OI	Stage III		Muscle in	High Grac	2010		-26430	Alive	WITH TUN	1454			Distant M	Bone		221	[Unknown]			0	1454	0	1454			1	221
TCGA-4Z-	TCGA-4Z-	BLCA	60	MALE	WHITE	Stage IV		Muscle in	High Grac	2011		-22271	Alive	WITH TUN	1029			New Prim	Lung		648	Complete Remission/Response			0	1029	0	1029			1	648
TCGA-5N	TCGA-5N	BLCA	76	FEMALE	WHITE	Stage III		Muscle in	High Grac	2011		-27777	Dead	TUMOR FREE		76						Complete Remission/Response			1	76	0	76	0	76	0	76
TCGA-5N	TCGA-5N	BLCA	73	FEMALE	WHITE	Stage III		Muscle in	High Grac	2012		-26906	Dead	WITH TUMOR		530					276	Complete Remission/Response			1	530	1	530	1	276	1	276
TCGA-BL-	TCGA-BL-	BLCA	73	MALE	WHITE	Stage I			High Grac	2009		-26819	Alive		1219			Locoregic	Bladder		630	Complete Remission/Response			0	1219	0	1219	1	630	1	630
TCGA-BL-	TCGA-BL-	BLCA	57	FEMALE	WHITE	Stage III		Muscle in	High Grac	2010		-20885	Dead	WITH TUMOR		223		Metastatic			216	Progressive Disease			1	223	1	223			1	216
TCGA-BL-	TCGA-BL-	BLCA	65	MALE	WHITE	Stage IV		Muscle in	High Grac	2010		-23927	Dead	WITH TUMOR		81						Stable Disease			1	81	1	81			1	81
TCGA-BL-	TCGA-BL-	BLCA	65	MALE	WHITE	Stage IV		Muscle in	High Grac	2010		-23927	Dead	WITH TUMOR		81						Stable Disease			1	81	1	81			1	81
TCGA-BL-	TCGA-BL-	BLCA	62	MALE	WHITE	Stage III		Muscle in	High Grac	2010		-23000	Dead	WITH TUMOR		205		Metastati	Bone		111	Progressive Disease			1	205	1	205			1	111
TCGA-BL-	TCGA-BL-	BLCA	80	FEMALE	WHITE	Stage III		Muscle in	High Grac	2012		-29247	Alive	WITH TUN	377			Distant M	Lung		242	Complete Remission/Response			0	377	0	377	1	242	1	242
TCGA-BT-	TCGA-BT-	BLCA	75	MALE	WHITE	Stage III		Muscle in	High Grac	2007		-27533	Dead	WITH TUMOR		200						Progressive Disease			1	200	1	200			1	200
TCGA-BT-	TCGA-BT-	BLCA	70	FEMALE	WHITE	Stage III		Muscle in	High Grac	2009		-25570	Dead	WITH TUMOR		400		Metastati	Other, sp	lower abc	326	Progressive Disease			1	400	1	400			1	326
TCGA-BT-	TCGA-BT-	BLCA	75	MALE	WHITE	Stage II		Muscle in	High Grac	1999		-27646	Dead	WITH TUMOR		579		Distant M	Other, sp	Adrenal	254	Progressive Disease			1	579	1	579			1	254
TCGA-BT-	TCGA-BT-	BLCA	75	MALE	WHITE	Stage II		Muscle in	High Grac	1999		-27646	Dead	WITH TUMOR		579		Distant M	Other, sp	Adrenal	254	Progressive Disease			1	579	1	579			1	254
TCGA-BT-	TCGA-BT-	BLCA	72	MALE		Stage III		Muscle in	High Grac	2003		-26456	Dead	WITH TUMOR		795						Progressive Disease			1	795	1	795			1	795
TCGA-BT-	TCGA-BT-	BLCA	72	MALE		Stage III		Muscle in	High Grac	2003		-26456	Dead	WITH TUMOR		795						Progressive Disease			1	795	1	795			1	795
TCGA-BT-	TCGA-BT-	BLCA	75	MALE	WHITE	Stage III		Muscle in	High Grac	2003		-27534	Dead	WITH TUMOR		370		Locoregic	Lymph Node Only		315	Progressive Disease			1	370	1	370			1	315
TCGA-BT-	TCGA-BT-	BLCA	81	FEMALE	WHITE	Stage III		Muscle in	High Grac	2005		-29634	Dead			544									1	544		544			0	544
TCGA-BT-	TCGA-BT-	BLCA	81	FEMALE	WHITE	Stage III		Muscle in	High Grac	2005		-29634	Dead			544									1	544		544			0	544
TCGA-BT-	TCGA-BT-	BLCA	73	MALE	WHITE	Stage IV		Muscle in	High Grac	2006		-26778	Dead	WITH TUMOR		593						Progressive Disease			1	593	1	593			1	593
TCGA-BT-	TCGA-BT-	BLCA	73	MALE	WHITE	Stage IV		Muscle in	High Grac	2006		-26778	Dead	WITH TUMOR		593						Progressive Disease			1	593	1	593			1	593
TCGA-BT-	TCGA-BT-	BLCA	79	FEMALE	WHITE	Stage IV		Muscle in	High Grac	2006		-28987	Dead			154									1	154		154			0	154
TCGA-BT-	TCGA-BT-	BLCA	79	FEMALE	WHITE	Stage IV		Muscle in	High Grac	2006		-28987	Dead			154									1	154		154			0	154
TCGA-BT-	TCGA-BT-	BLCA	63	MALE	WHITE	Stage IV		Muscle in	High Grac	2007		-23220	Dead	WITH TUMOR		453		Distant M	Liver		314	Progressive Disease			1	453	1	453			1	314
TCGA-BT-	TCGA-BT-	BLCA	70	FEMALE	WHITE	Stage III		Muscle in	High Grac	2008		-25761	Dead	TUMOR FREE		455						Complete Remission/Response			1	455	0	455	0	455	0	455
TCGA-BT-	TCGA-BT-	BLCA	70	FEMALE	WHITE	Stage III		Muscle in	High Grac	2008		-25761	Dead	TUMOR FREE		455						Complete Remission/Response			1	455	0	455	0	455	0	455
TCGA-BT-	TCGA-BT-	BLCA	59	FEMALE	BLACK OI	Stage IV		Muscle in	High Grac	2008		-21551	Dead			154									1	154		154			0	154
TCGA-BT-	TCGA-BT-	BLCA	59	FEMALE	BLACK OI	Stage IV		Muscle in	High Grac	2008		-21551	Dead			154									1	154		154			0	154
TCGA-BT-	TCGA-BT-	BLCA	71	MALE	WHITE	Stage II		Muscle in	High Grac	2009		-26078	Dead	TUMOR FREE		254						Complete Remission/Response			1	254	0	254	0	254	0	254
TCGA-BT-	TCGA-BT-	BLCA	71	MALE	WHITE	Stage II		Muscle in	High Grac	2009		-26078	Dead	TUMOR FREE		254						Complete Remission/Response			1	254	0	254	0	254	0	254
TCGA-BT-	TCGA-BT-	BLCA	56	MALE	WHITE	Stage IV		Muscle in	High Grac	2009		-20519	Dead	WITH TUMOR		251						Progressive Disease			1	251	1	251			1	251
TCGA-BT-	TCGA-BT-	BLCA	56	MALE	WHITE	Stage IV		Muscle in	High Grac	2009		-20519	Dead	WITH TUMOR		251						Progressive Disease			1	251	1	251			1	251
TCGA-BT-	TCGA-BT-	BLCA	54	MALE	WHITE	Stage III		Muscle in	High Grac	2010		-20032	Alive	TUMOR F	522							Complete Remission/Response			0	522	0	522	0	522	0	522
TCGA-BT-	TCGA-BT-	BLCA	54	MALE	WHITE	Stage III		Muscle in	High Grac	2010		-20032	Alive	TUMOR F	522							Complete Remission/Response			0	522	0	522	0	522	0	522
TCGA-BT-	TCGA-BT-	BLCA	73	FEMALE	WHITE	Stage III		Muscle in	High Grac	2010		-26846	Dead	TUMOR FREE		492						Complete Remission/Response			1	492	0	492	0	492	0	492
TCGA-BT-	TCGA-BT-	BLCA	73	FEMALE	WHITE	Stage III		Muscle in	High Grac	2010		-26846	Dead	TUMOR FREE		492						Complete Remission/Response			1	492	0	492	0	492	0	492
TCGA-BT-	TCGA-BT-	BLCA	78	FEMALE	WHITE	Stage IV		Muscle in	High Grac	2011		-28673	Dead	WITH TUMOR		623		Distant M	Other, sp	brain and	555	Progressive Disease			1	623	1	623			1	555
TCGA-BT-	TCGA-BT-	BLCA	76	MALE	WHITE	Stage IV		Muscle in	High Grac	2010		-28030	Dead	WITH TUMOR		142						Progressive Disease			1	142	1	142			1	142
TCGA-BT-	TCGA-BT-	BLCA	76	MALE	WHITE	Stage III		Muscle in	High Grac	2011		-27821	Alive	TUMOR F	789							Complete Remission/Response			0	789	0	789	0	789	0	789
TCGA-BT-	TCGA-BT-	BLCA	80	MALE	WHITE	Stage II		Muscle in	High Grac	2011		-29314	Dead	WITH TUMOR		303						Stable Disease			1	303	1	303			1	303
TCGA-BT-	TCGA-BT-	BLCA	59	MALE	WHITE	Stage IV		Muscle in	High Grac	2011		-21788	Dead	WITH TUMOR		534						Partial Remission/Response			1	534	1	534			1	534
TCGA-BT-	TCGA-BT-	BLCA	64	MALE	WHITE	Stage II		Muscle in	High Grac	2011		-23584	Alive	TUMOR F	873							Complete Remission/Response			0	873	0	873	0	873	0	873
TCGA-BT-	TCGA-BT-	BLCA	74	MALE	WHITE	Stage III		Muscle in	High Grac	2012		-27045	Alive	TUMOR F	1108							Complete Remission/Response			0	1108	0	1108	0	1108	0	1108
TCGA-BT-	TCGA-BT-	BLCA	64	MALE	WHITE	Stage IV		Muscle in	High Grac	2011		-23713	Alive	TUMOR F	864							Complete Remission/Response			0	864	0	864			0	864
TCGA-C4	TCGA-C4	BLCA	69	FEMALE		Stage IV		Muscle in	High Grac	2006		-25202	Dead	WITH TUMOR		273						Partial Remission/Response			1	273	1	273			1	273
TCGA-C4	TCGA-C4	BLCA	60	MALE		Stage II		Muscle in	High Grac	2007		-22068	Alive	TUMOR F	59										0	59	0	59			0	59
TCGA-C4	TCGA-C4	BLCA	71	MALE		Stage III		Muscle in	High Grac	2006		-26178	Alive	TUMOR F	89										0	89	0	89			0	89
TCGA-C4	TCGA-C4	BLCA	82	FEMALE		Stage III		Muscle in	High Grac	2008		-29951	Alive	TUMOR F	700										0	700	0	700			0	700
TCGA-C4	TCGA-C4	BLCA	77	MALE		Stage IV		Muscle in	High Grac	2008		-28428	Dead	WITH TUMOR		62									1	62	1	62			1	62
TCGA-CF	TCGA-CF	BLCA	62	MALE	ASIAN	Stage III		Muscle in	High Grac	2010		-22777	Alive	WITH TUN	389			Distant M	Liver		294	Complete Remission/Response			0	389	0	389	1	294	1	294
TCGA-CF	TCGA-CF	BLCA	75	FEMALE	ASIAN	Stage III		Muscle in	High Grac	2010		-27565	Alive	WITH TUN	382			Distant M	Other, sp	Pelvis	133	Complete Remission/Response			0	382	0	382	1	133	1	133
TCGA-CF	TCGA-CF	BLCA	52	MALE	ASIAN	Stage III		Muscle in	High Grac	2011		-19270	Alive	TUMOR F	425							Complete Remission/Response			0	425	0	425	0	425	0	425
TCGA-CF	TCGA-CF	BLCA	34	MALE	ASIAN	Stage III		Muscle in	Low Grad	2011		-12557	Alive	TUMOR F	383							Complete Remission/Response			0	383	0	383	0	383	0	383
TCGA-CF	TCGA-CF	BLCA	48	MALE	ASIAN	Stage II		Muscle in	Low Grad	2011		-17600	Alive	TUMOR F	369							Complete Remission/Response			0	369	0	369	0	369	0	369
TCGA-CF	TCGA-CF	BLCA	75	MALE	ASIAN	Stage II		Muscle in	Low Grad	2011		-27458	Alive	TUMOR F	398							Complete Remission/Response			0	398	0	398	0	398	0	398
TCGA-CF	TCGA-CF	BLCA	62	MALE	ASIAN	Stage II		Muscle in	Low Grad	2011		-22716	Alive	TUMOR F	370							Complete Remission/Response			0	370	0	370	0	370	0	370
TCGA-CF	TCGA-CF	BLCA	41	MALE	ASIAN	Stage II		Muscle in	Low Grad	2011		-15225	Alive	TUMOR F	333							Complete Remission/Response			0	333	0	333	0	333	0	333
TCGA-CF	TCGA-CF	BLCA	58	FEMALE	ASIAN	Stage II		Muscle in	Low Grad	2011		-21347	Dead	WITH TUMOR		385		Distant M	Bone		139	Complete Remission/Response			1	385	1	385	1	139	1	139
TCGA-CF	TCGA-CF	BLCA	52	MALE	ASIAN	Stage II		Muscle in	High Grac	2012		-19265	Alive	TUMOR F	379							Complete Remission/Response			0	379	0	379	0	379	0	379
TCGA-CF	TCGA-CF	BLCA	42	MALE	ASIAN	Stage II		Muscle in	Low Grad	2012		-15497	Alive	TUMOR F	368							Complete Remission/Response			0	368	0	368	0	368	0	368
TCGA-CF	TCGA-CF	BLCA	60	MALE	ASIAN	Stage II		Muscle in	Low Grad	2012		-22200	Alive	TUMOR F	384							Complete Remission/Response			0	384	0	384	0	384	0	384
TCGA-CF	TCGA-CF	BLCA	55	MALE	ASIAN	Stage II		Muscle in	Low Grad	2012		-20432	Alive	TUMOR F	373							Complete Remission/Response			0	373	0	373	0	373	0	373
TCGA-CF	TCGA-CF	BLCA	59	MALE	ASIAN	Stage II		Muscle in	Low Grad	2012		-21734	Alive	TUMOR F	399							Complete Remission/Response			0	399	0	399	0	399	0	399
TCGA-CF	TCGA-CF	BLCA	67	MALE	ASIAN	Stage II		Muscle in	Low Grad	2012		-24625	Alive	TUMOR F	365							Complete Remission/Response			0	365	0	365	0	365	0	365
TCGA-CF	TCGA-CF	BLCA	54	MALE	ASIAN	Stage II		Muscle in	Low Grad	2013		-20059	Alive	TUMOR F	368							Complete Remission/Response			0	368	0	368	0	368	0	368
TCGA-CF	TCGA-CF	BLCA	55	FEMALE	ASIAN	Stage II		Muscle in	High Grac	2013		-20259	Alive	TUMOR F	345							Complete Remission/Response			0	345	0	345	0	345	0	345
TCGA-CF	TCGA-CF	BLCA	59	MALE	ASIAN	Stage II		Muscle in	High Grac	2013		-21636	Alive	TUMOR F	345							Complete Remission/Response			0	345	0	345	0	345	0	345
TCGA-CF	TCGA-CF	BLCA	52	MALE	ASIAN	Stage II		Muscle in	High Grac	2013		-19195	Alive	TUMOR F	361							Complete Remission/Response			0	361	0	361	0	361	0	361
TCGA-CF	TCGA-CF	BLCA	85	MALE	ASIAN	Stage II		Muscle in	Low Grad	2013		-31236	Alive	TUMOR F	-64							Complete Remission/Response			0	0	0	0	0	0	0	0
TCGA-CF	TCGA-CF	BLCA	85	MALE	ASIAN	Stage III		Muscle in	High Grac	2013		-31099	Dead	WITH TUMOR		565		Locoregional (Urothelial tumor			322	Complete Remission/Response			1	565	1	565	1	322	1	322
TCGA-CF	TCGA-CF	BLCA	50	MALE	ASIAN	Stage I			High Grac	2013		-18445	Alive	TUMOR F	398							Complete Remission/Response			0	398	0	398	0	398	0	398
TCGA-CU	TCGA-CU	BLCA	60	MALE	WHITE	Stage III		Muscle in	High Grac	2005		-21927	Dead			393									1	393		393			0	393
TCGA-CU	TCGA-CU	BLCA	60	MALE	WHITE	Stage III		Muscle in	High Grac	2005		-21927	Dead			393									1	393		393			0	393
TCGA-CU	TCGA-CU	BLCA	84	MALE	WHITE	Stage IV		Muscle in	High Grac	2009		-30817	Dead	WITH TUMOR		149		Metastati	Other, sp	peritoneu	118	Complete Remission/Response			1	149	1	149			1	118
TCGA-CU	TCGA-CU	BLCA	83	MALE	WHITE	Stage IV		Muscle in	High Grac	2010		-30674	Dead	WITH TUMOR		460		Metastati	Lymph Node Only		219	Complete Remission/Response			1	460	1	460			1	219
TCGA-CU	TCGA-CU	BLCA	83	MALE	WHITE	Stage IV		Muscle in	High Grac	2010		-30674	Dead	WITH TUMOR		460		Metastati	Lymph Node Only		219	Complete Remission/Response			1	460	1	460			1	219
TCGA-CU	TCGA-CU	BLCA	75	MALE	WHITE	Stage III		Muscle in	High Grac	2011		-27692	Alive	WITH TUN	562			Distant M	Lung		166	Progressive Disease			0	562	0	562			1	166
TCGA-CU	TCGA-CU	BLCA	58	MALE	WHITE	Stage IV		Muscle in	High Grac	2011		-21233	Alive	TUMOR F	158							Complete Remission/Response			0	158	0	158			0	158
TCGA-CU	TCGA-CU	BLCA	67	MALE	WHITE	Stage III		Muscle in	High Grac	2011		-24524	Alive	TUMOR F	906			Distant M	Lung		370	Complete Remission/Response			0	906	0	906	1	370	1	370
TCGA-CU	TCGA-CU	BLCA	70	MALE	WHITE	Stage III		Muscle in	High Grac	2012		-25706	Dead	TUMOR FREE		56						Complete Remission/Response			1	56	0	56	0	56	0	56
TCGA-CU	TCGA-CU	BLCA	76	MALE	WHITE	Stage IV		Muscle in	High Grac	2013		-27963	Dead	WITH TUMOR		413		New Prim	Lymph Node Only		256	Partial Remission/Response			1	413	1	413			1	256
TCGA-DK	TCGA-DK	BLCA	60	MALE	WHITE	Stage IV		Muscle in	High Grac	2010		-22235	Dead	WITH TUMOR		665									1	665	1	665			1	665
TCGA-DK	TCGA-DK	BLCA	79	MALE	WHITE	Stage II		Muscle in	High Grac	2010		-29111	Dead	TUMOR FREE		65						Complete Remission/Response			1	65	0	65	0	65	0	65
TCGA-DK	TCGA-DK	BLCA	53	MALE	WHITE	Stage IV		Muscle in	High Grac	2008		-19554	Alive	TUMOR F	2020							Complete Remission/Response			0	2020	0	2020			0	2020
TCGA-DK	TCGA-DK	BLCA	53	MALE	WHITE	Stage IV		Muscle in	High Grac	2008		-19554	Alive	TUMOR F	2020							Complete Remission/Response			0	2020	0	2020			0	2020
TCGA-DK	TCGA-DK	BLCA	67	FEMALE	WHITE	Stage IV		Muscle in	High Grac	2010		-24499	Alive	WITH TUN	560			Metastati	Lung		112				0	560	0	560			1	112
TCGA-DK	TCGA-DK	BLCA	57	MALE	WHITE	Stage III		Muscle in	High Grac	2010		-21093	Alive	TUMOR F	578							Complete Remission/Response			0	578	0	578	0	578	0	578
TCGA-DK	TCGA-DK	BLCA	74	FEMALE	WHITE	Stage IV		Muscle in	High Grac	2010		-27126	Dead			508		Metastati	Lung		364	Progressive Disease			1	508		508			1	364
TCGA-DK	TCGA-DK	BLCA	72	MALE	WHITE	Stage III		Muscle in	High Grac	2000		-26307	Alive	TUMOR F	3981							Complete Remission/Response			0	3981	0	3981	0	3981	0	3981
TCGA-DK	TCGA-DK	BLCA	69	MALE	WHITE	Stage IV		Muscle in	High Grac	2002		-25317	Alive	TUMOR F	3420										0	3420	0	3420			0	3420
TCGA-DK	TCGA-DK	BLCA	84	MALE	WHITE	Stage III		Muscle in	High Grac	2010		-30707	Alive	WITH TUN	491			Metastati	Bone		211	Progressive Disease			0	491	0	491			1	211
TCGA-DK	TCGA-DK	BLCA	84	FEMALE	WHITE	Stage IV		Muscle in	High Grac	2010		-30914	Alive	TUMOR F	536			Metastati	Lymph Node Only		283	Progressive Disease			0	536	0	536			1	283
TCGA-DK	TCGA-DK	BLCA	65	MALE	WHITE	Stage III		Muscle in	High Grac	2010		-24095	Alive	TUMOR F	475							Complete Remission/Response			0	475	0	475	0	475	0	475
TCGA-DK	TCGA-DK	BLCA	80	FEMALE	WHITE	Stage IV		Muscle in	High Grac	2010		-29332	Dead	TUMOR FREE		1420									1	1420	0	1420			0	1420
TCGA-DK	TCGA-DK	BLCA	73	FEMALE	WHITE	Stage II		Muscle in	High Grac	2010		-26726	Alive	TUMOR F	546							Complete Remission/Response			0	546	0	546	0	546	0	546
TCGA-DK	TCGA-DK	BLCA	63	FEMALE	WHITE	Stage IV		Muscle in	High Grac	2010		-23116	Dead	WITH TUMOR		237		Distant M	Liver		163	Complete Remission/Response			1	237	1	237			1	163
TCGA-DK	TCGA-DK	BLCA	79	MALE	WHITE	Stage III		Muscle in	High Grac	2001		-29037	Alive	TUMOR F	4343							Complete Remission/Response			0	4343	0	4343	0	4343	0	4343
TCGA-DK	TCGA-DK	BLCA	81	MALE	WHITE	Stage IV		Muscle in	High Grac	2005		-29789	Alive	TUMOR F	2656							Complete Remission/Response			0	2656	0	2656			0	2656
TCGA-DK	TCGA-DK	BLCA	87	MALE	WHITE	Stage IV		Muscle in	High Grac	2011		-32076	Dead	TUMOR FREE		146						Complete Remission/Response			1	146	0	146			0	146
TCGA-DK	TCGA-DK	BLCA	79	FEMALE	WHITE	Stage IV		Muscle in	High Grac	2011		-29047	Dead	TUMOR FREE		413						Complete Remission/Response			1	413	0	413			0	413
TCGA-DK	TCGA-DK	BLCA	76	MALE	WHITE	Stage III		Muscle in	High Grac	2011		-28106	Dead	WITH TUMOR		248		Locoregic	Lymph Node Only		201	Complete Remission/Response			1	248	1	248	1	201	1	201
TCGA-DK	TCGA-DK	BLCA	72	MALE	WHITE	Stage III		Muscle in	High Grac	2011		-26562	Dead	TUMOR FREE		250						Complete Remission/Response			1	250	0	250	0	250	0	250
TCGA-DK	TCGA-DK	BLCA	74	MALE	WHITE	Stage III		Muscle in	High Grac	2011		-27194	Dead	WITH TUMOR		539		Locoregic	Lymph Node Only		250	Complete Remission/Response			1	539	1	539	1	250	1	250
TCGA-DK	TCGA-DK	BLCA	68	MALE	WHITE	Stage II		Muscle in	High Grac	2008		-25098	Alive	TUMOR F	1529							Complete Remission/Response			0	1529	0	1529	0	1529	0	1529
TCGA-DK	TCGA-DK	BLCA	62	MALE	WHITE	Stage III		Muscle in	High Grac	2011		-22786	Alive	TUMOR F	648							Complete Remission/Response			0	648	0	648	0	648	0	648
TCGA-DK	TCGA-DK	BLCA	58	MALE	WHITE	Stage II		Muscle in	High Grac	2011		-21369	Dead	WITH TUMOR		706		Distant M	Lung		462	Complete Remission/Response			1	706	1	706	1	462	1	462
TCGA-DK	TCGA-DK	BLCA	60	MALE	WHITE	Stage II		Muscle in	High Grac	2011		-22237	Dead	WITH TUMOR		294		Locoregic	Lymph Node Only		212	Stable Disease			1	294	1	294			1	212
TCGA-DK	TCGA-DK	BLCA	57	MALE	WHITE	Stage III		Muscle in	High Grac	2011		-20909	Alive	TUMOR F	633							Complete Remission/Response			0	633	0	633	0	633	0	633
TCGA-DK	TCGA-DK	BLCA	67	FEMALE	WHITE	Stage III		Muscle in	High Grac	2011		-24553	Dead	WITH TUMOR		321		Locoregic	Bladder		167	Progressive Disease			1	321	1	321			1	167
TCGA-DK	TCGA-DK	BLCA	67	FEMALE	WHITE	Stage III		Muscle in	High Grac	2001		-24536	Alive	TUMOR F	4967							Complete Remission/Response			0	4967	0	4967	0	4967	0	4967
TCGA-DK	TCGA-DK	BLCA	78	FEMALE	WHITE	Stage III		Muscle in	High Grac	2009		-28757	Alive	TUMOR F	2009							Complete Remission/Response			0	2009	0	2009	0	2009	0	2009
TCGA-DK	TCGA-DK	BLCA	85	MALE	WHITE	Stage IV		Muscle in	High Grac	2010		-31160	Dead	WITH TUMOR		547		Distant M	Liver		533	Complete Remission/Response			1	547	1	547			1	533
TCGA-DK	TCGA-DK	BLCA	82	FEMALE	ASIAN	Stage II		Muscle in	High Grac	2008		-30089	Alive	TUMOR F	1952							Complete Remission/Response			0	1952	0	1952	0	1952	0	1952
TCGA-DK	TCGA-DK	BLCA	70	MALE	WHITE	Stage II		Muscle in	High Grac	2010		-25644	Alive	TUMOR F	1621							Complete Remission/Response			0	1621	0	1621	0	1621	0	1621
TCGA-DK	TCGA-DK	BLCA	61	MALE	WHITE	Stage II		Muscle in	High Grac	2007		-22439	Alive	TUMOR F	2330							Complete Remission/Response			0	2330	0	2330	0	2330	0	2330
TCGA-DK	TCGA-DK	BLCA	67	MALE	WHITE	Stage II		Muscle in	High Grac	2007		-24607	Alive	TUMOR F	2049							Complete Remission/Response			0	2049	0	2049	0	2049	0	2049
TCGA-DK	TCGA-DK	BLCA	70	MALE	WHITE	Stage IV		Muscle in	High Grac	2012		-25625	Alive	WITH TUN	477			Distant M	Liver		268	Complete Remission/Response			0	477	0	477			1	268
TCGA-DK	TCGA-DK	BLCA	45	MALE	WHITE	Stage IV		Muscle in	High Grac	2010		-16453	Alive	TUMOR F	1542							Complete Remission/Response			0	1542	0	1542			0	1542
TCGA-DK	TCGA-DK	BLCA	57	MALE	WHITE	Stage II		Muscle in	High Grac	2011		-21007	Alive	TUMOR F	1115							Complete Remission/Response			0	1115	0	1115	0	1115	0	1115
TCGA-DK	TCGA-DK	BLCA	48	MALE	WHITE	Stage IV		Muscle in	High Grac	2010		-17635	Dead	WITH TUMOR		1163		Distant M	Bone		920	Progressive Disease			1	1163	1	1163			1	920
TCGA-DK	TCGA-DK	BLCA	64	FEMALE	WHITE	Stage II		Muscle in	High Grac	2010		-23574	Alive	TUMOR F	1582							Partial Remission/Response			0	1582	0	1582			0	1582
TCGA-DK	TCGA-DK	BLCA	72	FEMALE	WHITE	Stage II		Muscle in	High Grac	2013		-26560	Alive	TUMOR F	457			Distant M	Bone		16	Partial Remission/Response			0	457	0	457			1	16
TCGA-DK	TCGA-DK	BLCA	61	FEMALE	WHITE	Stage IV		Muscle in	High Grac	2012		-22314	Dead	WITH TUMOR		413		Distant M	Liver		285	Progressive Disease			1	413	1	413			1	285
TCGA-DK	TCGA-DK	BLCA	68	MALE	WHITE	Stage IV		Muscle in	High Grac	2001		-25196	Alive	WITH TUN	5041			New Prim	Other, sp	Colon	4907	Stable Disease			0	5041	0	5041			1	4907
TCGA-DK	TCGA-DK	BLCA	60	MALE	WHITE	Stage III		Muscle in	High Grac	2000		-22027	Alive	TUMOR F	5050			Distant M	Other, sp	soft tissue	539	Complete Remission/Response			0	5050	0	5050	1	539	1	539
TCGA-DK	TCGA-DK	BLCA	63	MALE	WHITE	Stage II		Muscle in	High Grac	2013		-23198	Alive	TUMOR F	572							Complete Remission/Response			0	572	0	572	0	572	0	572
TCGA-DK	TCGA-DK	BLCA	64	MALE	WHITE	Stage II		Muscle in	[Unknown	2013		-23611	Alive	TUMOR F	578							Complete Remission/Response			0	578	0	578	0	578	0	578
TCGA-DK	TCGA-DK	BLCA	78	MALE	WHITE	Stage II		Muscle in	High Grac	2013		-28853	Dead	WITH TUMOR		415		Distant M	Lung		379	Stable Disease			1	415	1	415			1	379
TCGA-DK	TCGA-DK	BLCA	63	MALE	WHITE	Stage II		Muscle in	High Grac	2013		-23208	Alive	TUMOR F	467							Progressive Disease			0	467	0	467			0	467
TCGA-DK	TCGA-DK	BLCA	71	MALE	WHITE	Stage II		Muscle in	High Grac	2013		-25950	Alive	WITH TUN	415							Stable Disease			0	415	0	415			0	415
TCGA-DK	TCGA-DK	BLCA	75	MALE	WHITE	Stage III		Muscle in	High Grac	2010		-27730	Alive	TUMOR F	1708							Complete Remission/Response			0	1708	0	1708	0	1708	0	1708
TCGA-DK	TCGA-DK	BLCA	82	MALE	WHITE	Stage III		Muscle in	High Grac	2013		-30090	Dead	WITH TUMOR		340		Distant M	Bone		328	Stable Disease			1	340	1	340			1	328
TCGA-DK	TCGA-DK	BLCA	64	FEMALE	WHITE	Stage II		Muscle in	High Grac	2013		-23542	Alive	WITH TUN	366							Complete Remission/Response			0	366	0	366	0	366	0	366
TCGA-DK	TCGA-DK	BLCA	61	MALE	WHITE	Stage II		Muscle in	High Grac	2013		-22365	Alive	TUMOR F	618			Distant M	Other, sp	Pelvis	467	Stable Disease			0	618	0	618			1	467
TCGA-E5-	TCGA-E5-	BLCA	61	FEMALE	WHITE	Stage IV		Muscle in	High Grac	2008		-22308	Alive	TUMOR F	1326							Complete Remission/Response			0	1326	0	1326			0	1326
TCGA-E5-	TCGA-E5-	BLCA	64	MALE	WHITE	Stage IV		Muscle in	High Grac	2010		-23566	Dead	WITH TUMOR		467		Locoregic	Other, sp	soft tissue	435	Progressive Disease			1	467	1	467			1	435
TCGA-E5-	TCGA-E5-	BLCA	72	MALE	[Unknown	Stage II		Muscle in	High Grac	2011		-26305	Alive	WITH TUN	1181			Distant M	Bone		705	Complete Remission/Response			0	1181	0	1181	1	705	1	705
TCGA-E7-	TCGA-E7-	BLCA	70	MALE	WHITE	Stage II		Muscle in	Low Grad	2011		-25584	Dead	WITH TUMOR		904		Distant M	Bone		369	Complete Remission/Response			1	904	1	904	1	369	1	369
TCGA-E7-	TCGA-E7-	BLCA	57	MALE	WHITE	Stage II		Muscle in	Low Grad	2012		-20917	Alive	TUMOR F	163							Complete Remission/Response			0	163	0	163	0	163	0	163
TCGA-E7-	TCGA-E7-	BLCA	56	MALE	ASIAN	Stage II		Muscle in	High Grac	2012		-20723	Dead	WITH TUMOR		674		Distant M	Bone		620	Complete Remission/Response			1	674	1	674	1	620	1	620
TCGA-E7-	TCGA-E7-	BLCA	66	MALE	ASIAN	Stage II		Muscle in	High Grac	2012		-24279	Dead	TUMOR FREE		68						Complete Remission/Response			1	68	0	68	0	68	0	68
TCGA-E7-	TCGA-E7-	BLCA	72	MALE	WHITE	Stage II		Muscle in	High Grac	2012		-26427	Alive	TUMOR F	508							Complete Remission/Response			0	508	0	508	0	508	0	508
TCGA-E7-	TCGA-E7-	BLCA	66	MALE	WHITE	Stage II		Muscle in	High Grac	2012		-24206	Dead	TUMOR FREE		778						Complete Remission/Response			1	778	0	778	0	778	0	778
TCGA-E7-	TCGA-E7-	BLCA	78	FEMALE	ASIAN	Stage II		Muscle in	High Grac	2012		-28714	Alive	TUMOR F	17										0	17	0	17			0	17
TCGA-E7-	TCGA-E7-	BLCA	67	MALE	ASIAN	Stage II		Muscle in	Low Grad	2012		-24528	Alive	TUMOR F	20										0	20	0	20			0	20
TCGA-E7-	TCGA-E7-	BLCA	81	MALE	ASIAN	Stage II		Muscle in	Low Grad	2012		-29613	Alive	TUMOR F	820							Complete Remission/Response			0	820	0	820	0	820	0	820
TCGA-E7-	TCGA-E7-	BLCA	55	MALE	ASIAN	Stage III		Muscle in	Low Grad	2012		-20169	Alive	TUMOR F	798							Complete Remission/Response			0	798	0	798	0	798	0	798
TCGA-E7-	TCGA-E7-	BLCA	66	MALE	WHITE	Stage IV		Muscle in	High Grac	2012		-24180	Alive		129							[Unknown]			0	129	0	129			0	129
TCGA-E7-	TCGA-E7-	BLCA	75	MALE	ASIAN	Stage II		Muscle in	High Grac	2013		-27670	Alive	TUMOR F	731							Complete Remission/Response			0	731	0	731	0	731	0	731
TCGA-E7-	TCGA-E7-	BLCA	37	MALE	ASIAN	Stage II		Muscle in	Low Grad	2013		-13867	Alive	TUMOR F	750							Complete Remission/Response			0	750	0	750	0	750	0	750
TCGA-E7-	TCGA-E7-	BLCA	73	MALE	WHITE	Stage III		Muscle in	Low Grad	2013		-27000	Alive	TUMOR F	28							Complete Remission/Response			0	28	0	28	0	28	0	28
TCGA-E7-	TCGA-E7-	BLCA	44	MALE	WHITE	Stage IV		Muscle in	High Grac	2013		-16145	Alive	WITH TUN	37							Stable Disease			0	37	0	37			0	37
TCGA-E7-	TCGA-E7-	BLCA	63	MALE	ASIAN	Stage III		Muscle in	High Grac	2013		-23057	Alive	TUMOR F	416			Distant M	Bone		174	Complete Remission/Response			0	416	0	416	1	174	1	174
TCGA-E7-	TCGA-E7-	BLCA	66	MALE	ASIAN	Stage III		Muscle in	High Grac	2013		-24466	Alive	WITH TUN	428			Locoregic	Bladder		190	Progressive Disease			0	428	0	428			1	190
TCGA-E7-	TCGA-E7-	BLCA	64	MALE	ASIAN	Stage III		Muscle in	High Grac	2013		-23497	Alive	TUMOR F	394							Complete Remission/Response			0	394	0	394	0	394	0	394
TCGA-E7-	TCGA-E7-	BLCA	52	MALE	ASIAN	Stage II		Muscle in	Low Grad	2013		-19063	Alive	TUMOR F	466							Complete Remission/Response			0	466	0	466	0	466	0	466
TCGA-E7-	TCGA-E7-	BLCA	59	MALE	ASIAN	Stage II		Muscle in	High Grac	2013		-21629	Alive	TUMOR F	13							Complete Remission/Response			0	13	0	13	0	13	0	13
TCGA-E7-	TCGA-E7-	BLCA	73	MALE	ASIAN	Stage II		Muscle in	High Grac	2013		-26956	Dead	TUMOR FREE		437						Complete Remission/Response			1	437	0	437	0	437	0	437
TCGA-E7-	TCGA-E7-	BLCA	60	FEMALE	WHITE	Stage IV		Muscle in	High Grac	2013		-22249	Dead	WITH TUMOR		246						Stable Disease			1	246	1	246			1	246
TCGA-FD	TCGA-FD	BLCA	74	FEMALE	WHITE	Stage III		Muscle in	High Grac	2011		-27068	Dead	TUMOR FREE		974						Complete Remission/Response			1	974	0	974	0	974	0	974
TCGA-FD	TCGA-FD	BLCA	55	FEMALE	BLACK OI	Stage III		Muscle in	High Grac	2011		-20379	Dead	WITH TUMOR		510		Locoregic	Other, sp	Left poste	359	Complete Remission/Response			1	510	1	510	1	359	1	359
TCGA-FD	TCGA-FD	BLCA	86	MALE	BLACK OI	Stage IV		Muscle in	High Grac	2011		-31442	Dead	WITH TUMOR		272						Progressive Disease			1	272	1	272			1	272
TCGA-FD	TCGA-FD	BLCA	75	MALE	WHITE	Stage II		Muscle in	High Grac	2011		-27541	Dead	TUMOR FREE		1005						Complete Remission/Response			1	1005	0	1005	0	1005	0	1005
TCGA-FD	TCGA-FD	BLCA	66	MALE	BLACK OI	Stage III		Muscle in	High Grac	2011		-24245	Dead	WITH TUMOR		122		Distant M	Lymph Node Only		110	Progressive Disease			1	122	1	122			1	110
TCGA-FD	TCGA-FD	BLCA	56	MALE	WHITE	Stage II		Muscle in	High Grac	2011		-20555	Alive	TUMOR F	384							Complete Remission/Response			0	384	0	384	0	384	0	384
TCGA-FD	TCGA-FD	BLCA	69	MALE	BLACK OI	Stage II		Muscle in	High Grac	2011		-25342	Dead	WITH TUMOR		685		Locoregic	Other, sp	Pelvis	346				1	685	1	685			1	346
TCGA-FD	TCGA-FD	BLCA	43	FEMALE	BLACK OI	Stage II		Muscle in	High Grac	2011		-15758	Alive	TUMOR F	851							Complete Remission/Response			0	851	0	851	0	851	0	851
TCGA-FD	TCGA-FD	BLCA	60	MALE	WHITE	Stage II		Muscle in	High Grac	2009		-21936	Alive	TUMOR F	1845			New Prim	Lung		1338	Complete Remission/Response			0	1845	0	1845	0	1845	1	1338
TCGA-FD	TCGA-FD	BLCA	59	MALE	WHITE	Stage IV		Muscle in	High Grac	2010		-21615	Dead	WITH TUMOR		739		Distant M	Lymph Node Only		281				1	739	1	739			1	281
TCGA-FD	TCGA-FD	BLCA	60	MALE	WHITE	Stage IV		Muscle in	High Grac	2011		-22020	Dead	WITH TUMOR		712		Distant M	Lung		260	Progressive Disease			1	712	1	712			1	260
TCGA-FD	TCGA-FD	BLCA	70	MALE	WHITE	Stage IV		Muscle in	High Grac	2011		-25852	Dead	WITH TUMOR		547		Distant M	Lymph Node Only		144	[Unknown]			1	547	1	547			1	144
TCGA-FD	TCGA-FD	BLCA	79	MALE	WHITE	Stage III		Muscle in	High Grac	2011		-29169	Alive	TUMOR F	887			Distant M	Lung		110	Progressive Disease			0	887	0	887			1	110
TCGA-FD	TCGA-FD	BLCA	68	MALE	WHITE	Stage IV		Muscle in	High Grac	2012		-25147	Dead	WITH TUMOR		168		Locoregic	Renal Pelvis		143	Progressive Disease			1	168	1	168			1	143
TCGA-FD	TCGA-FD	BLCA	60	MALE	WHITE	Stage III		Muscle in	High Grac	2012		-22005	Alive	TUMOR F	783							Complete Remission/Response			0	783	0	783	0	783	0	783
TCGA-FD	TCGA-FD	BLCA	62	MALE	WHITE	Stage IV		Muscle in	High Grac	2008		-22687	Dead	WITH TUMOR		1423		Locoregic	Other, sp	sigmoid c	991	Stable Disease			1	1423	1	1423			1	991
TCGA-FD	TCGA-FD	BLCA	68	MALE	WHITE	Stage IV		Muscle in	High Grac	2010		-24979	Dead	WITH TUMOR		602		Locoregic	Other, sp	pelvis	372	[Unknown]			1	602	1	602			1	372

TCGA-FD	TCGA-FD	BLCA	66	MALE	WHITE	Stage IV	Muscle in	High Grac	2012		-24408	Dead	WITH TUMOR		391	Locoregic	Other, sp	local & re	220	[Unknown]		1	391	1	391			1	220
TCGA-FD	TCGA-FD	BLCA	76	MALE	WHITE	Stage III	Muscle in	High Grac	2011		-27833	Alive	TUMOR F	699								0	699	0	699			0	699
TCGA-FD	TCGA-FD	BLCA	74	MALE	BLACK OI	Stage II	Muscle in	High Grac	2012		-27201	Alive	TUMOR F	832						Complete Remission/Response		0	832	0	832	0	832	0	832
TCGA-FD	TCGA-FD	BLCA	71	FEMALE	WHITE	Stage III	Muscle in	High Grac	2012		-25993	Alive	TUMOR F	455						Complete Remission/Response		0	455	0	455	0	455	0	455
TCGA-FD	TCGA-FD	BLCA	70	MALE	WHITE	Stage IV	Muscle in	High Grac	2012		-25841	Alive	WITH TUI	636		Distant M	Bone		497	Complete Remission/Response		0	636	0	636			1	497
TCGA-FD	TCGA-FD	BLCA	84	MALE	WHITE	Stage II	Muscle in	High Grac	2012		-30764	Alive	TUMOR F	110						Complete Remission/Response		0	110	0	110	0	110	0	110
TCGA-FD	TCGA-FD	BLCA	65	MALE	WHITE	Stage III	Muscle in	High Grac	2012		-23889	Dead	TUMOR FREE		474					Complete Remission/Response		1	474	0	474	0	474	0	474
TCGA-FD	TCGA-FD	BLCA	57	MALE	WHITE	Stage II	Muscle in	High Grac	2012		-20964	Alive	TUMOR F	813						Complete Remission/Response		0	813	0	813	0	813	0	813
TCGA-FD	TCGA-FD	BLCA	68	MALE	WHITE	Stage III	Muscle in	High Grac	2009		-24963	Alive	TUMOR F	1639						Complete Remission/Response		0	1639	0	1639	0	1639	0	1639
TCGA-FD	TCGA-FD	BLCA	84	MALE	WHITE	Stage III	Muscle in	High Grac	2011		-30686	Dead	TUMOR FREE		328							1	328	0	328			0	328
TCGA-FD	TCGA-FD	BLCA	76	FEMALE	BLACK OI	Stage II	Muscle in	High Grac	2012		-27882	Alive	TUMOR F	588						Complete Remission/Response		0	588	0	588	0	588	0	588
TCGA-FD	TCGA-FD	BLCA	47	FEMALE	WHITE	Stage III	Muscle in	High Grac	2012		-17501	Dead	WITH TUMOR		163	Locoregic	Other, sp	Peritonea	134	Progressive Disease		1	163	1	163			1	134
TCGA-FD	TCGA-FD	BLCA	82	MALE	WHITE	Stage IV	Muscle in	High Grac	2012		-30276	Dead	WITH TUMOR		173	Locoregic	Lymph Node Only		99	Progressive Disease		1	173	1	173			1	99
TCGA-FD	TCGA-FD	BLCA	63	FEMALE	WHITE	Stage III	Muscle in	High Grac	2012		-23080	Alive	TUMOR F	251						Complete Remission/Response		0	251	0	251	0	251	0	251
TCGA-FD	TCGA-FD	BLCA	77	FEMALE	WHITE	Stage IV	Muscle in	High Grac	2009		-28451	Dead	WITH TUMOR		835	Locoregic	Lymph Node Only		533			1	835	1	835			1	533
TCGA-FD	TCGA-FD	BLCA	61	MALE	WHITE	Stage IV	Muscle in	High Grac	2012		-22500	Dead	WITH TUMOR		550	New Prim	Lung		27	Complete Remission/Response		1	550	1	550			1	27
TCGA-FD	TCGA-FD	BLCA	61	FEMALE	WHITE	Stage III	Muscle in	High Grac	2009		-22505	Alive	TUMOR F	1792						Complete Remission/Response		0	1792	0	1792	0	1792	0	1792
TCGA-FD	TCGA-FD	BLCA	69	MALE	ASIAN	Stage III	Muscle in	High Grac	2012		-25297	Alive	TUMOR F	82						Complete Remission/Response		0	82	0	82	0	82	0	82
TCGA-FD	TCGA-FD	BLCA	74	MALE	WHITE	Stage IV	Muscle in	High Grac	2012		-27310	Dead	TUMOR FREE		216					[Unknown]		1	216	0	216			0	216
TCGA-FD	TCGA-FD	BLCA	76	MALE	WHITE	Stage II	Muscle in	High Grac	2012		-27843	Dead	WITH TUMOR		191							1	191	1	191			1	191
TCGA-FD	TCGA-FD	BLCA	60	FEMALE	BLACK OI	Stage III	Muscle in	High Grac	2013		-22000	Dead	WITH TUMOR		406	Distant M	Other, sp	sigmoid c	306	Progressive Disease		1	406	1	406			1	306
TCGA-FD	TCGA-FD	BLCA	58	MALE	WHITE	Stage IV	Muscle in	High Grac	2009		-21196	Alive	TUMOR F	1912						Complete Remission/Response		0	1912	0	1912			0	1912
TCGA-FD	TCGA-FD	BLCA	82	MALE	WHITE	Stage III	Muscle in	High Grac	2012		-30185	Alive	TUMOR F	572						Complete Remission/Response		0	572	0	572	0	572	0	572
TCGA-FD	TCGA-FD	BLCA	79	FEMALE	WHITE	Stage III	Muscle in	High Grac	2013		-28963	Alive	TUMOR F	187						Complete Remission/Response		0	187	0	187	0	187	0	187
TCGA-FD	TCGA-FD	BLCA	77	MALE	WHITE	Stage III	Muscle in	High Grac	2012		-28328	Dead	TUMOR FREE		386					Complete Remission/Response		1	386	0	386	0	386	0	386
TCGA-FD	TCGA-FD	BLCA	54	MALE	WHITE	Stage IV	Muscle in	High Grac	2013		-19874	Alive	TUMOR F	376		Distant M	Other, sp	sacrum	321	[Unknown]		0	376	0	376			1	321
TCGA-FD	TCGA-FD	BLCA	80	FEMALE	WHITE	Stage IV	Muscle in	High Grac	2013		-29317	Dead	TUMOR FREE		69					Progressive Disease		1	69	0	69			0	69
TCGA-FD	TCGA-FD	BLCA	73	MALE	WHITE	Stage IV	Muscle in	High Grac	2013		-26893	Dead	TUMOR FREE		93							1	93	0	93			0	93
TCGA-FD	TCGA-FD	BLCA	63	MALE	WHITE	Stage IV	Muscle in	High Grac	2013		-23236	Dead	WITH TUMOR		131	Distant M	Lung		126	Complete Remission/Response		1	131	1	131			1	126
TCGA-FD	TCGA-FD	BLCA	73	MALE	WHITE	Stage IV	Muscle in	High Grac	2013		-26874	Dead	TUMOR FREE		294					[Unknown]		1	294	0	294			0	294
TCGA-FD	TCGA-FD	BLCA	60	MALE	WHITE	Stage III	Muscle in	High Grac	2013		-21942	Alive	TUMOR F	330						Complete Remission/Response		0	330	0	330	0	330	0	330
TCGA-FJ-	TCGA-FJ-	BLCA	76	MALE	WHITE	Stage IV	Muscle in	High Grac	2010		-28052	Alive	TUMOR F	945						Complete Remission/Response		0	945	0	945			0	945
TCGA-FJ-	TCGA-FJ-	BLCA	72	MALE	WHITE		Muscle in	High Grac	2012		-26515	Dead	TUMOR FREE		385					Complete Remission/Response		1	385	0	385	0	385	0	385
TCGA-FJ-	TCGA-FJ-	BLCA	65	MALE	WHITE	Stage IV	Muscle in	High Grac	2012		-23866	Dead	WITH TUMOR		324	Distant M	Bone		152	Progressive Disease		1	324	1	324			1	152
TCGA-FJ-	TCGA-FJ-	BLCA	73	MALE	WHITE	Stage III	Muscle in	High Grac	2012		-26825	Alive	TUMOR F	524						Complete Remission/Response		0	524	0	524	0	524	0	524
TCGA-FJ-	TCGA-FJ-	BLCA	49	MALE	WHITE	Stage III	Muscle in	High Grac	2013		-18051	Dead	WITH TUMOR		272	Locoregic	Other, sp	Pelvic	106	Progressive Disease		1	272	1	272			1	106
TCGA-FT	TCGA-FT	BLCA	80	FEMALE	WHITE	Stage III	Muscle in	High Grac	2011		-29293	Dead	TUMOR FREE		99							1	99	0	99			0	99
TCGA-FT	TCGA-FT	BLCA	76	MALE	WHITE	Stage IV	Muscle in	High Grac	2013		-28082	Alive	TUMOR F	337						Complete Remission/Response		0	337	0	337			0	337
TCGA-G2	TCGA-G2	BLCA	58	FEMALE	BLACK OI	Stage II	Muscle in	High Grac	2010		-21215	Dead	WITH TUMOR		696	Distant M	Other, sp	small bow	679	Partial Remission/Response		1	696	1	696			1	679
TCGA-G2	TCGA-G2	BLCA	50	MALE	WHITE	Stage II	Muscle in	High Grac	2007		-18467	Alive	TUMOR F	1884						Partial Remission/Response		0	1884	0	1884			0	1884
TCGA-G2	TCGA-G2	BLCA	56	FEMALE	WHITE	Stage II	Muscle in	High Grac	2009		-20649	Alive	TUMOR F	1460		Distant M	Lung		910	Complete Remission/Response		0	1460	0	1460	1	910	1	910
TCGA-G2	TCGA-G2	BLCA	57	MALE	WHITE	Stage II	Muscle in	High Grac	2010		-20995	Alive	WITH TUI	485		Distant M	Renal Pelvis		485	Stable Disease		0	485	0	485			1	485
TCGA-G2	TCGA-G2	BLCA	77	MALE	WHITE	Stage II	Muscle in	High Grac	2009		-28370	Dead	WITH TUMOR		819	Distant M	Bone		105	Stable Disease		1	819	1	819			1	105
TCGA-G2	TCGA-G2	BLCA	69	MALE	WHITE	Stage III	Muscle in	High Grac	2009		-25440	Dead	TUMOR FREE		1804					Complete Remission/Response		1	1804	0	1804	0	1804	0	1804
TCGA-G2	TCGA-G2	BLCA	85	MALE	WHITE	Stage II	Muscle in	High Grac	2009		-31293	Dead	WITH TUMOR		1004	New Prim	Other, specify		897	Progressive Disease		1	1004	1	1004			1	897
TCGA-G2	TCGA-G2	BLCA	66	MALE	BLACK OI	Stage II	Muscle in	High Grac	2008		-24149	Dead	WITH TUMOR		220					Progressive Disease		1	220	1	220			1	220
TCGA-G2	TCGA-G2	BLCA	51	MALE	WHITE	Stage II	Muscle in	High Grac	2007		-18983	Dead	WITH TUMOR		612					Progressive Disease		1	612	1	612			1	612
TCGA-G2	TCGA-G2	BLCA	66	MALE	ASIAN	Stage II	Muscle in	High Grac	2012		-24112	Alive	WITH TUI	536						Stable Disease		0	536	0	536			0	536
TCGA-G2	TCGA-G2	BLCA	75	FEMALE	WHITE	Stage II	Muscle in	High Grac	2009		-27646	Alive	TUMOR F	2008						Complete Remission/Response		0	2008	0	2008	0	2008	0	2008
TCGA-G2	TCGA-G2	BLCA	66	MALE	WHITE	Stage IV	Muscle in	High Grac	2009		-24267	Dead	WITH TUMOR		211					[Unknown]		1	211	1	211			1	211
TCGA-G2	TCGA-G2	BLCA	60	MALE	WHITE	Stage IV	Muscle in	High Grac	2009		-22205	Alive	TUMOR F	2139						Complete Remission/Response		0	2139	0	2139			0	2139
TCGA-G2	TCGA-G2	BLCA	77	MALE	WHITE	Stage IV	Muscle in	High Grac	2012		-28457	Alive	TUMOR F	893						Partial Remission/Response		0	893	0	893			0	893
TCGA-GC	TCGA-GC	BLCA	70	MALE	WHITE	Stage II	Muscle in	High Grac	2010		-25609	Dead	WITH TUMOR		651					Progressive Disease		1	651	1	651			1	651
TCGA-GC	TCGA-GC	BLCA	70	MALE	WHITE	Stage II	Muscle in	High Grac	2010		-25609	Dead	WITH TUMOR		651					Progressive Disease		1	651	1	651			1	651
TCGA-GC	TCGA-GC	BLCA	45	MALE	WHITE	Stage III	Muscle in	High Grac	2011		-16504	Dead	WITH TUMOR		630	Locoregic	Bladder		546	Progressive Disease		1	630	1	630			1	546
TCGA-GC	TCGA-GC	BLCA	79	MALE	WHITE	Stage II	Muscle in	High Grac	2011		-29065	Alive	TUMOR F	481						Complete Remission/Response		0	481	0	481	0	481	0	481
TCGA-GC	TCGA-GC	BLCA	54	MALE	WHITE	Stage III	Muscle in	High Grac	2011		-20021	Alive	TUMOR F	582						Complete Remission/Response		0	582	0	582	0	582	0	582
TCGA-GC	TCGA-GC	BLCA	59	MALE	WHITE	Stage II	Muscle in	High Grac	2011		-21668	Alive	TUMOR F	484						Complete Remission/Response		0	484	0	484	0	484	0	484
TCGA-GC	TCGA-GC	BLCA	83	FEMALE	WHITE	Stage III	Muscle in	High Grac	2011		-30529	Alive	TUMOR F	428						Complete Remission/Response		0	428	0	428	0	428	0	428
TCGA-GC	TCGA-GC	BLCA	80	FEMALE	WHITE	Stage III	Muscle in	High Grac	2011		-29295	Alive	TUMOR F	540						Complete Remission/Response		0	540	0	540	0	540	0	540
TCGA-GC	TCGA-GC	BLCA	80	FEMALE	WHITE	Stage III	Muscle in	High Grac	2011		-29295	Alive	TUMOR F	540						Complete Remission/Response		0	540	0	540	0	540	0	540
TCGA-GC	TCGA-GC	BLCA	61	MALE	WHITE	Stage IV	Muscle in	High Grac	2012		-22515	Alive	TUMOR F	758						Complete Remission/Response		0	758	0	758			0	758
TCGA-GC	TCGA-GC	BLCA	71	MALE	WHITE	Stage III	Muscle in	High Grac	2012		-25964	Alive	TUMOR F	15						[Unknown]		0	15	0	15			0	15
TCGA-GC	TCGA-GC	BLCA	90	FEMALE	WHITE	Stage II	Muscle in	High Grac	2012		-32873	Dead	TUMOR FREE		272					Complete Remission/Response		1	272	0	272	0	272	0	272
TCGA-GC	TCGA-GC	BLCA	90	FEMALE	BLACK OI	Stage IV	Muscle in	High Grac	2013		-32873	Alive	TUMOR F	423						Complete Remission/Response		0	423	0	423			0	423
TCGA-GC	TCGA-GC	BLCA	90	FEMALE	BLACK OI	Stage IV	Muscle in	High Grac	2013		-32873	Alive	TUMOR F	423						Complete Remission/Response		0	423	0	423			0	423
TCGA-GD	TCGA-GD	BLCA	53	FEMALE	WHITE	Stage IV	Muscle in	High Grac	2011		-19498	Alive	WITH TUI	812		Metastati	Lung		214	Progressive Disease		0	812	0	812			1	214
TCGA-GD	TCGA-GD	BLCA	53	FEMALE	WHITE	Stage IV	Muscle in	High Grac	2011		-19498	Alive	WITH TUI	812		Metastati	Lung		214	Progressive Disease		0	812	0	812			1	214
TCGA-GD	TCGA-GD	BLCA	84	FEMALE	WHITE	Stage IV	Muscle in	High Grac	2011		-30956	Alive		64						[Unknown]		0	64	0	64			0	64
TCGA-GD	TCGA-GD	BLCA	84	FEMALE	WHITE	Stage IV	Muscle in	High Grac	2011		-30956	Alive		64						[Unknown]		0	64	0	64			0	64
TCGA-GD	TCGA-GD	BLCA	48	MALE	WHITE	Stage IV	Muscle in	High Grac	2011		-17682	Alive		95						[Unknown]		0	95	0	95			0	95
TCGA-GD	TCGA-GD	BLCA	48	MALE	WHITE	Stage IV	Muscle in	High Grac	2011		-17682	Alive		95						[Unknown]		0	95	0	95			0	95
TCGA-GD	TCGA-GD	BLCA	54	FEMALE	WHITE	Stage II	Muscle in	High Grac	2011		-19776	Alive	WITH TUI	638		Locoregic	Bladder		223	Progressive Disease		0	638	0	638			1	223
TCGA-GD	TCGA-GD	BLCA	64	MALE	WHITE	Stage III	Muscle in	High Grac	2012		-23631	Alive	TUMOR F	67						Complete Remission/Response		0	67	0	67	0	67	0	67
TCGA-GD	TCGA-GD	BLCA	86	FEMALE	WHITE	Stage II	Muscle in	High Grac	2013		-31571	Alive	TUMOR F	224						Complete Remission/Response		0	224	0	224	0	224	0	224
TCGA-GU	TCGA-GU	BLCA	72	MALE	WHITE	Stage IV	Muscle in	High Grac	2012		-26546	Dead	TUMOR FREE		332					Stable Disease		1	332	0	332			0	332
TCGA-GU	TCGA-GU	BLCA	67	MALE	WHITE	Stage III	Muscle in	High Grac	2012		-24534	Dead	TUMOR FREE		344					Complete Remission/Response		1	344	0	344	0	344	0	344
TCGA-GU	TCGA-GU	BLCA	68	MALE	WHITE	Stage IV	Muscle in	High Grac	2012		-25134	Dead	WITH TUMOR		577	Distant M	Lung		91	Progressive Disease		1	577	1	577			1	91
TCGA-GU	TCGA-GU	BLCA	87	MALE	WHITE	Stage IV	Muscle in	High Grac	2012		-31812	Dead	WITH TUMOR		232					Progressive Disease		1	232	1	232			1	232
TCGA-GU	TCGA-GU	BLCA	72	MALE	WHITE	Stage III	Muscle in	High Grac	2012		-26527	Alive	WITH TUI	997		Distant M	Lung		303	Stable Disease		0	997	0	997			1	303
TCGA-GU	TCGA-GU	BLCA	66	MALE	WHITE	Stage II	Muscle in	High Grac	2012		-24343	Alive	TUMOR F	610						Complete Remission/Response		0	610	0	610	0	610	0	610
TCGA-GU	TCGA-GU	BLCA	62	MALE	WHITE	Stage II	Muscle in	High Grac	2013		-22774	Alive	WITH TUI	480						Progressive Disease		0	480	0	480			0	480
TCGA-GU	TCGA-GU	BLCA	81	MALE	WHITE	Stage IV	Muscle in	High Grac	2012		-29739	Dead	WITH TUMOR		144					Progressive Disease		1	144	1	144			1	144
TCGA-GU	TCGA-GU	BLCA	75	MALE	WHITE	Stage IV	Muscle in	High Grac	2013		-27684	Dead	WITH TUMOR		324					Progressive Disease		1	324	1	324			1	324
TCGA-GU	TCGA-GU	BLCA	74	MALE	WHITE	Stage IV	Muscle in	High Grac	2012		-27049	Alive	WITH TUI	1003		New Prim	Urethra		596	Progressive Disease		0	1003	0	1003			1	596
TCGA-GU	TCGA-GU	BLCA	68	MALE	WHITE	Stage III	Muscle in	High Grac	2013		-25185	Dead	WITH TUMOR		213					Stable Disease		1	213	1	213			1	213
TCGA-GV	TCGA-GV	BLCA	66	MALE	WHITE	Stage IV	Muscle in	High Grac	2011		-24243	Dead	WITH TUMOR		434	Locoregic	Bladder		134	Partial Remission/Response		1	434	1	434			1	134
TCGA-GV	TCGA-GV	BLCA	74	MALE	WHITE	Stage II	Muscle in	High Grac	2011		-27091	Alive	WITH TUI	649		Distant M	Bone		271	Stable Disease		0	649	0	649			1	271
TCGA-GV	TCGA-GV	BLCA	59	MALE	WHITE	Stage III	Muscle in	High Grac	2011		-21888	Alive	TUMOR F	581						Complete Remission/Response		0	581	0	581	0	581	0	581
TCGA-GV	TCGA-GV	BLCA	55	MALE	WHITE	Stage IV	Muscle in	High Grac	2011		-20420	Alive	WITH TUI	603						Stable Disease		0	603	0	603			0	603
TCGA-GV	TCGA-GV	BLCA	79	MALE	WHITE	Stage IV	Muscle in	High Grac	2011		-28890	Dead	TUMOR FREE		617					Stable Disease		1	617	0	617			0	617
TCGA-GV	TCGA-GV	BLCA	65	MALE	WHITE	Stage IV	Muscle in	High Grac	2011		-23847	Dead	WITH TUMOR			Distant M	Other, sp	Brain	84			1		1				1	84
TCGA-GV	TCGA-GV	BLCA	67	MALE	WHITE	Stage II	Muscle in	High Grac	2011		-24681	Dead	TUMOR FREE		258	Locoregic	Bladder		125	Stable Disease		1	258	0	258			1	125
TCGA-GV	TCGA-GV	BLCA	47	MALE	WHITE	Stage III	Muscle in	High Grac	2011		-17383	Alive	WITH TUI	1110		Distant M	Other, sp	Abdomina	768	Complete Remission/Response		0	1110	0	1110	1	768	1	768
TCGA-GV	TCGA-GV	BLCA	56	FEMALE	WHITE	Stage IV	Muscle in	High Grac	2012		-20802	Alive	WITH TUI	832						Stable Disease		0	832	0	832			0	832
TCGA-GV	TCGA-GV	BLCA	75	MALE	WHITE	Stage II	Muscle in	High Grac	2012		-27569	Dead	WITH TUMOR		261	Distant M	Lung		219	Stable Disease		1	261	1	261			1	219
TCGA-GV	TCGA-GV	BLCA	77	MALE	WHITE	Stage II	Muscle in	High Grac	2012		-28204	Alive	TUMOR F	580						Complete Remission/Response		0	580	0	580	0	580	0	580
TCGA-GV	TCGA-GV	BLCA	54	MALE	WHITE	Stage II	Muscle in	High Grac	2012		-19871	Alive	TUMOR F	691						Complete Remission/Response		0	691	0	691	0	691	0	691
TCGA-H4	TCGA-H4	BLCA	53	MALE	WHITE	Stage III	Muscle in	High Grac	2010		-19630	Alive	TUMOR F	46						Complete Remission/Response		0	46	0	46	0	46	0	46
TCGA-H4	TCGA-H4	BLCA	64	FEMALE	BLACK OI	Stage IV	Muscle in	High Grac	2010		-23538	Alive	WITH TUI	590		Distant M	Lung		318	Complete Remission/Response		0	590	0	590			1	318
TCGA-HC	TCGA-HC	BLCA	69	MALE	[Not Evaluated]		Muscle in	[Unknowr	2007			Alive		1174						[Unknown]		0	1174	0	1174			0	1174
TCGA-HC	TCGA-HC	BLCA	52	FEMALE	[Not Evalu	Stage III	Muscle in	High Grade			-19016	Alive	TUMOR F	1947		Locoregic	Lymph Node Only		907	Complete Remission/Response		0	1947	0	1947	1	907	1	907
TCGA-HC	TCGA-HC	BLCA	78	MALE	[Not Evalu	Stage IV	Muscle in	High Grade			-28555	Dead	WITH TUMOR		274	Distant M	Lymph Node Only		84	Progressive Disease		1	274	1	274			1	84
TCGA-HC	TCGA-HC	BLCA	57	MALE	[Not Evalu	Stage III	Muscle in	High Grade			-20647	Dead	WITH TUMOR		370	Distant M	Renal Pelvis		211	Complete Remission/Response		1	370	1	370	1	211	1	211
TCGA-K4	TCGA-K4	BLCA	66	MALE	WHITE	Stage III	Muscle in	High Grac	2011		-24124	Alive	TUMOR F	761						Complete Remission/Response		0	761	0	761	0	761	0	761
TCGA-K4	TCGA-K4	BLCA	87	MALE	WHITE	Stage III	Muscle in	High Grac	2012		-32134	Alive	TUMOR F	105								0	105	0	105			0	105
TCGA-K4	TCGA-K4	BLCA	77	FEMALE	WHITE	Stage II	Muscle in	High Grac	2011		-28444	Alive	TUMOR F	646								0	646	0	646			0	646
TCGA-K4	TCGA-K4	BLCA	77	FEMALE	WHITE	Stage II	Muscle in	High Grac	2011		-28444	Alive	TUMOR F	646								0	646	0	646			0	646
TCGA-K4	TCGA-K4	BLCA	76	MALE	WHITE	Stage III	Muscle in	High Grac	2012		-27922	Alive		84						Partial Remission/Response		0	84	0	84			0	84
TCGA-K4	TCGA-K4	BLCA	83	MALE	WHITE	Stage II	Muscle in	High Grac	2012		-30655	Dead	WITH TUMOR		278	Distant M	Other, sp	psoas	170	Partial Remission/Response		1	278	1	278			1	170
TCGA-K4	TCGA-K4	BLCA	59	MALE	WHITE	Stage II	Muscle in	High Grac	2012		-21601	Alive	TUMOR F	842						Complete Remission/Response		0	842	0	842	0	842	0	842
TCGA-K4	TCGA-K4	BLCA	59	MALE	WHITE	Stage II	Muscle in	High Grac	2012		-21601	Alive	TUMOR F	842						Complete Remission/Response		0	842	0	842	0	842	0	842
TCGA-K4	TCGA-K4	BLCA	69	MALE	WHITE	Stage III	Muscle in	High Grac	2012		-25305	Alive	WITH TUI	276		Distant M	Lung		196	Progressive Disease		0	276	0	276			1	196
TCGA-K4	TCGA-K4	BLCA	67	FEMALE	WHITE	Stage III	Muscle in	High Grac	2012		-24614	Dead	WITH TUMOR		356	Locoregic	Other, sp	ileum	236	Progressive Disease		1	356	1	356			1	236
TCGA-K4	TCGA-K4	BLCA	67	FEMALE	WHITE	Stage III	Muscle in	High Grac	2012		-24614	Dead	WITH TUMOR		356	Locoregic	Other, sp	ileum	236	Progressive Disease		1	356	1	356			1	236
TCGA-K4	TCGA-K4	BLCA	75	MALE	WHITE	Stage II	Muscle in	High Grac	2012		-27518	Alive	TUMOR F	539						Complete Remission/Response		0	539	0	539	0	539	0	539
TCGA-K4	TCGA-K4	BLCA	75	FEMALE	WHITE	Stage III	Muscle in	High Grac	2012		-27686	Alive	TUMOR F	55						Complete Remission/Response		0	55	0	55	0	55	0	55
TCGA-K4	TCGA-K4	BLCA	64	MALE	WHITE	Stage IV	Muscle in	High Grac	2013		-23664	Alive	TUMOR F	469						Complete Remission/Response		0	469	0	469			0	469
TCGA-K4	TCGA-K4	BLCA	77	MALE	WHITE	Stage IV	Muscle in	[Unknowr	2013		-28463	Alive	WITH TUI	495		Distant M	Liver		70	Partial Remission/Response		0	495	0	495			1	70
TCGA-K4	TCGA-K4	BLCA	56	MALE	WHITE	Stage III	Muscle in	High Grac	2013		-20683	Alive		359						[Unknown]		0	359	0	359			0	359
TCGA-KQ	TCGA-KQ	BLCA	73	MALE	BLACK OI	Stage III	Muscle in	High Grac	2011		-26837	Alive	TUMOR F	1604						Complete Remission/Response		0	1604	0	1604	0	1604	0	1604
TCGA-KQ	TCGA-KQ	BLCA	84	MALE	[Unknowr	Stage IV	Muscle in	High Grac	2011		-30774	Alive	TUMOR F	1538						Complete Remission/Response		0	1538	0	1538			0	1538
TCGA-KQ	TCGA-KQ	BLCA	76	MALE	WHITE	Stage IV	Muscle in	High Grac	2011		-27816	Alive	WITH TUI	1094		Locoregic	Lymph Node Only		178	Stable Disease		0	1094	0	1094			1	178
TCGA-KQ	TCGA-KQ	BLCA	89	MALE	WHITE	Stage III	Muscle in	High Grac	2009		-32511	Alive		1429		Locoregic	Lymph Node Only		1314	[Unknown]		0	1429	0	1429			1	1314
TCGA-KQ	TCGA-KQ	BLCA	80	FEMALE	WHITE	Stage II	Muscle in	High Grac	2011		-29351	Alive	TUMOR F	1350						Complete Remission/Response		0	1350	0	1350	0	1350	0	1350
TCGA-KQ	TCGA-KQ	BLCA	87	FEMALE	WHITE	Stage III	Muscle in	High Grac	2011		-32041	Alive	TUMOR F	35						Complete Remission/Response		0	35	0	35	0	35	0	35
TCGA-LC	TCGA-LC	BLCA	78	MALE	WHITE	Stage IV	Muscle in	High Grac	2012		-28793	Alive	TUMOR F	466						Complete Remission/Response		0	466	0	466			0	466
TCGA-LT-	TCGA-LT-	BLCA	56	MALE	WHITE	Stage II	Muscle in	High Grac	2013		-20746	Alive	TUMOR F	474						Complete Remission/Response		0	474	0	474	0	474	0	474
TCGA-LT-	TCGA-LT-	BLCA	69	FEMALE	WHITE	Stage II	Muscle in	High Grac	2013		-25498	Alive	TUMOR F	641						Complete Remission/Response		0	641	0	641	0	641	0	641
TCGA-MV	TCGA-MV	BLCA	75	MALE	WHITE	Stage III	Muscle in	High Grac	2012		-27430	Alive	TUMOR F	410						Complete Remission/Response		0	410	0	410	0	410	0	410
TCGA-PC	TCGA-PC	BLCA	70	MALE	WHITE	Stage II	Muscle in	High Grac	2011		-25672	Alive	TUMOR F	372						Complete Remission/Response		0	372	0	372	0	372	0	372
TCGA-PC	TCGA-PC	BLCA	78	FEMALE	WHITE	Stage III	Muscle in	High Grac	2011		-28546	Alive		507						[Unknown]		0	507	0	507			0	507
TCGA-R3	TCGA-R3	BLCA	70	MALE	WHITE	Stage III	Muscle in	High Grac	2012		-25576	Alive	TUMOR F	433						Complete Remission/Response		0	433	0	433	0	433	0	433
TCGA-S5	TCGA-S5	BLCA	83	MALE	WHITE	Stage IV	Muscle in	High Grac	2013		-30594	Dead	WITH TUMOR		56					[Unknown]		1	56	1	56			1	56
TCGA-S5	TCGA-S5	BLCA	46	MALE	BLACK OI	Stage III	Muscle in	High Grac	2013		-16972	Alive	TUMOR F	503						Complete Remission/Response		0	503	0	503	0	503	0	503
TCGA-SY	TCGA-SY	BLCA	82	MALE	WHITE	Stage IV	Muscle in	High Grac	2011		-30205	Dead	TUMOR FREE		1008					Complete Remission/Response		1	1008	0	1008			0	1008
TCGA-SY	TCGA-SY	BLCA	66	MALE	WHITE	Stage III	Muscle in	High Grac	2011		-24260	Alive	TUMOR F	1186						Complete Remission/Response		0	1186	0	1186	0	1186	0	1186
TCGA-UY	TCGA-UY	BLCA	60	MALE	WHITE	Stage IV	Muscle in	High Grade			-22020	Dead			536					Complete Remission/Response		1	536		536			0	536
TCGA-UY	TCGA-UY	BLCA	62	MALE	WHITE	Stage IV	Muscle in	High Grade			-22717	Alive	WITH TUI	1127					355	Progressive Disease		0	1127	0	1127			1	355
TCGA-UY	TCGA-UY	BLCA	81	FEMALE	ASIAN	Stage IV	Muscle in	High Grade			-29706	Dead	WITH TUMOR		690					[Unknown]		1	690	1	690			1	690
TCGA-UY	TCGA-UY	BLCA	80	MALE	WHITE	Stage IV	Muscle in	High Grade			-29132	Dead	WITH TUMOR		2641	Distant M	Renal Pelvis		2472	Complete Remission/Response		1	2641	1	2641			1	2472
TCGA-UY	TCGA-UY	BLCA	75	FEMALE	WHITE	Stage II	Muscle in	High Grade			-27234	Alive	TUMOR F	2312						Complete Remission/Response		0	2312	0	2312	0	2312	0	2312
TCGA-UY	TCGA-UY	BLCA	78	FEMALE	WHITE	Stage II	Muscle in	High Grade			-28719	Alive	TUMOR F	2380						Complete Remission/Response		0	2380	0	2380	0	2380	0	2380
TCGA-UY	TCGA-UY	BLCA	63	MALE	WHITE	Stage IV	Muscle in	High Grade			-23360	Alive	TUMOR F	2109						Complete Remission/Response		0	2109	0	2109			0	2109
TCGA-UY	TCGA-UY	BLCA	90	MALE	WHITE	Stage IV	Muscle in	High Grade			-32918	Dead	WITH TUMOR		92	Distant M	Lymph Node Only		32	Partial Remission/Response		1	92	1	92			1	32
TCGA-UY	TCGA-UY	BLCA	68	FEMALE	BLACK OI	Stage II	Muscle in	High Grade			-24588	Alive	TUMOR F	3432						Complete Remission/Response		0	3432	0	3432	0	3432	0	3432
TCGA-UY	TCGA-UY	BLCA	48	MALE	WHITE	Stage III	Muscle in	High Grade			-17520	Alive	TUMOR F	1072						Complete Remission/Response		0	1072	0	1072	0	1072	0	1072
TCGA-UY	TCGA-UY	BLCA	49	MALE	WHITE	Stage III	Muscle in	High Grade			-17885	Alive	TUMOR F	899						Complete Remission/Response		0	899	0	899	0	899	0	899
TCGA-UY	TCGA-UY	BLCA	80	MALE	WHITE	Stage III	Muscle in	High Grade			-29565	Alive	TUMOR F	542						Complete Remission/Response		0	542	0	542	0	542	0	542
TCGA-UY	TCGA-UY	BLCA	86	MALE	WHITE	Stage IV	Muscle in	High Grade			-31755	Alive	WITH TUI	189		Distant M	Lung		473	Complete Remission/Response		0	189	0	189			1	473
TCGA-UY	TCGA-UY	BLCA	77	MALE	WHITE	Stage IV	Muscle in	High Grade			-28470	Alive	TUMOR F	117						Complete Remission/Response		0	117	0	117			0	117
TCGA-UY	TCGA-UY	BLCA	73	MALE	WHITE	Stage II	Muscle in	High Grade			-26654	Alive	TUMOR F	1561						Complete Remission/Response		0	1561	0	1561	0	1561	0	1561
TCGA-XF	TCGA-XF	BLCA	50	FEMALE	[Unknowr	Stage II	Muscle in	High Grac	2006		-18544	Alive	TUMOR F	1370						Complete Remission/Response		0	1370	0	1370	0	1370	0	1370
TCGA-XF	TCGA-XF	BLCA	79	MALE	ASIAN	Stage IV	Muscle in	High Grac	2005		-28996	Dead			200					[Unknown]		1	200		200			0	200
TCGA-XF	TCGA-XF	BLCA	77	MALE	WHITE	Stage III	Muscle in	High Grac	2006		-28305	Alive	TUMOR F	2964						Complete Remission/Response		0	2964	0	2964	0	2964	0	2964
TCGA-XF	TCGA-XF	BLCA	47	MALE	WHITE	Stage III	Muscle in	High Grac	2004		-17212	Alive	TUMOR F	3817						Complete Remission/Response		0	3817	0	3817	0	3817	0	3817
TCGA-XF	TCGA-XF	BLCA	80	MALE	WHITE	Stage III	Muscle in	High Grac	2004		-29288	Dead	TUMOR FREE		2954					Complete Remission/Response		1	2954	0	2954	0	2954	0	2954
TCGA-XF	TCGA-XF	BLCA	69	MALE	WHITE	Stage III	Muscle in	High Grac	2005		-25421	Dead	WITH TUMOR		467	Distant M	Liver		398	Complete Remission/Response		1	467	1	467	1	398	1	398
TCGA-XF	TCGA-XF	BLCA	61	FEMALE	WHITE	Stage IV	Muscle in	High Grac	2004		-22393	Dead	WITH TUMOR		57					Partial Remission/Response		1	57	1	57			1	57
TCGA-XF	TCGA-XF	BLCA	57	FEMALE	WHITE	Stage IV	Muscle in	High Grac	2005		-21059	Dead	WITH TUMOR		544	Distant M	Lung		468	Progressive Disease		1	544	1	544			1	468
TCGA-XF	TCGA-XF	BLCA	81	MALE	[Unknowr	Stage II	Muscle in	High Grac	2006		-29825	Alive	TUMOR F	1479						Complete Remission/Response		0	1479	0	1479	0	1479	0	1479
TCGA-XF	TCGA-XF	BLCA	65	FEMALE	ASIAN	Stage II	Muscle in	High Grac	2005		-24060	Dead	WITH TUMOR		1971	Locoregic	Lymph Node Only		547	Stable Disease		1	1971	1	1971			1	547
TCGA-XF	TCGA-XF	BLCA	73	FEMALE	ASIAN	Stage II	Muscle in	High Grac	2006		-26719	Alive	TUMOR F	2423						Complete Remission/Response		0	2423	0	2423	0	2423	0	2423
TCGA-XF	TCGA-XF	BLCA	83	MALE	WHITE	Stage IV	Muscle in	High Grac	2004		-30362	Dead	WITH TUMOR		98					Progressive Disease		1	98	1	98			1	98
TCGA-XF	TCGA-XF	BLCA	65	MALE	WHITE	Stage IV	Muscle in	High Grac	2004		-24019	Dead	WITH TUMOR		486	Distant M	Bone		382	Stable Disease		1	486	1	486			1	382
TCGA-XF	TCGA-XF	BLCA	69	MALE	WHITE	Stage IV	Muscle in	High Grac	2004		-25460	Dead	TUMOR FREE		2020					Complete Remission/Response		1	2020	0	2020			0	2020
TCGA-XF	TCGA-XF	BLCA	76	MALE	WHITE	Stage III	Muscle in	High Grac	2004		-27826	Alive	TUMOR F	1048						Complete Remission/Response		0	1048	0	1048	0	1048	0	1048
TCGA-XF	TCGA-XF	BLCA	59	MALE	WHITE	Stage III	Muscle in	High Grac	2004		-21776	Dead	WITH TUMOR		454	Locoregic	Renal Pelvis		310	Stable Disease		1	454	1	454			1	310
TCGA-XF	TCGA-XF	BLCA	68	MALE	WHITE	Stage III	Muscle in	High Grac	2005		-25080	Dead			128					[Unknown]		1	128		128			0	128
TCGA-XF	TCGA-XF	BLCA	74	FEMALE	WHITE	Stage IV	Muscle in	High Grac	2006		-27068	Dead	WITH TUMOR		182	Distant M	Liver		103	Progressive Disease		1	182	1	182			1	103
TCGA-XF	TCGA-XF	BLCA	82	MALE	WHITE	Stage IV	Muscle in	High Grac	2006		-30265	Dead	WITH TUMOR		388	Distant M	Lung		237	Progressive Disease		1	388	1	388			1	237
TCGA-XF	TCGA-XF	BLCA	85	MALE	WHITE	Stage IV	Muscle in	High Grac	2006		-31049	Dead	WITH TUMOR		362					Partial Remission/Response		1	362	1	362			1	362
TCGA-XF	TCGA-XF	BLCA	63	FEMALE	WHITE	Stage IV	Muscle in	High Grac	2006		-23349	Dead	WITH TUMOR		719	Distant M	Lung		475	Progressive Disease		1	719	1	719			1	475
TCGA-XF	TCGA-XF	BLCA	60	FEMALE	WHITE	Stage IV	Muscle in	High Grac	2006		-22212	Alive	WITH TUI	640		Distant M	Other, sp	Groin	168	Progressive Disease		0	640	0	640			1	168
TCGA-XF	TCGA-XF	BLCA	79	MALE	WHITE	Stage IV	Muscle in	High Grac	2007		-29100	Dead	WITH TUMOR		859	Distant M	Lung		854	Complete Remission/Response		1	859	1	859			1	854
TCGA-XF	TCGA-XF	BLCA	68	MALE	WHITE	Stage III	Muscle in	High Grac	2007		-25190	Alive	TUMOR F	799						Complete Remission/Response		0	799	0	799	0	799	0	799
TCGA-XF	TCGA-XF	BLCA	54	MALE	WHITE	Stage III	Muscle in	High Grac	2007		-19945	Dead			575					[Unknown]		1	575		575			0	575
TCGA-XF	TCGA-XF	BLCA	69	FEMALE	WHITE	Stage IV	Muscle in	High Grac	2008		-25532	Alive	TUMOR F	68						[Unknown]		0	68	0	68			0	68
TCGA-XF	TCGA-XF	BLCA	48	MALE	WHITE	Stage IV	Muscle in	High Grac	2008		-17728	Dead	WITH TUMOR		495	Distant M	Lung		182	Partial Remission/Response		1	495	1	495			1	182
TCGA-XF	TCGA-XF	BLCA	78	FEMALE	WHITE	Stage IV	Muscle in	High Grac	2008		-28578	Alive	TUMOR F	2027		Locoregic	Other, sp	Vagina	664	Complete Remission/Response		0	2027	0	2027			1	664
TCGA-XF	TCGA-XF	BLCA	88	FEMALE	WHITE	Stage III	Muscle in	High Grac	2008		-32207	Alive		64						[Unknown]		0	64	0	64			0	64
TCGA-XF	TCGA-XF	BLCA	64	MALE	WHITE	Stage III	Muscle in	High Grac	2008		-23403	Dead			418					[Unknown]		1	418		418			0	418
TCGA-XF	TCGA-XF	BLCA	64	FEMALE	WHITE	Stage II	Muscle in	High Grac	2006		-23448	Dead	WITH TUMOR		2828	Locoregic	Other, sp	Vagina	1651	Progressive Disease		1	2828	1	2828			1	1651
TCGA-XF	TCGA-XF	BLCA	68	MALE	WHITE	Stage IV	Muscle in	High Grac	2004		-25023	Dead	WITH TUMOR		329	Distant M	Lung		157	Stable Disease		1	329	1	329			1	157
TCGA-XF	TCGA-XF	BLCA	49	MALE	WHITE	Stage III	Muscle in	High Grac	2004		-18021	Alive	TUMOR F	3364						Complete Remission/Response		0	3364	0	3364	0	3364	0	3364
TCGA-XF	TCGA-XF	BLCA	80	MALE	WHITE	Stage IV	Muscle in	High Grac	2005		-29547	Dead	WITH TUMOR		344	Distant M	Other, sp	lumbar sc	313	Progressive Disease		1	344	1	344			1	313
TCGA-XF	TCGA-XF	BLCA	70	MALE	ASIAN	Stage III	Muscle in	High Grac	2006		-25838	Dead	TUMOR FREE		1670					Complete Remission/Response		1	1670	0	1670	0	1670	0	1670
TCGA-XF	TCGA-XF	BLCA	75	MALE	WHITE	Stage II	Muscle in	High Grac	2007		-27422	Dead	TUMOR FREE		232					Complete Remission/Response		1	232	0	232	0	232	0	232
TCGA-XF	TCGA-XF	BLCA	59	FEMALE	WHITE	Stage II	Muscle in	High Grac	2006		-21660	Alive	TUMOR F	2177						Complete Remission/Response		0	2177	0	2177	0	2177	0	2177
TCGA-XF	TCGA-XF	BLCA	48	MALE	WHITE	Stage III	Muscle in	High Grac	2006		-17670	Alive	TUMOR F	2790						Complete Remission/Response		0	2790	0	2790	0	2790	0	2790
TCGA-XF	TCGA-XF	BLCA	75	FEMALE	WHITE	Stage IV	Muscle in	High Grac	2006		-27397	Dead	WITH TUMOR		90					Partial Remission/Response		1	90	1	90			1	90
TCGA-XF	TCGA-XF	BLCA	79	FEMALE	WHITE	Stage IV	Muscle in	High Grac	2006		-28903	Dead	WITH TUMOR		253					Partial Remission/Response		1	253	1	253			1	253
TCGA-XF	TCGA-XF	BLCA	87	FEMALE	BLACK OI	Stage III	Muscle in	High Grac	2006		-31884	Dead	TUMOR FREE		206					Complete Remission/Response		1	206	0	206	0	206	0	206
TCGA-XF	TCGA-XF	BLCA	78	MALE	WHITE	Stage III	Muscle in	High Grac	2006		-28509	Alive	TUMOR F	3011		Distant M	Lung		637	Complete Remission/Response		0	3011	0	3011	1	637	1	637
TCGA-XF	TCGA-XF	BLCA	81	FEMALE	WHITE	Stage IV	Muscle in	High Grac	2007		-29667	Dead	WITH TUMOR		1348	Locoregic	Lymph Node Only		1344	Partial Remission/Response		1	1348	1	1348			1	1344
TCGA-XF	TCGA-XF	BLCA	68	MALE	WHITE	Stage IV	Muscle in	High Grac	2007		-24877	Dead	WITH TUMOR		1718	Locoregic	Other, sp	Scrotum a	603	Complete Remission/Response		1	1718	1	1718			1	603
TCGA-XF	TCGA-XF	BLCA	75	FEMALE	[Unknowr	Stage III	Muscle in	High Grac	2006		-27687	Dead	WITH TUMOR		941	Locoregic	Other, sp	Pelvis and	650	Partial Remission/Response		1	941	1	941			1	650
TCGA-XF	TCGA-XF	BLCA	73	MALE	WHITE	Stage II	Muscle in	High Grac	2007		-26720	Dead	TUMOR FREE		1869					Complete Remission/Response		1	1869	0	1869	0	1869	0	1869
TCGA-XF	TCGA-XF	BLCA	76	MALE	WHITE	Stage IV	Muscle in	High Grac	2007		-27909	Alive	TUMOR F	2625						Complete Remission/Response		0	2625	0	2625			0	2625
TCGA-XF	TCGA-XF	BLCA	77	FEMALE	WHITE	Stage III	Muscle in	High Grac	2007		-28302	Dead	TUMOR FREE		823					Complete Remission/Response		1	823	0	823	0	823	0	823
TCGA-XF	TCGA-XF	BLCA	61	FEMALE	WHITE	Stage III	Muscle in	High Grac	2008		-22411	Alive	TUMOR F	2293						Complete Remission/Response		0	2293	0	2293	0	2293	0	2293
TCGA-XF	TCGA-XF	BLCA	60	MALE	WHITE	Stage IV	Muscle in	High Grac	2008		-21951	Dead	WITH TUMOR		565	Locoregic	Bladder		321	Complete Remission/Response		1	565	1	565			1	321
TCGA-XF	TCGA-XF	BLCA	74	FEMALE	WHITE	Stage III	Muscle in	High Grac	2007		-27309	Dead			118					[Unknown]		1	118		118			0	118
TCGA-YC	TCGA-YC	BLCA	78	FEMALE	WHITE	Stage II	Muscle in	High Grac	2013		-28741	Alive	TUMOR F	573						Complete Remission/Response		0	573	0	573	0	573	0	573
TCGA-YC	TCGA-YC	BLCA	71	MALE	WHITE	Stage II	Muscle in	High Grac	2013		-26130	Alive	TUMOR F	293						Complete Remission/Response		0	293	0	293	0	293	0	293
TCGA-YC	TCGA-YC	BLCA	71	MALE	WHITE	Stage IV	Muscle in	High Grac	2013		-26283	Dead	WITH TUMOR		20					Progressive Disease		1	20	1	20			1	20
TCGA-YF	TCGA-YF	BLCA	47	FEMALE	[Unknowr	Stage II	Muscle in	High Grac	2013		-17268	Alive	TUMOR F	364						Complete Remission/Response		0	364	0	364	0	364	0	364
TCGA-YF	TCGA-YF	BLCA	57	MALE	[Unknowr	Stage II	Muscle in	High Grac	2013		-21097	Alive	WITH TUI	415		Locoregic	Other, sp	Pelvis	381	Complete Remission/Response		0	415	0	415	1	381	1	381
TCGA-ZF	TCGA-ZF	BLCA	82	MALE	WHITE	Stage III	Muscle in	High Grac	2013		-30222	Dead	WITH TUMOR		680	Locoregic	Other, sp	right hem	559	Progressive Disease		1	680	1	680			1	559
TCGA-ZF	TCGA-ZF	BLCA	81	MALE	WHITE	Stage IV	Muscle in	High Grac	2013		-29610	Alive	WITH TUI	773		Distant M	Bone		758	Complete Remission/Response		0	773	0	773			1	758
TCGA-ZF	TCGA-ZF	BLCA	75	MALE	WHITE	Stage II	Muscle in	High Grac	2013		-27537	Alive	TUMOR F	642						Complete Remission/Response		0	642	0	642	0	642	0	642
TCGA-ZF	TCGA-ZF	BLCA	53	FEMALE	WHITE	Stage II	Muscle in	High Grac	2012		-19384	Dead	WITH TUMOR		949	Distant M	Bone		917	Complete Remission/Response		1	949	1	949	1	917	1	917
TCGA-ZF	TCGA-ZF	BLCA	83	MALE	WHITE	Stage II	Muscle in	High Grac	2012		-30398	Alive	TUMOR F	921						Complete Remission/Response		0	921	0	921	0	921	0	921
TCGA-ZF	TCGA-ZF	BLCA	59	MALE	WHITE	Stage III	Muscle in	High Grac	2011		-21811	Alive	TUMOR F	1090						Complete Remission/Response		0	1090	0	1090	0	1090	0	1090
TCGA-ZF	TCGA-ZF	BLCA	76	FEMALE	WHITE	Stage II	Muscle in	High Grac	2011		-28063	Alive	TUMOR F	665						Complete Remission/Response		0	665	0	665	0	665	0	665
TCGA-ZF	TCGA-ZF	BLCA	58	MALE	WHITE	Stage IV	Muscle in	High Grac	2010		-21491	Dead	WITH TUMOR		864	Distant M	Other, sp	penis	629	Complete Remission/Response		1	864	1	864			1	629
TCGA-ZF	TCGA-ZF	BLCA	77	MALE	WHITE	Stage III	Muscle in	High Grac	2007		-28275	Alive	TUMOR F	2868						Complete Remission/Response		0	2868	0	2868	0	2868	0	2868
TCGA-ZF	TCGA-ZF	BLCA	75	FEMALE	WHITE	Stage IV	Muscle in	High Grac	2008		-27496	Dead			408					[Unknown]		1	408		408			0	408
TCGA-ZF	TCGA-ZF	BLCA	78	FEMALE	WHITE	Stage II	Muscle in	High Grac	2010		-28622	Dead	WITH TUMOR		106					Progressive Disease		1	106	1	106			1	106
TCGA-ZF	TCGA-ZF	BLCA	74	MALE	WHITE	Stage II	Muscle in	High Grac	2009		-27281	Alive	TUMOR F	1949		Locoregic	Bladder		1565	Complete Remission/Response		0	1949	0	1949	1	1565	1	1565
TCGA-ZF	TCGA-ZF	BLCA	70	FEMALE	WHITE	Stage III	Muscle in	High Grac	2010		-25656	Alive	WITH TUI	1786		Distant M	Other, sp	neck lymp	440	Complete Remission/Response		0	1786	0	1786	1	440	1	440
TCGA-ZF	TCGA-ZF	BLCA	61	MALE	WHITE	Stage II	Muscle in	High Grac	2007		-22480	Alive	TUMOR F	2703		Locoregic	Bladder		1064	Complete Remission/Response		0	2703	0	2703	1	1064	1	1064
TCGA-ZF	TCGA-ZF	BLCA	70	MALE	WHITE	Stage II	Muscle in	High Grac	2010		-25642	Alive	TUMOR F	1455						Complete Remission/Response		0	1455	0	1455	0	1455	0	1455
TCGA-ZF	TCGA-ZF	BLCA	67	FEMALE	WHITE	Stage III	Muscle in	High Grac	2010		-24795	Dead	WITH TUMOR		615	Distant M	Lung		516	Partial Remission/Response		1	615	1	615			1	516
TCGA-ZF	TCGA-ZF	BLCA	74	MALE	WHITE	Stage II		High Grac	2011		-27056	Dead	WITH TUMOR		88					Progressive Disease		1	88	1	88			1	88
TCGA-ZF	TCGA-ZF	BLCA	67	MALE	WHITE	Stage IV	Muscle in	High Grac	2008		-24537	Dead	WITH TUMOR		1036	Locoregic	Lymph Node Only		750	[Unknown]		1	1036	1	1036			1	750
TCGA-ZF	TCGA-ZF	BLCA	65	MALE	WHITE	Stage IV	Muscle in	High Grac	2008		-23974	Dead	WITH TUMOR		599	Distant M	Lung		511	[Unknown]		1	599	1	599			1	511
TCGA-ZF	TCGA-ZF	BLCA	70	MALE	WHITE	Stage III	Muscle in	High Grac	2008		-25682	Dead	WITH TUMOR		262	Distant M	Bone		199	Progressive Disease		1	262	1	262			1	199
TCGA-ZF	TCGA-ZF	BLCA	66	MALE	WHITE	Stage III	Muscle in	High Grac	2008		-24448	Alive	TUMOR F	1806						Complete Remission/Response		0	1806	0	1806	0	1806	0	1806
TCGA-ZF	TCGA-ZF	BLCA	56	MALE	WHITE	Stage III	Muscle in	High Grac	2009		-20611	Alive		1830		Locoregic	Other, sp	penile ure	1679	Complete Remission/Response		0	1830	0	1830	1	1679	1	1679
TCGA-ZF	TCGA-ZF	BLCA	56	MALE	WHITE	Stage II	Muscle in	High Grac	2009		-20579	Alive	WITH TUI	2044		Locoregic	Bladder		1665	Complete Remission/Response		0	2044	0	2044	1	1665	1	1665
TCGA-ZF	TCGA-ZF	BLCA	69	FEMALE	WHITE	Stage II	Muscle in	High Grac	2009		-25484	Alive	TUMOR F	1714						Complete Remission/Response		0	1714	0	1714	0	1714	0	1714
TCGA-ZF	TCGA-ZF	BLCA	70	MALE	WHITE	Stage III	Muscle in	High Grac	2009		-25579	Dead			1077					Partial Remission/Response		1	1077		1077			0	1077
TCGA-ZF	TCGA-ZF	BLCA	60	MALE	WHITE	Stage II	Muscle in	High Grac	2010		-22035	Alive	TUMOR F	1761						Complete Remission/Response		0	1761	0	1761	0	1761	0	1761
TCGA-ZF	TCGA-ZF	BLCA	71	MALE	WHITE	Stage III	Muscle in	High Grac	2009		-26128	Dead			590					[Unknown]		1	590		590			0	590
TCGA-ZF	TCGA-ZF	BLCA	79	FEMALE	WHITE	Stage III	Muscle in	High Grac	2010		-28937	Dead	WITH TUMOR		259	Distant M	Liver		248	Progressive Disease		1	259	1	259			1	248
TCGA-ZF	TCGA-ZF	BLCA	61	FEMALE	WHITE	Stage IV	Muscle in	High Grac	2010		-22549	Alive	TUMOR F	1649						Complete Remission/Response		0	1649	0	1649			0	1649
TCGA-ZF	TCGA-ZF	BLCA	60	FEMALE	WHITE	Stage IV	Muscle in	High Grac	2012		-22122	Alive	WITH TUI	897		Locoregic	Other, sp	peritoneu	177	Stable Disease		0	897	0	897			1	177
TCGA-ZF	TCGA-ZF	BLCA	62	FEMALE	WHITE	Stage IV	Muscle in	High Grac	2013		-22741	Dead	WITH TUMOR		168					Progressive Disease		1	168	1	168			1	168
TCGA-ZF	TCGA-ZF	BLCA	65	MALE	WHITE	Stage IV	Muscle in	High Grac	2013		-23971	Alive	TUMOR F	372						Complete Remission/Response		0	372	0	372			0	372
TCGA-3C	TCGA-3C	BRCA	55	FEMALE	WHITE	Stage X	Infiltrating Lobular C		2004	Pre (<6 m	-20211	Alive	WITH TUI	4047		Distant M	Lung		1808		Negative	0	4047	0	4047	1	1808	1	1808
TCGA-3C	TCGA-3C	BRCA	50	FEMALE	BLACK OI	Stage IIB	Infiltrating Ductal Ca		2003	Post (prio	-18538	Alive	TUMOR F	4005								Negative	0	4005	0	4005	0	4005	0	4005
TCGA-3C	TCGA-3C	BRCA	62	FEMALE	BLACK OI	Stage IIB	Infiltrating Ductal Ca		2011	Post (prio	-22848	Alive	TUMOR F	1474								Negative	0	1474	0	1474	0	1474	0	1474
TCGA-3C	TCGA-3C	BRCA	52	FEMALE	BLACK OI	Stage IA	Infiltrating Ductal Ca		2011	[Unknowr	-19074	Alive	TUMOR F	1448								Close	0	1448	0	1448			0	1448
TCGA-4H	TCGA-4H	BRCA	50	FEMALE	WHITE	Stage IIIA	Infiltrating Lobular C		2013	Post (prio	-18371	Alive	TUMOR F	348								Negative	0	348	0	348	0	348	0	348
TCGA-5L-	TCGA-5L-	BRCA	42	FEMALE	WHITE	Stage IIA	Infiltrating Lobular C		2010	Post (prio	-15393	Alive	TUMOR F	1477								Positive	0	1477	0	1477			0	1477
TCGA-5L-	TCGA-5L-	BRCA	63	FEMALE	WHITE	Stage IV	Infiltrating Lobular C		2010	Post (prio	-23225	Alive	WITH TUI	1471								Negative	0	1471	0	1471			0	1471
TCGA-5T-	TCGA-5T-	BRCA	52	FEMALE	BLACK OI	Stage IIA	Other, specify		2013	[Unknowr	-19031	Alive		303								[Unknown]	0	303	0	303			0	303
TCGA-A1	TCGA-A1	BRCA	70	FEMALE	WHITE	Stage I	Other, specify		2008	Post (prio	-25833	Alive	TUMOR F	259								Negative	0	259	0	259	0	259	0	259
TCGA-A1	TCGA-A1	BRCA	59	FEMALE	WHITE	Stage IIA	Infiltrating Ductal Ca		2005		-21793	Alive		437								Negative	0	437	0	437	0	437	0	437
TCGA-A1	TCGA-A1	BRCA	56	FEMALE	WHITE	Stage I	Mixed Histology (ple		2005	Pre (<6 m	-20717	Alive	TUMOR F	1321								Negative	0	1321	0	1321	0	1321	0	1321
TCGA-A1	TCGA-A1	BRCA	54	FEMALE	WHITE	Stage IIA	Infiltrating Ductal Ca		2006	Pre (<6 m	-19731	Alive	TUMOR F	1463								Negative	0	1463	0	1463	0	1463	0	1463
TCGA-A1	TCGA-A1	BRCA	61	FEMALE	WHITE	Stage IIB	Other, specify		2006	Post (prio	-22380	Alive		434								Negative	0	434	0	434	0	434	0	434
TCGA-A1	TCGA-A1	BRCA	39	FEMALE	WHITE	Stage IIA	Infiltrating Ductal Ca		2006	Pre (<6 m	-14595	Alive	TUMOR F	1437								Negative	0	1437	0	1437	0	1437	0	1437
TCGA-A1	TCGA-A1	BRCA	52	FEMALE	WHITE	Stage IIB	Infiltrating Ductal Ca		2007		-19250	Alive	TUMOR F	635								Negative	0	635	0	635	0	635	0	635
TCGA-A1	TCGA-A1	BRCA	39	FEMALE	BLACK OI	Stage IIIA	Infiltrating Ductal Ca		2006		-14383	Alive	TUMOR F	416								Negative	0	416	0	416	0	416	0	416
TCGA-A1	TCGA-A1	BRCA	54	FEMALE	ASIAN	Stage IIA	Other, specify		2007	Indetermi	-20048	Dead	WITH TUMOR		967							Negative	1	967	1	967			1	967
TCGA-A1	TCGA-A1	BRCA	77	MALE	WHITE	Stage IIA	Infiltrating Ductal Ca		2007		-28198	Alive	TUMOR F	242								Negative	0	242	0	242	0	242	0	242
TCGA-A1	TCGA-A1	BRCA	50	FEMALE	WHITE	Stage IIA	Infiltrating Ductal Ca		2007	Post (prio	-18401	Alive	TUMOR F	1196								Negative	0	1196	0	1196	0	1196	0	1196
TCGA-A1	TCGA-A1	BRCA	67	FEMALE	WHITE	Stage IIB	Infiltrating Ductal Ca		2007	Post (prio	-24826	Alive	TUMOR F	852								Negative	0	852	0	852	0	852	0	852
TCGA-A1	TCGA-A1	BRCA	40	FEMALE		Stage IIA	Infiltrating Ductal Ca		2007		-14954	Alive	TUMOR F	584								Negative	0	584	0	584	0	584	0	584
TCGA-A1	TCGA-A1	BRCA	45	FEMALE	WHITE	Stage IIB	Infiltrating Ductal Ca		2007	Pre (<6 m	-16458	Alive	TUMOR F	554								Negative	0	554	0	554	0	554	0	554
TCGA-A2	TCGA-A2	BRCA	66	FEMALE	WHITE	Stage IA	Infiltrating Ductal Ca		2002	Post (prio	-24155	Alive	TUMOR F	4354								Negative	0	4354	0	4354	0	4354	0	4354
TCGA-A2	TCGA-A2	BRCA	36	FEMALE	BLACK OI	Stage IIIC	Infiltrating Ductal Ca		2003	Pre (<6 m	-13238	Dead	WITH TUMOR		548	Distant M	Bone		102		Positive	1	548	1	548			1	102
TCGA-A2	TCGA-A2	BRCA	48	FEMALE	WHITE	Stage IA	Infiltrating Ductal Ca		2004	Post (prio	-17660	Alive	TUMOR F	2385								Negative	0	2385	0	2385	0	2385	0	2385
TCGA-A2	TCGA-A2	BRCA	36	FEMALE	BLACK OI	Stage IA	Infiltrating Ductal Ca		2004	Pre (<6 m	-13483	Alive	TUMOR F	3709								Negative	0	3709	0	3709	0	3709	0	3709
TCGA-A2	TCGA-A2	BRCA	62	FEMALE	WHITE	Stage IIA	Infiltrating Ductal Ca		2004	Post (prio	-22707	Alive	TUMOR F	2246								Negative	0	2246	0	2246	0	2246	0	2246
TCGA-A2	TCGA-A2	BRCA	47	FEMALE	WHITE	Stage IIA	Infiltrating Ductal Ca		2004	Peri (6-12	-17474	Alive	TUMOR F	2654								Negative	0	2654	0	2654	0	2654	0	2654
TCGA-A2	TCGA-A2	BRCA	39	FEMALE	WHITE	Stage IIA	Infiltrating Ductal Ca		2005	Pre (<6 m	-14250	Dead	WITH TUMOR		1920				1470		Negative	1	1920	1	1920	1	1470	1	1470
TCGA-A2	TCGA-A2	BRCA	50	FEMALE	WHITE	Stage IIB	Other, specify		2005	Pre (<6 m	-18345	Alive	TUMOR F	3102								Negative	0	3102	0	3102	0	3102	0	3102
TCGA-A2	TCGA-A2	BRCA	34	FEMALE	WHITE	Stage IIA	Infiltrating Ductal Ca		2006	Pre (<6 m	-12642	Alive	TUMOR F	1686								Negative	0	1686	0	1686	0	1686	0	1686
TCGA-A2	TCGA-A2	BRCA	53	FEMALE	WHITE	Stage IIB	Infiltrating Ductal Ca		2008	Post (prio	-19533	Alive	TUMOR F	1099								Negative	0	1099	0	1099	0	1099	0	1099
TCGA-A2	TCGA-A2	BRCA	60	FEMALE	WHITE	Stage IIIA	Infiltrating Lobular C		2001	Post (prio	-22094	Alive	TUMOR F	4159		New Prim	Other, sp	LEFT BRE/	1364		Negative	0	4159	0	4159	1	1364	1	1364
TCGA-A2	TCGA-A2	BRCA	37	FEMALE	BLACK OI	Stage IIIA	Infiltrating Ductal Ca		2006	Pre (<6 m	-13556	Alive	TUMOR F	3015								Positive	0	3015	0	3015			0	3015
TCGA-A2	TCGA-A2	BRCA	40	FEMALE	BLACK OI	Stage IIA	Infiltrating Ductal Ca		2003	Pre (<6 m	-14969	Dead	WITH TUMOR		754				382		Negative	1	754	1	754	1	382	1	382
TCGA-A2	TCGA-A2	BRCA	85	FEMALE	WHITE	Stage IIB	Infiltrating Lobular C		2002	Post (prio	-31404	Dead	TUMOR FREE		3492							Positive	1	3492	0	3492			0	3492
TCGA-A2	TCGA-A2	BRCA	60	FEMALE	WHITE	Stage IA	Infiltrating Ductal Ca		2003	Post (prio	-22133	Alive	TUMOR F	2813								Negative	0	2813	0	2813	0	2813	0	2813
TCGA-A2	TCGA-A2	BRCA	62	FEMALE	BLACK OI	Stage IA	Infiltrating Ductal Ca		2003	Post (prio	-22810	Alive	TUMOR F	2695								Negative	0	2695	0	2695	0	2695	0	2695
TCGA-A2	TCGA-A2	BRCA	54	FEMALE	ASIAN	Stage IIB	Infiltrating Lobular C		2004	Post (prio	-20075	Alive	TUMOR F	3283								Close	0	3283	0	3283			0	3283
TCGA-A2	TCGA-A2	BRCA	73	FEMALE	WHITE	Stage IV	Infiltrating Ductal Ca		2004	Post (prio	-26817	Dead	TUMOR FREE		2348							Positive	1	2348	0	2348			0	2348
TCGA-A2	TCGA-A2	BRCA	71	FEMALE	WHITE	Stage IIA	Infiltrating Ductal Ca		2005	Post (prio	-25959	Alive	TUMOR F	2289								Negative	0	2289	0	2289	0	2289	0	2289
TCGA-A2	TCGA-A2	BRCA	73	FEMALE	WHITE	Stage IIA	Infiltrating Ductal Ca		2005	Post (prio	-26823	Dead	TUMOR FREE		158							Close	1	158	0	158			0	158
TCGA-A2	TCGA-A2	BRCA	41	FEMALE	ASIAN	Stage IIB	Infiltrating Ductal Ca		2005	Post (prio	-15289	Alive	TUMOR F	3011								Negative	0	3011	0	3011	0	3011	0	3011
TCGA-A2	TCGA-A2	BRCA	67	FEMALE	WHITE	Stage IIB	Infiltrating Ductal Ca		2006	Post (prio	-24541	Alive	TUMOR F	3283								Negative	0	3283	0	3283	0	3283	0	3283
TCGA-A2	TCGA-A2	BRCA	52	FEMALE	WHITE	Stage IIA	Infiltrating Ductal Ca		2006	Pre (<6 m	-19152	Alive	TUMOR F	1728								Negative	0	1728	0	1728	0	1728	0	1728
TCGA-A2	TCGA-A2	BRCA	63	FEMALE	WHITE	Stage IIB	Infiltrating Ductal Ca		2006	Post (prio	-23229	Alive	TUMOR F	1673								Negative	0	1673	0	1673	0	1673	0	1673
TCGA-A2	TCGA-A2	BRCA	46	FEMALE	WHITE	Stage IIA	Infiltrating Ductal Ca		2007	Peri (6-12	-17026	Alive	TUMOR F	1616								Positive	0	1616	0	1616			0	1616
TCGA-A2	TCGA-A2	BRCA	60	FEMALE	BLACK OI	Stage IIA	Infiltrating Ductal Ca		2008	Post (prio	-22115	Alive	TUMOR F	2048								Negative	0	2048	0	2048	0	2048	0	2048
TCGA-A2	TCGA-A2	BRCA	76	FEMALE	WHITE	Stage IIA	Infiltrating Ductal Ca		2008	Post (prio	-28084	Alive	TUMOR F	1051								Negative	0	1051	0	1051	0	1051	0	1051
TCGA-A2	TCGA-A2	BRCA	45	FEMALE	WHITE	Stage IIA	Infiltrating Ductal Ca		2008	Pre (<6 m	-16623	Alive	TUMOR F	1027								Negative	0	1027	0	1027	0	1027	0	1027

TCGA-A2	TCGA-A2	BRCA	42	FEMALE	WHITE	Stage IA	Infiltrating Ductal Ca	2008	Post (prio	-15524	Alive	TUMOR F	1873						Negative	0	1873	0	1873	0	1873	0	1873
TCGA-A2	TCGA-A2	BRCA	37	FEMALE	BLACK OI	Stage IIB	Infiltrating Ductal Ca	2009	Pre (<6 m	-13805	Alive	TUMOR F	767						Negative	0	767	0	767	0	767	0	767
TCGA-A2	TCGA-A2	BRCA	73	FEMALE	WHITE	Stage IA	Infiltrating Ductal Ca	2002	Post (prio	-26845	Alive	TUMOR F	3094						Negative	0	3094	0	3094	0	3094	0	3094
TCGA-A2	TCGA-A2	BRCA	70	FEMALE	WHITE	Stage IIA	Infiltrating Lobular C	2003	Post (prio	-25771	Alive	TUMOR F	4088						Negative	0	4088	0	4088	0	4088	0	4088
TCGA-A2	TCGA-A2	BRCA	54	FEMALE	WHITE	Stage IA	Infiltrating Ductal Ca	2003	Peri (6-12	-19880	Alive	TUMOR F	2442						Close	0	2442	0	2442			0	2442
TCGA-A2	TCGA-A2	BRCA	56	FEMALE	WHITE	Stage I	Infiltrating Lobular C	2004	Post (prio	-20585	Alive	TUMOR F	3603						Negative	0	3603	0	3603	0	3603	0	3603
TCGA-A2	TCGA-A2	BRCA	64	FEMALE	BLACK OI	Stage IIA	Infiltrating Ductal Ca	2004	Post (prio	-23543	Alive	TUMOR F	2426						Negative	0	2426	0	2426	0	2426	0	2426
TCGA-A2	TCGA-A2	BRCA	63	FEMALE	WHITE	Stage IB	Infiltrating Ductal Ca	2005	Post (prio	-23021	Alive	TUMOR F	2263						Negative	0	2263	0	2263	0	2263	0	2263
TCGA-A2	TCGA-A2	BRCA	52	FEMALE	WHITE	Stage IIA	Infiltrating Lobular C	2008	Post (prio	-19160	Alive	TUMOR F	2190						Negative	0	2190	0	2190	0	2190	0	2190
TCGA-A2	TCGA-A2	BRCA	58	FEMALE	WHITE	Stage IIIA	Infiltrating Ductal Ca	2008	Post (prio	-21464	Alive	TUMOR F	1066						Negative	0	1066	0	1066	0	1066	0	1066
TCGA-A2	TCGA-A2	BRCA	79	FEMALE	WHITE	Stage IA	Infiltrating Ductal Ca	2008	Post (prio	-29144	Alive	TUMOR F	1043						Negative	0	1043	0	1043	0	1043	0	1043
TCGA-A2	TCGA-A2	BRCA	80	FEMALE	WHITE	Stage IA	Infiltrating Ductal Ca	2008	Post (prio	-29285	Alive	TUMOR F	968						Negative	0	968	0	968	0	968	0	968
TCGA-A2	TCGA-A2	BRCA	53	FEMALE	WHITE	Stage IIIC	Infiltrating Lobular C	2008	Post (prio	-19413	Dead	WITH TUMOR		1884	Distant M	Bone		1353	Positive	1	1884	1	1884			1	1353
TCGA-A2	TCGA-A2	BRCA	46	FEMALE	WHITE	Stage IIB	Infiltrating Lobular C	2009	Post (prio	-16858	Alive	TUMOR F	752						Negative	0	752	0	752	0	752	0	752
TCGA-A2	TCGA-A2	BRCA	62	FEMALE	WHITE	Stage IIB	Infiltrating Ductal Ca	2009	Post (prio	-22872	Alive	TUMOR F	1925						Negative	0	1925	0	1925	0	1925	0	1925
TCGA-A2	TCGA-A2	BRCA	62	FEMALE	WHITE	Stage IIA	Infiltrating Ductal Ca	2003	Post (prio	-22922	Alive	WITH TUM	3017						Negative	0	3017	0	3017	0	3017	0	3017
TCGA-A2	TCGA-A2	BRCA	66	FEMALE	WHITE	Stage IIA	Infiltrating Ductal Ca	2006	Post (prio	-24219	Alive	TUMOR F	1662						Negative	0	1662	0	1662	0	1662	0	1662
TCGA-A2	TCGA-A2	BRCA	63	FEMALE	WHITE	Stage IV	Infiltrating Ductal Ca	2006	Post (prio	-23126	Dead	WITH TUMOR		825					Positive	1	825	1	825			1	825
TCGA-A2	TCGA-A2	BRCA	82	FEMALE	WHITE	Stage IV	Infiltrating Ductal Ca	2006	Post (prio	-30085	Dead	WITH TUMOR		1365				653	Negative	1	1365	1	1365			1	653
TCGA-A2	TCGA-A2	BRCA	48	FEMALE	WHITE	Stage IA	Infiltrating Ductal Ca	2007	Pre (<6 m	-17774	Alive	WITH TUM	1534					1239	Negative	0	1534	0	1534	1	1239	1	1239
TCGA-A2	TCGA-A2	BRCA	62	FEMALE	WHITE	Stage IIIA	Infiltrating Lobular C	2007	Post (prio	-22928	Alive	TUMOR F	1347						Negative	0	1347	0	1347	0	1347	0	1347
TCGA-A2	TCGA-A2	BRCA	59	FEMALE	WHITE	Stage IIB	Infiltrating Ductal Ca	2009	Post (prio	-21575	Alive	TUMOR F	533						Negative	0	533	0	533	0	533	0	533
TCGA-A2	TCGA-A2	BRCA	55	FEMALE	AMERICA	Stage IIIC	Infiltrating Ductal Ca	2009	Post (prio	-20227	Alive	TUMOR F	521						Negative	0	521	0	521	0	521	0	521
TCGA-A2	TCGA-A2	BRCA	66	FEMALE	WHITE	Stage IV	Infiltrating Ductal Ca	2009	Post (prio	-24235	Dead	WITH TUMOR		255					Close	1	255	1	255			1	255
TCGA-A2	TCGA-A2	BRCA	37	FEMALE	WHITE	Stage IB	Infiltrating Ductal Ca	2009	Pre (<6 m	-13680	Alive	TUMOR F	1516						Negative	0	1516	0	1516	0	1516	0	1516
TCGA-A2	TCGA-A2	BRCA	62	FEMALE	WHITE	Stage IIA	Infiltrating Lobular C	2009	Post (prio	-22849	Alive	TUMOR F	624						Negative	0	624	0	624	0	624	0	624
TCGA-A2	TCGA-A2	BRCA	39	FEMALE	WHITE	Stage IIA	Infiltrating Ductal Ca	2009	Pre (<6 m	-14503	Alive	TUMOR F	531						Negative	0	531	0	531	0	531	0	531
TCGA-A2	TCGA-A2	BRCA	50	FEMALE	WHITE	Stage IIB	Infiltrating Lobular C	2009	Pre (<6 m	-18334	Alive	TUMOR F	575						Negative	0	575	0	575	0	575	0	575
TCGA-A2	TCGA-A2	BRCA	51	FEMALE	WHITE	Stage IIA	Infiltrating Ductal Ca	2009	Pre (<6 m	-18756	Alive	TUMOR F	631						Negative	0	631	0	631	0	631	0	631
TCGA-A2	TCGA-A2	BRCA	59	FEMALE	WHITE	Stage IIB	Infiltrating Ductal Ca	2008	Post (prio	-21685	Alive	WITH TUM	990		Locoregional Disease			756	Close	0	990	0	990			1	756
TCGA-A2	TCGA-A2	BRCA	63	FEMALE	WHITE	Stage IIB	Infiltrating Lobular C	2009	Post (prio	-23021	Alive	TUMOR F	769						Negative	0	769	0	769	0	769	0	769
TCGA-A2	TCGA-A2	BRCA	48	FEMALE	WHITE	Stage IIB	Infiltrating Ductal Ca	2010	Post (prio	-17727	Alive	TUMOR F	554						Negative	0	554	0	554	0	554	0	554
TCGA-A2	TCGA-A2	BRCA	67	FEMALE	BLACK OI	Stage I	Infiltrating Ductal Ca	2009	Post (prio	-24660	Alive	TUMOR F	1535						Negative	0	1535	0	1535	0	1535	0	1535
TCGA-A2	TCGA-A2	BRCA	63	FEMALE	WHITE	Stage IIIC	Infiltrating Ductal Ca	2009	Post (prio	-23058	Alive	TUMOR F	666						Negative	0	666	0	666	0	666	0	666
TCGA-A2	TCGA-A2	BRCA	53	FEMALE	WHITE	Stage IIIA	Infiltrating Ductal Ca	2009	Post (prio	-19596	Alive	TUMOR F	659						Negative	0	659	0	659	0	659	0	659
TCGA-A2	TCGA-A2	BRCA	62	FEMALE	WHITE	Stage I	Infiltrating Ductal Ca	2009	Post (prio	-22961	Alive	TUMOR F	1505						Negative	0	1505	0	1505	0	1505	0	1505
TCGA-A2	TCGA-A2	BRCA	39	FEMALE	WHITE	Stage IIIA	Infiltrating Ductal Ca	2010	Pre (<6 m	-14448	Alive	WITH TUM	566		Distant M	Other, sp	Left Cervi	559	Negative	0	566	0	566	1	559	1	559
TCGA-A2	TCGA-A2	BRCA	61	FEMALE	WHITE	Stage IIA	Infiltrating Lobular C	2009	Post (prio	-22625	Alive	TUMOR F	588						Close	0	588	0	588			0	588
TCGA-A2	TCGA-A2	BRCA	48	FEMALE	WHITE	Stage IIIA	Infiltrating Lobular C	2010	Pre (<6 m	-17702	Alive	TUMOR F	1474						Negative	0	1474	0	1474	0	1474	0	1474
TCGA-A2	TCGA-A2	BRCA	67	FEMALE	WHITE	Stage IIA	Infiltrating Ductal Ca	2008	Post (prio	-24831	Alive	TUMOR F	965						Negative	0	965	0	965	0	965	0	965
TCGA-A2	TCGA-A2	BRCA	56	FEMALE	WHITE	Stage IIIB	Infiltrating Ductal Ca	2008	Post (prio	-20482	Dead	TUMOR FREE		723					Negative	1	723	0	723	0	723	0	723
TCGA-A2	TCGA-A2	BRCA	74	FEMALE	WHITE	Stage IIB	Infiltrating Lobular C	2010	Post (prio	-27287	Alive	TUMOR F	714						Negative	0	714	0	714	0	714	0	714
TCGA-A2	TCGA-A2	BRCA	62	FEMALE	WHITE	Stage IIIA	Infiltrating Ductal Ca	2010	Post (prio	-22779	Alive	TUMOR F	528						Negative	0	528	0	528	0	528	0	528
TCGA-A2	TCGA-A2	BRCA	61	FEMALE	WHITE	Stage IIIA	Infiltrating Ductal Ca	2008	Post (prio	-22344	Alive	TUMOR F	1847						Negative	0	1847	0	1847	0	1847	0	1847
TCGA-A2	TCGA-A2	BRCA	63	FEMALE	WHITE	Stage IIA	Infiltrating Ductal Ca	2010	Post (prio	-23055	Alive	TUMOR F	683						Negative	0	683	0	683	0	683	0	683
TCGA-A2	TCGA-A2	BRCA	49	FEMALE	WHITE	Stage IIB	Other, specify	2010	Pre (<6 m	-18002	Alive	TUMOR F	616						Negative	0	616	0	616	0	616	0	616
TCGA-A2	TCGA-A2	BRCA	85	FEMALE	WHITE	Stage IIB	Infiltrating Ductal Ca	2010	Post (prio	-31223	Alive	TUMOR F	584						Negative	0	584	0	584	0	584	0	584
TCGA-A2	TCGA-A2	BRCA	71	FEMALE	WHITE	Stage IIIA	Infiltrating Ductal Ca	2010	Post (prio	-25966	Alive	TUMOR F	595						Negative	0	595	0	595	0	595	0	595
TCGA-A2	TCGA-A2	BRCA	50	FEMALE	WHITE	Stage IIIA	Infiltrating Ductal Ca	2010	Pre (<6 m	-18514	Alive	TUMOR F	501						Negative	0	501	0	501	0	501	0	501
TCGA-A2	TCGA-A2	BRCA	70	FEMALE	BLACK OI	Stage I	Infiltrating Ductal Ca	2007	Post (prio	-25666	Alive	TUMOR F	1596						Close	0	1596	0	1596			0	1596
TCGA-A2	TCGA-A2	BRCA	44	FEMALE	WHITE	Stage IIA	Infiltrating Lobular C	2003	Pre (<6 m	-16084	Alive	TUMOR F	3276						Negative	0	3276	0	3276	0	3276	0	3276
TCGA-A2	TCGA-A2	BRCA	39	FEMALE	BLACK OI	Stage IIB	Infiltrating Ductal Ca	2010	Pre (<6 m	-14454	Alive	WITH TUM	1291		Distant M	Bone		1290	Close	0	1291	0	1291			1	1290
TCGA-A2	TCGA-A2	BRCA	50	FEMALE	WHITE	Stage IIB	Infiltrating Ductal Ca	2010	Pre (<6 m	-18508	Alive	TUMOR F	523						Negative	0	523	0	523	0	523	0	523
TCGA-A2	TCGA-A2	BRCA	90	FEMALE	WHITE	Stage IIIC	Infiltrating Lobular C	2010	Post (prio	-32872	Alive	TUMOR F	552						Positive	0	552	0	552			0	552
TCGA-A2	TCGA-A2	BRCA	34	FEMALE	BLACK OI	Stage IIIA	Infiltrating Ductal Ca	2004	Pre (<6 m	-12601	Alive	TUMOR F	3204		Locoregic	Other, sp	Left Chest	2989	Negative	0	3204	0	3204	1	2989	1	2989
TCGA-A2	TCGA-A2	BRCA	66	FEMALE	WHITE	Stage IIA	Infiltrating Ductal Ca	2011	Post (prio	-24319	Alive	TUMOR F	322						Negative	0	322	0	322	0	322	0	322
TCGA-A2	TCGA-A2	BRCA	55	FEMALE	BLACK OI	Stage IIB	Infiltrating Lobular C	2011	Pre (<6 m	-20401	Alive	TUMOR F	1102						Negative	0	1102	0	1102	0	1102	0	1102
TCGA-A2	TCGA-A2	BRCA	47	FEMALE	WHITE	Stage IIIA	Mucinous Carcinoma	2010	Pre (<6 m	-17372	Alive	TUMOR F	1206						Positive	0	1206	0	1206			0	1206
TCGA-A2	TCGA-A2	BRCA	62	FEMALE	BLACK OI	Stage IIIA	Infiltrating Ductal Ca	2003	Post (prio	-23000	Dead	WITH TUMOR		1032	Distant M	Lung		682	Negative	1	1032	1	1032	1	682	1	682
TCGA-A2	TCGA-A2	BRCA	45	FEMALE	BLACK OI	Stage IIB	Infiltrating Ductal Ca	2006	Pre (<6 m	-16649	Alive	TUMOR F	2770						Negative	0	2770	0	2770	0	2770	0	2770
TCGA-A2	TCGA-A2	BRCA	35	FEMALE	BLACK OI	Stage IIB	Infiltrating Ductal Ca	2006	Post (prio	-13046	Dead	WITH TUMOR		912	Distant M	Bone		574	Negative	1	912	1	912	1	574	1	574
TCGA-A2	TCGA-A2	BRCA	46	FEMALE	BLACK OI	Stage IIA	Infiltrating Ductal Ca	2011	Pre (<6 m	-16971	Alive	TUMOR F	996						Negative	0	996	0	996	0	996	0	996
TCGA-A2	TCGA-A2	BRCA	42	FEMALE	BLACK OI	Stage IIB	Infiltrating Ductal Ca	2009	Pre (<6 m	-15670	Alive	TUMOR F	1712						Negative	0	1712	0	1712	0	1712	0	1712
TCGA-A2	TCGA-A2	BRCA	49	FEMALE	BLACK OI	Stage IIA	Infiltrating Ductal Ca	2009	Post (prio	-17981	Dead	TUMOR FREE		1439					Negative	1	1439	0	1439	0	1439	0	1439
TCGA-A2	TCGA-A2	BRCA	49	FEMALE	BLACK OI	Stage IIB	Infiltrating Ductal Ca	2010	Post (prio	-18059	Dead	WITH TUMOR		1093	Distant M	Lung		802	Negative	1	1093	1	1093	1	802	1	802
TCGA-A2	TCGA-A2	BRCA	46	FEMALE	BLACK OI	Stage I	Infiltrating Ductal Ca	2009	Pre (<6 m	-16978	Alive	TUMOR F	1532						Negative	0	1532	0	1532	0	1532	0	1532
TCGA-A2	TCGA-A2	BRCA	57	FEMALE	BLACK OI	Stage IIB	Infiltrating Ductal Ca	2010	Post (prio	-21024	Alive	TUMOR F	1546						Close	0	1546	0	1546			0	1546
TCGA-A2	TCGA-A2	BRCA	49	FEMALE	WHITE	Stage IIIC	Infiltrating Lobular C	2011	Pre (<6 m	-18060	Alive	TUMOR F	222						Negative	0	222	0	222	0	222	0	222
TCGA-A2	TCGA-A2	BRCA	67	FEMALE	BLACK OI	Stage IIA	Metaplastic Carcinor	2012	Post (prio	-24779	Alive	TUMOR F	742						Negative	0	742	0	742	0	742	0	742
TCGA-A2	TCGA-A2	BRCA	46	FEMALE	WHITE	Stage IIIC	Infiltrating Lobular C	2012	Pre (<6 m	-17157	Alive	TUMOR F	648						Negative	0	648	0	648	0	648	0	648
TCGA-A2	TCGA-A2	BRCA	77	FEMALE	WHITE	Stage IIA	Mucinous Carcinoma	2012	Post (prio	-28416	Alive	TUMOR F	706						Negative	0	706	0	706	0	706	0	706
TCGA-A2	TCGA-A2	BRCA	66	FEMALE	WHITE	Stage IIA	Metaplastic Carcinor	2012	Post (prio	-24353	Alive	TUMOR F	820						Negative	0	820	0	820	0	820	0	820
TCGA-A2	TCGA-A2	BRCA	62	FEMALE	WHITE	Stage IIIA	Infiltrating Lobular C	2012	Post (prio	-22936	Alive	TUMOR F	643						Negative	0	643	0	643	0	643	0	643
TCGA-A2	TCGA-A2	BRCA	59	FEMALE	BLACK OI	Stage IIB	Infiltrating Ductal Ca	2012	Post (prio	-21762	Alive	TUMOR F	666						Negative	0	666	0	666	0	666	0	666
TCGA-A7	TCGA-A7	BRCA	66	FEMALE	WHITE	Stage I	Infiltrating Ductal Ca	2009	Indetermi	-24242	Alive	TUMOR F	1165						Negative	0	1165	0	1165	0	1165	0	1165
TCGA-A7	TCGA-A7	BRCA	57	FEMALE	WHITE	Stage IIA	Infiltrating Ductal Ca	2009	Indetermi	-20863	Alive	TUMOR F	1074						Negative	0	1074	0	1074	0	1074	0	1074
TCGA-A7	TCGA-A7	BRCA	57	FEMALE	WHITE	Stage IIA	Infiltrating Ductal Ca	2009	Indetermi	-20863	Alive	TUMOR F	1074						Negative	0	1074	0	1074	0	1074	0	1074
TCGA-A7	TCGA-A7	BRCA	78	FEMALE	WHITE	Stage IIA	Mixed Histology (ple	2010	Post (prio	-28596	Alive	TUMOR F	1043						Close	0	1043	0	1043			0	1043
TCGA-A7	TCGA-A7	BRCA	79	FEMALE	WHITE	Stage IIA	Infiltrating Ductal Ca	2009	Post (prio	-29104	Alive	TUMOR F	1079						Negative	0	1079	0	1079	0	1079	0	1079
TCGA-A7	TCGA-A7	BRCA	79	FEMALE	WHITE	Stage IIA	Infiltrating Ductal Ca	2009	Post (prio	-29104	Alive	TUMOR F	1079						Negative	0	1079	0	1079	0	1079	0	1079
TCGA-A7	TCGA-A7	BRCA	57	FEMALE	WHITE	Stage IIA	Infiltrating Ductal Ca	2010	Post (prio	-21014	Alive	TUMOR F	931						Positive	0	931	0	931			0	931
TCGA-A7	TCGA-A7	BRCA	37	FEMALE	WHITE	Stage IIA	Infiltrating Ductal Ca	2009	Pre (<6 m	-13836	Alive	TUMOR F	1139						Negative	0	1139	0	1139	0	1139	0	1139
TCGA-A7	TCGA-A7	BRCA	37	FEMALE	WHITE	Stage IIA	Infiltrating Ductal Ca	2009	Pre (<6 m	-13836	Alive	TUMOR F	1139						Negative	0	1139	0	1139	0	1139	0	1139
TCGA-A7	TCGA-A7	BRCA	62	FEMALE	WHITE	Stage IIA	Infiltrating Ductal Ca	2009	Post (prio	-22708	Alive	TUMOR F	1085						Negative	0	1085	0	1085	0	1085	0	1085
TCGA-A7	TCGA-A7	BRCA	56	FEMALE	WHITE	Stage IIA	Infiltrating Ductal Ca	2009	Post (prio	-20570	Alive	TUMOR F	1007						Close	0	1007	0	1007			0	1007
TCGA-A7	TCGA-A7	BRCA	56	FEMALE	WHITE	Stage IIA	Infiltrating Ductal Ca	2009	Post (prio	-20570	Alive	TUMOR F	1007						Close	0	1007	0	1007			0	1007
TCGA-A7	TCGA-A7	BRCA	63	FEMALE	WHITE	Stage IA	Infiltrating Ductal Ca	2009	Post (prio	-23294	Alive	TUMOR F	906						Negative	0	906	0	906	0	906	0	906
TCGA-A7	TCGA-A7	BRCA	63	FEMALE	WHITE	Stage IA	Infiltrating Ductal Ca	2009	Post (prio	-23294	Alive	TUMOR F	906						Negative	0	906	0	906	0	906	0	906
TCGA-A7	TCGA-A7	BRCA	46	FEMALE	BLACK OI	Stage IIA	Infiltrating Ductal Ca	2010	Post (prio	-17070	Alive	TUMOR F	965						Negative	0	965	0	965	0	965	0	965
TCGA-A7	TCGA-A7	BRCA	62	FEMALE	WHITE	Stage IIB	Infiltrating Ductal Ca	2010	Post (prio	-22690	Dead	WITH TUMOR		614	Distant M	Other, sp	Lung, Bor	556	Negative	1	614	1	614	1	556	1	556
TCGA-A7	TCGA-A7	BRCA	62	FEMALE	WHITE	Stage IIB	Infiltrating Ductal Ca	2010	Post (prio	-22690	Dead	WITH TUMOR		614	Distant M	Other, sp	Lung, Bor	556	Negative	1	614	1	614	1	556	1	556
TCGA-A7	TCGA-A7	BRCA	44	FEMALE	WHITE	Stage IIIA	Infiltrating Ductal Ca	2010	Pre (<6 m	-16363	Alive	TUMOR F	765						Negative	0	765	0	765	0	765	0	765
TCGA-A7	TCGA-A7	BRCA	44	FEMALE	WHITE	Stage IIIA	Infiltrating Ductal Ca	2010	Pre (<6 m	-16363	Alive	TUMOR F	765						Negative	0	765	0	765	0	765	0	765
TCGA-A7	TCGA-A7	BRCA	79	FEMALE	WHITE	Stage IIA	Infiltrating Ductal Ca	2010	Post (prio	-28969	Alive	TUMOR F	718		New Prim	Other, sp	Skin, right	586	Negative	0	718	0	718	0	718	1	586
TCGA-A7	TCGA-A7	BRCA	79	FEMALE	WHITE	Stage IIA	Infiltrating Ductal Ca	2010	Post (prio	-28969	Alive	TUMOR F	718		New Prim	Other, sp	Skin, right	586	Negative	0	718	0	718	0	718	1	586
TCGA-A7	TCGA-A7	BRCA	61	FEMALE	WHITE	Stage IIB	Infiltrating Lobular C	2010	Post (prio	-22643	Alive	TUMOR F	899		New Prim	Other, sp	Skin lesior	795	Negative	0	899	0	899	0	899	1	795
TCGA-A7	TCGA-A7	BRCA	71	FEMALE	WHITE	Stage IIIA	Infiltrating Lobular C	2010	Post (prio	-26274	Alive	TUMOR F	954						Negative	0	954	0	954	0	954	0	954
TCGA-A7	TCGA-A7	BRCA	55	FEMALE	WHITE	Stage IA	Other, specify	2010	Post (prio	-20210	Alive	TUMOR F	738						Negative	0	738	0	738	0	738	0	738
TCGA-A7	TCGA-A7	BRCA	50	FEMALE	BLACK OI	Stage IIA	Other, specify	2010	Pre (<6 m	-18362	Alive	TUMOR F	722						Negative	0	722	0	722	0	722	0	722
TCGA-A7	TCGA-A7	BRCA	72	FEMALE	WHITE	Stage IIA	Infiltrating Ductal Ca	2011	Post (prio	-26423	Alive	WITH TUM	724		Distant M	Liver		291	Negative	0	724	0	724	1	291	1	291
TCGA-A7	TCGA-A7	BRCA	65	FEMALE	WHITE	Stage IIA	Infiltrating Ductal Ca	2011	Post (prio	-23948	Alive	TUMOR F	661						Negative	0	661	0	661	0	661	0	661
TCGA-A7	TCGA-A7	BRCA	49	FEMALE	WHITE	Stage IIA	Infiltrating Ductal Ca	2011	Pre (<6 m	-17930	Alive	TUMOR F	627						Negative	0	627	0	627	0	627	0	627
TCGA-A7	TCGA-A7	BRCA	53	FEMALE	BLACK OI	Stage IIIA	Infiltrating Ductal Ca	2011	Post (prio	-19563	Alive	TUMOR F	679						Negative	0	679	0	679	0	679	0	679
TCGA-A7	TCGA-A7	BRCA	71	FEMALE	WHITE	Stage I	Mucinous Carcinoma	2011	Post (prio	-26042	Alive	TUMOR F	345						Negative	0	345	0	345	0	345	0	345
TCGA-A7	TCGA-A7	BRCA	62	FEMALE	WHITE	Stage IIA	Infiltrating Lobular C	2011	Post (prio	-22997	Alive	TUMOR F	322						Negative	0	322	0	322	0	322	0	322
TCGA-A7	TCGA-A7	BRCA	62	FEMALE	WHITE	Stage IIA	Mucinous Carcinoma	2011	Post (prio	-22672	Alive	TUMOR F	313						Negative	0	313	0	313	0	313	0	313
TCGA-A7	TCGA-A7	BRCA	63	FEMALE	WHITE	Stage IA	Infiltrating Lobular C	2011	Post (prio	-23209	Alive	TUMOR F	343						Negative	0	343	0	343	0	343	0	343
TCGA-A7	TCGA-A7	BRCA	79	FEMALE	WHITE	Stage IIA	Mucinous Carcinoma	2012	Post (prio	-29184	Alive	WITH TUM	408		Distant M	Bone		174	Negative	0	408	0	408	1	174	1	174
TCGA-A7	TCGA-A7	BRCA	70	FEMALE	WHITE	Stage IIIC	Infiltrating Lobular C	2012	Post (prio	-25678	Alive	WITH TUM	447		Locoregic	Other, sp	Left Breas	419	Negative	0	447	0	447	1	419	1	419
TCGA-A7	TCGA-A7	BRCA	50	FEMALE	WHITE	Stage IIIC	Infiltrating Lobular C	2012	Pre (<6 m	-18296	Alive	TUMOR F	364						Negative	0	364	0	364	0	364	0	364
TCGA-A7	TCGA-A7	BRCA	40	FEMALE	WHITE	Stage IIB	Infiltrating Lobular C	2012	Pre (<6 m	-14906	Alive	TUMOR F	454						Negative	0	454	0	454	0	454	0	454
TCGA-A7	TCGA-A7	BRCA	56	FEMALE	WHITE	Stage IIIA	Infiltrating Lobular C	2012	Post (prio	-20539	Alive	TUMOR F	418						Negative	0	418	0	418	0	418	0	418
TCGA-A7	TCGA-A7	BRCA	62	FEMALE	WHITE	Stage IIB	Infiltrating Lobular C	2012	Post (prio	-23008	Alive	TUMOR F	446						Negative	0	446	0	446	0	446	0	446
TCGA-A7	TCGA-A7	BRCA	52	FEMALE	BLACK OI	Stage IIA	Infiltrating Ductal Ca	2012	Post (prio	-19131	Alive	TUMOR F	441						Negative	0	441	0	441	0	441	0	441
TCGA-A7	TCGA-A7	BRCA	54	FEMALE	BLACK OI	Stage IIA	Infiltrating Ductal Ca	2011	Post (prio	-20080	Alive	TUMOR F	644						Negative	0	644	0	644	0	644	0	644
TCGA-A7	TCGA-A7	BRCA	54	FEMALE	BLACK OI	Stage IIA	Infiltrating Ductal Ca	2012	Post (prio	-19985	Alive	TUMOR F	545						Positive	0	545	0	545			0	545
TCGA-A7	TCGA-A7	BRCA	84	FEMALE	BLACK OI	Stage IIA	Infiltrating Ductal Ca	2012	Post (prio	-30783	Alive	TUMOR F	448						Negative	0	448	0	448	0	448	0	448
TCGA-A7	TCGA-A7	BRCA	62	FEMALE	BLACK OI	Stage IIA	Infiltrating Carcinom	2012	Post (prio	-22966	Alive	TUMOR F	368						Negative	0	368	0	368	0	368	0	368
TCGA-A7	TCGA-A7	BRCA	47	FEMALE	BLACK OI	Stage IIA	Infiltrating Ductal Ca	2012	Peri (6-12	-17529	Alive	TUMOR F	326						Negative	0	326	0	326	0	326	0	326
TCGA-A7	TCGA-A7	BRCA	48	FEMALE	WHITE	Stage IIIC	Infiltrating Lobular C	2012	Pre (<6 m	-17683	Alive	TUMOR F	336						[Unknown]	0	336	0	336			0	336
TCGA-A7	TCGA-A7	BRCA	51	FEMALE	BLACK OI	Stage IIA	Infiltrating Ductal Ca	2013	Peri (6-12	-18695	Alive	TUMOR F	313						Negative	0	313	0	313	0	313	0	313
TCGA-A7	TCGA-A7	BRCA	48	FEMALE	BLACK OI	Stage IIA	Infiltrating Ductal Ca	2013	Post (prio	-17539	Alive	TUMOR F	285						Close	0	285	0	285			0	285
TCGA-A7	TCGA-A7	BRCA	68	FEMALE	BLACK OI	Stage IIA	Infiltrating Ductal Ca	2013	Post (prio	-25066	Alive	TUMOR F	317						Negative	0	317	0	317	0	317	0	317
TCGA-A7	TCGA-A7	BRCA	48	FEMALE	BLACK OI	Stage IIB	Infiltrating Ductal Ca	2013	Post (prio	-17640	Alive	TUMOR F	266						Negative	0	266	0	266	0	266	0	266
TCGA-A8	TCGA-A8	BRCA	66	FEMALE		Stage IIIB	Infiltrating Ductal Ca	2009	Post (prio	-24288	Alive	TUMOR F	0						Negative	0	0	0	0	0	0	0	0
TCGA-A8	TCGA-A8	BRCA	60	FEMALE		Stage I	Infiltrating Ductal Ca	2009	Post (prio	-22157	Alive	TUMOR F	396						Negative	0	396	0	396	0	396	0	396
TCGA-A8	TCGA-A8	BRCA	63	FEMALE		Stage IIIA	Infiltrating Ductal Ca	2009	Post (prio	-23011	Alive	TUMOR F	396						Negative	0	396	0	396	0	396	0	396
TCGA-A8	TCGA-A8	BRCA	63	FEMALE		Stage IIIA	Infiltrating Ductal Ca	2007	Post (prio	-23164	Alive	TUMOR F	31						Negative	0	31	0	31	0	31	0	31
TCGA-A8	TCGA-A8	BRCA	69	FEMALE		Stage IIB	Infiltrating Ductal Ca	2007	Post (prio	-25477	Alive	TUMOR F	547						Negative	0	547	0	547	0	547	0	547
TCGA-A8	TCGA-A8	BRCA	75	FEMALE		Stage IIIA	Infiltrating Ductal Ca	2007	Post (prio	-27393	Alive	TUMOR F	1614						Negative	0	1614	0	1614	0	1614	0	1614
TCGA-A8	TCGA-A8	BRCA	80	FEMALE		Stage IIB	Infiltrating Ductal Ca	2008	Post (prio	-29525	Dead	TUMOR FREE		883					Negative	1	883	0	883	0	883	0	883
TCGA-A8	TCGA-A8	BRCA	77	FEMALE		Stage IIB	Infiltrating Ductal Ca	2006	Post (prio	-28338	Dead	TUMOR FREE		943					Negative	1	943	0	943	0	943	0	943
TCGA-A8	TCGA-A8	BRCA	66	FEMALE		Stage IIA	Infiltrating Ductal Ca	2008	Post (prio	-24442	Alive	TUMOR F	791					791	Negative	0	791	0	791	1	791	1	791
TCGA-A8	TCGA-A8	BRCA	84	FEMALE		Stage IIB	Infiltrating Ductal Ca	2007	Post (prio	-30984	Alive		31							0	31	0	31			0	31
TCGA-A8	TCGA-A8	BRCA	42	FEMALE		Stage IIB	Infiltrating Ductal Ca	2009	Pre (<6 m	-15369	Alive	TUMOR F	518						Negative	0	518	0	518	0	518	0	518
TCGA-A8	TCGA-A8	BRCA	66	FEMALE		Stage IIA	Infiltrating Ductal Ca	2006	Post (prio	-24230	Alive	TUMOR F	1642						Negative	0	1642	0	1642	0	1642	0	1642
TCGA-A8	TCGA-A8	BRCA	69	FEMALE		Stage IIIB	Infiltrating Ductal Ca	2007	Post (prio	-25294	Alive	TUMOR F	274						Negative	0	274	0	274	0	274	0	274
TCGA-A8	TCGA-A8	BRCA	69	FEMALE		Stage IIA	Infiltrating Ductal Ca	2007	Post (prio	-25475	Alive	TUMOR F	1308						Negative	0	1308	0	1308	0	1308	0	1308
TCGA-A8	TCGA-A8	BRCA	57	FEMALE		Stage IIA	Infiltrating Ductal Ca	2008	Post (prio	-20820	Alive	TUMOR F	1034						Negative	0	1034	0	1034	0	1034	0	1034
TCGA-A8	TCGA-A8	BRCA	81	FEMALE		Stage X	Infiltrating Ductal Ca	2008	Post (prio	-29800	Alive	TUMOR F	608						Negative	0	608	0	608	0	608	0	608
TCGA-A8	TCGA-A8	BRCA	65	FEMALE		Stage IIB	Infiltrating Ductal Ca	2009	Post (prio	-23742	Alive	TUMOR F	577						Negative	0	577	0	577	0	577	0	577
TCGA-A8	TCGA-A8	BRCA	65	FEMALE		Stage IIA	Infiltrating Ductal Ca	2009	Post (prio	-23926	Alive	TUMOR F	577						Negative	0	577	0	577	0	577	0	577
TCGA-A8	TCGA-A8	BRCA	69	FEMALE		Stage IIIA	Infiltrating Ductal Ca	2009	Post (prio	-25263	Alive	TUMOR F	426						Negative	0	426	0	426	0	426	0	426
TCGA-A8	TCGA-A8	BRCA	35	FEMALE		Stage IIB	Infiltrating Ductal Ca	2009	Pre (<6 m	-12965	Alive	TUMOR F	365						Negative	0	365	0	365	0	365	0	365
TCGA-A8	TCGA-A8	BRCA	58	FEMALE		Stage IIIA	Infiltrating Ductal Ca	2009	Post (prio	-21428	Alive	TUMOR F	975						Negative	0	975	0	975	0	975	0	975
TCGA-A8	TCGA-A8	BRCA	51	FEMALE		Stage IIA	Infiltrating Ductal Ca	2009	Post (prio	-18870	Alive	TUMOR F	304						Negative	0	304	0	304	0	304	0	304
TCGA-A8	TCGA-A8	BRCA	68	FEMALE		Stage IIB	Infiltrating Ductal Ca	2009	Post (prio	-24929	Alive	TUMOR F	334						Negative	0	334	0	334	0	334	0	334
TCGA-A8	TCGA-A8	BRCA	80	FEMALE		Stage IIIC	Infiltrating Ductal Ca	2009		-29251	Alive		273						Positive	0	273	0	273			0	273
TCGA-A8	TCGA-A8	BRCA	73	FEMALE		Stage IIA	Mixed Histology (ple	2009	Post (prio	-26998	Alive	WITH TUM	243						Positive	0	243	0	243			0	243
TCGA-A8	TCGA-A8	BRCA	66	FEMALE		Stage IIIA	Infiltrating Ductal Ca	2009	Post (prio	-24411	Alive	TUMOR F	760						Negative	0	760	0	760	0	760	0	760
TCGA-A8	TCGA-A8	BRCA	76	FEMALE		Stage IV	Infiltrating Ductal Ca	2009	Post (prio	-27942	Alive	WITH TUM	304						Positive	0	304	0	304			0	304
TCGA-A8	TCGA-A8	BRCA	85	FEMALE		Stage IIA	Infiltrating Ductal Ca	2008	Post (prio	-31351	Alive	TUMOR F	1371						Negative	0	1371	0	1371	0	1371	0	1371
TCGA-A8	TCGA-A8	BRCA	80	FEMALE		Stage IIA	Infiltrating Ductal Ca	2008	Post (prio	-29555	Alive	TUMOR F	0						Negative	0	0	0	0	0	0	0	0
TCGA-A8	TCGA-A8	BRCA	58	FEMALE		Stage IIB	Infiltrating Ductal Ca	2008	Post (prio	-21338	Alive	TUMOR F	549						Negative	0	549	0	549	0	549	0	549
TCGA-A8	TCGA-A8	BRCA	67	FEMALE		Stage IIB	Infiltrating Ductal Ca	2008	Post (prio	-24746	Alive	TUMOR F	0						Negative	0	0	0	0	0	0	0	0
TCGA-A8	TCGA-A8	BRCA	81	FEMALE		Stage IIB	Infiltrating Ductal Ca	2007	Post (prio	-29858	Alive	WITH TUM	458						Positive	0	458	0	458			0	458
TCGA-A8	TCGA-A8	BRCA	44	MALE		Stage IIB	Infiltrating Ductal Ca	2008		-16377	Alive	TUMOR F	1124						Negative	0	1124	0	1124	0	1124	0	1124
TCGA-A8	TCGA-A8	BRCA	59	FEMALE		Stage IIA	Infiltrating Ductal Ca	2009	Post (prio	-21731	Alive	TUMOR F	396						Negative	0	396	0	396	0	396	0	396
TCGA-A8	TCGA-A8	BRCA	89	FEMALE		Stage I	Infiltrating Ductal Ca	2007	Post (prio	-32750	Alive	TUMOR F	30						Negative	0	30	0	30	0	30	0	30
TCGA-A8	TCGA-A8	BRCA	52	FEMALE		Stage IIA	Infiltrating Ductal Ca	2008	Pre (<6 m	-19114	Alive	TUMOR F	1156						Negative	0	1156	0	1156	0	1156	0	1156
TCGA-A8	TCGA-A8	BRCA	65	FEMALE		Stage IIA	Infiltrating Ductal Ca	2008	Post (prio	-24017	Alive	TUMOR F	881						Negative	0	881	0	881	0	881	0	881
TCGA-A8	TCGA-A8	BRCA	59	FEMALE		Stage IIIC	Infiltrating Ductal Ca	2008	Post (prio	-21641	Alive	TUMOR F	1004						Negative	0	1004	0	1004	0	1004	0	1004
TCGA-A8	TCGA-A8	BRCA	41	FEMALE		Stage IIA	Infiltrating Ductal Ca	2009	Pre (<6 m	-14976	Alive	TUMOR F	607						Negative	0	607	0	607	0	607	0	607
TCGA-A8	TCGA-A8	BRCA	66	FEMALE		Stage IIA	Infiltrating Ductal Ca	2007	Post (prio	-24137	Alive	TUMOR F	0						Negative	0	0	0	0	0	0	0	0
TCGA-A8	TCGA-A8	BRCA	53	FEMALE		Stage IIA	Infiltrating Ductal Ca	2009	Post (prio	-19389	Alive	TUMOR F	365						Negative	0	365	0	365	0	365	0	365
TCGA-A8	TCGA-A8	BRCA	52	FEMALE		Stage IV	Infiltrating Ductal Ca	2005	Post (prio	-19266	Dead	WITH TUMOR		1127					Positive	1	1127	1	1127			1	1127
TCGA-A8	TCGA-A8	BRCA	89	FEMALE		Stage IIIA	Infiltrating Ductal Ca	2008	Post (prio	-32842	Dead	TUMOR FREE		304					Negative	1	304	0	304	0	304	0	304
TCGA-A8	TCGA-A8	BRCA	45	FEMALE		Stage IV	Infiltrating Ductal Ca	2008	Pre (<6 m	-16681	Alive	WITH TUM	943					762	Positive	0	943	0	943			1	762
TCGA-A8	TCGA-A8	BRCA	70	FEMALE		Stage IIIA	Infiltrating Ductal Ca	2008	Post (prio	-25598	Alive	TUMOR F	943						Negative	0	943	0	943	0	943	0	943
TCGA-A8	TCGA-A8	BRCA	52	FEMALE		Stage IIB	Infiltrating Ductal Ca	2008	Post (prio	-19298	Alive	TUMOR F	30						Negative	0	30	0	30	0	30	0	30
TCGA-A8	TCGA-A8	BRCA	71	FEMALE		Stage IIA	Infiltrating Ductal Ca	2008	Post (prio	-25994	Alive	TUMOR F	1004						Negative	0	1004	0	1004	0	1004	0	1004
TCGA-A8	TCGA-A8	BRCA	64	FEMALE		Stage IV	Infiltrating Ductal Ca	2002	Post (prio	-23560	Dead	WITH TUMOR		3409				2557	Positive	1	3409	1	3409			1	2557
TCGA-A8	TCGA-A8	BRCA	43	FEMALE		Stage IIIC	Infiltrating Ductal Ca	2007	Pre (<6 m	-15705	Alive	TUMOR F	1308					366	Negative	0	1308	0	1308	1	366	1	366
TCGA-A8	TCGA-A8	BRCA	76	FEMALE		[Discrepancy]	Infiltrating Ductal Ca	2004	Post (prio	-28003	Alive	TUMOR F	1217						Negative	0	1217	0	1217	0	1217	0	1217
TCGA-A8	TCGA-A8	BRCA	74	FEMALE		Stage IIA	Infiltrating Ductal Ca	2008	Post (prio	-27121	Alive	TUMOR F	0						Negative	0	0	0	0	0	0	0	0
TCGA-A8	TCGA-A8	BRCA	61	FEMALE		Stage IIA	Infiltrating Ductal Ca	2008	Post (prio	-22554	Alive	TUMOR F	1004					243	Negative	0	1004	0	1004	1	243	1	243
TCGA-A8	TCGA-A8	BRCA	48	FEMALE		Stage IIIA	Infiltrating Ductal Ca	2008	Pre (<6 m	-17807	Alive	TUMOR F	942						Negative	0	942	0	942	0	942	0	942
TCGA-A8	TCGA-A8	BRCA	61	FEMALE		Stage IIA	Infiltrating Ductal Ca	2009	Post (prio	-22373	Alive	TUMOR F	546						Negative	0	546	0	546	0	546	0	546
TCGA-A8	TCGA-A8	BRCA	75	FEMALE		Stage IIA	Infiltrating Ductal Ca	2009	Post (prio	-27729	Alive	TUMOR F	0						Negative	0	0	0	0	0	0	0	0
TCGA-A8	TCGA-A8	BRCA	45	FEMALE		Stage I	Infiltrating Ductal Ca	2007	Pre (<6 m	-16742	Alive	TUMOR F	1277						Negative	0	1277	0	1277	0	1277	0	1277
TCGA-A8	TCGA-A8	BRCA	73	FEMALE		Stage IIA	Infiltrating Ductal Ca	2009	Post (prio	-26723	Alive	TUMOR F	0						Negative	0	0	0	0	0	0	0	0
TCGA-A8	TCGA-A8	BRCA	65	FEMALE		Stage IIB	Infiltrating Ductal Ca	2009	Post (prio	-23741	Alive	TUMOR F	365						Negative	0	365	0	365	0	365	0	365
TCGA-A8	TCGA-A8	BRCA	76	FEMALE		Stage X	Infiltrating Ductal Ca	2009	Post (prio	-28063	Alive		304							0	304	0	304			0	304
TCGA-A8	TCGA-A8	BRCA	40	FEMALE		Stage X	Infiltrating Ductal Ca	2009	Pre (<6 m	-14883	Alive	WITH TUM	304						Positive	0	304	0	304			0	304
TCGA-A8	TCGA-A8	BRCA	58	FEMALE		Stage IIIB	Infiltrating Ductal Ca	2009	Post (prio	-21520	Alive	TUMOR F	365						Negative	0	365	0	365	0	365	0	365
TCGA-A8	TCGA-A8	BRCA	69	FEMALE		Stage X	Infiltrating Ductal Ca	2009	Post (prio	-25355	Alive		31							0	31	0	31			0	31
TCGA-A8	TCGA-A8	BRCA	47	FEMALE		Stage IIB	Infiltrating Ductal Ca	2007	Post (prio	-17440	Alive	TUMOR F	1522						Negative	0	1522	0	1522	0	1522	0	1522
TCGA-A8	TCGA-A8	BRCA	73	FEMALE		Stage IIIB	Infiltrating Ductal Ca	2008	Post (prio	-26724	Alive	WITH TUM	1492						Positive	0	1492	0	1492			0	1492
TCGA-A8	TCGA-A8	BRCA	79	FEMALE		Stage IIIC	Infiltrating Ductal Ca	2008	Post (prio	-29006	Alive	TUMOR F	0						Negative	0	0	0	0	0	0	0	0
TCGA-A8	TCGA-A8	BRCA	84	FEMALE		Stage IIA	Infiltrating Ductal Ca	2008	Post (prio	-30711	Alive	TUMOR F	1371						Negative	0	1371	0	1371	0	1371	0	1371
TCGA-A8	TCGA-A8	BRCA	68	FEMALE		Stage IIA	Infiltrating Ductal Ca	2008	Post (prio	-24960	Alive	TUMOR F	912						Negative	0	912	0	912	0	912	0	912
TCGA-A8	TCGA-A8	BRCA	75	FEMALE		Stage IIIC	Infiltrating Ductal Ca	2009	Post (prio	-27455	Alive	TUMOR F	1006						Negative	0	1006	0	1006	0	1006	0	1006
TCGA-A8	TCGA-A8	BRCA	57	FEMALE		Stage IIIC	Infiltrating Ductal Ca	2006	Post (prio	-21002	Alive	TUMOR F	31						Negative	0	31	0	31	0	31	0	31
TCGA-A8	TCGA-A8	BRCA	83	FEMALE		Stage IIIB	Infiltrating Ductal Ca	2008	Post (prio	-30528	Alive	WITH TUM	761						Positive	0	761	0	761			0	761
TCGA-A8	TCGA-A8	BRCA	82	FEMALE		Stage IIB	Infiltrating Ductal Ca	2009	Post (prio	-30196	Alive	TUMOR F	273						Negative	0	273	0	273	0	273	0	273
TCGA-A8	TCGA-A8	BRCA	68	FEMALE		[Discrepancy]	Infiltrating Lobular C	2009	Post (prio	-25110	Alive	TUMOR F	579						Negative	0	579	0	579	0	579	0	579
TCGA-A8	TCGA-A8	BRCA	51	FEMALE		Stage IIA	Infiltrating Lobular C	2009	Pre (<6 m	-18628	Alive	TUMOR F	457						Negative	0	457	0	457	0	457	0	457
TCGA-A8	TCGA-A8	BRCA	70	FEMALE		Stage IIIB	Infiltrating Lobular C	2007	Post (prio	-25659	Alive		30							0	30	0	30			0	30
TCGA-A8	TCGA-A8	BRCA	62	FEMALE		Stage IIIC	Infiltrating Lobular C	2009	Post (prio	-22858	Dead			426						1	426		426			0	426
TCGA-A8	TCGA-A8	BRCA	83	FEMALE		Stage IIB	Infiltrating Lobular C	2008	Post (prio	-30590	Alive	WITH TUM	0						Positive	0	0	0	0			0	0
TCGA-A8	TCGA-A8	BRCA	84	FEMALE		Stage IIA	Infiltrating Lobular C	2009	Post (prio	-30742	Alive	TUMOR F	365						Negative	0	365	0	365	0	365	0	365
TCGA-A8	TCGA-A8	BRCA	66	FEMALE		Stage IIA	Infiltrating Lobular C	2009	Post (prio	-24380	Alive	TUMOR F	579						Negative	0	579	0	579	0	579	0	579
TCGA-A8	TCGA-A8	BRCA	73	FEMALE		Stage IIA	Infiltrating Lobular C	2009	Post (prio	-26693	Alive	TUMOR F	396						Negative	0	396	0	396	0	396	0	396
TCGA-A8	TCGA-A8	BRCA	64	FEMALE		Stage IIIC	Infiltrating Lobular C	2009	Post (prio	-23404	Alive	TUMOR F	640						Negative	0	640	0	640	0	640	0	640
TCGA-A8	TCGA-A8	BRCA	57	FEMALE		Stage IIB	Infiltrating Lobular C	2009	Post (prio	-20940	Alive	TUMOR F	30						Negative	0	30	0	30	0	30	0	30
TCGA-A8	TCGA-A8	BRCA	80	FEMALE		Stage IIA	Infiltrating Ductal Ca	2009	Post (prio	-29370	Alive	TUMOR F	822						Negative	0	822	0	822	0	822	0	822
TCGA-A8	TCGA-A8	BRCA	54	FEMALE		Stage IIA	Infiltrating Lobular C	2009	Post (prio	-19814	Alive	TUMOR F	518						Negative	0	518	0	518	0	518	0	518
TCGA-A8	TCGA-A8	BRCA	83	FEMALE		Stage I	Mixed Histology (ple	2007	Post (prio	-30407	Alive	TUMOR F	1157						Negative	0	1157	0	1157	0	1157	0	1157
TCGA-AC	TCGA-AC	BRCA	62	FEMALE	WHITE	Stage IIB	Infiltrating Ductal Ca	2010	Post (prio	-22751	Alive	TUMOR F	585						Negative	0	585	0	585	0	585	0	585
TCGA-AC	TCGA-AC	BRCA	50	FEMALE	WHITE	Stage IIB	Infiltrating Ductal Ca	2010		-18500	Alive	TUMOR F	698						Negative	0	698	0	698	0	698	0	698
TCGA-AC	TCGA-AC	BRCA	76	FEMALE	WHITE	Stage IIA	Infiltrating Lobular C	2006	Post (prio	-27934	Alive	TUMOR F	2248						Negative	0	2248	0	2248	0	2248	0	2248
TCGA-AC	TCGA-AC	BRCA	90	FEMALE	WHITE	Stage IIA	Infiltrating Ductal Ca	2009	Post (prio	-32873	Dead	WITH TUMOR		174					Negative	1	174	1	174			1	174
TCGA-AC	TCGA-AC	BRCA	90	FEMALE	WHITE	Stage IIA	Infiltrating Ductal Ca	2009	Post (prio	-32873	Dead	WITH TUMOR		174					Negative	1	174	1	174			1	174
TCGA-AC	TCGA-AC	BRCA	84	FEMALE	WHITE	Stage IIB	Infiltrating Lobular C	2011	Post (prio	-31003	Alive	TUMOR F	677		New Prim	Other, sp	Rectum	654	Negative	0	677	0	677	0	677	1	654
TCGA-AC	TCGA-AC	BRCA	78	FEMALE	BLACK OI	Stage IIIA	Infiltrating Ductal Ca	2006	Post (prio	-28625	Alive	TUMOR F	2222						Negative	0	2222	0	2222	0	2222	0	2222
TCGA-AC	TCGA-AC	BRCA	41	FEMALE	BLACK OI	Stage IIB	Infiltrating Ductal Ca	2005		-15212	Alive	TUMOR F	3022						Negative	0	3022	0	3022	0	3022	0	3022
TCGA-AC	TCGA-AC	BRCA	65	FEMALE	WHITE	Stage IIA	Infiltrating Lobular C	2009		-23851	Alive	TUMOR F	1234						Negative	0	1234	0	1234	0	1234	0	1234
TCGA-AC	TCGA-AC	BRCA	65	FEMALE	WHITE	Stage IIA	Infiltrating Lobular C	2009		-23851	Alive	TUMOR F	1234						Negative	0	1234	0	1234	0	1234	0	1234
TCGA-AC	TCGA-AC	BRCA	62	FEMALE	WHITE	Stage IIIC	Infiltrating Lobular C	2005	Post (prio	-22746	Dead	WITH TUMOR		2636	Distant M	Bone		766	Negative	1	2636	1	2636	1	766	1	766
TCGA-AC	TCGA-AC	BRCA	40	FEMALE	ASIAN	Stage IIB	Infiltrating Lobular C	2006			Alive	TUMOR F	2759						Negative	0	2759	0	2759	0	2759	0	2759
TCGA-AC	TCGA-AC	BRCA	40	FEMALE	ASIAN	Stage IIB	Infiltrating Lobular C	2006			Alive	TUMOR F	2759						Negative	0	2759	0	2759	0	2759	0	2759
TCGA-AC	TCGA-AC	BRCA	79	FEMALE	WHITE	Stage IIB	Infiltrating Lobular C	2007	Post (prio	-29211	Alive	TUMOR F	1853						Negative	0	1853	0	1853	0	1853	0	1853
TCGA-AC	TCGA-AC	BRCA	79	FEMALE	WHITE	Stage IIB	Infiltrating Lobular C	2007	Post (prio	-29211	Alive	TUMOR F	1853						Negative	0	1853	0	1853	0	1853	0	1853
TCGA-AC	TCGA-AC	BRCA	45	FEMALE	WHITE	Stage IIIC	Infiltrating Lobular C	2006		-16627	Alive	TUMOR F	2650						Negative	0	2650	0	2650	0	2650	0	2650
TCGA-AC	TCGA-AC	BRCA	87	FEMALE	WHITE	Stage IIB	Infiltrating Lobular C	2004	Post (prio	-31805	Dead	WITH TUMOR		792	Distant M	Other, sp	Bone Mar	780	Negative	1	792	1	792	1	780	1	780
TCGA-AC	TCGA-AC	BRCA	87	FEMALE	WHITE	Stage IIB	Infiltrating Lobular C	2004	Post (prio	-31805	Dead	WITH TUMOR		792	Distant M	Other, sp	Bone Mar	780	Negative	1	792	1	792	1	780	1	780
TCGA-AC	TCGA-AC	BRCA	65	FEMALE	WHITE	Stage IIB	Infiltrating Lobular C	2005	Post (prio	-23928	Alive	TUMOR F	2255						Negative	0	2255	0	2255	0	2255	0	2255
TCGA-AC	TCGA-AC	BRCA	58	FEMALE	WHITE	Stage IIB	Metaplastic Carcinor	2011	Post (prio	-21488	Alive	TUMOR F	1005						Negative	0	1005	0	1005	0	1005	0	1005
TCGA-AC	TCGA-AC	BRCA	76	FEMALE	WHITE	Stage IIIA	Infiltrating Lobular C	2011	Post (prio	-27865	Alive	TUMOR F	588						Negative	0	588	0	588	0	588	0	588
TCGA-AC	TCGA-AC	BRCA	48	FEMALE	WHITE	Stage IIIB	Metaplastic Carcinor	2011	Peri (6-12	-17569	Dead	WITH TUMOR		446	Distant M	Bone		237	Negative	1	446	1	446	1	237	1	237
TCGA-AC	TCGA-AC	BRCA	46	FEMALE	BLACK OI	Stage IIIA	Infiltrating Lobular C	2011		-16983	Alive	TUMOR F	987						Negative	0	987	0	987	0	987	0	987
TCGA-AC	TCGA-AC	BRCA	79	FEMALE	WHITE	Stage IIIC	Infiltrating Lobular C	2011	Post (prio	-29014	Dead	TUMOR FREE		197					Negative	1	197	0	197	0	197	0	197
TCGA-AC	TCGA-AC	BRCA	87	FEMALE	WHITE	Stage IIB	Infiltrating Lobular C	2011	Post (prio	-32061	Alive	TUMOR F	496						Negative	0	496	0	496	0	496	0	496
TCGA-AC	TCGA-AC	BRCA	68	FEMALE	WHITE	Stage IIB	Infiltrating Lobular C	2011	Post (prio	-25186	Alive	TUMOR F	451						Negative	0	451	0	451	0	451	0	451
TCGA-AC	TCGA-AC	BRCA	79	FEMALE	WHITE	Stage IIB	Infiltrating Lobular C	2011	Post (prio	-29113	Alive	TUMOR F	675						Negative	0	675	0	675	0	675	0	675
TCGA-AC	TCGA-AC	BRCA	54	FEMALE	WHITE	Stage IA	Infiltrating Lobular C	2011	Peri (6-12	-19723	Alive	TUMOR F	734						Close	0	734	0	734			0	734
TCGA-AC	TCGA-AC	BRCA	50	FEMALE	WHITE	Stage IIIA	Infiltrating Lobular C	2011	Peri (6-12	-18535	Alive	TUMOR F	762						Negative	0	762	0	762	0	762	0	762
TCGA-AC	TCGA-AC	BRCA	75	FEMALE	WHITE	Stage IIB	Infiltrating Lobular C	2012	Post (prio	-27421	Alive	TUMOR F	456						Negative	0	456	0	456	0	456	0	456
TCGA-AC	TCGA-AC	BRCA	65	FEMALE	BLACK OI	Stage IIA	Infiltrating Lobular C	2011	[Not Evalu	-24029	Alive	TUMOR F	504						Close	0	504	0	504			0	504
TCGA-AC	TCGA-AC	BRCA	90	FEMALE	WHITE	Stage IIIA	Infiltrating Lobular C	2012	Post (prio	-32872	Alive	TUMOR F	602						Negative	0	602	0	602	0	602	0	602
TCGA-AC	TCGA-AC	BRCA	66	FEMALE	WHITE	Stage IIB	Infiltrating Lobular C	2012	Post (prio	-24327	Alive	TUMOR F	471						Negative	0	471	0	471	0	471	0	471
TCGA-AC	TCGA-AC	BRCA	74	FEMALE	WHITE	Stage IIIC	Infiltrating Lobular C	2012	Post (prio	-27143	Alive	TUMOR F	707						Negative	0	707	0	707	0	707	0	707
TCGA-AC	TCGA-AC	BRCA	66	FEMALE	WHITE	Stage IIA	Other, specify	2012	Post (prio	-24439	Alive	TUMOR F	754						Negative	0	754	0	754	0	754	0	754
TCGA-AC	TCGA-AC	BRCA	63	FEMALE	WHITE	Stage IIB	Infiltrating Lobular C	2012	Post (prio	-23284	Alive	TUMOR F	890						Negative	0	890	0	890	0	890	0	890
TCGA-AC	TCGA-AC	BRCA	76	FEMALE	BLACK OI	Stage IIB	Infiltrating Ductal Ca	2012		-27964	Alive	TUMOR F	511						Negative	0	511	0	511	0	511	0	511
TCGA-AC	TCGA-AC	BRCA	88	FEMALE	WHITE	Stage IB	Other, specify	2012		-32375	Alive	TUMOR F	442						Negative	0	442	0	442	0	442	0	442
TCGA-AC	TCGA-AC	BRCA	74	FEMALE	WHITE	Stage IIA	Infiltrating Lobular C	2012		-27160	Alive	TUMOR F	588						Negative	0	588	0	588	0	588	0	588
TCGA-AC	TCGA-AC	BRCA	74	FEMALE	BLACK OI	Stage IIB	Infiltrating Ductal Ca	2013		-27191	Alive	TUMOR F	455						Negative	0	455	0	455	0	455	0	455
TCGA-AC	TCGA-AC	BRCA	58	MALE	BLACK OI	Stage IV	Infiltrating Ductal Ca	2013	[Not Evalu	-21389	Dead	WITH TUMOR		348					Negative	1	348	1	348			1	348
TCGA-AC	TCGA-AC	BRCA	72	FEMALE	BLACK OI	Stage IIA	Infiltrating Ductal Ca	2013	Post (prio	-26627	Alive	TUMOR F	417						Negative	0	417	0	417	0	417	0	417
TCGA-AC	TCGA-AC	BRCA	79	FEMALE	WHITE	Stage IIB	Infiltrating Lobular C	2012	Post (prio	-28876	Alive	TUMOR F	530						Positive	0	530	0	530			0	530
TCGA-AC	TCGA-AC	BRCA	47	FEMALE	WHITE	Stage IIB	Infiltrating Lobular C	2012		-17521	Alive	TUMOR F	568						Negative	0	568	0	568	0	568	0	568
TCGA-AC	TCGA-AC	BRCA	73	FEMALE	BLACK OI	Stage IIA	Infiltrating Ductal Ca	2013	Post (prio	-26896	Alive	TUMOR F	413						Negative	0	413	0	413	0	413	0	413
TCGA-AC	TCGA-AC	BRCA	49	FEMALE	WHITE	Stage IIIC	Infiltrating Lobular C	2013	Peri (6-12	-17969	Alive	TUMOR F	373						Negative	0	373	0	373	0	373	0	373
TCGA-AC	TCGA-AC	BRCA	49	FEMALE	WHITE	Stage IIIC	Infiltrating Lobular C	2013	Peri (6-12	-17969	Alive	TUMOR F	373						Negative	0	373	0	373	0	373	0	373
TCGA-AC	TCGA-AC	BRCA	43	FEMALE	BLACK OI	Stage IIB	Infiltrating Ductal Ca	2011	[Unknowr	-15996	Alive	TUMOR F	51						Negative	0	51	0	51	0	51	0	51
TCGA-AC	TCGA-AC	BRCA	51	FEMALE	BLACK OI	Stage IIA	Infiltrating Ductal Ca	2013	[Unknowr	-18806	Alive	TUMOR F	250						Negative	0	250	0	250	0	250	0	250
TCGA-AC	TCGA-AC	BRCA	56	FEMALE	WHITE	Stage IIB	Metaplastic Carcinor	2013	Post (prio	-20479	Alive	TUMOR F	1						Negative	0	1	0	1	0	1	0	1
TCGA-AC	TCGA-AC	BRCA	72	FEMALE	BLACK OI	Stage IA	Infiltrating Ductal Ca	2011	Post (prio	-26550	Alive	TUMOR F	614						Negative	0	614	0	614	0	614	0	614
TCGA-AC	TCGA-AC	BRCA	72	FEMALE	BLACK OI	Stage IIB	Infiltrating Ductal Ca	2011	Post (prio	-26535	Alive	TUMOR F	34						Negative	0	34	0	34	0	34	0	34
TCGA-AC	TCGA-AC	BRCA	75	FEMALE	WHITE	Stage IA	Mucinous Carcinoma	2013	Post (prio	-27605	Alive	TUMOR F	40						Negative	0	40	0	40	0	40	0	40
TCGA-AC	TCGA-AC	BRCA	71	FEMALE	WHITE	Stage IIA	Infiltrating Lobular C	2013	Post (prio	-26248	Alive	TUMOR F	70						Negative	0	70	0	70	0	70	0	70
TCGA-AN	TCGA-AN	BRCA	74	FEMALE	WHITE	Stage IIA	Infiltrating Ductal Ca	2010	Post (prio	-27163	Alive	TUMOR F	10						Negative	0	10	0	10	0	10	0	10
TCGA-AN	TCGA-AN	BRCA	66	FEMALE	WHITE	Stage IIA	Infiltrating Ductal Ca	2010	Post (prio	-24232	Alive	TUMOR F	10						Negative	0	10	0	10	0	10	0	10
TCGA-AN	TCGA-AN	BRCA	29	FEMALE	WHITE	Stage IIB	Infiltrating Ductal Ca	2010	Pre (<6 m	-10812	Alive	TUMOR F	7						Negative	0	7	0	7	0	7	0	7
TCGA-AN	TCGA-AN	BRCA	68	FEMALE	WHITE	Stage IIA	Infiltrating Ductal Ca	2010	Post (prio	-25158	Alive	TUMOR F	10						Negative	0	10	0	10	0	10	0	10
TCGA-AN	TCGA-AN	BRCA	62	FEMALE	WHITE	Stage IIA	Infiltrating Ductal Ca	2008	Post (prio	-22710	Alive	TUMOR F	19						Positive	0	19	0	19			0	19
TCGA-AN	TCGA-AN	BRCA	36	FEMALE	WHITE	Stage IIIA	Infiltrating Ductal Ca	2008	Peri (6-12	-13445	Alive	TUMOR F	90						Positive	0	90	0	90			0	90
TCGA-AN	TCGA-AN	BRCA	51	FEMALE	WHITE	Stage IIB	Infiltrating Ductal Ca	2008	Post (prio	-18851	Alive	TUMOR F	54						Positive	0	54	0	54			0	54
TCGA-AN	TCGA-AN	BRCA	58	FEMALE	WHITE	Stage IIB	Infiltrating Ductal Ca	2008	Post (prio	-21426	Alive	TUMOR F	52						Positive	0	52	0	52			0	52
TCGA-AN	TCGA-AN	BRCA	79	FEMALE	WHITE	Stage IIB	Infiltrating Ductal Ca	2010	Post (prio	-29131	Alive	TUMOR F	303						Negative	0	303	0	303	0	303	0	303
TCGA-AN	TCGA-AN	BRCA	76	FEMALE	WHITE	Stage IIA	Infiltrating Ductal Ca	2010	Post (prio	-27977	Alive	TUMOR F	224						Negative	0	224	0	224	0	224	0	224
TCGA-AN	TCGA-AN	BRCA	41	FEMALE	WHITE	Stage IIIB	Infiltrating Ductal Ca	2010	Pre (<6 m	-15286	Alive	TUMOR F	227						Negative	0	227	0	227	0	227	0	227
TCGA-AN	TCGA-AN	BRCA	56	FEMALE	WHITE	Stage IIA	Infiltrating Ductal Ca	2010	Post (prio	-20713	Alive	TUMOR F	5						Negative	0	5	0	5	0	5	0	5
TCGA-AN	TCGA-AN	BRCA	55	FEMALE	WHITE	Stage IIA	Infiltrating Ductal Ca	2010	Pre (<6 m	-20152	Alive	TUMOR F	10						Negative	0	10	0	10	0	10	0	10
TCGA-AN	TCGA-AN	BRCA	70	FEMALE	WHITE	Stage IIIA	Infiltrating Ductal Ca	2010	Post (prio	-25658	Alive	TUMOR F	10						Negative	0	10	0	10	0	10	0	10
TCGA-AN	TCGA-AN	BRCA	62	FEMALE	WHITE	Stage IIA	Infiltrating Ductal Ca	2010	Post (prio	-22798	Alive	TUMOR F	10						Negative	0	10	0	10	0	10	0	10
TCGA-AN	TCGA-AN	BRCA	71	FEMALE	WHITE	Stage IIA	Infiltrating Ductal Ca	2010	Post (prio	-26007	Alive	TUMOR F	196						Negative	0	196	0	196	0	196	0	196
TCGA-AN	TCGA-AN	BRCA	32	FEMALE	WHITE	Stage IA	Infiltrating Ductal Ca	2010	Pre (<6 m	-11929	Alive	TUMOR F	172						Negative	0	172	0	172	0	172	0	172
TCGA-AN	TCGA-AN	BRCA	59	FEMALE	WHITE	Stage IV	Infiltrating Ductal Ca	2010	Peri (6-12	-21910	Alive	TUMOR F	242						Negative	0	242	0	242			0	242
TCGA-AN	TCGA-AN	BRCA	88	FEMALE	WHITE	Stage IIIB	Infiltrating Ductal Ca	2010	Post (prio	-32462	Alive	TUMOR F	213						Negative	0	213	0	213	0	213	0	213
TCGA-AN	TCGA-AN	BRCA	62	FEMALE	WHITE	Stage IIA	Infiltrating Ductal Ca	2010	Post (prio	-22805	Alive	TUMOR F	231						Negative	0	231	0	231	0	231	0	231
TCGA-AN	TCGA-AN	BRCA	61	FEMALE	WHITE	Stage IA	Infiltrating Lobular C	2010	Post (prio	-22629	Alive	TUMOR F	218						Negative	0	218	0	218	0	218	0	218
TCGA-AN	TCGA-AN	BRCA	55	FEMALE	WHITE	Stage IA	Infiltrating Lobular C	2010	Peri (6-12	-20352	Alive	TUMOR F	210						Negative	0	210	0	210	0	210	0	210
TCGA-AN	TCGA-AN	BRCA	63	FEMALE	WHITE	Stage IIB	Infiltrating Ductal Ca	2010	Post (prio	-23300	Alive	TUMOR F	214						Negative	0	214	0	214	0	214	0	214
TCGA-AN	TCGA-AN	BRCA	58	FEMALE	WHITE	Stage IIA	Infiltrating Ductal Ca	2010	Post (prio	-21479	Alive	TUMOR F	10						Negative	0	10	0	10	0	10	0	10
TCGA-AN	TCGA-AN	BRCA	67	FEMALE	WHITE	Stage IIIA	Infiltrating Ductal Ca	2010	Post (prio	-24692	Alive	TUMOR F	11						Negative	0	11	0	11	0	11	0	11
TCGA-AN	TCGA-AN	BRCA	52	FEMALE	WHITE	Stage IIA	Infiltrating Ductal Ca	2010	Post (prio	-19309	Alive	TUMOR F	10						Negative	0	10	0	10	0	10	0	10
TCGA-AN	TCGA-AN	BRCA	55	FEMALE	WHITE	Stage IA	Infiltrating Ductal Ca	2010	Post (prio	-20347	Alive	TUMOR F	10						Negative	0	10	0	10	0	10	0	10
TCGA-AN	TCGA-AN	BRCA	45	FEMALE	WHITE	Stage IIIA	Infiltrating Ductal Ca	2010	Pre (<6 m	-16533	Alive	TUMOR F	10						Negative	0	10	0	10	0	10	0	10

TCGA-AN	TCGA-AN	BRCA	56	FEMALE	WHITE	Stage IIA	Infiltrating Ductal Ca	2010	Post (prio	-20504	Alive	TUMOR F	16						Negative	0	16	0	16	0	16	0	16
TCGA-AN	TCGA-AN	BRCA	61	FEMALE	WHITE	Stage IIA	Infiltrating Ductal Ca	2010	Post (prio	-22341	Alive	TUMOR F	163						Negative	0	163	0	163	0	163	0	163
TCGA-AN	TCGA-AN	BRCA	68	FEMALE	WHITE	Stage IIIA	Infiltrating Ductal Ca	2010	Post (prio	-25029	Alive	TUMOR F	10						Negative	0	10	0	10	0	10	0	10
TCGA-AN	TCGA-AN	BRCA	59	FEMALE	WHITE	Stage IIIA	Infiltrating Ductal Ca	2010	Post (prio	-21640	Alive	TUMOR F	375						Negative	0	375	0	375	0	375	0	375
TCGA-AN	TCGA-AN	BRCA	69	FEMALE	WHITE	Stage IIIA	Infiltrating Ductal Ca	2010	Post (prio	-25305	Alive	TUMOR F	9						Negative	0	9	0	9	0	9	0	9
TCGA-AN	TCGA-AN	BRCA	55	FEMALE	WHITE	Stage IIIA	Infiltrating Ductal Ca	2010	Post (prio	-20381	Alive	TUMOR F	10						Negative	0	10	0	10	0	10	0	10
TCGA-AN	TCGA-AN	BRCA	63	FEMALE	WHITE	Stage IIIA	Infiltrating Ductal Ca	2010	Post (prio	-23358	Alive	TUMOR F	10						Negative	0	10	0	10	0	10	0	10
TCGA-AN	TCGA-AN	BRCA	54	FEMALE	WHITE	Stage IIIA	Infiltrating Ductal Ca	2010	Post (prio	-19794	Alive	TUMOR F	10						Negative	0	10	0	10	0	10	0	10
TCGA-AN	TCGA-AN	BRCA	54	FEMALE	WHITE	Stage IIA	Infiltrating Ductal Ca	2010	Post (prio	-20052	Alive	TUMOR F	10						Negative	0	10	0	10	0	10	0	10
TCGA-AN	TCGA-AN	BRCA	67	FEMALE	WHITE	Stage IIIA	Infiltrating Ductal Ca	2010	Post (prio	-24779	Alive	TUMOR F	162						Negative	0	162	0	162	0	162	0	162
TCGA-AN	TCGA-AN	BRCA	36	FEMALE	WHITE	Stage IIIA	Infiltrating Ductal Ca	2010	Pre (<6 m	-13208	Alive	TUMOR F	170						Negative	0	170	0	170	0	170	0	170
TCGA-AC	TCGA-AC	BRCA	34	FEMALE	ASIAN	Stage IIIA	Infiltrating Ductal Ca	2003	Pre (<6 m	-12443	Alive	TUMOR F	2442						Negative	0	2442	0	2442	0	2442	0	2442
TCGA-AC	TCGA-AC	BRCA	29	FEMALE	WHITE	Stage I	Infiltrating Ductal Ca	2006	Pre (<6 m	-10616	Alive	TUMOR F	1866						Negative	0	1866	0	1866	0	1866	0	1866
TCGA-AC	TCGA-AC	BRCA	59	FEMALE	WHITE	Stage IIB	Infiltrating Ductal Ca	2006	Post (prio	-21816	Alive	WITH TUN	2031		Locoregional Disease			1645	Negative	0	2031	0	2031	1	1645	1	1645
TCGA-AC	TCGA-AC	BRCA	69	FEMALE	BLACK OI	Stage IIA	Infiltrating Ductal Ca	2002	Post (prio	-25362	Dead	TUMOR FREE		2483					Negative	1	2483	0	2483	0	2483	0	2483
TCGA-AC	TCGA-AC	BRCA	54	FEMALE	BLACK OI	Stage IIB	Infiltrating Ductal Ca	2003	Post (prio	-19724	Dead	WITH TUMOR		2911	Distant M	Liver		1922	Close	1	2911	1	2911			1	1922
TCGA-AC	TCGA-AC	BRCA	57	FEMALE	WHITE	Stage IIB	Infiltrating Ductal Ca	2005	Post (prio	-20930	Alive	TUMOR F	2091						Negative	0	2091	0	2091	0	2091	0	2091
TCGA-AC	TCGA-AC	BRCA	42	FEMALE	WHITE	Stage IIB	Infiltrating Ductal Ca	2007	Pre (<6 m	-15540	Alive	TUMOR F	2124						Negative	0	2124	0	2124	0	2124	0	2124
TCGA-AC	TCGA-AC	BRCA	31	FEMALE	WHITE	Stage I	Other, specify	2001	Pre (<6 m	-11354	Dead	TUMOR FREE		1793	New Prim	Lung		1614	Negative	1	1793	0	1793	0	1793	1	1614
TCGA-AC	TCGA-AC	BRCA	41	FEMALE	WHITE	Stage I	Infiltrating Ductal Ca	2008	Peri (6-12	-15284	Alive	TUMOR F	1351						Negative	0	1351	0	1351	0	1351	0	1351
TCGA-AC	TCGA-AC	BRCA	41	FEMALE	WHITE	Stage IA	Infiltrating Ductal Ca	2008	Pre (<6 m	-15324	Alive	TUMOR F	997						Negative	0	997	0	997	0	997	0	997
TCGA-AC	TCGA-AC	BRCA	67	FEMALE	WHITE	Stage IIB	Other, specify	2009	Post (prio	-24519	Alive	TUMOR F	651						Negative	0	651	0	651	0	651	0	651
TCGA-AC	TCGA-AC	BRCA	41	FEMALE	BLACK OI	Stage IA	Infiltrating Ductal Ca	2009	Pre (<6 m	-15052	Alive	TUMOR F	1587						Negative	0	1587	0	1587	0	1587	0	1587
TCGA-AC	TCGA-AC	BRCA	48	FEMALE	WHITE	Stage IV	Other, specify	2008	Pre (<6 m	-17815	Dead	WITH TUMOR		792					Close	1	792	1	792			1	792
TCGA-AC	TCGA-AC	BRCA	61	FEMALE	WHITE	Stage IIA	Infiltrating Ductal Ca	2008	Post (prio	-22462	Alive	TUMOR F	1140						Negative	0	1140	0	1140	0	1140	0	1140
TCGA-AC	TCGA-AC	BRCA	71	FEMALE	WHITE	Stage IIB	Other, specify	2009	Post (prio	-26281	Alive	TUMOR F	618						Negative	0	618	0	618	0	618	0	618
TCGA-AC	TCGA-AC	BRCA	61	FEMALE	WHITE	Stage IIA	Infiltrating Lobular C	2009	Post (prio	-22284	Alive	TUMOR F	680						Negative	0	680	0	680	0	680	0	680
TCGA-AC	TCGA-AC	BRCA	61	FEMALE	WHITE	Stage IIIC	Infiltrating Lobular C	2007	Post (prio	-22642	Alive	WITH TUN	1613		Distant M	Bone		1066	Negative	0	1613	0	1613	1	1066	1	1066
TCGA-AC	TCGA-AC	BRCA	36	FEMALE	BLACK OI	Stage IIIC	Infiltrating Ductal Ca	2009	Pre (<6 m	-13307	Alive	WITH TUN	655		Distant M	Bone		143	Negative	0	655	0	655	1	143	1	143
TCGA-AC	TCGA-AC	BRCA	50	FEMALE	WHITE	Stage IIIA	Infiltrating Ductal Ca	2007	Pre (<6 m	-18561	Alive	TUMOR F	1542						Negative	0	1542	0	1542	0	1542	0	1542
TCGA-AC	TCGA-AC	BRCA	64	FEMALE	WHITE	Stage IIA	Infiltrating Ductal Ca	2006	Post (prio	-23404	Alive	TUMOR F	1547						Negative	0	1547	0	1547	0	1547	0	1547
TCGA-AC	TCGA-AC	BRCA	59	FEMALE	WHITE	Stage IIIA	Infiltrating Ductal Ca	2005	Post (prio	-21831	Alive	TUMOR F	2190						Negative	0	2190	0	2190	0	2190	0	2190
TCGA-AC	TCGA-AC	BRCA	53	FEMALE	BLACK OI	Stage IIIA	Infiltrating Ductal Ca	2005	Post (prio	-19516	Alive	TUMOR F	2335						Positive	0	2335	0	2335			0	2335
TCGA-AC	TCGA-AC	BRCA	68	FEMALE	WHITE	Stage IIA	Infiltrating Ductal Ca	2009	Post (prio	-24923	Alive	TUMOR F	1980						Negative	0	1980	0	1980	0	1980	0	1980
TCGA-AC	TCGA-AC	BRCA	49	FEMALE	WHITE	Stage IIIA	Other, specify	2009	Post (prio	-18021	Alive	TUMOR F	798						Close	0	798	0	798			0	798
TCGA-AC	TCGA-AC	BRCA	56	FEMALE	WHITE	Stage IIA	Infiltrating Ductal Ca	2007	Post (prio	-20694	Alive	TUMOR F	1528						Negative	0	1528	0	1528	0	1528	0	1528
TCGA-AC	TCGA-AC	BRCA	54	FEMALE	WHITE	Stage IIB	Infiltrating Lobular C	2006	Post (prio	-19758	Alive	TUMOR F	1887						Negative	0	1887	0	1887	0	1887	0	1887
TCGA-AC	TCGA-AC	BRCA	59	FEMALE	WHITE	Stage IIIA	Infiltrating Ductal Ca	2006	Post (prio	-21895	Alive	TUMOR F	1683						Negative	0	1683	0	1683	0	1683	0	1683
TCGA-AC	TCGA-AC	BRCA	40	FEMALE		Stage IIB	Infiltrating Ductal Ca	2005	Pre (<6 m	-14653	Alive	TUMOR F	2184						Negative	0	2184	0	2184	0	2184	0	2184
TCGA-AC	TCGA-AC	BRCA	38	FEMALE	WHITE	Stage IIA	Other, specify	2002	Pre (<6 m	-14174	Alive	TUMOR F	3506						Negative	0	3506	0	3506	0	3506	0	3506
TCGA-AC	TCGA-AC	BRCA	72	FEMALE	BLACK OI	Stage IIA	Other, specify	2002	Post (prio	-26466	Alive	TUMOR F	3456						Negative	0	3456	0	3456	0	3456	0	3456
TCGA-AC	TCGA-AC	BRCA	39	FEMALE	ASIAN	Stage IIA	Infiltrating Ductal Ca	2002	Pre (<6 m	-14264	Alive	TUMOR F	3307		Locoregional Disease			267	Negative	0	3307	0	3307	1	267	1	267
TCGA-AC	TCGA-AC	BRCA	61	FEMALE	WHITE	Stage IIA	Infiltrating Ductal Ca	2002	Post (prio	-22340	Alive	TUMOR F	3248						Negative	0	3248	0	3248	0	3248	0	3248
TCGA-AC	TCGA-AC	BRCA	29	FEMALE	WHITE	Stage IIB	Infiltrating Ductal Ca	2002	Pre (<6 m	-10898	Alive	TUMOR F	3286						Negative	0	3286	0	3286	0	3286	0	3286
TCGA-AC	TCGA-AC	BRCA	47	FEMALE	WHITE	Stage IIA	Infiltrating Ductal Ca	2003	Pre (<6 m	-17420	Alive	TUMOR F	3112						Close	0	3112	0	3112			0	3112
TCGA-AC	TCGA-AC	BRCA	63	FEMALE	WHITE	Stage IIA	Infiltrating Ductal Ca	2004	Post (prio	-23150	Alive	TUMOR F	2989						Negative	0	2989	0	2989	0	2989	0	2989
TCGA-AC	TCGA-AC	BRCA	42	FEMALE	WHITE	Stage IIB	Infiltrating Ductal Ca	2005	Pre (<6 m	-15469	Alive	TUMOR F	2372						Negative	0	2372	0	2372	0	2372	0	2372
TCGA-AC	TCGA-AC	BRCA	43	FEMALE	WHITE	Stage IIA	Infiltrating Ductal Ca	2005	Pre (<6 m	-15774	Alive	TUMOR F	2515						Negative	0	2515	0	2515	0	2515	0	2515
TCGA-AC	TCGA-AC	BRCA	51	FEMALE	WHITE	Stage IIB	Infiltrating Lobular C	2005	Pre (<6 m	-18721	Alive	TUMOR F	2142						Negative	0	2142	0	2142	0	2142	0	2142
TCGA-AC	TCGA-AC	BRCA	36	FEMALE	WHITE	Stage IIA	Infiltrating Ductal Ca	2006	Pre (<6 m	-13409	Alive	TUMOR F	1842						Negative	0	1842	0	1842	0	1842	0	1842
TCGA-AC	TCGA-AC	BRCA	75	FEMALE	WHITE	Stage IIA	Other, specify	2006	Post (prio	-27705	Alive	TUMOR F	1639						Negative	0	1639	0	1639	0	1639	0	1639
TCGA-AC	TCGA-AC	BRCA	69	FEMALE	BLACK OI	Stage IIA	Other, specify	2008	Pre (<6 m	-25515	Alive	TUMOR F	1234						Negative	0	1234	0	1234	0	1234	0	1234
TCGA-AC	TCGA-AC	BRCA	46	FEMALE	WHITE	Stage IIB	Infiltrating Lobular C	2008	Pre (<6 m	-16817	Alive	TUMOR F	622						Negative	0	622	0	622	0	622	0	622
TCGA-AC	TCGA-AC	BRCA	77	FEMALE	WHITE	Stage IIA	Infiltrating Ductal Ca	2003	Post (prio	-28247	Alive	TUMOR F	2953						Negative	0	2953	0	2953	0	2953	0	2953
TCGA-AC	TCGA-AC	BRCA	84	MALE	WHITE	Stage IIIB	Infiltrating Ductal Ca	2006		-30859	Alive	TUMOR F	1882						Negative	0	1882	0	1882	0	1882	0	1882
TCGA-AC	TCGA-AC	BRCA	51	FEMALE	WHITE	Stage IIA	Infiltrating Ductal Ca	2005	Pre (<6 m	-18900	Alive	TUMOR F	2513						Negative	0	2513	0	2513	0	2513	0	2513
TCGA-AC	TCGA-AC	BRCA	69	FEMALE	WHITE	Stage IIA	Infiltrating Lobular C	2010	Post (prio	-25230	Alive	TUMOR F	350						Negative	0	350	0	350	0	350	0	350
TCGA-AC	TCGA-AC	BRCA	78	FEMALE	WHITE	Stage IIA	Infiltrating Ductal Ca	2009	Post (prio	-28660	Alive	TUMOR F	541						Negative	0	541	0	541	0	541	0	541
TCGA-AC	TCGA-AC	BRCA	61	FEMALE	WHITE	Stage IIIA	Infiltrating Ductal Ca	2009	Post (prio	-22539	Alive	TUMOR F	754						Negative	0	754	0	754	0	754	0	754
TCGA-AC	TCGA-AC	BRCA	45	FEMALE	WHITE	Stage IIA	Infiltrating Ductal Ca	2009	Pre (<6 m	-16615	Alive	TUMOR F	819						Negative	0	819	0	819	0	819	0	819
TCGA-AC	TCGA-AC	BRCA	48	FEMALE	WHITE	Stage IIA	Infiltrating Ductal Ca	2001	Peri (6-12	-17759	Alive	TUMOR F	3957						Negative	0	3957	0	3957	0	3957	0	3957
TCGA-AC	TCGA-AC	BRCA	70	FEMALE	WHITE	Stage IIIA	Infiltrating Ductal Ca	2010	Post (prio	-25793	Dead	TUMOR FREE		172					Negative	1	172	0	172	0	172	0	172
TCGA-AC	TCGA-AC	BRCA	84	FEMALE	WHITE	Stage IIIA	Infiltrating Ductal Ca	2010	Post (prio	-30958	Alive	TUMOR F	475						Negative	0	475	0	475	0	475	0	475
TCGA-AC	TCGA-AC	BRCA	49	FEMALE	WHITE	Stage IIIC	Infiltrating Ductal Ca	2010	Pre (<6 m	-18177	Alive	TUMOR F	989						Negative	0	989	0	989	0	989	0	989
TCGA-AC	TCGA-AC	BRCA	51	FEMALE	BLACK OI	Stage IIA	Infiltrating Ductal Ca	2011	Post (prio	-18741	Alive	TUMOR F	78						Negative	0	78	0	78	0	78	0	78
TCGA-AC	TCGA-AC	BRCA	51	MALE	BLACK OI	Stage IIA	Infiltrating Ductal Ca	2011		-18694	Alive	TUMOR F	1001						Negative	0	1001	0	1001	0	1001	0	1001
TCGA-AC	TCGA-AC	BRCA	55	FEMALE	WHITE	Stage IIIA	Infiltrating Lobular C	2013	Post (prio	-20239	Dead	TUMOR FREE		584					Negative	1	584	0	584	0	584	0	584
TCGA-AR	TCGA-AR	BRCA	43	FEMALE	WHITE	Stage IIA	Infiltrating Ductal Ca	2003	Pre (<6 m	-15812	Alive	TUMOR F	4275						Negative	0	4275	0	4275	0	4275	0	4275
TCGA-AR	TCGA-AR	BRCA	27	FEMALE	WHITE	Stage IIIA	Infiltrating Ductal Ca	2006	Pre (<6 m	-9873	Alive	TUMOR F	2991						Negative	0	2991	0	2991	0	2991	0	2991
TCGA-AR	TCGA-AR	BRCA	68	FEMALE	WHITE	Stage IIB	Infiltrating Ductal Ca	2006	Post (prio	-25190	Dead			160					Negative	1	160		160	0	160	0	160
TCGA-AR	TCGA-AR	BRCA	46	FEMALE	WHITE	Stage IIB	Infiltrating Ductal Ca	2006	Pre (<6 m	-17141	Alive	TUMOR F	2558						Negative	0	2558	0	2558	0	2558	0	2558
TCGA-AR	TCGA-AR	BRCA	53	FEMALE	BLACK OI	Stage IIIA	Infiltrating Ductal Ca	2006	Pre (<6 m	-19679	Alive	TUMOR F	3316						Negative	0	3316	0	3316	0	3316	0	3316
TCGA-AR	TCGA-AR	BRCA	35	FEMALE		Stage IIA	Infiltrating Ductal Ca	2006	Pre (<6 m	-13039	Alive	TUMOR F	709						Negative	0	709	0	709	0	709	0	709
TCGA-AR	TCGA-AR	BRCA	66	FEMALE		Stage IIA	Infiltrating Ductal Ca	2005	Post (prio	-24279	Alive	TUMOR F	2288						Negative	0	2288	0	2288	0	2288	0	2288
TCGA-AR	TCGA-AR	BRCA	50	FEMALE	WHITE	Stage IIIA	Infiltrating Ductal Ca	2005	Pre (<6 m	-18263	Alive	TUMOR F	3009					739	Negative	0	3009	0	3009	1	739	1	739
TCGA-AR	TCGA-AR	BRCA	64	FEMALE		Stage IIA	Infiltrating Ductal Ca	2005	Post (prio	-23653	Alive	TUMOR F	1972						Negative	0	1972	0	1972	0	1972	0	1972
TCGA-AR	TCGA-AR	BRCA	54	FEMALE	WHITE	Stage IIA	Infiltrating Ductal Ca	2004	Post (prio	-19836	Dead	WITH TUMOR		1699	Distant M	Bone		544	Negative	1	1699	1	1699	1	544	1	544
TCGA-AR	TCGA-AR	BRCA	43	FEMALE	WHITE	[Discrepancy]	Infiltrating Ductal Ca	2004	Pre (<6 m	-15720	Dead			3262	Distant M	Bone		1313	Negative	1	3262		3262	1	1313	1	1313
TCGA-AR	TCGA-AR	BRCA	73	FEMALE	WHITE	Stage IIB	Infiltrating Ductal Ca	2004	Post (prio	-26993	Alive	TUMOR F	1988						Negative	0	1988	0	1988	0	1988	0	1988
TCGA-AR	TCGA-AR	BRCA	36	FEMALE	WHITE	Stage IIB	Infiltrating Ductal Ca	2004	Pre (<6 m	-13467	Alive	TUMOR F	4052						Negative	0	4052	0	4052	0	4052	0	4052
TCGA-AR	TCGA-AR	BRCA	47	FEMALE	WHITE	Stage IIIA	Infiltrating Ductal Ca	2004	Pre (<6 m	-17390	Dead	WITH TUMOR		2551	Distant M	Bone	Liver	1609	Negative	1	2551	1	2551	1	1609	1	1609
TCGA-AR	TCGA-AR	BRCA	59	FEMALE	WHITE	Stage IIB	Infiltrating Ductal Ca	2004	Post (prio	-21755	Alive	TUMOR F	4080						Negative	0	4080	0	4080	0	4080	0	4080
TCGA-AR	TCGA-AR	BRCA	54	FEMALE	WHITE	Stage IIA	Infiltrating Ductal Ca	2006	Pre (<6 m	-19729	Alive	TUMOR F	3261						Negative	0	3261	0	3261	0	3261	0	3261
TCGA-AR	TCGA-AR	BRCA	51	FEMALE	WHITE	Stage IIB	Infiltrating Ductal Ca	2004	Post (prio	-18991	Alive	TUMOR F	3807		New Prim	Other, sp	Renal	1136	Negative	0	3807	0	3807	0	3807	1	1136
TCGA-AR	TCGA-AR	BRCA	47	FEMALE	WHITE	Stage IIA	Medullary Carcinom	2005	Post (prio	-17229	Alive	TUMOR F	3296		New Prim	Other, sp	Contralate	588	Negative	0	3296	0	3296	1	588	1	588
TCGA-AR	TCGA-AR	BRCA	83	FEMALE	WHITE	Stage I	Infiltrating Ductal Ca	2005	Post (prio	-30489	Alive	TUMOR F	3072						Negative	0	3072	0	3072	0	3072	0	3072
TCGA-AR	TCGA-AR	BRCA	70	FEMALE	WHITE	Stage I	Infiltrating Lobular C	2006	Post (prio	-25825	Alive	TUMOR F	3159						Negative	0	3159	0	3159	0	3159	0	3159
TCGA-AR	TCGA-AR	BRCA	60	FEMALE	WHITE	Stage IIIA	Infiltrating Lobular C	2006	Post (prio	-22023	Alive	TUMOR F	2971						Negative	0	2971	0	2971	0	2971	0	2971
TCGA-AR	TCGA-AR	BRCA	52	FEMALE	WHITE	Stage IIIA	Infiltrating Lobular C	2006	Post (prio	-19123	Alive	TUMOR F	2991						Negative	0	2991	0	2991	0	2991	0	2991
TCGA-AR	TCGA-AR	BRCA	46	FEMALE	WHITE	Stage IIA	Infiltrating Ductal Ca	2006	Pre (<6 m	-16974	Alive	TUMOR F	2920						Negative	0	2920	0	2920	0	2920	0	2920
TCGA-AR	TCGA-AR	BRCA	47	FEMALE	WHITE	Stage IIA	Infiltrating Ductal Ca	2006	Pre (<6 m	-17332	Alive	TUMOR F	2618						Negative	0	2618	0	2618	0	2618	0	2618
TCGA-AR	TCGA-AR	BRCA	80	FEMALE	WHITE	Stage I	Infiltrating Ductal Ca	2007	Post (prio	-29406	Alive	TUMOR F	2856						Negative	0	2856	0	2856	0	2856	0	2856
TCGA-AR	TCGA-AR	BRCA	49	FEMALE	WHITE	Stage IIA	Infiltrating Ductal Ca	2007	Post (prio	-18109	Alive	TUMOR F	3021						Negative	0	3021	0	3021	0	3021	0	3021
TCGA-AR	TCGA-AR	BRCA	50	FEMALE	WHITE	Stage IIIA	Infiltrating Ductal Ca	2007	Post (prio	-18482	Dead	WITH TUMOR		524	New Prim	Other, sp	Breast	268	Negative	1	524	1	524	1	268	1	268
TCGA-AR	TCGA-AR	BRCA	54	FEMALE	ASIAN	Stage IIB	Infiltrating Ductal Ca	2007	Post (prio	-20028	Alive	TUMOR F	1150						Negative	0	1150	0	1150	0	1150	0	1150
TCGA-AR	TCGA-AR	BRCA	62	FEMALE	WHITE	Stage IIA	Infiltrating Lobular C	2007	Post (prio	-22942	Dead			1272					Negative	1	1272		1272	0	1272	0	1272
TCGA-AR	TCGA-AR	BRCA	39	FEMALE	WHITE	Stage IIIA	Infiltrating Ductal Ca	2007	Pre (<6 m	-14564	Alive	TUMOR F	2868						Negative	0	2868	0	2868	0	2868	0	2868
TCGA-AR	TCGA-AR	BRCA	68	MALE	WHITE	Stage IIB	Infiltrating Ductal Ca	2007		-24875	Alive	TUMOR F	1864						Negative	0	1864	0	1864	0	1864	0	1864
TCGA-AR	TCGA-AR	BRCA	65	FEMALE	WHITE	Stage IIA	Infiltrating Ductal Ca	2007	Post (prio	-23855	Alive	TUMOR F	2632						Negative	0	2632	0	2632	0	2632	0	2632
TCGA-AR	TCGA-AR	BRCA	64	FEMALE	WHITE	Stage I	Infiltrating Ductal Ca	2007	Post (prio	-23507	Alive	TUMOR F	2629						Negative	0	2629	0	2629	0	2629	0	2629
TCGA-AR	TCGA-AR	BRCA	65	FEMALE	WHITE	Stage I	Infiltrating Ductal Ca	2008	Post (prio	-23927	Alive	TUMOR F	1026						Negative	0	1026	0	1026	0	1026	0	1026
TCGA-AR	TCGA-AR	BRCA	65	FEMALE	WHITE	Stage IIA	Infiltrating Lobular C	2001	Post (prio	-24046	Alive	TUMOR F	4894						Negative	0	4894	0	4894	0	4894	0	4894
TCGA-AR	TCGA-AR	BRCA	46	FEMALE	WHITE	Stage IIA	Infiltrating Ductal Ca	2004	Pre (<6 m	-16961	Alive	TUMOR F	1548						Negative	0	1548	0	1548	0	1548	0	1548
TCGA-AR	TCGA-AR	BRCA	26	FEMALE	WHITE	Stage IIB	Infiltrating Ductal Ca	2005	Pre (<6 m	-9706	Dead	WITH TUMOR		2866	Locoregional Disease			1414	Negative	1	2866	1	2866	1	1414	1	1414
TCGA-AR	TCGA-AR	BRCA	38	FEMALE	WHITE	Stage IIIA	Mixed Histology (ple	2005	Pre (<6 m	-14119	Alive	TUMOR F	3660						Negative	0	3660	0	3660	0	3660	0	3660
TCGA-AR	TCGA-AR	BRCA	54	FEMALE	WHITE	Stage I	Infiltrating Ductal Ca	2005	Peri (6-12	-19989	Alive	TUMOR F	3035						Negative	0	3035	0	3035	0	3035	0	3035
TCGA-AR	TCGA-AR	BRCA	43	FEMALE	WHITE	Stage IIIA	Mixed Histology (ple	2005	Pre (<6 m	-15977	Alive	WITH TUN	3607					2561	Negative	0	3607	0	3607	1	2561	1	2561
TCGA-AR	TCGA-AR	BRCA	47	FEMALE	WHITE	Stage I	Infiltrating Ductal Ca	2005	Post (prio	-17490	Alive		84						Negative	0	84	0	84	0	84	0	84
TCGA-AR	TCGA-AR	BRCA	49	FEMALE	WHITE	Stage IIB	Infiltrating Ductal Ca	2006	Post (prio	-18084	Alive	TUMOR F	3172						Negative	0	3172	0	3172	0	3172	0	3172
TCGA-AR	TCGA-AR	BRCA	45	FEMALE	WHITE	Stage IIIA	Infiltrating Ductal Ca	2006	Pre (<6 m	-16642	Alive	TUMOR F	3430						Negative	0	3430	0	3430	0	3430	0	3430
TCGA-AR	TCGA-AR	BRCA	61	FEMALE	WHITE	Stage I	Infiltrating Ductal Ca	2006	Post (prio	-22587	Alive		2976						Negative	0	2976	0	2976	0	2976	0	2976
TCGA-AR	TCGA-AR	BRCA	46	FEMALE	WHITE	Stage IIIC	Infiltrating Lobular C	2006	Pre (<6 m	-17135	Alive	TUMOR F	3202						Negative	0	3202	0	3202	0	3202	0	3202
TCGA-AR	TCGA-AR	BRCA	47	FEMALE	WHITE	Stage IIA	Infiltrating Ductal Ca	2006	Pre (<6 m	-17523	Alive	TUMOR F	3128						Negative	0	3128	0	3128	0	3128	0	3128
TCGA-AR	TCGA-AR	BRCA	52	FEMALE	WHITE	Stage IIB	Infiltrating Ductal Ca	2006	Peri (6-12	-19027	Alive	TUMOR F	3203						Negative	0	3203	0	3203	0	3203	0	3203
TCGA-AR	TCGA-AR	BRCA	55	FEMALE	WHITE	Stage IIB	Infiltrating Ductal Ca	2006	Post (prio	-20123	Alive	TUMOR F	1550						Negative	0	1550	0	1550	0	1550	0	1550
TCGA-AR	TCGA-AR	BRCA	52	FEMALE	WHITE	Stage IIA	Mixed Histology (ple	2006	Pre (<6 m	-19030	Alive	TUMOR F	3004						Negative	0	3004	0	3004	0	3004	0	3004
TCGA-AR	TCGA-AR	BRCA	57	FEMALE	WHITE	Stage IIA	Infiltrating Ductal Ca	2006	Post (prio	-20900	Alive	TUMOR F	3001						Negative	0	3001	0	3001	0	3001	0	3001
TCGA-AR	TCGA-AR	BRCA	58	FEMALE	WHITE	Stage IIA	Infiltrating Ductal Ca	2006	Post (prio	-21309	Alive	TUMOR F	2707						Negative	0	2707	0	2707	0	2707	0	2707
TCGA-AR	TCGA-AR	BRCA	51	FEMALE	WHITE	Stage IIIA	Infiltrating Ductal Ca	2006	Pre (<6 m	-18771	Alive	TUMOR F	3030						Negative	0	3030	0	3030	0	3030	0	3030
TCGA-AR	TCGA-AR	BRCA	50	FEMALE	WHITE	Stage I	Infiltrating Ductal Ca	2007	Pre (<6 m	-18611	Alive	TUMOR F	2838						Negative	0	2838	0	2838	0	2838	0	2838
TCGA-AR	TCGA-AR	BRCA	50	FEMALE	WHITE	Stage IIIA	Infiltrating Ductal Ca	2007	Pre (<6 m	-18270	Alive	TUMOR F	2605						Negative	0	2605	0	2605	0	2605	0	2605
TCGA-AR	TCGA-AR	BRCA	62	FEMALE	WHITE	Stage I	Infiltrating Ductal Ca	2007	Post (prio	-22809	Alive	TUMOR F	2161						Negative	0	2161	0	2161	0	2161	0	2161
TCGA-AR	TCGA-AR	BRCA	45	FEMALE	WHITE	Stage IIA	Infiltrating Ductal Ca	2001	Pre (<6 m	-16642	Dead			2854	Locoregional Disease			1640	Negative	1	2854		2854	1	1640	1	1640
TCGA-AR	TCGA-AR	BRCA	69	FEMALE	WHITE	Stage I	Infiltrating Lobular C	2001	Post (prio	-25499	Alive	TUMOR F	5062		New Prim	Other, sp	Endometr	4203	Negative	0	5062	0	5062	0	5062	1	4203
TCGA-AR	TCGA-AR	BRCA	55	FEMALE	WHITE	Stage III	Infiltrating Lobular C	2007	Post (prio	-20288	Dead			616	Distant M	Bone		434	Negative	1	616		616	1	434	1	434
TCGA-AR	TCGA-AR	BRCA	40	FEMALE	WHITE	Stage IIIA	Infiltrating Lobular C	2007	Pre (<6 m	-14723	Alive	TUMOR F	2632						Negative	0	2632	0	2632	0	2632	0	2632
TCGA-AR	TCGA-AR	BRCA	62	FEMALE	WHITE	Stage III	Infiltrating Lobular C	2007	Post (prio	-22800	Dead			1649	Distant M	Bone		1304	Negative	1	1649		1649	1	1304	1	1304
TCGA-AR	TCGA-AR	BRCA	70	FEMALE	WHITE	Stage IIB	Infiltrating Lobular C	2007	Post (prio	-25747	Alive	TUMOR F	2012						Negative	0	2012	0	2012	0	2012	0	2012
TCGA-AR	TCGA-AR	BRCA	49	FEMALE	WHITE	Stage II	Infiltrating Lobular C	2007	Pre (<6 m	-18053	Alive	TUMOR F	1935						Negative	0	1935	0	1935	0	1935	0	1935
TCGA-AR	TCGA-AR	BRCA	65	FEMALE	WHITE	Stage IIA	Infiltrating Lobular C	2010	Post (prio	-23992	Alive	TUMOR F	1161						Negative	0	1161	0	1161	0	1161	0	1161
TCGA-AR	TCGA-AR	BRCA	46	FEMALE	WHITE	Stage IIB	Infiltrating Lobular C	2010	Pre (<6 m	-17080	Alive	TUMOR F	1198						Negative	0	1198	0	1198	0	1198	0	1198
TCGA-AR	TCGA-AR	BRCA	59	FEMALE	WHITE	Stage IIB	Infiltrating Lobular C	2010	Post (prio	-21865	Alive	TUMOR F	1233						Negative	0	1233	0	1233	0	1233	0	1233
TCGA-AR	TCGA-AR	BRCA	49	FEMALE	WHITE	Stage I	Metaplastic Carcinor	2010	Post (prio	-18214	Alive	TUMOR F	1742						Negative	0	1742	0	1742	0	1742	0	1742
TCGA-AR	TCGA-AR	BRCA	62	FEMALE	WHITE	Stage IIA	Infiltrating Lobular C	2008	Post (prio	-22780	Alive	TUMOR F	2231		New Prim	Other, sp	Malignant	968		0	2231	0	2231			1	968
TCGA-AR	TCGA-AR	BRCA	68	FEMALE	WHITE	Stage IIIC	Infiltrating Lobular C	2012	Post (prio	-25089	Alive	TUMOR F	1013							0	1013	0	1013			0	1013
TCGA-AR	TCGA-AR	BRCA	54	FEMALE	WHITE	Stage IIB	Infiltrating Lobular C	2011	Post (prio	-19805	Alive	TUMOR F	1185							0	1185	0	1185			0	1185
TCGA-AR	TCGA-AR	BRCA	68	FEMALE	WHITE	Stage IIIA	Other, specify	2011	Post (prio	-24841	Dead	WITH TUMOR		322	Distant M	Lung	Liver, bon	301		1	322	1	322			1	301
TCGA-B6	TCGA-B6	BRCA	73	FEMALE	BLACK OI	Stage IIA	Infiltrating Ductal Ca	1998	Post (prio	-26902	Dead	TUMOR FREE		2361	Distant M	Lung		1120	Negative	1	2361	0	2361	1	1120	1	1120
TCGA-B6	TCGA-B6	BRCA	45	FEMALE	WHITE	Stage IA	Infiltrating Ductal Ca	1995	Indetermi	-16702	Alive	TUMOR F	4361							0	4361	0	4361			0	4361
TCGA-B6	TCGA-B6	BRCA	49	FEMALE	WHITE	Stage IIB	Infiltrating Ductal Ca	1988	Indetermi	-18038	Alive	TUMOR F	8556						Negative	0	8556	0	8556	0	8556	0	8556
TCGA-B6	TCGA-B6	BRCA	49	FEMALE	BLACK OI	Stage IIA	Infiltrating Ductal Ca	1990	Indetermi	-18014	Dead	WITH TUMOR		991	Distant M	Other, sp	Skin and I	859	Negative	1	991	1	991	1	859	1	859
TCGA-B6	TCGA-B6	BRCA	46	FEMALE	WHITE	Stage X	Infiltrating Ductal Ca	1992	Indetermi	-17073	Dead	WITH TUMOR		749	Locoregic	Other, sp	Pectoral r	746		1	749	1	749			1	746
TCGA-B6	TCGA-B6	BRCA	62	FEMALE	WHITE	Stage IV	Infiltrating Ductal Ca	1992	Indetermi	-22798	Dead	WITH TUMOR		362						1	362	1	362			1	362
TCGA-B6	TCGA-B6	BRCA	51	FEMALE	WHITE	Stage IIA	Other, specify	1992	Indetermi	-18748	Alive	TUMOR F	8391						Negative	0	8391	0	8391	0	8391	0	8391
TCGA-B6	TCGA-B6	BRCA	64	FEMALE	WHITE	Stage IV	Infiltrating Ductal Ca	1992	Indetermi	-23646	Dead	WITH TUMOR		3941	Distant M	Other, sp	lung, bon	2717	Negative	1	3941	1	3941			1	2717
TCGA-B6	TCGA-B6	BRCA	90	FEMALE	WHITE	Stage X	Other, specify	1992	Post (prio	-32872	Dead	TUMOR FREE		1542					Negative	1	1542	0	1542	0	1542	0	1542
TCGA-B6	TCGA-B6	BRCA	38	FEMALE	BLACK OI	Stage IIIA	Mixed Histology (ple	1995	Pre (<6 m	-13982	Dead	WITH TUMOR		1993	Distant M	Bone		834	Negative	1	1993	1	1993	1	834	1	834
TCGA-B6	TCGA-B6	BRCA	50	FEMALE	WHITE	Stage IIB	Infiltrating Ductal Ca	1994	Indetermi	-18433	Dead	WITH TUMOR		4456					Negative	1	4456	1	4456			1	4456
TCGA-B6	TCGA-B6	BRCA	81	FEMALE	WHITE	Stage IIIA	Infiltrating Lobular C	1995	Post (prio	-29907	Dead			3418					Negative	1	3418		3418	0	3418	0	3418
TCGA-B6	TCGA-B6	BRCA	42	FEMALE	BLACK OI	Stage IIB	Infiltrating Ductal Ca	1995	Indetermi	-15625	Alive	TUMOR F	7106						Negative	0	7106	0	7106	0	7106	0	7106
TCGA-B6	TCGA-B6	BRCA	63	FEMALE	WHITE	Stage IIIB	Infiltrating Ductal Ca	1994	Indetermi	-23150	Dead	WITH TUMOR		571	Distant M	Brain		489	Negative	1	571	1	571	1	489	1	489
TCGA-B6	TCGA-B6	BRCA	75	FEMALE	BLACK OI	Stage IIB	Other, specify	1997	Post (prio	-27557	Dead	TUMOR FREE		3873					Negative	1	3873	0	3873	0	3873	0	3873
TCGA-B6	TCGA-B6	BRCA	45	FEMALE	WHITE	Stage X	Other, specify	1996	Indetermi	-16705	Dead	WITH TUMOR		2573	Distant M	Other, sp	bone, bra	1412	Negative	1	2573	1	2573	1	1412	1	1412
TCGA-B6	TCGA-B6	BRCA	66	FEMALE	WHITE	Stage IIA	Infiltrating Ductal Ca	2001	Indetermi	-24322	Alive	TUMOR F	5042						Negative	0	5042	0	5042	0	5042	0	5042
TCGA-B6	TCGA-B6	BRCA	74	FEMALE	WHITE	Stage IA	Infiltrating Lobular C	1997	Post (prio	-27047	Dead	TUMOR FREE		3926					Negative	1	3926	0	3926	0	3926	0	3926
TCGA-B6	TCGA-B6	BRCA	40	FEMALE	WHITE	Stage IIIA	Infiltrating Ductal Ca	1996	Indetermi	-14669	Alive	TUMOR F	4285						Negative	0	4285	0	4285	0	4285	0	4285
TCGA-B6	TCGA-B6	BRCA	61	FEMALE	BLACK OI	Stage X	Infiltrating Ductal Ca	1989	Indetermi	-22449	Alive	TUMOR F	7777						Negative	0	7777	0	7777	0	7777	0	7777
TCGA-B6	TCGA-B6	BRCA	26	FEMALE	WHITE	Stage IIB	Infiltrating Ductal Ca	1991	Indetermi	-9840	Alive	TUMOR F	2082						Negative	0	2082	0	2082	0	2082	0	2082
TCGA-B6	TCGA-B6	BRCA	51	FEMALE	BLACK OI	Stage IIA	Infiltrating Ductal Ca	1991	Indetermi	-18759	Dead	TUMOR FREE		6456	Locoregic	Other, sp	Chest Wa	2165	Negative	1	6456	0	6456	1	2165	1	2165
TCGA-B6	TCGA-B6	BRCA	44	FEMALE	WHITE	Stage IIIB	Infiltrating Ductal Ca	1992	Indetermi	-16138	Alive	WITH TUN	7126		Locoregic	Other, sp	Chest Wa	1100	Negative	0	7126	0	7126	1	1100	1	1100
TCGA-B6	TCGA-B6	BRCA	60	FEMALE	BLACK OI	Stage IIA	Infiltrating Ductal Ca	1993		-22238	Dead	TUMOR FREE		2469					Negative	1	2469	0	2469	0	2469	0	2469
TCGA-B6	TCGA-B6	BRCA	57	FEMALE	BLACK OI	Stage X	Infiltrating Ductal Ca	1993	Indetermi	-21047	Dead	TUMOR FREE		2373						1	2373	0	2373			0	2373
TCGA-B6	TCGA-B6	BRCA	60	FEMALE	WHITE	Stage IA	Infiltrating Ductal Ca	1993	Post (prio	-22077	Alive	TUMOR F	8008						Negative	0	8008	0	8008	0	8008	0	8008
TCGA-B6	TCGA-B6	BRCA	71	FEMALE	WHITE	Stage IIIB	Infiltrating Ductal Ca	1993	Post (prio	-26269	Alive	TUMOR F	4929						Negative	0	4929	0	4929	0	4929	0	4929
TCGA-B6	TCGA-B6	BRCA	73	FEMALE	WHITE	[Discrepancy]	Mixed Histology (ple	1994	Post (prio	-26941	Dead	WITH TUMOR		3126	Distant M	Bone		2787	Negative	1	3126	1	3126	1	2787	1	2787
TCGA-B6	TCGA-B6	BRCA	68	FEMALE	WHITE	Stage IIB	Infiltrating Lobular C	1994	Post (prio	-25010	Dead	TUMOR FREE		4267					Negative	1	4267	0	4267	0	4267	0	4267
TCGA-B6	TCGA-B6	BRCA	38	FEMALE	WHITE	Stage IIA	Infiltrating Ductal Ca	1988	Indetermi	-14134	Dead	WITH TUMOR		3063	New Prim	Other, sp	Breast	2041	Negative	1	3063	1	3063	1	2041	1	2041
TCGA-B6	TCGA-B6	BRCA	39	FEMALE	WHITE	Stage IIIA	Infiltrating Ductal Ca	1996	Indetermi	-14374	Alive	TUMOR F	2721						Close	0	2721	0	2721			0	2721
TCGA-B6	TCGA-B6	BRCA	40	FEMALE	WHITE	Stage IA	Other, specify	1990	Indetermi	-14920	Alive	TUMOR F	8605		New Prim	Other, sp	Right Brea	3076	Negative	0	8605	0	8605	1	3076	1	3076
TCGA-B6	TCGA-B6	BRCA	42	FEMALE	WHITE	Stage IIIA	Mixed Histology (ple	1996	Indetermi	-15507	Alive	TUMOR F	5156						Negative	0	5156	0	5156	0	5156	0	5156
TCGA-B6	TCGA-B6	BRCA	58	FEMALE	BLACK OI	Stage X	Infiltrating Ductal Ca	1996	Indetermi	-21230	Dead	TUMOR FREE		2965					Positive	1	2965	0	2965			0	2965
TCGA-B6	TCGA-B6	BRCA	61	FEMALE	WHITE	Stage IIB	Infiltrating Ductal Ca	1995	Post (prio	-22362	Alive	TUMOR F	5739		New Prim	Other, sp	right brea	3862	Negative	0	5739	0	5739	1	3862	1	3862
TCGA-B6	TCGA-B6	BRCA	67	FEMALE	WHITE	Stage IIB	Infiltrating Ductal Ca	1995	Post (prio	-24803	Dead	TUMOR FREE		2417					Negative	1	2417	0	2417	0	2417	0	2417
TCGA-B6	TCGA-B6	BRCA	58	FEMALE	BLACK OI	Stage X	Infiltrating Ductal Ca	1997	Indetermi	-21479	Dead	WITH TUMOR		558	Distant M	Liver		447	Negative	1	558	1	558	1	447	1	447
TCGA-B6	TCGA-B6	BRCA	40	FEMALE	BLACK OI	Stage IIIA	Other, specify	1998	Pre (<6 m	-14784	Dead	WITH TUMOR		639	Distant M	Bone		563	Negative	1	639	1	639	1	563	1	563
TCGA-B6	TCGA-B6	BRCA	40	FEMALE	WHITE	Stage IIIA	Infiltrating Ductal Ca	1996	Pre (<6 m	-14778	Dead	WITH TUMOR		3461	Distant M	Bone		2011	Negative	1	3461	1	3461	1	2011	1	2011
TCGA-B6	TCGA-B6	BRCA	50	FEMALE	BLACK OI	Stage II	Infiltrating Ductal Ca	1996	Indetermi	-18584	Alive	TUMOR F	6292		New Prim	Bone		5117	Negative	0	6292	0	6292	0	6292	1	5117
TCGA-B6	TCGA-B6	BRCA	54	FEMALE	WHITE	Stage I	Other, specify	1996	Indetermi	-19802	Dead			3945					Negative	1	3945		3945	0	3945	0	3945
TCGA-B6	TCGA-B6	BRCA	48	FEMALE	WHITE	[Discrepancy]	Infiltrating Ductal Ca	1994	Indetermi	-17624	Dead	TUMOR FREE		7455	Distant M	Brain		490	Negative	1	7455	0	7455	1	490	1	490
TCGA-B6	TCGA-B6	BRCA	62	FEMALE	WHITE	Stage IIB	Infiltrating Ductal Ca	1996	Post (prio	-22688	Dead	WITH TUMOR		860	Distant M	Bone		716	Negative	1	860	1	860	1	716	1	716
TCGA-B6	TCGA-B6	BRCA	61	FEMALE	BLACK OI	Stage IIB	Infiltrating Ductal Ca	1995	Post (prio	-22380	Dead	WITH TUMOR		2097	Distant M	Other, sp	Liver and	1343	Negative	1	2097	1	2097	1	1343	1	1343
TCGA-B6	TCGA-B6	BRCA	62	FEMALE	WHITE	Stage X	Mixed Histology (ple	1994	Post (prio	-22723	Dead	TUMOR FREE		1781	New Prim	Other, sp	colon	1104		1	1781	0	1781			1	1104
TCGA-B6	TCGA-B6	BRCA	67	FEMALE	WHITE	Stage IIB	Infiltrating Ductal Ca	2003	Post (prio	-24791	Alive	TUMOR F	1326						Negative	0	1326	0	1326	0	1326	0	1326
TCGA-B6	TCGA-B6	BRCA	68	FEMALE	WHITE	Stage IIB	Infiltrating Ductal Ca	1998	Post (prio	-24891	Alive	TUMOR F	3088						Negative	0	3088	0	3088	0	3088	0	3088
TCGA-B6	TCGA-B6	BRCA	63	FEMALE	WHITE	Stage I	Infiltrating Ductal Ca	2003	Post (prio	-23031	Alive	TUMOR F	2236						Negative	0	2236	0	2236	0	2236	0	2236
TCGA-B6	TCGA-B6	BRCA	57	FEMALE	WHITE	Stage IIIB	Infiltrating Ductal Ca	1998	Post (prio	-21183	Alive	TUMOR F	4233						Negative	0	4233	0	4233	0	4233	0	4233
TCGA-B6	TCGA-B6	BRCA	62	FEMALE	WHITE	Stage IIA	Infiltrating Lobular C	1998	Indetermi	-22905	Alive	WITH TUN	5176		New Prim	Other, sp	Lung	4224	Negative	0	5176	0	5176	0	5176	1	4224
TCGA-B6	TCGA-B6	BRCA	50	FEMALE	BLACK OI	Stage IV	Mixed Histology (ple	2007	[Unknowr	-18434	Dead	WITH TUMOR		1152	Distant M	Bone		994	Negative	1	1152	1	1152			1	994
TCGA-B6	TCGA-B6	BRCA	43	FEMALE	BLACK OI	Stage IIIA	Infiltrating Ductal Ca	2008	[Unknowr	-16022	Alive	TUMOR F	215						Negative	0	215	0	215	0	215	0	215
TCGA-B6	TCGA-B6	BRCA	47	FEMALE	BLACK OI	Stage IIA	Infiltrating Ductal Ca	2007	[Unknowr	-17438	Alive	TUMOR F	2596						Negative	0	2596	0	2596	0	2596	0	2596
TCGA-B6	TCGA-B6	BRCA	47	FEMALE	BLACK OI	Stage I	Infiltrating Ductal Ca	2008	[Unknowr	-17314	Alive	WITH TUN	2281		Locoregic	Other, sp	breast	1516	Negative	0	2281	0	2281	1	1516	1	1516
TCGA-B6	TCGA-B6	BRCA	55	FEMALE	BLACK OI	Stage IIIC	Mixed Histology (ple	2008	[Unknowr	-20102	Alive	TUMOR F	2072						Negative	0	2072	0	2072	0	2072	0	2072
TCGA-B6	TCGA-B6	BRCA	44	FEMALE	BLACK OI	Stage IIIA	Infiltrating Ductal Ca	2008	Pre (<6 m	-16178	Dead	WITH TUMOR		573	Distant M	Lung		456	Negative	1	573	1	573	1	456	1	456
TCGA-B6	TCGA-B6	BRCA	76	FEMALE	WHITE	Stage I	Infiltrating Lobular C	2005	Post (prio	-27811	Alive	TUMOR F	3152						Negative	0	3152	0	3152	0	3152	0	3152
TCGA-B6	TCGA-B6	BRCA	51	FEMALE	WHITE	Stage IIB	Infiltrating Lobular C	2005	[Unknowr	-18852	Alive	TUMOR F	2164						Negative	0	2164	0	2164	0	2164	0	2164
TCGA-BH	TCGA-BH	BRCA	45	FEMALE	WHITE	Stage IIA	Infiltrating Ductal Ca	2008	Pre (<6 m	-16688	Alive	TUMOR F	1914						Negative	0	1914	0	1914	0	1914	0	1914
TCGA-BH	TCGA-BH	BRCA	45	FEMALE	WHITE	Stage IIA	Infiltrating Ductal Ca	2008	Pre (<6 m	-16688	Alive	TUMOR F	1914						Negative	0	1914	0	1914	0	1914	0	1914
TCGA-BH	TCGA-BH	BRCA	52	FEMALE	BLACK OI	Stage I	Infiltrating Ductal Ca	2008		-19314	Alive	TUMOR F	1820						Negative	0	1820	0	1820	0	1820	0	1820
TCGA-BH	TCGA-BH	BRCA	56	FEMALE	WHITE	Stage IIA	Infiltrating Ductal Ca	2008	Post (prio	-20468	Alive	TUMOR F	622						Negative	0	622	0	622	0	622	0	622
TCGA-BH	TCGA-BH	BRCA	62	FEMALE	WHITE	Stage IIA	Infiltrating Ductal Ca	2008	Post (prio	-22703	Alive	TUMOR F	777						Negative	0	777	0	777	0	777	0	777
TCGA-BH	TCGA-BH	BRCA	62	FEMALE	WHITE	Stage IIA	Infiltrating Ductal Ca	2008	Post (prio	-22703	Alive	TUMOR F	777						Negative	0	777	0	777	0	777	0	777
TCGA-BH	TCGA-BH	BRCA	47	FEMALE	WHITE	Stage IIIA	Infiltrating Ductal Ca	2008	Pre (<6 m	-17465	Alive	TUMOR F	1919						Negative	0	1919	0	1919	0	1919	0	1919
TCGA-BH	TCGA-BH	BRCA	47	FEMALE	WHITE	Stage IIIA	Infiltrating Ductal Ca	2008	Pre (<6 m	-17465	Alive	TUMOR F	1919						Negative	0	1919	0	1919	0	1919	0	1919
TCGA-BH	TCGA-BH	BRCA	56	FEMALE	BLACK OI	Stage I	Infiltrating Ductal Ca	2006	Peri (6-12	-20776	Alive	TUMOR F	2477						Negative	0	2477	0	2477	0	2477	0	2477
TCGA-BH	TCGA-BH	BRCA	66	FEMALE	WHITE	Stage IIB	Infiltrating Ductal Ca	2007	Post (prio	-24315	Alive	TUMOR F	1148						Negative	0	1148	0	1148	0	1148	0	1148
TCGA-BH	TCGA-BH	BRCA	53	FEMALE	WHITE	Stage IIB	Infiltrating Ductal Ca	2007	Post (prio	-19479	Alive	TUMOR F	1203						Negative	0	1203	0	1203	0	1203	0	1203
TCGA-BH	TCGA-BH	BRCA	53	FEMALE	WHITE	Stage IIB	Infiltrating Ductal Ca	2007	Post (prio	-19479	Alive	TUMOR F	1203						Negative	0	1203	0	1203	0	1203	0	1203
TCGA-BH	TCGA-BH	BRCA	65	MALE	WHITE	Stage IIIA	Infiltrating Ductal Ca	2007		-23980	Alive	TUMOR F	1191						Negative	0	1191	0	1191	0	1191	0	1191
TCGA-BH	TCGA-BH	BRCA	40	FEMALE	WHITE	Stage IIIA	Infiltrating Lobular C	2007	Pre (<6 m	-14920	Alive	TUMOR F	2136						Positive	0	2136	0	2136			0	2136
TCGA-BH	TCGA-BH	BRCA	40	FEMALE	WHITE	Stage IIIA	Infiltrating Lobular C	2007	Pre (<6 m	-14920	Alive	TUMOR F	2136						Positive	0	2136	0	2136			0	2136
TCGA-BH	TCGA-BH	BRCA	47	FEMALE	WHITE	Stage I	Infiltrating Ductal Ca	2006		-17346	Alive	TUMOR F	2483						Negative	0	2483	0	2483	0	2483	0	2483
TCGA-BH	TCGA-BH	BRCA	42	FEMALE	WHITE	Stage IIB	Infiltrating Ductal Ca	2006	Pre (<6 m	-15593	Alive	TUMOR F	2559						Negative	0	2559	0	2559	0	2559	0	2559
TCGA-BH	TCGA-BH	BRCA	42	FEMALE	WHITE	Stage IIB	Infiltrating Ductal Ca	2006	Pre (<6 m	-15593	Alive	TUMOR F	2559						Negative	0	2559	0	2559	0	2559	0	2559
TCGA-BH	TCGA-BH	BRCA	64	FEMALE	WHITE	Stage I	Infiltrating Ductal Ca	2006	Post (prio	-23380	Alive	TUMOR F	1569						Negative	0	1569	0	1569	0	1569	0	1569
TCGA-BH	TCGA-BH	BRCA	64	FEMALE	WHITE	Stage I	Infiltrating Ductal Ca	2006	Post (prio	-23380	Alive	TUMOR F	1569						Negative	0	1569	0	1569	0	1569	0	1569
TCGA-BH	TCGA-BH	BRCA	44	FEMALE	WHITE	Stage IA	Infiltrating Ductal Ca	2006	Pre (<6 m	-16310	Alive	TUMOR F	1572						Negative	0	1572	0	1572	0	1572	0	1572
TCGA-BH	TCGA-BH	BRCA	51	FEMALE	WHITE	Stage IIIC	Mixed Histology (ple	2006	Pre (<6 m	-18839	Alive	TUMOR F	1132						Negative	0	1132	0	1132	0	1132	0	1132
TCGA-BH	TCGA-BH	BRCA	51	FEMALE	WHITE	Stage IIIC	Mixed Histology (ple	2006	Pre (<6 m	-18839	Alive	TUMOR F	1132						Negative	0	1132	0	1132	0	1132	0	1132
TCGA-BH	TCGA-BH	BRCA	60	FEMALE	WHITE	Stage IIIC	Infiltrating Ductal Ca	2007	Post (prio	-21949	Alive	TUMOR F	974						Negative	0	974	0	974	0	974	0	974
TCGA-BH	TCGA-BH	BRCA	60	FEMALE	WHITE	Stage IIIC	Infiltrating Ductal Ca	2007	Post (prio	-21949	Alive	TUMOR F	974						Negative	0	974	0	974	0	974	0	974
TCGA-BH	TCGA-BH	BRCA	47	FEMALE	WHITE	Stage IIA	Infiltrating Ductal Ca	2009	Pre (<6 m	-17275	Alive	TUMOR F	554						Negative	0	554	0	554	0	554	0	554
TCGA-BH	TCGA-BH	BRCA	56	FEMALE	WHITE	Stage IIB	Infiltrating Ductal Ca	2009	Post (prio	-20691	Dead	TUMOR FREE		1324					Negative	1	1324	0	1324	0	1324	0	1324
TCGA-BH	TCGA-BH	BRCA	56	FEMALE	WHITE	Stage IIB	Infiltrating Ductal Ca	2009	Post (prio	-20691	Dead	TUMOR FREE		1324					Negative	1	1324	0	1324	0	1324	0	1324
TCGA-BH	TCGA-BH	BRCA	73	FEMALE	WHITE	Stage I	Infiltrating Ductal Ca	2008	Post (prio	-26988	Alive	TUMOR F	1871						Negative	0	1871	0	1871	0	1871	0	1871
TCGA-BH	TCGA-BH	BRCA	41	FEMALE	WHITE	Stage IIB	Infiltrating Ductal Ca	2008	Pre (<6 m	-15027	Alive	TUMOR F	660						Negative	0	660	0	660	0	660	0	660
TCGA-BH	TCGA-BH	BRCA	41	FEMALE	WHITE	Stage IIB	Infiltrating Ductal Ca	2008	Pre (<6 m	-15027	Alive	TUMOR F	660						Negative	0	660	0	660	0	660	0	660
TCGA-BH	TCGA-BH	BRCA	35	FEMALE	WHITE	Stage I	Infiltrating Ductal Ca	2007	Pre (<6 m	-12841	Alive	TUMOR F	2278						Negative	0	2278	0	2278	0	2278	0	2278
TCGA-BH	TCGA-BH	BRCA	35	FEMALE	WHITE	Stage I	Infiltrating Ductal Ca	2007	Pre (<6 m	-12841	Alive	TUMOR F	2278						Negative	0	2278	0	2278	0	2278	0	2278
TCGA-BH	TCGA-BH	BRCA	54	FEMALE	WHITE	Stage IIB	Infiltrating Ductal Ca	2007	Post (prio	-20049	Alive	TUMOR F	1876						Negative	0	1876	0	1876	0	1876	0	1876
TCGA-BH	TCGA-BH	BRCA	54	FEMALE	WHITE	Stage IIB	Infiltrating Ductal Ca	2007	Post (prio	-20049	Alive	TUMOR F	1876						Negative	0	1876	0	1876	0	1876	0	1876
TCGA-BH	TCGA-BH	BRCA	54	FEMALE	WHITE	Stage I	Infiltrating Ductal Ca	2007	Pre (<6 m	-19767	Alive	TUMOR F	2197						Negative	0	2197	0	2197	0	2197	0	2197
TCGA-BH	TCGA-BH	BRCA	54	FEMALE	WHITE	Stage I	Infiltrating Ductal Ca	2007	Pre (<6 m	-19767	Alive	TUMOR F	2197						Negative	0	2197	0	2197	0	2197	0	2197
TCGA-BH	TCGA-BH	BRCA	76	FEMALE	WHITE	Stage I	Infiltrating Ductal Ca	2006	Post (prio	-27917	Dead	TUMOR FREE		2296					Negative	1	2296	0	2296	0	2296	0	2296
TCGA-BH	TCGA-BH	BRCA	39	FEMALE	WHITE	Stage I	Infiltrating Ductal Ca	2007	Pre (<6 m	-14494	Alive	TUMOR F	2255		Locoregic	Other, sp	Dermis ar	1709	Negative	0	2255	0	2255	1	1709	1	1709
TCGA-BH	TCGA-BH	BRCA	39	FEMALE	WHITE	Stage I	Infiltrating Ductal Ca	2007	Pre (<6 m	-14494	Alive	TUMOR F	2255		Locoregic	Other, sp	Dermis ar	1709	Negative	0	2255	0	2255	1	1709	1	1709
TCGA-BH	TCGA-BH	BRCA	59	FEMALE	WHITE	Stage I	Infiltrating Ductal Ca	2007	Post (prio	-21622	Alive	TUMOR F	2330						Negative	0	2330	0	2330	0	2330	0	2330
TCGA-BH	TCGA-BH	BRCA	55	FEMALE	WHITE	Stage IIIA	Infiltrating Ductal Ca	2007	Post (prio	-20374	Alive	TUMOR F	2612						Negative	0	2612	0	2612	0	2612	0	2612
TCGA-BH	TCGA-BH	BRCA	55	FEMALE	WHITE	Stage IIIA	Infiltrating Ductal Ca	2007	Post (prio	-20374	Alive	TUMOR F	2612						Negative	0	2612	0	2612	0	2612	0	2612
TCGA-BH	TCGA-BH	BRCA	56	FEMALE	WHITE	Stage IIA	Infiltrating Ductal Ca	2007		-20625	Alive	TUMOR F	2365						Negative	0	2365	0	2365	0	2365	0	2365
TCGA-BH	TCGA-BH	BRCA	56	FEMALE	WHITE	Stage IIA	Infiltrating Ductal Ca	2007		-20625	Alive	TUMOR F	2365						Negative	0	2365	0	2365	0	2365	0	2365
TCGA-BH	TCGA-BH	BRCA	78	FEMALE	WHITE	Stage IIB	Infiltrating Ductal Ca	2006	Post (prio	-28699	Alive	TUMOR F	1519						Negative	0	1519	0	1519	0	1519	0	1519
TCGA-BH	TCGA-BH	BRCA	78	FEMALE	WHITE	Stage IIB	Infiltrating Ductal Ca	2006	Post (prio	-28699	Alive	TUMOR F	1519						Negative	0	1519	0	1519	0	1519	0	1519
TCGA-BH	TCGA-BH	BRCA	71	FEMALE	BLACK OI	Stage I	Infiltrating Ductal Ca	2006	Post (prio	-25974	Alive	TUMOR F	2371						Negative	0	2371	0	2371	0	2371	0	2371
TCGA-BH	TCGA-BH	BRCA	71	FEMALE	BLACK OI	Stage I	Infiltrating Ductal Ca	2006	Post (prio	-25974	Alive	TUMOR F	2371						Negative	0	2371	0	2371	0	2371	0	2371
TCGA-BH	TCGA-BH	BRCA	59	FEMALE	WHITE	Stage IIIA	Infiltrating Ductal Ca	2007	Post (prio	-21856	Alive	TUMOR F	2255						Negative	0	2255	0	2255	0	2255	0	2255
TCGA-BH	TCGA-BH	BRCA	59	FEMALE	WHITE	Stage IIIA	Infiltrating Ductal Ca	2007	Post (prio	-21856	Alive	TUMOR F	2255						Negative	0	2255	0	2255	0	2255	0	2255
TCGA-BH	TCGA-BH	BRCA	62	FEMALE	WHITE	Stage IIA	Infiltrating Ductal Ca	2007	Post (prio	-23006	Alive	TUMOR F	1270						Negative	0	1270	0	1270	0	1270	0	1270
TCGA-BH	TCGA-BH	BRCA	62	FEMALE	WHITE	Stage IIA	Infiltrating Ductal Ca	2007	Post (prio	-23006	Alive	TUMOR F	1270						Negative	0	1270	0	1270	0	1270	0	1270
TCGA-BH	TCGA-BH	BRCA	61	FEMALE	WHITE	Stage IIIA	Infiltrating Lobular C	2007	Post (prio	-22377	Dead	WITH TUMOR		1411	Distant M	Bone		1123	Negative	1	1411	1	1411	1	1123	1	1123
TCGA-BH	TCGA-BH	BRCA	47	FEMALE	WHITE	Stage I	Mixed Histology (ple	2006	Pre (<6 m	-17221	Alive	WITH TUN	2709		Distant M	Bone		2674	Negative	0	2709	0	2709	1	2674	1	2674
TCGA-BH	TCGA-BH	BRCA	47	FEMALE	WHITE	Stage I	Mixed Histology (ple	2006	Pre (<6 m	-17221	Alive	WITH TUN	2709		Distant M	Bone		2674	Negative	0	2709	0	2709	1	2674	1	2674
TCGA-BH	TCGA-BH	BRCA	48	FEMALE	WHITE	Stage IIB	Infiltrating Ductal Ca	2006	Peri (6-12	-17871	Alive	TUMOR F	2767						Negative	0	2767	0	2767	0	2767	0	2767
TCGA-BH	TCGA-BH	BRCA	58	MALE	WHITE	Stage IIB	Infiltrating Ductal Ca	2006		-21386	Alive	TUMOR F	2486						Negative	0	2486	0	2486	0	2486	0	2486
TCGA-BH	TCGA-BH	BRCA	58	MALE	WHITE	Stage IIB	Infiltrating Ductal Ca	2006		-21386	Alive	TUMOR F	2486						Negative	0	2486	0	2486	0	2486	0	2486
TCGA-BH	TCGA-BH	BRCA	62	FEMALE	WHITE	Stage IIA	Infiltrating Ductal Ca	2007	Post (prio	-22656	Alive	TUMOR F	2372						Negative	0	2372	0	2372	0	2372	0	2372
TCGA-BH	TCGA-BH	BRCA	62	FEMALE	WHITE	Stage IIA	Infiltrating Ductal Ca	2007	Post (prio	-22656	Alive	TUMOR F	2372						Negative	0	2372	0	2372	0	2372	0	2372
TCGA-BH	TCGA-BH	BRCA	30	FEMALE	BLACK OI	Stage IIA	Infiltrating Ductal Ca	2007	Pre (<6 m	-11204	Alive	TUMOR F	2041						Negative	0	2041	0	2041	0	2041	0	2041
TCGA-BH	TCGA-BH	BRCA	30	FEMALE	BLACK OI	Stage IIA	Infiltrating Ductal Ca	2007	Pre (<6 m	-11204	Alive	TUMOR F	2041						Negative	0	2041	0	2041	0	2041	0	2041
TCGA-BH	TCGA-BH	BRCA	63	FEMALE	WHITE	Stage IIB	Infiltrating Ductal Ca	2007	Post (prio	-23366	Alive	TUMOR F	1156						Negative	0	1156	0	1156	0	1156	0	1156
TCGA-BH	TCGA-BH	BRCA	63	FEMALE	WHITE	Stage IIB	Infiltrating Ductal Ca	2007	Post (prio	-23366	Alive	TUMOR F	1156						Negative	0	1156	0	1156	0	1156	0	1156
TCGA-BH	TCGA-BH	BRCA	63	FEMALE	WHITE	Stage IIB	Infiltrating Ductal Ca	2009	Post (prio	-23327	Alive	TUMOR F	912						Negative	0	912	0	912	0	912	0	912
TCGA-BH	TCGA-BH	BRCA	63	FEMALE	WHITE	Stage IIB	Infiltrating Ductal Ca	2009	Post (prio	-23327	Alive	TUMOR F	912						Negative	0	912	0	912	0	912	0	912
TCGA-BH	TCGA-BH	BRCA	49	FEMALE	WHITE	Stage IIA	Infiltrating Ductal Ca	2009	Pre (<6 m	-18237	Alive	TUMOR F	423						Negative	0	423	0	423	0	423	0	423
TCGA-BH	TCGA-BH	BRCA	49	FEMALE	WHITE	Stage IIA	Infiltrating Ductal Ca	2009	Pre (<6 m	-18237	Alive	TUMOR F	423						Negative	0	423	0	423	0	423	0	423
TCGA-BH	TCGA-BH	BRCA	64	FEMALE	WHITE	Stage IIA	Other, specify	2007	Post (prio	-23628	Alive	TUMOR F	2381						Negative	0	2381	0	2381	0	2381	0	2381
TCGA-BH	TCGA-BH	BRCA	64	FEMALE	WHITE	Stage IIA	Other, specify	2007	Post (prio	-23628	Alive	TUMOR F	2381						Negative	0	2381	0	2381	0	2381	0	2381
TCGA-BH	TCGA-BH	BRCA	78	FEMALE	WHITE	Stage I	Infiltrating Ductal Ca	2009	Post (prio	-28529	Alive	TUMOR F	1644						Negative	0	1644	0	1644	0	1644	0	1644
TCGA-BH	TCGA-BH	BRCA	78	FEMALE	WHITE	Stage I	Infiltrating Ductal Ca	2009	Post (prio	-28529	Alive	TUMOR F	1644						Negative	0	1644	0	1644	0	1644	0	1644
TCGA-BH	TCGA-BH	BRCA	60	FEMALE	WHITE	Stage IIB	Infiltrating Lobular C	2009	Post (prio	-22199	Alive	TUMOR F	476						Negative	0	476	0	476	0	476	0	476
TCGA-BH	TCGA-BH	BRCA	60	FEMALE	WHITE	Stage IIB	Infiltrating Lobular C	2009	Post (prio	-22199	Alive	TUMOR F	476						Negative	0	476	0	476	0	476	0	476
TCGA-BH	TCGA-BH	BRCA	42	FEMALE	WHITE	Stage IIB	Infiltrating Ductal Ca	2009	Pre (<6 m	-15533	Alive	TUMOR F	98						Negative	0	98	0	98	0	98	0	98
TCGA-BH	TCGA-BH	BRCA	42	FEMALE	WHITE	Stage IIB	Infiltrating Ductal Ca	2009	Pre (<6 m	-15533	Alive	TUMOR F	98						Negative	0	98	0	98	0	98	0	98
TCGA-BH	TCGA-BH	BRCA	71	FEMALE	WHITE	Stage IIIA	Infiltrating Ductal Ca	2010	Post (prio	-25944	Alive	TUMOR F	78						Negative	0	78	0	78	0	78	0	78
TCGA-BH	TCGA-BH	BRCA	41	FEMALE	WHITE	Stage IIA	Infiltrating Ductal Ca	2007	Pre (<6 m	-15078	Alive	TUMOR F	2403						Negative	0	2403	0	2403	0	2403	0	2403
TCGA-BH	TCGA-BH	BRCA	41	FEMALE	WHITE	Stage IIA	Infiltrating Ductal Ca	2007	Pre (<6 m	-15078	Alive	TUMOR F	2403						Negative	0	2403	0	2403	0	2403	0	2403
TCGA-BH	TCGA-BH	BRCA	54	FEMALE	WHITE	Stage IIIA	Infiltrating Ductal Ca	2007	Peri (6-12	-19781	Alive	TUMOR F	2064						Negative	0	2064	0	2064	0	2064	0	2064
TCGA-BH	TCGA-BH	BRCA	54	FEMALE	WHITE	Stage IIIA	Infiltrating Ductal Ca	2007	Peri (6-12	-19781	Alive	TUMOR F	2064						Negative	0	2064	0	2064	0	2064	0	2064
TCGA-BH	TCGA-BH	BRCA	62	FEMALE	WHITE	Stage I	Infiltrating Ductal Ca	2007	Post (prio	-22833	Alive	TUMOR F	2156						Negative	0	2156	0	2156	0	2156	0	2156
TCGA-BH	TCGA-BH	BRCA	43	FEMALE	WHITE	Stage IIB	Infiltrating Ductal Ca	2009	Pre (<6 m	-15980	Alive	TUMOR F	495						Negative	0	495	0	495	0	495	0	495
TCGA-BH	TCGA-BH	BRCA	43	FEMALE	WHITE	Stage IIB	Infiltrating Ductal Ca	2009	Pre (<6 m	-15980	Alive	TUMOR F	495						Negative	0	495	0	495	0	495	0	495
TCGA-BH	TCGA-BH	BRCA	38	FEMALE	WHITE	Stage IIIC	Infiltrating Ductal Ca	2009	Pre (<6 m	-14175	Alive	TUMOR F	134						Negative	0	134	0	134	0	134	0	134
TCGA-BH	TCGA-BH	BRCA	38	FEMALE	WHITE	Stage IIIC	Infiltrating Ductal Ca	2009	Pre (<6 m	-14175	Alive	TUMOR F	134						Negative	0	134	0	134	0	134	0	134
TCGA-BH	TCGA-BH	BRCA	52	FEMALE	WHITE	Stage IIB	Infiltrating Ductal Ca	2009	Peri (6-12	-19192	Alive	TUMOR F	477						Negative	0	477	0	477	0	477	0	477
TCGA-BH	TCGA-BH	BRCA	52	FEMALE	WHITE	Stage IIB	Infiltrating Ductal Ca	2009	Peri (6-12	-19192	Alive	TUMOR F	477						Negative	0	477	0	477	0	477	0	477
TCGA-BH	TCGA-BH	BRCA	49	FEMALE	BLACK OI	Stage IIIA	Infiltrating Ductal Ca	2009	Pre (<6 m	-18152	Alive	TUMOR F	435						Negative	0	435	0	435	0	435	0	435
TCGA-BH	TCGA-BH	BRCA	69	FEMALE	WHITE	Stage IA	Infiltrating Ductal Ca	2009	Post (prio	-25408	Alive	TUMOR F	293						Negative	0	293	0	293	0	293	0	293
TCGA-BH	TCGA-BH	BRCA	79	FEMALE	WHITE	Stage IIB	Infiltrating Ductal Ca	2006	Post (prio	-29115	Alive	TUMOR F	1363						Negative	0	1363	0	1363	0	1363	0	1363
TCGA-BH	TCGA-BH	BRCA	53	FEMALE	WHITE	Stage IIB	Infiltrating Lobular C	2006	Post (prio	-19415	Alive	TUMOR F	2489						Negative	0	2489	0	2489	0	2489	0	2489
TCGA-BH	TCGA-BH	BRCA	72	FEMALE	WHITE	Stage IIA	Infiltrating Ductal Ca	2008	Post (prio	-26548	Dead	TUMOR FREE		991					Negative	1	991	0	991	0	991	0	991
TCGA-BH	TCGA-BH	BRCA	69	FEMALE	WHITE	Stage IA	Infiltrating Ductal Ca	2008	Post (prio	-25259	Alive	TUMOR F	745						Negative	0	745	0	745	0	745	0	745

TCGA-BH	TCGA-BH	BRCA	68	FEMALE	WHITE	Stage IIB	Infiltrating Ductal Ca	2007	Post (prio	-24850	Alive	TUMOR F	943						Negative	0	943	0	943	0	943	0	943
TCGA-BH	TCGA-BH	BRCA	51	FEMALE	WHITE	Stage IIA	Infiltrating Ductal Ca	2008	Peri (6-12	-18741	Alive	TUMOR F	1926						Negative	0	1926	0	1926	0	1926	0	1926
TCGA-BH	TCGA-BH	BRCA	67	FEMALE	WHITE	Stage IIA	Infiltrating Ductal Ca	2008	Post (prio	-24493	Alive	TUMOR F	923						Negative	0	923	0	923	0	923	0	923
TCGA-BH	TCGA-BH	BRCA	62	FEMALE	WHITE	Stage IIA	Infiltrating Ductal Ca	2009	Post (prio	-22714	Alive	TUMOR F	328						Negative	0	328	0	328	0	328	0	328
TCGA-BH	TCGA-BH	BRCA	69	FEMALE	WHITE	Stage IA	Infiltrating Ductal Ca	2009	Post (prio	-25223	Alive	TUMOR F	461						Negative	0	461	0	461	0	461	0	461
TCGA-BH	TCGA-BH	BRCA	46	FEMALE	WHITE	Stage I	Infiltrating Ductal Ca	2008	Pre (<6 m	-17152	Alive	TUMOR F	1928						Negative	0	1928	0	1928	0	1928	0	1928
TCGA-BH	TCGA-BH	BRCA	45	FEMALE	WHITE	Stage I	Infiltrating Ductal Ca	2008	Post (prio	-16719	Alive	TUMOR F	1620						Negative	0	1620	0	1620	0	1620	0	1620
TCGA-BH	TCGA-BH	BRCA	45	FEMALE	WHITE	Stage I	Infiltrating Ductal Ca	2008	Post (prio	-16719	Alive	TUMOR F	1620						Negative	0	1620	0	1620	0	1620	0	1620
TCGA-BH	TCGA-BH	BRCA	82	FEMALE	WHITE	Stage I	Infiltrating Ductal Ca	2008	Post (prio	-30125	Alive	TUMOR F	747						Negative	0	747	0	747	0	747	0	747
TCGA-BH	TCGA-BH	BRCA	65	FEMALE	WHITE	Stage IIIA	Infiltrating Ductal Ca	2008	Post (prio	-23972	Alive	TUMOR F	702						Negative	0	702	0	702	0	702	0	702
TCGA-BH	TCGA-BH	BRCA	65	FEMALE	WHITE	Stage IIIA	Infiltrating Ductal Ca	2008	Post (prio	-23972	Alive	TUMOR F	702						Negative	0	702	0	702	0	702	0	702
TCGA-BH	TCGA-BH	BRCA	69	FEMALE	WHITE	Stage IIA	Infiltrating Ductal Ca	2007	Post (prio	-25289	Alive	TUMOR F	1247						Negative	0	1247	0	1247	0	1247	0	1247
TCGA-BH	TCGA-BH	BRCA	69	FEMALE	WHITE	Stage IIA	Infiltrating Ductal Ca	2007	Post (prio	-25289	Alive	TUMOR F	1247						Negative	0	1247	0	1247	0	1247	0	1247
TCGA-BH	TCGA-BH	BRCA	31	FEMALE	WHITE	Stage I	Infiltrating Ductal Ca	2008	Pre (<6 m	-11379	Alive	TUMOR F	1611						Negative	0	1611	0	1611	0	1611	0	1611
TCGA-BH	TCGA-BH	BRCA	31	FEMALE	WHITE	Stage I	Infiltrating Ductal Ca	2008	Pre (<6 m	-11379	Alive	TUMOR F	1611						Negative	0	1611	0	1611	0	1611	0	1611
TCGA-BH	TCGA-BH	BRCA	55	FEMALE	WHITE	Stage IA	Infiltrating Ductal Ca	2008	Post (prio	-20448	Alive	TUMOR F	806						Negative	0	806	0	806	0	806	0	806
TCGA-BH	TCGA-BH	BRCA	77	FEMALE	WHITE	Stage IA	Infiltrating Ductal Ca	2008	Post (prio	-28233	Alive	TUMOR F	727						Negative	0	727	0	727	0	727	0	727
TCGA-BH	TCGA-BH	BRCA	78	FEMALE	WHITE	Stage IA	Infiltrating Ductal Ca	2008	Post (prio	-28644	Alive	TUMOR F	620						Negative	0	620	0	620	0	620	0	620
TCGA-BH	TCGA-BH	BRCA	81	FEMALE	WHITE	Stage IIB	Infiltrating Ductal Ca	2010	Post (prio	-29751	Alive	TUMOR F	178						Negative	0	178	0	178	0	178	0	178
TCGA-BH	TCGA-BH	BRCA	81	FEMALE	WHITE	Stage IIB	Infiltrating Ductal Ca	2010	Post (prio	-29751	Alive	TUMOR F	178						Negative	0	178	0	178	0	178	0	178
TCGA-BH	TCGA-BH	BRCA	56	FEMALE	WHITE	Stage IIB	Infiltrating Ductal Ca	2009	Post (prio	-20804	Alive	TUMOR F	72						Negative	0	72	0	72	0	72	0	72
TCGA-BH	TCGA-BH	BRCA	67	FEMALE	WHITE	Stage IA	Infiltrating Ductal Ca	2009	Post (prio	-24731	Alive	TUMOR F	516						Negative	0	516	0	516	0	516	0	516
TCGA-BH	TCGA-BH	BRCA	48	FEMALE	WHITE	Stage IIA	Infiltrating Ductal Ca	2009	Pre (<6 m	-17710	Alive	TUMOR F	76						Negative	0	76	0	76	0	76	0	76
TCGA-BH	TCGA-BH	BRCA	65	FEMALE	WHITE	Stage IIIA	Infiltrating Lobular C	2009	Post (prio	-23883	Alive	TUMOR F	414						Negative	0	414	0	414	0	414	0	414
TCGA-BH	TCGA-BH	BRCA	56	FEMALE	WHITE	Stage IIA	Infiltrating Ductal Ca	2007	Post (prio	-20719	Alive	TUMOR F	1121						Negative	0	1121	0	1121	0	1121	0	1121
TCGA-BH	TCGA-BH	BRCA	52	FEMALE	WHITE	Stage IA	Infiltrating Ductal Ca	2009	Peri (6-12	-19216	Alive	TUMOR F	392						Negative	0	392	0	392	0	392	0	392
TCGA-BH	TCGA-BH	BRCA	62	FEMALE	ASIAN	Stage IA	Infiltrating Ductal Ca	2006	Post (prio	-22962	Alive	TUMOR F	1561						Negative	0	1561	0	1561	0	1561	0	1561
TCGA-BH	TCGA-BH	BRCA	54	FEMALE	WHITE	Stage IIB	Infiltrating Ductal Ca	2008	Post (prio	-19900	Alive	TUMOR F	829						Negative	0	829	0	829	0	829	0	829
TCGA-BH	TCGA-BH	BRCA	60	FEMALE	WHITE	Stage I	Infiltrating Ductal Ca	2009	Post (prio	-22179	Alive	TUMOR F	1545						Negative	0	1545	0	1545	0	1545	0	1545
TCGA-BH	TCGA-BH	BRCA	59	FEMALE	WHITE	Stage IIA	Infiltrating Ductal Ca	2010	Post (prio	-21857	Alive	TUMOR F	170						Negative	0	170	0	170	0	170	0	170
TCGA-BH	TCGA-BH	BRCA	58	FEMALE	WHITE	Stage IIA	Infiltrating Ductal Ca	2010	Indetermi	-21369	Alive	TUMOR F	728							0	728	0	728			0	728
TCGA-BH	TCGA-BH	BRCA	46	FEMALE	WHITE	Stage IIA	Infiltrating Ductal Ca	2010		-16911	Alive	TUMOR F	759							0	759	0	759			0	759
TCGA-BH	TCGA-BH	BRCA	77	FEMALE	WHITE	Stage IIA	Infiltrating Ductal Ca	2008		-28304	Alive	TUMOR F	1288						Negative	0	1288	0	1288	0	1288	0	1288
TCGA-BH	TCGA-BH	BRCA	49	FEMALE	WHITE	Stage I	Infiltrating Ductal Ca	2008		-17996	Alive	TUMOR F	1363						Negative	0	1363	0	1363	0	1363	0	1363
TCGA-BH	TCGA-BH	BRCA	82	FEMALE	WHITE	Stage I		2008		-30025	Alive	TUMOR F	701						Negative	0	701	0	701	0	701	0	701
TCGA-BH	TCGA-BH	BRCA	50	FEMALE	WHITE	Stage IIA	Infiltrating Ductal Ca	2010	Peri (6-12	-18594	Alive	TUMOR F	1001						Negative	0	1001	0	1001	0	1001	0	1001
TCGA-BH	TCGA-BH	BRCA	50	FEMALE	WHITE	Stage IIA	Infiltrating Ductal Ca	2010	Peri (6-12	-18594	Alive	TUMOR F	1001						Negative	0	1001	0	1001	0	1001	0	1001
TCGA-BH	TCGA-BH	BRCA	81	FEMALE	WHITE	Stage IA	Infiltrating Ductal Ca	2010	Post (prio	-29820	Alive	TUMOR F	149						Negative	0	149	0	149	0	149	0	149
TCGA-BH	TCGA-BH	BRCA	63	FEMALE	WHITE	Stage IA	Infiltrating Ductal Ca	2010	Post (prio	-23065	Alive	TUMOR F	652						Negative	0	652	0	652	0	652	0	652
TCGA-BH	TCGA-BH	BRCA	53	FEMALE	WHITE	Stage IIA	Infiltrating Ductal Ca	2010	Post (prio	-19489	Alive	TUMOR F	1093						Negative	0	1093	0	1093	0	1093	0	1093
TCGA-BH	TCGA-BH	BRCA	56	FEMALE	WHITE	Stage IV	Infiltrating Ductal Ca	2000		-20678	Dead	WITH TUMOR		612					Negative	1	612	1	612			1	612
TCGA-BH	TCGA-BH	BRCA	56	FEMALE	WHITE	Stage IV	Infiltrating Ductal Ca	2000		-20678	Dead	WITH TUMOR		612					Negative	1	612	1	612			1	612
TCGA-BH	TCGA-BH	BRCA	46	FEMALE	WHITE	Stage I	Infiltrating Ductal Ca	1999		-16824	Dead	WITH TUMOR		2763					Negative	1	2763	1	2763			1	2763
TCGA-BH	TCGA-BH	BRCA	46	FEMALE	WHITE	Stage I	Infiltrating Ductal Ca	1999		-16824	Dead	WITH TUMOR		2763					Negative	1	2763	1	2763			1	2763
TCGA-BH	TCGA-BH	BRCA	50	FEMALE	WHITE	Stage IIIA	Infiltrating Ductal Ca	2003		-18519	Dead			811					Negative	1	811		811	0	811	0	811
TCGA-BH	TCGA-BH	BRCA	50	FEMALE	WHITE	Stage IIIA	Infiltrating Ductal Ca	2003		-18519	Dead			811					Negative	1	811		811	0	811	0	811
TCGA-BH	TCGA-BH	BRCA	39	FEMALE	WHITE	Stage IIIA	Infiltrating Ductal Ca	2001		-14532	Dead			2207					Negative	1	2207		2207	0	2207	0	2207
TCGA-BH	TCGA-BH	BRCA	39	FEMALE	WHITE	Stage IIIA	Infiltrating Ductal Ca	2001		-14532	Dead			2207					Negative	1	2207		2207	0	2207	0	2207
TCGA-BH	TCGA-BH	BRCA	88	FEMALE	WHITE	Stage IIA	Infiltrating Ductal Ca	2001		-32404	Dead			1148					Negative	1	1148		1148	0	1148	0	1148
TCGA-BH	TCGA-BH	BRCA	88	FEMALE	WHITE	Stage IIA	Infiltrating Ductal Ca	2001		-32404	Dead			1148					Negative	1	1148		1148	0	1148	0	1148
TCGA-BH	TCGA-BH	BRCA	60	FEMALE	WHITE	Stage I	Infiltrating Ductal Ca	2003		-22222	Dead	WITH TUMOR		921					Negative	1	921	1	921			1	921
TCGA-BH	TCGA-BH	BRCA	60	FEMALE	WHITE	Stage I	Infiltrating Ductal Ca	2003		-22222	Dead	WITH TUMOR		921					Negative	1	921	1	921			1	921
TCGA-BH	TCGA-BH	BRCA	56	FEMALE	WHITE	Stage IIB	Infiltrating Ductal Ca	2001		-20675	Dead			1692					Negative	1	1692		1692	0	1692	0	1692
TCGA-BH	TCGA-BH	BRCA	56	FEMALE	WHITE	Stage IIB	Infiltrating Ductal Ca	2001		-20675	Dead			1692					Negative	1	1692		1692	0	1692	0	1692
TCGA-BH	TCGA-BH	BRCA	50	FEMALE	WHITE	Stage IIA	Infiltrating Ductal Ca	2001		-18524	Dead	TUMOR FREE		1142					Negative	1	1142	0	1142	0	1142	0	1142
TCGA-BH	TCGA-BH	BRCA	50	FEMALE	WHITE	Stage IIA	Infiltrating Ductal Ca	2001		-18524	Dead	TUMOR FREE		1142					Negative	1	1142	0	1142	0	1142	0	1142
TCGA-BH	TCGA-BH	BRCA	79	FEMALE	WHITE	Stage I	Mucinous Carcinoma	2003	Post (prio	-28974	Dead	TUMOR FREE		2009					Negative	1	2009	0	2009	0	2009	0	2009
TCGA-BH	TCGA-BH	BRCA	79	FEMALE	WHITE	Stage I	Mucinous Carcinoma	2003	Post (prio	-28974	Dead	TUMOR FREE		2009					Negative	1	2009	0	2009	0	2009	0	2009
TCGA-BH	TCGA-BH	BRCA	70	FEMALE	WHITE	Stage IIA	Infiltrating Ductal Ca	2003	Post (prio	-25674	Dead	WITH TUMOR		224					Negative	1	224	1	224			1	224
TCGA-BH	TCGA-BH	BRCA	72	FEMALE	WHITE	Stage IIIA	Infiltrating Ductal Ca	2003	Post (prio	-26426	Dead	TUMOR FREE		1563					Negative	1	1563	0	1563	0	1563	0	1563
TCGA-BH	TCGA-BH	BRCA	72	FEMALE	WHITE	Stage IIIA	Infiltrating Ductal Ca	2003	Post (prio	-26426	Dead	TUMOR FREE		1563					Negative	1	1563	0	1563	0	1563	0	1563
TCGA-BH	TCGA-BH	BRCA	48	FEMALE	WHITE	Stage IIB	Infiltrating Ductal Ca	2001		-17682	Dead			1556					Negative	1	1556		1556	0	1556	0	1556
TCGA-BH	TCGA-BH	BRCA	48	FEMALE	WHITE	Stage IIB	Infiltrating Ductal Ca	2001		-17682	Dead			1556					Negative	1	1556		1556	0	1556	0	1556
TCGA-BH	TCGA-BH	BRCA	48	FEMALE	WHITE	Stage IIB	Infiltrating Ductal Ca	2001		-17682	Dead			1556					Negative	1	1556		1556	0	1556	0	1556
TCGA-BH	TCGA-BH	BRCA	78	FEMALE	WHITE	Stage IIA	Infiltrating Ductal Ca	1999	Post (prio	-28724	Dead	TUMOR FREE		2127					Negative	1	2127	0	2127	0	2127	0	2127
TCGA-BH	TCGA-BH	BRCA	78	FEMALE	WHITE	Stage IIA	Infiltrating Ductal Ca	1999	Post (prio	-28724	Dead	TUMOR FREE		2127					Negative	1	2127	0	2127	0	2127	0	2127
TCGA-BH	TCGA-BH	BRCA	68	FEMALE	WHITE	Stage IIA	Mixed Histology (ple	1999	Post (prio	-25136	Dead	TUMOR FREE		2798						1	2798	0	2798			0	2798
TCGA-BH	TCGA-BH	BRCA	68	FEMALE	WHITE	Stage IIA	Mixed Histology (ple	1999	Post (prio	-25136	Dead	TUMOR FREE		2798						1	2798	0	2798			0	2798
TCGA-BH	TCGA-BH	BRCA	35	FEMALE	WHITE	Stage IIB	Infiltrating Ductal Ca	1999	Pre (<6 m	-13138	Dead	WITH TUMOR		3462						1	3462	1	3462			1	3462
TCGA-BH	TCGA-BH	BRCA	35	FEMALE	WHITE	Stage IIB	Infiltrating Ductal Ca	1999	Pre (<6 m	-13138	Dead	WITH TUMOR		3462						1	3462	1	3462			1	3462
TCGA-BH	TCGA-BH	BRCA	35	FEMALE	WHITE	Stage IIB	Infiltrating Ductal Ca	1999	Pre (<6 m	-13138	Dead	WITH TUMOR		3462						1	3462	1	3462			1	3462
TCGA-BH	TCGA-BH	BRCA	55	FEMALE	WHITE	Stage I	Infiltrating Ductal Ca	1999		-20425	Dead	TUMOR FREE		2520						1	2520	0	2520			0	2520
TCGA-BH	TCGA-BH	BRCA	55	FEMALE	WHITE	Stage I	Infiltrating Ductal Ca	1999		-20425	Dead	TUMOR FREE		2520						1	2520	0	2520			0	2520
TCGA-BH	TCGA-BH	BRCA	83	FEMALE	WHITE	Stage I	Infiltrating Ductal Ca	1999	Post (prio	-30339	Dead	TUMOR FREE		1286						1	1286	0	1286			0	1286
TCGA-BH	TCGA-BH	BRCA	83	FEMALE	WHITE	Stage I	Infiltrating Ductal Ca	1999	Post (prio	-30339	Dead	TUMOR FREE		1286						1	1286	0	1286			0	1286
TCGA-BH	TCGA-BH	BRCA	45	FEMALE	WHITE	Stage IIIA	Infiltrating Ductal Ca	1999		-16504	Dead	WITH TUMOR		365						1	365	1	365			1	365
TCGA-BH	TCGA-BH	BRCA	45	FEMALE	WHITE	Stage IIIA	Infiltrating Ductal Ca	1999		-16504	Dead	WITH TUMOR		365						1	365	1	365			1	365
TCGA-BH	TCGA-BH	BRCA	38	FEMALE	WHITE	Stage IIA	Infiltrating Ductal Ca	1999	Pre (<6 m	-14090	Dead	WITH TUMOR		1694						1	1694	1	1694			1	1694
TCGA-BH	TCGA-BH	BRCA	38	FEMALE	WHITE	Stage IIA	Infiltrating Ductal Ca	1999	Pre (<6 m	-14090	Dead	WITH TUMOR		1694						1	1694	1	1694			1	1694
TCGA-BH	TCGA-BH	BRCA	67	FEMALE	WHITE	Stage IIB	Infiltrating Ductal Ca	2000	Post (prio	-24544	Dead	WITH TUMOR		1508						1	1508	1	1508			1	1508
TCGA-BH	TCGA-BH	BRCA	79	FEMALE	WHITE	Stage IIA	Infiltrating Ductal Ca	2000	Post (prio	-28940	Dead	WITH TUMOR		538	Distant M	Lung	Liver	396		1	538	1	538			1	396
TCGA-BH	TCGA-BH	BRCA	79	FEMALE	WHITE	Stage IIA	Infiltrating Ductal Ca	2000	Post (prio	-28940	Dead	WITH TUMOR		538	Distant M	Lung	Liver	396		1	538	1	538			1	396
TCGA-BH	TCGA-BH	BRCA	80	FEMALE	WHITE	Stage IIA	Infiltrating Ductal Ca	2000	Post (prio	-29335	Dead	WITH TUMOR		785						1	785	1	785			1	785
TCGA-BH	TCGA-BH	BRCA	80	FEMALE	WHITE	Stage IIA	Infiltrating Ductal Ca	2000	Post (prio	-29335	Dead	WITH TUMOR		785						1	785	1	785			1	785
TCGA-BH	TCGA-BH	BRCA	53	FEMALE	WHITE	Stage IIIB	Infiltrating Ductal Ca	1999		-19701	Dead	TUMOR FREE		959						1	959	0	959			0	959
TCGA-BH	TCGA-BH	BRCA	53	FEMALE	WHITE	Stage IIIB	Infiltrating Ductal Ca	1999		-19701	Dead	TUMOR FREE		959						1	959	0	959			0	959
TCGA-BH	TCGA-BH	BRCA	62	FEMALE	WHITE	Stage IIA	Infiltrating Ductal Ca	2002		-22842	Dead	TUMOR FREE		2712						1	2712	0	2712			0	2712
TCGA-BH	TCGA-BH	BRCA	62	FEMALE	WHITE	Stage IIA	Infiltrating Ductal Ca	2002		-22842	Dead	TUMOR FREE		2712						1	2712	0	2712			0	2712
TCGA-BH	TCGA-BH	BRCA	51	FEMALE	WHITE		Infiltrating Ductal Ca	1996		-18691	Dead			2965						1	2965		2965			0	2965
TCGA-BH	TCGA-BH	BRCA	51	FEMALE	WHITE		Infiltrating Ductal Ca	1996		-18691	Dead			2965						1	2965		2965			0	2965
TCGA-BH	TCGA-BH	BRCA	90	FEMALE	WHITE	Stage IIIB	Infiltrating Ductal Ca	1999	Post (prio	-32871	Dead	WITH TUMOR		763						1	763	1	763			1	763
TCGA-BH	TCGA-BH	BRCA	90	FEMALE	WHITE	Stage IIIB	Infiltrating Ductal Ca	1999	Post (prio	-32871	Dead	WITH TUMOR		763						1	763	1	763			1	763
TCGA-BH	TCGA-BH	BRCA	60	FEMALE	WHITE	Stage IIB	Infiltrating Ductal Ca	2000	Post (prio	-21933	Dead	WITH TUMOR		3669						1	3669	1	3669			1	3669
TCGA-BH	TCGA-BH	BRCA	60	FEMALE	WHITE	Stage IIB	Infiltrating Ductal Ca	2000	Post (prio	-21933	Dead	WITH TUMOR		3669						1	3669	1	3669			1	3669
TCGA-BH	TCGA-BH	BRCA	78	FEMALE	BLACK OI	Stage IIA	Medullary Carcinoma	1998	Post (prio	-28706	Dead	TUMOR FREE		3472						1	3472	0	3472			0	3472
TCGA-BH	TCGA-BH	BRCA	78	FEMALE	BLACK OI	Stage IIA	Medullary Carcinoma	1998	Post (prio	-28706	Dead	TUMOR FREE		3472						1	3472	0	3472			0	3472
TCGA-BH	TCGA-BH	BRCA	68	FEMALE	WHITE	Stage I	Infiltrating Ductal Ca	1998	Post (prio	-24940	Dead	WITH TUMOR		1009						1	1009	1	1009			1	1009
TCGA-BH	TCGA-BH	BRCA	68	FEMALE	WHITE	Stage I	Infiltrating Ductal Ca	1998	Post (prio	-24940	Dead	WITH TUMOR		1009						1	1009	1	1009			1	1009
TCGA-BH	TCGA-BH	BRCA	31	FEMALE	WHITE	Stage IIB	Infiltrating Ductal Ca	1998		-11650	Dead	WITH TUMOR		2273						1	2273	1	2273			1	2273
TCGA-BH	TCGA-BH	BRCA	31	FEMALE	WHITE	Stage IIB	Infiltrating Ductal Ca	1998		-11650	Dead	WITH TUMOR		2273						1	2273	1	2273			1	2273
TCGA-BH	TCGA-BH	BRCA	31	FEMALE	WHITE	Stage IIB	Infiltrating Ductal Ca	1998		-11650	Dead	WITH TUMOR		2273						1	2273	1	2273			1	2273
TCGA-BH	TCGA-BH	BRCA	88	FEMALE	WHITE	Stage I	Infiltrating Ductal Ca	1998	Post (prio	-32295	Dead			3736						1	3736		3736			0	3736
TCGA-BH	TCGA-BH	BRCA	88	FEMALE	WHITE	Stage I	Infiltrating Ductal Ca	1998	Post (prio	-32295	Dead			3736						1	3736		3736			0	3736
TCGA-BH	TCGA-BH	BRCA	47	FEMALE	WHITE	Stage IV	Infiltrating Ductal Ca	1998		-17172	Dead	WITH TUMOR		1034						1	1034	1	1034			1	1034
TCGA-BH	TCGA-BH	BRCA	47	FEMALE	WHITE	Stage IV	Infiltrating Ductal Ca	1998		-17172	Dead	WITH TUMOR		1034						1	1034	1	1034			1	1034
TCGA-BH	TCGA-BH	BRCA	66	FEMALE	WHITE	Stage IIIA	Infiltrating Ductal Ca	1998		-24262	Dead			1927						1	1927		1927			0	1927
TCGA-BH	TCGA-BH	BRCA	66	FEMALE	WHITE	Stage IIIA	Infiltrating Ductal Ca	1998		-24262	Dead			1927						1	1927		1927			0	1927
TCGA-BH	TCGA-BH	BRCA	69	FEMALE	WHITE	Stage IIB	Infiltrating Ductal Ca	1998		-25558	Dead	WITH TUMOR		1673						1	1673	1	1673			1	1673
TCGA-BH	TCGA-BH	BRCA	44	FEMALE	WHITE	Stage IIIA	Infiltrating Ductal Ca	1998		-16289	Dead			1388						1	1388		1388			0	1388
TCGA-BH	TCGA-BH	BRCA	44	FEMALE	WHITE	Stage IIIA	Infiltrating Ductal Ca	1998		-16289	Dead			1388						1	1388		1388			0	1388
TCGA-BH	TCGA-BH	BRCA	34	FEMALE	WHITE	Stage IIA	Infiltrating Ductal Ca	1997		-12525	Dead	WITH TUMOR		2192						1	2192	1	2192			1	2192
TCGA-BH	TCGA-BH	BRCA	34	FEMALE	WHITE	Stage IIA	Infiltrating Ductal Ca	1997		-12525	Dead	WITH TUMOR		2192						1	2192	1	2192			1	2192
TCGA-BH	TCGA-BH	BRCA	73	FEMALE	WHITE	Stage IIIB	Other, specify	1997		-26684	Dead	WITH TUMOR		1642						1	1642	1	1642			1	1642
TCGA-BH	TCGA-BH	BRCA	73	FEMALE	WHITE	Stage IIIB	Other, specify	1997		-26684	Dead	WITH TUMOR		1642						1	1642	1	1642			1	1642
TCGA-BH	TCGA-BH	BRCA	44	FEMALE	WHITE	Stage IA	Infiltrating Ductal Ca	1997		-16174	Dead	WITH TUMOR		1688						1	1688	1	1688			1	1688
TCGA-BH	TCGA-BH	BRCA	44	FEMALE	WHITE	Stage IA	Infiltrating Ductal Ca	1997		-16174	Dead	WITH TUMOR		1688						1	1688	1	1688			1	1688
TCGA-BH	TCGA-BH	BRCA	64	FEMALE	WHITE	Stage IA	Infiltrating Ductal Ca	2011	Post (prio	-23423	Alive	TUMOR F	856							0	856	0	856			0	856
TCGA-BH	TCGA-BH	BRCA	60	FEMALE	WHITE	Stage II	Infiltrating Ductal Ca	2011	Post (prio	-22052	Alive	TUMOR F	795							0	795	0	795			0	795
TCGA-BH	TCGA-BH	BRCA	78	FEMALE	WHITE	Stage IIB	Infiltrating Ductal Ca	1996	Post (prio	-28609	Dead	WITH TUMOR		1174						1	1174	1	1174			1	1174
TCGA-BH	TCGA-BH	BRCA	78	FEMALE	WHITE	Stage IIB	Infiltrating Ductal Ca	1996	Post (prio	-28609	Dead	WITH TUMOR		1174						1	1174	1	1174			1	1174
TCGA-BH	TCGA-BH	BRCA	80	FEMALE	WHITE	Stage IIB	Infiltrating Ductal Ca	1996	Post (prio	-29529	Dead			2534						1	2534		2534			0	2534
TCGA-BH	TCGA-BH	BRCA	80	FEMALE	WHITE	Stage IIB	Infiltrating Ductal Ca	1996	Post (prio	-29529	Dead			2534						1	2534		2534			0	2534
TCGA-BH	TCGA-BH	BRCA	48	FEMALE	WHITE	Stage IIB	Infiltrating Ductal Ca	1995		-17715	Dead	TUMOR FREE		1759						1	1759	0	1759			0	1759
TCGA-BH	TCGA-BH	BRCA	48	FEMALE	WHITE	Stage IIB	Infiltrating Ductal Ca	1995		-17715	Dead	TUMOR FREE		1759						1	1759	0	1759			0	1759
TCGA-BH	TCGA-BH	BRCA	77	FEMALE	BLACK OI	Stage I	Medullary Carcinoma	1996	Post (prio	-28403	Dead	TUMOR FREE		3959						1	3959	0	3959			0	3959
TCGA-BH	TCGA-BH	BRCA	77	FEMALE	BLACK OI	Stage I	Medullary Carcinoma	1996	Post (prio	-28403	Dead	TUMOR FREE		3959						1	3959	0	3959			0	3959
TCGA-BH	TCGA-BH	BRCA	50	FEMALE	WHITE	Stage IIIC	Infiltrating Lobular C	2010		-18501	Alive	TUMOR F	1120						Negative	0	1120	0	1120	0	1120	0	1120
TCGA-BH	TCGA-BH	BRCA	46	FEMALE	WHITE	Stage IIB	Infiltrating Lobular C	2010	Pre (<6 m	-16882	Alive	TUMOR F	1119							0	1119	0	1119			0	1119
TCGA-BH	TCGA-BH	BRCA	45	FEMALE	WHITE	Stage IIA	Infiltrating Lobular C	2011	Pre (<6 m	-16658	Alive	TUMOR F	612						Negative	0	612	0	612	0	612	0	612
TCGA-BH	TCGA-BH	BRCA	75	FEMALE	BLACK OI	Stage IIA	Infiltrating Lobular C	2012	Post (prio	-27731	Dead	TUMOR FREE		320					Negative	1	320	0	320	0	320	0	320
TCGA-BH	TCGA-BH	BRCA	80	FEMALE	BLACK OI	Stage IIA	Infiltrating Lobular C	2004	Post (prio	-29330	Alive	TUMOR F	3364						Negative	0	3364	0	3364	0	3364	0	3364
TCGA-BH	TCGA-BH	BRCA	41	FEMALE	BLACK OI	Stage IB	Infiltrating Ductal Ca	2012	Pre (<6 m	-15158	Alive	TUMOR F	635						Negative	0	635	0	635	0	635	0	635
TCGA-BH	TCGA-BH	BRCA	51	FEMALE	BLACK OI	Stage IIB	Infiltrating Ductal Ca	2012	Post (prio	-18927	Alive	TUMOR F	567						Negative	0	567	0	567	0	567	0	567
TCGA-BH	TCGA-BH	BRCA	63	FEMALE	BLACK OI	Stage IA	Infiltrating Ductal Ca	2012	Post (prio	-23072	Alive	TUMOR F	715						Negative	0	715	0	715	0	715	0	715
TCGA-BH	TCGA-BH	BRCA	46	FEMALE	BLACK OI	Stage IIA	Infiltrating Ductal Ca	2012	[Unknowr	-16829	Alive	TUMOR F	293						Negative	0	293	0	293	0	293	0	293
TCGA-BH	TCGA-BH	BRCA	61	FEMALE	WHITE	Stage IIA	Metaplastic Carcinor	2012	Post (prio	-22325	Alive	TUMOR F	160						Negative	0	160	0	160	0	160	0	160
TCGA-BH	TCGA-BH	BRCA	87	FEMALE	WHITE	Stage IA	Infiltrating Lobular C	2012	Post (prio	-31932	Dead	TUMOR FREE		295					Negative	1	295	0	295	0	295	0	295
TCGA-BH	TCGA-BH	BRCA	58	FEMALE	WHITE	Stage IA	Infiltrating Lobular C	2012	Post (prio	-21343	Alive	TUMOR F	574						Negative	0	574	0	574	0	574	0	574
TCGA-BH	TCGA-BH	BRCA	54	FEMALE	WHITE	Stage IIB	Infiltrating Lobular C	2012	Peri (6-12	-19975	Alive	TUMOR F	662						Negative	0	662	0	662	0	662	0	662
TCGA-BH	TCGA-BH	BRCA	53	FEMALE	WHITE	Stage IIIA	Infiltrating Lobular C	2013	[Unknowr	-19570	Alive	TUMOR F	287						Negative	0	287	0	287	0	287	0	287
TCGA-C8	TCGA-C8	BRCA	80	FEMALE	ASIAN	Stage IIB	Infiltrating Ductal Ca	2010	Post (prio	-29463	Alive	TUMOR F	0						Negative	0	0	0	0	0	0	0	0
TCGA-C8	TCGA-C8	BRCA	67	FEMALE	ASIAN	Stage IIA	Infiltrating Ductal Ca	2010	Post (prio	-24710	Alive	TUMOR F	363						Negative	0	363	0	363	0	363	0	363
TCGA-C8	TCGA-C8	BRCA	70	FEMALE	ASIAN	Stage IIA	Infiltrating Ductal Ca	2010	Post (prio	-25811	Alive	TUMOR F	358						Negative	0	358	0	358	0	358	0	358
TCGA-C8	TCGA-C8	BRCA	58	FEMALE	ASIAN	Stage IIA	Infiltrating Ductal Ca	2010	Post (prio	-21428	Alive	TUMOR F	358						Negative	0	358	0	358	0	358	0	358
TCGA-C8	TCGA-C8	BRCA	50	FEMALE	ASIAN	Stage IIA	Infiltrating Ductal Ca	2010	Pre (<6 m	-18506	Alive	TUMOR F	385						Negative	0	385	0	385	0	385	0	385
TCGA-C8	TCGA-C8	BRCA	55	FEMALE	ASIAN	Stage IIB	Infiltrating Ductal Ca	2010	Post (prio	-20332	Alive	TUMOR F	358						Negative	0	358	0	358	0	358	0	358
TCGA-C8	TCGA-C8	BRCA	78	FEMALE	ASIAN	Stage IIIA	Infiltrating Ductal Ca	2010	Post (prio	-28733	Dead	WITH TUMOR		385					Negative	1	385	1	385			1	385
TCGA-C8	TCGA-C8	BRCA	43	FEMALE	ASIAN	Stage IIA	Infiltrating Ductal Ca	2010	Pre (<6 months sinc		Alive	TUMOR F	0						Negative	0	0	0	0	0	0	0	0
TCGA-C8	TCGA-C8	BRCA	46	FEMALE	ASIAN	Stage IIB	Infiltrating Ductal Ca	2010	Pre (<6 m	-17045	Alive	TUMOR F	385						Negative	0	385	0	385	0	385	0	385
TCGA-C8	TCGA-C8	BRCA	55	FEMALE	ASIAN	Stage IIA	Infiltrating Ductal Ca	2010	Post (prio	-20346	Alive	TUMOR F	385							0	385	0	385			0	385
TCGA-C8	TCGA-C8	BRCA	49	FEMALE	ASIAN	Stage IIIB	Infiltrating Ductal Ca	2010	Pre (<6 m	-18140	Alive	TUMOR F	385						Negative	0	385	0	385	0	385	0	385
TCGA-C8	TCGA-C8	BRCA	62	FEMALE	ASIAN	Stage IIB	Mucinous Carcinoma	2010	Post (prio	-22889	Alive	TUMOR F	385						Negative	0	385	0	385	0	385	0	385
TCGA-C8	TCGA-C8	BRCA	44	FEMALE	ASIAN	Stage IIB	Infiltrating Ductal Ca	2010	Pre (<6 months sinc		Alive	TUMOR F	1476						Negative	0	1476	0	1476	0	1476	0	1476
TCGA-C8	TCGA-C8	BRCA	45	FEMALE	ASIAN	Stage IIB	Infiltrating Ductal Ca	2010	Peri (6-12	-16508	Alive	TUMOR F	382						Negative	0	382	0	382	0	382	0	382
TCGA-C8	TCGA-C8	BRCA	52	FEMALE	ASIAN	Stage IIIA	Infiltrating Ductal Ca	2010	Peri (6-12	-19117	Alive	TUMOR F	370						Negative	0	370	0	370	0	370	0	370
TCGA-C8	TCGA-C8	BRCA	82	FEMALE	ASIAN	Stage IIIA	Infiltrating Ductal Ca	2010	Post (prio	-29966	Alive	TUMOR F	411						Negative	0	411	0	411	0	411	0	411
TCGA-C8	TCGA-C8	BRCA	56	FEMALE	ASIAN	Stage IIB	Infiltrating Ductal Ca	2010	Post (prio	-20471	Alive	TUMOR F	383						Negative	0	383	0	383	0	383	0	383
TCGA-C8	TCGA-C8	BRCA	65	FEMALE	WHITE	Stage IIIA	Other, specify	2010	Post (prio	-23922	Alive	TUMOR F	0						Negative	0	0	0	0	0	0	0	0
TCGA-C8	TCGA-C8	BRCA	52	FEMALE	ASIAN	Stage IIA	Infiltrating Ductal Ca	2010	Peri (6-12	-19010	Alive	TUMOR F	383						Negative	0	383	0	383	0	383	0	383
TCGA-C8	TCGA-C8	BRCA	64	FEMALE	ASIAN	Stage IIB	Infiltrating Ductal Ca	2010	Post (prio	-23667	Alive	TUMOR F	393						Negative	0	393	0	393	0	393	0	393
TCGA-C8	TCGA-C8	BRCA	34	FEMALE	ASIAN	Stage IIB	Infiltrating Ductal Ca	2010	Pre (<6 m	-12729	Alive	WITH TUI	379		Locoregional Recurrence			136	Negative	0	379	0	379	1	136	1	136
TCGA-C8	TCGA-C8	BRCA	54	FEMALE	ASIAN	Stage IIIA	Infiltrating Ductal Ca	2010	Post (prio	-20053	Alive	TUMOR F	380						Negative	0	380	0	380	0	380	0	380
TCGA-C8	TCGA-C8	BRCA	59	FEMALE	ASIAN	Stage IIA	Infiltrating Ductal Ca	2010	Post (prior bilateral c		Alive	TUMOR F	375						Negative	0	375	0	375	0	375	0	375
TCGA-C8	TCGA-C8	BRCA	48	FEMALE	ASIAN	Stage IIA	Infiltrating Ductal Ca	2010	Pre (<6 months sinc		Alive	TUMOR F	332						Negative	0	332	0	332	0	332	0	332
TCGA-C8	TCGA-C8	BRCA	50	FEMALE	ASIAN	Stage IIA	Infiltrating Ductal Ca	2010	Pre (<6 months sinc		Alive	TUMOR F	345						Negative	0	345	0	345	0	345	0	345
TCGA-C8	TCGA-C8	BRCA	40	FEMALE	ASIAN	Stage IIIA	Infiltrating Ductal Ca	2010	Pre (<6 months sinc		Alive	TUMOR F	343						Negative	0	343	0	343	0	343	0	343
TCGA-C8	TCGA-C8	BRCA	53	FEMALE	ASIAN	Stage IIA	Infiltrating Ductal Ca	2010	Peri (6-12	-19629	Alive	TUMOR F	5						Negative	0	5	0	5	0	5	0	5
TCGA-C8	TCGA-C8	BRCA	53	FEMALE	ASIAN	Stage IIB	Infiltrating Ductal Ca	2010	Peri (6-12 months si		Alive	TUMOR F	366						Negative	0	366	0	366	0	366	0	366
TCGA-C8	TCGA-C8	BRCA	38	FEMALE	ASIAN	Stage IIIA	Other, specify	2010	Pre (<6 months sinc		Alive	TUMOR F	317						Negative	0	317	0	317	0	317	0	317
TCGA-C8	TCGA-C8	BRCA	74	FEMALE	ASIAN	Stage IIA	Infiltrating Ductal Ca	2010	Post (prio	-27063	Alive	TUMOR F	375						Negative	0	375	0	375	0	375	0	375
TCGA-C8	TCGA-C8	BRCA	56	FEMALE	ASIAN	Stage IIA	Infiltrating Ductal Ca	2010	Post (prio	-20712	Alive	TUMOR F	394						Negative	0	394	0	394	0	394	0	394
TCGA-C8	TCGA-C8	BRCA	34	FEMALE	ASIAN	Stage IIIC	Infiltrating Ductal Ca	2010	Pre (<6 m	-12715	Alive	TUMOR F	375						Negative	0	375	0	375	0	375	0	375
TCGA-C8	TCGA-C8	BRCA	47	FEMALE	ASIAN	Stage IIIA	Infiltrating Ductal Ca	2010	Peri (6-12	-17468	Alive	TUMOR F	616						Negative	0	616	0	616	0	616	0	616
TCGA-C8	TCGA-C8	BRCA	58	FEMALE	ASIAN	Stage IIB	Infiltrating Ductal Ca	2010	Post (prio	-21391	Alive	TUMOR F	381						Negative	0	381	0	381	0	381	0	381
TCGA-C8	TCGA-C8	BRCA	58	FEMALE	ASIAN	Stage IIA	Infiltrating Ductal Ca	2010	Post (prio	-21463	Alive	TUMOR F	376						Negative	0	376	0	376	0	376	0	376
TCGA-C8	TCGA-C8	BRCA	90	FEMALE	ASIAN	Stage IIA	Infiltrating Ductal Ca	2010	Post (prio	-32872	Alive	TUMOR F	394						Negative	0	394	0	394	0	394	0	394
TCGA-C8	TCGA-C8	BRCA	59	FEMALE	ASIAN	Stage IIA	Infiltrating Ductal Ca	2010	Post (prior bilateral c		Alive	TUMOR F	470						Negative	0	470	0	470	0	470	0	470
TCGA-C8	TCGA-C8	BRCA	29	FEMALE	ASIAN	Stage IIB	Infiltrating Ductal Ca	2010	Pre (<6 months sinc		Alive	TUMOR F	513						Negative	0	513	0	513	0	513	0	513
TCGA-C8	TCGA-C8	BRCA	63	FEMALE	ASIAN	Stage IIA	Infiltrating Ductal Ca	2010	Post (prio	-23337	Alive	TUMOR F	508						Negative	0	508	0	508	0	508	0	508
TCGA-C8	TCGA-C8	BRCA	56	FEMALE	ASIAN	Stage IIA	Infiltrating Ductal Ca	2010	Post (prio	-20774	Alive	TUMOR F	1						Negative	0	1	0	1	0	1	0	1
TCGA-C8	TCGA-C8	BRCA	61	FEMALE	ASIAN	Stage IIIA	Infiltrating Ductal Ca	2010	Post (prio	-22598	Alive	TUMOR F	297						Negative	0	297	0	297	0	297	0	297
TCGA-C8	TCGA-C8	BRCA	48	FEMALE	ASIAN	Stage IIB	Infiltrating Ductal Ca	2010	Peri (6-12	-17779	Alive	TUMOR F	747						Negative	0	747	0	747	0	747	0	747
TCGA-C8	TCGA-C8	BRCA	48	FEMALE	ASIAN	Stage IIB	Infiltrating Ductal Ca	2011	Peri (6-12	-17763	Alive	TUMOR F	439						Negative	0	439	0	439	0	439	0	439
TCGA-C8	TCGA-C8	BRCA	60	FEMALE	ASIAN	Stage IIIB	Infiltrating Lobular C	2010	Post (prio	-22096	Dead	WITH TUMOR		1034	Locoregional Recurrence			1034	Negative	1	1034	1	1034	1	1034	1	1034
TCGA-C8	TCGA-C8	BRCA	68	FEMALE	ASIAN	Stage IA	Infiltrating Lobular C	2011	Post (prio	-24877	Alive	TUMOR F	394						Negative	0	394	0	394	0	394	0	394
TCGA-C8	TCGA-C8	BRCA	59	FEMALE	ASIAN	Stage IIIA	Metaplastic Carcinor	2013	Post (prio	-21613	Alive	TUMOR F	396						Negative	0	396	0	396	0	396	0	396
TCGA-C8	TCGA-C8	BRCA	53	FEMALE	ASIAN	Stage IIB	Mucinous Carcinoma	2013	Post (prio	-19432	Alive	TUMOR F	380						Negative	0	380	0	380	0	380	0	380
TCGA-C8	TCGA-C8	BRCA	49	FEMALE	ASIAN	Stage IIIA	Infiltrating Lobular C	2013	Peri (6-12	-17921	Alive	TUMOR F	408						Negative	0	408	0	408	0	408	0	408
TCGA-C8	TCGA-C8	BRCA	49	FEMALE	WHITE	Stage IIB	Mixed Histology (ple	2013	Pre (<6 m	-18038	Alive	TUMOR F	592						Negative	0	592	0	592	0	592	0	592
TCGA-D8	TCGA-D8	BRCA	52	FEMALE	WHITE	Stage IA	Infiltrating Ductal Ca	2010	Post (prio	-19028	Alive	TUMOR F	1728						Negative	0	1728	0	1728	0	1728	0	1728
TCGA-D8	TCGA-D8	BRCA	51	FEMALE	WHITE		Infiltrating Ductal Ca	2010	Post (prio	-18942	Alive	TUMOR F	635						Negative	0	635	0	635	0	635	0	635
TCGA-D8	TCGA-D8	BRCA	62	FEMALE	WHITE	Stage IIB	Infiltrating Ductal Ca	2010	Post (prio	-22900	Alive	TUMOR F	403						Negative	0	403	0	403	0	403	0	403
TCGA-D8	TCGA-D8	BRCA	40	FEMALE	WHITE		Infiltrating Ductal Ca	2010	Indetermi	-14626	Alive	TUMOR F	626						Negative	0	626	0	626	0	626	0	626
TCGA-D8	TCGA-D8	BRCA	74	FEMALE	WHITE	Stage IIB	Infiltrating Ductal Ca	2010	Post (prio	-27313	Alive	TUMOR F	425						Negative	0	425	0	425	0	425	0	425
TCGA-D8	TCGA-D8	BRCA	51	FEMALE	WHITE	Stage IIA	Infiltrating Ductal Ca	2010	Post (prio	-18984	Alive	TUMOR F	431						Negative	0	431	0	431	0	431	0	431
TCGA-D8	TCGA-D8	BRCA	80	FEMALE	WHITE	Stage IIA	Infiltrating Ductal Ca	2010	Post (prio	-29280	Alive	TUMOR F	410						Negative	0	410	0	410	0	410	0	410
TCGA-D8	TCGA-D8	BRCA	57	FEMALE	WHITE	Stage IIA	Infiltrating Ductal Ca	2010	Post (prio	-20861	Alive	TUMOR F	643						Negative	0	643	0	643	0	643	0	643
TCGA-D8	TCGA-D8	BRCA	45	FEMALE	WHITE		Infiltrating Ductal Ca	2010	Indetermi	-16736	Alive	TUMOR F	584						Negative	0	584	0	584	0	584	0	584
TCGA-D8	TCGA-D8	BRCA	77	FEMALE	WHITE	Stage IIB	Infiltrating Ductal Ca	2010	Post (prio	-28444	Alive	TUMOR F	431						Negative	0	431	0	431	0	431	0	431
TCGA-D8	TCGA-D8	BRCA	48	FEMALE	WHITE	Stage IA	Infiltrating Ductal Ca	2010	Post (prio	-17879	Alive	TUMOR F	532						Negative	0	532	0	532	0	532	0	532
TCGA-D8	TCGA-D8	BRCA	60	FEMALE	WHITE		Infiltrating Ductal Ca	2010	Post (prio	-21918	Alive	WITH TUI	502		Locoregional Disease			431	Negative	0	502	0	502	1	431	1	431
TCGA-D8	TCGA-D8	BRCA	54	FEMALE	WHITE	Stage IIB	Infiltrating Ductal Ca	2010	Post (prio	-19822	Alive	TUMOR F	1688						Negative	0	1688	0	1688	0	1688	0	1688
TCGA-D8	TCGA-D8	BRCA	59	FEMALE	WHITE	Stage IIIA	Infiltrating Ductal Ca	2010	Post (prio	-21898	Alive	TUMOR F	480						Negative	0	480	0	480	0	480	0	480
TCGA-D8	TCGA-D8	BRCA	41	FEMALE	WHITE	Stage IIB	Infiltrating Ductal Ca	2010	Pre (<6 m	-15166	Alive	TUMOR F	552						Negative	0	552	0	552	0	552	0	552
TCGA-D8	TCGA-D8	BRCA	62	FEMALE	WHITE	Stage IIA	Infiltrating Ductal Ca	2010	Post (prio	-22683	Alive	TUMOR F	575						Negative	0	575	0	575	0	575	0	575
TCGA-D8	TCGA-D8	BRCA	79	FEMALE	WHITE	Stage IIIA	Infiltrating Ductal Ca	2010	Post (prio	-29199	Alive	TUMOR F	366						Negative	0	366	0	366	0	366	0	366
TCGA-D8	TCGA-D8	BRCA	62	FEMALE	WHITE	Stage IIA	Infiltrating Ductal Ca	2010	Post (prio	-22878	Alive	TUMOR F	1612						Negative	0	1612	0	1612	0	1612	0	1612
TCGA-D8	TCGA-D8	BRCA	56	FEMALE	WHITE	Stage IA	Infiltrating Ductal Ca	2010	Post (prio	-20497	Alive	TUMOR F	426						Negative	0	426	0	426	0	426	0	426
TCGA-D8	TCGA-D8	BRCA	54	FEMALE	WHITE	Stage IIA	Infiltrating Ductal Ca	2010	Post (prio	-20072	Alive	TUMOR F	577						Negative	0	577	0	577	0	577	0	577
TCGA-D8	TCGA-D8	BRCA	54	FEMALE	WHITE	Stage IIA	Infiltrating Ductal Ca	2010	Post (prio	-19966	Alive	TUMOR F	611						Negative	0	611	0	611	0	611	0	611
TCGA-D8	TCGA-D8	BRCA	90	FEMALE	WHITE	Stage IIA	Infiltrating Ductal Ca	2010	Post (prio	-32872	Alive	TUMOR F	612						Negative	0	612	0	612	0	612	0	612
TCGA-D8	TCGA-D8	BRCA	72	FEMALE	WHITE	Stage IIA	Infiltrating Ductal Ca	2010	Post (prio	-26484	Alive	TUMOR F	611						Negative	0	611	0	611	0	611	0	611
TCGA-D8	TCGA-D8	BRCA	59	FEMALE	WHITE	Stage IIB	Infiltrating Ductal Ca	2010	Post (prio	-21602	Alive	TUMOR F	590						Negative	0	590	0	590	0	590	0	590
TCGA-D8	TCGA-D8	BRCA	80	FEMALE	WHITE	Stage IIIC	Infiltrating Lobular C	2010	Post (prio	-29331	Alive	TUMOR F	620						Negative	0	620	0	620	0	620	0	620
TCGA-D8	TCGA-D8	BRCA	73	FEMALE	WHITE	Stage IA	Infiltrating Ductal Ca	2010	Post (prio	-26862	Alive	TUMOR F	639						Negative	0	639	0	639	0	639	0	639
TCGA-D8	TCGA-D8	BRCA	77	FEMALE	WHITE	Stage IA	Other, specify	2010	Post (prio	-28178	Alive	TUMOR F	371						Negative	0	371	0	371	0	371	0	371
TCGA-D8	TCGA-D8	BRCA	70	FEMALE	WHITE	Stage IIA	Other, specify	2010	Post (prio	-25677	Alive	TUMOR F	405						Negative	0	405	0	405	0	405	0	405
TCGA-D8	TCGA-D8	BRCA	51	FEMALE	WHITE	Stage IA	Infiltrating Ductal Ca	2010	Post (prio	-18733	Alive	TUMOR F	447						Negative	0	447	0	447	0	447	0	447
TCGA-D8	TCGA-D8	BRCA	81	FEMALE	WHITE	Stage IIIC	Infiltrating Ductal Ca	2010	Post (prio	-29734	Alive	TUMOR F	565						Negative	0	565	0	565	0	565	0	565
TCGA-D8	TCGA-D8	BRCA	80	FEMALE	WHITE	Stage IIIA	Infiltrating Ductal Ca	2010	Post (prio	-29256	Alive	TUMOR F	541						Negative	0	541	0	541	0	541	0	541
TCGA-D8	TCGA-D8	BRCA	40	FEMALE	WHITE	Stage IIA	Other, specify	2010	Pre (<6 m	-14858	Alive	TUMOR F	509						Negative	0	509	0	509	0	509	0	509
TCGA-D8	TCGA-D8	BRCA	62	FEMALE	WHITE	Stage IIIA	Infiltrating Lobular C	2010	Post (prio	-22995	Alive	TUMOR F	783						Negative	0	783	0	783	0	783	0	783
TCGA-D8	TCGA-D8	BRCA	66	FEMALE	WHITE	Stage IIB	Infiltrating Ductal Ca	2010	Post (prio	-24417	Alive	TUMOR F	727							0	727	0	727			0	727
TCGA-D8	TCGA-D8	BRCA	64	FEMALE	WHITE	Stage IA	Infiltrating Ductal Ca	2010	Post (prio	-23387	Alive	TUMOR F	839						Negative	0	839	0	839	0	839	0	839
TCGA-D8	TCGA-D8	BRCA	62	FEMALE	WHITE	Stage IIB	Infiltrating Ductal Ca	2010	Post (prio	-22976	Alive	TUMOR F	552						Negative	0	552	0	552	0	552	0	552
TCGA-D8	TCGA-D8	BRCA	85	FEMALE	WHITE	Stage IIIB	Mucinous Carcinoma	2010	Post (prio	-31220	Dead	WITH TUMOR		377					Negative	1	377	1	377			1	377
TCGA-D8	TCGA-D8	BRCA	36	FEMALE	WHITE	Stage IIIA	Infiltrating Ductal Ca	2010	Pre (<6 m	-13510	Alive	TUMOR F	522						Negative	0	522	0	522	0	522	0	522
TCGA-D8	TCGA-D8	BRCA	45	FEMALE	WHITE	Stage IIA	Infiltrating Ductal Ca	2010	Pre (<6 m	-16787	Alive	TUMOR F	463							0	463	0	463			0	463
TCGA-D8	TCGA-D8	BRCA	86	FEMALE	WHITE	Stage IIIB	Infiltrating Ductal Ca	2010	Post (prio	-31748	Alive	TUMOR F	448						Negative	0	448	0	448	0	448	0	448
TCGA-D8	TCGA-D8	BRCA	76	FEMALE	WHITE	Stage IIIA	Other, specify	2010	Post (prio	-27967	Alive	TUMOR F	664						Negative	0	664	0	664	0	664	0	664
TCGA-D8	TCGA-D8	BRCA	55	FEMALE	WHITE	Stage IIB	Infiltrating Ductal Ca	2010	Post (prio	-20092	Alive	TUMOR F	441						Negative	0	441	0	441	0	441	0	441
TCGA-D8	TCGA-D8	BRCA	34	FEMALE	WHITE	Stage IIB	Infiltrating Ductal Ca	2010	Pre (<6 m	-12668	Alive	TUMOR F	606						Negative	0	606	0	606	0	606	0	606
TCGA-D8	TCGA-D8	BRCA	57	FEMALE	WHITE	Stage IA	Infiltrating Ductal Ca	2010	Post (prio	-21056	Alive	TUMOR F	538						Negative	0	538	0	538	0	538	0	538
TCGA-D8	TCGA-D8	BRCA	56	FEMALE	WHITE	Stage IIB	Infiltrating Lobular C	2010	Post (prio	-20606	Alive	TUMOR F	1682						Negative	0	1682	0	1682	0	1682	0	1682
TCGA-D8	TCGA-D8	BRCA	69	FEMALE	WHITE	Stage IIA	Infiltrating Ductal Ca	2010	Post (prio	-25345	Alive	TUMOR F	499						Negative	0	499	0	499	0	499	0	499
TCGA-D8	TCGA-D8	BRCA	56	FEMALE	WHITE	Stage IIB	Infiltrating Ductal Ca	2010	Post (prio	-20488	Alive	TUMOR F	482						Negative	0	482	0	482	0	482	0	482
TCGA-D8	TCGA-D8	BRCA	48	MALE	WHITE	Stage IIIC	Other, specify	2010		-17708	Alive	TUMOR F	496						Negative	0	496	0	496	0	496	0	496
TCGA-D8	TCGA-D8	BRCA	61	FEMALE	WHITE	Stage IIA	Infiltrating Ductal Ca	2010	Post (prio	-22448	Alive	TUMOR F	506						Negative	0	506	0	506	0	506	0	506
TCGA-D8	TCGA-D8	BRCA	56	FEMALE	WHITE	Stage IA	Infiltrating Ductal Ca	2010	Post (prio	-20715	Alive	TUMOR F	395						Negative	0	395	0	395	0	395	0	395
TCGA-D8	TCGA-D8	BRCA	84	FEMALE	WHITE	Stage IIA	Mucinous Carcinoma	2010	Post (prio	-30830	Alive	TUMOR F	461						Negative	0	461	0	461	0	461	0	461
TCGA-D8	TCGA-D8	BRCA	53	FEMALE	WHITE	Stage IIA	Infiltrating Ductal Ca	2011	Post (prio	-19478	Alive	TUMOR F	1309						Negative	0	1309	0	1309	0	1309	0	1309
TCGA-D8	TCGA-D8	BRCA	74	FEMALE	WHITE	Stage IIA	Infiltrating Ductal Ca	2010	Post (prio	-27106	Alive	TUMOR F	503							0	503	0	503			0	503
TCGA-D8	TCGA-D8	BRCA	81	FEMALE	WHITE	Stage IIIA	Infiltrating Ductal Ca	2010	Post (prio	-29916	Alive	TUMOR F	466						Negative	0	466	0	466	0	466	0	466
TCGA-D8	TCGA-D8	BRCA	65	FEMALE	WHITE	Stage IIIA	Infiltrating Ductal Ca	2010	Post (prio	-24083	Alive	TUMOR F	472							0	472	0	472			0	472
TCGA-D8	TCGA-D8	BRCA	80	FEMALE	WHITE	Stage IIIA	Infiltrating Ductal Ca	2010	Post (prio	-29474	Dead	WITH TUMOR		302						1	302	1	302			1	302
TCGA-D8	TCGA-D8	BRCA	71	FEMALE	WHITE	Stage IIA	Infiltrating Ductal Ca	2011	Post (prio	-26021	Alive	TUMOR F	433						Negative	0	433	0	433	0	433	0	433
TCGA-D8	TCGA-D8	BRCA	61	FEMALE	WHITE	Stage IIIA	Infiltrating Ductal Ca	2011	Post (prio	-22317	Alive	TUMOR F	430						Negative	0	430	0	430	0	430	0	430
TCGA-D8	TCGA-D8	BRCA	66	FEMALE	WHITE	Stage IA	Other, specify	2010	Post (prio	-24315	Alive	TUMOR F	530						Negative	0	530	0	530	0	530	0	530
TCGA-D8	TCGA-D8	BRCA	40	FEMALE	WHITE	Stage IIA	Infiltrating Ductal Ca	2010	Pre (<6 m	-14731	Alive	TUMOR F	488						Negative	0	488	0	488	0	488	0	488
TCGA-D8	TCGA-D8	BRCA	75	FEMALE	WHITE	Stage IIA	Infiltrating Lobular C	2010	Post (prio	-27521	Alive	TUMOR F	409						Negative	0	409	0	409	0	409	0	409
TCGA-D8	TCGA-D8	BRCA	72	FEMALE	WHITE	Stage IIA	Infiltrating Ductal Ca	2011	Post (prio	-26588	Alive	TUMOR F	397						Negative	0	397	0	397	0	397	0	397
TCGA-D8	TCGA-D8	BRCA	58	FEMALE	WHITE	Stage IIIA	Infiltrating Lobular C	2010	Post (prio	-21327	Alive	TUMOR F	439						Negative	0	439	0	439	0	439	0	439
TCGA-D8	TCGA-D8	BRCA	47	FEMALE	WHITE	Stage IIB	Infiltrating Ductal Ca	2011	Post (prio	-17426	Alive	TUMOR F	1461						Negative	0	1461	0	1461	0	1461	0	1461
TCGA-D8	TCGA-D8	BRCA	49	FEMALE	WHITE	Stage IIIA	Infiltrating Ductal Ca	2011	Post (prio	-18025	Alive	TUMOR F	499						Negative	0	499	0	499	0	499	0	499
TCGA-D8	TCGA-D8	BRCA	59	FEMALE	WHITE	Stage IA	Infiltrating Ductal Ca	2011	Post (prio	-21910	Alive	TUMOR F	410						Negative	0	410	0	410	0	410	0	410
TCGA-D8	TCGA-D8	BRCA	36	FEMALE	WHITE	Stage IIIA	Infiltrating Ductal Ca	2011	Pre (<6 m	-13243	Alive	TUMOR F	519						Negative	0	519	0	519	0	519	0	519
TCGA-D8	TCGA-D8	BRCA	64	FEMALE	WHITE	Stage IA	Infiltrating Ductal Ca	2011	Post (prio	-23576	Alive	TUMOR F	49						Negative	0	49	0	49	0	49	0	49
TCGA-D8	TCGA-D8	BRCA	41	FEMALE	WHITE	Stage IIIC	Infiltrating Ductal Ca	2011	Post (prio	-15122	Alive	TUMOR F	307						Negative	0	307	0	307	0	307	0	307
TCGA-D8	TCGA-D8	BRCA	53	FEMALE	WHITE	Stage IIIC	Infiltrating Lobular C	2011	Post (prio	-19591	Alive	TUMOR F	398						Negative	0	398	0	398	0	398	0	398
TCGA-D8	TCGA-D8	BRCA	62	FEMALE	WHITE	Stage IIA	Infiltrating Lobular C	2011	Post (prio	-22958	Alive	TUMOR F	381						Negative	0	381	0	381	0	381	0	381
TCGA-D8	TCGA-D8	BRCA	55	FEMALE	WHITE	Stage IIIB	Mucinous Carcinoma	2011	Post (prio	-20173	Alive	TUMOR F	373						Negative	0	373	0	373	0	373	0	373
TCGA-D8	TCGA-D8	BRCA	54	FEMALE	WHITE	Stage IIIC	Infiltrating Lobular C	2011	Post (prio	-19846	Alive	TUMOR F	1015						Negative	0	1015	0	1015	0	1015	0	1015
TCGA-D8	TCGA-D8	BRCA	56	FEMALE	WHITE	Stage IIIC	Infiltrating Lobular C	2011	Post (prio	-20629	Alive	TUMOR F	563						Negative	0	563	0	563	0	563	0	563
TCGA-D8	TCGA-D8	BRCA	68	FEMALE	WHITE	Stage IA	Infiltrating Lobular C	2012	Post (prio	-25097	Alive	TUMOR F	659						Negative	0	659	0	659	0	659	0	659
TCGA-D8	TCGA-D8	BRCA	88	FEMALE	WHITE	Stage IIA	Infiltrating Lobular C	2012	Post (prio	-32208	Alive	WITH TUI	492						Negative	0	492	0	492	0	492	0	492
TCGA-D8	TCGA-D8	BRCA	79	FEMALE	WHITE	Stage IIIA	Mucinous Carcinoma	2013	Post (prio	-29125	Dead	WITH TUMOR		385	Distant M	Lung		21	Negative	1	385	1	385	1	21	1	21
TCGA-D8	TCGA-D8	BRCA	53	FEMALE	WHITE	Stage IIA	Other, specify	2013	Post (prio	-19607	Alive	TUMOR F	767						Negative	0	767	0	767	0	767	0	767
TCGA-E2-	TCGA-E2-	BRCA	79	FEMALE	WHITE	Stage IIA	Infiltrating Ductal Ca	2007	Post (prio	-28855	Alive	TUMOR F	1308						Negative	0	1308	0	1308	0	1308	0	1308
TCGA-E2-	TCGA-E2-	BRCA	34	FEMALE	ASIAN	Stage IB	Infiltrating Ductal Ca	2006	Pre (<6 m	-12528	Alive	TUMOR F	2541						Negative	0	2541	0	2541	0	2541	0	2541
TCGA-E2-	TCGA-E2-	BRCA	54	FEMALE	WHITE	Stage IIIA	Infiltrating Lobular C	2008	Post (prio	-20065	Alive	TUMOR F	1047						Negative	0	1047	0	1047	0	1047	0	1047
TCGA-E2-	TCGA-E2-	BRCA	64	FEMALE	WHITE	Stage IIIA	Infiltrating Ductal Ca	2009	Post (prio	-23530	Alive	WITH TUI	837						Negative	0	837	0	837	0	837	0	837
TCGA-E2-	TCGA-E2-	BRCA	64	FEMALE	WHITE	Stage IIA	Infiltrating Ductal Ca	2007	Post (prio	-23521	Alive	TUMOR F	1417						Negative	0	1417	0	1417	0	1417	0	1417
TCGA-E2-	TCGA-E2-	BRCA	41	FEMALE	WHITE	Stage IIB	Infiltrating Ductal Ca	2007	Pre (<6 m	-15085	Alive	WITH TUI	1229		Distant M	Bone		75	Negative	0	1229	0	1229	1	75	1	75
TCGA-E2-	TCGA-E2-	BRCA	67	FEMALE	WHITE	Stage IIB	Infiltrating Ductal Ca	2007	Post (prio	-24577	Alive	TUMOR F	1141		New Prim	Lung		195	Close	0	1141	0	1141			1	195
TCGA-E2-	TCGA-E2-	BRCA	54	FEMALE	WHITE	Stage IIB	Infiltrating Ductal Ca	2008	Post (prio	-19849	Alive	TUMOR F	1220						Negative	0	1220	0	1220	0	1220	0	1220
TCGA-E2-	TCGA-E2-	BRCA	64	FEMALE	WHITE	Stage IIA	Infiltrating Ductal Ca	2008	Post (prio	-23684	Alive	TUMOR F	865						Negative	0	865	0	865	0	865	0	865
TCGA-E2-	TCGA-E2-	BRCA	47	FEMALE	WHITE	Stage IIA	Infiltrating Lobular C	2008	Pre (<6 m	-17257	Alive	TUMOR F	878						Negative	0	878	0	878	0	878	0	878
TCGA-E2-	TCGA-E2-	BRCA	37	FEMALE	WHITE	Stage IIB	Infiltrating Ductal Ca	2007	Pre (<6 m	-13743	Alive	TUMOR F	1434						Negative	0	1434	0	1434	0	1434	0	1434
TCGA-E2-	TCGA-E2-	BRCA	76	FEMALE	WHITE	Stage IIIA	Infiltrating Ductal Ca	2008	Post (prio	-27837	Alive	TUMOR F	1359						Negative	0	1359	0	1359	0	1359	0	1359
TCGA-E2-	TCGA-E2-	BRCA	79	FEMALE	WHITE	Stage IIIC	Infiltrating Ductal Ca	2008	Post (prio	-28923	Alive	TUMOR F	1246						Negative	0	1246	0	1246	0	1246	0	1246
TCGA-E2-	TCGA-E2-	BRCA	50	FEMALE	WHITE	Stage IIB	Infiltrating Ductal Ca	2008	Pre (<6 m	-18507	Alive	TUMOR F	1163						Negative	0	1163	0	1163	0	1163	0	1163
TCGA-E2-	TCGA-E2-	BRCA	62	FEMALE	WHITE	Stage IIA	Infiltrating Ductal Ca	2008	Post (prio	-22686	Alive	TUMOR F	1174		New Prim	Other, sp	Endometr	971	Negative	0	1174	0	1174	0	1174	1	971
TCGA-E2-	TCGA-E2-	BRCA	65	FEMALE	WHITE	Stage I	Infiltrating Ductal Ca	2008	Post (prio	-23878	Alive	TUMOR F	1009						Negative	0	1009	0	1009	0	1009	0	1009
TCGA-E2-	TCGA-E2-	BRCA	52	FEMALE	WHITE	Stage IIA	Infiltrating Ductal Ca	2008	Post (prio	-19237	Alive	TUMOR F	2311						Negative	0	2311	0	2311	0	2311	0	2311
TCGA-E2-	TCGA-E2-	BRCA	74	FEMALE	WHITE	Stage I	Infiltrating Lobular C	2008	Post (prio	-27389	Alive	TUMOR F	1318						Negative	0	1318	0	1318	0	1318	0	1318
TCGA-E2-	TCGA-E2-	BRCA	53	FEMALE	WHITE	Stage IIB	Infiltrating Ductal Ca	2008	Post (prio	-19643	Alive	TUMOR F	1042						Negative	0	1042	0	1042	0	1042	0	1042
TCGA-E2-	TCGA-E2-	BRCA	78	MALE	WHITE	Stage IIA	Infiltrating Ductal Ca	2008		-28664	Alive	TUMOR F	974						Negative	0	974	0	974	0	974	0	974
TCGA-E2-	TCGA-E2-	BRCA	55	FEMALE	BLACK OI	Stage IIIA	Infiltrating Ductal Ca	2009	Post (prio	-20105	Alive	TUMOR F	972						Negative	0	972	0	972	0	972	0	972
TCGA-E2-	TCGA-E2-	BRCA	35	FEMALE	WHITE	Stage IIA	Infiltrating Ductal Ca	2009	Pre (<6 m	-13004	Alive	TUMOR F	2109						Negative	0	2109	0	2109	0	2109	0	2109
TCGA-E2-	TCGA-E2-	BRCA	35	FEMALE	WHITE	Stage IIA	Infiltrating Ductal Ca	2009	Pre (<6 m	-13004	Alive	TUMOR F	2109						Negative	0	2109	0	2109	0	2109	0	2109

TCGA-E2-	TCGA-E2-	BRCA	64	FEMALE	WHITE	Stage I	Infiltrating Ductal Ca	2009	Post (prio	-23682	Dead	WITH TUMOR		563	Distant M	Bone		188	Negative	1	563	1	563	1	188	1	188
TCGA-E2-	TCGA-E2-	BRCA	48	FEMALE	WHITE	Stage IIA	Infiltrating Ductal Ca	2009	Post (prio	-17580	Alive	TUMOR F	1935						Negative	0	1935	0	1935	0	1935	0	1935
TCGA-E2-	TCGA-E2-	BRCA	56	FEMALE	WHITE	Stage I	Infiltrating Ductal Ca	2009	Post (prio	-20705	Alive	TUMOR F	2128		Distant M	Liver		54	Negative	0	2128	0	2128	1	54	1	54
TCGA-E2-	TCGA-E2-	BRCA	51	FEMALE	WHITE	Stage IIB	Infiltrating Ductal Ca	2009	Pre (<6 m	-18875	Alive	TUMOR F	707						Negative	0	707	0	707	0	707	0	707
TCGA-E2-	TCGA-E2-	BRCA	51	FEMALE	WHITE	Stage IIB	Infiltrating Ductal Ca	2009	Pre (<6 m	-18875	Alive	TUMOR F	707						Negative	0	707	0	707	0	707	0	707
TCGA-E2-	TCGA-E2-	BRCA	68	FEMALE	WHITE	Stage I	Infiltrating Ductal Ca	2009	Post (prio	-24999	Alive	TUMOR F	591						Negative	0	591	0	591	0	591	0	591
TCGA-E2-	TCGA-E2-	BRCA	58	FEMALE	WHITE	Stage IIB	Infiltrating Ductal Ca	2009	Post (prio	-21348	Alive	TUMOR F	640						Negative	0	640	0	640	0	640	0	640
TCGA-E2-	TCGA-E2-	BRCA	61	FEMALE	WHITE	Stage I	Infiltrating Ductal Ca	2009	Post (prio	-22422	Alive	TUMOR F	726						Negative	0	726	0	726	0	726	0	726
TCGA-E2-	TCGA-E2-	BRCA	43	FEMALE	WHITE	Stage IIA	Infiltrating Ductal Ca	2009	Pre (<6 m	-15903	Alive	TUMOR F	450						Negative	0	450	0	450	0	450	0	450
TCGA-E2-	TCGA-E2-	BRCA	43	FEMALE	WHITE	Stage IIA	Infiltrating Ductal Ca	2009	Pre (<6 m	-15903	Alive	TUMOR F	450						Negative	0	450	0	450	0	450	0	450
TCGA-E2-	TCGA-E2-	BRCA	50	FEMALE	WHITE	Stage IIA	Infiltrating Ductal Ca	2009	Pre (<6 m	-18621	Alive	TUMOR F	762						Negative	0	762	0	762	0	762	0	762
TCGA-E2-	TCGA-E2-	BRCA	45	FEMALE	WHITE	Stage IIIC	Infiltrating Ductal Ca	2009	Pre (<6 m	-16750	Alive	TUMOR F	710						Negative	0	710	0	710	0	710	0	710
TCGA-E2-	TCGA-E2-	BRCA	45	FEMALE	WHITE	Stage IIIC	Infiltrating Ductal Ca	2009	Pre (<6 m	-16750	Alive	TUMOR F	710						Negative	0	710	0	710	0	710	0	710
TCGA-E2-	TCGA-E2-	BRCA	61	FEMALE	WHITE	Stage I	Infiltrating Ductal Ca	2009	Post (prio	-22583	Alive	TUMOR F	694						Negative	0	694	0	694	0	694	0	694
TCGA-E2-	TCGA-E2-	BRCA	47	FEMALE	WHITE	Stage IIA	Infiltrating Ductal Ca	2009	Pre (<6 m	-17224	Alive	TUMOR F	526						Negative	0	526	0	526	0	526	0	526
TCGA-E2-	TCGA-E2-	BRCA	40	FEMALE	WHITE	Stage IIA	Infiltrating Ductal Ca	2009	Pre (<6 m	-14894	Alive	TUMOR F	630						Negative	0	630	0	630	0	630	0	630
TCGA-E2-	TCGA-E2-	BRCA	40	FEMALE	WHITE	Stage IIA	Infiltrating Ductal Ca	2009	Pre (<6 m	-14894	Alive	TUMOR F	630						Negative	0	630	0	630	0	630	0	630
TCGA-E2-	TCGA-E2-	BRCA	64	FEMALE	WHITE	Stage I	Infiltrating Ductal Ca	2009	Post (prio	-23660	Alive	TUMOR F	658						Negative	0	658	0	658	0	658	0	658
TCGA-E2-	TCGA-E2-	BRCA	76	FEMALE	WHITE	Stage IIA	Mixed Histology (ple	2009	Post (prio	-28013	Alive	TUMOR F	554						Negative	0	554	0	554	0	554	0	554
TCGA-E2-	TCGA-E2-	BRCA	38	FEMALE	WHITE	Stage IIA	Infiltrating Ductal Ca	2009	Pre (<6 m	-13985	Alive	TUMOR F	393						Negative	0	393	0	393	0	393	0	393
TCGA-E2-	TCGA-E2-	BRCA	44	FEMALE	WHITE	Stage IIA	Infiltrating Ductal Ca	2009	Pre (<6 m	-16163	Alive	TUMOR F	1692						Negative	0	1692	0	1692	0	1692	0	1692
TCGA-E2-	TCGA-E2-	BRCA	44	FEMALE	WHITE	Stage IIA	Infiltrating Ductal Ca	2009	Pre (<6 m	-16163	Alive	TUMOR F	1692						Negative	0	1692	0	1692	0	1692	0	1692
TCGA-E2-	TCGA-E2-	BRCA	51	FEMALE	WHITE	Stage I	Infiltrating Ductal Ca	2009	Pre (<6 m	-18771	Alive	TUMOR F	1640						Negative	0	1640	0	1640	0	1640	0	1640
TCGA-E2-	TCGA-E2-	BRCA	58	FEMALE	WHITE	Stage IIB	Infiltrating Ductal Ca	2010	Post (prio	-21514	Alive	TUMOR F	275						Negative	0	275	0	275	0	275	0	275
TCGA-E2-	TCGA-E2-	BRCA	58	FEMALE	WHITE	Stage IIB	Infiltrating Ductal Ca	2010	Post (prio	-21514	Alive	TUMOR F	275						Negative	0	275	0	275	0	275	0	275
TCGA-E2-	TCGA-E2-	BRCA	58	FEMALE	WHITE	Stage IIB	Infiltrating Ductal Ca	2010	Post (prio	-21514	Alive	TUMOR F	275						Negative	0	275	0	275	0	275	0	275
TCGA-E2-	TCGA-E2-	BRCA	65	FEMALE	BLACK OI	Stage IIA	Infiltrating Lobular C	2010	Post (prio	-23845	Alive	TUMOR F	626						Negative	0	626	0	626	0	626	0	626
TCGA-E2-	TCGA-E2-	BRCA	65	FEMALE	BLACK OI	Stage IIA	Infiltrating Lobular C	2010	Post (prio	-23845	Alive	TUMOR F	626						Negative	0	626	0	626	0	626	0	626
TCGA-E2-	TCGA-E2-	BRCA	66	FEMALE	WHITE	Stage IIA	Infiltrating Lobular C	2010	Post (prio	-24266	Dead	TUMOR FREE		336					Negative	1	336	0	336	0	336	0	336
TCGA-E2-	TCGA-E2-	BRCA	66	FEMALE	WHITE	Stage IIA	Infiltrating Lobular C	2010	Post (prio	-24266	Dead	TUMOR FREE		336					Negative	1	336	0	336	0	336	0	336
TCGA-E2-	TCGA-E2-	BRCA	89	FEMALE	BLACK OI	Stage I	Infiltrating Ductal Ca	2010	Post (prio	-32583	Alive	TUMOR F	1545		Locoregional Recurrence			1210	Negative	0	1545	0	1545	1	1210	1	1210
TCGA-E2-	TCGA-E2-	BRCA	61	FEMALE	WHITE	Stage IA	Infiltrating Ductal Ca	2010	Post (prio	-22524	Alive	TUMOR F	595						Negative	0	595	0	595	0	595	0	595
TCGA-E2-	TCGA-E2-	BRCA	64	FEMALE	WHITE	Stage IIA	Infiltrating Ductal Ca	2010	Post (prio	-23570	Alive	TUMOR F	1732						Negative	0	1732	0	1732	0	1732	0	1732
TCGA-E2-	TCGA-E2-	BRCA	34	FEMALE	WHITE	Stage IIB	Infiltrating Ductal Ca	2010	Post (prio	-12671	Alive	TUMOR F	428						Negative	0	428	0	428	0	428	0	428
TCGA-E2-	TCGA-E2-	BRCA	65	FEMALE	WHITE	Stage IIA	Infiltrating Ductal Ca	2010	Post (prio	-24054	Alive	TUMOR F	1563						Negative	0	1563	0	1563	0	1563	0	1563
TCGA-E2-	TCGA-E2-	BRCA	63	FEMALE	WHITE	Stage IIB	Infiltrating Ductal Ca	2004	Post (prio	-23230	Alive	TUMOR F	2329						Negative	0	2329	0	2329	0	2329	0	2329
TCGA-E2-	TCGA-E2-	BRCA	50	FEMALE	WHITE	Stage IIIA	Infiltrating Ductal Ca	2007	Post (prio	-18346	Alive	TUMOR F	1631						Negative	0	1631	0	1631	0	1631	0	1631
TCGA-E2-	TCGA-E2-	BRCA	45	FEMALE	WHITE	Stage IIB	Infiltrating Ductal Ca	2007	Post (prio	-16603	Alive	TUMOR F	2653						Negative	0	2653	0	2653	0	2653	0	2653
TCGA-E2-	TCGA-E2-	BRCA	74	FEMALE	WHITE	Stage IIIA	Infiltrating Ductal Ca	2008	Post (prio	-27241	Dead	TUMOR FREE		1004					Negative	1	1004	0	1004	0	1004	0	1004
TCGA-E2-	TCGA-E2-	BRCA	46	FEMALE	WHITE	Stage IIA	Infiltrating Lobular C	2008	Pre (<6 m	-17039	Alive	TUMOR F	984						Negative	0	984	0	984	0	984	0	984
TCGA-E2-	TCGA-E2-	BRCA	46	FEMALE	WHITE	Stage IIA	Infiltrating Lobular C	2008	Pre (<6 m	-17039	Alive	TUMOR F	984						Negative	0	984	0	984	0	984	0	984
TCGA-E2-	TCGA-E2-	BRCA	44	FEMALE	WHITE	Stage IIA	Infiltrating Ductal Ca	2008	Pre (<6 m	-16370	Alive	TUMOR F	867						Negative	0	867	0	867	0	867	0	867
TCGA-E2-	TCGA-E2-	BRCA	63	FEMALE		Stage IA	Infiltrating Ductal Ca	2010	Post (prio	-23168	Alive	TUMOR F	501						Positive	0	501	0	501			0	501
TCGA-E2-	TCGA-E2-	BRCA	63	FEMALE		Stage IA	Infiltrating Ductal Ca	2010	Post (prio	-23168	Alive	TUMOR F	501						Positive	0	501	0	501			0	501
TCGA-E2-	TCGA-E2-	BRCA	53	FEMALE	WHITE	Stage IIA	Infiltrating Ductal Ca	2010	Post (prio	-19627	Alive	TUMOR F	1133						Negative	0	1133	0	1133	0	1133	0	1133
TCGA-E2-	TCGA-E2-	BRCA	61	FEMALE	WHITE	Stage IIB	Infiltrating Ductal Ca	2008	Post (prio	-22510	Alive	WITH TUN	2362		Distant M	Liver		1810	Negative	0	2362	0	2362	1	1810	1	1810
TCGA-E2-	TCGA-E2-	BRCA	74	FEMALE	WHITE	Stage I	Infiltrating Ductal Ca	2008	Post (prio	-27341	Alive	TUMOR F	1138						Negative	0	1138	0	1138	0	1138	0	1138
TCGA-E2-	TCGA-E2-	BRCA	74	FEMALE	WHITE	Stage I	Infiltrating Ductal Ca	2008	Post (prio	-27341	Alive	TUMOR F	1138						Negative	0	1138	0	1138	0	1138	0	1138
TCGA-E2-	TCGA-E2-	BRCA	45	FEMALE	WHITE	Stage IIB	Infiltrating Ductal Ca	2009	Pre (<6 m	-16757	Alive	TUMOR F	2140						Negative	0	2140	0	2140	0	2140	0	2140
TCGA-E2-	TCGA-E2-	BRCA	45	FEMALE	WHITE	Stage IIB	Infiltrating Ductal Ca	2009	Pre (<6 m	-16757	Alive	TUMOR F	2140						Negative	0	2140	0	2140	0	2140	0	2140
TCGA-E2-	TCGA-E2-	BRCA	80	FEMALE	WHITE	Stage I	Infiltrating Lobular C	2008	Post (prio	-29444	Alive	TUMOR F	1026						Negative	0	1026	0	1026	0	1026	0	1026
TCGA-E2-	TCGA-E2-	BRCA	51	FEMALE	WHITE	Stage I	Infiltrating Ductal Ca	2008	Post (prio	-18788	Alive	TUMOR F	1025						Negative	0	1025	0	1025	0	1025	0	1025
TCGA-E2-	TCGA-E2-	BRCA	51	FEMALE	WHITE	Stage I	Infiltrating Ductal Ca	2008	Post (prio	-18788	Alive	TUMOR F	1025						Negative	0	1025	0	1025	0	1025	0	1025
TCGA-E2-	TCGA-E2-	BRCA	57	FEMALE	WHITE	Stage I	Infiltrating Lobular C	2009	Post (prio	-20956	Alive	TUMOR F	865						Negative	0	865	0	865	0	865	0	865
TCGA-E2-	TCGA-E2-	BRCA	71	FEMALE	WHITE	Stage IIA	Infiltrating Ductal Ca	2009	Post (prio	-26052	Alive	TUMOR F	1800						Negative	0	1800	0	1800	0	1800	0	1800
TCGA-E2-	TCGA-E2-	BRCA	78	FEMALE	WHITE	Stage IIA	Infiltrating Lobular C	2009	Post (prio	-28495	Alive	TUMOR F	118						Negative	0	118	0	118	0	118	0	118
TCGA-E2-	TCGA-E2-	BRCA	60	FEMALE	WHITE	Stage I	Infiltrating Ductal Ca	2010	Post (prio	-22199	Alive	TUMOR F	675						Negative	0	675	0	675	0	675	0	675
TCGA-E2-	TCGA-E2-	BRCA	37	FEMALE	WHITE	Stage I	Infiltrating Ductal Ca	2009	Pre (<6 m	-13671	Alive	TUMOR F	1855						Negative	0	1855	0	1855	0	1855	0	1855
TCGA-E2-	TCGA-E2-	BRCA	37	FEMALE	WHITE	Stage I	Infiltrating Ductal Ca	2009	Pre (<6 m	-13671	Alive	TUMOR F	1855						Negative	0	1855	0	1855	0	1855	0	1855
TCGA-E2-	TCGA-E2-	BRCA	60	FEMALE	WHITE	Stage IA	Infiltrating Ductal Ca	2010	Post (prio	-22279	Alive	TUMOR F	337						Negative	0	337	0	337	0	337	0	337
TCGA-E2-	TCGA-E2-	BRCA	44	FEMALE	WHITE	Stage IIA	Infiltrating Ductal Ca	2007	Pre (<6 m	-16403	Alive	TUMOR F	1648						Negative	0	1648	0	1648	0	1648	0	1648
TCGA-E2-	TCGA-E2-	BRCA	40	FEMALE	WHITE	Stage IIIA	Infiltrating Ductal Ca	2009	Pre (<6 m	-14854	Alive	TUMOR F	1836		New Prim	Other, sp	left breast	1619	Negative	0	1836	0	1836	1	1619	1	1619
TCGA-E2-	TCGA-E2-	BRCA	40	FEMALE	WHITE	Stage IIIA	Infiltrating Ductal Ca	2009	Pre (<6 m	-14854	Alive	TUMOR F	1836		New Prim	Other, sp	left breast	1619	Negative	0	1836	0	1836	1	1619	1	1619
TCGA-E2-	TCGA-E2-	BRCA	52	FEMALE	BLACK OI	Stage IIB	Infiltrating Lobular C	2008	Post (prio	-19187	Alive	TUMOR F	2240						Negative	0	2240	0	2240	0	2240	0	2240
TCGA-E2-	TCGA-E2-	BRCA	40	FEMALE	WHITE	Stage IIA	Infiltrating Ductal Ca	2009	Pre (<6 m	-14868	Alive	TUMOR F	598						Negative	0	598	0	598	0	598	0	598
TCGA-E2-	TCGA-E2-	BRCA	59	FEMALE		Stage IIA	Infiltrating Ductal Ca	2009	Post (prio	-21773	Alive	TUMOR F	748						Negative	0	748	0	748	0	748	0	748
TCGA-E2-	TCGA-E2-	BRCA	41	FEMALE	BLACK OI	Stage IIB	Infiltrating Ductal Ca	2008	Pre (<6 m	-15283	Alive	TUMOR F	2306						Negative	0	2306	0	2306	0	2306	0	2306
TCGA-E2-	TCGA-E2-	BRCA	41	FEMALE	BLACK OI	Stage IIB	Infiltrating Ductal Ca	2008	Pre (<6 m	-15283	Alive	TUMOR F	2306						Negative	0	2306	0	2306	0	2306	0	2306
TCGA-E2-	TCGA-E2-	BRCA	71	FEMALE	WHITE	Stage IIIC	Infiltrating Ductal Ca	2008	Post (prio	-26239	Dead	WITH TUMOR		879	Distant M	Lung		361	Negative	1	879	1	879	1	361	1	361
TCGA-E2-	TCGA-E2-	BRCA	50	FEMALE	BLACK OI	Stage IIIA	Infiltrating Lobular C	2010	Peri (6-12	-18492	Alive	TUMOR F	1523						Negative	0	1523	0	1523	0	1523	0	1523
TCGA-E2-	TCGA-E2-	BRCA	59	FEMALE	WHITE	Stage I	Infiltrating Ductal Ca	2002	Post (prio	-21628	Alive	TUMOR F	3247						Negative	0	3247	0	3247	0	3247	0	3247
TCGA-E2-	TCGA-E2-	BRCA	59	FEMALE	WHITE	Stage I	Infiltrating Ductal Ca	2002	Post (prio	-21628	Alive	TUMOR F	3247						Negative	0	3247	0	3247	0	3247	0	3247
TCGA-E2-	TCGA-E2-	BRCA	57	FEMALE	WHITE	Stage IIB	Infiltrating Ductal Ca	2003	Post (prio	-21064	Alive	TUMOR F	3121						Close	0	3121	0	3121			0	3121
TCGA-E2-	TCGA-E2-	BRCA	57	FEMALE	WHITE	Stage IIB	Infiltrating Ductal Ca	2003	Post (prio	-21064	Alive	TUMOR F	3121						Close	0	3121	0	3121			0	3121
TCGA-E2-	TCGA-E2-	BRCA	84	FEMALE	BLACK OI	Stage IIIC	Infiltrating Ductal Ca	2008	Post (prio	-30757	Dead	WITH TUMOR		266	Locoregic	Other, sp	Left axilla	155	Negative	1	266	1	266	1	155	1	155
TCGA-E2-	TCGA-E2-	BRCA	73	FEMALE	BLACK OI	Stage IIIA	Infiltrating Ductal Ca	2008	Post (prio	-27028	Alive	WITH TUN	1309		Distant M	Liver		1303	Negative	0	1309	0	1309	1	1303	1	1303
TCGA-E2-	TCGA-E2-	BRCA	46	FEMALE	WHITE	Stage IA	Infiltrating Ductal Ca	2010	Pre (<6 m	-16887	Alive	TUMOR F	1604						Negative	0	1604	0	1604	0	1604	0	1604
TCGA-E2-	TCGA-E2-	BRCA	46	FEMALE	WHITE	Stage IA	Infiltrating Ductal Ca	2010	Pre (<6 m	-16887	Alive	TUMOR F	1604						Negative	0	1604	0	1604	0	1604	0	1604
TCGA-E2-	TCGA-E2-	BRCA	78	FEMALE	BLACK OI	Stage IIIC	Infiltrating Lobular C	2011	Post (prio	-28621	Dead	WITH TUMOR		821	Distant M	Bone		597	Negative	1	821	1	821	1	597	1	597
TCGA-E2-	TCGA-E2-	BRCA	77	FEMALE		Stage IIB	Infiltrating Lobular C	2011	Post (prio	-28411	Alive	TUMOR F	1051						Negative	0	1051	0	1051	0	1051	0	1051
TCGA-E2-	TCGA-E2-	BRCA	43	FEMALE	WHITE	Stage IIIC	Infiltrating Lobular C	2011	Pre (<6 m	-15969	Alive	TUMOR F	1325						Positive	0	1325	0	1325			0	1325
TCGA-E2-	TCGA-E2-	BRCA	69	FEMALE	BLACK OI	Stage IIB	Infiltrating Ductal Ca	2012	Post (prio	-25282	Alive	TUMOR F	252						Negative	0	252	0	252	0	252	0	252
TCGA-E2-	TCGA-E2-	BRCA	47	FEMALE	BLACK OI	Stage IB	Infiltrating Ductal Ca	2011	Pre (<6 m	-17456	Alive	TUMOR F	931						Negative	0	931	0	931	0	931	0	931
TCGA-E2-	TCGA-E2-	BRCA	72	FEMALE	BLACK OI	Stage IIIA	Infiltrating Ductal Ca	2011	Post (prio	-26399	Alive	TUMOR F	1208						Negative	0	1208	0	1208	0	1208	0	1208
TCGA-E2-	TCGA-E2-	BRCA	48	FEMALE	BLACK OI	Stage IA	Infiltrating Ductal Ca	2011	Peri (6-12	-17689	Alive	TUMOR F	1062						Negative	0	1062	0	1062	0	1062	0	1062
TCGA-E2-	TCGA-E2-	BRCA	44	FEMALE	BLACK OI	Stage IIA	Infiltrating Ductal Ca	2011	Pre (<6 m	-16195	Alive	TUMOR F	1179						Negative	0	1179	0	1179	0	1179	0	1179
TCGA-E2-	TCGA-E2-	BRCA	69	FEMALE	BLACK OI	Stage IA	Infiltrating Lobular C	2011	Post (prio	-25350	Alive	TUMOR F	1043						Negative	0	1043	0	1043	0	1043	0	1043
TCGA-E2-	TCGA-E2-	BRCA	90	FEMALE	BLACK OI	Stage IIIC	Infiltrating Ductal Ca	2013	Post (prio	-32873	Alive	TUMOR F	538						Negative	0	538	0	538	0	538	0	538
TCGA-E9-	TCGA-E9-	BRCA	70	FEMALE	WHITE	Stage IIIA	Mixed Histology (ple	2010	Post (prio	-25714	Alive	TUMOR F	1059						Negative	0	1059	0	1059	0	1059	0	1059
TCGA-E9-	TCGA-E9-	BRCA	41	FEMALE	WHITE	Stage IIIA	Mixed Histology (ple	2010	Pre (<6 m	-15055	Alive	TUMOR F	1000						Negative	0	1000	0	1000	0	1000	0	1000
TCGA-E9-	TCGA-E9-	BRCA	41	FEMALE	WHITE	Stage IIIA	Mixed Histology (ple	2010	Pre (<6 m	-15055	Alive	TUMOR F	1000						Negative	0	1000	0	1000	0	1000	0	1000
TCGA-E9-	TCGA-E9-	BRCA	45	FEMALE	WHITE	Stage IIB	Mixed Histology (ple	2010	Post (prio	-16736	Alive	TUMOR F	1120						Negative	0	1120	0	1120	0	1120	0	1120
TCGA-E9-	TCGA-E9-	BRCA	45	FEMALE	WHITE	Stage IIB	Mixed Histology (ple	2010	Post (prio	-16736	Alive	TUMOR F	1120						Negative	0	1120	0	1120	0	1120	0	1120
TCGA-E9-	TCGA-E9-	BRCA	52	FEMALE	WHITE	Stage IIB	Infiltrating Ductal Ca	2010	Post (prio	-19327	Dead	WITH TUMOR		678	Distant M	Bone		443	Negative	1	678	1	678	1	443	1	443
TCGA-E9-	TCGA-E9-	BRCA	52	FEMALE	WHITE	Stage IIB	Infiltrating Ductal Ca	2010	Post (prio	-19327	Dead	WITH TUMOR		678	Distant M	Bone		443	Negative	1	678	1	678	1	443	1	443
TCGA-E9-	TCGA-E9-	BRCA	48	FEMALE	WHITE	Stage IIA	Infiltrating Ductal Ca	2010	Post (prio	-17652	Alive	TUMOR F	1039						Negative	0	1039	0	1039	0	1039	0	1039
TCGA-E9-	TCGA-E9-	BRCA	48	FEMALE	WHITE	Stage IIA	Infiltrating Ductal Ca	2010	Post (prio	-17652	Alive	TUMOR F	1039						Negative	0	1039	0	1039	0	1039	0	1039
TCGA-E9-	TCGA-E9-	BRCA	58	FEMALE	WHITE	Stage IIA	Mixed Histology (ple	2011	Post (prio	-21228	Alive	TUMOR F	1101						Negative	0	1101	0	1101	0	1101	0	1101
TCGA-E9-	TCGA-E9-	BRCA	58	FEMALE	WHITE	Stage IIA	Mixed Histology (ple	2011	Post (prio	-21228	Alive	TUMOR F	1101						Negative	0	1101	0	1101	0	1101	0	1101
TCGA-E9-	TCGA-E9-	BRCA	58	FEMALE	WHITE	Stage IIA	Mixed Histology (ple	2011	Post (prio	-21397	Alive	TUMOR F	1112						Negative	0	1112	0	1112	0	1112	0	1112
TCGA-E9-	TCGA-E9-	BRCA	58	FEMALE	WHITE	Stage IIA	Mixed Histology (ple	2011	Post (prio	-21397	Alive	TUMOR F	1112						Negative	0	1112	0	1112	0	1112	0	1112
TCGA-E9-	TCGA-E9-	BRCA	61	FEMALE	WHITE	Stage IIB	Mixed Histology (ple	2011	Post (prio	-22355	Alive	TUMOR F	1203						Negative	0	1203	0	1203	0	1203	0	1203
TCGA-E9-	TCGA-E9-	BRCA	75	FEMALE	WHITE	Stage IIB	Infiltrating Ductal Ca	2011	Post (prio	-27400	Alive	TUMOR F	1266						Negative	0	1266	0	1266	0	1266	0	1266
TCGA-E9-	TCGA-E9-	BRCA	75	FEMALE	WHITE	Stage IIB	Infiltrating Ductal Ca	2011	Post (prio	-27400	Alive	TUMOR F	1266						Negative	0	1266	0	1266	0	1266	0	1266
TCGA-E9-	TCGA-E9-	BRCA	28	FEMALE	WHITE	Stage IIB	Infiltrating Ductal Ca	2011	Pre (<6 m	-10564	Alive	TUMOR F	1088						Negative	0	1088	0	1088	0	1088	0	1088
TCGA-E9-	TCGA-E9-	BRCA	28	FEMALE	WHITE	Stage IIB	Infiltrating Ductal Ca	2011	Pre (<6 m	-10564	Alive	TUMOR F	1088						Negative	0	1088	0	1088	0	1088	0	1088
TCGA-E9-	TCGA-E9-	BRCA	60	FEMALE	WHITE	Stage IIA	Mixed Histology (ple	2011	Post (prio	-21981	Dead	WITH TUMOR		1072	Distant M	Lung		90	Negative	1	1072	1	1072	1	90	1	90
TCGA-E9-	TCGA-E9-	BRCA	60	FEMALE	WHITE	Stage IIA	Mixed Histology (ple	2011	Post (prio	-21981	Dead	WITH TUMOR		1072	Distant M	Lung		90	Negative	1	1072	1	1072	1	90	1	90
TCGA-E9-	TCGA-E9-	BRCA	62	FEMALE	WHITE	Stage IIA	Infiltrating Ductal Ca	2011	Post (prio	-22896	Dead	TUMOR FREE		786					Negative	1	786	0	786	0	786	0	786
TCGA-E9-	TCGA-E9-	BRCA	62	FEMALE	WHITE	Stage IIA	Infiltrating Ductal Ca	2011	Post (prio	-22896	Dead	TUMOR FREE		786					Negative	1	786	0	786	0	786	0	786
TCGA-E9-	TCGA-E9-	BRCA	71	FEMALE	WHITE	Stage IIB	Infiltrating Ductal Ca	2011	Post (prio	-26221	Alive	TUMOR F	576						Negative	0	576	0	576	0	576	0	576
TCGA-E9-	TCGA-E9-	BRCA	51	FEMALE	WHITE	Stage IIA	Mixed Histology (ple	2011	Post (prio	-18985	Alive	TUMOR F	300						Negative	0	300	0	300	0	300	0	300
TCGA-E9-	TCGA-E9-	BRCA	61	FEMALE	WHITE	Stage IIA	Infiltrating Ductal Ca	2011	Post (prio	-22397	Alive	TUMOR F	755						Negative	0	755	0	755	0	755	0	755
TCGA-E9-	TCGA-E9-	BRCA	58	FEMALE	WHITE	Stage IIA	Infiltrating Ductal Ca	2011	Post (prio	-21394	Alive	TUMOR F	860							0	860	0	860			0	860
TCGA-E9-	TCGA-E9-	BRCA	51	FEMALE	WHITE	Stage IIIC	Infiltrating Ductal Ca	2011	Post (prio	-18936	Alive	TUMOR F	1063						Positive	0	1063	0	1063			0	1063
TCGA-E9-	TCGA-E9-	BRCA	70	FEMALE	WHITE	Stage IIIC	Infiltrating Ductal Ca	2010	Post (prio	-25693	Alive	TUMOR F	78						Negative	0	78	0	78	0	78	0	78
TCGA-E9-	TCGA-E9-	BRCA	66	FEMALE	WHITE	Stage IA	Infiltrating Ductal Ca	2011	Post (prio	-24181	Alive	TUMOR F	186						Negative	0	186	0	186	0	186	0	186
TCGA-E9-	TCGA-E9-	BRCA	63	FEMALE	WHITE	Stage IA	Infiltrating Ductal Ca	2011	Post (prio	-23116	Alive	TUMOR F	92						Negative	0	92	0	92	0	92	0	92
TCGA-E9-	TCGA-E9-	BRCA	63	FEMALE	WHITE	Stage IIA	Infiltrating Ductal Ca	2010	Post (prio	-23347	Alive	TUMOR F	339						Negative	0	339	0	339	0	339	0	339
TCGA-E9-	TCGA-E9-	BRCA	64	FEMALE	WHITE	Stage IIA	Infiltrating Ductal Ca	2011	Post (prio	-23397	Alive	TUMOR F	1467						Negative	0	1467	0	1467	0	1467	0	1467
TCGA-E9-	TCGA-E9-	BRCA	64	FEMALE	WHITE	Stage IIA	Infiltrating Ductal Ca	2011	Post (prio	-23397	Alive	TUMOR F	1467						Negative	0	1467	0	1467	0	1467	0	1467
TCGA-E9-	TCGA-E9-	BRCA	48	FEMALE	WHITE	Stage IA	Infiltrating Ductal Ca	2011	Pre (<6 m	-17699	Alive	TUMOR F	1369						Negative	0	1369	0	1369	0	1369	0	1369
TCGA-E9-	TCGA-E9-	BRCA	40	FEMALE	WHITE	Stage IIA	Infiltrating Ductal Ca	2011	Pre (<6 m	-14755	Dead	TUMOR FREE		976					Negative	1	976	0	976	0	976	0	976
TCGA-E9-	TCGA-E9-	BRCA	40	FEMALE	WHITE	Stage IIA	Infiltrating Ductal Ca	2011	Pre (<6 m	-14755	Dead	TUMOR FREE		976					Negative	1	976	0	976	0	976	0	976
TCGA-E9-	TCGA-E9-	BRCA	56	FEMALE	WHITE	Stage IIIC	Mixed Histology (ple	2007	Post (prio	-20795	Alive	TUMOR F	1224						Negative	0	1224	0	1224	0	1224	0	1224
TCGA-E9-	TCGA-E9-	BRCA	56	FEMALE	WHITE	Stage IIIC	Mixed Histology (ple	2007	Post (prio	-20795	Alive	TUMOR F	1224						Negative	0	1224	0	1224	0	1224	0	1224
TCGA-E9-	TCGA-E9-	BRCA	67	FEMALE	WHITE	Stage IIA	Infiltrating Ductal Ca	2011	Post (prio	-24719	Alive	TUMOR F	34						Negative	0	34	0	34	0	34	0	34
TCGA-E9-	TCGA-E9-	BRCA	67	FEMALE	WHITE	Stage IIA	Infiltrating Ductal Ca	2011	Post (prio	-24719	Alive	TUMOR F	34						Negative	0	34	0	34	0	34	0	34
TCGA-E9-	TCGA-E9-	BRCA	74	FEMALE	WHITE	Stage IIIA	Infiltrating Ductal Ca	2011	Post (prio	-27392	Alive	TUMOR F	1419						Negative	0	1419	0	1419	0	1419	0	1419
TCGA-E9-	TCGA-E9-	BRCA	68	FEMALE	WHITE	Stage IIIA	Infiltrating Ductal Ca	2011	Post (prio	-25119	Alive	TUMOR F	200						Negative	0	200	0	200	0	200	0	200
TCGA-E9-	TCGA-E9-	BRCA	68	FEMALE	WHITE	Stage IIIA	Infiltrating Ductal Ca	2011	Post (prio	-25119	Alive	TUMOR F	200						Negative	0	200	0	200	0	200	0	200
TCGA-E9-	TCGA-E9-	BRCA	62	FEMALE	WHITE	Stage IIIA	Infiltrating Ductal Ca	2011	Post (prio	-22788	Alive	TUMOR F	647						Negative	0	647	0	647	0	647	0	647
TCGA-E9-	TCGA-E9-	BRCA	63	FEMALE	WHITE	Stage IIA	Infiltrating Ductal Ca	2011	Post (prio	-23224	Alive	TUMOR F	1417						Negative	0	1417	0	1417	0	1417	0	1417
TCGA-E9-	TCGA-E9-	BRCA	63	FEMALE	WHITE	Stage IIA	Infiltrating Ductal Ca	2011	Post (prio	-23224	Alive	TUMOR F	1417						Negative	0	1417	0	1417	0	1417	0	1417
TCGA-E9-	TCGA-E9-	BRCA	43	FEMALE	WHITE	Stage IIIA	Mixed Histology (ple	2011	Pre (<6 m	-15942	Alive	TUMOR F	1449						Negative	0	1449	0	1449	0	1449	0	1449
TCGA-E9-	TCGA-E9-	BRCA	43	FEMALE	WHITE	Stage IIIA	Mixed Histology (ple	2011	Pre (<6 m	-15942	Alive	TUMOR F	1449						Negative	0	1449	0	1449	0	1449	0	1449
TCGA-E9-	TCGA-E9-	BRCA	45	FEMALE	WHITE	Stage IIIA	Infiltrating Ductal Ca	2011	Pre (<6 m	-16511	Dead	WITH TUMOR		1048	Distant M	Bone		790	Negative	1	1048	1	1048	1	790	1	790
TCGA-E9-	TCGA-E9-	BRCA	42	FEMALE	WHITE	Stage IIB	Infiltrating Ductal Ca	2011	Pre (<6 m	-15602	Alive	TUMOR F	975						Negative	0	975	0	975	0	975	0	975
TCGA-E9-	TCGA-E9-	BRCA	58	FEMALE	WHITE	Stage IIB	Infiltrating Ductal Ca	2011	Post (prio	-21294	Alive	TUMOR F	1285						Negative	0	1285	0	1285	0	1285	0	1285
TCGA-E9-	TCGA-E9-	BRCA	37	FEMALE	WHITE	Stage IA	Infiltrating Ductal Ca	2011	Pre (<6 m	-13817	Alive	TUMOR F	1148						Negative	0	1148	0	1148	0	1148	0	1148
TCGA-E9-	TCGA-E9-	BRCA	74	FEMALE	WHITE	Stage IIA	Infiltrating Ductal Ca	2011	Post (prio	-27288	Alive	TUMOR F	1189						Negative	0	1189	0	1189	0	1189	0	1189
TCGA-E9-	TCGA-E9-	BRCA	71	FEMALE	WHITE	Stage IA	Mixed Histology (ple	2011	Post (prio	-26278	Alive	TUMOR F	1167						Negative	0	1167	0	1167	0	1167	0	1167
TCGA-E9-	TCGA-E9-	BRCA	38	FEMALE	WHITE	Stage IIA	Infiltrating Ductal Ca	2011	Pre (<6 m	-14115	Alive	TUMOR F	1248						Negative	0	1248	0	1248	0	1248	0	1248
TCGA-E9-	TCGA-E9-	BRCA	56	FEMALE	WHITE	Stage IIIA	Infiltrating Ductal Ca	2011	Post (prio	-20574	Alive	TUMOR F	1269						Negative	0	1269	0	1269	0	1269	0	1269
TCGA-E9-	TCGA-E9-	BRCA	47	FEMALE	WHITE	Stage IIA	Infiltrating Ductal Ca	2011	Post (prio	-17300	Alive	TUMOR F	1239						Negative	0	1239	0	1239	0	1239	0	1239
TCGA-E9-	TCGA-E9-	BRCA	42	FEMALE	WHITE	Stage IIB	Infiltrating Ductal Ca	2011	Pre (<6 m	-15504	Alive	TUMOR F	1232						Negative	0	1232	0	1232	0	1232	0	1232
TCGA-E9-	TCGA-E9-	BRCA	52	FEMALE	WHITE	Stage IIA	Infiltrating Ductal Ca	2011	Peri (6-12	-19035	Alive	WITH TUN	612		Distant M	Bone		305	Negative	0	612	0	612	1	305	1	305
TCGA-E9-	TCGA-E9-	BRCA	54	FEMALE	WHITE	Stage IIA	Infiltrating Ductal Ca	2011	Post (prio	-19778	Alive	TUMOR F	21						Negative	0	21	0	21	0	21	0	21
TCGA-E9-	TCGA-E9-	BRCA	47	FEMALE	WHITE	Stage IIB	Infiltrating Ductal Ca	2011	Pre (<6 m	-17363	Alive	TUMOR F	26						Negative	0	26	0	26	0	26	0	26
TCGA-E9-	TCGA-E9-	BRCA	59	FEMALE	WHITE	Stage IA	Infiltrating Ductal Ca	2011	Post (prio	-21562	Alive	TUMOR F	1186						Negative	0	1186	0	1186	0	1186	0	1186
TCGA-E9-	TCGA-E9-	BRCA	51	FEMALE	WHITE	Stage IIA	Infiltrating Ductal Ca	2011	Peri (6-12	-18684	Alive	TUMOR F	59						Negative	0	59	0	59	0	59	0	59
TCGA-E9-	TCGA-E9-	BRCA	45	FEMALE	WHITE	Stage IIA	Infiltrating Ductal Ca	2011	Pre (<6 m	-16689	Alive	TUMOR F	217						Negative	0	217	0	217	0	217	0	217
TCGA-E9-	TCGA-E9-	BRCA	69	FEMALE	WHITE	Stage IIA	Infiltrating Ductal Ca	2011	Post (prio	-25370	Alive	TUMOR F	747						Negative	0	747	0	747	0	747	0	747
TCGA-E9-	TCGA-E9-	BRCA	71	FEMALE	WHITE	Stage IIA	Infiltrating Lobular C	2011	Post (prio	-25957	Alive	TUMOR F	375						Negative	0	375	0	375	0	375	0	375
TCGA-E9-	TCGA-E9-	BRCA	72	FEMALE	WHITE	Stage IIB	Infiltrating Lobular C	2011	Post (prio	-26407	Dead	WITH TUMOR		904	Distant M	Bone	Liver	524	Negative	1	904	1	904	1	524	1	524
TCGA-E9-	TCGA-E9-	BRCA	63	FEMALE	WHITE	Stage IIA	Infiltrating Lobular C	2011	Post (prio	-23318	Alive	TUMOR F	288						Negative	0	288	0	288	0	288	0	288
TCGA-E9-	TCGA-E9-	BRCA	49	FEMALE	WHITE	Stage IIA	Infiltrating Lobular C	2011	Pre (<6 m	-18046	Alive	TUMOR F	1158						Negative	0	1158	0	1158	0	1158	0	1158
TCGA-E9-	TCGA-E9-	BRCA	78	FEMALE	WHITE	Stage IIIC	Infiltrating Lobular C	2011	Post (prio	-28715	Alive	TUMOR F	1001						Negative	0	1001	0	1001	0	1001	0	1001
TCGA-E9-	TCGA-E9-	BRCA	33	FEMALE	WHITE	Stage IIA	Medullary Carcinoma	2011	Pre (<6 m	-12142	Alive	TUMOR F	918						Negative	0	918	0	918	0	918	0	918
TCGA-E9-	TCGA-E9-	BRCA	48	FEMALE	WHITE	Stage IIB	Infiltrating Lobular C	2011	Post (prio	-17588	Alive	TUMOR F	926						Negative	0	926	0	926	0	926	0	926
TCGA-E9-	TCGA-E9-	BRCA	85	FEMALE	WHITE	Stage IA	Mucinous Carcinoma	2012	Post (prio	-31173	Alive	TUMOR F	727						Negative	0	727	0	727	0	727	0	727
TCGA-E9-	TCGA-E9-	BRCA	63	FEMALE	WHITE	Stage IIIA	Infiltrating Lobular C	2012	Post (prio	-23057	Alive	TUMOR F	725						Negative	0	725	0	725	0	725	0	725
TCGA-E9-	TCGA-E9-	BRCA	60	FEMALE	WHITE	Stage IIIC	Infiltrating Lobular C	2012	Post (prio	-22199	Alive	TUMOR F	812						Negative	0	812	0	812	0	812	0	812
TCGA-E9-	TCGA-E9-	BRCA	65	FEMALE	WHITE	Stage IIB	Metaplastic Carcinor	2012	Post (prio	-24053	Alive	TUMOR F	24						Negative	0	24	0	24	0	24	0	24
TCGA-E9-	TCGA-E9-	BRCA	41	FEMALE	WHITE	Stage IIA	Mucinous Carcinoma	2012	Pre (<6 m	-15050	Alive	TUMOR F	785						Negative	0	785	0	785	0	785	0	785
TCGA-E9-	TCGA-E9-	BRCA	63	FEMALE	WHITE	Stage IIA	Other, specify	2012	Post (prio	-23371	Alive	TUMOR F	803						Negative	0	803	0	803	0	803	0	803
TCGA-E9-	TCGA-E9-	BRCA	45	FEMALE	WHITE	Stage IIIC	Infiltrating Lobular C	2012	Pre (<6 m	-16697	Alive	TUMOR F	847						Negative	0	847	0	847	0	847	0	847
TCGA-EW	TCGA-EW	BRCA	80	FEMALE	WHITE	Stage IIB	Infiltrating Lobular C	2010	Post (prio	-29529	Alive	TUMOR F	371						Negative	0	371	0	371	0	371	0	371
TCGA-EW	TCGA-EW	BRCA	48	FEMALE	ASIAN	Stage IIA	Other, specify	2008	Peri (6-12	-17597	Alive	TUMOR F	1208						Negative	0	1208	0	1208	0	1208	0	1208
TCGA-EW	TCGA-EW	BRCA	38	FEMALE	ASIAN	Stage I	Infiltrating Ductal Ca	2009	Pre (<6 m	-14122	Alive	TUMOR F	258						Negative	0	258	0	258	0	258	0	258
TCGA-EW	TCGA-EW	BRCA	53	FEMALE	WHITE	Stage IIIA	Infiltrating Ductal Ca	2010	Post (prio	-19706	Alive	TUMOR F	554						Negative	0	554	0	554	0	554	0	554
TCGA-EW	TCGA-EW	BRCA	38	FEMALE	WHITE	Stage IIB	Infiltrating Ductal Ca	2010	Pre (<6 m	-14122	Alive	TUMOR F	575						Negative	0	575	0	575	0	575	0	575
TCGA-EW	TCGA-EW	BRCA	50	FEMALE	BLACK OI	[Discrepancy]	Infiltrating Lobular C	2010	Pre (<6 m	-18558	Alive	TUMOR F	403						Negative	0	403	0	403	0	403	0	403
TCGA-EW	TCGA-EW	BRCA	61	FEMALE	WHITE	Stage IA	Infiltrating Lobular C	2010	Post (prio	-22332	Alive	TUMOR F	504						Negative	0	504	0	504	0	504	0	504
TCGA-EW	TCGA-EW	BRCA	59	FEMALE	WHITE	Stage IIB	Infiltrating Lobular C	2010	Post (prio	-21559	Alive	TUMOR F	477						Negative	0	477	0	477	0	477	0	477
TCGA-EW	TCGA-EW	BRCA	70	FEMALE	WHITE	Stage I	Infiltrating Ductal Ca	2009	Post (prio	-25830	Alive	TUMOR F	875						Negative	0	875	0	875	0	875	0	875
TCGA-EW	TCGA-EW	BRCA	56	FEMALE	WHITE	Stage IIB	Infiltrating Ductal Ca	2009	Post (prio	-20764	Alive	TUMOR F	789						Negative	0	789	0	789	0	789	0	789
TCGA-EW	TCGA-EW	BRCA	58	FEMALE	BLACK OI	Stage IIA	Infiltrating Ductal Ca	2009	Post (prio	-21465	Alive	TUMOR F	694						Negative	0	694	0	694	0	694	0	694
TCGA-EW	TCGA-EW	BRCA	43	FEMALE	WHITE	Stage IIA	Mucinous Carcinoma	2009	Pre (<6 m	-15837	Alive	TUMOR F	911						Negative	0	911	0	911	0	911	0	911
TCGA-EW	TCGA-EW	BRCA	63	FEMALE	WHITE	Stage IIA	Infiltrating Ductal Ca	2009	Post (prio	-23266	Alive	TUMOR F	908						Negative	0	908	0	908	0	908	0	908
TCGA-EW	TCGA-EW	BRCA	56	FEMALE	BLACK OI	Stage IIA	Infiltrating Ductal Ca	2008	Post (prio	-20664	Alive	TUMOR F	1229						Negative	0	1229	0	1229	0	1229	0	1229
TCGA-EW	TCGA-EW	BRCA	55	FEMALE	WHITE	Stage IIB	Other, specify	2008	Post (prio	-20107	Alive	TUMOR F	1251		Distant M	Other, sp	mediastin	1094	Negative	0	1251	0	1251	1	1094	1	1094
TCGA-EW	TCGA-EW	BRCA	68	FEMALE	WHITE	Stage IIIC	Infiltrating Ductal Ca	2008	Post (prio	-24923	Alive	TUMOR F	1210		Distant M	Lung		1014	Negative	0	1210	0	1210	1	1014	1	1014
TCGA-EW	TCGA-EW	BRCA	48	FEMALE	WHITE	Stage IIA	Infiltrating Ductal Ca	2008	Pre (<6 m	-17637	Alive	TUMOR F	1611						Negative	0	1611	0	1611	0	1611	0	1611
TCGA-EW	TCGA-EW	BRCA	43	FEMALE	WHITE	Stage IIA	Medullary Carcinoma	2009	Pre (<6 m	-15989	Alive	TUMOR F	907						Negative	0	907	0	907	0	907	0	907
TCGA-EW	TCGA-EW	BRCA	77	FEMALE	WHITE	Stage IIB	Infiltrating Ductal Ca	2010	Post (prio	-28177	Alive	TUMOR F	703						Negative	0	703	0	703	0	703	0	703
TCGA-EW	TCGA-EW	BRCA	64	FEMALE	WHITE	Stage IIB	Infiltrating Ductal Ca	2010	Post (prio	-23485	Alive	TUMOR F	562						Close	0	562	0	562			0	562
TCGA-EW	TCGA-EW	BRCA	59	FEMALE	WHITE	Stage IIA	Infiltrating Ductal Ca	2009	Post (prio	-21750	Alive	WITH TUN	915		Distant M	Other, sp	mediastin	729	Negative	0	915	0	915	1	729	1	729
TCGA-EW	TCGA-EW	BRCA	58	FEMALE	WHITE	Stage IIIC	Infiltrating Ductal Ca	2010	Post (prio	-21501	Dead	WITH TUMOR		239	Distant M	Lung		207	Negative	1	239	1	239	1	207	1	207
TCGA-EW	TCGA-EW	BRCA	59	FEMALE	WHITE	Stage IIB	Infiltrating Ductal Ca	2010	Post (prio	-21837	Alive	TUMOR F	575						Negative	0	575	0	575	0	575	0	575
TCGA-EW	TCGA-EW	BRCA	70	FEMALE	BLACK OI	Stage IIIA	Infiltrating Ductal Ca	2009	Post (prio	-25770	Alive	TUMOR F	608						Negative	0	608	0	608	0	608	0	608
TCGA-EW	TCGA-EW	BRCA	66	FEMALE	BLACK OI	Stage IIB	Infiltrating Ductal Ca	2011	Post (prio	-24434	Alive	TUMOR F	187						Negative	0	187	0	187	0	187	0	187
TCGA-EW	TCGA-EW	BRCA	61	MALE	WHITE	Stage IIA	Infiltrating Ductal Ca	2010		-22536	Alive	TUMOR F	424						Negative	0	424	0	424	0	424	0	424
TCGA-EW	TCGA-EW	BRCA	56	FEMALE	WHITE	Stage IIA	Infiltrating Ductal Ca	2011	Post (prio	-20694	Alive	TUMOR F	320						Negative	0	320	0	320	0	320	0	320
TCGA-EW	TCGA-EW	BRCA	50	FEMALE	WHITE	Stage IA	Infiltrating Ductal Ca	2010	Post (prio	-18384	Alive	TUMOR F	439						Negative	0	439	0	439	0	439	0	439
TCGA-EW	TCGA-EW	BRCA	53	FEMALE	WHITE	Stage IIB	Infiltrating Lobular C	2008	Post (prio	-19464	Alive	TUMOR F	1051						Negative	0	1051	0	1051	0	1051	0	1051
TCGA-EW	TCGA-EW	BRCA	52	FEMALE	WHITE	Stage IIA	Infiltrating Ductal Ca	2010	Post (prio	-19073	Alive	TUMOR F	607						Negative	0	607	0	607	0	607	0	607
TCGA-EW	TCGA-EW	BRCA	59	FEMALE	BLACK OI	Stage IIIC	Infiltrating Ductal Ca	2008	Post (prio	-21628	Alive	TUMOR F	1673						Negative	0	1673	0	1673	0	1673	0	1673
TCGA-EW	TCGA-EW	BRCA	41	FEMALE	BLACK OI	Stage IIB	Infiltrating Ductal Ca	2008	Pre (<6 m	-15302	Alive	TUMOR F	1604						Negative	0	1604	0	1604	0	1604	0	1604
TCGA-EW	TCGA-EW	BRCA	39	FEMALE	WHITE	Stage IIIC	Other, specify	2011	Pre (<6 m	-14316	Alive	TUMOR F	788						Negative	0	788	0	788	0	788	0	788
TCGA-EW	TCGA-EW	BRCA	52	FEMALE	WHITE	Stage IIA	Other, specify	2011	Post (prio	-19085	Alive	TUMOR F	672						Negative	0	672	0	672	0	672	0	672
TCGA-EW	TCGA-EW	BRCA	60	FEMALE	WHITE	Stage IIB	Infiltrating Lobular C	2011	[Not Evalu	-21922	Alive	TUMOR F	1035						Negative	0	1035	0	1035	0	1035	0	1035
TCGA-EW	TCGA-EW	BRCA	61	FEMALE	BLACK OI	Stage IIIA	Infiltrating Ductal Ca	2011	Post (prio	-22520	Alive	TUMOR F	532						Negative	0	532	0	532	0	532	0	532
TCGA-EW	TCGA-EW	BRCA	75	FEMALE	WHITE	Stage IIA	Infiltrating Lobular C	2012	Post (prio	-27627	Alive	TUMOR F	533						Negative	0	533	0	533	0	533	0	533
TCGA-EW	TCGA-EW	BRCA	51	FEMALE	WHITE	Stage IIIA	Infiltrating Lobular C	2012	[Not Evalu	-18850	Alive	TUMOR F	715						Negative	0	715	0	715	0	715	0	715
TCGA-EW	TCGA-EW	BRCA	34	FEMALE	BLACK OI	Stage IIA	Infiltrating Ductal Ca	2013		-12755	Alive	TUMOR F	463							0	463	0	463			0	463
TCGA-EW	TCGA-EW	BRCA	59	MALE	BLACK OI	Stage II	Infiltrating Ductal Ca	2013		-21623	Alive	TUMOR F	510						Negative	0	510	0	510	0	510	0	510
TCGA-EW	TCGA-EW	BRCA	62	FEMALE	BLACK OI	Stage II	Infiltrating Ductal Ca	2012		-22844	Alive	TUMOR F	760						Negative	0	760	0	760	0	760	0	760
TCGA-EW	TCGA-EW	BRCA	60	FEMALE	BLACK OI	Stage IIA	Infiltrating Lobular C	2012		-21918	Alive	TUMOR F	952						Negative	0	952	0	952	0	952	0	952
TCGA-EW	TCGA-EW	BRCA	32	FEMALE	BLACK OI	Stage IIB	Infiltrating Ductal Ca	2012		-12006	Alive	TUMOR F	1010						Negative	0	1010	0	1010	0	1010	0	1010
TCGA-GI-	TCGA-GI-	BRCA	63	FEMALE	WHITE	Stage IIIB	Infiltrating Ductal Ca	2004		-23103	Alive		225						Negative	0	225	0	225	0	225	0	225
TCGA-GI-	TCGA-GI-	BRCA	63	FEMALE	WHITE	Stage IIIB	Infiltrating Ductal Ca	2004		-23103	Alive		225						Negative	0	225	0	225	0	225	0	225
TCGA-GI-	TCGA-GI-	BRCA	58	FEMALE	BLACK OI	Stage IIB	Infiltrating Ductal Ca	2003	Post (prio	-21534	Alive	TUMOR F	3342						Negative	0	3342	0	3342	0	3342	0	3342
TCGA-GI-	TCGA-GI-	BRCA	58	FEMALE	BLACK OI	Stage IIB	Infiltrating Ductal Ca	2003	Post (prio	-21534	Alive	TUMOR F	3342						Negative	0	3342	0	3342	0	3342	0	3342
TCGA-GM	TCGA-GM	BRCA	69	FEMALE	WHITE	Stage I	Infiltrating Ductal Ca	2006	Post (prio	-25490	Dead	WITH TUMOR		1812	Distant M	Bone		536	Negative	1	1812	1	1812	1	536	1	536
TCGA-GM	TCGA-GM	BRCA	46	FEMALE	WHITE	Stage IIB	Infiltrating Ductal Ca	1993	Post (prio	-16854	Dead	WITH TUMOR		6593	Distant M	Bone		3014	Negative	1	6593	1	6593	1	3014	1	3014
TCGA-GM	TCGA-GM	BRCA	62	FEMALE	WHITE	Stage IIA	Infiltrating Ductal Ca	2006	Post (prio	-22883	Alive	TUMOR F	2406						Negative	0	2406	0	2406	0	2406	0	2406
TCGA-GM	TCGA-GM	BRCA	57	FEMALE	WHITE	Stage IIA	Infiltrating Ductal Ca	2006	Post (prio	-21122	Alive	TUMOR F	2535						Negative	0	2535	0	2535	0	2535	0	2535
TCGA-GM	TCGA-GM	BRCA	53	FEMALE	WHITE	Stage I	Infiltrating Ductal Ca	2007	Post (prio	-19415	Alive	TUMOR F	2282						Negative	0	2282	0	2282	0	2282	0	2282
TCGA-GM	TCGA-GM	BRCA	53	FEMALE	BLACK OI	Stage IIA	Infiltrating Ductal Ca	2007	Post (prio	-19614	Alive	TUMOR F	2155						Negative	0	2155	0	2155	0	2155	0	2155
TCGA-GM	TCGA-GM	BRCA	58	FEMALE	WHITE	Stage I	Infiltrating Ductal Ca	2007	Post (prio	-21344	Alive	TUMOR F	2193						Negative	0	2193	0	2193	0	2193	0	2193
TCGA-GM	TCGA-GM	BRCA	52	FEMALE	WHITE	Stage I	Infiltrating Ductal Ca	2005	Pre (<6 m	-19030	Alive	TUMOR F	2590						Negative	0	2590	0	2590	0	2590	0	2590
TCGA-GM	TCGA-GM	BRCA	58	FEMALE	WHITE	Stage I	Infiltrating Ductal Ca	2005	Post (prio	-21198	Alive	TUMOR F	2645						Negative	0	2645	0	2645	0	2645	0	2645
TCGA-GM	TCGA-GM	BRCA	50	FEMALE	WHITE	Stage I	Infiltrating Ductal Ca	2003	Peri (6-12	-18475	Alive	TUMOR F	3519						Negative	0	3519	0	3519	0	3519	0	3519
TCGA-GM	TCGA-GM	BRCA	57	FEMALE	WHITE	Stage IIA	Infiltrating Ductal Ca	2004	Post (prio	-21160	Alive	TUMOR F	3226						Negative	0	3226	0	3226	0	3226	0	3226
TCGA-GM	TCGA-GM	BRCA	58	FEMALE	WHITE	Stage IIA	Infiltrating Ductal Ca	2004	Post (prio	-21288	Alive	TUMOR F	3091						Negative	0	3091	0	3091	0	3091	0	3091
TCGA-GM	TCGA-GM	BRCA	54	FEMALE	BLACK OI	Stage I	Infiltrating Lobular C	2006	Post (prio	-19842	Alive	TUMOR F	2596						Negative	0	2596	0	2596	0	2596	0	2596
TCGA-GM	TCGA-GM	BRCA	63	FEMALE	WHITE	Stage IIA	Infiltrating Lobular C	2003	Post (prio	-23041	Alive	TUMOR F	3361						Negative	0	3361	0	3361	0	3361	0	3361
TCGA-GM	TCGA-GM	BRCA	72	FEMALE	WHITE	Stage IIB	Infiltrating Lobular C	2009	Post (prio	-26642	Alive	TUMOR F	1162						Negative	0	1162	0	1162	0	1162	0	1162
TCGA-GM	TCGA-GM	BRCA	46	FEMALE	WHITE	Stage IIIA	Infiltrating Lobular C	2009	Pre (<6 m	-16948	Alive	TUMOR F	1330						Negative	0	1330	0	1330	0	1330	0	1330
TCGA-GM	TCGA-GM	BRCA	49	FEMALE	BLACK OI	Stage IIA	Infiltrating Ductal Ca	2007	Post (prio	-18171	Alive	TUMOR F	2108						Negative	0	2108	0	2108	0	2108	0	2108
TCGA-GM	TCGA-GM	BRCA	44	FEMALE	WHITE	Stage IIIA	Infiltrating Lobular C	2007	Post (prio	-16109	Alive	TUMOR F	2019						Negative	0	2019	0	2019	0	2019	0	2019
TCGA-GM	TCGA-GM	BRCA	67	FEMALE	WHITE	Stage IIIC	Infiltrating Lobular C	2007	Post (prio	-24788	Alive	TUMOR F	2191						Negative	0	2191	0	2191	0	2191	0	2191
TCGA-GM	TCGA-GM	BRCA	63	FEMALE	WHITE	Stage IIB	Infiltrating Lobular C	2011	Post (prio	-23215	Alive	TUMOR F	412						Negative	0	412	0	412	0	412	0	412
TCGA-GM	TCGA-GM	BRCA	65	FEMALE	WHITE	Stage IIB	Infiltrating Lobular C	2011	Post (prio	-23753	Alive	TUMOR F	551						Negative	0	551	0	551	0	551	0	551
TCGA-HN	TCGA-HN	BRCA	56	FEMALE	[Not Evalu	Stage IIA	Infiltrating Lobular Carcinoma		Post (prio	-20483	Alive	TUMOR F	79						Negative	0	79	0	79	0	79	0	79
TCGA-HN	TCGA-HN	BRCA	45	FEMALE	[Not Evalu	Stage IIB	Infiltrating Lobular Carcinoma		Pre (<6 m	-16672	Dead	WITH TUMOR		1900	Distant M	Bone		1543	Negative	1	1900	1	1900	1	1543	1	1543
TCGA-JL-	TCGA-JL-	BRCA	49	FEMALE	ASIAN	Stage IIB	Infiltrating Lobular C	2011	Post (prior bilateral c		Alive		360						Negative	0	360	0	360	0	360	0	360
TCGA-JL-	TCGA-JL-	BRCA	46	FEMALE	ASIAN	Stage IIA	Infiltrating Lobular C	2011	Post (prior bilateral c		Alive		352						Negative	0	352	0	352	0	352	0	352
TCGA-LD	TCGA-LD	BRCA	44	FEMALE	WHITE	Stage IIB	Infiltrating Lobular C	2012	[Not Evalu	-16101	Alive	TUMOR F	646						Negative	0	646	0	646	0	646	0	646
TCGA-LD	TCGA-LD	BRCA	79	FEMALE	WHITE	Stage IIIC	Other, specify	2013		-28981	Alive	WITH TUN	402						Negative	0	402	0	402	0	402	0	402
TCGA-LD	TCGA-LD	BRCA	52	FEMALE	BLACK OI	Stage IIIC	Infiltrating Ductal Ca	2013	Pre (<6 m	-19169	Alive	WITH TUN	216						Positive	0	216	0	216			0	216
TCGA-LD	TCGA-LD	BRCA	54	FEMALE	WHITE	Stage IIB	Other, specify	2013	Post (prio	-20077	Alive	TUMOR F	404						Negative	0	404	0	404	0	404	0	404
TCGA-LD	TCGA-LD	BRCA	73	FEMALE	BLACK OI	Stage IA	Infiltrating Ductal Ca	2013	Post (prio	-26869	Alive	TUMOR F	323						Negative	0	323	0	323	0	323	0	323
TCGA-LL-	TCGA-LL-	BRCA	61	FEMALE	WHITE	Stage IA	Infiltrating Lobular C	2012	Post (prio	-22497	Alive	TUMOR F	759						Negative	0	759	0	759	0	759	0	759
TCGA-LL-	TCGA-LL-	BRCA	62	FEMALE	BLACK OI	Stage IA	Infiltrating Ductal Ca	2012	Post (prio	-22772	Alive	TUMOR F	996						Negative	0	996	0	996	0	996	0	996
TCGA-LL-	TCGA-LL-	BRCA	56	FEMALE	BLACK OI	Stage IIA	Infiltrating Ductal Ca	2012	Post (prio	-20495	Alive	TUMOR F	889						Negative	0	889	0	889	0	889	0	889
TCGA-LL-	TCGA-LL-	BRCA	84	FEMALE	WHITE	Stage IIA	Infiltrating Lobular C	2012	Post (prio	-30934	Alive	TUMOR F	762						Negative	0	762	0	762	0	762	0	762
TCGA-LL-	TCGA-LL-	BRCA	64	FEMALE	WHITE	Stage IIB	Infiltrating Lobular C	2012	Post (prio	-23395	Alive		519						Negative	0	519	0	519	0	519	0	519
TCGA-LL-	TCGA-LL-	BRCA	88	FEMALE	BLACK OI	Stage IIIB	Infiltrating Ductal Ca	2012	Post (prio	-32478	Alive	WITH TUN	466		Locoregic	Other, sp	Skin left c	258	Negative	0	466	0	466	1	258	1	258
TCGA-LL-	TCGA-LL-	BRCA	46	FEMALE	BLACK OI	Stage IIA	Infiltrating Ductal Ca	2013	Post (prio	-16884	Alive	TUMOR F	447						Negative	0	447	0	447	0	447	0	447
TCGA-LL-	TCGA-LL-	BRCA	50	FEMALE	BLACK OI	Stage IA	Infiltrating Ductal Ca	2013	Pre (<6 m	-18354	Alive	TUMOR F	440						Negative	0	440	0	440	0	440	0	440
TCGA-LL-	TCGA-LL-	BRCA	49	FEMALE	BLACK OI	Stage IIB	Infiltrating Ductal Ca	2013	Post (prio	-18041	Alive		450						Negative	0	450	0	450	0	450	0	450
TCGA-LL-	TCGA-LL-	BRCA	90	FEMALE	WHITE	Stage IIA	Infiltrating Lobular C	2012	Post (prio	-32873	Alive	TUMOR F	677						Negative	0	677	0	677	0	677	0	677
TCGA-LL-	TCGA-LL-	BRCA	77	FEMALE	BLACK OI	Stage IIIA	Infiltrating Ductal Ca	2013	Post (prio	-28375	Alive		80						Negative	0	80	0	80	0	80	0	80
TCGA-LL-	TCGA-LL-	BRCA	50	FEMALE	BLACK OI	Stage IIA	Infiltrating Ductal Ca	2013	Post (prio	-18520	Alive	TUMOR F	489						Negative	0	489	0	489	0	489	0	489
TCGA-LL-	TCGA-LL-	BRCA	67	FEMALE	BLACK OI	Stage IA	Infiltrating Ductal Ca	2013	Post (prio	-24647	Alive	TUMOR F	477						Negative	0	477	0	477	0	477	0	477
TCGA-LL-	TCGA-LL-	BRCA	55	FEMALE	BLACK OI	Stage IV	Infiltrating Ductal Ca	2013	Post (prio	-20118	Dead	WITH TUMOR		227	Distant M	Brain		192	Positive	1	227	1	227			1	192
TCGA-LL-	TCGA-LL-	BRCA	61	FEMALE	BLACK OI	Stage IA	Infiltrating Ductal Ca	2013	Post (prio	-22416	Alive	TUMOR F	441						Negative	0	441	0	441	0	441	0	441
TCGA-LL-	TCGA-LL-	BRCA	49	FEMALE	BLACK OI	Stage IIB	Infiltrating Ductal Ca	2013	Pre (<6 m	-18161	Alive	TUMOR F	594						Negative	0	594	0	594	0	594	0	594
TCGA-LL-	TCGA-LL-	BRCA	70	FEMALE	BLACK OI	Stage IIB	Infiltrating Ductal Ca	2013	Post (prio	-25867	Alive	TUMOR F	376						Negative	0	376	0	376	0	376	0	376
TCGA-LL-	TCGA-LL-	BRCA	61	FEMALE	BLACK OI	Stage IIA	Infiltrating Ductal Ca	2013	Post (prio	-22544	Alive	TUMOR F	596						Negative	0	596	0	596	0	596	0	596
TCGA-LL-	TCGA-LL-	BRCA	69	FEMALE	WHITE	Stage IIIC	Infiltrating Lobular C	2013	Post (prio	-25477	Alive	TUMOR F	532						Negative	0	532	0	532	0	532	0	532
TCGA-LO	TCGA-LO	BRCA	73	FEMALE	WHITE	Stage IIIA	Infiltrating Lobular C	2011	Post (prio	-26883	Alive	WITH TUN	849		Distant M	Bone		681	Positive	0	849	0	849			1	681
TCGA-MS	TCGA-MS	BRCA	44	FEMALE	WHITE	Stage IIB	Infiltrating Lobular C	2012		-16213	Alive	TUMOR F	681						Negative	0	681	0	681	0	681	0	681
TCGA-OK	TCGA-OK	BRCA	59	FEMALE	[Not Evalu	Stage II	Infiltrating Lobular C	2012	[Unknown	-21555	Alive		64						Negative	0	64	0	64	0	64	0	64
TCGA-OL	TCGA-OL	BRCA	71	FEMALE	BLACK OI	Stage IIA	Infiltrating Ductal Ca	2009	Post (prio	-26052	Dead	TUMOR FREE		1104					Negative	1	1104	0	1104	0	1104	0	1104
TCGA-OL	TCGA-OL	BRCA	70	FEMALE	BLACK OI	Stage IIA	Infiltrating Ductal Ca	2009	Post (prio	-25643	Alive	TUMOR F	1780						Negative	0	1780	0	1780	0	1780	0	1780
TCGA-OL	TCGA-OL	BRCA	40	FEMALE	BLACK OI	Stage IIB	Infiltrating Ductal Ca	2009	Pre (<6 m	-14853	Alive	TUMOR F	973						[Unknown]	0	973	0	973			0	973
TCGA-OL	TCGA-OL	BRCA	61	FEMALE	WHITE	Stage IIA	Infiltrating Lobular C	2009	Post (prio	-22411	Alive	TUMOR F	1783						Negative	0	1783	0	1783	0	1783	0	1783
TCGA-OL	TCGA-OL	BRCA	63	FEMALE	BLACK OI	Stage IIA	Infiltrating Ductal Ca	2010	Post (prio	-23108	Alive	TUMOR F	1219						Negative	0	1219	0	1219	0	1219	0	1219
TCGA-OL	TCGA-OL	BRCA	43	FEMALE	BLACK OI	Stage IIIC	Infiltrating Ductal Ca	2011	Pre (<6 m	-15820	Alive	TUMOR F	1062						Negative	0	1062	0	1062	0	1062	0	1062
TCGA-OL	TCGA-OL	BRCA	40	FEMALE	BLACK OI	Stage IIA	Infiltrating Ductal Ca	2010	Pre (<6 m	-14638	Alive	TUMOR F	1106						Negative	0	1106	0	1106	0	1106	0	1106
TCGA-OL	TCGA-OL	BRCA	51	FEMALE	BLACK OI	Stage IA	Infiltrating Ductal Ca	2011	Pre (<6 m	-18942	Alive	TUMOR F	878						Negative	0	878	0	878	0	878	0	878
TCGA-OL	TCGA-OL	BRCA	52	FEMALE	BLACK OI	Stage IIA	Infiltrating Ductal Ca	2011	Post (prio	-19216	Alive	TUMOR F	752						Negative	0	752	0	752	0	752	0	752
TCGA-OL	TCGA-OL	BRCA	57	FEMALE	BLACK OI	Stage IA	Infiltrating Ductal Ca	2012	Post (prio	-21038	Alive	TUMOR F	679						Negative	0	679	0	679	0	679	0	679
TCGA-OL	TCGA-OL	BRCA	66	FEMALE	BLACK OI	Stage IIB	Infiltrating Ductal Ca	2012	Post (prio	-24139	Alive	TUMOR F	620						Negative	0	620	0	620	0	620	0	620
TCGA-OL	TCGA-OL	BRCA		FEMALE	BLACK OI	Stage IB	Mucinous Carcinoma		Post (prior bilateral c		Alive	TUMOR FREE							Negative	0		0		0		0	
TCGA-OL	TCGA-OL	BRCA	36	FEMALE	BLACK OI	Stage IIA	Infiltrating Ductal Ca	2008	[Not Evalu	-13458	Alive	TUMOR F	714						Negative	0	714	0	714	0	714	0	714
TCGA-OL	TCGA-OL	BRCA	80	FEMALE	WHITE	Stage I	Infiltrating Lobular C	2008	Post (prio	-29345	Alive	TUMOR F	1996						Negative	0	1996	0	1996	0	1996	0	1996
TCGA-OL	TCGA-OL	BRCA	72	FEMALE	WHITE	Stage IIA	Infiltrating Lobular C	2010	Post (prio	-26299	Dead	TUMOR FREE		1275					Negative	1	1275	0	1275	0	1275	0	1275
TCGA-OL	TCGA-OL	BRCA	71	FEMALE	BLACK OI	Stage IA	Infiltrating Ductal Ca	2010	Post (prio	-26186	Alive	TUMOR F	1301						Negative	0	1301	0	1301	0	1301	0	1301
TCGA-OL	TCGA-OL	BRCA	59	FEMALE	WHITE	Stage IIIC	Infiltrating Lobular C	2011	Post (prio	-21608	Alive	TUMOR F	792						Negative	0	792	0	792	0	792	0	792
TCGA-OL	TCGA-OL	BRCA	39	FEMALE	BLACK OI	Stage IIB	Infiltrating Ductal Ca	2012	Pre (<6 m	-14272	Alive	TUMOR F	528						Negative	0	528	0	528	0	528	0	528
TCGA-OL	TCGA-OL	BRCA	75	FEMALE	BLACK OI	Stage IIA	Infiltrating Ductal Ca	2013		-27489	Alive	TUMOR F	428						Negative	0	428	0	428	0	428	0	428
TCGA-OL	TCGA-OL	BRCA	43	FEMALE	BLACK OI	Stage IA	Infiltrating Ductal Ca	2012	[Unknown	-15780	Alive	TUMOR F	858						Negative	0	858	0	858	0	858	0	858
TCGA-OL	TCGA-OL	BRCA	49	FEMALE	WHITE	Stage IIA	Infiltrating Lobular C	2012	Pre (<6 m	-18151	Alive	TUMOR F	600						Negative	0	600	0	600	0	600	0	600
TCGA-OL	TCGA-OL	BRCA	48	FEMALE	BLACK OI	Stage IA	Infiltrating Ductal Ca	2011	[Unknown	-17702	Alive	TUMOR F	1220						Negative	0	1220	0	1220	0	1220	0	1220
TCGA-OL	TCGA-OL	BRCA	67	FEMALE	BLACK OI	Stage IIB	Other, specify	2013	Post (prio	-24735	Alive	TUMOR F	271		Distant M	Lung		275		0	271	0	271			1	275
TCGA-PE	TCGA-PE	BRCA	72	FEMALE	WHITE	Stage IIIA	Infiltrating Lobular C	2005	Post (prio	-26514	Dead	TUMOR FREE		1430						1	1430	0	1430			0	1430

TCGA-PE-	TCGA-PE-	BRCA	64	FEMALE	ASIAN	Stage IIA		Infiltrating Lobular C		2006	Post (prio	-23686	Alive	TUMOR F	1953								Negative	0	1953	0	1953	0	1953	0	1953
TCGA-PE-	TCGA-PE-	BRCA	41	FEMALE	WHITE	Stage IIA		Infiltrating Lobular C		2007	Pre (<6 m	-15216	Alive	TUMOR F	2645									0	2645	0	2645			0	2645
TCGA-PL-	TCGA-PL-	BRCA	54	FEMALE	BLACK OI	Stage IIIB		Medullary Carcinom		2013	[Unknowr	-19746	Alive	WITH TUN	-7									0	0	0	0			0	0
TCGA-PL-	TCGA-PL-	BRCA	35	FEMALE	BLACK OI	Stage IV		Infiltrating Ductal Ca		2013	Pre (<6 m	-12840	Alive	WITH TUN	5									0	5	0	5			0	5
TCGA-PL-	TCGA-PL-	BRCA	30	FEMALE	BLACK OI	Stage IIB		Infiltrating Ductal Ca		2013	Pre (<6 m	-11199	Alive	WITH TUN	8									0	8	0	8			0	8
TCGA-PL-	TCGA-PL-	BRCA	29	FEMALE	BLACK OI	Stage IIIB		Infiltrating Ductal Ca		2013	Pre (<6 m	-10869	Alive	TUMOR F	302									0	302	0	302			0	302
TCGA-S3-	TCGA-S3-	BRCA	64	FEMALE	BLACK OI	Stage IIA		Infiltrating Ductal Ca		2013	Post (prio	-23650	Alive	TUMOR F	572								Negative	0	572	0	572	0	572	0	572
TCGA-S3-	TCGA-S3-	BRCA	71	FEMALE	BLACK OI	Stage IIB		Mixed Histology (ple		2013	Post (prio	-26141	Alive	TUMOR F	562								Negative	0	562	0	562	0	562	0	562
TCGA-S3-	TCGA-S3-	BRCA	29	FEMALE	BLACK OI	Stage IIIC		Infiltrating Ductal Ca		2013	[Unknowr	-10832	Alive	TUMOR F	641								Negative	0	641	0	641	0	641	0	641
TCGA-S3-	TCGA-S3-	BRCA	63	FEMALE	BLACK OI	Stage IIB		Infiltrating Ductal Ca		2013	Post (prio	-23314	Alive	TUMOR F	629								Negative	0	629	0	629	0	629	0	629
TCGA-S3-	TCGA-S3-	BRCA	65	FEMALE	BLACK OI	Stage IIA		Infiltrating Ductal Ca		2013	Post (prio	-24075	Alive	TUMOR F	586								Negative	0	586	0	586	0	586	0	586
TCGA-S3-	TCGA-S3-	BRCA	67	FEMALE	BLACK OI	Stage IIA		Infiltrating Ductal Ca		2013	Post (prio	-24813	Alive	TUMOR F	421								Negative	0	421	0	421	0	421	0	421
TCGA-S3-	TCGA-S3-	BRCA	82	FEMALE	BLACK OI	Stage IIIA		Infiltrating Ductal Ca		2013	Post (prio	-30101	Alive	TUMOR F	574								Negative	0	574	0	574	0	574	0	574
TCGA-S3-	TCGA-S3-	BRCA	47	FEMALE	BLACK OI	Stage I		Infiltrating Ductal Ca		2013	Post (prio	-17352	Alive	TUMOR F	529								Negative	0	529	0	529	0	529	0	529
TCGA-S3-	TCGA-S3-	BRCA	51	FEMALE	BLACK OI	Stage IIB		Infiltrating Ductal Ca		2013	Post (prio	-18659	Alive	TUMOR F	525								Negative	0	525	0	525	0	525	0	525
TCGA-S3-	TCGA-S3-	BRCA	64	FEMALE	BLACK OI	Stage IIB		Infiltrating Ductal Ca		2013	Post (prio	-23642	Alive	TUMOR F	424								Negative	0	424	0	424	0	424	0	424
TCGA-UL	TCGA-UL	BRCA	73	FEMALE	BLACK OI	Stage IIA		Infiltrating Ductal Ca		2013	Post (prio	-26999	Alive	TUMOR F	518									0	518	0	518			0	518
TCGA-UU	TCGA-UU	BRCA	63	FEMALE	BLACK OI	Stage IV		Infiltrating Ductal Ca		2013	Post (prio	-23278	Dead	WITH TUMOR		116							Positive	1	116	1	116			1	116
TCGA-V7	TCGA-V7	BRCA	75	FEMALE	BLACK OI	Stage IIIA		Infiltrating Ductal Ca		2009	Post (prio	-27684	Alive	TUMOR F	2033								Negative	0	2033	0	2033	0	2033	0	2033
TCGA-W8	TCGA-W8	BRCA	66	FEMALE	WHITE	Stage IIA		Infiltrating Lobular C		2013	Post (prio	-24125	Alive	TUMOR F	347								Negative	0	347	0	347	0	347	0	347
TCGA-WT	TCGA-WT	BRCA	55	FEMALE	BLACK OI	Stage IIB		Infiltrating Ductal Ca		2010	Post (prior bilateral c		Alive	TUMOR F	1611								Negative	0	1611	0	1611	0	1611	0	1611
TCGA-WT	TCGA-WT	BRCA	77	FEMALE	WHITE	Stage IA		Infiltrating Lobular C		2012	Post (prior bilateral c		Alive	TUMOR F	883								Negative	0	883	0	883	0	883	0	883
TCGA-XX	TCGA-XX	BRCA	46	FEMALE	WHITE	Stage IIIA		Infiltrating Lobular C		2013	Post (prio	-17022	Alive	TUMOR F	467								Negative	0	467	0	467	0	467	0	467
TCGA-XX	TCGA-XX	BRCA	68	FEMALE	WHITE	Stage IIB		Infiltrating Lobular C		2013	Post (prio	-25000	Alive	TUMOR F	488								Negative	0	488	0	488	0	488	0	488
TCGA-Z7-	TCGA-Z7-	BRCA	61	FEMALE	WHITE	Stage IIIA		Infiltrating Lobular C		2005	Post (prio	-22280	Alive	WITH TUN	3287			Locoregional Recurrence			181		Positive	0	3287	0	3287			1	181
TCGA-Z7-	TCGA-Z7-	BRCA	46	FEMALE	WHITE	Stage I		Infiltrating Lobular C		2005	Pre (<6 m	-16955	Alive	TUMOR F	3256								Negative	0	3256	0	3256	0	3256	0	3256
TCGA-2W	TCGA-2W	CESC	51	FEMALE	WHITE		Stage IB2	Adenosqu	G3	2013	Pre (<6 m	-18886	Alive	TUMOR F	533							Complete Remission/Response		0	533	0	533	0	533	0	533
TCGA-4J-	TCGA-4J-	CESC	31	FEMALE	WHITE		Stage IB2	Cervical S	G3	2013	Pre (<6 m	-11611	Alive	TUMOR F	542							Complete Remission/Response		0	542	0	542	0	542	0	542
TCGA-BI-	TCGA-BI-	CESC	53	FEMALE	WHITE		Stage IIIB	Cervical S	G3	2006	Post (prio	-19473	Alive	TUMOR F	1505									0	1505	0	1505			0	1505
TCGA-BI-	TCGA-BI-	CESC	48	FEMALE	WHITE		Stage IB	Cervical S	G3	2007	Pre (<6 m	-17839	Alive	TUMOR F	1735							Complete Remission/Response		0	1735	0	1735	0	1735	0	1735
TCGA-BI-	TCGA-BI-	CESC	49	FEMALE	WHITE		Stage IB1	Cervical S	G3	2010		-18215	Alive	TUMOR F	720							Complete Remission/Response		0	720	0	720	0	720	0	720
TCGA-C5	TCGA-C5	CESC	21	FEMALE	BLACK OR AFRICAN		Stage IB2	Cervical S	G3	1997	Pre (<6 m	-7760	Dead	WITH TUMOR		348	Cervical C	Distant M	Other, sp	Pelvis/Live	62	Progressive Disease		1	348	1	348			1	62
TCGA-C5	TCGA-C5	CESC	64	FEMALE	WHITE		Stage IB2	Cervical S	G2	2000	Post (prio	-23727	Dead	WITH TUMOR		2094	Cervical Cancer				1674	Complete Remission/Response		1	2094	1	2094	1	1674	1	1674
TCGA-C5	TCGA-C5	CESC	46	FEMALE	WHITE		Stage IB	Cervical S	G1	1997		-16975	Dead	WITH TUMOR		570	Cervical C	Distant M	Other, sp	Lung, Ster	93	Complete Remission/Response		1	570	1	570	1	93	1	93
TCGA-C5	TCGA-C5	CESC	31	FEMALE	WHITE		Stage IIIB	Cervical S	G2	2001	Pre (<6 m	-11564	Alive	TUMOR F	1112							Complete Remission/Response		0	1112	0	1112	0	1112	0	1112
TCGA-C5	TCGA-C5	CESC	34	FEMALE	WHITE		Stage IIB	Cervical S	G2	1997	Pre (<6 m	-12611	Alive	TUMOR F	4385							Complete Remission/Response		0	4385	0	4385	0	4385	0	4385
TCGA-C5	TCGA-C5	CESC	36	FEMALE	BLACK OR AFRICAN		Stage IB	Cervical S	G2	1994	Pre (<6 m	-13297	Alive	TUMOR F	5385							Complete Remission/Response		0	5385	0	5385	0	5385	0	5385
TCGA-C5	TCGA-C5	CESC	32	FEMALE	WHITE		Stage IB	Cervical S	G2	1994	Pre (<6 m	-11723	Alive	TUMOR F	5271							Complete Remission/Response		0	5271	0	5271	0	5271	0	5271
TCGA-C5	TCGA-C5	CESC	78	FEMALE	BLACK OR AFRICAN		Stage IB	Cervical S	G2	1997	Post (prio	-28815	Dead	TUMOR FREE		2520						Complete Remission/Response		1	2520	0	2520	0	2520	0	2520
TCGA-C5	TCGA-C5	CESC	26	FEMALE	WHITE		Stage IB2	Cervical S	G3	2007	Pre (<6 m	-9783	Dead	WITH TUMOR		166	Cervical Cancer					Progressive Disease		1	166	1	166			1	166
TCGA-C5	TCGA-C5	CESC	65	FEMALE	WHITE		Stage IIIB	Cervical S	G2	2002	Post (prio	-24018	Dead	TUMOR FREE		604	Cervical Cancer					Partial Remission/Response		1	604	1	604			0	604
TCGA-C5	TCGA-C5	CESC	53	FEMALE	WHITE		Stage IB	Cervical S	G2	1996	Indetermi	-19672	Dead	WITH TUMOR		2052	Cervical C	Distant M	Other, sp	Regional (	693	Complete Remission/Response		1	2052	1	2052	1	693	1	693
TCGA-C5	TCGA-C5	CESC	55	FEMALE	WHITE		Stage IIB	Cervical S	G3	1998	Post (prio	-20090	Dead	WITH TUMOR		955	Cervical C	Distant M	Lung		316	Complete Remission/Response		1	955	1	955	1	316	1	316
TCGA-C5	TCGA-C5	CESC	37	FEMALE	WHITE		Stage IB	Cervical S	G2	1999	Pre (<6 m	-13808	Alive	TUMOR F	1409							Complete Remission/Response		0	1409	0	1409	0	1409	0	1409
TCGA-C5	TCGA-C5	CESC	43	FEMALE	WHITE		Stage IB1	Cervical S	G2	2001	Indetermi	-15848	Alive	TUMOR F	919							Complete Remission/Response		0	919	0	919	0	919	0	919
TCGA-C5	TCGA-C5	CESC	46	FEMALE	WHITE		Stage IB1	Endocervi	G3	2002	Pre (<6 m	-17020	Dead	WITH TUMOR		1065	Cervical C	Distant M	Lung		555	Complete Remission/Response		1	1065	1	1065	1	555	1	555
TCGA-C5	TCGA-C5	CESC	40	FEMALE	WHITE		Stage IB1	Endocervi	G1	2003	Pre (<6 m	-14970	Alive	TUMOR F	1756							Complete Remission/Response		0	1756	0	1756	0	1756	0	1756
TCGA-C5	TCGA-C5	CESC	49	FEMALE	WHITE		Stage IB2	Cervical S	G3	2004	Pre (<6 m	-17911	Alive	TUMOR F	1617							Complete Remission/Response		0	1617	0	1617	0	1617	0	1617
TCGA-C5	TCGA-C5	CESC	71	FEMALE	WHITE		Stage IIIB	Cervical S	G3	2004	Post (prio	-26234	Dead	WITH TUMOR		1186	Cervical C	Distant M	Lung		178	Complete Remission/Response		1	1186	1	1186	1	178	1	178
TCGA-C5	TCGA-C5	CESC	50	FEMALE	WHITE		Stage IB1	Cervical S	G2	2004	Peri (6-12	-18336	Dead	WITH TUMOR		1083	Cervical C	Distant M	Other, sp	distant re	805	Complete Remission/Response		1	1083	1	1083	1	805	1	805
TCGA-C5	TCGA-C5	CESC	61	FEMALE	WHITE		Stage IB1	Endocervi	G2	2005	Post (prio	-22308	Dead	TUMOR FREE		14								1	14	0	14			0	14
TCGA-C5	TCGA-C5	CESC	79	FEMALE	WHITE		Stage IIIB	Cervical S	G3	2005	Post (prio	-28939	Dead	WITH TUMOR		74	Cervical Cancer					Progressive Disease		1	74	1	74			1	74
TCGA-C5	TCGA-C5	CESC	49	FEMALE	WHITE		Stage IB2	Cervical S	G3	2005	Post (prio	-18067	Dead	WITH TUMOR		636	Cervical Cancer					Progressive Disease		1	636	1	636			1	636
TCGA-C5	TCGA-C5	CESC	42	FEMALE	WHITE		Stage IIIB	Cervical S	G2	2007	Pre (<6 m	-15499	Dead	TUMOR FREE		1245						Complete Remission/Response		1	1245	0	1245	0	1245	0	1245
TCGA-C5	TCGA-C5	CESC	34	FEMALE	BLACK OR AFRICAN		Stage IB2	Cervical S	G3	2009	Pre (<6 m	-12692	Alive	TUMOR F	109							Complete Remission/Response		0	109	0	109	0	109	0	109
TCGA-C5	TCGA-C5	CESC	35	FEMALE	WHITE		Stage IIA	Cervical S	G2	2009		-12836	Alive	TUMOR F	1031							Complete Remission/Response		0	1031	0	1031	0	1031	0	1031
TCGA-C5	TCGA-C5	CESC	37	FEMALE	WHITE		Stage IB2	Endocervi	G1	2005		-13857	Alive	TUMOR F	1345							Complete Remission/Response		0	1345	0	1345	0	1345	0	1345
TCGA-C5	TCGA-C5	CESC	38	FEMALE	WHITE		Stage IB	Cervical S	G3	2001	Pre (<6 m	-13966	Alive	TUMOR F	2226							Complete Remission/Response		0	2226	0	2226	0	2226	0	2226
TCGA-C5	TCGA-C5	CESC	36	FEMALE	BLACK OR AFRICAN		Stage IB	Cervical S	G3	2003	Pre (<6 m	-13447	Alive	TUMOR F	2234							Complete Remission/Response		0	2234	0	2234	0	2234	0	2234
TCGA-C5	TCGA-C5	CESC	54	FEMALE	WHITE		Stage IB1	Cervical S	G2	2007	Post (prio	-20070	Alive	TUMOR F	2526							Complete Remission/Response		0	2526	0	2526	0	2526	0	2526
TCGA-C5	TCGA-C5	CESC	30	FEMALE	WHITE		Stage IB1	Cervical S	G2	2002	Pre (<6 m	-11018	Alive	TUMOR F	2383							Complete Remission/Response		0	2383	0	2383	0	2383	0	2383
TCGA-C5	TCGA-C5	CESC	65	FEMALE	WHITE		Stage IIIB	Cervical S	G2	1998	Post (prio	-24059	Dead	WITH TUMOR		3046	Cervical C	Distant Metastasis			2893	Complete Remission/Response		1	3046	1	3046	1	2893	1	2893
TCGA-C5	TCGA-C5	CESC	37	FEMALE	WHITE		Stage IB1	Endocervi	G2	2005	Pre (<6 m	-13835	Alive	TUMOR F	1169							Complete Remission/Response		0	1169	0	1169	0	1169	0	1169
TCGA-C5	TCGA-C5	CESC	56	FEMALE	WHITE		Stage IB2	Endocervi	G2	2009	Post (prio	-20480	Dead			1011								1	1011		1011			0	1011
TCGA-C5	TCGA-C5	CESC	51	FEMALE	WHITE		Stage IIB	Cervical S	G2	2004		-18690	Alive	TUMOR F	1582							Complete Remission/Response		0	1582	0	1582	0	1582	0	1582
TCGA-C5	TCGA-C5	CESC	44	FEMALE	WHITE		Stage IB2	Cervical S	G3	2006		-16139	Alive		548			Locoregic	Cervix		252			0	548	0	548			1	252
TCGA-C5	TCGA-C5	CESC	24	FEMALE	WHITE		Stage IB2	Mucinous	G2	1996	Pre (<6 m	-9115	Dead	WITH TUMOR		543	Cervical C	Distant M	Other, sp	Pelvis	450			1	543	1	543			1	450
TCGA-C5	TCGA-C5	CESC	76	FEMALE	WHITE		Stage IB2	Cervical S	G2	2007	Post (prio	-27963	Alive	TUMOR F	621							Complete Remission/Response		0	621	0	621	0	621	0	621
TCGA-C5	TCGA-C5	CESC	55	FEMALE	WHITE		Stage IB	Cervical S	G2	1997		-20242	Alive	TUMOR F	6408									0	6408	0	6408			0	6408
TCGA-C5	TCGA-C5	CESC	43	FEMALE	WHITE		Stage IIB	Cervical S	G2	2001		-15777	Alive	TUMOR F	4694									0	4694	0	4694			0	4694
TCGA-C5	TCGA-C5	CESC	42	FEMALE	WHITE		Stage IIA	Cervical S	G2	2002		-15652	Dead	TUMOR FREE		3097								1	3097	0	3097			0	3097
TCGA-C5	TCGA-C5	CESC	58	FEMALE	WHITE		Stage IVA	Cervical S	G2	2001		-21520	Dead	TUMOR FREE		4086								1	4086	0	4086			0	4086
TCGA-C5	TCGA-C5	CESC	48	FEMALE	WHITE		Stage IIIB	Cervical S	G2	2002		-17869	Dead	WITH TUMOR		471	Cervical Cancer							1	471	1	471			1	471
TCGA-C5	TCGA-C5	CESC	35	FEMALE	WHITE		Stage IB2	Endocervi	G2	2012		-12853	Alive	TUMOR F	619							Complete Remission/Response		0	619	0	619	0	619	0	619
TCGA-C5	TCGA-C5	CESC	68	FEMALE	WHITE		Stage IB2	Cervical S	G2	2002	Post (prio	-25045	Alive	TUMOR F	4482							Complete Remission/Response		0	4482	0	4482	0	4482	0	4482
TCGA-C5	TCGA-C5	CESC	48	FEMALE	WHITE		Stage IB	Cervical S	G3	2002		-17765	Dead	TUMOR FREE		523								1	523	0	523			0	523
TCGA-C5	TCGA-C5	CESC	45	FEMALE	WHITE		Stage IB1	Cervical S	G2	2001		-16473	Alive	TUMOR F	4738							Complete Remission/Response		0	4738	0	4738	0	4738	0	4738
TCGA-C5	TCGA-C5	CESC	55	FEMALE	WHITE		Stage IIIB	Cervical S	G3	2003		-20189	Alive	TUMOR F	3988									0	3988	0	3988			0	3988
TCGA-C5	TCGA-C5	CESC	42	FEMALE	BLACK OR AFRICAN		Stage IB1	Cervical S	G3	2004		-15349	Dead	TUMOR FREE		2888								1	2888	0	2888			0	2888
TCGA-C5	TCGA-C5	CESC	70	FEMALE	WHITE		Stage IIIB	Cervical S	G2	1997		-25665	Dead	TUMOR FREE		284								1	284	0	284			0	284
TCGA-C5	TCGA-C5	CESC	72	FEMALE	BLACK OR AFRICAN		Stage IVB	Cervical S	G3	2003		-26542	Dead	WITH TUMOR		414	Cervical Cancer							1	414	1	414			1	414
TCGA-C5	TCGA-C5	CESC	35	FEMALE	WHITE		Stage IB1	Mucinous	G2	2003		-12977	Alive	TUMOR F	83									0	83	0	83			0	83
TCGA-C5	TCGA-C5	CESC	26	FEMALE	WHITE		Stage IB1	Cervical S	G2	2004		-9776	Alive	TUMOR F	1551									0	1551	0	1551			0	1551
TCGA-C5	TCGA-C5	CESC	39	FEMALE	WHITE		Stage IB1	Cervical Squamous C		2007		-14444	Dead	TUMOR FREE		1394								1	1394	0	1394			0	1394
TCGA-C5	TCGA-C5	CESC	69	FEMALE	BLACK OR AFRICAN		Stage IB2	Cervical S	G3	2008	Post (prio	-25220	Alive	TUMOR F	254									0	254	0	254			0	254
TCGA-C5	TCGA-C5	CESC	74	FEMALE	WHITE		Stage IB	Cervical Squamous C		2002		-27087	Alive	TUMOR F	4467									0	4467	0	4467			0	4467
TCGA-C5	TCGA-C5	CESC	30	FEMALE	BLACK OR AFRICAN AMERICAN			Cervical S	G3	2000		-11268	Alive	TUMOR F	3039									0	3039	0	3039			0	3039
TCGA-C5	TCGA-C5	CESC	79	FEMALE	WHITE		Stage IB1	Cervical S	G2	2006	Post (prio	-28873	Dead	WITH TUMOR		715	Cervical Cancer							1	715	1	715			1	715
TCGA-C5	TCGA-C5	CESC	56	FEMALE	WHITE		Stage IB	Cervical S	G3	1994		-20672	Dead			837		Locoregional Recurre	regional r		272			1	837		837			1	272
TCGA-C5	TCGA-C5	CESC	36	FEMALE	WHITE		Stage IB1	Cervical S	G3	2008		-13253	Dead	WITH TUMOR		633								1	633	1	633			1	633
TCGA-C5	TCGA-C5	CESC	41	FEMALE	WHITE		Stage IIB	Cervical S	G2	2005	Pre (<6 m	-15262	Alive	TUMOR F	636									0	636	0	636			0	636
TCGA-C5	TCGA-C5	CESC	44	FEMALE	WHITE			Cervical S	G2	2000		-16186	Alive	TUMOR F	518									0	518	0	518			0	518
TCGA-C5	TCGA-C5	CESC	35	FEMALE	BLACK OR AFRICAN		Stage IB2	Cervical S	G3	2008		-12959	Alive	TUMOR F	149									0	149	0	149			0	149
TCGA-C5	TCGA-C5	CESC	37	FEMALE	BLACK OR AFRICAN		Stage IB	Cervical S	G2	1994		-13584	Alive	TUMOR F	4879									0	4879	0	4879			0	4879
TCGA-C5	TCGA-C5	CESC	47	FEMALE	WHITE		Stage IB2	Cervical S	G2	2013		-17304	Alive	TUMOR F	448									0	448	0	448			0	448
TCGA-DG	TCGA-DG	CESC	25	FEMALE	[Not Evaluated]		Stage IB1	Adenosqu	GX		Pre (<6 m	-9209	Alive		34							[Unknown]		0	34	0	34			0	34
TCGA-DG	TCGA-DG	CESC	50	FEMALE	[Not Evaluated]		Stage IIIB	Cervical S	G3	2006	[Unknowr	-18453	Alive	TUMOR F	2893							Complete Remission/Response		0	2893	0	2893	0	2893	0	2893
TCGA-DG	TCGA-DG	CESC	51	FEMALE	[Not Evaluated]		Stage IIIB	Cervical S	G3	2007	Post (prio	-18834	Alive	TUMOR F	2496							Complete Remission/Response		0	2496	0	2496	0	2496	0	2496
TCGA-DG	TCGA-DG	CESC	53	FEMALE	[Not Evaluated]		Stage IIA	Cervical S	G1		Post (prio	-19539	Alive	TUMOR F	1367							Complete Remission/Response		0	1367	0	1367	0	1367	0	1367
TCGA-DG	TCGA-DG	CESC	46	FEMALE	[Not Evaluated]		Stage IB1	Cervical S	G2	2008	[Unknowr	-16808	Alive	TUMOR F	1946							Complete Remission/Response		0	1946	0	1946	0	1946	0	1946
TCGA-DR	TCGA-DR	CESC	53	FEMALE	ASIAN		Stage IB	Cervical S	G3	2004	Post (prio	-19384	Alive	TUMOR F	2669							Complete Remission/Response		0	2669	0	2669	0	2669	0	2669
TCGA-DR	TCGA-DR	CESC	61	FEMALE	WHITE		Stage IIIB	Cervical S	G2	2006	Post (prio	-22464	Alive	TUMOR F	1791							Complete Remission/Response		0	1791	0	1791	0	1791	0	1791
TCGA-DS	TCGA-DS	CESC	45	FEMALE	WHITE		Stage IB	Cervical S	G3	1996	Pre (<6 m	-16792	Dead	WITH TUMOR		1118		Distant M	Other, sp	Vagina	209	Progressive Disease		1	1118	1	1118			1	209
TCGA-DS	TCGA-DS	CESC	25	FEMALE	WHITE		Stage IB	Cervical S	G2	2001	Pre (<6 m	-9186	Alive	TUMOR F	81							Complete Remission/Response		0	81	0	81	0	81	0	81
TCGA-DS	TCGA-DS	CESC	51	FEMALE	WHITE		Stage IB	Cervical S	G3	2003	Post (prio	-18839	Alive	TUMOR F	3589			Distant M	Lung		868	Complete Remission/Response		0	3589	0	3589	1	868	1	868
TCGA-DS	TCGA-DS	CESC	47	FEMALE	WHITE		Stage IB	Cervical S	G2	2002	Pre (<6 m	-17433	Alive	TUMOR F	3609							Complete Remission/Response		0	3609	0	3609	0	3609	0	3609
TCGA-DS	TCGA-DS	CESC	75	FEMALE	WHITE		Stage IVA	Cervical S	G3	1999	Post (prio	-27433	Dead	WITH TUN	266	266	Cervical Cancer							1	266	1	266			1	266
TCGA-DS	TCGA-DS	CESC	77	FEMALE	ASIAN		Stage IB	Cervical S	G3	2001	Post (prio	-28160	Dead	WITH TUN	879	879	Cervical C	Locoregional Recurrence			379			1	879	1	879			1	379
TCGA-DS	TCGA-DS	CESC	45	FEMALE	WHITE		Stage IB	Cervical S	G2	2007	Pre (<6 m	-16685	Dead	WITH TUMOR		861	Cervical C	Distant M	Head & Neck		652	Complete Remission/Response		1	861	1	861	1	652	1	652
TCGA-DS	TCGA-DS	CESC	47	FEMALE	WHITE		Stage IB	Cervical S	G2	2010	Pre (<6 m	-17357	Alive	TUMOR F	376							Complete Remission/Response		0	376	0	376	0	376	0	376
TCGA-DS	TCGA-DS	CESC	49	FEMALE	WHITE		Stage IB1	Cervical S	G3	2003	Pre (<6 m	-17954	Alive	TUMOR F	3874							Complete Remission/Response		0	3874	0	3874	0	3874	0	3874
TCGA-DS	TCGA-DS	CESC	46	FEMALE			Stage IIIB	Cervical S	G3	2011	Pre (<6 m	-16861	Alive	TUMOR F	699							Complete Remission/Response		0	699	0	699	0	699	0	699
TCGA-DS	TCGA-DS	CESC	80	FEMALE	WHITE		Stage IB1	Cervical S	G2	2012	Post (prio	-29526	Alive	TUMOR F	512							Complete Remission/Response		0	512	0	512	0	512	0	512
TCGA-DS	TCGA-DS	CESC	41	FEMALE	[Unknown]		Stage IB2	Adenosqu	G3	2006	Pre (<6 m	-15319	Dead	WITH TUMOR		492	Cervical C	Distant M	Lung		321	Partial Remission/Response		1	492	1	492			1	321
TCGA-DS	TCGA-DS	CESC	34	FEMALE	WHITE		Stage IB1	Mucinous	G2	2013	Pre (<6 m	-12644	Alive	TUMOR F	533							Complete Remission/Response		0	533	0	533	0	533	0	533
TCGA-DS	TCGA-DS	CESC	43	FEMALE	WHITE		Stage IIA2	Cervical S	G2	2013	Pre (<6 m	-15970	Dead	WITH TUMOR		252	Cervical C	Locoregic	Other, sp	Retroperit	206	Complete Remission/Response		1	252	1	252	1	206	1	206
TCGA-EA	TCGA-EA	CESC	46	FEMALE	WHITE		Stage IB1	Cervical S	G2	2010	Post (prio	-16923	Alive	TUMOR F	1203							Complete Remission/Response		0	1203	0	1203	0	1203	0	1203
TCGA-EA	TCGA-EA	CESC	47	FEMALE	WHITE		Stage IB	Cervical S	G2	2011	Post (prio	-17475	Alive	TUMOR F	1243							Complete Remission/Response		0	1243	0	1243	0	1243	0	1243
TCGA-EA	TCGA-EA	CESC	60	FEMALE	WHITE		Stage II	Cervical S	G2	2011	Post (prio	-21966	Alive	TUMOR F	1136							Complete Remission/Response		0	1136	0	1136	0	1136	0	1136
TCGA-EA	TCGA-EA	CESC	57	FEMALE	WHITE		Stage II	Cervical S	G2	2011	Post (prio	-20980	Alive	TUMOR F	940							Complete Remission/Response		0	940	0	940	0	940	0	940
TCGA-EA	TCGA-EA	CESC	35	FEMALE	WHITE		Stage IB	Cervical S	G1	2011	Pre (<6 m	-13117	Alive	TUMOR F	959							Complete Remission/Response		0	959	0	959	0	959	0	959
TCGA-EA	TCGA-EA	CESC	68	FEMALE	WHITE		Stage IB	Cervical S	G1	2011	Post (prio	-25138	Alive	TUMOR F	954							Complete Remission/Response		0	954	0	954	0	954	0	954
TCGA-EA	TCGA-EA	CESC	43	FEMALE	WHITE		Stage II	Cervical S	G2	2011	Pre (<6 m	-15909	Alive	TUMOR F	1013							Complete Remission/Response		0	1013	0	1013	0	1013	0	1013
TCGA-EA	TCGA-EA	CESC	59	FEMALE	WHITE		Stage IIIB	Cervical S	G3	2011	Post (prio	-21655	Alive	TUMOR F	397							Complete Remission/Response		0	397	0	397	0	397	0	397
TCGA-EA	TCGA-EA	CESC	45	FEMALE	WHITE		Stage IB	Cervical S	G2	2011	Pre (<6 m	-16460	Alive	TUMOR F	761							Complete Remission/Response		0	761	0	761	0	761	0	761
TCGA-EA	TCGA-EA	CESC	40	FEMALE	WHITE		Stage IB	Cervical S	G3	2012	Pre (<6 m	-14706	Alive	TUMOR F	1122							Complete Remission/Response		0	1122	0	1122	0	1122	0	1122
TCGA-EA	TCGA-EA	CESC	51	FEMALE	WHITE		Stage IIA2	Cervical S	G2	2012	Pre (<6 m	-18720	Alive	TUMOR F	803							Complete Remission/Response		0	803	0	803	0	803	0	803
TCGA-EA	TCGA-EA	CESC	50	FEMALE	WHITE		Stage IB1	Cervical S	G2	2012	Post (prio	-18561	Alive	TUMOR F	747							Complete Remission/Response		0	747	0	747	0	747	0	747
TCGA-EA	TCGA-EA	CESC	50	FEMALE	ASIAN		Stage IIA1	Cervical S	G3	2012	Pre (<6 m	-18529	Alive	TUMOR F	965			Locoregic	Cervix		316	Complete Remission/Response		0	965	0	965	1	316	1	316
TCGA-EA	TCGA-EA	CESC	43	FEMALE	WHITE		Stage IB1	Cervical S	G2	2012	Pre (<6 m	-15871	Alive	TUMOR F	791							Complete Remission/Response		0	791	0	791	0	791	0	791
TCGA-EA	TCGA-EA	CESC	31	FEMALE	WHITE		Stage IIIB	Cervical S	G2	2012	Pre (<6 m	-11621	Alive	TUMOR F	369							Stable Disease		0	369	0	369			0	369
TCGA-EA	TCGA-EA	CESC	49	FEMALE	WHITE		Stage IB2	Endocervi	G2	2012	Pre (<6 m	-17995	Alive	TUMOR F	755							Complete Remission/Response		0	755	0	755	0	755	0	755
TCGA-EA	TCGA-EA	CESC	45	FEMALE	ASIAN		Stage IVA	Cervical S	G2	2012	Pre (<6 m	-16609	Dead	WITH TUMOR		227	Cervical C	Locoregic	Vulva		208	Progressive Disease		1	227	1	227			1	208
TCGA-EA	TCGA-EA	CESC	38	FEMALE	WHITE		Stage IB1	Endometr	G3	2012	Pre (<6 m	-13988	Alive	TUMOR F	453							Complete Remission/Response		0	453	0	453	0	453	0	453
TCGA-EA	TCGA-EA	CESC	59	FEMALE	WHITE		Stage IB1	Cervical S	G2	2012	Post (prio	-21561	Alive	TUMOR F	815							Complete Remission/Response		0	815	0	815	0	815	0	815
TCGA-EA	TCGA-EA	CESC	39	FEMALE	WHITE		Stage IB2	Cervical S	G2	2012	Pre (<6 m	-14472	Alive	TUMOR F	788							Complete Remission/Response		0	788	0	788	0	788	0	788
TCGA-EA	TCGA-EA	CESC	40	FEMALE	ASIAN		Stage IB1	Cervical S	G2	2012	Pre (<6 m	-14629	Alive	TUMOR F	830							Complete Remission/Response		0	830	0	830	0	830	0	830
TCGA-EA	TCGA-EA	CESC	54	FEMALE	ASIAN		Stage IB1	Cervical S	G3	2012	Peri (6-12	-19868	Alive	TUMOR F	829			Distant M	Other, sp	Lymph nc	425	Complete Remission/Response		0	829	0	829	1	425	1	425
TCGA-EA	TCGA-EA	CESC	56	FEMALE	ASIAN		Stage IB1	Cervical S	G2	2012	Peri (6-12	-20506	Alive	WITH TUN	828			Locoregic	Cervix		440	Complete Remission/Response		0	828	0	828	1	440	1	440
TCGA-EA	TCGA-EA	CESC	49	FEMALE	ASIAN		Stage IIIB	Cervical S	G3	2013	Peri (6-12	-18035	Alive	TUMOR F	730							Complete Remission/Response		0	730	0	730	0	730	0	730
TCGA-EA	TCGA-EA	CESC	54	FEMALE	ASIAN		Stage IB1	Cervical S	G3	2013	Peri (6-12	-19942	Alive	TUMOR F	410							Complete Remission/Response		0	410	0	410	0	410	0	410
TCGA-EA	TCGA-EA	CESC	38	FEMALE	WHITE		Stage IB2	Cervical S	G2	2013	Pre (<6 m	-13984	Alive	TUMOR F	11							Complete Remission/Response		0	11	0	11	0	11	0	11
TCGA-EK-	TCGA-EK-	CESC	64	FEMALE	BLACK OR AFRICAN		Stage IIIB	Cervical S	G2	2008	Post (prio	-23599	Alive	WITH TUN	383									0	383	0	383			0	383
TCGA-EK-	TCGA-EK-	CESC	24	FEMALE	WHITE		Stage IIB	Cervical S	G3	2007	Pre (<6 m	-8918	Alive	TUMOR F	1847							Complete Remission/Response		0	1847	0	1847	0	1847	0	1847
TCGA-EK-	TCGA-EK-	CESC	20	FEMALE	WHITE		Stage IB2	Cervical S	G3	2007	Pre (<6 m	-7649	Alive	TUMOR F	799									0	799	0	799			0	799
TCGA-EK-	TCGA-EK-	CESC	28	FEMALE	WHITE		Stage IB1	Cervical S	G3	2006		-10347	Alive		202									0	202	0	202			0	202
TCGA-EK-	TCGA-EK-	CESC	48	FEMALE	WHITE		Stage IB2	Cervical S	G3	1997		-17581	Alive	TUMOR F	3442							Complete Remission/Response		0	3442	0	3442	0	3442	0	3442
TCGA-EK-	TCGA-EK-	CESC	88	FEMALE	BLACK OR AFRICAN AMERICAN			Cervical S	G3	2005	Post (prio	-32500	Alive		46									0	46	0	46			0	46
TCGA-EK-	TCGA-EK-	CESC	44	FEMALE	WHITE		Stage IIIB	Cervical S	G2	2009		-16324	Alive	WITH TUN	586									0	586	0	586			0	586
TCGA-EK-	TCGA-EK-	CESC	43	FEMALE	WHITE		Stage IB1	Cervical S	G3	2006	Indetermi	-15939	Alive	TUMOR F	12									0	12	0	12			0	12
TCGA-EK-	TCGA-EK-	CESC	36	FEMALE	WHITE		Stage IIIB	Cervical Squamous C		2008		-13384	Alive	WITH TUN	13									0	13	0	13			0	13
TCGA-EK-	TCGA-EK-	CESC	81	FEMALE	WHITE		Stage IIB	Cervical S	G3	2008	Post (prio	-29747	Alive	WITH TUN	18									0	18	0	18			0	18
TCGA-EK-	TCGA-EK-	CESC	45	FEMALE	WHITE		Stage IB	Cervical S	G3	2010	Pre (<6 m	-16487	Alive	TUMOR F	27									0	27	0	27			0	27
TCGA-EK-	TCGA-EK-	CESC	48	FEMALE	WHITE		Stage IB2	Cervical S	G3	2010	Pre (<6 m	-17692	Alive	WITH TUN	44									0	44	0	44			0	44
TCGA-EK-	TCGA-EK-	CESC	58	FEMALE	WHITE		Stage IB1	Cervical S	G3	2011		-21242	Alive	WITH TUN	4									0	4	0	4			0	4
TCGA-EK-	TCGA-EK-	CESC	74	FEMALE			Stage IIA2	Cervical S	G3	2011		-27209	Alive	WITH TUN	1246									0	1246	0	1246			0	1246
TCGA-EK-	TCGA-EK-	CESC	48	FEMALE	WHITE		Stage IVB	Cervical S	G3	2011		-17752	Alive	TUMOR F	9									0	9	0	9			0	9
TCGA-EK-	TCGA-EK-	CESC	33	FEMALE			Stage IB1	Cervical S	G3	2009		-12353	Alive	TUMOR F	129									0	129	0	129			0	129
TCGA-EK-	TCGA-EK-	CESC	47	FEMALE	AMERICAN INDIAN (		Stage IB1	Cervical S	G2	2009	Post (prio	-17191	Alive	TUMOR F	2									0	2	0	2			0	2
TCGA-EK-	TCGA-EK-	CESC	26	FEMALE	WHITE		Stage IIA	Cervical S	G2	2010	Pre (<6 m	-9743	Alive	TUMOR F	57									0	57	0	57			0	57
TCGA-EK-	TCGA-EK-	CESC	51	FEMALE	WHITE		Stage IB2	Cervical S	G3	2009		-18664	Alive	TUMOR F	53									0	53	0	53			0	53
TCGA-EK-	TCGA-EK-	CESC	67	FEMALE	WHITE		Stage IA2	Cervical S	G3	2009	Post (prio	-24541	Alive		13									0	13	0	13			0	13
TCGA-EK-	TCGA-EK-	CESC	32	FEMALE	BLACK OR AFRICAN		Stage IB	Endocervi	G2	2009		-12047	Dead	WITH TUMOR		1453	Cervical C	Locoregic	Cervix		1143	Progressive Disease		1	1453	1	1453			1	1143
TCGA-EK-	TCGA-EK-	CESC	40	FEMALE	WHITE		Stage IB	Cervical S	G3	2009		-14842	Alive	WITH TUN	50									0	50	0	50			0	50
TCGA-EK-	TCGA-EK-	CESC	45	FEMALE	WHITE		Stage IB1	Cervical S	G2	2009	Post (prio	-16487	Alive	WITH TUN	71									0	71	0	71			0	71
TCGA-EK-	TCGA-EK-	CESC	59	FEMALE	AMERICAN INDIAN (		Stage IIB	Cervical S	G1	2010	Post (prio	-21844	Alive	WITH TUN	2									0	2	0	2			0	2
TCGA-EK-	TCGA-EK-	CESC	51	FEMALE	NATIVE HAWAIIAN (		Stage IB1	Cervical S	G3	2010		-18630	Alive	[Discrepar	3									0	3	0	3			0	3
TCGA-EK-	TCGA-EK-	CESC	33	FEMALE	WHITE		Stage IB1	Endocervi	G3	2009		-12207	Alive	TUMOR F	15									0	15	0	15			0	15
TCGA-EK-	TCGA-EK-	CESC	65	FEMALE	WHITE		Stage IIA	Endocervi	G2	2006	Post (prio	-23879	Alive		0									0	0	0	0			0	0
TCGA-EK-	TCGA-EK-	CESC	47	FEMALE	WHITE		Stage IIIB	Cervical S	G3	2005		-17425	Alive	WITH TUN	27									0	27	0	27			0	27
TCGA-EX-	TCGA-EX-	CESC	58	FEMALE	BLACK OR AFRICAN		Stage IIB	Cervical S	G3	2010	Post (prio	-21338	Alive	TUMOR F	619							Complete Remission/Response		0	619	0	619	0	619	0	619
TCGA-EX-	TCGA-EX-	CESC	38	FEMALE	WHITE		Stage IB1	Endocervi	G1	2009	Pre (<6 m	-14214	Alive	TUMOR F	241							Complete Remission/Response		0	241	0	241	0	241	0	241
TCGA-EX-	TCGA-EX-	CESC	32	FEMALE	WHITE		Stage IIA1	Cervical S	G3	2011	Pre (<6 m	-11943	Alive	TUMOR F	463							Complete Remission/Response		0	463	0	463	0	463	0	463
TCGA-EX-	TCGA-EX-	CESC	42	FEMALE	WHITE		Stage IVB	Endocervi	G1	2012	Pre (<6 m	-15559	Alive	TUMOR F	447							Complete Remission/Response		0	447	0	447			0	447
TCGA-EX-	TCGA-EX-	CESC	41	FEMALE	ASIAN		Stage IB1	Cervical S	G3	2012	Pre (<6 m	-15121	Alive	TUMOR F	602							Complete Remission/Response		0	602	0	602	0	602	0	602
TCGA-EX-	TCGA-EX-	CESC	62	FEMALE	WHITE		Stage IB2	Cervical S	G3	2012	Post (prio	-22917	Dead	TUMOR FREE		253		New Prim	Lung		77	Complete Remission/Response		1	253	0	253	0	253	1	77
TCGA-EX-	TCGA-EX-	CESC	44	FEMALE	WHITE		Stage IB1	Cervical S	G3	2013	Pre (<6 m	-16258	Alive	WITH TUN	473			Locoregic	Other, sp	Vagina	396	Complete Remission/Response		0	473	0	473	1	396	1	396
TCGA-FU	TCGA-FU	CESC	28	FEMALE	WHITE		Stage IIIB	Cervical S	G3	2010	Pre (<6 m	-10551	Alive	TUMOR F	372							Complete Remission/Response		0	372	0	372	0	372	0	372
TCGA-FU	TCGA-FU	CESC	60	FEMALE	WHITE		Stage IIA1	Cervical S	G3	2010	Post (prio	-21962	Alive	TUMOR F	725							Complete Remission/Response		0	725	0	725	0	725	0	725
TCGA-FU	TCGA-FU	CESC	29	FEMALE	WHITE		Stage IB1	Cervical S	G2	2011	Peri (6-12	-10865	Alive	TUMOR F	579							Complete Remission/Response		0	579	0	579	0	579	0	579
TCGA-FU	TCGA-FU	CESC	55	FEMALE	WHITE		Stage IIB	Endocervi	G2	2011	Post (prio	-20207	Alive	TUMOR F	490							Complete Remission/Response		0	490	0	490	0	490	0	490
TCGA-FU	TCGA-FU	CESC	55	FEMALE	WHITE		Stage IIB	Endocervi	G2	2011	Post (prio	-20207	Alive	TUMOR F	490							Complete Remission/Response		0	490	0	490	0	490	0	490
TCGA-FU	TCGA-FU	CESC	47	FEMALE	WHITE		Stage IB2	Cervical S	G2	2011	Pre (<6 m	-17228	Alive	TUMOR F	954							Complete Remission/Response		0	954	0	954	0	954	0	954
TCGA-FU	TCGA-FU	CESC	64	FEMALE	WHITE		Stage IIA2	Cervical S	G3	2010	Post (prio	-23565	Alive	TUMOR F	1103							Complete Remission/Response		0	1103	0	1103	0	1103	0	1103
TCGA-FU	TCGA-FU	CESC	45	FEMALE	WHITE		Stage IB1	Cervical S	G2	2010	Pre (<6 m	-16533	Dead	TUMOR FREE		638								1	638	0	638			0	638
TCGA-FU	TCGA-FU	CESC	55	FEMALE	WHITE		Stage IIIB	Cervical S	G2	2010	Post (prio	-20302	Alive	TUMOR F	795							Complete Remission/Response		0	795	0	795	0	795	0	795
TCGA-FU	TCGA-FU	CESC	78	FEMALE	[Not Evaluated]		Stage IB2	Cervical S	G3	2012	Post (prio	-28825	Alive		45							[Unknown]		0	45	0	45			0	45
TCGA-FU	TCGA-FU	CESC	43	FEMALE	WHITE		Stage IIA2	Cervical S	G2	2012	[Unknowr	-15833	Alive	TUMOR F	491							Complete Remission/Response		0	491	0	491	0	491	0	491
TCGA-FU	TCGA-FU	CESC	35	FEMALE	WHITE		Stage IB1	Cervical S	G1	2012	Pre (<6 m	-12816	Alive	TUMOR F	861							Complete Remission/Response		0	861	0	861	0	861	0	861
TCGA-FU	TCGA-FU	CESC	38	FEMALE	WHITE		Stage IIIB	Endocervi	G3	2012	Pre (<6 m	-14182	Alive	TUMOR F	426							Complete Remission/Response		0	426	0	426	0	426	0	426
TCGA-FU	TCGA-FU	CESC	49	FEMALE	WHITE		Stage IB2	Endocervi	G2	2011	Pre (<6 m	-17905	Alive	TUMOR F	1078							Complete Remission/Response		0	1078	0	1078	0	1078	0	1078
TCGA-FU	TCGA-FU	CESC	32	FEMALE	WHITE		Stage IIIB	Cervical S	G3	2012	Pre (<6 m	-11692	Alive	TUMOR F	321							Complete Remission/Response		0	321	0	321	0	321	0	321
TCGA-FU	TCGA-FU	CESC	33	FEMALE	WHITE		Stage IIIB	Endocervi	G2	2013	Pre (<6 m	-12248	Alive	TUMOR F	34							[Unknown]		0	34	0	34			0	34
TCGA-GH	TCGA-GH	CESC	27	FEMALE	WHITE		Stage IB1	Cervical S	G3	2013	[Not Evalu	-10160	Alive	TUMOR F	540							Complete Remission/Response		0	540	0	540	0	540	0	540
TCGA-HG	TCGA-HG	CESC	38	FEMALE	WHITE		Stage IB2	Cervical S	G2	2009	Pre (<6 m	-14025	Dead	WITH TUMOR		773						Complete Remission/Response		1	773	1	773			1	773
TCGA-HG	TCGA-HG	CESC	49	FEMALE	WHITE		Stage IIB	Adenosqu	G3	2013	Pre (<6 m	-17963	Alive	TUMOR F	504							Complete Remission/Response		0	504	0	504	0	504	0	504
TCGA-HM	TCGA-HM	CESC	40	FEMALE	BLACK OR AFRICAN		Stage IB1	Cervical S	G3	2010	Pre (<6 m	-14736	Dead	WITH TUMOR		659	Cervical C	Distant Metastasis			385	Complete Remission/Response		1	659	1	659	1	385	1	385
TCGA-HM	TCGA-HM	CESC	40	FEMALE	BLACK OR AFRICAN		Stage IB1	Cervical S	G3	2010	Pre (<6 m	-14736	Dead	WITH TUMOR		659	Cervical C	Distant Metastasis			385	Complete Remission/Response		1	659	1	659	1	385	1	385
TCGA-HM	TCGA-HM	CESC	64	FEMALE	WHITE		Stage IIA2	Cervical S	G3	2011	Post (prio	-23618	Alive	TUMOR F	632							Complete Remission/Response		0	632	0	632	0	632	0	632
TCGA-HM	TCGA-HM	CESC	51	FEMALE	WHITE		Stage IIIB	Cervical S	G3	2012	Pre (<6 m	-18903	Alive	TUMOR F	454							Complete Remission/Response		0	454	0	454	0	454	0	454
TCGA-HM	TCGA-HM	CESC	34	FEMALE	BLACK OR AFRICAN		Stage IVB	Adenosqu	G3	2013	Pre (<6 m	-12775	Alive	TUMOR F	287							Complete Remission/Response		0	287	0	287			0	287
TCGA-HM	TCGA-HM	CESC	34	FEMALE	BLACK OR AFRICAN		Stage IVB	Adenosqu	G3	2013	Pre (<6 m	-12775	Alive	TUMOR F	287							Complete Remission/Response		0	287	0	287			0	287
TCGA-IR-	TCGA-IR-	CESC	37	FEMALE	WHITE		Stage IB1	Cervical S	G3	2000	Pre (<6 m	-13559	Alive	TUMOR F	4483							Complete Remission/Response		0	4483	0	4483	0	4483	0	4483
TCGA-IR-	TCGA-IR-	CESC	60	FEMALE	WHITE		Stage IB1	Endometr	G3	2002	Post (prio	-21949	Alive	TUMOR F	4172							Complete Remission/Response		0	4172	0	4172	0	4172	0	4172
TCGA-IR-	TCGA-IR-	CESC	53	FEMALE	WHITE		Stage IB1	Endocervi	G3	2002	Post (prio	-19374	Dead	WITH TUMOR		2032		Distant M	Lung		317	Complete Remission/Response		1	2032	1	2032	1	317	1	317
TCGA-IR-	TCGA-IR-	CESC	40	FEMALE	WHITE		Stage IB1	Cervical S	G3	2003	Pre (<6 m	-14697	Alive	TUMOR F	3935							Complete Remission/Response		0	3935	0	3935	0	3935	0	3935
TCGA-IR-	TCGA-IR-	CESC	64	FEMALE	WHITE		Stage IB1	Endocervi	G2	2004	Post (prio	-23721	Alive	TUMOR F	2949							Complete Remission/Response		0	2949	0	2949	0	2949	0	2949
TCGA-IR-	TCGA-IR-	CESC	49	FEMALE	BLACK OR AFRICAN		Stage IIA1	Cervical S	G4	2005	Pre (<6 m	-18118	Alive	TUMOR F	2394							Complete Remission/Response		0	2394	0	2394	0	2394	0	2394
TCGA-IR-	TCGA-IR-	CESC	48	FEMALE	WHITE		Stage IVB	Endocervi	G2	2005	Peri (6-12	-17741	Alive	TUMOR F	2493							Complete Remission/Response		0	2493	0	2493			0	2493
TCGA-IR-	TCGA-IR-	CESC	69	FEMALE	WHITE		Stage IB2	Cervical S	G3	2009	Post (prio	-25440	Dead	WITH TUMOR		908	Cervical C	Distant M	Other, sp	thoracic l	574	Complete Remission/Response		1	908	1	908	1	574	1	574
TCGA-IR-	TCGA-IR-	CESC	60	FEMALE	WHITE		Stage IB1	Cervical S	G2	2010	Post (prio	-22123	Alive	TUMOR F	1106							Complete Remission/Response		0	1106	0	1106	0	1106	0	1106
TCGA-JW	TCGA-JW	CESC	35	FEMALE	AMERICAN INDIAN (		Stage IIA	Cervical S	G3	2012	Pre (<6 m	-13109	Alive	TUMOR F	834							Complete Remission/Response		0	834	0	834	0	834	0	834
TCGA-JW	TCGA-JW	CESC	53	FEMALE	AMERICAN INDIAN (		Stage IVB	Cervical S	G2	2012	Pre (<6 m	-19423	Dead	WITH TUMOR		100	Cervical C	Distant M	Other, sp	Brain	94	Progressive Disease		1	100	1	100			1	94
TCGA-JW	TCGA-JW	CESC	45	FEMALE	AMERICAN INDIAN (		Stage IIB	Cervical S	G3	2012	Pre (<6 m	-16444	Alive	WITH TUN	747			Distant M	Lung		558	Complete Remission/Response		0	747	0	747	1	558	1	558
TCGA-JW	TCGA-JW	CESC	56	FEMALE	AMERICAN INDIAN (		Stage IIB	Cervical S	G3	2012	Post (prio	-20714	Alive	WITH TUN	652			Distant M	Head & Neck		267	Progressive Disease		0	652	0	652			1	267
TCGA-JW	TCGA-JW	CESC	43	FEMALE	BLACK OR AFRICAN		Stage IB2	Cervical S	G3	2012	Pre (<6 m	-15965	Alive	TUMOR F	623							Complete Remission/Response		0	623	0	623	0	623	0	623
TCGA-JW	TCGA-JW	CESC	37	FEMALE	AMERICAN INDIAN (		Stage IB2	Cervical S	G1	2012	Pre (<6 m	-13604	Alive	TUMOR F	474							Complete Remission/Response		0	474	0	474	0	474	0	474
TCGA-JW	TCGA-JW	CESC	44	FEMALE	WHITE		Stage IB2	Endocervi	GX	2012	Pre (<6 m	-16080	Alive	TUMOR F	863							Complete Remission/Response		0	863	0	863	0	863	0	863
TCGA-JW	TCGA-JW	CESC	42	FEMALE	WHITE		Stage IIB	Cervical S	G2	2013	Pre (<6 m	-15682	Dead	WITH TUMOR		252	Cervical C	New Primary Tumor		Breast	153	Complete Remission/Response		1	252	1	252	0	252	1	153
TCGA-JW	TCGA-JW	CESC	46	FEMALE	WHITE		Stage IB1	Cervical S	G2	2013	Pre (<6 m	-16894	Alive	TUMOR F	552							Complete Remission/Response		0	552	0	552	0	552	0	552
TCGA-JX-	TCGA-JX-	CESC	25	FEMALE	WHITE		Stage IB	Cervical S	G2	1995	Pre (<6 m	-9303	Dead	WITH TUMOR		642	Cervical C	Locoregic	Other, sp	Pelvis	491	Complete Remission/Response		1	642	1	642	1	491	1	491
TCGA-JX-	TCGA-JX-	CESC	63	FEMALE	BLACK OR AFRICAN		Stage III	Cervical S	G2	1997	Post (prio	-23313	Alive	TUMOR F	6375							Complete Remission/Response		0	6375	0	6375	0	6375	0	6375
TCGA-JX-	TCGA-JX-	CESC	40	FEMALE	ASIAN		Stage IB1	Endocervi	G3	2011	Pre (<6 m	-14839	Alive	TUMOR F	1357							Complete Remission/Response		0	1357	0	1357	0	1357	0	1357
TCGA-JX-	TCGA-JX-	CESC	37	FEMALE	WHITE		Stage IB1	Cervical S	G3	2012	Pre (<6 m	-13667	Alive	TUMOR F	636							Complete Remission/Response		0	636	0	636	0	636	0	636
TCGA-LP-	TCGA-LP-	CESC	35	FEMALE	ASIAN		Stage IIIB	Cervical S	G3	2011	Pre (<6 m	-13074	Alive	TUMOR F	343									0	343	0	343			0	343
TCGA-LP-	TCGA-LP-	CESC	63	FEMALE	ASIAN		Stage IB	Cervical S	G2	2011	[Not Evaluated]		Alive	TUMOR F	0									0	0	0	0			0	0
TCGA-LP-	TCGA-LP-	CESC	52	FEMALE	ASIAN		Stage IA	Cervical S	G1	2011	Peri (6-12	-19079	Alive	TUMOR F	27									0	27	0	27			0	27
TCGA-LP-	TCGA-LP-	CESC	45	FEMALE	ASIAN		Stage IB1	Cervical S	G3	2011	Peri (6-12	-16544	Alive	TUMOR F	380							Complete Remission/Response		0	380	0	380	0	380	0	380
TCGA-LP-	TCGA-LP-	CESC	30	FEMALE	ASIAN		Stage IIIB	Endocervi	G3	2012	Pre (<6 m	-11049	Alive	TUMOR F	9							Complete Remission/Response		0	9	0	9	0	9	0	9
TCGA-LP-	TCGA-LP-	CESC	40	FEMALE	ASIAN		Stage IB1	Cervical S	G3	2012	Pre (<6 m	-14698	Alive	TUMOR F	25							Complete Remission/Response		0	25	0	25	0	25	0	25
TCGA-LP-	TCGA-LP-	CESC	53	FEMALE	ASIAN		Stage II	Endocervi	G3	2013	Post (prio	-19595	Alive	TUMOR F	406							Complete Remission/Response		0	406	0	406	0	406	0	406
TCGA-MA	TCGA-MA	CESC	54	FEMALE	WHITE		Stage IB1	Cervical S	G3	2012	Post (prio	-20066	Alive	TUMOR F	685							Complete Remission/Response		0	685	0	685	0	685	0	685
TCGA-MA	TCGA-MA	CESC	50	FEMALE	BLACK OR AFRICAN		Stage IIIB	Cervical S	G2	2012	Post (prio	-18412	Alive	TUMOR F	617							Complete Remission/Response		0	617	0	617	0	617	0	617
TCGA-MA	TCGA-MA	CESC	48	FEMALE	WHITE		Stage IB1	Cervical S	G3	2012	Pre (<6 m	-17795	Alive	TUMOR F	542							Complete Remission/Response		0	542	0	542	0	542	0	542
TCGA-MA	TCGA-MA	CESC	43	FEMALE	WHITE		Stage IB2	Cervical S	GX	2013	Pre (<6 m	-15762	Alive	TUMOR F	595							Complete Remission/Response		0	595	0	595	0	595	0	595
TCGA-MA	TCGA-MA	CESC	33	FEMALE	WHITE		Stage IIB	Cervical S	G2	2013	Pre (<6 m	-12398	Alive	WITH TUN	279							Progressive Disease		0	279	0	279			0	279
TCGA-MA	TCGA-MA	CESC	75	FEMALE	WHITE		Stage IIB	Cervical S	G3	2013	Post (prio	-27460	Alive	TUMOR F	259							Complete Remission/Response		0	259	0	259	0	259	0	259
TCGA-MA	TCGA-MA	CESC	48	FEMALE	WHITE		Stage IIIB	Cervical S	G3	2013	Peri (6-12	-17658	Alive	TUMOR F	346							Complete Remission/Response		0	346	0	346	0	346	0	346
TCGA-MU	TCGA-MU	CESC	27	FEMALE	WHITE		Stage IIA1	Cervical S	G2	2012	Pre (<6 m	-10199	Alive	TUMOR F	854							Complete Remission/Response		0	854	0	854	0	854	0	854
TCGA-MU	TCGA-MU	CESC	60	FEMALE	BLACK OR AFRICAN		Stage IA1	Cervical S	G2	2011	Post (prio	-21927	Alive	WITH TUN	1053			Distant M	Other, sp	Thoracic S	630			0	1053	0	1053			1	630
TCGA-MU	TCGA-MU	CESC	46	FEMALE	WHITE		Stage IB1	Cervical S	G2	2013	[Unknowr	-17156	Alive	TUMOR F	607							Complete Remission/Response		0	607	0	607	0	607	0	607
TCGA-MY	TCGA-MY	CESC	62	FEMALE	WHITE		Stage IIB	Cervical S	G1	2010	Post (prio	-22901	Alive	TUMOR F	1667							Stable Disease		0	1667	0	1667			0	1667
TCGA-MY	TCGA-MY	CESC	42	FEMALE	BLACK OR AFRICAN		Stage IB1	Cervical S	G3	2011	Post (prio	-15466	Alive	TUMOR F	1066							Complete Remission/Response		0	1066	0	1066	0	1066	0	1066
TCGA-MY	TCGA-MY	CESC	68	FEMALE	WHITE		Stage IIA2	Cervical S	G1	2012	Post (prio	-25082	Alive	TUMOR F	634							Stable Disease		0	634	0	634			0	634
TCGA-MY	TCGA-MY	CESC	68	FEMALE	WHITE		Stage IIA2	Cervical S	G1	2012	Post (prio	-25082	Alive	TUMOR F	634							Stable Disease		0	634	0	634			0	634
TCGA-MY	TCGA-MY	CESC	28	FEMALE	BLACK OR AFRICAN		Stage IIA	Cervical S	G3	2013	Pre (<6 m	-10379	Alive	TUMOR F	524							Complete Remission/Response		0	524	0	524	0	524	0	524
TCGA-PN	TCGA-PN	CESC	43	FEMALE	BLACK OR AFRICAN		Stage IIB	Cervical S	G3	2013	Pre (<6 m	-15918	Alive	WITH TUN	90							Stable Disease		0	90	0	90			0	90
TCGA-O1	TCGA-O1	CESC	32	FEMALE	WHITE		Stage IB1	Mucinous	G2	2012	Pre (<6 m	-11705	Alive	TUMOR F	474							Complete Remission/Response		0	474	0	474	0	474	0	474
TCGA-O1	TCGA-O1	CESC	64	FEMALE	WHITE			Cervical S	G3	2012	Post (prio	-23523	Alive	TUMOR F	499									0	499	0	499			0	499
TCGA-O1	TCGA-O1	CESC	56	FEMALE	WHITE			Cervical S	G2	2012	Post (prio	-20574	Alive	TUMOR F	485							Complete Remission/Response		0	485	0	485	0	485	0	485
TCGA-O1	TCGA-O1	CESC	55	FEMALE	WHITE		Stage I	Cervical S	GX	2013	Post (prio	-20286	Dead	WITH TUMOR		275	Cervical Cancer							1	275	1	275			1	275
TCGA-Q1	TCGA-Q1	CESC	36	FEMALE	ASIAN		Stage IB1	Mucinous	G2	2013	Pre (<6 m	-13166	Alive	TUMOR F	491							Complete Remission/Response		0	491	0	491	0	491	0	491
TCGA-O1	TCGA-O1	CESC	44	FEMALE	WHITE		Stage I	Cervical S	GX	2013	Pre (<6 m	-16200	Alive		534							[Unknown]		0	534	0	534			0	534
TCGA-O1	TCGA-O1	CESC	37	FEMALE	[Not Evaluated]		Stage IB2	Cervical S	GX	2013	Pre (<6 m	-13601	Alive	TUMOR F	428							Complete Remission/Response		0	428	0	428	0	428	0	428
TCGA-O1	TCGA-O1	CESC	45	FEMALE	WHITE		Stage IB1	Endocervi	G1	2013	Pre (<6 m	-16450	Alive	TUMOR F	483							Complete Remission/Response		0	483	0	483	0	483	0	483
TCGA-O1	TCGA-O1	CESC	46	FEMALE	WHITE		Stage I	Cervical S	GX	2013	Pre (<6 m	-16851	Alive	WITH TUN	284							[Unknown]		0	284	0	284			0	284
TCGA-O1	TCGA-O1	CESC	45	FEMALE	WHITE		Stage I	Endocervi	GX	2013	Pre (<6 m	-16701	Alive	TUMOR F	567							Complete Remission/Response		0	567	0	567	0	567	0	567
TCGA-O1	TCGA-O1	CESC	33	FEMALE	WHITE		Stage IB1	Adenosqu	G2	2012	Pre (<6 m	-12229	Alive	TUMOR F	688							Complete Remission/Response		0	688	0	688	0	688	0	688
TCGA-R2-	TCGA-R2-	CESC	42	FEMALE	WHITE		Stage IB	Cervical S	G3	2011	Peri (6-12	-15479	Alive	TUMOR F	596							Complete Remission/Response		0	596	0	596	0	596	0	596
TCGA-RA	TCGA-RA	CESC	34	FEMALE	WHITE		Stage IIB	Cervical S	GX	2013	Pre (<6 m	-12607	Alive	TUMOR F	444							Complete Remission/Response		0	444	0	444	0	444	0	444
TCGA-UC	TCGA-UC	CESC	21	FEMALE	AMERICAN INDIAN (		Stage IB	Cervical S	G2	1997	Pre (<6 m	-7855	Dead	WITH TUMOR		355	Cervical C	Locoregional Recurre		vaginal cu	202	Complete Remission/Response		1	355	1	355	1	202	1	202
TCGA-UC	TCGA-UC	CESC	44	FEMALE	[Not Evaluated]		Stage IB1	Cervical S	G2	1999	Pre (<6 m	-16412	Dead			2859								1	2859		2859			0	2859
TCGA-UC	TCGA-UC	CESC	44	FEMALE	WHITE		Stage IIIB	Cervical S	G1	2003	Pre (<6 m	-16231	Dead	WITH TUMOR		370	Cervical Cancer					Progressive Disease		1	370	1	370			1	370
TCGA-UC	TCGA-UC	CESC	44	FEMALE	WHITE		Stage IIIB	Cervical S	G1	2003	Pre (<6 m	-16231	Dead	WITH TUMOR		370	Cervical Cancer					Progressive Disease		1	370	1	370			1	370
TCGA-UC	TCGA-UC	CESC	44	FEMALE	WHITE		Stage IB1	Endometr	G1	2009	Pre (<6 m	-16427	Alive	TUMOR F	2114			Locoregic	Other, specify		778	Complete Remission/Response		0	2114	0	2114	1	778	1	778
TCGA-VS	TCGA-VS	CESC	41	FEMALE	WHITE		Stage IIIB	Cervical S	GX	2010	Pre (<6 m	-15215	Dead	WITH TUMOR		305	Cervical Cancer					Progressive Disease		1	305	1	305			1	305
TCGA-VS	TCGA-VS	CESC	55	FEMALE	WHITE		Stage IIIB	Cervical S	G2	2011	Peri (6-12	-20286	Alive	TUMOR F	1415							Complete Remission/Response		0	1415	0	1415	0	1415	0	1415
TCGA-VS	TCGA-VS	CESC	36	FEMALE	[Unknown]		Stage IB1	Cervical S	GX	2011	Peri (6-12	-13166	Alive	TUMOR F	1386							Complete Remission/Response		0	1386	0	1386	0	1386	0	1386
TCGA-VS	TCGA-VS	CESC	56	FEMALE	[Unknown]		Stage IIIB	Cervical S	G2	2012	Peri (6-12	-20728	Alive	TUMOR F	986							Complete Remission/Response		0	986	0	986	0	986	0	986
TCGA-VS	TCGA-VS	CESC	38	FEMALE	WHITE		Stage IIB	Cervical S	GX	2012	Pre (<6 m	-14129	Alive	TUMOR F	729							Complete Remission/Response		0	729	0	729	0	729	0	729
TCGA-VS	TCGA-VS	CESC	60	FEMALE	WHITE		Stage IIB	Cervical S	G3	2008	Post (prio	-22000	Dead	WITH TUMOR		607	Cervical Cancer					Progressive Disease		1	607	1	607			1	607
TCGA-VS	TCGA-VS	CESC	65	FEMALE	WHITE		Stage IVA	Cervical S	G2	2008	[Not Evalu	-23817	Dead	WITH TUMOR		829	Cervical Cancer					Partial Remission/Response		1	829	1	829			1	829
TCGA-VS	TCGA-VS	CESC	38	FEMALE	BLACK OR AFRICAN		Stage IIB	Cervical S	G3	2009	Pre (<6 m	-14162	Alive	TUMOR F	1992							Complete Remission/Response		0	1992	0	1992	0	1992	0	1992
TCGA-VS	TCGA-VS	CESC	26	FEMALE	WHITE		Stage IB	Cervical S	G2	2010	Pre (<6 m	-9571	Dead	WITH TUMOR		978	Cervical C	Locoregional Recurre		Pelvis	488	Progressive Disease		1	978	1	978			1	488
TCGA-VS	TCGA-VS	CESC	79	FEMALE	WHITE		Stage IB1	Cervical S	G2	2010	Post (prio	-28881	Alive	TUMOR F	1630							Complete Remission/Response		0	1630	0	1630	0	1630	0	1630
TCGA-VS	TCGA-VS	CESC	44	FEMALE	[Unknown]		Stage IB1	Cervical S	GX	2011	Peri (6-12	-16072	Alive	TUMOR F	1099							Complete Remission/Response		0	1099	0	1099	0	1099	0	1099
TCGA-VS	TCGA-VS	CESC	51	FEMALE	WHITE		Stage IVA	Cervical S	G2	2009	Peri (6-12	-18743	Dead	WITH TUMOR		350	Cervical C	Distant Metastasis		Liver	263	Progressive Disease		1	350	1	350			1	263
TCGA-VS	TCGA-VS	CESC	42	FEMALE	WHITE		Stage IIB	Cervical S	G2	2010	Pre (<6 m	-15374	Alive	TUMOR F	1800							Complete Remission/Response		0	1800	0	1800	0	1800	0	1800
TCGA-VS	TCGA-VS	CESC	76	FEMALE	WHITE		Stage IB1	Mucinous	G2	2010	Post (prio	-27909	Dead	WITH TUMOR		1210	Cervical Cancer					Partial Remission/Response		1	1210	1	1210			1	1210
TCGA-VS	TCGA-VS	CESC	47	FEMALE	[Unknown]		Stage IVB	Cervical S	GX	2009		-17168	Dead	WITH TUMOR		951	Cervical Cancer					Progressive Disease		1	951	1	951			1	951
TCGA-VS	TCGA-VS	CESC	39	FEMALE	WHITE		Stage IIB	Cervical S	G2	2011	Pre (<6 m	-14399	Alive	TUMOR F	1243							Complete Remission/Response		0	1243	0	1243	0	1243	0	1243
TCGA-VS	TCGA-VS	CESC	40	FEMALE	BLACK OR AFRICAN		Stage IIB	Cervical S	G2	2010	[Not Evalu	-14955	Dead	WITH TUMOR		506	Cervical Cancer					Partial Remission/Response		1	506	1	506			1	506
TCGA-VS	TCGA-VS	CESC	47	FEMALE	WHITE		Stage IIB	Cervical S	GX	2010	Pre (<6 m	-17465	Dead	WITH TUMOR		144	Cervical Cancer					Progressive Disease		1	144	1	144			1	144
TCGA-VS	TCGA-VS	CESC	38	FEMALE	WHITE		Stage IIB	Cervical S	G2	2012	Pre (<6 m	-14046	Alive	TUMOR F	1015							Complete Remission/Response		0	1015	0	1015	0	1015	0	1015
TCGA-VS	TCGA-VS	CESC	42	FEMALE	[Unknown]		Stage IIIA	Cervical S	G3	2011	Pre (<6 m	-15365	Alive	WITH TUN	1221							Partial Remission/Response		0	1221	0	1221			0	1221
TCGA-VS	TCGA-VS	CESC	66	FEMALE	WHITE		Stage IB1	Mucinous	G2	2010	Post (prio	-24345	Alive	TUMOR F	1778							Complete Remission/Response		0	1778	0	1778	0	1778	0	1778
TCGA-VS	TCGA-VS	CESC	63	FEMALE	[Unknown]		Stage IVA	Cervical S	GX	2010	Post (prio	-23277	Dead	WITH TUMOR		477	Cervical Cancer					Partial Remission/Response		1	477	1	477			1	477
TCGA-VS	TCGA-VS	CESC	67	FEMALE	[Unknown]		Stage IIIB	Cervical S	G2	2010	Post (prio	-24498	Alive	TUMOR F	1714							Complete Remission/Response		0	1714	0	1714	0	1714	0	1714
TCGA-VS	TCGA-VS	CESC	64	FEMALE	WHITE		Stage IB1	Cervical S	G3	2010	Post (prio	-23585	Alive	TUMOR F	1688							Complete Remission/Response		0	1688	0	1688	0	1688	0	1688
TCGA-VS	TCGA-VS	CESC	46	FEMALE	WHITE		Stage IIB	Cervical S	G2	2010	Pre (<6 m	-16905	Alive	TUMOR F	1525							Complete Remission/Response		0	1525	0	1525	0	1525	0	1525

TCGA-VS	TCGA-VS	CESC	76	FEMALE	[Unknown]		Stage IIB	Cervical S	G3	2010	Post (prio	-27897	Alive	TUMOR F	1561							Complete Remission/Response		0	1561	0	1561	0	1561	0	1561
TCGA-VS	TCGA-VS	CESC	57	FEMALE	WHITE		Stage IIB	Cervical S	G3	2010	Peri (6-12	-21149	Alive	TUMOR F	1535							Complete Remission/Response		0	1535	0	1535	0	1535	0	1535
TCGA-VS	TCGA-VS	CESC	52	FEMALE	WHITE		Stage IVB	Cervical S	GX	2011	Pre (<6 m	-19109	Alive	WITH TUM	1320			Distant Metastasis		Diaphragm	711	Stable Disease		0	1320	0	1320			1	711
TCGA-VS	TCGA-VS	CESC	30	FEMALE	WHITE		Stage IB1	Cervical S	G3	2011	Pre (<6 m	-11210	Alive	TUMOR F	1472							Complete Remission/Response		0	1472	0	1472	0	1472	0	1472
TCGA-VS	TCGA-VS	CESC	60	FEMALE	WHITE		Stage IIIA	Cervical S	G3	2012	Post (prio	-22221	Alive	TUMOR F	867							Complete Remission/Response		0	867	0	867	0	867	0	867
TCGA-VS	TCGA-VS	CESC	54	FEMALE	[Unknown]		Stage IIB	Cervical S	G3	2012	Post (prio	-19737	Alive	TUMOR F	911							Complete Remission/Response		0	911	0	911	0	911	0	911
TCGA-VS	TCGA-VS	CESC	32	FEMALE	[Unknown]		Stage IIB	Cervical S	G2	2012	Peri (6-12	-11790	Alive	WITH TUM	825							Stable Disease		0	825	0	825			0	825
TCGA-VS	TCGA-VS	CESC	73	FEMALE	WHITE		Stage IIIA	Cervical S	G2	2013	Post (prio	-26815	Alive	TUMOR F	739							Complete Remission/Response		0	739	0	739	0	739	0	739
TCGA-VS	TCGA-VS	CESC	53	FEMALE	BLACK OR AFRICAN		Stage IVA	Cervical S	GX	2010		-19454	Alive	TUMOR F	1427							Complete Remission/Response		0	1427	0	1427			0	1427
TCGA-VS	TCGA-VS	CESC	76	FEMALE	WHITE		Stage IIB	Cervical S	GX	2011	Post (prio	-27838	Dead	TUMOR FREE		1372						Complete Remission/Response		1	1372	0	1372	0	1372	0	1372
TCGA-VS	TCGA-VS	CESC	55	FEMALE	[Unknown]		Stage IIB	Cervical S	GX	2012	Pre (<6 m	-20264	Dead	WITH TUMOR		52	Cervical Cancer							1	52	1	52			1	52
TCGA-VS	TCGA-VS	CESC	79	FEMALE	WHITE		Stage IIIB	Cervical S	G3	2010	Post (prio	-29033	Dead	WITH TUMOR		442	Cervical C	Distant Metastasis		Bone	336	Progressive Disease		1	442	1	442			1	336
TCGA-VS	TCGA-VS	CESC	39	FEMALE	BLACK OR AFRICAN		Stage IVB	Cervical S	G2	2011	Peri (6-12	-14255	Dead	TUMOR FREE		829						Complete Remission/Response		1	829	0	829			0	829
TCGA-VS	TCGA-VS	CESC	43	FEMALE	WHITE		Stage IIB	Mucinous	G2	2011	Peri (6-12	-15813	Alive	TUMOR F	1456							Complete Remission/Response		0	1456	0	1456	0	1456	0	1456
TCGA-VS	TCGA-VS	CESC	43	FEMALE	[Unknown]		Stage IIA	Mucinous	G3	2011	Peri (6-12	-15849	Alive	WITH TUM	1444			Distant M	Other, sp	Paraortic	352	Complete Remission/Response		0	1444	0	1444	1	352	1	352
TCGA-VS	TCGA-VS	CESC	32	FEMALE	[Unknown]		Stage IB	Mucinous	G2	2011	Peri (6-12	-11853	Alive	TUMOR F	1263			Distant M	Other, sp	Ovary	561	Complete Remission/Response		0	1263	0	1263	1	561	1	561
TCGA-VS	TCGA-VS	CESC	53	FEMALE	WHITE		Stage IIA	Mucinous	GX	2013	[Unknowr	-19688	Alive	WITH TUM	793			Distant M	Other, sp	Retroperit	675	Complete Remission/Response		0	793	0	793	1	675	1	675
TCGA-VS	TCGA-VS	CESC	72	FEMALE	ASIAN		Stage IB	Mucinous	G3	2013	Post (prio	-26603	Alive	TUMOR F	482							Complete Remission/Response		0	482	0	482	0	482	0	482
TCGA-VS	TCGA-VS	CESC	42	FEMALE	[Unknown]		Stage IIB	Cervical S	G1	2013	Pre (<6 m	-15534	Alive	TUMOR F	442							Complete Remission/Response		0	442	0	442	0	442	0	442
TCGA-VS	TCGA-VS	CESC	74	FEMALE	[Unknown]		Stage IVA	Cervical S	GX	2013	Post (prio	-27348	Dead	WITH TUMOR		104	Cervical Cancer					[Unknown]		1	104	1	104			1	104
TCGA-VS	TCGA-VS	CESC	29	FEMALE	[Unknown]		Stage IVB	Cervical S	G2	2012	[Unknowr	-10707	Dead	WITH TUMOR		555	Cervical C	Distant M	Head & Neck		412	Progressive Disease		1	555	1	555			1	412
TCGA-VS	TCGA-VS	CESC	61	FEMALE	WHITE		Stage IB1	Mucinous	G2	2009	Peri (6-12	-22344	Alive	TUMOR F	2044							Complete Remission/Response		0	2044	0	2044	0	2044	0	2044
TCGA-VS	TCGA-VS	CESC	58	FEMALE	[Unknown]		Stage IB	Mucinous	G3	2013	Pre (<6 m	-21215	Alive	WITH TUM	573			Distant M	Other, sp	Retroperit	475	Complete Remission/Response		0	573	0	573	1	475	1	475
TCGA-VS	TCGA-VS	CESC	46	FEMALE	WHITE		Stage IVB	Mucinous	G2	2013	Pre (<6 m	-17001	Dead	WITH TUMOR		157	Cervical C	Distant Metastasis		Retroperit	58	Progressive Disease		1	157	1	157			1	58
TCGA-VS	TCGA-VS	CESC	29	FEMALE	WHITE		Stage IB1	Cervical S	G2	2013	Peri (6-12	-10637	Alive	TUMOR F	555							Complete Remission/Response		0	555	0	555	0	555	0	555
TCGA-VS	TCGA-VS	CESC	62	FEMALE	WHITE		Stage IVB	Cervical S	G3	2013	[Not Evalu	-22990	Alive	WITH TUM	540			Distant M	Other, sp	Mediastin	456	Complete Remission/Response		0	540	0	540			1	456
TCGA-VS	TCGA-VS	CESC	63	FEMALE	WHITE		Stage IVA	Mucinous	G2	2013	Post (prio	-23334	Dead	WITH TUMOR		132	Cervical C	New Primary Tumor		Sigmoid c	100	[Unknown]		1	132	1	132			1	100
TCGA-VS	TCGA-VS	CESC	50	FEMALE	WHITE		Stage IIB	Mucinous	G2	2011	Pre (<6 m	-18577	Dead	WITH TUMOR		494						Progressive Disease		1	494	1	494			1	494
TCGA-VS	TCGA-VS	CESC	51	FEMALE	WHITE		Stage IIB	Cervical S	G2	2009	Peri (6-12	-18792	Dead	WITH TUMOR		469	Cervical Cancer					Progressive Disease		1	469	1	469			1	469
TCGA-WL	TCGA-WL	CESC	57	FEMALE	WHITE			Cervical S	G3	2012	Pre (<6 months sinc		Alive	TUMOR F	791							Partial Remission/Response		0	791	0	791			0	791
TCGA-XS	TCGA-XS	CESC	41	FEMALE	BLACK OR AFRICAN		Stage IB1	Cervical S	G2	2012	Pre (<6 m	-15298	Alive	TUMOR F	890							Complete Remission/Response		0	890	0	890	0	890	0	890
TCGA-ZJ-	TCGA-ZJ-	CESC	73	FEMALE	[Unknown]			Cervical Squamous C		2007	[Unknowr	-26723	Alive		0							[Unknown]		0	0	0	0			0	0
TCGA-ZJ-	TCGA-ZJ-	CESC	24	FEMALE	WHITE		Stage IIB	Cervical S	GX	2007	Post (prio	-8918	Alive	TUMOR F	2056							Complete Remission/Response		0	2056	0	2056	0	2056	0	2056
TCGA-ZJ-	TCGA-ZJ-	CESC	38	FEMALE	NATIVE HAWAIIAN (		Stage I	Cervical S	GX	2007	Pre (<6 m	-14225	Dead			582						[Unknown]		1	582		582			0	582
TCGA-ZJ-	TCGA-ZJ-	CESC	85	FEMALE	WHITE		Stage II	Cervical S	G3	2008	[Unknowr	-31258	Alive		21							[Unknown]		0	21	0	21			0	21
TCGA-ZJ-	TCGA-ZJ-	CESC	58	FEMALE	[Unknown]		Stage IIIB	Cervical S	G2	2009	[Unknowr	-21481	Alive		0							[Unknown]		0	0	0	0			0	0
TCGA-ZJ-	TCGA-ZJ-	CESC	64	FEMALE	WHITE		Stage IB1	Cervical S	G2	2009	[Unknowr	-23603	Alive		43							[Unknown]		0	43	0	43			0	43
TCGA-ZJ-	TCGA-ZJ-	CESC	42	FEMALE	WHITE		Stage IB2	Endocervi	G3	2009	[Unknowr	-15492	Alive		0							[Unknown]		0	0	0	0			0	0
TCGA-ZJ-	TCGA-ZJ-	CESC	35	FEMALE	[Unknown]		Stage IIIB	Cervical S	G2	2010	[Unknowr	-12968	Alive		0							[Unknown]		0	0	0	0			0	0
TCGA-ZJ-	TCGA-ZJ-	CESC	62	FEMALE	[Unknown]		Stage IIB	Cervical S	G3	2010	[Unknowr	-22709	Alive		0							[Unknown]		0	0	0	0			0	0
TCGA-ZJ-	TCGA-ZJ-	CESC	67	FEMALE	[Unknown]		Stage IIB	Cervical S	G2	2010	[Unknowr	-24497	Alive		0							[Unknown]		0	0	0	0			0	0
TCGA-ZJ-	TCGA-ZJ-	CESC	43	FEMALE	[Unknown]		Stage IIB	Cervical Squamous C		2010	[Unknowr	-16007	Alive		0							[Unknown]		0	0	0	0			0	0
TCGA-ZJ-	TCGA-ZJ-	CESC	34	FEMALE	WHITE		Stage IB2	Cervical Squamous C		2010	[Unknowr	-12701	Alive		0							[Unknown]		0	0	0	0			0	0
TCGA-ZJ-	TCGA-ZJ-	CESC	54	FEMALE	WHITE		Stage IIIB	Cervical S	G2	2009	[Unknowr	-19847	Alive		0							[Unknown]		0	0	0	0			0	0
TCGA-ZJ-	TCGA-ZJ-	CESC	51	FEMALE	WHITE		Stage IIB	Cervical S	G2	2010	[Unknowr	-18773	Alive		5							[Unknown]		0	5	0	5			0	5
TCGA-ZJ-	TCGA-ZJ-	CESC	48	FEMALE	[Unknown]		Stage IIIB	Cervical Squamous C		2006	[Unknowr	-17869	Alive		0							[Unknown]		0	0	0	0			0	0
TCGA-ZJ-	TCGA-ZJ-	CESC	25	FEMALE	WHITE		Stage IIB	Cervical Squamous C		2006	[Unknowr	-9486	Alive		0							[Unknown]		0	0	0	0			0	0
TCGA-ZX	TCGA-ZX	CESC	64	FEMALE	WHITE		Stage IIIB	Cervical S	G2	2013	Post (prio	-23440	Alive	WITH TUM	119									0	119	0	119			0	119
TCGA-3X	TCGA-3X	CHOL	72	MALE	ASIAN	Stage I		Cholangic	G2	2010		-26349	Dead	WITH TUMOR		339		Locoregic	Liver		216		R0	1	339	1	339	1	216	1	216
TCGA-3X	TCGA-3X	CHOL	50	FEMALE	WHITE	Stage II		Cholangic	G2	2012		-18303	Dead	WITH TUMOR		445		Extrahepa	Bone		264		R0	1	445	1	445	1	264	1	264
TCGA-3X	TCGA-3X	CHOL	70	FEMALE	WHITE	Stage IVB		Cholangic	G1	2012		-25819	Alive		402								R0	0	402	0	402			0	402
TCGA-3X	TCGA-3X	CHOL	72	FEMALE	WHITE	Stage I		Cholangic	G3	2012		-26493	Alive	TUMOR F	709								R0	0	709	0	709	0	709	0	709
TCGA-3X	TCGA-3X	CHOL	60	MALE	ASIAN	Stage II		Cholangic	G2	2013		-21943	Alive	TUMOR F	650								R0	0	650	0	650	0	650	0	650
TCGA-4G	TCGA-4G	CHOL	71	FEMALE	WHITE	Stage II		Cholangic	G2	2012		-25989	Alive	WITH TUM	1177			Intrahepa	Liver		424		R1	0	1177	0	1177			1	424
TCGA-4G	TCGA-4G	CHOL	62	MALE	WHITE	Stage I		Cholangic	G2	2013		-22777	Alive	TUMOR F	420								R0	0	420	0	420	0	420	0	420
TCGA-W5	TCGA-W5	CHOL	62	FEMALE	WHITE	Stage I		Cholangic	G3	2008		-22933	Alive	TUMOR F	1976								R0	0	1976	0	1976	0	1976	0	1976
TCGA-W5	TCGA-W5	CHOL	70	FEMALE	WHITE	Stage III		Cholangic	G3	2012		-25775	Alive	TUMOR F	1077								R0	0	1077	0	1077	0	1077	0	1077
TCGA-W5	TCGA-W5	CHOL	66	MALE	WHITE	Stage I		Cholangic	G2	2005		-24388	Dead	WITH TUMOR		1939		New Prim	Other, sp	bladder	217		R0	1	1939	1	1939	0	1939	1	217
TCGA-W5	TCGA-W5	CHOL	66	MALE	WHITE	Stage I		Cholangic	G2	2005		-24388	Dead	WITH TUMOR		1939		New Prim	Other, sp	bladder	217		R0	1	1939	1	1939	0	1939	1	217
TCGA-W5	TCGA-W5	CHOL	57	MALE	WHITE	Stage I		Cholangic	G3	2006		-21118	Dead	WITH TUMOR		640		Intrahepa	Liver		215		R0	1	640	1	640	1	215	1	215
TCGA-W5	TCGA-W5	CHOL	68	MALE	WHITE	Stage II		Cholangic	G2	2006		-25069	Alive	TUMOR F	50								R0	0	50	0	50	0	50	0	50
TCGA-W5	TCGA-W5	CHOL	68	MALE	WHITE	Stage II		Cholangic	G2	2006		-25069	Alive	TUMOR F	50								R0	0	50	0	50	0	50	0	50
TCGA-W5	TCGA-W5	CHOL	77	FEMALE	WHITE	Stage I		Cholangic	G3	2006		-28367	Alive	TUMOR F	1542								R0	0	1542	0	1542	0	1542	0	1542
TCGA-W5	TCGA-W5	CHOL	77	FEMALE	WHITE	Stage I		Cholangic	G3	2006		-28367	Alive	TUMOR F	1542								R0	0	1542	0	1542	0	1542	0	1542
TCGA-W5	TCGA-W5	CHOL	64	FEMALE	WHITE	Stage II		Cholangic	G3	2008		-23560	Dead	WITH TUMOR		1220		Extrahepa	Bone		295		R0	1	1220	1	1220	1	295	1	295
TCGA-W5	TCGA-W5	CHOL	78	FEMALE	WHITE	Stage I		Cholangic	G3	2010		-28552	Dead	WITH TUMOR		627		New Prim	Lung		95		R0	1	627	1	627	0	627	1	95
TCGA-W5	TCGA-W5	CHOL	78	FEMALE	WHITE	Stage I		Cholangic	G3	2010		-28552	Dead	WITH TUMOR		627		New Prim	Lung		95		R0	1	627	1	627	0	627	1	95
TCGA-W5	TCGA-W5	CHOL	31	FEMALE	WHITE	Stage IVA		Cholangic	G3	2010		-11438	Dead	WITH TUMOR		924		Intrahepa	Liver		237		R0	1	924	1	924			1	237
TCGA-W5	TCGA-W5	CHOL	67	MALE	WHITE	Stage IVB		Cholangic	G4	2010		-24797	Dead			271							RX	1	271		271			0	271
TCGA-W5	TCGA-W5	CHOL	67	MALE	WHITE	Stage IVB		Cholangic	G4	2010		-24797	Dead			271							RX	1	271		271			0	271
TCGA-W5	TCGA-W5	CHOL	29	FEMALE	WHITE	Stage II		Cholangic	G3	2010		-10659	Alive	WITH TUM	1614								R1	0	1614	0	1614			0	1614
TCGA-W5	TCGA-W5	CHOL	82	MALE	WHITE	Stage I		Cholangic	G3	2010		-30039	Alive	TUMOR F	1153								R0	0	1153	0	1153	0	1153	0	1153
TCGA-W5	TCGA-W5	CHOL	82	MALE	WHITE	Stage I		Cholangic	G3	2010		-30039	Alive	TUMOR F	1153								R0	0	1153	0	1153	0	1153	0	1153
TCGA-W5	TCGA-W5	CHOL	71	MALE	WHITE	Stage I		Cholangic	G3	2010		-26139	Alive	TUMOR F	10								R0	0	10	0	10	0	10	0	10
TCGA-W5	TCGA-W5	CHOL	71	MALE	WHITE	Stage I		Cholangic	G3	2010		-26139	Alive	TUMOR F	10								R0	0	10	0	10	0	10	0	10
TCGA-W5	TCGA-W5	CHOL	60	MALE	WHITE	Stage I		Cholangic	G3	2011		-22090	Alive	TUMOR F	1449								R0	0	1449	0	1449	0	1449	0	1449
TCGA-W5	TCGA-W5	CHOL	75	FEMALE	WHITE	Stage I		Cholangic	G2	2012		-27535	Dead	WITH TUMOR		555		Intrahepa	Liver		40		R0	1	555	1	555	1	40	1	40
TCGA-W5	TCGA-W5	CHOL	75	FEMALE	WHITE	Stage I		Cholangic	G2	2012		-27535	Dead	WITH TUMOR		555		Intrahepa	Liver		40		R0	1	555	1	555	1	40	1	40
TCGA-W5	TCGA-W5	CHOL	51	FEMALE	WHITE	Stage IV		Cholangic	G3	2008		-18882	Dead	WITH TUMOR		1402		Intrahepa	Liver		362		R0	1	1402	1	1402			1	362
TCGA-W5	TCGA-W5	CHOL	55	FEMALE	WHITE	Stage I		Cholangic	G3	2011		-20285	Alive	TUMOR F	1471			Locoregic	Liver		377		R0	0	1471	0	1471	1	377	1	377
TCGA-W5	TCGA-W5	CHOL	81	MALE	WHITE	Stage II		Cholangic	G4	2010		-29594	Dead	WITH TUMOR		170							RX	1	170	1	170			1	170
TCGA-W6	TCGA-W6	CHOL	46	FEMALE	WHITE	Stage I		Cholangic	G2	2013		-16951	Alive	TUMOR F	808			Extrahepa	Non-regional / Dista		549		R0	0	808	0	808	1	549	1	549
TCGA-WD	TCGA-WD	CHOL	71	FEMALE	WHITE	Stage II		Cholangic	G2	2013		-26006	Dead	TUMOR FREE		21							RX	1	21	0	21			0	21
TCGA-YR	TCGA-YR	CHOL	52	MALE	WHITE	Stage IV		Cholangic	G2	2009		-19292	Dead	WITH TUMOR		26		New Prim	Other, sp	Adrenal g	17		R1	1	26	1	26			1	17
TCGA-ZD	TCGA-ZD	CHOL	73	FEMALE	ASIAN	Stage II		Cholangic	G3	2013		-26754	Dead	WITH TUMOR		169		Locoregic	Liver		59		R0	1	169	1	169	1	59	1	59
TCGA-ZH	TCGA-ZH	CHOL	74	FEMALE	WHITE	Stage IVA		Cholangic	G2	2012		-27279	Dead	WITH TUMOR		385		Locoregional Recurrence			49		R1	1	385	1	385			1	49
TCGA-ZH	TCGA-ZH	CHOL	59	FEMALE	BLACK OI	Stage I		Cholangic	G2	2011		-21743	Dead	WITH TUMOR		701		Locoregional Recurrence			198		R0	1	701	1	701	1	198	1	198
TCGA-ZH	TCGA-ZH	CHOL	58	MALE	WHITE	Stage I		Cholangic	G3	2010		-21503	Dead	WITH TUMOR		741		Locoregional Recurrence			281		R1	1	741	1	741			1	281
TCGA-ZH	TCGA-ZH	CHOL	69	MALE	WHITE	Stage IVB		Cholangic	G3	2012		-25268	Alive	WITH TUM	1229			Extrahepa	Lung		242		R0	0	1229	0	1229			1	242
TCGA-ZH	TCGA-ZH	CHOL	41	FEMALE	BLACK OI	Stage I		Cholangic	G2	2013		-14976	Alive	TUMOR F	519								R0	0	519	0	519	0	519	0	519
TCGA-ZH	TCGA-ZH	CHOL	73	MALE	WHITE	Stage I		Cholangic	G2	2013		-26744	Alive	TUMOR F	602								R0	0	602	0	602	0	602	0	602
TCGA-ZU	TCGA-ZU	CHOL	52	MALE	WHITE	Stage I		Cholangic	G3	2012		-19264	Dead	WITH TUMOR		98		Intrahepa	Liver		72		R0	1	98	1	98	1	72	1	72
TCGA-ZU	TCGA-ZU	CHOL	52	MALE	WHITE	Stage I		Cholangic	G3	2012		-19264	Dead	WITH TUMOR		98		Intrahepa	Liver		72		R0	1	98	1	98	1	72	1	72
TCGA-3L-	TCGA-3L-	COAD	61	FEMALE	BLACK OI	Stage I		Colon Adenocarcino		2013		-22379	Alive	TUMOR F	475							Complete Remission/Response		0	475	0	475	0	475	0	475
TCGA-4N	TCGA-4N	COAD	67	MALE	BLACK OI	Stage IIIB		Colon Adenocarcino		2013		-24523	Alive	WITH TUM	146							Stable Disease		0	146	0	146			0	146
TCGA-4T-	TCGA-4T-	COAD	42	FEMALE	BLACK OI	Stage IIA		Colon Mucinous Ade		2013		-15494	Alive	TUMOR F	385							Complete Remission/Response		0	385	0	385	0	385	0	385
TCGA-5M	TCGA-5M	COAD	74	MALE	BLACK OI	Stage IV		Colon Adenocarcino		2009		-27095	Dead	WITH TUMOR		49						Progressive Disease		1	49	1	49			1	49
TCGA-5M	TCGA-5M	COAD	40	FEMALE	BLACK OI	Stage IV		Colon Adenocarcino		2009		-14852	Dead	WITH TUMOR		290					219	Progressive Disease		1	290	1	290			1	219
TCGA-5M	TCGA-5M	COAD	76	MALE	BLACK OI	Stage IIA		Colon Adenocarcino		2011		-27870	Alive	WITH TUM	1200						810	Progressive Disease		0	1200	0	1200			1	810
TCGA-A6	TCGA-A6	COAD	45	MALE	WHITE	Stage IIA		Colon Adenocarcino		2009		-16512	Alive	TUMOR F	775									0	775	0	775			0	775
TCGA-A6	TCGA-A6	COAD	85	MALE	WHITE	Stage IV		Colon Adenocarcino		2009		-31329	Dead	WITH TUMOR		1331					535			1	1331	1	1331			1	535
TCGA-A6	TCGA-A6	COAD	85	MALE	WHITE	Stage IV		Colon Adenocarcino		2009		-31329	Dead	WITH TUMOR		1331					535			1	1331	1	1331			1	535
TCGA-A6	TCGA-A6	COAD	82	FEMALE	WHITE	Stage IIIB		Colon Adenocarcino		2009		-30237	Alive	TUMOR F	1419							Complete Remission/Response		0	1419	0	1419	0	1419	0	1419
TCGA-A6	TCGA-A6	COAD	82	FEMALE	WHITE	Stage IIIB		Colon Adenocarcino		2009		-30237	Alive	TUMOR F	1419							Complete Remission/Response		0	1419	0	1419	0	1419	0	1419
TCGA-A6	TCGA-A6	COAD	71	MALE	WHITE	Stage IV		Colon Mucinous Ade		2009		-26292	Alive	TUMOR F	1331						735	Complete Remission/Response		0	1331	0	1331			1	735
TCGA-A6	TCGA-A6	COAD	71	MALE	WHITE	Stage IV		Colon Mucinous Ade		2009		-26292	Alive	TUMOR F	1331						735	Complete Remission/Response		0	1331	0	1331			1	735
TCGA-A6	TCGA-A6	COAD	78	MALE	WHITE	Stage IIA		Colon Adenocarcino		2009		-28813	Alive	TUMOR F	1321							Complete Remission/Response		0	1321	0	1321	0	1321	0	1321
TCGA-A6	TCGA-A6	COAD	78	MALE	WHITE	Stage IIA		Colon Adenocarcino		2009		-28813	Alive	TUMOR F	1321							Complete Remission/Response		0	1321	0	1321	0	1321	0	1321
TCGA-A6	TCGA-A6	COAD	75	FEMALE	WHITE	Stage IIB		Colon Adenocarcino		2009		-27403	Dead	TUMOR FREE		1305						Complete Remission/Response		1	1305	0	1305	0	1305	0	1305
TCGA-A6	TCGA-A6	COAD	75	FEMALE	WHITE	Stage IIB		Colon Adenocarcino		2009		-27403	Dead	TUMOR FREE		1305						Complete Remission/Response		1	1305	0	1305	0	1305	0	1305
TCGA-A6	TCGA-A6	COAD	68	FEMALE	WHITE	Stage IIIC		Colon Adenocarcino		2009		-25143	Dead	TUMOR FREE		740								1	740	0	740			0	740
TCGA-A6	TCGA-A6	COAD	68	FEMALE	WHITE	Stage IIIC		Colon Adenocarcino		2009		-25143	Dead	TUMOR FREE		740								1	740	0	740			0	740
TCGA-A6	TCGA-A6	COAD	43	FEMALE	WHITE	Stage IIIB		Colon Adenocarcino		2009		-16030	Alive	TUMOR F	1286							Complete Remission/Response		0	1286	0	1286	0	1286	0	1286
TCGA-A6	TCGA-A6	COAD	43	FEMALE	WHITE	Stage IIIB		Colon Adenocarcino		2009		-16030	Alive	TUMOR F	1286							Complete Remission/Response		0	1286	0	1286	0	1286	0	1286
TCGA-A6	TCGA-A6	COAD	73	FEMALE	WHITE	Stage IIB		Colon Adenocarcino		2009		-26820	Alive	TUMOR F	1366							Complete Remission/Response		0	1366	0	1366	0	1366	0	1366
TCGA-A6	TCGA-A6	COAD	73	FEMALE	WHITE	Stage IIB		Colon Adenocarcino		2009		-26820	Alive	TUMOR F	1366							Complete Remission/Response		0	1366	0	1366	0	1366	0	1366
TCGA-A6	TCGA-A6	COAD	72	FEMALE	BLACK OI	Stage II		Colon Adenocarcino		2009		-26533	Alive	TUMOR F	1068							Complete Remission/Response		0	1068	0	1068	0	1068	0	1068
TCGA-A6	TCGA-A6	COAD	72	FEMALE	BLACK OI	Stage II		Colon Adenocarcino		2009		-26533	Alive	TUMOR F	1068							Complete Remission/Response		0	1068	0	1068	0	1068	0	1068
TCGA-A6	TCGA-A6	COAD	73	FEMALE	WHITE	Stage IIA		Colon Adenocarcino		2009		-26929	Alive	TUMOR F	1387						1150	Complete Remission/Response		0	1387	0	1387	1	1150	1	1150
TCGA-A6	TCGA-A6	COAD	73	FEMALE	WHITE	Stage IIA		Colon Adenocarcino		2009		-26929	Alive	TUMOR F	1387						1150	Complete Remission/Response		0	1387	0	1387	1	1150	1	1150
TCGA-A6	TCGA-A6	COAD	70	MALE	WHITE	Stage IV		Colon Adenocarcino		2009		-25683	Dead	WITH TUMOR		424					381			1	424	1	424			1	381
TCGA-A6	TCGA-A6	COAD	70	MALE	WHITE	Stage IV		Colon Adenocarcino		2009		-25683	Dead	WITH TUMOR		424					381			1	424	1	424			1	381
TCGA-A6	TCGA-A6	COAD	57	FEMALE	WHITE	Stage IV		Colon Adenocarcino		2009		-20871	Dead	WITH TUMOR		504					300			1	504	1	504			1	300
TCGA-A6	TCGA-A6	COAD	57	FEMALE	WHITE	Stage IV		Colon Adenocarcino		2009		-20871	Dead	WITH TUMOR		504					300			1	504	1	504			1	300
TCGA-A6	TCGA-A6	COAD	75	FEMALE	WHITE	Stage I		Colon Adenocarcino		2009		-27708	Alive	TUMOR F	1127			Locoregional Disease			976			0	1127	0	1127			1	976
TCGA-A6	TCGA-A6	COAD	75	FEMALE	WHITE	Stage I		Colon Adenocarcino		2009		-27708	Alive	TUMOR F	1127			Locoregional Disease			976			0	1127	0	1127			1	976
TCGA-A6	TCGA-A6	COAD	48	FEMALE	BLACK OI	Stage IIA		Colon Adenocarcino		2009		-17757	Alive	TUMOR F	1133			Locoregional Disease			948			0	1133	0	1133			1	948
TCGA-A6	TCGA-A6	COAD	48	FEMALE	BLACK OI	Stage IIA		Colon Adenocarcino		2009		-17757	Alive	TUMOR F	1133			Locoregional Disease			948			0	1133	0	1133			1	948
TCGA-A6	TCGA-A6	COAD	81	FEMALE	WHITE	Stage IIA		Colon Adenocarcino		2009		-29623	Dead	TUMOR FREE		1126						Complete Remission/Response		1	1126	0	1126	0	1126	0	1126
TCGA-A6	TCGA-A6	COAD	81	FEMALE	WHITE	Stage IIA		Colon Adenocarcino		2009		-29623	Dead	TUMOR FREE		1126						Complete Remission/Response		1	1126	0	1126	0	1126	0	1126
TCGA-A6	TCGA-A6	COAD	53	FEMALE	WHITE	Stage IIIC		Colon Adenocarcino		2009		-19624	Alive	TUMOR F	1054									0	1054	0	1054			0	1054
TCGA-A6	TCGA-A6	COAD	53	FEMALE	WHITE	Stage IIIC		Colon Adenocarcino		2009		-19624	Alive	TUMOR F	1054									0	1054	0	1054			0	1054
TCGA-A6	TCGA-A6	COAD	73	MALE	WHITE	Stage IIA		Colon Adenocarcino		2009		-26666	Alive	TUMOR F	1014									0	1014	0	1014			0	1014
TCGA-A6	TCGA-A6	COAD	73	MALE	WHITE	Stage IIA		Colon Adenocarcino		2009		-26666	Alive	TUMOR F	1014									0	1014	0	1014			0	1014
TCGA-A6	TCGA-A6	COAD	71	FEMALE	WHITE	Stage IIB		Colon Mucinous Ade		2009		-26274	Alive	TUMOR F	996									0	996	0	996			0	996
TCGA-A6	TCGA-A6	COAD	71	FEMALE	WHITE	Stage IIB		Colon Mucinous Ade		2009		-26274	Alive	TUMOR F	996									0	996	0	996			0	996
TCGA-A6	TCGA-A6	COAD	62	MALE	WHITE	Stage IIA		Colon Adenocarcino		2009		-22999	Alive	TUMOR F	1111							Complete Remission/Response		0	1111	0	1111	0	1111	0	1111
TCGA-A6	TCGA-A6	COAD	62	MALE	WHITE	Stage IIA		Colon Adenocarcino		2009		-22999	Alive	TUMOR F	1111							Complete Remission/Response		0	1111	0	1111	0	1111	0	1111
TCGA-A6	TCGA-A6	COAD	79	MALE	WHITE	Stage IIA		Colon Adenocarcino		2010		-29097	Dead	TUMOR FREE		442					364			1	442	0	442			1	364
TCGA-A6	TCGA-A6	COAD	57	FEMALE	WHITE	Stage IIIB		Colon Adenocarcino		2010		-21064	Alive	TUMOR F	987							Complete Remission/Response		0	987	0	987	0	987	0	987
TCGA-A6	TCGA-A6	COAD	57	FEMALE	WHITE	Stage IIIB		Colon Adenocarcino		2010		-21064	Alive	TUMOR F	987							Complete Remission/Response		0	987	0	987	0	987	0	987
TCGA-A6	TCGA-A6	COAD	74	MALE	WHITE	Stage I		Colon Adenocarcino		2010		-27184	Alive	TUMOR F	1001							Complete Remission/Response		0	1001	0	1001	0	1001	0	1001
TCGA-A6	TCGA-A6	COAD	65	MALE	BLACK OI	Stage IIIB		Colon Adenocarcino		2010		-23920	Alive	TUMOR F	962							Complete Remission/Response		0	962	0	962	0	962	0	962
TCGA-A6	TCGA-A6	COAD	82	MALE	WHITE	Stage I		Colon Adenocarcino		2010		-30028	Alive	TUMOR F	926							Complete Remission/Response		0	926	0	926	0	926	0	926
TCGA-A6	TCGA-A6	COAD	82	MALE	WHITE	Stage I		Colon Adenocarcino		2010		-30028	Alive	TUMOR F	926							Complete Remission/Response		0	926	0	926	0	926	0	926
TCGA-A6	TCGA-A6	COAD	73	MALE	BLACK OI	Stage IIIC		Colon Adenocarcino		2010		-26803	Alive	TUMOR F	888							Complete Remission/Response		0	888	0	888	0	888	0	888
TCGA-A6	TCGA-A6	COAD	80	FEMALE	BLACK OI	Stage IIA		Colon Adenocarcino		2010		-29446	Alive	TUMOR F	1020							Complete Remission/Response		0	1020	0	1020	0	1020	0	1020
TCGA-A6	TCGA-A6	COAD	46	MALE	WHITE	Stage IVA		Colon Adenocarcino		2010		-16873	Alive	WITH TUM	718						495			0	718	0	718			1	495
TCGA-A6	TCGA-A6	COAD	46	MALE	WHITE	Stage IVA		Colon Adenocarcino		2010		-16873	Alive	WITH TUM	718						495			0	718	0	718			1	495
TCGA-A6	TCGA-A6	COAD	80	MALE	BLACK OI	Stage IIIC		Colon Adenocarcino		2010		-29426	Alive	WITH TUM	672						183			0	672	0	672			1	183
TCGA-A6	TCGA-A6	COAD	84	FEMALE	WHITE	Stage IIA		Colon Adenocarcino		2010		-30915	Alive	TUMOR F	671						529			0	671	0	671			1	529
TCGA-A6	TCGA-A6	COAD	84	FEMALE	WHITE	Stage IIA		Colon Adenocarcino		2010		-30915	Alive	TUMOR F	671						529			0	671	0	671			1	529
TCGA-A6	TCGA-A6	COAD	78	MALE	WHITE	Stage IIC		Colon Adenocarcino		2010		-28514	Alive	WITH TUM	995						509	Complete Remission/Response		0	995	0	995	1	509	1	509
TCGA-A6	TCGA-A6	COAD	40	FEMALE	WHITE	Stage IIIB		Colon Adenocarcino		2010		-14756	Alive	TUMOR F	887							Complete Remission/Response		0	887	0	887	0	887	0	887
TCGA-A6	TCGA-A6	COAD	40	FEMALE	WHITE	Stage IIIB		Colon Adenocarcino		2010		-14756	Alive	TUMOR F	887							Complete Remission/Response		0	887	0	887	0	887	0	887
TCGA-A6	TCGA-A6	COAD	55	MALE	WHITE	Stage IIIB		Colon Adenocarcino		2010		-20095	Alive	TUMOR F	824							Complete Remission/Response		0	824	0	824	0	824	0	824
TCGA-A6	TCGA-A6	COAD	61	MALE	WHITE	Stage I		Colon Adenocarcino		2010		-22523	Alive	TUMOR F	685									0	685	0	685			0	685
TCGA-A6	TCGA-A6	COAD	62	MALE	WHITE	Stage IIA		Colon Adenocarcino		2011		-22835	Alive	TUMOR F	734							Complete Remission/Response		0	734	0	734	0	734	0	734
TCGA-A6	TCGA-A6	COAD	31	MALE	BLACK OI	Stage IIA		Colon Adenocarcino		2011		-11391	Alive	TUMOR F	255									0	255	0	255			0	255
TCGA-A6	TCGA-A6	COAD	56	FEMALE	WHITE	Stage IVA		Colon Adenocarcino		2011		-20485	Alive	WITH TUM	763						654	Complete Remission/Response		0	763	0	763			1	654
TCGA-A6	TCGA-A6	COAD	56	MALE	WHITE	Stage IVA		Colon Adenocarcino		2010		-20641	Alive	WITH TUM	766						686			0	766	0	766			1	686
TCGA-A6	TCGA-A6	COAD	66	MALE	WHITE	Stage IIIB		Colon Adenocarcino		2010		-24237	Alive	TUMOR F	735							Complete Remission/Response		0	735	0	735	0	735	0	735
TCGA-A6	TCGA-A6	COAD	69	FEMALE	BLACK OI	Stage IIA		Colon Adenocarcino		2010		-25516	Alive	TUMOR F	627									0	627	0	627			0	627
TCGA-A6	TCGA-A6	COAD	55	FEMALE	WHITE	Stage IIIB		Colon Adenocarcino		2010		-20110	Alive	TUMOR F	662									0	662	0	662			0	662
TCGA-A6	TCGA-A6	COAD	59	MALE	BLACK OI	Stage IVA		Colon Adenocarcino		2011		-21638	Alive	WITH TUM	751									0	751	0	751			0	751
TCGA-A6	TCGA-A6	COAD	82	MALE	WHITE	Stage I		Colon Adenocarcino		2011		-29996	Alive	TUMOR F	742							Complete Remission/Response		0	742	0	742	0	742	0	742
TCGA-A6	TCGA-A6	COAD	65	FEMALE	WHITE	Stage IIIB		Colon Adenocarcino		2011		-23953	Alive	TUMOR F	726							Complete Remission/Response		0	726	0	726	0	726	0	726
TCGA-A6	TCGA-A6	COAD	74	MALE	WHITE	Stage IIA		Colon Mucinous Ade		2011		-27250	Alive	TUMOR F	612							Complete Remission/Response		0	612	0	612	0	612	0	612
TCGA-A6	TCGA-A6	COAD	43	MALE	WHITE	Stage IIIC		Colon Mucinous Ade		2011		-15842	Alive	TUMOR F	598							Complete Remission/Response		0	598	0	598	0	598	0	598
TCGA-A6	TCGA-A6	COAD	82	MALE	WHITE	Stage IIB		Colon Adenocarcino		2011		-30012	Alive	TUMOR F	617							Complete Remission/Response		0	617	0	617	0	617	0	617
TCGA-A6	TCGA-A6	COAD	34	FEMALE	BLACK OI	Stage IIIC		Colon Mucinous Ade		2008		-12526	Dead	WITH TUMOR		494					301	Progressive Disease		1	494	1	494			1	301
TCGA-A6	TCGA-A6	COAD	55	FEMALE	BLACK OI	Stage IIIB		Colon Mucinous Ade		2007		-20181	Dead	WITH TUMOR		758					257	Progressive Disease		1	758	1	758			1	257
TCGA-A6	TCGA-A6	COAD	56	MALE	BLACK OI	Stage IV		Colon Adenocarcino		2007		-20493	Dead	WITH TUMOR		1881		Metastatic			612	[Unknown]		1	1881	1	1881			1	612
TCGA-A6	TCGA-A6	COAD	57	MALE	BLACK OI	Stage IIIB		Colon Adenocarcino		2008		-20903	Dead	TUMOR FREE		1711					1678	Complete Remission/Response		1	1711	0	1711	1	1678	1	1678
TCGA-A6	TCGA-A6	COAD	59	MALE	BLACK OI	Stage IIIB		Colon Adenocarcino		2013		-21554	Alive	TUMOR F	293							Complete Remission/Response		0	293	0	293	0	293	0	293
TCGA-AA	TCGA-AA	COAD	58	MALE		Stage IV		Colon Adenocarcino		2002		-21519	Dead	WITH TUMOR		153								1	153	1	153			1	153
TCGA-AA	TCGA-AA	COAD	58	MALE		Stage IV		Colon Adenocarcino		2002		-21519	Dead	WITH TUMOR		153								1	153	1	153			1	153
TCGA-AA	TCGA-AA	COAD	75	MALE		Stage II		Colon Adenocarcino		2002		-27606	Dead	WITH TUMOR		214						[Unknown]		1	214	1	214			1	214
TCGA-AA	TCGA-AA	COAD	75	MALE		Stage II		Colon Adenocarcino		2002		-27606	Dead	WITH TUMOR		214						[Unknown]		1	214	1	214			1	214
TCGA-AA	TCGA-AA	COAD	90	FEMALE		Stage II		Colon Adenocarcino		2002		-32871	Dead	TUMOR FREE		92						[Unknown]		1	92	0	92			0	92
TCGA-AA	TCGA-AA	COAD	90	FEMALE		Stage II		Colon Adenocarcino		2002		-32871	Dead	TUMOR FREE		92						[Unknown]		1	92	0	92			0	92
TCGA-AA	TCGA-AA	COAD	55	MALE		Stage IV		Colon Adenocarcino		2002		-20089	Alive	WITH TUM	31									0	31	0	31			0	31
TCGA-AA	TCGA-AA	COAD	55	MALE		Stage IV		Colon Adenocarcino		2002		-20089	Alive	WITH TUM	31									0	31	0	31			0	31
TCGA-AA	TCGA-AA	COAD	79	MALE		Stage I		Colon Adenocarcino		2002		-28886	Alive	TUMOR F	1127							Complete Remission/Response		0	1127	0	1127	0	1127	0	1127
TCGA-AA	TCGA-AA	COAD	79	MALE		Stage I		Colon Adenocarcino		2002		-28886	Alive	TUMOR F	1127							Complete Remission/Response		0	1127	0	1127	0	1127	0	1127
TCGA-AA	TCGA-AA	COAD	83	FEMALE		Stage II		Colon Adenocarcino		2002		-30438	Alive	WITH TUM	31									0	31	0	31			0	31
TCGA-AA	TCGA-AA	COAD	83	FEMALE		Stage II		Colon Adenocarcino		2002		-30438	Alive	WITH TUM	31									0	31	0	31			0	31
TCGA-AA	TCGA-AA	COAD	73	MALE		Stage I		Colon Adenocarcino		2003		-26724	Alive	WITH TUM	1065							Complete Remission/Response		0	1065	0	1065	0	1065	0	1065
TCGA-AA	TCGA-AA	COAD	73	MALE		Stage I		Colon Adenocarcino		2003		-26724	Alive	WITH TUM	1065							Complete Remission/Response		0	1065	0	1065	0	1065	0	1065
TCGA-AA	TCGA-AA	COAD	77	MALE		Stage I		Colon Adenocarcino		2004		-28275	Alive	WITH TUM	1765							[Unknown]		0	1765	0	1765			0	1765
TCGA-AA	TCGA-AA	COAD	77	MALE		Stage I		Colon Adenocarcino		2004		-28275	Alive	WITH TUM	1765							[Unknown]		0	1765	0	1765			0	1765
TCGA-AA	TCGA-AA	COAD	54	FEMALE		Stage II		Colon Adenocarcino		2005		-19786	Alive	TUMOR F	1915							Complete Remission/Response		0	1915	0	1915	0	1915	0	1915
TCGA-AA	TCGA-AA	COAD	54	FEMALE		Stage II		Colon Adenocarcino		2005		-19786	Alive	TUMOR F	1915							Complete Remission/Response		0	1915	0	1915	0	1915	0	1915
TCGA-AA	TCGA-AA	COAD	70	MALE		Stage II		Colon Adenocarcino		2005		-25902	Alive	TUMOR F	1946							Complete Remission/Response		0	1946	0	1946	0	1946	0	1946
TCGA-AA	TCGA-AA	COAD	70	MALE		Stage II		Colon Adenocarcino		2005		-25902	Alive	TUMOR F	1946							Complete Remission/Response		0	1946	0	1946	0	1946	0	1946
TCGA-AA	TCGA-AA	COAD	64	MALE		Stage II		Colon Adenocarcino		2005		-23407	Alive	TUMOR F	212						59	Complete Remission/Response		0	212	0	212	1	59	1	59
TCGA-AA	TCGA-AA	COAD	64	MALE		Stage II		Colon Adenocarcino		2005		-23407	Alive	TUMOR F	212						59	Complete Remission/Response		0	212	0	212	1	59	1	59
TCGA-AA	TCGA-AA	COAD	81	FEMALE		Stage I		Colon Adenocarcino		2006		-29738	Alive	TUMOR F	31							[Unknown]		0	31	0	31			0	31
TCGA-AA	TCGA-AA	COAD	81	FEMALE		Stage I		Colon Adenocarcino		2006		-29738	Alive	TUMOR F	31							[Unknown]		0	31	0	31			0	31
TCGA-AA	TCGA-AA	COAD	74	FEMALE		Stage III		Colon Mucinous Ade		2007		-27303	Dead	WITH TUMOR		396						[Unknown]		1	396	1	396			1	396
TCGA-AA	TCGA-AA	COAD	74	FEMALE		Stage III		Colon Mucinous Ade		2007		-27303	Dead	WITH TUMOR		396						[Unknown]		1	396	1	396			1	396
TCGA-AA	TCGA-AA	COAD	60	MALE		Stage IIA		Colon Adenocarcino		2007		-22099	Alive	TUMOR F	1186						821	Complete Remission/Response		0	1186	0	1186	1	821	1	821
TCGA-AA	TCGA-AA	COAD	60	MALE		Stage IIA		Colon Adenocarcino		2007		-22099	Alive	TUMOR F	1186						821	Complete Remission/Response		0	1186	0	1186	1	821	1	821
TCGA-AA	TCGA-AA	COAD	81	FEMALE		Stage IIA		Colon Adenocarcino		2007		-29769	Alive	WITH TUM	31									0	31	0	31			0	31
TCGA-AA	TCGA-AA	COAD	81	FEMALE		Stage IIA		Colon Adenocarcino		2007		-29769	Alive	WITH TUM	31									0	31	0	31			0	31
TCGA-AA	TCGA-AA	COAD	63	MALE		Stage III		Colon Adenocarcino		2007		-23131	Alive	TUMOR F	276							Complete Remission/Response		0	276	0	276	0	276	0	276
TCGA-AA	TCGA-AA	COAD	63	MALE		Stage III		Colon Adenocarcino		2007		-23131	Alive	TUMOR F	276							Complete Remission/Response		0	276	0	276	0	276	0	276
TCGA-AA	TCGA-AA	COAD	86	FEMALE		Stage II		Colon Adenocarcino		2007		-31531	Alive	WITH TUM	731							[Unknown]		0	731	0	731			0	731
TCGA-AA	TCGA-AA	COAD	86	FEMALE		Stage II		Colon Adenocarcino		2007		-31531	Alive	WITH TUM	731							[Unknown]		0	731	0	731			0	731
TCGA-AA	TCGA-AA	COAD	87	MALE		Stage II		Colon Adenocarcino		2007		-31805	Alive	WITH TUMOR										0		0				0	
TCGA-AA	TCGA-AA	COAD	87	MALE		Stage II		Colon Adenocarcino		2007		-31805	Alive	WITH TUMOR										0		0				0	
TCGA-AA	TCGA-AA	COAD	67	MALE		Stage IIA		Colon Adenocarcino		2007		-24653	Alive	TUMOR F	1127							Complete Remission/Response		0	1127	0	1127	0	1127	0	1127
TCGA-AA	TCGA-AA	COAD	67	MALE		Stage IIA		Colon Adenocarcino		2007		-24653	Alive	TUMOR F	1127							Complete Remission/Response		0	1127	0	1127	0	1127	0	1127
TCGA-AA	TCGA-AA	COAD	85	MALE		Stage II		Colon Adenocarcino		2007		-31197	Alive	TUMOR F	1096							Complete Remission/Response		0	1096	0	1096	0	1096	0	1096
TCGA-AA	TCGA-AA	COAD	85	MALE		Stage II		Colon Adenocarcino		2007		-31197	Alive	TUMOR F	1096							Complete Remission/Response		0	1096	0	1096	0	1096	0	1096
TCGA-AA	TCGA-AA	COAD	90	MALE		Stage IIIB		Colon Adenocarcino		2007		-32871	Alive	TUMOR F	245							Partial Remission/Response		0	245	0	245			0	245
TCGA-AA	TCGA-AA	COAD	90	MALE		Stage IIIB		Colon Adenocarcino		2007		-32871	Alive	TUMOR F	245							Partial Remission/Response		0	245	0	245			0	245
TCGA-AA	TCGA-AA	COAD	57	MALE		Stage I		Colon Adenocarcino		2007		-21123	Alive	TUMOR F	580							Complete Remission/Response		0	580	0	580	0	580	0	580
TCGA-AA	TCGA-AA	COAD	57	MALE		Stage I		Colon Adenocarcino		2007		-21123	Alive	TUMOR F	580							Complete Remission/Response		0	580	0	580	0	580	0	580
TCGA-AA	TCGA-AA	COAD	90	FEMALE		Stage IIA		Colon Adenocarcino		2007		-32871	Alive	WITH TUM	0									0	0	0	0			0	0
TCGA-AA	TCGA-AA	COAD	90	FEMALE		Stage IIA		Colon Adenocarcino		2007		-32871	Alive	WITH TUM	0									0	0	0	0			0	0
TCGA-AA	TCGA-AA	COAD	78	FEMALE		Stage IIIC		Colon Adenocarcino		2007		-28609	Dead	WITH TUMOR		0								1	0	1	0			1	0
TCGA-AA	TCGA-AA	COAD	78	FEMALE		Stage IIIC		Colon Adenocarcino		2007		-28609	Dead	WITH TUMOR		0								1	0	1	0			1	0
TCGA-AA	TCGA-AA	COAD	80	MALE		Stage I		Colon Adenocarcino		2007		-29402	Alive	TUMOR F	580							Complete Remission/Response		0	580	0	580	0	580	0	580
TCGA-AA	TCGA-AA	COAD	75	FEMALE		Stage IIA		Colon Adenocarcino		2007		-27514	Alive	TUMOR F	1035							Complete Remission/Response		0	1035	0	1035	0	1035	0	1035
TCGA-AA	TCGA-AA	COAD	75	FEMALE		Stage IIA		Colon Adenocarcino		2007		-27514	Alive	TUMOR F	1035							Complete Remission/Response		0	1035	0	1035	0	1035	0	1035
TCGA-AA	TCGA-AA	COAD	63	MALE		Stage IIA		Colon Adenocarcino		2007		-23133	Alive	WITH TUM	882							[Unknown]		0	882	0	882			0	882
TCGA-AA	TCGA-AA	COAD	63	MALE		Stage IIA		Colon Adenocarcino		2007		-23133	Alive	WITH TUM	882							[Unknown]		0	882	0	882			0	882
TCGA-AA	TCGA-AA	COAD	78	FEMALE		Stage IIA		Colon Adenocarcino		2007		-28489	Alive	TUMOR F	882							Complete Remission/Response		0	882	0	882	0	882	0	882
TCGA-AA	TCGA-AA	COAD	78	FEMALE		Stage IIA		Colon Adenocarcino		2007		-28489	Alive	TUMOR F	882							Complete Remission/Response		0	882	0	882	0	882	0	882
TCGA-AA	TCGA-AA	COAD	54	FEMALE		Stage I		Colon Adenocarcino		2008		-19752	Alive	TUMOR F	791							Complete Remission/Response		0	791	0	791	0	791	0	791
TCGA-AA	TCGA-AA	COAD	69	MALE		Stage IIIC		Colon Adenocarcino		2008		-25324	Alive	TUMOR F	395							Complete Remission/Response		0	395	0	395	0	395	0	395
TCGA-AA	TCGA-AA	COAD	84	MALE		Stage I		Colon Mucinous Ade		2008		-30772	Alive	TUMOR F	30							[Unknown]		0	30	0	30			0	30
TCGA-AA	TCGA-AA	COAD	68	MALE		Stage I		Colon Adenocarcino		2008		-24959	Alive	TUMOR F	426							Complete Remission/Response		0	426	0	426	0	426	0	426
TCGA-AA	TCGA-AA	COAD	71	FEMALE		Stage IIIC		Colon Adenocarcino		2008		-26175	Alive	TUMOR F	1034							Complete Remission/Response		0	1034	0	1034	0	1034	0	1034
TCGA-AA	TCGA-AA	COAD	69	MALE		Stage I		Colon Adenocarcino		2008		-25415	Alive	TUMOR F	639							Complete Remission/Response		0	639	0	639	0	639	0	639
TCGA-AA	TCGA-AA	COAD	85	MALE		Stage IIIC		Colon Adenocarcino		2008		-31290	Dead	TUMOR FREE		396					122	Complete Remission/Response		1	396	0	396	1	122	1	122
TCGA-AA	TCGA-AA	COAD	61	FEMALE		Stage I		Colon Adenocarcino		2008		-22524	Alive	TUMOR F	730							Complete Remission/Response		0	730	0	730	0	730	0	730
TCGA-AA	TCGA-AA	COAD	62	FEMALE		Stage IIA		Colon Adenocarcino		2008		-22799	Alive	TUMOR F	546							Complete Remission/Response		0	546	0	546	0	546	0	546
TCGA-AA	TCGA-AA	COAD	81	FEMALE		Stage IIA		Colon Mucinous Ade		2008		-29617	Alive	TUMOR F	911						91	Complete Remission/Response		0	911	0	911	1	91	1	91
TCGA-AA	TCGA-AA	COAD	78	MALE		Stage I				2008		-28704	Alive	WITH TUM	700							[Unknown]		0	700	0	700			0	700
TCGA-AA	TCGA-AA	COAD	70	MALE		Stage I		Colon Adenocarcino		2008		-25752	Alive	TUMOR F	638						92	Complete Remission/Response		0	638	0	638	1	92	1	92
TCGA-AA	TCGA-AA	COAD	72	FEMALE		Stage IIIC		Colon Adenocarcino		2009		-26359	Alive	TUMOR F	608							Complete Remission/Response		0	608	0	608	0	608	0	608
TCGA-AA	TCGA-AA	COAD	72	MALE		Stage IIA		Colon Adenocarcino		2009		-26420	Alive	TUMOR F	424							Complete Remission/Response		0	424	0	424	0	424	0	424
TCGA-AA	TCGA-AA	COAD	82	MALE		Stage IIIC		Colon Adenocarcino		2008		-30254	Alive	WITH TUM	608							[Unknown]		0	608	0	608			0	608
TCGA-AA	TCGA-AA	COAD	68	MALE		Stage II		Colon Adenocarcino		2005		-24896	Alive	TUMOR F	1856							Complete Remission/Response		0	1856	0	1856	0	1856	0	1856
TCGA-AA	TCGA-AA	COAD	68	MALE		Stage II		Colon Adenocarcino		2005		-24896	Alive	TUMOR F	1856							Complete Remission/Response		0	1856	0	1856	0	1856	0	1856
TCGA-AA	TCGA-AA	COAD	51	FEMALE		Stage II		Colon Adenocarcino		2005		-18932	Alive	TUMOR F	2375							Complete Remission/Response		0	2375	0	2375	0	2375	0	2375
TCGA-AA	TCGA-AA	COAD	51	FEMALE		Stage II		Colon Adenocarcino		2005		-18932	Alive	TUMOR F	2375							Complete Remission/Response		0	2375	0	2375	0	2375	0	2375
TCGA-AA	TCGA-AA	COAD	80	FEMALE		Stage IV		Colon Adenocarcino		2005		-29554	Alive	WITH TUM	184							Partial Remission/Response		0	184	0	184			0	184
TCGA-AA	TCGA-AA	COAD	80	FEMALE		Stage IV		Colon Adenocarcino		2005		-29554	Alive	WITH TUM	184							Partial Remission/Response		0	184	0	184			0	184
TCGA-AA	TCGA-AA	COAD	42	MALE		Stage II		Colon Adenocarcino		2005		-15675	Alive	TUMOR F	212							Complete Remission/Response		0	212	0	212	0	212	0	212
TCGA-AA	TCGA-AA	COAD	42	MALE		Stage II		Colon Adenocarcino		2005		-15675	Alive	TUMOR F	212							Complete Remission/Response		0	212	0	212	0	212	0	212
TCGA-AA	TCGA-AA	COAD	74	FEMALE		Stage II		Colon Adenocarcino		2005		-27363	Alive	WITH TUM	1643							[Unknown]		0	1643	0	1643			0	1643
TCGA-AA	TCGA-AA	COAD	68	MALE		Stage III		Colon Adenocarcino		2005		-25020	Dead	WITH TUMOR		61								1	61	1	61			1	61
TCGA-AA	TCGA-AA	COAD	36	FEMALE		Stage I		Colon Adenocarcino		2005		-13302	Alive	TUMOR F	426							Complete Remission/Response		0	426	0	426	0	426	0	426
TCGA-AA	TCGA-AA	COAD	90	MALE		Stage III		Colon Adenocarcino		2006		-32873	Alive	TUMOR F	28							[Unknown]		0	28	0	28			0	28
TCGA-AA	TCGA-AA	COAD	53	FEMALE		Stage II		Colon Adenocarcino		2006		-19509	Alive	TUMOR F	1522							Complete Remission/Response		0	1522	0	1522	0	1522	0	1522
TCGA-AA	TCGA-AA	COAD	84	MALE		Stage II		Colon Adenocarcino		2006		-30801	Alive	TUMOR F	1431							Complete Remission/Response		0	1431	0	1431	0	1431	0	1431
TCGA-AA	TCGA-AA	COAD	60	FEMALE		Stage III		Colon Adenocarcino		2006		-22219	Alive	TUMOR F	1430							Complete Remission/Response		0	1430	0	1430	0	1430	0	1430
TCGA-AA	TCGA-AA	COAD	59	MALE		Stage IV		Colon Adenocarcino		2006		-21611	Alive	WITH TUM	457							Progressive Disease		0	457	0	457			0	457
TCGA-AA	TCGA-AA	COAD	67	FEMALE		Stage IV		Colon Adenocarcino		2006		-24655	Dead	WITH TUMOR		335						Progressive Disease		1	335	1	335			1	335
TCGA-AA	TCGA-AA	COAD	77	FEMALE		Stage III		Colon Adenocarcino		2006		-28399	Alive	TUMOR F	182							Complete Remission/Response		0	182	0	182	0	182	0	182
TCGA-AA	TCGA-AA	COAD	65	FEMALE		Stage IV		Colon Mucinous Ade		2007		-23741	Alive	WITH TUM	0									0	0	0	0			0	0
TCGA-AA	TCGA-AA	COAD	69	MALE		Stage II		Colon Adenocarcino		2007		-25506	Alive	TUMOR F	1127							Complete Remission/Response		0	1127	0	1127	0	1127	0	1127
TCGA-AA	TCGA-AA	COAD	80	MALE		Stage IV		Colon Adenocarcino		2007		-29220	Alive	WITH TUM	578							Progressive Disease		0	578	0	578			0	578
TCGA-AA	TCGA-AA	COAD	47	FEMALE		Stage IV		Colon Mucinous Ade		2008		-17167	Dead	WITH TUMOR		1095					396	Progressive Disease		1	1095	1	1095			1	396
TCGA-AA	TCGA-AA	COAD	77	FEMALE		Stage IV		Colon Adenocarcino		2008		-28459	Alive	WITH TUM	0									0	0	0	0			0	0
TCGA-AA	TCGA-AA	COAD	63	FEMALE		Stage IV		Colon Adenocarcino		2009		-23164	Alive	WITH TUM	0									0	0	0	0			0	0
TCGA-AA	TCGA-AA	COAD	75	FEMALE		Stage IV		Colon Adenocarcino		2009		-27667	Dead	WITH TUMOR		153						[Unknown]		1	153	1	153			1	153
TCGA-AA	TCGA-AA	COAD	77	MALE		Stage II		Colon Adenocarcino		2003		-28367	Alive	WITH TUM	2587							[Unknown]		0	2587	0	2587			0	2587
TCGA-AA	TCGA-AA	COAD	77	MALE		Stage II		Colon Adenocarcino		2003		-28367	Alive	WITH TUM	2587							[Unknown]		0	2587	0	2587			0	2587
TCGA-AA	TCGA-AA	COAD	80	FEMALE		Stage IIA		Colon Adenocarcino		2008		-29465	Alive	TUMOR F	821							Complete Remission/Response		0	821	0	821	0	821	0	821
TCGA-AA	TCGA-AA	COAD	65	MALE		Stage III		Colon Adenocarcino		2004		-23831	Alive	WITH TUM	0									0	0	0	0			0	0
TCGA-AA	TCGA-AA	COAD	65	MALE		Stage III		Colon Adenocarcino		2004		-23831	Alive	WITH TUM	0									0	0	0	0			0	0
TCGA-AA	TCGA-AA	COAD	68	MALE		Stage IV		Colon Adenocarcino		2005		-24927	Alive	TUMOR F	579							Partial Remission/Response		0	579	0	579			0	579
TCGA-AA	TCGA-AA	COAD	68	MALE		Stage IV		Colon Adenocarcino		2005		-24927	Alive	TUMOR F	579							Partial Remission/Response		0	579	0	579			0	579
TCGA-AA	TCGA-AA	COAD	77	MALE		Stage II		Colon Adenocarcino		2006		-28428	Dead	WITH TUMOR		579					153	[Unknown]		1	579	1	579			1	153
TCGA-AA	TCGA-AA	COAD	84	FEMALE		Stage III		Colon Adenocarcino		2007		-30893	Dead	WITH TUMOR		306						[Unknown]		1	306	1	306			1	306
TCGA-AA	TCGA-AA	COAD	82	FEMALE		Stage IIA		Colon Adenocarcino		2007		-29980	Alive	TUMOR F	1066						762	Complete Remission/Response		0	1066	0	1066	1	762	1	762
TCGA-AA	TCGA-AA	COAD	85	FEMALE		Stage IIA		Colon Adenocarcino		2007		-31380	Alive	WITH TUM	0									0	0	0	0			0	0
TCGA-AA	TCGA-AA	COAD	65	FEMALE		Stage IIA		Colon Adenocarcino		2007		-23922	Alive	TUMOR F	1005							Complete Remission/Response		0	1005	0	1005	0	1005	0	1005
TCGA-AA	TCGA-AA	COAD	78	FEMALE		Stage IIA		Colon Adenocarcino		2007		-28763	Dead	WITH TUMOR		30								1	30	1	30			1	30
TCGA-AA	TCGA-AA	COAD	41	FEMALE		Stage IIA		Colon Adenocarcino		2008		-15280	Alive	TUMOR F	761							Complete Remission/Response		0	761	0	761	0	761	0	761
TCGA-AA	TCGA-AA	COAD	81	FEMALE		Stage I		Colon Mucinous Ade		2008		-29706	Alive	TUMOR F	31							[Unknown]		0	31	0	31			0	31
TCGA-AA	TCGA-AA	COAD	66	MALE		Stage IIA		Colon Adenocarcino		2008		-24411	Alive	TUMOR F	547							Complete Remission/Response		0	547	0	547	0	547	0	547
TCGA-AA	TCGA-AA	COAD	63	FEMALE		Stage IIA		Colon Adenocarcino		2008		-23011	Alive	TUMOR F	485							Complete Remission/Response		0	485	0	485	0	485	0	485
TCGA-AA	TCGA-AA	COAD	67	MALE		Stage IIA		Colon Mucinous Ade		2008		-24655	Alive	TUMOR F	1186							Complete Remission/Response		0	1186	0	1186	0	1186	0	1186
TCGA-AA	TCGA-AA	COAD	66	MALE		Stage IIA		Colon Adenocarcino		2009		-24230	Alive	TUMOR F	1124							Complete Remission/Response		0	1124	0	1124	0	1124	0	1124
TCGA-AA	TCGA-AA	COAD	51	MALE		Stage IIIA		Colon Adenocarcino		2009		-18842	Alive	TUMOR F	1126						396	Complete Remission/Response		0	1126	0	1126	1	396	1	396

TCGA-AA	TCGA-AA	COAD	78	FEMALE		Stage IIIC	Colon Adenocarcino	2009	-28521	Alive	WITH TUM	454			365	Progressive Disease	0	454	0	454			1	365
TCGA-AA	TCGA-AA	COAD	86	FEMALE		Stage IIA	Colon Adenocarcino	2009	-31593	Dead	WITH TUMOR		0				1	0	1	0			1	0
TCGA-AA	TCGA-AA	COAD	74	FEMALE		Stage IIA	Colon Adenocarcino	2009	-27057	Alive	WITH TUM	518				[Unknown]	0	518	0	518			0	518
TCGA-AA	TCGA-AA	COAD	82	FEMALE		Stage IIIC	Colon Adenocarcino	2009	-30194	Dead	WITH TUMOR		306			[Unknown]	1	306	1	306			1	306
TCGA-AA	TCGA-AA	COAD	74	MALE		Stage I	Colon Adenocarcino	2009	-27090	Dead	WITH TUMOR		0				1	0	1	0			1	0
TCGA-AA	TCGA-AA	COAD	74	MALE		Stage IIA	Colon Adenocarcino	2009	-27090	Alive	WITH TUM	1006				[Unknown]	0	1006	0	1006			0	1006
TCGA-AA	TCGA-AA	COAD	88	MALE		Stage IIA	Colon Mucinous Ade	2009	-32203	Dead	WITH TUMOR		0				1	0	1	0			1	0
TCGA-AA	TCGA-AA	COAD	71	FEMALE		Stage I	Colon Mucinous Ade	2009	-26022	Alive	TUMOR F	1096				Complete Remission/Response	0	1096	0	1096	0	1096	0	1096
TCGA-AA	TCGA-AA	COAD	72	MALE		Stage I	Colon Adenocarcino	2009	-26541	Alive	WITH TUM	975				[Unknown]	0	975	0	975			0	975
TCGA-AA	TCGA-AA	COAD	59	MALE		Stage IIA	Colon Adenocarcino	2009	-21670	Alive	WITH TUM	30					0	30	0	30			0	30
TCGA-AA	TCGA-AA	COAD	67	MALE		Stage I	Colon Adenocarcino	2009	-24806	Alive	TUMOR F	945				Complete Remission/Response	0	945	0	945	0	945	0	945
TCGA-AA	TCGA-AA	COAD	53	FEMALE		Stage IIIB	Colon Adenocarcino	2009	-19449	Alive	TUMOR F	945				Complete Remission/Response	0	945	0	945	0	945	0	945
TCGA-AA	TCGA-AA	COAD	72	MALE		Stage IIA	Colon Adenocarcino	2009	-26602	Alive	TUMOR F	914				Complete Remission/Response	0	914	0	914	0	914	0	914
TCGA-AA	TCGA-AA	COAD	82	MALE		Stage IIA	Colon Adenocarcino	2009	-30163	Alive	TUMOR F	914				Complete Remission/Response	0	914	0	914	0	914	0	914
TCGA-AA	TCGA-AA	COAD	71	MALE		Stage II	Colon Adenocarcino	2005	-26237	Alive	TUMOR F	1612				Complete Remission/Response	0	1612	0	1612	0	1612	0	1612
TCGA-AA	TCGA-AA	COAD	78	FEMALE		Stage I	Colon Adenocarcino	2006	-28672	Alive	TUMOR F	518				Complete Remission/Response	0	518	0	518	0	518	0	518
TCGA-AA	TCGA-AA	COAD	74	MALE		Stage IV	Colon Adenocarcino	2007	-27028	Alive	WITH TUM	731			394	[Unknown]	0	731	0	731			1	394
TCGA-AA	TCGA-AA	COAD	76	MALE		Stage IV	Colon Adenocarcino	2009	-28062	Dead	WITH TUMOR		822			Partial Remission/Response	1	822	1	822			1	822
TCGA-AA	TCGA-AA	COAD	71	FEMALE		Stage IV	Colon Adenocarcino	2009	-26086	Alive	TUMOR F	912				Complete Remission/Response	0	912	0	912			0	912
TCGA-AA	TCGA-AA	COAD	45	MALE		Stage IV	Colon Adenocarcino	2009	-16651	Alive	WITH TUM	0					0	0	0	0			0	0
TCGA-AA	TCGA-AA	COAD	78	FEMALE		Stage I	Colon Adenocarcino	2007	-28640	Alive	WITH TUM	549				[Unknown]	0	549	0	549			0	549
TCGA-AA	TCGA-AA	COAD	83	FEMALE		Stage I	Colon Mucinous Ade	2007	-30377	Alive	TUMOR F	943				Complete Remission/Response	0	943	0	943	0	943	0	943
TCGA-AA	TCGA-AA	COAD	66	MALE		Stage IV	Colon Adenocarcino	2008	-24321	Dead	WITH TUMOR		61			Progressive Disease	1	61	1	61			1	61
TCGA-AA	TCGA-AA	COAD	83	MALE		Stage IIA	Colon Adenocarcino	2009	-30439	Alive	WITH TUM	395				[Unknown]	0	395	0	395			0	395
TCGA-AA	TCGA-AA	COAD	84	FEMALE		Stage IVA	Colon Adenocarcino	2010	-31015	Alive	WITH TUM	730				[Unknown]	0	730	0	730			0	730
TCGA-AA	TCGA-AA	COAD	60	FEMALE		Stage IIB	Colon Mucinous Ade	2009	-22035	Alive	TUMOR F	1004				Complete Remission/Response	0	1004	0	1004	0	1004	0	1004
TCGA-AA	TCGA-AA	COAD	87	FEMALE		Stage IIIB	Colon Mucinous Ade	2009	-32081	Alive	TUMOR F	791				Complete Remission/Response	0	791	0	791	0	791	0	791
TCGA-AA	TCGA-AA	COAD	79	FEMALE		Stage IIA	Colon Mucinous Ade	2010	-29130	Alive	TUMOR F	730				Complete Remission/Response	0	730	0	730	0	730	0	730
TCGA-AA	TCGA-AA	COAD	68	MALE		Stage IIIC	Colon Adenocarcino	2008	-24868	Dead	WITH TUMOR		61				1	61	1	61			1	61
TCGA-AA	TCGA-AA	COAD	38	MALE		Stage IIIB	Colon Adenocarcino	2010	-14186	Alive	TUMOR F	638				Complete Remission/Response	0	638	0	638	0	638	0	638
TCGA-AA	TCGA-AA	COAD	65	MALE		Stage IIA	Colon Adenocarcino	2007	-24045	Alive	WITH TUM	1035				[Unknown]	0	1035	0	1035			0	1035
TCGA-AA	TCGA-AA	COAD	89	FEMALE		Stage IIA	Colon Mucinous Ade	2009	-32537	Alive	WITH TUM	61					0	61	0	61			0	61
TCGA-AA	TCGA-AA	COAD	55	FEMALE		Stage I	Colon Adenocarcino	2008	-20089	Alive	TUMOR F	669				Complete Remission/Response	0	669	0	669	0	669	0	669
TCGA-AA	TCGA-AA	COAD	65	MALE		Stage IIA	Colon Adenocarcino	2009	-23741	Alive	TUMOR F	1096				Complete Remission/Response	0	1096	0	1096	0	1096	0	1096
TCGA-AA	TCGA-AA	COAD	58	MALE		Stage III	Colon Adenocarcino	2007	-21518	Alive	TUMOR F	489				Complete Remission/Response	0	489	0	489	0	489	0	489
TCGA-AA	TCGA-AA	COAD	72	MALE		Stage IV	Colon Adenocarcino	2008	-26360	Alive	WITH TUM	1551			1216	Progressive Disease	0	1551	0	1551			1	1216
TCGA-AA	TCGA-AA	COAD	69	MALE		Stage IV	Colon Adenocarcino	2007	-25536	Alive	WITH TUM	397				Stable Disease	0	397	0	397			0	397
TCGA-AA	TCGA-AA	COAD	80	MALE		Stage I	Colon Adenocarcino	2009	-29310	Alive	TUMOR F	1036				Complete Remission/Response	0	1036	0	1036	0	1036	0	1036
TCGA-AA	TCGA-AA	COAD	70	MALE		Stage IIIA	Colon Adenocarcino	2009	-25599	Alive	WITH TUM	791				Complete Remission/Response	0	791	0	791	0	791	0	791
TCGA-AA	TCGA-AA	COAD	65	MALE			Colon Adenocarcino	2009	-23832	Alive	TUMOR F	761				Complete Remission/Response	0	761	0	761	0	761	0	761
TCGA-AA	TCGA-AA	COAD	84	MALE		Stage IIA	Colon Adenocarcino	2010	-30742	Alive	TUMOR F	730				Complete Remission/Response	0	730	0	730	0	730	0	730
TCGA-AA	TCGA-AA	COAD	89	FEMALE		Stage I	Colon Adenocarcino	2009	-32630	Alive	TUMOR F	822				Complete Remission/Response	0	822	0	822	0	822	0	822
TCGA-AA	TCGA-AA	COAD	75	MALE		Stage IIIB	Colon Adenocarcino	2009	-27608	Alive	WITH TUM	822				[Unknown]	0	822	0	822			0	822
TCGA-AA	TCGA-AA	COAD	61	FEMALE		Stage IIA	Colon Adenocarcino	2009	-22585	Alive	WITH TUM	0					0	0	0	0			0	0
TCGA-AA	TCGA-AA	COAD	73	MALE		Stage I	Colon Adenocarcino	2007	-26967	Alive	TUMOR F	580				Complete Remission/Response	0	580	0	580	0	580	0	580
TCGA-AA	TCGA-AA	COAD	84	MALE		Stage IV	Colon Adenocarcino	2009	-30712	Dead	WITH TUMOR		242			[Unknown]	1	242	1	242			1	242
TCGA-AA	TCGA-AA	COAD	69	MALE		Stage IIIB	Colon Mucinous Ade	2008	-25323	Alive	TUMOR F	822				Complete Remission/Response	0	822	0	822	0	822	0	822
TCGA-AA	TCGA-AA	COAD	76	MALE		Stage IIA	Colon Adenocarcino	2009	-27943	Alive	TUMOR F	424				Complete Remission/Response	0	424	0	424	0	424	0	424
TCGA-AA	TCGA-AA	COAD	80	MALE		Stage IIA	Colon Adenocarcino	2009	-29463	Alive	WITH TUM	1157				[Unknown]	0	1157	0	1157			0	1157
TCGA-AA	TCGA-AA	COAD	70	MALE		Stage I	Colon Adenocarcino	2007	-25689	Alive	TUMOR F	578				Complete Remission/Response	0	578	0	578	0	578	0	578
TCGA-AA	TCGA-AA	COAD	65	MALE		Stage IIA	Colon Adenocarcino	2007	-23741	Alive	TUMOR F	913				Complete Remission/Response	0	913	0	913	0	913	0	913
TCGA-AA	TCGA-AA	COAD	66	MALE		Stage IIIC	Colon Adenocarcino	2007	-24379	Alive	TUMOR F	1035				Partial Remission/Response	0	1035	0	1035			0	1035
TCGA-AA	TCGA-AA	COAD	80	MALE		Stage IIIB	Colon Mucinous Ade	2009	-29371	Alive	TUMOR F	549				Partial Remission/Response	0	549	0	549			0	549
TCGA-AA	TCGA-AA	COAD	79	MALE		Stage IIA	Colon Adenocarcino	2009	-29189	Alive	WITH TUM	549				[Unknown]	0	549	0	549			0	549
TCGA-AA	TCGA-AA	COAD	66	MALE		Stage IIA	Colon Adenocarcino	2009	-24319	Alive	TUMOR F	1157				Complete Remission/Response	0	1157	0	1157	0	1157	0	1157
TCGA-AA	TCGA-AA	COAD	75	MALE		Stage IIB	Colon Mucinous Ade	2008	-27667	Dead	WITH TUMOR		122				1	122	1	122			1	122
TCGA-AA	TCGA-AA	COAD	83	FEMALE		Stage IIIC	Colon Adenocarcino	2008	-30316	Alive	[Discrepan	822				[Discrepancy]	0	822	0	822			0	822
TCGA-AA	TCGA-AA	COAD	66	FEMALE		Stage IIIB	Colon Adenocarcino	2008	-24199	Alive	TUMOR F	1278				Complete Remission/Response	0	1278	0	1278	0	1278	0	1278
TCGA-AA	TCGA-AA	COAD	64	FEMALE		Stage I	Colon Adenocarcino	2008	-23528	Alive	WITH TUM	30					0	30	0	30			0	30
TCGA-AA	TCGA-AA	COAD	50	MALE		Stage IIIB	Colon Adenocarcino	2009	-18263	Alive	TUMOR F	518				Complete Remission/Response	0	518	0	518	0	518	0	518
TCGA-AA	TCGA-AA	COAD	80	MALE		Stage I	Colon Adenocarcino	2008	-29311	Alive	TUMOR F	456				Partial Remission/Response	0	456	0	456			0	456
TCGA-AA	TCGA-AA	COAD	70	MALE		Stage IIA	Colon Adenocarcino	2008	-25872	Alive	TUMOR F	669				Complete Remission/Response	0	669	0	669	0	669	0	669
TCGA-AA	TCGA-AA	COAD	46	FEMALE		Stage IIB	Colon Adenocarcino	2008	-16922	Alive	TUMOR F	1064				Complete Remission/Response	0	1064	0	1064	0	1064	0	1064
TCGA-AA	TCGA-AA	COAD	57	FEMALE		Stage IIA	Colon Adenocarcino	2009	-21032	Alive	TUMOR F	457				Complete Remission/Response	0	457	0	457	0	457	0	457
TCGA-AA	TCGA-AA	COAD	75	MALE		Stage IIIA	Colon Adenocarcino	2009	-27729	Alive	TUMOR F	457				[Discrepancy]	0	457	0	457			0	457
TCGA-AA	TCGA-AA	COAD	47	FEMALE		Stage IIIC	Colon Mucinous Ade	2009	-17318	Dead	WITH TUMOR		334		183	Progressive Disease	1	334	1	334			1	183
TCGA-AA	TCGA-AA	COAD	72	MALE		Stage IIIB	Colon Adenocarcino	2009	-26298	Alive	WITH TUM	974				[Unknown]	0	974	0	974			0	974
TCGA-AA	TCGA-AA	COAD	63	MALE		Stage IIA	Colon Mucinous Ade	2009	-23133	Alive	TUMOR F	365				Complete Remission/Response	0	365	0	365	0	365	0	365
TCGA-AA	TCGA-AA	COAD	76	MALE		Stage I	Colon Adenocarcino	2009	-27759	Alive	TUMOR F	943				Complete Remission/Response	0	943	0	943	0	943	0	943
TCGA-AA	TCGA-AA	COAD	74	FEMALE		Stage IIIC	Colon Adenocarcino	2009	-27029	Alive	TUMOR F	943				Complete Remission/Response	0	943	0	943	0	943	0	943
TCGA-AA	TCGA-AA	COAD	80	FEMALE		Stage III	Colon Adenocarcino	2003	-29554	Dead	WITH TUMOR		1158		762	[Unknown]	1	1158	1	1158			1	762
TCGA-AA	TCGA-AA	COAD	80	FEMALE		Stage III	Colon Adenocarcino	2003	-29554	Dead	WITH TUMOR		1158		762	[Unknown]	1	1158	1	1158			1	762
TCGA-AA	TCGA-AA	COAD	48	FEMALE		Stage II	Colon Adenocarcino	2003	-17715	Alive	WITH TUM	31					0	31	0	31			0	31
TCGA-AA	TCGA-AA	COAD	47	MALE		Stage III	Colon Mucinous Ade	2004	-17289	Alive	WITH TUM	1065			608	Partial Remission/Response	0	1065	0	1065			1	608
TCGA-AA	TCGA-AA	COAD	47	MALE		Stage III	Colon Mucinous Ade	2004	-17289	Alive	WITH TUM	1065			608	Partial Remission/Response	0	1065	0	1065			1	608
TCGA-AA	TCGA-AA	COAD	47	FEMALE		Stage III	Colon Adenocarcino	2004	-17257	Alive	WITH TUM	31					0	31	0	31			0	31
TCGA-AA	TCGA-AA	COAD	47	FEMALE		Stage III	Colon Adenocarcino	2004	-17257	Alive	WITH TUM	31					0	31	0	31			0	31
TCGA-AA	TCGA-AA	COAD	63	FEMALE		Stage III	Colon Adenocarcino	2004	-23192	Alive	TUMOR F	1005				Complete Remission/Response	0	1005	0	1005	0	1005	0	1005
TCGA-AA	TCGA-AA	COAD	63	FEMALE		Stage III	Colon Adenocarcino	2004	-23192	Alive	TUMOR F	1005				Complete Remission/Response	0	1005	0	1005	0	1005	0	1005
TCGA-AA	TCGA-AA	COAD	59	MALE		Stage I	Colon Adenocarcino	2004	-21792	Alive	WITH TUM	31					0	31	0	31			0	31
TCGA-AA	TCGA-AA	COAD	59	MALE		Stage I	Colon Adenocarcino	2004	-21792	Alive	WITH TUM	31					0	31	0	31			0	31
TCGA-AA	TCGA-AA	COAD	80	FEMALE		Stage III	Colon Adenocarcino	2004	-29281	Alive	WITH TUM	791			791	[Unknown]	0	791	0	791			1	791
TCGA-AA	TCGA-AA	COAD	80	FEMALE		Stage III	Colon Adenocarcino	2004	-29281	Alive	WITH TUM	791			791	[Unknown]	0	791	0	791			1	791
TCGA-AA	TCGA-AA	COAD	68	MALE		Stage II	Colon Adenocarcino	2004	-24990	Alive	WITH TUM	1126			1126	Progressive Disease	0	1126	0	1126			1	1126
TCGA-AA	TCGA-AA	COAD	68	MALE		Stage II	Colon Adenocarcino	2004	-24990	Alive	WITH TUM	1126			1126	Progressive Disease	0	1126	0	1126			1	1126
TCGA-AA	TCGA-AA	COAD	88	FEMALE		Stage II	Colon Adenocarcino	2005	-32446	Alive	WITH TUM	0					0	0	0	0			0	0
TCGA-AA	TCGA-AA	COAD	88	FEMALE		Stage II	Colon Adenocarcino	2005	-32446	Alive	WITH TUM	0					0	0	0	0			0	0
TCGA-AA	TCGA-AA	COAD	81	MALE		Stage II	Colon Mucinous Ade	2005	-29708	Dead	TUMOR FREE		1188			Complete Remission/Response	1	1188	0	1188	0	1188	0	1188
TCGA-AA	TCGA-AA	COAD	67	MALE		Stage II	Colon Adenocarcino	2006	-24686	Alive	TUMOR F	1581				Complete Remission/Response	0	1581	0	1581	0	1581	0	1581
TCGA-AA	TCGA-AA	COAD	82	FEMALE		Stage IV	Colon Adenocarcino	2007	-30256	Dead	WITH TUMOR		90				1	90	1	90			1	90
TCGA-AA	TCGA-AA	COAD	68	FEMALE		Stage IV	Colon Adenocarcino	2007	-25051	Alive	WITH TUM	1216				Partial Remission/Response	0	1216	0	1216			0	1216
TCGA-AA	TCGA-AA	COAD	74	FEMALE		Stage IV	Colon Adenocarcino	2007	-27362	Dead	WITH TUMOR		61				1	61	1	61			1	61
TCGA-AA	TCGA-AA	COAD	70	FEMALE		Stage IV	Colon Adenocarcino	2008	-25749	Dead	WITH TUMOR		153				1	153	1	153			1	153
TCGA-AA	TCGA-AA	COAD	50	MALE		Stage IV	Colon Adenocarcino	2009	-18506	Dead	WITH TUMOR		426		306	Progressive Disease	1	426	1	426			1	306
TCGA-AA	TCGA-AA	COAD	82	MALE		Stage II	Colon Adenocarcino	2005	-30288	Alive	WITH TUM	28					0	28	0	28			0	28
TCGA-AA	TCGA-AA	COAD	82	MALE		Stage II	Colon Adenocarcino	2005	-30288	Alive	WITH TUM	28					0	28	0	28			0	28
TCGA-AA	TCGA-AA	COAD	84	FEMALE		Stage IIA	Colon Adenocarcino	2006	-30834	Dead	WITH TUMOR		670				1	670	1	670			1	670
TCGA-AA	TCGA-AA	COAD	73	FEMALE		Stage I	Colon Adenocarcino	2006	-26693	Alive	TUMOR F	1247			1005	Complete Remission/Response	0	1247	0	1247	1	1005	1	1005
TCGA-AA	TCGA-AA	COAD	73	MALE		Stage I	Colon Adenocarcino	2007	-26724	Alive	TUMOR F	1216				Complete Remission/Response	0	1216	0	1216	0	1216	0	1216
TCGA-AA	TCGA-AA	COAD	90	FEMALE		Stage III	Colon Mucinous Ade	2004	-32873	Dead	WITH TUMOR		549			[Unknown]	1	549	1	549			1	549
TCGA-AA	TCGA-AA	COAD	90	FEMALE		Stage III	Colon Mucinous Ade	2004	-32873	Dead	WITH TUMOR		549			[Unknown]	1	549	1	549			1	549
TCGA-AA	TCGA-AA	COAD	65	FEMALE		Stage I	Colon Adenocarcino	2004	-23986	Alive	TUMOR F	1246				Complete Remission/Response	0	1246	0	1246	0	1246	0	1246
TCGA-AA	TCGA-AA	COAD	65	FEMALE		Stage I	Colon Adenocarcino	2004	-23986	Alive	TUMOR F	1246				Complete Remission/Response	0	1246	0	1246	0	1246	0	1246
TCGA-AD	TCGA-AD	COAD	67	MALE	WHITE	Stage I	Colon Mucinous Ade	2010	-24776	Alive	TUMOR F	370				Complete Remission/Response	0	370	0	370	0	370	0	370
TCGA-AD	TCGA-AD	COAD	81	FEMALE	WHITE	Stage I	Colon Adenocarcino	2011	-29758	Alive	TUMOR F	650				Complete Remission/Response	0	650	0	650	0	650	0	650
TCGA-AD	TCGA-AD	COAD	73	MALE	WHITE	Stage IIIB	Colon Adenocarcino	2010	-26816	Dead	WITH TUMOR		472		343	Complete Remission/Response	1	472	1	472	1	343	1	343
TCGA-AD	TCGA-AD	COAD	76	MALE	ASIAN	Stage IIA	Colon Adenocarcino	2005		Dead	TUMOR FREE		2532		1516	Complete Remission/Response	1	2532	0	2532	1	1516	1	1516
TCGA-AD	TCGA-AD	COAD	65	MALE	WHITE		Colon Adenocarcino	2010	-23769	Alive	TUMOR F	746				Complete Remission/Response	0	746	0	746	0	746	0	746
TCGA-AD	TCGA-AD	COAD	84	MALE	WHITE	Stage IIIB	Colon Adenocarcino	2011	-30879	Alive	TUMOR F	763				Complete Remission/Response	0	763	0	763	0	763	0	763
TCGA-AD	TCGA-AD	COAD	84	MALE	WHITE	Stage IIIC	Colon Mucinous Ade	2011	-30693	Dead	TUMOR FREE		176			Complete Remission/Response	1	176	0	176	0	176	0	176
TCGA-AD	TCGA-AD	COAD	78	MALE	WHITE		Colon Adenocarcino	2011	-28579	Dead	WITH TUMOR		682	Metastatic	468	Complete Remission/Response	1	682	1	682	1	468	1	468
TCGA-AD	TCGA-AD	COAD	58	MALE	WHITE		Colon Adenocarcino	2011	-21294	Alive	TUMOR F	834				Complete Remission/Response	0	834	0	834	0	834	0	834
TCGA-AD	TCGA-AD	COAD	58	MALE	WHITE		Colon Adenocarcino	2011	-21457	Dead	WITH TUMOR		331	Metastatic	325	Progressive Disease	1	331	1	331			1	325
TCGA-AD	TCGA-AD	COAD	62	MALE	WHITE	Stage IIIC	Colon Adenocarcino	2011	-22853	Alive	WITH TUM	805			648	Complete Remission/Response	0	805	0	805	1	648	1	648
TCGA-AD	TCGA-AD	COAD	74	FEMALE	BLACK OR	Stage IIA	Colon Adenocarcino	2012	-27180	Alive	TUMOR F	0				[Unknown]	0	0	0	0			0	0
TCGA-AD	TCGA-AD	COAD	51	MALE	BLACK OR	Stage I	Colon Adenocarcino	2012	-18731	Alive	TUMOR F	500				Complete Remission/Response	0	500	0	500	0	500	0	500
TCGA-AM	TCGA-AM	COAD	59	FEMALE	WHITE	Stage IVA	Colon Adenocarcino	2010	-21902	Alive	TUMOR F	14					0	14	0	14			0	14
TCGA-AM	TCGA-AM	COAD	68	FEMALE	WHITE	Stage IIA	Colon Adenocarcino	2010	-24903	Alive	TUMOR F	28					0	28	0	28			0	28
TCGA-AU	TCGA-AU	COAD	80	FEMALE	WHITE	Stage IIA	Colon Adenocarcino	2010	-29460	Alive	TUMOR F	441				[Unknown]	0	441	0	441			0	441
TCGA-AU	TCGA-AU	COAD	69	FEMALE	WHITE	Stage I	Colon Adenocarcino	2010	-25259	Alive	TUMOR F	824					0	824	0	824			0	824
TCGA-AY	TCGA-AY	COAD	50	FEMALE	BLACK OR	Stage IIIC	Colon Adenocarcino	2007	-18621	Dead	WITH TUMOR		496		186		1	496	1	496			1	186
TCGA-AY	TCGA-AY	COAD	63	FEMALE	WHITE	Stage I		2009	-23320	Dead	TUMOR FREE		29				1	29	0	29			0	29
TCGA-AY	TCGA-AY	COAD	65	FEMALE	BLACK OR	Stage IVA	Colon Adenocarcino	2010	-23870	Alive	TUMOR F	1004				Complete Remission/Response	0	1004	0	1004			0	1004
TCGA-AY	TCGA-AY	COAD	47	MALE	WHITE	Stage IIIC	Colon Mucinous Ade	2010	-17311	Alive	TUMOR F	6					0	6	0	6			0	6
TCGA-AY	TCGA-AY	COAD	60	MALE	WHITE	Stage IIA	Colon Adenocarcino	2010	-21957	Alive	TUMOR F	652				Complete Remission/Response	0	652	0	652	0	652	0	652
TCGA-AY	TCGA-AY	COAD	66	FEMALE	BLACK OR	Stage IIIB	Colon Adenocarcino	2010	-24453	Alive	TUMOR F	542				Complete Remission/Response	0	542	0	542	0	542	0	542
TCGA-AY	TCGA-AY	COAD	74	FEMALE	BLACK OR	Stage I	Colon Adenocarcino	2012	-27074	Alive	WITH TUM	525			396	Complete Remission/Response	0	525	0	525	1	396	1	396
TCGA-AY	TCGA-AY	COAD	55	FEMALE	BLACK OR	Stage IIA	Colon Adenocarcino	2012	-20299	Alive	TUMOR F	543				Complete Remission/Response	0	543	0	543	0	543	0	543
TCGA-AY	TCGA-AY	COAD	54	FEMALE	BLACK OR	Stage I	Colon Adenocarcino	2013	-19904	Alive	TUMOR F	588				Complete Remission/Response	0	588	0	588	0	588	0	588
TCGA-AY	TCGA-AY	COAD	44	MALE	BLACK OR	Stage IVA	Colon Adenocarcino	2013	-16140	Alive	TUMOR F	573				Complete Remission/Response	0	573	0	573			0	573
TCGA-AZ	TCGA-AZ	COAD	47	FEMALE	WHITE	Stage IIIB	Colon Adenocarcino	2006	-17321	Alive	TUMOR F	3324				Complete Remission/Response	0	3324	0	3324	0	3324	0	3324
TCGA-AZ	TCGA-AZ	COAD	51	FEMALE	WHITE	Stage I	Colon Adenocarcino	2007	-18765	Alive	TUMOR F	2310				Complete Remission/Response	0	2310	0	2310	0	2310	0	2310
TCGA-AZ	TCGA-AZ	COAD	61	MALE	WHITE	Stage IIA	Colon Adenocarcino	2006	-22340	Alive	TUMOR F	1776					0	1776	0	1776			0	1776
TCGA-AZ	TCGA-AZ	COAD	37	MALE	WHITE	Stage IV	Colon Adenocarcino	2002	-13755	Dead	WITH TUMOR		43				1	43	1	43			1	43
TCGA-AZ	TCGA-AZ	COAD	71	FEMALE	WHITE	Stage IVA	Colon Adenocarcino	2008	-26267	Dead	WITH TUMOR		172				1	172	1	172			1	172
TCGA-AZ	TCGA-AZ	COAD	84	MALE	WHITE	Stage IIIB	Colon Adenocarcino	2009	-30908	Alive	WITH TUM	1002			688		0	1002	0	1002			1	688
TCGA-AZ	TCGA-AZ	COAD	82	FEMALE	WHITE	Stage IV	Colon Adenocarcino	2009	-30043	Dead	WITH TUMOR		156				1	156	1	156			1	156
TCGA-AZ	TCGA-AZ	COAD	79	FEMALE	WHITE	Stage IIA	Colon Adenocarcino	2004	-28906	Alive	TUMOR F	3247				Complete Remission/Response	0	3247	0	3247	0	3247	0	3247
TCGA-AZ	TCGA-AZ	COAD	61	MALE	WHITE	Stage IVA	Colon Adenocarcino	2006	-22409	Dead	WITH TUMOR		680				1	680	1	680			1	680
TCGA-AZ	TCGA-AZ	COAD	49	MALE	WHITE	Stage IVA	Colon Adenocarcino	2006	-18252	Alive	WITH TUM	1977			411		0	1977	0	1977			1	411
TCGA-AZ	TCGA-AZ	COAD	43	MALE	WHITE	Stage II	Colon Adenocarcino	2000	-15983	Dead	WITH TUMOR		1910		304		1	1910	1	1910			1	304
TCGA-AZ	TCGA-AZ	COAD	51	FEMALE	WHITE	Stage I	Colon Adenocarcino	2007	-18820	Alive	TUMOR F	2683				Complete Remission/Response	0	2683	0	2683	0	2683	0	2683
TCGA-AZ	TCGA-AZ	COAD	77	FEMALE	WHITE	Stage II	Colon Adenocarcino	2000	-28182	Dead	TUMOR FREE		1503			Complete Remission/Response	1	1503	0	1503	0	1503	0	1503
TCGA-AZ	TCGA-AZ	COAD	77	FEMALE	WHITE	Stage II	Colon Adenocarcino	2000	-28182	Dead	TUMOR FREE		1503			Complete Remission/Response	1	1503	0	1503	0	1503	0	1503
TCGA-AZ	TCGA-AZ	COAD	72	MALE	WHITE	Stage I	Colon Adenocarcino	2001	-26438	Dead	TUMOR FREE		206			Complete Remission/Response	1	206	0	206	0	206	0	206
TCGA-AZ	TCGA-AZ	COAD	72	MALE	WHITE	Stage I	Colon Adenocarcino	2001	-26438	Dead	TUMOR FREE		206			Complete Remission/Response	1	206	0	206	0	206	0	206
TCGA-AZ	TCGA-AZ	COAD	64	MALE	BLACK OR	Stage IV	Colon Adenocarcino	2001	-23581	Dead	WITH TUMOR		368			Progressive Disease	1	368	1	368			1	368
TCGA-AZ	TCGA-AZ	COAD	64	MALE	BLACK OR	Stage IV	Colon Adenocarcino	2001	-23581	Dead	WITH TUMOR		368			Progressive Disease	1	368	1	368			1	368
TCGA-AZ	TCGA-AZ	COAD	68	MALE	WHITE	Stage II	Colon Adenocarcino	2001	-25082	Dead	WITH TUMOR		3042	Locoregional Disease	2270	Progressive Disease	1	3042	1	3042			1	2270
TCGA-AZ	TCGA-AZ	COAD	68	MALE	WHITE	Stage II	Colon Adenocarcino	2001	-25082	Dead	WITH TUMOR		3042	Locoregional Disease	2270	Progressive Disease	1	3042	1	3042			1	2270
TCGA-AZ	TCGA-AZ	COAD	77	FEMALE	WHITE		Colon Adenocarcino	2002	-28424	Dead	TUMOR FREE		899			Complete Remission/Response	1	899	0	899	0	899	0	899
TCGA-AZ	TCGA-AZ	COAD	77	FEMALE	WHITE		Colon Adenocarcino	2002	-28424	Dead	TUMOR FREE		899			Complete Remission/Response	1	899	0	899	0	899	0	899
TCGA-AZ	TCGA-AZ	COAD	77	MALE	WHITE	Stage IIIB	Colon Adenocarcino	2008	-28485	Dead	TUMOR FREE		159			Stable Disease	1	159	0	159			0	159
TCGA-AZ	TCGA-AZ	COAD	77	MALE	WHITE	Stage IIIB	Colon Adenocarcino	2008	-28485	Dead	TUMOR FREE		159			Stable Disease	1	159	0	159			0	159
TCGA-AZ	TCGA-AZ	COAD	81	MALE	WHITE	Stage IV	Colon Adenocarcino	2009	-29704	Dead	WITH TUMOR		357			Progressive Disease	1	357	1	357			1	357
TCGA-AZ	TCGA-AZ	COAD	69	MALE	WHITE	Stage IV	Colon Adenocarcino	2009	-25448	Dead	WITH TUMOR		97			Complete Remission/Response	1	97	1	97			1	97
TCGA-AZ	TCGA-AZ	COAD	55	FEMALE	WHITE	Stage IIIA	Colon Adenocarcino	2009	-20094	Dead	[Discrepancy]		59			Progressive Disease	1	59		59			0	59
TCGA-CA	TCGA-CA	COAD	42	FEMALE	ASIAN	Stage IIA	Colon Adenocarcino	2010	-15415	Alive	TUMOR F	386				Complete Remission/Response	0	386	0	386	0	386	0	386
TCGA-CA	TCGA-CA	COAD	45	MALE	ASIAN	Stage IIA	Colon Adenocarcino	2010	-16743	Alive	TUMOR F	376				Complete Remission/Response	0	376	0	376	0	376	0	376
TCGA-CA	TCGA-CA	COAD	54	FEMALE	ASIAN	Stage IIA	Colon Adenocarcino	2010	-19785	Alive	TUMOR F	379				Complete Remission/Response	0	379	0	379	0	379	0	379
TCGA-CA	TCGA-CA	COAD	52	FEMALE	ASIAN	Stage IIA	Colon Mucinous Ade	2010	-19353	Alive	TUMOR F	377				Complete Remission/Response	0	377	0	377	0	377	0	377
TCGA-CA	TCGA-CA	COAD	56	MALE	ASIAN	Stage IIA	Colon Adenocarcino	2010	-20662	Alive	TUMOR F	383				Complete Remission/Response	0	383	0	383	0	383	0	383
TCGA-CA	TCGA-CA	COAD	63	MALE	ASIAN	Stage IIIB	Colon Adenocarcino	2010	-23075	Alive	TUMOR F	383				Complete Remission/Response	0	383	0	383	0	383	0	383
TCGA-CA	TCGA-CA	COAD	65	MALE	ASIAN	Stage IIA	Colon Adenocarcino	2010	-23943	Alive	TUMOR F	371				Complete Remission/Response	0	371	0	371	0	371	0	371
TCGA-CA	TCGA-CA	COAD	57	MALE	ASIAN	Stage IIA	Colon Mucinous Ade	2010	-20984	Alive	TUMOR F	388				Complete Remission/Response	0	388	0	388	0	388	0	388
TCGA-CA	TCGA-CA	COAD	46	MALE	ASIAN	Stage IIA	Colon Adenocarcino	2010	-17109	Dead	WITH TUMOR		306		207	Complete Remission/Response	1	306	1	306	1	207	1	207
TCGA-CA	TCGA-CA	COAD	77	MALE	ASIAN	Stage IIA	Colon Adenocarcino	2011	-28343	Alive	WITH TUM	435			354	Progressive Disease	0	435	0	435			1	354
TCGA-CK	TCGA-CK	COAD	46	FEMALE	WHITE	Stage IIIB	Colon Adenocarcino	2003	-16980	Alive	WITH TUM	534			21	Stable Disease	0	534	0	534			1	21
TCGA-CK	TCGA-CK	COAD	45	FEMALE	WHITE	Stage III	Colon Adenocarcino	2002	-16736	Alive	TUMOR F	4502				Complete Remission/Response	0	4502	0	4502	0	4502	0	4502
TCGA-CK	TCGA-CK	COAD	68	FEMALE	BLACK OR	Stage IIIB	Colon Mucinous Ade	2008	-24944	Alive	TUMOR F	2599				Complete Remission/Response	0	2599	0	2599	0	2599	0	2599
TCGA-CK	TCGA-CK	COAD	79	FEMALE	WHITE	Stage IIA	Colon Mucinous Ade	2007	-28982	Dead	TUMOR FREE		2134	Locoregional Disease	480		1	2134	0	2134			1	480
TCGA-CK	TCGA-CK	COAD	48	FEMALE	WHITE	Stage IIIC	Colon Mucinous Ade	2007	-17826	Alive	TUMOR F	475				[Unknown]	0	475	0	475			0	475
TCGA-CK	TCGA-CK	COAD	81	MALE	WHITE	Stage I	Colon Adenocarcino	2006	-29938	Dead	TUMOR FREE		1493			Complete Remission/Response	1	1493	0	1493	0	1493	0	1493
TCGA-CK	TCGA-CK	COAD	58	FEMALE	WHITE	Stage IIA	Colon Adenocarcino	2008	-21399	Alive	TUMOR F	1561				Complete Remission/Response	0	1561	0	1561	0	1561	0	1561
TCGA-CK	TCGA-CK	COAD	81	MALE	WHITE	Stage IIIB	Colon Adenocarcino	2008	-29586	Alive	TUMOR F	669					0	669	0	669			0	669
TCGA-CK	TCGA-CK	COAD	63	MALE	WHITE	Stage I	Colon Adenocarcino	2007	-23040	Alive	TUMOR F	0					0	0	0	0			0	0
TCGA-CK	TCGA-CK	COAD	71	FEMALE	WHITE	Stage I	Colon Adenocarcino	2003	-26024	Dead	WITH TUMOR		643		292	Progressive Disease	1	643	1	643			1	292
TCGA-CK	TCGA-CK	COAD	84	FEMALE	WHITE	Stage IIB	Colon Adenocarcino	2009	-30957	Alive	TUMOR F	0					0	0	0	0			0	0
TCGA-CK	TCGA-CK	COAD	87	FEMALE	WHITE	Stage IIA	Colon Adenocarcino	2008	-32052	Alive	TUMOR F	2523				Complete Remission/Response	0	2523	0	2523	0	2523	0	2523
TCGA-CK	TCGA-CK	COAD	45	FEMALE	WHITE	Stage IV	Colon Mucinous Ade	2008	-16529	Alive	WITH TUM	61				[Unknown]	0	61	0	61			0	61
TCGA-CK	TCGA-CK	COAD	88	FEMALE	WHITE	Stage I	Colon Mucinous Ade	2002	-32354	Alive	TUMOR F	3780					0	3780	0	3780			0	3780
TCGA-CM	TCGA-CM	COAD	69	MALE	WHITE	Stage IIA	Colon Adenocarcino	2010	-25291	Alive	TUMOR F	701				Complete Remission/Response	0	701	0	701	0	701	0	701
TCGA-CM	TCGA-CM	COAD	69	MALE	WHITE	Stage I	Colon Adenocarcino	2010	-25506	Alive	TUMOR F	609				Complete Remission/Response	0	609	0	609	0	609	0	609
TCGA-CM	TCGA-CM	COAD	61	MALE	BLACK OR	Stage I	Colon Adenocarcino	2009	-22403	Alive	TUMOR F	1126				Complete Remission/Response	0	1126	0	1126	0	1126	0	1126
TCGA-CM	TCGA-CM	COAD	47	MALE	WHITE	Stage IVA	Colon Adenocarcino	2010	-17198	Alive	[Discrepan	761				[Discrepancy]	0	761	0	761			0	761
TCGA-CM	TCGA-CM	COAD	53	MALE	WHITE	Stage IIIB	Colon Mucinous Ade	2010	-19509	Alive	TUMOR F	792				Complete Remission/Response	0	792	0	792	0	792	0	792
TCGA-CM	TCGA-CM	COAD	34	FEMALE	WHITE	Stage IIIA	Colon Adenocarcino	2010	-12478	Alive	TUMOR F	244				Complete Remission/Response	0	244	0	244	0	244	0	244
TCGA-CM	TCGA-CM	COAD	62	MALE	WHITE	Stage IIIB	Colon Adenocarcino	2009	-22891	Alive	TUMOR F	822				Complete Remission/Response	0	822	0	822	0	822	0	822
TCGA-CM	TCGA-CM	COAD	58	MALE	BLACK OR	Stage IIA	Colon Adenocarcino	2010	-21519	Alive	TUMOR F	396				Complete Remission/Response	0	396	0	396	0	396	0	396
TCGA-CM	TCGA-CM	COAD	82	FEMALE	WHITE	Stage IIIA	Colon Adenocarcino	2010	-30163	Alive	TUMOR F	884				Complete Remission/Response	0	884	0	884	0	884	0	884
TCGA-CM	TCGA-CM	COAD	39	FEMALE	WHITE	Stage IIIB	Colon Adenocarcino	2010	-14426	Alive	TUMOR F	670				[Unknown]	0	670	0	670			0	670
TCGA-CM	TCGA-CM	COAD	72	MALE	WHITE	Stage IIIB	Colon Adenocarcino	2010	-26543	Alive	TUMOR F	699				[Unknown]	0	699	0	699			0	699
TCGA-CM	TCGA-CM	COAD	68	FEMALE	WHITE	Stage IIA	Colon Adenocarcino	2010	-24867	Alive	TUMOR F	915				[Unknown]	0	915	0	915			0	915
TCGA-CM	TCGA-CM	COAD	44	MALE	WHITE	Stage IIA	Colon Adenocarcino	2010	-16344	Alive	TUMOR F	974					0	974	0	974			0	974
TCGA-CM	TCGA-CM	COAD	63	FEMALE	WHITE	Stage IIA	Colon Adenocarcino	2010	-23253	Alive	TUMOR F	457		Locoregional Disease	335		0	457	0	457			1	335
TCGA-CM	TCGA-CM	COAD	80	MALE	WHITE	Stage IVA	Colon Adenocarcino	2010	-29493	Dead	WITH TUMOR		153		31		1	153	1	153			1	31
TCGA-CM	TCGA-CM	COAD	60	FEMALE	WHITE	Stage IIIB	Colon Mucinous Ade	2010	-22127	Alive	TUMOR F	457					0	457	0	457			0	457
TCGA-CM	TCGA-CM	COAD	60	MALE	WHITE	Stage I	Colon Adenocarcino	2010	-22219	Alive	TUMOR F	457					0	457	0	457			0	457
TCGA-CM	TCGA-CM	COAD	59	FEMALE	WHITE	Stage IVA	Colon Adenocarcino	2010	-21672	Alive	WITH TUM	518					0	518	0	518			0	518
TCGA-CM	TCGA-CM	COAD	36	FEMALE	WHITE	Stage I	Colon Adenocarcino	2010	-13363	Alive	TUMOR F	457					0	457	0	457			0	457
TCGA-CM	TCGA-CM	COAD	48	FEMALE	WHITE	Stage IIIB	Colon Mucinous Ade	2010	-17624	Alive	TUMOR F	365					0	365	0	365			0	365
TCGA-CM	TCGA-CM	COAD	74	MALE	WHITE	Stage I	Colon Adenocarcino	2010	-27150	Alive	TUMOR F	427					0	427	0	427			0	427
TCGA-CM	TCGA-CM	COAD	46	FEMALE	WHITE	Stage IIA	Colon Adenocarcino	2009	-16802	Alive	TUMOR F	883					0	883	0	883			0	883
TCGA-CM	TCGA-CM	COAD	74	MALE	WHITE	Stage IIA	Colon Adenocarcino	2010	-27302	Alive	TUMOR F	488					0	488	0	488			0	488
TCGA-CM	TCGA-CM	COAD	48	FEMALE	WHITE	Stage I	Colon Adenocarcino	2010	-17807	Alive	TUMOR F	669					0	669	0	669			0	669
TCGA-CM	TCGA-CM	COAD	57	FEMALE	BLACK OR	Stage IIIC	Colon Adenocarcino	2010	-21031	Alive	TUMOR F	456					0	456	0	456			0	456
TCGA-CM	TCGA-CM	COAD	84	FEMALE	BLACK OR	Stage IIA	Colon Adenocarcino	2010	-30834	Alive	TUMOR F	395					0	395	0	395			0	395
TCGA-CM	TCGA-CM	COAD	67	MALE	BLACK OR	Stage IIA	Colon Adenocarcino	2010	-24564	Alive	TUMOR F	396					0	396	0	396			0	396
TCGA-CM	TCGA-CM	COAD	73	FEMALE	WHITE	Stage I	Colon Adenocarcino	2010	-26663	Alive	TUMOR F	457					0	457	0	457			0	457
TCGA-CM	TCGA-CM	COAD	77	FEMALE	WHITE	Stage I	Colon Adenocarcino	2010	-28307	Alive	TUMOR F	427					0	427	0	427			0	427
TCGA-CM	TCGA-CM	COAD	70	FEMALE	WHITE	Stage IIIB	Colon Adenocarcino	2010	-25902	Alive	TUMOR F	335					0	335	0	335			0	335
TCGA-CM	TCGA-CM	COAD	39	MALE	WHITE	Stage IIA	Colon Adenocarcino	2011	-14368	Alive	TUMOR F	394					0	394	0	394			0	394
TCGA-CM	TCGA-CM	COAD	35	MALE	BLACK OR	Stage IVB	Colon Adenocarcino	2011	-12935	Alive	WITH TUM	397			337		0	397	0	397			1	337
TCGA-CM	TCGA-CM	COAD	82	MALE	WHITE	Stage I	Colon Adenocarcino	2011	-30223	Alive	TUMOR F	337					0	337	0	337			0	337
TCGA-CM	TCGA-CM	COAD	75	FEMALE	WHITE	Stage IIA	Colon Adenocarcino	2011	-27575	Alive	TUMOR F	337					0	337	0	337			0	337
TCGA-CM	TCGA-CM	COAD	63	FEMALE	WHITE	Stage IVA	Colon Adenocarcino	2011	-23070	Alive	WITH TUM	335		Metastatic	0		0	335	0	335			1	0
TCGA-CM	TCGA-CM	COAD	58	MALE	WHITE	Stage IIA	Colon Adenocarcino	2011	-21519	Alive	TUMOR F	306					0	306	0	306			0	306
TCGA-CM	TCGA-CM	COAD	78	FEMALE	WHITE	Stage IIIB	Colon Adenocarcino	2011	-28701	Alive	TUMOR F	366					0	366	0	366			0	366
TCGA-D5	TCGA-D5	COAD	83	MALE	WHITE	[Discrepancy]	Colon Adenocarcino	2010	-30505	Dead	WITH TUMOR		1381		722	Complete Remission/Response	1	1381	1	1381	1	722	1	722
TCGA-D5	TCGA-D5	COAD	60	FEMALE	WHITE	Stage IIIB	Colon Adenocarcino	2010	-21974	Dead	WITH TUMOR		1661		1007	Progressive Disease	1	1661	1	1661			1	1007
TCGA-D5	TCGA-D5	COAD	60	MALE	WHITE	Stage IIIA	Colon Mucinous Ade	2010	-22097	Alive	WITH TUM	596			579		0	596	0	596			1	579
TCGA-D5	TCGA-D5	COAD	73	MALE	WHITE	Stage IIA	Colon Adenocarcino	2010	-26972	Alive	TUMOR F	1706				Complete Remission/Response	0	1706	0	1706	0	1706	0	1706
TCGA-D5	TCGA-D5	COAD	63	MALE	WHITE	Stage IIIB	Colon Adenocarcino	2010	-23109	Alive	TUMOR F	1701				Complete Remission/Response	0	1701	0	1701	0	1701	0	1701
TCGA-D5	TCGA-D5	COAD	69	MALE	WHITE	Stage IIA	Colon Adenocarcino	2010	-25394	Alive	WITH TUM	614			386		0	614	0	614			1	386
TCGA-D5	TCGA-D5	COAD	53	MALE	WHITE	Stage I	Colon Adenocarcino	2010	-19591	Alive	TUMOR F	621					0	621	0	621			0	621
TCGA-D5	TCGA-D5	COAD	75	MALE	WHITE	Stage IIA	Colon Adenocarcino	2010	-27633	Alive	TUMOR F	540					0	540	0	540			0	540
TCGA-D5	TCGA-D5	COAD	61	MALE	WHITE	Stage IIA	Colon Adenocarcino	2010	-22344	Alive	TUMOR F	555					0	555	0	555			0	555
TCGA-D5	TCGA-D5	COAD	68	FEMALE	WHITE	[Discrepancy]	Colon Adenocarcino	2010	-24971	Alive	TUMOR F	775				Complete Remission/Response	0	775	0	775	0	775	0	775
TCGA-D5	TCGA-D5	COAD	62	FEMALE	WHITE	Stage IIA	Colon Mucinous Ade	2010	-22778	Alive	TUMOR F	1316				Complete Remission/Response	0	1316	0	1316	0	1316	0	1316
TCGA-D5	TCGA-D5	COAD	80	FEMALE	WHITE	Stage IIIB	Colon Adenocarcino	2010	-29278	Alive	TUMOR F	460					0	460	0	460			0	460
TCGA-D5	TCGA-D5	COAD	73	MALE	WHITE	Stage IIA	Colon Adenocarcino	2010	-26890	Alive	WITH TUM	543		Locoregional Disease	493		0	543	0	543			1	493
TCGA-D5	TCGA-D5	COAD	64	MALE	WHITE	Stage IIIB	Colon Adenocarcino	2010	-23381	Dead	WITH TUMOR		146		123		1	146	1	146			1	123
TCGA-D5	TCGA-D5	COAD	79	FEMALE	WHITE	Stage IIIB	Colon Adenocarcino	2010	-29194	Alive	TUMOR F	521					0	521	0	521			0	521
TCGA-D5	TCGA-D5	COAD	45	FEMALE	WHITE	[Discrepancy]	Colon Adenocarcino	2010	-16703	Alive	TUMOR F	380					0	380	0	380			0	380
TCGA-D5	TCGA-D5	COAD	66	MALE	WHITE	Stage I	Colon Mucinous Ade	2010	-24282	Alive	TUMOR F	491					0	491	0	491			0	491
TCGA-D5	TCGA-D5	COAD	49	MALE	WHITE	Stage IIA	Colon Adenocarcino	2010	-18029	Alive	TUMOR F	474					0	474	0	474			0	474
TCGA-D5	TCGA-D5	COAD	51	FEMALE	WHITE	Stage I	Colon Adenocarcino	2011	-18874	Alive	TUMOR F	229					0	229	0	229			0	229
TCGA-D5	TCGA-D5	COAD	77	FEMALE	WHITE	Stage IIA	Colon Adenocarcino	2011	-28124	Alive	TUMOR F	377					0	377	0	377			0	377
TCGA-D5	TCGA-D5	COAD	76	MALE	WHITE	Stage IIIA	Colon Adenocarcino	2011	-27771	Alive	TUMOR F	308					0	308	0	308			0	308
TCGA-D5	TCGA-D5	COAD	57	MALE	WHITE	Stage I	Colon Adenocarcino	2011	-21118	Alive	TUMOR F	378					0	378	0	378			0	378
TCGA-D5	TCGA-D5	COAD	68	MALE	WHITE	Stage IIA	Colon Adenocarcino	2011	-24964	Alive	TUMOR F	435					0	435	0	435			0	435
TCGA-D5	TCGA-D5	COAD	65	MALE	WHITE	Stage IIIB	Colon Adenocarcino	2011	-23768	Alive	TUMOR F	275					0	275	0	275			0	275
TCGA-D5	TCGA-D5	COAD	34	MALE	WHITE	Stage IIA	Colon Adenocarcino	2011	-12737	Alive	TUMOR F	287				[Unknown]	0	287	0	287			0	287
TCGA-D5	TCGA-D5	COAD	80	MALE	WHITE	Stage IIA	Colon Mucinous Ade	2011	-29411	Alive	TUMOR F	354					0	354	0	354			0	354
TCGA-D5	TCGA-D5	COAD	49	FEMALE	WHITE	Stage IV	Colon Adenocarcino	2011	-17919	Alive	TUMOR F	408					0	408	0	408			0	408
TCGA-D5	TCGA-D5	COAD	67	MALE	WHITE	Stage IIA	Colon Mucinous Ade	2011	-24764	Alive	TUMOR F	406					0	406	0	406			0	406
TCGA-D5	TCGA-D5	COAD	77	MALE	WHITE	Stage IIIC	Colon Adenocarcino	2011	-28292	Alive	TUMOR F	365					0	365	0	365			0	365
TCGA-D5	TCGA-D5	COAD	69	MALE	WHITE	Stage IIA	Colon Adenocarcino	2011	-25466	Alive	TUMOR F	346					0	346	0	346			0	346
TCGA-D5	TCGA-D5	COAD	79	FEMALE	WHITE	Stage I	Colon Mucinous Ade	2011	-28913	Alive	TUMOR F	312					0	312	0	312			0	312
TCGA-DM	TCGA-DM	COAD	71	FEMALE	WHITE	Stage IIA	Colon Adenocarcino	2001	-26217	Alive		3641					0	3641	0	3641			0	3641
TCGA-DM	TCGA-DM	COAD	65	MALE	WHITE	Stage IIA	Colon Adenocarcino	2000	-23966	Dead			743		484		1	743		743			1	484
TCGA-DM	TCGA-DM	COAD	68	FEMALE	WHITE	Stage IIIC	[Discrepancy]	1999	-25032	Dead			1162				1	1162		1162			0	1162
TCGA-DM	TCGA-DM	COAD	79	FEMALE	WHITE	Stage IIA	Colon Adenocarcino	2001	-28875	Alive		3974					0	3974	0	3974			0	3974
TCGA-DM	TCGA-DM	COAD	80	MALE	WHITE	Stage IIA	Colon Adenocarcino	1999	-29403	Dead		2821	2821		1920		1	2821		2821			1	1920
TCGA-DM	TCGA-DM	COAD	88	MALE	WHITE	Stage IIA	Colon Mucinous Ade	2000	-32302	Dead		1518	1518				1	1518		1518			0	1518
TCGA-DM	TCGA-DM	COAD	82	MALE	WHITE	Stage IIA	[Discrepancy]	2000	-30142	Dead			405		80		1	405		405			1	80
TCGA-DM	TCGA-DM	COAD	50	FEMALE	WHITE		Colon Adenocarcino	2000	-18311	Dead		383	383				1	383		383			0	383
TCGA-DM	TCGA-DM	COAD	67	FEMALE	WHITE	Stage IIA	Colon Adenocarcino	2000	-24514	Alive		4270					0	4270	0	4270			0	4270
TCGA-DM	TCGA-DM	COAD	71	FEMALE	WHITE	Stage IIIC	Colon Adenocarcino	1999	-26169	Dead		228	228				1	228		228			0	228
TCGA-DM	TCGA-DM	COAD	68	MALE	WHITE	Stage IIA	Colon Adenocarcino	2000	-24888	Dead		1348	1348				1	1348		1348			0	1348
TCGA-DM	TCGA-DM	COAD	82	MALE	WHITE	Stage IIIC	Colon Adenocarcino	2000	-30272	Alive		4000					0	4000	0	4000			0	4000
TCGA-DM	TCGA-DM	COAD	75	MALE	WHITE	Stage IIIB	[Discrepancy]	2000	-27708	Alive		4126					0	4126	0	4126			0	4126
TCGA-DM	TCGA-DM	COAD	70	FEMALE	WHITE	Stage IIA	Colon Mucinous Ade	1998	-25649	Dead		236	236				1	236		236			0	236
TCGA-DM	TCGA-DM	COAD	60	FEMALE	WHITE	Stage IIA	Colon Adenocarcino	2000	-22265	Alive		4233					0	4233	0	4233			0	4233
TCGA-DM	TCGA-DM	COAD	71	FEMALE	WHITE	Stage IV	Colon Mucinous Ade	2001	-26021	Dead			179				1	179		179			0	179
TCGA-DM	TCGA-DM	COAD	68	MALE	WHITE	Stage IIIC	Colon Mucinous Ade	2001	-25084	Dead		427	427		320		1	427		427			1	320
TCGA-DM	TCGA-DM	COAD	78	MALE	WHITE	Stage IIIC	Colon Adenocarcino	2001	-28833	Dead		805	805				1	805		805			0	805
TCGA-DM	TCGA-DM	COAD	74	MALE	WHITE	Stage IIA	Colon Adenocarcino	2001	-27073	Dead		2475	2475		1929		1	2475		2475			1	1929
TCGA-DM	TCGA-DM	COAD	72	FEMALE	WHITE	Stage IIA	Colon Adenocarcino	2001	-26602	Alive		3648					0	3648	0	3648			0	3648
TCGA-DM	TCGA-DM	COAD	73	MALE	WHITE	Stage IIIB	Colon Adenocarcino	2002	-26856	Dead		1094	1094				1	1094		1094			0	1094
TCGA-DM	TCGA-DM	COAD	75	MALE	WHITE	Stage IIA	Colon Adenocarcino	2001	-27641	Dead		1849	1849				1	1849		1849			0	1849
TCGA-DM	TCGA-DM	COAD	50	MALE	WHITE	Stage IIIC	Colon Adenocarcino	2002	-18325	Alive		3561		Locoregional Disease	484		0	3561	0	3561			1	484
TCGA-DM	TCGA-DM	COAD	75	MALE	WHITE	Stage IIA	Colon Mucinous Ade	2003	-27428	Alive		2988					0	2988	0	2988			0	2988
TCGA-DM	TCGA-DM	COAD	63	MALE	WHITE	Stage IIA	Colon Adenocarcino	2003	-23043	Alive		2895					0	2895	0	2895			0	2895
TCGA-F4	TCGA-F4	COAD	61	FEMALE	WHITE	Stage IIIB	Colon Adenocarcino	2011	-22574	Dead	TUMOR FREE		262				1	262	0	262			0	262
TCGA-F4	TCGA-F4	COAD	51	FEMALE	WHITE	Stage IIIB	Colon Adenocarcino	2011	-18637	Dead	WITH TUMOR		972		313	Complete Remission/Response	1	972	1	972	1	313	1	313
TCGA-F4	TCGA-F4	COAD	41	FEMALE	WHITE	Stage IIIC	Colon Adenocarcino	2011	-15151	Dead	WITH TUMOR		338		288		1	338	1	338			1	288
TCGA-F4	TCGA-F4	COAD	51	MALE	WHITE	Stage IIA	Colon Mucinous Ade	2011	-18804	Alive	TUMOR F	1087				Complete Remission/Response	0	1087	0	1087	0	1087	0	1087
TCGA-F4	TCGA-F4	COAD	60	MALE	WHITE	Stage I	Colon Adenocarcino	2011	-22203	Alive	TUMOR F	1087				Complete Remission/Response	0	1087	0	1087	0	1087	0	1087
TCGA-F4	TCGA-F4	COAD	78	FEMALE	WHITE	Stage IIA	Colon Adenocarcino	2011	-28772	Dead	TUMOR FREE		188				1	188	0	188			0	188
TCGA-F4	TCGA-F4	COAD	64	MALE	WHITE	Stage IIA	Colon Adenocarcino	2011	-23644	Alive	TUMOR F	1456				Complete Remission/Response	0	1456	0	1456	0	1456	0	1456
TCGA-F4	TCGA-F4	COAD	60	MALE	AMERICA	Stage IIIC	Colon Mucinous Ade	2011	-21949	Alive	TUMOR F	47					0	47	0	47			0	47
TCGA-F4	TCGA-F4	COAD	60	MALE	AMERICA	Stage IIIC	Colon Mucinous Ade	2011	-21949	Alive	TUMOR F	47					0	47	0	47			0	47
TCGA-F4	TCGA-F4	COAD	58	FEMALE	WHITE	Stage IIA	Colon Adenocarcino	2011	-21241	Alive	TUMOR F	1047				Complete Remission/Response	0	1047	0	1047	0	1047	0	1047
TCGA-F4	TCGA-F4	COAD	59	FEMALE	WHITE	Stage I	Colon Adenocarcino	2011	-21625	Alive	WITH TUM	1260		Metastatic	336	Complete Remission/Response	0	1260	0	1260	1	336	1	336
TCGA-F4	TCGA-F4	COAD	51	FEMALE	WHITE	Stage IIIC	Colon Adenocarcino	2011	-18690	Alive	TUMOR F	1309				Complete Remission/Response	0	1309	0	1309	0	1309	0	1309
TCGA-F4	TCGA-F4	COAD	54	FEMALE	WHITE	Stage I	Colon Adenocarcino	2011	-19803	Alive	TUMOR F	1024				Complete Remission/Response	0	1024	0	1024	0	1024	0	1024
TCGA-F4	TCGA-F4	COAD	52	FEMALE	WHITE	Stage IVA	Colon Adenocarcino	2011	-19039	Dead	WITH TUMOR		403				1	403	1	403			1	403
TCGA-F4	TCGA-F4	COAD	77	FEMALE	WHITE	Stage IIA	Colon Adenocarcino	2011	-28272	Alive	TUMOR F	16				Complete Remission/Response	0	16	0	16	0	16	0	16
TCGA-F4	TCGA-F4	COAD	70	FEMALE	WHITE	Stage IIA	Colon Adenocarcino	2011	-25890	Alive	TUMOR F	1442				Complete Remission/Response	0	1442	0	1442	0	1442	0	1442

TCGA-F4-	TCGA-F4-	COAD	45	MALE	WHITE	Stage I		Colon Mucinous Ade		2011	-16519	Alive	TUMOR F	1074						Complete Remission/Response	0	1074	0	1074	0	1074	0	1074
TCGA-G4	TCGA-G4	COAD	49	FEMALE	WHITE	Stage III		Colon Adenocarcino		2002	-18076	Alive	TUMOR F	4051						Complete Remission/Response	0	4051	0	4051	0	4051	0	4051
TCGA-G4	TCGA-G4	COAD	75	MALE	WHITE	Stage IV		Colon Adenocarcino		2004	-27755	Dead	WITH TUMOR		858				517		1	858	1	858			1	517
TCGA-G4	TCGA-G4	COAD	70	FEMALE	WHITE	Stage II		Colon Adenocarcino		2004	-25758	Alive	TUMOR F	254							0	254	0	254			0	254
TCGA-G4	TCGA-G4	COAD	70	FEMALE	WHITE	Stage II		Colon Adenocarcino		2004	-25758	Alive	TUMOR F	254							0	254	0	254			0	254
TCGA-G4	TCGA-G4	COAD	55	FEMALE	WHITE	Stage IV		Colon Adenocarcino		2005	-20103	Alive	WITH TUI	2506					439		0	2506	0	2506			1	439
TCGA-G4	TCGA-G4	COAD	55	FEMALE	WHITE	Stage IV		Colon Adenocarcino		2005	-20103	Alive	WITH TUI	2506					439		0	2506	0	2506			1	439
TCGA-G4	TCGA-G4	COAD	90	MALE	BLACK OI	Stage IIIB		Colon Adenocarcino		2005	-32873	Dead	WITH TUMOR		715				291		1	715	1	715			1	291
TCGA-G4	TCGA-G4	COAD	90	MALE	BLACK OI	Stage IIIB		Colon Adenocarcino		2005	-32873	Dead	WITH TUMOR		715				291		1	715	1	715			1	291
TCGA-G4	TCGA-G4	COAD	69	MALE	WHITE	Stage IIIC		Colon Adenocarcino		2005	-25318	Alive	TUMOR F	2268							0	2268	0	2268			0	2268
TCGA-G4	TCGA-G4	COAD	90	FEMALE	WHITE	Stage IIA		Colon Mucinous Ade		2006	-32872	Dead	TUMOR FREE		2047						1	2047	0	2047			0	2047
TCGA-G4	TCGA-G4	COAD	90	FEMALE	WHITE	Stage IIA		Colon Mucinous Ade		2006	-32872	Dead	TUMOR FREE		2047						1	2047	0	2047			0	2047
TCGA-G4	TCGA-G4	COAD	54	FEMALE	WHITE	Stage IV		Colon Adenocarcino		2006	-19800	Dead	WITH TUMOR		2003				872		1	2003	1	2003			1	872
TCGA-G4	TCGA-G4	COAD	66	FEMALE	WHITE	Stage IIB		Colon Adenocarcino		2006	-24172	Alive	TUMOR F	1631					859		0	1631	0	1631			1	859
TCGA-G4	TCGA-G4	COAD	71	MALE	WHITE	[Discrepancy]		Colon Adenocarcino		2007	-26163	Alive	TUMOR F	1359							0	1359	0	1359			0	1359
TCGA-G4	TCGA-G4	COAD	37	FEMALE	WHITE	Stage IIIB		Colon Adenocarcino		2007	-13648	Alive	TUMOR F	1674							0	1674	0	1674			0	1674
TCGA-G4	TCGA-G4	COAD	40	FEMALE	BLACK OI	Stage IIIB		Colon Adenocarcino		2007	-14729	Alive	TUMOR F	2600					2176	Complete Remission/Response	0	2600	0	2600	1	2176	1	2176
TCGA-G4	TCGA-G4	COAD	69	MALE	WHITE	Stage IIIB		Colon Adenocarcino		2008	-25243	Alive	TUMOR F	1935						Complete Remission/Response	0	1935	0	1935	0	1935	0	1935
TCGA-G4	TCGA-G4	COAD	80	MALE	WHITE	Stage III		Colon Adenocarcino		2008	-29297	Alive	TUMOR F	1199							0	1199	0	1199			0	1199
TCGA-G4	TCGA-G4	COAD	80	MALE	WHITE	Stage III		Colon Adenocarcino		2008	-29297	Alive	TUMOR F	1199							0	1199	0	1199			0	1199
TCGA-G4	TCGA-G4	COAD	76	FEMALE	WHITE	Stage IV		Colon Adenocarcino		2008	-27958	Alive	WITH TUI	1093					38		0	1093	0	1093			1	38
TCGA-G4	TCGA-G4	COAD	76	FEMALE	WHITE	Stage IV		Colon Adenocarcino		2008	-27958	Alive	WITH TUI	1093					38		0	1093	0	1093			1	38
TCGA-G4	TCGA-G4	COAD	66	MALE	WHITE	Stage IV		Colon Adenocarcino		2008	-24174	Alive	TUMOR F	1883						Complete Remission/Response	0	1883	0	1883			0	1883
TCGA-G4	TCGA-G4	COAD	51	FEMALE	WHITE	Stage IIIC		Colon Adenocarcino		2008	-18822	Alive	TUMOR F	1095		Metastatic			592		0	1095	0	1095			1	592
TCGA-G4	TCGA-G4	COAD	51	FEMALE	WHITE	Stage IIIC		Colon Adenocarcino		2008	-18822	Alive	TUMOR F	1095		Metastatic			592		0	1095	0	1095			1	592
TCGA-G4	TCGA-G4	COAD	73	MALE	WHITE	Stage III		Colon Adenocarcino		2009	-26840	Alive	TUMOR F	804							0	804	0	804			0	804
TCGA-G4	TCGA-G4	COAD	73	MALE	WHITE	Stage III		Colon Adenocarcino		2009	-26840	Alive	TUMOR F	804							0	804	0	804			0	804
TCGA-G4	TCGA-G4	COAD	60	FEMALE	WHITE	Stage III		Colon Adenocarcino		2009	-21921	Alive	TUMOR F	672							0	672	0	672			0	672
TCGA-G4	TCGA-G4	COAD	65	MALE	WHITE	Stage IIIB		Colon Mucinous Ade		2009	-23825	Alive	WITH TUI	792					789		0	792	0	792			1	789
TCGA-G4	TCGA-G4	COAD	65	MALE	WHITE	Stage IIIB		Colon Mucinous Ade		2009	-23825	Alive	WITH TUI	792					789		0	792	0	792			1	789
TCGA-G4	TCGA-G4	COAD	50	MALE	BLACK OI	Stage IA		Colon Adenocarcino		2010	-18334	Alive	TUMOR F	419							0	419	0	419			0	419
TCGA-G4	TCGA-G4	COAD	73	FEMALE	WHITE	Stage IIA		Colon Adenocarcino		2008	-27020	Alive	TUMOR F	1089							0	1089	0	1089			0	1089
TCGA-G4	TCGA-G4	COAD	58	FEMALE	WHITE	Stage IIA		Colon Adenocarcino		2009	-21532	Alive	TUMOR F	796							0	796	0	796			0	796
TCGA-G4	TCGA-G4	COAD	77	FEMALE	WHITE	Stage IIA		Colon Adenocarcino		2004	-28163	Alive	TUMOR F	2792					2564		0	2792	0	2792			1	2564
TCGA-G4	TCGA-G4	COAD	77	FEMALE	WHITE	Stage IIA		Colon Adenocarcino		2004	-28163	Alive	TUMOR F	2792					2564		0	2792	0	2792			1	2564
TCGA-G4	TCGA-G4	COAD	90	MALE	WHITE	Stage IIA		Colon Adenocarcino		2006	-32871	Dead	TUMOR FREE		1422						1	1422	0	1422			0	1422
TCGA-G4	TCGA-G4	COAD	84	MALE	WHITE	Stage IIA		Colon Adenocarcino		2007	-30697	Alive	TUMOR F	2275					1003	Complete Remission/Response	0	2275	0	2275	1	1003	1	1003
TCGA-G4	TCGA-G4	COAD	78	MALE	WHITE	Stage I		Colon Adenocarcino		2006	-28772	Alive	TUMOR F	2424						Complete Remission/Response	0	2424	0	2424	0	2424	0	2424
TCGA-NH	TCGA-NH	COAD	68	FEMALE	BLACK OI	Stage IIA		Colon Adenocarcino		2012	-25089	Alive	TUMOR F	553						Complete Remission/Response	0	553	0	553	0	553	0	553
TCGA-NH	TCGA-NH	COAD	42	MALE	BLACK OI	Stage IVA		Colon Mucinous Ade		2012	-15450	Dead	WITH TUMOR		334				276	Partial Remission/Response	1	334	1	334			1	276
TCGA-NH	TCGA-NH	COAD	69	MALE	BLACK OI	Stage IIIB		Colon Adenocarcino		2012	-25467	Alive	TUMOR F	588						Complete Remission/Response	0	588	0	588	0	588	0	588
TCGA-NH	TCGA-NH	COAD	90	FEMALE	BLACK OI	Stage IIA		Colon Adenocarcino		2012	-32872	Alive	TUMOR F	588						Complete Remission/Response	0	588	0	588	0	588	0	588
TCGA-NH	TCGA-NH	COAD	58	MALE	BLACK OI	Stage IIIC		Colon Adenocarcino		2013	-21313	Dead	WITH TUMOR		302				6	Progressive Disease	1	302	1	302			1	6
TCGA-NH	TCGA-NH	COAD	71	FEMALE	BLACK OI	Stage IIIC		Colon Adenocarcino		2013	-26100	Alive	WITH TUI	476						Progressive Disease	0	476	0	476			0	476
TCGA-NH	TCGA-NH	COAD	66	FEMALE	BLACK OI	Stage IVB		Colon Mucinous Ade		2013	-24355	Alive		389						[Unknown]	0	389	0	389			0	389
TCGA-NH	TCGA-NH	COAD	53	FEMALE	BLACK OI	Stage IIA		Colon Adenocarcino		2013	-19535	Alive	WITH TUI	543						Partial Remission/Response	0	543	0	543			0	543
TCGA-NH	TCGA-NH	COAD	53	FEMALE	BLACK OI	Stage IIA		Colon Adenocarcino		2013	-19535	Alive	WITH TUI	543						Partial Remission/Response	0	543	0	543			0	543
TCGA-NH	TCGA-NH	COAD	79	MALE	BLACK OI	Stage IV		Colon Adenocarcino		2013	-29185	Dead	WITH TUMOR		511					[Unknown]	1	511	1	511			1	511
TCGA-OC	TCGA-OC	COAD	64	FEMALE	BLACK OI	Stage IIIC		Colon Adenocarcino		2011	-23499	Alive	TUMOR F	1301						Complete Remission/Response	0	1301	0	1301	0	1301	0	1301
TCGA-OC	TCGA-OC	COAD	55	FEMALE	BLACK OI	Stage IIIC		Colon Mucinous Ade		2011	-20391	Alive	TUMOR F	896						Complete Remission/Response	0	896	0	896	0	896	0	896
TCGA-OC	TCGA-OC	COAD	61	FEMALE	BLACK OI	Stage IIA		Colon Adenocarcino		2011	-22617	Alive	TUMOR F	1003						Complete Remission/Response	0	1003	0	1003	0	1003	0	1003
TCGA-OC	TCGA-OC	COAD	71	MALE	BLACK OI	Stage IIIB		Colon Adenocarcino		2011	-26220	Dead	TUMOR FREE		256						1	256	0	256			0	256
TCGA-OC	TCGA-OC	COAD	61	MALE	BLACK OI	Stage I		Colon Adenocarcino		2012	-22367	Alive	TUMOR F	952						Complete Remission/Response	0	952	0	952	0	952	0	952
TCGA-QL	TCGA-QL	COAD	84	FEMALE	BLACK OI	Stage I		Colon Adenocarcino		2012	-30914	Alive	TUMOR F	666						Complete Remission/Response	0	666	0	666	0	666	0	666
TCGA-RU	TCGA-RU	COAD	51	MALE	BLACK OI	Stage IIIB		Colon Adenocarcino		2011	-18975	Alive	WITH TUI	1177		Metastatic			256	Partial Remission/Response	0	1177	0	1177			1	256
TCGA-SS-	TCGA-SS-	COAD	44	FEMALE	BLACK OI	Stage IIB		Colon Adenocarcino		2010	-16416	Alive	WITH TUI	1829		Locoregional Disease			506	Complete Remission/Response	0	1829	0	1829	1	506	1	506
TCGA-T9-	TCGA-T9-	COAD	82	MALE	BLACK OI	Stage IIA		Colon Adenocarcino		2013	-30058	Alive	WITH TUI	362					81	Complete Remission/Response	0	362	0	362	1	81	1	81
TCGA-WS	TCGA-WS	COAD	52	FEMALE	BLACK OI	Stage IIA		Colon Mucinous Ade		2008		Alive	TUMOR F	2130						Complete Remission/Response	0	2130	0	2130	0	2130	0	2130
TCGA-FA	TCGA-FA	DLBC	75	MALE	WHITE		Stage I	Diffuse large B-cell l		2012	-27468	Alive	TUMOR F	132						Complete Remission/Response	0	132	0	132	0	132	0	132
TCGA-FA	TCGA-FA	DLBC	67	MALE	WHITE		Stage IV	Diffuse large B-cell l		2012	-24590	Alive	TUMOR F	132						Complete Remission/Response	0	132	0	132			0	132
TCGA-FA	TCGA-FA	DLBC	40	MALE	ASIAN		Stage II	Diffuse large B-cell l		2012	-14723	Dead	TUMOR FREE		708					Complete Remission/Response	1	708	0	708	0	708	0	708
TCGA-FA	TCGA-FA	DLBC	73	MALE	ASIAN			Diffuse large B-cell l		2013	-27025	Dead	TUMOR FREE		58					Progressive Disease	1	58	0	58			0	58
TCGA-FA	TCGA-FA	DLBC	58	FEMALE	ASIAN			Diffuse large B-cell l		2013	-21330	Alive	WITH TUI	749			Paraaortic lymph noc		739	Complete Remission/Response	0	749	0	749	1	739	1	739
TCGA-FA	TCGA-FA	DLBC	54	FEMALE	ASIAN		Stage II	Diffuse large B-cell l		2013	-20087	Alive	TUMOR F	719						Complete Remission/Response	0	719	0	719	0	719	0	719
TCGA-FA	TCGA-FA	DLBC	61	FEMALE	ASIAN		Stage I	Diffuse large B-cell l		2013	-22454	Dead	TUMOR FREE		248					Complete Remission/Response	1	248	0	248	0	248	0	248
TCGA-FA	TCGA-FA	DLBC	48	FEMALE	ASIAN		Stage II	Diffuse large B-cell l		2013	-17819	Alive	TUMOR F	427						Complete Remission/Response	0	427	0	427	0	427	0	427
TCGA-FA	TCGA-FA	DLBC	64	FEMALE	WHITE		Stage III	Diffuse large B-cell l		2013	-23641	Alive	WITH TUI	553			Other, sp	Inguinal, r	150	Partial Remission/Response	0	553	0	553			1	150
TCGA-FF-	TCGA-FF-	DLBC	28	FEMALE	ASIAN		Stage I	Primary mediastinal (		2010	-10466	Alive	TUMOR F	1081						Complete Remission/Response	0	1081	0	1081	0	1081	0	1081
TCGA-FF-	TCGA-FF-	DLBC	57	FEMALE	ASIAN		Stage II	Diffuse large B-cell l		2010	-21005	Alive	TUMOR F	946						Complete Remission/Response	0	946	0	946	0	946	0	946
TCGA-FF-	TCGA-FF-	DLBC	70	FEMALE	ASIAN		Stage II	Diffuse large B-cell l		2010	-25612	Alive	TUMOR F	965						Complete Remission/Response	0	965	0	965	0	965	0	965
TCGA-FF-	TCGA-FF-	DLBC	51	MALE	ASIAN		Stage II	Diffuse large B-cell l		2011	-18853	Alive	TUMOR F	751						Complete Remission/Response	0	751	0	751	0	751	0	751
TCGA-FF-	TCGA-FF-	DLBC	38	FEMALE	ASIAN		Stage II	Diffuse large B-cell l		2011	-13996	Alive	WITH TUI	126			Leptomeninges		24	Progressive Disease	0	126	0	126			1	24
TCGA-FF-	TCGA-FF-	DLBC	42	MALE	ASIAN		Stage II	Diffuse large B-cell l		2010	-15358	Alive	TUMOR F	832						Complete Remission/Response	0	832	0	832	0	832	0	832
TCGA-FF-	TCGA-FF-	DLBC	60	FEMALE	ASIAN		Stage I	Diffuse large B-cell l		2011	-21959	Alive	TUMOR F	679						Complete Remission/Response	0	679	0	679	0	679	0	679
TCGA-FF-	TCGA-FF-	DLBC	74	MALE	ASIAN		Stage IV	Diffuse large B-cell l		2010	-27255	Alive		298			Brain		255	Stable Disease	0	298	0	298			1	255
TCGA-FF-	TCGA-FF-	DLBC	61	MALE	ASIAN		Stage IV	Diffuse large B-cell l		2012	-22471	Dead	WITH TUMOR		313		Other, sp	Skin (Calf	290	Progressive Disease	1	313	1	313			1	290
TCGA-FF-	TCGA-FF-	DLBC	72	MALE	ASIAN		Stage II	Diffuse large B-cell l		2012	-26520	Alive	TUMOR F	791						Complete Remission/Response	0	791	0	791	0	791	0	791
TCGA-FF-	TCGA-FF-	DLBC	46	MALE	ASIAN		Stage IV	Diffuse large B-cell l		2011	-16924	Alive	TUMOR F	385						Complete Remission/Response	0	385	0	385			0	385
TCGA-FM	TCGA-FM	DLBC	70	FEMALE	WHITE		Stage II	Diffuse large B-cell l		2011	-25724	Alive	TUMOR F	972						Complete Remission/Response	0	972	0	972	0	972	0	972
TCGA-G8	TCGA-G8	DLBC	43	FEMALE	BLACK OR AFRICAN		Stage IV	Diffuse large B-cell l		2006	-15750	Dead	WITH TUMOR		1252		Supraclavicular lymp		338	Complete Remission/Response	1	1252	1	1252			1	338
TCGA-G8	TCGA-G8	DLBC	69	MALE	WHITE		Stage IV	Diffuse large B-cell l		2008	-25256	Alive	TUMOR F	1581						Stable Disease	0	1581	0	1581			0	1581
TCGA-G8	TCGA-G8	DLBC	52	MALE	WHITE		Stage IV	Diffuse large B-cell l		2009	-19238	Alive	TUMOR F	2131						Progressive Disease	0	2131	0	2131			0	2131
TCGA-G8	TCGA-G8	DLBC	58	MALE	WHITE			Diffuse large B-cell l		1987	-21272	Dead	TUMOR FREE		6425				6383	Complete Remission/Response	1	6425	0	6425	1	6383	1	6383
TCGA-G8	TCGA-G8	DLBC	57	FEMALE	WHITE		Stage IV	Diffuse large B-cell l		2000	-21021	Dead	WITH TUMOR		595		Ovary		279	Stable Disease	1	595	1	595			1	279
TCGA-G8	TCGA-G8	DLBC	67	MALE	WHITE		Stage III	Diffuse large B-cell l		2002	-24661	Dead	TUMOR FREE		3553					Complete Remission/Response	1	3553	0	3553	0	3553	0	3553
TCGA-G8	TCGA-G8	DLBC	67	MALE	WHITE		Stage III	Diffuse large B-cell l		1996	-24569	Alive	TUMOR F	5980					3669	Complete Remission/Response	0	5980	0	5980	1	3669	1	3669
TCGA-GR	TCGA-GR	DLBC	56	FEMALE	WHITE		Stage I	Diffuse large B-cell l		2001	-20812	Alive	TUMOR F	4578						Complete Remission/Response	0	4578	0	4578	0	4578	0	4578
TCGA-GR	TCGA-GR	DLBC	36	MALE	WHITE		Stage II	Diffuse large B-cell l		2005	-13317	Alive	TUMOR F	2983						Complete Remission/Response	0	2983	0	2983	0	2983	0	2983
TCGA-GR	TCGA-GR	DLBC	57	FEMALE	WHITE		Stage IV	Diffuse large B-cell l		2010	-20888	Alive	TUMOR F	1581						Complete Remission/Response	0	1581	0	1581			0	1581
TCGA-GR	TCGA-GR	DLBC	46	FEMALE	WHITE		Stage IV	Diffuse large B-cell l		2010	-17023	Alive	TUMOR F	1299						Complete Remission/Response	0	1299	0	1299			0	1299
TCGA-GR	TCGA-GR	DLBC	54	FEMALE	ASIAN		Stage I	Diffuse large B-cell l		2010	-19838	Alive	TUMOR F	1334						Complete Remission/Response	0	1334	0	1334	0	1334	0	1334
TCGA-GR	TCGA-GR	DLBC	66	FEMALE	WHITE		Stage IV	Diffuse large B-cell l		2009	-24405	Alive	WITH TUI	1739			Soft Tissue		1218	Complete Remission/Response	0	1739	0	1739			1	1218
TCGA-GS	TCGA-GS	DLBC	69	FEMALE	WHITE		Stage II	Diffuse large B-cell l		2012	-25292	Alive	TUMOR F	982						Complete Remission/Response	0	982	0	982	0	982	0	982
TCGA-GS	TCGA-GS	DLBC	70	MALE	WHITE		Stage IV	Diffuse large B-cell l		2011	-25794	Alive	TUMOR F	1163						Complete Remission/Response	0	1163	0	1163			0	1163
TCGA-GS	TCGA-GS	DLBC	45	MALE	WHITE		Stage II	Diffuse large B-cell l		2004	-16720	Alive	TUMOR F	3897						Complete Remission/Response	0	3897	0	3897	0	3897	0	3897
TCGA-GS	TCGA-GS	DLBC	40	FEMALE	WHITE		Stage II	Primary mediastinal (		2013	-14838	Alive	TUMOR F	730						Complete Remission/Response	0	730	0	730	0	730	0	730
TCGA-GS	TCGA-GS	DLBC	23	FEMALE	WHITE		Stage II	Primary mediastinal (		2010	-8493	Alive	TUMOR F	1617						Complete Remission/Response	0	1617	0	1617	0	1617	0	1617
TCGA-GS	TCGA-GS	DLBC	37	MALE	WHITE		Stage I	Diffuse large B-cell l		2011	-13708	Alive	TUMOR F	1373						Complete Remission/Response	0	1373	0	1373	0	1373	0	1373
TCGA-GS	TCGA-GS	DLBC	82	FEMALE	WHITE		Stage I	Diffuse large B-cell l		2005	-30256	Alive	WITH TUI	3333			Pleura/Pleural effusic		3255	Complete Remission/Response	0	3333	0	3333	1	3255	1	3255
TCGA-GS	TCGA-GS	DLBC	51	MALE	WHITE		Stage II	Primary mediastinal (		2012	-18691	Alive	TUMOR F	765						Partial Remission/Response	0	765	0	765			0	765
TCGA-GS	TCGA-GS	DLBC	77	FEMALE	WHITE		Stage III	Diffuse large B-cell l		2005	-28474	Alive	TUMOR F	3394						Complete Remission/Response	0	3394	0	3394	0	3394	0	3394
TCGA-GS	TCGA-GS	DLBC	27	FEMALE	WHITE		Stage II	Diffuse large B-cell l		2011	-10075	Dead	WITH TUMOR		391		Cervical Lymph Node		106	Progressive Disease	1	391	1	391			1	106
TCGA-RQ	TCGA-RQ	DLBC	63	MALE	WHITE			Primary DLBCL of the		2012	-23127	Alive	TUMOR F	788						Partial Remission/Response	0	788	0	788			0	788
TCGA-RQ	TCGA-RQ	DLBC	62	FEMALE	WHITE			Primary DLBCL of the		2011	-22859	Alive		1						[Unknown]	0	1	0	1			0	1
TCGA-RQ	TCGA-RQ	DLBC	63	FEMALE	WHITE			Primary DLBCL of the		2013	-23178	Alive	TUMOR F	17						Complete Remission/Response	0	17	0	17	0	17	0	17
TCGA-VB	TCGA-VB	DLBC	55	MALE	WHITE		Stage III	Diffuse large B-cell l		2013	-20270	Alive	TUMOR F	0						[Unknown]	0	0	0	0			0	0
TCGA-2H	TCGA-2H	ESCA	67	MALE	[Unknown	Stage III		Esophagu	G3	2001	-24487	Dead	WITH TUMOR		784	Distant M	Brain		172		1	784	1	784			1	172
TCGA-2H	TCGA-2H	ESCA	66	MALE	[Unknown	Stage III		Esophagu	G2	1999	-24328	Dead	WITH TUMOR		610	Distant M	Brain		504		1	610	1	610			1	504
TCGA-2H	TCGA-2H	ESCA	44	MALE	[Unknown	Stage IIB		Esophagu	G2	1998	-16197	Dead	WITH TUMOR		951	Locoregional Recurrence			800		1	951	1	951			1	800
TCGA-2H	TCGA-2H	ESCA	68	MALE	[Unknown	Stage III		Esophagu	G2	1999	-25097	Dead	WITH TUMOR		435	Locoregional Recurrence			400		1	435	1	435			1	400
TCGA-2H	TCGA-2H	ESCA	57	MALE	[Unknown	Stage I		Esophagu	G2	2000	-21180	Dead	WITH TUMOR		1781	Locoregional Recurrence			1261		1	1781	1	1781			1	1261
TCGA-2H	TCGA-2H	ESCA	43	MALE	[Unknown	Stage III		Esophagu	G3	2000	-16067	Dead	WITH TUMOR		232	Distant M	Other, sp	Lymph nc	113		1	232	1	232			1	113
TCGA-2H	TCGA-2H	ESCA	74	MALE	[Unknown	Stage III		Esophagu	G3	2000	-27115	Dead	WITH TUMOR		180	Distant Metastasis			109		1	180	1	180			1	109
TCGA-2H	TCGA-2H	ESCA	53	MALE	[Unknown	Stage IIB		Esophagu	G2	2000	-19484	Dead	WITH TUMOR		424	Distant M	Other, sp	peritoneu	367		1	424	1	424			1	367
TCGA-2H	TCGA-2H	ESCA	70	MALE	[Unknown	Stage III		Esophagu	G3	2000	-25664	Dead	WITH TUMOR		272	Distant M	Brain		242		1	272	1	272			1	242
TCGA-2H	TCGA-2H	ESCA	58	MALE	[Unknown	Stage IVA		Esophagu	G3	2000	-21486	Dead	WITH TUMOR		494	Locoregional Recurrence			362		1	494	1	494			1	362
TCGA-2H	TCGA-2H	ESCA	80	MALE	[Unknown	Stage III		Esophagu	G3	2001	-29497	Dead	WITH TUMOR		128	Locoregional Recurrence			112		1	128	1	128			1	112
TCGA-2H	TCGA-2H	ESCA	80	MALE	[Unknown	Stage IIA		Esophagu	G2	1999	-29451	Dead	WITH TUMOR		987						1	987	1	987			1	987
TCGA-IC-	TCGA-IC-	ESCA	59	MALE	WHITE	Stage IIB		Esophagu	G2	2013	-21640	Alive	WITH TUI	234						Complete Remission/Response	0	234	0	234	0	234	0	234
TCGA-IC-	TCGA-IC-	ESCA	59	MALE	WHITE	Stage IIB		Esophagu	G2	2013	-21640	Alive	WITH TUI	234						Complete Remission/Response	0	234	0	234	0	234	0	234
TCGA-IC-	TCGA-IC-	ESCA	69	FEMALE	WHITE	Stage IA	Stage IIB	Esophagu	GX	2013	-25417	Alive	TUMOR F	477		Locoregic	Other, sp	regional l	293	Complete Remission/Response	0	477	0	477	1	293	1	293
TCGA-IC-	TCGA-IC-	ESCA	69	FEMALE	WHITE	Stage IA	Stage IIB	Esophagu	GX	2013	-25417	Alive	TUMOR F	477		Locoregic	Other, sp	regional l	293	Complete Remission/Response	0	477	0	477	1	293	1	293
TCGA-IG-	TCGA-IG-	ESCA	51	FEMALE	WHITE	Stage IIA		Esophagu	G2	2011	-18786	Alive	TUMOR F	1012						Complete Remission/Response	0	1012	0	1012	0	1012	0	1012
TCGA-IG-	TCGA-IG-	ESCA	51	FEMALE	WHITE	Stage IIA		Esophagu	G2	2011	-18786	Alive	TUMOR F	1012						Complete Remission/Response	0	1012	0	1012	0	1012	0	1012
TCGA-IG-	TCGA-IG-	ESCA	54	MALE	WHITE	Stage IIA		Esophagu	G2	2011	-19755	Alive	TUMOR F	1071						Complete Remission/Response	0	1071	0	1071	0	1071	0	1071
TCGA-IG-	TCGA-IG-	ESCA	72	MALE	WHITE	Stage IIIA		Esophagu	G2	2012	-26330	Dead	TUMOR FREE		26					Complete Remission/Response	1	26	0	26	0	26	0	26
TCGA-IG-	TCGA-IG-	ESCA	53	MALE	WHITE	Stage IIIA		Esophagu	G2	2012	-19567	Alive	TUMOR F	632						Complete Remission/Response	0	632	0	632	0	632	0	632
TCGA-IG-	TCGA-IG-	ESCA	61	MALE	WHITE	Stage IIIA		Esophagu	G2	2012	-22316	Alive	TUMOR F	80						Complete Remission/Response	0	80	0	80	0	80	0	80
TCGA-IG-	TCGA-IG-	ESCA	62	MALE	WHITE	Stage IIIA		Esophagu	G2	2012	-22774	Alive	WITH TUI	612		New Prim	Lung		542	Stable Disease	0	612	0	612			1	542
TCGA-IG-	TCGA-IG-	ESCA	48	MALE	WHITE	Stage IIB		Esophagu	G2	2012	-17860	Dead	WITH TUMOR		567					Progressive Disease	1	567	1	567			1	567
TCGA-IG-	TCGA-IG-	ESCA	71	MALE	WHITE	Stage IIIB		Esophagu	G3	2012	-26273	Dead	TUMOR FREE		118					Complete Remission/Response	1	118	0	118	0	118	0	118
TCGA-IG-	TCGA-IG-	ESCA	56	MALE	WHITE	Stage IIA		Esophagu	G2	2012	-20506	Dead	TUMOR FREE		283					Complete Remission/Response	1	283	0	283	0	283	0	283
TCGA-IG-	TCGA-IG-	ESCA	58	MALE	ASIAN	Stage IIIA	Stage IIIA	Esophagu	G2	2012	-21214	Alive	TUMOR F	16							0	16	0	16			0	16
TCGA-IG-	TCGA-IG-	ESCA	63	MALE	WHITE	Stage IIB	Stage IV	Esophagu	G2	2012	-23259	Alive	TUMOR F	518						Complete Remission/Response	0	518	0	518			0	518
TCGA-IG-	TCGA-IG-	ESCA	72	MALE	WHITE	Stage IB		Esophagu	G1	2012	-26574	Dead	TUMOR FREE		24						1	24	0	24			0	24
TCGA-IG-	TCGA-IG-	ESCA	69	FEMALE	WHITE	Stage IIB		Esophagu	G2	2012	-25414	Alive	TUMOR F	712						Complete Remission/Response	0	712	0	712	0	712	0	712
TCGA-IG-	TCGA-IG-	ESCA	60	MALE	ASIAN	Stage IIIB		Esophagu	G2	2012	-22070	Dead	TUMOR FREE		390					[Unknown]	1	390	0	390			0	390
TCGA-IG-	TCGA-IG-	ESCA	54	MALE	WHITE	Stage IIB		Esophagu	G1	2013	-19900	Dead	TUMOR FREE		303					Complete Remission/Response	1	303	0	303	0	303	0	303
TCGA-IG-	TCGA-IG-	ESCA	50	FEMALE	WHITE	Stage IIIA		Esophagu	G2	2013	-18364	Alive	TUMOR F	452						Complete Remission/Response	0	452	0	452	0	452	0	452
TCGA-IG-	TCGA-IG-	ESCA	62	MALE	ASIAN	Stage IIIB		Esophagu	G3	2013	-22869	Dead	TUMOR FREE		142					Complete Remission/Response	1	142	0	142	0	142	0	142
TCGA-IG-	TCGA-IG-	ESCA	36	MALE	ASIAN	Stage IIA		Esophagu	G3	2013	-13166	Alive	TUMOR F	441						Complete Remission/Response	0	441	0	441	0	441	0	441
TCGA-IG-	TCGA-IG-	ESCA	58	MALE	WHITE	Stage IIA		Esophagu	G2	2013	-21271	Alive	TUMOR F	370						Complete Remission/Response	0	370	0	370	0	370	0	370
TCGA-JY-	TCGA-JY-	ESCA	56	MALE	WHITE	Stage I		Esophagu	G1	2003	-20550	Alive	TUMOR F	3714						Complete Remission/Response	0	3714	0	3714	0	3714	0	3714
TCGA-JY-	TCGA-JY-	ESCA	51	MALE	ASIAN	Stage IIB		Esophagu	G2	2006	-18978	Dead	WITH TUMOR		1361	Distant M	Lung		477		1	1361	1	1361			1	477
TCGA-JY-	TCGA-JY-	ESCA	77	MALE	WHITE	Stage I		Esophagu	G2	2007	-28269	Alive	TUMOR F	1837						Complete Remission/Response	0	1837	0	1837	0	1837	0	1837
TCGA-JY-	TCGA-JY-	ESCA	51	FEMALE	ASIAN	Stage IIA		Esophagu	G1	2008	-18808	Alive	TUMOR F	2069						Complete Remission/Response	0	2069	0	2069	0	2069	0	2069
TCGA-JY-	TCGA-JY-	ESCA	49	MALE	WHITE	Stage III		Esophagu	G3	2009	-18105	Dead	WITH TUMOR		112	Distant M	Other, sp	Pleural M	55		1	112	1	112			1	55
TCGA-JY-	TCGA-JY-	ESCA	50	MALE	WHITE	Stage III		Esophagu	G2	2009	-18348	Dead	TUMOR FREE		1263						1	1263	0	1263			0	1263
TCGA-JY-	TCGA-JY-	ESCA	53	MALE	WHITE	Stage IIB		Esophagu	G2	2010	-19549	Alive	TUMOR F	1441		Distant M	Lung		749	Progressive Disease	0	1441	0	1441			1	749
TCGA-JY-	TCGA-JY-	ESCA	75	MALE	WHITE	Stage IIB		Esophagu	G3	2012	-27633	Alive	WITH TUI	1060		Distant M	Other, sp	Adrenal G	493	Complete Remission/Response	0	1060	0	1060	1	493	1	493
TCGA-JY-	TCGA-JY-	ESCA	77	MALE	WHITE	Stage IIB		Esophagu	G3	2012	-28181	Alive	WITH TUI	660						Stable Disease	0	660	0	660			0	660
TCGA-JY-	TCGA-JY-	ESCA	47	MALE	ASIAN	Stage IIIB		Esophagu	G3	2011	-17228	Alive	WITH TUI	705		Locoregional Recurrence			652	Complete Remission/Response	0	705	0	705	1	652	1	652
TCGA-JY-	TCGA-JY-	ESCA	51	MALE	WHITE	Stage IIIC		Esophagu	G3	2012	-18813	Dead	WITH TUMOR		960	Locoregic	Other, sp	Mediastin	732	Complete Remission/Response	1	960	1	960	1	732	1	732
TCGA-JY-	TCGA-JY-	ESCA	61	MALE	WHITE	Stage IIIA		Esophagu	G3	2012	-22427	Alive	WITH TUI	767						Stable Disease	0	767	0	767			0	767
TCGA-JY-	TCGA-JY-	ESCA	58	FEMALE	WHITE	Stage IB		Esophagu	G1	2012	-21257	Alive	TUMOR F	731						Complete Remission/Response	0	731	0	731	0	731	0	731
TCGA-KH	TCGA-KH	ESCA	82	MALE	WHITE	Stage IA	Stage IA	Esophagu	GX	2013	-30167	Alive	TUMOR F	191						Complete Remission/Response	0	191	0	191	0	191	0	191
TCGA-L5-	TCGA-L5-	ESCA	81	MALE	WHITE		Stage III	Esophagu	GX	2009	-29753	Alive	TUMOR F	96						[Unknown]	0	96	0	96			0	96
TCGA-L5-	TCGA-L5-	ESCA	81	MALE	WHITE		Stage III	Esophagu	GX	2009	-29753	Alive	TUMOR F	96						[Unknown]	0	96	0	96			0	96
TCGA-L5-	TCGA-L5-	ESCA	74	MALE	WHITE	Stage I		Esophagu	GX	2011	-27143	Alive	TUMOR F	920		New Prim	Other, sp	Right diac	885	Complete Remission/Response	0	920	0	920	0	920	1	885
TCGA-L5-	TCGA-L5-	ESCA	75	MALE	WHITE	Stage III		Esophagu	GX	2011	-27592	Dead	WITH TUMOR		9					Complete Remission/Response	1	9	1	9			1	9
TCGA-L5-	TCGA-L5-	ESCA	77	MALE	WHITE	Stage IIIA		Esophagu	GX	2011	-28367	Dead	TUMOR FREE		556					Complete Remission/Response	1	556	0	556	0	556	0	556
TCGA-L5-	TCGA-L5-	ESCA	90	MALE	WHITE	Stage IIB		Esophagu	G3	2011	-32872	Dead	WITH TUMOR		131	Distant M	Liver		98	Progressive Disease	1	131	1	131			1	98
TCGA-L5-	TCGA-L5-	ESCA	84	FEMALE	[Not Evalu	[Unknown]		Esophagu	GX	2009	-30783	Alive		272						[Unknown]	0	272	0	272			0	272
TCGA-L5-	TCGA-L5-	ESCA	81	MALE	WHITE	Stage IIIB		Esophagu	G2	2011	-29591	Dead			730					Complete Remission/Response	1	730		730	0	730	0	730
TCGA-L5-	TCGA-L5-	ESCA	81	MALE	WHITE	Stage IIIB		Esophagu	G2	2011	-29591	Dead			730					Complete Remission/Response	1	730		730	0	730	0	730
TCGA-L5-	TCGA-L5-	ESCA	63	MALE	WHITE	Stage IIB		Esophagu	G3	2012	-23194	Dead			801					Complete Remission/Response	1	801		801	0	801	0	801
TCGA-L5-	TCGA-L5-	ESCA	63	MALE	WHITE	Stage IIB		Esophagu	G3	2012	-23194	Dead			801					Complete Remission/Response	1	801		801	0	801	0	801
TCGA-L5-	TCGA-L5-	ESCA	79	FEMALE	WHITE	Stage I		Esophagu	G1	2012	-29105	Alive		143						[Unknown]	0	143	0	143			0	143
TCGA-L5-	TCGA-L5-	ESCA	79	FEMALE	WHITE	Stage I		Esophagu	G1	2012	-29105	Alive		143						[Unknown]	0	143	0	143			0	143
TCGA-L5-	TCGA-L5-	ESCA	71	MALE	WHITE	Stage I		Esophagu	GX	2012	-26180	Alive	TUMOR F	992						Complete Remission/Response	0	992	0	992	0	992	0	992
TCGA-L5-	TCGA-L5-	ESCA	71	MALE	WHITE	Stage I		Esophagu	GX	2012	-26180	Alive	TUMOR F	992						Complete Remission/Response	0	992	0	992	0	992	0	992
TCGA-L5-	TCGA-L5-	ESCA	79	MALE	WHITE	Stage IIIC		Esophagu	G2	2012	-29190	Alive	WITH TUI	608		Locoregic	Other, sp	Lymph Nc	267	Stable Disease	0	608	0	608			1	267
TCGA-L5-	TCGA-L5-	ESCA	79	MALE	WHITE	Stage IIIC		Esophagu	G2	2012	-29190	Alive	WITH TUI	608		Locoregic	Other, sp	Lymph Nc	267	Stable Disease	0	608	0	608			1	267
TCGA-L5-	TCGA-L5-	ESCA	70	FEMALE	WHITE	Stage I		Esophagu	G3	2012	-25657	Alive	TUMOR F	639						Complete Remission/Response	0	639	0	639	0	639	0	639
TCGA-L5-	TCGA-L5-	ESCA	70	FEMALE	WHITE	Stage I		Esophagu	G3	2012	-25657	Alive	TUMOR F	639						Complete Remission/Response	0	639	0	639	0	639	0	639
TCGA-L5-	TCGA-L5-	ESCA	54	FEMALE	WHITE	Stage IA		Esophagu	GX	2010	-19845	Dead	TUMOR FREE		1458					Complete Remission/Response	1	1458	0	1458	0	1458	0	1458
TCGA-L5-	TCGA-L5-	ESCA	54	FEMALE	WHITE	Stage IA		Esophagu	GX	2010	-19845	Dead	TUMOR FREE		1458					Complete Remission/Response	1	1458	0	1458	0	1458	0	1458
TCGA-L5-	TCGA-L5-	ESCA	65	MALE	WHITE	Stage IIB		Esophagu	GX	2010	-23970	Dead	TUMOR FREE		558					Complete Remission/Response	1	558	0	558	0	558	0	558
TCGA-L5-	TCGA-L5-	ESCA	65	MALE	WHITE	Stage IIB		Esophagu	GX	2010	-23970	Dead	TUMOR FREE		558					Complete Remission/Response	1	558	0	558	0	558	0	558
TCGA-L5-	TCGA-L5-	ESCA	75	MALE	WHITE	Stage IIIC		Esophagu	G2	2010	-27757	Alive		101						[Unknown]	0	101	0	101			0	101
TCGA-L5-	TCGA-L5-	ESCA	75	MALE	WHITE	Stage IIIC		Esophagu	G2	2010	-27757	Alive		101						[Unknown]	0	101	0	101			0	101
TCGA-L5-	TCGA-L5-	ESCA	67	FEMALE	WHITE	Stage IA		Esophagu	G2	2010	-24829	Alive	TUMOR F	218						[Unknown]	0	218	0	218			0	218
TCGA-L5-	TCGA-L5-	ESCA	67	FEMALE	WHITE	Stage IA		Esophagu	G2	2010	-24829	Alive	TUMOR F	218						[Unknown]	0	218	0	218			0	218
TCGA-L5-	TCGA-L5-	ESCA	75	MALE	WHITE	Stage IIIA		Esophagu	GX	2010	-27565	Dead	WITH TUMOR		42	Distant M	Brain		29		1	42	1	42			1	29
TCGA-L5-	TCGA-L5-	ESCA	75	MALE	WHITE	Stage IIIA		Esophagu	GX	2010	-27565	Dead	WITH TUMOR		42	Distant M	Brain		29		1	42	1	42			1	29
TCGA-L5-	TCGA-L5-	ESCA	83	MALE	WHITE	Stage IA		Esophagu	GX	2010	-30417	Dead	TUMOR FREE		96					Complete Remission/Response	1	96	0	96	0	96	0	96
TCGA-L5-	TCGA-L5-	ESCA	83	MALE	WHITE	Stage IA		Esophagu	GX	2010	-30417	Dead	TUMOR FREE		96					Complete Remission/Response	1	96	0	96	0	96	0	96
TCGA-L5-	TCGA-L5-	ESCA	86	FEMALE	WHITE	Stage IIB		Esophagu	G3	2010	-31601	Alive	TUMOR F	1782						[Unknown]	0	1782	0	1782			0	1782
TCGA-L5-	TCGA-L5-	ESCA	77	MALE	WHITE	Stage IV		Esophagu	GX	2006	-28162	Dead	WITH TUMOR		149					Stable Disease	1	149	1	149			1	149
TCGA-L5-	TCGA-L5-	ESCA	81	MALE	WHITE	Stage IIA		Esophagu	GX	2009	-29674	Alive	TUMOR F	882						[Unknown]	0	882	0	882			0	882
TCGA-L5-	TCGA-L5-	ESCA	56	FEMALE	[Not Evalu	Stage IIB		Esophagu	GX	2006	-20796	Dead	TUMOR FREE		217					Complete Remission/Response	1	217	0	217	0	217	0	217
TCGA-L5-	TCGA-L5-	ESCA	60	MALE	WHITE	Stage IIB		Esophagu	GX	2006	-22015	Dead	TUMOR FREE		226					Complete Remission/Response	1	226	0	226	0	226	0	226
TCGA-L5-	TCGA-L5-	ESCA	84	MALE	WHITE	Stage IB		Esophagu	GX	2013	-30954	Alive	TUMOR F	471						Complete Remission/Response	0	471	0	471	0	471	0	471
TCGA-L5-	TCGA-L5-	ESCA	86	MALE	WHITE	Stage IIB		Esophagu	GX	2012	-31480	Alive	TUMOR F	694						Complete Remission/Response	0	694	0	694	0	694	0	694
TCGA-L5-	TCGA-L5-	ESCA	60	MALE	WHITE	Stage III		Esophagu	GX	2007	-22066	Alive	TUMOR F	79						[Unknown]	0	79	0	79			0	79
TCGA-L5-	TCGA-L5-	ESCA	67	MALE	WHITE	Stage IIA		Esophagu	GX	2007	-24716	Dead	WITH TUMOR		764	Distant M	Liver		315		1	764	1	764			1	315
TCGA-L5-	TCGA-L5-	ESCA	76	MALE	WHITE	[Unknown]		Esophagu	GX	2006	-28061	Alive		11						[Unknown]	0	11	0	11			0	11
TCGA-L5-	TCGA-L5-	ESCA	70	FEMALE	WHITE	Stage IIA		Esophagu	GX	2005	-25890	Alive	TUMOR F	225						[Unknown]	0	225	0	225			0	225
TCGA-L5-	TCGA-L5-	ESCA	51	MALE	WHITE	[Unknown]		Esophagu	GX	2005	-18925	Alive	TUMOR F	114						[Unknown]	0	114	0	114			0	114
TCGA-L5-	TCGA-L5-	ESCA	71	FEMALE	WHITE	Stage I		Esophagu	GX	2005	-26143	Alive	TUMOR F	92						[Unknown]	0	92	0	92			0	92
TCGA-L5-	TCGA-L5-	ESCA	77	MALE	WHITE	Stage IIB		Esophagu	GX	2000	-28243	Alive	TUMOR F	1688						[Unknown]	0	1688	0	1688			0	1688
TCGA-L5-	TCGA-L5-	ESCA	57	MALE	WHITE	Stage IVA		Esophagu	GX	2001	-21134	Dead	TUMOR FREE		81						1	81	0	81			0	81
TCGA-L5-	TCGA-L5-	ESCA	77	MALE	WHITE	Stage III		Esophagu	GX	2001	-28219	Alive	TUMOR F	1094						[Unknown]	0	1094	0	1094			0	1094
TCGA-L5-	TCGA-L5-	ESCA	54	MALE	WHITE	Stage IV		Esophagu	GX	2001	-19991	Dead	TUMOR FREE		393						1	393	0	393			0	393
TCGA-L5-	TCGA-L5-	ESCA	79	MALE	WHITE	Stage III		Esophagu	G3	2004	-29135	Dead	WITH TUMOR		410	Locoregional Recurrence			401		1	410	1	410			1	401
TCGA-L5-	TCGA-L5-	ESCA	77	MALE	WHITE	Stage III		Esophagu	GX	2000	-28200	Alive	WITH TUI	501		Distant M	Lung		396	[Unknown]	0	501	0	501			1	396
TCGA-L5-	TCGA-L5-	ESCA	84	FEMALE	WHITE	Stage IIA		Esophagu	G1	2004	-30719	Alive	WITH TUI	412		Locoregional Recurrence			325	[Unknown]	0	412	0	412			1	325
TCGA-L5-	TCGA-L5-	ESCA	56	MALE	WHITE	Stage III		Esophagu	GX	2004	-20542	Alive	TUMOR F	402						[Unknown]	0	402	0	402			0	402
TCGA-L5-	TCGA-L5-	ESCA	84	FEMALE	WHITE	Stage IIB		Esophagu	GX	2004	-30936	Dead	TUMOR FREE		236						1	236	0	236			0	236
TCGA-L5-	TCGA-L5-	ESCA	81	MALE	WHITE	Stage III		Esophagu	GX	2004	-29891	Alive		167						[Unknown]	0	167	0	167			0	167
TCGA-L5-	TCGA-L5-	ESCA	71	MALE	WHITE	Stage IIA		Esophagu	G3	2001	-26250	Dead	WITH TUMOR		650	Locoregional Recurrence			337		1	650	1	650			1	337
TCGA-L5-	TCGA-L5-	ESCA	81	FEMALE	[Not Evalu	Stage III		Esophagu	GX	2001	-29730	Alive	WITH TUI	265		Distant M	Liver		265	[Unknown]	0	265	0	265			1	265
TCGA-L5-	TCGA-L5-	ESCA	76	MALE	WHITE	Stage IIB		Esophagu	GX	2001	-27800	Alive	WITH TUI	408		Distant M	Lung		300	[Unknown]	0	408	0	408			1	300
TCGA-L5-	TCGA-L5-	ESCA	69	MALE	WHITE	Stage IIB		Esophagu	GX	2001	-25259	Alive	WITH TUI	825		Distant M	Liver		825	[Unknown]	0	825	0	825			1	825
TCGA-L5-	TCGA-L5-	ESCA	84	MALE	WHITE	Stage IIA		Esophagu	G3	2001	-30738	Dead	TUMOR FREE		2532						1	2532	0	2532			0	2532
TCGA-L5-	TCGA-L5-	ESCA	75	MALE	WHITE	Stage IIA		Esophagu	G3	2001	-27546	Dead	WITH TUMOR		1599	Locoregional Recurrence			1252		1	1599	1	1599			1	1252
TCGA-L5-	TCGA-L5-	ESCA	55	MALE	WHITE	[Discrepancy]		Esophagu	GX	2001	-20194	Dead	WITH TUMOR		1402	Locoregional Recurrence			1147		1	1402	1	1402			1	1147
TCGA-L7-	TCGA-L7-	ESCA	65	MALE	BLACK OR AFRICAN AMERICAN			Esophagu	G3	2012	-24106	Dead	WITH TUMOR		330	Distant M	Lung		270	Stable Disease	1	330	1	330			1	270
TCGA-L7-	TCGA-L7-	ESCA	62	MALE	WHITE	Stage IIIC		Esophagu	G2	2013	-22730	Alive	TUMOR F	315						Complete Remission/Response	0	315	0	315	0	315	0	315
TCGA-LN	TCGA-LN	ESCA	66	MALE	ASIAN	Stage IIA	Stage IIA	Esophagu	G2	2010	-24379	Dead	TUMOR FREE		180					Complete Remission/Response	1	180	0	180	0	180	0	180
TCGA-LN	TCGA-LN	ESCA	44	MALE	ASIAN	Stage IIA	Stage IIA	Esophagu	G2	2010	-16164	Dead	TUMOR FREE		318					Complete Remission/Response	1	318	0	318	0	318	0	318
TCGA-LN	TCGA-LN	ESCA	62	MALE	ASIAN	Stage IIA	Stage IIA	Esophagu	G1	2010	-22933	Alive	TUMOR F	385						Complete Remission/Response	0	385	0	385	0	385	0	385
TCGA-LN	TCGA-LN	ESCA	50	MALE	ASIAN	Stage IIB	Stage IIB	Esophagu	G2	2010	-18503	Alive	TUMOR F	378						Complete Remission/Response	0	378	0	378	0	378	0	378
TCGA-LN	TCGA-LN	ESCA	47	MALE	ASIAN	Stage IIA	Stage IIA	Esophagu	G3	2011	-17237	Alive	WITH TUI	408		Locoregional Recurrence			193	Complete Remission/Response	0	408	0	408	1	193	1	193
TCGA-LN	TCGA-LN	ESCA	71	MALE	ASIAN	Stage IIA	Stage IIA	Esophagu	G2	2011	-26189	Alive	WITH TUI	375		Locoregional Recurrence			320	Complete Remission/Response	0	375	0	375	1	320	1	320
TCGA-LN	TCGA-LN	ESCA	46	MALE	ASIAN	Stage III	Stage III	Esophagu	G3	2011	-17109	Alive	TUMOR F	407						Complete Remission/Response	0	407	0	407	0	407	0	407
TCGA-LN	TCGA-LN	ESCA	59	MALE	ASIAN	Stage IIA	Stage IIA	Esophagu	G2	2011	-21865	Alive	WITH TUI	400		Locoregional Recurrence			49	Complete Remission/Response	0	400	0	400	1	49	1	49
TCGA-LN	TCGA-LN	ESCA	62	MALE	ASIAN	Stage IIA	Stage IIA	Esophagu	G1	2011	-22969	Alive	TUMOR F	467						Complete Remission/Response	0	467	0	467	0	467	0	467
TCGA-LN	TCGA-LN	ESCA	49	MALE	ASIAN	Stage IIA	Stage IIA	Esophagu	G3	2011	-17922	Alive	TUMOR F	383						Complete Remission/Response	0	383	0	383	0	383	0	383
TCGA-LN	TCGA-LN	ESCA	73	MALE	ASIAN	Stage III	Stage III	Esophagu	G3	2011	-26910	Alive	TUMOR F	403						Complete Remission/Response	0	403	0	403	0	403	0	403
TCGA-LN	TCGA-LN	ESCA	44	MALE	ASIAN	Stage IIA	Stage IIA	Esophagu	G2	2011	-16154	Alive	TUMOR F	384						Complete Remission/Response	0	384	0	384	0	384	0	384
TCGA-LN	TCGA-LN	ESCA	77	MALE	ASIAN	Stage IIA	Stage IIA	Esophagu	G2	2011	-28125	Alive	WITH TUI	379		Locoregional Recurrence			90	Complete Remission/Response	0	379	0	379	1	90	1	90
TCGA-LN	TCGA-LN	ESCA	60	MALE	ASIAN	Stage IIA	Stage IIA	Esophagu	G3	2011	-21954	Alive	WITH TUI	383		Locoregional Recurrence			205	Complete Remission/Response	0	383	0	383	1	205	1	205
TCGA-LN	TCGA-LN	ESCA	57	MALE	ASIAN	Stage IIA	Stage IIA	Esophagu	G1	2011	-21122	Alive	TUMOR F	380						Complete Remission/Response	0	380	0	380	0	380	0	380
TCGA-LN	TCGA-LN	ESCA	61	MALE	ASIAN	Stage III	Stage III	Esophagu	G2	2011	-22313	Alive	TUMOR F	388						Complete Remission/Response	0	388	0	388	0	388	0	388
TCGA-LN	TCGA-LN	ESCA	36	MALE	ASIAN	Stage III	Stage III	Esophagu	GX	2011	-13419	Alive	TUMOR F	383						Complete Remission/Response	0	383	0	383	0	383	0	383
TCGA-LN	TCGA-LN	ESCA	49	MALE	ASIAN	Stage IIA	Stage IIA	Esophagu	G2	2011	-18160	Dead	WITH TUMOR		681	Locoregional Recurrence			224	Complete Remission/Response	1	681	1	681	1	224	1	224
TCGA-LN	TCGA-LN	ESCA	65	MALE	ASIAN	Stage II	Stage II	Esophagu	G2	2012	-24011	Alive	TUMOR F	391		Locoregional Recurrence			158	Complete Remission/Response	0	391	0	391	1	158	1	158
TCGA-LN	TCGA-LN	ESCA	52	MALE	ASIAN	Stage IIA	Stage IIA	Esophagu	G2	2012	-19046	Alive	TUMOR F	472		Locoregional Recurrence			250	Complete Remission/Response	0	472	0	472	1	250	1	250
TCGA-LN	TCGA-LN	ESCA	58	MALE	ASIAN	Stage IIA	Stage IIA	Esophagu	G1	2012	-21217	Dead	WITH TUMOR		351	Locoregional Recurrence			274	Complete Remission/Response	1	351	1	351	1	274	1	274
TCGA-LN	TCGA-LN	ESCA	46	MALE	ASIAN	Stage III	Stage III	Esophagu	G3	2010	-16990	Alive	TUMOR F	375		Locoregional Recurrence			25	Complete Remission/Response	0	375	0	375	1	25	1	25
TCGA-LN	TCGA-LN	ESCA	57	MALE	ASIAN	Stage IIA	Stage IIA	Esophagu	G2	2012	-21142	Alive	TUMOR F	402		Locoregional Recurrence			226	Complete Remission/Response	0	402	0	402	1	226	1	226
TCGA-LN	TCGA-LN	ESCA	57	MALE	ASIAN	Stage IV		Esophagu	G2	2012	-20993	Dead	WITH TUMOR		136					Progressive Disease	1	136	1	136			1	136
TCGA-LN	TCGA-LN	ESCA	54	MALE	ASIAN	Stage IIB		Esophagu	G2	2012	-19840	Alive	TUMOR F	375						Complete Remission/Response	0	375	0	375	0	375	0	375
TCGA-LN	TCGA-LN	ESCA	46	MALE	ASIAN	Stage IIA		Esophagu	G1	2012	-16928	Alive	TUMOR F	768		Locoregional Recurrence			535	Complete Remission/Response	0	768	0	768	1	535	1	535
TCGA-LN	TCGA-LN	ESCA	58	MALE	ASIAN	Stage IIA		Esophagu	G1	2012	-21462	Alive	TUMOR F	320						Complete Remission/Response	0	320	0	320	0	320	0	320
TCGA-LN	TCGA-LN	ESCA	59	MALE	ASIAN	Stage IIA		Esophagu	G2	2013	-21840	Alive	TUMOR F	365		Locoregional Recurrence			97	Complete Remission/Response	0	365	0	365	1	97	1	97
TCGA-LN	TCGA-LN	ESCA	72	MALE	ASIAN	Stage IIA		Esophagu	G2	2013	-26517	Alive	TUMOR F	372						Complete Remission/Response	0	372	0	372	0	372	0	372
TCGA-LN	TCGA-LN	ESCA	50	MALE	ASIAN	Stage III		Esophagu	G2	2013	-18296	Alive	TUMOR F	366		Locoregional Recurrence			88	Complete Remission/Response	0	366	0	366	1	88	1	88
TCGA-LN	TCGA-LN	ESCA	49	MALE	ASIAN	Stage IIA		Esophagu	G2	2013	-17931	Alive	TUMOR F	401		Locoregional Recurrence			117	Complete Remission/Response	0	401	0	401	1	117	1	117
TCGA-LN	TCGA-LN	ESCA	56	MALE	ASIAN	Stage IIA		Esophagu	G3	2013	-20510	Alive	TUMOR F	375						Complete Remission/Response	0	375	0	375	0	375	0	375
TCGA-LN	TCGA-LN	ESCA	52	MALE	ASIAN	Stage IIA		Esophagu	G2	2013	-19328	Alive	TUMOR F	407		Locoregional Recurrence			57	Complete Remission/Response	0	407	0	407	1	57	1	57
TCGA-LN	TCGA-LN	ESCA	67	FEMALE	ASIAN	Stage IIA		Esophagu	G3	2013	-24551	Alive	TUMOR F	401						Complete Remission/Response	0	401	0	401	0	401	0	401
TCGA-LN	TCGA-LN	ESCA	42	MALE	ASIAN	Stage IIA		Esophagu	G2	2013	-15551	Alive	TUMOR F	4							0	4	0	4			0	4
TCGA-LN	TCGA-LN	ESCA	60	FEMALE	ASIAN	Stage IIA		Esophagu	G3	2013	-22104	Alive	WITH TUI	366		Locoregional Recurrence			290	Complete Remission/Response	0	366	0	366	1	290	1	290
TCGA-LN	TCGA-LN	ESCA	62	MALE	ASIAN	Stage IIA		Esophagu	G3	2013	-22717	Alive	TUMOR F	391		Locoregional Recurrence			82	Complete Remission/Response	0	391	0	391	1	82	1	82
TCGA-LN	TCGA-LN	ESCA	70	MALE	ASIAN	Stage IIB		Esophagu	G2	2013	-25657	Alive	TUMOR F	373						Complete Remission/Response	0	373	0	373	0	373	0	373
TCGA-MS	TCGA-MS	ESCA	58	MALE	[Not Evalu	Stage IIA		Esophagu	G2		-21433	Alive	TUMOR F	1007						Complete Remission/Response	0	1007	0	1007	0	1007	0	1007
TCGA-Q9	TCGA-Q9	ESCA	57	FEMALE	WHITE	Stage IIIB		Esophagu	G2	2012	-21108	Dead	WITH TUMOR		157	Distant M	Liver		99		1	157	1	157			1	99
TCGA-Q9	TCGA-Q9	ESCA	61	MALE	WHITE	Stage IIIB		Esophagu	G2	2013	-22586	Alive	TUMOR F	238						Complete Remission/Response	0	238	0	238	0	238	0	238
TCGA-R6-	TCGA-R6-	ESCA	58	MALE	WHITE		Stage IV	Esophagu	G3	2011	-21259	Dead	WITH TUMOR		243	Distant M	Other, sp	Brain	199	Partial Remission/Response	1	243	1	243			1	199
TCGA-R6-	TCGA-R6-	ESCA	74	FEMALE	WHITE		Stage IIIA	Esophagu	G3	2010	-27098	Dead	WITH TUMOR		231						1	231	1	231			1	231
TCGA-R6-	TCGA-R6-	ESCA	42	MALE	WHITE		Stage IV	Esophagu	G3	2010	-15512	Dead	WITH TUMOR		154						1	154	1	154			1	154
TCGA-R6-	TCGA-R6-	ESCA	27	MALE	WHITE		Stage IIIA	Esophagu	G2	2012	-10222	Dead	WITH TUMOR		496	Distant M	Liver		160	Progressive Disease	1	496	1	496			1	160
TCGA-R6-	TCGA-R6-	ESCA	68	MALE	WHITE		Stage IV	Esophagu	G3	2010	-25092	Dead	WITH TUMOR		214						1	214	1	214			1	214
TCGA-R6-	TCGA-R6-	ESCA	64	MALE	WHITE		Stage IIIA	Esophagu	G2	2011	-23484	Alive	WITH TUI	1168		Distant M	Other, sp	brain	572	Complete Remission/Response	0	1168	0	1168	1	572	1	572
TCGA-R6-	TCGA-R6-	ESCA	58	MALE	WHITE		Stage IVB	Esophagu	G3	2008	-21410	Dead	WITH TUMOR		193						1	193	1	193			1	193
TCGA-R6-	TCGA-R6-	ESCA	54	MALE	WHITE		Stage IVA	Esophagu	G2	2005	-20045	Alive	WITH TUI	1641						Stable Disease	0	1641	0	1641			0	1641
TCGA-R6-	TCGA-R6-	ESCA	71	MALE	WHITE		Stage IIB	Esophagu	G3	2005	-25940	Dead	WITH TUMOR		283	Distant M	Bone		102		1	283	1	283			1	102
TCGA-R6-	TCGA-R6-	ESCA	60	MALE	WHITE	Stage IVA		Esophagu	G2	2004	-21917	Dead	WITH TUMOR		480	Distant M	Liver		336		1	480	1	480			1	336
TCGA-R6-	TCGA-R6-	ESCA	72	MALE	WHITE		Stage IIIA	Esophagu	G2	2012	-26535	Dead	WITH TUMOR		88						1	88	1	88			1	88
TCGA-R6-	TCGA-R6-	ESCA	56	MALE	WHITE		Stage IIA	Esophagu	G2	2013	-20812	Alive	WITH TUI	70							0	70	0	70			0	70
TCGA-R6-	TCGA-R6-	ESCA	60	MALE	WHITE		Stage IIA	Esophagu	G3	2013	-22093	Dead	WITH TUMOR		386	Distant M	Other, sp	left suprac	107		1	386	1	386			1	107
TCGA-RE	TCGA-RE	ESCA	72	FEMALE	WHITE	Stage IIB		Esophagu	G3	2006	-26348	Dead	TUMOR FREE		213						1	213	0	213			0	213
TCGA-S8-	TCGA-S8-	ESCA	76	MALE	WHITE	Stage IIIA	Stage IB	Esophagu	G2	2012	-28111	Alive	WITH TUI	609		Distant M	Liver		267	Progressive Disease	0	609	0	609			1	267
TCGA-S8-	TCGA-S8-	ESCA	51	MALE	WHITE	Stage IB		Esophagu	G1	2012	-18944	Alive	TUMOR F	620						Complete Remission/Response	0	620	0	620	0	620	0	620
TCGA-V5	TCGA-V5	ESCA	59	MALE	[Unknown	[Unknown	Stage IIB	Esophagu	GX		-21654	Dead	TUMOR FREE		161						1	161	0	161			0	161
TCGA-V5	TCGA-V5	ESCA	55	MALE	BLACK OR AFRICAN		Stage IV	Esophagu	GX		-20439	Dead	WITH TUMOR		104	Distant M	Liver		88	Progressive Disease	1	104	1	104			1	88
TCGA-V5	TCGA-V5	ESCA	55	MALE	BLACK OR AFRICAN		Stage IV	Esophagu	GX		-20439	Dead	WITH TUMOR		104	Distant M	Liver		88	Progressive Disease	1	104	1	104			1	88
TCGA-V5	TCGA-V5	ESCA	45	MALE	WHITE	Stage IB	Stage IIIA	Esophagu	G2		-16643	Alive	WITH TUI	500		Distant M	Other, sp	bone	294	Partial Remission/Response	0	500	0	500			1	294
TCGA-V5	TCGA-V5	ESCA	45	MALE	WHITE	Stage IB	Stage IIIA	Esophagu	G2		-16643	Alive	WITH TUI	500		Distant M	Other, sp	bone	294	Partial Remission/Response	0	500	0	500			1	294
TCGA-V5	TCGA-V5	ESCA	67	MALE	BLACK OI	Stage IIB		Esophagu	G3		-24559	Alive	WITH TUI	467		Distant M	Lung		322	Partial Remission/Response	0	467	0	467			1	322
TCGA-V5	TCGA-V5	ESCA	72	MALE	WHITE		Stage IIIA	Esophagu	G3		-26571	Alive	WITH TUI	282		Distant M	Lung		282	[Unknown]	0	282	0	282			1	282
TCGA-V5	TCGA-V5	ESCA	74	MALE	WHITE		Stage IV	Esophagu	G2		-27081	Alive	WITH TUI	273		Distant M	Other, sp	bone	273	[Unknown]	0	273	0	273			1	273
TCGA-V5	TCGA-V5	ESCA	74	MALE	WHITE		Stage IV	Esophagu	G2		-27081	Alive	WITH TUI	273		Distant M	Other, sp	bone	273	[Unknown]	0	273	0	273			1	273
TCGA-VR	TCGA-VR	ESCA	49	MALE	WHITE	Stage IIA	Stage IIA	Esophagu	G3	2013	-18123	Alive	TUMOR F	785						Complete Remission/Response	0	785	0	785	0	785	0	785
TCGA-VR	TCGA-VR	ESCA	51	MALE	WHITE	Stage IIIB	Stage IIB	Esophagu	G3	2013	-18942	Alive	TUMOR F	824		Locoregional Recurrence			242	Progressive Disease	0	824	0	824			1	242
TCGA-VR	TCGA-VR	ESCA	73	MALE	WHITE	Stage III	Stage III	Esophagu	G2	2009	-26912	Dead	WITH TUMOR		694						1	694	1	694			1	694
TCGA-VR	TCGA-VR	ESCA	54	MALE	[Unknown	Stage III	Stage III	Esophagu	GX	2009	-19951	Dead	WITH TUMOR		378						1	378	1	378			1	378
TCGA-VR	TCGA-VR	ESCA	64	MALE	WHITE	Stage IIA	Stage IIA	Esophagu	G2	2010	-23517	Dead	TUMOR FREE		47						1	47	0	47			0	47

TCGA-VR-	TCGA-VR-	ESCA	51	MALE	WHITE	Stage IV	Stage IV	Esophagu	G2	2011	-18711	Dead	WITH TUMOR		557						1	557	1	557			1	557
TCGA-VR-	TCGA-VR-	ESCA	57	MALE	ASIAN	Stage IIIB	Stage IIIB	Esophagu	G2	2012	-20902	Dead	WITH TUMOR		247						1	247	1	247			1	247
TCGA-VR-	TCGA-VR-	ESCA	63	MALE	[Unknown	Stage IVA	Stage IVA	Esophagu	G2	2009	-23226	Dead	WITH TUMOR		855						1	855	1	855			1	855
TCGA-VR-	TCGA-VR-	ESCA	44	FEMALE	WHITE	Stage IIA	Stage IIA	Esophagu	G1	2012	-16255	Alive	TUMOR F	1025						Complete Remission/Response	0	1025	0	1025	0	1025	0	1025
TCGA-VR-	TCGA-VR-	ESCA	47	MALE	WHITE	Stage IIIC	Stage IIA	Esophagu	G2	2013	-17449	Dead	WITH TUMOR		553	Distant M	Bone		379		1	553	1	553			1	379
TCGA-VR-	TCGA-VR-	ESCA	60	MALE	WHITE	Stage IIIA	Stage IIIA	Esophagu	G3	2010	-22149	Alive	TUMOR F	1590						Complete Remission/Response	0	1590	0	1590	0	1590	0	1590
TCGA-VR-	TCGA-VR-	ESCA	53	MALE	WHITE	Stage IIB	Stage IA	Esophagu	G2	2010	-19714	Dead	WITH TUMOR		1405	Distant M	Lung		832	Progressive Disease	1	1405	1	1405			1	832
TCGA-VR-	TCGA-VR-	ESCA	51	FEMALE	WHITE	Stage IIIA	Stage IIIA	Esophagu	G2	2013	-18909	Alive	TUMOR F	549						Complete Remission/Response	0	549	0	549	0	549	0	549
TCGA-VR-	TCGA-VR-	ESCA	65	FEMALE	WHITE	Stage IV	Stage IIB	Esophagu	G2	2011	-23915	Alive	WITH TUM	342		Distant M	Brain		207		0	342	0	342			1	207
TCGA-VR-	TCGA-VR-	ESCA	58	MALE	[Unknown	Stage IIIC	Stage IIIC	Esophagu	G2	2012	-21468	Dead	WITH TUMOR		279	Distant M	Other, sp	Head and	244		1	279	1	279			1	244
TCGA-VR-	TCGA-VR-	ESCA	70	MALE	WHITE	Stage III	Stage III	Esophagu	G1	2010	-25855	Dead	WITH TUMOR		484						1	484	1	484			1	484
TCGA-X8-	TCGA-X8-	ESCA	69	MALE	WHITE	[Unknown]		Esophagu	G1	2013	-25370	Alive	TUMOR F	554						Complete Remission/Response	0	554	0	554	0	554	0	554
TCGA-XP-	TCGA-XP-	ESCA	54	MALE	BLACK OI	Stage IIB		Esophagu	G2	2009	-19886	Dead	TUMOR FREE		763					Complete Remission/Response	1	763	0	763	0	763	0	763
TCGA-XP-	TCGA-XP-	ESCA	63	FEMALE	WHITE	Stage IIA		Esophagu	G2	2010	-23375	Alive	TUMOR F	1254						Complete Remission/Response	0	1254	0	1254	0	1254	0	1254
TCGA-XP-	TCGA-XP-	ESCA	49	MALE	BLACK OI	Stage IIB	Stage IIB	Esophagu	G1	2011	-18048	Alive	TUMOR F	437						Complete Remission/Response	0	437	0	437	0	437	0	437
TCGA-Z6-	TCGA-Z6-	ESCA	53	MALE	WHITE	Stage IIB		Esophagu	G2	2013	-19523	Alive	TUMOR F	104						Complete Remission/Response	0	104	0	104	0	104	0	104
TCGA-Z6-	TCGA-Z6-	ESCA	57	MALE	WHITE	Stage IIIA	Stage IIIA	Esophagu	G3	2013	-20855	Alive	TUMOR F	64						Complete Remission/Response	0	64	0	64	0	64	0	64
TCGA-Z6-	TCGA-Z6-	ESCA	53	MALE	WHITE	Stage IIIA		Esophagu	G2	2013	-19470	Alive	TUMOR F	40						Complete Remission/Response	0	40	0	40	0	40	0	40
TCGA-Z6-	TCGA-Z6-	ESCA	57	MALE	WHITE	Stage IIA		Esophagu	G1	2013	-20926	Alive	TUMOR F	81						Complete Remission/Response	0	81	0	81	0	81	0	81
TCGA-ZR-	TCGA-ZR-	ESCA	65	MALE	WHITE	Stage IIIC		Esophagu	G3	2012	-23752	Dead	WITH TUMOR		600	Distant M	Liver		163	[Unknown]	1	600	1	600			1	163
TCGA-02-	TCGA-02-	GBM	44	FEMALE	WHITE			Untreated primary (c		2002	-16179	Dead	WITH TUM	279	358	Recurrence			137		1	358	1	358			1	137
TCGA-02-	TCGA-02-	GBM	50	MALE	WHITE			Untreated primary (c		2003	-18341	Dead	WITH TUM	144	144	Recurrence			40		1	144	1	144			1	40
TCGA-02-	TCGA-02-	GBM	56	FEMALE	WHITE			Untreated primary (c		2002	-20516	Dead	WITH TUM	558	558	Recurrence			302		1	558	1	558			1	302
TCGA-02-	TCGA-02-	GBM	40	FEMALE	WHITE			Treated primary GBM		2002	-14806	Dead	WITH TUM	705	705	Progression of Disease			518		1	705	1	705			1	518
TCGA-02-	TCGA-02-	GBM	61	FEMALE	WHITE			Untreated primary (c		2003	-22457	Dead	WITH TUM	322	322	Progression of Disease			264		1	322	1	322			1	264
TCGA-02-	TCGA-02-	GBM	20	FEMALE	WHITE			Treated primary GBM		2002	-7452	Dead	WITH TUM	1077	1077	Progression of Disease			351		1	1077	1	1077			1	351
TCGA-02-	TCGA-02-	GBM	18	FEMALE	WHITE			Untreated primary (c		2004	-6926	Dead	WITH TUM	630	630	Progression of Disease			145		1	630	1	630			1	145
TCGA-02-	TCGA-02-	GBM	25	MALE	WHITE			Untreated primary (c		1997	-9369	Dead	WITH TUM	2512	2512	Progression of Disease			2243		1	2512	1	2512			1	2243
TCGA-02-	TCGA-02-	GBM	50	MALE	WHITE			Untreated primary (c		2002	-18404	Dead	WITH TUM	627	627	Recurrence			456		1	627	1	627			1	456
TCGA-02-	TCGA-02-	GBM	50	MALE	WHITE			Untreated primary (c		2004	-18271	Dead	WITH TUMOR		2648	Recurrence			1699		1	2648	1	2648			1	1699
TCGA-02-	TCGA-02-	GBM	43	FEMALE	WHITE			Untreated primary (c		1999	-16035	Dead	WITH TUM	2362	2362	Progression of Disease			255		1	2362	1	2362			1	255
TCGA-02-	TCGA-02-	GBM	38	FEMALE	WHITE			Untreated primary (c		2003	-14003	Dead	WITH TUM	612	612	Progression of Disease			435		1	612	1	612			1	435
TCGA-02-	TCGA-02-	GBM	35	MALE	WHITE			Treated primary GBM		2000	-13116	Dead	WITH TUM	1615	1615	Recurrence			1401		1	1615	1	1615			1	1401
TCGA-02-	TCGA-02-	GBM	47	MALE	WHITE			Untreated primary (c		2002	-17402	Dead	WITH TUM	1300	1300	Progression of Disease			656		1	1300	1	1300			1	656
TCGA-02-	TCGA-02-	GBM	27	MALE	WHITE			Untreated primary (c		2003	-10022	Dead	WITH TUM	748	748	Progression of Disease			462		1	748	1	748			1	462
TCGA-02-	TCGA-02-	GBM	33	FEMALE	WHITE			Untreated primary (c		2005	-12369	Dead	WITH TUM	315	370	Progression of Disease			257		1	370	1	370			1	257
TCGA-02-	TCGA-02-	GBM	39	MALE	WHITE			Treated primary GBM		1998	-14304	Dead	WITH TUM	2755	2755	Progression of Disease			1921		1	2755	1	2755			1	1921
TCGA-02-	TCGA-02-	GBM	54	MALE	WHITE			Untreated primary (c		2003	-20070	Dead	WITH TUM	86	86	Progression of Disease			32		1	86	1	86			1	32
TCGA-02-	TCGA-02-	GBM	60	MALE	WHITE			Untreated primary (c		2003	-22166	Dead	WITH TUM	430	430	Progression of Disease			386		1	430	1	430			1	386
TCGA-02-	TCGA-02-	GBM	74	FEMALE	WHITE			Untreated primary (c		2004	-27063	Dead	WITH TUM	110	110	Progression of Disease			37		1	110	1	110			1	37
TCGA-02-	TCGA-02-	GBM	48	FEMALE	WHITE			Untreated primary (c		2004	-17749	Dead	WITH TUM	326	326	Progression of Disease			239		1	326	1	326			1	239
TCGA-02-	TCGA-02-	GBM	54	MALE	WHITE			Untreated primary (c		2005	-20065	Dead	WITH TUM	320	320	Progression of Disease			102		1	320	1	320			1	102
TCGA-02-	TCGA-02-	GBM	54	FEMALE	WHITE			Treated primary GBM		2004	-19882	Dead	WITH TUM	557	557	Progression of Disease			282		1	557	1	557			1	282
TCGA-02-	TCGA-02-	GBM	61	MALE	BLACK OR AFRICAN AMERICAN			Untreated primary (c		2004	-22417	Dead	WITH TUM	209	209	Progression of Disease			195		1	209	1	209			1	195
TCGA-02-	TCGA-02-	GBM	78	MALE	WHITE			Untreated primary (c		2005	-28759	Dead	WITH TUM	448	448	Progression of Disease			57		1	448	1	448			1	57
TCGA-02-	TCGA-02-	GBM	80	MALE				Untreated primary (c		2005	-29299	Dead		98	98	Progression of Disease			23		1	98		98			1	23
TCGA-02-	TCGA-02-	GBM	43	MALE	WHITE			Untreated primary (c		2005	-15983	Dead	WITH TUM	459	459	Progression of Disease			89		1	459	1	459			1	89
TCGA-02-	TCGA-02-	GBM	49	MALE	WHITE			Untreated primary (c		2005	-18061	Dead	WITH TUM	383	383	Progression of Disease			204		1	383	1	383			1	204
TCGA-02-	TCGA-02-	GBM	44	FEMALE	WHITE			Untreated primary (c		2005	-16224	Dead	WITH TUM	199	199	Progression of Disease			72		1	199	1	199			1	72
TCGA-02-	TCGA-02-	GBM	62	FEMALE	WHITE			Untreated primary (c		2005	-22798	Dead	WITH TUM	76	76	Progression of Disease			6		1	76	1	76			1	6
TCGA-02-	TCGA-02-	GBM	66	FEMALE	WHITE			Treated primary GBM		2004	-24140	Dead	WITH TUM	604	604	Progression of Disease			473		1	604	1	604			1	473
TCGA-02-	TCGA-02-	GBM	28	FEMALE	WHITE			Treated primary GBM		2005	-10517	Dead	WITH TUM	254	254	Progression of Disease			172		1	254	1	254			1	172
TCGA-02-	TCGA-02-	GBM	68	FEMALE	WHITE			Untreated primary (c		2005	-25097	Dead	WITH TUM	291	291	Recurrence			21		1	291	1	291			1	21
TCGA-02-	TCGA-02-	GBM	66	FEMALE	ASIAN			Untreated primary (c		2006	-24151	Dead	WITH TUM	183	183	Progression of Disease			183		1	183	1	183			1	183
TCGA-02-	TCGA-02-	GBM	50	MALE	WHITE			Untreated primary (c		2005	-18280	Dead	WITH TUM	600	600	Recurrence			496		1	600	1	600			1	496
TCGA-02-	TCGA-02-	GBM	57	MALE	WHITE			Untreated primary (c		2006	-21160	Dead	WITH TUM	804	804	Recurrence			175		1	804	1	804			1	175
TCGA-02-	TCGA-02-	GBM	31	FEMALE	ASIAN			Untreated primary (c		2006	-11668	Alive	WITH TUM	873		Progression of Disease			873		0	873	0	873			1	873
TCGA-02-	TCGA-02-	GBM	70	MALE	WHITE			Untreated primary (c		2006	-25916	Alive	WITH TUM	762		Recurrence			762		0	762	0	762			1	762
TCGA-02-	TCGA-02-	GBM	53	MALE	WHITE			Untreated primary (c		2005	-19425	Dead	WITH TUM	167	167	Progression of Disease			8		1	167	1	167			1	8
TCGA-02-	TCGA-02-	GBM	68	FEMALE	WHITE			Untreated primary (c		2005	-24906	Dead	WITH TUM	310	310	Progression of Disease			154		1	310	1	310			1	154
TCGA-02-	TCGA-02-	GBM	63	MALE	WHITE			Untreated primary (c		2005	-23205	Dead	WITH TUM	634	634	Progression of Disease			336		1	634	1	634			1	336
TCGA-02-	TCGA-02-	GBM	57	MALE	WHITE			Untreated primary (c		2005	-21173	Dead	WITH TUM	829	829	Progression of Disease			798		1	829	1	829			1	798
TCGA-02-	TCGA-02-	GBM	28	MALE	WHITE			Untreated primary (c		1999	-10310	Dead	WITH TUM	2729	2729	Progression of Disease			2339		1	2729	1	2729			1	2339
TCGA-02-	TCGA-02-	GBM	59	FEMALE	WHITE			Treated primary GBM		2005	-21626	Dead	WITH TUM	691	691	Progression of Disease			463		1	691	1	691			1	463
TCGA-02-	TCGA-02-	GBM	36	FEMALE	WHITE			Untreated primary (c		2005	-13263	Dead		7	384						1	384		384			0	384
TCGA-02-	TCGA-02-	GBM	63	FEMALE	WHITE			Untreated primary (c		2003	-23289	Dead	WITH TUM	1561	1561	Progression of Disease			976		1	1561	1	1561			1	976
TCGA-02-	TCGA-02-	GBM	45	FEMALE	WHITE			Untreated primary (c		2005	-16763	Dead	WITH TUM	268	268	Progression of Disease			97		1	268	1	268			1	97
TCGA-02-	TCGA-02-	GBM	27	FEMALE	WHITE			Untreated primary (c		2004	-10185	Alive		1757		Recurrence			1757		0	1757	0	1757			1	1757
TCGA-02-	TCGA-02-	GBM	52	MALE	WHITE			Treated primary GBM		2005	-19233	Dead	WITH TUM	516	516	Progression of Disease			358		1	516	1	516			1	358
TCGA-02-	TCGA-02-	GBM	46	MALE	WHITE			Untreated primary (c		2004	-17080	Dead	WITH TUM	106	106						1	106	1	106			1	106
TCGA-02-	TCGA-02-	GBM	42	MALE	WHITE			Treated primary GBM		2004	-15660	Dead	WITH TUM	822	822	Progression of Disease			450		1	822	1	822			1	450
TCGA-02-	TCGA-02-	GBM	29	FEMALE	WHITE			Untreated primary (c		2001	-10699	Dead	WITH TUM	1977	1977	Recurrence			1457		1	1977	1	1977			1	1457
TCGA-02-	TCGA-02-	GBM	54	MALE	WHITE			Untreated primary (c		2004	-19883	Dead	WITH TUM	355	355	Recurrence			196		1	355	1	355			1	196
TCGA-02-	TCGA-02-	GBM	56	MALE	WHITE			Treated primary GBM		2004	-20557	Dead	WITH TUM	537	537	Recurrence			146		1	537	1	537			1	146
TCGA-02-	TCGA-02-	GBM	56	MALE	ASIAN			Untreated primary (c		2004	-20813	Dead	WITH TUM	705	705	Progression of Disease			74		1	705	1	705			1	74
TCGA-02-	TCGA-02-	GBM	43	FEMALE	WHITE			Treated primary GBM		2001	-15836	Alive	WITH TUM	2818		Recurrence			1560		0	2818	0	2818			1	1560
TCGA-02-	TCGA-02-	GBM	37	FEMALE	BLACK OR AFRICAN AMERICAN			Treated primary GBM		1998	-13681	Dead	WITH TUM	3041	3041	Recurrence			2296		1	3041	1	3041			1	2296
TCGA-02-	TCGA-02-	GBM	52	MALE	ASIAN			Treated primary GBM		2006	-19257	Dead	WITH TUM	476	476	Progression of Disease			91		1	476	1	476			1	91
TCGA-02-	TCGA-02-	GBM	51	MALE	WHITE			Treated primary GBM		2003	-18657	Dead	WITH TUM	1489	1489	Progression of Disease			1231		1	1489	1	1489			1	1231
TCGA-02-	TCGA-02-	GBM	36	FEMALE	WHITE			Untreated primary (c		1989	-13269	Dead	WITH TUM	503	503	Recurrence			503		1	503	1	503			1	503
TCGA-02-	TCGA-02-	GBM	54	MALE	WHITE			Untreated primary (c		1989	-19956	Dead	WITH TUM	515	515						1	515	1	515			1	515
TCGA-02-	TCGA-02-	GBM	14	MALE				Untreated primary (c		1990	-5303	Dead	WITH TUM	539	539	Progression of Disease			293		1	539	1	539			1	293
TCGA-02-	TCGA-02-	GBM	68	MALE	WHITE			Untreated primary (c		1990	-25194	Dead	WITH TUM	327	327	Progression of Disease			99		1	327	1	327			1	99
TCGA-02-	TCGA-02-	GBM	26	MALE				Untreated primary (c		1990	-9578	Dead		440	440						1	440		440			0	440
TCGA-02-	TCGA-02-	GBM	78	FEMALE	WHITE			Untreated primary (c		1991	-28696	Dead	WITH TUM	121	121						1	121	1	121			1	121
TCGA-02-	TCGA-02-	GBM	50	FEMALE	WHITE			Untreated primary (c		1991	-18353	Dead	WITH TUM	422	422						1	422	1	422			1	422
TCGA-02-	TCGA-02-	GBM	57	MALE	WHITE			Untreated primary (c		1992	-21031	Dead	WITH TUM	432	432	Progression of Disease			245		1	432	1	432			1	245
TCGA-02-	TCGA-02-	GBM	49	MALE	WHITE			Untreated primary (c		1992	-18066	Dead	WITH TUM	485	485	Progression of Disease			374		1	485	1	485			1	374
TCGA-02-	TCGA-02-	GBM	40	MALE	WHITE			Untreated primary (c		1994	-14636	Dead	WITH TUM	372	372	Progression of Disease			238		1	372	1	372			1	238
TCGA-02-	TCGA-02-	GBM	74	MALE	WHITE			Untreated primary (c		1995	-27125	Dead	WITH TUM	301	301	Progression of Disease			73		1	301	1	301			1	73
TCGA-02-	TCGA-02-	GBM	69	FEMALE				Untreated primary (c		1995	-25352	Dead	WITH TUM	235	235	Progression of Disease			164		1	235	1	235			1	164
TCGA-02-	TCGA-02-	GBM	61	MALE	WHITE			Untreated primary (c		1996	-22598	Dead	WITH TUM	323	323	Progression of Disease			284		1	323	1	323			1	284
TCGA-02-	TCGA-02-	GBM	82	FEMALE	WHITE			Untreated primary (c		1995	-30160	Dead	WITH TUM	5	223	Progression of Disease			5		1	223	1	223			1	5
TCGA-02-	TCGA-02-	GBM	51	FEMALE	BLACK OR AFRICAN AMERICAN			Untreated primary (c		1995	-18654	Dead	WITH TUM	484	484	Progression of Disease			120		1	484	1	484			1	120
TCGA-02-	TCGA-02-	GBM	46	FEMALE	WHITE			Untreated primary (c		1995	-17021	Dead	WITH TUM	782	782	Progression of Disease			438		1	782	1	782			1	438
TCGA-02-	TCGA-02-	GBM	77	FEMALE	WHITE			Untreated primary (c		1996	-28449	Dead	WITH TUM	133	133	Progression of Disease			4		1	133	1	133			1	4
TCGA-02-	TCGA-02-	GBM	48	MALE	WHITE			Untreated primary (c		1996	-17643	Dead	WITH TUM	764	764	Progression of Disease			691		1	764	1	764			1	691
TCGA-02-	TCGA-02-	GBM	41	MALE	WHITE			Untreated primary (c		1996	-15231	Dead	WITH TUM	322	322	Progression of Disease			168		1	322	1	322			1	168
TCGA-02-	TCGA-02-	GBM	67	MALE	WHITE			Untreated primary (c		1995	-24524	Dead	WITH TUM	377	377	Progression of Disease			148		1	377	1	377			1	148
TCGA-02-	TCGA-02-	GBM	50	MALE	WHITE			Untreated primary (c		1996	-18368	Dead	WITH TUM	441	441	Progression of Disease			125		1	441	1	441			1	125
TCGA-02-	TCGA-02-	GBM	67	FEMALE	WHITE			Untreated primary (c		1997	-24641	Dead	WITH TUM	321	321						1	321	1	321			1	321
TCGA-02-	TCGA-02-	GBM	36	MALE	WHITE			Untreated primary (c		1997	-13260	Dead	WITH TUM	1433	1433	Progression of Disease			1053		1	1433	1	1433			1	1053
TCGA-02-	TCGA-02-	GBM	62	MALE	WHITE			Untreated primary (c		1997	-22955	Dead	WITH TUM	345	345	Progression of Disease			212		1	345	1	345			1	212
TCGA-02-	TCGA-02-	GBM	61	MALE	WHITE			Untreated primary (c		1997	-22541	Dead	WITH TUM	282	282	Recurrence			15		1	282	1	282			1	15
TCGA-02-	TCGA-02-	GBM	62	FEMALE	WHITE			Untreated primary (c		1998	-22662	Dead	WITH TUM	493	493	Recurrence			428		1	493	1	493			1	428
TCGA-02-	TCGA-02-	GBM	67	FEMALE	WHITE			Untreated primary (c		1998	-24796	Dead	WITH TUM	102	102	Recurrence			12		1	102	1	102			1	12
TCGA-02-	TCGA-02-	GBM	61	MALE	WHITE			Treated primary GBM		2007	-22457	Dead	WITH TUM	511	511	Progression of Disease			128		1	511	1	511			1	128
TCGA-02-	TCGA-02-	GBM	57	MALE	WHITE			Treated primary GBM		2007	-21021	Dead	WITH TUM	393	393	Progression of Disease			183		1	393	1	393			1	183
TCGA-02-	TCGA-02-	GBM	43	MALE	ASIAN			Untreated primary (c		2008	-15964	Alive	WITH TUM	466							0	466	0	466			0	466
TCGA-02-	TCGA-02-	GBM	53	MALE	BLACK OR AFRICAN AMERICAN			Untreated primary (c		2009	-19494	Alive	WITH TUM	470		Progression of Disease			186		0	470	0	470			1	186
TCGA-02-	TCGA-02-	GBM	64	MALE	WHITE			Untreated primary (c		2008	-23394	Dead	WITH TUMOR		618						1	618	1	618			1	618
TCGA-06-	TCGA-06-	GBM	81	FEMALE	WHITE			Glioblastoma Multifo		2005	-29606	Dead	WITH TUMOR		82						1	82	1	82			1	82
TCGA-06-	TCGA-06-	GBM	84	FEMALE	WHITE			Untreated primary (c		2006	-30968	Dead		8	187						1	187		187			0	187
TCGA-06-	TCGA-06-	GBM	67	MALE	WHITE			Untreated primary (c		1998	-24591	Dead	WITH TUM	123	620						1	620	1	620			1	620
TCGA-06-	TCGA-06-	GBM	63	FEMALE	WHITE			Untreated primary (c		2001	-23343	Dead	WITH TUM	1439	1448	Progression of Disease			797		1	1448	1	1448			1	797
TCGA-06-	TCGA-06-	GBM	63	FEMALE	WHITE			Untreated primary (c		2001	-23343	Dead	WITH TUM	1439	1448	Progression of Disease			797		1	1448	1	1448			1	797
TCGA-06-	TCGA-06-	GBM	86	MALE	WHITE			Untreated primary (c		2001	-31627	Dead		3	211					[Unknown]	1	211		211			0	211
TCGA-06-	TCGA-06-	GBM	67	MALE	WHITE			Untreated primary (c		2002	-24502	Dead	WITH TUM	109	121	Progression of Disease			91		1	121	1	121			1	91
TCGA-06-	TCGA-06-	GBM	66	MALE	WHITE			Untreated primary (c		2000	-24217	Dead	WITH TUM	691	691	Progression of Disease			190		1	691	1	691			1	190
TCGA-06-	TCGA-06-	GBM	30	MALE	ASIAN			Untreated primary (c		2002	-11284	Dead	WITH TUM	989	1024	Progression of Disease			148		1	1024	1	1024			1	148
TCGA-06-	TCGA-06-	GBM	54	MALE	WHITE			Untreated primary (c		2003	-19812	Dead	WITH TUM	320	394	Progression of Disease			244		1	394	1	394			1	244
TCGA-06-	TCGA-06-	GBM	49	MALE	WHITE			Untreated primary (c		2007	-18125	Dead	WITH TUM	570	771	Progression of Disease			482		1	771	1	771			1	482
TCGA-06-	TCGA-06-	GBM	64	MALE	WHITE			Untreated primary (c		2007	-23402	Dead	WITH TUM	428	435	Progression of Disease			78		1	435	1	435			1	78
TCGA-06-	TCGA-06-	GBM	63	FEMALE	WHITE			Untreated primary (c		2003	-23273	Dead	WITH TUM	702	812	Progression of Disease			487		1	812	1	812			1	487
TCGA-06-	TCGA-06-	GBM	43	MALE	WHITE			Untreated primary (c		2004	-15736	Dead	WITH TUM	674	737	Progression of Disease			394		1	737	1	737			1	394
TCGA-06-	TCGA-06-	GBM	40	MALE	WHITE			Untreated primary (c		2006	-14729	Dead	WITH TUM	327	362	Progression of Disease			152		1	362	1	362			1	152
TCGA-06-	TCGA-06-	GBM	86	MALE	WHITE			Glioblastoma Multifo		2003	-31566	Dead	WITH TUMOR		6						1	6	1	6			1	6
TCGA-06-	TCGA-06-	GBM	62	MALE	WHITE			Untreated primary (c		2000	-22926	Dead	WITH TUM	280	313	Progression of Disease			145		1	313	1	313			1	145
TCGA-06-	TCGA-06-	GBM	81	MALE	WHITE			Glioblastoma Multifo		2002	-29642	Dead	WITH TUMOR		67						1	67	1	67			1	67
TCGA-06-	TCGA-06-	GBM	58	MALE	WHITE			Untreated primary (c		2006	-21386	Dead	WITH TUM	357	357	Recurrence			265		1	357	1	357			1	265
TCGA-06-	TCGA-06-	GBM	53	FEMALE	WHITE			Untreated primary (c		2002	-19661	Dead	WITH TUM	71	71						1	71	1	71			1	71
TCGA-06-	TCGA-06-	GBM	33	FEMALE	WHITE			Untreated primary (c		1995	-12253	Dead	WITH TUM	611	611	Progression of Disease			530		1	611	1	611			1	530
TCGA-06-	TCGA-06-	GBM	51	FEMALE	WHITE			Untreated primary (c		1999	-18743	Dead	WITH TUM	508	541	Progression of Disease			92		1	541	1	541			1	92
TCGA-06-	TCGA-06-	GBM	76	MALE	BLACK OR AFRICAN AMERICAN			Untreated primary (c		2002	-27842	Dead	WITH TUM	298	307	Progression of Disease			189		1	307	1	307			1	189
TCGA-06-	TCGA-06-	GBM	74	FEMALE	WHITE			Untreated primary (c		2005	-27315	Dead	WITH TUM	239	262	Progression of Disease			204		1	262	1	262			1	204
TCGA-06-	TCGA-06-	GBM	45	MALE	WHITE			Glioblastoma Multifo		1995	-16442	Dead	WITH TUMOR		592						1	592	1	592			1	592
TCGA-06-	TCGA-06-	GBM	32	FEMALE	WHITE			Glioblastoma Multifo		1995	-11956	Dead	WITH TUMOR		1417						1	1417	1	1417			1	1417
TCGA-06-	TCGA-06-	GBM	68	MALE	WHITE			Untreated primary (c		1995	-24844	Dead	WITH TUM	359	375	Progression of Disease			299		1	375	1	375			1	299
TCGA-06-	TCGA-06-	GBM	68	MALE	WHITE			Untreated primary (c		1995	-24844	Dead	WITH TUM	359	375	Progression of Disease			299		1	375	1	375			1	299
TCGA-06-	TCGA-06-	GBM	54	MALE	WHITE			Untreated primary (c		1998	-20018	Dead	WITH TUM	389	424	Progression of Disease			208		1	424	1	424			1	208
TCGA-06-	TCGA-06-	GBM	61	MALE	WHITE			Untreated primary (c		1998	-22498	Dead	WITH TUM	263	318	Progression of Disease			256		1	318	1	318			1	256
TCGA-06-	TCGA-06-	GBM	57	MALE	WHITE			Untreated primary (c		1998	-20932	Dead	WITH TUM	164	178						1	178	1	178			1	178
TCGA-06-	TCGA-06-	GBM	63	FEMALE	WHITE			Untreated primary (c		1998	-23128	Dead	WITH TUM	97	97						1	97	1	97			1	97
TCGA-06-	TCGA-06-	GBM	73	MALE	WHITE			Untreated primary (c		1998	-26856	Dead	WITH TUM	166	329	Progression of Disease			90		1	329	1	329			1	90
TCGA-06-	TCGA-06-	GBM	74	MALE	WHITE			Glioblastoma Multifo		1998	-27368	Dead	WITH TUMOR		287					[Unknown]	1	287	1	287			1	287
TCGA-06-	TCGA-06-	GBM	56	FEMALE	WHITE			Glioblastoma Multifo		1999	-20684	Dead	WITH TUMOR		359					[Discrepancy]	1	359	1	359			1	359
TCGA-06-	TCGA-06-	GBM	47	FEMALE	WHITE			Untreated primary (c		2000	-17272	Dead	WITH TUM	78	104						1	104	1	104			1	104
TCGA-06-	TCGA-06-	GBM	47	MALE	ASIAN			Untreated primary (c		2000	-17510	Dead	WITH TUM	1730	1731	Progression of Disease			1428		1	1731	1	1731			1	1428
TCGA-06-	TCGA-06-	GBM	52	MALE	WHITE			Glioblastoma Multifo		2001	-19198	Dead	WITH TUMOR		324					[Unknown]	1	324	1	324			1	324
TCGA-06-	TCGA-06-	GBM	51	MALE	WHITE			Untreated primary (c		2001	-18902	Dead	WITH TUM	161	178	Progression of Disease			66		1	178	1	178			1	66
TCGA-06-	TCGA-06-	GBM	44	MALE	BLACK OR AFRICAN AMERICAN			Glioblastoma Multifo		2001	-16170	Dead	WITH TUMOR		347					[Unknown]	1	347	1	347			1	347
TCGA-06-	TCGA-06-	GBM	59	FEMALE	WHITE			Untreated primary (c		2002	-21776	Dead	WITH TUM	579	598	Progression of Disease			461		1	598	1	598			1	461
TCGA-06-	TCGA-06-	GBM	68	MALE	WHITE			Untreated primary (c		2002	-25127	Dead	WITH TUM	95	100	Progression of Disease			92		1	100	1	100			1	92
TCGA-06-	TCGA-06-	GBM	65	MALE	WHITE			Untreated primary (c		2002	-24085	Dead	WITH TUM	396	399	Progression of Disease			117		1	399	1	399			1	117
TCGA-06-	TCGA-06-	GBM	65	MALE	WHITE			Untreated primary (c		2002	-24085	Dead	WITH TUM	396	399	Progression of Disease			117		1	399	1	399			1	117
TCGA-06-	TCGA-06-	GBM	72	FEMALE	WHITE			Untreated primary (c		2003	-26549	Dead	WITH TUM	7	136						1	136	1	136			1	136
TCGA-06-	TCGA-06-	GBM	54	MALE	WHITE			Untreated primary (c		2003	-19824	Dead	WITH TUM	67	98	Progression of Disease			47		1	98	1	98			1	47
TCGA-06-	TCGA-06-	GBM	69	MALE	WHITE			Untreated primary (c		2003	-25559	Dead	WITH TUM	83	123	Progression of Disease			39		1	123	1	123			1	39
TCGA-06-	TCGA-06-	GBM	34	MALE	ASIAN			Untreated primary (c		2004	-12777	Alive	TUMOR F	2768		Progression of Disease			41		0	2768	0	2768			1	41
TCGA-06-	TCGA-06-	GBM	64	MALE	WHITE			Untreated primary (c		2004	-23498	Dead	WITH TUM	61	127						1	127	1	127			1	127
TCGA-06-	TCGA-06-	GBM	38	MALE	WHITE			Untreated primary (c		2004	-14235	Dead	WITH TUMOR		2681	Progression of Disease			192		1	2681	1	2681			1	192
TCGA-06-	TCGA-06-	GBM	64	MALE	WHITE			Untreated primary (c		2004	-23450	Dead	WITH TUM	578	616	Progression of Disease			250		1	616	1	616			1	250
TCGA-06-	TCGA-06-	GBM	76	MALE	WHITE			Untreated primary (c		2004	-27964	Dead	WITH TUM	77	111						1	111	1	111			1	111
TCGA-06-	TCGA-06-	GBM	63	MALE	WHITE			Untreated primary (c		2005	-23317	Dead	WITH TUMOR		2126				1276		1	2126	1	2126			1	1276
TCGA-06-	TCGA-06-	GBM	54	MALE	WHITE			Untreated primary (c		2005	-19923	Alive	WITH TUM	2246		Progression of Disease			711		0	2246	0	2246			1	711
TCGA-06-	TCGA-06-	GBM	69	MALE	WHITE			Untreated primary (c		2006	-25318	Dead	WITH TUM	801	828	Recurrence			531		1	828	1	828			1	531
TCGA-06-	TCGA-06-	GBM	71	MALE	WHITE			Untreated primary (c		2006	-26080	Dead	WITH TUMOR		1356				310		1	1356	1	1356			1	310
TCGA-06-	TCGA-06-	GBM	55	MALE	WHITE			Untreated primary (c		2006	-20297	Dead	WITH TUM	454	469						1	469	1	469			1	469
TCGA-06-	TCGA-06-	GBM	62	MALE	WHITE			Untreated primary (c		2006	-22835	Dead	WITH TUM	313	317	Progression of Disease			88		1	317	1	317			1	88
TCGA-06-	TCGA-06-	GBM	62	MALE	WHITE			Untreated primary (c		2006	-22835	Dead	WITH TUM	313	317	Progression of Disease			88		1	317	1	317			1	88
TCGA-06-	TCGA-06-	GBM	58	MALE	WHITE			Untreated primary (c		2007	-21243	Dead	WITH TUMOR		1185	Locoregional Disease			648		1	1185	1	1185			1	648
TCGA-06-	TCGA-06-	GBM	37	FEMALE	WHITE			Untreated primary (c		1994	-13852	Dead	WITH TUM	142	142	Progression of Disease			125		1	142	1	142			1	125
TCGA-06-	TCGA-06-	GBM	63	MALE	WHITE			Untreated primary (c		1994	-23132	Dead	WITH TUM	214	225	Progression of Disease			147		1	225	1	225			1	147
TCGA-06-	TCGA-06-	GBM	65	FEMALE	WHITE			Untreated primary (c		1994	-24095	Dead	WITH TUM	79	169	Progression of Disease			47		1	169	1	169			1	47
TCGA-06-	TCGA-06-	GBM	51	FEMALE	WHITE			Untreated primary (c		1995	-18649	Dead	WITH TUM	12	12						1	12	1	12			1	12
TCGA-06-	TCGA-06-	GBM	40	MALE	WHITE			Untreated primary (c		1996	-14937	Dead	WITH TUM	197	233	Recurrence			106		1	233	1	233			1	106
TCGA-06-	TCGA-06-	GBM	52	FEMALE	WHITE			Untreated primary (c		1997	-19108	Dead	WITH TUM	167	256	Progression of Disease			149		1	256	1	256			1	149
TCGA-06-	TCGA-06-	GBM	76	MALE	WHITE			Untreated primary (c		1997	-27877	Dead	WITH TUM	119	232						1	232	1	232			1	232
TCGA-06-	TCGA-06-	GBM	72	FEMALE	WHITE			Untreated primary (c		1997	-26600	Dead	WITH TUM	151	225	Progression of Disease			67		1	225	1	225			1	67
TCGA-06-	TCGA-06-	GBM	72	FEMALE	WHITE			Untreated primary (c		1997	-26600	Dead	WITH TUM	151	225	Progression of Disease			67		1	225	1	225			1	67
TCGA-06-	TCGA-06-	GBM	47	MALE	WHITE			Untreated primary (c		1997	-17515	Dead	WITH TUM	360	360	Progression of Disease			53		1	360	1	360			1	53
TCGA-06-	TCGA-06-	GBM	47	MALE	WHITE			Untreated primary (c		1997	-17515	Dead	WITH TUM	360	360	Progression of Disease			53		1	360	1	360			1	53
TCGA-06-	TCGA-06-	GBM	55	FEMALE	WHITE			Untreated primary (c		1998	-20134	Dead	WITH TUM	6	16						1	16	1	16			1	16
TCGA-06-	TCGA-06-	GBM	66	MALE	WHITE			Untreated primary (c		1998	-24187	Dead	WITH TUM	378	457	Progression of Disease			48		1	457	1	457			1	48
TCGA-06-	TCGA-06-	GBM	61	FEMALE	WHITE			Untreated primary (c		1999	-22524	Dead	WITH TUM	684	735	Progression of Disease			175		1	735	1	735			1	175
TCGA-06-	TCGA-06-	GBM	67	MALE	WHITE			Untreated primary (c		2000	-24488	Dead	WITH TUM	11	22						1	22	1	22			1	22
TCGA-06-	TCGA-06-	GBM	31	MALE	WHITE			Untreated primary (c		2000	-11333	Dead	WITH TUM	548	603	Progression of Disease			260		1	603	1	603			1	260
TCGA-06-	TCGA-06-	GBM	31	MALE	WHITE			Untreated primary (c		2000	-11333	Dead	WITH TUM	548	603	Progression of Disease			260		1	603	1	603			1	260
TCGA-06-	TCGA-06-	GBM	75	FEMALE	WHITE			Untreated primary (c		2006	-27735	Dead	WITH TUM	315	415						1	415	1	415			1	415
TCGA-06-	TCGA-06-	GBM	46	MALE	WHITE			Untreated primary (c		2007	-17037	Dead	WITH TUM	359	405	Progression of Disease			311		1	405	1	405			1	311
TCGA-06-	TCGA-06-	GBM	57	MALE	WHITE			Glioblastoma Multifo		2007	-20937	Dead	WITH TUMOR		621					[Unknown]	1	621	1	621			1	621
TCGA-06-	TCGA-06-	GBM	65	FEMALE	WHITE			Untreated primary (c		2007	-24101	Dead	WITH TUMOR		1481	Recurrence			196		1	1481	1	1481			1	196
TCGA-06-	TCGA-06-	GBM	51	MALE	BLACK OR AFRICAN AMERICAN			Untreated primary (c		1992	-18913	Dead	WITH TUM	314	329	Progression of Disease			87		1	329	1	329			1	87
TCGA-06-	TCGA-06-	GBM	57	FEMALE	WHITE			Untreated primary (c		1993	-20845	Dead	WITH TUM	168	274						1	274	1	274			1	274
TCGA-06-	TCGA-06-	GBM	71	MALE	WHITE			Untreated primary (c		1993	-26059	Dead	WITH TUM	8	8						1	8	1	8			1	8
TCGA-06-	TCGA-06-	GBM	43	MALE	BLACK OR AFRICAN AMERICAN			Untreated primary (c		1994	-16023	Dead	WITH TUM	2145	2201	Progression of Disease			334		1	2201	1	2201			1	334
TCGA-06-	TCGA-06-	GBM	76	FEMALE	WHITE			Untreated primary (c		1995	-28084	Dead	WITH TUM	143	143						1	143	1	143			1	143
TCGA-06-	TCGA-06-	GBM	56	FEMALE	WHITE			Untreated primary (c		1995	-20619	Dead	WITH TUM	245	291	Progression of Disease			130		1	291	1	291			1	130
TCGA-06-	TCGA-06-	GBM	77	FEMALE	WHITE			Untreated primary (c		1995	-28433	Dead	WITH TUM	5	96						1	96	1	96			1	96
TCGA-06-	TCGA-06-	GBM	63	MALE	WHITE			Untreated primary (c		1996	-23215	Dead	TUMOR F	1065	1068	Recurrence			1013		1	1068	0	1068			1	1013
TCGA-06-	TCGA-06-	GBM	71	MALE	BLACK OR AFRICAN AMERICAN			Untreated primary (c		2007	-26247	Dead	WITH TUMOR		384	Recurrence			85		1	384	1	384			1	85
TCGA-06-	TCGA-06-	GBM	55	FEMALE	WHITE			Untreated primary (c		2007	-20449	Dead	WITH TUM	98	175						1	175	1	175			1	175
TCGA-06-	TCGA-06-	GBM	60	MALE	WHITE			Untreated primary (c		2007	-22273	Dead	WITH TUM	136	175	Progression of Disease			90		1	175	1	175			1	90
TCGA-06-	TCGA-06-	GBM	77	MALE	WHITE			Untreated primary (c		2007	-28477	Dead	WITH TUM	294	298	Progression of Disease			202		1	298	1	298			1	202
TCGA-06-	TCGA-06-	GBM	73	FEMALE	BLACK OR AFRICAN AMERICAN			Untreated primary (c		2007	-26796	Dead	WITH TUM	62	64						1	64	1	64			1	64
TCGA-06-	TCGA-06-	GBM	39	FEMALE	WHITE			Untreated primary (c		2007	-14560	Dead	WITH TUM	717	717	Progression of Disease			351		1	717	1	717			1	351
TCGA-06-	TCGA-06-	GBM	53	MALE	WHITE			Untreated primary (c		2008	-19503	Dead	WITH TUMOR		432	Progression of Disease			160		1	432	1	432			1	160
TCGA-06-	TCGA-06-	GBM	69	MALE	WHITE			Untreated primary (c		2006	-25434	Dead	WITH TUMOR		803	Progression of Disease			176		1	803	1	803			1	176
TCGA-06-	TCGA-06-	GBM	66	MALE	WHITE			Untreated primary (c		2007	-24471	Dead	WITH TUMOR		1426				1277		1	1426	1	1426			1	1277
TCGA-06-	TCGA-06-	GBM	59	MALE	WHITE			Untreated primary (c		2007	-21884	Dead	WITH TUM	164	239	Progression of Disease			92		1	239	1	239			1	92
TCGA-06-	TCGA-06-	GBM	53	MALE	WHITE			Untreated primary (c		2007	-19657	Dead	WITH TUM	82	82						1	82	1	82			1	82
TCGA-06-	TCGA-06-	GBM	50	MALE	BLACK OR AFRICAN AMERICAN			Untreated primary (c		2008	-18571	Dead	WITH TUM	66	82						1	82	1	82			1	82
TCGA-06-	TCGA-06-	GBM	43	MALE	WHITE			Untreated primary (c		2008	-16018	Dead	WITH TUM	28	28						1	28	1	28			1	28
TCGA-06-	TCGA-06-	GBM	61	FEMALE	WHITE			Untreated primary (c		2008	-22312	Alive	TUMOR F	1322		Progression of Disease			279		0	1322	0	1322			1	279
TCGA-06-	TCGA-06-	GBM	72	FEMALE	BLACK OR AFRICAN AMERICAN			Untreated primary (c		2008	-26372	Alive	WITH TUM	1405					424		0	1405	0	1405			1	424
TCGA-06-	TCGA-06-	GBM	78	MALE	WHITE			Untreated primary (c		2008	-28760	Alive	WITH TUM	204							0	204	0	204			0	204
TCGA-06-	TCGA-06-	GBM	74	MALE	WHITE			Untreated primary (c		2008	-27319	Alive	WITH TUM	218		Recurrence			66		0	218	0	218			1	66
TCGA-06-	TCGA-06-	GBM	52	MALE	WHITE			Untreated primary (c		2008	-19154	Dead	WITH TUMOR		1229	Recurrence			699		1	1229	1	1229			1	699
TCGA-06-	TCGA-06-	GBM	50	MALE	WHITE			Untreated primary (c		2008	-18278	Dead	WITH TUMOR		504	Recurrence			141		1	504	1	504			1	141
TCGA-06-	TCGA-06-	GBM	30	MALE	WHITE			Untreated primary (c		2008	-11112	Dead	WITH TUMOR		632	Recurrence			213		1	632	1	632			1	213
TCGA-06-	TCGA-06-	GBM	79	FEMALE	WHITE			Untreated primary (c		2008	-28893	Dead	WITH TUMOR		814	Progression of Disease			329		1	814	1	814			1	329
TCGA-06-	TCGA-06-	GBM	54	MALE	BLACK OR AFRICAN AMERICAN			Untreated primary (c		2008	-19899	Dead	WITH TUMOR		728	Progression of Disease			208		1	728	1	728			1	208
TCGA-06-	TCGA-06-	GBM	42	MALE	BLACK OR AFRICAN AMERICAN			Untreated primary (c		2008	-15645	Dead	WITH TUM	193	208						1	208	1	208			1	208
TCGA-06-	TCGA-06-	GBM	75	MALE	WHITE			Untreated primary (c		2008	-27658	Dead	WITH TUM	109	123	Recurrence			109		1	123	1	123			1	109
TCGA-06-	TCGA-06-	GBM	61	MALE	WHITE			Untreated primary (c		2006	-22332	Dead	WITH TUM	815	815	Recurrence			750		1	815	1	815			1	750
TCGA-06-	TCGA-06-	GBM	56	FEMALE	WHITE			Untreated primary (c		2008	-20538	Dead	WITH TUMOR		360	Recurrence			232		1	360	1	360			1	232
TCGA-06-	TCGA-06-	GBM	61	MALE	WHITE			Untreated primary (c		2008	-22537	Dead	WITH TUMOR		466	Recurrence			81		1	466	1	466			1	81
TCGA-06-	TCGA-06-	GBM	81	FEMALE	WHITE			Untreated primary (c		2009	-29802	Dead	WITH TUM	414	414						1	414	1	414			1	414
TCGA-06-	TCGA-06-	GBM	28	FEMALE	WHITE			Untreated primary (c		2009	-10542	Alive	TUMOR F	1031							0	1031	0	1031			0	1031
TCGA-06-	TCGA-06-	GBM	47	MALE	WHITE			Glioblastoma Multifo		2009	-17296	Dead	WITH TUMOR		466	Progression of Disease			256	Progressive Disease	1	466	1	466			1	256
TCGA-06-	TCGA-06-	GBM	76	MALE	BLACK OR AFRICAN AMERICAN			Untreated primary (c		2008	-27980	Dead	WITH TUM	33	33						1	33	1	33			1	33
TCGA-06-	TCGA-06-	GBM	75	FEMALE	WHITE			Untreated primary (c		2008	-27511	Dead		23	380						1	380		380			0	380
TCGA-06-	TCGA-06-	GBM	83	MALE	WHITE			Untreated primary (c		2009	-30580	Dead	WITH TUM	150	150						1	150	1	150			1	150
TCGA-06-	TCGA-06-	GBM	53	FEMALE	WHITE			Untreated primary (c		2009	-19505	Dead	WITH TUMOR		537	Recurrence			78		1	537	1	537			1	78
TCGA-06-	TCGA-06-	GBM	81	MALE	WHITE			Untreated primary (c		2009	-29753	Dead	WITH TUMOR		382	Progression of Disease			151		1	382	1	382			1	151
TCGA-06-	TCGA-06-	GBM	72	FEMALE	WHITE			Untreated primary (c		2009	-26643	Alive	WITH TUM	932					554		0	932	0	932			1	554
TCGA-06-	TCGA-06-	GBM	50	MALE	WHITE			Untreated primary (c		2009	-18435	Alive	WITH TUM	181							0	181	0	181			0	181
TCGA-06-	TCGA-06-	GBM	59	MALE	ASIAN			Untreated primary (c		2009	-21559	Dead	WITH TUMOR		506	Progression of Disease			178		1	506	1	506			1	178
TCGA-06-	TCGA-06-	GBM	23	FEMALE	WHITE			Untreated primary (c		2009	-8428	Dead	WITH TUM	182	182						1	182	1	182			1	182
TCGA-06-	TCGA-06-	GBM	65	MALE	WHITE			Untreated primary (c		2009	-23759	Dead	WITH TUM	133	133						1	133	1	133			1	133
TCGA-06-	TCGA-06-	GBM	24	FEMALE	BLACK OR AFRICAN AMERICAN			Untreated primary (c		2009	-8922	Alive	WITH TUM	13							0	13	0	13			0	13
TCGA-06-	TCGA-06-	GBM	21	FEMALE	WHITE			Untreated primary (c		2009	-7936	Alive	TUMOR F	958							0	958	0	958			0	958
TCGA-06-	TCGA-06-	GBM	54	FEMALE	WHITE			Untreated primary (c		2009	-20063	Dead	WITH TUM	342	357	Progression of Disease			158		1	357	1	357			1	158
TCGA-06-	TCGA-06-	GBM	72	FEMALE	WHITE			Untreated primary (c		2010	-26395	Dead	WITH TUM	108	108						1	108	1	108			1	108
TCGA-06-	TCGA-06-	GBM	51	MALE	WHITE			Untreated primary (c		2010	-18832	Dead	WITH TUM	254	254	Progression of Disease			214		1	254	1	254			1	214
TCGA-06-	TCGA-06-	GBM	78	FEMALE	WHITE			Untreated primary (c		2010	-28762	Dead	WITH TUM	138	138	Progression of Disease			88		1	138	1	138			1	88
TCGA-06-	TCGA-06-	GBM	67	MALE	WHITE			Untreated primary (c		2010	-24477	Alive	WITH TUM	268		Progression of Disease			195		0	268	0	268			1	195
TCGA-06-	TCGA-06-	GBM	61	MALE	WHITE			Untreated primary (c		2010	-22527	Alive	WITH TUM	273		Progression of Disease			167		0	273	0	273			1	167
TCGA-06-	TCGA-06-	GBM	60	MALE	WHITE			Untreated primary (c		2010	-22213	Alive	WITH TUM	260							0	260	0	260			0	260
TCGA-06-	TCGA-06-	GBM	23	FEMALE	WHITE			Untreated primary (c		2010	-8533	Alive	WITH TUM	204							0	204	0	204			0	204
TCGA-06-	TCGA-06-	GBM	45	FEMALE	WHITE			Untreated primary (c		2010	-16572	Alive	WITH TUM	155							0	155	0	155			0	155
TCGA-06-	TCGA-06-	GBM	75	FEMALE	WHITE			Untreated primary (c		2010	-27584	Dead	WITH TUM	83	83						1	83	1	83			1	83
TCGA-06-	TCGA-06-	GBM	58	MALE	WHITE			Untreated primary (c		2010	-21232	Dead	WITH TUM	114	114						1	114	1	114			1	114
TCGA-06-	TCGA-06-	GBM	45	FEMALE	WHITE			Untreated primary (c		2010	-16662	Alive	WITH TUM	187		Progression of Disease			97		0	187	0	187			1	97
TCGA-06-	TCGA-06-	GBM	63	MALE	WHITE			Untreated primary (c		2011	-23211	Alive	WITH TUM	139							0	139	0	139			0	139
TCGA-06-	TCGA-06-	GBM	64	FEMALE	WHITE			Untreated primary (c		2011	-23519	Dead	WITH TUM	120	159	Progression of Disease			85		1	159	1	159			1	85
TCGA-06-	TCGA-06-	GBM	49	FEMALE	WHITE			Untreated primary (c		2011	-18248	Alive	WITH TUM	237							0	237	0	237			0	237
TCGA-06-	TCGA-06-	GBM	58	MALE	WHITE			Untreated primary (c		2011	-21328	Dead	WITH TUM	164	164						1	164	1	164			1	164
TCGA-06-	TCGA-06-	GBM	44	FEMALE	WHITE			Untreated primary (c		2011	-16206	Dead	WITH TUMOR		45						1	45	1	45			1	45
TCGA-06-	TCGA-06-	GBM	64	FEMALE	WHITE			Untreated primary (c		1993	-23585	Dead	WITH TUM	15	3667						1	3667	1	3667			1	3667
TCGA-06-	TCGA-06-	GBM	76	FEMALE	WHITE			Untreated primary (c		1994	-27909	Dead	WITH TUM	224	224						1	224	1	224			1	224
TCGA-06-	TCGA-06-	GBM	64	MALE	WHITE			Untreated primary (c		2010	-23426	Alive	WITH TUM	253		Progression of Disease			133		0	253	0	253			1	133
TCGA-06-	TCGA-06-	GBM	65	MALE	WHITE			Untreated primary (c		2010	-23763	Alive	WITH TUM	391		Progression of Disease			306		0	391	0	391			1	306
TCGA-06-	TCGA-06-	GBM	53	FEMALE	WHITE			Untreated primary (c		2011	-19591	Alive	WITH TUM	145							0	145	0	145			0	145
TCGA-06-	TCGA-06-	GBM	58	FEMALE	WHITE			Untreated primary (c		2011	-21190	Alive	WITH TUM	47							0	47	0	47			0	47
TCGA-06-	TCGA-06-	GBM	76	MALE	WHITE			Untreated primary (c		2011	-27850	Alive	WITH TUM	145							0	145	0	145			0	145
TCGA-06-	TCGA-06-	GBM	60	MALE	WHITE			Untreated primary (c		2011	-22051	Alive	WITH TUM	151							0	151	0	151			0	151
TCGA-06-	TCGA-06-	GBM	21	FEMALE	BLACK OR AFRICAN AMERICAN			Glioblastoma Multifo		2012	-7958	Alive	WITH TUM	815		Progression of Disease			100	Progressive Disease	0	815	0	815			1	100
TCGA-06-	TCGA-06-	GBM	78	FEMALE	BLACK OR AFRICAN AMERICAN			Glioblastoma Multifo		2011	-28545	Dead	WITH TUMOR		57					Progressive Disease	1	57	1	57			1	57
TCGA-06-	TCGA-06-	GBM	79	MALE	BLACK OR AFRICAN AMERICAN			Glioblastoma Multifo		2011	-29037	Dead	WITH TUMOR		95					Stable Disease	1	95	1	95			1	95
TCGA-06-	TCGA-06-	GBM	53	FEMALE	BLACK OR AFRICAN AMERICAN			Glioblastoma Multifo		2012	-19443	Dead	WITH TUMOR		78	Progression of Disease			46	Progressive Disease	1	78	1	78			1	46
TCGA-06-	TCGA-06-	GBM	64	MALE	BLACK OR AFRICAN AMERICAN			Glioblastoma Multifo		2013	-23674	Dead	WITH TUMOR		444	Progression of Disease			342	Progressive Disease	1	444	1	444			1	342
TCGA-06-	TCGA-06-	GBM	30	FEMALE	BLACK OR AFRICAN AMERICAN			Glioblastoma Multifo		2013	-11297	Alive	WITH TUM	539		Progression of Disease			188	Stable Disease	0	539	0	539			1	188
TCGA-08-	TCGA-08-	GBM	62	MALE	WHITE			Untreated primary (c		2001	-22684	Dead	WITH TUM	690	690	Recurrence			484		1	690	1	690			1	484
TCGA-08-	TCGA-08-	GBM	31	FEMALE	BLACK OR AFRICAN AMERICAN			Untreated primary (c		2001	-11613	Dead	WITH TUM	1057	1151	Progression of Disease			463		1	1151	1	1151			1	463
TCGA-08-	TCGA-08-	GBM	57	FEMALE	WHITE			Untreated primary (c		2001	-20974	Dead	WITH TUM	119	127	Progression of Disease			103		1	127	1	127			1	103
TCGA-08-	TCGA-08-	GBM	66	MALE	WHITE			Untreated primary (c		1992	-24253	Dead	WITH TUM	3310	3524	Recurrence			1880		1	3524	1	3524			1	1880
TCGA-08-	TCGA-08-	GBM	71	FEMALE				Untreated primary (c		1992	-25960	Dead	WITH TUM	53	53						1	53	1	53			1	53
TCGA-08-	TCGA-08-	GBM	69	MALE				Untreated primary (c		1993	-25409	Dead	WITH TUM	132	256	Progression of Disease			75		1	256	1	256			1	75
TCGA-08-	TCGA-08-	GBM	50	MALE				Untreated primary (c		1993	-18384	Dead	WITH TUM	419	782	Progression of Disease			59		1	782	1	782			1	59
TCGA-08-	TCGA-08-	GBM	63	MALE	WHITE			Untreated primary (c		2000	-23373	Dead	WITH TUM	316	370	Recurrence			198		1	370	1	370			1	198
TCGA-08-	TCGA-08-	GBM	46	MALE				Untreated primary (c		2000	-16964	Dead	WITH TUM	231	298	Progression of Disease			93		1	298	1	298			1	93
TCGA-08-	TCGA-08-	GBM	32	MALE	WHITE			Untreated primary (c		2001	-11905	Dead	WITH TUM	104	889	Recurrence			439		1	889	1	889			1	439
TCGA-08-	TCGA-08-	GBM	40	MALE	WHITE			Untreated primary (c		2001	-14838	Dead	WITH TUM	1637	1987	Recurrence			1542		1	1987	1	1987			1	1542
TCGA-08-	TCGA-08-	GBM	79	MALE	WHITE			Untreated primary (c		2003	-29106	Dead	WITH TUM	39	39						1	39	1	39			1	39
TCGA-08-	TCGA-08-	GBM	58	MALE	WHITE			Untreated primary (c		2003	-21332	Dead	WITH TUM	195	256	Progression of Disease			164		1	256	1	256			1	164
TCGA-08-	TCGA-08-	GBM	52	FEMALE	WHITE			Untreated primary (c		2003	-19199	Dead	WITH TUM	458	546	Progression of Disease			253		1	546	1	546			1	253
TCGA-08-	TCGA-08-	GBM	30	FEMALE	WHITE			Untreated primary (c		2003	-11099	Dead	WITH TUM	685	747	Recurrence			519		1	747	1	747			1	519
TCGA-08-	TCGA-08-	GBM	59	FEMALE	WHITE			Untreated primary (c		2004	-21900	Dead	WITH TUM	447	946	Recurrence			447		1	946	1	946			1	447
TCGA-08-	TCGA-08-	GBM	49	MALE	WHITE			Untreated primary (c		2004	-18144	Dead	WITH TUM	1084	1143	Recurrence			155		1	1143	1	1143			1	155

TCGA-08-	TCGA-08-	GBM	50	MALE	WHITE	Untreated primary (c	2005	-18383	Dead	WITH TU	594	678	Recurrence	264		1	678	1	678			1	264
TCGA-08-	TCGA-08-	GBM	59	FEMALE	WHITE	Untreated primary (c	2005	-21748	Dead	WITH TU	29	103				1	103	1	103			1	103
TCGA-08-	TCGA-08-	GBM	51	FEMALE		Untreated primary (c	1994	-18941	Dead	WITH TU	279	372	Recurrence	161		1	372	1	372			1	161
TCGA-08-	TCGA-08-	GBM	74	FEMALE		Untreated primary (c	1997	-27277	Dead	WITH TU	330	454	Progression of Disease	151		1	454	1	454			1	151
TCGA-08-	TCGA-08-	GBM	74	MALE	WHITE	Untreated primary (c	2005	-27053	Dead	WITH TU	478	548	Recurrence	427		1	548	1	548			1	427
TCGA-08-	TCGA-08-	GBM	59	MALE		Untreated primary (c	2005	-21638	Dead	WITH TU	97	467				1	467	1	467			1	467
TCGA-08-	TCGA-08-	GBM	69	MALE	WHITE	Untreated primary (c	2005	-25304	Dead	WITH TU	322	425				1	425	1	425			1	425
TCGA-08-	TCGA-08-	GBM	60	MALE	WHITE	Untreated primary (c	2005	-22056	Dead	WITH TU	9	22				1	22	1	22			1	22
TCGA-08-	TCGA-08-	GBM	63	MALE	BLACK OR AFRICAN AMERICAN	Untreated primary (c	1999	-23273	Dead	WITH TU	17	382				1	382	1	382			1	382
TCGA-08-	TCGA-08-	GBM	75	MALE	WHITE	Untreated primary (c	2000	-27675	Dead	WITH TU	107	130	Recurrence	90		1	130	1	130			1	90
TCGA-08-	TCGA-08-	GBM	48	MALE	WHITE	Untreated primary (c	2000	-17821	Dead	WITH TU	358	1282	Progression of Disease	231		1	1282	1	1282			1	231
TCGA-08-	TCGA-08-	GBM	69	FEMALE	WHITE	Untreated primary (c	2001	-25457	Dead	WITH TU	259	337				1	337	1	337			1	337
TCGA-08-	TCGA-08-	GBM	14	MALE	WHITE	Untreated primary (c	2001	-5309	Dead	WITH TU	561	596	Progression of Disease	40		1	596	1	596			1	40
TCGA-08-	TCGA-08-	GBM	34	FEMALE	WHITE	Untreated primary (c	1996	-12771	Dead	WITH TU	1770	1785	Recurrence	1024		1	1785	1	1785			1	1024
TCGA-08-	TCGA-08-	GBM	60	FEMALE	WHITE	Untreated primary (c	2001	-21995	Dead	WITH TU	39	588	Progression of Disease	134		1	588	1	588			1	134
TCGA-08-	TCGA-08-	GBM	70	MALE	WHITE	Untreated primary (c	2001	-25766	Dead	WITH TU	327	327	Recurrence	105		1	327	1	327			1	105
TCGA-08-	TCGA-08-	GBM	17	MALE	BLACK OR AFRICAN AMERICAN	Untreated primary (c	2002	-6377	Dead	WITH TU	146	146	Progression of Disease	125		1	146	1	146			1	125
TCGA-08-	TCGA-08-	GBM	17	FEMALE	WHITE	Untreated primary (c	2002	-6464	Dead	WITH TU	199	221	Progression of Disease	61		1	221	1	221			1	61
TCGA-08-	TCGA-08-	GBM	52	MALE		Untreated primary (c	2001	-19013	Dead	WITH TU	486	486	Progression of Disease	343		1	486	1	486			1	343
TCGA-08-	TCGA-08-	GBM	56	FEMALE		Untreated primary (c	2004	-20556	Dead	WITH TU	527	560	Recurrence	328		1	560	1	560			1	328
TCGA-08-	TCGA-08-	GBM	64	MALE	WHITE	Untreated primary (c	2005	-23482	Dead	WITH TU	168	230				1	230	1	230			1	230
TCGA-12-	TCGA-12-	GBM	78	FEMALE	WHITE	Untreated primary (c	2005	-28583	Dead	WITH TU	426	467	Progression of Disease	160		1	467	1	467			1	160
TCGA-12-	TCGA-12-	GBM	36	FEMALE	WHITE	Untreated primary (c	2006	-13451	Dead	WITH TU	439	448	Progression of Disease	398		1	448	1	448			1	398
TCGA-12-	TCGA-12-	GBM	49	MALE	WHITE	Untreated primary (c	2003	-18071	Dead		50	395				1	395		395			0	395
TCGA-12-	TCGA-12-	GBM	60	MALE	WHITE	Untreated primary (c	2003	-21920	Dead	WITH TU	316	1062	Progression of Disease	203		1	1062	1	1062			1	203
TCGA-12-	TCGA-12-	GBM	57	MALE	WHITE	Untreated primary (c	2005	-21069	Dead	WITH TU	181	318				1	318	1	318			1	318
TCGA-12-	TCGA-12-	GBM	48	FEMALE	WHITE	Untreated primary (c	2000	-17625	Dead	WITH TU	218	231	Progression of Disease	216		1	231	1	231			1	216
TCGA-12-	TCGA-12-	GBM	48	FEMALE	BLACK OR AFRICAN AMERICAN	Untreated primary (c	1997	-17865	Dead	WITH TU	2005	2883	Progression of Disease	1467		1	2883	1	2883			1	1467
TCGA-12-	TCGA-12-	GBM	69	MALE	WHITE	Untreated primary (c	1996	-25413	Dead	WITH TU	3	3				1	3	1	3			1	3
TCGA-12-	TCGA-12-	GBM	51	MALE	WHITE	Untreated primary (c	1998	-18717	Dead	WITH TU	1161	1161	Progression of Disease	600		1	1161	1	1161			1	600
TCGA-12-	TCGA-12-	GBM	60	MALE	WHITE	Untreated primary (c	2001	-22189	Dead		790	790	Progression of Disease	379		1	790		790			1	379
TCGA-12-	TCGA-12-	GBM	74	MALE	WHITE	Untreated primary (c	1998	-27085	Dead	WITH TU	734	811	Progression of Disease	228		1	811	1	811			1	228
TCGA-12-	TCGA-12-	GBM	63	MALE	WHITE	Untreated primary (c	1999	-23019	Dead	WITH TU	186	369	Progression of Disease	98		1	369	1	369			1	98
TCGA-12-	TCGA-12-	GBM	75	FEMALE	WHITE	Untreated primary (c	2000	-27519	Dead	WITH TU	80	111	Progression of Disease	35		1	111	1	111			1	35
TCGA-12-	TCGA-12-	GBM	46	MALE	WHITE	Untreated primary (c	1996	-17125	Dead	WITH TU	551	620	Progression of Disease	208		1	620	1	620			1	208
TCGA-12-	TCGA-12-	GBM	55	MALE	WHITE	Untreated primary (c	1997	-20136	Dead		0	864				1	864		864			0	864
TCGA-12-	TCGA-12-	GBM	52	MALE	WHITE	Untreated primary (c	2002	-19171	Dead	WITH TU	378	458	Progression of Disease	378		1	458	1	458			1	378
TCGA-12-	TCGA-12-	GBM	46	MALE	WHITE	Untreated primary (c	2003	-17019	Dead	WITH TU	1615	1638	Progression of Disease	607		1	1638	1	1638			1	607
TCGA-12-	TCGA-12-	GBM	24	MALE	WHITE	Untreated primary (c	2003	-8849	Dead	WITH TU	1311	1315	Progression of Disease	725		1	1315	1	1315			1	725
TCGA-12-	TCGA-12-	GBM	75	FEMALE	BLACK OR AFRICAN AMERICAN	Untreated primary (c	2004	-27565	Dead	WITH TU	167	232	Progression of Disease	94		1	232	1	232			1	94
TCGA-12-	TCGA-12-	GBM	52	MALE	WHITE	Untreated primary (c	2005	-19228	Dead	WITH TU	277	296	Progression of Disease	127		1	296	1	296			1	127
TCGA-12-	TCGA-12-	GBM	53	MALE	WHITE	Untreated primary (c	2006	-19460	Dead	WITH TU	452	454	Progression of Disease	232		1	454	1	454			1	232
TCGA-12-	TCGA-12-	GBM	51	FEMALE	WHITE	Untreated primary (c	2006	-18646	Dead	WITH TU	434	452	Progression of Disease	91		1	452	1	452			1	91
TCGA-12-	TCGA-12-	GBM	45	FEMALE	WHITE	Untreated primary (c	1994	-16666	Dead		2791	2791				1	2791		2791			0	2791
TCGA-12-	TCGA-12-	GBM	49	FEMALE	BLACK OR AFRICAN AMERICAN	Untreated primary (c	1995	-18160	Dead	WITH TU	754	754	Recurrence	428		1	754	1	754			1	428
TCGA-12-	TCGA-12-	GBM	40	MALE	WHITE	Untreated primary (c	1997	-14878	Dead	WITH TU	562	562	Recurrence	272		1	562	1	562			1	272
TCGA-12-	TCGA-12-	GBM	62	MALE	WHITE	Untreated primary (c	1998	-22949	Dead	WITH TU	323	323	Recurrence	259		1	323	1	323			1	259
TCGA-12-	TCGA-12-	GBM	46	MALE	WHITE	Untreated primary (c	1998	-16803	Dead	WITH TU	715	715	Recurrence	91		1	715	1	715			1	91
TCGA-12-	TCGA-12-	GBM	38	FEMALE	WHITE	Untreated primary (c	1999	-13887	Dead		845	845				1	845		845			0	845
TCGA-12-	TCGA-12-	GBM	38	FEMALE	WHITE	Untreated primary (c	1999	-14093	Dead	WITH TU	1179	1179				1	1179	1	1179			1	1179
TCGA-12-	TCGA-12-	GBM	57	MALE	WHITE	Untreated primary (c	2000	-20909	Dead	WITH TU	272	272	Progression of Disease	13		1	272	1	272			1	13
TCGA-12-	TCGA-12-	GBM	75	MALE	BLACK OR AFRICAN AMERICAN	Untreated primary (c	2006	-27471	Dead	WITH TU	626	626	Recurrence	473		1	626	1	626			1	473
TCGA-12-	TCGA-12-	GBM	53	FEMALE	WHITE	Untreated primary (c	1989	-19667	Dead		3881	3881				1	3881		3881			0	3881
TCGA-12-	TCGA-12-	GBM	64	MALE	WHITE	Untreated primary (c	2000	-23419	Dead	WITH TU	177	177				1	177	1	177			1	177
TCGA-12-	TCGA-12-	GBM	58	MALE	WHITE	Untreated primary (c	2001	-21377	Dead	WITH TU	190	231				1	231	1	231			1	231
TCGA-12-	TCGA-12-	GBM	10	FEMALE	WHITE	Untreated primary (c	2001	-3982	Dead	WITH TU	929	1010	Recurrence	56		1	1010	1	1010			1	56
TCGA-12-	TCGA-12-	GBM	58	MALE	WHITE	Untreated primary (c	2006	-21411	Dead	WITH TU	658	661	Recurrence	637		1	661	1	661			1	637
TCGA-12-	TCGA-12-	GBM	66	FEMALE	WHITE	Untreated primary (c	2006	-24297	Dead	WITH TU	364	486	Recurrence	322		1	486	1	486			1	322
TCGA-12-	TCGA-12-	GBM	56	MALE	WHITE	Untreated primary (c	2006	-20586	Dead	WITH TU	346	372	Recurrence	186		1	372	1	372			1	186
TCGA-12-	TCGA-12-	GBM	65	FEMALE	WHITE	Untreated primary (c	2006	-23775	Dead	WITH TU	482	482	Progression of Disease	96		1	482	1	482			1	96
TCGA-12-	TCGA-12-	GBM	55	MALE	WHITE	Untreated primary (c	2007	-20321	Dead	WITH TUMOR		277	Recurrence	270		1	277	1	277			1	270
TCGA-12-	TCGA-12-	GBM	61	MALE	WHITE	Untreated primary (c	2007	-22526	Dead	WITH TU	442	442	Progression of Disease	166		1	442	1	442			1	166
TCGA-12-	TCGA-12-	GBM	75	FEMALE	WHITE	Untreated primary (c	2007	-27726	Dead	WITH TU	121	121	Recurrence	93		1	121	1	121			1	93
TCGA-12-	TCGA-12-	GBM	68	FEMALE	WHITE	Untreated primary (c	2008	-25034	Dead	WITH TU	104	126	Recurrence	62		1	126	1	126			1	62
TCGA-12-	TCGA-12-	GBM	62	FEMALE	WHITE	Untreated primary (c	2002	-22865	Dead	WITH TU	427	675	Progression of Disease	180		1	675	1	675			1	180
TCGA-12-	TCGA-12-	GBM	75	FEMALE	WHITE	Untreated primary (c	2006	-27730	Dead	WITH TU	287	476				1	476	1	476			1	476
TCGA-12-	TCGA-12-	GBM	47	FEMALE	WHITE	Untreated primary (c	2006	-17432	Dead	WITH TU	769	781	Progression of Disease	89		1	781	1	781			1	89
TCGA-12-	TCGA-12-	GBM	86	MALE	WHITE	Untreated primary (c	2006	-31419	Dead	WITH TU	414	448	Progression of Disease	404		1	448	1	448			1	404
TCGA-12-	TCGA-12-	GBM	58	MALE	WHITE	Untreated primary (c	2008	-21492	Dead	WITH TU	195	206	Progression of Disease	140		1	206	1	206			1	140
TCGA-12-	TCGA-12-	GBM	47	FEMALE	WHITE	Untreated primary (c	2004	-17468	Dead	WITH TU	1807	1818	Progression of Disease	1142		1	1818	1	1818			1	1142
TCGA-12-	TCGA-12-	GBM	59	FEMALE	WHITE	Untreated primary (c	2005	-21855	Dead	WITH TU	1315	1339	Progression of Disease	1315		1	1339	1	1339			1	1315
TCGA-12-	TCGA-12-	GBM	61	FEMALE	WHITE	Untreated primary (c	2007	-22463	Dead	WITH TU	784	819	Progression of Disease	86		1	819	1	819			1	86
TCGA-12-	TCGA-12-	GBM	76	MALE	WHITE	Untreated primary (c	2008	-27846	Dead	WITH TU	351	463	Progression of Disease	351		1	463	1	463			1	351
TCGA-12-	TCGA-12-	GBM	46	MALE	WHITE	Untreated primary (c	2008	-17138	Dead	WITH TU	333	333	Progression of Disease	239		1	333	1	333			1	239
TCGA-12-	TCGA-12-	GBM	77	MALE	WHITE	Untreated primary (c	2008	-28277	Dead	WITH TU	358	386	Progression of Disease	358		1	386	1	386			1	358
TCGA-12-	TCGA-12-	GBM	60	MALE	WHITE	Untreated primary (c	2003	-21920	Dead	WITH TU	1050	1062	Progression of Disease	203		1	1062	1	1062			1	203
TCGA-12-	TCGA-12-	GBM	34	FEMALE	WHITE	Untreated primary (c	2008	-12529	Dead	WITH TU	357	442	Progression of Disease	224		1	442	1	442			1	224
TCGA-12-	TCGA-12-	GBM	60	FEMALE	WHITE	Untreated primary (c	2008	-22161	Dead	WITH TU	399	454	Progression of Disease	399		1	454	1	454			1	399
TCGA-12-	TCGA-12-	GBM	56	FEMALE	WHITE	Untreated primary (c	2009	-20463	Dead	WITH TU	98	98				1	98	1	98			1	98
TCGA-12-	TCGA-12-	GBM	59	MALE	WHITE	Untreated primary (c	2010	-21768	Dead	WITH TU	62	62				1	62	1	62			1	62
TCGA-14-	TCGA-14-	GBM	49	MALE	BLACK OR AFRICAN AMERICAN	Untreated primary (c	1999	-18219	Dead	WITH TU	460	460	Recurrence	128		1	460	1	460			1	128
TCGA-14-	TCGA-14-	GBM	49	MALE	BLACK OR AFRICAN AMERICAN	Untreated primary (c	1999	-18219	Dead	WITH TU	460	460	Recurrence	128		1	460	1	460			1	128
TCGA-14-	TCGA-14-	GBM	70	MALE	WHITE	Untreated primary (c	2002	-25627	Dead	WITH TU	364	364				1	364	1	364			1	364
TCGA-14-	TCGA-14-	GBM	49	MALE	BLACK OR AFRICAN AMERICAN	Untreated primary (c	2003	-17903	Dead	WITH TU	29	29				1	29	1	29			1	29
TCGA-14-	TCGA-14-	GBM	36	FEMALE	WHITE	Untreated primary (c	2000	-13159	Dead	WITH TU	189	189				1	189	1	189			1	189
TCGA-14-	TCGA-14-	GBM	50	FEMALE	WHITE	Untreated primary (c	1998	-18592	Dead	WITH TU	701	701	Recurrence	127		1	701	1	701			1	127
TCGA-14-	TCGA-14-	GBM	69	MALE	ASIAN	Untreated primary (c	1999	-25326	Dead		68	68				1	68		68			0	68
TCGA-14-	TCGA-14-	GBM	54	MALE	WHITE	Untreated primary (c	2005	-20050	Dead		342	342	Progression of Disease	105		1	342		342			1	105
TCGA-14-	TCGA-14-	GBM	64	FEMALE	WHITE	Untreated primary (c	2006	-23674	Dead	WITH TU	419	419				1	419	1	419			1	419
TCGA-14-	TCGA-14-	GBM	65	MALE	WHITE	Untreated primary (c	2003	-24085	Dead	WITH TU	99	99				1	99	1	99			1	99
TCGA-14-	TCGA-14-	GBM	78	MALE	WHITE	Untreated primary (c	2004	-28582	Dead		41	41				1	41		41			0	41
TCGA-14-	TCGA-14-	GBM	69	FEMALE	WHITE	Untreated primary (c	2002	-25425	Dead		164	164				1	164		164			0	164
TCGA-14-	TCGA-14-	GBM	60	MALE	WHITE	Untreated primary (c	2002	-22179	Dead	WITH TU	88	88				1	88	1	88			1	88
TCGA-14-	TCGA-14-	GBM	67	MALE	WHITE	Untreated primary (c	2002	-24797	Dead	WITH TU	502	502	Progression of Disease	215		1	502	1	502			1	215
TCGA-14-	TCGA-14-	GBM	55	MALE	WHITE	Untreated primary (c	2002	-20179	Dead		801	801	Recurrence	97		1	801		801			1	97
TCGA-14-	TCGA-14-	GBM	67	MALE	WHITE	Untreated primary (c	2002	-24815	Dead		62	62				1	62		62			0	62
TCGA-14-	TCGA-14-	GBM	74	FEMALE	WHITE	Untreated primary (c	1998	-27387	Dead		880	880				1	880		880			0	880
TCGA-14-	TCGA-14-	GBM	60	FEMALE		Untreated primary (c	1999	-22029	Dead	WITH TU	485	485	Progression of Disease	282		1	485	1	485			1	282
TCGA-14-	TCGA-14-	GBM	60	FEMALE		Untreated primary (c	1999	-22029	Dead	WITH TU	485	485	Progression of Disease	282		1	485	1	485			1	282
TCGA-14-	TCGA-14-	GBM	66	FEMALE	WHITE	Untreated primary (c	2002	-24426	Dead	WITH TU	587	587	Recurrence	326		1	587	1	587			1	326
TCGA-14-	TCGA-14-	GBM	61	MALE	WHITE	Untreated primary (c	2004	-22392	Dead	WITH TU	24	24				1	24	1	24			1	24
TCGA-14-	TCGA-14-	GBM	52	MALE	WHITE	Untreated primary (c	2003	-19237	Dead	WITH TU	42	42				1	42	1	42			1	42
TCGA-14-	TCGA-14-	GBM	78	FEMALE	WHITE	Untreated primary (c	2003	-28753	Dead	WITH TU	34	34				1	34	1	34			1	34
TCGA-14-	TCGA-14-	GBM	54	MALE	WHITE	Untreated primary (c	2004	-19921	Dead	WITH TU	114	114				1	114	1	114			1	114
TCGA-14-	TCGA-14-	GBM	58	FEMALE	WHITE	Untreated primary (c	2005	-21421	Dead	WITH TU	975	975	Progression of Disease	333		1	975	1	975			1	333
TCGA-14-	TCGA-14-	GBM	58	FEMALE	WHITE	Untreated primary (c	2005	-21421	Dead	WITH TU	975	975	Progression of Disease	333		1	975	1	975			1	333
TCGA-14-	TCGA-14-	GBM	57	FEMALE	BLACK OR AFRICAN AMERICAN	Untreated primary (c	2005	-20960	Alive	WITH TU	1788		Progression of Disease	826		0	1788	0	1788			1	826
TCGA-14-	TCGA-14-	GBM	39	MALE	WHITE	Untreated primary (c	2005	-14401	Dead	WITH TU	703	703	Recurrence	19		1	703	1	703			1	19
TCGA-14-	TCGA-14-	GBM	60	MALE	WHITE	Untreated primary (c	2005	-22106	Dead	WITH TU	216	216	Recurrence	172		1	216	1	216			1	172
TCGA-14-	TCGA-14-	GBM	69	MALE	WHITE	Untreated primary (c	2005	-25297	Dead	WITH TU	35	35				1	35	1	35			1	35
TCGA-14-	TCGA-14-	GBM	54	FEMALE	WHITE	Untreated primary (c	2005	-19887	Dead	WITH TU	918	918	Recurrence	291		1	918	1	918			1	291
TCGA-14-	TCGA-14-	GBM	61	MALE	WHITE	Untreated primary (c	2005	-22562	Dead	WITH TU	28	28				1	28	1	28			1	28
TCGA-14-	TCGA-14-	GBM	23	MALE	WHITE	Untreated primary (c	2006	-8710	Alive	WITH TU	1246		Progression of Disease	98		0	1246	0	1246			1	98
TCGA-14-	TCGA-14-	GBM	54	MALE	WHITE	Untreated primary (c	2008	-19899	Dead		187	203	Recurrence	81		1	203		203			1	81
TCGA-14-	TCGA-14-	GBM	63	FEMALE	WHITE	Untreated primary (c	2007	-23231	Dead	WITH TU	378	378	Progression of Disease	275		1	378	1	378			1	275
TCGA-14-	TCGA-14-	GBM	59	MALE		Untreated primary (c	2005	-21628	Dead	WITH TU	30	30				1	30	1	30			1	30
TCGA-14-	TCGA-14-	GBM	66	MALE	WHITE	Untreated primary (c	2006	-24375	Dead	WITH TU	60	60				1	60	1	60			1	60
TCGA-14-	TCGA-14-	GBM	31	MALE	WHITE	Untreated primary (c	2005	-11329	Dead	WITH TU	541	541	Recurrence	331		1	541	1	541			1	331
TCGA-14-	TCGA-14-	GBM	58	FEMALE	WHITE	Untreated primary (c	2007	-21384	Dead	WITH TU	543	543	Recurrence	140		1	543	1	543			1	140
TCGA-14-	TCGA-14-	GBM	70	MALE	WHITE	Untreated primary (c	2007	-25684	Dead	WITH TUMOR		232	Progression of Disease	82		1	232	1	232			1	82
TCGA-14-	TCGA-14-	GBM	51	MALE	WHITE	Untreated primary (c	1998	-18828	Dead		179	179				1	179		179			0	179
TCGA-14-	TCGA-14-	GBM	57	MALE	BLACK OR AFRICAN AMERICAN	Untreated primary (c	2009	-20929	Alive	WITH TU	218					0	218	0	218			0	218
TCGA-14-	TCGA-14-	GBM	52	FEMALE	WHITE	Untreated primary (c	2002	-19090	Dead	WITH TU	532	532				1	532	1	532			1	532
TCGA-14-	TCGA-14-	GBM	44	FEMALE	WHITE	Untreated primary (c	2009	-16259	Alive		242					0	242	0	242			0	242
TCGA-14-	TCGA-14-	GBM	74	MALE	WHITE	Untreated primary (c	2009	-27172	Dead		12	12	Progression of Disease	9		1	12		12			1	9
TCGA-14-	TCGA-14-	GBM	38	FEMALE	BLACK OR AFRICAN AMERICAN	Untreated primary (c	2009	-14082	Alive	WITH TU	115					0	115	0	115			0	115
TCGA-14-	TCGA-14-	GBM	40	MALE	BLACK OR AFRICAN AMERICAN	Untreated primary (c	2010	-14816	Alive		104		Progression of Disease	96		0	104	0	104			1	96
TCGA-15-	TCGA-15-	GBM	65	MALE	WHITE	Untreated primary (c	2005	-24050	Dead			419	Progression of Disease	232		1	419		419			1	232
TCGA-15-	TCGA-15-	GBM	21	MALE	WHITE	Glioblastoma Multif	2004	-7827	Dead	WITH TUMOR		1537	Progression of Disease	1550	Complete Remission/Response	1	1537	1	1537	1	1550	1	1550
TCGA-15-	TCGA-15-	GBM	55	MALE	WHITE	Untreated primary (c	2006	-20355	Alive	WITH TU	581		Recurrence	443		0	581	0	581			1	443
TCGA-15-	TCGA-15-	GBM	36	FEMALE	WHITE	Untreated primary (c	2004	-13326	Alive	WITH TU	1618		Recurrence	340		0	1618	0	1618			1	340
TCGA-15-	TCGA-15-	GBM	65	MALE	WHITE	Untreated primary (c	2006	-23828	Dead	WITH TUMOR		404	Recurrence	74		1	404	1	404			1	74
TCGA-16-	TCGA-16-	GBM	85	MALE	WHITE	Untreated primary (c	2006	-31111	Dead	WITH TU	119	119				1	119	1	119			1	119
TCGA-16-	TCGA-16-	GBM	57	MALE	WHITE	Untreated primary (c	2007	-20848	Dead	WITH TU	302	535	Recurrence	129		1	535	1	535			1	129
TCGA-16-	TCGA-16-	GBM	54	MALE	WHITE	Untreated primary (c	2007	-19939	Alive		793					0	793	0	793			0	793
TCGA-16-	TCGA-16-	GBM	52	FEMALE	WHITE	Untreated primary (c	2007	-19281	Dead	WITH TU	496	498	Recurrence	83		1	498	1	498			1	83
TCGA-16-	TCGA-16-	GBM	65	MALE	WHITE	Untreated primary (c	2008	-23778			131		Recurrence	94		0	131		131			1	94
TCGA-16-	TCGA-16-	GBM	49	FEMALE	WHITE	Untreated primary (c	2006	-18195	Dead	WITH TU	883	883	Recurrence	654		1	883	1	883			1	654
TCGA-16-	TCGA-16-	GBM	66	FEMALE		Untreated primary (c	2005	-24301	Dead	WITH TU	25	139	Progression of Disease	25		1	139	1	139			1	25
TCGA-16-	TCGA-16-	GBM	48	MALE	WHITE	Untreated primary (c	2004	-17840	Dead	WITH TU	301	313	Recurrence	288		1	313	1	313			1	288
TCGA-16-	TCGA-16-	GBM	55	MALE	WHITE	Untreated primary (c	2006	-20310	Dead	WITH TU	406	426	Recurrence	239		1	426	1	426			1	239
TCGA-16-	TCGA-16-	GBM	70	FEMALE	WHITE	Untreated primary (c	2005	-25813	Dead	WITH TU	111	278	Progression of Disease	111		1	278	1	278			1	111
TCGA-16-	TCGA-16-	GBM	57	FEMALE	WHITE	Untreated primary (c	2006	-21148	Dead	WITH TU	556	646	Progression of Disease	518		1	646	1	646			1	518
TCGA-16-	TCGA-16-	GBM	72	MALE	WHITE	Untreated primary (c	2006	-26313	Dead	WITH TU	425	425	Progression of Disease	369		1	425	1	425			1	369
TCGA-16-	TCGA-16-	GBM	36	FEMALE	WHITE	Untreated primary (c	2008	-13208	Alive		195					0	195	0	195			0	195
TCGA-19-	TCGA-19-	GBM	71	MALE	ASIAN	Untreated primary (c	2007	-25974	Alive	TUMOR F	358					0	358	0	358			0	358
TCGA-19-	TCGA-19-	GBM	48	FEMALE	WHITE	Untreated primary (c	2007	-17714	Dead	WITH TU	666	666	Recurrence	50		1	666	1	666			1	50
TCGA-19-	TCGA-19-	GBM	48	FEMALE	WHITE	Untreated primary (c	2007	-17714	Dead	WITH TU	666	666	Recurrence	50		1	666	1	666			1	50
TCGA-19-	TCGA-19-	GBM	83	FEMALE	WHITE	Untreated primary (c	2008	-30364	Alive		262					0	262	0	262			0	262
TCGA-19-	TCGA-19-	GBM	77	FEMALE	WHITE	Untreated primary (c	2008	-28382	Dead		13	20				1	20		20			0	20
TCGA-19-	TCGA-19-	GBM	61	MALE	WHITE	Untreated primary (c	2008	-22326	Dead	WITH TU	434	434	Recurrence	310		1	434	1	434			1	310
TCGA-19-	TCGA-19-	GBM	70	MALE	WHITE	Untreated primary (c	2008	-25800	Alive	WITH TU	103					0	103	0	103			0	103
TCGA-19-	TCGA-19-	GBM	69	MALE	WHITE	Untreated primary (c	2008	-25459	Dead	WITH TU	327	327	Recurrence	177		1	327	1	327			1	177
TCGA-19-	TCGA-19-	GBM	51	MALE	WHITE	Untreated primary (c	2008	-18678	Dead	WITH TU	276	276	Recurrence	237		1	276	1	276			1	237
TCGA-19-	TCGA-19-	GBM	81	MALE	WHITE	Untreated primary (c	2008	-29892	Alive	WITH TU	181		Recurrence	79		0	181	0	181			1	79
TCGA-19-	TCGA-19-	GBM	58	MALE	WHITE	Untreated primary (c	2008	-21368	Dead	WITH TU	394	394	Progression of Disease	148		1	394	1	394			1	148
TCGA-19-	TCGA-19-	GBM	51	MALE	WHITE	Untreated primary (c	2008	-18659	Dead	WITH TU	141	141	Recurrence	81		1	141	1	141			1	81
TCGA-19-	TCGA-19-	GBM	51	MALE	WHITE	Untreated primary (c	2008	-18659	Dead	WITH TU	141	141	Recurrence	81		1	141	1	141			1	81
TCGA-19-	TCGA-19-	GBM	63	FEMALE	WHITE	Untreated primary (c	2007	-23063	Dead		772	772				1	772		772			0	772
TCGA-19-	TCGA-19-	GBM	72	FEMALE	WHITE	Untreated primary (c	2008	-26593	Dead		106	111				1	111		111			0	111
TCGA-19-	TCGA-19-	GBM	64	FEMALE	ASIAN	Untreated primary (c	2008	-23568	Alive	TUMOR F	218					0	218	0	218			0	218
TCGA-19-	TCGA-19-	GBM	48	MALE	WHITE	Untreated primary (c	2008	-17894	Dead	WITH TU	385	385	Recurrence	308		1	385	1	385			1	308
TCGA-19-	TCGA-19-	GBM	39	MALE	WHITE	Untreated primary (c	2009	-14301	Dead		112	112				1	112		112			0	112
TCGA-19-	TCGA-19-	GBM	69	FEMALE	WHITE	Untreated primary (c	2009	-25282	Dead		99	99				1	99		99			0	99
TCGA-19-	TCGA-19-	GBM	56	MALE	WHITE	Untreated primary (c	2008	-20626	Dead	WITH TU	154	154	Progression of Disease	88		1	154	1	154			1	88
TCGA-19-	TCGA-19-	GBM	82	FEMALE	WHITE	Untreated primary (c	2009	-30065	Alive	WITH TU	4					0	4	0	4			0	4
TCGA-19-	TCGA-19-	GBM	55	FEMALE	BLACK OR AFRICAN AMERICAN	Untreated primary (c	2009	-20159	Alive	WITH TU	294		Progression of Disease	146		0	294	0	294			1	146
TCGA-19-	TCGA-19-	GBM	70	MALE	WHITE	Untreated primary (c	2009	-25665	Dead	TUMOR F	148	148				1	148	0	148			0	148
TCGA-19-	TCGA-19-	GBM	83	MALE	WHITE	Untreated primary (c	2009	-30429	Dead		33	33				1	33		33			0	33
TCGA-19-	TCGA-19-	GBM	65	MALE	WHITE	Untreated primary (c	2009	-23835	Alive	TUMOR F	229					0	229	0	229			0	229
TCGA-19-	TCGA-19-	GBM	51	MALE	WHITE	Untreated primary (c	2009	-18891	Dead	TUMOR F	5	5				1	5	0	5			0	5
TCGA-19-	TCGA-19-	GBM	76	FEMALE	WHITE	Untreated primary (c	2009	-27930	Dead	TUMOR F	124	124				1	124	0	124			0	124
TCGA-19-	TCGA-19-	GBM	60	MALE	WHITE	Untreated primary (c	2009	-22257	Dead	WITH TU	501	737	Progression of Disease	145		1	737	1	737			1	145
TCGA-19-	TCGA-19-	GBM	74	FEMALE	WHITE	Untreated primary (c	2009	-27200	Dead	TUMOR F	213	213				1	213	0	213			0	213
TCGA-19-	TCGA-19-	GBM	36	MALE	WHITE	Untreated primary (c	2009	-13257	Alive	WITH TU	214		Recurrence	70		0	214	0	214			1	70
TCGA-19-	TCGA-19-	GBM	36	MALE	WHITE	Untreated primary (c	2009	-13257	Alive	WITH TU	214		Recurrence	70		0	214	0	214			1	70
TCGA-19-	TCGA-19-	GBM	68	MALE	WHITE	Untreated primary (c	2009	-24945	Alive	TUMOR F	137					0	137	0	137			0	137
TCGA-19-	TCGA-19-	GBM	47	FEMALE	BLACK OR AFRICAN AMERICAN	Untreated primary (c	2010	-17218	Dead	WITH TU	202	202	Progression of Disease	43		1	202	1	202			1	43
TCGA-19-	TCGA-19-	GBM	52	FEMALE	WHITE	Untreated primary (c	2010	-19244	Dead	WITH TUMOR		523	Recurrence	294	Progressive Disease	1	523	1	523			1	294
TCGA-19-	TCGA-19-	GBM	76	FEMALE	WHITE	Untreated primary (c	2010	-27995	Dead	WITH TU	244	244				1	244	1	244			1	244
TCGA-19-	TCGA-19-	GBM	62	MALE	WHITE	Untreated primary (c	2010	-22841	Dead		567	575				1	575		575			0	575
TCGA-19-	TCGA-19-	GBM	58	MALE	WHITE	Untreated primary (c	2010	-21204	Dead	WITH TU	144	144	Progression of Disease	119		1	144	1	144			1	119
TCGA-19-	TCGA-19-	GBM	72	FEMALE	WHITE	Untreated primary (c	2010	-26355	Dead	WITH TUMOR		368			Stable Disease	1	368	1	368			1	368
TCGA-19-	TCGA-19-	GBM	83	MALE	WHITE	Untreated primary (c	2010	-30550	Dead		54	54				1	54		54			0	54
TCGA-19-	TCGA-19-	GBM	53	FEMALE	WHITE	Untreated primary (c	2010	-19591	Dead	WITH TUMOR		684	Progression of Disease	647	Stable Disease	1	684	1	684			1	647
TCGA-19-	TCGA-19-	GBM	56	MALE	WHITE	Untreated primary (c	2010	-20466	Dead	WITH TUMOR		428	Recurrence	356	Progressive Disease	1	428	1	428			1	356
TCGA-19-	TCGA-19-	GBM	77	FEMALE	WHITE	Untreated primary (c	2010	-28449	Dead	WITH TUMOR		511	Recurrence	119	Progressive Disease	1	511	1	511			1	119
TCGA-19-	TCGA-19-	GBM	56	MALE	WHITE	Untreated primary (c	2010	-20641	Dead	WITH TUMOR		455	Recurrence	382	Stable Disease	1	455	1	455			1	382
TCGA-19-	TCGA-19-	GBM	39	MALE	BLACK OR AFRICAN AMERICAN	Glioblastoma Multif	2012	-14437	Alive	WITH TU	541				Stable Disease	0	541	0	541			0	541
TCGA-19-	TCGA-19-	GBM	68	MALE	BLACK OR AFRICAN AMERICAN	Glioblastoma Multif	2013	-25058	Dead	WITH TUMOR		121			[Unknown]	1	121	1	121			1	121
TCGA-19-	TCGA-19-	GBM	40	MALE	BLACK OR AFRICAN AMERICAN	Glioblastoma Multif	2012	-14970	Alive	WITH TU	868		Progression of Disease	784	Stable Disease	0	868	0	868			1	784
TCGA-26-	TCGA-26-	GBM	36	MALE	WHITE	Untreated primary (c	2008	-13489	Alive	WITH TU	305		Progression of Disease	158		0	305	0	305			1	158
TCGA-26-	TCGA-26-	GBM	63	MALE	WHITE	Untreated primary (c	2007	-23302	Dead	WITH TUMOR		422	Progression of Disease	284		1	422	1	422			1	284
TCGA-26-	TCGA-26-	GBM	48	MALE	WHITE	Untreated primary (c	2008	-17684	Alive	WITH TU	296		Recurrence	233		0	296	0	296			1	233
TCGA-26-	TCGA-26-	GBM	43	MALE	WHITE	Untreated primary (c	2008	-15950	Alive	WITH TU	953					0	953	0	953			0	953
TCGA-26-	TCGA-26-	GBM	60	FEMALE	WHITE	Untreated primary (c	2008	-21990	Alive	WITH TU	217		Progression of Disease	217		0	217	0	217			1	217
TCGA-26-	TCGA-26-	GBM	68	MALE	WHITE	Untreated primary (c	2008	-25171	Dead	WITH TU	285	285	Recurrence	213		1	285	1	285			1	213
TCGA-26-	TCGA-26-	GBM	74	MALE	WHITE	Untreated primary (c	2009	-27196	Alive	TUMOR F	286					0	286	0	286			0	286
TCGA-26-	TCGA-26-	GBM	59	MALE	WHITE	Untreated primary (c	2009	-21560	Alive	TUMOR F	452		Progression of Disease	370		0	452	0	452			1	370
TCGA-26-	TCGA-26-	GBM	74	MALE	WHITE	Untreated primary (c	2009	-27111	Alive	TUMOR F	167					0	167	0	167			0	167
TCGA-26-	TCGA-26-	GBM	72	FEMALE	WHITE	Untreated primary (c	2009	-26490	Dead			270			[Unknown]	1	270		270			0	270
TCGA-26-	TCGA-26-	GBM	78	FEMALE	WHITE	Untreated primary (c	2009	-28496	Dead	WITH TUMOR		577			[Unknown]	1	577	1	577			1	577
TCGA-26-	TCGA-26-	GBM	65	FEMALE	WHITE	Untreated primary (c	2010	-23782	Alive	WITH TU	48					0	48	0	48			0	48
TCGA-26-	TCGA-26-	GBM	57	MALE	WHITE	Untreated primary (c	2010	-21093	Alive	TUMOR F	241					0	241	0	241			0	241
TCGA-26-	TCGA-26-	GBM	65	FEMALE	WHITE	Untreated primary (c	2011	-23884	Alive		71					0	71	0	71			0	71
TCGA-26-	TCGA-26-	GBM	47	MALE	BLACK OR AFRICAN AMERICAN	Glioblastoma Multif	2013	-17365	Alive	WITH TU	588		Recurrence	407	Stable Disease	0	588	0	588			1	407
TCGA-27-	TCGA-27-	GBM	57	MALE	WHITE	Untreated primary (c	2007	-21131	Dead	WITH TU	146	154	Recurrence	124		1	154	1	154			1	124
TCGA-27-	TCGA-27-	GBM	66	MALE	WHITE	Untreated primary (c	2006	-24447	Dead	WITH TU	505	505	Progression of Disease	144		1	505	1	505			1	144
TCGA-27-	TCGA-27-	GBM	59	FEMALE	WHITE	Untreated primary (c	2007	-21678	Dead	WITH TU	0	300	Progression of Disease	176		1	300	1	300			1	176
TCGA-27-	TCGA-27-	GBM	67	FEMALE	WHITE	Untreated primary (c	2006	-24592	Dead	WITH TU	555	737	Recurrence	469		1	737	1	737			1	469
TCGA-27-	TCGA-27-	GBM	56	MALE	WHITE	Untreated primary (c	2006	-20568	Dead	WITH TU	1008	1233	Recurrence	335		1	1233	1	1233			1	335
TCGA-27-	TCGA-27-	GBM	53	FEMALE	WHITE	Untreated primary (c	2007	-19444	Dead	WITH TU	648	648	Progression of Disease	157		1	648	1	648			1	157
TCGA-27-	TCGA-27-	GBM	33	FEMALE	WHITE	Untreated primary (c	2007	-12060	Dead	WITH TUMOR		914	Progression of Disease	172		1	914	1	914			1	172
TCGA-27-	TCGA-27-	GBM	36	MALE	WHITE	Untreated primary (c	2007	-13368	Dead	WITH TU	427	427	Progression of Disease	136		1	427	1	427			1	136
TCGA-27-	TCGA-27-	GBM	59	FEMALE	WHITE	Untreated primary (c	2008	-21706	Dead	WITH TU	350	350	Progression of Disease	119		1	350	1	350			1	119
TCGA-27-	TCGA-27-	GBM	52	MALE	WHITE	Untreated primary (c	2007	-19130	Dead	WITH TU	753	753	Progression of Disease	539		1	753	1	753			1	539
TCGA-27-	TCGA-27-	GBM	48	MALE	WHITE	Untreated primary (c	2009	-17715	Dead	WITH TUMOR		550	Progression of Disease	256		1	550	1	550			1	256
TCGA-27-	TCGA-27-	GBM	34	MALE	WHITE	Untreated primary (c	2009	-12685	Dead	WITH TUMOR		510				1	510	1	510			1	510
TCGA-27-	TCGA-27-	GBM	63	MALE	WHITE	Untreated primary (c	2005	-23034	Dead	WITH TU	489	489	Progression of Disease	402		1	489	1	489			1	402
TCGA-27-	TCGA-27-	GBM	56	MALE	WHITE	Untreated primary (c	2008	-20555	Dead	WITH TU	231	231				1	231	1	231			1	231
TCGA-27-	TCGA-27-	GBM	79	FEMALE	WHITE	Untreated primary (c	2007	-28977	Dead	WITH TU	87	87				1	87	1	87			1	87
TCGA-27-	TCGA-27-	GBM	81	MALE	WHITE	Untreated primary (c	2007	-29619	Dead	WITH TU	438	438				1	438	1	438			1	438
TCGA-27-	TCGA-27-	GBM	62	MALE	WHITE	Untreated primary (c	2008	-22867	Dead	WITH TU	480	480	Progression of Disease	72		1	480	1	480			1	72
TCGA-28-	TCGA-28-	GBM	76	MALE	WHITE	Untreated primary (c	2008	-27817	Alive	WITH TU	345		Progression of Disease	317		0	345	0	345			1	317
TCGA-28-	TCGA-28-	GBM	77	FEMALE	WHITE	Untreated primary (c	2008	-28302	Alive		6					0	6	0	6			0	6
TCGA-28-	TCGA-28-	GBM	44	MALE	WHITE	Untreated primary (c	2008	-16301	Dead		77	77				1	77		77			0	77
TCGA-28-	TCGA-28-	GBM	73	MALE	WHITE	Untreated primary (c	2009	-26765	Alive	WITH TU	280		Progression of Disease	280		0	280	0	280			1	280
TCGA-28-	TCGA-28-	GBM	40	FEMALE	WHITE	Untreated primary (c	2009	-14817	Alive	WITH TU	254		Progression of Disease	116		0	254	0	254			1	116
TCGA-28-	TCGA-28-	GBM	61	FEMALE	WHITE	Untreated primary (c	2009	-22291	Alive	WITH TU	232		Progression of Disease	219		0	232	0	232			1	219
TCGA-28-	TCGA-28-	GBM	42	FEMALE	WHITE	Untreated primary (c	2009	-15565	Alive	WITH TU	258					0	258	0	258			0	258
TCGA-28-	TCGA-28-	GBM	53	MALE	WHITE	Untreated primary (c	2009	-19366	Alive	WITH TU	37					0	37	0	37			0	37
TCGA-28-	TCGA-28-	GBM	52	FEMALE	WHITE	Untreated primary (c	2009	-19263	Dead	WITH TU	47	47				1	47	1	47			1	47
TCGA-28-	TCGA-28-	GBM	78	MALE	WHITE	Untreated primary (c	2009	-28649	Alive	TUMOR F	86					0	86	0	86			0	86
TCGA-28-	TCGA-28-	GBM	71	MALE	WHITE	Untreated primary (c	2009	-26189	Alive		4					0	4	0	4			0	4
TCGA-28-	TCGA-28-	GBM	48	MALE	WHITE	Untreated primary (c	2009	-17758	Alive	WITH TU	143		Progression of Disease	105		0	143	0	143			1	105
TCGA-28-	TCGA-28-	GBM	59	MALE	WHITE	Untreated primary (c	2009	-21838			95					0	95		95			0	95
TCGA-28-	TCGA-28-	GBM	65	MALE	WHITE	Untreated primary (c	2009	-23807	Alive		20					0	20	0	20			0	20
TCGA-28-	TCGA-28-	GBM	63	FEMALE	WHITE	Untreated primary (c	2009	-23292	Alive	WITH TU	205		Progression of Disease	84		0	205	0	205			1	84
TCGA-28-	TCGA-28-	GBM	77	FEMALE	WHITE	Untreated primary (c	2009	-28240	Alive		145					0	145	0	145			0	145
TCGA-28-	TCGA-28-	GBM	37	MALE	WHITE	Untreated primary (c	2009	-13664	Dead	WITH TU	343	343	Progression of Disease	193		1	343	1	343			1	193
TCGA-28-	TCGA-28-	GBM	69	FEMALE	WHITE	Untreated primary (c	2009	-25421	Alive	WITH TU	222		Progression of Disease	75		0	222	0	222			1	75
TCGA-28-	TCGA-28-	GBM	45	MALE	ASIAN	Untreated primary (c	2009	-16737	Alive		160					0	160	0	160			0	160
TCGA-28-	TCGA-28-	GBM	72	MALE	WHITE	Untreated primary (c	2009	-26490	Dead	WITH TUMOR		454				1	454	1	454			1	454
TCGA-28-	TCGA-28-	GBM	71	MALE	WHITE	Untreated primary (c	2010	-25975	Dead	WITH TU	275	343				1	343	1	343			1	343
TCGA-28-	TCGA-28-	GBM	52	MALE	WHITE	Untreated primary (c	2010	-19053	Dead	WITH TUMOR		544		148	Progressive Disease	1	544	1	544			1	148
TCGA-28-	TCGA-28-	GBM	66	FEMALE	WHITE	Untreated primary (c	2010	-24362	Alive		442					0	442	0	442			0	442
TCGA-28-	TCGA-28-	GBM	42	MALE	BLACK OR AFRICAN AMERICAN	Untreated primary (c	2010	-15507	Alive	WITH TU	358			225		0	358	0	358			1	225
TCGA-28-	TCGA-28-	GBM	72	MALE	WHITE	Untreated primary (c	2010	-26491	Alive	WITH TU	951					0	951	0	951			0	951
TCGA-28-	TCGA-28-	GBM	53	MALE	WHITE	Untreated primary (c	2010	-19520	Dead	WITH TUMOR		713	Locoregional Disease	247		1	713	1	713			1	247
TCGA-28-	TCGA-28-	GBM	62	FEMALE	WHITE	Untreated primary (c	2010	-22686	Dead	WITH TU	237	335		164		1	335	1	335			1	164
TCGA-28-	TCGA-28-	GBM	52	MALE	WHITE	Untreated primary (c	2010	-19239	Alive	TUMOR F	415					0	415	0	415			0	415
TCGA-28-	TCGA-28-	GBM	63	MALE	WHITE	Untreated primary (c	2010	-23096	Dead		128	157				1	157		157			0	157
TCGA-28-	TCGA-28-	GBM	47	FEMALE	WHITE	Untreated primary (c	2010	-17254	Alive	TUMOR F	690			258		0	690	0	690			1	258
TCGA-28-	TCGA-28-	GBM	67	MALE	WHITE	Untreated primary (c	2010	-24622	Dead	WITH TUMOR		388		262		1	388	1	388			1	262
TCGA-28-	TCGA-28-	GBM	60	MALE	WHITE	Untreated primary (c	2010	-22177	Dead	WITH TU	165	165				1	165	1	165			1	165
TCGA-32-	TCGA-32-	GBM	59	MALE	WHITE	Untreated primary (c	2005	-21613	Dead	WITH TU	468	468	Progression of Disease	408		1	468	1	468			1	408
TCGA-32-	TCGA-32-	GBM	21	MALE	WHITE	Untreated primary (c	2008	-7756	Dead	WITH TU	641	641	Progression of Disease	507		1	641	1	641			1	507
TCGA-32-	TCGA-32-	GBM	65	MALE	WHITE	Untreated primary (c	2008	-23798	Dead		15	15				1	15		15			0	15
TCGA-32-	TCGA-32-	GBM	63	FEMALE	WHITE	Untreated primary (c	2009	-23205	Alive	WITH TU	3					0	3	0	3			0	3
TCGA-32-	TCGA-32-	GBM	57	MALE	WHITE	Untreated primary (c	2008	-21054	Dead	WITH TU	482	482				1	482	1	482			1	482
TCGA-32-	TCGA-32-	GBM	69	FEMALE	WHITE	Untreated primary (c	2008	-25252	Dead	WITH TU	593	593	Progression of Disease	265		1	593	1	593			1	265
TCGA-32-	TCGA-32-	GBM	72	MALE	WHITE	Untreated primary (c	2008	-26467	Dead	WITH TU	36	36				1	36	1	36			1	36
TCGA-32-	TCGA-32-	GBM	76	FEMALE	WHITE	Untreated primary (c	2008	-27764	Dead	WITH TU	142	142				1	142	1	142			1	142
TCGA-32-	TCGA-32-	GBM	68	MALE	WHITE	Untreated primary (c	2008	-25061	Dead	WITH TU	386	386	Progression of Disease	233		1	386	1	386			1	233
TCGA-32-	TCGA-32-	GBM	49	FEMALE	WHITE	Untreated primary (c	2009	-18091	Dead	WITH TU	452	452	Recurrence	68		1	452	1	452			1	68
TCGA-32-	TCGA-32-	GBM	60	MALE	WHITE	Untreated primary (c	2009	-22021	Dead	WITH TU	515	515	Recurrence	345		1	515	1	515			1	345
TCGA-32-	TCGA-32-	GBM	63	MALE	WHITE	Untreated primary (c	2005	-23131	Dead	WITH TU	372	372	Recurrence	70		1	372	1	372			1	70
TCGA-32-	TCGA-32-	GBM	58	FEMALE	WHITE	Untreated primary (c	2008	-21198	Dead	WITH TU	632	632	Progression of Disease	488		1	632	1	632			1	488
TCGA-32-	TCGA-32-	GBM	59	FEMALE	WHITE	Treated primary GBM	2008	-21860	Dead	WITH TU	457	457	Recurrence	252		1	457	1	457			1	252
TCGA-32-	TCGA-32-	GBM	62	MALE	WHITE	Untreated primary (c	2009	-22916	Dead	WITH TU	485	485	Progression of Disease	131		1	485	1	485			1	131
TCGA-32-	TCGA-32-	GBM	48	FEMALE	WHITE	Untreated primary (c	2008	-17781	Dead	WITH TU	224	224	Progression of Disease	182		1	224	1	224			1	182
TCGA-32-	TCGA-32-	GBM	80	MALE	WHITE	Untreated primary (c	2008	-29281	Dead	WITH TUMOR		269			Progressive Disease	1	269	1	269			1	269
TCGA-32-	TCGA-32-	GBM	82	MALE	WHITE	Untreated primary (c	2009	-29964	Alive	TUMOR F	693					0	693	0	693			0	693
TCGA-32-	TCGA-32-	GBM	67	MALE	WHITE	Untreated primary (c	2009	-24658	Dead	WITH TUMOR		766				1	766	1	766			1	766
TCGA-32-	TCGA-32-	GBM	25	MALE	WHITE	Untreated primary (c	2009	-9213	Alive	WITH TU	643					0	643	0	643			0	643
TCGA-32-	TCGA-32-	GBM	40	MALE	WHITE	Treated primary GBM	2009	-14903	Dead	WITH TUMOR		618	Recurrence	94		1	618	1	618			1	94
TCGA-32-	TCGA-32-	GBM	73	MALE	WHITE	Untreated primary (c	2010	-26768	Dead	WITH TU	113	113				1	113	1	113			1	113
TCGA-32-	TCGA-32-	GBM	56	MALE	WHITE	Untreated primary (c	2010	-20748	Dead	WITH TU	383	383				1	383	1	383			1	383
TCGA-32-	TCGA-32-	GBM	47	FEMALE	WHITE	Untreated primary (c	2009	-17237	Alive	WITH TU	604		Recurrence	353		0	604	0	604			1	353
TCGA-32-	TCGA-32-	GBM	73	MALE	WHITE	Untreated primary (c	2010	-26691	Dead	WITH TU	330	330				1	330	1	330			1	330
TCGA-32-	TCGA-32-	GBM	66	MALE	WHITE	Untreated primary (c	2010	-24134	Dead	WITH TUMOR		585	Progression of Disease	118		1	585	1	585			1	118
TCGA-41-	TCGA-41-	GBM	89	MALE	WHITE	Untreated primary (c	2009	-32612	Dead	WITH TU	26	26	Progression of Disease	26		1	26	1	26			1	26
TCGA-41-	TCGA-41-	GBM	67	MALE	WHITE	Untreated primary (c	2009	-24505	Dead	WITH TU	406	406	Progression of Disease	122		1	406	1	406			1	122
TCGA-41-	TCGA-41-	GBM	59	MALE	WHITE	Untreated primary (c	2009	-21911	Alive	WITH TU	272		Progression of Disease	167		0	272	0	272			1	167
TCGA-41-	TCGA-41-	GBM	75	MALE	WHITE	Untreated primary (c	2009	-27587	Dead	WITH TU	290	290	Progression of Disease	131		1	290	1	290			1	131
TCGA-41-	TCGA-41-	GBM	88	MALE	WHITE	Untreated primary (c	2009	-32351	Dead	WITH TU	30	30				1	30	1	30			1	30
TCGA-41-	TCGA-41-	GBM	81	FEMALE	WHITE	Untreated primary (c	2009	-29904	Dead	WITH TU	135	135				1	135	1	135			1	135
TCGA-41-	TCGA-41-	GBM	48	MALE	WHITE	Untreated primary (c	2010	-17880	Dead	WITH TUMOR		360	Locoregional Disease	288		1	360	1	360			1	288
TCGA-41-	TCGA-41-	GBM	63	FEMALE	WHITE	Untreated primary (c	2010	-23107	Dead	WITH TU	6	6				1	6	1	6			1	6
TCGA-41-	TCGA-41-	GBM	59	FEMALE	BLACK OR AFRICAN AMERICAN	Untreated primary (c	2010	-21698	Dead	WITH TUMOR		460	Progression of Disease	210		1	460	1	460			1	210

TCGA-41	TCGA-41	GBM	73	FEMALE	WHITE			Untreated primary (c		2011	-26667	Dead	WITH TUMOR		379	Progression of Disease			200		1	379	1	379			1	200
TCGA-4W	TCGA-4W	GBM	61	MALE	BLACK OR AFRICAN AMERICAN			Glioblastoma Multifc		2012	-22317	Alive	TUMOR F	124						Progressive Disease	0	124	0	124			0	124
TCGA-4W	TCGA-4W	GBM	69	MALE	BLACK OR AFRICAN AMERICAN			Glioblastoma Multifc		2013	-25545	Alive	TUMOR F	44						Stable Disease	0	44	0	44			0	44
TCGA-4W	TCGA-4W	GBM	54	FEMALE	BLACK OR AFRICAN AMERICAN			Glioblastoma Multifc		2012	-19801	Alive	TUMOR F	778					749	Progressive Disease	0	778	0	778			1	749
TCGA-74	TCGA-74	GBM	67	MALE				Untreated primary (c		2009	-24632	Dead	WITH TUMOR		105						1	105	1	105			1	105
TCGA-74	TCGA-74	GBM	67	MALE				Untreated primary (c		2009	-24632	Dead	WITH TUMOR		105						1	105	1	105			1	105
TCGA-74	TCGA-74	GBM	73	FEMALE				Untreated primary (c		2009	-26827	Alive	WITH TU	636					93		0	636	0	636			1	93
TCGA-74	TCGA-74	GBM	51	MALE				Untreated primary (c		2010	-18740	Alive	WITH TU	132					132		0	132	0	132			1	132
TCGA-74	TCGA-74	GBM	58	MALE				Untreated primary (c		2010	-21464	Alive	WITH TU	436					222		0	436	0	436			1	222
TCGA-74	TCGA-74	GBM	77	MALE				Untreated primary (c		2010	-28263	Alive	WITH TU	250							0	250	0	250			0	250
TCGA-74	TCGA-74	GBM	55	FEMALE				Untreated primary (c		2010	-20232	Alive	WITH TU	228					91		0	228	0	228			1	91
TCGA-76	TCGA-76	GBM	76	MALE	WHITE			Untreated primary (c		2005	-28011	Dead		146	146	Progression of Disease			88		1	146		146			1	88
TCGA-76	TCGA-76	GBM	68	MALE	WHITE			Untreated primary (c		2005	-25085	Dead	WITH TU	138	138	Progression of Disease			34		1	138	1	138			1	34
TCGA-76	TCGA-76	GBM	58	MALE	WHITE			Untreated primary (c		2005	-21193	Dead	WITH TU	535	535	Progression of Disease			416		1	535	1	535			1	416
TCGA-76	TCGA-76	GBM	85	FEMALE	WHITE			Untreated primary (c		2005	-31267	Dead	WITH TU	94	94						1	94	1	94			1	94
TCGA-76	TCGA-76	GBM	76	FEMALE	WHITE			Untreated primary (c		2005	-27831	Dead	WITH TU	111	111						1	111	1	111			1	111
TCGA-76	TCGA-76	GBM	70	FEMALE	WHITE			Untreated primary (c		2005	-25812	Dead	WITH TU	279	279	Progression of Disease			112		1	279	1	279			1	112
TCGA-76	TCGA-76	GBM	50	FEMALE	WHITE			Treated primary GBM		2006	-18433	Dead	WITH TU	1458	1458						1	1458	1	1458			1	1458
TCGA-76	TCGA-76	GBM	66	FEMALE	WHITE			Untreated primary (c		2009	-24319	Dead	TUMOR F	77	77						1	77	0	77			0	77
TCGA-76	TCGA-76	GBM	52	FEMALE	WHITE			Untreated primary (c		2010	-19231	Dead	TUMOR FREE		1121					[Unknown]	1	1121	0	1121			0	1121
TCGA-76	TCGA-76	GBM	57	MALE	WHITE			Untreated primary (c		2009	-20937	Dead	WITH TU	508	508	Progression of Disease			323		1	508	1	508			1	323
TCGA-76	TCGA-76	GBM	74	MALE	WHITE			Untreated primary (c		2010	-27322	Dead	WITH TU	100	100	Progression of Disease			76		1	100	1	100			1	76
TCGA-76	TCGA-76	GBM	78	MALE				Untreated primary (c		2010	-28742	Dead		82	82	Progression of Disease			64		1	82		82			1	64
TCGA-76	TCGA-76	GBM	57	MALE	WHITE			Untreated primary (c		2007	-20931	Dead	WITH TU	346	346	Progression of Disease			108		1	346	1	346			1	108
TCGA-76	TCGA-76	GBM	63	MALE	WHITE			Untreated primary (c		2007	-23323	Dead	WITH TU	519	519	Progression of Disease			458		1	519	1	519			1	458
TCGA-76	TCGA-76	GBM	71	FEMALE	WHITE			Glioblastoma Multifc		2007	-26002	Dead	WITH TUMOR		165						1	165	1	165			1	165
TCGA-76	TCGA-76	GBM	64	FEMALE	WHITE			Untreated primary (c		2008	-23674	Dead	WITH TU	254	254						1	254	1	254			1	254
TCGA-76	TCGA-76	GBM	60	MALE	WHITE			Untreated primary (c		2009	-22173	Dead	WITH TU	638	638	Progression of Disease			180		1	638	1	638			1	180
TCGA-76	TCGA-76	GBM	66	MALE	BLACK OR AFRICAN AMERICAN			Untreated primary (c		2010	-24265	Dead		147	147						1	147		147			0	147
TCGA-76	TCGA-76	GBM	74	MALE	WHITE			Untreated primary (c		2010	-27341	Dead	WITH TU	153	153	Progression of Disease			135		1	153	1	153			1	135
TCGA-76	TCGA-76	GBM	73	MALE	WHITE			Glioblastoma Multifc		2010	-26913	Dead	WITH TUMOR		114						1	114	1	114			1	114
TCGA-76	TCGA-76	GBM	54	MALE	WHITE			Untreated primary (c		2011	-19965	Dead	WITH TUMOR		727					[Unknown]	1	727	1	727			1	727
TCGA-76	TCGA-76	GBM	58	MALE	WHITE			Untreated primary (c		2011	-21511	Dead	WITH TUMOR		1048	Progression of Disease			205		1	1048	1	1048			1	205
TCGA-76	TCGA-76	GBM	44	FEMALE	WHITE			Untreated primary (c		2011	-16126	Dead	WITH TUMOR		567						1	567	1	567			1	567
TCGA-76	TCGA-76	GBM	49	FEMALE	WHITE			Untreated primary (c		2011	-18054	Alive	WITH TU	1101						[Unknown]	0	1101	0	1101			0	1101
TCGA-81	TCGA-81	GBM	64	MALE	WHITE			Untreated primary (c		2009	-23567	Dead	WITH TUMOR		49						1	49	1	49			1	49
TCGA-81	TCGA-81	GBM	33	MALE	WHITE			Untreated primary (c		2010	-12292	Alive	WITH TU	539		Progression of Disease			277		0	539	0	539			1	277
TCGA-87	TCGA-87	GBM	50	FEMALE	WHITE			Untreated primary (c		2010	-18391	Alive	TUMOR F	800						Complete Remission/Response	0	800	0	800	0	800	0	800
TCGA-OX	TCGA-OX	GBM	68	MALE	BLACK OR AFRICAN AMERICAN			Glioblastoma Multifc		2012	-25178	Dead	WITH TUMOR		180					Partial Remission/Response	1	180	1	180			1	180
TCGA-RR	TCGA-RR	GBM	72	MALE	BLACK OR AFRICAN AMERICAN			Glioblastoma Multifc		2012	-26582	Dead	WITH TUMOR		191					Partial Remission/Response	1	191	1	191			1	191
TCGA-RR	TCGA-RR	GBM	51	MALE	BLACK OR AFRICAN AMERICAN			Glioblastoma Multifc		2007	-18713	Alive		0						[Unknown]	0	0	0	0			0	0
TCGA-RR	TCGA-RR	GBM	55	MALE	BLACK OR AFRICAN AMERICAN			Glioblastoma Multifc		2010	-20302	Dead			625	Recurrence			369	Complete Remission/Response	1	625		625	1	369	1	369
TCGA-4P	TCGA-4P	HNSC	66	MALE	BLACK OI	Stage IVA	Stage IVA	Head & N	G2	2013	-24222	Alive	TUMOR F	102						Complete Remission/Response	0	102	0	102			0	102
TCGA-BA	TCGA-BA	HNSC	69	MALE	WHITE	Stage IVA	Stage IVA	Head & N	G3	2003	-25282	Dead	WITH TUMOR		462		Distant M	bone (hur	396	Complete Remission/Response	1	462	1	462			1	396
TCGA-BA	TCGA-BA	HNSC	49	MALE	BLACK OI	Stage III	Stage IVA	Head & N	G2	2004	-17951	Dead	WITH TUMOR		283		Oral Cavity		236	Progressive Disease	1	283	1	283			1	236
TCGA-BA	TCGA-BA	HNSC	39	MALE	WHITE		Stage IVA	Head & N	G2	2003	-14405	Dead	WITH TUMOR		415		Larynx		286	Complete Remission/Response	1	415	1	415			1	286
TCGA-BA	TCGA-BA	HNSC	45	FEMALE	WHITE	Stage IVA	Stage IVB	Head & N	G2	2003	-16536	Dead	WITH TUMOR		1134					Persistent Disease	1	1134	1	1134			1	1134
TCGA-BA	TCGA-BA	HNSC	83	MALE	WHITE		Stage IVA	Head & N	G2	2003	-30480	Dead	WITH TUMOR		276						1	276	1	276			1	276
TCGA-BA	TCGA-BA	HNSC	47	MALE	WHITE	Stage IVA	Stage IVA	Head & N	G2	2010	-17177	Dead	WITH TUMOR		806		Distant M	Lung	389	Complete Remission/Response	1	806	1	806			1	389
TCGA-BA	TCGA-BA	HNSC	72	MALE	WHITE	Stage IVA	Stage IVA	Head & N	G1	2010	-26569	Alive	TUMOR F	722					517	Complete Remission/Response	0	722	0	722			1	517
TCGA-BA	TCGA-BA	HNSC	56	MALE	WHITE	Stage IVA	Stage IVA	Head & N	G2	2009	-20783	Alive	TUMOR F	1288						Complete Remission/Response	0	1288	0	1288			0	1288
TCGA-BA	TCGA-BA	HNSC	51	MALE	WHITE		Stage III	Head & N	G2	2005	-18743	Dead	WITH TUMOR		1762		Distant M	bone (hip	1522	Complete Remission/Response	1	1762	1	1762	1	1522	1	1522
TCGA-BA	TCGA-BA	HNSC	54	MALE	BLACK OI	Stage IVA	Stage IVA	Head & N	G2	2010	-19956	Alive	TUMOR F	520			Orophary	L tonsil	248	Complete Remission/Response	0	520	0	520			1	248
TCGA-BA	TCGA-BA	HNSC	58	FEMALE	WHITE	Stage II	Stage II	Head & N	G3	2010	-21444	Alive	TUMOR F	725						Complete Remission/Response	0	725	0	725	0	725	0	725
TCGA-BA	TCGA-BA	HNSC	41	FEMALE		Stage III	Stage IVA	Head & N	G2	2010	-15247	Alive	TUMOR F	623						Complete Remission/Response	0	623	0	623			0	623
TCGA-BA	TCGA-BA	HNSC	65	MALE	WHITE		Stage IVA	Head & N	G1	2006	-23984	Alive	TUMOR F	1995						Complete Remission/Response	0	1995	0	1995			0	1995
TCGA-BA	TCGA-BA	HNSC	71	MALE	WHITE		Stage IVA	Head & N	G3	2006	-26286	Dead	WITH TUMOR		2083		Oropharynx		1614	Complete Remission/Response	1	2083	1	2083			1	1614
TCGA-BA	TCGA-BA	HNSC	53	MALE	BLACK OR AFRICAN		Stage IVB	Head & N	G2	2011	-19608	Dead	WITH TUMOR		472		Distant M	L neck sul	344	Partial Remission/Response	1	472	1	472			1	344
TCGA-BA	TCGA-BA	HNSC	62	MALE	WHITE	Stage III	Stage III	Head & N	G2	2010	-22765	Alive	TUMOR F	644						Complete Remission/Response	0	644	0	644	0	644	0	644
TCGA-BA	TCGA-BA	HNSC	60	FEMALE	BLACK OR AFRICAN		Stage IVC	Head & N	G2	2010	-22222	Dead			451						1	451		451			0	451
TCGA-BA	TCGA-BA	HNSC	75	MALE	WHITE		Stage IVA	Head & N	G2	2011	-27708	Dead	WITH TUMOR		108					Stable Disease	1	108	1	108			1	108
TCGA-BA	TCGA-BA	HNSC	47	MALE	WHITE		Stage IVA	Head & N	G2	2004	-17296	Dead	WITH TUMOR		384						1	384	1	384			1	384
TCGA-BA	TCGA-BA	HNSC	28	MALE	WHITE	Stage IVA	Stage IVA	Head & N	G2	2011	-10512	Alive	WITH TU	122			Oral Cavit	L Tongue	66	Progressive Disease	0	122	0	122			1	66
TCGA-BA	TCGA-BA	HNSC	61	MALE	WHITE	Stage III	Stage III	Head & N	G1	2010	-22609	Alive	TUMOR F	1273						Complete Remission/Response	0	1273	0	1273	0	1273	0	1273
TCGA-BA	TCGA-BA	HNSC	59	MALE	WHITE		Stage IVA	Head & N	G2	2011	-21635	Alive	TUMOR F	895		New Primary Tumor		LUL Lung	463	Complete Remission/Response	0	895	0	895			1	463
TCGA-BA	TCGA-BA	HNSC	77	MALE	WHITE		Stage IVA	Head & N	G3	2011	-28402	Alive	TUMOR F	855						Complete Remission/Response	0	855	0	855			0	855
TCGA-BA	TCGA-BA	HNSC	57	MALE	WHITE	[Discrepa	Stage IVA	Head & N	G3	2012	-21094	Alive	TUMOR F	622						Complete Remission/Response	0	622	0	622			0	622
TCGA-BA	TCGA-BA	HNSC	46	MALE	WHITE	Stage II	Stage I	Head & N	G1	2011	-17011	Alive	WITH TU	918		Locoregic	Oral Cavit	L FOM	197	Complete Remission/Response	0	918	0	918	1	197	1	197
TCGA-BA	TCGA-BA	HNSC	59	MALE	BLACK OI	Stage IVA	Stage IVA	Head & N	G2	2012	-21778	Alive	WITH TU	850		Distant M	Oral Cavit	R Floor of	849	Complete Remission/Response	0	850	0	850			1	849
TCGA-BA	TCGA-BA	HNSC	41	FEMALE	WHITE	Stage IVA	Stage IVA	Head & N	G2	2012	-15004	Alive	TUMOR F	351						Complete Remission/Response	0	351	0	351			0	351
TCGA-BA	TCGA-BA	HNSC	24	FEMALE	WHITE	Stage I	Stage II	Head & N	G1	2012	-8967	Alive	WITH TU	216		Locoregic	Cervical Lymph Node		208	Complete Remission/Response	0	216	0	216	1	208	1	208
TCGA-BA	TCGA-BA	HNSC	44	MALE	BLACK OI	Stage IVA	Stage IVA	Head & N	G2	2013	-16401	Dead	WITH TUMOR		173		Cervical Lymph Node		96	Progressive Disease	1	173	1	173			1	96
TCGA-BA	TCGA-BA	HNSC	70	FEMALE	WHITE	Stage II	Stage III	Head & N	G2	2013	-25874	Alive	TUMOR F	440						Complete Remission/Response	0	440	0	440	0	440	0	440
TCGA-BA	TCGA-BA	HNSC	80	FEMALE	WHITE	Stage IVA	Stage IVA	Head & N	G2	2012	-29283	Dead	TUMOR FREE		238					Complete Remission/Response	1	238	0	238			0	238
TCGA-BA	TCGA-BA	HNSC	49	MALE	WHITE		Stage IVA	Head & N	G2	2012	-18052	Dead	WITH TUMOR		69					Progressive Disease	1	69	1	69			1	69
TCGA-BA	TCGA-BA	HNSC	62	MALE	BLACK OR AFRICAN		Stage III	Head & N	G2	2012	-22857	Dead	WITH TUMOR		336		Larynx		266	Complete Remission/Response	1	336	1	336	1	266	1	266
TCGA-BA	TCGA-BA	HNSC	62	MALE	ASIAN	Stage IVA	Stage IVA	Head & N	G2	2012	-22967	Dead	WITH TUMOR		407		Distant M	Lungs	240	Complete Remission/Response	1	407	1	407			1	240
TCGA-BA	TCGA-BA	HNSC	59	MALE	WHITE		Stage III	Head & N	G2	2012	-21886	Alive	TUMOR F	623						Complete Remission/Response	0	623	0	623	0	623	0	623
TCGA-BA	TCGA-BA	HNSC	50	MALE	BLACK OI	Stage IVB	Stage IVB	Head & N	G2	2013	-18355	Alive	TUMOR F	499						Complete Remission/Response	0	499	0	499			0	499
TCGA-BB	TCGA-BB	HNSC	68	MALE	BLACK OI	Stage IVA	Stage IVA	Head & N	G3	2008	-25020	Alive	WITH TU	187						Partial Remission/Response	0	187	0	187			0	187
TCGA-BB	TCGA-BB	HNSC	48	MALE	WHITE	Stage IVA	Stage IVA	Head & N	G3	2003	-17608	Alive	TUMOR F	3221						Complete Remission/Response	0	3221	0	3221			0	3221
TCGA-BB	TCGA-BB	HNSC	52	MALE	WHITE	Stage IVA	Stage III	Head & N	G2	2004	-19119	Alive	WITH TU	278			Oral Cavity		175	Progressive Disease	0	278	0	278			1	175
TCGA-BB	TCGA-BB	HNSC	73	MALE	WHITE		Stage IVA	Head & N	G3	2003	-26687	Alive	WITH TU	146						Partial Remission/Response	0	146	0	146			0	146
TCGA-BB	TCGA-BB	HNSC	66	MALE	WHITE	Stage IVA	Stage IVA	Head & N	G2	2010	-24322	Alive	WITH TU	134		Locoregic	Hypopharynx		85	Stable Disease	0	134	0	134			1	85
TCGA-BB	TCGA-BB	HNSC	50	MALE	WHITE	Stage III	Stage II	Head & N	GX	2010	-18536	Alive	TUMOR F	559						Complete Remission/Response	0	559	0	559	0	559	0	559
TCGA-BB	TCGA-BB	HNSC	56	MALE	WHITE	Stage III	Stage III	Head & Neck Squam		2011	-20563	Alive	TUMOR F	682						Complete Remission/Response	0	682	0	682	0	682	0	682
TCGA-BB	TCGA-BB	HNSC	67	MALE	WHITE	Stage IVA	Stage IVA	Head & N	G3	2011	-24823	Alive	TUMOR F	1117						Complete Remission/Response	0	1117	0	1117			0	1117
TCGA-BB	TCGA-BB	HNSC	43	FEMALE	WHITE	Stage III	Stage III	Head & N	G2	2011	-15899	Alive	TUMOR F	1025						Complete Remission/Response	0	1025	0	1025	0	1025	0	1025
TCGA-BB	TCGA-BB	HNSC	61	MALE	WHITE	Stage IVA	Stage IVA	Head & N	G2	2010	-22317	Alive	TUMOR F	1527						Complete Remission/Response	0	1527	0	1527			0	1527
TCGA-BB	TCGA-BB	HNSC	40	MALE	WHITE		Stage III	Head & N	GX	2010	-14810	Alive	TUMOR F	1368						Complete Remission/Response	0	1368	0	1368	0	1368	0	1368
TCGA-BB	TCGA-BB	HNSC	58	MALE	WHITE	Stage IVA	Stage IVA	Head & N	G3	2009	-21443	Alive	TUMOR F	2016						Complete Remission/Response	0	2016	0	2016			0	2016
TCGA-BB	TCGA-BB	HNSC	64	FEMALE	WHITE	Stage IVA	Stage IVA	Head & N	G2	2011	-23409	Alive	WITH TU	750		Distant M	Distant M	Pulmonar	428	Complete Remission/Response	0	750	0	750			1	428
TCGA-BB	TCGA-BB	HNSC	63	MALE	ASIAN	Stage I	Stage IVA	Head & N	G3	2011	-23185	Alive	TUMOR F	1168						Complete Remission/Response	0	1168	0	1168			0	1168
TCGA-BB	TCGA-BB	HNSC	69	FEMALE	WHITE	Stage IVA	Stage IVA	Head & N	G3	2005	-25408	Alive	TUMOR F	2161						Complete Remission/Response	0	2161	0	2161			0	2161
TCGA-BB	TCGA-BB	HNSC	84	MALE	BLACK OI	Stage III	Stage II	Head & N	G2	2010	-30721	Alive	WITH TU	624		Locoregic	Oral Cavity		440	Progressive Disease	0	624	0	624			1	440
TCGA-BB	TCGA-BB	HNSC	47	MALE	WHITE	Stage IVA	Stage IVA	Head & N	G2	2012	-17502	Alive	TUMOR F	782						Complete Remission/Response	0	782	0	782			0	782
TCGA-BB	TCGA-BB	HNSC	64	MALE	WHITE	Stage IVA	Stage IVA	Head & N	G3	2012	-23640	Dead	WITH TUMOR		321	Distant M	Distant M	Cerebellu	230	Complete Remission/Response	1	321	1	321			1	230
TCGA-BB	TCGA-BB	HNSC	65	MALE	WHITE	Stage IVA	Stage IVA	Head & N	G2	2012	-23906	Alive	WITH TU	827		Distant M	Distant M	Adrenal G	438	Complete Remission/Response	0	827	0	827			1	438
TCGA-BB	TCGA-BB	HNSC	52	MALE	WHITE	Stage III	Stage III	Head & N	GX	2010	-19194	Alive	TUMOR F	393						Complete Remission/Response	0	393	0	393	0	393	0	393
TCGA-BB	TCGA-BB	HNSC	61	FEMALE	WHITE	Stage IVA	Stage IVA	Head & N	G2	2012	-22506	Dead	WITH TUMOR		268		Distant M	Lung	212	Complete Remission/Response	1	268	1	268			1	212
TCGA-C9	TCGA-C9	HNSC	72	FEMALE	ASIAN	Stage III	Stage III	Head & N	G1	2011	-26592	Dead	TUMOR FREE		191					Complete Remission/Response	1	191	0	191	0	191	0	191
TCGA-C9	TCGA-C9	HNSC	45	FEMALE	ASIAN	Stage III	Stage III	Head & N	G1	2011	-16567	Alive	TUMOR F	386						Complete Remission/Response	0	386	0	386	0	386	0	386
TCGA-CN	TCGA-CN	HNSC	61	FEMALE	WHITE	Stage II	Stage II	Head & N	G1	2010	-22299	Alive	TUMOR F	1483						Complete Remission/Response	0	1483	0	1483	0	1483	0	1483
TCGA-CN	TCGA-CN	HNSC	67	MALE	WHITE	Stage IVA	Stage IVA	Head & N	G2	2010	-24815	Alive	TUMOR F	1699						Complete Remission/Response	0	1699	0	1699			0	1699
TCGA-CN	TCGA-CN	HNSC	60	MALE	WHITE	Stage II	Stage II	Head & N	G2	2010	-22171	Alive	TUMOR F	1157						Complete Remission/Response	0	1157	0	1157	0	1157	0	1157
TCGA-CN	TCGA-CN	HNSC	68	MALE	WHITE	Stage IVA	Stage IVA	Head & N	G2	2010	-25194	Dead	WITH TUMOR		142		Distant M	SKIN NEC	79	Progressive Disease	1	142	1	142			1	79
TCGA-CN	TCGA-CN	HNSC	56	MALE	WHITE	Stage IVA	Stage IVA	Head & N	G2	2010	-20748	Alive	TUMOR F	1560						Complete Remission/Response	0	1560	0	1560			0	1560
TCGA-CN	TCGA-CN	HNSC	56	MALE	WHITE	Stage IVA	Stage IVA	Head & N	G2	2010	-20660	Alive	TUMOR F	1724						Complete Remission/Response	0	1724	0	1724			0	1724
TCGA-CN	TCGA-CN	HNSC	73	MALE	WHITE	Stage III	Stage IVA	Head & N	G2	2010	-26713	Alive	TUMOR F	392						Complete Remission/Response	0	392	0	392			0	392
TCGA-CN	TCGA-CN	HNSC	62	MALE	WHITE	Stage IVA	Stage IVA	Head & N	G2	2010	-22828	Alive	TUMOR F	817						Complete Remission/Response	0	817	0	817			0	817
TCGA-CN	TCGA-CN	HNSC	63	FEMALE	WHITE	Stage IVA	Stage IVA	Head & N	G3	2010	-23194	Dead	WITH TUMOR		998		Oral Cavity		243	Persistent Disease	1	998	1	998			1	243
TCGA-CN	TCGA-CN	HNSC	61	MALE	WHITE	Stage III	Stage I	Head & N	G3	2010	-22561	Alive	TUMOR F	1586						Complete Remission/Response	0	1586	0	1586	0	1586	0	1586
TCGA-CN	TCGA-CN	HNSC	70	MALE	WHITE	Stage II	Stage II	Head & N	G2	2010	-25753	Alive	TUMOR F	1690						Complete Remission/Response	0	1690	0	1690	0	1690	0	1690
TCGA-CN	TCGA-CN	HNSC	52	MALE	WHITE	Stage IVA	Stage IVA	Head & N	G3	2010	-19107	Alive	TUMOR F	1737						Complete Remission/Response	0	1737	0	1737			0	1737
TCGA-CN	TCGA-CN	HNSC	70	FEMALE	WHITE		Stage II	Head & N	G2	2010	-25683	Dead	WITH TUMOR		395		Cervical L	R NECK	209	Complete Remission/Response	1	395	1	395	1	209	1	209
TCGA-CN	TCGA-CN	HNSC	19	MALE	WHITE	Stage IVA	Stage II	Head & N	G2	2010	-7301	Alive	TUMOR F	625						Complete Remission/Response	0	625	0	625			0	625
TCGA-CN	TCGA-CN	HNSC	53	MALE	WHITE	Stage IVA	Stage IVA	Head & N	G2	2009	-19621	Dead			436						1	436		436			0	436
TCGA-CN	TCGA-CN	HNSC	71	MALE	WHITE	Stage IVA	Stage IVA	Head & N	G2	2009	-26081	Dead	WITH TUMOR		1394	Locoregic	Orophary	OROPHAI	1302	Complete Remission/Response	1	1394	1	1394			1	1302
TCGA-CN	TCGA-CN	HNSC	79	FEMALE	WHITE	Stage IVA	Stage IVA	Head & N	G2	2009	-29186	Dead	WITH TUMOR		839			PTERYGO	224	Complete Remission/Response	1	839	1	839			1	224
TCGA-CN	TCGA-CN	HNSC	75	MALE	WHITE	Stage IVA	Stage IVA	Head & N	G2	2008	-27506	Alive	TUMOR F	2239		Locoregic	Cervical L	R NECK	1716	Complete Remission/Response	0	2239	0	2239			1	1716
TCGA-CN	TCGA-CN	HNSC	48	FEMALE	WHITE	Stage IVA	Stage IVA	Head & N	G3	2008	-17793	Dead	WITH TUMOR		397					Progressive Disease	1	397	1	397			1	397
TCGA-CN	TCGA-CN	HNSC	64	MALE	BLACK OI	Stage IVA	Stage IVA	Head & N	G2	2010	-23670	Alive	TUMOR F	1278						Complete Remission/Response	0	1278	0	1278			0	1278
TCGA-CN	TCGA-CN	HNSC	56	MALE	WHITE	Stage III	Stage III	Head & N	G2	2010	-20540	Alive	TUMOR F	1409						Complete Remission/Response	0	1409	0	1409	0	1409	0	1409
TCGA-CN	TCGA-CN	HNSC	60	MALE	WHITE	Stage II	Stage III	Head & N	G2	2010	-22138	Dead	TUMOR FREE		261		Cervical Lymph Node		145	Complete Remission/Response	1	261	0	261	1	145	1	145
TCGA-CN	TCGA-CN	HNSC	59	MALE	WHITE	Stage IVA	Stage IVA	Head & N	G2	2009	-21858	Dead	WITH TUMOR		377		Distant M	LUNG, BC	356	Complete Remission/Response	1	377	1	377			1	356
TCGA-CN	TCGA-CN	HNSC	68	MALE	WHITE	Stage IVA	Stage IVA	Head & N	G2	2008	-25034	Alive	TUMOR F	2169						Complete Remission/Response	0	2169	0	2169			0	2169
TCGA-CN	TCGA-CN	HNSC	80	MALE	WHITE	Stage IVA		Head & N	G2	2007	-29383	Dead	TUMOR FREE		2120	New Primary Tumor			2062	Complete Remission/Response	1	2120	0	2120			1	2062
TCGA-CN	TCGA-CN	HNSC	48	MALE	BLACK OI	Stage IVB	Stage IVB	Head & N	G3	2007	-17635	Dead	WITH TUMOR		253		Distant M	BONE	53	Complete Remission/Response	1	253	1	253			1	53
TCGA-CN	TCGA-CN	HNSC	55	MALE	WHITE	Stage IVA	Stage IVA	Head & N	G2	2006	-20362	Dead	TUMOR FREE		493					Complete Remission/Response	1	493	0	493			0	493
TCGA-CN	TCGA-CN	HNSC	38	MALE	WHITE	Stage IVB	Stage IVC	Head & N	G2	2006	-14190	Dead	WITH TUMOR		351					Progressive Disease	1	351	1	351			1	351
TCGA-CN	TCGA-CN	HNSC	51	MALE	WHITE	Stage IVA	Stage IVA	Head & N	G2	2006	-18904	Dead	WITH TUMOR		360		Cervical L	L NECK	276	Complete Remission/Response	1	360	1	360			1	276
TCGA-CN	TCGA-CN	HNSC	60	FEMALE	WHITE	Stage IVA	Stage IVA	Head & N	G2	2006	-22246	Dead	TUMOR FREE		352					Complete Remission/Response	1	352	0	352			0	352
TCGA-CN	TCGA-CN	HNSC	90	FEMALE	WHITE	Stage IVA	Stage IVA	Head & N	G3	2006	-32871	Dead	TUMOR FREE		380					Complete Remission/Response	1	380	0	380			0	380
TCGA-CN	TCGA-CN	HNSC	78	MALE	WHITE	Stage III	Stage II	Head & N	G3	2006	-28549	Dead	WITH TUMOR		259		Oral Cavity		123	Complete Remission/Response	1	259	1	259	1	123	1	123
TCGA-CN	TCGA-CN	HNSC	55	FEMALE	WHITE	Stage I	Stage II	Head & N	G1	2010	-20143	Alive	TUMOR F	1584						Complete Remission/Response	0	1584	0	1584	0	1584	0	1584
TCGA-CN	TCGA-CN	HNSC	56	FEMALE	WHITE	Stage IVA	Stage IVA	Head & N	G3	2007	-20464	Dead	TUMOR FREE		1732		Distant M	lung	1369	Complete Remission/Response	1	1732	0	1732			1	1369
TCGA-CN	TCGA-CN	HNSC	53	MALE	WHITE	Stage IVA	Stage IVA	Head & N	G2	2010	-19475	Alive	TUMOR F	1523		New Primary Tumor		nasal cavi	540	Complete Remission/Response	0	1523	0	1523			1	540
TCGA-CN	TCGA-CN	HNSC	57	MALE	WHITE	Stage IVA	Stage IVA	Head & N	G3	2010	-21016	Alive	TUMOR F	933						Complete Remission/Response	0	933	0	933			0	933
TCGA-CN	TCGA-CN	HNSC	66	MALE	WHITE	Stage IVA	Stage III	Head & N	G2	2010	-24286	Alive	TUMOR F	1460						Complete Remission/Response	0	1460	0	1460			0	1460
TCGA-CN	TCGA-CN	HNSC	56	MALE	WHITE	Stage IVA	Stage IVA	Head & N	G3	2010	-20703	Dead	WITH TUMOR		727		Distant M	LUNG	323	Complete Remission/Response	1	727	1	727			1	323
TCGA-CN	TCGA-CN	HNSC	64	MALE	WHITE	Stage IVA	Stage IVA	Head & N	G2	2010	-23664	Alive	TUMOR F	1443						Complete Remission/Response	0	1443	0	1443			0	1443
TCGA-CN	TCGA-CN	HNSC	55	MALE	WHITE	Stage IVA	Stage III	Head & N	G2	2010	-20358	Dead	TUMOR FREE		853					Complete Remission/Response	1	853	0	853			0	853
TCGA-CN	TCGA-CN	HNSC	85	FEMALE	WHITE	Stage IVA	Stage IVA	Head & N	G2	2011	-31242	Dead	TUMOR FREE		580					Complete Remission/Response	1	580	0	580			0	580
TCGA-CN	TCGA-CN	HNSC	61	MALE	WHITE	Stage IVA	Stage IVA	Head & N	G2	2011	-22485	Alive	TUMOR F	1038						Complete Remission/Response	0	1038	0	1038			0	1038
TCGA-CN	TCGA-CN	HNSC	58	MALE	BLACK OI	Stage III	Stage IVA	Head & N	G2	2011	-21232	Dead	TUMOR FREE		205					Complete Remission/Response	1	205	0	205			0	205
TCGA-CN	TCGA-CN	HNSC	63	FEMALE	WHITE		Stage III	Head & N	G2	2010	-23212	Dead	WITH TUMOR		276					Progressive Disease	1	276	1	276			1	276
TCGA-CN	TCGA-CN	HNSC	49	MALE	WHITE	Stage IVA	Stage IVA	Head & N	G3	2010	-17971	Dead	WITH TUMOR		281		Cervical Lymph Node		101	Progressive Disease	1	281	1	281			1	101
TCGA-CN	TCGA-CN	HNSC	73	MALE	WHITE	Stage IVA	Stage IVA	Head & N	GX	2010	-26769	Alive	TUMOR F	1584						Complete Remission/Response	0	1584	0	1584			0	1584
TCGA-CN	TCGA-CN	HNSC	66	MALE	WHITE	Stage IVA	Stage IVA	Head & N	G2	2010	-24259	Dead	WITH TUMOR		337		Cervical Lymph Node		156	Progressive Disease	1	337	1	337			1	156
TCGA-CN	TCGA-CN	HNSC	47	MALE	WHITE	Stage IVA	Stage IVA	Head & N	G3	2011	-17308	Alive	TUMOR F	318						Complete Remission/Response	0	318	0	318			0	318
TCGA-CN	TCGA-CN	HNSC	64	MALE	WHITE	Stage IVA	Stage IVA	Head & N	G2	2011	-23515	Dead	WITH TUMOR		980				436	Complete Remission/Response	1	980	1	980			1	436
TCGA-CN	TCGA-CN	HNSC	61	MALE	WHITE	Stage IVA	Stage IVA	Head & N	G2	2011	-22598	Alive	TUMOR F	1066						Complete Remission/Response	0	1066	0	1066			0	1066
TCGA-CN	TCGA-CN	HNSC	67	MALE	WHITE	Stage IVA	Stage IVA	Head & N	G2	2011	-24778	Alive	TUMOR F	1183						Complete Remission/Response	0	1183	0	1183			0	1183
TCGA-CN	TCGA-CN	HNSC	78	MALE	WHITE	Stage IVA	Stage IVA	Head & N	G2	2011	-28850	Dead	TUMOR FREE		112					Complete Remission/Response	1	112	0	112			0	112
TCGA-CN	TCGA-CN	HNSC	58	FEMALE	WHITE	Stage IVA	Stage IVA	Head & N	G2	2011	-21447	Dead	WITH TUMOR		530		Cervical L	SUPRASTI	350	Complete Remission/Response	1	530	1	530			1	350
TCGA-CN	TCGA-CN	HNSC	66	MALE	WHITE	Stage IVA	Stage IVA	Head & N	G3	2011	-24298	Dead	TUMOR FREE		988		Distant M	LUNG	279	Complete Remission/Response	1	988	0	988			1	279
TCGA-CN	TCGA-CN	HNSC	53	MALE	WHITE	Stage IVA	Stage IVA	Head & N	G2	2011	-19447	Dead	WITH TUMOR		357		Orophary	tonsil	244	Progressive Disease	1	357	1	357			1	244
TCGA-CN	TCGA-CN	HNSC	63	MALE	[Unknowr	Stage III	Stage IVA	Head & N	G2	2011	-23310	Alive	TUMOR F	1065						Complete Remission/Response	0	1065	0	1065			0	1065
TCGA-CN	TCGA-CN	HNSC	61	FEMALE	WHITE	Stage III	Stage II	Head & N	G1	2011	-22330	Dead	WITH TUMOR		773	Locoregic	Oral Cavit	tongue, r	186	Complete Remission/Response	1	773	1	773	1	186	1	186
TCGA-CN	TCGA-CN	HNSC	60	FEMALE	WHITE	Stage II	Stage I	Head & N	G2	2011	-21971	Alive	TUMOR F	717						Complete Remission/Response	0	717	0	717	0	717	0	717
TCGA-CN	TCGA-CN	HNSC	60	MALE	WHITE	Stage IVB	Stage IVA	Head & N	G2	2011	-21995	Dead	WITH TUMOR		526	Distant M	Distant M	spine	344	Complete Remission/Response	1	526	1	526			1	344
TCGA-CN	TCGA-CN	HNSC	71	MALE	WHITE	Stage III	Stage III	Head & N	G3	2011	-26155	Alive	TUMOR F	904						Complete Remission/Response	0	904	0	904	0	904	0	904
TCGA-CN	TCGA-CN	HNSC	67	MALE	WHITE		Stage IVA	Head & N	GX	2012	-24495	Alive	TUMOR F	645						Complete Remission/Response	0	645	0	645			0	645
TCGA-CN	TCGA-CN	HNSC	60	MALE	WHITE	Stage IVA	Stage IVA	Head & N	G3	2011	-22201	Alive	TUMOR F	225						Complete Remission/Response	0	225	0	225			0	225
TCGA-CN	TCGA-CN	HNSC	50	MALE	WHITE	Stage IVA	Stage III	Head & N	G3	2011	-18272	Alive	TUMOR F	964						Complete Remission/Response	0	964	0	964			0	964
TCGA-CN	TCGA-CN	HNSC	59	MALE	WHITE	Stage IVA	Stage IVA	Head & N	G2	2011	-21734	Alive	TUMOR F	679						Complete Remission/Response	0	679	0	679			0	679
TCGA-CN	TCGA-CN	HNSC	48	FEMALE	WHITE	Stage IVA	Stage IVA	Head & N	G2	2012	-17693	Dead	TUMOR FREE		377					Complete Remission/Response	1	377	0	377			0	377
TCGA-CN	TCGA-CN	HNSC	46	MALE	WHITE	Stage II	Stage I	Head & N	G2	2012	-17125	Alive	TUMOR F	504						Complete Remission/Response	0	504	0	504	0	504	0	504
TCGA-CN	TCGA-CN	HNSC	40	FEMALE	WHITE	Stage IVA	Stage IVA	Head & N	G2	2012	-14758	Dead	TUMOR FREE		134					Complete Remission/Response	1	134	0	134			0	134
TCGA-CN	TCGA-CN	HNSC	47	MALE	WHITE	Stage IVA	Stage IVA	Head & N	G2	2012	-17454	Alive	TUMOR F	367						Complete Remission/Response	0	367	0	367			0	367
TCGA-CN	TCGA-CN	HNSC	57	MALE	WHITE	Stage IVB	Stage IVA	Head & N	G3	2012	-21011	Dead	WITH TUMOR		82					Progressive Disease	1	82	1	82			1	82
TCGA-CN	TCGA-CN	HNSC	57	MALE	WHITE	Stage IVA	Stage IVA	Head & N	G2	2012	-20967	Alive	TUMOR F	713						Complete Remission/Response	0	713	0	713			0	713
TCGA-CN	TCGA-CN	HNSC	59	MALE	WHITE	Stage IVA	Stage IVA	Head & N	GX	2013	-21730	Alive	TUMOR F	603						Complete Remission/Response	0	603	0	603			0	603
TCGA-CN	TCGA-CN	HNSC	61	MALE	WHITE	Stage IVA	Stage IVA	Head & N	G3	2012	-22397	Alive	TUMOR F	742						Complete Remission/Response	0	742	0	742			0	742
TCGA-CN	TCGA-CN	HNSC	59	MALE	WHITE	Stage IVA	Stage IVA	Head & N	GX	2013	-21811	Alive	TUMOR F	635						Complete Remission/Response	0	635	0	635			0	635
TCGA-CN	TCGA-CN	HNSC	40	MALE	WHITE	Stage IVA	Stage IVA	Head & N	GX	2013	-14895	Alive	TUMOR F	594						Complete Remission/Response	0	594	0	594			0	594
TCGA-CC	TCGA-CC	HNSC	82	MALE	WHITE	Stage I	Stage III	Head & N	G2	2007	-30173	Alive	TUMOR F	1466						Complete Remission/Response	0	1466	0	1466	0	1466	0	1466
TCGA-CC	TCGA-CC	HNSC	59	MALE	WHITE	Stage III	Stage IVA	Head & N	G2	2007	-21803	Alive	TUMOR F	1593						Complete Remission/Response	0	1593	0	1593			0	1593
TCGA-CC	TCGA-CC	HNSC	65	MALE	WHITE	Stage I	Stage I	Head & N	G2	2007	-23833	Dead	WITH TUMOR		654		Oral Cavity		204	Complete Remission/Response	1	654	1	654	1	204	1	204
TCGA-CQ	TCGA-CQ	HNSC	67	MALE	WHITE	Stage IVA	Stage IVA	Head & N	G3	2007	-24473	Dead	WITH TUMOR		89					Progressive Disease	1	89	1	89			1	89
TCGA-CC	TCGA-CC	HNSC	61	FEMALE	ASIAN	Stage IVA	Stage IVA	Head & N	G2	2008	-22307	Alive	TUMOR F	1660						Complete Remission/Response	0	1660	0	1660			0	1660
TCGA-CC	TCGA-CC	HNSC	46	FEMALE	WHITE	Stage II	Stage III	Head & N	G2	2008	-17028	Alive	TUMOR F	2143						Complete Remission/Response	0	2143	0	2143	0	2143	0	2143
TCGA-CC	TCGA-CC	HNSC	69	FEMALE	WHITE	Stage IVA	Stage III	Head & N	G3	2008	-25255	Alive	TUMOR F	1897						Complete Remission/Response	0	1897	0	1897			0	1897
TCGA-CC	TCGA-CC	HNSC	73	FEMALE	WHITE		Stage IVA	Head & N	G2	2008	-26901	Alive	TUMOR F	1399						Complete Remission/Response	0	1399	0	1399			0	1399
TCGA-CC	TCGA-CC	HNSC	87	MALE	WHITE	Stage III	Stage III	Head & N	G2	2008	-32032	Dead		317	317						1	317		317			0	317
TCGA-CC	TCGA-CC	HNSC	74	MALE	WHITE	Stage II	Stage I	Head & N	G3	2008	-27110	Dead	WITH TUMOR		341		Oropharynx		176	Complete Remission/Response	1	341	1	341	1	176	1	176
TCGA-CQ	TCGA-CQ	HNSC	87	MALE	WHITE	Stage IVA	Stage IVB	Head & N	G2	2008	-32024	Dead	WITH TUMOR		129		Oral Cavity		50	Progressive Disease	1	129	1	129			1	50
TCGA-CC	TCGA-CC	HNSC	52	FEMALE	WHITE	Stage IVA	Stage III	Head & N	G2	2009	-19096	Alive	TUMOR F	1253						Complete Remission/Response	0	1253	0	1253			0	1253
TCGA-CQ	TCGA-CQ	HNSC	50	FEMALE	WHITE	Stage IVA	Stage IVA	Head & N	G2	2007	-18578	Dead	WITH TUMOR		479		Cervical Lymph Node		195	Complete Remission/Response	1	479	1	479			1	195
TCGA-CC	TCGA-CC	HNSC	69	MALE	WHITE	Stage III	Stage III	Head & N	G2	2007	-25366	Dead			985					Complete Remission/Response	1	985		985	0	985	0	985
TCGA-CC	TCGA-CC	HNSC	79	MALE	WHITE		Stage II	Head & N	G3	2008	-29026	Alive	TUMOR F	1000						Complete Remission/Response	0	1000	0	1000	0	1000	0	1000
TCGA-CC	TCGA-CC	HNSC	63	MALE	WHITE	Stage IVA	Stage IVA	Head & N	G2	2008	-23326	Alive	TUMOR F	2016						Complete Remission/Response	0	2016	0	2016			0	2016
TCGA-CC	TCGA-CC	HNSC	69	MALE	WHITE	Stage II	Stage IVA	Head & N	G2	2008	-25266	Alive	TUMOR F	1428						Complete Remission/Response	0	1428	0	1428			0	1428
TCGA-CC	TCGA-CC	HNSC	52	MALE	WHITE	Stage IVA	Stage II	Head & N	G3	2009	-19051	Alive	TUMOR F	1721						Complete Remission/Response	0	1721	0	1721			0	1721
TCGA-CC	TCGA-CC	HNSC	65	MALE	WHITE	Stage III	Stage II	Head & N	G2	2008	-23988	Dead			403		Oral Cavity		180	Persistent Disease	1	403		403			1	180
TCGA-CQ	TCGA-CQ	HNSC	77	MALE	WHITE	Stage IVA	Stage III	Head & N	G2	2008	-28466	Dead	WITH TUMOR		129					Progressive Disease	1	129	1	129			1	129
TCGA-CC	TCGA-CC	HNSC	71	FEMALE	WHITE	Stage IVA	Stage III	Head & N	G2	2009	-25942	Dead			456		Distant M	left axilla	273	Complete Remission/Response	1	456		456			1	273
TCGA-CC	TCGA-CC	HNSC	61	MALE	WHITE	Stage II	Stage III	Head & N	G2	2009	-22541	Alive	TUMOR F	1179						Complete Remission/Response	0	1179	0	1179	0	1179	0	1179
TCGA-CC	TCGA-CC	HNSC	59	FEMALE	WHITE	Stage I		Head & N	G1	2008	-21723	Alive	TUMOR F	2133		Locoregic	Oral Cavit	left oral tc	1160	Complete Remission/Response	0	2133	0	2133	1	1160	1	1160
TCGA-CC	TCGA-CC	HNSC	83	FEMALE	WHITE	Stage III	Stage III	Head & N	G2	2008	-30640	Alive	TUMOR F	1973						Complete Remission/Response	0	1973	0	1973	0	1973	0	1973
TCGA-CC	TCGA-CC	HNSC	40	MALE	ASIAN	Stage II	Stage II	Head & N	G2	2009	-14676	Alive	TUMOR F	1628			Oral Cavity		230	Complete Remission/Response	0	1628	0	1628	1	230	1	230
TCGA-CC	TCGA-CC	HNSC	75	FEMALE	WHITE	Stage I	Stage I	Head & N	G3	2009	-27707	Alive	TUMOR F	509						Complete Remission/Response	0	509	0	509	0	509	0	509
TCGA-CC	TCGA-CC	HNSC	80	FEMALE	WHITE	Stage II	Stage II	Head & N	G2	2010	-29530	Alive	TUMOR F	1309		New Prim	Oral Cavit	left bucca	1148	Complete Remission/Response	0	1309	0	1309	0	1309	1	1148
TCGA-CC	TCGA-CC	HNSC	77	FEMALE	WHITE	Stage II	Stage II	Head & N	G2	2010	-28138	Alive	TUMOR F	1274						Complete Remission/Response	0	1274	0	1274	0	1274	0	1274
TCGA-CQ	TCGA-CQ	HNSC	76	FEMALE	WHITE	Stage III	Stage IVA	Head & N	G2	2010	-27928	Alive	TUMOR F	1311						Complete Remission/Response	0	1311	0	1311			0	1311
TCGA-CC	TCGA-CC	HNSC	51	MALE	WHITE	Stage II	Stage IVA	Head & N	G3	2007	-18841	Alive	TUMOR F	2359						Complete Remission/Response	0	2359	0	2359			0	2359
TCGA-CC	TCGA-CC	HNSC	63	MALE	ASIAN	Stage II	Stage II	Head & N	G2	2010	-23205	Alive	TUMOR F	1353						Complete Remission/Response	0	1353	0	1353	0	1353	0	1353
TCGA-CC	TCGA-CC	HNSC	88	MALE	WHITE	Stage III	Stage II	Head & N	G3	2010	-32194	Dead			353		Oral Cavit	left lateral	207	Complete Remission/Response	1	353		353	1	207	1	207
TCGA-CC	TCGA-CC	HNSC	56	MALE	[Not Evalu	Stage III		Head & N	G2	2011	-20732	Alive	WITH TU	707		Locoregic	Oral Cavity		287	Complete Remission/Response	0	707	0	707	1	287	1	287
TCGA-CC	TCGA-CC	HNSC		MALE	[Not Evalu	Stage II	Stage II	Head & N	G2	2011		Alive	TUMOR FREE							Complete Remission/Response	0		0		0		0	
TCGA-CC	TCGA-CC	HNSC	59	MALE	[Not Evalu	Stage III	Stage II	Head & N	G2	2011	-21574	Alive	TUMOR F	893						Complete Remission/Response	0	893	0	893	0	893	0	893
TCGA-CC	TCGA-CC	HNSC	69	MALE	WHITE	Stage IVA	Stage IVB	Head & N	G3	2011	-25497	Alive	TUMOR F	1022						Complete Remission/Response	0	1022	0	1022			0	1022
TCGA-CC	TCGA-CC	HNSC	76	FEMALE	[Not Evalu	Stage II	Stage II	Head & N	G2	2011	-27793	Alive	TUMOR F	897						Complete Remission/Response	0	897	0	897	0	897	0	897
TCGA-CQ	TCGA-CQ	HNSC	78	FEMALE	WHITE	Stage III	Stage II	Head & N	G2	2011	-28637	Dead	WITH TUMOR		430		Distant M	new prim	350	Complete Remission/Response	1	430	1	430	1	350	1	350
TCGA-CC	TCGA-CC	HNSC	58	MALE	ASIAN	Stage II	Stage IVA	Head & N	G2	2012	-21455	Dead	WITH TUMOR		379			trachea	210	Complete Remission/Response	1	379	1	379			1	210
TCGA-CC	TCGA-CC	HNSC	73	MALE	WHITE	Stage III		Head & N	G3	2011	-26664	Alive	WITH TU	950			Distant M	lung	517	Complete Remission/Response	0	950	0	950	1	517	1	517
TCGA-CR	TCGA-CR	HNSC	51	MALE	WHITE		Stage IVA	Head & N	G3	2003	-18812	Alive	TUMOR F	2562						Complete Remission/Response	0	2562	0	2562			0	2562
TCGA-CR	TCGA-CR	HNSC	48	MALE	BLACK OR AFRICAN		Stage III	Head & N	G2	2006	-17810	Alive	TUMOR F	358						Complete Remission/Response	0	358	0	358	0	358	0	358
TCGA-CR	TCGA-CR	HNSC	53	MALE	WHITE		Stage IVA	Head & N	G3	2006	-19441	Alive	WITH TU	1663			Cervical Lymph Node		936	Complete Remission/Response	0	1663	0	1663			1	936
TCGA-CR	TCGA-CR	HNSC	35	FEMALE	WHITE	Stage II	Stage II	Head & N	GX	2007	-13047	Alive	TUMOR F	1152						Complete Remission/Response	0	1152	0	1152	0	1152	0	1152
TCGA-CR	TCGA-CR	HNSC	71	MALE	WHITE		Stage II	Head & N	G3	2009	-26097	Alive	TUMOR F	799						Complete Remission/Response	0	799	0	799	0	799	0	799
TCGA-CR	TCGA-CR	HNSC	59	MALE	WHITE	Stage III	Stage IVA	Head & N	G3	2006	-21689	Alive	TUMOR F	1777						Complete Remission/Response	0	1777	0	1777			0	1777
TCGA-CR	TCGA-CR	HNSC	38	MALE	WHITE	Stage II	Stage IVA	Head & N	G2	2007	-14133	Alive	TUMOR F	1521						Complete Remission/Response	0	1521	0	1521			0	1521
TCGA-CR	TCGA-CR	HNSC	58	MALE	WHITE	Stage IVA	Stage IVA	Head & N	G2	2007	-21315	Dead	WITH TUMOR		1202		Oropharynx		498	Complete Remission/Response	1	1202	1	1202			1	498
TCGA-CR	TCGA-CR	HNSC	59	MALE	WHITE		Stage IVB	Head & N	G3	2008	-21654	Alive	TUMOR F	1050						Complete Remission/Response	0	1050	0	1050			0	1050
TCGA-CR	TCGA-CR	HNSC	68	MALE	WHITE		Stage IVA	Head & N	G3	2008	-24857	Alive	TUMOR F	1125						Complete Remission/Response	0	1125	0	1125			0	1125
TCGA-CR	TCGA-CR	HNSC	51	MALE	WHITE	Stage IVA	Stage IVA	Head & N	G2	2009	-18840	Dead	WITH TUMOR		564		Cervical L	Right nec	220	Complete Remission/Response	1	564	1	564			1	220
TCGA-CR	TCGA-CR	HNSC	56	FEMALE	WHITE		Stage IVA	Head & N	G2	2010	-20815	Alive	TUMOR F	514						Complete Remission/Response	0	514	0	514			0	514
TCGA-CR	TCGA-CR	HNSC	66	FEMALE	WHITE		Stage IVA	Head & N	G3	2010	-24244	Dead			183					Complete Remission/Response	1	183		183			0	183
TCGA-CR	TCGA-CR	HNSC	53	MALE	WHITE		Stage IVA	Head & N	G2	2010	-19472	Alive	TUMOR F	362						Complete Remission/Response	0	362	0	362			0	362
TCGA-CR	TCGA-CR	HNSC	47	MALE	WHITE		Stage IVA	Head & N	G2	2010	-17203	Alive	TUMOR F	311						Complete Remission/Response	0	311	0	311			0	311
TCGA-CR	TCGA-CR	HNSC	62	MALE	BLACK OR AFRICAN		Stage IVA	Head & N	G3	2010	-22741	Alive	TUMOR F	345						Complete Remission/Response	0	345	0	345			0	345
TCGA-CR	TCGA-CR	HNSC	67	FEMALE	ASIAN	Stage IVA	Stage IVA	Head & N	G2	2010	-24569	Alive	TUMOR F	354						Complete Remission/Response	0	354	0	354			0	354
TCGA-CR	TCGA-CR	HNSC	50	MALE	WHITE	Stage I	Stage II	Head & N	G3	2010	-18331	Alive	TUMOR F	234						Complete Remission/Response	0	234	0	234	0	234	0	234
TCGA-CR	TCGA-CR	HNSC	68	FEMALE	WHITE	Stage II	Stage II	Head & N	G2	2010	-25176	Alive	TUMOR F	379						Complete Remission/Response	0	379	0	379	0	379	0	379
TCGA-CR	TCGA-CR	HNSC	60	MALE	BLACK OI	Stage IVA	Stage IVA	Head & N	G2	2010	-22125	Alive	WITH TU	693		New Prim	Distant M	Lung	499	Complete Remission/Response	0	693	0	693			1	499
TCGA-CR	TCGA-CR	HNSC	78	MALE	WHITE	Stage III	Stage IVA	Head & N	G3	2010	-28600	Alive	TUMOR F	479						Complete Remission/Response	0	479	0	479			0	479
TCGA-CR	TCGA-CR	HNSC	69	MALE	BLACK OI	Stage IVA	Stage IVA	Head & N	G2	2010	-25432	Dead	WITH TUMOR		282	Locoregic	Oral Cavit	Right ginc	247	Complete Remission/Response	1	282	1	282			1	247
TCGA-CR	TCGA-CR	HNSC	66	MALE	WHITE	Stage III	Stage III	Head & N	G2	2008	-24192	Alive	TUMOR F	1435						Complete Remission/Response	0	1435	0	1435	0	1435	0	1435
TCGA-CR	TCGA-CR	HNSC	60	MALE	WHITE	Stage IVA	Stage IVA	Head & N	G2	2008	-22101	Alive	TUMOR F	1191						Complete Remission/Response	0	1191	0	1191			0	1191
TCGA-CR	TCGA-CR	HNSC	52	MALE	WHITE	Stage IVA	Stage IVB	Head & N	G1	2008	-19116	Alive	TUMOR F	1440						Complete Remission/Response	0	1440	0	1440			0	1440
TCGA-CR	TCGA-CR	HNSC	54	MALE	WHITE	Stage IVA	Stage IVA	Head & N	G2	2008	-19876	Alive	TUMOR F	1245						Complete Remission/Response	0	1245	0	1245			0	1245
TCGA-CR	TCGA-CR	HNSC	59	MALE	WHITE	Stage IVA	Stage IVA	Head & N	G2	2008	-21897	Dead	TUMOR FREE		1090					Complete Remission/Response	1	1090	0	1090			0	1090
TCGA-CR	TCGA-CR	HNSC	72	FEMALE	WHITE		Stage II	Head & N	G2	2009	-26468	Alive	TUMOR F	105						Complete Remission/Response	0	105	0	105	0	105	0	105
TCGA-CR	TCGA-CR	HNSC	45	FEMALE	WHITE	Stage III	Stage III	Head & N	G2	2009	-16568	Dead			94						1	94		94			0	94
TCGA-CR	TCGA-CR	HNSC	45	MALE	WHITE	Stage I	Stage II	Head & N	G1	2009	-16610	Alive	TUMOR F	759						Complete Remission/Response	0	759	0	759	0	759	0	759
TCGA-CR	TCGA-CR	HNSC	66	MALE	WHITE	Stage IVA	Stage IVA	Head & N	G2	2009	-24448	Alive	TUMOR F	889						Complete Remission/Response	0	889	0	889			0	889
TCGA-CR	TCGA-CR	HNSC	67	FEMALE	WHITE		Stage II	Head & N	G2	2009	-24558	Alive	WITH TU	30							0	30	0	30			0	30
TCGA-CR	TCGA-CR	HNSC	83	MALE	WHITE	Stage III	Stage II	Head & N	G2	2009	-30670	Alive	TUMOR F	972						Complete Remission/Response	0	972	0	972	0	972	0	972
TCGA-CR	TCGA-CR	HNSC	58	MALE	WHITE	Stage IVA	Stage IVA	Head & N	G3	2008	-21371	Dead			279					Complete Remission/Response	1	279		279			0	279
TCGA-CR	TCGA-CR	HNSC	78	FEMALE	WHITE	Stage IVA	Stage IVA	Head & N	G2	2009	-28763	Alive	TUMOR F	1036						Complete Remission/Response	0	1036	0	1036			0	1036
TCGA-CR	TCGA-CR	HNSC	58	MALE	WHITE	Stage IVB	Stage III	Head & N	G2	2010	-21215	Dead	WITH TUMOR		606		Distant M	Lung	561	Complete Remission/Response	1	606	1	606			1	561
TCGA-CR	TCGA-CR	HNSC	49	MALE	WHITE	Stage IVA	Stage IVA	Head & N	G2	2008	-18078	Alive	TUMOR F	796			Oral Cavit	Tongue	614	Complete Remission/Response	0	796	0	796			1	614
TCGA-CR	TCGA-CR	HNSC	79	FEMALE	WHITE	Stage I	Stage I	Head & N	G2	2008	-29122	Dead	WITH TUMOR		521		Oropharynx		454	Complete Remission/Response	1	521	1	521	1	454	1	454
TCGA-CR	TCGA-CR	HNSC	42	MALE	WHITE		Stage IVA	Head & N	G2	2008	-15477	Alive	TUMOR F	997						Complete Remission/Response	0	997	0	997			0	997
TCGA-CR	TCGA-CR	HNSC	69	MALE	WHITE	Stage IVA	Stage IVA	Head & N	G1	2008	-25238	Alive	WITH TU	1430			Distant M	Lung (dist	1028	Complete Remission/Response	0	1430	0	1430			1	1028
TCGA-CR	TCGA-CR	HNSC	70	FEMALE	WHITE		Stage IVA	Head & N	G2	2008	-25728	Dead	WITH TUMOR		823		Cervical L	Right nec	334	Complete Remission/Response	1	823	1	823			1	334
TCGA-CR	TCGA-CR	HNSC	55	MALE	BLACK OR AFRICAN		Stage III	Head & N	G2	2007	-20169	Alive	TUMOR F	392						Complete Remission/Response	0	392	0	392	0	392	0	392
TCGA-CR	TCGA-CR	HNSC	67	MALE	WHITE	Stage IVA	Stage III	Head & N	G2	2009	-24575	Alive	TUMOR F	1508						Complete Remission/Response	0	1508	0	1508			0	1508
TCGA-CR	TCGA-CR	HNSC	36	FEMALE	WHITE	Stage I	Stage I	Head & N	G1	2009	-13508	Alive	TUMOR F	913						Complete Remission/Response	0	913	0	913	0	913	0	913
TCGA-CR	TCGA-CR	HNSC	67	FEMALE	WHITE	Stage IVA	Stage IVA	Head & N	G1	2009	-24650	Alive	TUMOR F	1425						Complete Remission/Response	0	1425	0	1425			0	1425
TCGA-CR	TCGA-CR	HNSC	26	MALE	WHITE	Stage III	Stage I	Head & N	G2	2009	-9750	Alive	TUMOR F	993						Complete Remission/Response	0	993	0	993	0	993	0	993
TCGA-CR	TCGA-CR	HNSC	70	MALE	WHITE	Stage IVA	Stage IVA	Head & N	G2	2009	-25581	Alive	TUMOR F	1346						Complete Remission/Response	0	1346	0	1346			0	1346
TCGA-CR	TCGA-CR	HNSC	80	FEMALE	WHITE	Stage II	Stage II	Head & N	G2	2009	-29527	Alive	TUMOR F	930						Complete Remission/Response	0	930	0	930	0	930	0	930
TCGA-CR	TCGA-CR	HNSC	44	MALE	WHITE	Stage IVA	Stage IVA	Head & N	G2	2011	-16351	Alive	TUMOR F	754						Complete Remission/Response	0	754	0	754			0	754
TCGA-CR	TCGA-CR	HNSC	53	FEMALE	WHITE	Stage II	Stage II	Head & N	G2	2011	-19468	Alive	TUMOR F	156						Complete Remission/Response	0	156	0	156	0	156	0	156
TCGA-CR	TCGA-CR	HNSC	60	FEMALE	WHITE	Stage IVA	Stage IVA	Head & N	G3	2008	-22182	Alive	TUMOR F	181						Complete Remission/Response	0	181	0	181			0	181
TCGA-CR	TCGA-CR	HNSC	64	MALE	WHITE	Stage I	Stage I	Head & N	G2	2008	-23420	Alive	TUMOR F	1077						Complete Remission/Response	0	1077	0	1077	0	1077	0	1077
TCGA-CR	TCGA-CR	HNSC	68	MALE	WHITE		Stage III	Head & N	G2	2009	-25138	Alive	TUMOR F	911						Complete Remission/Response	0	911	0	911	0	911	0	911
TCGA-CR	TCGA-CR	HNSC	53	MALE	WHITE		Stage IVA	Head & N	G2	2008	-19532	Alive	WITH TU	1472			Oropharynx		234	Complete Remission/Response	0	1472	0	1472			1	234
TCGA-CV	TCGA-CV	HNSC	61	MALE	WHITE	Stage IVA	Stage IVA	Head & N	G3	1999	-22577	Alive	TUMOR F	4241			Distant M	Lung	783	Complete Remission/Response	0	4241	0	4241			1	783
TCGA-CV	TCGA-CV	HNSC	61	MALE	WHITE	Stage IVA	Stage IVA	Head & N	G3	1999	-22577	Alive	TUMOR F	4241			Distant M	Lung	783	Complete Remission/Response	0	4241	0	4241			1	783
TCGA-CV	TCGA-CV	HNSC	73	MALE	WHITE	Stage IVA	Stage IVA	Head & N	G3	2000	-26953	Dead	TUMOR F	522	522					Complete Remission/Response	1	522	0	522			0	522
TCGA-CV	TCGA-CV	HNSC	73	MALE	WHITE	Stage IVA	Stage IVA	Head & N	G3	2000	-26953	Dead	TUMOR F	522	522					Complete Remission/Response	1	522	0	522			0	522
TCGA-CV	TCGA-CV	HNSC	68	MALE	WHITE	Stage III	Stage III	Head & N	G3	2000	-25006	Alive	TUMOR F	3930							0	3930	0	3930			0	3930
TCGA-CV	TCGA-CV	HNSC	68	MALE	WHITE	Stage III	Stage III	Head & N	G3	2000	-25006	Alive	TUMOR F	3930							0	3930	0	3930			0	3930
TCGA-CV	TCGA-CV	HNSC	60	MALE	BLACK OI	Stage IVA	Stage IVA	Head & N	G2	2000	-22182	Dead	TUMOR F	3295	3314		Distant M	Lung	1859	Complete Remission/Response	1	3314	0	3314			1	1859
TCGA-CV	TCGA-CV	HNSC	60	MALE	BLACK OI	Stage IVA	Stage IVA	Head & N	G2	2000	-22182	Dead	TUMOR F	3295	3314		Distant M	Lung	1859	Complete Remission/Response	1	3314	0	3314			1	1859
TCGA-CV	TCGA-CV	HNSC	57	MALE	WHITE	Stage IVA	Stage IVA	Head & N	G3	2000	-20926	Dead	WITH TU	1438	2319		Distant M	Lung	284		1	2319	1	2319			1	284
TCGA-CV	TCGA-CV	HNSC	57	MALE	WHITE	Stage IVA	Stage IVA	Head & N	G3	2000	-20926	Dead	WITH TU	1438	2319		Distant M	Lung	284		1	2319	1	2319			1	284
TCGA-CV	TCGA-CV	HNSC	65	MALE		Stage IVA	Stage IVA	Head & N	G2	2001	-23923	Dead	WITH TU	548	584					Persistent Disease	1	584	1	584			1	584
TCGA-CV	TCGA-CV	HNSC	65	MALE		Stage IVA	Stage IVA	Head & N	G2	2001	-23923	Dead	WITH TU	548	584					Persistent Disease	1	584	1	584			1	584
TCGA-CV	TCGA-CV	HNSC	62	MALE	WHITE	Stage IVA	Stage II	Head & N	G2	2002	-22686	Dead	WITH TU	546	546		Oropharynx		211		1	546	1	546			1	211
TCGA-CV	TCGA-CV	HNSC	62	MALE	WHITE	Stage IVA	Stage II	Head & N	G2	2002	-22686	Dead	WITH TU	546	546		Oropharynx		211		1	546	1	546			1	211
TCGA-CV	TCGA-CV	HNSC	52	MALE	WHITE	Stage IVA	Stage IVA	Head & N	G3	2002	-19197	Alive	TUMOR F	3270						Complete Remission/Response	0	3270	0	3270			0	3270
TCGA-CV	TCGA-CV	HNSC	52	MALE	WHITE	Stage IVA	Stage IVA	Head & N	G3	2002	-19197	Alive	TUMOR F	3270						Complete Remission/Response	0	3270	0	3270			0	3270
TCGA-CV	TCGA-CV	HNSC	58	MALE	WHITE	Stage IVA	Stage IVA	Head & N	G3	2003	-21305	Alive	TUMOR F	2886						Complete Remission/Response	0	2886	0	2886			0	2886
TCGA-CV	TCGA-CV	HNSC	58	MALE	WHITE	Stage IVA	Stage IVA	Head & N	G3	2003	-21305	Alive	TUMOR F	2886						Complete Remission/Response	0	2886	0	2886			0	2886
TCGA-CV	TCGA-CV	HNSC	76	FEMALE	WHITE	Stage IVA	Stage IVA	Head & N	G3	2004	-28040	Alive	TUMOR F	2327						Complete Remission/Response	0	2327	0	2327			0	2327
TCGA-CV	TCGA-CV	HNSC	76	FEMALE	WHITE	Stage IVA	Stage IVA	Head & N	G3	2004	-28040	Alive	TUMOR F	2327						Complete Remission/Response	0	2327	0	2327			0	2327

TCGA-CV	TCGA-CV	HNSC	63	MALE	BLACK OI	Stage III	Stage III	Head & N	G3	2004	-23287	Alive	TUMOR F	2784							0	2784	0	2784			0	2784
TCGA-CV	TCGA-CV	HNSC	63	MALE	BLACK OI	Stage III	Stage III	Head & N	G3	2004	-23287	Alive	TUMOR F	2784							0	2784	0	2784			0	2784
TCGA-CV	TCGA-CV	HNSC	64	MALE	BLACK OI	Stage IVA	Stage IVA	Head & N	G3	2004	-23650	Alive	TUMOR F	2437							0	2437	0	2437			0	2437
TCGA-CV	TCGA-CV	HNSC	64	MALE	BLACK OI	Stage IVA	Stage IVA	Head & N	G3	2004	-23650	Alive	TUMOR F	2437							0	2437	0	2437			0	2437
TCGA-CV	TCGA-CV	HNSC	63	FEMALE	WHITE	Stage IVA	Stage IVA	Head & N	G3	2005	-23119	Dead	TUMOR FREE		545					Complete Remission/Response	1	545	0	545			0	545
TCGA-CV	TCGA-CV	HNSC	63	FEMALE	WHITE	Stage IVA	Stage IVA	Head & N	G3	2005	-23119	Dead	TUMOR FREE		545					Complete Remission/Response	1	545	0	545			0	545
TCGA-CV	TCGA-CV	HNSC	59	MALE	WHITE	Stage IVA	Stage IVA	Head & N	G2	2009	-21890	Dead	WITH TUI	406	406						1	406	1	406			1	406
TCGA-CV	TCGA-CV	HNSC	59	MALE	WHITE	Stage IVA	Stage IVA	Head & N	G2	2009	-21890	Dead	WITH TUI	406	406						1	406	1	406			1	406
TCGA-CV	TCGA-CV	HNSC	60	MALE	WHITE	Stage IVA	Stage IVA	Head & N	G2	2009	-22279	Alive	TUMOR F	701							0	701	0	701			0	701
TCGA-CV	TCGA-CV	HNSC	60	MALE	WHITE	Stage IVA	Stage IVA	Head & N	G2	2009	-22279	Alive	TUMOR F	701							0	701	0	701			0	701
TCGA-CV	TCGA-CV	HNSC	62	FEMALE	WHITE	Stage III	Stage III	Head & N	G3	2005	-22771	Alive	TUMOR F	2641						Complete Remission/Response	0	2641	0	2641	0	2641	0	2641
TCGA-CV	TCGA-CV	HNSC	62	FEMALE	WHITE	Stage III	Stage III	Head & N	G3	2005	-22771	Alive	TUMOR F	2641						Complete Remission/Response	0	2641	0	2641	0	2641	0	2641
TCGA-CV	TCGA-CV	HNSC	50	MALE	WHITE	Stage IVA	Stage III	Head & N	G2	2006	-18267	Alive	TUMOR F	1478							0	1478	0	1478			0	1478
TCGA-CV	TCGA-CV	HNSC	50	MALE	WHITE	Stage IVA	Stage III	Head & N	G2	2006	-18267	Alive	TUMOR F	1478							0	1478	0	1478			0	1478
TCGA-CV	TCGA-CV	HNSC	66	MALE	WHITE	Stage IVA	Stage III	Head & N	G2	2007	-24115	Alive	TUMOR F	1840						Complete Remission/Response	0	1840	0	1840			0	1840
TCGA-CV	TCGA-CV	HNSC	66	MALE	WHITE	Stage IVA	Stage III	Head & N	G2	2007	-24115	Alive	TUMOR F	1840						Complete Remission/Response	0	1840	0	1840			0	1840
TCGA-CV	TCGA-CV	HNSC	53	FEMALE	BLACK OI	Stage IVB	Stage IVB	Head & N	G2	2008	-19480	Dead	WITH TUI	215	215						1	215	1	215			1	215
TCGA-CV	TCGA-CV	HNSC	53	FEMALE	BLACK OI	Stage IVB	Stage IVB	Head & N	G2	2008	-19480	Dead	WITH TUI	215	215						1	215	1	215			1	215
TCGA-CV	TCGA-CV	HNSC	26	MALE	WHITE	Stage IVA	Stage III	Head & N	G2	2007	-9567	Alive	TUMOR F	1315						Complete Remission/Response	0	1315	0	1315			0	1315
TCGA-CV	TCGA-CV	HNSC	26	MALE	WHITE	Stage IVA	Stage III	Head & N	G2	2007	-9567	Alive	TUMOR F	1315						Complete Remission/Response	0	1315	0	1315			0	1315
TCGA-CV	TCGA-CV	HNSC	50	FEMALE	WHITE	Stage III	Stage II	Head & N	G2	2007	-18600	Alive	TUMOR F	1665							0	1665	0	1665			0	1665
TCGA-CV	TCGA-CV	HNSC	50	FEMALE	WHITE	Stage III	Stage II	Head & N	G2	2007	-18600	Alive	TUMOR F	1665							0	1665	0	1665			0	1665
TCGA-CV	TCGA-CV	HNSC	57	MALE	WHITE	Stage II	Stage II	Head & N	G3	2010	-20855	Alive	WITH TUI	641						Complete Remission/Response	0	641	0	641	0	641	0	641
TCGA-CV	TCGA-CV	HNSC	57	MALE	WHITE	Stage II	Stage II	Head & N	G3	2010	-20855	Alive	WITH TUI	641						Complete Remission/Response	0	641	0	641	0	641	0	641
TCGA-CV	TCGA-CV	HNSC	62	MALE	WHITE	Stage IVA	Stage III	Head & N	G1	2007	-22885	Alive	TUMOR F	1899							0	1899	0	1899			0	1899
TCGA-CV	TCGA-CV	HNSC	62	MALE	WHITE	Stage IVA	Stage III	Head & N	G1	2007	-22885	Alive	TUMOR F	1899							0	1899	0	1899			0	1899
TCGA-CV	TCGA-CV	HNSC	60	MALE	WHITE	Stage III	Stage III	Head & N	G3	2010	-22025	Dead	WITH TUMOR		292	Locoregic	Oral Cavit	pharynge	95	Progressive Disease	1	292	1	292			1	95
TCGA-CV	TCGA-CV	HNSC	60	MALE	WHITE	Stage III	Stage III	Head & N	G3	2010	-22025	Dead	WITH TUMOR		292	Locoregic	Oral Cavit	pharynge	95	Progressive Disease	1	292	1	292			1	95
TCGA-CV	TCGA-CV	HNSC	53	MALE	WHITE	Stage III	Stage IVA	Head & N	G2	1998	-19518	Dead	TUMOR FREE		2741						1	2741	0	2741			0	2741
TCGA-CV	TCGA-CV	HNSC	53	MALE	WHITE	Stage III	Stage IVA	Head & N	G2	1998	-19518	Dead	TUMOR FREE		2741						1	2741	0	2741			0	2741
TCGA-CV	TCGA-CV	HNSC	66	FEMALE	WHITE	Stage IVA	Stage IVA	Head & N	G2	1998	-24180	Dead	WITH TUMOR		65					Complete Remission/Response	1	65	1	65			1	65
TCGA-CV	TCGA-CV	HNSC	66	FEMALE	WHITE	Stage IVA	Stage IVA	Head & N	G2	1998	-24180	Dead	WITH TUMOR		65					Complete Remission/Response	1	65	1	65			1	65
TCGA-CV	TCGA-CV	HNSC	67	MALE	WHITE	Stage IVA	Stage III	Head & N	G3	1999	-24799	Dead	WITH TUMOR		295						1	295	1	295			1	295
TCGA-CV	TCGA-CV	HNSC	67	MALE	WHITE	Stage IVA	Stage III	Head & N	G3	1999	-24799	Dead	WITH TUMOR		295						1	295	1	295			1	295
TCGA-CV	TCGA-CV	HNSC	68	MALE	WHITE	Stage IVA	Stage IVA	Head & N	G2	2004	-25020	Dead	WITH TUMOR		166	Locoregic	Oral Cavit	mandibul	157	Progressive Disease	1	166	1	166			1	157
TCGA-CV	TCGA-CV	HNSC	68	MALE	WHITE	Stage IVA	Stage IVA	Head & N	G2	2004	-25020	Dead	WITH TUMOR		166	Locoregic	Oral Cavit	mandibul	157	Progressive Disease	1	166	1	166			1	157
TCGA-CV	TCGA-CV	HNSC	71	MALE	WHITE	Stage II	Stage II	Head & N	G2	2003	-26156	Dead	TUMOR FREE		624						1	624	0	624			0	624
TCGA-CV	TCGA-CV	HNSC	87	MALE	WHITE	Stage II	Stage II	Head & N	G1	1996	-31825	Dead	TUMOR FREE		144						1	144	0	144			0	144
TCGA-CV	TCGA-CV	HNSC	87	MALE	WHITE	Stage II	Stage II	Head & N	G1	1996	-31825	Dead	TUMOR FREE		144						1	144	0	144			0	144
TCGA-CV	TCGA-CV	HNSC	60	MALE	WHITE	Stage IVA	Stage IVA	Head & N	G3	1994	-21947	Dead	WITH TUMOR		666						1	666	1	666			1	666
TCGA-CV	TCGA-CV	HNSC	60	MALE	WHITE	Stage IVA	Stage IVA	Head & N	G3	1994	-21947	Dead	WITH TUMOR		666						1	666	1	666			1	666
TCGA-CV	TCGA-CV	HNSC	80	FEMALE	WHITE	Stage III	Stage I	Head & N	G2	2002	-29382	Dead	TUMOR FREE		804					Complete Remission/Response	1	804	0	804	0	804	0	804
TCGA-CV	TCGA-CV	HNSC	51	MALE	WHITE	Stage III	Stage III	Head & N	G2	1999	-18812	Dead	TUMOR FREE		342					Complete Remission/Response	1	342	0	342	0	342	0	342
TCGA-CV	TCGA-CV	HNSC	73	FEMALE	WHITE	Stage II	Stage II	Head & N	G3	2000	-26941	Alive	TUMOR F	4282						Complete Remission/Response	0	4282	0	4282	0	4282	0	4282
TCGA-CV	TCGA-CV	HNSC	74	MALE	WHITE	Stage III	Stage III	Head & N	G2	1999	-27310	Dead	WITH TUMOR		602						1	602	1	602			1	602
TCGA-CV	TCGA-CV	HNSC	74	MALE	WHITE	Stage III	Stage III	Head & N	G2	1999	-27310	Dead	WITH TUMOR		602						1	602	1	602			1	602
TCGA-CV	TCGA-CV	HNSC	41	MALE	WHITE	Stage IVA	Stage IVA	Head & N	G2	1993	-15158	Dead	WITH TUMOR		366						1	366	1	366			1	366
TCGA-CV	TCGA-CV	HNSC	79	FEMALE	WHITE	Stage IVB	Stage IVA	Head & N	G2	1994	-29153	Dead	TUMOR FREE		1289						1	1289	0	1289			0	1289
TCGA-CV	TCGA-CV	HNSC	64	MALE	WHITE	Stage IVA	Stage IVA	Head & N	G2	1994	-23549	Dead	WITH TUMOR		459	Locoregic	Oral Cavit	Tonsil	258	Complete Remission/Response	1	459	1	459			1	258
TCGA-CV	TCGA-CV	HNSC	57	MALE	WHITE	Stage IVA	Stage IVA	Head & N	G2	1996	-21166	Dead	WITH TUMOR		915						1	915	1	915			1	915
TCGA-CV	TCGA-CV	HNSC	57	MALE	WHITE	Stage IVA	Stage IVA	Head & N	G2	1996	-21166	Dead	WITH TUMOR		915						1	915	1	915			1	915
TCGA-CV	TCGA-CV	HNSC	65	FEMALE	WHITE	Stage IVA	Stage III	Head & N	G1	1996	-23892	Dead	TUMOR FREE		185						1	185	0	185			0	185
TCGA-CV	TCGA-CV	HNSC	65	FEMALE	WHITE	Stage IVA	Stage III	Head & N	G1	1996	-23892	Dead	TUMOR FREE		185						1	185	0	185			0	185
TCGA-CV	TCGA-CV	HNSC	80	FEMALE	WHITE	Stage III	Stage III	Head & N	G1	1997	-29465	Dead	WITH TUMOR		1641	Locoregic	Oral Cavit	oral tongu	1521	Complete Remission/Response	1	1641	1	1641	1	1521	1	1521
TCGA-CV	TCGA-CV	HNSC	80	FEMALE	WHITE	Stage III	Stage III	Head & N	G1	1997	-29465	Dead	WITH TUMOR		1641	Locoregic	Oral Cavit	oral tongu	1521	Complete Remission/Response	1	1641	1	1641	1	1521	1	1521
TCGA-CV	TCGA-CV	HNSC	59	MALE	BLACK OI	Stage IVA	Stage IVA	Head & N	G2	1998	-21557	Dead	TUMOR FREE		2002					Complete Remission/Response	1	2002	0	2002			0	2002
TCGA-CV	TCGA-CV	HNSC	59	MALE	BLACK OI	Stage IVA	Stage IVA	Head & N	G2	1998	-21557	Dead	TUMOR FREE		2002					Complete Remission/Response	1	2002	0	2002			0	2002
TCGA-CV	TCGA-CV	HNSC	74	FEMALE	WHITE	Stage II	Stage II	Head & N	G3	2000	-27261	Dead	WITH TUMOR		334						1	334	1	334			1	334
TCGA-CV	TCGA-CV	HNSC	74	FEMALE	WHITE	Stage II	Stage II	Head & N	G3	2000	-27261	Dead	WITH TUMOR		334						1	334	1	334			1	334
TCGA-CV	TCGA-CV	HNSC	67	MALE	WHITE	Stage III	Stage IVA	Head & N	G2	1993	-24720	Dead	WITH TUMOR		217					Complete Remission/Response	1	217	1	217			1	217
TCGA-CV	TCGA-CV	HNSC	67	MALE	WHITE	Stage III	Stage IVA	Head & N	G2	1993	-24720	Dead	WITH TUMOR		217					Complete Remission/Response	1	217	1	217			1	217
TCGA-CV	TCGA-CV	HNSC	48	MALE	WHITE	Stage III	Stage III	Head & N	G2	1999	-17679	Dead	WITH TUMOR		256	Locoregional Recurre	Rt. Neck r		103		1	256	1	256			1	103
TCGA-CV	TCGA-CV	HNSC	48	MALE	WHITE	Stage III	Stage III	Head & N	G2	1999	-17679	Dead	WITH TUMOR		256	Locoregional Recurre	Rt. Neck r		103		1	256	1	256			1	103
TCGA-CV	TCGA-CV	HNSC	49	MALE	BLACK OI	Stage III	Stage III	Head & N	G3	1999	-18194	Dead	TUMOR FREE		862						1	862	0	862			0	862
TCGA-CV	TCGA-CV	HNSC	49	MALE	BLACK OI	Stage III	Stage III	Head & N	G3	1999	-18194	Dead	TUMOR FREE		862						1	862	0	862			0	862
TCGA-CV	TCGA-CV	HNSC	61	MALE	WHITE	Stage II	Stage II	Head & N	G3	1999	-22532	Dead	WITH TUMOR		76					Complete Remission/Response	1	76	1	76			1	76
TCGA-CV	TCGA-CV	HNSC	61	MALE	WHITE	Stage II	Stage II	Head & N	G3	1999	-22532	Dead	WITH TUMOR		76					Complete Remission/Response	1	76	1	76			1	76
TCGA-CV	TCGA-CV	HNSC	65	MALE	WHITE	Stage IVA	Stage III	Head & N	G2	1999	-24103	Dead	TUMOR FREE		126						1	126	0	126			0	126
TCGA-CV	TCGA-CV	HNSC	65	MALE	WHITE	Stage IVA	Stage III	Head & N	G2	1999	-24103	Dead	TUMOR FREE		126						1	126	0	126			0	126
TCGA-CV	TCGA-CV	HNSC	74	MALE	WHITE	Stage IVA	Stage IVA	Head & N	G2	2001	-27149	Dead	TUMOR FREE		1972						1	1972	0	1972			0	1972
TCGA-CV	TCGA-CV	HNSC	74	MALE	WHITE	Stage IVA	Stage IVA	Head & N	G2	2001	-27149	Dead	TUMOR FREE		1972						1	1972	0	1972			0	1972
TCGA-CV	TCGA-CV	HNSC	39	MALE	WHITE	Stage II	Stage II	Head & N	G2	2001	-14515	Alive	TUMOR F	5252						Complete Remission/Response	0	5252	0	5252	0	5252	0	5252
TCGA-CV	TCGA-CV	HNSC	54	MALE	WHITE	Stage I	Stage I	Head & N	G3	2001	-19938	Alive	TUMOR F	3381							0	3381	0	3381			0	3381
TCGA-CV	TCGA-CV	HNSC	54	MALE	WHITE	Stage I	Stage I	Head & N	G3	2001	-19938	Alive	TUMOR F	3381							0	3381	0	3381			0	3381
TCGA-CV	TCGA-CV	HNSC	87	FEMALE		Stage II	Stage IVA	Head & N	G2	2001	-32022	Dead	TUMOR FREE		572						1	572	0	572			0	572
TCGA-CV	TCGA-CV	HNSC	53	MALE	WHITE	Stage II	Stage III	Head & N	G3	2002	-19382	Dead	WITH TUMOR		385						1	385	1	385			1	385
TCGA-CV	TCGA-CV	HNSC	53	MALE	WHITE	Stage II	Stage III	Head & N	G3	2002	-19382	Dead	WITH TUMOR		385						1	385	1	385			1	385
TCGA-CV	TCGA-CV	HNSC	85	FEMALE	WHITE	Stage II	Stage II	Head & N	G2	2002	-31134	Dead	TUMOR FREE		243					Complete Remission/Response	1	243	0	243	0	243	0	243
TCGA-CV	TCGA-CV	HNSC	66	MALE	WHITE	Stage III	Stage II	Head & N	G3	2001	-24183	Dead	WITH TUMOR		274						1	274	1	274			1	274
TCGA-CV	TCGA-CV	HNSC	80	MALE	WHITE	Stage II	Stage II	Head & N	G2	1999	-29550	Dead	TUMOR FREE		160						1	160	0	160			0	160
TCGA-CV	TCGA-CV	HNSC	80	MALE	WHITE	Stage II	Stage II	Head & N	G2	1999	-29550	Dead	TUMOR FREE		160						1	160	0	160			0	160
TCGA-CV	TCGA-CV	HNSC	76	FEMALE	WHITE	Stage IVA	Stage III	Head & N	G3	2000	-27805	Dead			56					Complete Remission/Response	1	56		56			0	56
TCGA-CV	TCGA-CV	HNSC	49	MALE	WHITE	Stage IVA	Stage II	Head & N	G2	2000	-18219	Dead	WITH TUMOR		1591						1	1591	1	1591			1	1591
TCGA-CV	TCGA-CV	HNSC	49	MALE	WHITE	Stage IVA	Stage II	Head & N	G2	2000	-18219	Dead	WITH TUMOR		1591						1	1591	1	1591			1	1591
TCGA-CV	TCGA-CV	HNSC	61	FEMALE	WHITE	Stage IVA	Stage IVA	Head & N	G2	2000	-22349	Dead	TUMOR FREE		393					Complete Remission/Response	1	393	0	393			0	393
TCGA-CV	TCGA-CV	HNSC	82	FEMALE	WHITE	Stage II	Stage I	Head & N	G2	1999	-30180	Dead	WITH TUMOR		663						1	663	1	663			1	663
TCGA-CV	TCGA-CV	HNSC	82	FEMALE	WHITE	Stage II	Stage I	Head & N	G2	1999	-30180	Dead	WITH TUMOR		663						1	663	1	663			1	663
TCGA-CV	TCGA-CV	HNSC	64	FEMALE	WHITE	Stage IVA	Stage IVA	Head & N	G3	2000	-23430	Dead	WITH TUMOR		2166						1	2166	1	2166			1	2166
TCGA-CV	TCGA-CV	HNSC	64	FEMALE	WHITE	Stage IVA	Stage IVA	Head & N	G3	2000	-23430	Dead	WITH TUMOR		2166						1	2166	1	2166			1	2166
TCGA-CV	TCGA-CV	HNSC	34	MALE	WHITE	Stage II	Stage II	Head & N	G2	1996	-12523	Dead	WITH TUMOR		327						1	327	1	327			1	327
TCGA-CV	TCGA-CV	HNSC	53	MALE	WHITE	Stage II	Stage II	Head & N	G1	2002	-19358	Alive	TUMOR F	3981						Complete Remission/Response	0	3981	0	3981	0	3981	0	3981
TCGA-CV	TCGA-CV	HNSC	53	MALE	WHITE	Stage II	Stage II	Head & N	G1	2002	-19358	Alive	TUMOR F	3981						Complete Remission/Response	0	3981	0	3981	0	3981	0	3981
TCGA-CV	TCGA-CV	HNSC	67	MALE	WHITE	Stage II	Stage III	Head & N	G3	2007	-24800	Alive	TUMOR F	2347						Complete Remission/Response	0	2347	0	2347	0	2347	0	2347
TCGA-CV	TCGA-CV	HNSC	67	MALE	WHITE	Stage II	Stage III	Head & N	G3	2007	-24800	Alive	TUMOR F	2347						Complete Remission/Response	0	2347	0	2347	0	2347	0	2347
TCGA-CV	TCGA-CV	HNSC	77	FEMALE	WHITE	Stage IVA	Stage IVA	Head & N	G3	2007	-28250	Dead	TUMOR FREE		144						1	144	0	144			0	144
TCGA-CV	TCGA-CV	HNSC	69	FEMALE	WHITE	Stage II	Stage II	Head & N	G2	2007	-25471	Alive	TUMOR F	2727						Complete Remission/Response	0	2727	0	2727	0	2727	0	2727
TCGA-CV	TCGA-CV	HNSC	69	FEMALE	WHITE	Stage II	Stage II	Head & N	G2	2007	-25471	Alive	TUMOR F	2727						Complete Remission/Response	0	2727	0	2727	0	2727	0	2727
TCGA-CV	TCGA-CV	HNSC	60	FEMALE	WHITE	Stage II	Stage III	Head & N	G2	2008	-22136	Alive	TUMOR F	1095							0	1095	0	1095			0	1095
TCGA-CV	TCGA-CV	HNSC	60	FEMALE	WHITE	Stage II	Stage III	Head & N	G2	2008	-22136	Alive	TUMOR F	1095							0	1095	0	1095			0	1095
TCGA-CV	TCGA-CV	HNSC	50	MALE	WHITE	Stage III	Stage II	Head & N	G2	2008	-18466	Alive	TUMOR F	954							0	954	0	954			0	954
TCGA-CV	TCGA-CV	HNSC	62	MALE	WHITE	Stage IVA	Stage III	Head & N	G2	2009	-22646	Alive	TUMOR F	797							0	797	0	797			0	797
TCGA-CV	TCGA-CV	HNSC	62	MALE	WHITE	Stage IVA	Stage III	Head & N	G2	2009	-22646	Alive	TUMOR F	797							0	797	0	797			0	797
TCGA-CV	TCGA-CV	HNSC	55	MALE	WHITE	Stage III	Stage II	Head & N	G2	1995	-20433	Dead	WITH TUMOR		577						1	577	1	577			1	577
TCGA-CV	TCGA-CV	HNSC	63	FEMALE	BLACK OI	Stage IVA	Stage IVA	Head & N	G2	2001	-23082	Dead	TUMOR FREE		521						1	521	0	521			0	521
TCGA-CV	TCGA-CV	HNSC	64	MALE	WHITE	Stage IVA	Stage III	Head & N	G1	2000	-23489	Dead	TUMOR FREE		2900	Distant M	Distant M	supraster	111	Complete Remission/Response	1	2900	0	2900			1	111
TCGA-CV	TCGA-CV	HNSC	64	MALE	WHITE	Stage IVA	Stage III	Head & N	G1	2000	-23489	Dead	TUMOR FREE		2900	Distant M	Distant M	supraster	111	Complete Remission/Response	1	2900	0	2900			1	111
TCGA-CV	TCGA-CV	HNSC	62	FEMALE	BLACK OI	Stage IVA	Stage III	Head & N	G2	1994	-22934	Dead	WITH TUMOR		151					Progressive Disease	1	151	1	151			1	151
TCGA-CV	TCGA-CV	HNSC	62	FEMALE	BLACK OI	Stage IVA	Stage III	Head & N	G2	1994	-22934	Dead	WITH TUMOR		151					Progressive Disease	1	151	1	151			1	151
TCGA-CV	TCGA-CV	HNSC	58	MALE	BLACK OI	Stage IVA	Stage II	Head & N	G2	1993	-21473	Dead	WITH TUMOR		361					Partial Remission/Response	1	361	1	361			1	361
TCGA-CV	TCGA-CV	HNSC	55	MALE	WHITE	Stage II	Stage II	Head & N	G3	1996	-20306	Dead	WITH TUMOR		1459						1	1459	1	1459			1	1459
TCGA-CV	TCGA-CV	HNSC	32	FEMALE	WHITE	Stage IVA	Stage II	Head & N	G2	1995	-11931	Dead	WITH TUMOR		64					Complete Remission/Response	1	64	1	64			1	64
TCGA-CV	TCGA-CV	HNSC	32	FEMALE	WHITE	Stage IVA	Stage II	Head & N	G2	1995	-11931	Dead	WITH TUMOR		64					Complete Remission/Response	1	64	1	64			1	64
TCGA-CV	TCGA-CV	HNSC	57	MALE	WHITE	Stage IVA	Stage III	Head & N	G2	2008	-20992	Alive	TUMOR F	1512							0	1512	0	1512			0	1512
TCGA-CV	TCGA-CV	HNSC	57	MALE	WHITE	Stage IVA	Stage III	Head & N	G2	2008	-20992	Alive	TUMOR F	1512							0	1512	0	1512			0	1512
TCGA-CV	TCGA-CV	HNSC	64	MALE	BLACK OI	Stage II	Stage II	Head & N	G1	1997	-23513	Dead	WITH TUMOR		560						1	560	1	560			1	560
TCGA-CV	TCGA-CV	HNSC	64	MALE	BLACK OI	Stage II	Stage II	Head & N	G1	1997	-23513	Dead	WITH TUMOR		560						1	560	1	560			1	560
TCGA-CV	TCGA-CV	HNSC	49	MALE	WHITE	Stage II	Stage II	Head & N	GX	1999	-18250	Dead			1748						1	1748		1748			0	1748
TCGA-CV	TCGA-CV	HNSC	49	MALE	WHITE	Stage II	Stage II	Head & N	GX	1999	-18250	Dead			1748						1	1748		1748			0	1748
TCGA-CV	TCGA-CV	HNSC	67	FEMALE	WHITE	Stage II	Stage II	Head & N	G2	1992	-24475	Dead			1081						1	1081		1081			0	1081
TCGA-CV	TCGA-CV	HNSC	43	MALE	BLACK OI	Stage IVB	Stage IVB	Head & N	G3	1994	-15888	Dead			543					Complete Remission/Response	1	543		543			0	543
TCGA-CV	TCGA-CV	HNSC	61	MALE	WHITE	Stage III	Stage II	Head & N	GX	1993	-22302	Dead	WITH TUMOR		6417						1	6417	1	6417			1	6417
TCGA-CV	TCGA-CV	HNSC	64	FEMALE	WHITE	Stage IVA	Stage IVA	Head & N	G1	1994	-23696	Dead			2717						1	2717		2717			0	2717
TCGA-CV	TCGA-CV	HNSC	74	FEMALE	WHITE	Stage II	Stage II	Head & N	G2	1995	-27231	Dead			294					Complete Remission/Response	1	294		294	0	294	0	294
TCGA-CV	TCGA-CV	HNSC	78	MALE	WHITE	Stage IVA	Stage III	Head & N	G2	1995	-28603	Dead			14					Complete Remission/Response	1	14		14			0	14
TCGA-CV	TCGA-CV	HNSC	60	MALE	WHITE	Stage IVA	Stage III	Head & N	G2	1995	-22062	Dead	WITH TUMOR		695						1	695	1	695			1	695
TCGA-CV	TCGA-CV	HNSC	29	FEMALE	WHITE	Stage IVA	Stage IVA	Head & N	GX	1994	-10936	Dead			763						1	763		763			0	763
TCGA-CV	TCGA-CV	HNSC	29	FEMALE	WHITE	Stage IVA	Stage IVA	Head & N	GX	1994	-10936	Dead			763						1	763		763			0	763
TCGA-CV	TCGA-CV	HNSC	62	MALE	BLACK OI	Stage IVA	Stage IVA	Head & N	G2	1996	-22805	Dead	TUMOR FREE		789	Locoregic	Hypophar	nasophar	494	Complete Remission/Response	1	789	0	789			1	494
TCGA-CV	TCGA-CV	HNSC	76	MALE	WHITE	Stage IVA	Stage IVA	Head & N	G2	1997	-27961	Dead			2						1	2		2			0	2
TCGA-CV	TCGA-CV	HNSC	60	FEMALE	WHITE	Stage IVA	Stage IVA	Head & N	G3	1997	-22098	Dead	WITH TUMOR		1037	Locoregic	Larynx	tracheal	733	Complete Remission/Response	1	1037	1	1037			1	733
TCGA-CV	TCGA-CV	HNSC	65	MALE	WHITE	Stage II	Stage II	Head & N	G1	1997	-24071	Dead			3059						1	3059		3059			0	3059
TCGA-CV	TCGA-CV	HNSC	65	MALE	WHITE	Stage II	Stage II	Head & N	G1	1997	-24071	Dead			3059						1	3059		3059			0	3059
TCGA-CV	TCGA-CV	HNSC	67	MALE	ASIAN	Stage IVA	Stage IVA	Head & N	G2	1997	-24775	Dead	WITH TUMOR		453						1	453	1	453			1	453
TCGA-CV	TCGA-CV	HNSC	67	MALE	ASIAN	Stage IVA	Stage IVA	Head & N	G2	1997	-24775	Dead	WITH TUMOR		453						1	453	1	453			1	453
TCGA-CV	TCGA-CV	HNSC	77	FEMALE	WHITE	Stage III	Stage III	Head & N	G1	2000	-28368	Dead	WITH TUMOR		1718						1	1718	1	1718			1	1718
TCGA-CV	TCGA-CV	HNSC	77	FEMALE	WHITE	Stage III	Stage III	Head & N	G1	2000	-28368	Dead	WITH TUMOR		1718						1	1718	1	1718			1	1718
TCGA-CV	TCGA-CV	HNSC	73	FEMALE	WHITE	Stage II	Stage II	Head & N	G1	1997	-26975	Dead	TUMOR FREE		4760	Locoregic	Oral Cavit	Maxilla	3282	Complete Remission/Response	1	4760	0	4760	1	3282	1	3282
TCGA-CV	TCGA-CV	HNSC	47	MALE	WHITE	Stage IVA	Stage IVA	Head & N	G2	1998	-17429	Dead	WITH TUMOR		1671						1	1671	1	1671			1	1671
TCGA-CV	TCGA-CV	HNSC	55	MALE	WHITE	Stage IVA	Stage III	Head & N	G3	1998	-20188	Dead	WITH TUMOR		107					Partial Remission/Response	1	107	1	107			1	107
TCGA-CV	TCGA-CV	HNSC	56	MALE	WHITE	Stage IVA	Stage III	Head & N	G2	1996	-20763	Dead	WITH TUMOR		495						1	495	1	495			1	495
TCGA-CV	TCGA-CV	HNSC	79	MALE	WHITE	Stage II	Stage III	Head & N	G1	1998	-28875	Dead	WITH TUMOR		2570					Complete Remission/Response	1	2570	1	2570			1	2570
TCGA-CV	TCGA-CV	HNSC	79	MALE	WHITE	Stage II	Stage III	Head & N	G1	1998	-28875	Dead	WITH TUMOR		2570					Complete Remission/Response	1	2570	1	2570			1	2570
TCGA-CV	TCGA-CV	HNSC	49	MALE	WHITE	Stage IVA	Stage IVA	Head & N	G3	1999	-18004	Dead	WITH TUMOR		601	Distant M	Distant M	Lt. Deltoic	445	Progressive Disease	1	601	1	601			1	445
TCGA-CV	TCGA-CV	HNSC	64	MALE	WHITE	Stage IVA	Stage III	Head & N	G2	1999	-23465	Dead	TUMOR FREE		218					Complete Remission/Response	1	218	0	218			0	218
TCGA-CV	TCGA-CV	HNSC	64	MALE	WHITE	Stage IVA	Stage III	Head & N	G2	1999	-23465	Dead	TUMOR FREE		218					Complete Remission/Response	1	218	0	218			0	218
TCGA-CV	TCGA-CV	HNSC	57	FEMALE	WHITE	Stage IVA	Stage IVA	Head & N	G2	1999	-21049	Dead	TUMOR FREE		4680					Complete Remission/Response	1	4680	0	4680			0	4680
TCGA-CV	TCGA-CV	HNSC	77	MALE		Stage II	Stage II	Head & N	GX	1996	-28221	Dead	WITH TUMOR		506						1	506	1	506			1	506
TCGA-CV	TCGA-CV	HNSC	77	MALE		Stage II	Stage II	Head & N	GX	1996	-28221	Dead	WITH TUMOR		506						1	506	1	506			1	506
TCGA-CV	TCGA-CV	HNSC	87	FEMALE	WHITE	Stage I	Stage II	Head & N	G3	2000	-32021	Dead	WITH TUMOR		194						1	194	1	194			1	194
TCGA-CV	TCGA-CV	HNSC	87	FEMALE	WHITE	Stage I	Stage II	Head & N	G3	2000	-32021	Dead	WITH TUMOR		194						1	194	1	194			1	194
TCGA-CV	TCGA-CV	HNSC	38	MALE	WHITE	Stage II	Stage II	Head & N	GX	2000	-13992	Dead			675						1	675		675			0	675
TCGA-CV	TCGA-CV	HNSC	38	MALE	WHITE	Stage II	Stage II	Head & N	GX	2000	-13992	Dead			675						1	675		675			0	675
TCGA-CV	TCGA-CV	HNSC	66	MALE	WHITE	Stage IVA	Stage II	Head & N	G2	1998	-24170	Dead	WITH TUMOR		1093						1	1093	1	1093			1	1093
TCGA-CV	TCGA-CV	HNSC	48	FEMALE	WHITE	Stage IVA	Stage IVA	Head & N	GX	1995	-17670	Dead			927						1	927		927			0	927
TCGA-CV	TCGA-CV	HNSC	57	MALE	WHITE		Stage III	Head & N	G2	1994	-20868	Alive	TUMOR F	851							0	851	0	851			0	851
TCGA-CV	TCGA-CV	HNSC	82	FEMALE	WHITE	Stage I	Stage II	Head & N	G2	1998	-30297	Alive	WITH TUI	639		Locoregic	Oral Cavit	Floor of m	639	Complete Remission/Response	0	639	0	639	1	639	1	639
TCGA-CV	TCGA-CV	HNSC	69	FEMALE	WHITE		Stage IVC	Head & N	G1	1995	-25379	Dead	TUMOR FREE		5152					Complete Remission/Response	1	5152	0	5152			0	5152
TCGA-CV	TCGA-CV	HNSC	46	MALE	WHITE	Stage III	Stage II	Head & N	G1	1997	-17125	Alive	TUMOR F	5480						Complete Remission/Response	0	5480	0	5480	0	5480	0	5480
TCGA-CV	TCGA-CV	HNSC	64	FEMALE	WHITE	[Discrepa	Stage II	Head & N	G3	1998	-23452	Dead	TUMOR FREE		4856						1	4856	0	4856			0	4856
TCGA-CV	TCGA-CV	HNSC	59	MALE	WHITE	Stage IVA	Stage IVA	Head & N	G1	1999	-21746	Dead	WITH TUMOR		1079		Distant M	pelvis	1064	Complete Remission/Response	1	1079	1	1079			1	1064
TCGA-CV	TCGA-CV	HNSC	87	FEMALE	WHITE	Stage IVA	Stage IVA	Head & N	G1	1999	-31936	Dead			32						1	32		32			0	32
TCGA-CV	TCGA-CV	HNSC	75	MALE	WHITE	Stage II	Stage III	Head & N	G2	1999	-27743	Dead	TUMOR FREE		1398						1	1398	0	1398			0	1398
TCGA-CV	TCGA-CV	HNSC	47	MALE	WHITE	Stage IVA	Stage IVA	Head & N	G2	2000	-17464	Dead	TUMOR FREE		198					Complete Remission/Response	1	198	0	198			0	198
TCGA-CV	TCGA-CV	HNSC	61	MALE	AMERICA	Stage IVA	Stage IVA	Head & N	G3	2000	-22442	Dead	TUMOR FREE		2703			R. Lung	2318	Complete Remission/Response	1	2703	0	2703			1	2318
TCGA-CV	TCGA-CV	HNSC	75	MALE	WHITE	[Discrepa	Stage II	Head & N	G3	2000	-27397	Dead	WITH TUMOR		1466						1	1466	1	1466			1	1466
TCGA-CV	TCGA-CV	HNSC	72	MALE	WHITE	[Discrepa	Stage IVA	Head & N	G3	2001	-26539	Dead	[Discrepancy]		1838	New Primary Tumor		mediastin	1425	Complete Remission/Response	1	1838		1838			1	1425
TCGA-CV	TCGA-CV	HNSC	65	MALE	WHITE		Stage III	Head & N	G2	2004	-23955	Dead	TUMOR FREE		2064					Complete Remission/Response	1	2064	0	2064	0	2064	0	2064
TCGA-CV	TCGA-CV	HNSC	82	FEMALE	WHITE	Stage IVA	Stage IVA	Head & N	G2	2007	-30095	Dead	TUMOR FREE		23					Complete Remission/Response	1	23	0	23			0	23
TCGA-CV	TCGA-CV	HNSC	48	MALE	WHITE	Stage III	Stage IVA	Head & N	G2	2008	-17669	Alive	TUMOR F	1722		Locoregional Disease		pterygoid	28	Complete Remission/Response	0	1722	0	1722			1	28
TCGA-CV	TCGA-CV	HNSC	24	MALE	WHITE	Stage III	Stage II	Head & N	G1	2008	-9015	Dead	TUMOR FREE		215					Complete Remission/Response	1	215	0	215	0	215	0	215
TCGA-CV	TCGA-CV	HNSC	42	MALE		Stage IVA	Stage III	Head & N	G2	2001	-15372	Dead	TUMOR FREE		464					Complete Remission/Response	1	464	0	464			0	464
TCGA-CV	TCGA-CV	HNSC	82	FEMALE	WHITE		Stage IVA	Head & N	G3	2012	-29988	Dead	TUMOR FREE		182					Complete Remission/Response	1	182	0	182			0	182
TCGA-CV	TCGA-CV	HNSC	78	MALE	WHITE		Stage II	Head & N	G2	2011	-28658	Alive	TUMOR F	1075						Complete Remission/Response	0	1075	0	1075	0	1075	0	1075
TCGA-CV	TCGA-CV	HNSC	85	MALE	WHITE		Stage IVA	Head & N	G2	2011	-31147	Dead	TUMOR FREE		194	Locoregic	Oropharynx		107	Complete Remission/Response	1	194	0	194			1	107
TCGA-CV	TCGA-CV	HNSC	53	MALE	WHITE		Stage II	Head & N	G1	2011	-19635	Alive	TUMOR F	906						Complete Remission/Response	0	906	0	906	0	906	0	906
TCGA-CV	TCGA-CV	HNSC	69	MALE	WHITE	Stage IVA	Stage IVA	Head & N	G2	2011	-25377	Dead	WITH TUMOR		197		Oral Cavit	submandi	127	Progressive Disease	1	197	1	197			1	127
TCGA-CV	TCGA-CV	HNSC	65	MALE	WHITE	Stage II	Stage IVA	Head & N	G2	2011	-23806	Alive	TUMOR F	852						Complete Remission/Response	0	852	0	852	0	852	0	852
TCGA-CV	TCGA-CV	HNSC	61	FEMALE	WHITE	Stage IVB	Stage IVA	Head & N	G2	2011	-22591	Alive	TUMOR F	110						Complete Remission/Response	0	110	0	110			0	110
TCGA-CV	TCGA-CV	HNSC	69	MALE	WHITE	Stage IVA	Stage IVA	Head & N	G1	2011	-25321	Alive	TUMOR F	646						Complete Remission/Response	0	646	0	646			0	646
TCGA-CV	TCGA-CV	HNSC	68	MALE	WHITE	Stage II	Stage IVA	Head & N	G2	2011	-25182	Alive	WITH TUI	714		Locoregic	Oral Cavit	submandi	393	Complete Remission/Response	0	714	0	714			1	393
TCGA-CV	TCGA-CV	HNSC	58	MALE	WHITE	Stage I	Stage II	Head & N	G3	2011	-21488	Alive	TUMOR F	606			Cervical L	neck	146	Complete Remission/Response	0	606	0	606	1	146	1	146
TCGA-CV	TCGA-CV	HNSC	65	MALE	WHITE	Stage IVA	Stage IVA	Head & N	G2	2011	-23848	Alive	TUMOR F	685		New Prim	Oral Cavit	Tonsil	401	Complete Remission/Response	0	685	0	685			1	401
TCGA-CV	TCGA-CV	HNSC	79	MALE	WHITE	Stage IVA	Stage II	Head & N	G1	2011	-29114	Dead	WITH TUMOR		317	Locoregional Recurre		Infratemp	134	Progressive Disease	1	317	1	317			1	134
TCGA-CX	TCGA-CX	HNSC	82	MALE	WHITE	Stage IVA	Stage II	Head & N	G2	2010	-30174	Dead	TUMOR FREE		11					Complete Remission/Response	1	11	0	11			0	11
TCGA-CX	TCGA-CX	HNSC	77	FEMALE	WHITE	Stage I	Stage III	Head & N	G2	2010	-28221	Alive	WITH TUI	321		Locoregic	Oropharv	Submand	92	Complete Remission/Response	0	321	0	321	1	92	1	92
TCGA-CX	TCGA-CX	HNSC	53	MALE	BLACK OI	Stage III	Stage IVA	Head & N	G2	2011	-19434	Alive	TUMOR F	573						Complete Remission/Response	0	573	0	573			0	573
TCGA-CX	TCGA-CX	HNSC	47	MALE	BLACK OI	Stage IVA	Stage IVA	Head & N	G2	2009	-17222	Alive	TUMOR F	1045						Complete Remission/Response	0	1045	0	1045			0	1045
TCGA-CX	TCGA-CX	HNSC	56	MALE	WHITE	Stage IVA	Stage IVA	Head & N	G3	2008	-20709	Alive	TUMOR F	1555						Complete Remission/Response	0	1555	0	1555			0	1555
TCGA-D6	TCGA-D6	HNSC	82	FEMALE	WHITE	Stage II	Stage II	Head & N	G3	2010	-30099	Dead	TUMOR FREE		403		Cervical Lymph Node		195	Complete Remission/Response	1	403	0	403	1	195	1	195
TCGA-D6	TCGA-D6	HNSC	69	MALE	WHITE	Stage I	Stage III	Head & N	G2	2010	-25242	Alive	TUMOR F	773						Complete Remission/Response	0	773	0	773	0	773	0	773
TCGA-D6	TCGA-D6	HNSC	59	MALE	WHITE	Stage III	Stage III	Head & N	G2	2010	-21791	Alive	WITH TUI	292			Distant M	tracheost	161	Progressive Disease	0	292	0	292			1	161
TCGA-D6	TCGA-D6	HNSC	50	MALE	WHITE	Stage II	Stage III	Head & N	G2	2010	-18611	Alive	TUMOR F	701						Complete Remission/Response	0	701	0	701	0	701	0	701
TCGA-D6	TCGA-D6	HNSC	61	MALE	WHITE	Stage IVA	Stage IVA	Head & N	G2	2010	-22633	Alive	TUMOR F	77						Complete Remission/Response	0	77	0	77			0	77
TCGA-D6	TCGA-D6	HNSC	73	MALE	WHITE	Stage I	Stage III	Head & N	G2	2011	-26976	Alive	TUMOR F	491						Complete Remission/Response	0	491	0	491	0	491	0	491
TCGA-D6	TCGA-D6	HNSC	64	FEMALE	WHITE	Stage IVA	Stage IVA	Head & N	G2	2011	-23722	Dead	TUMOR FREE		348					Complete Remission/Response	1	348	0	348			0	348
TCGA-D6	TCGA-D6	HNSC	55	FEMALE	WHITE	Stage I	Stage III	Head & N	G3	2011	-20175	Alive	TUMOR F	568						Complete Remission/Response	0	568	0	568	0	568	0	568
TCGA-D6	TCGA-D6	HNSC	62	MALE	WHITE	Stage II	Stage II	Head & N	G2	2012	-22796	Alive	TUMOR F	759						Stable Disease	0	759	0	759			0	759
TCGA-D6	TCGA-D6	HNSC	52	MALE	WHITE	Stage II	Stage II	Head & N	G2	2012	-19212	Alive	TUMOR F	770						Stable Disease	0	770	0	770			0	770
TCGA-D6	TCGA-D6	HNSC	59	MALE	WHITE	Stage IVA	Stage IVA	Head & N	G2	2012	-21659	Alive	TUMOR F	539						Complete Remission/Response	0	539	0	539			0	539
TCGA-D6	TCGA-D6	HNSC	61	MALE	WHITE	Stage III	Stage III	Head & N	G2	2012	-22455	Alive	TUMOR F	376						Complete Remission/Response	0	376	0	376	0	376	0	376
TCGA-D6	TCGA-D6	HNSC	67	MALE	WHITE	Stage III	Stage IVA	Head & N	G1	2012	-24558	Alive	TUMOR F	875						Partial Remission/Response	0	875	0	875			0	875
TCGA-D6	TCGA-D6	HNSC	65	FEMALE	WHITE	Stage III	Stage III	Head & N	G2	2012	-23865	Alive	WITH TUI	232			Oral Cavit	oral tongu	232	Complete Remission/Response	0	232	0	232	1	232	1	232
TCGA-D6	TCGA-D6	HNSC	71	MALE	WHITE	Stage III	Stage III	Head & N	G3	2013	-26294	Alive	TUMOR F	687						Complete Remission/Response	0	687	0	687	0	687	0	687
TCGA-D6	TCGA-D6	HNSC	44	MALE	WHITE	Stage IVA	Stage IVA	Head & N	G2	2013	-16185	Alive	TUMOR F	759						Stable Disease	0	759	0	759			0	759
TCGA-D6	TCGA-D6	HNSC	62	MALE	WHITE	Stage III	Stage III	Head & N	G3	2013	-22674	Alive	TUMOR F	424						Complete Remission/Response	0	424	0	424	0	424	0	424
TCGA-D6	TCGA-D6	HNSC	57	MALE	WHITE	Stage IVA	Stage IVA	Head & N	G3	2013	-20876	Alive	TUMOR F	368						Complete Remission/Response	0	368	0	368			0	368
TCGA-D6	TCGA-D6	HNSC	50	MALE	WHITE	Stage IVA	Stage IVA	Head & N	G2	2013	-18408	Alive	TUMOR F	389						Complete Remission/Response	0	389	0	389			0	389
TCGA-D6	TCGA-D6	HNSC	67	MALE	WHITE	Stage IVA	Stage IVA	Head & N	G3	2013	-24702	Alive	TUMOR F	710						Complete Remission/Response	0	710	0	710			0	710
TCGA-DC	TCGA-DC	HNSC	43	FEMALE	WHITE		Stage IVA	Head & N	G2	2007	-15867	Alive	TUMOR F	1778						Complete Remission/Response	0	1778	0	1778			0	1778
TCGA-DC	TCGA-DC	HNSC	52	FEMALE	WHITE		Stage II	Head & N	G2	2008	-19211	Dead	TUMOR FREE		1133		Oral Cavity		1058	Complete Remission/Response	1	1133	0	1133	1	1058	1	1058
TCGA-DC	TCGA-DC	HNSC	64	MALE	WHITE		Stage IVA	Head & N	G2	2009	-23550	Dead	TUMOR FREE		941					Complete Remission/Response	1	941	0	941			0	941
TCGA-DC	TCGA-DC	HNSC	73	MALE	WHITE		Stage III	Head & N	G2	2009	-26855	Alive	TUMOR F	1030						Complete Remission/Response	0	1030	0	1030	0	1030	0	1030
TCGA-DC	TCGA-DC	HNSC	52	MALE	WHITE		Stage IVA	Head & N	G3	2009	-19172	Dead	WITH TUI	548	548		Distant M	Lung	447	Complete Remission/Response	1	548	1	548			1	447
TCGA-DC	TCGA-DC	HNSC	66	MALE	WHITE		Stage III	Head & N	G3	2010	-24106	Dead	WITH TUMOR		427		Oral Cavity		79	Persistent Disease	1	427	1	427			1	79
TCGA-DC	TCGA-DC	HNSC	70	MALE	WHITE		Stage IVC	Head & N	G4	2010	-25606	Alive	TUMOR F	1409						Complete Remission/Response	0	1409	0	1409			0	1409
TCGA-DC	TCGA-DC	HNSC	51	MALE	WHITE		Stage IVA	Head & N	G4	2010	-18718	Alive	TUMOR F	1413						Complete Remission/Response	0	1413	0	1413			0	1413
TCGA-DC	TCGA-DC	HNSC	62	MALE	WHITE		Stage IVA	Head & N	G4	2011	-22798	Alive	TUMOR F	622						Complete Remission/Response	0	622	0	622			0	622
TCGA-DC	TCGA-DC	HNSC	57	MALE	AMERICAN INDIAN (		Stage IVA	Head & N	G2	2011	-21041	Alive	TUMOR F	1143						Complete Remission/Response	0	1143	0	1143			0	1143
TCGA-DC	TCGA-DC	HNSC	58	MALE	WHITE		Stage IVA	Head & N	G4	2011	-21439	Alive	TUMOR F	1224						Complete Remission/Response	0	1224	0	1224			0	1224
TCGA-DC	TCGA-DC	HNSC	47	MALE	WHITE		Stage IVB	Head & N	G4	2011	-17471	Alive	TUMOR F	1218		New Prim	Oral Cavit	mandibul	1084	Complete Remission/Response	0	1218	0	1218			1	1084
TCGA-DC	TCGA-DC	HNSC	53	MALE	WHITE		Stage II	Head & N	G3	2011	-19530	Alive	TUMOR F	1190						Complete Remission/Response	0	1190	0	1190	0	1190	0	1190
TCGA-DC	TCGA-DC	HNSC	48	MALE	WHITE		Stage IVA	Head & N	G4	2011	-17846	Alive	TUMOR F	1265			Cervical Lymph Node		137	Persistent Disease	0	1265	0	1265			1	137
TCGA-F7	TCGA-F7	HNSC	47	MALE	WHITE	Stage IVA	Stage IVA	Head & N	G2	2011	-17251	Alive	TUMOR F	1131						Complete Remission/Response	0	1131	0	1131			0	1131
TCGA-F7	TCGA-F7	HNSC	58	MALE	WHITE	Stage I	Stage I	Head & N	G1	2011	-21445	Alive	TUMOR F	995						Complete Remission/Response	0	995	0	995	0	995	0	995
TCGA-F7	TCGA-F7	HNSC	48	MALE	WHITE	Stage II	Stage II	Head & N	G1	2012	-17873	Alive	TUMOR F	658						Complete Remission/Response	0	658	0	658	0	658	0	658
TCGA-F7	TCGA-F7	HNSC	66	MALE	WHITE	Stage III		Head & N	G1	2012	-24285	Alive	TUMOR F	616						Complete Remission/Response	0	616	0	616	0	616	0	616
TCGA-F7	TCGA-F7	HNSC	72	MALE	WHITE	Stage IVA		Head & N	G2	2011	-26373	Alive	TUMOR F	92							0	92	0	92			0	92
TCGA-F7	TCGA-F7	HNSC	67	FEMALE	WHITE	Stage III		Head & N	G2	2011	-24758	Alive	TUMOR F	947						Complete Remission/Response	0	947	0	947	0	947	0	947
TCGA-F7	TCGA-F7	HNSC	62	MALE	WHITE	Stage III		Head & N	G1	2012	-22655	Alive	TUMOR F	576		Locoregic	Cervical Lymph Node		213	Complete Remission/Response	0	576	0	576	1	213	1	213
TCGA-F7	TCGA-F7	HNSC	54	MALE	WHITE	Stage II		Head & N	G1	2012	-19857	Alive	TUMOR F	759						Complete Remission/Response	0	759	0	759	0	759	0	759
TCGA-F7	TCGA-F7	HNSC	51	MALE	ASIAN	Stage IVA		Head & N	G2	2012	-18879	Alive	TUMOR F	14						[Unknown]	0	14	0	14			0	14
TCGA-F7	TCGA-F7	HNSC	47	MALE	WHITE	Stage III		Head & N	G1	2012	-17475	Alive	TUMOR F	543						Complete Remission/Response	0	543	0	543	0	543	0	543
TCGA-F7	TCGA-F7	HNSC	75	MALE	WHITE	Stage III		Head & N	G1	2012	-27456	Dead	WITH TUMOR		359					Complete Remission/Response	1	359	1	359			1	359
TCGA-F7	TCGA-F7	HNSC	70	MALE	WHITE	Stage IVA		Head & N	G1	2012	-25592	Alive	TUMOR F	616						Complete Remission/Response	0	616	0	616			0	616
TCGA-F7	TCGA-F7	HNSC	73	MALE	WHITE	Stage II		Head & N	G3	2012	-26667	Alive	TUMOR F	378						Complete Remission/Response	0	378	0	378	0	378	0	378
TCGA-H7	TCGA-H7	HNSC	75	FEMALE	WHITE	Stage IVA	Stage III	Head & N	G2	2011	-27519	Alive	TUMOR F	407		New Prim	Oral Cavit	hard palat	252	Complete Remission/Response	0	407	0	407			1	252
TCGA-H7	TCGA-H7	HNSC	54	MALE	WHITE	Stage IVA	Stage IVA	Head & N	GX	2012	-19808	Alive	TUMOR F	461						Complete Remission/Response	0	461	0	461			0	461
TCGA-H7	TCGA-H7	HNSC	50	MALE	WHITE	Stage IVA	Stage IVA	Head & N	G2	2012	-18286	Alive	TUMOR F	458						Complete Remission/Response	0	458	0	458			0	458
TCGA-H7	TCGA-H7	HNSC	35	FEMALE	WHITE	Stage IVA	Stage III	Head & N	G2	2012	-13137	Alive	TUMOR F	414		Locoregic	Distant M	stoma site	235	Complete Remission/Response	0	414	0	414			1	235
TCGA-H7	TCGA-H7	HNSC	35	FEMALE	WHITE	Stage IVA	Stage III	Head & N	G2	2012	-13137	Alive	TUMOR F	414		Locoregic	Distant M	stoma site	235	Complete Remission/Response	0	414	0	414			1	235
TCGA-H7	TCGA-H7	HNSC	65	MALE	WHITE	Stage II	Stage III	Head & N	G2	2012	-23808	Alive	TUMOR F	642						Complete Remission/Response	0	642	0	642	0	642	0	642
TCGA-H7	TCGA-H7	HNSC	65	MALE	WHITE	Stage II	Stage III	Head & N	G2	2012	-23808	Alive	TUMOR F	642						Complete Remission/Response	0	642	0	642	0	642	0	642
TCGA-H7	TCGA-H7	HNSC	57	MALE	WHITE		Stage IVA	Head & N	G2	2013	-20946	Alive	TUMOR F	637						Complete Remission/Response	0	637	0	637			0	637
TCGA-HD	TCGA-HD	HNSC	60	MALE	WHITE	Stage IVA	Stage IVA	Head & N	G3	2011	-22076	Alive	WITH TUI	1027		Locoregic	Larynx		162	Progressive Disease	0	1027	0	1027			1	162
TCGA-HD	TCGA-HD	HNSC	62	MALE	BLACK OI	Stage I	Stage II	Head & N	G2	2011	-22774	Alive	TUMOR F	866						Complete Remission/Response	0	866	0	866	0	866	0	866
TCGA-HD	TCGA-HD	HNSC	69	MALE	WHITE	Stage IVA	Stage IVA	Head & N	G1	2011	-25365	Alive	TUMOR F	783						Complete Remission/Response	0	783	0	783			0	783
TCGA-HD	TCGA-HD	HNSC	74	MALE	WHITE	Stage IVA	Stage III	Head & N	G2	2011	-27334	Alive	WITH TUI	667		Distant M	Distant M	Lung	667	Complete Remission/Response	0	667	0	667			1	667
TCGA-HD	TCGA-HD	HNSC	52	MALE	WHITE	Stage IVA	Stage IVA	Head & N	G2	2011	-19192	Alive	TUMOR F	836						Complete Remission/Response	0	836	0	836			0	836
TCGA-HD	TCGA-HD	HNSC	62	MALE	WHITE	Stage II	Stage II	Head & N	G1	2011	-22979	Dead	TUMOR FREE		836					Complete Remission/Response	1	836	0	836	0	836	0	836
TCGA-HD	TCGA-HD	HNSC	63	MALE	WHITE	Stage IVA	Stage III	Head & N	G3	2011	-23069	Dead	WITH TUMOR		446	Distant M	Distant M	Lung	171	Progressive Disease	1	446	1	446			1	171
TCGA-HD	TCGA-HD	HNSC	58	MALE	WHITE	Stage III	Stage III	Head & N	G4	2012	-21517	Alive	TUMOR F	670						Complete Remission/Response	0	670	0	670	0	670	0	670
TCGA-HD	TCGA-HD	HNSC	51	FEMALE	WHITE	Stage I	Stage I	Head & N	G2	2012	-18718	Dead	TUMOR FREE		385					Complete Remission/Response	1	385	0	385	0	385	0	385
TCGA-HD	TCGA-HD	HNSC	61	FEMALE	WHITE	Stage III	Stage III	Head & N	G2	2012	-22340	Alive	TUMOR F	695						Complete Remission/Response	0	695	0	695	0	695	0	695
TCGA-HD	TCGA-HD	HNSC	61	FEMALE	WHITE	Stage III	Stage III	Head & N	G2	2012	-22340	Alive	TUMOR F	695						Complete Remission/Response	0	695	0	695	0	695	0	695
TCGA-HD	TCGA-HD	HNSC	41	FEMALE	BLACK OI	Stage IVA	Stage IVA	Head & N	G1	2012	-15096	Alive		11							0	11	0	11			0	11
TCGA-HD	TCGA-HD	HNSC	74	MALE	WHITE	Stage IVA	Stage IVA	Head & N	G2	2012	-27057	Alive	WITH TUI	421		Locoregic	Oral Cavit	Buccal Sp	419	Complete Remission/Response	0	421	0	421			1	419
TCGA-HD	TCGA-HD	HNSC	56	MALE	WHITE	Stage IVA	Stage III	Head & N	G3	2013	-20810	Dead	WITH TUMOR		130					[Unknown]	1	130	1	130			1	130
TCGA-HD	TCGA-HD	HNSC	79	FEMALE	WHITE	Stage III	Stage II	Head & N	G2	2012	-29045	Alive	TUMOR F	111						[Unknown]	0	111	0	111			0	111
TCGA-HD	TCGA-HD	HNSC	79	FEMALE	WHITE	Stage III	Stage II	Head & N	G2	2012	-29045	Alive	TUMOR F	111						[Unknown]	0	111	0	111			0	111
TCGA-HD	TCGA-HD	HNSC	56	MALE	WHITE	Stage IVA	Stage III	Head & N	G1	2013	-20491	Alive	TUMOR F	210						Complete Remission/Response	0	210	0	210			0	210
TCGA-HD	TCGA-HD	HNSC	56	MALE	WHITE	Stage IVA	Stage III	Head & N	G1	2013	-20491	Alive	TUMOR F	210						Complete Remission/Response	0	210	0	210			0	210
TCGA-HL	TCGA-HL	HNSC	65	MALE	WHITE		Stage IVA	Head & N	G3	2008	-23950	Alive	TUMOR F	1057						Complete Remission/Response	0	1057	0	1057			0	1057
TCGA-IO	TCGA-IO	HNSC	49	MALE	WHITE	Stage II	Stage III	Head & N	G3	2011	-18131	Alive	TUMOR F	485						Complete Remission/Response	0	485	0	485	0	485	0	485
TCGA-IO	TCGA-IO	HNSC	60	FEMALE	WHITE	Stage II	Stage II	Head & N	G1	2011	-22151	Alive	TUMOR F	1172						Complete Remission/Response	0	1172	0	1172	0	1172	0	1172
TCGA-IO	TCGA-IO	HNSC	68	FEMALE	WHITE	Stage IVA	Stage IVA	Head & N	G1	2011	-24986	Alive	TUMOR F	441						Complete Remission/Response	0	441	0	441			0	441
TCGA-IO	TCGA-IO	HNSC	55	FEMALE	WHITE	Stage III	Stage III	Head & N	G2	2011	-20417	Alive	TUMOR F	1147						Complete Remission/Response	0	1147	0	1147	0	1147	0	1147
TCGA-IO	TCGA-IO	HNSC	57	MALE	WHITE	Stage IVA	Stage IVA	Head & N	G2	2012	-21066	Alive	WITH TUI	360			Oral Cavity		249	Progressive Disease	0	360	0	360			1	249
TCGA-IQ	TCGA-IQ	HNSC	76	MALE	WHITE	Stage II	Stage II	Head & N	G2	2012	-27990	Alive	TUMOR F	1138						Complete Remission/Response	0	1138	0	1138	0	1138	0	1138
TCGA-IO	TCGA-IO	HNSC	63	MALE	BLACK OI	Stage IVA	Stage IVA	Head & N	G3	2012	-23035	Dead	TUMOR FREE		2					Complete Remission/Response	1	2	0	2			0	2
TCGA-IO	TCGA-IO	HNSC	54	MALE	WHITE	Stage IVA	Stage IVA	Head & N	G1	2012	-19913	Alive	TUMOR F	1021						Complete Remission/Response	0	1021	0	1021			0	1021
TCGA-IO	TCGA-IO	HNSC	70	FEMALE	WHITE	Stage IVA	Stage IVA	Head & N	G2	2012	-25697	Dead	TUMOR FREE		161						1	161	0	161			0	161
TCGA-IO	TCGA-IO	HNSC	72	FEMALE	WHITE	Stage II	Stage II	Head & N	G1	2012	-26506	Alive	WITH TUI	416		Locoregic	Oral Cavity		156	Progressive Disease	0	416	0	416			1	156
TCGA-IO	TCGA-IO	HNSC	43	MALE	WHITE	Stage IVA	Stage IVA	Head & N	GX	2012	-15920	Dead	TUMOR FREE		421					Complete Remission/Response	1	421	0	421			0	421
TCGA-IO	TCGA-IO	HNSC	61	FEMALE	WHITE	Stage III	Stage III	Head & N	G2	2013	-22529	Alive	TUMOR F	579						Complete Remission/Response	0	579	0	579	0	579	0	579
TCGA-IO	TCGA-IO	HNSC	55	MALE	BLACK OI	Stage III	Stage IVC	Head & N	G1	2013	-20109	Alive	TUMOR F	471						Complete Remission/Response	0	471	0	471			0	471
TCGA-KU	TCGA-KU	HNSC	69	FEMALE	WHITE	Stage III	Stage III	Head & N	G2	2012	-25454	Dead	WITH TUMOR		406	Distant M	Distant M	Brain Met	389	Complete Remission/Response	1	406	1	406	1	389	1	389
TCGA-KU	TCGA-KU	HNSC	53	FEMALE	WHITE	Stage IVA	Stage IVA	Head & N	G2	2012	-19405	Alive	TUMOR F	552		Locoregic	Oral Cavit	Toungue	552	Complete Remission/Response	0	552	0	552			1	552
TCGA-KU	TCGA-KU	HNSC	55	FEMALE	WHITE	Stage IVA	Stage IVA	Head & N	G2	2013	-20314	Alive	TUMOR F	586						Complete Remission/Response	0	586	0	586			0	586
TCGA-KU	TCGA-KU	HNSC	55	FEMALE	WHITE	Stage IVA	Stage IVA	Head & N	G2	2013	-20314	Alive	TUMOR F	586						Complete Remission/Response	0	586	0	586			0	586
TCGA-KU	TCGA-KU	HNSC	41	MALE	WHITE	Stage IVA	Stage I	Head & N	G3	2013	-15066	Dead	WITH TUMOR		327	Locoregic	Oral Cavit	Right bas	110	Progressive Disease	1	327	1	327			1	110
TCGA-MT	TCGA-MT	HNSC	52	FEMALE	WHITE	Stage I	Stage I	Head & N	G2	2012	-19098	Alive	TUMOR F	437						Complete Remission/Response	0	437	0	437	0	437	0	437
TCGA-MT	TCGA-MT	HNSC	30	MALE	WHITE	Stage IVA	Stage IVA	Head & N	G1	2012	-10976	Alive	TUMOR F	242						[Unknown]	0	242	0	242			0	242
TCGA-MT	TCGA-MT	HNSC	85	FEMALE	WHITE	Stage I	Stage I	Head & N	G2	2011	-31161	Alive	TUMOR F	914						Complete Remission/Response	0	914	0	914	0	914	0	914
TCGA-MT	TCGA-MT	HNSC	55	MALE	WHITE	Stage II	Stage II	Head & N	G2	2012	-20386	Alive		56						Complete Remission/Response	0	56	0	56	0	56	0	56
TCGA-MT	TCGA-MT	HNSC	60	FEMALE	WHITE	Stage IVA	Stage IVA	Head & N	G2	2012	-22057	Alive	TUMOR F	384						Complete Remission/Response	0	384	0	384			0	384
TCGA-MT	TCGA-MT	HNSC	53	FEMALE	WHITE	Stage IVA	Stage IVA	Head & N	G2	2013	-19394	Alive	[Discrepar	190						[Unknown]	0	190	0	190			0	190
TCGA-MT	TCGA-MT	HNSC	74	MALE	WHITE	Stage IVA	Stage IVA	Head & N	G3	2013	-27162	Alive	WITH TUI	469		Distant M	Distant M	lung	179	Progressive Disease	0	469	0	469			1	179
TCGA-MZ	TCGA-MZ	HNSC	53	MALE	WHITE		Stage IVA	Head & Neck Squam		2008	-19500	Dead			217					Complete Remission/Response	1	217		217			0	217
TCGA-MZ	TCGA-MZ	HNSC	68	MALE	WHITE		Stage IVA	Head & N	G2	2012	-24939	Dead	WITH TUMOR		489	Distant M	Distant M	lung, mec	246		1	489	1	489			1	246
TCGA-MZ	TCGA-MZ	HNSC	51	MALE	WHITE	Stage IVA	Stage IVA	Head & Neck Squam		2013	-18974	Alive	WITH TUI	547		Distant M	Distant M	lung	339	Complete Remission/Response	0	547	0	547			1	339

TCGA-P3-	TCGA-P3-	HNSC	54	MALE	WHITE	Stage IVA	Stage IVA	Head & N	G3	2004	-19728	Alive	TUMOR F	910						Complete Remission/Response	0	910	0	910			0	910
TCGA-P3-	TCGA-P3-	HNSC	49	MALE	WHITE	Stage IVA	Stage III	Head & N	G3	2005	-17917	Dead	WITH TUMOR		480		Distant M	Lung	280	Complete Remission/Response	1	480	1	480			1	280
TCGA-P3-	TCGA-P3-	HNSC	41	MALE	[Unknown	Stage I	Stage II	Head & N	G3	2008	-15125	Alive	TUMOR F	2182		Locoregio	Oral Cavit	tongue	1601	Complete Remission/Response	0	2182	0	2182	1	1601	1	1601
TCGA-P3-	TCGA-P3-	HNSC	49	MALE	WHITE	Stage III	Stage III	Head & N	G2	2009	-18002	Alive	TUMOR F	1559						Complete Remission/Response	0	1559	0	1559	0	1559	0	1559
TCGA-P3-	TCGA-P3-	HNSC	49	MALE	WHITE	Stage IVA	Stage IVA	Head & N	G2	2009	-18035	Dead	WITH TUMOR		330		Distant M	Lung	140	[Unknown]	1	330	1	330			1	140
TCGA-P3-	TCGA-P3-	HNSC	50	MALE	WHITE	Stage IVB	Stage IVA	Head & N	G3	2005	-18536	Alive	TUMOR F	1120						Complete Remission/Response	0	1120	0	1120			0	1120
TCGA-P3-	TCGA-P3-	HNSC	67	MALE	WHITE	Stage III	Stage IVA	Head & N	G2	2005	-24715	Dead	TUMOR FREE		1430					Complete Remission/Response	1	1430	0	1430			0	1430
TCGA-P3-	TCGA-P3-	HNSC	47	FEMALE	WHITE	Stage IVA	Stage IVA	Head & N	G2	2007	-17259	Alive	TUMOR F	578		Locoregio	Oral Cavity		365	Complete Remission/Response	0	578	0	578			1	365
TCGA-P3-	TCGA-P3-	HNSC	45	MALE	WHITE	Stage IVA	Stage II	Head & N	G2	2008	-16588	Alive	TUMOR F	2298						Complete Remission/Response	0	2298	0	2298			0	2298
TCGA-P3-	TCGA-P3-	HNSC	49	MALE	WHITE	Stage IVA	Stage IVA	Head & N	G2	2008	-18143	Dead	WITH TUMOR		577	Distant M	Distant M	Lung	242	Complete Remission/Response	1	577	1	577			1	242
TCGA-P3-	TCGA-P3-	HNSC	54	MALE	WHITE	Stage IVA	Stage IVA	Head & N	G2	2008	-20027	Dead	WITH TUMOR		62					Progressive Disease	1	62	1	62			1	62
TCGA-P3-	TCGA-P3-	HNSC	79	FEMALE	WHITE	Stage IVA	Stage IVA	Head & N	G2	2009	-28977	Dead	WITH TUMOR		882	Locoregio	Oral Cavity		335	Complete Remission/Response	1	882	1	882			1	335
TCGA-P3-	TCGA-P3-	HNSC	53	MALE	WHITE	Stage IVA	Stage IVA	Head & N	G3	2009	-19694	Dead	WITH TUMOR		395	Distant M	Distant M	lung	273	Complete Remission/Response	1	395	1	395			1	273
TCGA-P3-	TCGA-P3-	HNSC	55	MALE	WHITE	Stage IVA	Stage III	Head & N	G2	2010	-20301	Dead			487					Complete Remission/Response	1	487		487			0	487
TCGA-P3-	TCGA-P3-	HNSC	54	MALE	WHITE	Stage IVA	Stage IVA	Head & N	G3	2010	-19754	Alive	TUMOR F	400						Complete Remission/Response	0	400	0	400			0	400
TCGA-OK	TCGA-OK	HNSC	79	FEMALE	WHITE	Stage IVA	Stage II	Head & N	G1	2011	-29037	Dead	WITH TUMOR		641	Locoregio	Cervical L	bilateral	553	Progressive Disease	1	641	1	641			1	553
TCGA-OK	TCGA-OK	HNSC	60	MALE	WHITE	Stage III	Stage II	Head & N	G2	2013	-22023	Alive	TUMOR F	645						Complete Remission/Response	0	645	0	645	0	645	0	645
TCGA-QK	TCGA-QK	HNSC	61	MALE	WHITE		Stage IVA	Head & N	GX	2013	-22463	Alive	TUMOR F	704		Distant M	Distant M	pulmonar	502	Complete Remission/Response	0	704	0	704			1	502
TCGA-OK	TCGA-OK	HNSC	69	MALE	WHITE	Stage III	Stage II	Head & N	G2	2013	-25334	Dead	WITH TUMOR		222	Locoregio	Cervical L	Left level I	142	Progressive Disease	1	222	1	222			1	142
TCGA-QK	TCGA-QK	HNSC	65	FEMALE	WHITE	Stage IVB	Stage IVA	Head & N	G2	2013	-23936	Alive	WITH TUM	653			Distant M	Lung	228	Progressive Disease	0	653	0	653			1	228
TCGA-OK	TCGA-OK	HNSC	52	MALE	WHITE	Stage IVA	Stage III	Head & N	G2	2013	-19113	Dead	WITH TUMOR		284	Locoregio	Cervical Lymph Node		176	Progressive Disease	1	284	1	284			1	176
TCGA-OK	TCGA-OK	HNSC	71	MALE	WHITE	Stage III	Stage II	Head & N	G3	2013	-25966	Alive	TUMOR F	387						[Unknown]	0	387	0	387			0	387
TCGA-OK	TCGA-OK	HNSC	56	MALE	WHITE	Stage II	Stage II	Head & N	G2	2012	-20461	Alive	TUMOR F	833						Complete Remission/Response	0	833	0	833	0	833	0	833
TCGA-OK	TCGA-OK	HNSC	66	MALE	WHITE	Stage IVA	Stage IVA	Head & N	G2	2013	-24144	Alive	TUMOR F	641		New Primary Tumor		Acute my	613	Complete Remission/Response	0	641	0	641			1	613
TCGA-OK	TCGA-OK	HNSC	62	FEMALE	BLACK OR	Stage IVA	Stage IVA	Head & N	G2	2013	-22865	Alive	TUMOR F	600						Complete Remission/Response	0	600	0	600			0	600
TCGA-OK	TCGA-OK	HNSC	59	MALE	BLACK OR	Stage IVA	Stage IVA	Head & Neck Squam		2013	-21711	Alive	TUMOR F	392						Complete Remission/Response	0	392	0	392			0	392
TCGA-QK	TCGA-QK	HNSC	60	FEMALE	BLACK OR	Stage IVC	Stage IVC	Head & N	G2	2013	-22107	Dead	WITH TUMOR		171					[Unknown]	1	171	1	171			1	171
TCGA-OK	TCGA-OK	HNSC	56	MALE	BLACK OR	Stage IVA	Stage IVA	Head & N	G2	2013	-20471	Dead	WITH TUMOR		449	Distant M	Distant M	esophagu	205	Complete Remission/Response	1	449	1	449			1	205
TCGA-OK	TCGA-OK	HNSC	60	MALE	BLACK OR AFRICAN		Stage IVA	Head & N	G2	2013	-21979	Dead	TUMOR FREE		371					Complete Remission/Response	1	371	0	371			0	371
TCGA-OK	TCGA-OK	HNSC	68	MALE	BLACK OR	Stage IVA	Stage IVA	Head & N	G2	2013	-25192	Alive	TUMOR F	542						Complete Remission/Response	0	542	0	542			0	542
TCGA-OK	TCGA-OK	HNSC	69	MALE	BLACK OR	Stage I	Stage I	Head & N	G3	2013	-25469	Alive	TUMOR F	466						Complete Remission/Response	0	466	0	466	0	466	0	466
TCGA-OK	TCGA-OK	HNSC	60	MALE	BLACK OR	Stage IVA	Stage IVA	Head & N	G2	2013	-22093	Alive		253						[Unknown]	0	253	0	253			0	253
TCGA-RS-	TCGA-RS-	HNSC	82	FEMALE	WHITE	Stage IVA	Stage IVA	Head & N	G2	2013	-30104	Dead	WITH TUMOR		387	Locoregio	Oral Cavit	L floor of	338	Complete Remission/Response	1	387	1	387			1	338
TCGA-RS-	TCGA-RS-	HNSC	58	MALE	WHITE	Stage II	Stage II	Head & N	G3	2013	-21393	Alive	TUMOR F	516						Complete Remission/Response	0	516	0	516	0	516	0	516
TCGA-T2-	TCGA-T2-	HNSC	73	FEMALE	WHITE		Stage III	Head & N	G1	2010	-27005	Dead	WITH TUMOR		209					Progressive Disease	1	209	1	209			1	209
TCGA-T2-	TCGA-T2-	HNSC	53	MALE	WHITE		Stage IVA	Head & N	G2	2012	-19397	Dead	WITH TUMOR		484	Distant M	Distant M	bilateral p	229	Stable Disease	1	484	1	484			1	229
TCGA-T2-	TCGA-T2-	HNSC	49	MALE	WHITE		Stage IVA	Head & N	G2	2012	-18114	Alive	TUMOR F	216						Complete Remission/Response	0	216	0	216			0	216
TCGA-T2-	TCGA-T2-	HNSC	82	MALE	WHITE	Stage III	Stage III	Head & N	G1	2011	-30013	Alive	TUMOR F	987						Complete Remission/Response	0	987	0	987	0	987	0	987
TCGA-T3-	TCGA-T3-	HNSC	52	MALE	BLACK OR	Stage IVA	Stage IVA	Head & N	G2	2013	-19247	Alive	WITH TUM	417		Distant M	Distant M	(Rt) Lung	417	[Unknown]	0	417	0	417			1	417
TCGA-T3-	TCGA-T3-	HNSC	79	MALE	BLACK OR	Stage IVA	Stage IVC	Head & N	G3	2013	-28879	Dead	WITH TUMOR		95	Distant M	Distant M	Lung	46	Progressive Disease	1	95	1	95			1	46
TCGA-TN	TCGA-TN	HNSC	56	MALE	WHITE	Stage IVA	Stage I	Head & N	G3	2013	-20666	Alive	TUMOR F	412						Complete Remission/Response	0	412	0	412			0	412
TCGA-TN	TCGA-TN	HNSC	51	MALE	WHITE	Stage IVA	Stage III	Head & N	G2	2013	-18809	Alive	TUMOR F	403						Complete Remission/Response	0	403	0	403			0	403
TCGA-TN	TCGA-TN	HNSC	59	MALE	WHITE	Stage IVA	Stage IVA	Head & N	G2	2013	-21731	Alive	TUMOR F	619						Complete Remission/Response	0	619	0	619			0	619
TCGA-UF	TCGA-UF	HNSC	62	MALE	WHITE	Stage IVA	Stage IVA	Head & N	G2	2009	-22836	Alive	TUMOR F	1971						Complete Remission/Response	0	1971	0	1971			0	1971
TCGA-UF	TCGA-UF	HNSC	54	MALE	[Unknown	Stage II	Stage III	Head & N	G1	2009	-19797	Alive	TUMOR F	1663						Complete Remission/Response	0	1663	0	1663	0	1663	0	1663
TCGA-UF	TCGA-UF	HNSC	67	MALE	WHITE	Stage IVA	Stage IVA	Head & N	G1	2009	-24623	Dead	WITH TUMOR		86					Complete Remission/Response	1	86	1	86			1	86
TCGA-UF	TCGA-UF	HNSC	67	MALE	WHITE	Stage IVA	Stage IVA	Head & N	G1	2009	-24623	Dead	WITH TUMOR		86					Complete Remission/Response	1	86	1	86			1	86
TCGA-UF	TCGA-UF	HNSC	50	MALE	WHITE	Stage IVA	Stage IVA	Head & N	G2	2010	-18404	Alive	TUMOR F	1506						Complete Remission/Response	0	1506	0	1506			0	1506
TCGA-UF	TCGA-UF	HNSC	54	FEMALE	WHITE	Stage IVA	Stage IVA	Head & N	G2	2010	-19747	Alive	TUMOR F	1461						Complete Remission/Response	0	1461	0	1461			0	1461
TCGA-UF	TCGA-UF	HNSC	63	MALE	WHITE	Stage IVA	Stage IVA	Head & N	G3	2010	-23071	Dead	TUMOR FREE		1504					Complete Remission/Response	1	1504	0	1504			0	1504
TCGA-UF	TCGA-UF	HNSC	75	MALE	WHITE	Stage IVA	Stage IVA	Head & N	G2	2011	-27459	Alive	TUMOR F	1358						Complete Remission/Response	0	1358	0	1358			0	1358
TCGA-UF	TCGA-UF	HNSC	66	FEMALE	WHITE	Stage IVA	Stage IVA	Head & N	G2	2008	-24121	Alive	TUMOR F	2265						Complete Remission/Response	0	2265	0	2265			0	2265
TCGA-UF	TCGA-UF	HNSC	42	MALE	WHITE	Stage IVA	Stage III	Head & N	G1	2012	-15452	Dead	WITH TUMOR		546		Oral Cavity		229	Complete Remission/Response	1	546	1	546			1	229
TCGA-UF	TCGA-UF	HNSC	71	MALE	[Unknown	Stage IVA	Stage IVA	Head & N	G3	2012	-26101	Dead	TUMOR FREE		739					Complete Remission/Response	1	739	0	739			0	739
TCGA-UF	TCGA-UF	HNSC	80	MALE	WHITE	Stage IVA	Stage IVA	Head & N	G2	2010	-29468	Alive	TUMOR F	1686						Complete Remission/Response	0	1686	0	1686			0	1686
TCGA-UF	TCGA-UF	HNSC	59	MALE	BLACK OR	Stage IVA	Stage IVA	Head & N	G1	2012	-21558	Alive	TUMOR F	896						Complete Remission/Response	0	896	0	896			0	896
TCGA-UF	TCGA-UF	HNSC	68	MALE	[Unknown	Stage IVA	Stage IVA	Head & N	G1	2013	-25201	Alive	TUMOR F	549						Complete Remission/Response	0	549	0	549			0	549
TCGA-UF	TCGA-UF	HNSC	59	MALE	[Unknown	Stage IVA	Stage IVA	Head & N	G2	2010	-21696	Dead	WITH TUMOR		424		Larynx		341	Complete Remission/Response	1	424	1	424			1	341
TCGA-UF	TCGA-UF	HNSC	79	FEMALE	WHITE	Stage IVA	Stage IVA	Head & N	G2	2009	-28951	Dead	WITH TUMOR		631	Locoregio	Oral Cavit	Floor of m	525	Complete Remission/Response	1	631	1	631			1	525
TCGA-UF	TCGA-UF	HNSC	59	MALE	WHITE	Stage IVA	Stage IVA	Head & N	G2	2012	-21676	Dead	WITH TUMOR		680	Locoregio	Cervical Lymph Node		519	Complete Remission/Response	1	680	1	680			1	519
TCGA-UF	TCGA-UF	HNSC	72	FEMALE	WHITE	Stage IVA	Stage IVA	Head & N	G3	2009	-26375	Dead	WITH TUMOR		993			Cervix	718	Complete Remission/Response	1	993	1	993			1	718
TCGA-UF	TCGA-UF	HNSC	62	FEMALE	WHITE	Stage IVA	Stage IVA	Head & N	G2	2011	-22985	Dead	WITH TUMOR		90		Oropharv	Oropharv	55	Complete Remission/Response	1	90	1	90			1	55
TCGA-UP	TCGA-UP	HNSC	58	MALE	WHITE		Stage IVA	Head & N	G2	2013	-21427	Alive	TUMOR F	518						Complete Remission/Response	0	518	0	518			0	518
TCGA-WA	TCGA-WA	HNSC	58	MALE	WHITE		Stage II	Head & N	G2	2012	-21207	Dead	TUMOR FREE		625					Complete Remission/Response	1	625	0	625	0	625	0	625
TCGA-WA	TCGA-WA	HNSC	58	MALE	WHITE		Stage II	Head & N	G2	2012	-21207	Dead	TUMOR FREE		625					Complete Remission/Response	1	625	0	625	0	625	0	625
TCGA-WA	TCGA-WA	HNSC	69	MALE	WHITE	Stage II	Stage II	Head & N	G3	2013	-25363	Alive	TUMOR F	443						Complete Remission/Response	0	443	0	443	0	443	0	443
TCGA-KL-	TCGA-KL-	KICH	57	FEMALE	WHITE	Stage III		Kidney Chromophob		2000	-20849	Dead	WITH TUMOR		1158				758	[Unknown]	1	1158	1	1158			1	758
TCGA-KL-	TCGA-KL-	KICH	67	FEMALE	WHITE	Stage II		Kidney Chromophob		2000	-24650	Alive	TUMOR F	4676							0	4676	0	4676			0	4676
TCGA-KL-	TCGA-KL-	KICH	67	FEMALE	WHITE	Stage II		Kidney Chromophob		2000	-24650	Alive	TUMOR F	4676							0	4676	0	4676			0	4676
TCGA-KL-	TCGA-KL-	KICH	56	FEMALE	WHITE	Stage II		Kidney Chromophob		2000	-20768	Dead	TUMOR FREE		725						1	725	0	725			0	725
TCGA-KL-	TCGA-KL-	KICH	69	MALE	WHITE	Stage III		Kidney Chromophob		2001	-25267	Alive	TUMOR F	3322						[Unknown]	0	3322	0	3322			0	3322
TCGA-KL-	TCGA-KL-	KICH	69	MALE	WHITE	Stage III		Kidney Chromophob		2001	-25267	Alive	TUMOR F	3322						[Unknown]	0	3322	0	3322			0	3322
TCGA-KL-	TCGA-KL-	KICH	46	FEMALE	WHITE	Stage I		Kidney Chromophob		2001	-17121	Alive	TUMOR F	4169						[Unknown]	0	4169	0	4169			0	4169
TCGA-KL-	TCGA-KL-	KICH	60	MALE	WHITE	Stage II		Kidney Chromophob		2003	-21976	Alive	TUMOR F	3127						[Not Evaluated]	0	3127	0	3127			0	3127
TCGA-KL-	TCGA-KL-	KICH	57	FEMALE	ASIAN	Stage I		Kidney Chromophob		2003	-20972	Alive	TUMOR F	2752						[Unknown]	0	2752	0	2752			0	2752
TCGA-KL-	TCGA-KL-	KICH	57	FEMALE	ASIAN	Stage I		Kidney Chromophob		2003	-20972	Alive	TUMOR F	2752						[Unknown]	0	2752	0	2752			0	2752
TCGA-KL-	TCGA-KL-	KICH	44	FEMALE	BLACK OR	Stage II		Kidney Chromophob		2003	-16250	Alive	TUMOR F	3304						[Unknown]	0	3304	0	3304			0	3304
TCGA-KL-	TCGA-KL-	KICH	48	FEMALE	WHITE	Stage II		Kidney Chromophob		2004	-17710	Alive	TUMOR F	3010						[Unknown]	0	3010	0	3010			0	3010
TCGA-KL-	TCGA-KL-	KICH	55	MALE	WHITE	Stage I		Kidney Chromophob		2004	-20128	Alive	TUMOR F	2582						[Unknown]	0	2582	0	2582			0	2582
TCGA-KL-	TCGA-KL-	KICH	55	MALE	WHITE	Stage I		Kidney Chromophob		2004	-20128	Alive	TUMOR F	2582						[Unknown]	0	2582	0	2582			0	2582
TCGA-KL-	TCGA-KL-	KICH	69	MALE	WHITE	Stage II		Kidney Chromophob		2005	-25229	Alive	TUMOR F	2991						[Unknown]	0	2991	0	2991			0	2991
TCGA-KL-	TCGA-KL-	KICH	37	FEMALE	WHITE	Stage III		Kidney Chromophob		2005	-13602	Alive	TUMOR F	2949						[Unknown]	0	2949	0	2949			0	2949
TCGA-KL-	TCGA-KL-	KICH	67	MALE	WHITE	Stage III		Kidney Chromophob		2005	-24832	Alive	TUMOR F	2815						[Unknown]	0	2815	0	2815			0	2815
TCGA-KL-	TCGA-KL-	KICH	51	FEMALE	WHITE	Stage IV		Kidney Chromophob		2006	-18821	Dead	WITH TUMOR		920				47	[Unknown]	1	920	1	920			1	47
TCGA-KL-	TCGA-KL-	KICH	51	FEMALE	WHITE	Stage IV		Kidney Chromophob		2006	-18821	Dead	WITH TUMOR		920				47	[Unknown]	1	920	1	920			1	47
TCGA-KL-	TCGA-KL-	KICH	26	MALE	BLACK OR	Stage II		Kidney Chromophob		2006	-9819	Alive	TUMOR F	1364						[Unknown]	0	1364	0	1364			0	1364
TCGA-KL-	TCGA-KL-	KICH	51	MALE	WHITE	Stage III		Kidney Chromophob		2006	-18667	Alive	TUMOR F	2633						[Unknown]	0	2633	0	2633			0	2633
TCGA-KL-	TCGA-KL-	KICH	67	MALE	WHITE	Stage IV		Kidney Chromophob		2006	-24545	Dead	WITH TUMOR		855				64	[Not Evaluated]	1	855	1	855			1	64
TCGA-KL-	TCGA-KL-	KICH	67	MALE	WHITE	Stage IV		Kidney Chromophob		2006	-24545	Dead	WITH TUMOR		855				64	[Not Evaluated]	1	855	1	855			1	64
TCGA-KL-	TCGA-KL-	KICH	34	MALE	WHITE	Stage II		Kidney Chromophob		2006	-12696	Alive	TUMOR F	2024						[Unknown]	0	2024	0	2024			0	2024
TCGA-KL-	TCGA-KL-	KICH	41	MALE	ASIAN	Stage IV		Kidney Chromophob		2006	-15045	Dead	WITH TUMOR		754				175	[Unknown]	1	754	1	754			1	175
TCGA-KL-	TCGA-KL-	KICH	45	FEMALE	WHITE	Stage II		Kidney Chromophob		2006	-16550	Alive	TUMOR F	2248						[Unknown]	0	2248	0	2248			0	2248
TCGA-KL-	TCGA-KL-	KICH	72	MALE	WHITE	Stage III		Kidney Chromophob		2007	-26562	Dead	WITH TUMOR		2172					[Unknown]	1	2172	1	2172			1	2172
TCGA-KL-	TCGA-KL-	KICH	51	MALE	WHITE	Stage III		Kidney Chromophob		2007	-18742	Alive	TUMOR F	876						[Unknown]	0	876	0	876			0	876
TCGA-KL-	TCGA-KL-	KICH	75	MALE	WHITE	Stage III		Kidney Chromophob		2008	-27412	Alive	TUMOR F	1665						[Unknown]	0	1665	0	1665			0	1665
TCGA-KL-	TCGA-KL-	KICH	62	MALE	WHITE	Stage II		Kidney Chromophob		2008	-22651	Alive	TUMOR F	1897						[Unknown]	0	1897	0	1897			0	1897
TCGA-KM	TCGA-KM	KICH	45	FEMALE	WHITE	Stage II		Kidney Chromophob		2000	-16683	Alive	WITH TUM	4622					2317	Complete Remission/Response	0	4622	0	4622	1	2317	1	2317
TCGA-KM	TCGA-KM	KICH	30	MALE	WHITE	Stage I		Kidney Chromophob		2011	-11266	Alive	WITH TUM	708						Partial Remission/Response	0	708	0	708			0	708
TCGA-KM	TCGA-KM	KICH	37	MALE	BLACK OR	Stage III		Kidney Chromophob		2009	-13860	Alive	TUMOR F	1366						Complete Remission/Response	0	1366	0	1366	0	1366	0	1366
TCGA-KM	TCGA-KM	KICH	61	FEMALE	WHITE	Stage I		Kidney Chromophob		2011	-22303	Alive	WITH TUM	876					876	Complete Remission/Response	0	876	0	876	1	876	1	876
TCGA-KM	TCGA-KM	KICH	38	MALE	[Unknown	Stage II		Kidney Chromophob		2009	-14212	Alive	TUMOR F	1437						Partial Remission/Response	0	1437	0	1437			0	1437
TCGA-KM	TCGA-KM	KICH	57	MALE	WHITE	Stage II		Kidney Chromophob		2009	-21183	Alive	TUMOR F	1520						Complete Remission/Response	0	1520	0	1520	0	1520	0	1520
TCGA-KM	TCGA-KM	KICH	47	MALE	WHITE	Stage I		Kidney Chromophob		2002	-17240	Alive	TUMOR F	3745						Complete Remission/Response	0	3745	0	3745	0	3745	0	3745
TCGA-KM	TCGA-KM	KICH	56	MALE	WHITE	Stage I		Kidney Chromophob		2005	-20491	Alive	TUMOR F	1553						Complete Remission/Response	0	1553	0	1553	0	1553	0	1553
TCGA-KM	TCGA-KM	KICH	67	MALE	WHITE	Stage I		Kidney Chromophob		2005	-24640	Alive	TUMOR F	953						Complete Remission/Response	0	953	0	953	0	953	0	953
TCGA-KN	TCGA-KN	KICH	41	FEMALE	WHITE	Stage II		Kidney Chromophob		2002	-15086	Alive	TUMOR F	3935							0	3935	0	3935			0	3935
TCGA-KN	TCGA-KN	KICH	43	MALE	BLACK OR	Stage II		Kidney Chromophob		2004	-15769	Alive	TUMOR F	714						Complete Remission/Response	0	714	0	714	0	714	0	714
TCGA-KN	TCGA-KN	KICH	43	MALE	BLACK OR	Stage II		Kidney Chromophob		2004	-15769	Alive	TUMOR F	714						Complete Remission/Response	0	714	0	714	0	714	0	714
TCGA-KN	TCGA-KN	KICH	47	FEMALE	WHITE	Stage II		Kidney Chromophob		2004	-17524	Alive	TUMOR F	2674							0	2674	0	2674			0	2674
TCGA-KN	TCGA-KN	KICH	28	FEMALE	WHITE	Stage I		Kidney Chromophob		2005	-10303	Alive	TUMOR F	775						Complete Remission/Response	0	775	0	775	0	775	0	775
TCGA-KN	TCGA-KN	KICH	28	FEMALE	WHITE	Stage I		Kidney Chromophob		2005	-10303	Alive	TUMOR F	775						Complete Remission/Response	0	775	0	775	0	775	0	775
TCGA-KN	TCGA-KN	KICH	44	MALE	WHITE	Stage I		Kidney Chromophob		2005	-16335	Alive	TUMOR F	788						Complete Remission/Response	0	788	0	788	0	788	0	788
TCGA-KN	TCGA-KN	KICH	44	MALE	WHITE	Stage I		Kidney Chromophob		2005	-16335	Alive	TUMOR F	788						Complete Remission/Response	0	788	0	788	0	788	0	788
TCGA-KN	TCGA-KN	KICH	42	FEMALE	WHITE	Stage I		Kidney Chromophob		2005	-15384	Alive	TUMOR F	1983						Complete Remission/Response	0	1983	0	1983	0	1983	0	1983
TCGA-KN	TCGA-KN	KICH	42	FEMALE	WHITE	Stage I		Kidney Chromophob		2005	-15384	Alive	TUMOR F	1983						Complete Remission/Response	0	1983	0	1983	0	1983	0	1983
TCGA-KN	TCGA-KN	KICH	53	MALE	WHITE	Stage I		Kidney Chromophob		2005	-19692	Alive	TUMOR F	3179						Complete Remission/Response	0	3179	0	3179	0	3179	0	3179
TCGA-KN	TCGA-KN	KICH	53	MALE	WHITE	Stage I		Kidney Chromophob		2005	-19692	Alive	TUMOR F	3179						Complete Remission/Response	0	3179	0	3179	0	3179	0	3179
TCGA-KN	TCGA-KN	KICH	50	MALE	WHITE	Stage IV		Kidney Chromophob		2006	-18345	Alive	WITH TUM	108						Progressive Disease	0	108	0	108			0	108
TCGA-KN	TCGA-KN	KICH	50	MALE	WHITE	Stage IV		Kidney Chromophob		2006	-18345	Alive	WITH TUM	108						Progressive Disease	0	108	0	108			0	108
TCGA-KN	TCGA-KN	KICH	54	MALE	WHITE	Stage IV		Kidney Chromophob		2006	-19854	Alive	TUMOR F	30							0	30	0	30			0	30
TCGA-KN	TCGA-KN	KICH	54	MALE	WHITE	Stage IV		Kidney Chromophob		2006	-19854	Alive	TUMOR F	30							0	30	0	30			0	30
TCGA-KN	TCGA-KN	KICH	71	MALE	WHITE	Stage II		Kidney Chromophob		2006	-26252	Dead	WITH TUMOR		1590				478	Stable Disease	1	1590	1	1590			1	478
TCGA-KN	TCGA-KN	KICH	71	MALE	WHITE	Stage II		Kidney Chromophob		2006	-26252	Dead	WITH TUMOR		1590				478	Stable Disease	1	1590	1	1590			1	478
TCGA-KN	TCGA-KN	KICH	38	FEMALE	WHITE	Stage III		Kidney Chromophob		2006	-13903	Alive	TUMOR F	2650					1989	Complete Remission/Response	0	2650	0	2650	1	1989	1	1989
TCGA-KN	TCGA-KN	KICH	38	FEMALE	WHITE	Stage III		Kidney Chromophob		2006	-13903	Alive	TUMOR F	2650					1989	Complete Remission/Response	0	2650	0	2650	1	1989	1	1989
TCGA-KN	TCGA-KN	KICH	75	MALE	WHITE	Stage I		Kidney Chromophob		2007	-27467	Dead	WITH TUMOR							[Unknown]	1		1				1	
TCGA-KN	TCGA-KN	KICH	75	MALE	WHITE	Stage I		Kidney Chromophob		2007	-27467	Dead	WITH TUMOR							[Unknown]	1		1				1	
TCGA-KN	TCGA-KN	KICH	35	FEMALE	[Not Eval	Stage II		Kidney Chromophob		2007	-13043	Alive	TUMOR F	588							0	588	0	588			0	588
TCGA-KN	TCGA-KN	KICH	35	FEMALE	[Not Eval	Stage II		Kidney Chromophob		2007	-13043	Alive	TUMOR F	588							0	588	0	588			0	588
TCGA-KN	TCGA-KN	KICH	86	FEMALE	WHITE	Stage II		Kidney Chromophob		2010	-31591	Alive		76						Complete Remission/Response	0	76	0	76	0	76	0	76
TCGA-KN	TCGA-KN	KICH	86	FEMALE	WHITE	Stage II		Kidney Chromophob		2010	-31591	Alive		76						Complete Remission/Response	0	76	0	76	0	76	0	76
TCGA-KN	TCGA-KN	KICH	46	FEMALE	WHITE	Stage III		Kidney Chromophob		2008	-17108	Alive	TUMOR F	341						Complete Remission/Response	0	341	0	341	0	341	0	341
TCGA-KN	TCGA-KN	KICH	46	FEMALE	WHITE	Stage III		Kidney Chromophob		2008	-17108	Alive	TUMOR F	341						Complete Remission/Response	0	341	0	341	0	341	0	341
TCGA-KN	TCGA-KN	KICH	65	FEMALE	WHITE	Stage II		Kidney Chromophob		2009	-23934	Alive	TUMOR F	1797						Complete Remission/Response	0	1797	0	1797	0	1797	0	1797
TCGA-KN	TCGA-KN	KICH	65	FEMALE	WHITE	Stage II		Kidney Chromophob		2009	-23934	Alive	TUMOR F	1797						Complete Remission/Response	0	1797	0	1797	0	1797	0	1797
TCGA-KN	TCGA-KN	KICH	59	MALE	WHITE	Stage II		Kidney Chromophob		2004	-21891	Alive	TUMOR F	3498						Complete Remission/Response	0	3498	0	3498	0	3498	0	3498
TCGA-KN	TCGA-KN	KICH	59	MALE	WHITE	Stage II		Kidney Chromophob		2004	-21891	Alive	TUMOR F	3498						Complete Remission/Response	0	3498	0	3498	0	3498	0	3498
TCGA-KN	TCGA-KN	KICH	62	MALE	WHITE	Stage II		Kidney Chromophob		2009	-22997	Alive	TUMOR F	1288						Complete Remission/Response	0	1288	0	1288	0	1288	0	1288
TCGA-KN	TCGA-KN	KICH	62	MALE	WHITE	Stage II		Kidney Chromophob		2009	-22997	Alive	TUMOR F	1288						Complete Remission/Response	0	1288	0	1288	0	1288	0	1288
TCGA-KN	TCGA-KN	KICH	42	FEMALE	WHITE	Stage I		Kidney Chromophob		2006	-15475	Alive	TUMOR F	3068						Complete Remission/Response	0	3068	0	3068	0	3068	0	3068
TCGA-KN	TCGA-KN	KICH	42	FEMALE	WHITE	Stage I		Kidney Chromophob		2006	-15475	Alive	TUMOR F	3068						Complete Remission/Response	0	3068	0	3068	0	3068	0	3068
TCGA-KO	TCGA-KO	KICH	49	MALE	WHITE	Stage I		Kidney Chromophob		2005	-18171	Alive	TUMOR F	3208							0	3208	0	3208			0	3208
TCGA-KO	TCGA-KO	KICH	49	MALE	WHITE	Stage I		Kidney Chromophob		2005	-18171	Alive	TUMOR F	3208							0	3208	0	3208			0	3208
TCGA-KO	TCGA-KO	KICH	78	MALE	WHITE	Stage IV		Kidney Chromophob		2005	-28705	Dead	WITH TUMOR		325				148	Progressive Disease	1	325	1	325			1	148
TCGA-KO	TCGA-KO	KICH	29	MALE	WHITE	Stage III		Kidney Chromophob		2005	-10671	Alive	TUMOR F	3056						Complete Remission/Response	0	3056	0	3056	0	3056	0	3056
TCGA-KO	TCGA-KO	KICH	17	FEMALE	WHITE	Stage I		Kidney Chromophob		2005	-6556	Alive	TUMOR F	3145						Complete Remission/Response	0	3145	0	3145	0	3145	0	3145
TCGA-KO	TCGA-KO	KICH	42	MALE	WHITE	Stage II		Kidney Chromophob		2005	-15407	Alive	TUMOR F	2820						Complete Remission/Response	0	2820	0	2820	0	2820	0	2820
TCGA-KO	TCGA-KO	KICH	47	MALE	WHITE	Stage III		Kidney Chromophob		2004	-17299	Dead	TUMOR FREE		507				46		1	507	0	507			1	46
TCGA-KO	TCGA-KO	KICH	69	MALE	WHITE	Stage II		Kidney Chromophob		2004	-25419	Alive	TUMOR F	3180						Complete Remission/Response	0	3180	0	3180	0	3180	0	3180
TCGA-KO	TCGA-KO	KICH	74	FEMALE	WHITE	Stage I		Kidney Chromophob		2004	-27084	Alive	TUMOR F	3136							0	3136	0	3136			0	3136
TCGA-KO	TCGA-KO	KICH	58	MALE	WHITE	Stage I		Kidney Chromophob		2004	-21371	Alive	TUMOR F	3958						Complete Remission/Response	0	3958	0	3958	0	3958	0	3958
TCGA-KO	TCGA-KO	KICH	46	MALE	WHITE	Stage I		Kidney Chromophob		2006	-17067	Alive	TUMOR F	3038						Complete Remission/Response	0	3038	0	3038	0	3038	0	3038
TCGA-KO	TCGA-KO	KICH	50	FEMALE	WHITE	Stage II		Kidney Chromophob		2006	-18502	Alive	TUMOR F	3212						Complete Remission/Response	0	3212	0	3212	0	3212	0	3212
TCGA-KO	TCGA-KO	KICH	44	FEMALE	WHITE	Stage I		Kidney Chromophob		2007	-16395	Alive	TUMOR F	2939						Complete Remission/Response	0	2939	0	2939	0	2939	0	2939
TCGA-KO	TCGA-KO	KICH	44	FEMALE	WHITE	Stage I		Kidney Chromophob		2007	-16395	Alive	TUMOR F	2939						Complete Remission/Response	0	2939	0	2939	0	2939	0	2939
TCGA-KO	TCGA-KO	KICH	41	MALE	WHITE	Stage III		Kidney Chromophob		2007	-15261	Alive	TUMOR F	2752						Complete Remission/Response	0	2752	0	2752	0	2752	0	2752
TCGA-KO	TCGA-KO	KICH	30	FEMALE	WHITE	Stage I		Kidney Chromophob		2007	-11139	Alive	TUMOR F	2815						Complete Remission/Response	0	2815	0	2815	0	2815	0	2815
TCGA-3Z	TCGA-3Z	KIRC	69	MALE	BLACK OR	Stage I		Kidney Cl	G2	2013	-25205	Alive	TUMOR F	385						Complete Remission/Response	0	385	0	385	0	385	0	385
TCGA-6D	TCGA-6D	KIRC	68	FEMALE	BLACK OR	Stage I		Kidney Cl	G2	2013	-25043	Alive	TUMOR F	362						[Unknown]	0	362	0	362			0	362
TCGA-A3	TCGA-A3	KIRC	67	MALE	WHITE	Stage I		Kidney Cl	G3	2005	-24569	Alive		1120							0	1120	0	1120			0	1120
TCGA-A3	TCGA-A3	KIRC	67	MALE	WHITE	Stage I		Kidney Cl	G3	2005	-24569	Alive		1120							0	1120	0	1120			0	1120
TCGA-A3	TCGA-A3	KIRC	66	MALE		Stage III		Kidney Cl	G3	2005	-24315	Alive	WITH TUM	1436							0	1436	0	1436			0	1436
TCGA-A3	TCGA-A3	KIRC	77	FEMALE	WHITE	Stage III		Kidney Cl	G2	2006	-28287	Alive	TUMOR F	16							0	16	0	16			0	16
TCGA-A3	TCGA-A3	KIRC	77	FEMALE	WHITE	Stage III		Kidney Cl	G2	2006	-28287	Alive	TUMOR F	16							0	16	0	16			0	16
TCGA-A3	TCGA-A3	KIRC	57	MALE		Stage I		Kidney Cl	G2	2005	-21183	Dead	TUMOR FREE		1191						1	1191	0	1191			0	1191
TCGA-A3	TCGA-A3	KIRC	57	MALE		Stage I		Kidney Cl	G2	2005	-21183	Dead	TUMOR FREE		1191						1	1191	0	1191			0	1191
TCGA-A3	TCGA-A3	KIRC	59	MALE	BLACK OR	Stage I		Kidney Cl	G3	2005	-21556	Dead	TUMOR F	735	735						1	735	0	735			0	735
TCGA-A3	TCGA-A3	KIRC	59	MALE	BLACK OR	Stage I		Kidney Cl	G3	2005	-21556	Dead	TUMOR F	735	735						1	735	0	735			0	735
TCGA-A3	TCGA-A3	KIRC	57	MALE	WHITE	Stage II		Kidney Cl	G3	2005	-20931	Alive	TUMOR F	1493							0	1493	0	1493			0	1493
TCGA-A3	TCGA-A3	KIRC	57	MALE	WHITE	Stage II		Kidney Cl	G3	2005	-20931	Alive	TUMOR F	1493							0	1493	0	1493			0	1493
TCGA-A3	TCGA-A3	KIRC	67	MALE		Stage II		Kidney Cl	G2	2005	-24803	Alive	TUMOR F	1491					560		0	1491	0	1491			1	560
TCGA-A3	TCGA-A3	KIRC	67	MALE		Stage II		Kidney Cl	G2	2005	-24803	Alive	TUMOR F	1491					560		0	1491	0	1491			1	560
TCGA-A3	TCGA-A3	KIRC	70	MALE	WHITE	Stage I		Kidney Cl	G2	2006	-25699	Alive	TUMOR F	1130							0	1130	0	1130			0	1130
TCGA-A3	TCGA-A3	KIRC	70	MALE	WHITE	Stage I		Kidney Cl	G2	2006	-25699	Alive	TUMOR F	1130							0	1130	0	1130			0	1130
TCGA-A3	TCGA-A3	KIRC	52	FEMALE	WHITE	Stage I		Kidney Cl	G1	2005	-19138	Alive	TUMOR F	1508							0	1508	0	1508			0	1508
TCGA-A3	TCGA-A3	KIRC	52	FEMALE	WHITE	Stage I		Kidney Cl	G1	2005	-19138	Alive	TUMOR F	1508							0	1508	0	1508			0	1508
TCGA-A3	TCGA-A3	KIRC	51	MALE	WHITE	Stage I		Kidney Cl	G2	2006	-18754	Alive	TUMOR F	1478							0	1478	0	1478			0	1478
TCGA-A3	TCGA-A3	KIRC	51	MALE	WHITE	Stage I		Kidney Cl	G2	2006	-18754	Alive	TUMOR F	1478							0	1478	0	1478			0	1478
TCGA-A3	TCGA-A3	KIRC	53	MALE	WHITE	Stage I		Kidney Cl	G1	2006	-19456	Alive	TUMOR F	1106							0	1106	0	1106			0	1106
TCGA-A3	TCGA-A3	KIRC	53	MALE	WHITE	Stage I		Kidney Cl	G1	2006	-19456	Alive	TUMOR F	1106							0	1106	0	1106			0	1106
TCGA-A3	TCGA-A3	KIRC	51	MALE	WHITE	Stage I		Kidney Cl	G3	2006	-18843	Alive	TUMOR F	1186							0	1186	0	1186			0	1186
TCGA-A3	TCGA-A3	KIRC	51	MALE	WHITE	Stage I		Kidney Cl	G3	2006	-18843	Alive	TUMOR F	1186							0	1186	0	1186			0	1186
TCGA-A3	TCGA-A3	KIRC	52	MALE	WHITE	Stage I		Kidney Cl	G2	2006	-19126	Dead	TUMOR FREE		1170					Partial Remission/Response	1	1170	0	1170			0	1170
TCGA-A3	TCGA-A3	KIRC	52	MALE	WHITE	Stage I		Kidney Cl	G2	2006	-19126	Dead	TUMOR FREE		1170					Partial Remission/Response	1	1170	0	1170			0	1170
TCGA-A3	TCGA-A3	KIRC	47	MALE	WHITE	Stage I		Kidney Cl	G1	2006	-17285	Alive	TUMOR F	1137							0	1137	0	1137			0	1137
TCGA-A3	TCGA-A3	KIRC	47	MALE	WHITE	Stage I		Kidney Cl	G1	2006	-17285	Alive	TUMOR F	1137							0	1137	0	1137			0	1137
TCGA-A3	TCGA-A3	KIRC	79	MALE	WHITE	Stage I		Kidney Cl	G2	2007	-29168	Alive	TUMOR F	1385						Complete Remission/Response	0	1385	0	1385	0	1385	0	1385
TCGA-A3	TCGA-A3	KIRC	79	MALE	WHITE	Stage I		Kidney Cl	G2	2007	-29168	Alive	TUMOR F	1385						Complete Remission/Response	0	1385	0	1385	0	1385	0	1385
TCGA-A3	TCGA-A3	KIRC	75	MALE	WHITE	Stage I		Kidney Cl	G2	2007	-27650	Alive	TUMOR F	1624						Complete Remission/Response	0	1624	0	1624	0	1624	0	1624
TCGA-A3	TCGA-A3	KIRC	75	MALE	WHITE	Stage I		Kidney Cl	G2	2007	-27650	Alive	TUMOR F	1624						Complete Remission/Response	0	1624	0	1624	0	1624	0	1624
TCGA-A3	TCGA-A3	KIRC	86	FEMALE	WHITE	Stage I		Kidney Cl	G2	2005	-31615	Alive	TUMOR F	1485							0	1485	0	1485			0	1485
TCGA-A3	TCGA-A3	KIRC	86	FEMALE	WHITE	Stage I		Kidney Cl	G2	2005	-31615	Alive	TUMOR F	1485							0	1485	0	1485			0	1485
TCGA-A3	TCGA-A3	KIRC	41	MALE	WHITE	Stage II		Kidney Cl	G4	2006	-15250	Alive	TUMOR F	1886					1098	Complete Remission/Response	0	1886	0	1886	1	1098	1	1098
TCGA-A3	TCGA-A3	KIRC	41	MALE	WHITE	Stage II		Kidney Cl	G4	2006	-15250	Alive	TUMOR F	1886					1098	Complete Remission/Response	0	1886	0	1886	1	1098	1	1098
TCGA-A3	TCGA-A3	KIRC	75	FEMALE	WHITE	Stage I		Kidney Cl	G4	2007	-27538	Alive	WITH TUM	1043							0	1043	0	1043			0	1043
TCGA-A3	TCGA-A3	KIRC	75	FEMALE	WHITE	Stage I		Kidney Cl	G4	2007	-27538	Alive	WITH TUM	1043							0	1043	0	1043			0	1043
TCGA-A3	TCGA-A3	KIRC	79	MALE	WHITE	Stage II		Kidney Cl	G3	2005	-28930	Alive	TUMOR F	945							0	945	0	945			0	945
TCGA-A3	TCGA-A3	KIRC	79	MALE	WHITE	Stage II		Kidney Cl	G3	2005	-28930	Alive	TUMOR F	945							0	945	0	945			0	945
TCGA-A3	TCGA-A3	KIRC	68	MALE	WHITE	Stage I		Kidney Cl	G3	2005	-25118	Dead	WITH TUMOR		137				114	Progressive Disease	1	137	1	137			1	114
TCGA-A3	TCGA-A3	KIRC	68	MALE	WHITE	Stage I		Kidney Cl	G3	2005	-25118	Dead	WITH TUMOR		137				114	Progressive Disease	1	137	1	137			1	114
TCGA-A3	TCGA-A3	KIRC	76	FEMALE	WHITE	Stage III		Kidney Cl	G2	2005	-27966	Dead	WITH TUMOR		1610				610		1	1610	1	1610			1	610
TCGA-A3	TCGA-A3	KIRC	76	FEMALE	WHITE	Stage III		Kidney Cl	G2	2005	-27966	Dead	WITH TUMOR		1610				610		1	1610	1	1610			1	610
TCGA-A3	TCGA-A3	KIRC	34	FEMALE	WHITE	Stage I		Kidney Cl	G2	2006	-12431	Alive	TUMOR F	1385							0	1385	0	1385			0	1385
TCGA-A3	TCGA-A3	KIRC	34	FEMALE	WHITE	Stage I		Kidney Cl	G2	2006	-12431	Alive	TUMOR F	1385							0	1385	0	1385			0	1385
TCGA-A3	TCGA-A3	KIRC	42	MALE	WHITE	Stage II		Kidney Cl	G2	2007	-15562	Alive	TUMOR F	910							0	910	0	910			0	910
TCGA-A3	TCGA-A3	KIRC	42	MALE	WHITE	Stage II		Kidney Cl	G2	2007	-15562	Alive	TUMOR F	910							0	910	0	910			0	910
TCGA-A3	TCGA-A3	KIRC	74	MALE	WHITE	Stage III		Kidney Cl	G3	2005		Dead	TUMOR FREE		561					Complete Remission/Response	1	561	0	561	0	561	0	561
TCGA-A3	TCGA-A3	KIRC	74	MALE	WHITE	Stage III		Kidney Cl	G3	2005		Dead	TUMOR FREE		561					Complete Remission/Response	1	561	0	561	0	561	0	561
TCGA-A3	TCGA-A3	KIRC	62	MALE	WHITE	Stage II		Kidney Cl	G3	2005	-22825	Alive	TUMOR F	2688						Complete Remission/Response	0	2688	0	2688	0	2688	0	2688
TCGA-A3	TCGA-A3	KIRC	62	MALE	WHITE	Stage II		Kidney Cl	G3	2005	-22825	Alive	TUMOR F	2688						Complete Remission/Response	0	2688	0	2688	0	2688	0	2688
TCGA-A3	TCGA-A3	KIRC	57	FEMALE	WHITE	Stage I		Kidney Cl	G2	2005	-21087	Alive	TUMOR F	1307							0	1307	0	1307			0	1307
TCGA-A3	TCGA-A3	KIRC	57	FEMALE	WHITE	Stage I		Kidney Cl	G2	2005	-21087	Alive	TUMOR F	1307							0	1307	0	1307			0	1307
TCGA-A3	TCGA-A3	KIRC	82	FEMALE	WHITE	Stage I		Kidney Cl	G2	2005	-30269	Alive	TUMOR F	2504						Complete Remission/Response	0	2504	0	2504	0	2504	0	2504
TCGA-A3	TCGA-A3	KIRC	82	FEMALE	WHITE	Stage I		Kidney Cl	G2	2005	-30269	Alive	TUMOR F	2504						Complete Remission/Response	0	2504	0	2504	0	2504	0	2504
TCGA-A3	TCGA-A3	KIRC	60	FEMALE	WHITE	Stage I		Kidney Cl	G2	2005	-21988	Alive	TUMOR F	1559							0	1559	0	1559			0	1559
TCGA-A3	TCGA-A3	KIRC	60	FEMALE	WHITE	Stage I		Kidney Cl	G2	2005	-21988	Alive	TUMOR F	1559							0	1559	0	1559			0	1559
TCGA-A3	TCGA-A3	KIRC	50	MALE	ASIAN	Stage II		Kidney Cl	G2	2006	-18596	Alive	TUMOR F	319							0	319	0	319			0	319
TCGA-A3	TCGA-A3	KIRC	50	MALE	ASIAN	Stage II		Kidney Cl	G2	2006	-18596	Alive	TUMOR F	319							0	319	0	319			0	319
TCGA-A3	TCGA-A3	KIRC	46	MALE	WHITE	Stage I		Kidney Cl	G2	2006	-16915	Alive		873							0	873	0	873			0	873
TCGA-A3	TCGA-A3	KIRC	46	MALE	WHITE	Stage I		Kidney Cl	G2	2006	-16915	Alive		873							0	873	0	873			0	873
TCGA-A3	TCGA-A3	KIRC	72	MALE	WHITE	Stage I		Kidney Cl	G3	2006	-26489	Alive	TUMOR F	2270						Complete Remission/Response	0	2270	0	2270	0	2270	0	2270
TCGA-A3	TCGA-A3	KIRC	72	MALE	WHITE	Stage I		Kidney Cl	G3	2006	-26489	Alive	TUMOR F	2270						Complete Remission/Response	0	2270	0	2270	0	2270	0	2270
TCGA-A3	TCGA-A3	KIRC	48	FEMALE	WHITE	Stage I		Kidney Cl	G2	2006	-17621	Alive	TUMOR F	2274						Complete Remission/Response	0	2274	0	2274	0	2274	0	2274
TCGA-A3	TCGA-A3	KIRC	48	FEMALE	WHITE	Stage I		Kidney Cl	G2	2006	-17621	Alive	TUMOR F	2274						Complete Remission/Response	0	2274	0	2274	0	2274	0	2274
TCGA-A3	TCGA-A3	KIRC	64	MALE	WHITE	Stage III		Kidney Cl	G2	2007	-23528	Alive	TUMOR F	735							0	735	0	735			0	735
TCGA-A3	TCGA-A3	KIRC	64	MALE	WHITE	Stage III		Kidney Cl	G2	2007	-23528	Alive	TUMOR F	735							0	735	0	735			0	735
TCGA-A3	TCGA-A3	KIRC	54	FEMALE	WHITE	Stage I		Kidney Cl	G3	2007	-19964	Alive	TUMOR F	1621						Complete Remission/Response	0	1621	0	1621	0	1621	0	1621
TCGA-A3	TCGA-A3	KIRC	54	FEMALE	WHITE	Stage I		Kidney Cl	G3	2007	-19964	Alive	TUMOR F	1621						Complete Remission/Response	0	1621	0	1621	0	1621	0	1621
TCGA-A3	TCGA-A3	KIRC	51	FEMALE	BLACK OR	Stage I		Kidney Cl	G2	2005	-18890	Alive		1314							0	1314	0	1314			0	1314
TCGA-A3	TCGA-A3	KIRC	51	FEMALE	BLACK OR	Stage I		Kidney Cl	G2	2005	-18890	Alive		1314							0	1314	0	1314			0	1314
TCGA-A3	TCGA-A3	KIRC	51	MALE	BLACK OR	Stage I		Kidney Cl	G2	2006	-18954	Dead	TUMOR FREE		1696					Complete Remission/Response	1	1696	0	1696	0	1696	0	1696
TCGA-A3	TCGA-A3	KIRC	51	MALE	BLACK OR	Stage I		Kidney Cl	G2	2006	-18954	Dead	TUMOR FREE		1696					Complete Remission/Response	1	1696	0	1696	0	1696	0	1696
TCGA-A3	TCGA-A3	KIRC	60	MALE	WHITE	Stage I		Kidney Cl	G3	2006	-22090	Alive	TUMOR F	630							0	630	0	630			0	630
TCGA-A3	TCGA-A3	KIRC	60	MALE	WHITE	Stage I		Kidney Cl	G3	2006	-22090	Alive	TUMOR F	630							0	630	0	630			0	630
TCGA-A3	TCGA-A3	KIRC	54	MALE		Stage I		Kidney Cl	G2	2007	-19828	Alive	TUMOR F	567							0	567	0	567			0	567
TCGA-A3	TCGA-A3	KIRC	54	MALE		Stage I		Kidney Cl	G2	2007	-19828	Alive	TUMOR F	567							0	567	0	567			0	567
TCGA-A3	TCGA-A3	KIRC	69	MALE	WHITE	Stage I		Kidney Cl	G3	2007	-25207	Alive	WITH TUM	574					514		0	574	0	574			1	514
TCGA-A3	TCGA-A3	KIRC	69	MALE	WHITE	Stage I		Kidney Cl	G3	2007	-25207	Alive	WITH TUM	574					514		0	574	0	574			1	514
TCGA-A3	TCGA-A3	KIRC	52	MALE	WHITE	Stage I		Kidney Cl	G2	2006	-19324	Alive		861							0	861	0	861			0	861
TCGA-A3	TCGA-A3	KIRC	52	MALE	WHITE	Stage I		Kidney Cl	G2	2006	-19324	Alive		861							0	861	0	861			0	861
TCGA-A3	TCGA-A3	KIRC	46	FEMALE	WHITE	Stage I		Kidney Cl	G2	2007	-17114	Alive	TUMOR F	1993						Complete Remission/Response	0	1993	0	1993	0	1993	0	1993
TCGA-A3	TCGA-A3	KIRC	46	FEMALE	WHITE	Stage I		Kidney Cl	G2	2007	-17114	Alive	TUMOR F	1993						Complete Remission/Response	0	1993	0	1993	0	1993	0	1993
TCGA-A3	TCGA-A3	KIRC	49	MALE	WHITE	Stage I		Kidney Cl	G2	2006	-17920	Alive	TUMOR F	617							0	617	0	617			0	617
TCGA-A3	TCGA-A3	KIRC	49	MALE	WHITE	Stage I		Kidney Cl	G2	2006	-17920	Alive	TUMOR F	617							0	617	0	617			0	617
TCGA-A3	TCGA-A3	KIRC	47	MALE	BLACK OR	Stage I		Kidney Cl	G3	2011	-17190	Alive	TUMOR F	1018						Complete Remission/Response	0	1018	0	1018	0	1018	0	1018
TCGA-A3	TCGA-A3	KIRC	57	FEMALE	BLACK OR	Stage I		Kidney Cl	G1	2012	-20918	Alive	TUMOR F	468						Complete Remission/Response	0	468	0	468	0	468	0	468
TCGA-A3	TCGA-A3	KIRC	49	FEMALE	BLACK OR	Stage I		Kidney Cl	G2	2012	-17952	Alive	TUMOR F	689						Complete Remission/Response	0	689	0	689	0	689	0	689
TCGA-A3	TCGA-A3	KIRC	78	MALE	BLACK OR	Stage I		Kidney Cl	G2	2012	-28689	Alive	TUMOR F	3						Complete Remission/Response	0	3	0	3	0	3	0	3
TCGA-A3	TCGA-A3	KIRC	59	FEMALE	BLACK OR	Stage I		Kidney Cl	G2	2011	-21572	Alive	TUMOR F	3						[Unknown]	0	3	0	3			0	3
TCGA-A3	TCGA-A3	KIRC	74	FEMALE	BLACK OR	Stage I		Kidney Cl	G1	2011	-27377	Alive	TUMOR F	0						Complete Remission/Response	0	0	0	0	0	0	0	0
TCGA-A3	TCGA-A3	KIRC	75	MALE	BLACK OR	Stage I		Kidney Cl	G2	2011	-27526	Alive	TUMOR F	340						Complete Remission/Response	0	340	0	340	0	340	0	340
TCGA-A3	TCGA-A3	KIRC	37	MALE	BLACK OR	Stage III		Kidney Cl	G2	2011	-13546	Alive	TUMOR F	323						Complete Remission/Response	0	323	0	323	0	323	0	323
TCGA-A3	TCGA-A3	KIRC	65	FEMALE	BLACK OR	Stage I		Kidney Cl	G3	2012	-23998	Alive	TUMOR F	0						Complete Remission/Response	0	0	0	0	0	0	0	0
TCGA-AK	TCGA-AK	KIRC	68	MALE	WHITE	Stage I		Kidney Cl	G2	2000	-24912	Alive	TUMOR F	3343							0	3343	0	3343			0	3343
TCGA-AK	TCGA-AK	KIRC	37	MALE	WHITE	Stage III		Kidney Cl	G3	2004	-13770	Dead	WITH TUMOR		885				256		1	885	1	885			1	256
TCGA-AK	TCGA-AK	KIRC	65	MALE	WHITE	Stage I		Kidney Cl	GX	2004	-24067	Alive	TUMOR F	3583						Complete Remission/Response	0	3583	0	3583	0	3583	0	3583
TCGA-AK	TCGA-AK	KIRC	62	MALE	WHITE	Stage III		Kidney Cl	G2	2004	-22653	Alive	TUMOR F	3728						Complete Remission/Response	0	3728	0	3728	0	3728	0	3728
TCGA-AK	TCGA-AK	KIRC	54	FEMALE	WHITE	Stage II		Kidney Cl	G2	2004	-19849	Alive	TUMOR F	3328						Complete Remission/Response	0	3328	0	3328	0	3328	0	3328
TCGA-AK	TCGA-AK	KIRC	61	MALE	WHITE	Stage III		Kidney Cl	G3	2004	-22612	Dead			480						1	480		480			0	480
TCGA-AK	TCGA-AK	KIRC	62	FEMALE	WHITE	Stage II		Kidney Cl	G3	2004	-22852	Dead	TUMOR FREE		2241					Complete Remission/Response	1	2241	0	2241	0	2241	0	2241
TCGA-AK	TCGA-AK	KIRC	48	FEMALE	WHITE	Stage II		Kidney Cl	GX	2004	-17853	Alive	TUMOR F	3409						Complete Remission/Response	0	3409	0	3409	0	3409	0	3409
TCGA-AK	TCGA-AK	KIRC	72	MALE	WHITE	Stage I		Kidney Cl	G2	2004	-26490	Alive	WITH TUM	2087					1640		0	2087	0	2087			1	1640
TCGA-AK	TCGA-AK	KIRC	40	MALE	WHITE	Stage IV		Kidney Cl	G2	2005	-14842	Alive	TUMOR F	3331					1189	Progressive Disease	0	3331	0	3331			1	1189
TCGA-AK	TCGA-AK	KIRC	58	MALE	WHITE	Stage I		Kidney Cl	G3	2005	-21337	Alive	TUMOR F	2865						Complete Remission/Response	0	2865	0	2865	0	2865	0	2865
TCGA-AK	TCGA-AK	KIRC	45	MALE	WHITE	Stage II		Kidney Cl	GX	2006	-16578	Alive	TUMOR F	1423							0	1423	0	1423			0	1423
TCGA-AK	TCGA-AK	KIRC	80	FEMALE	WHITE	Stage I		Kidney Cl	G2	2006	-29497	Alive	TUMOR F	1471							0	1471	0	1471			0	1471
TCGA-AK	TCGA-AK	KIRC	69	MALE	WHITE	Stage III		Kidney Cl	G3	2006	-25494	Alive	TUMOR F	2392						Complete Remission/Response	0	2392	0	2392	0	2392	0	2392
TCGA-AK	TCGA-AK	KIRC	83	MALE	WHITE	Stage II		Kidney Cl	G2	2006	-30676	Alive	TUMOR F	1217							0	1217	0	1217			0	1217
TCGA-AK	TCGA-AK	KIRC	85	FEMALE	WHITE	Stage I		Kidney Cl	G2	2006	-31364	Alive	TUMOR F	1779							0	1779	0	1779			0	1779
TCGA-AK	TCGA-AK	KIRC	48	MALE	WHITE	Stage II		Kidney Cl	G3	2006	-17859	Alive	TUMOR F	2868						Complete Remission/Response	0	2868	0	2868	0	2868	0	2868
TCGA-AK	TCGA-AK	KIRC	58	FEMALE	WHITE	Stage II		Kidney Cl	G2	2006	-21271	Alive	TUMOR F	2531						Complete Remission/Response	0	2531	0	2531	0	2531	0	2531
TCGA-AK	TCGA-AK	KIRC	84	MALE	WHITE	Stage I		Kidney Cl	G3	2006	-30862	Alive	TUMOR F	874							0	874	0	874			0	874
TCGA-AK	TCGA-AK	KIRC	71	FEMALE	WHITE	Stage III		Kidney Cl	G3	2007	-26221	Dead			683						1	683		683			0	683
TCGA-AK	TCGA-AK	KIRC	48	MALE	BLACK OR	Stage II		Kidney Cl	G3	2007	-17821	Alive		1143							0	1143	0	1143			0	1143
TCGA-AK	TCGA-AK	KIRC	48	MALE	WHITE	Stage I		Kidney Cl	G3	2007	-17557	Alive	TUMOR F	1168							0	1168	0	1168			0	1168
TCGA-AK	TCGA-AK	KIRC	58	MALE	WHITE	Stage I		Kidney Cl	G2	2007	-21281	Alive	TUMOR F	2508						Complete Remission/Response	0	2508	0	2508	0	2508	0	2508
TCGA-AK	TCGA-AK	KIRC	72	MALE	WHITE	Stage I		Kidney Cl	G2	2007	-26464	Alive	TUMOR F	2217						Complete Remission/Response	0	2217	0	2217	0	2217	0	2217
TCGA-AK	TCGA-AK	KIRC	71	FEMALE	BLACK OR	Stage I		Kidney Cl	GX	2008	-26232	Alive	TUMOR F	369							0	369	0	369			0	369
TCGA-AS	TCGA-AS	KIRC	63	MALE	WHITE	Stage I		Kidney Clear Cell Rer		2009	-23039	Alive	TUMOR F	1238						[Unknown]	0	1238	0	1238			0	1238
TCGA-AS	TCGA-AS	KIRC	35	MALE	WHITE	Stage I		Kidney Cl	G1	2010	-12937	Alive	TUMOR F	43						[Unknown]	0	43	0	43			0	43
TCGA-B0	TCGA-B0	KIRC	46	MALE	WHITE	Stage IV		Kidney Cl	G4	2008	-16804	Dead	WITH TUMOR		101						1	101	1	101			1	101
TCGA-B0	TCGA-B0	KIRC	46	MALE	WHITE	Stage IV		Kidney Cl	G4	2008	-16804	Dead	WITH TUMOR		101						1	101	1	101			1	101
TCGA-B0	TCGA-B0	KIRC	65	MALE	WHITE	Stage IV		Kidney Cl	G3	2008	-23985	Dead	WITH TUMOR		43						1	43	1	43			1	43
TCGA-B0	TCGA-B0	KIRC	65	MALE	WHITE	Stage IV		Kidney Cl	G3	2008	-23985	Dead	WITH TUMOR		43						1	43	1	43			1	43
TCGA-B0	TCGA-B0	KIRC	55	MALE	WHITE	Stage IV		Kidney Cl	G3	2009	-20176	Dead	WITH TUMOR		139						1	139	1	139			1	139
TCGA-B0	TCGA-B0	KIRC	55	MALE	WHITE	Stage IV		Kidney Cl	G3	2009	-20176	Dead	WITH TUMOR		139						1	139	1	139			1	139
TCGA-B0	TCGA-B0	KIRC	72	FEMALE	WHITE	Stage III		Kidney Cl	G4	2009	-26659	Dead	TUMOR FREE		77						1	77	0	77			0	77
TCGA-B0	TCGA-B0	KIRC	72	FEMALE	WHITE	Stage III		Kidney Cl	G4	2009	-26659	Dead	TUMOR FREE		77						1	77	0	77			0	77
TCGA-B0	TCGA-B0	KIRC	72	MALE	WHITE	Stage III		Kidney Cl	G4	2009	-26582	Dead	TUMOR FREE		106						1	106	0	106			0	106
TCGA-B0	TCGA-B0	KIRC	72	MALE	WHITE	Stage III		Kidney Cl	G4	2009	-26582	Dead	TUMOR FREE		106						1	106	0	106			0	106

TCGA-B0	TCGA-B0	KIRC	58	MALE	WHITE	Stage III	Kidney Cl	G3	2001	-21317	Dead	WITH TUMOR		866	794		1	866	1	866			1	794
TCGA-B0	TCGA-B0	KIRC	58	MALE	WHITE	Stage III	Kidney Cl	G3	2001	-21317	Dead	WITH TUMOR		866	794		1	866	1	866			1	794
TCGA-B0	TCGA-B0	KIRC	46	FEMALE	WHITE	Stage IV	Kidney Cl	G4	2003	-16844	Dead	WITH TUMOR		578			1	578	1	578			1	578
TCGA-B0	TCGA-B0	KIRC	46	FEMALE	WHITE	Stage IV	Kidney Cl	G4	2003	-16844	Dead	WITH TUMOR		578			1	578	1	578			1	578
TCGA-B0	TCGA-B0	KIRC	75	MALE	WHITE	Stage IV	Kidney Cl	G4	2003	-27417	Dead	WITH TUMOR		42			1	42	1	42			1	42
TCGA-B0	TCGA-B0	KIRC	75	MALE	WHITE	Stage IV	Kidney Cl	G4	2003	-27417	Dead	WITH TUMOR		42			1	42	1	42			1	42
TCGA-B0	TCGA-B0	KIRC	74	MALE	WHITE	Stage IV	Kidney Cl	G4	2004	-27225	Dead	[Discrepancy]		110			1	110		110			0	110
TCGA-B0	TCGA-B0	KIRC	74	MALE	WHITE	Stage IV	Kidney Cl	G4	2004	-27225	Dead	[Discrepancy]		110			1	110		110			0	110
TCGA-B0	TCGA-B0	KIRC	60	MALE	WHITE	Stage IV	Kidney Cl	G4	2004	-22034	Dead	WITH TUMOR		1980			1	1980	1	1980			1	1980
TCGA-B0	TCGA-B0	KIRC	60	MALE	WHITE	Stage IV	Kidney Cl	G4	2004	-22034	Dead	WITH TUMOR		1980			1	1980	1	1980			1	1980
TCGA-B0	TCGA-B0	KIRC	66	FEMALE	WHITE	Stage IV	Kidney Cl	G3	2005	-24232	Dead	WITH TUMOR		238			1	238	1	238			1	238
TCGA-B0	TCGA-B0	KIRC	66	FEMALE	WHITE	Stage IV	Kidney Cl	G3	2005	-24232	Dead	WITH TUMOR		238			1	238	1	238			1	238
TCGA-B0	TCGA-B0	KIRC	51	MALE	WHITE	Stage IV	Kidney Cl	G4	2005	-18921	Dead	WITH TUMOR		182			1	182	1	182			1	182
TCGA-B0	TCGA-B0	KIRC	51	MALE	WHITE	Stage IV	Kidney Cl	G4	2005	-18921	Dead	WITH TUMOR		182			1	182	1	182			1	182
TCGA-B0	TCGA-B0	KIRC	61	MALE	WHITE	Stage III	Kidney Cl	G4	2007	-22331	Dead	TUMOR F	26	65			1	65	0	65			0	65
TCGA-B0	TCGA-B0	KIRC	61	MALE	WHITE	Stage III	Kidney Cl	G4	2007	-22331	Dead	TUMOR F	26	65			1	65	0	65			0	65
TCGA-B0	TCGA-B0	KIRC	63	MALE	WHITE	Stage III	Kidney Cl	G4	2008	-23322	Dead	WITH TUMOR		600			1	600	1	600			1	600
TCGA-B0	TCGA-B0	KIRC	63	MALE	WHITE	Stage III	Kidney Cl	G4	2008	-23322	Dead	WITH TUMOR		600			1	600	1	600			1	600
TCGA-B0	TCGA-B0	KIRC	75	FEMALE	WHITE	Stage III	Kidney Cl	G3	2010	-27396	Alive	TUMOR F	1755			Complete Remission/Response	0	1755	0	1755	0	1755	0	1755
TCGA-B0	TCGA-B0	KIRC	75	FEMALE	WHITE	Stage III	Kidney Cl	G3	2010	-27396	Alive	TUMOR F	1755			Complete Remission/Response	0	1755	0	1755	0	1755	0	1755
TCGA-B0	TCGA-B0	KIRC	76	MALE	WHITE	Stage IV	Kidney Cl	G3	2006	-28095	Dead	WITH TUMOR		1337			1	1337	1	1337			1	1337
TCGA-B0	TCGA-B0	KIRC	76	MALE	WHITE	Stage IV	Kidney Cl	G3	2006	-28095	Dead	WITH TUMOR		1337			1	1337	1	1337			1	1337
TCGA-B0	TCGA-B0	KIRC	76	FEMALE	WHITE	Stage III	Kidney Cl	G2	2010	-27899	Dead	WITH TUMOR		202			1	202	1	202			1	202
TCGA-B0	TCGA-B0	KIRC	76	FEMALE	WHITE	Stage III	Kidney Cl	G2	2010	-27899	Dead	WITH TUMOR		202			1	202	1	202			1	202
TCGA-B0	TCGA-B0	KIRC	81	MALE	WHITE	Stage IV	Kidney Cl	G3	2010	-29873	Dead	WITH TUMOR		99			1	99	1	99			1	99
TCGA-B0	TCGA-B0	KIRC	81	MALE	WHITE	Stage IV	Kidney Cl	G3	2010	-29873	Dead	WITH TUMOR		99			1	99	1	99			1	99
TCGA-B0	TCGA-B0	KIRC	57	MALE	WHITE	Stage III	Kidney Cl	G2	2010	-20910	Alive	WITH TUM	1778		768	Progressive Disease	0	1778	0	1778			1	768
TCGA-B0	TCGA-B0	KIRC	57	MALE	WHITE	Stage III	Kidney Cl	G2	2010	-20910	Alive	WITH TUM	1778		768	Progressive Disease	0	1778	0	1778			1	768
TCGA-B0	TCGA-B0	KIRC	47	MALE	WHITE	Stage III	Kidney Cl	G3	2001	-17402	Dead	WITH TUMOR		478	205		1	478	1	478			1	205
TCGA-B0	TCGA-B0	KIRC	47	MALE	WHITE	Stage III	Kidney Cl	G3	2001	-17402	Dead	WITH TUMOR		478	205		1	478	1	478			1	205
TCGA-B0	TCGA-B0	KIRC	48	MALE	WHITE	Stage III	Kidney Cl	G3	2001	-17792	Dead	WITH TUMOR		1417	458		1	1417	1	1417			1	458
TCGA-B0	TCGA-B0	KIRC	48	MALE	WHITE	Stage III	Kidney Cl	G3	2001	-17792	Dead	WITH TUMOR		1417	458		1	1417	1	1417			1	458
TCGA-B0	TCGA-B0	KIRC	68	MALE	WHITE	Stage III	Kidney Cl	G3	2001	-25018	Dead	TUMOR F	18	18			1	18	0	18			0	18
TCGA-B0	TCGA-B0	KIRC	68	MALE	WHITE	Stage III	Kidney Cl	G3	2001	-25018	Dead	TUMOR F	18	18			1	18	0	18			0	18
TCGA-B0	TCGA-B0	KIRC	58	MALE	WHITE	Stage IV	Kidney Cl	G3	2001	-21374	Dead	WITH TUMOR		168			1	168	1	168			1	168
TCGA-B0	TCGA-B0	KIRC	58	MALE	WHITE	Stage IV	Kidney Cl	G3	2001	-21374	Dead	WITH TUMOR		168			1	168	1	168			1	168
TCGA-B0	TCGA-B0	KIRC	65	MALE	WHITE	Stage III	Kidney Cl	G4	2001	-23873	Dead	WITH TUMOR		1588			1	1588	1	1588			1	1588
TCGA-B0	TCGA-B0	KIRC	65	MALE	WHITE	Stage III	Kidney Cl	G4	2001	-23873	Dead	WITH TUMOR		1588			1	1588	1	1588			1	1588
TCGA-B0	TCGA-B0	KIRC	49	MALE	WHITE	Stage II	Kidney Cl	G3	2002	-18133	Dead	WITH TUMOR		1371			1	1371	1	1371			1	1371
TCGA-B0	TCGA-B0	KIRC	49	MALE	WHITE	Stage II	Kidney Cl	G3	2002	-18133	Dead	WITH TUMOR		1371			1	1371	1	1371			1	1371
TCGA-B0	TCGA-B0	KIRC	81	MALE	WHITE	Stage III	Kidney Cl	G3	2002	-29873	Dead	TUMOR FREE		1019			1	1019	0	1019			0	1019
TCGA-B0	TCGA-B0	KIRC	81	MALE	WHITE	Stage III	Kidney Cl	G3	2002	-29873	Dead	TUMOR FREE		1019			1	1019	0	1019			0	1019
TCGA-B0	TCGA-B0	KIRC	68	FEMALE	WHITE	Stage II	Kidney Cl	G3	2002	-24853	Dead	WITH TUMOR		510	127		1	510	1	510			1	127
TCGA-B0	TCGA-B0	KIRC	68	FEMALE	WHITE	Stage II	Kidney Cl	G3	2002	-24853	Dead	WITH TUMOR		510	127		1	510	1	510			1	127
TCGA-B0	TCGA-B0	KIRC	60	FEMALE	WHITE	Stage IV	Kidney Cl	G4	2002	-22220	Dead	TUMOR F	183	183			1	183	0	183			0	183
TCGA-B0	TCGA-B0	KIRC	60	FEMALE	WHITE	Stage IV	Kidney Cl	G4	2002	-22220	Dead	TUMOR F	183	183			1	183	0	183			0	183
TCGA-B0	TCGA-B0	KIRC	68	FEMALE	ASIAN	Stage III	Kidney Cl	G3	2002	-25013	Dead	WITH TUMOR		1230			1	1230	1	1230			1	1230
TCGA-B0	TCGA-B0	KIRC	68	FEMALE	ASIAN	Stage III	Kidney Cl	G3	2002	-25013	Dead	WITH TUMOR		1230			1	1230	1	1230			1	1230
TCGA-B0	TCGA-B0	KIRC	78	MALE	WHITE	Stage II	Kidney Cl	G4	2002	-28774	Dead	WITH TUMOR		1111			1	1111	1	1111			1	1111
TCGA-B0	TCGA-B0	KIRC	78	MALE	WHITE	Stage II	Kidney Cl	G4	2002	-28774	Dead	WITH TUMOR		1111			1	1111	1	1111			1	1111
TCGA-B0	TCGA-B0	KIRC	88	MALE	WHITE	Stage I	Kidney Cl	G2	2002	-32290	Dead	TUMOR FREE		454			1	454	0	454			0	454
TCGA-B0	TCGA-B0	KIRC	88	MALE	WHITE	Stage I	Kidney Cl	G2	2002	-32290	Dead	TUMOR FREE		454			1	454	0	454			0	454
TCGA-B0	TCGA-B0	KIRC	49	FEMALE	WHITE	Stage I	Kidney Cl	G3	2002	-18132	Dead	TUMOR FREE		1657			1	1657	0	1657			0	1657
TCGA-B0	TCGA-B0	KIRC	49	FEMALE	WHITE	Stage I	Kidney Cl	G3	2002	-18132	Dead	TUMOR FREE		1657			1	1657	0	1657			0	1657
TCGA-B0	TCGA-B0	KIRC	77	FEMALE	WHITE	Stage III	Kidney Cl	G4	2002	-28467	Dead	WITH TUMOR		885	668		1	885	1	885			1	668
TCGA-B0	TCGA-B0	KIRC	77	FEMALE	WHITE	Stage III	Kidney Cl	G4	2002	-28467	Dead	WITH TUMOR		885	668		1	885	1	885			1	668
TCGA-B0	TCGA-B0	KIRC	79	MALE	WHITE	Stage IV	Kidney Cl	G3	2003	-28948	Dead	WITH TUMOR		307			1	307	1	307			1	307
TCGA-B0	TCGA-B0	KIRC	79	MALE	WHITE	Stage IV	Kidney Cl	G3	2003	-28948	Dead	WITH TUMOR		307			1	307	1	307			1	307
TCGA-B0	TCGA-B0	KIRC	82	FEMALE	WHITE	Stage I	Kidney Cl	G2	2003	-30190	Dead	WITH TUMOR		2386			1	2386	1	2386			1	2386
TCGA-B0	TCGA-B0	KIRC	82	FEMALE	WHITE	Stage I	Kidney Cl	G2	2003	-30190	Dead	WITH TUMOR		2386			1	2386	1	2386			1	2386
TCGA-B0	TCGA-B0	KIRC	49	MALE	WHITE	Stage I	Kidney Cl	G3	2003	-18224	Dead	TUMOR FREE		2090			1	2090	0	2090			0	2090
TCGA-B0	TCGA-B0	KIRC	49	MALE	WHITE	Stage I	Kidney Cl	G3	2003	-18224	Dead	TUMOR FREE		2090			1	2090	0	2090			0	2090
TCGA-B0	TCGA-B0	KIRC	61	MALE	WHITE	Stage IV	Kidney Cl	G3	2003	-22488	Dead	WITH TUMOR		1238			1	1238	1	1238			1	1238
TCGA-B0	TCGA-B0	KIRC	61	MALE	WHITE	Stage IV	Kidney Cl	G3	2003	-22488	Dead	WITH TUMOR		1238			1	1238	1	1238			1	1238
TCGA-B0	TCGA-B0	KIRC	63	MALE	WHITE	Stage I	Kidney Cl	G3	2003	-23129	Dead	WITH TUMOR		1378	1208		1	1378	1	1378			1	1208
TCGA-B0	TCGA-B0	KIRC	63	MALE	WHITE	Stage I	Kidney Cl	G3	2003	-23129	Dead	WITH TUMOR		1378	1208		1	1378	1	1378			1	1208
TCGA-B0	TCGA-B0	KIRC	69	FEMALE	WHITE	Stage I	Kidney Cl	G3	2003	-25268	Dead	TUMOR FREE		834			1	834	0	834			0	834
TCGA-B0	TCGA-B0	KIRC	69	FEMALE	WHITE	Stage I	Kidney Cl	G3	2003	-25268	Dead	TUMOR FREE		834			1	834	0	834			0	834
TCGA-B0	TCGA-B0	KIRC	80	FEMALE	WHITE	Stage I	Kidney Cl	G2	2003	-29380	Dead	TUMOR FREE		1639			1	1639	0	1639			0	1639
TCGA-B0	TCGA-B0	KIRC	80	FEMALE	WHITE	Stage I	Kidney Cl	G2	2003	-29380	Dead	TUMOR FREE		1639			1	1639	0	1639			0	1639
TCGA-B0	TCGA-B0	KIRC	63	MALE	WHITE	Stage IV	Kidney Cl	G3	2004	-23097	Dead	WITH TUMOR		204	108		1	204	1	204			1	108
TCGA-B0	TCGA-B0	KIRC	63	MALE	WHITE	Stage IV	Kidney Cl	G3	2004	-23097	Dead	WITH TUMOR		204	108		1	204	1	204			1	108
TCGA-B0	TCGA-B0	KIRC	73	FEMALE	WHITE	Stage III	Kidney Cl	G4	2004	-27019	Dead	WITH TUMOR		1724	250		1	1724	1	1724			1	250
TCGA-B0	TCGA-B0	KIRC	73	FEMALE	WHITE	Stage III	Kidney Cl	G4	2004	-27019	Dead	WITH TUMOR		1724	250		1	1724	1	1724			1	250
TCGA-B0	TCGA-B0	KIRC	57	MALE	WHITE	Stage III	Kidney Cl	G3	2004	-21088	Dead	WITH TUMOR		320			1	320	1	320			1	320
TCGA-B0	TCGA-B0	KIRC	57	MALE	WHITE	Stage III	Kidney Cl	G3	2004	-21088	Dead	WITH TUMOR		320			1	320	1	320			1	320
TCGA-B0	TCGA-B0	KIRC	60	MALE	WHITE	Stage IV	Kidney Cl	G3	2004	-22220	Dead	WITH TUMOR		313	62		1	313	1	313			1	62
TCGA-B0	TCGA-B0	KIRC	60	MALE	WHITE	Stage IV	Kidney Cl	G3	2004	-22220	Dead	WITH TUMOR		313	62		1	313	1	313			1	62
TCGA-B0	TCGA-B0	KIRC	70	MALE	WHITE	Stage IV	Kidney Cl	G2	2003	-25582	Dead	WITH TUMOR		1986	748		1	1986	1	1986			1	748
TCGA-B0	TCGA-B0	KIRC	70	MALE	WHITE	Stage IV	Kidney Cl	G2	2003	-25582	Dead	WITH TUMOR		1986	748		1	1986	1	1986			1	748
TCGA-B0	TCGA-B0	KIRC	52	MALE	WHITE	Stage IV	Kidney Cl	G2	2004	-19139	Dead	WITH TUMOR		1200	163		1	1200	1	1200			1	163
TCGA-B0	TCGA-B0	KIRC	52	MALE	WHITE	Stage IV	Kidney Cl	G2	2004	-19139	Dead	WITH TUMOR		1200	163		1	1200	1	1200			1	163
TCGA-B0	TCGA-B0	KIRC	60	MALE	WHITE	Stage IV	Kidney Cl	G3	2004	-22147	Dead	[Discrepancy]		793			1	793		793			0	793
TCGA-B0	TCGA-B0	KIRC	60	MALE	WHITE	Stage IV	Kidney Cl	G3	2004	-22147	Dead	[Discrepancy]		793			1	793		793			0	793
TCGA-B0	TCGA-B0	KIRC	54	MALE	WHITE	Stage III	Kidney Cl	G3	2004	-19892	Dead	WITH TUMOR		883	494		1	883	1	883			1	494
TCGA-B0	TCGA-B0	KIRC	54	MALE	WHITE	Stage III	Kidney Cl	G3	2004	-19892	Dead	WITH TUMOR		883	494		1	883	1	883			1	494
TCGA-B0	TCGA-B0	KIRC	51	MALE	WHITE	Stage III	Kidney Cl	G3	2004	-18843	Dead	WITH TUMOR		69	4		1	69	1	69			1	4
TCGA-B0	TCGA-B0	KIRC	51	MALE	WHITE	Stage III	Kidney Cl	G3	2004	-18843	Dead	WITH TUMOR		69	4		1	69	1	69			1	4
TCGA-B0	TCGA-B0	KIRC	78	FEMALE	WHITE	Stage II	Kidney Cl	G2	2004	-28552	Dead	WITH TUMOR		1121	860		1	1121	1	1121			1	860
TCGA-B0	TCGA-B0	KIRC	78	FEMALE	WHITE	Stage II	Kidney Cl	G2	2004	-28552	Dead	WITH TUMOR		1121	860		1	1121	1	1121			1	860
TCGA-B0	TCGA-B0	KIRC	75	FEMALE	WHITE	Stage I	Kidney Cl	G2	2003	-27523	Dead	TUMOR FREE		2145			1	2145	0	2145			0	2145
TCGA-B0	TCGA-B0	KIRC	75	FEMALE	WHITE	Stage I	Kidney Cl	G2	2003	-27523	Dead	TUMOR FREE		2145			1	2145	0	2145			0	2145
TCGA-B0	TCGA-B0	KIRC	77	FEMALE	WHITE	Stage III	Kidney Cl	G2	2005	-28205	Dead	TUMOR FREE		637			1	637	0	637			0	637
TCGA-B0	TCGA-B0	KIRC	77	FEMALE	WHITE	Stage III	Kidney Cl	G2	2005	-28205	Dead	TUMOR FREE		637			1	637	0	637			0	637
TCGA-B0	TCGA-B0	KIRC	77	MALE	WHITE	Stage I	Kidney Cl	G3	2005	-28405	Dead	TUMOR FREE		1317			1	1317	0	1317			0	1317
TCGA-B0	TCGA-B0	KIRC	77	MALE	WHITE	Stage I	Kidney Cl	G3	2005	-28405	Dead	TUMOR FREE		1317			1	1317	0	1317			0	1317
TCGA-B0	TCGA-B0	KIRC	63	MALE	WHITE	Stage IV	Kidney Cl	G3	2005	-23052	Dead	WITH TUMOR		342	103		1	342	1	342			1	103
TCGA-B0	TCGA-B0	KIRC	63	MALE	WHITE	Stage IV	Kidney Cl	G3	2005	-23052	Dead	WITH TUMOR		342	103		1	342	1	342			1	103
TCGA-B0	TCGA-B0	KIRC	79	FEMALE	WHITE	Stage III	Kidney Cl	G2	2005	-29219	Dead	WITH TUMOR		362	213		1	362	1	362			1	213
TCGA-B0	TCGA-B0	KIRC	79	FEMALE	WHITE	Stage III	Kidney Cl	G2	2005	-29219	Dead	WITH TUMOR		362	213		1	362	1	362			1	213
TCGA-B0	TCGA-B0	KIRC	63	MALE	WHITE	Stage I	Kidney Cl	G3	2005	-23034	Dead	WITH TUMOR		1045			1	1045	1	1045			1	1045
TCGA-B0	TCGA-B0	KIRC	63	MALE	WHITE	Stage I	Kidney Cl	G3	2005	-23034	Dead	WITH TUMOR		1045			1	1045	1	1045			1	1045
TCGA-B0	TCGA-B0	KIRC	33	MALE	WHITE	Stage IV	Kidney Cl	G3	2006	-12219	Dead	WITH TUMOR		222	65		1	222	1	222			1	65
TCGA-B0	TCGA-B0	KIRC	33	MALE	WHITE	Stage IV	Kidney Cl	G3	2006	-12219	Dead	WITH TUMOR		222	65		1	222	1	222			1	65
TCGA-B0	TCGA-B0	KIRC	76	FEMALE	WHITE	Stage III	Kidney Cl	G3	2005	-27793	Dead	TUMOR FREE		770			1	770	0	770			0	770
TCGA-B0	TCGA-B0	KIRC	76	FEMALE	WHITE	Stage III	Kidney Cl	G3	2005	-27793	Dead	TUMOR FREE		770			1	770	0	770			0	770
TCGA-B0	TCGA-B0	KIRC	53	MALE	WHITE	Stage I	Kidney Cl	G3	2006	-19677	Dead	TUMOR FREE		563			1	563	0	563			0	563
TCGA-B0	TCGA-B0	KIRC	53	MALE	WHITE	Stage I	Kidney Cl	G3	2006	-19677	Dead	TUMOR FREE		563			1	563	0	563			0	563
TCGA-B0	TCGA-B0	KIRC	53	FEMALE	WHITE	Stage IV	Kidney Cl	G3	2008	-19611	Dead	WITH TUMOR		459			1	459	1	459			1	459
TCGA-B0	TCGA-B0	KIRC	53	FEMALE	WHITE	Stage IV	Kidney Cl	G3	2008	-19611	Dead	WITH TUMOR		459			1	459	1	459			1	459
TCGA-B0	TCGA-B0	KIRC	62	MALE	WHITE	Stage IV	Kidney Cl	G2	2008	-22965	Dead	WITH TUMOR		333	75		1	333	1	333			1	75
TCGA-B0	TCGA-B0	KIRC	62	MALE	WHITE	Stage IV	Kidney Cl	G2	2008	-22965	Dead	WITH TUMOR		333	75		1	333	1	333			1	75
TCGA-B0	TCGA-B0	KIRC	81	MALE	WHITE	Stage III	Kidney Cl	G3	2008	-29921	Dead	TUMOR FREE		245			1	245	0	245			0	245
TCGA-B0	TCGA-B0	KIRC	81	MALE	WHITE	Stage III	Kidney Cl	G3	2008	-29921	Dead	TUMOR FREE		245			1	245	0	245			0	245
TCGA-B0	TCGA-B0	KIRC	72	FEMALE	WHITE	Stage III	Kidney Cl	GX	1998	-26574	Dead	TUMOR FREE		68			1	68	0	68			0	68
TCGA-B0	TCGA-B0	KIRC	72	FEMALE	WHITE	Stage III	Kidney Cl	GX	1998	-26574	Dead	TUMOR FREE		68			1	68	0	68			0	68
TCGA-B0	TCGA-B0	KIRC	59	FEMALE	WHITE	Stage III	Kidney Cl	G2	2010	-21852	Alive	WITH TUM	665		359		0	665	0	665			1	359
TCGA-B0	TCGA-B0	KIRC	59	FEMALE	WHITE	Stage III	Kidney Cl	G2	2010	-21852	Alive	WITH TUM	665		359		0	665	0	665			1	359
TCGA-B0	TCGA-B0	KIRC	53	FEMALE	BLACK OI	Stage I	Kidney Cl	G3	1998	-19620	Dead	[Discrepancy]		1584			1	1584		1584			0	1584
TCGA-B0	TCGA-B0	KIRC	53	FEMALE	BLACK OI	Stage I	Kidney Cl	G3	1998	-19620	Dead	[Discrepancy]		1584			1	1584		1584			0	1584
TCGA-B0	TCGA-B0	KIRC	88	FEMALE	WHITE	Stage III	Kidney Cl	G3	2002	-32364	Dead	[Discrepancy]		485			1	485		485			0	485
TCGA-B0	TCGA-B0	KIRC	88	FEMALE	WHITE	Stage III	Kidney Cl	G3	2002	-32364	Dead	[Discrepancy]		485			1	485		485			0	485
TCGA-B0	TCGA-B0	KIRC	72	MALE	BLACK OI	Stage III	Kidney Cl	G3	1998	-26478	Dead	WITH TUMOR		1913	1426		1	1913	1	1913			1	1426
TCGA-B0	TCGA-B0	KIRC	72	MALE	BLACK OI	Stage III	Kidney Cl	G3	1998	-26478	Dead	WITH TUMOR		1913	1426		1	1913	1	1913			1	1426
TCGA-B0	TCGA-B0	KIRC	74	FEMALE	WHITE	Stage I	Kidney Cl	G3	1999	-27152	Dead	[Discrepancy]		2764	2734		1	2764		2764			1	2734
TCGA-B0	TCGA-B0	KIRC	74	FEMALE	WHITE	Stage I	Kidney Cl	G3	1999	-27152	Dead	[Discrepancy]		2764	2734		1	2764		2764			1	2734
TCGA-B0	TCGA-B0	KIRC	90	FEMALE	WHITE	Stage I	Kidney Cl	G2	2002	-32873	Dead	TUMOR FREE		2752			1	2752	0	2752			0	2752
TCGA-B0	TCGA-B0	KIRC	90	FEMALE	WHITE	Stage I	Kidney Cl	G2	2002	-32873	Dead	TUMOR FREE		2752			1	2752	0	2752			0	2752
TCGA-B0	TCGA-B0	KIRC	64	MALE	WHITE	Stage I	Kidney Cl	G2	2004	-23547	Dead	TUMOR FREE		1598			1	1598	0	1598			0	1598
TCGA-B0	TCGA-B0	KIRC	64	MALE	WHITE	Stage I	Kidney Cl	G2	2004	-23547	Dead	TUMOR FREE		1598			1	1598	0	1598			0	1598
TCGA-B0	TCGA-B0	KIRC	65	FEMALE	WHITE	Stage IV	Kidney Cl	G4	2007	-23929	Dead	WITH TUMOR		927	549		1	927	1	927			1	549
TCGA-B0	TCGA-B0	KIRC	65	FEMALE	WHITE	Stage IV	Kidney Cl	G4	2007	-23929	Dead	WITH TUMOR		927	549		1	927	1	927			1	549
TCGA-B0	TCGA-B0	KIRC	54	MALE	WHITE	Stage III	Kidney Cl	G2	2009	-19932	Alive	TUMOR F	1782			Complete Remission/Response	0	1782	0	1782	0	1782	0	1782
TCGA-B0	TCGA-B0	KIRC	54	MALE	WHITE	Stage III	Kidney Cl	G2	2009	-19932	Alive	TUMOR F	1782			Complete Remission/Response	0	1782	0	1782	0	1782	0	1782
TCGA-B0	TCGA-B0	KIRC	69	MALE	WHITE	Stage III	Kidney Cl	G4	2009	-25428	Dead	WITH TUMOR		587	70		1	587	1	587			1	70
TCGA-B0	TCGA-B0	KIRC	69	MALE	WHITE	Stage III	Kidney Cl	G4	2009	-25428	Dead	WITH TUMOR		587	70		1	587	1	587			1	70
TCGA-B0	TCGA-B0	KIRC	71	FEMALE	WHITE	Stage I	Kidney Cl	G2	2009	-25976	Alive	TUMOR F	2009			Complete Remission/Response	0	2009	0	2009	0	2009	0	2009
TCGA-B0	TCGA-B0	KIRC	71	FEMALE	WHITE	Stage I	Kidney Cl	G2	2009	-25976	Alive	TUMOR F	2009			Complete Remission/Response	0	2009	0	2009	0	2009	0	2009
TCGA-B0	TCGA-B0	KIRC	69	FEMALE	WHITE	Stage III	Kidney Cl	G2	2010	-25369	Alive	TUMOR F	1175			Complete Remission/Response	0	1175	0	1175	0	1175	0	1175
TCGA-B0	TCGA-B0	KIRC	69	FEMALE	WHITE	Stage III	Kidney Cl	G2	2010	-25369	Alive	TUMOR F	1175			Complete Remission/Response	0	1175	0	1175	0	1175	0	1175
TCGA-B0	TCGA-B0	KIRC	43	MALE	WHITE	Stage IV	Kidney Cl	G3	2009	-15935	Alive	WITH TUM	1604		75	Progressive Disease	0	1604	0	1604			1	75
TCGA-B0	TCGA-B0	KIRC	43	MALE	WHITE	Stage IV	Kidney Cl	G3	2009	-15935	Alive	WITH TUM	1604		75	Progressive Disease	0	1604	0	1604			1	75
TCGA-B0	TCGA-B0	KIRC	52	MALE	WHITE	Stage III	Kidney Cl	G3	2010	-19352	Alive	WITH TUM	1274		1129	Complete Remission/Response	0	1274	0	1274	1	1129	1	1129
TCGA-B0	TCGA-B0	KIRC	52	MALE	WHITE	Stage III	Kidney Cl	G3	2010	-19352	Alive	WITH TUM	1274		1129	Complete Remission/Response	0	1274	0	1274	1	1129	1	1129
TCGA-B0	TCGA-B0	KIRC	40	MALE	WHITE	Stage I	Kidney Cl	G2	2010	-14612	Alive	TUMOR F	1608			Complete Remission/Response	0	1608	0	1608	0	1608	0	1608
TCGA-B0	TCGA-B0	KIRC	40	MALE	WHITE	Stage I	Kidney Cl	G2	2010	-14612	Alive	TUMOR F	1608			Complete Remission/Response	0	1608	0	1608	0	1608	0	1608
TCGA-B0	TCGA-B0	KIRC	61	FEMALE	WHITE	Stage I	Kidney Cl	G2	2010	-22607	Alive	TUMOR F	1552			Complete Remission/Response	0	1552	0	1552	0	1552	0	1552
TCGA-B0	TCGA-B0	KIRC	61	FEMALE	WHITE	Stage I	Kidney Cl	G2	2010	-22607	Alive	TUMOR F	1552			Complete Remission/Response	0	1552	0	1552	0	1552	0	1552
TCGA-B0	TCGA-B0	KIRC	72	FEMALE	WHITE	Stage I	Kidney Cl	G2	2010	-26559	Alive	TUMOR F	1169			Complete Remission/Response	0	1169	0	1169	0	1169	0	1169
TCGA-B0	TCGA-B0	KIRC	72	FEMALE	WHITE	Stage I	Kidney Cl	G2	2010	-26559	Alive	TUMOR F	1169			Complete Remission/Response	0	1169	0	1169	0	1169	0	1169
TCGA-B0	TCGA-B0	KIRC	56	MALE	WHITE	Stage I	Kidney Cl	G2	2010	-20635	Alive	TUMOR F	1485			Complete Remission/Response	0	1485	0	1485	0	1485	0	1485
TCGA-B0	TCGA-B0	KIRC	56	MALE	WHITE	Stage I	Kidney Cl	G2	2010	-20635	Alive	TUMOR F	1485			Complete Remission/Response	0	1485	0	1485	0	1485	0	1485
TCGA-B0	TCGA-B0	KIRC	46	MALE	WHITE	Stage I	Kidney Cl	G2	2010	-17145	Alive	TUMOR F	1411			Complete Remission/Response	0	1411	0	1411	0	1411	0	1411
TCGA-B0	TCGA-B0	KIRC	59	FEMALE	WHITE	Stage III	Kidney Cl	G4	2008	-21819	Alive	TUMOR F	1733			Complete Remission/Response	0	1733	0	1733	0	1733	0	1733
TCGA-B0	TCGA-B0	KIRC	59	FEMALE	WHITE	Stage III	Kidney Cl	G4	2008	-21819	Alive	TUMOR F	1733			Complete Remission/Response	0	1733	0	1733	0	1733	0	1733
TCGA-B0	TCGA-B0	KIRC	64	MALE	WHITE	Stage IV	Kidney Cl	G4	2010	-23477	Alive	WITH TUM	1290		444	Progressive Disease	0	1290	0	1290			1	444
TCGA-B0	TCGA-B0	KIRC	64	MALE	WHITE	Stage IV	Kidney Cl	G4	2010	-23477	Alive	WITH TUM	1290		444	Progressive Disease	0	1290	0	1290			1	444
TCGA-B0	TCGA-B0	KIRC	53	FEMALE	WHITE	Stage I	Kidney Cl	G1	2004	-19685	Alive	WITH TUM	3392			Partial Remission/Response	0	3392	0	3392			0	3392
TCGA-B0	TCGA-B0	KIRC	53	FEMALE	WHITE	Stage I	Kidney Cl	G1	2004	-19685	Alive	WITH TUM	3392			Partial Remission/Response	0	3392	0	3392			0	3392
TCGA-B0	TCGA-B0	KIRC	66	FEMALE	WHITE	Stage I	Kidney Cl	G3	2002	-24120	Alive	WITH TUM	3431		3250		0	3431	0	3431			1	3250
TCGA-B0	TCGA-B0	KIRC	66	FEMALE	WHITE	Stage I	Kidney Cl	G3	2002	-24120	Alive	WITH TUM	3431		3250		0	3431	0	3431			1	3250
TCGA-B0	TCGA-B0	KIRC	66	FEMALE	WHITE	Stage III	Kidney Cl	G3	2004	-24140	Alive	TUMOR F	3944			Complete Remission/Response	0	3944	0	3944	0	3944	0	3944
TCGA-B0	TCGA-B0	KIRC	47	FEMALE	WHITE	Stage I	Kidney Cl	G2	2003	-17177	Alive	TUMOR F	4074			Complete Remission/Response	0	4074	0	4074	0	4074	0	4074
TCGA-B0	TCGA-B0	KIRC	71	MALE	WHITE	Stage III	Kidney Cl	G3	2008	-26060	Dead	WITH TUMOR		480	288		1	480	1	480			1	288
TCGA-B0	TCGA-B0	KIRC	71	MALE	WHITE	Stage III	Kidney Cl	G3	2008	-26060	Dead	WITH TUMOR		480	288		1	480	1	480			1	288
TCGA-B0	TCGA-B0	KIRC	61	FEMALE	WHITE	Stage I	Kidney Cl	G2	2007	-22320	Alive	TUMOR F	2150			Complete Remission/Response	0	2150	0	2150	0	2150	0	2150
TCGA-B0	TCGA-B0	KIRC	69	MALE	WHITE	Stage III	Kidney Cl	G4	2007	-25552	Alive	WITH TUM	2609		1717	Complete Remission/Response	0	2609	0	2609	1	1717	1	1717
TCGA-B0	TCGA-B0	KIRC	69	MALE	WHITE	Stage III	Kidney Cl	G4	2007	-25552	Alive	WITH TUM	2609		1717	Complete Remission/Response	0	2609	0	2609	1	1717	1	1717
TCGA-B0	TCGA-B0	KIRC	50	MALE	WHITE	Stage I	Kidney Cl	G2	2007	-18486	Alive	TUMOR F	2630			Complete Remission/Response	0	2630	0	2630	0	2630	0	2630
TCGA-B0	TCGA-B0	KIRC	50	MALE	WHITE	Stage I	Kidney Cl	G2	2007	-18486	Alive	TUMOR F	2630			Complete Remission/Response	0	2630	0	2630	0	2630	0	2630
TCGA-B0	TCGA-B0	KIRC	77	MALE	BLACK OI	Stage I	Kidney Cl	G3	2005	-28438	Alive	TUMOR F	3631			Complete Remission/Response	0	3631	0	3631	0	3631	0	3631
TCGA-B0	TCGA-B0	KIRC	53	MALE	WHITE	Stage I	Kidney Cl	G2	2003	-19647	Alive	TUMOR F	3841		94	Complete Remission/Response	0	3841	0	3841	1	94	1	94
TCGA-B0	TCGA-B0	KIRC	53	MALE	WHITE	Stage I	Kidney Cl	G2	2003	-19647	Alive	TUMOR F	3841		94	Complete Remission/Response	0	3841	0	3841	1	94	1	94
TCGA-B0	TCGA-B0	KIRC	77	MALE	WHITE	Stage I	Kidney Cl	G2	2009	-28184	Alive	TUMOR F	1790			Complete Remission/Response	0	1790	0	1790	0	1790	0	1790
TCGA-B0	TCGA-B0	KIRC	65	MALE	WHITE	Stage III	Kidney Cl	G4	2007	-23915	Alive	TUMOR F	2461		224	Complete Remission/Response	0	2461	0	2461	1	224	1	224
TCGA-B0	TCGA-B0	KIRC	65	MALE	WHITE	Stage III	Kidney Cl	G4	2007	-23915	Alive	TUMOR F	2461		224	Complete Remission/Response	0	2461	0	2461	1	224	1	224
TCGA-B0	TCGA-B0	KIRC	71	MALE	WHITE	Stage I	Kidney Cl	G2	2007	-26186	Alive	TUMOR F	2172			Complete Remission/Response	0	2172	0	2172	0	2172	0	2172
TCGA-B0	TCGA-B0	KIRC	73	MALE	WHITE	Stage I	Kidney Cl	G3	2009	-26897	Alive	TUMOR F	2246			Complete Remission/Response	0	2246	0	2246	0	2246	0	2246
TCGA-B0	TCGA-B0	KIRC	73	MALE	WHITE	Stage I	Kidney Cl	G3	2009	-26897	Alive	TUMOR F	2246			Complete Remission/Response	0	2246	0	2246	0	2246	0	2246
TCGA-B0	TCGA-B0	KIRC	65	FEMALE	WHITE	Stage I	Kidney Cl	G2	2002	-23746	Alive	TUMOR F	4537		842	Complete Remission/Response	0	4537	0	4537	1	842	1	842
TCGA-B0	TCGA-B0	KIRC	65	FEMALE	WHITE	Stage I	Kidney Cl	G2	2002	-23746	Alive	TUMOR F	4537		842	Complete Remission/Response	0	4537	0	4537	1	842	1	842
TCGA-B0	TCGA-B0	KIRC	45	MALE	WHITE	Stage II	Kidney Cl	G2	2004	-16442	Alive	TUMOR F	3205			Complete Remission/Response	0	3205	0	3205	0	3205	0	3205
TCGA-B0	TCGA-B0	KIRC	45	MALE	WHITE	Stage II	Kidney Cl	G2	2004	-16442	Alive	TUMOR F	3205			Complete Remission/Response	0	3205	0	3205	0	3205	0	3205
TCGA-B0	TCGA-B0	KIRC	39	FEMALE	WHITE	Stage I	Kidney Cl	G3	2004	-14598	Alive	TUMOR F	3744			Complete Remission/Response	0	3744	0	3744	0	3744	0	3744
TCGA-B0	TCGA-B0	KIRC	62	FEMALE	WHITE	Stage III	Kidney Cl	G3	2003	-22843	Alive	TUMOR F	3974			Complete Remission/Response	0	3974	0	3974	0	3974	0	3974
TCGA-B0	TCGA-B0	KIRC	62	FEMALE	WHITE	Stage III	Kidney Cl	G3	2003	-22843	Alive	TUMOR F	3974			Complete Remission/Response	0	3974	0	3974	0	3974	0	3974
TCGA-B0	TCGA-B0	KIRC	57	MALE	WHITE	Stage I	Kidney Cl	G2	2008	-21168	Alive	WITH TUM	2430		1441	Progressive Disease	0	2430	0	2430			1	1441
TCGA-B0	TCGA-B0	KIRC	57	MALE	WHITE	Stage I	Kidney Cl	G2	2008	-21168	Alive	WITH TUM	2430		1441	Progressive Disease	0	2430	0	2430			1	1441
TCGA-B0	TCGA-B0	KIRC	50	MALE	WHITE	Stage III	Kidney Cl	G3	2004	-18444	Alive	WITH TUM	3989		3766	Complete Remission/Response	0	3989	0	3989	1	3766	1	3766
TCGA-B0	TCGA-B0	KIRC	50	MALE	WHITE	Stage III	Kidney Cl	G3	2004	-18444	Alive	WITH TUM	3989		3766	Complete Remission/Response	0	3989	0	3989	1	3766	1	3766
TCGA-B0	TCGA-B0	KIRC	68	FEMALE	BLACK OI	Stage IV	Kidney Cl	G3	2004	-24988	Alive	WITH TUM	2722		725		0	2722	0	2722			1	725
TCGA-B0	TCGA-B0	KIRC	68	FEMALE	BLACK OI	Stage IV	Kidney Cl	G3	2004	-24988	Alive	WITH TUM	2722		725		0	2722	0	2722			1	725
TCGA-B0	TCGA-B0	KIRC	75	FEMALE	WHITE	Stage III	Kidney Cl	G3	2006	-27644	Alive	TUMOR F	2782			Complete Remission/Response	0	2782	0	2782	0	2782	0	2782
TCGA-B0	TCGA-B0	KIRC	75	FEMALE	WHITE	Stage III	Kidney Cl	G3	2006	-27644	Alive	TUMOR F	2782			Complete Remission/Response	0	2782	0	2782	0	2782	0	2782
TCGA-B0	TCGA-B0	KIRC	53	MALE	WHITE	Stage I	Kidney Cl	G3	2003	-19600	Alive	TUMOR F	3834			Complete Remission/Response	0	3834	0	3834	0	3834	0	3834
TCGA-B2	TCGA-B2	KIRC	59	MALE	WHITE	Stage II	Kidney Cl	G2	2010	-21793	Alive	TUMOR F	992			Complete Remission/Response	0	992	0	992	0	992	0	992
TCGA-B2	TCGA-B2	KIRC	59	MALE	WHITE	Stage II	Kidney Cl	G2	2010	-21793	Alive	TUMOR F	992			Complete Remission/Response	0	992	0	992	0	992	0	992
TCGA-B2	TCGA-B2	KIRC	73	MALE	WHITE	Stage I	Kidney Cl	G2	2010	-26846	Alive	TUMOR F	1092			Complete Remission/Response	0	1092	0	1092	0	1092	0	1092
TCGA-B2	TCGA-B2	KIRC	73	MALE	WHITE	Stage I	Kidney Cl	G2	2010	-26846	Alive	TUMOR F	1092			Complete Remission/Response	0	1092	0	1092	0	1092	0	1092
TCGA-B2	TCGA-B2	KIRC	72	FEMALE	BLACK OI	Stage I	Kidney Cl	G2	2010	-26606	Dead	TUMOR FREE		51			1	51	0	51			0	51
TCGA-B2	TCGA-B2	KIRC	72	FEMALE	BLACK OI	Stage I	Kidney Cl	G2	2010	-26606	Dead	TUMOR FREE		51			1	51	0	51			0	51
TCGA-B2	TCGA-B2	KIRC	83	MALE	WHITE	Stage I	Kidney Cl	G3	2010	-30355	Alive	TUMOR F	972			Complete Remission/Response	0	972	0	972	0	972	0	972
TCGA-B2	TCGA-B2	KIRC	83	MALE	WHITE	Stage I	Kidney Cl	G3	2010	-30355	Alive	TUMOR F	972			Complete Remission/Response	0	972	0	972	0	972	0	972
TCGA-B2	TCGA-B2	KIRC	52	MALE	WHITE	Stage II	Kidney Cl	G3	2010	-19059	Alive	TUMOR F	648			Complete Remission/Response	0	648	0	648	0	648	0	648
TCGA-B2	TCGA-B2	KIRC	61	MALE	WHITE	Stage I	Kidney Cl	G2	2010	-22492	Alive	TUMOR F	952			Complete Remission/Response	0	952	0	952	0	952	0	952
TCGA-B2	TCGA-B2	KIRC	61	MALE	WHITE	Stage I	Kidney Cl	G2	2010	-22492	Alive	TUMOR F	952			Complete Remission/Response	0	952	0	952	0	952	0	952
TCGA-B2	TCGA-B2	KIRC	56	MALE	WHITE	Stage I	Kidney Cl	G2	2010	-20493	Alive	TUMOR F	963			Complete Remission/Response	0	963	0	963	0	963	0	963
TCGA-B2	TCGA-B2	KIRC	74	MALE	WHITE	Stage I	Kidney Cl	G2	2010	-27183	Alive	TUMOR F	755			Complete Remission/Response	0	755	0	755	0	755	0	755
TCGA-B2	TCGA-B2	KIRC	79	MALE	WHITE	Stage I	Kidney Cl	G2	2010	-28901	Alive	TUMOR F	919			Complete Remission/Response	0	919	0	919	0	919	0	919
TCGA-B2	TCGA-B2	KIRC	79	MALE	WHITE	Stage I	Kidney Cl	G2	2010	-28901	Alive	TUMOR F	919			Complete Remission/Response	0	919	0	919	0	919	0	919
TCGA-B2	TCGA-B2	KIRC	46	MALE	WHITE	Stage IV	Kidney Cl	G3	2010	-17045	Dead	WITH TUMOR		1003	163	Progressive Disease	1	1003	1	1003			1	163
TCGA-B2	TCGA-B2	KIRC	79	MALE	WHITE	Stage I	Kidney Cl	G3	2010	-28953	Alive	TUMOR F	656			Complete Remission/Response	0	656	0	656	0	656	0	656
TCGA-B2	TCGA-B2	KIRC	79	MALE	WHITE	Stage I	Kidney Cl	G3	2010	-28953	Alive	TUMOR F	656			Complete Remission/Response	0	656	0	656	0	656	0	656
TCGA-B2	TCGA-B2	KIRC	61	MALE	BLACK OI	Stage II	Kidney Clear Cell Rer		2012	-22637	Alive	TUMOR F	507		266	Complete Remission/Response	0	507	0	507	1	266	1	266
TCGA-B4	TCGA-B4	KIRC	68	FEMALE	WHITE	Stage IV	Kidney Cl	G3	2010	-25115	Alive	TUMOR F	365				0	365	0	365			0	365
TCGA-B4	TCGA-B4	KIRC	62	MALE	WHITE	Stage I	Kidney Cl	G2	2010	-22980	Alive	TUMOR F	175				0	175	0	175			0	175
TCGA-B4	TCGA-B4	KIRC	65	MALE	WHITE	Stage III	Kidney Cl	G2	2010	-23806	Alive	TUMOR F	155			Complete Remission/Response	0	155	0	155	0	155	0	155
TCGA-B4	TCGA-B4	KIRC	59	MALE	WHITE	Stage I	Kidney Cl	G1	2010	-21713	Alive	TUMOR F	38				0	38	0	38			0	38
TCGA-B4	TCGA-B4	KIRC	64	FEMALE	WHITE	Stage I	Kidney Cl	G2	2010	-23408	Alive	TUMOR F	16				0	16	0	16			0	16
TCGA-B4	TCGA-B4	KIRC	61	FEMALE	WHITE	Stage I	Kidney Cl	G2	2010	-22341	Alive	TUMOR F	141				0	141	0	141			0	141
TCGA-B4	TCGA-B4	KIRC	52	MALE	WHITE	[Discrepancy]	Kidney Cl	G2	2010	-19242	Alive	WITH TUM	166				0	166	0	166			0	166
TCGA-B4	TCGA-B4	KIRC	45	MALE	WHITE	Stage I	Kidney Cl	G2	2010	-16779	Alive	TUMOR F	11				0	11	0	11			0	11
TCGA-B4	TCGA-B4	KIRC	61	FEMALE	WHITE	Stage II	Kidney Cl	G1	2010	-22409	Alive	TUMOR F	7			[Unknown]	0	7	0	7			0	7
TCGA-B8	TCGA-B8	KIRC	66	FEMALE	WHITE	Stage IV	Kidney Cl	G3	2002	-24132	Dead	WITH TUMOR		709			1	709	1	709			1	709
TCGA-B8	TCGA-B8	KIRC	66	FEMALE	WHITE	Stage IV	Kidney Cl	G3	2002	-24132	Dead	WITH TUMOR		709			1	709	1	709			1	709
TCGA-B8	TCGA-B8	KIRC	41	FEMALE	WHITE	Stage I	Kidney Cl	G2	2009	-15063	Alive	TUMOR F	511			Complete Remission/Response	0	511	0	511	0	511	0	511
TCGA-B8	TCGA-B8	KIRC	63	FEMALE	WHITE	Stage I	Kidney Cl	G3	2009	-23062	Alive	TUMOR F	1520			Complete Remission/Response	0	1520	0	1520	0	1520	0	1520
TCGA-B8	TCGA-B8	KIRC	51	FEMALE	WHITE	Stage III	Kidney Cl	G2	2009	-18760	Alive	TUMOR F	1299			Complete Remission/Response	0	1299	0	1299	0	1299	0	1299
TCGA-B8	TCGA-B8	KIRC	74	MALE	WHITE	Stage III	Kidney Cl	G3	2010	-27031	Alive	WITH TUM	762		223		0	762	0	762			1	223
TCGA-B8	TCGA-B8	KIRC	73	FEMALE	WHITE	Stage I	Kidney Cl	G2	2010	-26882	Alive	TUMOR F	1380			Complete Remission/Response	0	1380	0	1380	0	1380	0	1380
TCGA-B8	TCGA-B8	KIRC	73	FEMALE	WHITE	Stage I	Kidney Cl	G2	2010	-26882	Alive	TUMOR F	1380			Complete Remission/Response	0	1380	0	1380	0	1380	0	1380
TCGA-B8	TCGA-B8	KIRC	58	MALE	WHITE	Stage I	Kidney Cl	G2	2010	-21206	Alive	TUMOR F	523			Complete Remission/Response	0	523	0	523	0	523	0	523
TCGA-B8	TCGA-B8	KIRC	58	MALE	WHITE	Stage I	Kidney Cl	G2	2010	-21206	Alive	TUMOR F	523			Complete Remission/Response	0	523	0	523	0	523	0	523
TCGA-B8	TCGA-B8	KIRC	70	FEMALE	WHITE	Stage III	Kidney Cl	G2	2010	-25786	Alive	WITH TUM	777		604	Complete Remission/Response	0	777	0	777	1	604	1	604
TCGA-B8	TCGA-B8	KIRC	70	FEMALE	WHITE	Stage III	Kidney Cl	G2	2010	-25786	Alive	WITH TUM	777		604	Complete Remission/Response	0	777	0	777	1	604	1	604
TCGA-B8	TCGA-B8	KIRC	63	MALE	BLACK OI	Stage I	Kidney Cl	G3	2010	-23176	Alive	TUMOR F	788			Complete Remission/Response	0	788	0	788	0	788	0	788
TCGA-B8	TCGA-B8	KIRC	57	MALE	WHITE	Stage IV	Kidney Cl	G3	2010	-21135	Alive	WITH TUM	1525		1227	Complete Remission/Response	0	1525	0	1525			1	1227
TCGA-B8	TCGA-B8	KIRC	57	MALE	WHITE	Stage IV	Kidney Cl	G3	2010	-21135	Alive	WITH TUM	1525		1227	Complete Remission/Response	0	1525	0	1525			1	1227
TCGA-B8	TCGA-B8	KIRC	56	MALE	WHITE	Stage III	Kidney Cl	G4	2010	-20764	Alive	TUMOR F	1218			Complete Remission/Response	0	1218	0	1218	0	1218	0	1218
TCGA-B8	TCGA-B8	KIRC	61	FEMALE	WHITE	Stage I	Kidney Cl	G3	2010	-22462	Alive	TUMOR F	722			Complete Remission/Response	0	722	0	722	0	722	0	722
TCGA-B8	TCGA-B8	KIRC	62	MALE	WHITE	Stage II	Kidney Cl	G2	2010	-23008	Alive	TUMOR F	36				0	36	0	36			0	36
TCGA-B8	TCGA-B8	KIRC	63	FEMALE	WHITE	Stage III	Kidney Cl	G3	2010	-23293	Alive	TUMOR F	822			Complete Remission/Response	0	822	0	822	0	822	0	822
TCGA-B8	TCGA-B8	KIRC	65	MALE	WHITE	Stage III	Kidney Cl	G3	2010	-23863	Alive	TUMOR F	26				0	26	0	26			0	26
TCGA-B8	TCGA-B8	KIRC	43	MALE	WHITE	Stage I	Kidney Cl	G2	2010	-15898	Alive	TUMOR F	737			Complete Remission/Response	0	737	0	737	0	737	0	737
TCGA-B8	TCGA-B8	KIRC	42	MALE	BLACK OI	Stage I	Kidney Cl	G2	2010	-15376	Alive	TUMOR F	1525			Complete Remission/Response	0	1525	0	1525	0	1525	0	1525
TCGA-B8	TCGA-B8	KIRC	38	FEMALE	BLACK OI	Stage I	Kidney Cl	G2	2010	-14113	Alive	TUMOR F	505				0	505	0	505			0	505
TCGA-B8	TCGA-B8	KIRC	53	MALE	WHITE	Stage I	Kidney Cl	G3	2010	-19561	Alive	TUMOR F	194			Complete Remission/Response	0	194	0	194	0	194	0	194
TCGA-B8	TCGA-B8	KIRC	53	MALE	WHITE	Stage I	Kidney Cl	G3	2010	-19561	Alive	TUMOR F	194			Complete Remission/Response	0	194	0	194	0	194	0	194
TCGA-B8	TCGA-B8	KIRC	71	MALE	WHITE	Stage III	Kidney Cl	G3	2010	-25937	Alive	WITH TUM	1476		694	Complete Remission/Response	0	1476	0	1476	1	694	1	694
TCGA-B8	TCGA-B8	KIRC	65	FEMALE	BLACK OI	Stage I	Kidney Cl	G3	2010	-23976	Alive	TUMOR F	16				0	16	0	16			0	16
TCGA-B8	TCGA-B8	KIRC	41	FEMALE	WHITE	Stage I	Kidney Cl	G2	2010	-15219	Alive	TUMOR F	1046			Complete Remission/Response	0	1046	0	1046	0	1046	0	1046
TCGA-B8	TCGA-B8	KIRC	41	FEMALE	WHITE	Stage I	Kidney Cl	G2	2010	-15219	Alive	TUMOR F	1046			Complete Remission/Response	0	1046	0	1046	0	1046	0	1046
TCGA-B8	TCGA-B8	KIRC	67	FEMALE	WHITE	Stage I	Kidney Cl	G2	2010	-24729	Alive	TUMOR F	435				0	435	0	435			0	435
TCGA-B8	TCGA-B8	KIRC	69	MALE	BLACK OI	Stage III	Kidney Cl	G2	2011	-25429	Alive	TUMOR F	830			Complete Remission/Response	0	830	0	830	0	830	0	830
TCGA-B8	TCGA-B8	KIRC	62	FEMALE	BLACK OI	Stage I	Kidney Cl	G3	2011	-22900	Alive	TUMOR F	909			Complete Remission/Response	0	909	0	909	0	909	0	909
TCGA-B8	TCGA-B8	KIRC	49	FEMALE	BLACK OI	Stage I	Kidney Cl	G2	2012	-18028	Alive	TUMOR F	519			Complete Remission/Response	0	519	0	519	0	519	0	519
TCGA-B8	TCGA-B8	KIRC	50	MALE	BLACK OI	Stage I	Kidney Cl	G3	2012	-18612	Alive	TUMOR F	53			Complete Remission/Response	0	53	0	53	0	53	0	53
TCGA-B8	TCGA-B8	KIRC	69	FEMALE	BLACK OI	Stage II	Kidney Cl	G3	2012	-25243	Alive	TUMOR F	256			Complete Remission/Response	0	256	0	256	0	256	0	256
TCGA-B8	TCGA-B8	KIRC	48	MALE	BLACK OI	Stage I	Kidney Cl	G3	2012	-17722	Alive	TUMOR F	150			Complete Remission/Response	0	150	0	150	0	150	0	150
TCGA-B8	TCGA-B8	KIRC	60	MALE	BLACK OI	Stage II	Kidney Cl	G2	2012	-22071	Alive	TUMOR F	528			Complete Remission/Response	0	528	0	528	0	528	0	528
TCGA-B8	TCGA-B8	KIRC	61	MALE	BLACK OI	Stage I	Kidney Cl	G1	2012	-22627	Alive	TUMOR F	469			Complete Remission/Response	0	469	0	469	0	469	0	469
TCGA-B8	TCGA-B8	KIRC	54	FEMALE	BLACK OI	Stage I	Kidney Cl	G3	2013	-19803	Alive	TUMOR F	495			Complete Remission/Response	0	495	0	495	0	495	0	495
TCGA-B8	TCGA-B8	KIRC	60	FEMALE	BLACK OI	Stage I	Kidney Cl	G2	2013	-21994	Alive	TUMOR F	431			Complete Remission/Response	0	431	0	431	0	431	0	431
TCGA-BP	TCGA-BP	KIRC	69	MALE	WHITE	Stage I	Kidney Cl	G2	2001	-25210	Alive	TUMOR F	3377				0	3377	0	3377			0	3377
TCGA-BP	TCGA-BP	KIRC	69	MALE	WHITE	Stage I	Kidney Cl	G2	2001	-25210	Alive	TUMOR F	3377				0	3377	0	3377			0	3377
TCGA-BP	TCGA-BP	KIRC	70	MALE	WHITE	Stage I	Kidney Cl	G2	2002	-25677	Dead	WITH TUMOR		2601	2352		1	2601	1	2601			1	2352
TCGA-BP	TCGA-BP	KIRC	70	MALE	WHITE	Stage I	Kidney Cl	G2	2002	-25677	Dead	WITH TUMOR		2601	2352		1	2601	1	2601			1	2352
TCGA-BP	TCGA-BP	KIRC	67	MALE	WHITE	Stage III	Kidney Cl	G2	2002	-24720	Alive	TUMOR F	2881				0	2881	0	2881			0	2881
TCGA-BP	TCGA-BP	KIRC	67	MALE	WHITE	Stage III	Kidney Cl	G2	2002	-24720	Alive	TUMOR F	2881				0	2881	0	2881			0	2881
TCGA-BP	TCGA-BP	KIRC	74	MALE	WHITE	Stage I	Kidney Cl	G3	2002	-27119	Alive	WITH TUM	2746		2685		0	2746	0	2746			1	2685
TCGA-BP	TCGA-BP	KIRC	74	MALE	WHITE	Stage I	Kidney Cl	G3	2002	-27119	Alive	WITH TUM	2746		2685		0	2746	0	2746			1	2685
TCGA-BP	TCGA-BP	KIRC	65	FEMALE	WHITE	Stage I	Kidney Cl	G2	2002	-23966	Alive	TUMOR F	3074				0	3074	0	3074			0	3074
TCGA-BP	TCGA-BP	KIRC	65	FEMALE	WHITE	Stage I	Kidney Cl	G2	2002	-23966	Alive	TUMOR F	3074				0	3074	0	3074			0	3074
TCGA-BP	TCGA-BP	KIRC	60	FEMALE	WHITE	Stage III	Kidney Cl	G3	2002	-21980	Alive	TUMOR F	2839				0	2839	0	2839			0	2839
TCGA-BP	TCGA-BP	KIRC	60	FEMALE	WHITE	Stage III	Kidney Cl	G3	2002	-21980	Alive	TUMOR F	2839				0	2839	0	2839			0	2839
TCGA-BP	TCGA-BP	KIRC	51	FEMALE	WHITE	Stage III	Kidney Cl	G2	2002	-18898	Dead	TUMOR FREE		992			1	992	0	992			0	992
TCGA-BP	TCGA-BP	KIRC	51	FEMALE	WHITE	Stage III	Kidney Cl	G2	2002	-18898	Dead	TUMOR FREE		992			1	992	0	992			0	992
TCGA-BP	TCGA-BP	KIRC	64	FEMALE	WHITE	Stage I	Kidney Cl	G1	2002	-23685	Alive	WITH TUM	3037		1049		0	3037	0	3037			1	1049
TCGA-BP	TCGA-BP	KIRC	64	FEMALE	WHITE	Stage I	Kidney Cl	G1	2002	-23685	Alive	WITH TUM	3037		1049		0	3037	0	3037			1	1049
TCGA-BP	TCGA-BP	KIRC	69	MALE	WHITE	Stage III	Kidney Cl	G3	2003	-25300	Alive	TUMOR F	13				0	13	0	13			0	13
TCGA-BP	TCGA-BP	KIRC	69	MALE	WHITE	Stage III	Kidney Cl	G3	2003	-25300	Alive	TUMOR F	13				0	13	0	13			0	13
TCGA-BP	TCGA-BP	KIRC	59	MALE	WHITE	Stage III	Kidney Cl	G2	2003	-21901	Alive	TUMOR F	2718				0	2718	0	2718			0	2718
TCGA-BP	TCGA-BP	KIRC	59	MALE	WHITE	Stage III	Kidney Cl	G2	2003	-21901	Alive	TUMOR F	2718				0	2718	0	2718			0	2718
TCGA-BP	TCGA-BP	KIRC	76	FEMALE	WHITE	Stage II	Kidney Cl	G2	2003	-27778	Dead	WITH TUMOR		701	78		1	701	1	701			1	78
TCGA-BP	TCGA-BP	KIRC	76	FEMALE	WHITE	Stage II	Kidney Cl	G2	2003	-27778	Dead	WITH TUMOR		701	78		1	701	1	701			1	78
TCGA-BP	TCGA-BP	KIRC	72	FEMALE	WHITE	Stage I	Kidney Cl	G2	2003	-26596	Dead	TUMOR FREE		2343			1	2343	0	2343			0	2343
TCGA-BP	TCGA-BP	KIRC	72	FEMALE	WHITE	Stage I	Kidney Cl	G2	2003	-26596	Dead	TUMOR FREE		2343			1	2343	0	2343			0	2343
TCGA-BP	TCGA-BP	KIRC	47	MALE	WHITE	Stage II	Kidney Cl	G3	2004	-17449	Alive	TUMOR F	1893				0	1893	0	1893			0	1893
TCGA-BP	TCGA-BP	KIRC	47	MALE	WHITE	Stage II	Kidney Cl	G3	2004	-17449	Alive	TUMOR F	1893				0	1893	0	1893			0	1893
TCGA-BP	TCGA-BP	KIRC	49	MALE	WHITE	Stage II	Kidney Cl	G3	2004	-18054	Alive	TUMOR F	1879				0	1879	0	1879			0	1879

TCGA-BP	TCGA-BP	KIRC	49	MALE	WHITE	Stage II	Kidney Cl	G3	2004	-18054	Alive	TUMOR F	1879				0	1879	0	1879			0	1879
TCGA-BP	TCGA-BP	KIRC	64	MALE	WHITE	Stage I	Kidney Cl	G2	2005	-23609	Alive	TUMOR F	1955				0	1955	0	1955			0	1955
TCGA-BP	TCGA-BP	KIRC	64	MALE	WHITE	Stage I	Kidney Cl	G2	2005	-23609	Alive	TUMOR F	1955				0	1955	0	1955			0	1955
TCGA-BP	TCGA-BP	KIRC	65	MALE	WHITE	Stage I	Kidney Cl	G2	2005	-23955	Alive	TUMOR F	1670				0	1670	0	1670			0	1670
TCGA-BP	TCGA-BP	KIRC	65	MALE	WHITE	Stage I	Kidney Cl	G2	2005	-23955	Alive	TUMOR F	1670				0	1670	0	1670			0	1670
TCGA-BP	TCGA-BP	KIRC	64	FEMALE	WHITE	Stage I	Kidney Cl	G2	2001	-23588	Alive	TUMOR F	2964				0	2964	0	2964			0	2964
TCGA-BP	TCGA-BP	KIRC	64	FEMALE	WHITE	Stage I	Kidney Cl	G2	2001	-23588	Alive	TUMOR F	2964				0	2964	0	2964			0	2964
TCGA-BP	TCGA-BP	KIRC	53	FEMALE	WHITE	Stage I	Kidney Cl	G2	2002	-19376	Dead	WITH TUMOR		1625	958		1	1625	1	1625			1	958
TCGA-BP	TCGA-BP	KIRC	53	FEMALE	WHITE	Stage I	Kidney Cl	G2	2002	-19376	Dead	WITH TUMOR		1625	958		1	1625	1	1625			1	958
TCGA-BP	TCGA-BP	KIRC	75	FEMALE	WHITE	Stage II	Kidney Cl	G2	2002	-27556	Dead	TUMOR FREE		109			1	109	0	109			0	109
TCGA-BP	TCGA-BP	KIRC	75	FEMALE	WHITE	Stage II	Kidney Cl	G2	2002	-27556	Dead	TUMOR FREE		109			1	109	0	109			0	109
TCGA-BP	TCGA-BP	KIRC	75	MALE	WHITE	Stage III	Kidney Cl	G2	2002	-27699	Dead	WITH TUMOR		845	134		1	845	1	845			1	134
TCGA-BP	TCGA-BP	KIRC	75	MALE	WHITE	Stage III	Kidney Cl	G2	2002	-27699	Dead	WITH TUMOR		845	134		1	845	1	845			1	134
TCGA-BP	TCGA-BP	KIRC	60	FEMALE	BLACK OI	Stage III	Kidney Cl	G2	2002	-22239	Alive	TUMOR F	1888				0	1888	0	1888			0	1888
TCGA-BP	TCGA-BP	KIRC	60	FEMALE	BLACK OI	Stage III	Kidney Cl	G2	2002	-22239	Alive	TUMOR F	1888				0	1888	0	1888			0	1888
TCGA-BP	TCGA-BP	KIRC	52	MALE	WHITE	Stage I	Kidney Cl	G2	2002	-19022	Dead	TUMOR FREE		2454			1	2454	0	2454			0	2454
TCGA-BP	TCGA-BP	KIRC	52	MALE	WHITE	Stage I	Kidney Cl	G2	2002	-19022	Dead	TUMOR FREE		2454			1	2454	0	2454			0	2454
TCGA-BP	TCGA-BP	KIRC	36	MALE	WHITE	Stage III	Kidney Cl	G2	2002	-13404	Alive	TUMOR F	1133				0	1133	0	1133			0	1133
TCGA-BP	TCGA-BP	KIRC	36	MALE	WHITE	Stage III	Kidney Cl	G2	2002	-13404	Alive	TUMOR F	1133				0	1133	0	1133			0	1133
TCGA-BP	TCGA-BP	KIRC	56	MALE	WHITE	Stage III	Kidney Cl	G3	2003	-20609	Dead	WITH TUMOR		645	442		1	645	1	645			1	442
TCGA-BP	TCGA-BP	KIRC	56	MALE	WHITE	Stage III	Kidney Cl	G3	2003	-20609	Dead	WITH TUMOR		645	442		1	645	1	645			1	442
TCGA-BP	TCGA-BP	KIRC	65	FEMALE	WHITE	Stage IV	Kidney Cl	G3	2003	-23860	Dead	WITH TUMOR		475	160		1	475	1	475			1	160
TCGA-BP	TCGA-BP	KIRC	65	FEMALE	WHITE	Stage IV	Kidney Cl	G3	2003	-23860	Dead	WITH TUMOR		475	160		1	475	1	475			1	160
TCGA-BP	TCGA-BP	KIRC	76	FEMALE	WHITE	Stage III	Kidney Cl	G4	2003	-27906	Dead	TUMOR FREE		2			1	2	0	2			0	2
TCGA-BP	TCGA-BP	KIRC	76	FEMALE	WHITE	Stage III	Kidney Cl	G4	2003	-27906	Dead	TUMOR FREE		2			1	2	0	2			0	2
TCGA-BP	TCGA-BP	KIRC	43	MALE	WHITE	Stage I	Kidney Cl	G3	2003	-15891	Alive	WITH TUI	2859		1757		0	2859	0	2859			1	1757
TCGA-BP	TCGA-BP	KIRC	43	MALE	WHITE	Stage I	Kidney Cl	G3	2003	-15891	Alive	WITH TUI	2859		1757		0	2859	0	2859			1	1757
TCGA-BP	TCGA-BP	KIRC	70	FEMALE	WHITE	Stage I	Kidney Cl	G2	2003	-25752	Dead	TUMOR FREE		562			1	562	0	562			0	562
TCGA-BP	TCGA-BP	KIRC	70	FEMALE	WHITE	Stage I	Kidney Cl	G2	2003	-25752	Dead	TUMOR FREE		562			1	562	0	562			0	562
TCGA-BP	TCGA-BP	KIRC	67	MALE	WHITE	Stage III	Kidney Cl	G2	2003	-24727	Dead	WITH TUMOR		1589			1	1589	1	1589			1	1589
TCGA-BP	TCGA-BP	KIRC	67	MALE	WHITE	Stage III	Kidney Cl	G2	2003	-24727	Dead	WITH TUMOR		1589			1	1589	1	1589			1	1589
TCGA-BP	TCGA-BP	KIRC	79	MALE	WHITE	Stage II	Kidney Cl	G3	2003	-29032	Dead	WITH TUMOR		2256	240		1	2256	1	2256			1	240
TCGA-BP	TCGA-BP	KIRC	79	MALE	WHITE	Stage II	Kidney Cl	G3	2003	-29032	Dead	WITH TUMOR		2256	240		1	2256	1	2256			1	240
TCGA-BP	TCGA-BP	KIRC	64	MALE	WHITE	Stage III	Kidney Cl	G3	2003	-23498	Dead	WITH TUMOR		1912	330		1	1912	1	1912			1	330
TCGA-BP	TCGA-BP	KIRC	64	MALE	WHITE	Stage III	Kidney Cl	G3	2003	-23498	Dead	WITH TUMOR		1912	330		1	1912	1	1912			1	330
TCGA-BP	TCGA-BP	KIRC	75	FEMALE	WHITE	Stage I	Kidney Cl	G2	2006	-27561	Alive	TUMOR F	1666				0	1666	0	1666			0	1666
TCGA-BP	TCGA-BP	KIRC	75	FEMALE	WHITE	Stage I	Kidney Cl	G2	2006	-27561	Alive	TUMOR F	1666				0	1666	0	1666			0	1666
TCGA-BP	TCGA-BP	KIRC	62	MALE	WHITE	Stage III	Kidney Cl	G3	2006	-22879	Alive	TUMOR F	1516				0	1516	0	1516			0	1516
TCGA-BP	TCGA-BP	KIRC	62	MALE	WHITE	Stage III	Kidney Cl	G3	2006	-22879	Alive	TUMOR F	1516				0	1516	0	1516			0	1516
TCGA-BP	TCGA-BP	KIRC	57	MALE	WHITE	Stage III	Kidney Cl	G3	2006	-21057	Dead	TUMOR FREE		1493			1	1493	0	1493			0	1493
TCGA-BP	TCGA-BP	KIRC	57	MALE	WHITE	Stage III	Kidney Cl	G3	2006	-21057	Dead	TUMOR FREE		1493			1	1493	0	1493			0	1493
TCGA-BP	TCGA-BP	KIRC	74	MALE	WHITE	Stage III	Kidney Cl	G2	2006	-27146	Alive	TUMOR F	1367				0	1367	0	1367			0	1367
TCGA-BP	TCGA-BP	KIRC	74	MALE	WHITE	Stage III	Kidney Cl	G2	2006	-27146	Alive	TUMOR F	1367				0	1367	0	1367			0	1367
TCGA-BP	TCGA-BP	KIRC	68	FEMALE	WHITE	Stage I	Kidney Cl	G2	2006	-24853	Alive		372				0	372	0	372			0	372
TCGA-BP	TCGA-BP	KIRC	68	FEMALE	WHITE	Stage I	Kidney Cl	G2	2006	-24853	Alive		372				0	372	0	372			0	372
TCGA-BP	TCGA-BP	KIRC	51	FEMALE	WHITE	Stage III	Kidney Cl	G2	2006	-18662	Alive	WITH TUI	970		873		0	970	0	970			1	873
TCGA-BP	TCGA-BP	KIRC	51	FEMALE	WHITE	Stage III	Kidney Cl	G2	2006	-18662	Alive	WITH TUI	970		873		0	970	0	970			1	873
TCGA-BP	TCGA-BP	KIRC	74	FEMALE	WHITE	Stage IV	Kidney Cl	G4	2007	-27253	Dead	WITH TUMOR		344	13		1	344	1	344			1	13
TCGA-BP	TCGA-BP	KIRC	74	FEMALE	WHITE	Stage IV	Kidney Cl	G4	2007	-27253	Dead	WITH TUMOR		344	13		1	344	1	344			1	13
TCGA-BP	TCGA-BP	KIRC	61	MALE	WHITE	Stage I	Kidney Cl	G2	2007	-22415	Dead	TUMOR FREE		375			1	375	0	375			0	375
TCGA-BP	TCGA-BP	KIRC	61	MALE	WHITE	Stage I	Kidney Cl	G2	2007	-22415	Dead	TUMOR FREE		375			1	375	0	375			0	375
TCGA-BP	TCGA-BP	KIRC	40	MALE	WHITE	Stage IV	Kidney Cl	G4	2006	-14973	Dead	WITH TUMOR		1034	685		1	1034	1	1034			1	685
TCGA-BP	TCGA-BP	KIRC	40	MALE	WHITE	Stage IV	Kidney Cl	G4	2006	-14973	Dead	WITH TUMOR		1034	685		1	1034	1	1034			1	685
TCGA-BP	TCGA-BP	KIRC	59	FEMALE	WHITE	Stage III	Kidney Cl	G4	2008	-21642	Dead			953			1	953		953			0	953
TCGA-BP	TCGA-BP	KIRC	59	FEMALE	WHITE	Stage III	Kidney Cl	G4	2008	-21642	Dead			953			1	953		953			0	953
TCGA-BP	TCGA-BP	KIRC	62	FEMALE	ASIAN	Stage I	Kidney Cl	G2	2003	-23001	Alive	TUMOR F	374				0	374	0	374			0	374
TCGA-BP	TCGA-BP	KIRC	62	FEMALE	ASIAN	Stage I	Kidney Cl	G2	2003	-23001	Alive	TUMOR F	374				0	374	0	374			0	374
TCGA-BP	TCGA-BP	KIRC	40	MALE	WHITE	Stage I	Kidney Cl	G2	2004	-14824	Alive	TUMOR F	2208				0	2208	0	2208			0	2208
TCGA-BP	TCGA-BP	KIRC	40	MALE	WHITE	Stage I	Kidney Cl	G2	2004	-14824	Alive	TUMOR F	2208				0	2208	0	2208			0	2208
TCGA-BP	TCGA-BP	KIRC	50	MALE	WHITE	Stage I	Kidney Cl	G2	2004	-18569	Alive	TUMOR F	2372				0	2372	0	2372			0	2372
TCGA-BP	TCGA-BP	KIRC	50	MALE	WHITE	Stage I	Kidney Cl	G2	2004	-18569	Alive	TUMOR F	2372				0	2372	0	2372			0	2372
TCGA-BP	TCGA-BP	KIRC	69	MALE	BLACK OI	Stage I	Kidney Cl	G2	2004	-25268	Alive	WITH TUI	2361		1457		0	2361	0	2361			1	1457
TCGA-BP	TCGA-BP	KIRC	69	MALE	BLACK OI	Stage I	Kidney Cl	G2	2004	-25268	Alive	WITH TUI	2361		1457		0	2361	0	2361			1	1457
TCGA-BP	TCGA-BP	KIRC	57	MALE	WHITE	Stage III	Kidney Cl	G4	2004	-21019	Alive	TUMOR F	182				0	182	0	182			0	182
TCGA-BP	TCGA-BP	KIRC	57	MALE	WHITE	Stage III	Kidney Cl	G4	2004	-21019	Alive	TUMOR F	182				0	182	0	182			0	182
TCGA-BP	TCGA-BP	KIRC	42	MALE	WHITE	Stage I	Kidney Cl	G3	2004	-15358	Dead	TUMOR FREE		1343			1	1343	0	1343			0	1343
TCGA-BP	TCGA-BP	KIRC	42	MALE	WHITE	Stage I	Kidney Cl	G3	2004	-15358	Dead	TUMOR FREE		1343			1	1343	0	1343			0	1343
TCGA-BP	TCGA-BP	KIRC	79	FEMALE	WHITE	Stage I	Kidney Cl	G2	2004	-29150	Dead	TUMOR FREE		1270			1	1270	0	1270			0	1270
TCGA-BP	TCGA-BP	KIRC	79	FEMALE	WHITE	Stage I	Kidney Cl	G2	2004	-29150	Dead	TUMOR FREE		1270			1	1270	0	1270			0	1270
TCGA-BP	TCGA-BP	KIRC	43	MALE	WHITE	Stage I	Kidney Cl	G2	2004	-15814	Alive	TUMOR F	2184				0	2184	0	2184			0	2184
TCGA-BP	TCGA-BP	KIRC	43	MALE	WHITE	Stage I	Kidney Cl	G2	2004	-15814	Alive	TUMOR F	2184				0	2184	0	2184			0	2184
TCGA-BP	TCGA-BP	KIRC	43	FEMALE	WHITE	Stage I	Kidney Cl	G3	2004	-15799	Alive	TUMOR F	1462				0	1462	0	1462			0	1462
TCGA-BP	TCGA-BP	KIRC	43	FEMALE	WHITE	Stage I	Kidney Cl	G3	2004	-15799	Alive	TUMOR F	1462				0	1462	0	1462			0	1462
TCGA-BP	TCGA-BP	KIRC	72	FEMALE	WHITE	Stage I	Kidney Cl	G2	2005	-26474	Alive	TUMOR F	400				0	400	0	400			0	400
TCGA-BP	TCGA-BP	KIRC	72	FEMALE	WHITE	Stage I	Kidney Cl	G2	2005	-26474	Alive	TUMOR F	400				0	400	0	400			0	400
TCGA-BP	TCGA-BP	KIRC	63	MALE	WHITE	Stage I	Kidney Cl	G2	2005	-23190	Alive	TUMOR F	1876				0	1876	0	1876			0	1876
TCGA-BP	TCGA-BP	KIRC	63	MALE	WHITE	Stage I	Kidney Cl	G2	2005	-23190	Alive	TUMOR F	1876				0	1876	0	1876			0	1876
TCGA-BP	TCGA-BP	KIRC	73	FEMALE	WHITE	Stage IV	Kidney Cl	G4	2005	-26824	Dead	WITH TUMOR		329	169		1	329	1	329			1	169
TCGA-BP	TCGA-BP	KIRC	73	FEMALE	WHITE	Stage IV	Kidney Cl	G4	2005	-26824	Dead	WITH TUMOR		329	169		1	329	1	329			1	169
TCGA-BP	TCGA-BP	KIRC	62	MALE	WHITE	Stage IV	Kidney Cl	G4	2005	-22771	Dead	WITH TUMOR		162	23		1	162	1	162			1	23
TCGA-BP	TCGA-BP	KIRC	62	MALE	WHITE	Stage IV	Kidney Cl	G4	2005	-22771	Dead	WITH TUMOR		162	23		1	162	1	162			1	23
TCGA-BP	TCGA-BP	KIRC	57	FEMALE	WHITE	Stage I	Kidney Cl	G2	2005	-20827	Alive	TUMOR F	1885				0	1885	0	1885			0	1885
TCGA-BP	TCGA-BP	KIRC	57	FEMALE	WHITE	Stage I	Kidney Cl	G2	2005	-20827	Alive	TUMOR F	1885				0	1885	0	1885			0	1885
TCGA-BP	TCGA-BP	KIRC	55	FEMALE	WHITE	Stage I	Kidney Cl	G2	2005	-20369	Alive	[Discrepar	1843				0	1843	0	1843			0	1843
TCGA-BP	TCGA-BP	KIRC	55	FEMALE	WHITE	Stage I	Kidney Cl	G2	2005	-20369	Alive	[Discrepar	1843				0	1843	0	1843			0	1843
TCGA-BP	TCGA-BP	KIRC	52	MALE	WHITE	Stage I	Kidney Cl	G2	2005	-19143	Alive	TUMOR F	411				0	411	0	411			0	411
TCGA-BP	TCGA-BP	KIRC	52	MALE	WHITE	Stage I	Kidney Cl	G2	2005	-19143	Alive	TUMOR F	411				0	411	0	411			0	411
TCGA-BP	TCGA-BP	KIRC	46	MALE	WHITE	Stage I	Kidney Cl	G3	2005	-17133	Alive	TUMOR F	1731				0	1731	0	1731			0	1731
TCGA-BP	TCGA-BP	KIRC	46	MALE	WHITE	Stage I	Kidney Cl	G3	2005	-17133	Alive	TUMOR F	1731				0	1731	0	1731			0	1731
TCGA-BP	TCGA-BP	KIRC	78	MALE	WHITE	Stage I	Kidney Cl	G3	2005	-28701	Alive	TUMOR F	2080				0	2080	0	2080			0	2080
TCGA-BP	TCGA-BP	KIRC	78	MALE	WHITE	Stage I	Kidney Cl	G3	2005	-28701	Alive	TUMOR F	2080				0	2080	0	2080			0	2080
TCGA-BP	TCGA-BP	KIRC	55	FEMALE	WHITE	Stage I	Kidney Cl	G2	2005	-20102	Alive	[Discrepar	354				0	354	0	354			0	354
TCGA-BP	TCGA-BP	KIRC	55	FEMALE	WHITE	Stage I	Kidney Cl	G2	2005	-20102	Alive	[Discrepar	354				0	354	0	354			0	354
TCGA-BP	TCGA-BP	KIRC	67	FEMALE	WHITE	Stage I	Kidney Cl	G2	2005	-24603	Alive	TUMOR F	1854				0	1854	0	1854			0	1854
TCGA-BP	TCGA-BP	KIRC	67	FEMALE	WHITE	Stage I	Kidney Cl	G2	2005	-24603	Alive	TUMOR F	1854				0	1854	0	1854			0	1854
TCGA-BP	TCGA-BP	KIRC	59	FEMALE	WHITE	Stage IV	Kidney Cl	G4	2007	-21697	Dead	WITH TUMOR		480	259		1	480	1	480			1	259
TCGA-BP	TCGA-BP	KIRC	59	FEMALE	WHITE	Stage IV	Kidney Cl	G4	2007	-21697	Dead	WITH TUMOR		480	259		1	480	1	480			1	259
TCGA-BP	TCGA-BP	KIRC	48	MALE	WHITE	Stage I	Kidney Cl	G2	2007	-17540	Alive	TUMOR F	1489				0	1489	0	1489			0	1489
TCGA-BP	TCGA-BP	KIRC	48	MALE	WHITE	Stage I	Kidney Cl	G2	2007	-17540	Alive	TUMOR F	1489				0	1489	0	1489			0	1489
TCGA-BP	TCGA-BP	KIRC	76	MALE	WHITE	Stage I	Kidney Cl	G2	2007	-28076	Dead	TUMOR FREE		1111			1	1111	0	1111			0	1111
TCGA-BP	TCGA-BP	KIRC	76	MALE	WHITE	Stage I	Kidney Cl	G2	2007	-28076	Dead	TUMOR FREE		1111			1	1111	0	1111			0	1111
TCGA-BP	TCGA-BP	KIRC	74	FEMALE	WHITE	Stage I	Kidney Cl	G2	2007	-27162	Alive	TUMOR F	620				0	620	0	620			0	620
TCGA-BP	TCGA-BP	KIRC	74	FEMALE	WHITE	Stage I	Kidney Cl	G2	2007	-27162	Alive	TUMOR F	620				0	620	0	620			0	620
TCGA-BP	TCGA-BP	KIRC	34	MALE	WHITE	Stage III	Kidney Cl	G3	2007	-12508	Alive	TUMOR F	1107				0	1107	0	1107			0	1107
TCGA-BP	TCGA-BP	KIRC	34	MALE	WHITE	Stage III	Kidney Cl	G3	2007	-12508	Alive	TUMOR F	1107				0	1107	0	1107			0	1107
TCGA-BP	TCGA-BP	KIRC	74	MALE	WHITE	[Discrepancy]	Kidney Cl	G4	2007	-27335	Dead	WITH TUMOR		334			1	334	1	334			1	334
TCGA-BP	TCGA-BP	KIRC	74	MALE	WHITE	[Discrepancy]	Kidney Cl	G4	2007	-27335	Dead	WITH TUMOR		334			1	334	1	334			1	334
TCGA-BP	TCGA-BP	KIRC	70	MALE	WHITE	Stage III	Kidney Cl	G3	2007	-25842	Dead	WITH TUMOR		1133	352		1	1133	1	1133			1	352
TCGA-BP	TCGA-BP	KIRC	70	MALE	WHITE	Stage III	Kidney Cl	G3	2007	-25842	Dead	WITH TUMOR		1133	352		1	1133	1	1133			1	352
TCGA-BP	TCGA-BP	KIRC	57	MALE	WHITE	Stage I	Kidney Cl	G2	2007	-20825	Alive	TUMOR F	1124				0	1124	0	1124			0	1124
TCGA-BP	TCGA-BP	KIRC	57	MALE	WHITE	Stage I	Kidney Cl	G2	2007	-20825	Alive	TUMOR F	1124				0	1124	0	1124			0	1124
TCGA-BP	TCGA-BP	KIRC	79	MALE	WHITE	Stage III	Kidney Cl	G3	2008	-28934	Alive	TUMOR F	204				0	204	0	204			0	204
TCGA-BP	TCGA-BP	KIRC	79	MALE	WHITE	Stage III	Kidney Cl	G3	2008	-28934	Alive	TUMOR F	204				0	204	0	204			0	204
TCGA-BP	TCGA-BP	KIRC	59	MALE	WHITE	Stage I	Kidney Cl	G2	2008	-21727	Alive	WITH TUI	1459		295		0	1459	0	1459			1	295
TCGA-BP	TCGA-BP	KIRC	59	MALE	WHITE	Stage I	Kidney Cl	G2	2008	-21727	Alive	WITH TUI	1459		295		0	1459	0	1459			1	295
TCGA-BP	TCGA-BP	KIRC	42	MALE	WHITE	Stage I	Kidney Cl	G3	2008	-15360	Alive	TUMOR F	211				0	211	0	211			0	211
TCGA-BP	TCGA-BP	KIRC	42	MALE	WHITE	Stage I	Kidney Cl	G3	2008	-15360	Alive	TUMOR F	211				0	211	0	211			0	211
TCGA-BP	TCGA-BP	KIRC	49	MALE	WHITE	Stage I	Kidney Cl	G3	2003	-17968	Alive	TUMOR F	2660				0	2660	0	2660			0	2660
TCGA-BP	TCGA-BP	KIRC	49	MALE	WHITE	Stage I	Kidney Cl	G3	2003	-17968	Alive	TUMOR F	2660				0	2660	0	2660			0	2660
TCGA-BP	TCGA-BP	KIRC	46	MALE	WHITE	Stage II	Kidney Cl	G3	2004	-17099	Alive	TUMOR F	2172				0	2172	0	2172			0	2172
TCGA-BP	TCGA-BP	KIRC	46	MALE	WHITE	Stage II	Kidney Cl	G3	2004	-17099	Alive	TUMOR F	2172				0	2172	0	2172			0	2172
TCGA-BP	TCGA-BP	KIRC	47	MALE	WHITE	Stage I	Kidney Cl	G2	2005	-17284	Alive	TUMOR F	1935				0	1935	0	1935			0	1935
TCGA-BP	TCGA-BP	KIRC	47	MALE	WHITE	Stage I	Kidney Cl	G2	2005	-17284	Alive	TUMOR F	1935				0	1935	0	1935			0	1935
TCGA-BP	TCGA-BP	KIRC	58	MALE	WHITE	Stage II	Kidney Cl	G2	2005	-21255	Alive	TUMOR F	1785				0	1785	0	1785			0	1785
TCGA-BP	TCGA-BP	KIRC	58	MALE	WHITE	Stage II	Kidney Cl	G2	2005	-21255	Alive	TUMOR F	1785				0	1785	0	1785			0	1785
TCGA-BP	TCGA-BP	KIRC	63	MALE	WHITE	Stage I	Kidney Cl	G3	2005	-23373	Alive	TUMOR F	1834				0	1834	0	1834			0	1834
TCGA-BP	TCGA-BP	KIRC	63	MALE	WHITE	Stage I	Kidney Cl	G3	2005	-23373	Alive	TUMOR F	1834				0	1834	0	1834			0	1834
TCGA-BP	TCGA-BP	KIRC	54	FEMALE	WHITE	Stage I	Kidney Cl	G2	2005	-20084	Alive	TUMOR F	1862				0	1862	0	1862			0	1862
TCGA-BP	TCGA-BP	KIRC	54	FEMALE	WHITE	Stage I	Kidney Cl	G2	2005	-20084	Alive	TUMOR F	1862				0	1862	0	1862			0	1862
TCGA-BP	TCGA-BP	KIRC	46	MALE	WHITE	Stage I	Kidney Cl	G2	2005	-16992	Alive	TUMOR F	1871				0	1871	0	1871			0	1871
TCGA-BP	TCGA-BP	KIRC	46	MALE	WHITE	Stage I	Kidney Cl	G2	2005	-16992	Alive	TUMOR F	1871				0	1871	0	1871			0	1871
TCGA-BP	TCGA-BP	KIRC	76	MALE	WHITE	Stage III	Kidney Cl	G2	2005	-27857	Alive	TUMOR F	205				0	205	0	205			0	205
TCGA-BP	TCGA-BP	KIRC	76	MALE	WHITE	Stage III	Kidney Cl	G2	2005	-27857	Alive	TUMOR F	205				0	205	0	205			0	205
TCGA-BP	TCGA-BP	KIRC	40	MALE	WHITE	Stage I	Kidney Cl	G3	2006	-14651	Alive	TUMOR F	1746				0	1746	0	1746			0	1746
TCGA-BP	TCGA-BP	KIRC	40	MALE	WHITE	Stage I	Kidney Cl	G3	2006	-14651	Alive	TUMOR F	1746				0	1746	0	1746			0	1746
TCGA-BP	TCGA-BP	KIRC	63	FEMALE	WHITE	Stage I	Kidney Cl	G2	2006	-23216	Alive	TUMOR F	1794				0	1794	0	1794			0	1794
TCGA-BP	TCGA-BP	KIRC	63	FEMALE	WHITE	Stage I	Kidney Cl	G2	2006	-23216	Alive	TUMOR F	1794				0	1794	0	1794			0	1794
TCGA-BP	TCGA-BP	KIRC	44	MALE	ASIAN	Stage III	Kidney Cl	G3	2006	-16228	Alive	TUMOR F	433				0	433	0	433			0	433
TCGA-BP	TCGA-BP	KIRC	44	MALE	ASIAN	Stage III	Kidney Cl	G3	2006	-16228	Alive	TUMOR F	433				0	433	0	433			0	433
TCGA-BP	TCGA-BP	KIRC	40	MALE	WHITE	Stage III	Kidney Cl	G3	2006	-14675	Alive	TUMOR F	1487				0	1487	0	1487			0	1487
TCGA-BP	TCGA-BP	KIRC	40	MALE	WHITE	Stage III	Kidney Cl	G3	2006	-14675	Alive	TUMOR F	1487				0	1487	0	1487			0	1487
TCGA-BP	TCGA-BP	KIRC	43	FEMALE	WHITE	Stage III	Kidney Cl	G3	2006	-16038	Alive	TUMOR F	1502				0	1502	0	1502			0	1502
TCGA-BP	TCGA-BP	KIRC	43	FEMALE	WHITE	Stage III	Kidney Cl	G3	2006	-16038	Alive	TUMOR F	1502				0	1502	0	1502			0	1502
TCGA-BP	TCGA-BP	KIRC	47	MALE	WHITE	Stage III	Kidney Cl	G3	2006	-17461	Alive	TUMOR F	1384				0	1384	0	1384			0	1384
TCGA-BP	TCGA-BP	KIRC	47	MALE	WHITE	Stage III	Kidney Cl	G3	2006	-17461	Alive	TUMOR F	1384				0	1384	0	1384			0	1384
TCGA-BP	TCGA-BP	KIRC	58	MALE	WHITE	Stage IV	Kidney Cl	G4	2006	-21310	Dead	WITH TUMOR		211	35		1	211	1	211			1	35
TCGA-BP	TCGA-BP	KIRC	58	MALE	WHITE	Stage IV	Kidney Cl	G4	2006	-21310	Dead	WITH TUMOR		211	35		1	211	1	211			1	35
TCGA-BP	TCGA-BP	KIRC	40	MALE	WHITE	Stage I	Kidney Cl	G3	2006	-14710	Alive	TUMOR F	1433				0	1433	0	1433			0	1433
TCGA-BP	TCGA-BP	KIRC	40	MALE	WHITE	Stage I	Kidney Cl	G3	2006	-14710	Alive	TUMOR F	1433				0	1433	0	1433			0	1433
TCGA-BP	TCGA-BP	KIRC	77	MALE	WHITE	Stage I	Kidney Cl	G3	2006	-28380	Alive	TUMOR F	1632				0	1632	0	1632			0	1632
TCGA-BP	TCGA-BP	KIRC	77	MALE	WHITE	Stage I	Kidney Cl	G3	2006	-28380	Alive	TUMOR F	1632				0	1632	0	1632			0	1632
TCGA-BP	TCGA-BP	KIRC	57	MALE	WHITE	Stage I	Kidney Cl	G3	2006	-20950	Alive	TUMOR F	454				0	454	0	454			0	454
TCGA-BP	TCGA-BP	KIRC	57	MALE	WHITE	Stage I	Kidney Cl	G3	2006	-20950	Alive	TUMOR F	454				0	454	0	454			0	454
TCGA-BP	TCGA-BP	KIRC	75	FEMALE	WHITE	Stage III	Kidney Cl	G3	2006	-27676	Dead	TUMOR FREE		1097			1	1097	0	1097			0	1097
TCGA-BP	TCGA-BP	KIRC	75	FEMALE	WHITE	Stage III	Kidney Cl	G3	2006	-27676	Dead	TUMOR FREE		1097			1	1097	0	1097			0	1097
TCGA-BP	TCGA-BP	KIRC	42	MALE	WHITE	Stage I	Kidney Cl	G3	2006	-15451	Alive	TUMOR F	1014				0	1014	0	1014			0	1014
TCGA-BP	TCGA-BP	KIRC	42	MALE	WHITE	Stage I	Kidney Cl	G3	2006	-15451	Alive	TUMOR F	1014				0	1014	0	1014			0	1014
TCGA-BP	TCGA-BP	KIRC	67	FEMALE	ASIAN	Stage III	Kidney Cl	G4	2006	-24558	Alive	TUMOR F	1413				0	1413	0	1413			0	1413
TCGA-BP	TCGA-BP	KIRC	67	FEMALE	ASIAN	Stage III	Kidney Cl	G4	2006	-24558	Alive	TUMOR F	1413				0	1413	0	1413			0	1413
TCGA-BP	TCGA-BP	KIRC	72	MALE	WHITE	Stage III	Kidney Cl	G4	2006	-26611	Dead	WITH TUMOR		952	205		1	952	1	952			1	205
TCGA-BP	TCGA-BP	KIRC	72	MALE	WHITE	Stage III	Kidney Cl	G4	2006	-26611	Dead	WITH TUMOR		952	205		1	952	1	952			1	205
TCGA-BP	TCGA-BP	KIRC	75	MALE	WHITE	Stage I	Kidney Cl	G3	2006	-27612	Alive	TUMOR F	785				0	785	0	785			0	785
TCGA-BP	TCGA-BP	KIRC	75	MALE	WHITE	Stage I	Kidney Cl	G3	2006	-27612	Alive	TUMOR F	785				0	785	0	785			0	785
TCGA-BP	TCGA-BP	KIRC	41	FEMALE	ASIAN	Stage I	Kidney Cl	G2	2006	-15041	Alive	TUMOR F	1124				0	1124	0	1124			0	1124
TCGA-BP	TCGA-BP	KIRC	41	FEMALE	ASIAN	Stage I	Kidney Cl	G2	2006	-15041	Alive	TUMOR F	1124				0	1124	0	1124			0	1124
TCGA-BP	TCGA-BP	KIRC	72	MALE	WHITE	Stage I	Kidney Cl	G2	2007	-26576	Dead	TUMOR FREE		828			1	828	0	828			0	828
TCGA-BP	TCGA-BP	KIRC	72	MALE	WHITE	Stage I	Kidney Cl	G2	2007	-26576	Dead	TUMOR FREE		828			1	828	0	828			0	828
TCGA-BP	TCGA-BP	KIRC	58	MALE	WHITE	Stage III	Kidney Cl	G3	2007	-21382	Alive	TUMOR F	118				0	118	0	118			0	118
TCGA-BP	TCGA-BP	KIRC	58	MALE	WHITE	Stage III	Kidney Cl	G3	2007	-21382	Alive	TUMOR F	118				0	118	0	118			0	118
TCGA-BP	TCGA-BP	KIRC	54	MALE	WHITE	Stage I	Kidney Cl	G2	2007	-19739	Alive	TUMOR F	1413				0	1413	0	1413			0	1413
TCGA-BP	TCGA-BP	KIRC	54	MALE	WHITE	Stage I	Kidney Cl	G2	2007	-19739	Alive	TUMOR F	1413				0	1413	0	1413			0	1413
TCGA-BP	TCGA-BP	KIRC	66	MALE	WHITE	Stage I	Kidney Cl	G4	2007	-24438	Alive	TUMOR F	501				0	501	0	501			0	501
TCGA-BP	TCGA-BP	KIRC	66	MALE	WHITE	Stage I	Kidney Cl	G4	2007	-24438	Alive	TUMOR F	501				0	501	0	501			0	501
TCGA-BP	TCGA-BP	KIRC	58	MALE	WHITE	Stage I	Kidney Cl	G3	2007	-21407	Alive	TUMOR F	177				0	177	0	177			0	177
TCGA-BP	TCGA-BP	KIRC	58	MALE	WHITE	Stage I	Kidney Cl	G3	2007	-21407	Alive	TUMOR F	177				0	177	0	177			0	177
TCGA-BP	TCGA-BP	KIRC	54	MALE	WHITE	Stage I	Kidney Cl	G3	2007	-19946	Alive	TUMOR F	1308				0	1308	0	1308			0	1308
TCGA-BP	TCGA-BP	KIRC	54	MALE	WHITE	Stage I	Kidney Cl	G3	2007	-19946	Alive	TUMOR F	1308				0	1308	0	1308			0	1308
TCGA-BP	TCGA-BP	KIRC	68	MALE	WHITE	Stage I	Kidney Cl	G3	2007	-24850	Alive	TUMOR F	1371				0	1371	0	1371			0	1371
TCGA-BP	TCGA-BP	KIRC	68	MALE	WHITE	Stage I	Kidney Cl	G3	2007	-24850	Alive	TUMOR F	1371				0	1371	0	1371			0	1371
TCGA-BP	TCGA-BP	KIRC	49	MALE	ASIAN	Stage I	Kidney Cl	G3	2007	-17997	Alive	TUMOR F	932				0	932	0	932			0	932
TCGA-BP	TCGA-BP	KIRC	49	MALE	ASIAN	Stage I	Kidney Cl	G3	2007	-17997	Alive	TUMOR F	932				0	932	0	932			0	932
TCGA-BP	TCGA-BP	KIRC	56	MALE	WHITE	Stage I	Kidney Cl	G2	2007	-20518	Alive	TUMOR F	1266				0	1266	0	1266			0	1266
TCGA-BP	TCGA-BP	KIRC	56	MALE	WHITE	Stage I	Kidney Cl	G2	2007	-20518	Alive	TUMOR F	1266				0	1266	0	1266			0	1266
TCGA-BP	TCGA-BP	KIRC	40	MALE	WHITE	Stage I	Kidney Cl	G3	2007	-14868	Alive	TUMOR F	563				0	563	0	563			0	563
TCGA-BP	TCGA-BP	KIRC	40	MALE	WHITE	Stage I	Kidney Cl	G3	2007	-14868	Alive	TUMOR F	563				0	563	0	563			0	563
TCGA-BP	TCGA-BP	KIRC	43	FEMALE	WHITE	Stage I	Kidney Cl	G2	2007	-15858	Alive	TUMOR F	1177				0	1177	0	1177			0	1177
TCGA-BP	TCGA-BP	KIRC	43	FEMALE	WHITE	Stage I	Kidney Cl	G2	2007	-15858	Alive	TUMOR F	1177				0	1177	0	1177			0	1177
TCGA-BP	TCGA-BP	KIRC	53	MALE	WHITE	Stage I	Kidney Cl	G3	2007	-19383	Alive	TUMOR F	1126				0	1126	0	1126			0	1126
TCGA-BP	TCGA-BP	KIRC	53	MALE	WHITE	Stage I	Kidney Cl	G3	2007	-19383	Alive	TUMOR F	1126				0	1126	0	1126			0	1126
TCGA-BP	TCGA-BP	KIRC	61	MALE	WHITE	Stage I	Kidney Cl	G2	2007	-22324	Alive	TUMOR F	840				0	840	0	840			0	840
TCGA-BP	TCGA-BP	KIRC	61	MALE	WHITE	Stage I	Kidney Cl	G2	2007	-22324	Alive	TUMOR F	840				0	840	0	840			0	840
TCGA-BP	TCGA-BP	KIRC	45	MALE	WHITE	Stage II	Kidney Cl	G2	2007	-16793	Alive	TUMOR F	1140				0	1140	0	1140			0	1140
TCGA-BP	TCGA-BP	KIRC	45	MALE	WHITE	Stage II	Kidney Cl	G2	2007	-16793	Alive	TUMOR F	1140				0	1140	0	1140			0	1140
TCGA-BP	TCGA-BP	KIRC	46	MALE	WHITE	Stage I	Kidney Cl	G2	2007	-17008	Alive	TUMOR F	1071				0	1071	0	1071			0	1071
TCGA-BP	TCGA-BP	KIRC	46	MALE	WHITE	Stage I	Kidney Cl	G2	2007	-17008	Alive	TUMOR F	1071				0	1071	0	1071			0	1071
TCGA-BP	TCGA-BP	KIRC	52	MALE	WHITE	Stage I	Kidney Cl	G3	2007	-19052	Dead	WITH TUMOR		1092	324		1	1092	1	1092			1	324
TCGA-BP	TCGA-BP	KIRC	52	MALE	WHITE	Stage I	Kidney Cl	G3	2007	-19052	Dead	WITH TUMOR		1092	324		1	1092	1	1092			1	324
TCGA-BP	TCGA-BP	KIRC	63	MALE	WHITE	Stage III	Kidney Cl	G4	2007	-23130	Dead	WITH TUMOR		878	582		1	878	1	878			1	582
TCGA-BP	TCGA-BP	KIRC	63	MALE	WHITE	Stage III	Kidney Cl	G4	2007	-23130	Dead	WITH TUMOR		878	582		1	878	1	878			1	582
TCGA-BP	TCGA-BP	KIRC	75	MALE	WHITE	Stage I	Kidney Cl	G2	2002	-27692	Dead	TUMOR FREE		1463			1	1463	0	1463			0	1463
TCGA-BP	TCGA-BP	KIRC	75	MALE	WHITE	Stage I	Kidney Cl	G2	2002	-27692	Dead	TUMOR FREE		1463			1	1463	0	1463			0	1463
TCGA-BP	TCGA-BP	KIRC	70	MALE	WHITE	Stage I	Kidney Cl	G4	2004	-25615	Alive	TUMOR F	193				0	193	0	193			0	193
TCGA-BP	TCGA-BP	KIRC	70	MALE	WHITE	Stage I	Kidney Cl	G4	2004	-25615	Alive	TUMOR F	193				0	193	0	193			0	193
TCGA-BP	TCGA-BP	KIRC	55	MALE	WHITE	Stage I	Kidney Cl	G2	2004	-20229	Alive	TUMOR F	2412				0	2412	0	2412			0	2412
TCGA-BP	TCGA-BP	KIRC	55	MALE	WHITE	Stage I	Kidney Cl	G2	2004	-20229	Alive	TUMOR F	2412				0	2412	0	2412			0	2412
TCGA-BP	TCGA-BP	KIRC	75	MALE	WHITE	Stage I	Kidney Cl	G2	2004	-27436	Dead	TUMOR FREE		62			1	62	0	62			0	62
TCGA-BP	TCGA-BP	KIRC	75	MALE	WHITE	Stage I	Kidney Cl	G2	2004	-27436	Dead	TUMOR FREE		62			1	62	0	62			0	62
TCGA-BP	TCGA-BP	KIRC	45	FEMALE	WHITE	Stage I	Kidney Cl	G2	2004	-16693	Alive	TUMOR F	2257				0	2257	0	2257			0	2257
TCGA-BP	TCGA-BP	KIRC	45	FEMALE	WHITE	Stage I	Kidney Cl	G2	2004	-16693	Alive	TUMOR F	2257				0	2257	0	2257			0	2257
TCGA-BP	TCGA-BP	KIRC	60	MALE	WHITE	Stage I	Kidney Cl	G3	2004	-21949	Alive	TUMOR F	932				0	932	0	932			0	932
TCGA-BP	TCGA-BP	KIRC	60	MALE	WHITE	Stage I	Kidney Cl	G3	2004	-21949	Alive	TUMOR F	932				0	932	0	932			0	932
TCGA-BP	TCGA-BP	KIRC	78	FEMALE	WHITE	Stage I	Kidney Cl	G2	2004	-28727	Dead	TUMOR FREE		1590			1	1590	0	1590			0	1590
TCGA-BP	TCGA-BP	KIRC	78	FEMALE	WHITE	Stage I	Kidney Cl	G2	2004	-28727	Dead	TUMOR FREE		1590			1	1590	0	1590			0	1590
TCGA-BP	TCGA-BP	KIRC	46	FEMALE	WHITE	Stage I	Kidney Cl	G3	2004	-17147	Alive	TUMOR F	293				0	293	0	293			0	293
TCGA-BP	TCGA-BP	KIRC	46	FEMALE	WHITE	Stage I	Kidney Cl	G3	2004	-17147	Alive	TUMOR F	293				0	293	0	293			0	293
TCGA-BP	TCGA-BP	KIRC	71	MALE	WHITE	Stage IV	Kidney Cl	G4	2004	-26070	Dead	WITH TUMOR		1912	396		1	1912	1	1912			1	396
TCGA-BP	TCGA-BP	KIRC	71	MALE	WHITE	Stage IV	Kidney Cl	G4	2004	-26070	Dead	WITH TUMOR		1912	396		1	1912	1	1912			1	396
TCGA-BP	TCGA-BP	KIRC	53	MALE	WHITE	Stage I	Kidney Cl	G2	2004	-19413	Alive	TUMOR F	2263				0	2263	0	2263			0	2263
TCGA-BP	TCGA-BP	KIRC	53	MALE	WHITE	Stage I	Kidney Cl	G2	2004	-19413	Alive	TUMOR F	2263				0	2263	0	2263			0	2263
TCGA-BP	TCGA-BP	KIRC	58	FEMALE	WHITE	Stage I	Kidney Cl	G2	2006	-21514	Alive	TUMOR F	1495				0	1495	0	1495			0	1495
TCGA-BP	TCGA-BP	KIRC	58	FEMALE	WHITE	Stage I	Kidney Cl	G2	2006	-21514	Alive	TUMOR F	1495				0	1495	0	1495			0	1495
TCGA-BP	TCGA-BP	KIRC	56	MALE	WHITE	Stage I	Kidney Cl	G3	2007	-20597	Alive	TUMOR F	1165				0	1165	0	1165			0	1165
TCGA-BP	TCGA-BP	KIRC	56	MALE	WHITE	Stage I	Kidney Cl	G3	2007	-20597	Alive	TUMOR F	1165				0	1165	0	1165			0	1165
TCGA-BP	TCGA-BP	KIRC	57	MALE	WHITE	Stage III	Kidney Cl	G3	2007	-20881	Alive	TUMOR F	1291		485		0	1291	0	1291			1	485
TCGA-BP	TCGA-BP	KIRC	57	MALE	WHITE	Stage III	Kidney Cl	G3	2007	-20881	Alive	TUMOR F	1291		485		0	1291	0	1291			1	485
TCGA-BP	TCGA-BP	KIRC	54	MALE	WHITE	Stage I	Kidney Cl	G3	2007	-19746	Alive	TUMOR F	1133				0	1133	0	1133			0	1133
TCGA-BP	TCGA-BP	KIRC	54	MALE	WHITE	Stage I	Kidney Cl	G3	2007	-19746	Alive	TUMOR F	1133				0	1133	0	1133			0	1133
TCGA-BP	TCGA-BP	KIRC	56	MALE	WHITE	Stage I	Kidney Cl	G3	2007	-20470	Alive	WITH TUI	1132		736		0	1132	0	1132			1	736
TCGA-BP	TCGA-BP	KIRC	56	MALE	WHITE	Stage I	Kidney Cl	G3	2007	-20470	Alive	WITH TUI	1132		736		0	1132	0	1132			1	736
TCGA-BP	TCGA-BP	KIRC	50	FEMALE	WHITE	Stage I	Kidney Cl	G2	2007	-18348	Alive	TUMOR F	693				0	693	0	693			0	693
TCGA-BP	TCGA-BP	KIRC	50	FEMALE	WHITE	Stage I	Kidney Cl	G2	2007	-18348	Alive	TUMOR F	693				0	693	0	693			0	693
TCGA-BP	TCGA-BP	KIRC	54	MALE	WHITE	Stage I	Kidney Cl	G2	2007	-19999	Alive	TUMOR F	406				0	406	0	406			0	406
TCGA-BP	TCGA-BP	KIRC	54	MALE	WHITE	Stage I	Kidney Cl	G2	2007	-19999	Alive	TUMOR F	406				0	406	0	406			0	406
TCGA-BP	TCGA-BP	KIRC	60	MALE	WHITE	Stage I	Kidney Cl	G4	2007	-22156	Dead	WITH TUMOR		822	634		1	822	1	822			1	634
TCGA-BP	TCGA-BP	KIRC	60	MALE	WHITE	Stage I	Kidney Cl	G4	2007	-22156	Dead	WITH TUMOR		822	634		1	822	1	822			1	634
TCGA-BP	TCGA-BP	KIRC	61	MALE	WHITE	Stage I	Kidney Cl	G3	2007	-22285	Alive	TUMOR F	1011				0	1011	0	1011			0	1011
TCGA-BP	TCGA-BP	KIRC	61	MALE	WHITE	Stage I	Kidney Cl	G3	2007	-22285	Alive	TUMOR F	1011				0	1011	0	1011			0	1011
TCGA-BP	TCGA-BP	KIRC	79	MALE	WHITE	Stage III	Kidney Cl	G2	2007	-29207	Alive	TUMOR F	967				0	967	0	967			0	967
TCGA-BP	TCGA-BP	KIRC	79	MALE	WHITE	Stage III	Kidney Cl	G2	2007	-29207	Alive	TUMOR F	967				0	967	0	967			0	967
TCGA-BP	TCGA-BP	KIRC	59	MALE	WHITE	Stage I	Kidney Cl	G2	2007	-21891	Alive	TUMOR F	714				0	714	0	714			0	714
TCGA-BP	TCGA-BP	KIRC	59	MALE	WHITE	Stage I	Kidney Cl	G2	2007	-21891	Alive	TUMOR F	714				0	714	0	714			0	714
TCGA-BP	TCGA-BP	KIRC	39	MALE	WHITE	Stage I	Kidney Cl	G2	2008	-14551	Alive	TUMOR F	408				0	408	0	408			0	408
TCGA-BP	TCGA-BP	KIRC	39	MALE	WHITE	Stage I	Kidney Cl	G2	2008	-14551	Alive	TUMOR F	408				0	408	0	408			0	408
TCGA-BP	TCGA-BP	KIRC	75	MALE	WHITE	Stage I	Kidney Cl	G2	2008	-27547	Alive	TUMOR F	749				0	749	0	749			0	749
TCGA-BP	TCGA-BP	KIRC	75	MALE	WHITE	Stage I	Kidney Cl	G2	2008	-27547	Alive	TUMOR F	749				0	749	0	749			0	749
TCGA-BP	TCGA-BP	KIRC	53	MALE	WHITE	Stage I	Kidney Cl	G2	2008	-19719	Alive	TUMOR F	1018				0	1018	0	1018			0	1018
TCGA-BP	TCGA-BP	KIRC	53	MALE	WHITE	Stage I	Kidney Cl	G2	2008	-19719	Alive	TUMOR F	1018				0	1018	0	1018			0	1018
TCGA-BP	TCGA-BP	KIRC	72	MALE	WHITE	Stage III	Kidney Cl	G3	2008	-26360	Alive	TUMOR F	603				0	603	0	603			0	603
TCGA-BP	TCGA-BP	KIRC	72	MALE	WHITE	Stage III	Kidney Cl	G3	2008	-26360	Alive	TUMOR F	603				0	603	0	603			0	603
TCGA-BP	TCGA-BP	KIRC	58	MALE	WHITE	Stage II	Kidney Cl	G4	2006	-21256	Alive	TUMOR F	1355				0	1355	0	1355			0	1355
TCGA-BP	TCGA-BP	KIRC	58	MALE	WHITE	Stage II	Kidney Cl	G4	2006	-21256	Alive	TUMOR F	1355				0	1355	0	1355			0	1355
TCGA-BP	TCGA-BP	KIRC	44	MALE	WHITE	Stage II	Kidney Cl	G4	2008	-16197	Alive	TUMOR F	1063				0	1063	0	1063			0	1063
TCGA-BP	TCGA-BP	KIRC	44	MALE	WHITE	Stage II	Kidney Cl	G4	2008	-16197	Alive	TUMOR F	1063				0	1063	0	1063			0	1063
TCGA-BP	TCGA-BP	KIRC	63	MALE	WHITE	Stage IV	Kidney Cl	G4	2008	-23125	Alive	WITH TUI	951		-19		0	951	0	951			1	
TCGA-BP	TCGA-BP	KIRC	63	MALE	WHITE	Stage IV	Kidney Cl	G4	2008	-23125	Alive	WITH TUI	951		-19		0	951	0	951			1	
TCGA-BP	TCGA-BP	KIRC	75	MALE	WHITE	Stage III	Kidney Cl	G2	2008	-27556	Alive	TUMOR F	29				0	29	0	29			0	29
TCGA-BP	TCGA-BP	KIRC	75	MALE	WHITE	Stage III	Kidney Cl	G2	2008	-27556	Alive	TUMOR F	29				0	29	0	29			0	29
TCGA-CJ-	TCGA-CJ-	KIRC	60	FEMALE	WHITE	Stage I	Kidney Cl	G2	2004	-22155	Alive	TUMOR F	3498			Complete Remission/Response	0	3498	0	3498	0	3498	0	3498
TCGA-CJ-	TCGA-CJ-	KIRC	60	FEMALE	WHITE	Stage I	Kidney Cl	G2	2004	-22155	Alive	TUMOR F	3498			Complete Remission/Response	0	3498	0	3498	0	3498	0	3498
TCGA-CJ-	TCGA-CJ-	KIRC	48	MALE	WHITE	Stage I	Kidney Cl	G3	2004	-17620	Alive	TUMOR F	1416				0	1416	0	1416			0	1416
TCGA-CJ-	TCGA-CJ-	KIRC	48	MALE	WHITE	Stage I	Kidney Cl	G3	2004	-17620	Alive	TUMOR F	1416				0	1416	0	1416			0	1416
TCGA-CJ-	TCGA-CJ-	KIRC	51	MALE	WHITE	Stage III	Kidney Cl	G3	2004	-18878	Alive	TUMOR F	1924				0	1924	0	1924			0	1924
TCGA-CJ-	TCGA-CJ-	KIRC	51	MALE	WHITE	Stage III	Kidney Cl	G3	2004	-18878	Alive	TUMOR F	1924				0	1924	0	1924			0	1924
TCGA-CJ-	TCGA-CJ-	KIRC	52	FEMALE	WHITE	Stage IV	Kidney Cl	G4	2004	-19101	Dead	WITH TUMOR		2227	1821		1	2227	1	2227			1	1821
TCGA-CJ-	TCGA-CJ-	KIRC	52	FEMALE	WHITE	Stage IV	Kidney Cl	G4	2004	-19101	Dead	WITH TUMOR		2227	1821		1	2227	1	2227			1	1821
TCGA-CJ-	TCGA-CJ-	KIRC	46	FEMALE	WHITE	Stage IV	Kidney Cl	G4	2004	-17110	Dead	WITH TUMOR		431	70		1	431	1	431			1	70
TCGA-CJ-	TCGA-CJ-	KIRC	46	FEMALE	WHITE	Stage IV	Kidney Cl	G4	2004	-17110	Dead	WITH TUMOR		431	70		1	431	1	431			1	70
TCGA-CJ-	TCGA-CJ-	KIRC	49	FEMALE	WHITE	Stage II	Kidney Cl	G2	2004	-18066	Alive	TUMOR F	3229			Complete Remission/Response	0	3229	0	3229	0	3229	0	3229
TCGA-CJ-	TCGA-CJ-	KIRC	49	FEMALE	WHITE	Stage II	Kidney Cl	G2	2004	-18066	Alive	TUMOR F	3229			Complete Remission/Response	0	3229	0	3229	0	3229	0	3229
TCGA-CJ-	TCGA-CJ-	KIRC	49	MALE	WHITE	Stage III	Kidney Cl	G4	2004	-18178	Alive	TUMOR F	3480			Complete Remission/Response	0	3480	0	3480	0	3480	0	3480
TCGA-CJ-	TCGA-CJ-	KIRC	49	MALE	WHITE	Stage III	Kidney Cl	G4	2004	-18178	Alive	TUMOR F	3480			Complete Remission/Response	0	3480	0	3480	0	3480	0	3480
TCGA-CJ-	TCGA-CJ-	KIRC	55	FEMALE	WHITE	Stage IV	Kidney Cl	G4	2005	-20410	Dead	WITH TUMOR		1661	-359		1	1661	1	1661			1	
TCGA-CJ-	TCGA-CJ-	KIRC	55	FEMALE	WHITE	Stage IV	Kidney Cl	G4	2005	-20410	Dead	WITH TUMOR		1661	-359		1	1661	1	1661			1	
TCGA-CJ-	TCGA-CJ-	KIRC	47	MALE	WHITE	Stage II	Kidney Cl	G2	2005	-17367	Alive	TUMOR F	3205			Complete Remission/Response	0	3205	0	3205	0	3205	0	3205
TCGA-CJ-	TCGA-CJ-	KIRC	47	MALE	WHITE	Stage II	Kidney Cl	G2	2005	-17367	Alive	TUMOR F	3205			Complete Remission/Response	0	3205	0	3205	0	3205	0	3205
TCGA-CJ-	TCGA-CJ-	KIRC	67	FEMALE	WHITE	Stage II	Kidney Cl	G3	2005	-24477	Alive	TUMOR F	1793				0	1793	0	1793			0	1793
TCGA-CJ-	TCGA-CJ-	KIRC	67	FEMALE	WHITE	Stage II	Kidney Cl	G3	2005	-24477	Alive	TUMOR F	1793				0	1793	0	1793			0	1793
TCGA-CJ-	TCGA-CJ-	KIRC	48	FEMALE	WHITE	Stage IV	Kidney Cl	G3	2004	-17882	Dead	WITH TUMOR		336	93		1	336	1	336			1	93
TCGA-CJ-	TCGA-CJ-	KIRC	48	FEMALE	WHITE	Stage IV	Kidney Cl	G3	2004	-17882	Dead	WITH TUMOR		336	93		1	336	1	336			1	93
TCGA-CJ-	TCGA-CJ-	KIRC	42	MALE	WHITE	Stage IV	Kidney Cl	G3	2005	-15601	Dead	WITH TUI	646	646	37		1	646	1	646			1	37
TCGA-CJ-	TCGA-CJ-	KIRC	42	MALE	WHITE	Stage IV	Kidney Cl	G3	2005	-15601	Dead	WITH TUI	646	646	37		1	646	1	646			1	37
TCGA-CJ-	TCGA-CJ-	KIRC	49	MALE	WHITE	Stage III	Kidney Cl	G2	2004	-17923	Alive	WITH TUI	2554		1754		0	2554	0	2554			1	1754
TCGA-CJ-	TCGA-CJ-	KIRC	49	MALE	WHITE	Stage III	Kidney Cl	G2	2004	-17923	Alive	WITH TUI	2554		1754		0	2554	0	2554			1	1754
TCGA-CJ-	TCGA-CJ-	KIRC	58	FEMALE	BLACK OI	Stage III	Kidney Cl	G2	2004	-21340	Alive	TUMOR F	1498				0	1498	0	1498			0	1498
TCGA-CJ-	TCGA-CJ-	KIRC	58	FEMALE	BLACK OI	Stage III	Kidney Cl	G2	2004	-21340	Alive	TUMOR F	1498				0	1498	0	1498			0	1498
TCGA-CJ-	TCGA-CJ-	KIRC	63	MALE	WHITE	Stage IV	Kidney Cl	G4	2004	-23195	Alive	TUMOR F	2423		169		0	2423	0	2423			1	169
TCGA-CJ-	TCGA-CJ-	KIRC	63	MALE	WHITE	Stage IV	Kidney Cl	G4	2004	-23195	Alive	TUMOR F	2423		169		0	2423	0	2423			1	169
TCGA-CJ-	TCGA-CJ-	KIRC	51	MALE	WHITE	Stage I	Kidney Cl	G4	2004	-18862	Alive	TUMOR F	1435				0	1435	0	1435			0	1435
TCGA-CJ-	TCGA-CJ-	KIRC	51	MALE	WHITE	Stage I	Kidney Cl	G4	2004	-18862	Alive	TUMOR F	1435				0	1435	0	1435			0	1435
TCGA-CJ-	TCGA-CJ-	KIRC	85	FEMALE	WHITE	Stage III	Kidney Cl	G3	2004	-31096	Alive	TUMOR F	2259				0	2259	0	2259			0	2259

TCGA-CJ-	TCGA-CJ-	KIRC	85	FEMALE	WHITE	Stage III		Kidney Cl	G3	2004	-31096	Alive	TUMOR F	2259				0	2259	0	2259			0	2259
TCGA-CJ-	TCGA-CJ-	KIRC	73	FEMALE	WHITE	Stage I		Kidney Cl	G3	2004	-26761	Alive	TUMOR F	2283				0	2283	0	2283			0	2283
TCGA-CJ-	TCGA-CJ-	KIRC	73	FEMALE	WHITE	Stage I		Kidney Cl	G3	2004	-26761	Alive	TUMOR F	2283				0	2283	0	2283			0	2283
TCGA-CJ-	TCGA-CJ-	KIRC	67	MALE	WHITE	Stage IV		Kidney Cl	G3	2004	-24510	Dead	WITH TUMOR		3554	531	Complete Remission/Response	1	3554	1	3554			1	531
TCGA-CJ-	TCGA-CJ-	KIRC	67	MALE	WHITE	Stage IV		Kidney Cl	G3	2004	-24510	Dead	WITH TUMOR		3554	531	Complete Remission/Response	1	3554	1	3554			1	531
TCGA-CJ-	TCGA-CJ-	KIRC	57	MALE	WHITE	Stage II		Kidney Cl	G3	2004	-20993	Alive	TUMOR F	1955				0	1955	0	1955			0	1955
TCGA-CJ-	TCGA-CJ-	KIRC	57	MALE	WHITE	Stage II		Kidney Cl	G3	2004	-20993	Alive	TUMOR F	1955				0	1955	0	1955			0	1955
TCGA-CJ-	TCGA-CJ-	KIRC	71	FEMALE	WHITE	Stage III		Kidney Cl	G2	2004	-26224	Alive	TUMOR F	2186				0	2186	0	2186			0	2186
TCGA-CJ-	TCGA-CJ-	KIRC	71	FEMALE	WHITE	Stage III		Kidney Cl	G2	2004	-26224	Alive	TUMOR F	2186				0	2186	0	2186			0	2186
TCGA-CJ-	TCGA-CJ-	KIRC	41	MALE	WHITE	Stage III		Kidney Cl	G3	2004	-15197	Alive	WITH TUI	2014		1881		0	2014	0	2014			1	1881
TCGA-CJ-	TCGA-CJ-	KIRC	41	MALE	WHITE	Stage III		Kidney Cl	G3	2004	-15197	Alive	WITH TUI	2014		1881		0	2014	0	2014			1	1881
TCGA-CJ-	TCGA-CJ-	KIRC	57	MALE	BLACK OI	Stage III		Kidney Cl	G3	2005	-21074	Alive	TUMOR F	1883				0	1883	0	1883			0	1883
TCGA-CJ-	TCGA-CJ-	KIRC	57	MALE	BLACK OI	Stage III		Kidney Cl	G3	2005	-21074	Alive	TUMOR F	1883				0	1883	0	1883			0	1883
TCGA-CJ-	TCGA-CJ-	KIRC	72	FEMALE	WHITE	Stage III		Kidney Cl	G3	2005	-26475	Alive	TUMOR F	1759				0	1759	0	1759			0	1759
TCGA-CJ-	TCGA-CJ-	KIRC	72	FEMALE	WHITE	Stage III		Kidney Cl	G3	2005	-26475	Alive	TUMOR F	1759				0	1759	0	1759			0	1759
TCGA-CJ-	TCGA-CJ-	KIRC	64	MALE	WHITE	Stage IV		Kidney Cl	G3	2005	-23437	Alive	TUMOR F	3451		2041	Complete Remission/Response	0	3451	0	3451			1	2041
TCGA-CJ-	TCGA-CJ-	KIRC	64	MALE	WHITE	Stage IV		Kidney Cl	G3	2005	-23437	Alive	TUMOR F	3451		2041	Complete Remission/Response	0	3451	0	3451			1	2041
TCGA-CJ-	TCGA-CJ-	KIRC	42	FEMALE	WHITE	Stage I		Kidney Cl	G3	2005	-15698	Alive	TUMOR F	1952				0	1952	0	1952			0	1952
TCGA-CJ-	TCGA-CJ-	KIRC	42	FEMALE	WHITE	Stage I		Kidney Cl	G3	2005	-15698	Alive	TUMOR F	1952				0	1952	0	1952			0	1952
TCGA-CJ-	TCGA-CJ-	KIRC	48	MALE	WHITE	Stage IV		Kidney Cl	G3	2005	-17611	Dead	WITH TUMOR		932	322		1	932	1	932			1	322
TCGA-CJ-	TCGA-CJ-	KIRC	48	MALE	WHITE	Stage IV		Kidney Cl	G3	2005	-17611	Dead	WITH TUMOR		932	322		1	932	1	932			1	322
TCGA-CJ-	TCGA-CJ-	KIRC	59	MALE	WHITE	Stage IV		Kidney Cl	G4	2005	-21701	Dead	WITH TUMOR		1567	608		1	1567	1	1567			1	608
TCGA-CJ-	TCGA-CJ-	KIRC	59	MALE	WHITE	Stage IV		Kidney Cl	G4	2005	-21701	Dead	WITH TUMOR		1567	608		1	1567	1	1567			1	608
TCGA-CJ-	TCGA-CJ-	KIRC	63	FEMALE	WHITE	Stage I		Kidney Cl	G4	2005	-23051	Alive	TUMOR F	1946				0	1946	0	1946			0	1946
TCGA-CJ-	TCGA-CJ-	KIRC	63	FEMALE	WHITE	Stage I		Kidney Cl	G4	2005	-23051	Alive	TUMOR F	1946				0	1946	0	1946			0	1946
TCGA-CJ-	TCGA-CJ-	KIRC	72	MALE	WHITE	Stage IV		Kidney Cl	G4	2005	-26604	Alive	TUMOR F	3519		70	Progressive Disease	0	3519	0	3519			1	70
TCGA-CJ-	TCGA-CJ-	KIRC	72	MALE	WHITE	Stage IV		Kidney Cl	G4	2005	-26604	Alive	TUMOR F	3519		70	Progressive Disease	0	3519	0	3519			1	70
TCGA-CJ-	TCGA-CJ-	KIRC	57	FEMALE	WHITE	Stage III		Kidney Cl	G4	2005	-21059	Dead	TUMOR FREE		819			1	819	0	819			0	819
TCGA-CJ-	TCGA-CJ-	KIRC	57	FEMALE	WHITE	Stage III		Kidney Cl	G4	2005	-21059	Dead	TUMOR FREE		819			1	819	0	819			0	819
TCGA-CJ-	TCGA-CJ-	KIRC	65	FEMALE	WHITE	Stage I		Kidney Cl	G2	2005	-23764	Alive	TUMOR F	1521				0	1521	0	1521			0	1521
TCGA-CJ-	TCGA-CJ-	KIRC	65	FEMALE	WHITE	Stage I		Kidney Cl	G2	2005	-23764	Alive	TUMOR F	1521				0	1521	0	1521			0	1521
TCGA-CJ-	TCGA-CJ-	KIRC	76	FEMALE	WHITE	Stage I		Kidney Cl	G3	2005	-27885	Alive	TUMOR F	750				0	750	0	750			0	750
TCGA-CJ-	TCGA-CJ-	KIRC	76	FEMALE	WHITE	Stage I		Kidney Cl	G3	2005	-27885	Alive	TUMOR F	750				0	750	0	750			0	750
TCGA-CJ-	TCGA-CJ-	KIRC	58	MALE	WHITE	Stage III		Kidney Cl	G3	2005	-21524	Dead	WITH TUI	841	841	158		1	841	1	841			1	158
TCGA-CJ-	TCGA-CJ-	KIRC	58	MALE	WHITE	Stage III		Kidney Cl	G3	2005	-21524	Dead	WITH TUI	841	841	158		1	841	1	841			1	158
TCGA-CJ-	TCGA-CJ-	KIRC	62	MALE	WHITE	Stage IV		Kidney Cl	G4	2005	-22892	Dead	WITH TUMOR		1200	61		1	1200	1	1200			1	61
TCGA-CJ-	TCGA-CJ-	KIRC	62	MALE	WHITE	Stage IV		Kidney Cl	G4	2005	-22892	Dead	WITH TUMOR		1200	61		1	1200	1	1200			1	61
TCGA-CJ-	TCGA-CJ-	KIRC	79	FEMALE	WHITE	Stage III		Kidney Cl	G3	2005	-29124	Alive	WITH TUI	3341		1423	Complete Remission/Response	0	3341	0	3341	1	1423	1	1423
TCGA-CJ-	TCGA-CJ-	KIRC	79	FEMALE	WHITE	Stage III		Kidney Cl	G3	2005	-29124	Alive	WITH TUI	3341		1423	Complete Remission/Response	0	3341	0	3341	1	1423	1	1423
TCGA-CJ-	TCGA-CJ-	KIRC	42	MALE	ASIAN	Stage I		Kidney Cl	G2	2006	-15637	Alive	TUMOR F	1528				0	1528	0	1528			0	1528
TCGA-CJ-	TCGA-CJ-	KIRC	42	MALE	ASIAN	Stage I		Kidney Cl	G2	2006	-15637	Alive	TUMOR F	1528				0	1528	0	1528			0	1528
TCGA-CJ-	TCGA-CJ-	KIRC	69	FEMALE	WHITE	Stage IV		Kidney Cl	G4	2006	-25508	Dead	WITH TUMOR		1714			1	1714	1	1714			1	1714
TCGA-CJ-	TCGA-CJ-	KIRC	69	FEMALE	WHITE	Stage IV		Kidney Cl	G4	2006	-25508	Dead	WITH TUMOR		1714			1	1714	1	1714			1	1714
TCGA-CJ-	TCGA-CJ-	KIRC	47	MALE	WHITE	Stage III		Kidney Cl	G3	2006	-17396	Alive	TUMOR F	1450				0	1450	0	1450			0	1450
TCGA-CJ-	TCGA-CJ-	KIRC	47	MALE	WHITE	Stage III		Kidney Cl	G3	2006	-17396	Alive	TUMOR F	1450				0	1450	0	1450			0	1450
TCGA-CJ-	TCGA-CJ-	KIRC	61	MALE	WHITE	Stage III		Kidney Cl	G3	2006	-22424	Alive	TUMOR F	1520				0	1520	0	1520			0	1520
TCGA-CJ-	TCGA-CJ-	KIRC	61	MALE	WHITE	Stage III		Kidney Cl	G3	2006	-22424	Alive	TUMOR F	1520				0	1520	0	1520			0	1520
TCGA-CJ-	TCGA-CJ-	KIRC	50	MALE	WHITE	Stage I		Kidney Cl	G3	2006	-18510	Alive	TUMOR F	1560				0	1560	0	1560			0	1560
TCGA-CJ-	TCGA-CJ-	KIRC	50	MALE	WHITE	Stage I		Kidney Cl	G3	2006	-18510	Alive	TUMOR F	1560				0	1560	0	1560			0	1560
TCGA-CJ-	TCGA-CJ-	KIRC	60	FEMALE	WHITE	Stage IV		Kidney Cl	G3	2006	-22036	Alive	WITH TUI	3302		278	Stable Disease	0	3302	0	3302			1	278
TCGA-CJ-	TCGA-CJ-	KIRC	60	FEMALE	WHITE	Stage IV		Kidney Cl	G3	2006	-22036	Alive	WITH TUI	3302		278	Stable Disease	0	3302	0	3302			1	278
TCGA-CJ-	TCGA-CJ-	KIRC	62	FEMALE	WHITE	Stage I		Kidney Cl	G2	2006	-22864	Alive	TUMOR F	1496				0	1496	0	1496			0	1496
TCGA-CJ-	TCGA-CJ-	KIRC	62	FEMALE	WHITE	Stage I		Kidney Cl	G2	2006	-22864	Alive	TUMOR F	1496				0	1496	0	1496			0	1496
TCGA-CJ-	TCGA-CJ-	KIRC	58	MALE	WHITE	Stage III		Kidney Cl	G3	2006	-21208	Alive	TUMOR F	1499				0	1499	0	1499			0	1499
TCGA-CJ-	TCGA-CJ-	KIRC	58	MALE	WHITE	Stage III		Kidney Cl	G3	2006	-21208	Alive	TUMOR F	1499				0	1499	0	1499			0	1499
TCGA-CJ-	TCGA-CJ-	KIRC	38	MALE	WHITE	Stage I		Kidney Cl	G2	2006	-14226	Alive	TUMOR F	1531				0	1531	0	1531			0	1531
TCGA-CJ-	TCGA-CJ-	KIRC	38	MALE	WHITE	Stage I		Kidney Cl	G2	2006	-14226	Alive	TUMOR F	1531				0	1531	0	1531			0	1531
TCGA-CJ-	TCGA-CJ-	KIRC	61	MALE	WHITE	Stage II		Kidney Cl	G3	2006	-22623	Alive	TUMOR F	1657				0	1657	0	1657			0	1657
TCGA-CJ-	TCGA-CJ-	KIRC	61	MALE	WHITE	Stage II		Kidney Cl	G3	2006	-22623	Alive	TUMOR F	1657				0	1657	0	1657			0	1657
TCGA-CJ-	TCGA-CJ-	KIRC	45	FEMALE	WHITE	Stage III		Kidney Cl	G4	2006	-16757	Dead	WITH TUMOR		1173	538		1	1173	1	1173			1	538
TCGA-CJ-	TCGA-CJ-	KIRC	45	FEMALE	WHITE	Stage III		Kidney Cl	G4	2006	-16757	Dead	WITH TUMOR		1173	538		1	1173	1	1173			1	538
TCGA-CJ-	TCGA-CJ-	KIRC	69	FEMALE	WHITE	Stage III		Kidney Cl	G3	2007	-25470	Alive	TUMOR F	1373				0	1373	0	1373			0	1373
TCGA-CJ-	TCGA-CJ-	KIRC	69	FEMALE	WHITE	Stage III		Kidney Cl	G3	2007	-25470	Alive	TUMOR F	1373				0	1373	0	1373			0	1373
TCGA-CJ-	TCGA-CJ-	KIRC	64	MALE	WHITE	Stage IV		Kidney Cl	G4	2005	-23379	Dead	WITH TUMOR		93	33		1	93	1	93			1	33
TCGA-CJ-	TCGA-CJ-	KIRC	64	MALE	WHITE	Stage IV		Kidney Cl	G4	2005	-23379	Dead	WITH TUMOR		93	33		1	93	1	93			1	33
TCGA-CJ-	TCGA-CJ-	KIRC	64	FEMALE	WHITE	Stage I		Kidney Cl	G2	2004	-23656	Dead	TUMOR FREE		139			1	139	0	139			0	139
TCGA-CJ-	TCGA-CJ-	KIRC	64	FEMALE	WHITE	Stage I		Kidney Cl	G2	2004	-23656	Dead	TUMOR FREE		139			1	139	0	139			0	139
TCGA-CJ-	TCGA-CJ-	KIRC	63	FEMALE	WHITE	Stage IV		Kidney Cl	G4	2004	-23353	Dead	WITH TUMOR		572	25		1	572	1	572			1	25
TCGA-CJ-	TCGA-CJ-	KIRC	63	FEMALE	WHITE	Stage IV		Kidney Cl	G4	2004	-23353	Dead	WITH TUMOR		572	25		1	572	1	572			1	25
TCGA-CJ-	TCGA-CJ-	KIRC	51	MALE	WHITE	Stage I		Kidney Cl	G3	2004	-18954	Alive	TUMOR F	3987			Complete Remission/Response	0	3987	0	3987	0	3987	0	3987
TCGA-CJ-	TCGA-CJ-	KIRC	84	MALE	WHITE	Stage I		Kidney Cl	G3	2004	-30900	Dead	TUMOR FREE		2190			1	2190	0	2190			0	2190
TCGA-CJ-	TCGA-CJ-	KIRC	84	MALE	WHITE	Stage I		Kidney Cl	G3	2004	-30900	Dead	TUMOR FREE		2190			1	2190	0	2190			0	2190
TCGA-CJ-	TCGA-CJ-	KIRC	70	MALE	WHITE	Stage II		Kidney Cl	G3	2004	-25617	Alive	WITH TUI	3936		900	Complete Remission/Response	0	3936	0	3936	1	900	1	900
TCGA-CJ-	TCGA-CJ-	KIRC	47	MALE	WHITE	Stage III		Kidney Cl	G3	2004	-17170	Alive	WITH TUI	4067		2219	Complete Remission/Response	0	4067	0	4067	1	2219	1	2219
TCGA-CJ-	TCGA-CJ-	KIRC	47	MALE	WHITE	Stage III		Kidney Cl	G3	2004	-17170	Alive	WITH TUI	4067		2219	Complete Remission/Response	0	4067	0	4067	1	2219	1	2219
TCGA-CJ-	TCGA-CJ-	KIRC	54	FEMALE	WHITE	Stage IV		Kidney Cl	G4	2004	-19849	Dead	WITH TUMOR		782	288		1	782	1	782			1	288
TCGA-CJ-	TCGA-CJ-	KIRC	54	FEMALE	WHITE	Stage IV		Kidney Cl	G4	2004	-19849	Dead	WITH TUMOR		782	288		1	782	1	782			1	288
TCGA-CJ-	TCGA-CJ-	KIRC	62	MALE	BLACK OI	Stage IV		Kidney Cl	G3	2003	-22944	Dead	WITH TUMOR		574	399		1	574	1	574			1	399
TCGA-CJ-	TCGA-CJ-	KIRC	62	MALE	BLACK OI	Stage IV		Kidney Cl	G3	2003	-22944	Dead	WITH TUMOR		574	399		1	574	1	574			1	399
TCGA-CJ-	TCGA-CJ-	KIRC	73	MALE	WHITE	Stage III		Kidney Cl	G4	2003	-26858	Dead	WITH TUMOR		679	595		1	679	1	679			1	595
TCGA-CJ-	TCGA-CJ-	KIRC	73	MALE	WHITE	Stage III		Kidney Cl	G4	2003	-26858	Dead	WITH TUMOR		679	595		1	679	1	679			1	595
TCGA-CJ-	TCGA-CJ-	KIRC	65	FEMALE	WHITE	Stage IV		Kidney Cl	G4	2003	-23778	Dead	WITH TUMOR		768	153		1	768	1	768			1	153
TCGA-CJ-	TCGA-CJ-	KIRC	65	FEMALE	WHITE	Stage IV		Kidney Cl	G4	2003	-23778	Dead	WITH TUMOR		768	153		1	768	1	768			1	153
TCGA-CJ-	TCGA-CJ-	KIRC	44	FEMALE	WHITE	Stage IV		Kidney Cl	G3	2003	-16121	Dead	WITH TUMOR		552	17		1	552	1	552			1	17
TCGA-CJ-	TCGA-CJ-	KIRC	44	FEMALE	WHITE	Stage IV		Kidney Cl	G3	2003	-16121	Dead	WITH TUMOR		552	17		1	552	1	552			1	17
TCGA-CJ-	TCGA-CJ-	KIRC	60	MALE	WHITE	Stage IV		Kidney Cl	G4	2004	-22244	Alive	WITH TUI	3736		55	Stable Disease	0	3736	0	3736			1	55
TCGA-CJ-	TCGA-CJ-	KIRC	78	MALE	WHITE	Stage I		Kidney Cl	G3	2004	-28745	Alive	TUMOR F	1889				0	1889	0	1889			0	1889
TCGA-CJ-	TCGA-CJ-	KIRC	61	MALE	WHITE	Stage III		Kidney Cl	G2	2005	-22561	Alive	TUMOR F	2231				0	2231	0	2231			0	2231
TCGA-CJ-	TCGA-CJ-	KIRC	59	FEMALE	WHITE	Stage I		Kidney Cl	G3	2005	-21836	Alive	TUMOR F	2038				0	2038	0	2038			0	2038
TCGA-CJ-	TCGA-CJ-	KIRC	90	MALE	WHITE	Stage I		Kidney Cl	G4	2005	-32872	Dead	TUMOR FREE		1620			1	1620	0	1620			0	1620
TCGA-CJ-	TCGA-CJ-	KIRC	90	MALE	WHITE	Stage I		Kidney Cl	G4	2005	-32872	Dead	TUMOR FREE		1620			1	1620	0	1620			0	1620
TCGA-CJ-	TCGA-CJ-	KIRC	77	MALE	WHITE	Stage I		Kidney Cl	G4	2003	-28359	Dead	TUMOR FREE		3615		Complete Remission/Response	1	3615	0	3615	0	3615	0	3615
TCGA-CJ-	TCGA-CJ-	KIRC	58	MALE	WHITE	Stage IV		Kidney Cl	G4	2003	-21380	Dead	WITH TUMOR		1625	137		1	1625	1	1625			1	137
TCGA-CJ-	TCGA-CJ-	KIRC	65	MALE	WHITE	Stage I		Kidney Cl	G3	2003	-24096	Dead	TUMOR FREE		2299			1	2299	0	2299			0	2299
TCGA-CJ-	TCGA-CJ-	KIRC	65	MALE	WHITE	Stage I		Kidney Cl	G3	2003	-24096	Dead	TUMOR FREE		2299			1	2299	0	2299			0	2299
TCGA-CJ-	TCGA-CJ-	KIRC	54	MALE	WHITE	Stage I		Kidney Cl	G3	2003	-19905	Alive	TUMOR F	1906				0	1906	0	1906			0	1906
TCGA-CJ-	TCGA-CJ-	KIRC	63	FEMALE	WHITE	Stage II		Kidney Cl	G3	2004	-23310	Alive	TUMOR F	3639			Complete Remission/Response	0	3639	0	3639	0	3639	0	3639
TCGA-CJ-	TCGA-CJ-	KIRC	54	FEMALE	WHITE	Stage IV		Kidney Cl	G4	2004	-19919	Dead	WITH TUMOR		224	144		1	224	1	224			1	144
TCGA-CJ-	TCGA-CJ-	KIRC	54	FEMALE	WHITE	Stage IV		Kidney Cl	G4	2004	-19919	Dead	WITH TUMOR		224	144		1	224	1	224			1	144
TCGA-CW	TCGA-CW	KIRC	73	FEMALE	WHITE	Stage IV		Kidney Cl	G3	2003	-26696	Dead			1964			1	1964		1964			0	1964
TCGA-CW	TCGA-CW	KIRC	73	FEMALE	WHITE	Stage IV		Kidney Cl	G3	2003	-26696	Dead			1964			1	1964		1964			0	1964
TCGA-CW	TCGA-CW	KIRC	44	MALE	WHITE	Stage I		Kidney Cl	G3	2003	-16195	Alive	TUMOR F	2799				0	2799	0	2799			0	2799
TCGA-CW	TCGA-CW	KIRC	44	MALE	WHITE	Stage I		Kidney Cl	G3	2003	-16195	Alive	TUMOR F	2799				0	2799	0	2799			0	2799
TCGA-CW	TCGA-CW	KIRC	51	FEMALE	WHITE	Stage I		Kidney Cl	G2	2003	-18666	Alive	TUMOR F	2489				0	2489	0	2489			0	2489
TCGA-CW	TCGA-CW	KIRC	74	MALE	WHITE	Stage III		Kidney Cl	G3	2003	-27352	Dead			164	102		1	164		164			1	102
TCGA-CW	TCGA-CW	KIRC	74	MALE	WHITE	Stage III		Kidney Cl	G3	2003	-27352	Dead			164	102		1	164		164			1	102
TCGA-CW	TCGA-CW	KIRC	51	MALE	WHITE	Stage IV		Kidney Cl	G2	2003	-18898	Alive	WITH TUI	2609				0	2609	0	2609			0	2609
TCGA-CW	TCGA-CW	KIRC	51	MALE	WHITE	Stage IV		Kidney Cl	G2	2003	-18898	Alive	WITH TUI	2609				0	2609	0	2609			0	2609
TCGA-CW	TCGA-CW	KIRC	62	FEMALE	WHITE	Stage III		Kidney Cl	G2	2003	-22693	Alive	TUMOR F	2226		2003		0	2226	0	2226			1	2003
TCGA-CW	TCGA-CW	KIRC	62	FEMALE	WHITE	Stage III		Kidney Cl	G2	2003	-22693	Alive	TUMOR F	2226		2003		0	2226	0	2226			1	2003
TCGA-CW	TCGA-CW	KIRC	78	FEMALE	WHITE	Stage I		Kidney Cl	G2	2004	-28714	Alive		2017				0	2017	0	2017			0	2017
TCGA-CW	TCGA-CW	KIRC	52	MALE	WHITE	Stage I		Kidney Cl	G2	2004	-19312	Alive	TUMOR F	2378				0	2378	0	2378			0	2378
TCGA-CW	TCGA-CW	KIRC	52	MALE	WHITE	Stage I		Kidney Cl	G2	2004	-19312	Alive	TUMOR F	2378				0	2378	0	2378			0	2378
TCGA-CW	TCGA-CW	KIRC	51	MALE	WHITE	Stage IV		Kidney Cl	G3	2004	-18905	Dead			1075	672		1	1075		1075			1	672
TCGA-CW	TCGA-CW	KIRC	56	MALE	WHITE	Stage IV		Kidney Cl	G2	2004	-20712	Alive	WITH TUI	2271				0	2271	0	2271			0	2271
TCGA-CW	TCGA-CW	KIRC	56	MALE	WHITE	Stage IV		Kidney Cl	G2	2004	-20712	Alive	WITH TUI	2271				0	2271	0	2271			0	2271
TCGA-CW	TCGA-CW	KIRC	61	MALE	WHITE	Stage IV		Kidney Cl	G4	2003	-22438	Dead	TUMOR FREE		41			1	41	0	41			0	41
TCGA-CW	TCGA-CW	KIRC	61	MALE	WHITE	Stage IV		Kidney Cl	G4	2003	-22438	Dead	TUMOR FREE		41			1	41	0	41			0	41
TCGA-CW	TCGA-CW	KIRC	60	MALE	WHITE	Stage I		Kidney Cl	G2	2003	-22020	Alive	TUMOR F	3222			Stable Disease	0	3222	0	3222			0	3222
TCGA-CW	TCGA-CW	KIRC	60	MALE	WHITE	Stage I		Kidney Cl	G2	2003	-22020	Alive	TUMOR F	3222			Stable Disease	0	3222	0	3222			0	3222
TCGA-CW	TCGA-CW	KIRC	68	MALE	WHITE	Stage I		Kidney Cl	G3	2003	-24909	Alive	TUMOR F	2552		2780		0	2552	0	2552			1	2780
TCGA-CW	TCGA-CW	KIRC	68	MALE	WHITE	Stage I		Kidney Cl	G3	2003	-24909	Alive	TUMOR F	2552		2780		0	2552	0	2552			1	2780
TCGA-CW	TCGA-CW	KIRC	73	MALE	WHITE	Stage I		Kidney Cl	G1	2004	-26909	Alive	TUMOR F	3146			Stable Disease	0	3146	0	3146			0	3146
TCGA-CW	TCGA-CW	KIRC	32	MALE	WHITE	Stage III		Kidney Cl	G4	2004	-11701	Dead	WITH TUMOR		571	457		1	571	1	571			1	457
TCGA-CZ	TCGA-CZ	KIRC	82	MALE	WHITE	Stage I		Kidney Cl	G2	2005	-30191	Alive	TUMOR F	774				0	774	0	774			0	774
TCGA-CZ	TCGA-CZ	KIRC	82	MALE	WHITE	Stage I		Kidney Cl	G2	2005	-30191	Alive	TUMOR F	774				0	774	0	774			0	774
TCGA-CZ	TCGA-CZ	KIRC	68	MALE	WHITE	Stage I		Kidney Cl	G2	2005	-24876	Dead	TUMOR FREE		1404			1	1404	0	1404			0	1404
TCGA-CZ	TCGA-CZ	KIRC	68	MALE	WHITE	Stage I		Kidney Cl	G2	2005	-24876	Dead	TUMOR FREE		1404			1	1404	0	1404			0	1404
TCGA-CZ	TCGA-CZ	KIRC	62	FEMALE	WHITE	Stage I		Kidney Cl	G2	2005	-22684	Alive		18				0	18	0	18			0	18
TCGA-CZ	TCGA-CZ	KIRC	62	FEMALE	WHITE	Stage I		Kidney Cl	G2	2005	-22684	Alive		18				0	18	0	18			0	18
TCGA-CZ	TCGA-CZ	KIRC	56	MALE	WHITE	Stage IV		Kidney Cl	G3	2005	-20718	Dead	WITH TUMOR		1432	188		1	1432	1	1432			1	188
TCGA-CZ	TCGA-CZ	KIRC	56	MALE	WHITE	Stage IV		Kidney Cl	G3	2005	-20718	Dead	WITH TUMOR		1432	188		1	1432	1	1432			1	188
TCGA-CZ	TCGA-CZ	KIRC	39	MALE	WHITE	Stage II		Kidney Cl	G4	2005	-14343	Dead	WITH TUMOR		2105	597		1	2105	1	2105			1	597
TCGA-CZ	TCGA-CZ	KIRC	39	MALE	WHITE	Stage II		Kidney Cl	G4	2005	-14343	Dead	WITH TUMOR		2105	597		1	2105	1	2105			1	597
TCGA-CZ	TCGA-CZ	KIRC	59	FEMALE	WHITE	Stage I		Kidney Cl	G2	2005	-21842	Alive	TUMOR F	1787				0	1787	0	1787			0	1787
TCGA-CZ	TCGA-CZ	KIRC	59	FEMALE	WHITE	Stage I		Kidney Cl	G2	2005	-21842	Alive	TUMOR F	1787				0	1787	0	1787			0	1787
TCGA-CZ	TCGA-CZ	KIRC	60	MALE	WHITE	Stage IV		Kidney Cl	G4	2005	-22115	Dead	WITH TUMOR		206			1	206	1	206			1	206
TCGA-CZ	TCGA-CZ	KIRC	60	MALE	WHITE	Stage IV		Kidney Cl	G4	2005	-22115	Dead	WITH TUMOR		206			1	206	1	206			1	206
TCGA-CZ	TCGA-CZ	KIRC	63	MALE	WHITE	Stage II		Kidney Cl	G2	2005	-23053	Dead	WITH TUMOR		446			1	446	1	446			1	446
TCGA-CZ	TCGA-CZ	KIRC	63	MALE	WHITE	Stage II		Kidney Cl	G2	2005	-23053	Dead	WITH TUMOR		446			1	446	1	446			1	446
TCGA-CZ	TCGA-CZ	KIRC	46	MALE	WHITE	Stage I		Kidney Cl	G2	2006	-17077	Alive	[Discrepai	3271				0	3271	0	3271			0	3271
TCGA-CZ	TCGA-CZ	KIRC	46	MALE	WHITE	Stage I		Kidney Cl	G2	2006	-17077	Alive	[Discrepai	3271				0	3271	0	3271			0	3271
TCGA-CZ	TCGA-CZ	KIRC	51	FEMALE	WHITE	Stage III		Kidney Cl	G3	2006	-18730	Alive	TUMOR F	1928				0	1928	0	1928			0	1928
TCGA-CZ	TCGA-CZ	KIRC	51	FEMALE	WHITE	Stage III		Kidney Cl	G3	2006	-18730	Alive	TUMOR F	1928				0	1928	0	1928			0	1928
TCGA-CZ	TCGA-CZ	KIRC	86	MALE	WHITE	Stage II		Kidney Cl	G3	2002	-31557	Dead	TUMOR F	2830	2830			1	2830	0	2830			0	2830
TCGA-CZ	TCGA-CZ	KIRC	86	MALE	WHITE	Stage II		Kidney Cl	G3	2002	-31557	Dead	TUMOR F	2830	2830			1	2830	0	2830			0	2830
TCGA-CZ	TCGA-CZ	KIRC	70	FEMALE	WHITE	Stage I		Kidney Cl	G2	2006	-25709	Dead	TUMOR FREE		166			1	166	0	166			0	166
TCGA-CZ	TCGA-CZ	KIRC	70	FEMALE	WHITE	Stage I		Kidney Cl	G2	2006	-25709	Dead	TUMOR FREE		166			1	166	0	166			0	166
TCGA-CZ	TCGA-CZ	KIRC	79	FEMALE	WHITE	Stage I		Kidney Cl	G3	2006	-28878	Alive	WITH TUI	3267				0	3267	0	3267			0	3267
TCGA-CZ	TCGA-CZ	KIRC	79	FEMALE	WHITE	Stage I		Kidney Cl	G3	2006	-28878	Alive	WITH TUI	3267				0	3267	0	3267			0	3267
TCGA-CZ	TCGA-CZ	KIRC	74	MALE	WHITE	Stage II		Kidney Cl	G3	2006	-27312	Alive	TUMOR F	1929				0	1929	0	1929			0	1929
TCGA-CZ	TCGA-CZ	KIRC	74	MALE	WHITE	Stage II		Kidney Cl	G3	2006	-27312	Alive	TUMOR F	1929				0	1929	0	1929			0	1929
TCGA-CZ	TCGA-CZ	KIRC	69	MALE	WHITE	Stage II		Kidney Cl	G2	2006	-25486	Alive	TUMOR F	1789				0	1789	0	1789			0	1789
TCGA-CZ	TCGA-CZ	KIRC	69	MALE	WHITE	Stage II		Kidney Cl	G2	2006	-25486	Alive	TUMOR F	1789				0	1789	0	1789			0	1789
TCGA-CZ	TCGA-CZ	KIRC	67	MALE	WHITE	Stage II		Kidney Cl	G2	2006	-24640	Dead			2419			1	2419		2419			0	2419
TCGA-CZ	TCGA-CZ	KIRC	67	MALE	WHITE	Stage II		Kidney Cl	G2	2006	-24640	Dead			2419			1	2419		2419			0	2419
TCGA-CZ	TCGA-CZ	KIRC	63	MALE	WHITE	Stage IV		Kidney Cl	G2	2006	-23083	Dead	WITH TUMOR		722	423		1	722	1	722			1	423
TCGA-CZ	TCGA-CZ	KIRC	63	MALE	WHITE	Stage IV		Kidney Cl	G2	2006	-23083	Dead	WITH TUMOR		722	423		1	722	1	722			1	423
TCGA-CZ	TCGA-CZ	KIRC	63	MALE	WHITE	Stage IV		Kidney Cl	G4	2006	-23150	Dead	WITH TUMOR		561			1	561	1	561			1	561
TCGA-CZ	TCGA-CZ	KIRC	63	MALE	WHITE	Stage IV		Kidney Cl	G4	2006	-23150	Dead	WITH TUMOR		561			1	561	1	561			1	561
TCGA-CZ	TCGA-CZ	KIRC	57	MALE	WHITE	Stage II		Kidney Cl	G3	2006	-21164	Alive	WITH TUI	2422		951		0	2422	0	2422			1	951
TCGA-CZ	TCGA-CZ	KIRC	57	MALE	WHITE	Stage II		Kidney Cl	G3	2006	-21164	Alive	WITH TUI	2422		951		0	2422	0	2422			1	951
TCGA-CZ	TCGA-CZ	KIRC	62	MALE	WHITE	Stage III		Kidney Cl	G4	2006	-22763	Alive	WITH TUI	2754		174		0	2754	0	2754			1	174
TCGA-CZ	TCGA-CZ	KIRC	62	MALE	WHITE	Stage III		Kidney Cl	G4	2006	-22763	Alive	WITH TUI	2754		174		0	2754	0	2754			1	174
TCGA-CZ	TCGA-CZ	KIRC	43	MALE	WHITE	Stage III		Kidney Cl	G3	2006	-15790	Alive	TUMOR F	2789				0	2789	0	2789			0	2789
TCGA-CZ	TCGA-CZ	KIRC	43	MALE	WHITE	Stage III		Kidney Cl	G3	2006	-15790	Alive	TUMOR F	2789				0	2789	0	2789			0	2789
TCGA-CZ	TCGA-CZ	KIRC	63	MALE	WHITE	Stage III		Kidney Cl	G3	2006	-23318	Alive	TUMOR F	1683				0	1683	0	1683			0	1683
TCGA-CZ	TCGA-CZ	KIRC	63	MALE	WHITE	Stage III		Kidney Cl	G3	2006	-23318	Alive	TUMOR F	1683				0	1683	0	1683			0	1683
TCGA-CZ	TCGA-CZ	KIRC	55	MALE	WHITE	Stage IV		Kidney Cl	G2	2006	-20392	Alive	WITH TUI	2873				0	2873	0	2873			0	2873
TCGA-CZ	TCGA-CZ	KIRC	55	MALE	WHITE	Stage IV		Kidney Cl	G2	2006	-20392	Alive	WITH TUI	2873				0	2873	0	2873			0	2873
TCGA-CZ	TCGA-CZ	KIRC	52	MALE	WHITE	Stage IV		Kidney Cl	G4	2006	-19030	Dead	WITH TUI	330	330	48		1	330	1	330			1	48
TCGA-CZ	TCGA-CZ	KIRC	52	MALE	WHITE	Stage IV		Kidney Cl	G4	2006	-19030	Dead	WITH TUI	330	330	48		1	330	1	330			1	48
TCGA-CZ	TCGA-CZ	KIRC	83	MALE	WHITE	Stage IV		Kidney Cl	G3	2007	-30659	Dead	WITH TUMOR		311			1	311	1	311			1	311
TCGA-CZ	TCGA-CZ	KIRC	83	MALE	WHITE	Stage IV		Kidney Cl	G3	2007	-30659	Dead	WITH TUMOR		311			1	311	1	311			1	311
TCGA-CZ	TCGA-CZ	KIRC	76	MALE	WHITE	Stage II		Kidney Cl	G2	2007	-27911	Alive		662				0	662	0	662			0	662
TCGA-CZ	TCGA-CZ	KIRC	76	MALE	WHITE	Stage II		Kidney Cl	G2	2007	-27911	Alive		662				0	662	0	662			0	662
TCGA-CZ	TCGA-CZ	KIRC	69	MALE	WHITE	Stage IV		Kidney Cl	G2	2007	-25463	Alive	TUMOR F	2128				0	2128	0	2128			0	2128
TCGA-CZ	TCGA-CZ	KIRC	69	MALE	WHITE	Stage IV		Kidney Cl	G2	2007	-25463	Alive	TUMOR F	2128				0	2128	0	2128			0	2128
TCGA-CZ	TCGA-CZ	KIRC	76	FEMALE		Stage III		Kidney Cl	G2	2007	-27838	Dead	TUMOR FREE		2564			1	2564	0	2564			0	2564
TCGA-CZ	TCGA-CZ	KIRC	76	FEMALE		Stage III		Kidney Cl	G2	2007	-27838	Dead	TUMOR FREE		2564			1	2564	0	2564			0	2564
TCGA-CZ	TCGA-CZ	KIRC	67	MALE		Stage III		Kidney Cl	G2	2007	-24669	Alive	TUMOR F	685				0	685	0	685			0	685
TCGA-CZ	TCGA-CZ	KIRC	67	MALE		Stage III		Kidney Cl	G2	2007	-24669	Alive	TUMOR F	685				0	685	0	685			0	685
TCGA-CZ	TCGA-CZ	KIRC	86	FEMALE	WHITE	Stage III		Kidney Cl	G4	2007	-31473	Dead	WITH TUMOR		73	62		1	73	1	73			1	62
TCGA-CZ	TCGA-CZ	KIRC	86	FEMALE	WHITE	Stage III		Kidney Cl	G4	2007	-31473	Dead	WITH TUMOR		73	62		1	73	1	73			1	62
TCGA-CZ	TCGA-CZ	KIRC	84	MALE	WHITE	Stage IV		Kidney Cl	G4	2007	-30729	Dead	WITH TUI	59	59			1	59	1	59			1	59
TCGA-CZ	TCGA-CZ	KIRC	84	MALE	WHITE	Stage IV		Kidney Cl	G4	2007	-30729	Dead	WITH TUI	59	59			1	59	1	59			1	59
TCGA-CZ	TCGA-CZ	KIRC	41	MALE	WHITE	Stage II		Kidney Cl	G2	2007	-15242	Dead	WITH TUMOR		946	110		1	946	1	946			1	110
TCGA-CZ	TCGA-CZ	KIRC	41	MALE	WHITE	Stage II		Kidney Cl	G2	2007	-15242	Dead	WITH TUMOR		946	110		1	946	1	946			1	110
TCGA-CZ	TCGA-CZ	KIRC	72	FEMALE		Stage II		Kidney Cl	G3	2008	-26494	Alive		386				0	386	0	386			0	386
TCGA-CZ	TCGA-CZ	KIRC	72	FEMALE		Stage II		Kidney Cl	G3	2008	-26494	Alive		386				0	386	0	386			0	386
TCGA-CZ	TCGA-CZ	KIRC	59	FEMALE	WHITE	Stage I		Kidney Cl	G2	2005	-21592	Alive	TUMOR F	2439				0	2439	0	2439			0	2439
TCGA-CZ	TCGA-CZ	KIRC	59	FEMALE	WHITE	Stage I		Kidney Cl	G2	2005	-21592	Alive	TUMOR F	2439				0	2439	0	2439			0	2439
TCGA-CZ	TCGA-CZ	KIRC	51	MALE	WHITE	Stage I		Kidney Cl	G3	2005	-18775	Alive	TUMOR F	2067				0	2067	0	2067			0	2067
TCGA-CZ	TCGA-CZ	KIRC	51	MALE	WHITE	Stage I		Kidney Cl	G3	2005	-18775	Alive	TUMOR F	2067				0	2067	0	2067			0	2067
TCGA-CZ	TCGA-CZ	KIRC	58	MALE	WHITE	Stage II		Kidney Cl	G2	2006	-21242	Alive	TUMOR F	1997				0	1997	0	1997			0	1997
TCGA-CZ	TCGA-CZ	KIRC	58	MALE	WHITE	Stage II		Kidney Cl	G2	2006	-21242	Alive	TUMOR F	1997				0	1997	0	1997			0	1997
TCGA-CZ	TCGA-CZ	KIRC	61	MALE	WHITE	Stage I		Kidney Cl	G3	2006	-22456	Alive	TUMOR F	373				0	373	0	373			0	373
TCGA-CZ	TCGA-CZ	KIRC	61	MALE	WHITE	Stage I		Kidney Cl	G3	2006	-22456	Alive	TUMOR F	373				0	373	0	373			0	373
TCGA-CZ	TCGA-CZ	KIRC	60	MALE	WHITE	Stage IV		Kidney Cl	G2	2006	-21928	Dead	WITH TUMOR		445	377		1	445	1	445			1	377
TCGA-CZ	TCGA-CZ	KIRC	60	MALE	WHITE	Stage IV		Kidney Cl	G2	2006	-21928	Dead	WITH TUMOR		445	377		1	445	1	445			1	377
TCGA-CZ	TCGA-CZ	KIRC	38	MALE	WHITE	Stage I		Kidney Cl	G2	2006	-14131	Alive	TUMOR F	693				0	693	0	693			0	693
TCGA-CZ	TCGA-CZ	KIRC	38	MALE	WHITE	Stage I		Kidney Cl	G2	2006	-14131	Alive	TUMOR F	693				0	693	0	693			0	693
TCGA-CZ	TCGA-CZ	KIRC	60	MALE	WHITE	Stage II		Kidney Cl	G2	2006	-22170	Alive	TUMOR F	1905				0	1905	0	1905			0	1905
TCGA-CZ	TCGA-CZ	KIRC	60	MALE	WHITE	Stage II		Kidney Cl	G2	2006	-22170	Alive	TUMOR F	1905				0	1905	0	1905			0	1905
TCGA-DV	TCGA-DV	KIRC	59	MALE	WHITE	Stage I		Kidney Cl	G2	2007	-21642	Alive	WITH TUI	1329				0	1329	0	1329			0	1329
TCGA-DV	TCGA-DV	KIRC	67	FEMALE	WHITE	Stage I		Kidney Cl	G2	2007	-24717	Alive	WITH TUI	1398				0	1398	0	1398			0	1398
TCGA-DV	TCGA-DV	KIRC	40	FEMALE	BLACK OI	Stage I		Kidney Cl	G2	2008	-14642	Alive	WITH TUI	2004		2004	Complete Remission/Response	0	2004	0	2004	1	2004	1	2004
TCGA-DV	TCGA-DV	KIRC	26	MALE	WHITE	Stage I		Kidney Cl	G2	2009	-9714	Alive	WITH TUI	370				0	370	0	370			0	370
TCGA-DV	TCGA-DV	KIRC	29	FEMALE	WHITE	Stage I		Kidney Cl	G2	2009	-10698	Alive	TUMOR F	355				0	355	0	355			0	355
TCGA-DV	TCGA-DV	KIRC	41	MALE	WHITE	Stage I		Kidney Cl	G2	2008	-15264	Alive	WITH TUI	1130				0	1130	0	1130			0	1130
TCGA-DV	TCGA-DV	KIRC	37	MALE	WHITE	Stage I		Kidney Cl	G2	2008	-13517	Alive	WITH TUI	2016		357	Complete Remission/Response	0	2016	0	2016	1	357	1	357
TCGA-DV	TCGA-DV	KIRC	52	FEMALE	WHITE	Stage I		Kidney Cl	G2	2007	-19022	Alive	WITH TUI	1729				0	1729	0	1729			0	1729
TCGA-DV	TCGA-DV	KIRC	55	FEMALE	BLACK OI	Stage I		Kidney Cl	G2	2008	-20310	Dead	WITH TUMOR		727			1	727	1	727			1	727
TCGA-DV	TCGA-DV	KIRC	59	MALE	BLACK OI	Stage IV		Kidney Cl	G4	2008	-21670	Dead	WITH TUMOR		1626		Progressive Disease	1	1626	1	1626			1	1626
TCGA-DV	TCGA-DV	KIRC	53	MALE	BLACK OI	Stage I		Kidney Cl	G2	2007	-19560	Alive	TUMOR F	365			Complete Remission/Response	0	365	0	365	0	365	0	365
TCGA-DV	TCGA-DV	KIRC	55	MALE	BLACK OI	Stage I		Kidney Cl	G3	2008	-20101	Alive	WITH TUI	2470		2008	Stable Disease	0	2470	0	2470			1	2008
TCGA-DV	TCGA-DV	KIRC	55	MALE	BLACK OI	Stage I		Kidney Cl	G3	2008	-20101	Alive	WITH TUI	2470		2008	Stable Disease	0	2470	0	2470			1	2008
TCGA-EU	TCGA-EU	KIRC	47	FEMALE	WHITE	Stage I		Kidney Cl	G1	2009	-17468	Alive	TUMOR F	551				0	551	0	551			0	551
TCGA-EU	TCGA-EU	KIRC	67	FEMALE	WHITE	Stage I		Kidney Cl	G3	2010	-24475	Alive	TUMOR F	119				0	119	0	119			0	119
TCGA-EU	TCGA-EU	KIRC	55	MALE	WHITE	Stage I		Kidney Cl	G2	2010	-20246	Alive	TUMOR F	206				0	206	0	206			0	206
TCGA-EU	TCGA-EU	KIRC	81	MALE	WHITE	Stage III		Kidney Cl	G3	2010	-29863	Alive	TUMOR F	127				0	127	0	127			0	127
TCGA-G6	TCGA-G6	KIRC	54	FEMALE	BLACK OI	Stage IV		Kidney Cl	G4	2012	-20029	Dead	WITH TUMOR		242		Progressive Disease	1	242	1	242			1	242
TCGA-G6	TCGA-G6	KIRC	55	MALE	BLACK OI	Stage IV		Kidney Cl	G3	2013	-20306	Dead	WITH TUMOR		313	304	Progressive Disease	1	313	1	313			1	304
TCGA-G6	TCGA-G6	KIRC	81	FEMALE	BLACK OI	Stage I		Kidney Cl	G3	2008	-29883	Alive	TUMOR F	2133			Complete Remission/Response	0	2133	0	2133	0	2133	0	2133
TCGA-G6	TCGA-G6	KIRC	62	FEMALE	BLACK OI	Stage I		Kidney Cl	G3	2008	-22649	Dead	TUMOR FREE		1091		Complete Remission/Response	1	1091	0	1091	0	1091	0	1091
TCGA-GK	TCGA-GK	KIRC	76	FEMALE	BLACK OI	Stage I		Kidney Clear Cell Rer		2012	-28107	Alive	TUMOR F	61			Complete Remission/Response	0	61	0	61	0	61	0	61
TCGA-MI	TCGA-MI	KIRC	41	MALE	BLACK OI	[Discrepancy]		Kidney Cl	G2	2012	-15037	Alive	TUMOR F	591			Complete Remission/Response	0	591	0	591	0	591	0	591
TCGA-MI	TCGA-MI	KIRC	68	MALE	BLACK OI	Stage II		Kidney Cl	G2	2012	-24916	Alive	TUMOR F	607			Complete Remission/Response	0	607	0	607	0	607	0	607
TCGA-MI	TCGA-MI	KIRC	58	FEMALE	BLACK OI	Stage I		Kidney Cl	G2	2013	-21375	Alive	TUMOR F	700			Complete Remission/Response	0	700	0	700	0	700	0	700
TCGA-MV	TCGA-MV	KIRC	72	FEMALE	BLACK OI	Stage I		Kidney Cl	G2	2012	-26649	Alive	TUMOR F	498			Complete Remission/Response	0	498	0	498	0	498	0	498
TCGA-T7-	TCGA-T7-	KIRC	47	FEMALE	BLACK OI	Stage I		Kidney Cl	G1	2013	-17331	Alive	TUMOR F	356			Complete Remission/Response	0	356	0	356	0	356	0	356
TCGA-2K	TCGA-2K	KIRP	53	MALE	WHITE	Stage II		Kidney Papillary Ren		2013	-19603	Alive	TUMOR F	214			Complete Remission/Response	0	214	0	214	0	214	0	214
TCGA-2Z	TCGA-2Z	KIRP	71	MALE	WHITE	Stage I	Stage I	Kidney Papillary Ren		2008	-26244	Alive	TUMOR F	2298			Complete Remission/Response	0	2298	0	2298	0	2298	0	2298
TCGA-2Z	TCGA-2Z	KIRP	71	FEMALE	WHITE	Stage I	Stage I	Kidney Papillary Ren		2008	-26134	Alive	TUMOR F	1795			Complete Remission/Response	0	1795	0	1795	0	1795	0	1795
TCGA-2Z	TCGA-2Z	KIRP	67	MALE	WHITE	Stage II	Stage II	Kidney Papillary Ren		2008	-24626	Dead	WITH TUMOR		1771	790	Progressive Disease	1	1771	1	1771			1	790
TCGA-2Z	TCGA-2Z	KIRP	80	MALE	WHITE	Stage II	Stage II	Kidney Papillary Ren		2006	-29275	Alive	TUMOR F	3050			[Unknown]	0	3050	0	3050			0	3050
TCGA-2Z	TCGA-2Z	KIRP	60	MALE	WHITE	Stage I	Stage I	Kidney Papillary Ren		2009	-22265	Alive	TUMOR F	1731			[Unknown]	0	1731	0	1731			0	1731
TCGA-2Z	TCGA-2Z	KIRP	63	MALE	WHITE	Stage IV	Stage IV	Kidney Papillary Ren		2007	-23230	Dead	WITH TUMOR		139	96	Progressive Disease	1	139	1	139			1	96
TCGA-2Z	TCGA-2Z	KIRP	50	MALE	WHITE	Stage I	Stage I	Kidney Papillary Ren		2007	-18498	Alive	WITH TUI	2790		506	Complete Remission/Response	0	2790	0	2790	1	506	1	506
TCGA-2Z	TCGA-2Z	KIRP	77	MALE	BLACK OI	Stage I	Stage I	Kidney Papillary Ren		2006	-28346	Alive	TUMOR F	2294			Complete Remission/Response	0	2294	0	2294	0	2294	0	2294
TCGA-2Z	TCGA-2Z	KIRP	66	MALE	WHITE	Stage I	Stage I	Kidney Papillary Ren		2009	-24370	Alive	TUMOR F	1060			[Unknown]	0	1060	0	1060			0	1060
TCGA-2Z	TCGA-2Z	KIRP	59	MALE	WHITE	Stage I	Stage I	Kidney Papillary Ren		2010	-21557	Alive	TUMOR F	1728			Complete Remission/Response	0	1728	0	1728	0	1728	0	1728
TCGA-2Z	TCGA-2Z	KIRP	64	MALE	WHITE	Stage I	Stage I	Kidney Papillary Ren		2010	-23458	Alive	TUMOR F	1662			Complete Remission/Response	0	1662	0	1662	0	1662	0	1662
TCGA-2Z	TCGA-2Z	KIRP	38	MALE	WHITE	Stage III	Stage I	Kidney Papillary Ren		2010	-14196	Alive	TUMOR F	1567		286	Complete Remission/Response	0	1567	0	1567	1	286	1	286
TCGA-2Z	TCGA-2Z	KIRP	69	FEMALE	WHITE	Stage I	Stage I	Kidney Papillary Ren		2011	-25240	Alive	TUMOR F	1213			Complete Remission/Response	0	1213	0	1213	0	1213	0	1213
TCGA-2Z	TCGA-2Z	KIRP	50	MALE	WHITE	Stage III	Stage III	Kidney Papillary Ren		2011	-18540	Alive	WITH TUI	1186		324	Stable Disease	0	1186	0	1186			1	324
TCGA-2Z	TCGA-2Z	KIRP	47	MALE	BLACK OI	Stage I	Stage I	Kidney Papillary Ren		2012	-17367	Alive	TUMOR F	1006			Complete Remission/Response	0	1006	0	1006	0	1006	0	1006
TCGA-2Z	TCGA-2Z	KIRP	28	MALE	WHITE	Stage I	Stage I	Kidney Papillary Ren		2012	-10323	Alive	TUMOR F	934			[Unknown]	0	934	0	934			0	934
TCGA-2Z	TCGA-2Z	KIRP	50	FEMALE	BLACK OI	Stage I	Stage I	Kidney Papillary Ren		2012	-18264	Alive	TUMOR F	740			Complete Remission/Response	0	740	0	740	0	740	0	740
TCGA-2Z	TCGA-2Z	KIRP	56	FEMALE	BLACK OI	Stage I	Stage I	Kidney Papillary Ren		2012	-20466	Alive	TUMOR F	949			Complete Remission/Response	0	949	0	949	0	949	0	949
TCGA-2Z	TCGA-2Z	KIRP	63	MALE	WHITE	Stage I	Stage I	Kidney Papillary Ren		2011	-23207	Alive	TUMOR F	893			Complete Remission/Response	0	893	0	893	0	893	0	893
TCGA-2Z	TCGA-2Z	KIRP	66	MALE	WHITE	Stage I	Stage I	Kidney Papillary Ren		2012	-24244	Alive	WITH TUI	778		600	[Unknown]	0	778	0	778			1	600
TCGA-2Z	TCGA-2Z	KIRP	57	MALE	WHITE	Stage I	Stage I	Kidney Papillary Ren		2012	-20932	Alive	TUMOR F	161			[Unknown]	0	161	0	161			0	161
TCGA-2Z	TCGA-2Z	KIRP	74	MALE	WHITE	Stage I	Stage I	Kidney Papillary Ren		2012	-27053	Dead	TUMOR FREE		492		Complete Remission/Response	1	492	0	492	0	492	0	492
TCGA-2Z	TCGA-2Z	KIRP	71	MALE	WHITE	Stage I	Stage I	Kidney Papillary Ren		2012	-26128	Alive	TUMOR F	684			Complete Remission/Response	0	684	0	684	0	684	0	684
TCGA-4A	TCGA-4A	KIRP	66	FEMALE	WHITE	Stage I	Stage I	Kidney Papillary Ren		2013	-24117	Alive	TUMOR F	4			Complete Remission/Response	0	4	0	4	0	4	0	4
TCGA-4A	TCGA-4A	KIRP	58	MALE	WHITE	Stage IV	Stage IV	Kidney Papillary Ren		2013	-21348	Alive	WITH TUI	390			Stable Disease	0	390	0	390			0	390
TCGA-4A	TCGA-4A	KIRP	63	FEMALE	BLACK OI	Stage II	Stage II	Kidney Papillary Ren		2013	-23226	Alive	TUMOR F	352			Complete Remission/Response	0	352	0	352	0	352	0	352
TCGA-5P	TCGA-5P	KIRP	81	MALE	WHITE	Stage III		Kidney Papillary Ren		2012	-29594	Alive	TUMOR F	373			Complete Remission/Response	0	373	0	373	0	373	0	373
TCGA-5P	TCGA-5P	KIRP	49	MALE	WHITE			Kidney Papillary Ren		2005	-17973	Alive		2043			Complete Remission/Response	0	2043	0	2043	0	2043	0	2043
TCGA-5P	TCGA-5P	KIRP	64	MALE	WHITE			Kidney Papillary Ren		2006	-23570	Alive		3241		3228	Complete Remission/Response	0	3241	0	3241	1	3228	1	3228
TCGA-5P	TCGA-5P	KIRP	79	MALE	WHITE			Kidney Papillary Ren		2006	-28881	Dead	TUMOR FREE		1508		Complete Remission/Response	1	1508	0	1508	0	1508	0	1508
TCGA-5P	TCGA-5P	KIRP	51	FEMALE	WHITE			Kidney Papillary Ren		2006	-18885	Alive	TUMOR F	2744			Complete Remission/Response	0	2744	0	2744	0	2744	0	2744
TCGA-5P	TCGA-5P	KIRP	75	MALE	WHITE			Kidney Papillary Ren		2007	-27434	Alive		677				0	677	0	677			0	677
TCGA-5P	TCGA-5P	KIRP	62	MALE	WHITE			Kidney Papillary Ren		2007	-22964	Alive	TUMOR F	2392			Complete Remission/Response	0	2392	0	2392	0	2392	0	2392
TCGA-5P	TCGA-5P	KIRP	59	MALE	WHITE			Kidney Papillary Ren		2007	-21829	Dead	WITH TUMOR		471	17	Partial Remission/Response	1	471	1	471			1	17
TCGA-5P	TCGA-5P	KIRP	54	MALE	WHITE			Kidney Papillary Ren		2008	-19986	Alive	TUMOR F	2184			Complete Remission/Response	0	2184	0	2184	0	2184	0	2184
TCGA-5P	TCGA-5P	KIRP	64	MALE	WHITE			Kidney Papillary Ren		2009	-23384	Alive		1914			Complete Remission/Response	0	1914	0	1914	0	1914	0	1914
TCGA-5P	TCGA-5P	KIRP	51	FEMALE	WHITE			Kidney Papillary Ren		2010	-18880	Alive	TUMOR F	1309			Complete Remission/Response	0	1309	0	1309	0	1309	0	1309
TCGA-5P	TCGA-5P	KIRP	59	FEMALE	WHITE			Kidney Papillary Ren		2011	-21600	Dead	WITH TUMOR		589	548	Complete Remission/Response	1	589	1	589	1	548	1	548
TCGA-5P	TCGA-5P	KIRP	61	MALE	WHITE			Kidney Papillary Ren		2013	-22545	Alive	TUMOR F	395			Complete Remission/Response	0	395	0	395	0	395	0	395
TCGA-5P	TCGA-5P	KIRP	51	FEMALE	WHITE			Kidney Papillary Ren		2013	-18975	Alive	TUMOR F	377			Complete Remission/Response	0	377	0	377	0	377	0	377
TCGA-5P	TCGA-5P	KIRP	70	MALE	WHITE		Stage I	Kidney Papillary Ren		2011	-25805	Alive	TUMOR F	824			Complete Remission/Response	0	824	0	824	0	824	0	824
TCGA-5P	TCGA-5P	KIRP	53	MALE	WHITE		Stage I	Kidney Papillary Ren		2011	-19631	Alive	TUMOR F	845		728	[Unknown]	0	845	0	845			1	728
TCGA-5P	TCGA-5P	KIRP	77	MALE	WHITE			Kidney Papillary Ren		2005	-28193	Alive	TUMOR F	2051			Complete Remission/Response	0	2051	0	2051	0	2051	0	2051
TCGA-A4	TCGA-A4	KIRP	42	MALE	WHITE		Stage I	Kidney Papillary Ren		2011	-15631	Alive	TUMOR F	554			Complete Remission/Response	0	554	0	554	0	554	0	554
TCGA-A4	TCGA-A4	KIRP	75	FEMALE	WHITE	Stage I	Stage I	Kidney Papillary Ren		2011	-27621	Dead	WITH TUMOR		597	234	Complete Remission/Response	1	597	1	597	1	234	1	234
TCGA-A4	TCGA-A4	KIRP	71	FEMALE	WHITE		Stage I	Kidney Papillary Ren		2008	-26268	Alive	TUMOR F	1857			Complete Remission/Response	0	1857	0	1857	0	1857	0	1857
TCGA-A4	TCGA-A4	KIRP	71	FEMALE	WHITE		Stage I	Kidney Papillary Ren		2008	-26268	Alive	TUMOR F	1857			Complete Remission/Response	0	1857	0	1857	0	1857	0	1857
TCGA-A4	TCGA-A4	KIRP	60	MALE	BLACK OI	Stage I	Stage I	Kidney Papillary Ren		2011	-21962	Alive	TUMOR F	3				0	3	0	3			0	3
TCGA-A4	TCGA-A4	KIRP	59	MALE	BLACK OI	Stage I	Stage I	Kidney Papillary Ren		2011	-21782	Alive	TUMOR F	650			Complete Remission/Response	0	650	0	650	0	650	0	650
TCGA-A4	TCGA-A4	KIRP	67	MALE	WHITE	Stage III	Stage I	Kidney Papillary Ren		2007	-24778	Dead	TUMOR FREE		1070		Complete Remission/Response	1	1070	0	1070	0	1070	0	1070
TCGA-A4	TCGA-A4	KIRP	67	MALE	WHITE	Stage III	Stage I	Kidney Papillary Ren		2007	-24778	Dead	TUMOR FREE		1070		Complete Remission/Response	1	1070	0	1070	0	1070	0	1070
TCGA-A4	TCGA-A4	KIRP	55	MALE	BLACK OR AFRICAN		Stage II	Kidney Papillary Ren		2011	-20226	Alive	TUMOR F	582			Complete Remission/Response	0	582	0	582	0	582	0	582
TCGA-A4	TCGA-A4	KIRP	55	MALE	BLACK OR AFRICAN		Stage II	Kidney Papillary Ren		2011	-20226	Alive	TUMOR F	582			Complete Remission/Response	0	582	0	582	0	582	0	582
TCGA-A4	TCGA-A4	KIRP	53	FEMALE	WHITE	Stage I	Stage I	Kidney Papillary Ren		2011	-19612	Alive	TUMOR F	27				0	27	0	27			0	27
TCGA-A4	TCGA-A4	KIRP	74	FEMALE	BLACK OI	Stage I	Stage I	Kidney Papillary Ren		2011	-27122	Alive	TUMOR F	238			Complete Remission/Response	0	238	0	238	0	238	0	238
TCGA-A4	TCGA-A4	KIRP	35	FEMALE	BLACK OI	Stage I	Stage II	Kidney Papillary Ren		2011	-12832	Alive	TUMOR F	7				0	7	0	7			0	7
TCGA-A4	TCGA-A4	KIRP	61	FEMALE	WHITE	Stage I	Stage I	Kidney Papillary Ren		2011	-22559	Alive	TUMOR F	506		493	Complete Remission/Response	0	506	0	506	1	493	1	493
TCGA-A4	TCGA-A4	KIRP	67	MALE	WHITE	Stage I	Stage I	Kidney Papillary Ren		2011	-24486	Alive	TUMOR F	927			[Unknown]	0	927	0	927			0	927

TCGA-A4	TCGA-A4	KIRP	73	MALE	BLACK O	Stage I	Stage I	Kidney Papillary Ren	2011	-26685	Alive	TUMOR F	896			Complete Remission/Response	0	896	0	896	0	896	0	896
TCGA-A4	TCGA-A4	KIRP	58	MALE	WHITE	Stage III	Stage I	Kidney Papillary Ren	2012	-21312	Alive	TUMOR F	780			Complete Remission/Response	0	780	0	780	0	780	0	780
TCGA-A4	TCGA-A4	KIRP	60	MALE	WHITE	Stage I	Stage I	Kidney Papillary Ren	2012	-22220	Alive	TUMOR F	729			Complete Remission/Response	0	729	0	729	0	729	0	729
TCGA-A4	TCGA-A4	KIRP	60	MALE	BLACK O	Stage I	Stage I	Kidney Papillary Ren	2012	-22231	Alive	TUMOR F	708			Complete Remission/Response	0	708	0	708	0	708	0	708
TCGA-A4	TCGA-A4	KIRP	47	MALE	AMERICA	Stage I	Stage I	Kidney Papillary Ren	2012	-17345	Alive	TUMOR F	709			Complete Remission/Response	0	709	0	709	0	709	0	709
TCGA-A4	TCGA-A4	KIRP	62	MALE	WHITE	Stage III	Stage I	Kidney Papillary Ren	2012	-22879	Alive		23				0	23	0	23			0	23
TCGA-A4	TCGA-A4	KIRP	71	MALE	WHITE	Stage I	Stage I	Kidney Papillary Ren	2012	-26090	Alive	TUMOR F	597			Complete Remission/Response	0	597	0	597	0	597	0	597
TCGA-A4	TCGA-A4	KIRP	48	MALE	WHITE	Stage I	Stage I	Kidney Papillary Ren	2012	-17713	Alive	TUMOR F	600			Complete Remission/Response	0	600	0	600	0	600	0	600
TCGA-A4	TCGA-A4	KIRP	59	FEMALE	WHITE	Stage I	Stage I	Kidney Papillary Ren	2012	-21884	Alive	TUMOR F	436			Complete Remission/Response	0	436	0	436	0	436	0	436
TCGA-A4	TCGA-A4	KIRP	84	MALE	BLACK O	Stage I	Stage I	Kidney Papillary Ren	2012	-31038	Alive	TUMOR F	379			Complete Remission/Response	0	379	0	379	0	379	0	379
TCGA-A4	TCGA-A4	KIRP	71	FEMALE	WHITE	Stage I	Stage II	Kidney Papillary Ren	2012	-26058	Alive	TUMOR F	516			Complete Remission/Response	0	516	0	516	0	516	0	516
TCGA-A4	TCGA-A4	KIRP	71	FEMALE	WHITE	Stage I	Stage II	Kidney Papillary Ren	2012	-26058	Alive	TUMOR F	516			Complete Remission/Response	0	516	0	516	0	516	0	516
TCGA-A4	TCGA-A4	KIRP	59	MALE	WHITE	Stage IV	Stage IV	Kidney Papillary Ren	2011	-21885	Dead	WITH TUMOR		258		Progressive Disease	1	258	1	258			1	258
TCGA-A4	TCGA-A4	KIRP	59	MALE	WHITE	Stage IV	Stage IV	Kidney Papillary Ren	2011	-21885	Dead	WITH TUMOR		258		Progressive Disease	1	258	1	258			1	258
TCGA-A4	TCGA-A4	KIRP	45	FEMALE	WHITE	Stage I	Stage I	Kidney Papillary Ren	2012	-16779	Alive	TUMOR F	496			Complete Remission/Response	0	496	0	496	0	496	0	496
TCGA-A4	TCGA-A4	KIRP	65	FEMALE	WHITE	Stage II	Stage II	Kidney Papillary Ren	2012	-24006	Alive	TUMOR F	479			Complete Remission/Response	0	479	0	479	0	479	0	479
TCGA-A4	TCGA-A4	KIRP	61	FEMALE	WHITE	Stage I	Stage I	Kidney Papillary Ren	2012	-22341	Alive	TUMOR F	252			Complete Remission/Response	0	252	0	252	0	252	0	252
TCGA-A4	TCGA-A4	KIRP	75	MALE	WHITE	Stage III	Stage III	Kidney Papillary Ren	2012	-27541	Dead	WITH TUMOR		395	296	Complete Remission/Response	1	395	1	395	1	296	1	296
TCGA-A4	TCGA-A4	KIRP	75	MALE	WHITE	Stage III	Stage III	Kidney Papillary Ren	2012	-27541	Dead	WITH TUMOR		395	296	Complete Remission/Response	1	395	1	395	1	296	1	296
TCGA-A4	TCGA-A4	KIRP	66	MALE	WHITE	Stage I	Stage I	Kidney Papillary Ren	2013	-24447	Alive	TUMOR F	379			Complete Remission/Response	0	379	0	379	0	379	0	379
TCGA-A4	TCGA-A4	KIRP	39	MALE	WHITE	Stage I		Kidney Papillary Ren	2013	-14278	Alive	TUMOR F	0				0	0	0	0			0	0
TCGA-A4	TCGA-A4	KIRP	71	MALE	WHITE	Stage III		Kidney Papillary Ren	2013	-26264	Alive		0				0	0	0	0			0	0
TCGA-AL	TCGA-AL	KIRP	41	MALE	WHITE	Stage IV	Stage IV	Kidney Papillary Ren	2004	-15181	Dead	WITH TUMOR		293	138		1	293	1	293			1	138
TCGA-AL	TCGA-AL	KIRP	54	FEMALE	WHITE		Stage I	Kidney Papillary Ren	2005	-19783	Alive	TUMOR F	2135			Complete Remission/Response	0	2135	0	2135	0	2135	0	2135
TCGA-AL	TCGA-AL	KIRP	74	MALE	WHITE	Stage II	Stage I	Kidney Papillary Ren	2005	-27205	Dead	TUMOR FREE		2331		Complete Remission/Response	1	2331	0	2331	0	2331	0	2331
TCGA-AL	TCGA-AL	KIRP	74	MALE	WHITE	Stage I	Stage I	Kidney Papillary Ren	2006	-27249	Dead	TUMOR FREE		1500		[Unknown]	1	1500	0	1500			0	1500
TCGA-AL	TCGA-AL	KIRP	57	MALE	BLACK OR AFRICAN		Stage II	Kidney Papillary Ren	2008	-21166	Alive	TUMOR F	2029			Complete Remission/Response	0	2029	0	2029	0	2029	0	2029
TCGA-AL	TCGA-AL	KIRP	55	MALE	WHITE	Stage II	Stage II	Kidney Papillary Ren	2008	-20104	Alive	TUMOR F	1906			Complete Remission/Response	0	1906	0	1906	0	1906	0	1906
TCGA-AL	TCGA-AL	KIRP	72	FEMALE	WHITE	Stage IV	Stage IV	Kidney Papillary Ren	2008	-26326	Alive	WITH TU	2060		332	Progressive Disease	0	2060	0	2060			1	332
TCGA-AL	TCGA-AL	KIRP	54	FEMALE	WHITE	Stage III	Stage I	Kidney Papillary Ren	2009	-20079	Dead	WITH TUMOR		1498		Progressive Disease	1	1498	1	1498			1	1498
TCGA-AT	TCGA-AT	KIRP	77	MALE	WHITE	Stage I		Kidney Papillary Ren	2011	-28137	Alive		67			[Unknown]	0	67	0	67			0	67
TCGA-B1	TCGA-B1	KIRP	73	MALE	WHITE	Stage III	Stage II	Kidney Papillary Ren	2010	-26820	Alive	TUMOR F	1251			Complete Remission/Response	0	1251	0	1251	0	1251	0	1251
TCGA-B1	TCGA-B1	KIRP	60	FEMALE	BLACK O	Stage II		Kidney Papillary Ren	2011	-22277	Alive	TUMOR F	1141			Complete Remission/Response	0	1141	0	1141	0	1141	0	1141
TCGA-B1	TCGA-B1	KIRP	79	MALE	BLACK O	Stage III		Kidney Papillary Ren	2011	-29068	Alive	WITH TU	659		408	[Unknown]	0	659	0	659			1	408
TCGA-B1	TCGA-B1	KIRP	79	MALE	BLACK O	Stage III		Kidney Papillary Ren	2011	-29068	Alive	WITH TU	659		408	[Unknown]	0	659	0	659			1	408
TCGA-B1	TCGA-B1	KIRP	62	MALE	WHITE	Stage I	Stage I	Kidney Papillary Ren	2012	-22800	Alive	TUMOR F	1110			Complete Remission/Response	0	1110	0	1110	0	1110	0	1110
TCGA-B1	TCGA-B1	KIRP	54	FEMALE	WHITE	Stage I	Stage I	Kidney Papillary Ren	2012	-19823	Alive	TUMOR F	842			Complete Remission/Response	0	842	0	842	0	842	0	842
TCGA-B1	TCGA-B1	KIRP	43	FEMALE	BLACK O	Stage I		Kidney Papillary Ren	2012	-15721	Alive	TUMOR F	616			Complete Remission/Response	0	616	0	616	0	616	0	616
TCGA-B1	TCGA-B1	KIRP	77	FEMALE	WHITE	Stage I		Kidney Papillary Ren	2012	-28433	Alive	TUMOR F	510			Complete Remission/Response	0	510	0	510	0	510	0	510
TCGA-B1	TCGA-B1	KIRP	64	MALE	WHITE	Stage I		Kidney Papillary Ren	2012	-23702	Alive	TUMOR F	618			Complete Remission/Response	0	618	0	618	0	618	0	618
TCGA-B1	TCGA-B1	KIRP	54	MALE	WHITE	Stage I		Kidney Papillary Ren	2012	-19806	Alive	TUMOR F	641			Complete Remission/Response	0	641	0	641	0	641	0	641
TCGA-B3	TCGA-B3	KIRP	52	MALE	WHITE	Stage III		Kidney Papillary Ren	2010	-19224	Alive	TUMOR F	1029			Complete Remission/Response	0	1029	0	1029	0	1029	0	1029
TCGA-B3	TCGA-B3	KIRP	52	MALE	WHITE	Stage III		Kidney Papillary Ren	2010	-19224	Alive	TUMOR F	1029			Complete Remission/Response	0	1029	0	1029	0	1029	0	1029
TCGA-B3	TCGA-B3	KIRP	59	FEMALE	WHITE	Stage I		Kidney Papillary Ren	2010	-21876	Alive	TUMOR F	883			Complete Remission/Response	0	883	0	883	0	883	0	883
TCGA-B3	TCGA-B3	KIRP	59	FEMALE	WHITE	Stage I		Kidney Papillary Ren	2010	-21876	Alive	TUMOR F	883			Complete Remission/Response	0	883	0	883	0	883	0	883
TCGA-B3	TCGA-B3	KIRP	56	MALE	WHITE	Stage I		Kidney Papillary Ren	2010	-20708	Alive	TUMOR F	930			Complete Remission/Response	0	930	0	930	0	930	0	930
TCGA-B3	TCGA-B3	KIRP	56	MALE	WHITE	Stage I		Kidney Papillary Ren	2010	-20708	Alive	TUMOR F	930			Complete Remission/Response	0	930	0	930	0	930	0	930
TCGA-B3	TCGA-B3	KIRP	75	MALE	WHITE	Stage II		Kidney Papillary Ren	2010	-27709	Alive	TUMOR F	1049				0	1049	0	1049			0	1049
TCGA-B3	TCGA-B3	KIRP	75	MALE	WHITE	Stage II		Kidney Papillary Ren	2010	-27709	Alive	TUMOR F	1049				0	1049	0	1049			0	1049
TCGA-B3	TCGA-B3	KIRP	48	FEMALE	BLACK O	Stage I		Kidney Papillary Ren	2011	-17760	Alive	TUMOR F	379			Complete Remission/Response	0	379	0	379	0	379	0	379
TCGA-B3	TCGA-B3	KIRP	43	MALE	BLACK O	Stage I		Kidney Papillary Ren	2013	-15945	Alive	TUMOR F	309			Complete Remission/Response	0	309	0	309	0	309	0	309
TCGA-B9	TCGA-B9	KIRP	64	MALE	BLACK O	Stage I	Stage I	Kidney Papillary Ren	2002	-23613	Dead	TUMOR FREE		1536			1	1536	0	1536			0	1536
TCGA-B9	TCGA-B9	KIRP	64	MALE	BLACK O	Stage I	Stage I	Kidney Papillary Ren	2002	-23613	Dead	TUMOR FREE		1536			1	1536	0	1536			0	1536
TCGA-B9	TCGA-B9	KIRP	49	MALE	BLACK OR AFRICAN		Stage IV	Kidney Papillary Ren	2009	-17989	Alive	WITH TU	857		637		0	857	0	857			1	637
TCGA-B9	TCGA-B9	KIRP	53	MALE	BLACK O	Stage I	Stage I	Kidney Papillary Ren	2009	-19571	Alive	TUMOR F	803				0	803	0	803			0	803
TCGA-B9	TCGA-B9	KIRP	53	MALE	BLACK O	Stage I	Stage I	Kidney Papillary Ren	2009	-19571	Alive	TUMOR F	803				0	803	0	803			0	803
TCGA-B9	TCGA-B9	KIRP	75	FEMALE	BLACK OR AFRICAN		Stage II	Kidney Papillary Ren	2010	-27692	Alive	TUMOR F	627				0	627	0	627			0	627
TCGA-B9	TCGA-B9	KIRP	48	FEMALE	BLACK OR AFRICAN		Stage II	Kidney Papillary Ren	2009	-17622	Alive	TUMOR F	14				0	14	0	14			0	14
TCGA-B9	TCGA-B9	KIRP	41	MALE	BLACK OR AFRICAN		Stage I	Kidney Papillary Ren	2010	-15053	Alive	TUMOR F	35				0	35	0	35			0	35
TCGA-B9	TCGA-B9	KIRP	71	MALE	WHITE	Stage III	Stage III	Kidney Papillary Ren	2010	-26149	Alive	TUMOR F	665			Complete Remission/Response	0	665	0	665	0	665	0	665
TCGA-B9	TCGA-B9	KIRP	51	MALE	WHITE		Stage I	Kidney Papillary Ren	2010	-18841	Alive	TUMOR F	1338			Complete Remission/Response	0	1338	0	1338	0	1338	0	1338
TCGA-B9	TCGA-B9	KIRP	59	MALE	WHITE	Stage II	Stage I	Kidney Papillary Ren	2011	-21705	Alive	TUMOR F	671			Complete Remission/Response	0	671	0	671	0	671	0	671
TCGA-B9	TCGA-B9	KIRP	70	MALE	BLACK O	Stage III	Stage III	Kidney Papillary Ren	2012	-25763	Alive	TUMOR F	361			Partial Remission/Response	0	361	0	361			0	361
TCGA-B9	TCGA-B9	KIRP	47	MALE	BLACK O	Stage I	Stage I	Kidney Papillary Ren	2012	-17465	Alive	TUMOR F	585		564	Complete Remission/Response	0	585	0	585	1	564	1	564
TCGA-B9	TCGA-B9	KIRP	50	MALE	BLACK O	Stage II	Stage II	Kidney Papillary Ren	2012	-18440	Alive	TUMOR F	524			Complete Remission/Response	0	524	0	524	0	524	0	524
TCGA-B9	TCGA-B9	KIRP	40	MALE	WHITE	Stage I	Stage I	Kidney Papillary Ren	2012	-14668	Alive	TUMOR F	5			Complete Remission/Response	0	5	0	5	0	5	0	5
TCGA-B9	TCGA-B9	KIRP	71	MALE	WHITE	Stage III	Stage II	Kidney Papillary Ren	2013	-26198	Alive	TUMOR F	452			Complete Remission/Response	0	452	0	452	0	452	0	452
TCGA-B9	TCGA-B9	KIRP	52	MALE	BLACK O	Stage I	Stage I	Kidney Papillary Ren	2013	-19051	Alive	TUMOR F	532			Complete Remission/Response	0	532	0	532	0	532	0	532
TCGA-B9	TCGA-B9	KIRP	79	MALE	BLACK O	Stage I	Stage I	Kidney Papillary Ren	2013	-28917	Alive	TUMOR F	374			Complete Remission/Response	0	374	0	374	0	374	0	374
TCGA-BQ	TCGA-BQ	KIRP	66	FEMALE	WHITE	Stage III	Stage II	Kidney Papillary Ren	2004	-24153	Alive	TUMOR F	2639				0	2639	0	2639			0	2639
TCGA-BQ	TCGA-BQ	KIRP	66	FEMALE	WHITE	Stage III	Stage II	Kidney Papillary Ren	2004	-24153	Alive	TUMOR F	2639				0	2639	0	2639			0	2639
TCGA-BQ	TCGA-BQ	KIRP	75	MALE	WHITE	Stage I	Stage I	Kidney Papillary Ren	2004	-27409	Alive	TUMOR F	586				0	586	0	586			0	586
TCGA-BQ	TCGA-BQ	KIRP	75	MALE	WHITE	Stage I	Stage I	Kidney Papillary Ren	2004	-27409	Alive	TUMOR F	586				0	586	0	586			0	586
TCGA-BQ	TCGA-BQ	KIRP	60	MALE	BLACK O	Stage IV	Stage IV	Kidney Papillary Ren	2004	-22095	Dead	WITH TUMOR		270	127		1	270	1	270			1	127
TCGA-BQ	TCGA-BQ	KIRP	60	MALE	BLACK O	Stage IV	Stage IV	Kidney Papillary Ren	2004	-22095	Dead	WITH TUMOR		270	127		1	270	1	270			1	127
TCGA-BQ	TCGA-BQ	KIRP	79	FEMALE	WHITE	Stage III	Stage III	Kidney Papillary Ren	2005	-29010	Alive	WITH TU	1938		1022		0	1938	0	1938			1	1022
TCGA-BQ	TCGA-BQ	KIRP	79	FEMALE	WHITE	Stage III	Stage III	Kidney Papillary Ren	2005	-29010	Alive	WITH TU	1938		1022		0	1938	0	1938			1	1022
TCGA-BQ	TCGA-BQ	KIRP	32	FEMALE	WHITE	Stage III	Stage III	Kidney Papillary Ren	2005	-11862	Dead	WITH TUMOR		697	130		1	697	1	697			1	130
TCGA-BQ	TCGA-BQ	KIRP	32	FEMALE	WHITE	Stage III	Stage III	Kidney Papillary Ren	2005	-11862	Dead	WITH TUMOR		697	130		1	697	1	697			1	130
TCGA-BQ	TCGA-BQ	KIRP	79	MALE	WHITE	Stage III	Stage III	Kidney Papillary Ren	2006	-29053	Dead	WITH TUMOR		1314	972		1	1314	1	1314			1	972
TCGA-BQ	TCGA-BQ	KIRP	79	MALE	WHITE	Stage III	Stage III	Kidney Papillary Ren	2006	-29053	Dead	WITH TUMOR		1314	972		1	1314	1	1314			1	972
TCGA-BQ	TCGA-BQ	KIRP	69	MALE	WHITE	Stage I	Stage I	Kidney Papillary Ren	2006	-25481	Alive	TUMOR F	1454				0	1454	0	1454			0	1454
TCGA-BQ	TCGA-BQ	KIRP	69	MALE	WHITE	Stage I	Stage I	Kidney Papillary Ren	2006	-25481	Alive	TUMOR F	1454				0	1454	0	1454			0	1454
TCGA-BQ	TCGA-BQ	KIRP	54	MALE		Stage III	Stage III	Kidney Papillary Ren	2007	-20042	Dead	WITH TUMOR		658	340		1	658	1	658			1	340
TCGA-BQ	TCGA-BQ	KIRP	54	MALE		Stage III	Stage III	Kidney Papillary Ren	2007	-20042	Dead	WITH TUMOR		658	340		1	658	1	658			1	340
TCGA-BQ	TCGA-BQ	KIRP	79	FEMALE	BLACK O	Stage I	Stage I	Kidney Papillary Ren	2007	-28877	Alive	TUMOR F	1242				0	1242	0	1242			0	1242
TCGA-BQ	TCGA-BQ	KIRP	79	FEMALE	BLACK O	Stage I	Stage I	Kidney Papillary Ren	2007	-28877	Alive	TUMOR F	1242				0	1242	0	1242			0	1242
TCGA-BQ	TCGA-BQ	KIRP	59	FEMALE	BLACK O	Stage I	Stage I	Kidney Papillary Ren	2008	-21730	Alive	TUMOR F	792				0	792	0	792			0	792
TCGA-BQ	TCGA-BQ	KIRP	59	FEMALE	BLACK O	Stage I	Stage I	Kidney Papillary Ren	2008	-21730	Alive	TUMOR F	792				0	792	0	792			0	792
TCGA-BQ	TCGA-BQ	KIRP	63	MALE	WHITE	Stage III	Stage II	Kidney Papillary Ren	2008	-23173	Alive	TUMOR F	921				0	921	0	921			0	921
TCGA-BQ	TCGA-BQ	KIRP	63	MALE	WHITE	Stage III	Stage II	Kidney Papillary Ren	2008	-23173	Alive	TUMOR F	921				0	921	0	921			0	921
TCGA-BQ	TCGA-BQ	KIRP	75	MALE	WHITE	Stage III	[Discrepar	Kidney Papillary Ren	2009	-27670	Alive	TUMOR F	792				0	792	0	792			0	792
TCGA-BQ	TCGA-BQ	KIRP	75	MALE	WHITE	Stage III	[Discrepar	Kidney Papillary Ren	2009	-27670	Alive	TUMOR F	792				0	792	0	792			0	792
TCGA-BQ	TCGA-BQ	KIRP	54	MALE	WHITE	Stage III	Stage III	Kidney Papillary Ren	2009	-19747	Alive	TUMOR F	771				0	771	0	771			0	771
TCGA-BQ	TCGA-BQ	KIRP	54	MALE	WHITE	Stage III	Stage III	Kidney Papillary Ren	2009	-19747	Alive	TUMOR F	771				0	771	0	771			0	771
TCGA-BQ	TCGA-BQ	KIRP	74	FEMALE	WHITE	Stage I	Stage I	Kidney Papillary Ren	2009	-27346	Alive	TUMOR F	403				0	403	0	403			0	403
TCGA-BQ	TCGA-BQ	KIRP	74	FEMALE	WHITE	Stage I	Stage I	Kidney Papillary Ren	2009	-27346	Alive	TUMOR F	403				0	403	0	403			0	403
TCGA-BQ	TCGA-BQ	KIRP	63	MALE	WHITE	Stage IV	Stage III	Kidney Papillary Ren	2009	-23267	Dead	WITH TUMOR		329	-35		1	329	1	329			1	
TCGA-BQ	TCGA-BQ	KIRP	63	MALE	WHITE	Stage IV	Stage III	Kidney Papillary Ren	2009	-23267	Dead	WITH TUMOR		329	-35		1	329	1	329			1	
TCGA-BQ	TCGA-BQ	KIRP	69	MALE	WHITE	Stage III	Stage III	Kidney Papillary Ren	2009	-25272	Alive	TUMOR F	233				0	233	0	233			0	233
TCGA-BQ	TCGA-BQ	KIRP	69	MALE	WHITE	Stage III	Stage III	Kidney Papillary Ren	2009	-25272	Alive	TUMOR F	233				0	233	0	233			0	233
TCGA-BQ	TCGA-BQ	KIRP	57	FEMALE	WHITE	Stage III	Stage III	Kidney Papillary Ren	2009	-20945	Alive	TUMOR F	29				0	29	0	29			0	29
TCGA-BQ	TCGA-BQ	KIRP	57	FEMALE	WHITE	Stage III	Stage III	Kidney Papillary Ren	2009	-20945	Alive	TUMOR F	29				0	29	0	29			0	29
TCGA-BQ	TCGA-BQ	KIRP	51	MALE	WHITE	Stage I	Stage I	Kidney Papillary Ren	2010	-18785	Alive	TUMOR F	364				0	364	0	364			0	364
TCGA-BQ	TCGA-BQ	KIRP	51	MALE	WHITE	Stage I	Stage I	Kidney Papillary Ren	2010	-18785	Alive	TUMOR F	364				0	364	0	364			0	364
TCGA-BQ	TCGA-BQ	KIRP	61	MALE	WHITE	Stage IV	Stage IV	Kidney Papillary Ren	2010	-22320	Dead	WITH TUMOR		241	188		1	241	1	241			1	188
TCGA-BQ	TCGA-BQ	KIRP	61	MALE	WHITE	Stage IV	Stage IV	Kidney Papillary Ren	2010	-22320	Dead	WITH TUMOR		241	188		1	241	1	241			1	188
TCGA-BQ	TCGA-BQ	KIRP	42	MALE		Stage IV	Stage III	Kidney Papillary Ren	2010	-15510	Alive	WITH TU	84				0	84	0	84			0	84
TCGA-BQ	TCGA-BQ	KIRP	42	MALE		Stage IV	Stage III	Kidney Papillary Ren	2010	-15510	Alive	WITH TU	84				0	84	0	84			0	84
TCGA-BQ	TCGA-BQ	KIRP	73	MALE	WHITE	Stage III	Stage III	Kidney Papillary Ren	2000	-26831	Alive	TUMOR F	3760				0	3760	0	3760			0	3760
TCGA-BQ	TCGA-BQ	KIRP	73	MALE	WHITE	Stage III	Stage III	Kidney Papillary Ren	2000	-26831	Alive	TUMOR F	3760				0	3760	0	3760			0	3760
TCGA-BQ	TCGA-BQ	KIRP	78	MALE	BLACK O	Stage I	Stage I	Kidney Papillary Ren	2001	-28709	Dead	TUMOR FREE		1468			1	1468	0	1468			0	1468
TCGA-BQ	TCGA-BQ	KIRP	78	MALE	BLACK O	Stage I	Stage I	Kidney Papillary Ren	2001	-28709	Dead	TUMOR FREE		1468			1	1468	0	1468			0	1468
TCGA-BQ	TCGA-BQ	KIRP	61	MALE	WHITE	Stage I	Stage I	Kidney Papillary Ren	2002	-22337	Alive	TUMOR F	2948				0	2948	0	2948			0	2948
TCGA-BQ	TCGA-BQ	KIRP	61	MALE	WHITE	Stage I	Stage I	Kidney Papillary Ren	2002	-22337	Alive	TUMOR F	2948				0	2948	0	2948			0	2948
TCGA-BQ	TCGA-BQ	KIRP	64	MALE	WHITE	Stage III	Stage III	Kidney Papillary Ren	2003	-23460	Alive	WITH TU	2839		229		0	2839	0	2839			1	229
TCGA-BQ	TCGA-BQ	KIRP	64	MALE	WHITE	Stage III	Stage III	Kidney Papillary Ren	2003	-23460	Alive	WITH TU	2839		229		0	2839	0	2839			1	229
TCGA-BQ	TCGA-BQ	KIRP	45	FEMALE	WHITE	Stage I	Stage I	Kidney Papillary Ren	2005	-16717	Dead	TUMOR FREE		1364			1	1364	0	1364			0	1364
TCGA-BQ	TCGA-BQ	KIRP	45	FEMALE	WHITE	Stage I	Stage I	Kidney Papillary Ren	2005	-16717	Dead	TUMOR FREE		1364			1	1364	0	1364			0	1364
TCGA-BQ	TCGA-BQ	KIRP	53	FEMALE	WHITE	Stage I	Stage I	Kidney Papillary Ren	2006	-19550	Alive	TUMOR F	1864				0	1864	0	1864			0	1864
TCGA-BQ	TCGA-BQ	KIRP	53	FEMALE	WHITE	Stage I	Stage I	Kidney Papillary Ren	2006	-19550	Alive	TUMOR F	1864				0	1864	0	1864			0	1864
TCGA-BQ	TCGA-BQ	KIRP	74	MALE	WHITE	Stage II	Stage II	Kidney Papillary Ren	2007	-27120	Alive	TUMOR F	1533				0	1533	0	1533			0	1533
TCGA-BQ	TCGA-BQ	KIRP	74	MALE	WHITE	Stage II	Stage II	Kidney Papillary Ren	2007	-27120	Alive	TUMOR F	1533				0	1533	0	1533			0	1533
TCGA-BQ	TCGA-BQ	KIRP	57	FEMALE	BLACK O	Stage III	Stage III	Kidney Papillary Ren	2007	-20837	Alive	TUMOR F	1516				0	1516	0	1516			0	1516
TCGA-BQ	TCGA-BQ	KIRP	57	FEMALE	BLACK O	Stage III	Stage III	Kidney Papillary Ren	2007	-20837	Alive	TUMOR F	1516				0	1516	0	1516			0	1516
TCGA-BQ	TCGA-BQ	KIRP	73	MALE	WHITE	Stage I	Stage I	Kidney Papillary Ren	2008	-26749	Alive	TUMOR F	799				0	799	0	799			0	799
TCGA-BQ	TCGA-BQ	KIRP	73	MALE	WHITE	Stage I	Stage I	Kidney Papillary Ren	2008	-26749	Alive	TUMOR F	799				0	799	0	799			0	799
TCGA-BQ	TCGA-BQ	KIRP	75	FEMALE	WHITE	Stage III	[Discrepar	Kidney Papillary Ren	2008	-27467	Alive	WITH TU	612		143		0	612	0	612			1	143
TCGA-BQ	TCGA-BQ	KIRP	75	FEMALE	WHITE	Stage III	[Discrepar	Kidney Papillary Ren	2008	-27467	Alive	WITH TU	612		143		0	612	0	612			1	143
TCGA-BQ	TCGA-BQ	KIRP	81	MALE	WHITE	Stage III	Stage III	Kidney Papillary Ren	2010	-29856	Dead	WITH TUMOR		343	50		1	343	1	343			1	50
TCGA-BQ	TCGA-BQ	KIRP	81	MALE	WHITE	Stage III	Stage III	Kidney Papillary Ren	2010	-29856	Dead	WITH TUMOR		343	50		1	343	1	343			1	50
TCGA-BQ	TCGA-BQ	KIRP	71	MALE	WHITE	Stage I	Stage I	Kidney Papillary Ren	2010	-26136	Alive	TUMOR F	232				0	232	0	232			0	232
TCGA-BQ	TCGA-BQ	KIRP	71	MALE	WHITE	Stage I	Stage I	Kidney Papillary Ren	2010	-26136	Alive	TUMOR F	232				0	232	0	232			0	232
TCGA-BQ	TCGA-BQ	KIRP	53	MALE	AMERICA	Stage I	Stage I	Kidney Papillary Ren	2011	-19479	Alive	TUMOR F	214				0	214	0	214			0	214
TCGA-BQ	TCGA-BQ	KIRP	53	MALE	AMERICA	Stage I	Stage I	Kidney Papillary Ren	2011	-19479	Alive	TUMOR F	214				0	214	0	214			0	214
TCGA-BQ	TCGA-BQ	KIRP	83	FEMALE	WHITE	Stage I	Stage I	Kidney Papillary Ren	2011	-30662	Alive	TUMOR F	218				0	218	0	218			0	218
TCGA-BQ	TCGA-BQ	KIRP	83	FEMALE	WHITE	Stage I	Stage I	Kidney Papillary Ren	2011	-30662	Alive	TUMOR F	218				0	218	0	218			0	218
TCGA-BQ	TCGA-BQ	KIRP	64	MALE	BLACK O	Stage I	Stage I	Kidney Papillary Ren	2011	-23559	Alive	TUMOR F	116				0	116	0	116			0	116
TCGA-BQ	TCGA-BQ	KIRP	64	MALE	BLACK O	Stage I	Stage I	Kidney Papillary Ren	2011	-23559	Alive	TUMOR F	116				0	116	0	116			0	116
TCGA-DV	TCGA-DV	KIRP	38	FEMALE	WHITE	Stage I	Stage I	Kidney Papillary Ren	2009	-13964	Alive	TUMOR F	1486			Complete Remission/Response	0	1486	0	1486	0	1486	0	1486
TCGA-DV	TCGA-DV	KIRP	51	MALE	BLACK O	Stage I	Stage I	Kidney Papillary Ren	2008	-18744	Alive	[Discrepar	414				0	414	0	414			0	414
TCGA-DV	TCGA-DV	KIRP	53	MALE	WHITE	Stage I	Stage I	Kidney Papillary Ren	2001	-19660	Alive	TUMOR F	3950				0	3950	0	3950			0	3950
TCGA-DV	TCGA-DV	KIRP	55	MALE	WHITE	Stage I	Stage I	Kidney Papillary Ren	2008	-20269	Alive	TUMOR F	1093				0	1093	0	1093			0	1093
TCGA-DV	TCGA-DV	KIRP	43	MALE	BLACK O	Stage I	Stage I	Kidney Papillary Ren	2007	-15891	Alive	WITH TU	1142				0	1142	0	1142			0	1142
TCGA-DV	TCGA-DV	KIRP	66	MALE	WHITE	Stage I	Stage I	Kidney Papillary Ren	2009	-24247	Alive	WITH TU	744		380		0	744	0	744			1	380
TCGA-DV	TCGA-DV	KIRP	58	FEMALE	WHITE	Stage I	Stage I	Kidney Papillary Ren	2010	-21347	Alive	TUMOR F	419				0	419	0	419			0	419
TCGA-DV	TCGA-DV	KIRP	60	MALE	BLACK O	Stage I	Stage I	Kidney Papillary Ren	2011	-22141	Alive		114				0	114	0	114			0	114
TCGA-DV	TCGA-DV	KIRP	61	MALE	BLACK O	Stage I	Stage I	Kidney Papillary Ren	2010	-22403	Alive	TUMOR F	445				0	445	0	445			0	445
TCGA-DV	TCGA-DV	KIRP	52	MALE	WHITE	Stage I	Stage I	Kidney Papillary Ren	2011	-19166	Alive	WITH TU	92				0	92	0	92			0	92
TCGA-DV	TCGA-DV	KIRP	43	MALE	BLACK O	Stage I	Stage I	Kidney Papillary Ren	2010	-15967	Alive	TUMOR F	1240			Complete Remission/Response	0	1240	0	1240	0	1240	0	1240
TCGA-DZ	TCGA-DZ	KIRP	31	MALE	WHITE	Stage III		Kidney Papillary Ren	2003	-11487	Alive	TUMOR F	2429				0	2429	0	2429			0	2429
TCGA-DZ	TCGA-DZ	KIRP	31	MALE	WHITE	Stage III		Kidney Papillary Ren	2003	-11487	Alive	TUMOR F	2429				0	2429	0	2429			0	2429
TCGA-DZ	TCGA-DZ	KIRP	69	MALE		Stage I		Kidney Papillary Ren	2003	-25417	Alive	TUMOR F	2460				0	2460	0	2460			0	2460
TCGA-DZ	TCGA-DZ	KIRP	69	MALE		Stage I		Kidney Papillary Ren	2003	-25417	Alive	TUMOR F	2460				0	2460	0	2460			0	2460
TCGA-DZ	TCGA-DZ	KIRP	66	FEMALE	WHITE	Stage I		Kidney Papillary Ren	2004	-24311	Dead	TUMOR FREE		1557			1	1557	0	1557			0	1557
TCGA-DZ	TCGA-DZ	KIRP	66	FEMALE	WHITE	Stage I		Kidney Papillary Ren	2004	-24311	Dead	TUMOR FREE		1557			1	1557	0	1557			0	1557
TCGA-DZ	TCGA-DZ	KIRP	69	MALE	WHITE	Stage I		Kidney Papillary Ren	2005	-25529	Alive	TUMOR F	1856				0	1856	0	1856			0	1856
TCGA-DZ	TCGA-DZ	KIRP	69	MALE	WHITE	Stage I		Kidney Papillary Ren	2005	-25529	Alive	TUMOR F	1856				0	1856	0	1856			0	1856
TCGA-DZ	TCGA-DZ	KIRP	45	MALE		Stage I		Kidney Papillary Ren	2006	-16468	Alive	TUMOR F	2269			[Discrepancy]	0	2269	0	2269			0	2269
TCGA-DZ	TCGA-DZ	KIRP	45	MALE		Stage I		Kidney Papillary Ren	2006	-16468	Alive	TUMOR F	2269			[Discrepancy]	0	2269	0	2269			0	2269
TCGA-EV	TCGA-EV	KIRP	47	FEMALE	WHITE	Stage I	Stage I	Kidney Papillary Ren	2010	-17275	Alive	TUMOR F	1040				0	1040	0	1040			0	1040
TCGA-EV	TCGA-EV	KIRP	58	MALE	WHITE	Stage I	Stage I	Kidney Papillary Ren	2010	-21344	Alive	TUMOR F	578				0	578	0	578			0	578
TCGA-EV	TCGA-EV	KIRP	57	MALE	WHITE	Stage I	Stage I	Kidney Papillary Ren	2010	-20888	Alive	TUMOR F	757				0	757	0	757			0	757
TCGA-F9	TCGA-F9	KIRP	35	FEMALE	BLACK O	Stage III	Stage III	Kidney Papillary Ren	2012	-12893	Dead	WITH TUMOR		325	109	[Unknown]	1	325	1	325			1	109
TCGA-F9	TCGA-F9	KIRP	37	FEMALE	ASIAN	Stage I	Stage I	Kidney Papillary Ren	2013	-13523	Alive	TUMOR F	398			Complete Remission/Response	0	398	0	398	0	398	0	398
TCGA-F9	TCGA-F9	KIRP	75	FEMALE	WHITE	Stage I	Stage I	Kidney Papillary Ren	2013	-27446	Alive	TUMOR F	73			Complete Remission/Response	0	73	0	73	0	73	0	73
TCGA-F9	TCGA-F9	KIRP	38	FEMALE	ASIAN	Stage IV	Stage IV	Kidney Papillary Ren	2013	-14101	Alive	TUMOR F	36			Complete Remission/Response	0	36	0	36			0	36
TCGA-F9	TCGA-F9	KIRP	79	MALE	ASIAN	Stage III	Stage III	Kidney Papillary Ren	2013	-29156	Alive	TUMOR F	13			Complete Remission/Response	0	13	0	13	0	13	0	13
TCGA-G7	TCGA-G7	KIRP	53	FEMALE	WHITE	Stage IV		Kidney Papillary Ren	2004	-19593	Dead	WITH TUMOR		122			1	122	1	122			1	122
TCGA-G7	TCGA-G7	KIRP	57	MALE	WHITE	Stage I	Stage I	Kidney Papillary Ren	2007	-21169	Alive	TUMOR F	1575		1558		0	1575	0	1575			1	1558
TCGA-G7	TCGA-G7	KIRP	40	MALE	BLACK O	Stage I	Stage I	Kidney Papillary Ren	2007	-14664	Alive	TUMOR F	2765			Complete Remission/Response	0	2765	0	2765	0	2765	0	2765
TCGA-G7	TCGA-G7	KIRP	60	FEMALE	WHITE	Stage IV	Stage IV	Kidney Papillary Ren	2007	-22215	Dead	WITH TUMOR		336			1	336	1	336			1	336
TCGA-G7	TCGA-G7	KIRP	56	MALE	WHITE	Stage I	Stage I	Kidney Papillary Ren	2008	-20478	Alive	TUMOR F	1194				0	1194	0	1194			0	1194
TCGA-G7	TCGA-G7	KIRP	58	MALE	WHITE	Stage I	Stage I	Kidney Papillary Ren	2008	-21252	Alive	TUMOR F	2173			Complete Remission/Response	0	2173	0	2173	0	2173	0	2173
TCGA-G7	TCGA-G7	KIRP	46	MALE	WHITE	Stage III	Stage III	Kidney Papillary Ren	2010	-17004	Alive	TUMOR F	765				0	765	0	765			0	765
TCGA-G7	TCGA-G7	KIRP	55	FEMALE	BLACK O	Stage III	Stage III	Kidney Papillary Ren	2011	-20165	Dead	WITH TUMOR		624	110	Progressive Disease	1	624	1	624			1	110
TCGA-G7	TCGA-G7	KIRP	83	MALE	WHITE	Stage I	Stage I	Kidney Papillary Ren	2011	-30346	Alive	TUMOR F	786			Complete Remission/Response	0	786	0	786	0	786	0	786
TCGA-G7	TCGA-G7	KIRP	40	MALE	BLACK O	Stage I	Stage I	Kidney Papillary Ren	2011	-14966	Alive	TUMOR F	768			Complete Remission/Response	0	768	0	768	0	768	0	768
TCGA-G7	TCGA-G7	KIRP	70	MALE	WHITE	Stage IV		Kidney Papillary Ren	2013	-25782	Alive	WITH TU	547			Stable Disease	0	547	0	547			0	547
TCGA-G7	TCGA-G7	KIRP	61	MALE	WHITE	Stage I	Stage I	Kidney Papillary Ren	2013	-22553	Alive	TUMOR F	702			Complete Remission/Response	0	702	0	702	0	702	0	702
TCGA-G7	TCGA-G7	KIRP	71	MALE	WHITE	Stage III		Kidney Papillary Ren	2012	-25977	Dead	WITH TUMOR		514	209	Complete Remission/Response	1	514	1	514	1	209	1	209
TCGA-G7	TCGA-G7	KIRP	62	MALE	WHITE	Stage I		Kidney Papillary Ren	2008	-22810	Alive	TUMOR F	2453		1447	Complete Remission/Response	0	2453	0	2453	1	1447	1	1447
TCGA-GL	TCGA-GL	KIRP	52	MALE	WHITE	Stage IV		Kidney Papillary Ren	1996	-19012	Alive	TUMOR F	5925				0	5925	0	5925			0	5925
TCGA-GL	TCGA-GL	KIRP	52	MALE	WHITE	Stage IV		Kidney Papillary Ren	1996	-19012	Alive	TUMOR F	5925				0	5925	0	5925			0	5925
TCGA-GL	TCGA-GL	KIRP	63	MALE	WHITE	Stage I		Kidney Papillary Ren	2011	-23239	Alive	TUMOR F	393			Complete Remission/Response	0	393	0	393	0	393	0	393
TCGA-GL	TCGA-GL	KIRP	28	FEMALE	WHITE	Stage III		Kidney Papillary Ren	2011	-10428	Alive	WITH TU	112				0	112	0	112			0	112
TCGA-GL	TCGA-GL	KIRP	28	FEMALE	WHITE	Stage III		Kidney Papillary Ren	2011	-10428	Alive	WITH TU	112				0	112	0	112			0	112
TCGA-GL	TCGA-GL	KIRP	63	MALE	WHITE	Stage I		Kidney Papillary Ren	2011	-23338	Alive	TUMOR F	841			Complete Remission/Response	0	841	0	841	0	841	0	841
TCGA-GL	TCGA-GL	KIRP	61	MALE	WHITE	Stage I		Kidney Papillary Ren	2011	-22635	Alive	TUMOR F	32				0	32	0	32			0	32
TCGA-GL	TCGA-GL	KIRP	79	MALE	WHITE	Stage III	Stage III	Kidney Papillary Ren	2012	-29046	Alive	TUMOR F	378			Complete Remission/Response	0	378	0	378	0	378	0	378
TCGA-GL	TCGA-GL	KIRP	79	MALE	WHITE	Stage III	Stage III	Kidney Papillary Ren	2012	-29046	Alive	TUMOR F	378			Complete Remission/Response	0	378	0	378	0	378	0	378
TCGA-GL	TCGA-GL	KIRP	46	MALE	WHITE	Stage I		Kidney Papillary Ren	2012	-17142	Alive	TUMOR F	546			Complete Remission/Response	0	546	0	546	0	546	0	546
TCGA-GL	TCGA-GL	KIRP	66	FEMALE	WHITE	Stage I		Kidney Papillary Ren	2013	-24348	Alive	TUMOR F	392			Complete Remission/Response	0	392	0	392	0	392	0	392
TCGA-GL	TCGA-GL	KIRP	69	MALE	WHITE	Stage I		Kidney Papillary Ren	2013	-25483	Alive	TUMOR F	341			Complete Remission/Response	0	341	0	341	0	341	0	341
TCGA-GL	TCGA-GL	KIRP	74	MALE	BLACK O	Stage I		Kidney Papillary Ren	2013	-27330	Alive	TUMOR F	496			Complete Remission/Response	0	496	0	496	0	496	0	496
TCGA-GL	TCGA-GL	KIRP	74	MALE	BLACK O	Stage I		Kidney Papillary Ren	2013	-27330	Alive	TUMOR F	496			Complete Remission/Response	0	496	0	496	0	496	0	496
TCGA-HE	TCGA-HE	KIRP		MALE		Stage I		Kidney Papillary Ren	2008		Alive	TUMOR FREE					0		0				0	
TCGA-HE	TCGA-HE	KIRP		MALE		Stage II		Kidney Papillary Ren	2008		Alive	TUMOR F	2492		2399	Complete Remission/Response	0	2492	0	2492	1	2399	1	2399
TCGA-HE	TCGA-HE	KIRP		FEMALE		Stage III		Kidney Papillary Ren	2010		Alive	TUMOR F	1770			Complete Remission/Response	0	1770	0	1770	0	1770	0	1770
TCGA-HE	TCGA-HE	KIRP	58	MALE	[Not Eval	Stage I		Kidney Papillary Renal Cell Carcinoma		-21524	Alive	TUMOR F	2582			Complete Remission/Response	0	2582	0	2582	0	2582	0	2582
TCGA-HE	TCGA-HE	KIRP	62	MALE	[Not Eval	Stage I		Kidney Papillary Renal Cell Carcinoma		-22888	Alive	TUMOR F	791			Complete Remission/Response	0	791	0	791	0	791	0	791
TCGA-HE	TCGA-HE	KIRP	73	MALE	[Not Eval	Stage I		Kidney Papillary Renal Cell Carcinoma		-26985	Alive	TUMOR F	360			Complete Remission/Response	0	360	0	360	0	360	0	360
TCGA-HE	TCGA-HE	KIRP	58	MALE	[Not Evaluated]			Kidney Papillary Renal Cell Carcinoma		-21485	Alive	TUMOR F	1726			Complete Remission/Response	0	1726	0	1726	0	1726	0	1726
TCGA-HE	TCGA-HE	KIRP	82	MALE	[Not Evaluated]			Kidney Papillary Renal Cell Carcinoma		-30088	Alive	TUMOR F	2228			Complete Remission/Response	0	2228	0	2228	0	2228	0	2228
TCGA-HE	TCGA-HE	KIRP	51	MALE	[Not Eval	Stage I		Kidney Papillary Renal Cell Carcinoma		-18913	Alive	TUMOR F	767			Complete Remission/Response	0	767	0	767	0	767	0	767
TCGA-IA-	TCGA-IA-	KIRP	56	MALE	WHITE	Stage III	Stage III	Kidney Papillary Ren	2011	-20463	Alive	TUMOR F	511			Complete Remission/Response	0	511	0	511	0	511	0	511
TCGA-IA-	TCGA-IA-	KIRP	49	FEMALE	BLACK O	Stage I	Stage I	Kidney Papillary Ren	2011	-18239	Alive	TUMOR F	608			Complete Remission/Response	0	608	0	608	0	608	0	608
TCGA-IA-	TCGA-IA-	KIRP	60	FEMALE	WHITE	Stage III	Stage III	Kidney Papillary Ren	2011	-21996	Alive	TUMOR F	62			Complete Remission/Response	0	62	0	62	0	62	0	62
TCGA-IA-	TCGA-IA-	KIRP	81	MALE	WHITE	[Discrepancy]		Kidney Papillary Ren	2003	-29677	Dead			2609		[Unknown]	1	2609		2609			0	2609
TCGA-IA-	TCGA-IA-	KIRP	80	MALE	WHITE	[Discrepancy]		Kidney Papillary Ren	2005	-29430	Dead			2684		Stable Disease	1	2684		2684			0	2684
TCGA-IA-	TCGA-IA-	KIRP	63	MALE	WHITE	Stage I	Stage I	Kidney Papillary Ren	2002	-23238	Dead			2941		[Unknown]	1	2941		2941			0	2941
TCGA-IA-	TCGA-IA-	KIRP	70	MALE	WHITE	Stage I		Kidney Papillary Ren	2003	-25678	Dead	TUMOR FREE		2043	1408	Complete Remission/Response	1	2043	0	2043	1	1408	1	1408
TCGA-IZ-	TCGA-IZ-	KIRP	82	MALE	WHITE	Stage II	Stage II	Kidney Papillary Ren	2012	-29963	Alive	TUMOR F	677			Complete Remission/Response	0	677	0	677	0	677	0	677
TCGA-IZ-	TCGA-IZ-	KIRP	50	MALE	WHITE	Stage I	Stage I	Kidney Papillary Ren	2012	-18540	Alive	TUMOR F	649			Complete Remission/Response	0	649	0	649	0	649	0	649
TCGA-IZ-	TCGA-IZ-	KIRP	69	MALE	BLACK O	Stage I	Stage I	Kidney Papillary Ren	2013	-25545	Alive	TUMOR F	497			Complete Remission/Response	0	497	0	497	0	497	0	497
TCGA-IZ-	TCGA-IZ-	KIRP	63	MALE	WHITE	Stage I	Stage I	Kidney Papillary Ren	2013	-23149	Alive	TUMOR F	376			Complete Remission/Response	0	376	0	376	0	376	0	376
TCGA-J7-	TCGA-J7-	KIRP	42	MALE	ASIAN	Stage I		Kidney Papillary Ren	2010	-15620	Alive	TUMOR F	388			Complete Remission/Response	0	388	0	388	0	388	0	388
TCGA-J7-	TCGA-J7-	KIRP	37	FEMALE	ASIAN	Stage III	Stage III	Kidney Papillary Ren	2011	-13628	Dead	WITH TUMOR		321	220	Complete Remission/Response	1	321	1	321	1	220	1	220
TCGA-J7-	TCGA-J7-	KIRP	75	MALE	ASIAN	Stage I	Stage I	Kidney Papillary Ren	2013	-27431	Alive	WITH TU	307		268	Complete Remission/Response	0	307	0	307	1	268	1	268
TCGA-KV	TCGA-KV	KIRP	85	MALE	WHITE	Stage I	Stage I	Kidney Papillary Ren	2013	-31217	Alive	TUMOR F	560			Complete Remission/Response	0	560	0	560	0	560	0	560
TCGA-KV	TCGA-KV	KIRP	65	MALE	WHITE	Stage I	Stage I	Kidney Papillary Ren	2011	-23979	Alive	TUMOR F	885			Complete Remission/Response	0	885	0	885	0	885	0	885
TCGA-KV	TCGA-KV	KIRP	40	MALE	WHITE	Stage I	Stage I	Kidney Papillary Ren	2013	-14834	Alive	TUMOR F	347			Complete Remission/Response	0	347	0	347	0	347	0	347
TCGA-MI	TCGA-MI	KIRP	50	MALE	BLACK O	Stage I	Stage I	Kidney Papillary Ren	2011	-18573	Alive	TUMOR F	1043			Complete Remission/Response	0	1043	0	1043	0	1043	0	1043
TCGA-MI	TCGA-MI	KIRP	65	MALE	BLACK O	Stage I	Stage I	Kidney Papillary Ren	2011	-24017	Alive	TUMOR F	845			Complete Remission/Response	0	845	0	845	0	845	0	845
TCGA-MI	TCGA-MI	KIRP	62	MALE	WHITE	Stage I	Stage I	Kidney Papillary Ren	2011	-22866	Alive	TUMOR F	924			Complete Remission/Response	0	924	0	924	0	924	0	924
TCGA-MI	TCGA-MI	KIRP	66	MALE	WHITE	Stage I	Stage I	Kidney Papillary Ren	2012	-24271	Alive	TUMOR F	841			Complete Remission/Response	0	841	0	841	0	841	0	841
TCGA-MI	TCGA-MI	KIRP	57	MALE	BLACK O	Stage I	Stage I	Kidney Papillary Ren	2012	-20933	Alive	TUMOR F	616			Complete Remission/Response	0	616	0	616	0	616	0	616
TCGA-MI	TCGA-MI	KIRP	62	FEMALE	BLACK O	Stage I	Stage I	Kidney Papillary Ren	2013	-22804	Alive	TUMOR F	536			Complete Remission/Response	0	536	0	536	0	536	0	536
TCGA-MI	TCGA-MI	KIRP	52	FEMALE	BLACK O	Stage II	Stage II	Kidney Papillary Ren	2013	-19029	Alive	TUMOR F	369		0	Complete Remission/Response	0	369	0	369	1	0	1	0
TCGA-MI	TCGA-MI	KIRP	54	MALE	WHITE	Stage I	Stage I	Kidney Papillary Ren	2012	-20006	Alive	TUMOR F	947			Complete Remission/Response	0	947	0	947	0	947	0	947
TCGA-MI	TCGA-MI	KIRP	63	MALE	BLACK O	Stage I	Stage I	Kidney Papillary Ren	2012	-23114	Alive	TUMOR F	890			Stable Disease	0	890	0	890			0	890
TCGA-O9	TCGA-O9	KIRP	44	MALE	WHITE	Stage I		Kidney Papillary Ren	2013	-16183	Alive		471			[Unknown]	0	471	0	471			0	471
TCGA-P4	TCGA-P4	KIRP	67	MALE	BLACK O	Stage III	Stage I	Kidney Papillary Ren	2004	-24615	Alive	TUMOR F	3035			Complete Remission/Response	0	3035	0	3035	0	3035	0	3035
TCGA-P4	TCGA-P4	KIRP	74	FEMALE	WHITE	Stage I	Stage I	Kidney Papillary Ren	2004	-27029	Alive	TUMOR F	2649			Complete Remission/Response	0	2649	0	2649	0	2649	0	2649
TCGA-P4	TCGA-P4	KIRP	57	MALE	WHITE	Stage III	Stage III	Kidney Papillary Ren	2004	-21132	Dead	WITH TUMOR		1306	42		1	1306	1	1306			1	42
TCGA-P4	TCGA-P4	KIRP	57	MALE	WHITE	Stage III	Stage III	Kidney Papillary Ren	2004	-21132	Dead	WITH TUMOR		1306	42		1	1306	1	1306			1	42
TCGA-P4	TCGA-P4	KIRP	54	FEMALE	WHITE	Stage III	Stage III	Kidney Papillary Ren	2007	-19748	Dead	WITH TUMOR		201	44	Complete Remission/Response	1	201	1	201	1	44	1	44
TCGA-P4	TCGA-P4	KIRP	83	MALE	WHITE	Stage I	Stage I	Kidney Papillary Ren	2006	-30395	Alive	TUMOR F	3015			Complete Remission/Response	0	3015	0	3015	0	3015	0	3015
TCGA-P4	TCGA-P4	KIRP	51	MALE	WHITE	Stage I	Stage I	Kidney Papillary Ren	2005	-18698	Alive	TUMOR F	2816			Complete Remission/Response	0	2816	0	2816	0	2816	0	2816
TCGA-P4	TCGA-P4	KIRP	51	MALE	WHITE	Stage I	Stage I	Kidney Papillary Ren	2005	-18698	Alive	TUMOR F	2816			Complete Remission/Response	0	2816	0	2816	0	2816	0	2816
TCGA-P4	TCGA-P4	KIRP	74	MALE	WHITE	Stage III	Stage III	Kidney Papillary Ren	2010	-27073	Alive	WITH TU	1590		232	Complete Remission/Response	0	1590	0	1590	1	232	1	232
TCGA-P4	TCGA-P4	KIRP	85	MALE	WHITE	Stage III	Stage III	Kidney Papillary Ren	2010	-31082	Dead	WITH TUMOR		487	41	Complete Remission/Response	1	487	1	487	1	41	1	41
TCGA-P4	TCGA-P4	KIRP	69	MALE	WHITE	Stage I	Stage I	Kidney Papillary Ren	2010	-25280	Alive	TUMOR F	1539			Complete Remission/Response	0	1539	0	1539	0	1539	0	1539
TCGA-P4	TCGA-P4	KIRP	69	MALE	WHITE	Stage I	Stage I	Kidney Papillary Ren	2008	-25354	Alive	TUMOR F	2400			Complete Remission/Response	0	2400	0	2400	0	2400	0	2400
TCGA-PJ-	TCGA-PJ-	KIRP	60	FEMALE	WHITE	Stage I	Stage I	Kidney Papillary Ren	2013	-21968	Alive	TUMOR F	600			Complete Remission/Response	0	600	0	600	0	600	0	600
TCGA-PJ-	TCGA-PJ-	KIRP	80	FEMALE	WHITE	Stage I	Stage I	Kidney Papillary Ren	2012	-29365	Alive	TUMOR F	540			Complete Remission/Response	0	540	0	540	0	540	0	540
TCGA-PJ-	TCGA-PJ-	KIRP	81	FEMALE	WHITE	Stage I	Stage I	Kidney Papillary Ren	2013	-29830	Alive	TUMOR F	380			Complete Remission/Response	0	380	0	380	0	380	0	380
TCGA-O2	TCGA-O2	KIRP	61	FEMALE	BLACK O	Stage III		Kidney Papillary Ren	2012	-22531	Alive		428		412	Complete Remission/Response	0	428	0	428	1	412	1	412
TCGA-SX	TCGA-SX	KIRP	51	MALE	WHITE	Stage I	Stage I	Kidney Papillary Ren	2010	-18742	Alive	TUMOR F	1457			Complete Remission/Response	0	1457	0	1457	0	1457	0	1457
TCGA-SX	TCGA-SX	KIRP	66	FEMALE	WHITE	Stage I	Stage I	Kidney Papillary Ren	2010	-24211	Alive	TUMOR F	1504			Complete Remission/Response	0	1504	0	1504	0	1504	0	1504
TCGA-SX	TCGA-SX	KIRP	57	MALE	WHITE	Stage I	Stage I	Kidney Papillary Ren	2011	-21079	Alive	TUMOR F	1357			Complete Remission/Response	0	1357	0	1357	0	1357	0	1357
TCGA-SX	TCGA-SX	KIRP	80	MALE	WHITE	Stage I	Stage I	Kidney Papillary Ren	2011	-29332	Alive	TUMOR F	876			Complete Remission/Response	0	876	0	876	0	876	0	876
TCGA-SX	TCGA-SX	KIRP	66	MALE	WHITE	Stage I	Stage I	Kidney Papillary Ren	2012	-24110	Alive	TUMOR F	1076			Complete Remission/Response	0	1076	0	1076	0	1076	0	1076
TCGA-SX	TCGA-SX	KIRP	74	MALE	[Not Eval	Stage I	Stage I	Kidney Papillary Ren	2012	-27063	Alive	TUMOR F	823			Complete Remission/Response	0	823	0	823	0	823	0	823
TCGA-SX	TCGA-SX	KIRP	60	MALE	BLACK O	Stage IV	Stage IV	Kidney Papillary Ren	2012	-22044	Dead	WITH TUMOR		363		Progressive Disease	1	363	1	363			1	363
TCGA-SX	TCGA-SX	KIRP	52	MALE	WHITE	Stage I	Stage I	Kidney Papillary Ren	2012	-19116	Alive	TUMOR F	578			Complete Remission/Response	0	578	0	578	0	578	0	578
TCGA-SX	TCGA-SX	KIRP	64	MALE	WHITE	Stage I	Stage I	Kidney Papillary Ren	2011	-23534	Alive	TUMOR F	1257			Complete Remission/Response	0	1257	0	1257	0	1257	0	1257
TCGA-SX	TCGA-SX	KIRP	66	FEMALE	WHITE	Stage I	Stage I	Kidney Papillary Ren	2012	-24382	Dead	TUMOR FREE		496		Complete Remission/Response	1	496	0	496	0	496	0	496
TCGA-SX	TCGA-SX	KIRP	48	MALE	WHITE	Stage I	Stage I	Kidney Papillary Ren	2010	-17696	Alive	TUMOR F	1266			Complete Remission/Response	0	1266	0	1266	0	1266	0	1266
TCGA-SX	TCGA-SX	KIRP	66	MALE	WHITE	Stage II	Stage II	Kidney Papillary Ren	2012	-24237	Alive	TUMOR F	805			Complete Remission/Response	0	805	0	805	0	805	0	805
TCGA-SX	TCGA-SX	KIRP	67	MALE	WHITE	Stage I	Stage I	Kidney Papillary Ren	2010	-24660	Alive	TUMOR F	1615			Complete Remission/Response	0	1615	0	1615	0	1615	0	1615
TCGA-SX	TCGA-SX	KIRP	47	MALE	[Not Eval	Stage I	Stage I	Kidney Papillary Ren	2010	-17349	Alive	TUMOR F	1331			Complete Remission/Response	0	1331	0	1331	0	1331	0	1331
TCGA-UN	TCGA-UN	KIRP	76	FEMALE	BLACK O	Stage I	Stage I	Kidney Papillary Ren	2013	-27851	Alive	TUMOR F	548			Complete Remission/Response	0	548	0	548	0	548	0	548
TCGA-UZ	TCGA-UZ	KIRP	78	MALE	WHITE	Stage I		Kidney Papillary Renal Cell Carcinoma		-28490	Dead			797		Complete Remission/Response	1	797		797	0	797	0	797
TCGA-UZ	TCGA-UZ	KIRP	71	MALE	WHITE	Stage I		Kidney Papillary Renal Cell Carcinoma		-25933	Alive	TUMOR F	3571		3157	Complete Remission/Response	0	3571	0	3571	1	3157	1	3157
TCGA-UZ	TCGA-UZ	KIRP	59	MALE	WHITE	Stage II		Kidney Papillary Renal Cell Carcinoma		-21550	Alive	TUMOR F	3152			Complete Remission/Response	0	3152	0	3152	0	3152	0	3152
TCGA-UZ	TCGA-UZ	KIRP	62	MALE	WHITE	Stage II		Kidney Papillary Renal Cell Carcinoma		-22646	Alive	TUMOR F	3811			Complete Remission/Response	0	3811	0	3811	0	3811	0	3811
TCGA-UZ	TCGA-UZ	KIRP	54	MALE	WHITE	Stage III		Kidney Papillary Renal Cell Carcinoma		-19724	Dead	WITH TUMOR		1136	679	Complete Remission/Response	1	1136	1	1136	1	679	1	679
TCGA-UZ	TCGA-UZ	KIRP	80	MALE	WHITE	Stage I		Kidney Papillary Renal Cell Carcinoma		-29220	Alive	TUMOR F	1911			Complete Remission/Response	0	1911	0	1911	0	1911	0	1911
TCGA-UZ	TCGA-UZ	KIRP	60	MALE	WHITE	Stage I		Kidney Papillary Renal Cell Carcinoma		-21915	Alive	TUMOR F	3010			Complete Remission/Response	0	3010	0	3010	0	3010	0	3010
TCGA-UZ	TCGA-UZ	KIRP	59	MALE	BLACK O	Stage III		Kidney Papillary Renal Cell Carcinoma		-21550	Dead	WITH TUMOR		2624	2148	Complete Remission/Response	1	2624	1	2624	1	2148	1	2148
TCGA-UZ	TCGA-UZ	KIRP	84	MALE	WHITE	Stage I		Kidney Papillary Renal Cell Carcinoma		-30681	Alive	TUMOR F	2627		2494	Complete Remission/Response	0	2627	0	2627	1	2494	1	2494
TCGA-UZ	TCGA-UZ	KIRP	62	FEMALE	BLACK O	Stage II		Kidney Papillary Renal Cell Carcinoma		-22646	Alive	TUMOR F	2167		55	Complete Remission/Response	0	2167	0	2167	1	55	1	55
TCGA-UZ	TCGA-UZ	KIRP	62	FEMALE	BLACK O	Stage II		Kidney Papillary Renal Cell Carcinoma		-22646	Alive	TUMOR F	2167		55	Complete Remission/Response	0	2167	0	2167	1	55	1	55
TCGA-UZ	TCGA-UZ	KIRP	77	MALE	WHITE	Stage I		Kidney Papillary Renal Cell Carcinoma		-28124	Dead	TUMOR FREE		933		Complete Remission/Response	1	933	0	933	0	933	0	933
TCGA-UZ	TCGA-UZ	KIRP	65	MALE	WHITE	Stage I		Kidney Papillary Renal Cell Carcinoma		-23741	Alive	TUMOR F	1444			Complete Remission/Response	0	1444	0	1444	0	1444	0	1444
TCGA-UZ	TCGA-UZ	KIRP	73	MALE	WHITE	Stage I		Kidney Papillary Renal Cell Carcinoma		-26663	Alive	TUMOR F	1981		308	Complete Remission/Response	0	1981	0	1981	1	308	1	308
TCGA-UZ	TCGA-UZ	KIRP	65	MALE	WHITE	Stage III		Kidney Papillary Renal Cell Carcinoma		-23741	Alive	TUMOR F	657			Complete Remission/Response	0	657	0	657	0	657	0	657
TCGA-UZ	TCGA-UZ	KIRP	60	MALE	WHITE	Stage I		Kidney Papillary Renal Cell Carcinoma		-21915	Alive	TUMOR F	3368			Complete Remission/Response	0	3368	0	3368	0	3368	0	3368
TCGA-UZ	TCGA-UZ	KIRP	88	FEMALE	WHITE	Stage I		Kidney Papillary Renal Cell Carcinoma		-32142	Alive	TUMOR F	1562			Complete Remission/Response	0	1562	0	1562	0	1562	0	1562
TCGA-V9	TCGA-V9	KIRP	67	MALE	BLACK O	Stage II	Stage II	Kidney Papillary Ren	2009	-24610	Alive	TUMOR F	965			Complete Remission/Response	0	965	0	965	0	965	0	965
TCGA-WI	TCGA-WI	KIRP	55	MALE	WHITE	Stage I	Stage I	Kidney Papillary Ren	2012		Alive	TUMOR F	791		791	Complete Remission/Response	0	791	0	791	1	791	1	791
TCGA-WI	TCGA-WI	KIRP	60	FEMALE	WHITE	Stage I	Stage I	Kidney Papillary Ren	2011		Alive	WITH TU	1188		1157	Complete Remission/Response	0	1188	0	1188	1	1157	1	1157
TCGA-Y8	TCGA-Y8	KIRP	57	FEMALE	WHITE	Stage I		Kidney Papillary Ren	2013	-20964	Alive	TUMOR F	567			Complete Remission/Response	0	567	0	567	0	567	0	567
TCGA-Y8	TCGA-Y8	KIRP	74	MALE	WHITE	Stage I		Kidney Papillary Ren	2013	-27139	Alive	TUMOR F	484			Complete Remission/Response	0	484	0	484	0	484	0	484
TCGA-Y8	TCGA-Y8	KIRP	62	MALE	WHITE	Stage III		Kidney Papillary Ren	2013	-22750	Alive	TUMOR F	567			Complete Remission/Response	0	567	0	567	0	567	0	567
TCGA-Y8	TCGA-Y8	KIRP	68	FEMALE	WHITE	Stage I		Kidney Papillary Ren	2013	-25047	Alive	TUMOR F	549		450	Complete Remission/Response	0	549	0	549	1	450	1	450
TCGA-Y8	TCGA-Y8	KIRP	69	MALE	WHITE	Stage I		Kidney Papillary Ren	2013	-25344	Alive	TUMOR F	475			Complete Remission/Response	0	475	0	475	0	475	0	475
TCGA-Y8	TCGA-Y8	KIRP	63	MALE	WHITE	Stage I		Kidney Papillary Ren	2012	-23192	Alive	TUMOR F	769			Complete Remission/Response	0	769	0	769	0	769	0	769
TCGA-Y8	TCGA-Y8	KIRP	63	MALE	WHITE	Stage I		Kidney Papillary Ren	2012	-23192	Alive	TUMOR F	769			Complete Remission/Response	0	769	0	769	0	769	0	769

TCGA-Y8	TCGA-Y8	KIRP	55	MALE	WHITE	Stage I	Kidney Papillary Ren		2013	-20386	Alive	TUMOR F	205			Complete Remission/Response	0	205	0	205	0	205	0	205
TCGA-Y8	TCGA-Y8	KIRP	58	MALE	WHITE	Stage I	Kidney Papillary Ren		2013	-21383	Alive	TUMOR F	183			Complete Remission/Response	0	183	0	183	0	183	0	183
TCGA-Y8	TCGA-Y8	KIRP	61	MALE	WHITE	Stage I	Kidney Papillary Ren		2013	-22319	Alive	TUMOR F	404			Complete Remission/Response	0	404	0	404	0	404	0	404
TCGA-AB	TCGA-AB	LAML	50	MALE	WHITE				2001	-18385	Dead			365			1	365						
TCGA-AB	TCGA-AB	LAML	61	FEMALE	WHITE				2001	-22584	Dead			792			1	792						
TCGA-AB	TCGA-AB	LAML	30	MALE	WHITE				2001	-11203	Alive		2557				0	2557	0					
TCGA-AB	TCGA-AB	LAML	77	MALE	WHITE				2002	-28124	Dead			577			1	577						
TCGA-AB	TCGA-AB	LAML	46	MALE	WHITE				2002	-16892	Dead			945			1	945						
TCGA-AB	TCGA-AB	LAML	68	FEMALE	WHITE				2002	-25143	Dead			181			1	181						
TCGA-AB	TCGA-AB	LAML	23	MALE	WHITE				2002	-8521	Alive		2861				0	2861	0					
TCGA-AB	TCGA-AB	LAML	64	FEMALE	WHITE				2002	-23679	Dead			62			1	62						
TCGA-AB	TCGA-AB	LAML	76	FEMALE	BLACK OR AFRICAN AMERICAN				2002	-27971	Dead			31			1	31						
TCGA-AB	TCGA-AB	LAML	81	MALE	WHITE				2002	-29766	Dead			243			1	243						
TCGA-AB	TCGA-AB	LAML	25	FEMALE	BLACK OR AFRICAN AMERICAN				2004	-9253	Dead			366			1	366						
TCGA-AB	TCGA-AB	LAML	78	MALE	WHITE				2005	-28581	Dead			31			1	31						
TCGA-AB	TCGA-AB	LAML	39	FEMALE	WHITE				2003	-14427	Dead			792			1	792						
TCGA-AB	TCGA-AB	LAML	49	MALE	WHITE				2004	-17988	Dead			822			1	822						
TCGA-AB	TCGA-AB	LAML	57	FEMALE	WHITE				2003	-20939	Dead			245			1	245						
TCGA-AB	TCGA-AB	LAML	64	MALE	WHITE				2005	-23376	Dead			273			1	273						
TCGA-AB	TCGA-AB	LAML	62	FEMALE	ASIAN				2002	-22707	Dead			303			1	303						
TCGA-AB	TCGA-AB	LAML	52	FEMALE	WHITE				2003	-19054	Alive		2496				0	2496	0					
TCGA-AB	TCGA-AB	LAML	76	MALE	WHITE				2003	-27971	Dead			0			1	0						
TCGA-AB	TCGA-AB	LAML	64	MALE	WHITE				2005	-23527	Dead			822			1	822						
TCGA-AB	TCGA-AB	LAML	65	MALE	WHITE				2003	-24017	Dead			973			1	973						
TCGA-AB	TCGA-AB	LAML	61	FEMALE	WHITE				2006	-22522	Dead			0			1	0						
TCGA-AB	TCGA-AB	LAML	45	MALE	WHITE				2004	-16437	Dead			30			1	30						
TCGA-AB	TCGA-AB	LAML	31	FEMALE	WHITE				2006	-11384	Dead			212			1	212						
TCGA-AB	TCGA-AB	LAML	64	FEMALE	WHITE				2002	-23468	Dead			731			1	731						
TCGA-AB	TCGA-AB	LAML	33	MALE	WHITE				2003	-12237	Dead			273			1	273						
TCGA-AB	TCGA-AB	LAML	55	MALE	WHITE				2003	-20393	Alive		2284				0	2284	0					
TCGA-AB	TCGA-AB	LAML	50	MALE	BLACK OR AFRICAN AMERICAN				2004	-18386	Dead			273			1	273						
TCGA-AB	TCGA-AB	LAML	64	FEMALE	WHITE				2005	-23404	Dead			275			1	275						
TCGA-AB	TCGA-AB	LAML	59	MALE	WHITE				2004	-21550	Dead			640			1	640						
TCGA-AB	TCGA-AB	LAML	60	FEMALE	WHITE				2005	-21915	Dead			365			1	365						
TCGA-AB	TCGA-AB	LAML	77	MALE	WHITE				2002	-28308	Dead			456			1	456						
TCGA-AB	TCGA-AB	LAML	33	MALE	WHITE				2004	-12266	Dead			245			1	245						
TCGA-AB	TCGA-AB	LAML	48	MALE	WHITE				2005	-17624	Alive		1673				0	1673	0					
TCGA-AB	TCGA-AB	LAML	35	MALE	WHITE				2005	-12904	Dead			518			1	518						
TCGA-AB	TCGA-AB	LAML	66	FEMALE	WHITE				2002	-24381	Dead			0			1	0						
TCGA-AB	TCGA-AB	LAML	67	MALE	WHITE				2002	-24503	Dead			365			1	365						
TCGA-AB	TCGA-AB	LAML	51	FEMALE	WHITE				2003	-18963	Dead			486			1	486						
TCGA-AB	TCGA-AB	LAML	74	MALE	WHITE				2003	-27243	Dead			28			1	28						
TCGA-AB	TCGA-AB	LAML	51	FEMALE	WHITE				2004	-18809	Dead			1401			1	1401						
TCGA-AB	TCGA-AB	LAML	65	MALE	WHITE				2004	-23741	Dead			0			1	0						
TCGA-AB	TCGA-AB	LAML	77	MALE	WHITE				2004	-28275	Dead			215			1	215						
TCGA-AB	TCGA-AB	LAML	63	MALE	WHITE				2005	-23254	Dead			122			1	122						
TCGA-AB	TCGA-AB	LAML	37	FEMALE	WHITE				2002	-13728	Dead			304			1	304						
TCGA-AB	TCGA-AB	LAML	57	FEMALE	WHITE				2005	-20910	Dead			1402			1	1402						
TCGA-AB	TCGA-AB	LAML	53	MALE	WHITE				2004	-19481	Dead			608			1	608						
TCGA-AB	TCGA-AB	LAML	62	MALE	WHITE				2005	-22980	Dead			455			1	455						
TCGA-AB	TCGA-AB	LAML	39	MALE	WHITE				2004	-14520	Alive		2220				0	2220	0					
TCGA-AB	TCGA-AB	LAML	72	FEMALE	BLACK OR AFRICAN AMERICAN				2003	-26298	Dead			61			1	61						
TCGA-AB	TCGA-AB	LAML	66	FEMALE	WHITE				2005	-24319	Dead			242			1	242						
TCGA-AB	TCGA-AB	LAML	51	MALE	WHITE				2003	-18720	Dead			89			1	89						
TCGA-AB	TCGA-AB	LAML	51	FEMALE	WHITE				2003	-18809	Dead			366			1	366						
TCGA-AB	TCGA-AB	LAML	18	MALE	WHITE				2004	-6636	Dead			212			1	212						
TCGA-AB	TCGA-AB	LAML	63	MALE	WHITE				2006	-23317	Dead			150			1	150						
TCGA-AB	TCGA-AB	LAML	54	MALE	WHITE				2005	-19905	Dead			306			1	306						
TCGA-AB	TCGA-AB	LAML	75	FEMALE	WHITE				2004	-27547	Dead			577			1	577						
TCGA-AB	TCGA-AB	LAML	40	MALE	BLACK OR AFRICAN AMERICAN				2005	-14671	Dead			304			1	304						
TCGA-AB	TCGA-AB	LAML	60	FEMALE	WHITE				2003	-22035	Dead			427			1	427						
TCGA-AB	TCGA-AB	LAML	76	MALE	WHITE				2005	-27940	Dead			0			1	0						
TCGA-AB	TCGA-AB	LAML	33	FEMALE	BLACK OR AFRICAN AMERICAN				2005	-12266	Alive		1430				0	1430	0					
TCGA-AB	TCGA-AB	LAML	63	MALE	WHITE				2003	-23132	Dead			31			1	31						
TCGA-AB	TCGA-AB	LAML	54	FEMALE	WHITE				2005	-19724	Dead			577			1	577						
TCGA-AB	TCGA-AB	LAML	75	MALE	WHITE				2005	-27486	Dead			61			1	61						
TCGA-AB	TCGA-AB	LAML	67	MALE	WHITE				2005	-24684	Dead			153			1	153						
TCGA-AB	TCGA-AB	LAML	66	FEMALE	WHITE				2002	-24257	Dead			184			1	184						
TCGA-AB	TCGA-AB	LAML	77	MALE	WHITE				2002	-28216	Dead			151			1	151						
TCGA-AB	TCGA-AB	LAML	64	FEMALE	BLACK OR AFRICAN AMERICAN				2009	-23679	Alive		243				0	243	0					
TCGA-AB	TCGA-AB	LAML	76	MALE	WHITE				2007	-27787	Dead			153			1	153						
TCGA-AB	TCGA-AB	LAML	51	MALE	BLACK OR AFRICAN AMERICAN				2009	-18689	Alive		153				0	153	0					
TCGA-AB	TCGA-AB	LAML	42	MALE	BLACK OR AFRICAN AMERICAN				2008	-15461	Alive		638				0	638	0					
TCGA-AB	TCGA-AB	LAML	51	FEMALE	WHITE				2009	-18689	Alive		273				0	273	0					
TCGA-AB	TCGA-AB	LAML	59	MALE	WHITE				2009	-21764	Alive		396				0	396	0					
TCGA-AB	TCGA-AB	LAML	43	MALE	WHITE				2009	-16010	Alive		212				0	212	0					
TCGA-AB	TCGA-AB	LAML	45	FEMALE	WHITE				2006	-16648	Alive		1219				0	1219	0					
TCGA-AB	TCGA-AB	LAML	60	FEMALE	WHITE				2008	-21946	Alive		638				0	638	0					
TCGA-AB	TCGA-AB	LAML	47	FEMALE	WHITE				2006	-17501	Dead			365			1	365						
TCGA-AB	TCGA-AB	LAML	68	FEMALE	WHITE				2009	-25141	Alive		245				0	245	0					
TCGA-AB	TCGA-AB	LAML	24	MALE	WHITE				2009	-8827	Dead		424				1							
TCGA-AB	TCGA-AB	LAML	48	FEMALE	WHITE				2009	-17808	Alive		393				0	393	0					
TCGA-AB	TCGA-AB	LAML	73	FEMALE	WHITE				2006	-26844	Dead			365			1	365						
TCGA-AB	TCGA-AB	LAML	60	MALE	WHITE				2008	-22067	Alive		730				0	730	0					
TCGA-AB	TCGA-AB	LAML	44	FEMALE	WHITE				2008	-16285	Dead			731			1	731						
TCGA-AB	TCGA-AB	LAML	71	MALE	WHITE				2009	-26176	Dead			214			1	214						
TCGA-AB	TCGA-AB	LAML	26	MALE	WHITE				2009	-9497	Alive		181				0	181	0					
TCGA-AB	TCGA-AB	LAML	60	FEMALE	WHITE				2009	-21946	Dead						1							
TCGA-AB	TCGA-AB	LAML	57	MALE	WHITE				2009	-20879	Alive		334				0	334	0					
TCGA-AB	TCGA-AB	LAML	55	MALE	WHITE				2009	-20150	Alive		304				0	304	0					
TCGA-AB	TCGA-AB	LAML	69	MALE	WHITE				2009	-25234	Dead			0			1	0						
TCGA-AB	TCGA-AB	LAML	42	MALE	BLACK OR AFRICAN AMERICAN				2009	-15400	Dead						1							
TCGA-AB	TCGA-AB	LAML	42	FEMALE	WHITE				2006	-15614	Alive		943				0	943	0					
TCGA-AB	TCGA-AB	LAML	45	MALE	WHITE				2006	-16617	Dead			212			1	212						
TCGA-AB	TCGA-AB	LAML	50	FEMALE	WHITE				2007	-18323	Dead			181			1	181						
TCGA-AB	TCGA-AB	LAML	41	FEMALE	WHITE				2009	-15310	Dead			153			1	153						
TCGA-AB	TCGA-AB	LAML	21	FEMALE	WHITE				2007	-7882	Dead			214			1	214						
TCGA-AB	TCGA-AB	LAML	50	FEMALE	WHITE				2009	-18413	Alive		243				0	243	0					
TCGA-AB	TCGA-AB	LAML	69	FEMALE	WHITE				2009	-25326	Alive		393				0	393	0					
TCGA-AB	TCGA-AB	LAML	76	FEMALE	WHITE				2006	-27787	Dead			671			1	671						
TCGA-AB	TCGA-AB	LAML	70	MALE	BLACK OR AFRICAN AMERICAN				2009	-25627	Dead			184			1	184						
TCGA-AB	TCGA-AB	LAML	27	MALE	BLACK OR AFRICAN AMERICAN				2009	-10046	Alive		59				0	59	0					
TCGA-AB	TCGA-AB	LAML	76	FEMALE	WHITE				2006	-27971	Dead			0			1	0						
TCGA-AB	TCGA-AB	LAML	65	MALE	WHITE				2008	-23833	Dead			517			1	517						
TCGA-AB	TCGA-AB	LAML	48	MALE	WHITE				2005	-17869	Alive		1611				0	1611	0					
TCGA-AB	TCGA-AB	LAML	59	MALE	WHITE				2008	-21581	Alive		577				0	577	0					
TCGA-AB	TCGA-AB	LAML	69	MALE	WHITE				2008	-25264	Dead						1							
TCGA-AB	TCGA-AB	LAML	81	MALE	WHITE				2008	-29769	Dead			31			1	31						
TCGA-AB	TCGA-AB	LAML	22	MALE	ASIAN				2009	-8220	Alive		30				0	30	0					
TCGA-AB	TCGA-AB	LAML	61	FEMALE	WHITE				2008	-22311	Dead			0			1	0						
TCGA-AB	TCGA-AB	LAML	51	FEMALE	BLACK OR AFRICAN AMERICAN				2006	-18689	Alive		1186				0	1186	0					
TCGA-AB	TCGA-AB	LAML	63	MALE	WHITE				2007	-23345	Dead			274			1	274						
TCGA-AB	TCGA-AB	LAML	61	MALE	WHITE				2006	-22553	Alive		1216				0	1216	0					
TCGA-AB	TCGA-AB	LAML	22	FEMALE	WHITE				2007	-8280	Alive		792				0	792	0					
TCGA-AB	TCGA-AB	LAML	73	FEMALE	WHITE				2009	-26968	Dead			0			1	0						
TCGA-AB	TCGA-AB	LAML	48	FEMALE	WHITE				2007	-17866	Alive		882				0	882	0					
TCGA-AB	TCGA-AB	LAML	41	FEMALE	WHITE				2006	-15128	Alive		1216				0	1216	0					
TCGA-AB	TCGA-AB	LAML	47	FEMALE	WHITE				2009	-17198	Dead						1							
TCGA-AB	TCGA-AB	LAML	54	FEMALE	WHITE				2009	-19724	Alive		61				0	61	0					
TCGA-AB	TCGA-AB	LAML	44	MALE	WHITE				2007	-16071	Dead			366			1	366						
TCGA-AB	TCGA-AB	LAML	56	FEMALE	WHITE				2009	-20454	Dead						1							
TCGA-AB	TCGA-AB	LAML	83	MALE	WHITE				2008	-30438	Dead			0			1	0						
TCGA-AB	TCGA-AB	LAML	78	MALE	WHITE				2008	-28551	Dead			31			1	31						
TCGA-AB	TCGA-AB	LAML	59	MALE	WHITE				2009	-21825	Alive		90				0	90	0					
TCGA-AB	TCGA-AB	LAML	57	MALE	WHITE				2007	-20850	Dead			243			1	243						
TCGA-AB	TCGA-AB	LAML	57	FEMALE	WHITE				2008	-20820	Dead			457			1	457						
TCGA-AB	TCGA-AB	LAML	88	FEMALE	WHITE				2009	-32326	Dead			90			1	90						
TCGA-AB	TCGA-AB	LAML	43	FEMALE	BLACK OR AFRICAN AMERICAN				2010	-15890	Dead		59				1							
TCGA-AB	TCGA-AB	LAML	71	FEMALE	WHITE				2007	-26054	Dead			123			1	123						
TCGA-AB	TCGA-AB	LAML	63	FEMALE	WHITE				2009	-23011	Dead						1							
TCGA-AB	TCGA-AB	LAML	75	MALE	WHITE				2007	-27485	Dead			0			1	0						
TCGA-AB	TCGA-AB	LAML	62	MALE	WHITE				2008	-22738	Alive		0				0	0	0					
TCGA-AB	TCGA-AB	LAML	58	MALE	WHITE				2007	-21396	Dead			122			1	122						
TCGA-AB	TCGA-AB	LAML	65	MALE	WHITE				2010	-23956	Alive		28				0	28	0					
TCGA-AB	TCGA-AB	LAML	66	MALE	WHITE				2007	-24349	Dead			61			1	61						
TCGA-AB	TCGA-AB	LAML	61	FEMALE	WHITE				2010	-22280	Alive		59				0	59	0					
TCGA-AB	TCGA-AB	LAML	36	FEMALE	WHITE				2007	-13149	Dead			215			1	215						
TCGA-AB	TCGA-AB	LAML	76	MALE	WHITE				2005	-27881	Dead			304			1	304						
TCGA-AB	TCGA-AB	LAML	72	MALE	WHITE				2008	-26512	Alive		455				0	455	0					
TCGA-AB	TCGA-AB	LAML	35	MALE	WHITE				2006	-12876	Dead						1							
TCGA-AB	TCGA-AB	LAML	73	MALE	WHITE				2006	-26998	Dead			0			1	0						
TCGA-AB	TCGA-AB	LAML	67	FEMALE	WHITE				2008	-24776	Alive		641				0	641	0					
TCGA-AB	TCGA-AB	LAML	70	FEMALE	WHITE				2010	-25568	Dead						1							
TCGA-AB	TCGA-AB	LAML	48	MALE	WHITE				2010	-17622	Dead						1							
TCGA-AB	TCGA-AB	LAML	65	FEMALE	WHITE				2006	-23984	Dead			243			1	243						
TCGA-AB	TCGA-AB	LAML	41	MALE	WHITE				2010	-15156	Alive						0		0					
TCGA-AB	TCGA-AB	LAML	52	MALE	WHITE				2009	-19146	Alive		31				0	31	0					
TCGA-AB	TCGA-AB	LAML	67	MALE	WHITE				2007	-24714	Dead			580			1	580						
TCGA-AB	TCGA-AB	LAML	58	MALE	WHITE				2008	-21336	Alive		699				0	699	0					
TCGA-AB	TCGA-AB	LAML	34	FEMALE	WHITE				2008	-12693	Alive		306				0	306	0					
TCGA-AB	TCGA-AB	LAML	60	FEMALE	WHITE				2007	-22188	Dead			31			1	31						
TCGA-AB	TCGA-AB	LAML	55	FEMALE	WHITE				2006	-20211	Alive		1492				0	1492	0					
TCGA-AB	TCGA-AB	LAML	56	FEMALE	WHITE				2007	-20484	Dead			489			1	489						
TCGA-AB	TCGA-AB	LAML	61	MALE	WHITE				2007	-22523	Dead			183			1	183						
TCGA-AB	TCGA-AB	LAML	31	MALE	WHITE				2007	-11626	Alive		609				0	609	0					
TCGA-AB	TCGA-AB	LAML	71	MALE	WHITE				2007	-26114	Dead			489			1	489						
TCGA-AB	TCGA-AB	LAML	56	MALE	WHITE				2002	-20485	Dead			1642			1	1642						
TCGA-AB	TCGA-AB	LAML	58	FEMALE	WHITE				2006	-21184	Alive		1277				0	1277	0					
TCGA-AB	TCGA-AB	LAML	60	MALE	WHITE				2006	-22006	Dead			335			1	335						
TCGA-AB	TCGA-AB	LAML	57	FEMALE	WHITE				2007	-20880	Dead			854			1	854						
TCGA-AB	TCGA-AB	LAML	58	MALE	WHITE				2007	-21214	Alive		1035				0	1035	0					
TCGA-AB	TCGA-AB	LAML	79	MALE	WHITE				2007	-28975	Dead			458			1	458						
TCGA-AB	TCGA-AB	LAML	55	MALE	WHITE				2006	-20331	Alive		1127				0	1127	0					
TCGA-AB	TCGA-AB	LAML	34	FEMALE	WHITE				2007	-12569	Dead			305			1	305						
TCGA-AB	TCGA-AB	LAML	76	FEMALE	WHITE				2007	-27790	Dead			792			1	792						
TCGA-AB	TCGA-AB	LAML	82	FEMALE	WHITE				2007	-30134	Dead			30			1	30						
TCGA-AB	TCGA-AB	LAML	68	FEMALE	WHITE				2007	-24837	Dead			609			1	609						
TCGA-AB	TCGA-AB	LAML	67	FEMALE	WHITE				2007	-24715	Dead			92			1	92						
TCGA-AB	TCGA-AB	LAML	54	MALE	BLACK OR AFRICAN AMERICAN				2007	-20057	Dead						1							
TCGA-AB	TCGA-AB	LAML	53	MALE	WHITE				2007	-19570	Dead			915			1	915						
TCGA-AB	TCGA-AB	LAML	71	FEMALE	WHITE				2008	-26053	Dead			31			1	31						
TCGA-AB	TCGA-AB	LAML	61	FEMALE	WHITE				2008	-22402	Alive		699				0	699	0					
TCGA-AB	TCGA-AB	LAML	30	FEMALE	WHITE				2008	-11262	Alive		671				0	671	0					
TCGA-AB	TCGA-AB	LAML	50	MALE	WHITE				2008	-18506	Alive		699				0	699	0					
TCGA-AB	TCGA-AB	LAML	35	FEMALE	WHITE				2008	-13027	Alive		487				0	487	0					
TCGA-AB	TCGA-AB	LAML	29	FEMALE					2007	-10807	Alive		150				0	150	0					
TCGA-AB	TCGA-AB	LAML	45	MALE	WHITE				2006	-16589	Dead			335			1	335						
TCGA-AB	TCGA-AB	LAML	38	MALE	WHITE				2006	-14123	Alive		1157				0	1157	0					
TCGA-AB	TCGA-AB	LAML	81	FEMALE	WHITE				2008	-29890	Dead			273			1	273						
TCGA-AB	TCGA-AB	LAML	31	FEMALE	WHITE				2008	-11596	Dead			212			1	212						
TCGA-AB	TCGA-AB	LAML	75	FEMALE	WHITE				2008	-27667	Dead			184			1	184						
TCGA-AB	TCGA-AB	LAML	67	FEMALE	WHITE				2008	-24717	Dead			30			1	30						
TCGA-AB	TCGA-AB	LAML	29	MALE	WHITE				2008	-10774	Dead			365			1	365						
TCGA-AB	TCGA-AB	LAML	51	MALE	WHITE				2008	-18962	Alive		457				0	457	0					
TCGA-AB	TCGA-AB	LAML	40	FEMALE	WHITE				2004	-14885	Alive		1826				0	1826	0					
TCGA-AB	TCGA-AB	LAML	32	FEMALE	WHITE				2003	-11839	Dead			1706			1	1706						
TCGA-AB	TCGA-AB	LAML	57	FEMALE	WHITE				2004	-21124	Dead			761			1	761						
TCGA-AB	TCGA-AB	LAML	25	MALE	WHITE				2005	-9436	Alive		1798				0	1798	0					
TCGA-AB	TCGA-AB	LAML	63	MALE	WHITE				2005	-23164	Alive		1551				0	1551	0					
TCGA-AB	TCGA-AB	LAML	74	MALE	WHITE				2005	-27364	Alive		1581				0	1581	0					
TCGA-AB	TCGA-AB	LAML	25	FEMALE	WHITE				2006	-9162	Dead						1							
TCGA-AB	TCGA-AB	LAML	68	FEMALE	WHITE				2008	-24837	Dead			31			1	31						
TCGA-AB	TCGA-AB	LAML	62	MALE	WHITE				2004	-22980	Alive		1735				0	1735	0					
TCGA-AB	TCGA-AB	LAML	25	MALE	WHITE				2006	-9251	Alive		1249				0	1249	0					
TCGA-AB	TCGA-AB	LAML	31	FEMALE	WHITE				2005	-11446	Alive		1581				0	1581	0					
TCGA-AB	TCGA-AB	LAML	68	MALE	WHITE				2005	-25171	Dead			1431			1	1431						
TCGA-AB	TCGA-AB	LAML	45	MALE	WHITE				2003	-16528	Alive		2100				0	2100	0					
TCGA-AB	TCGA-AB	LAML	61	MALE	WHITE				2006	-22464	Dead			485			1	485						
TCGA-AB	TCGA-AB	LAML	35	MALE	WHITE				2005	-13029	Alive		1581				0	1581	0					
TCGA-AB	TCGA-AB	LAML	22	MALE	WHITE				2005	-8159	Dead			822			1	822						
TCGA-AB	TCGA-AB	LAML	23	MALE					2005	-8676	Dead			577			1	577						
TCGA-AB	TCGA-AB	LAML	21	FEMALE	WHITE				2005	-7916	Alive		1885				0	1885	0					
TCGA-AB	TCGA-AB	LAML	53	MALE	WHITE				2004	-19543	Alive		1887				0	1887	0					
TCGA-CS	TCGA-CS	LGG	31	FEMALE	WHITE		Astrocyto	G2	2005	-11509	Alive	WITH TUM	3574			Stable Disease	0	3574	0	3574			0	3574
TCGA-CS	TCGA-CS	LGG	67	MALE	WHITE		Astrocyto	G3	2005	-24578	Dead	WITH TUMOR		234	9	Stable Disease	1	234	1	234			1	9
TCGA-CS	TCGA-CS	LGG	44	FEMALE	BLACK OR AFRICAN AMERICAN		Astrocyto	G3	2006	-16297	Dead	WITH TUMOR		1335	1184	Progressive Disease	1	1335	1	1335			1	1184
TCGA-CS	TCGA-CS	LGG	37	MALE	WHITE		Astrocyto	G3	2009	-13565	Dead	WITH TUMOR		1106			1	1106	1	1106			1	1106
TCGA-CS	TCGA-CS	LGG	50	MALE	WHITE		Astrocyto	G2	2010	-18494	Alive	WITH TUM	1828			Stable Disease	0	1828	0	1828			0	1828
TCGA-CS	TCGA-CS	LGG	47	FEMALE	WHITE		Oligoden	G2	2005	-17460	Alive	TUMOR FREE				Complete Remission/Response	0		0		0		0	
TCGA-CS	TCGA-CS	LGG	39	MALE	WHITE		Astrocyto	G3	2008	-14418	Alive	TUMOR F	1222			Complete Remission/Response	0	1222	0	1222	0	1222	0	1222
TCGA-CS	TCGA-CS	LGG	40	MALE	WHITE		Astrocyto	G3	2005	-14920	Alive		8			[Unknown]	0	8	0	8			0	8
TCGA-CS	TCGA-CS	LGG	43	MALE	BLACK OR AFRICAN AMERICAN		Oligoden	G2	2007	-15848	Dead	WITH TUMOR		639	287	Progressive Disease	1	639	1	639			1	287
TCGA-CS	TCGA-CS	LGG	53	FEMALE	WHITE		Oligoden	G3	2010	-19399	Alive	WITH TUM	1631			Partial Remission/Response	0	1631	0	1631			0	1631
TCGA-CS	TCGA-CS	LGG	54	FEMALE	WHITE		Astrocyto	G3	2010	-19866	Dead	WITH TUMOR		194		Progressive Disease	1	194	1	194			1	194
TCGA-CS	TCGA-CS	LGG	58	MALE	WHITE		Oligoastr	G3	2009	-21321	Dead	WITH TUMOR		538	188	Progressive Disease	1	538	1	538			1	188
TCGA-CS	TCGA-CS	LGG	48	MALE	WHITE		Astrocyto	G3	2009	-17729	Dead	WITH TUMOR		814	647	Progressive Disease	1	814	1	814			1	647
TCGA-CS	TCGA-CS	LGG	31	MALE			Astrocyto	G3	2009	-11666	Dead			1137		Stable Disease	1	1137		1137			0	1137
TCGA-CS	TCGA-CS	LGG	51	FEMALE	WHITE		Astrocyto	G3	2010	-18977	Alive	WITH TUM	1568			Stable Disease	0	1568	0	1568			0	1568
TCGA-CS	TCGA-CS	LGG	22	MALE	WHITE		Astrocyto	G3	2010	-8340	Alive	TUMOR F	1428			Stable Disease	0	1428	0	1428			0	1428
TCGA-CS	TCGA-CS	LGG	39	FEMALE	WHITE		Astrocyto	G2	2010	-14375	Alive	WITH TUM	1469			Stable Disease	0	1469	0	1469			0	1469
TCGA-CS	TCGA-CS	LGG	57	FEMALE	WHITE		Oligoden	G2	2011	-20836	Alive	WITH TUM	1519			Stable Disease	0	1519	0	1519			0	1519
TCGA-CS	TCGA-CS	LGG	26	FEMALE	WHITE		Oligoden	G2	2011	-9809	Alive	WITH TUM	1494			Stable Disease	0	1494	0	1494			0	1494
TCGA-CS	TCGA-CS	LGG	43	MALE	WHITE		Oligoden	G3	2011	-16066	Alive	WITH TUM	1426			[Discrepancy]	0	1426	0	1426			0	1426
TCGA-DB	TCGA-DB	LGG	38	FEMALE	WHITE		Oligoastr	G3	2002	-13904	Alive	TUMOR F	3733			Complete Remission/Response	0	3733	0	3733	0	3733	0	3733
TCGA-DB	TCGA-DB	LGG	33	MALE	WHITE		Astrocyto	G3	2006	-12152	Alive	TUMOR F	2493			Complete Remission/Response	0	2493	0	2493	0	2493	0	2493
TCGA-DB	TCGA-DB	LGG	37	FEMALE	WHITE		Oligoastr	G3	2006	-13680	Alive	TUMOR F	2289			Complete Remission/Response	0	2289	0	2289	0	2289	0	2289
TCGA-DB	TCGA-DB	LGG	36	MALE	WHITE		Oligoastr	G3	2006	-13371	Alive	TUMOR F	1458			Complete Remission/Response	0	1458	0	1458	0	1458	0	1458
TCGA-DB	TCGA-DB	LGG	32	MALE	WHITE		Oligoastr	G3	2007	-11738	Alive	TUMOR F	2218			Complete Remission/Response	0	2218	0	2218	0	2218	0	2218
TCGA-DB	TCGA-DB	LGG	34	MALE	WHITE		Astrocyto	G3	2007	-12725	Dead	WITH TUMOR		1547	1156	Complete Remission/Response	1	1547	1	1547	1	1156	1	1156
TCGA-DB	TCGA-DB	LGG	17	MALE	WHITE		Oligoden	G2	2007	-6480	Alive	WITH TUM	2772		507	Stable Disease	0	2772	0	2772			1	507
TCGA-DB	TCGA-DB	LGG	59	MALE			Oligoden	G2	2007	-21908	Alive	TUMOR F	1354		695	Complete Remission/Response	0	1354	0	1354	1	695	1	695
TCGA-DB	TCGA-DB	LGG	43	MALE	WHITE		Oligoastr	G2	2007	-15836	Alive	TUMOR F	1112			Complete Remission/Response	0	1112	0	1112	0	1112	0	1112
TCGA-DB	TCGA-DB	LGG	61	MALE	WHITE		Oligoastr	G3	2006	-22342	Alive	WITH TUM	2381			Stable Disease	0	2381	0	2381			0	2381
TCGA-DB	TCGA-DB	LGG	33	FEMALE	WHITE		Oligoastr	G2	2010	-12131	Alive	TUMOR F	1412		501	Complete Remission/Response	0	1412	0	1412	1	501	1	501
TCGA-DB	TCGA-DB	LGG	30	MALE	WHITE		Oligoastr	G2	2012	-11195	Alive	WITH TUM	573			Stable Disease	0	573	0	573			0	573
TCGA-DB	TCGA-DB	LGG	38	MALE	WHITE		Astrocyto	G3	2011	-13990	Alive	TUMOR F	919			Stable Disease	0	919	0	919			0	919
TCGA-DB	TCGA-DB	LGG	26	MALE	WHITE		Oligoastr	G2	2010	-9705	Alive	TUMOR F	1421			Partial Remission/Response	0	1421	0	1421			0	1421
TCGA-DB	TCGA-DB	LGG	32	MALE	WHITE		Astrocyto	G3	2010	-11741	Alive	TUMOR F	1210			Complete Remission/Response	0	1210	0	1210	0	1210	0	1210
TCGA-DB	TCGA-DB	LGG	27	FEMALE	WHITE		Oligoastr	G3	2011	-10150	Alive	TUMOR F	1139			Complete Remission/Response	0	1139	0	1139	0	1139	0	1139
TCGA-DB	TCGA-DB	LGG	41	FEMALE	WHITE		Astrocyto	G3	2011	-15066	Alive	TUMOR F	1021		468	Stable Disease	0	1021	0	1021			1	468
TCGA-DB	TCGA-DB	LGG	34	MALE	WHITE		Oligoden	G3	2006	-12550	Alive	WITH TUM	2219			Stable Disease	0	2219	0	2219			0	2219
TCGA-DB	TCGA-DB	LGG	53	FEMALE	WHITE		Oligoastr	G2	2011	-19710	Alive	TUMOR F	962			Stable Disease	0	962	0	962			0	962
TCGA-DB	TCGA-DB	LGG	67	FEMALE	WHITE		Oligoden	G2	2012	-24690	Alive	WITH TUM	736		724	Complete Remission/Response	0	736	0	736	1	724	1	724
TCGA-DB	TCGA-DB	LGG	59	MALE	WHITE		Oligoastr	G2	2011	-21811	Dead	WITH TUMOR		775	430	Stable Disease	1	775	1	775			1	430
TCGA-DB	TCGA-DB	LGG	40	MALE	WHITE		Oligoden	G3	2012	-14936	Alive	TUMOR F	916			Complete Remission/Response	0	916	0	916	0	916	0	916
TCGA-DB	TCGA-DB	LGG	31	FEMALE	[Unknown]		Oligoastr	G2	2012	-11637	Alive	TUMOR F	182			Stable Disease	0	182	0	182			0	182
TCGA-DB	TCGA-DB	LGG	24	FEMALE	[Not Evaluated]		Oligoden	G2	2012	-8947	Alive	TUMOR F	955			Complete Remission/Response	0	955	0	955	0	955	0	955
TCGA-DB	TCGA-DB	LGG	20	MALE	WHITE		Oligoastr	G2	2012	-7389	Alive	TUMOR F	835			Complete Remission/Response	0	835	0	835	0	835	0	835
TCGA-DB	TCGA-DB	LGG	38	FEMALE	WHITE		Oligoastr	G2	2011	-14019	Alive	TUMOR F	846			Complete Remission/Response	0	846	0	846	0	846	0	846
TCGA-DB	TCGA-DB	LGG	54	MALE	WHITE		Oligoden	G2	2012	-19950	Alive	TUMOR F	889			Stable Disease	0	889	0	889			0	889
TCGA-DB	TCGA-DB	LGG	65	FEMALE	WHITE		Oligoastr	G3	2012	-23936	Dead	TUMOR FREE		438		Stable Disease	1	438	0	438			0	438
TCGA-DB	TCGA-DB	LGG	56	FEMALE	WHITE		Astrocyto	G3	2012	-20625	Alive	TUMOR F	563			Stable Disease	0	563	0	563			0	563
TCGA-DB	TCGA-DB	LGG	55	FEMALE	WHITE		Oligoastr	G3	2013	-20241	Alive	TUMOR F	368			Complete Remission/Response	0	368	0	368	0	368	0	368
TCGA-DB	TCGA-DB	LGG	36	FEMALE	WHITE		Astrocyto	G3	2013	-13338	Alive	TUMOR F	342			Complete Remission/Response	0	342	0	342	0	342	0	342
TCGA-DB	TCGA-DB	LGG	47	MALE	WHITE		Astrocyto	G2	2013	-17289	Alive	TUMOR F	544			Complete Remission/Response	0	544	0	544	0	544	0	544
TCGA-DB	TCGA-DB	LGG	29	MALE	WHITE		Astrocyto	G3	2010	-10812	Alive		935			Partial Remission/Response	0	935	0	935			0	935
TCGA-DB	TCGA-DB	LGG	25	FEMALE	[Unknown]		Astrocyto	G2	2013	-9413	Alive	TUMOR F	492			Complete Remission/Response	0	492	0	492	0	492	0	492
TCGA-DH	TCGA-DH	LGG	38	FEMALE	WHITE		Oligoastr	G3	2007	-13943	Dead	WITH TUMOR		607		Progressive Disease	1	607	1	607			1	607
TCGA-DH	TCGA-DH	LGG	32	MALE	WHITE		Oligoden	G3	2005	-11846	Alive		968			Stable Disease	0	968	0	968			0	968
TCGA-DH	TCGA-DH	LGG	29	MALE	WHITE		Astrocyto	G3	2008	-10678	Alive	WITH TUM	1943		1627	Stable Disease	0	1943	0	1943			1	1627
TCGA-DH	TCGA-DH	LGG	30	MALE	WHITE		Oligoastr	G3	2009	-11291	Alive	WITH TUM	1401			Stable Disease	0	1401	0	1401			0	1401
TCGA-DH	TCGA-DH	LGG	56	FEMALE	WHITE		Oligoden	G3	2009	-20558	Alive	TUMOR F	748			Complete Remission/Response	0	748	0	748	0	748	0	748
TCGA-DH	TCGA-DH	LGG	70	MALE	WHITE		Oligoden	G3	2007	-25809	Dead	TUMOR FREE		919	260	Stable Disease	1	919	0	919			1	260
TCGA-DH	TCGA-DH	LGG	70	MALE	WHITE		Oligoden	G3	2007	-25809	Dead	TUMOR FREE		919	260	Stable Disease	1	919	0	919			1	260
TCGA-DH	TCGA-DH	LGG	52	MALE	WHITE		Astrocyto	G3	2010	-19156	Alive	WITH TUM	1279			Stable Disease	0	1279	0	1279			0	1279
TCGA-DH	TCGA-DH	LGG	43	FEMALE	WHITE		Astrocyto	G3	2012	-15866	Alive	WITH TUM	846			Stable Disease	0	846	0	846			0	846
TCGA-DH	TCGA-DH	LGG	49	MALE	WHITE		Oligoden	G2	2012	-18193	Alive	TUMOR F	523			Complete Remission/Response	0	523	0	523	0	523	0	523
TCGA-DH	TCGA-DH	LGG	49	FEMALE	WHITE		Oligoden	G3	2013	-18199	Alive	WITH TUM	523			Stable Disease	0	523	0	523			0	523
TCGA-DH	TCGA-DH	LGG	59	FEMALE	WHITE		Oligoden	G3	2008	-21803	Alive	TUMOR F	1650			Complete Remission/Response	0	1650	0	1650	0	1650	0	1650
TCGA-DH	TCGA-DH	LGG	50	MALE	WHITE		Oligoden	G2	2013	-18429	Alive	WITH TUM	706			Stable Disease	0	706	0	706			0	706
TCGA-DH	TCGA-DH	LGG	30	MALE	WHITE		Astrocyto	G3	2013	-11014	Dead	WITH TUMOR		531	404	Stable Disease	1	531	1	531			1	404
TCGA-DH	TCGA-DH	LGG	43	MALE	WHITE		Astrocyto	G3	2013	-16037	Alive	WITH TUM	417			Stable Disease	0	417	0	417			0	417
TCGA-DH	TCGA-DH	LGG	49	MALE	WHITE		Astrocyto	G3	2013	-18161	Alive	WITH TUM	566			Stable Disease	0	566	0	566			0	566
TCGA-DU	TCGA-DU	LGG	34	FEMALE	WHITE		Astrocyto	G3	2009	-12606	Alive	WITH TUM	548			Stable Disease	0	548	0	548			0	548
TCGA-DU	TCGA-DU	LGG	48	MALE	WHITE		Oligoden	G2	2010	-17663	Alive	TUMOR F	443			Stable Disease	0	443	0	443			0	443
TCGA-DU	TCGA-DU	LGG	40	FEMALE	WHITE		Oligoastr	G3	2010	-14802	Alive	WITH TUM	531			Stable Disease	0	531	0	531			0	531
TCGA-DU	TCGA-DU	LGG	61	FEMALE	WHITE		Oligoastr	G3	2010	-22348	Dead	WITH TUMOR		205	24	Progressive Disease	1	205	1	205			1	24
TCGA-DU	TCGA-DU	LGG	29	MALE	WHITE		Oligoastr	G2	2010	-10624	Alive	WITH TUM	407			Stable Disease	0	407	0	407			0	407
TCGA-DU	TCGA-DU	LGG	57	FEMALE	WHITE		Astrocyto	G3	2010	-21106	Alive	WITH TUM	257		202	Progressive Disease	0	257	0	257			1	202
TCGA-DU	TCGA-DU	LGG	49	FEMALE	WHITE		Oligoastr	G3	2010	-18234	Alive	WITH TUM	207			Stable Disease	0	207	0	207			0	207
TCGA-DU	TCGA-DU	LGG	34	FEMALE	WHITE		Oligoden	G2	1995	-12764	Alive	WITH TUM	5546		4347	Progressive Disease	0	5546	0	5546			1	4347
TCGA-DU	TCGA-DU	LGG	34	FEMALE	WHITE		Oligoden	G2	1995	-12764	Alive	WITH TUM	5546		4347	Progressive Disease	0	5546	0	5546			1	4347
TCGA-DU	TCGA-DU	LGG	37	FEMALE	WHITE		Oligoastr	G2	2009	-13673	Alive	TUMOR F	576			Stable Disease	0	576	0	576			0	576
TCGA-DU	TCGA-DU	LGG	43	FEMALE	BLACK OR AFRICAN AMERICAN		Oligoden	G2	2009	-15986	Alive	WITH TUM	532		265	Progressive Disease	0	532	0	532			1	265
TCGA-DU	TCGA-DU	LGG	43	FEMALE	BLACK OR AFRICAN AMERICAN		Oligoastr	G2	2009	-15986	Alive	WITH TUM	532		265	Progressive Disease	0	532	0	532			1	265
TCGA-DU	TCGA-DU	LGG	62	FEMALE	WHITE		Oligoden	G2	2010	-22847	Alive	TUMOR F	461			Stable Disease	0	461	0	461			0	461
TCGA-DU	TCGA-DU	LGG	35	FEMALE	WHITE		Astrocyto	G3	1993	-12836	Alive	TUMOR F	6423		950	Progressive Disease	0	6423	0	6423			1	950
TCGA-DU	TCGA-DU	LGG	66	MALE	WHITE		Oligoden	G3	1993	-24282	Dead			1585			1	1585		1585			0	1585
TCGA-DU	TCGA-DU	LGG	53	MALE	WHITE		Oligoden	G3	1994	-19541	Dead	WITH TUMOR		682	353	Progressive Disease	1	682	1	682			1	353
TCGA-DU	TCGA-DU	LGG	31	MALE	WHITE		Oligoastr	G2	1994	-11584	Dead	WITH TUMOR		1491	1197	Progressive Disease	1	1491	1	1491			1	1197

TCGA-DU	TCGA-DU	LGG	31	FEMALE	WHITE	Oligoastr	G3	1996	-11427	Dead	WITH TUMOR		2286	106	Progressive Disease	1	2286	1	2286			1	106
TCGA-DU	TCGA-DU	LGG	45	MALE	WHITE	Oligoden	G3	1997	-16695	Dead	WITH TUMOR		1401	837	Progressive Disease	1	1401	1	1401			1	837
TCGA-DU	TCGA-DU	LGG	45	MALE	WHITE	Oligoden	G3	1997	-16695	Dead	WITH TUMOR		1401	837	Progressive Disease	1	1401	1	1401			1	837
TCGA-DU	TCGA-DU	LGG	54	MALE	WHITE	Oligoden	G2	1998	-19906	Dead	WITH TUMOR		2000	1629	Progressive Disease	1	2000	1	2000			1	1629
TCGA-DU	TCGA-DU	LGG	66	FEMALE	WHITE	Oligoden	G2	1998	-24122	Dead	WITH TUMOR		37		Progressive Disease	1	37	1	37			1	37
TCGA-DU	TCGA-DU	LGG	31	FEMALE	WHITE	Oligoden	G2	1998	-11595	Dead	WITH TUMOR		2660	1886	Progressive Disease	1	2660	1	2660			1	1886
TCGA-DU	TCGA-DU	LGG	52	MALE	WHITE	Astrocyto	G3	1998	-19207	Dead	WITH TUMOR		214	103	Progressive Disease	1	214	1	214			1	103
TCGA-DU	TCGA-DU	LGG	60	FEMALE	WHITE	Oligoastr	G3	1999	-22031	Dead	WITH TUMOR		354		Progressive Disease	1	354	1	354			1	354
TCGA-DU	TCGA-DU	LGG	24	FEMALE	WHITE	Oligoden	G3	2000	-9021	Dead	WITH TUMOR		4068	1154	Progressive Disease	1	4068	1	4068			1	1154
TCGA-DU	TCGA-DU	LGG	24	FEMALE	WHITE	Oligoden	G3	2000	-9021	Dead	WITH TUMOR		4068	1154	Progressive Disease	1	4068	1	4068			1	1154
TCGA-DU	TCGA-DU	LGG	51	FEMALE	WHITE	Astrocyto	G3	2000	-18686	Dead	WITH TUMOR		605	486	Progressive Disease	1	605	1	605			1	486
TCGA-DU	TCGA-DU	LGG	59	FEMALE	WHITE	Oligoastr	G3	2001	-21634	Dead			512			1	512		512			0	512
TCGA-DU	TCGA-DU	LGG	35	FEMALE	WHITE	Oligoden	G2	2001	-13021	Dead	WITH TUMOR		2875	2397	Progressive Disease	1	2875	1	2875			1	2397
TCGA-DU	TCGA-DU	LGG	35	FEMALE	WHITE	Oligoden	G2	2001	-13021	Dead	WITH TUMOR		2875	2397	Progressive Disease	1	2875	1	2875			1	2397
TCGA-DU	TCGA-DU	LGG	23	FEMALE	WHITE	Oligoden	G3	1998	-8752	Dead			3470	2097	Progressive Disease	1	3470		3470			1	2097
TCGA-DU	TCGA-DU	LGG	56	MALE	WHITE	Oligoden	G3	2010	-20567	Alive	WITH TUM	242				0	242	0	242			0	242
TCGA-DU	TCGA-DU	LGG	25	MALE	WHITE	Oligoastr	G3	2011	-9380	Alive	WITH TUM	73			Stable Disease	0	73	0	73			0	73
TCGA-DU	TCGA-DU	LGG	60	FEMALE	WHITE	Astrocyto	G3	1995	-21990	Dead	WITH TUMOR		349		Progressive Disease	1	349	1	349			1	349
TCGA-DU	TCGA-DU	LGG	33	MALE	WHITE	Astrocyto	G2	1997	-12245	Dead			1915			1	1915		1915			0	1915
TCGA-DU	TCGA-DU	LGG	41	FEMALE	WHITE	Oligoden	G2	1998	-15289	Alive	TUMOR F	4752		330	Progressive Disease	0	4752	0	4752			1	330
TCGA-DU	TCGA-DU	LGG	32	FEMALE	WHITE	Oligoden	G2	2000	-11854	Dead	WITH TUMOR		4695	2960	Progressive Disease	1	4695	1	4695			1	2960
TCGA-DU	TCGA-DU	LGG	58	FEMALE	WHITE	Astrocyto	G3	2001	-21199	Dead	WITH TUMOR		456	193	Progressive Disease	1	456	1	456			1	193
TCGA-DU	TCGA-DU	LGG	25	MALE	WHITE	Oligoastr	G2	2000	-9322	Dead	WITH TUMOR		3200		Stable Disease	1	3200	1	3200			1	3200
TCGA-DU	TCGA-DU	LGG	74	FEMALE	BLACK OR AFRICAN AMERICAN	Astrocyto	G3	2001	-27039	Dead	WITH TUMOR		199		Progressive Disease	1	199	1	199			1	199
TCGA-DU	TCGA-DU	LGG	59	MALE	WHITE	Astrocyto	G3	2001	-21824	Dead	WITH TUMOR		269	187	Progressive Disease	1	269	1	269			1	187
TCGA-DU	TCGA-DU	LGG	59	MALE	WHITE	Oligoden	G2	2001	-21865	Dead	WITH TUMOR		3571	3437	Progressive Disease	1	3571	1	3571			1	3437
TCGA-DU	TCGA-DU	LGG	41	FEMALE	WHITE	Oligoden	G2	2004	-15041	Alive	WITH TUM	2761		591	Progressive Disease	0	2761	0	2761			1	591
TCGA-DU	TCGA-DU	LGG	57	FEMALE	WHITE	Oligoden	G3	2006	-21019	Dead	WITH TUMOR		933	338	Progressive Disease	1	933	1	933			1	338
TCGA-DU	TCGA-DU	LGG	39	MALE	WHITE	Oligoastr	[Discrepa	2009	-14279	Alive	TUMOR F	800			Stable Disease	0	800	0	800			0	800
TCGA-DU	TCGA-DU	LGG	45	FEMALE	WHITE	Astrocyto	G3	1995	-16667	Dead	WITH TUMOR		315			1	315	1	315			1	315
TCGA-DU	TCGA-DU	LGG	69	MALE	WHITE	Astrocyto	G3	1997	-25266	Dead	WITH TUMOR		242	91	Progressive Disease	1	242	1	242			1	91
TCGA-DU	TCGA-DU	LGG	53	FEMALE	WHITE	Oligoden	G2	2003	-19616	Alive	TUMOR F	2869			Stable Disease	0	2869	0	2869			0	2869
TCGA-DU	TCGA-DU	LGG	38	FEMALE	BLACK OR AFRICAN AMERICAN	Astrocyto	G3	2006	-14205	Dead	WITH TUMOR		576	200	Progressive Disease	1	576	1	576			1	200
TCGA-DU	TCGA-DU	LGG	33	MALE	WHITE	Astrocyto	G3	2006	-12266	Dead	WITH TUMOR		1339	675	Progressive Disease	1	1339	1	1339			1	675
TCGA-DU	TCGA-DU	LGG	53	FEMALE	WHITE	Oligoden	G3	2006	-19610	Dead	WITH TUMOR		1886	317	Progressive Disease	1	1886	1	1886			1	317
TCGA-DU	TCGA-DU	LGG	53	MALE	WHITE	Oligoden	G2	2006	-19644	Dead	WITH TUMOR		788	366	Progressive Disease	1	788	1	788			1	366
TCGA-DU	TCGA-DU	LGG	48	FEMALE	WHITE	Oligoden	G3	2006	-17656	Alive	WITH TUM	1834		1374	Progressive Disease	0	1834	0	1834			1	1374
TCGA-DU	TCGA-DU	LGG	43	MALE	WHITE	Oligoastr	G3	2008	-15931	Dead	WITH TUMOR		709	309	Progressive Disease	1	709	1	709			1	309
TCGA-DU	TCGA-DU	LGG	43	MALE	WHITE	Oligoastr	G3	2008	-15931	Dead	WITH TUMOR		709	309	Progressive Disease	1	709	1	709			1	309
TCGA-DU	TCGA-DU	LGG	67	MALE	WHITE	Oligoastr	G2	2008	-24800	Alive	WITH TUM	1277		1250	Progressive Disease	0	1277	0	1277			1	1250
TCGA-DU	TCGA-DU	LGG	41	FEMALE	WHITE	Oligoden	G3	2011	-15197	Alive	WITH TUM	84			Stable Disease	0	84	0	84			0	84
TCGA-DU	TCGA-DU	LGG	57	FEMALE	WHITE	Astrocyto	G3	2001	-21129	Dead	WITH TUMOR		155			1	155	1	155			1	155
TCGA-DU	TCGA-DU	LGG	63	FEMALE	WHITE	Oligoastr	G3	2009	-23064	Dead	WITH TUMOR		722	111	Progressive Disease	1	722	1	722			1	111
TCGA-DU	TCGA-DU	LGG	61	FEMALE	WHITE	Oligoastr	G3	2011	-22596	Dead	WITH TUMOR		444	402	Progressive Disease	1	444	1	444			1	402
TCGA-DU	TCGA-DU	LGG	29	MALE	WHITE	Oligoastr	G3	2011	-10824	Alive	WITH TUM	629		509	Progressive Disease	0	629	0	629			1	509
TCGA-DU	TCGA-DU	LGG	51	MALE	WHITE	Oligoden	G2	2011	-18735	Alive	TUMOR F	651			Stable Disease	0	651	0	651			0	651
TCGA-DU	TCGA-DU	LGG	60	FEMALE	WHITE	Oligoden	G3	2011	-21964	Alive	TUMOR F	582			Stable Disease	0	582	0	582			0	582
TCGA-DU	TCGA-DU	LGG	29	FEMALE	WHITE	Oligoastr	G2	2012	-10641	Alive	WITH TUM	516		193	Progressive Disease	0	516	0	516			1	193
TCGA-DU	TCGA-DU	LGG	69	FEMALE	WHITE	Oligoastr	G2	2012	-25262	Alive	TUMOR F	471			Stable Disease	0	471	0	471			0	471
TCGA-DU	TCGA-DU	LGG	55	FEMALE	WHITE	Oligoden	G3	2012	-20136	Alive	TUMOR F	431			Stable Disease	0	431	0	431			0	431
TCGA-DU	TCGA-DU	LGG	33	MALE	WHITE	Astrocyto	G3	2012	-12111	Alive	TUMOR F	1012		203	[Discrepancy]	0	1012	0	1012			1	203
TCGA-DU	TCGA-DU	LGG	51	MALE	WHITE	Oligoastr	G2	2012	-18791	Alive	TUMOR F	908		848	[Discrepancy]	0	908	0	908			1	848
TCGA-DU	TCGA-DU	LGG	42	MALE	BLACK OR AFRICAN AMERICAN	Oligoden	G2	2012	-15650	Alive	TUMOR F	964		551	Stable Disease	0	964	0	964			1	551
TCGA-DU	TCGA-DU	LGG	70	MALE	WHITE	Oligoden	G3	2013	-25726	Alive	WITH TUM	743		584	[Discrepancy]	0	743	0	743			1	584
TCGA-DU	TCGA-DU	LGG	62	FEMALE	WHITE	Astrocyto	G2	2013	-22811	Alive	WITH TUM	796		186	Progressive Disease	0	796	0	796			1	186
TCGA-DU	TCGA-DU	LGG	33	FEMALE	BLACK OR AFRICAN AMERICAN	Astrocyto	G3	2012	-12107	Alive	WITH TUM	760			Progressive Disease	0	760	0	760			0	760
TCGA-DU	TCGA-DU	LGG	46	FEMALE	WHITE	Astrocyto	G3	2012	-16945	Dead	WITH TUMOR		1033	526	[Discrepancy]	1	1033	1	1033			1	526
TCGA-DU	TCGA-DU	LGG	37	FEMALE	BLACK OR AFRICAN AMERICAN	Oligoden	G2	2013	-13597	Alive	TUMOR F	777			Stable Disease	0	777	0	777			0	777
TCGA-DU	TCGA-DU	LGG	60	MALE	WHITE	Oligoden	G2	2013	-22038	Alive	TUMOR F	656			Stable Disease	0	656	0	656			0	656
TCGA-DU	TCGA-DU	LGG	35	FEMALE	WHITE	Oligoastr	G2	2007	-12809	Alive	TUMOR F	2893		2267	[Discrepancy]	0	2893	0	2893			1	2267
TCGA-DU	TCGA-DU	LGG	27	FEMALE	WHITE	Astrocyto	G3	2013	-10170	Alive	TUMOR F	638			Stable Disease	0	638	0	638			0	638
TCGA-DU	TCGA-DU	LGG	74	FEMALE	WHITE	Oligoden	G3	2013	-27084	Alive	WITH TUM	678		641	Progressive Disease	0	678	0	678			1	641
TCGA-DU	TCGA-DU	LGG	87	MALE	WHITE	Oligoden	G2	1999	-31776	Dead	WITH TUMOR		347	203	Progressive Disease	1	347	1	347			1	203
TCGA-DU	TCGA-DU	LGG	54	MALE	WHITE	Oligoden	G3	2000	-19823	Dead	WITH TUMOR		814	410	Progressive Disease	1	814	1	814			1	410
TCGA-DU	TCGA-DU	LGG	30	MALE	WHITE	Astrocyto	G2	2013	-10960	Alive	WITH TUM	522			[Unknown]	0	522	0	522			0	522
TCGA-DU	TCGA-DU	LGG	51	MALE	WHITE	Oligoden	G3	2006	-18939	Dead	WITH TUMOR		648	338	Progressive Disease	1	648	1	648			1	338
TCGA-DU	TCGA-DU	LGG	73	FEMALE	WHITE	Oligoden	G3	2002	-26766	Dead	WITH TUMOR		547		[Unknown]	1	547	1	547			1	547
TCGA-DU	TCGA-DU	LGG	35	MALE	WHITE	Oligoastr	G3	2003	-13108	Dead	WITH TUMOR		4229	3009	Stable Disease	1	4229	1	4229			1	3009
TCGA-DU	TCGA-DU	LGG	32	MALE	WHITE	Oligoden	G2	2007	-11764	Alive	TUMOR F	2565			Stable Disease	0	2565	0	2565			0	2565
TCGA-DU	TCGA-DU	LGG	56	MALE	ASIAN	Oligoden	G2	2010	-20704	Alive	TUMOR F	1567		74	Progressive Disease	0	1567	0	1567			1	74
TCGA-DU	TCGA-DU	LGG	32	MALE	WHITE	Astrocyto	G2	2011	-11884	Alive	WITH TUM	1137		1114	[Discrepancy]	0	1137	0	1137			1	1114
TCGA-DU	TCGA-DU	LGG	52	MALE	WHITE	Oligoastr	G3	2012	-19025	Dead	WITH TUMOR		228	188	Progressive Disease	1	228	1	228			1	188
TCGA-DU	TCGA-DU	LGG	40	MALE	WHITE	Oligoden	G2	2008	-14671	Dead			1351		[Unknown]	1	1351		1351			0	1351
TCGA-DU	TCGA-DU	LGG	32	MALE	WHITE	Astrocyto	G3	1998	-11906	Dead	WITH TUMOR		1183	822	Progressive Disease	1	1183	1	1183			1	822
TCGA-DU	TCGA-DU	LGG	55	MALE	BLACK OR AFRICAN AMERICAN	Astrocyto	G3	2013	-20394	Alive	WITH TUM	17			[Unknown]	0	17	0	17			0	17
TCGA-E1	TCGA-E1	LGG	41	MALE	WHITE	Astrocyto	G3	1998	-15230	Dead	WITH TUMOR		1525	1242	Stable Disease	1	1525	1	1525			1	1242
TCGA-E1	TCGA-E1	LGG	38	MALE	WHITE	Astrocyto	G3	1998	-14212	Dead	WITH TUMOR		2052	156	Progressive Disease	1	2052	1	2052			1	156
TCGA-E1	TCGA-E1	LGG	42	MALE	WHITE	Astrocyto	G3	1999	-15481	Dead	WITH TUMOR		1251	903	Stable Disease	1	1251	1	1251			1	903
TCGA-E1	TCGA-E1	LGG	34	MALE	WHITE	Astrocyto	G3	2000	-12655	Dead	WITH TUMOR		2433	1604	Stable Disease	1	2433	1	2433			1	1604
TCGA-E1	TCGA-E1	LGG	62	FEMALE	WHITE	Astrocyto	G3	2000	-22837	Dead	WITH TUMOR		1762	1452	Stable Disease	1	1762	1	1762			1	1452
TCGA-E1	TCGA-E1	LGG	31	MALE	WHITE	Oligoden	G3	1994	-11332	Dead	WITH TUMOR		4084	2602	Stable Disease	1	4084	1	4084			1	2602
TCGA-E1	TCGA-E1	LGG	42	FEMALE	WHITE	Oligoden	G2	1997	-15372	Dead	WITH TUM	2379	2379	362	Progressive Disease	1	2379	1	2379			1	362
TCGA-E1	TCGA-E1	LGG	48	FEMALE	WHITE	Oligoden	G2	1998	-17612	Dead	WITH TUMOR		2907	923	Stable Disease	1	2907	1	2907			1	923
TCGA-E1	TCGA-E1	LGG	38	FEMALE	WHITE	Oligoastr	G2	1999	-13903	Dead			3978	1085	Stable Disease	1	3978		3978			1	1085
TCGA-E1	TCGA-E1	LGG	57	MALE	WHITE	Astrocyto	G3	1998	-21020	Dead	WITH TUMOR		435	284	Stable Disease	1	435	1	435			1	284
TCGA-E1	TCGA-E1	LGG	32	FEMALE	WHITE	Astrocyto	G3	2001	-11971	Dead	WITH TUMOR		886	188	Stable Disease	1	886	1	886			1	188
TCGA-E1	TCGA-E1	LGG	47	FEMALE	WHITE	Astrocyto	G3	2005	-17465	Dead	WITH TUMOR		2835	689	Stable Disease	1	2835	1	2835			1	689
TCGA-E1	TCGA-E1	LGG	33	FEMALE	WHITE	Astrocyto	G3	2008	-12229	Dead	WITH TUMOR		111	56	[Unknown]	1	111	1	111			1	56
TCGA-E1	TCGA-E1	LGG	55	MALE	WHITE	Astrocyto	G3	2008	-20180	Dead	WITH TUMOR		592	516	Stable Disease	1	592	1	592			1	516
TCGA-E1	TCGA-E1	LGG	52	MALE	WHITE	Astrocyto	G3	2009	-19236	Dead			378		Stable Disease	1	378		378			0	378
TCGA-E1	TCGA-E1	LGG	46	MALE	WHITE	Astrocyto	G3	2009	-16982	Dead	WITH TUMOR		492	91	Progressive Disease	1	492	1	492			1	91
TCGA-E1	TCGA-E1	LGG	63	MALE	WHITE	Astrocyto	G3	2010	-23263	Dead	WITH TUMOR		648	88	Progressive Disease	1	648	1	648			1	88
TCGA-E1	TCGA-E1	LGG	63	FEMALE	WHITE	Astrocyto	G3	2010	-23180	Dead	WITH TUMOR		727	420	Stable Disease	1	727	1	727			1	420
TCGA-E1	TCGA-E1	LGG	45	MALE	WHITE	Oligoden	G3	1995	-16770	Dead	WITH TUMOR		2282	1977	Stable Disease	1	2282	1	2282			1	1977
TCGA-E1	TCGA-E1	LGG	58	FEMALE	BLACK OR AFRICAN AMERICAN	Oligoden	G3	2000	-21341	Dead	WITH TUMOR		1578	580	Stable Disease	1	1578	1	1578			1	580
TCGA-E1	TCGA-E1	LGG	71	MALE	BLACK OR AFRICAN AMERICAN	Oligoden	G3	2007	-26159	Dead	WITH TUMOR		466		Stable Disease	1	466	1	466			1	466
TCGA-E1	TCGA-E1	LGG	42	MALE	WHITE	Oligoastr	G3	1996	-15345	Dead	WITH TUMOR		23		[Unknown]	1	23	1	23			1	23
TCGA-E1	TCGA-E1	LGG	26	FEMALE	WHITE	Oligoastr	G3	2010	-9546	Dead	WITH TUMOR		987	97	Progressive Disease	1	987	1	987			1	97
TCGA-E1	TCGA-E1	LGG	28	MALE	WHITE	Oligoastr	G2	2002	-10571	Dead	WITH TUMOR		1120	409	Progressive Disease	1	1120	1	1120			1	409
TCGA-E1	TCGA-E1	LGG	27	FEMALE	WHITE	Oligoden	G2	1998	-9867	Dead	WITH TUMOR		4445	958	Stable Disease	1	4445	1	4445			1	958
TCGA-E1	TCGA-E1	LGG	58	FEMALE	WHITE	Oligoden	G2	2012	-21341	Dead	WITH TUMOR		398	213	Progressive Disease	1	398	1	398			1	213
TCGA-E1	TCGA-E1	LGG	31	FEMALE	BLACK OR AFRICAN AMERICAN	Astrocyto	G2	1992	-11330	Dead	WITH TUMOR		2235	1569	Progressive Disease	1	2235	1	2235			1	1569
TCGA-E1	TCGA-E1	LGG	35	MALE	WHITE	Astrocyto	G2	1999	-12849	Dead	WITH TUMOR		4412	846	Progressive Disease	1	4412	1	4412			1	846
TCGA-E1	TCGA-E1	LGG	41	FEMALE	WHITE	Astrocyto	G2	2009	-14985	Dead	WITH TUMOR		984	294	Progressive Disease	1	984	1	984			1	294
TCGA-EZ	TCGA-EZ	LGG	47	FEMALE	WHITE	Oligoden	G2	2011	-17432	Alive	WITH TUM	1201			Stable Disease	0	1201	0	1201			0	1201
TCGA-F6	TCGA-F6	LGG	34	MALE	ASIAN	Oligoden	G2	2013	-12578	Alive	TUMOR F	7			Complete Remission/Response	0	7	0	7	0	7	0	7
TCGA-F6	TCGA-F6	LGG	44	MALE	ASIAN	Astrocyto	G2	2013	-16202	Alive	TUMOR F	6			Complete Remission/Response	0	6	0	6	0	6	0	6
TCGA-FG	TCGA-FG	LGG	54	MALE	WHITE	Oligoden	G3	2009	-19818	Alive	WITH TUM	1453			Stable Disease	0	1453	0	1453			0	1453
TCGA-FG	TCGA-FG	LGG	23	MALE	WHITE	Astrocyto	G3	2009	-8571	Dead	WITH TUMOR		775	395	Progressive Disease	1	775	1	775			1	395
TCGA-FG	TCGA-FG	LGG	23	MALE	WHITE	Astrocyto	G3	2009	-8571	Dead	WITH TUMOR		775	395	Progressive Disease	1	775	1	775			1	395
TCGA-FG	TCGA-FG	LGG	62	MALE	WHITE	Oligoden	G2	2010	-22735	Alive	WITH TUM	1588			Stable Disease	0	1588	0	1588			0	1588
TCGA-FG	TCGA-FG	LGG	39	FEMALE	WHITE	Oligoastr	G2	2008	-14249	Dead	WITH TUMOR		1120	715	Stable Disease	1	1120	1	1120			1	715
TCGA-FG	TCGA-FG	LGG	39	FEMALE	WHITE	Oligoastr	G2	2008	-14249	Dead	WITH TUMOR		1120	715	Stable Disease	1	1120	1	1120			1	715
TCGA-FG	TCGA-FG	LGG	59	FEMALE	BLACK OR AFRICAN AMERICAN	Astrocyto	G3	2010	-21745	Alive	WITH TUM	571		405	Progressive Disease	0	571	0	571			1	405
TCGA-FG	TCGA-FG	LGG	30	MALE	WHITE	Astrocyto	G2	2011	-11312	Alive	WITH TUM	454		119	Progressive Disease	0	454	0	454			1	119
TCGA-FG	TCGA-FG	LGG	70	MALE	WHITE	Oligoden	G2	2011	-25667	Alive	WITH TUM	1294			Stable Disease	0	1294	0	1294			0	1294
TCGA-FG	TCGA-FG	LGG	23	FEMALE	WHITE	Astrocyto	G2	2011	-8403	Alive	WITH TUM	1257		913	Complete Remission/Response	0	1257	0	1257	1	913	1	913
TCGA-FG	TCGA-FG	LGG	63	MALE	WHITE	Oligoden	G3	2011	-23374	Dead	WITH TUMOR		561		Stable Disease	1	561	1	561			1	561
TCGA-FG	TCGA-FG	LGG	28	MALE	WHITE	Oligoden	G2	2008	-10476	Alive	WITH TUM	467			Stable Disease	0	467	0	467			0	467
TCGA-FG	TCGA-FG	LGG	48	MALE	WHITE	Astrocyto	G3	2011	-17843	Alive	TUMOR F	544			Complete Remission/Response	0	544	0	544	0	544	0	544
TCGA-FG	TCGA-FG	LGG	49	MALE	WHITE	Oligoastr	G2	2009	-18084	Alive	WITH TUM	1219			Stable Disease	0	1219	0	1219			0	1219
TCGA-FG	TCGA-FG	LGG	31	FEMALE	WHITE	Oligoden	G3	2011	-11528	Alive	WITH TUM	686		407	Stable Disease	0	686	0	686			1	407
TCGA-FG	TCGA-FG	LGG	31	MALE	WHITE	Oligoden	G2	2011	-11357	Alive	TUMOR F	627			Complete Remission/Response	0	627	0	627	0	627	0	627
TCGA-FG	TCGA-FG	LGG	49	FEMALE	WHITE	Oligoastr	G2	2011	-18206	Alive	WITH TUM	611		254	Stable Disease	0	611	0	611			1	254
TCGA-FG	TCGA-FG	LGG	23	MALE	WHITE	Oligoastr	G3	2012	-8642	Alive	WITH TUM	862			Stable Disease	0	862	0	862			0	862
TCGA-FG	TCGA-FG	LGG	35	MALE	WHITE	Oligoden	G2	2012	-12972	Alive	WITH TUM	416			Stable Disease	0	416	0	416			0	416
TCGA-FG	TCGA-FG	LGG	37	MALE	WHITE	Astrocyto	G3	2012	-13621	Alive	TUMOR F	433			Complete Remission/Response	0	433	0	433	0	433	0	433
TCGA-FG	TCGA-FG	LGG	42	FEMALE	WHITE	Oligoastr	G3	2011	-15431	Alive	WITH TUM	487			Stable Disease	0	487	0	487			0	487
TCGA-FG	TCGA-FG	LGG	31	FEMALE	WHITE	Oligoastr	G2	2011	-11684	Alive	TUMOR F	611			Complete Remission/Response	0	611	0	611	0	611	0	611
TCGA-FG	TCGA-FG	LGG	41	MALE	WHITE	Oligoastr	G2	2012	-15051	Alive	WITH TUM	455		384	Stable Disease	0	455	0	455			1	384
TCGA-FG	TCGA-FG	LGG	33	FEMALE	WHITE	Oligoden	G2	2012	-12384	Alive	WITH TUM	685			Stable Disease	0	685	0	685			0	685
TCGA-FG	TCGA-FG	LGG	30	MALE	WHITE	Oligoden	G3	2011	-11252	Alive	WITH TUM	992			Stable Disease	0	992	0	992			0	992
TCGA-FG	TCGA-FG	LGG	27	FEMALE	WHITE	Oligoden	G2	2010	-10082	Alive	TUMOR F	1164		499	Complete Remission/Response	0	1164	0	1164	1	499	1	499
TCGA-FG	TCGA-FG	LGG	27	FEMALE	WHITE	Oligoden	G2	2010	-10082	Alive	TUMOR F	1164		499	Complete Remission/Response	0	1164	0	1164	1	499	1	499
TCGA-FG	TCGA-FG	LGG	58	FEMALE	WHITE	Oligoastr	G3	2012	-21450	Alive	WITH TUM	326			[Unknown]	0	326	0	326			0	326
TCGA-FG	TCGA-FG	LGG	63	MALE	WHITE	Oligoastr	G3	2012	-23077	Dead	WITH TUMOR		559	226	Stable Disease	1	559	1	559			1	226
TCGA-FG	TCGA-FG	LGG	47	MALE	WHITE	Astrocyto	G2	2012	-17381	Alive	TUMOR F	569			Complete Remission/Response	0	569	0	569	0	569	0	569
TCGA-FG	TCGA-FG	LGG	44	FEMALE	WHITE	Oligoastr	G2	2012	-16310	Alive	WITH TUM	721			Stable Disease	0	721	0	721			0	721
TCGA-FG	TCGA-FG	LGG	47	FEMALE	WHITE	Oligoastr	G2	2012	-17339	Alive	TUMOR F	449			Complete Remission/Response	0	449	0	449	0	449	0	449
TCGA-FG	TCGA-FG	LGG	34	FEMALE	WHITE	Oligoastr	G2	2012	-12445	Alive	WITH TUM	542			Stable Disease	0	542	0	542			0	542
TCGA-FG	TCGA-FG	LGG	34	FEMALE	WHITE	Astrocyto	G2	2012	-12580	Alive	TUMOR F	655			Complete Remission/Response	0	655	0	655	0	655	0	655
TCGA-FG	TCGA-FG	LGG	60	MALE	WHITE	Oligoden	G2	2013	-22275	Alive	TUMOR F	457			Complete Remission/Response	0	457	0	457	0	457	0	457
TCGA-FG	TCGA-FG	LGG	44	FEMALE	WHITE	Oligoden	G2	2013	-16106	Alive	WITH TUM	500			Stable Disease	0	500	0	500			0	500
TCGA-FG	TCGA-FG	LGG	52	FEMALE	WHITE	Astrocyto	G3	2012	-19138	Alive	WITH TUM	677		285	Progressive Disease	0	677	0	677			1	285
TCGA-FG	TCGA-FG	LGG	20	FEMALE	WHITE	Oligoden	G2	2011	-7421	Alive	WITH TUM	868		490	Complete Remission/Response	0	868	0	868	1	490	1	490
TCGA-FG	TCGA-FG	LGG	53	FEMALE	BLACK OR AFRICAN AMERICAN	Oligoastr	G3	2011	-19717	Alive	WITH TUM	328			Stable Disease	0	328	0	328			0	328
TCGA-FG	TCGA-FG	LGG	50	FEMALE	WHITE	Oligoden	G2	2011	-18415	Alive	WITH TUM	1115			Stable Disease	0	1115	0	1115			0	1115
TCGA-FG	TCGA-FG	LGG	33	FEMALE	WHITE	Oligoden	G2	2010	-12248	Dead	WITH TUMOR		1481	347	Progressive Disease	1	1481	1	1481			1	347
TCGA-FG	TCGA-FG	LGG	74	FEMALE	WHITE	Oligoastr	G2	2013	-27232	Alive	TUMOR F	622			Complete Remission/Response	0	622	0	622	0	622	0	622
TCGA-FG	TCGA-FG	LGG	37	FEMALE	ASIAN	Astrocyto	G3	2013	-13606	Alive	WITH TUM	567			Stable Disease	0	567	0	567			0	567
TCGA-FG	TCGA-FG	LGG	61	FEMALE	WHITE	Astrocyto	G3	2013	-22565	Alive	TUMOR F	174			Complete Remission/Response	0	174	0	174	0	174	0	174
TCGA-FN	TCGA-FN	LGG	25	MALE	WHITE	Oligoastr	G3	2011	-9228	Alive	TUMOR F	837			Complete Remission/Response	0	837	0	837	0	837	0	837
TCGA-HT	TCGA-HT	LGG	54	MALE	WHITE	Oligoden	G2	2011	-19944	Alive	TUMOR F	3			Complete Remission/Response	0	3	0	3	0	3	0	3
TCGA-HT	TCGA-HT	LGG	30	MALE	WHITE	Oligoden	G3	2011	-11209	Alive	TUMOR F	203			Stable Disease	0	203	0	203			0	203
TCGA-HT	TCGA-HT	LGG	30	MALE	WHITE	Oligoden	G3	2011	-11097	Dead	WITH TUMOR		351	92	Progressive Disease	1	351	1	351			1	92
TCGA-HT	TCGA-HT	LGG	37	MALE	WHITE	Oligoden	G3	2011	-13611	Dead	TUMOR FREE		1220		Complete Remission/Response	1	1220	0	1220	0	1220	0	1220
TCGA-HT	TCGA-HT	LGG	37	FEMALE	WHITE	Oligoden	G3	2010	-13564	Alive	TUMOR F	4				0	4	0	4			0	4
TCGA-HT	TCGA-HT	LGG	38	MALE	WHITE	Oligoden	G2	2010	-13968	Alive	TUMOR F	1			Stable Disease	0	1	0	1			0	1
TCGA-HT	TCGA-HT	LGG	28	MALE	WHITE	Oligoastr	G2	2010	-10306	Alive	TUMOR F	503			Complete Remission/Response	0	503	0	503	0	503	0	503
TCGA-HT	TCGA-HT	LGG	52	MALE	WHITE	Oligoastr	G2	2011	-19149	Alive	TUMOR F	114			Stable Disease	0	114	0	114			0	114
TCGA-HT	TCGA-HT	LGG	67	MALE	WHITE	Oligoastr	G3	2010	-24686	Alive	TUMOR F	530			Complete Remission/Response	0	530	0	530	0	530	0	530
TCGA-HT	TCGA-HT	LGG	26	MALE	WHITE	Astrocyto	G2	2011	-9614	Alive	TUMOR F	199			Stable Disease	0	199	0	199			0	199
TCGA-HT	TCGA-HT	LGG	62	MALE	WHITE	Astrocyto	G3	2011	-22948	Alive	TUMOR F	738			Complete Remission/Response	0	738	0	738	0	738	0	738
TCGA-HT	TCGA-HT	LGG	36	MALE	WHITE	Astrocyto	G2	2011	-13216	Alive	TUMOR F	194			Complete Remission/Response	0	194	0	194	0	194	0	194
TCGA-HT	TCGA-HT	LGG	44	MALE	WHITE	Astrocyto	G3	2011	-16137	Alive	TUMOR F	1227		705	Complete Remission/Response	0	1227	0	1227	1	705	1	705
TCGA-HT	TCGA-HT	LGG	33	MALE	WHITE	Oligoden	G2	2005	-12291	Alive	WITH TUM	2287		1943	Stable Disease	0	2287	0	2287			1	1943
TCGA-HT	TCGA-HT	LGG	39	MALE	WHITE	Oligoden	G2	2006	-14306	Alive	WITH TUM	2918		880	Stable Disease	0	2918	0	2918			1	880
TCGA-HT	TCGA-HT	LGG	18	FEMALE	WHITE	Oligoastr	G2	2005	-6857	Alive	TUMOR F	3253		374	Partial Remission/Response	0	3253	0	3253			1	374
TCGA-HT	TCGA-HT	LGG	14	MALE	WHITE	Oligoastr	G2	1998	-5267	Alive	TUMOR F	5255			Complete Remission/Response	0	5255	0	5255	0	5255	0	5255
TCGA-HT	TCGA-HT	LGG	42	MALE	WHITE	Astrocyto	G2	2006	-15589	Alive	TUMOR F	122			Complete Remission/Response	0	122	0	122	0	122	0	122
TCGA-HT	TCGA-HT	LGG	30	FEMALE	WHITE	Astrocyto	G3	2008	-10989	Alive	TUMOR F	153			Partial Remission/Response	0	153	0	153			0	153
TCGA-HT	TCGA-HT	LGG	21	MALE	WHITE	Oligoden	G2	2008	-7946	Alive	TUMOR F	908				0	908	0	908			0	908
TCGA-HT	TCGA-HT	LGG	29	MALE	WHITE	Oligoden	G2	2008	-10883	Alive	TUMOR F	705			Complete Remission/Response	0	705	0	705	0	705	0	705
TCGA-HT	TCGA-HT	LGG	50	MALE	BLACK OR AFRICAN AMERICAN	Astrocyto	G2	2003	-18443	Alive	TUMOR F	3725			Partial Remission/Response	0	3725	0	3725			0	3725
TCGA-HT	TCGA-HT	LGG	38	MALE	WHITE	Oligoden	G2	2008	-13892	Alive		139				0	139	0	139			0	139
TCGA-HT	TCGA-HT	LGG	30	FEMALE	WHITE	Astrocyto	G2	2008	-11233	Alive	TUMOR F	526			Complete Remission/Response	0	526	0	526	0	526	0	526
TCGA-HT	TCGA-HT	LGG	61	FEMALE	WHITE	Astrocyto	G2	2008	-22585	Dead	WITH TUMOR		96		Progressive Disease	1	96	1	96			1	96
TCGA-HT	TCGA-HT	LGG	61	MALE	BLACK OR AFRICAN AMERICAN	Oligoastr	G2	2007	-22302	Alive	TUMOR F	671			Stable Disease	0	671	0	671			0	671
TCGA-HT	TCGA-HT	LGG	34	MALE	WHITE	Oligoastr	G3	2007	-12607	Alive	TUMOR F	1399			Stable Disease	0	1399	0	1399			0	1399
TCGA-HT	TCGA-HT	LGG	25	FEMALE	WHITE	Oligoastr	G2	2007	-9447	Alive	TUMOR F	1706		1205	Stable Disease	0	1706	0	1706			1	1205
TCGA-HT	TCGA-HT	LGG	36	MALE	WHITE	Oligoastr	G2	2007	-13314	Alive	TUMOR F	1752			Complete Remission/Response	0	1752	0	1752	0	1752	0	1752
TCGA-HT	TCGA-HT	LGG	75	MALE	WHITE	Oligoden	G3	2007	-27685	Dead	WITH TUMOR		7		Progressive Disease	1	7	1	7			1	7
TCGA-HT	TCGA-HT	LGG	40	MALE	WHITE	Oligoden	G3	2007	-14659	Alive	TUMOR F	434		280	Complete Remission/Response	0	434	0	434	1	280	1	280
TCGA-HT	TCGA-HT	LGG	26	MALE	WHITE	Oligoden	G2	2009	-9667	Alive	TUMOR F	5				0	5	0	5			0	5
TCGA-HT	TCGA-HT	LGG	53	MALE	WHITE	Oligoden	G3	2009	-19610	Alive	[Discrepar	494			Stable Disease	0	494	0	494			0	494
TCGA-HT	TCGA-HT	LGG	32	FEMALE	WHITE	Astrocyto	G2	2010	-11970	Alive	TUMOR F	23			Stable Disease	0	23	0	23			0	23
TCGA-HT	TCGA-HT	LGG	29	FEMALE	WHITE	Oligoastr	G2	2009	-10790	Alive	TUMOR F	1359			Complete Remission/Response	0	1359	0	1359	0	1359	0	1359
TCGA-HT	TCGA-HT	LGG	58	MALE	WHITE	Oligoastr	G3	2008	-21352	Alive	TUMOR F	184			Complete Remission/Response	0	184	0	184	0	184	0	184
TCGA-HT	TCGA-HT	LGG	29	FEMALE	WHITE	Astrocyto	G3	2008	-10724	Alive	TUMOR F	1300			Complete Remission/Response	0	1300	0	1300	0	1300	0	1300
TCGA-HT	TCGA-HT	LGG	74	MALE	WHITE	Oligoden	G3	2010	-27186	Alive	TUMOR F	3			Stable Disease	0	3	0	3			0	3
TCGA-HT	TCGA-HT	LGG	59	MALE	WHITE	Oligoden	G3	2009	-21844	Alive	TUMOR F	964			Complete Remission/Response	0	964	0	964	0	964	0	964
TCGA-HT	TCGA-HT	LGG	58	FEMALE	WHITE	Oligoden	G2	2009	-21331	Alive	TUMOR F	455			Complete Remission/Response	0	455	0	455	0	455	0	455
TCGA-HT	TCGA-HT	LGG	29	MALE	WHITE	Oligoastr	G3	2009	-10673	Alive	TUMOR F	3				0	3	0	3			0	3
TCGA-HT	TCGA-HT	LGG	31	FEMALE	WHITE	Astrocyto	G2	2008	-11465	Alive	TUMOR F	3				0	3	0	3			0	3
TCGA-HT	TCGA-HT	LGG	43	MALE	WHITE	Oligoastr	G2	2009	-15727	Alive	TUMOR F	90			Complete Remission/Response	0	90	0	90	0	90	0	90
TCGA-HT	TCGA-HT	LGG	51	FEMALE	WHITE	Oligoden	G2	2008	-18956	Alive	TUMOR F	533			Partial Remission/Response	0	533	0	533			0	533
TCGA-HT	TCGA-HT	LGG	60	MALE	WHITE	Oligoden	G3	2008	-21930	Alive	TUMOR F	210			Complete Remission/Response	0	210	0	210	0	210	0	210
TCGA-HT	TCGA-HT	LGG	29	FEMALE	WHITE	Oligoden	G2	2009	-10827	Alive	TUMOR F	442			Complete Remission/Response	0	442	0	442	0	442	0	442
TCGA-HT	TCGA-HT	LGG	62	MALE	WHITE	Astrocyto	G2	2010	-22738	Alive	TUMOR F	1201		1147	Stable Disease	0	1201	0	1201			1	1147
TCGA-HT	TCGA-HT	LGG	39	MALE	WHITE	Astrocyto	G3	2008	-14496	Alive	TUMOR F	585			Complete Remission/Response	0	585	0	585	0	585	0	585
TCGA-HT	TCGA-HT	LGG	35	MALE	WHITE	Oligoden	G3	2008	-13145	Alive	TUMOR F	1189			Complete Remission/Response	0	1189	0	1189	0	1189	0	1189
TCGA-HT	TCGA-HT	LGG	24	FEMALE	WHITE	Astrocyto	G3	2006	-8912	Alive	TUMOR F	7				0	7	0	7			0	7
TCGA-HT	TCGA-HT	LGG	28	MALE	WHITE	Astrocyto	G2	2007	-10422	Alive	TUMOR F	1540			Complete Remission/Response	0	1540	0	1540	0	1540	0	1540
TCGA-HT	TCGA-HT	LGG	60	FEMALE	WHITE	Astrocyto	G3	2009	-22034	Alive	TUMOR F	15				0	15	0	15			0	15
TCGA-HT	TCGA-HT	LGG	29	MALE	WHITE	Oligoastr	G2	2011	-10805	Alive	WITH TUM	718			Stable Disease	0	718	0	718			0	718
TCGA-HT	TCGA-HT	LGG	41	FEMALE	WHITE	Oligoden	G3	2008	-15045	Alive	TUMOR F	1130			Complete Remission/Response	0	1130	0	1130	0	1130	0	1130
TCGA-HT	TCGA-HT	LGG	56	MALE	WHITE	Oligoden	G2	2008	-20613	Alive	TUMOR F	10			Stable Disease	0	10	0	10			0	10
TCGA-HT	TCGA-HT	LGG	20	FEMALE	WHITE	Oligoden	G2	2011	-7581	Alive	TUMOR F	4				0	4	0	4			0	4
TCGA-HT	TCGA-HT	LGG	31	MALE	WHITE	Oligoastr	G3	2011	-11622	Alive	TUMOR F	112			Complete Remission/Response	0	112	0	112	0	112	0	112
TCGA-HT	TCGA-HT	LGG	30	MALE	WHITE	Oligoastr	G2	2011	-11043	Alive	TUMOR F	162				0	162	0	162			0	162
TCGA-HT	TCGA-HT	LGG	38	MALE	BLACK OR AFRICAN AMERICAN	Oligoden	G2	2011	-14171	Alive	TUMOR F	1079			Complete Remission/Response	0	1079	0	1079	0	1079	0	1079
TCGA-HT	TCGA-HT	LGG	66	MALE	WHITE	Oligoden	G3	2010	-24450	Dead	WITH TUMOR		113		Progressive Disease	1	113	1	113			1	113
TCGA-HT	TCGA-HT	LGG	44	FEMALE	WHITE	Astrocyto	G2	2011	-16280	Alive	[Discrepar	343			Stable Disease	0	343	0	343			0	343
TCGA-HT	TCGA-HT	LGG	30	FEMALE	WHITE	Oligoastr	G2	2009	-11118	Alive	TUMOR F	956			Stable Disease	0	956	0	956			0	956
TCGA-HT	TCGA-HT	LGG	64	FEMALE	WHITE	Oligoden	G2	2011	-23680	Alive	TUMOR F	50			Stable Disease	0	50	0	50			0	50
TCGA-HT	TCGA-HT	LGG	55	MALE	WHITE	Astrocyto	G3	2009	-20175	Alive	WITH TUM	494		278	Stable Disease	0	494	0	494			1	278
TCGA-HT	TCGA-HT	LGG	30	FEMALE	WHITE	Oligoden	G2	2010	-11295	Alive	WITH TUM	286		133	Stable Disease	0	286	0	286			1	133
TCGA-HT	TCGA-HT	LGG	37	FEMALE	WHITE	Oligoastr	G2	2005	-13753	Dead	WITH TUMOR		1933	1306	Stable Disease	1	1933	1	1933			1	1306
TCGA-HT	TCGA-HT	LGG	21	MALE	WHITE	Astrocyto	G2	2008	-7760	Alive	TUMOR F	3			Complete Remission/Response	0	3	0	3	0	3	0	3
TCGA-HT	TCGA-HT	LGG	40	FEMALE	WHITE	Oligoastr	G2	2010	-14910	Dead	TUMOR FREE		1152		Partial Remission/Response	1	1152	0	1152			0	1152
TCGA-HT	TCGA-HT	LGG	34	FEMALE	WHITE	Oligoden	G3	2009	-12669	Alive	TUMOR F	1004			Complete Remission/Response	0	1004	0	1004	0	1004	0	1004
TCGA-HT	TCGA-HT	LGG	51	FEMALE	WHITE	Astrocyto	G3	2010	-18748	Alive	TUMOR F	372				0	372	0	372			0	372
TCGA-HT	TCGA-HT	LGG	54	MALE	WHITE	Oligoden	G3	2011	-20041	Alive	TUMOR F	190			Stable Disease	0	190	0	190			0	190
TCGA-HT	TCGA-HT	LGG	53	MALE	WHITE	Astrocyto	G3	2010	-19555	Alive	TUMOR F	3			Stable Disease	0	3	0	3			0	3
TCGA-HT	TCGA-HT	LGG	62	MALE	WHITE	Oligoden	G2	2011	-22880	Alive	TUMOR F	14			Stable Disease	0	14	0	14			0	14
TCGA-HT	TCGA-HT	LGG	26	FEMALE	WHITE	Oligoden	G2	2011	-9539	Alive	TUMOR F	76			Stable Disease	0	76	0	76			0	76
TCGA-HT	TCGA-HT	LGG	64	MALE	WHITE	Oligoden	G3	2011	-23486	Alive	TUMOR F	169			[Unknown]	0	169	0	169			0	169
TCGA-HT	TCGA-HT	LGG	57	MALE	WHITE	Astrocyto	G3	2011	-21183	Alive	TUMOR F	419			Complete Remission/Response	0	419	0	419	0	419	0	419
TCGA-HT	TCGA-HT	LGG	32	MALE	WHITE	Oligoastr	G3	2011	-11810	Alive		7				0	7	0	7			0	7
TCGA-HT	TCGA-HT	LGG	49	FEMALE	BLACK OR AFRICAN AMERICAN	Oligoden	G2	2006	-18173	Alive	TUMOR F	900			Complete Remission/Response	0	900	0	900	0	900	0	900
TCGA-HT	TCGA-HT	LGG	36	MALE	WHITE	Oligoastr	G3	2011	-13198	Alive	TUMOR F	1040			Complete Remission/Response	0	1040	0	1040	0	1040	0	1040
TCGA-HT	TCGA-HT	LGG	29	FEMALE	WHITE	Oligoden	G2	2011	-10950	Alive	TUMOR F	418			Complete Remission/Response	0	418	0	418	0	418	0	418
TCGA-HT	TCGA-HT	LGG	30	FEMALE	WHITE	Astrocyto	G3	2011	-11305	Alive	TUMOR F	860			Complete Remission/Response	0	860	0	860	0	860	0	860
TCGA-HT	TCGA-HT	LGG	47	MALE	WHITE	Astrocyto	G3	2012	-17499	Alive	WITH TUM	478		120	Progressive Disease	0	478	0	478			1	120
TCGA-HT	TCGA-HT	LGG	55	FEMALE	WHITE	Astrocyto	G3	2012	-20296	Alive	TUMOR F	7				0	7	0	7			0	7
TCGA-HT	TCGA-HT	LGG	51	FEMALE	WHITE	Oligoden	G3	2012	-18799	Alive	TUMOR F	792			Complete Remission/Response	0	792	0	792	0	792	0	792
TCGA-HT	TCGA-HT	LGG	33	FEMALE	WHITE	Oligoden	G2	2012	-12403	Alive	TUMOR F	4			Complete Remission/Response	0	4	0	4	0	4	0	4
TCGA-HT	TCGA-HT	LGG	33	FEMALE	WHITE	Astrocyto	G3	2012	-12092	Alive	TUMOR F	615			Complete Remission/Response	0	615	0	615	0	615	0	615
TCGA-HT	TCGA-HT	LGG	48	FEMALE	WHITE	Oligoden	G3	2012	-17775	Alive		2				0	2	0	2			0	2
TCGA-HT	TCGA-HT	LGG	65	FEMALE	WHITE	Astrocyto	G3	2012	-23769	Alive	WITH TUM	832			Partial Remission/Response	0	832	0	832			0	832
TCGA-HT	TCGA-HT	LGG	24	MALE	WHITE	Astrocyto	G2	2011	-8820	Alive		3				0	3	0	3			0	3
TCGA-HT	TCGA-HT	LGG	70	FEMALE	WHITE	Astrocyto	G3	2012	-25772	Dead	WITH TUMOR		162		Progressive Disease	1	162	1	162			1	162
TCGA-HT	TCGA-HT	LGG	47	MALE	WHITE	Oligoastr	G2	2013	-17392	Alive	WITH TUM	82			Partial Remission/Response	0	82	0	82			0	82
TCGA-HT	TCGA-HT	LGG	38	FEMALE	WHITE	Oligoden	G2	2012	-13892	Alive	TUMOR F	515			Complete Remission/Response	0	515	0	515	0	515	0	515
TCGA-HT	TCGA-HT	LGG	36	FEMALE	WHITE	Astrocyto	G2	2013	-13222	Alive		55				0	55	0	55			0	55
TCGA-HT	TCGA-HT	LGG	47	MALE	AMERICAN INDIAN OR ALASKA	Oligoden	G2	2010	-17331	Alive		491		395	Progressive Disease	0	491	0	491			1	395
TCGA-HT	TCGA-HT	LGG	37	FEMALE	WHITE	Astrocyto	G3	2013	-13595	Alive	TUMOR F	512			Complete Remission/Response	0	512	0	512	0	512	0	512
TCGA-HT	TCGA-HT	LGG	51	FEMALE	ASIAN	Oligoden	G3	2012	-18857	Alive	TUMOR F	651			Complete Remission/Response	0	651	0	651	0	651	0	651
TCGA-HT	TCGA-HT	LGG	20	FEMALE	WHITE	Oligoden	G2	2013	-7664	Alive	TUMOR F	194			Complete Remission/Response	0	194	0	194	0	194	0	194
TCGA-HT	TCGA-HT	LGG	22	MALE	WHITE	Astrocyto	G3	2012	-8335	Alive	TUMOR F	533			Complete Remission/Response	0	533	0	533	0	533	0	533
TCGA-HT	TCGA-HT	LGG	66	MALE	WHITE	Oligoden	G3	2013	-24276	Dead	WITH TUMOR		537	476	Complete Remission/Response	1	537	1	537	1	476	1	476
TCGA-HT	TCGA-HT	LGG	62	MALE	WHITE	Astrocyto	G3	2013	-22850	Alive		74			Stable Disease	0	74	0	74			0	74
TCGA-HT	TCGA-HT	LGG	33	MALE	WHITE	Oligoastr	G2	2013	-12393	Alive		320			Partial Remission/Response	0	320	0	320			0	320
TCGA-HT	TCGA-HT	LGG	58	FEMALE	WHITE	Oligoden	G3	2012	-21189	Alive	TUMOR F	467			Complete Remission/Response	0	467	0	467	0	467	0	467
TCGA-HT	TCGA-HT	LGG	22	FEMALE	WHITE	Oligoastr	G2	2013	-8240	Alive	WITH TUM	336			Partial Remission/Response	0	336	0	336			0	336
TCGA-HT	TCGA-HT	LGG	34	MALE	WHITE	Astrocyto	G3	2013	-12460	Alive		3				0	3	0	3			0	3
TCGA-HW	TCGA-HW	LGG	37	MALE	WHITE	Oligoden	G2	2010	-13546	Alive	TUMOR F	1721			Stable Disease	0	1721	0	1721			0	1721
TCGA-HW	TCGA-HW	LGG	39	MALE	WHITE	Oligoden	G2	2010	-14535	Alive	WITH TUM	1382		840	Progressive Disease	0	1382	0	1382			1	840
TCGA-HW	TCGA-HW	LGG	38	MALE	WHITE	Oligoastr	G2	2011	-14201	Dead	WITH TUMOR		1262		Progressive Disease	1	1262	1	1262			1	1262
TCGA-HW	TCGA-HW	LGG	41	MALE	WHITE	Astrocyto	G2	2011	-15092	Alive	WITH TUM	1387			Progressive Disease	0	1387	0	1387			0	1387
TCGA-HW	TCGA-HW	LGG	35	MALE	WHITE	Oligoden	G2	2009	-12874	Alive	WITH TUM	2078		2197	Progressive Disease	0	2078	0	2078			1	2197
TCGA-HW	TCGA-HW	LGG	40	FEMALE	WHITE	Astrocyto	G2	2009	-14767	Alive	WITH TUM	2107			Stable Disease	0	2107	0	2107			0	2107
TCGA-HW	TCGA-HW	LGG	45	FEMALE	WHITE	Oligoden	G2	2011	-16460	Alive	WITH TUM	1078			Progressive Disease	0	1078	0	1078			0	1078
TCGA-HW	TCGA-HW	LGG	34	FEMALE	WHITE	Astrocyto	G3	2011	-12557	Dead	WITH TUMOR		1209	997	Partial Remission/Response	1	1209	1	1209			1	997
TCGA-HW	TCGA-HW	LGG	36	MALE	WHITE	Astrocyto	G3	2011	-13503	Alive	WITH TUM	1217			Stable Disease	0	1217	0	1217			0	1217
TCGA-HW	TCGA-HW	LGG	31	MALE	WHITE	Astrocyto	G3	2011	-11474	Alive	WITH TUM	1294			Stable Disease	0	1294	0	1294			0	1294
TCGA-HW	TCGA-HW	LGG	39	MALE	WHITE	Oligoden	G2	2012	-14548	Alive	WITH TUM	758			Stable Disease	0	758	0	758			0	758
TCGA-HW	TCGA-HW	LGG	68	MALE	WHITE	Oligoden	G3	2012	-24966	Dead	WITH TUMOR		962	615	Progressive Disease	1	962	1	962			1	615
TCGA-HW	TCGA-HW	LGG	64	MALE	WHITE	Astrocyto	G3	2012	-23484	Dead	WITH TUMOR		388		Progressive Disease	1	388	1	388			1	388
TCGA-HW	TCGA-HW	LGG	42	FEMALE	WHITE	Astrocyto	G2	2012	-15513	Alive	WITH TUM	878			Stable Disease	0	878	0	878			0	878
TCGA-HW	TCGA-HW	LGG	35	MALE	WHITE	Astrocyto	G2	2012	-12845	Alive	WITH TUM	630			Progressive Disease	0	630	0	630			0	630
TCGA-IK-	TCGA-IK-	LGG	43	MALE	WHITE	Oligoden	G2	2009	-15900	Dead	WITH TUMOR		578	123	Progressive Disease	1	578	1	578			1	123
TCGA-IK-	TCGA-IK-	LGG	62	MALE	WHITE	Oligoastr	G3	2009	-22745	Alive	TUMOR F	1301			Complete Remission/Response	0	1301	0	1301	0	1301	0	1301
TCGA-KT	TCGA-KT	LGG	26	MALE	WHITE	Oligoastr	G3	2013	-9590	Alive	WITH TUM	438			Stable Disease	0	438	0	438			0	438
TCGA-KT	TCGA-KT	LGG	45	FEMALE	WHITE	Astrocyto	G3	2013	-16560	Alive	TUMOR F	438		84	Progressive Disease	0	438	0	438			1	84
TCGA-P5	TCGA-P5	LGG	27	MALE	WHITE	Oligoden	G2	2012	-10027	Alive	TUMOR F	0				0	0	0	0			0	0
TCGA-P5	TCGA-P5	LGG	35	MALE	WHITE	Astrocyto	G3	2012	-12851	Alive		0				0	0	0	0			0	0
TCGA-P5	TCGA-P5	LGG	39	MALE	WHITE	Astrocyto	G2	2012	-14310	Alive	WITH TUM	7				0	7	0	7			0	7
TCGA-P5	TCGA-P5	LGG	20	FEMALE	WHITE	Astrocyto	G2	2012	-7612	Alive	TUMOR F	204				0	204	0	204			0	204
TCGA-P5	TCGA-P5	LGG	41	FEMALE	WHITE	Oligoden	G3	2012	-15091	Alive	TUMOR F	243			Partial Remission/Response	0	243	0	243			0	243
TCGA-P5	TCGA-P5	LGG	21	MALE	WHITE	Astrocyto	G2	2012	-7716	Alive		72				0	72	0	72			0	72
TCGA-P5	TCGA-P5	LGG	39	MALE	WHITE	Astrocyto	G3	2012	-14375	Alive	TUMOR F	70				0	70	0	70			0	70
TCGA-P5	TCGA-P5	LGG	33	MALE	WHITE	Oligoden	G2	2012	-12157	Alive	[Discrepar	287			Partial Remission/Response	0	287	0	287			0	287
TCGA-P5	TCGA-P5	LGG	30	MALE	WHITE	Astrocyto	G2	2012	-11070	Alive	TUMOR F	179				0	179	0	179			0	179
TCGA-P5	TCGA-P5	LGG	36	FEMALE	WHITE	Astrocyto	G2	2012	-13396	Alive	WITH TUM	242			Partial Remission/Response	0	242	0	242			0	242
TCGA-P5	TCGA-P5	LGG	35	FEMALE	WHITE	Oligoden	G3	2012	-12851	Alive	[Discrepar	231			Partial Remission/Response	0	231	0	231			0	231
TCGA-P5	TCGA-P5	LGG	26	MALE	WHITE	Oligoden	G2	2012	-9575	Alive	TUMOR F	39				0	39	0	39			0	39
TCGA-P5	TCGA-P5	LGG	71	FEMALE	WHITE	Oligoden	G3	2013	-26002	Alive	TUMOR F	7			Complete Remission/Response	0	7	0	7	0	7	0	7
TCGA-P5	TCGA-P5	LGG	35	MALE	WHITE	Astrocyto	G3	2013	-12848	Alive	TUMOR F	317			Complete Remission/Response	0	317	0	317	0	317	0	317
TCGA-P5	TCGA-P5	LGG	21	MALE	WHITE	Astrocyto	G3	2013	-7913	Alive	TUMOR F	403			Complete Remission/Response	0	403	0	403	0	403	0	403

TCGA-P5	TCGA-P5	LGG	64	FEMALE	WHITE		Oligoden	G3	2013	-23482	Alive	TUMOR F	354						Complete Remission/Response		0	354	0	354	0	354	0	354
TCGA-P5	TCGA-P5	LGG	22	MALE	WHITE		Oligoastr	G3	2013	-8043	Alive	TUMOR F	333						Complete Remission/Response		0	333	0	333	0	333	0	333
TCGA-P5	TCGA-P5	LGG	59	FEMALE	WHITE		Oligoastr	G2	2013	-21715	Alive	TUMOR F	274						[Unknown]		0	274	0	274			0	274
TCGA-P5	TCGA-P5	LGG	52	FEMALE	WHITE		Astrocyto	G2	2013	-19142	Alive	TUMOR F	414						Complete Remission/Response		0	414	0	414	0	414	0	414
TCGA-P5	TCGA-P5	LGG	38	FEMALE	WHITE		Astrocyto	G2	2013	-14152	Alive	TUMOR F	292						Complete Remission/Response		0	292	0	292	0	292	0	292
TCGA-P5	TCGA-P5	LGG	44	FEMALE	WHITE		Astrocyto	G3	2013	-16300	Alive	TUMOR F	230						Complete Remission/Response		0	230	0	230	0	230	0	230
TCGA-P5	TCGA-P5	LGG	47	MALE	WHITE		Oligoastr	G2	2012	-17390	Alive	TUMOR F	372						Complete Remission/Response		0	372	0	372	0	372	0	372
TCGA-P5	TCGA-P5	LGG	37	FEMALE	WHITE		Oligoastr	G3	2013	-13545	Alive	TUMOR F	584						Complete Remission/Response		0	584	0	584	0	584	0	584
TCGA-P5	TCGA-P5	LGG	55	FEMALE	WHITE		Oligoastr	G2	2013	-20128	Alive	TUMOR F	105						Complete Remission/Response		0	105	0	105	0	105	0	105
TCGA-P5	TCGA-P5	LGG	44	FEMALE	WHITE		Astrocyto	G3	2013	-16161	Alive	TUMOR F	72						Complete Remission/Response		0	72	0	72	0	72	0	72
TCGA-P5	TCGA-P5	LGG	34	FEMALE	WHITE		Astrocyto	G3	2013	-12757	Alive	TUMOR F	134						Complete Remission/Response		0	134	0	134	0	134	0	134
TCGA-OH	TCGA-OH	LGG	38	FEMALE	WHITE		Oligoden	G3	2013	-14073	Alive	WITH TUM	461						Partial Remission/Response		0	461	0	461			0	461
TCGA-OH	TCGA-OH	LGG	32	FEMALE	WHITE		Oligoastr	G2	2013	-11861	Alive	[Discrepar	77						Partial Remission/Response		0	77	0	77			0	77
TCGA-OH	TCGA-OH	LGG	43	FEMALE	WHITE		Oligoden	G2	2013	-15850	Alive	WITH TUM	384						Stable Disease		0	384	0	384			0	384
TCGA-OH	TCGA-OH	LGG	28	FEMALE	WHITE		Oligoastr	G3	2013	-10440	Alive	TUMOR F	482						Stable Disease		0	482	0	482			0	482
TCGA-OH	TCGA-OH	LGG	54	MALE	WHITE		Oligoden	G2	2013	-19921	Alive	TUMOR F	427						Stable Disease		0	427	0	427			0	427
TCGA-OH	TCGA-OH	LGG	41	MALE	WHITE		Astrocyto	G3	2013	-15092	Alive	WITH TUM	588					498	Stable Disease		0	588	0	588			1	498
TCGA-QH	TCGA-QH	LGG	52	FEMALE	WHITE		Oligoden	G3	2013	-19149	Alive	TUMOR F	451						Stable Disease		0	451	0	451			0	451
TCGA-OH	TCGA-OH	LGG	51	MALE	ASIAN		Oligoastr	G3	2013	-18955	Alive	WITH TUM	442						Stable Disease		0	442	0	442			0	442
TCGA-QH	TCGA-QH	LGG	43	MALE	WHITE		Oligoastr	G3	2013	-15847	Alive	TUMOR F	414						Partial Remission/Response		0	414	0	414			0	414
TCGA-OH	TCGA-OH	LGG	66	MALE	WHITE		Astrocyto	G2	2013	-24252	Dead	WITH TUMOR		372				273	Complete Remission/Response		1	372	1	372	1	273	1	273
TCGA-OH	TCGA-OH	LGG	38	MALE	WHITE		Oligoastr	G3	2013	-14140	Alive	WITH TUM	408						Stable Disease		0	408	0	408			0	408
TCGA-OH	TCGA-OH	LGG	38	MALE	WHITE		Oligoastr	G2	2013	-14007	Alive	TUMOR F	279						Partial Remission/Response		0	279	0	279			0	279
TCGA-OH	TCGA-OH	LGG	27	MALE	WHITE		Oligoastr	G2	2013	-10184	Alive	TUMOR F	313						Complete Remission/Response		0	313	0	313	0	313	0	313
TCGA-OH	TCGA-OH	LGG	47	MALE	WHITE		Oligoastr	G3	2013	-17258	Alive	TUMOR F	442						Complete Remission/Response		0	442	0	442	0	442	0	442
TCGA-OH	TCGA-OH	LGG	58	FEMALE	WHITE		Oligoastr	G2	2013	-21239	Alive		497						[Unknown]		0	497	0	497			0	497
TCGA-QH	TCGA-QH	LGG	56	FEMALE	WHITE		Oligoden	G3	2013	-20771	Alive	WITH TUM	509						Stable Disease		0	509	0	509			0	509
TCGA-OH	TCGA-OH	LGG	73	FEMALE	WHITE		Oligoden	G2	2013	-26992	Alive		58						[Unknown]		0	58	0	58			0	58
TCGA-OH	TCGA-OH	LGG	23	FEMALE	WHITE		Oligoastr	G2	2013	-8508	Alive	TUMOR F	502						Complete Remission/Response		0	502	0	502	0	502	0	502
TCGA-OH	TCGA-OH	LGG	48	MALE	WHITE		Astrocyto	G3	2013	-17692	Alive	WITH TUM	508					438	Complete Remission/Response		0	508	0	508	1	438	1	438
TCGA-OH	TCGA-OH	LGG	33	MALE	WHITE		Oligoden	G2	2013	-12385	Alive	TUMOR F	337						Complete Remission/Response		0	337	0	337	0	337	0	337
TCGA-OH	TCGA-OH	LGG	38	FEMALE	WHITE		Oligoastr	G3	2013	-14082	Alive	WITH TUM	395					335	Complete Remission/Response		0	395	0	395	1	335	1	335
TCGA-R8	TCGA-R8	LGG	40	MALE	WHITE		Oligoden	G2	2005	-14716	Alive		2702						[Unknown]		0	2702	0	2702			0	2702
TCGA-R8	TCGA-R8	LGG	52	MALE	WHITE		Oligoden	G3	2005	-19185	Alive		2860						[Unknown]		0	2860	0	2860			0	2860
TCGA-R8	TCGA-R8	LGG	53	FEMALE	ASIAN		Oligoden	G2	2009	-19432	Alive		993								0	993	0	993			0	993
TCGA-R8	TCGA-R8	LGG	48	FEMALE	WHITE		Oligoden	G2	2008	-17610	Alive		1806						[Unknown]		0	1806	0	1806			0	1806
TCGA-RY	TCGA-RY	LGG	46	FEMALE	WHITE		Oligoden	G2	2012	-16911	Alive	TUMOR F	939								0	939	0	939			0	939
TCGA-RY	TCGA-RY	LGG	45	MALE	WHITE		Oligoden	G2	2012	-16470	Alive	TUMOR F	166								0	166	0	166			0	166
TCGA-RY	TCGA-RY	LGG	54	FEMALE	WHITE		Astrocyto	G3	2012	-19903	Alive	WITH TUM	301						Stable Disease		0	301	0	301			0	301
TCGA-RY	TCGA-RY	LGG	47	MALE	[Unknown]		Oligoden	G3	2012	-17501	Alive	WITH TUM	854						Stable Disease		0	854	0	854			0	854
TCGA-RY	TCGA-RY	LGG	30	MALE	WHITE		Astrocyto	G3	2012	-11083	Alive	WITH TUM	63								0	63	0	63			0	63
TCGA-RY	TCGA-RY	LGG	40	FEMALE	WHITE		Oligoastr	G2	2013	-14617	Alive	TUMOR F	596								0	596	0	596			0	596
TCGA-RY	TCGA-RY	LGG	45	MALE	WHITE		Oligoden	G2	2012	-16732	Alive	TUMOR F	933								0	933	0	933			0	933
TCGA-S9	TCGA-S9	LGG	48	FEMALE	WHITE		Astrocyto	G3	2006	-17857	Dead	WITH TUMOR		1891				1324	Progressive Disease		1	1891	1	1891			1	1324
TCGA-S9	TCGA-S9	LGG	38	MALE	WHITE		Astrocyto	G2	2007	-14108	Alive	WITH TUM	2650					1475	Partial Remission/Response		0	2650	0	2650			1	1475
TCGA-S9	TCGA-S9	LGG	50	MALE	WHITE		Oligoastr	G3	2010	-18321	Alive	TUMOR F	571						Complete Remission/Response		0	571	0	571	0	571	0	571
TCGA-S9	TCGA-S9	LGG	40	MALE	WHITE		Oligoden	G3	2011	-14679	Alive	TUMOR F	1250					861	Partial Remission/Response		0	1250	0	1250			1	861
TCGA-S9	TCGA-S9	LGG	46	MALE	WHITE		Oligoden	G3	2011	-17025	Alive	WITH TUM	1245					856	Complete Remission/Response		0	1245	0	1245	1	856	1	856
TCGA-S9	TCGA-S9	LGG	50	MALE	WHITE		Oligoden	G2	2011	-18564	Alive	WITH TUM	1076						Partial Remission/Response		0	1076	0	1076			0	1076
TCGA-S9	TCGA-S9	LGG	39	FEMALE	WHITE		Astrocyto	G2	2011	-14592	Alive	WITH TUM	1120					552	Partial Remission/Response		0	1120	0	1120			1	552
TCGA-S9	TCGA-S9	LGG	46	MALE	WHITE		Astrocyto	G3	2012	-16890	Dead	WITH TUMOR		742				692	Partial Remission/Response		1	742	1	742			1	692
TCGA-S9	TCGA-S9	LGG	22	FEMALE	WHITE		Astrocyto	G3	2012	-8271	Alive	TUMOR F	785						Complete Remission/Response		0	785	0	785	0	785	0	785
TCGA-S9	TCGA-S9	LGG	48	FEMALE	WHITE		Oligoden	G2	2012	-17800	Alive	WITH TUM	908						Partial Remission/Response		0	908	0	908			0	908
TCGA-S9	TCGA-S9	LGG	33	MALE	WHITE		Astrocyto	G2	2011	-12417	Alive	TUMOR F	992						Complete Remission/Response		0	992	0	992	0	992	0	992
TCGA-S9	TCGA-S9	LGG	28	MALE	WHITE		Astrocyto	G3	2012	-10253	Alive	TUMOR F	1069						Complete Remission/Response		0	1069	0	1069	0	1069	0	1069
TCGA-S9	TCGA-S9	LGG	24	MALE	WHITE		Astrocyto	G2	2005	-9044	Dead	WITH TUMOR		2988				367	Partial Remission/Response		1	2988	1	2988			1	367
TCGA-S9	TCGA-S9	LGG	36	MALE	WHITE		Astrocyto	G3	2006	-13460	Alive	WITH TUM	2918						Partial Remission/Response		0	2918	0	2918			0	2918
TCGA-S9	TCGA-S9	LGG	66	MALE	WHITE		Astrocyto	G3	2008	-24242	Dead	WITH TUMOR		178				161	Progressive Disease		1	178	1	178			1	161
TCGA-S9	TCGA-S9	LGG	52	MALE	WHITE		Oligoden	G2	2005	-19223	Alive	TUMOR F	3761						Complete Remission/Response		0	3761	0	3761	0	3761	0	3761
TCGA-S9	TCGA-S9	LGG	58	MALE	WHITE		Oligoden	G3	2008	-21231	Alive	WITH TUM	2289						Partial Remission/Response		0	2289	0	2289			0	2289
TCGA-S9	TCGA-S9	LGG	34	MALE	WHITE		Oligoden	G2	2004	-12488	Alive	WITH TUM	4113						Partial Remission/Response		0	4113	0	4113			0	4113
TCGA-S9	TCGA-S9	LGG	31	MALE	WHITE		Astrocyto	G3	2007	-11509	Alive	WITH TUM	2602						Progressive Disease		0	2602	0	2602			0	2602
TCGA-S9	TCGA-S9	LGG	73	FEMALE	WHITE		Oligoastr	G2	2011	-26904	Alive	WITH TUM	1173						Partial Remission/Response		0	1173	0	1173			0	1173
TCGA-S9	TCGA-S9	LGG	56	FEMALE	WHITE		Oligoastr	G2	2006	-20609	Alive	WITH TUM	2585						Partial Remission/Response		0	2585	0	2585			0	2585
TCGA-S9	TCGA-S9	LGG	52	MALE	WHITE		Astrocyto	G3	2012	-19234	Alive	WITH TUM	946					240	Progressive Disease		0	946	0	946			1	240
TCGA-S9	TCGA-S9	LGG	59	FEMALE	WHITE		Astrocyto	G3	2012	-21761	Alive	WITH TUM	604					505	Partial Remission/Response		0	604	0	604			1	505
TCGA-S9	TCGA-S9	LGG	38	FEMALE	WHITE		Astrocyto	G3	2013	-13961	Alive	WITH TUM	805					303	Progressive Disease		0	805	0	805			1	303
TCGA-S9	TCGA-S9	LGG	29	MALE	WHITE		Astrocyto	G2	2013	-10933	Alive	WITH TUM	566					187	Progressive Disease		0	566	0	566			1	187
TCGA-S9	TCGA-S9	LGG	42	MALE	WHITE		Oligoastr	G3	2013	-15658	Alive	WITH TUM	567						Partial Remission/Response		0	567	0	567			0	567
TCGA-S9	TCGA-S9	LGG	57	FEMALE	WHITE		Oligoastr	G2	2013	-21133	Alive	WITH TUM	428						Partial Remission/Response		0	428	0	428			0	428
TCGA-S9	TCGA-S9	LGG	45	FEMALE	WHITE		Oligoastr	G2	2011	-16512	Alive	WITH TUM	1099						Partial Remission/Response		0	1099	0	1099			0	1099
TCGA-S9	TCGA-S9	LGG	33	FEMALE	WHITE		Astrocyto	G3	2004	-12402	Dead	WITH TUMOR		241					Progressive Disease		1	241	1	241			1	241
TCGA-S9	TCGA-S9	LGG	57	MALE	WHITE		Astrocyto	G3	2012	-21095	Dead	WITH TUMOR		819				350	Progressive Disease		1	819	1	819			1	350
TCGA-S9	TCGA-S9	LGG	39	MALE	WHITE		Oligoastr	G3	2012	-14422	Alive	WITH TUM	715						Partial Remission/Response		0	715	0	715			0	715
TCGA-S9	TCGA-S9	LGG	48	FEMALE	WHITE		Astrocyto	G3	2013	-17874	Alive	WITH TUM	608					467	Partial Remission/Response		0	608	0	608			1	467
TCGA-S9	TCGA-S9	LGG	30	FEMALE	WHITE		Oligoden	G3	2013	-10989	Alive	WITH TUM	609					390	Partial Remission/Response		0	609	0	609			1	390
TCGA-S9	TCGA-S9	LGG	43	MALE	WHITE		Oligoden	G2	2013	-15926	Alive	WITH TUM	249						Partial Remission/Response		0	249	0	249			0	249
TCGA-S9	TCGA-S9	LGG	25	MALE	WHITE		Oligoden	G3	2013	-9345	Alive	WITH TUM	62						Partial Remission/Response		0	62	0	62			0	62
TCGA-S9	TCGA-S9	LGG	52	FEMALE	WHITE		Oligoden	G3	2013	-19056	Alive	WITH TUM	629						Partial Remission/Response		0	629	0	629			0	629
TCGA-S9	TCGA-S9	LGG	54	FEMALE	WHITE		Astrocyto	G3	2013	-19869	Alive	WITH TUM	458					382	Complete Remission/Response		0	458	0	458	1	382	1	382
TCGA-S9	TCGA-S9	LGG	36	FEMALE	WHITE		Astrocyto	G3	2013	-13332	Alive	WITH TUM	564						Partial Remission/Response		0	564	0	564			0	564
TCGA-S9	TCGA-S9	LGG	35	FEMALE	WHITE		Oligoastr	G2	2012	-12853	Alive	WITH TUM	863						Partial Remission/Response		0	863	0	863			0	863
TCGA-S9	TCGA-S9	LGG	41	MALE	WHITE		Oligoden	G2	2012	-15300	Alive	WITH TUM	826					774	Partial Remission/Response		0	826	0	826			1	774
TCGA-S9	TCGA-S9	LGG	35	MALE	WHITE		Oligoden	G2	1999	-12828	Dead	WITH TUMOR		5166				3637	Stable Disease		1	5166	1	5166			1	3637
TCGA-S9	TCGA-S9	LGG	69	MALE	WHITE		Astrocyto	G3	2008	-25419	Dead	WITH TUMOR		316					Progressive Disease		1	316	1	316			1	316
TCGA-S9	TCGA-S9	LGG	28	FEMALE	WHITE		Astrocyto	G2	2006	-10342	Alive	TUMOR F	3013					970	Partial Remission/Response		0	3013	0	3013			1	970
TCGA-S9	TCGA-S9	LGG	46	MALE	WHITE		Astrocyto	G3	2012	-17134	Alive	WITH TUM	914						Partial Remission/Response		0	914	0	914			0	914
TCGA-S9	TCGA-S9	LGG	27	MALE	WHITE		Astrocyto	G2	2006	-10108	Alive	WITH TUM	3000					144	Progressive Disease		0	3000	0	3000			1	144
TCGA-S9	TCGA-S9	LGG	44	FEMALE	WHITE		Astrocyto	G3	2004	-16275	Dead	WITH TUMOR		961				698	Progressive Disease		1	961	1	961			1	698
TCGA-S9	TCGA-S9	LGG	70	MALE	WHITE		Astrocyto	G3	2012	-25570	Alive	WITH TUM	569					129	Progressive Disease		0	569	0	569			1	129
TCGA-S9	TCGA-S9	LGG	40	MALE	WHITE		Astrocyto	G3	2013	-14858	Alive	WITH TUM	623						Partial Remission/Response		0	623	0	623			0	623
TCGA-TM	TCGA-TM	LGG	43	FEMALE	WHITE		Astrocyto	G3	2009	-16021	Dead	WITH TUMOR		1666				713	Complete Remission/Response		1	1666	1	1666	1	713	1	713
TCGA-TM	TCGA-TM	LGG	39	FEMALE	WHITE		Astrocyto	G2	2010	-14603	Alive	TUMOR F	1470						Complete Remission/Response		0	1470	0	1470	0	1470	0	1470
TCGA-TM	TCGA-TM	LGG	30	MALE	WHITE		Oligoastr	G2	2010	-11286	Alive	TUMOR F	1500						Complete Remission/Response		0	1500	0	1500	0	1500	0	1500
TCGA-TM	TCGA-TM	LGG	44	MALE	WHITE		Astrocyto	G2	2011	-16415	Alive	TUMOR F	1058						Complete Remission/Response		0	1058	0	1058	0	1058	0	1058
TCGA-TM	TCGA-TM	LGG	41	FEMALE	WHITE		Astrocyto	G2	2009	-15178	Alive	WITH TUM	1989					924	Complete Remission/Response		0	1989	0	1989	1	924	1	924
TCGA-TM	TCGA-TM	LGG	41	FEMALE	WHITE		Astrocyto	G2	2009	-15178	Alive	WITH TUM	1989					924	Complete Remission/Response		0	1989	0	1989	1	924	1	924
TCGA-TM	TCGA-TM	LGG	40	MALE	WHITE		Astrocyto	G3	2012	-14933	Dead	WITH TUMOR		758					Progressive Disease		1	758	1	758			1	758
TCGA-TM	TCGA-TM	LGG	32	MALE	WHITE		Astrocyto	G2	2012	-11849	Dead	WITH TUMOR		492					Progressive Disease		1	492	1	492			1	492
TCGA-TM	TCGA-TM	LGG	48	MALE	WHITE		Astrocyto	G3	2009	-17744	Alive	TUMOR F	1796						Complete Remission/Response		0	1796	0	1796	0	1796	0	1796
TCGA-TM	TCGA-TM	LGG	54	FEMALE	WHITE		Oligoden	G3	2010	-20014	Alive	TUMOR F	1236						Complete Remission/Response		0	1236	0	1236	0	1236	0	1236
TCGA-TM	TCGA-TM	LGG	44	FEMALE	WHITE		Oligoastr	G3	2012	-16277	Alive	TUMOR F	926						Complete Remission/Response		0	926	0	926	0	926	0	926
TCGA-TM	TCGA-TM	LGG	30	MALE	WHITE		Astrocyto	G3	2012	-11221	Alive	TUMOR F	854						Complete Remission/Response		0	854	0	854	0	854	0	854
TCGA-TM	TCGA-TM	LGG	63	MALE	WHITE		Oligoden	G3	2012	-23091	Alive	WITH TUM	735					454	Progressive Disease		0	735	0	735			1	454
TCGA-TM	TCGA-TM	LGG	31	MALE	WHITE		Oligoastr	G2	2010	-11498	Dead	WITH TUMOR		1242				987	Complete Remission/Response		1	1242	1	1242	1	987	1	987
TCGA-TM	TCGA-TM	LGG	40	MALE	WHITE		Oligoden	G3	2012	-14660	Alive	TUMOR F	754						Complete Remission/Response		0	754	0	754	0	754	0	754
TCGA-TM	TCGA-TM	LGG	61	FEMALE	WHITE		Oligoden	G3	2011	-22327	Dead	WITH TUMOR		1011					Complete Remission/Response		1	1011	1	1011			1	1011
TCGA-TM	TCGA-TM	LGG	31	MALE	WHITE		Astrocyto	G2	2012	-11668	Alive	TUMOR F	772					455	Complete Remission/Response		0	772	0	772	1	455	1	455
TCGA-TM	TCGA-TM	LGG	46	MALE	WHITE		Oligoden	G2	2012	-16843	Alive	TUMOR F	566						Complete Remission/Response		0	566	0	566	0	566	0	566
TCGA-TM	TCGA-TM	LGG	36	MALE	WHITE		Oligoden	G3	2013	-13424	Alive	TUMOR F	454						Complete Remission/Response		0	454	0	454	0	454	0	454
TCGA-TM	TCGA-TM	LGG	19	MALE	WHITE		Oligoastr	G2	2012	-6994	Alive	WITH TUM	724						Stable Disease		0	724	0	724			0	724
TCGA-TO	TCGA-TO	LGG	27	FEMALE	WHITE		Oligoden	G3	2012	-9919	Alive	WITH TUM	650						Partial Remission/Response		0	650	0	650			0	650
TCGA-TO	TCGA-TO	LGG	36	MALE	WHITE		Oligoastr	G2	2012	-13227	Alive	WITH TUM	599						Partial Remission/Response		0	599	0	599			0	599
TCGA-TO	TCGA-TO	LGG	39	MALE	BLACK OR AFRICAN AMERICAN		Oligoastr	G2	2013	-14524	Alive	WITH TUM	486						Partial Remission/Response		0	486	0	486			0	486
TCGA-TO	TCGA-TO	LGG	37	FEMALE	WHITE		Oligoden	G2	2012	-13799	Alive	TUMOR F	62						[Unknown]		0	62	0	62			0	62
TCGA-TO	TCGA-TO	LGG	25	FEMALE	WHITE		Oligoastr	G2	2011	-9209	Alive	WITH TUM	1229						Stable Disease		0	1229	0	1229			0	1229
TCGA-TQ	TCGA-TQ	LGG	29	MALE	WHITE		Oligoastr	G2	2010	-10742	Alive	TUMOR F	1341					858	Complete Remission/Response		0	1341	0	1341	1	858	1	858
TCGA-TO	TCGA-TO	LGG	29	MALE	WHITE		Oligoastr	G2	2010	-10742	Alive	TUMOR F	1341					858	Complete Remission/Response		0	1341	0	1341	1	858	1	858
TCGA-TO	TCGA-TO	LGG	41	FEMALE	WHITE		Oligoastr	G3	2011	-15154	Alive	WITH TUM	1116						Partial Remission/Response		0	1116	0	1116			0	1116
TCGA-TO	TCGA-TO	LGG	32	MALE	WHITE		Oligoden	G2	2011	-11823	Alive	WITH TUM	1026						Partial Remission/Response		0	1026	0	1026			0	1026
TCGA-TO	TCGA-TO	LGG	29	MALE	BLACK OR AFRICAN AMERICAN		Oligoastr	G2	2011	-10890	Alive	WITH TUM	949						Partial Remission/Response		0	949	0	949			0	949
TCGA-TO	TCGA-TO	LGG	66	MALE	BLACK OR AFRICAN AMERICAN		Oligoastr	G2	2012	-24109	Alive	WITH TUM	914						Partial Remission/Response		0	914	0	914			0	914
TCGA-TO	TCGA-TO	LGG	38	FEMALE	WHITE		Oligoden	G2	2012	-14187	Alive	TUMOR F	795						Complete Remission/Response		0	795	0	795	0	795	0	795
TCGA-TO	TCGA-TO	LGG	38	MALE	WHITE		Oligoastr	G2	2012	-14022	Alive	TUMOR F	787						Partial Remission/Response		0	787	0	787			0	787
TCGA-TO	TCGA-TO	LGG	25	FEMALE	WHITE		Oligoden	G2	2012	-9172	Alive	TUMOR F	-1						Complete Remission/Response		0	0	0	0	0	0	0	0
TCGA-TQ	TCGA-TQ	LGG	51	MALE	WHITE		Oligoden	G2	2011	-18990	Alive	WITH TUM	1032						Partial Remission/Response		0	1032	0	1032			0	1032
TCGA-TO	TCGA-TO	LGG	27	MALE	WHITE		Astrocyto	G2	2009	-10167	Alive	WITH TUM	1868					530	Partial Remission/Response		0	1868	0	1868			1	530
TCGA-TO	TCGA-TO	LGG	27	MALE	WHITE		Astrocyto	G2	2009	-10167	Alive	WITH TUM	1868					530	Partial Remission/Response		0	1868	0	1868			1	530
TCGA-TO	TCGA-TO	LGG	32	MALE	WHITE		Oligoden	G2	2010	-11804	Dead	WITH TUMOR		821				511	Progressive Disease		1	821	1	821			1	511
TCGA-TO	TCGA-TO	LGG	42	FEMALE	WHITE		Oligoden	G2	2010	-15629	Dead	WITH TUMOR		954				455	Partial Remission/Response		1	954	1	954			1	455
TCGA-TO	TCGA-TO	LGG	42	FEMALE	WHITE		Oligoden	G2	2010	-15629	Dead	WITH TUMOR		954				455	Partial Remission/Response		1	954	1	954			1	455
TCGA-VM	TCGA-VM	LGG	50	FEMALE	WHITE		Oligoden	G2	2011	-18494	Alive		1397					456	[Unknown]		0	1397	0	1397			1	456
TCGA-VM	TCGA-VM	LGG	37	FEMALE	[Not Evaluated]		Astrocyto	G2	2011	-13663	Alive	TUMOR F	1314						Complete Remission/Response		0	1314	0	1314	0	1314	0	1314
TCGA-VM	TCGA-VM	LGG	54	MALE	[Unknown]		Oligoden	G2	2011	-19991	Alive	WITH TUM	411						[Unknown]		0	411	0	411			0	411
TCGA-VM	TCGA-VM	LGG	33	MALE	[Unknown]		Oligoden	G3	2011	-12340	Alive	TUMOR F	3						[Unknown]		0	3	0	3			0	3
TCGA-VM	TCGA-VM	LGG	58	MALE	WHITE		Astrocyto	G3	2012	-21200	Dead	WITH TUMOR		240					[Unknown]		1	240	1	240			1	240
TCGA-VM	TCGA-VM	LGG	25	MALE	WHITE		Oligoden	G2	2011	-9363	Alive	WITH TUM	1191					815	[Unknown]		0	1191	0	1191			1	815
TCGA-VM	TCGA-VM	LGG	44	FEMALE	WHITE		Astrocyto	G3	2013	-16334	Alive	TUMOR F	609						[Unknown]		0	609	0	609			0	609
TCGA-VM	TCGA-VM	LGG	24	FEMALE	WHITE		Astrocyto	G2	2013	-9005	Alive	TUMOR F	714						Complete Remission/Response		0	714	0	714	0	714	0	714
TCGA-VV	TCGA-VV	LGG	44	MALE	WHITE		Oligoastr	G3	2011	-16163	Alive	TUMOR F	1127						Stable Disease		0	1127	0	1127			0	1127
TCGA-VV	TCGA-VV	LGG	36	FEMALE	[Not Evaluated]		Astrocyto	G3	2013	-13453	Alive	TUMOR F	487						[Unknown]		0	487	0	487			0	487
TCGA-VW	TCGA-VW	LGG	35	FEMALE	ASIAN		Oligoden	G3	2011	-12820	Alive	WITH TUM	706						Stable Disease		0	706	0	706			0	706
TCGA-VW	TCGA-VW	LGG	66	MALE	WHITE		Astrocyto	G3	2013	-24411	Dead	WITH TUMOR		245					Stable Disease		1	245	1	245			1	245
TCGA-WS	TCGA-WS	LGG	47	MALE	WHITE		Oligoden	G2	2010		Alive	WITH TUM	1553						Stable Disease		0	1553	0	1553			0	1553
TCGA-WH	TCGA-WH	LGG	65	MALE	WHITE		Astrocyto	G2	2013	-24055	Alive	WITH TUM	405						Stable Disease		0	405	0	405			0	405
TCGA-WY	TCGA-WY	LGG	32	FEMALE	WHITE		Astrocyto	G3	2010	-11855	Alive	TUMOR F	1337						Complete Remission/Response		0	1337	0	1337	0	1337	0	1337
TCGA-WY	TCGA-WY	LGG	34	FEMALE	WHITE		Astrocyto	G2	2010	-12631	Alive	WITH TUM	1213						Stable Disease		0	1213	0	1213			0	1213
TCGA-WY	TCGA-WY	LGG	20	MALE	WHITE		Astrocyto	G2	2010	-7380	Alive	TUMOR F	1320						Partial Remission/Response		0	1320	0	1320			0	1320
TCGA-WY	TCGA-WY	LGG	24	MALE	WHITE		Astrocyto	G2	2010	-8817	Alive	TUMOR F	1393						Complete Remission/Response		0	1393	0	1393	0	1393	0	1393
TCGA-WY	TCGA-WY	LGG	36	MALE	WHITE		Astrocyto	G2	2010	-13506	Alive	WITH TUM	1426					809	Progressive Disease		0	1426	0	1426			1	809
TCGA-WY	TCGA-WY	LGG	60	MALE	WHITE		Oligoastr	G2	2010	-21979	Alive	WITH TUM	1147					887	Partial Remission/Response		0	1147	0	1147			1	887
TCGA-WY	TCGA-WY	LGG	48	FEMALE	WHITE		Oligoastr	G2	2011	-17878	Alive	WITH TUM	633					532	Partial Remission/Response		0	633	0	633			1	532
TCGA-2V	TCGA-2V	LIHC		MALE	ASIAN	Stage II	Hepatoce	G3			Alive	WITH TUMOR								R0	0		0		0		0	
TCGA-2Y	TCGA-2Y	LIHC	58	MALE	WHITE	[Discrepancy]	Hepatoce	G2	2006	-21318	Dead	WITH TUMOR		724	Extrahepa	Lung		102		R0	1	724	1	724	1	102	1	102
TCGA-2Y	TCGA-2Y	LIHC	51	MALE	WHITE	Stage I	Hepatoce	G2	2006	-18768	Dead	WITH TUMOR		1624	Locoregic	Liver		1083		R0	1	1624	1	1624	1	1083	1	1083
TCGA-2Y	TCGA-2Y	LIHC	55	FEMALE	WHITE	Stage I	Hepatoce	G2	2009	-20187	Alive	TUMOR F	1939							R0	0	1939	0	1939	0	1939	0	1939
TCGA-2Y	TCGA-2Y	LIHC	54	FEMALE	WHITE	Stage I	Hepatoce	G1	2007	-20011	Dead	WITH TUMOR		2532	New Prim	Other, sp	Renal met	1745		R0	1	2532	1	2532	0	2532	1	1745
TCGA-2Y	TCGA-2Y	LIHC	64	MALE	WHITE	Stage I	Hepatoce	G2	2007	-23587	Dead	WITH TUMOR		1271	Locoregic	Liver		1229		R0	1	1271	1	1271	1	1229	1	1229
TCGA-2Y	TCGA-2Y	LIHC	68	MALE	WHITE	Stage I	Hepatoce	G2	2007	-24963	Alive	WITH TUM	2442		Locoregic	Liver		2133		R1	0	2442	0	2442			1	2133
TCGA-2Y	TCGA-2Y	LIHC	64	FEMALE	WHITE	Stage II	Hepatoce	G3	2007	-23435	Dead	WITH TUMOR		757	Extrahepa	Lung		637		R0	1	757	1	757	1	637	1	637
TCGA-2Y	TCGA-2Y	LIHC	82	FEMALE	WHITE	Stage II	Hepatoce	G2	2007	-30082	Dead	WITH TUMOR		848	Locoregic	Liver		719		R0	1	848	1	848	1	719	1	719
TCGA-2Y	TCGA-2Y	LIHC	49	MALE	WHITE	Stage IIIA	Hepatoce	G1	2004	-18109	Alive	TUMOR F	3675							R0	0	3675	0	3675	0	3675	0	3675
TCGA-2Y	TCGA-2Y	LIHC	58	MALE	WHITE	Stage I	Hepatoce	G2	2010	-21222	Dead	WITH TUMOR		1229	Locoregic	Liver		321		R0	1	1229	1	1229	1	321	1	321
TCGA-2Y	TCGA-2Y	LIHC	64	FEMALE	WHITE	Stage I	Hepatoce	G3	2010	-23653	Alive	TUMOR F	1731							R0	0	1731	0	1731	0	1731	0	1731
TCGA-2Y	TCGA-2Y	LIHC	45	MALE	WHITE	Stage II	Hepatoce	G1	2010	-16443	Alive	TUMOR F	1516		Locoregic	Liver		22		R0	0	1516	0	1516	1	22	1	22
TCGA-2Y	TCGA-2Y	LIHC	68	MALE	BLACK OI	Stage I	Hepatoce	G2	2010	-25096	Alive	TUMOR F	1452							R0	0	1452	0	1452	0	1452	0	1452
TCGA-2Y	TCGA-2Y	LIHC	59	FEMALE	WHITE	Stage I	Hepatoce	G3	2010	-21818	Dead	WITH TUMOR		555	Locoregic	Liver		258		R0	1	555	1	555	1	258	1	258
TCGA-2Y	TCGA-2Y	LIHC	68	FEMALE	WHITE	Stage I	Hepatoce	G2	2011	-24982	Alive		357							R0	0	357	0	357	0	357	0	357
TCGA-2Y	TCGA-2Y	LIHC	81	FEMALE	WHITE	Stage I	Hepatoce	G2	2011	-29752	Alive	WITH TUM	1168		Locoregic	Liver		1117		R0	0	1168	0	1168	1	1117	1	1117
TCGA-2Y	TCGA-2Y	LIHC	85	FEMALE	WHITE	[Discrepancy]	Hepatoce	G2	2011	-31322	Dead	WITH TUMOR		633	Extrahepa	Other, sp	Kidney	398		R0	1	633	1	633	1	398	1	398
TCGA-2Y	TCGA-2Y	LIHC	70	MALE	WHITE	Stage I	Hepatoce	G2	2011	-25670	Alive	WITH TUM	697		Locoregic	Liver		59		R0	0	697	0	697	1	59	1	59
TCGA-2Y	TCGA-2Y	LIHC	70	MALE	WHITE	Stage II	Hepatoce	G2	2012	-25892	Dead	WITH TUMOR		36						R0	1	36	1	36			1	36
TCGA-2Y	TCGA-2Y	LIHC	66	MALE	[Not Evalu	Stage I	Hepatoce	G2	2012	-24359	Alive	TUMOR F	260							R0	0	260	0	260	0	260	0	260
TCGA-3K	TCGA-3K	LIHC	65	MALE	BLACK OI	Stage IIIB	Hepatoce	G1	2013	-24046	Alive	WITH TUM	396		Locoregic	Liver		151		RX	0	396	0	396			1	151
TCGA-4R	TCGA-4R	LIHC	66	MALE	WHITE	Stage II	Hepatoce	G2	2013	-24279	Dead	WITH TUMOR		262	Locoregic	Liver		158		R0	1	262	1	262	1	158	1	158
TCGA-5C	TCGA-5C	LIHC	58	MALE	WHITE	Stage II	Hepatoce	G2	2013	-21273	Alive	TUMOR F	328							R0	0	328	0	328	0	328	0	328
TCGA-5C	TCGA-5C	LIHC	70	MALE	WHITE	Stage I	Hepatoce	G2	2013	-25608	Alive	TUMOR F	322							R0	0	322	0	322	0	322	0	322
TCGA-5C	TCGA-5C	LIHC	61	MALE	ASIAN	Stage II	Hepatoch	G1	2013	-22552	Alive	TUMOR F	20							R0	0	20	0	20	0	20	0	20
TCGA-5R	TCGA-5R	LIHC	57	MALE	WHITE	Stage II	Hepatoce	G2	2013	-21073	Alive	TUMOR F	520							R0	0	520	0	520	0	520	0	520
TCGA-5R	TCGA-5R	LIHC	17	FEMALE	WHITE	Stage IIIA	Hepatoce	G3	2013	-6545	Alive	TUMOR F	449							R0	0	449	0	449	0	449	0	449
TCGA-5R	TCGA-5R	LIHC	65	FEMALE	WHITE	Stage II	Hepatoce	G2	2013	-24020	Dead	TUMOR FREE		46						R0	1	46	0	46	0	46	0	46
TCGA-BC	TCGA-BC	LIHC	74	FEMALE	WHITE	Stage IIIA	Hepatoce	G3	2005	-27162	Dead	WITH TUMOR		1490	Extrahepa	Lymph Node(s)		475		R1	1	1490	1	1490			1	475
TCGA-BC	TCGA-BC	LIHC	73	MALE	WHITE	Stage IIIA	Hepatoce	G3	2009	-26795	Alive	WITH TUM	849							R1	0	849	0	849			0	849
TCGA-BC	TCGA-BC	LIHC	72	FEMALE	WHITE		Hepatocellular Carci		1998	-26400	Dead	WITH TUMOR		1135	Intrahepatic Recurrence			340		R1	1	1135	1	1135			1	340
TCGA-BC	TCGA-BC	LIHC	72	FEMALE	WHITE		Hepatocellular Carci		1998	-26400	Dead	WITH TUMOR		1135	Intrahepatic Recurrence			340		R1	1	1135	1	1135			1	340
TCGA-BC	TCGA-BC	LIHC	66	FEMALE	WHITE		Hepatoce	G2	1999	-24274	Dead	WITH TUMOR		308	Intrahepatic Recurrence			199		R0	1	308	1	308	1	199	1	199
TCGA-BC	TCGA-BC	LIHC	66	FEMALE	WHITE		Hepatoce	G2	1999	-24274	Dead	WITH TUMOR		308	Intrahepatic Recurrence			199		R0	1	308	1	308	1	199	1	199
TCGA-BC	TCGA-BC	LIHC	81	MALE	WHITE		Hepatoce	G1	1999	-29842	Dead	WITH TUMOR		1423	Intrahepatic Recurrence			1130		R0	1	1423	1	1423	1	1130	1	1130
TCGA-BC	TCGA-BC	LIHC	81	MALE	WHITE		Hepatoce	G1	1999	-29842	Dead	WITH TUMOR		1423	Intrahepatic Recurrence			1130		R0	1	1423	1	1423	1	1130	1	1130
TCGA-BC	TCGA-BC	LIHC	76	MALE	WHITE		Hepatoce	G1	2000	-27944	Dead	WITH TUMOR		837	Extrahepa	Pulmonary		598		R1	1	837	1	837			1	598
TCGA-BC	TCGA-BC	LIHC	76	MALE	WHITE		Hepatoce	G1	2000	-27944	Dead	WITH TUMOR		837	Extrahepa	Pulmonary		598		R1	1	837	1	837			1	598
TCGA-BC	TCGA-BC	LIHC	69	MALE	WHITE		Hepatoce	G2	2000	-25305	Dead	WITH TUMOR		837	Intrahepatic Recurrence			586		R1	1	837	1	837			1	586
TCGA-BC	TCGA-BC	LIHC	69	MALE	WHITE		Hepatoce	G2	2000	-25305	Dead	WITH TUMOR		837	Intrahepatic Recurrence			586		R1	1	837	1	837			1	586
TCGA-BC	TCGA-BC	LIHC	50	MALE	ASIAN		Hepatoce	G3	2002	-18562	Dead	WITH TUMOR		91						R1	1	91	1	91			1	91
TCGA-BC	TCGA-BC	LIHC	50	MALE	ASIAN		Hepatoce	G3	2002	-18562	Dead	WITH TUMOR		91						R1	1	91	1	91			1	91
TCGA-BC	TCGA-BC	LIHC	52	FEMALE	WHITE	Stage IIIA	Hepatoce	G2	2002	-19140	Dead	TUMOR FREE		770						R0	1	770	0	770	0	770	0	770
TCGA-BC	TCGA-BC	LIHC	52	FEMALE	WHITE	Stage IIIA	Hepatoce	G2	2002	-19140	Dead	TUMOR FREE		770						R0	1	770	0	770	0	770	0	770
TCGA-BC	TCGA-BC	LIHC	76	MALE	WHITE		Hepatoce	G3	2002	-28049	Dead	WITH TUMOR		711	Intrahepatic Recurrence			400		R0	1	711	1	711	1	400	1	400
TCGA-BC	TCGA-BC	LIHC	76	MALE	WHITE		Hepatoce	G3	2002	-28049	Dead	WITH TUMOR		711	Intrahepatic Recurrence			400		R0	1	711	1	711	1	400	1	400
TCGA-BC	TCGA-BC	LIHC	62	FEMALE	WHITE	Stage I	Hepatoce	G2	2003	-22913	Dead	TUMOR FREE		34						R0	1	34	0	34	0	34	0	34
TCGA-BC	TCGA-BC	LIHC	62	FEMALE	WHITE	Stage I	Hepatoce	G2	2003	-22913	Dead	TUMOR FREE		34						R0	1	34	0	34	0	34	0	34
TCGA-BC	TCGA-BC	LIHC	51	FEMALE	BLACK OR AFRICAN AMERICAN		Hepatoce	G1	2003	-18951	Dead	WITH TUMOR		2116	Extrahepa	Other, sp	spleen	2057		R0	1	2116	1	2116	1	2057	1	2057
TCGA-BC	TCGA-BC	LIHC	51	FEMALE	BLACK OR AFRICAN AMERICAN		Hepatoce	G1	2003	-18951	Dead	WITH TUMOR		2116	Extrahepa	Other, sp	spleen	2057		R0	1	2116	1	2116	1	2057	1	2057
TCGA-BC	TCGA-BC	LIHC	80	MALE	WHITE		Hepatoce	G2	2009	-29511	Dead	TUMOR FREE		153						R0	1	153	0	153	0	153	0	153
TCGA-BC	TCGA-BC	LIHC	80	MALE	WHITE		Hepatoce	G2	2009	-29511	Dead	TUMOR FREE		153						R0	1	153	0	153	0	153	0	153
TCGA-BC	TCGA-BC	LIHC	62	FEMALE	WHITE	Stage IIIA	Hepatoce	G2	2008	-22968	Alive	TUMOR F	1351							R0	0	1351	0	1351	0	1351	0	1351
TCGA-BC	TCGA-BC	LIHC	62	FEMALE	WHITE	Stage IIIA	Hepatoce	G2	2008	-22968	Alive	TUMOR F	1351							R0	0	1351	0	1351	0	1351	0	1351
TCGA-BC	TCGA-BC	LIHC	75	FEMALE	WHITE	Stage II	Hepatoce	G3	2010	-27411	Dead	TUMOR FREE		1397						R0	1	1397	0	1397	0	1397	0	1397
TCGA-BC	TCGA-BC	LIHC	66	FEMALE	WHITE	Stage I	Hepatoce	G2	2011	-24328	Alive	TUMOR F	8							R0	0	8	0	8	0	8	0	8
TCGA-BC	TCGA-BC	LIHC	68	FEMALE	WHITE	Stage II	Hepatoce	G3	2011	-25061	Alive	WITH TUM	680		Intrahepa	Liver		209		R0	0	680	0	680	1	209	1	209
TCGA-BC	TCGA-BC	LIHC	69	MALE	WHITE	Stage IIIA	Hepatoce	G3	2012	-25503	Dead	TUMOR FREE		547	Locoregional Recurrence			384		R1	1	547	0	547			1	384
TCGA-BC	TCGA-BC	LIHC	64	MALE	WHITE	Stage II	Hepatoce	G3	2013	-23380	Alive	TUMOR F	444							R0	0	444	0	444	0	444	0	444
TCGA-BC	TCGA-BC	LIHC	69	MALE	WHITE	Stage I	Hepatoce	G1	2013	-25370	Alive	TUMOR F	387							R0	0	387	0	387	0	387	0	387
TCGA-BC	TCGA-BC	LIHC	66	FEMALE	WHITE	Stage IIIC	Hepatoce	G3	2013	-24189	Alive	WITH TUM	562		Locoregic	Liver		220		R0	0	562	0	562	1	220	1	220
TCGA-BD	TCGA-BD	LIHC	69	MALE	WHITE		Hepatoce	G2	2011	-25489	Alive	WITH TUM	1363		Intrahepa	Liver		415		R0	0	1363	0	1363	1	415	1	415
TCGA-BD	TCGA-BD	LIHC	69	MALE	WHITE		Hepatoce	G2	2011	-25489	Alive	WITH TUM	1363		Intrahepa	Liver		415		R0	0	1363	0	1363	1	415	1	415
TCGA-BD	TCGA-BD	LIHC	75	FEMALE	BLACK OI	Stage I	Hepatoce	G2	2010	-27756	Alive	TUMOR F	409							R0	0	409	0	409	0	409	0	409
TCGA-BD	TCGA-BD	LIHC	75	FEMALE	BLACK OI	Stage I	Hepatoce	G2	2010	-27756	Alive	TUMOR F	409							R0	0	409	0	409	0	409	0	409
TCGA-BD	TCGA-BD	LIHC	62	MALE	WHITE	Stage II	Hepatoce	G2	2011	-22657	Alive	WITH TUM	1115		Locoregic	Liver		226		R0	0	1115	0	1115	1	226	1	226
TCGA-BW	TCGA-BW	LIHC	50	MALE	BLACK OI	Stage IIIA	Hepatoce	G2	2010	-18510	Alive	TUMOR F	20							RX	0	20	0	20			0	20
TCGA-BW	TCGA-BW	LIHC	26	FEMALE	WHITE	Stage IV	Hepatoce	G3	2010	-9809	Alive	WITH TUM	0		Extrahepa	Liver		102		RX	0	0	0	0			1	102
TCGA-BW	TCGA-BW	LIHC	63	MALE	WHITE	Stage I	Hepatoce	G3	2010	-23353	Alive	TUMOR F	0							RX	0	0	0	0			0	0
TCGA-CC	TCGA-CC	LIHC	48	MALE	ASIAN	Stage II	Hepatoce	G2	2010	-17586	Dead	TUMOR FREE		129						RX	1	129	0	129			0	129
TCGA-CC	TCGA-CC	LIHC	60	FEMALE	ASIAN	Stage IIIC	Hepatoce	G2	2010	-21946	Alive	WITH TUM	250		Locoregic	Liver		180		R0	0	250	0	250	1	180	1	180
TCGA-CC	TCGA-CC	LIHC	61	FEMALE	ASIAN	Stage IIIC	Hepatoce	G1	2010	-22345	Dead	TUMOR FREE		87						RX	1	87	0	87			0	87
TCGA-CC	TCGA-CC	LIHC	44	MALE	ASIAN	Stage II	Hepatoce	G2	2010	-16373	Dead	TUMOR FREE		97						RX	1	97	0	97			0	97
TCGA-CC	TCGA-CC	LIHC	67	MALE	ASIAN	Stage IIIC	Hepatoce	G1	2010	-24787	Dead	TUMOR FREE		103							1	103	0	103			0	103
TCGA-CC	TCGA-CC	LIHC	35	MALE	ASIAN	Stage IIIA	Hepatoce	G1	2010	-12863	Dead	TUMOR FREE		129						RX	1	129	0	129			0	129
TCGA-CC	TCGA-CC	LIHC	71	MALE	ASIAN	Stage IIIA	Hepatoce	G2	2010	-25979	Dead	TUMOR FREE		102						RX	1	102	0	102			0	102
TCGA-CC	TCGA-CC	LIHC	24	FEMALE	ASIAN	Stage IIIA	Hepatoce	G1	2010	-9107	Alive	WITH TUM	219		New Prim	Lung		145		RX	0	219	0	219			1	145
TCGA-CC	TCGA-CC	LIHC	50	MALE	ASIAN	Stage IIIA	Hepatoce	G3	2010	-18553	Dead	TUMOR FREE		101							1	101	0	101			0	101
TCGA-CC	TCGA-CC	LIHC	45	MALE	ASIAN	Stage IIIA	Hepatoce	G3	2011	-16460	Dead	WITH TUMOR		300	Locoregional Recurrence			219		R0	1	300	1	300	1	219	1	219
TCGA-CC	TCGA-CC	LIHC	61	MALE	ASIAN	Stage IIIA	Hepatoce	G2	2011	-22375	Dead	WITH TUMOR		303	Locoregional Recurrence			261		R0	1	303	1	303	1	261	1	261
TCGA-CC	TCGA-CC	LIHC	36	MALE	ASIAN	Stage IIIA	Hepatoce	G1	2011	-13255	Dead	WITH TUMOR		315	New Prim	Lung		260		R0	1	315	1	315	0	315	1	260
TCGA-CC	TCGA-CC	LIHC	54	MALE	ASIAN	Stage IIIA	Hepatoce	G2	2011	-20067	Alive	WITH TUM	363		Locoregic	Liver		297		R0	0	363	0	363	1	297	1	297
TCGA-CC	TCGA-CC	LIHC	63	MALE	ASIAN	Stage IIIA	Hepatoce	G3	2012	-23156	Dead	WITH TUMOR		347	New Prim	Lung		312		R0	1	347	1	347	0	347	1	312
TCGA-CC	TCGA-CC	LIHC	45	MALE	ASIAN	Stage IIIA	Hepatoce	G2	2012	-16702	Dead	WITH TUMOR		304	Locoregional Recurrence			182		R0	1	304	1	304	1	182	1	182
TCGA-CC	TCGA-CC	LIHC	48	MALE	ASIAN	Stage IIIB	Hepatoce	G2	2012	-17805	Dead	WITH TUMOR		272	Locoregional Recurrence			175		R0	1	272	1	272	1	175	1	175
TCGA-CC	TCGA-CC	LIHC	57	MALE	ASIAN	Stage IIIA	Hepatoce	G2	2012	-20985	Dead	WITH TUMOR		217	New Prim	Lung		169		R0	1	217	1	217	0	217	1	169
TCGA-CC	TCGA-CC	LIHC	59	MALE	ASIAN	Stage IIIA	Hepatoce	G1	2013	-21845	Dead	WITH TUMOR		649	New Prim	Lung		254		R0	1	649	1	649	0	649	1	254
TCGA-CC	TCGA-CC	LIHC	47	MALE	ASIAN	Stage II	Hepatoce	G2	2013	-17453	Dead	WITH TUMOR		299	Locoregional Recurrence			195		R0	1	299	1	299	1	195	1	195
TCGA-CC	TCGA-CC	LIHC	58	MALE	ASIAN	Stage IIIA	Hepatoce	G1	2013	-21381	Alive	TUMOR F	365							R0	0	365	0	365	0	365	0	365
TCGA-CC	TCGA-CC	LIHC	54	MALE	ASIAN	Stage IIIA	Hepatoce	G3	2013	-20079	Alive	WITH TUM	399		Locoregional Recurrence			278		R0	0	399	0	399	1	278	1	278
TCGA-CC	TCGA-CC	LIHC	56	MALE	ASIAN	Stage II	Hepatoce	G3	2013	-20751	Alive	TUMOR F	382							R0	0	382	0	382	0	382	0	382
TCGA-CC	TCGA-CC	LIHC	59	MALE	ASIAN	Stage IIIA	Hepatoce	G3	2013	-21552	Dead	WITH TUMOR		262	Locoregional Recurrence			150		R0	1	262	1	262	1	150	1	150
TCGA-CC	TCGA-CC	LIHC	61	MALE	ASIAN	Stage IIIA	Hepatoce	G1	2013	-22319	Dead	WITH TUMOR		278	Locoregional Recurrence			179		R0	1	278	1	278	1	179	1	179
TCGA-CC	TCGA-CC	LIHC	18	MALE	ASIAN	Stage IIIC	Hepatoce	G1	2013	-6617	Dead	WITH TUMOR		300	Locoregional Recurrence			201		R0	1	300	1	300	1	201	1	201
TCGA-CC	TCGA-CC	LIHC	74	MALE	ASIAN	Stage IIIA	Hepatoce	G2	2013	-27334	Dead	WITH TUMOR		140	Locoregional Recurrence			83		R0	1	140	1	140	1	83	1	83
TCGA-CC	TCGA-CC	LIHC	39	FEMALE	ASIAN	Stage IIIA	Hepatoce	G3	2013	-14367	Dead	WITH TUMOR		344	Locoregional Recurrence			301		R0	1	344	1	344	1	301	1	301
TCGA-CC	TCGA-CC	LIHC	51	FEMALE	ASIAN	Stage II	Hepatoce	G2	2013	-18935	Dead	WITH TUMOR		279	Locoregional Recurrence			149		R0	1	279	1	279	1	149	1	149
TCGA-CC	TCGA-CC	LIHC	55	MALE	ASIAN	Stage II	Hepatoce	G2	2013	-20157	Alive	WITH TUM	211		New Prim	Lung		109		R0	0	211	0	211	0	211	1	109
TCGA-CC	TCGA-CC	LIHC	52	FEMALE	ASIAN	Stage IIIA	Hepatoce	G2	2013	-19326	Alive	TUMOR F	0							R0	0	0	0	0	0	0	0	0
TCGA-CC	TCGA-CC	LIHC	57	MALE	ASIAN	Stage IIIA	Hepatoce	G2	2013	-21044	Alive	TUMOR F	0							R0	0	0	0	0	0	0	0	0
TCGA-CC	TCGA-CC	LIHC	68	MALE	ASIAN	Stage IIIA	Hepatoce	G2	2013	-24891	Alive	WITH TUM	248		Locoregional Recurrence			153		R0	0	248	0	248	1	153	1	153
TCGA-DD	TCGA-DD	LIHC	55	FEMALE	WHITE	Stage II	Hepatoce	G3	2006	-20188	Alive	WITH TUM	2425		Intrahepa	Liver		1676		R0	0	2425	0	2425	1	1676	1	1676
TCGA-DD	TCGA-DD	LIHC	55	FEMALE	WHITE	Stage II	Hepatoce	G3	2006	-20188	Alive	WITH TUM	2425		Intrahepa	Liver		1676		R0	0	2425	0	2425	1	1676	1	1676
TCGA-DD	TCGA-DD	LIHC	42	MALE	BLACK OI	Stage II	Hepatoce	G3	2005	-15410	Dead	TUMOR FREE		1149						R0	1	1149	0	1149	0	1149	0	1149
TCGA-DD	TCGA-DD	LIHC	42	MALE	BLACK OI	Stage II	Hepatoce	G3	2005	-15410	Dead	TUMOR FREE		1149						R0	1	1149	0	1149	0	1149	0	1149
TCGA-DD	TCGA-DD	LIHC	53	MALE	WHITE	Stage IIIA	Hepatoce	G2	2002	-19697	Dead	WITH TUMOR		2542	Intrahepa	Liver		469		R0	1	2542	1	2542	1	469	1	469
TCGA-DD	TCGA-DD	LIHC	53	MALE	WHITE	Stage IIIA	Hepatoce	G2	2002	-19697	Dead	WITH TUMOR		2542	Intrahepa	Liver		469		R0	1	2542	1	2542	1	469	1	469
TCGA-DD	TCGA-DD	LIHC	68	MALE	ASIAN	Stage IIIA	Hepatoce	G3	2002	-24934	Dead	TUMOR FREE		1622	Extrahepa	Other, sp	sternum	828		R0	1	1622	0	1622	1	828	1	828
TCGA-DD	TCGA-DD	LIHC	68	MALE	ASIAN	Stage IIIA	Hepatoce	G3	2002	-24934	Dead	TUMOR FREE		1622	Extrahepa	Other, sp	sternum	828		R0	1	1622	0	1622	1	828	1	828
TCGA-DD	TCGA-DD	LIHC	77	FEMALE	WHITE	Stage II	Hepatoce	G2	2003	-28406	Alive		3437		Extrahepa	Other, sp	lateral to	658		R0	0	3437	0	3437	1	658	1	658
TCGA-DD	TCGA-DD	LIHC	77	FEMALE	WHITE	Stage II	Hepatoce	G2	2003	-28406	Alive		3437		Extrahepa	Other, sp	lateral to	658		R0	0	3437	0	3437	1	658	1	658
TCGA-DD	TCGA-DD	LIHC	40	MALE	ASIAN	Stage IV	Hepatoce	G3	2003	-14613	Dead			223						R2	1	223		223			0	223
TCGA-DD	TCGA-DD	LIHC	40	MALE	ASIAN	Stage IV	Hepatoce	G3	2003	-14613	Dead			223						R2	1	223		223			0	223
TCGA-DD	TCGA-DD	LIHC	67	MALE	BLACK OI	Stage I	Hepatoce	G3	2004	-24646	Alive		79							R0	0	79	0	79	0	79	0	79
TCGA-DD	TCGA-DD	LIHC	67	MALE	BLACK OI	Stage I	Hepatoce	G3	2004	-24646	Alive		79							R0	0	79	0	79	0	79	0	79
TCGA-DD	TCGA-DD	LIHC	73	MALE	WHITE	Stage I	Hepatoce	G2	2005	-26857	Dead	TUMOR FREE		14						RX	1	14	0	14			0	14
TCGA-DD	TCGA-DD	LIHC	73	MALE	WHITE	Stage I	Hepatoce	G2	2005	-26857	Dead	TUMOR FREE		14						RX	1	14	0	14			0	14
TCGA-DD	TCGA-DD	LIHC	69	MALE	WHITE	Stage I	Hepatoce	G3	2006	-25403	Alive	TUMOR F	662							RX	0	662	0	662			0	662
TCGA-DD	TCGA-DD	LIHC	69	MALE	WHITE	Stage I	Hepatoce	G3	2006	-25403	Alive	TUMOR F	662							RX	0	662	0	662			0	662
TCGA-DD	TCGA-DD	LIHC	57	FEMALE	WHITE	Stage I	Hepatoce	G2	2006	-21003	Dead	WITH TUMOR		1560	Intrahepa	Liver		498		R0	1	1560	1	1560	1	498	1	498
TCGA-DD	TCGA-DD	LIHC	57	FEMALE	WHITE	Stage I	Hepatoce	G2	2006	-21003	Dead	WITH TUMOR		1560	Intrahepa	Liver		498		R0	1	1560	1	1560	1	498	1	498
TCGA-DD	TCGA-DD	LIHC	70	MALE	WHITE	Stage I	Hepatoce	G2	2002	-25808	Dead			2759	Intrahepa	Liver		720		R0	1	2759		2759	1	720	1	720
TCGA-DD	TCGA-DD	LIHC	70	MALE	WHITE	Stage I	Hepatoce	G2	2002	-25808	Dead			2759	Intrahepa	Liver		720		R0	1	2759		2759	1	720	1	720
TCGA-DD	TCGA-DD	LIHC	68	MALE	ASIAN	Stage II	Hepatoce	G2	2002	-24925	Alive	WITH TUM	2415		Locoregic	Liver		754		R0	0	2415	0	2415	1	754	1	754
TCGA-DD	TCGA-DD	LIHC	72	FEMALE		Stage I	Hepatoce	G2	2001	-26471	Alive	[Discrepar	2017		Intrahepa	Liver		505		R0	0	2017	0	2017	1	505	1	505
TCGA-DD	TCGA-DD	LIHC	72	FEMALE		Stage I	Hepatoce	G2	2001	-26471	Alive	[Discrepar	2017		Intrahepa	Liver		505		R0	0	2017	0	2017	1	505	1	505
TCGA-DD	TCGA-DD	LIHC	20	FEMALE	WHITE	Stage I	Hepatoce	G3	2004	-7363	Alive		602		Extrahepa	Other, sp	peritonea	299		R0	0	602	0	602	1	299	1	299
TCGA-DD	TCGA-DD	LIHC	20	FEMALE	WHITE	Stage I	Hepatoce	G3	2004	-7363	Alive		602		Extrahepa	Other, sp	peritonea	299		R0	0	602	0	602	1	299	1	299
TCGA-DD	TCGA-DD	LIHC	68	MALE	WHITE	Stage I	Hepatoce	G1	2006	-25039	Alive	TUMOR F	2301							R0	0	2301	0	2301	0	2301	0	2301
TCGA-DD	TCGA-DD	LIHC	68	MALE	WHITE	Stage I	Hepatoce	G1	2006	-25039	Alive	TUMOR F	2301							R0	0	2301	0	2301	0	2301	0	2301
TCGA-DD	TCGA-DD	LIHC	73	MALE	WHITE	Stage IIIA	Hepatoce	G3	2006	-26858	Dead	WITH TUMOR		349	Intrahepa	Liver		44		R0	1	349	1	349	1	44	1	44
TCGA-DD	TCGA-DD	LIHC	73	MALE	WHITE	Stage IIIA	Hepatoce	G3	2006	-26858	Dead	WITH TUMOR		349	Intrahepa	Liver		44		R0	1	349	1	349	1	44	1	44
TCGA-DD	TCGA-DD	LIHC	57	FEMALE	WHITE	Stage I	Hepatoce	G3	2007	-21165	Dead	WITH TUMOR		394						R0	1	394	1	394			1	394
TCGA-DD	TCGA-DD	LIHC	57	FEMALE	WHITE	Stage I	Hepatoce	G3	2007	-21165	Dead	WITH TUMOR		394						R0	1	394	1	394			1	394
TCGA-DD	TCGA-DD	LIHC	76	MALE	WHITE	Stage I	Hepatoce	G3	2007	-28108	Dead	WITH TUMOR		1372	Intrahepa	Liver		875		R0	1	1372	1	1372	1	875	1	875
TCGA-DD	TCGA-DD	LIHC	76	MALE	WHITE	Stage I	Hepatoce	G3	2007	-28108	Dead	WITH TUMOR		1372	Intrahepa	Liver		875		R0	1	1372	1	1372	1	875	1	875
TCGA-DD	TCGA-DD	LIHC	23	MALE	WHITE	Stage III	Hepatoce	G3	2008	-8499	Alive	WITH TUM	1495		Intrahepa	Liver		120		R0	0	1495	0	1495	1	120	1	120

TCGA-DD	TCGA-DD	LIHC	23	MALE	WHITE	Stage III	Hepatoce	G3	2008	-8499	Alive	WITH TUN	1495		Intrahepa	Liver		120	R0	0	1495	0	1495	1	120	1	120
TCGA-DD	TCGA-DD	LIHC	46	MALE	ASIAN	Stage I	Hepatoce	G2	2008	-17097	Alive	TUMOR F	183						R0	0	183	0	183	0	183	0	183
TCGA-DD	TCGA-DD	LIHC	46	MALE	ASIAN	Stage I	Hepatoce	G2	2008	-17097	Alive	TUMOR F	183						R0	0	183	0	183	0	183	0	183
TCGA-DD	TCGA-DD	LIHC	71	FEMALE	WHITE	Stage IIIC	Hepatoce	G2	2008	-26009	Dead	WITH TUMOR		1005	Intrahepa	Liver		263	R0	1	1005	1	1005	1	263	1	263
TCGA-DD	TCGA-DD	LIHC	71	FEMALE	WHITE	Stage IIIC	Hepatoce	G2	2008	-26009	Dead	WITH TUMOR		1005	Intrahepa	Liver		263	R0	1	1005	1	1005	1	263	1	263
TCGA-DD	TCGA-DD	LIHC	64	FEMALE	WHITE	Stage IVB	Hepatoce	G2	2008	-23437	Dead	WITH TUMOR		558	Locoregic	Liver		167	R0	1	558	1	558			1	167
TCGA-DD	TCGA-DD	LIHC	23	MALE	BLACK OI	Stage II	Hepatoce	G3	2009	-8573	Dead	WITH TUMOR		415					R0	1	415	1	415			1	415
TCGA-DD	TCGA-DD	LIHC	23	MALE	BLACK OI	Stage II	Hepatoce	G3	2009	-8573	Dead	WITH TUMOR		415					R0	1	415	1	415			1	415
TCGA-DD	TCGA-DD	LIHC	77	MALE	WHITE	Stage II	Hepatoce	G3	2000	-28254	Dead	WITH TUMOR		643	Extrahepa	Other, sp	second or	455	R0	1	643	1	643	1	455	1	455
TCGA-DD	TCGA-DD	LIHC	77	MALE	WHITE	Stage II	Hepatoce	G3	2000	-28254	Dead	WITH TUMOR		643	Extrahepa	Other, sp	second or	455	R0	1	643	1	643	1	455	1	455
TCGA-DD	TCGA-DD	LIHC	29	FEMALE	WHITE	Stage III	Hepatoce	G2	1995	-10699	Dead			827					R0	1	827		827	0	827	0	827
TCGA-DD	TCGA-DD	LIHC	29	FEMALE	WHITE	Stage III	Hepatoce	G2	1995	-10699	Dead			827					R0	1	827		827	0	827	0	827
TCGA-DD	TCGA-DD	LIHC	78	FEMALE	WHITE	Stage I	Hepatoce	G2	1998	-28528	Dead	WITH TUMOR		1694	Intrahepa	Liver		1032	R0	1	1694	1	1694	1	1032	1	1032
TCGA-DD	TCGA-DD	LIHC	78	FEMALE	WHITE	Stage I	Hepatoce	G2	1998	-28528	Dead	WITH TUMOR		1694	Intrahepa	Liver		1032	R0	1	1694	1	1694	1	1032	1	1032
TCGA-DD	TCGA-DD	LIHC	67	MALE	ASIAN	Stage I	Hepatoce	G3	2002	-24769	Dead	TUMOR FREE		171					R0	1	171	0	171	0	171	0	171
TCGA-DD	TCGA-DD	LIHC	43	FEMALE		Stage II	Hepatoce	G2	1996	-15930	Dead	TUMOR FREE		601					R0	1	601	0	601	0	601	0	601
TCGA-DD	TCGA-DD	LIHC	43	FEMALE		Stage II	Hepatoce	G2	1996	-15930	Dead	TUMOR FREE		601					R0	1	601	0	601	0	601	0	601
TCGA-DD	TCGA-DD	LIHC	70	MALE	WHITE	Stage I	Hepatoce	G2	2001	-25889	Dead	WITH TUMOR		785	New Primary Tumor			359	R0	1	785	1	785	0	785	1	359
TCGA-DD	TCGA-DD	LIHC	65	MALE		Stage IIIA	Hepatoce	G2	2001	-24046	Dead	TUMOR FREE		233					R0	1	233	0	233	0	233	0	233
TCGA-DD	TCGA-DD	LIHC	65	MALE		Stage IIIA	Hepatoce	G2	2001	-24046	Dead	TUMOR FREE		233					R0	1	233	0	233	0	233	0	233
TCGA-DD	TCGA-DD	LIHC	76	FEMALE	WHITE	Stage I	Hepatoce	G1	1998	-27930	Dead	TUMOR FREE		2131					R0	1	2131	0	2131	0	2131	0	2131
TCGA-DD	TCGA-DD	LIHC	76	FEMALE	WHITE	Stage I	Hepatoce	G1	1998	-27930	Dead	TUMOR FREE		2131					R0	1	2131	0	2131	0	2131	0	2131
TCGA-DD	TCGA-DD	LIHC	45	MALE	ASIAN	Stage I	Hepatoce	G2	1999	-16708	Dead			535					R0	1	535		535	0	535	0	535
TCGA-DD	TCGA-DD	LIHC	45	MALE	ASIAN	Stage I	Hepatoce	G2	1999	-16708	Dead			535					R0	1	535		535	0	535	0	535
TCGA-DD	TCGA-DD	LIHC	37	MALE	WHITE	Stage IIIA	Hepatoce	G3	1998	-13739	Dead	WITH TUMOR		612	Intrahepa	Liver		87	R0	1	612	1	612	1	87	1	87
TCGA-DD	TCGA-DD	LIHC	37	MALE	WHITE	Stage IIIA	Hepatoce	G3	1998	-13739	Dead	WITH TUMOR		612	Intrahepa	Liver		87	R0	1	612	1	612	1	87	1	87
TCGA-DD	TCGA-DD	LIHC	66	FEMALE	WHITE	Stage III	Hepatoce	G2	1996	-24288	Dead	WITH TUMOR		3125	Intrahepa	Liver		84	R0	1	3125	1	3125	1	84	1	84
TCGA-DD	TCGA-DD	LIHC	66	FEMALE	WHITE	Stage III	Hepatoce	G2	1996	-24288	Dead	WITH TUMOR		3125	Intrahepa	Liver		84	R0	1	3125	1	3125	1	84	1	84
TCGA-DD	TCGA-DD	LIHC	72	FEMALE	WHITE	Stage II	Hepatoce	G2	1997	-26319	Dead	TUMOR FREE		3258					R0	1	3258	0	3258	0	3258	0	3258
TCGA-DD	TCGA-DD	LIHC	72	FEMALE	WHITE	Stage II	Hepatoce	G2	1997	-26319	Dead	TUMOR FREE		3258					R0	1	3258	0	3258	0	3258	0	3258
TCGA-DD	TCGA-DD	LIHC	67	MALE	[Unknowr	Stage IIIB	Hepatoce	G3	1998	-24599	Dead	WITH TUMOR		419	Intrahepa	Liver		144	R0	1	419	1	419	1	144	1	144
TCGA-DD	TCGA-DD	LIHC	75	MALE	WHITE	Stage II	Hepatoce	G2	2001	-27524	Dead	TUMOR FREE		11					R0	1	11	0	11	0	11	0	11
TCGA-DD	TCGA-DD	LIHC	75	MALE	WHITE	Stage II	Hepatoce	G2	2001	-27524	Dead	TUMOR FREE		11					R0	1	11	0	11	0	11	0	11
TCGA-DD	TCGA-DD	LIHC	64	FEMALE	WHITE	Stage IVB	Hepatoce	G2	2001	-23526	Dead	WITH TUMOR		931	Extrahepa	Lung		289	R0	1	931	1	931			1	289
TCGA-DD	TCGA-DD	LIHC	67	FEMALE	WHITE	Stage IIIC	Hepatoce	G3	2009	-24603	Alive	TUMOR F	1008						R0	0	1008	0	1008	0	1008	0	1008
TCGA-DD	TCGA-DD	LIHC	25	MALE	WHITE	Stage I	Fibrolame	G2	2011	-9426	Alive	TUMOR F	989		Extrahepa	Other, sp	retroperit	912	R0	0	989	0	989	1	912	1	912
TCGA-DD	TCGA-DD	LIHC	56	FEMALE	WHITE	Stage I	Hepatoce	G3	2007	-20782	Alive	TUMOR F	2746		New Prim	Other, sp	breast car	15	R0	0	2746	0	2746	0	2746	1	15
TCGA-DD	TCGA-DD	LIHC	75	FEMALE	WHITE	Stage IIIA	Hepatoce	G3	2011	-27549	Dead	WITH TUMOR		660	Locoregic	Liver		190	R0	1	660	1	660	1	190	1	190
TCGA-DD	TCGA-DD	LIHC	72	MALE	WHITE	Stage I	Hepatoce	G2	2011	-26330	Alive	WITH TUN	942		Extrahepa	Bone		656	R0	0	942	0	942	1	656	1	656
TCGA-DD	TCGA-DD	LIHC	77	MALE	WHITE	Stage IIIA	Hepatoce	G2	2011	-28234	Dead	WITH TUMOR		802	Locoregic	Liver		430	R0	1	802	1	802	1	430	1	430
TCGA-DD	TCGA-DD	LIHC	65	FEMALE	WHITE	Stage IIIB	Hepatoce	G3	2011	-23865	Alive	WITH TUN	917		Extrahepa	Lung		161	R0	0	917	0	917	1	161	1	161
TCGA-DD	TCGA-DD	LIHC	67	MALE	WHITE	Stage II	Hepatoce	G2	2011	-24516	Alive	TUMOR F	816						R0	0	816	0	816	0	816	0	816
TCGA-DD	TCGA-DD	LIHC	54	FEMALE	WHITE	Stage II	Hepatoce	G2	2011	-19964	Alive	WITH TUN	928		Intrahepa	Liver		549	R0	0	928	0	928	1	549	1	549
TCGA-DD	TCGA-DD	LIHC	80	FEMALE	WHITE	Stage IIIA	Hepatoce	G2	2004	-29244	Dead	WITH TUMOR		1210	Intrahepa	Liver		89	R0	1	1210	1	1210	1	89	1	89
TCGA-DD	TCGA-DD	LIHC	46	MALE	WHITE	Stage I	Hepatoce	G1	2007	-16874	Alive	TUMOR F	1711						R0	0	1711	0	1711	0	1711	0	1711
TCGA-DD	TCGA-DD	LIHC	56	FEMALE	WHITE	Stage I	Hepatoce	G3	2004	-20548	Dead	WITH TUMOR		899	Extrahepa	Lung		166	R0	1	899	1	899	1	166	1	166
TCGA-DD	TCGA-DD	LIHC	65	MALE	WHITE	Stage I	Hepatoce	G1	2007	-24090	Alive	WITH TUN	2245		Locoregic	Liver		990	R0	0	2245	0	2245	1	990	1	990
TCGA-DD	TCGA-DD	LIHC	32	MALE	WHITE	Stage I	Hepatoce	G3	2005	-11838	Alive	TUMOR F	3308		Intrahepa	Liver		1286	R0	0	3308	0	3308	1	1286	1	1286
TCGA-DD	TCGA-DD	LIHC	60	MALE	WHITE	Stage II	Hepatoce	G3	2007	-22111	Dead	WITH TUMOR		373	Intrahepa	Liver		126	R0	1	373	1	373	1	126	1	126
TCGA-DD	TCGA-DD	LIHC	85	FEMALE	WHITE	Stage I	Hepatoce	G3	2005	-31386	Dead	TUMOR FREE		9					R0	1	9	0	9	0	9	0	9
TCGA-DD	TCGA-DD	LIHC	61	FEMALE	WHITE	Stage I	Hepatoce	G2	2005	-22638	Dead	WITH TUMOR		2456	Extrahepa	Bone		893	R0	1	2456	1	2456	1	893	1	893
TCGA-DD	TCGA-DD	LIHC	61	MALE	WHITE	Stage IIIA	Hepatoce	G1	2007	-22438	Alive	TUMOR F	2398						R0	0	2398	0	2398	0	2398	0	2398
TCGA-DD	TCGA-DD	LIHC	71	MALE	WHITE	Stage I	Hepatoce	G2	2012	-26136	Alive	TUMOR F	728						R0	0	728	0	728	0	728	0	728
TCGA-DD	TCGA-DD	LIHC	72	FEMALE	WHITE	Stage I	Hepatoce	G2	2012	-26390	Dead	WITH TUMOR		283	Locoregic	Liver		240	R0	1	283	1	283	1	240	1	240
TCGA-DD	TCGA-DD	LIHC	65	FEMALE	WHITE	Stage IIIA	Hepatoce	G1	2012	-23782	Alive	WITH TUN	701		Locoregic	Liver		566	R0	0	701	0	701	1	566	1	566
TCGA-DD	TCGA-DD	LIHC	68	FEMALE	WHITE	Stage II	Hepatoce	G1	2012	-24941	Alive	WITH TUN	693		Intrahepa	Liver		592	R0	0	693	0	693	1	592	1	592
TCGA-DD	TCGA-DD	LIHC	66	MALE	WHITE	Stage I	Hepatoce	G1	2012	-24377	Alive	TUMOR F	44						R0	0	44	0	44	0	44	0	44
TCGA-DD	TCGA-DD	LIHC	77	FEMALE	WHITE	Stage I	Hepatoce	G1	2010	-28234	Alive	TUMOR F	1085						R0	0	1085	0	1085	0	1085	0	1085
TCGA-DD	TCGA-DD	LIHC	73	FEMALE	WHITE	Stage I	Hepatoce	G3	2005	-26949	Alive	TUMOR F	3478						R0	0	3478	0	3478	0	3478	0	3478
TCGA-DD	TCGA-DD	LIHC	81	FEMALE	WHITE	Stage I	Hepatoch	G4	2009	-29712	Dead	TUMOR FREE		410					R0	1	410	0	410	0	410	0	410
TCGA-DD	TCGA-DD	LIHC	72	MALE	ASIAN	Stage I	Hepatoce	G3	2008	-26322	Dead	TUMOR FREE		16					R0	1	16	0	16	0	16	0	16
TCGA-DD	TCGA-DD	LIHC	51	MALE	ASIAN	Stage I	Hepatoce	G2	2008	-18911	Alive	TUMOR F	347						R0	0	347	0	347	0	347	0	347
TCGA-DD	TCGA-DD	LIHC	65	MALE	ASIAN	Stage I	Hepatoce	G3	2008	-23973	Alive	WITH TUN	2301		Locoregic	Liver		776	R0	0	2301	0	2301	1	776	1	776
TCGA-DD	TCGA-DD	LIHC	65	MALE	ASIAN	Stage I	Hepatoce	G3	2008	-23973	Alive	WITH TUN	2301		Locoregic	Liver		776	R0	0	2301	0	2301	1	776	1	776
TCGA-DD	TCGA-DD	LIHC	74	FEMALE	ASIAN	Stage I	Hepatoce	G3	2008	-27103	Alive	TUMOR F	2324						R0	0	2324	0	2324	0	2324	0	2324
TCGA-DD	TCGA-DD	LIHC	61	MALE	ASIAN	Stage I	Hepatoce	G2	2008	-22499	Dead	WITH TUMOR		1685	Locoregic	Liver		537	R0	1	1685	1	1685	1	537	1	537
TCGA-DD	TCGA-DD	LIHC	48	MALE	ASIAN	Stage I	Hepatoce	G4	2008	-17828	Dead	WITH TUMOR		381	Locoregic	Lung		151	R0	1	381	1	381	1	151	1	151
TCGA-DD	TCGA-DD	LIHC	62	MALE	ASIAN	Stage I	Hepatoce	G3	2008	-22727	Alive	TUMOR F	2184		Intrahepa	Liver		1279	R0	0	2184	0	2184	1	1279	1	1279
TCGA-DD	TCGA-DD	LIHC	68	MALE	ASIAN	Stage I	Hepatoce	G3	2008	-25172	Dead	WITH TUMOR		365	Locoregic	Liver		119	R0	1	365	1	365	1	119	1	119
TCGA-DD	TCGA-DD	LIHC	52	MALE	ASIAN	Stage II	Hepatoce	G4	2008	-19174	Dead	WITH TUMOR		469	Intrahepa	Liver		125	R0	1	469	1	469	1	125	1	125
TCGA-DD	TCGA-DD	LIHC	69	MALE	ASIAN	Stage II	Hepatoce	G3	2008	-25544	Dead	TUMOR FREE		195	Intrahepa	Liver		83	R0	1	195	0	195	1	83	1	83
TCGA-DD	TCGA-DD	LIHC	69	MALE	ASIAN	Stage II	Hepatoce	G3	2008	-25236	Alive	WITH TUN	1618		Intrahepa	Liver		1432	R0	0	1618	0	1618	1	1432	1	1432
TCGA-DD	TCGA-DD	LIHC	75	MALE	ASIAN	Stage II	Hepatoce	G2	2008	-27528	Alive	WITH TUN	2102		Locoregic	Liver		100	R0	0	2102	0	2102	1	100	1	100
TCGA-DD	TCGA-DD	LIHC	70	MALE	ASIAN	Stage I	Hepatoce	G2	2008	-25796	Alive	TUMOR F	9						R0	0	9	0	9	0	9	0	9
TCGA-DD	TCGA-DD	LIHC	66	FEMALE	ASIAN	Stage I	Hepatoce	G3	2008	-24355	Dead			107					R0	1	107		107	0	107	0	107
TCGA-DD	TCGA-DD	LIHC	48	MALE	ASIAN	Stage II	Hepatoce	G3	2009	-17590	Alive	WITH TUN	1769		Extrahepa	Lung		1097	R0	0	1769	0	1769	1	1097	1	1097
TCGA-DD	TCGA-DD	LIHC	32	MALE	ASIAN	Stage I	Hepatoce	G3	2009	-12007	Alive	WITH TUN	1302		Intrahepa	Liver		357	R0	0	1302	0	1302	1	357	1	357
TCGA-DD	TCGA-DD	LIHC	40	MALE	ASIAN	Stage I	Hepatoce	G3	2009	-14796	Alive	WITH TUN	1876		Intrahepa	Liver		479	R0	0	1876	0	1876	1	479	1	479
TCGA-DD	TCGA-DD	LIHC	64	MALE	ASIAN	Stage I	Hepatoce	G3	2009	-23728	Alive		415						R0	0	415	0	415	0	415	0	415
TCGA-DD	TCGA-DD	LIHC	50	MALE	ASIAN	Stage II	Hepatoce	G3	2009	-18429	Dead	WITH TUMOR		432	Locoregic	Liver		105	R0	1	432	1	432	1	105	1	105
TCGA-DD	TCGA-DD	LIHC	39	MALE	ASIAN	Stage I	Hepatoce	G3	2009	-14473	Alive	TUMOR F	1804						R0	0	1804	0	1804	0	1804	0	1804
TCGA-DD	TCGA-DD	LIHC	69	FEMALE	ASIAN	Stage I	Hepatoce	G2	2010	-25292	Alive	TUMOR F	1562						R0	0	1562	0	1562	0	1562	0	1562
TCGA-DD	TCGA-DD	LIHC	59	MALE	ASIAN	Stage I	Hepatoce	G3	2010	-21606	Alive	TUMOR F	1567						R0	0	1567	0	1567	0	1567	0	1567
TCGA-DD	TCGA-DD	LIHC	53	MALE	ASIAN	Stage I	Hepatoce	G3	2010	-19660	Alive	WITH TUN	1531		Intrahepa	Liver		344	R0	0	1531	0	1531	1	344	1	344
TCGA-DD	TCGA-DD	LIHC	43	MALE	ASIAN	Stage I	Hepatoce	G3	2010	-15872	Alive	TUMOR F	1424						R0	0	1424	0	1424	0	1424	0	1424
TCGA-DD	TCGA-DD	LIHC	66	MALE	ASIAN	Stage II	Hepatoce	G3	2010	-24329	Alive	WITH TUN	170		Extrahepa	Other, sp	lymph no	42	R0	0	170	0	170	1	42	1	42
TCGA-DD	TCGA-DD	LIHC	61	MALE	ASIAN	Stage I	Hepatoce	G3	2010	-22332	Alive	WITH TUN	1450		Intrahepa	Liver		776	R0	0	1450	0	1450	1	776	1	776
TCGA-DD	TCGA-DD	LIHC	63	FEMALE	ASIAN	Stage I	Hepatoce	G4	2010	-23230	Dead	TUMOR FREE		171	Locoregic	Liver		88	R0	1	171	0	171	1	88	1	88
TCGA-DD	TCGA-DD	LIHC	73	FEMALE	ASIAN	Stage I	Hepatoce	G2	2010	-26781	Alive	WITH TUN	137		Intrahepa	Liver		47	R0	0	137	0	137	1	47	1	47
TCGA-DD	TCGA-DD	LIHC	51	FEMALE	ASIAN	Stage I	Hepatoce	G4	2010	-18794	Alive	WITH TUN	564		Intrahepa	Liver		127	R1	0	564	0	564			1	127
TCGA-DD	TCGA-DD	LIHC	66	MALE	ASIAN	Stage I	Hepatoce	G2	2010	-24221	Alive	TUMOR F	658						R0	0	658	0	658	0	658	0	658
TCGA-DD	TCGA-DD	LIHC	43	MALE	ASIAN	Stage I	Hepatoce	G2	2011	-15706	Alive	TUMOR F	1295						R0	0	1295	0	1295	0	1295	0	1295
TCGA-DD	TCGA-DD	LIHC	54	MALE	ASIAN	Stage I	Hepatoce	G3	2011	-19748	Alive	WITH TUN	1345		Intrahepa	Liver		130	R0	0	1345	0	1345	1	130	1	130
TCGA-DD	TCGA-DD	LIHC	66	MALE	ASIAN	Stage IIIA	Hepatoce	G3	2011	-24185	Alive	TUMOR F	672		Locoregic	Liver		50	R0	0	672	0	672	1	50	1	50
TCGA-DD	TCGA-DD	LIHC	73	FEMALE	ASIAN	Stage I	Hepatoce	G2	2011	-26781	Alive	TUMOR F	1219						R0	0	1219	0	1219	0	1219	0	1219
TCGA-DD	TCGA-DD	LIHC	66	FEMALE	ASIAN	Stage I	Hepatoce	G3	2011	-24288	Alive	TUMOR F	1233						R0	0	1233	0	1233	0	1233	0	1233
TCGA-DD	TCGA-DD	LIHC	51	MALE	ASIAN	Stage I	Hepatoce	G4	2011	-18728	Alive	TUMOR F	1242						R0	0	1242	0	1242	0	1242	0	1242
TCGA-DD	TCGA-DD	LIHC	53	MALE	ASIAN	Stage I	Hepatoce	G3	2011	-19527	Dead	WITH TUMOR		425	Intrahepa	Liver		101	R0	1	425	1	425	1	101	1	101
TCGA-DD	TCGA-DD	LIHC	51	MALE	ASIAN	Stage I	Hepatoce	G4	2011	-18752	Alive	TUMOR F	1231						R0	0	1231	0	1231	0	1231	0	1231
TCGA-DD	TCGA-DD	LIHC	50	MALE	ASIAN	Stage I	Hepatoce	G4	2011	-18482	Alive	WITH TUN	1202		Intrahepa	Liver		168	R0	0	1202	0	1202	1	168	1	168
TCGA-DD	TCGA-DD	LIHC	64	FEMALE	ASIAN	Stage I	Hepatoce	G4	2011	-23614	Dead	TUMOR FREE		115					R0	1	115	0	115	0	115	0	115
TCGA-DD	TCGA-DD	LIHC	70	MALE	ASIAN	Stage IIIA	Hepatoce	G3	2011	-25887	Alive	TUMOR F	1145		Intrahepa	Liver		270	R0	0	1145	0	1145	1	270	1	270
TCGA-DD	TCGA-DD	LIHC	43	FEMALE	ASIAN	Stage I	Hepatoce	G3	2011	-15928	Alive	TUMOR F	1085						R0	0	1085	0	1085	0	1085	0	1085
TCGA-DD	TCGA-DD	LIHC	70	FEMALE	ASIAN	Stage I	Hepatoce	G3	2011	-25863	Alive	TUMOR F	1066						R0	0	1066	0	1066	0	1066	0	1066
TCGA-DD	TCGA-DD	LIHC	68	FEMALE	ASIAN	Stage II	Hepatoce	G3	2011	-25046	Alive	TUMOR F	1049						R0	0	1049	0	1049	0	1049	0	1049
TCGA-DD	TCGA-DD	LIHC	58	MALE	ASIAN	Stage I	Hepatoce	G4	2012	-21512	Alive	TUMOR F	636						R0	0	636	0	636	0	636	0	636
TCGA-DD	TCGA-DD	LIHC	58	MALE	ASIAN	Stage II	Hepatoce	G3	2012	-21349	Dead	TUMOR FREE		12					R0	1	12	0	12	0	12	0	12
TCGA-DD	TCGA-DD	LIHC	59	MALE	ASIAN	Stage I	Hepatoce	G4	2012	-21632	Alive	TUMOR F	898						R0	0	898	0	898	0	898	0	898
TCGA-DD	TCGA-DD	LIHC	55	MALE	ASIAN	Stage I	Hepatoce	G3	2013	-20139	Alive	TUMOR F	453						R0	0	453	0	453	0	453	0	453
TCGA-DD	TCGA-DD	LIHC	45	MALE	ASIAN	Stage I	Hepatoce	G3	2013	-16760	Alive	TUMOR F	458						R0	0	458	0	458	0	458	0	458
TCGA-DD	TCGA-DD	LIHC	59	MALE	ASIAN	Stage II	Hepatoce	G3	2013	-21756	Alive	TUMOR F	436						R0	0	436	0	436	0	436	0	436
TCGA-DD	TCGA-DD	LIHC	58	MALE	ASIAN	Stage I	Hepatoce	G3	2009	-21217	Alive	TUMOR F	2028		Intrahepa	Liver		1700	R0	0	2028	0	2028	1	1700	1	1700
TCGA-DD	TCGA-DD	LIHC	63	MALE	ASIAN	Stage I	Hepatoce	G2	2013	-23227	Alive	TUMOR F	474						R0	0	474	0	474	0	474	0	474
TCGA-DD	TCGA-DD	LIHC	60	MALE	ASIAN	Stage II	Hepatoce	G3	2013	-21940	Alive	TUMOR F	554						R0	0	554	0	554	0	554	0	554
TCGA-DD	TCGA-DD	LIHC	50	MALE	ASIAN	Stage I	Hepatoce	G3	2013	-18300	Alive	TUMOR F	574						R0	0	574	0	574	0	574	0	574
TCGA-DD	TCGA-DD	LIHC	48	MALE	ASIAN	Stage I	Hepatoce	G3	2013	-17781	Alive	TUMOR F	587		Locoregic	Liver		386	R0	0	587	0	587	1	386	1	386
TCGA-DD	TCGA-DD	LIHC	55	FEMALE	ASIAN	Stage I	Hepatoce	G2	2013	-20233	Alive	TUMOR F	555						R0	0	555	0	555	0	555	0	555
TCGA-DD	TCGA-DD	LIHC	45	FEMALE	ASIAN	Stage IIIA	Hepatoce	G4	2013	-16732	Alive	TUMOR F	555						R0	0	555	0	555	0	555	0	555
TCGA-DD	TCGA-DD	LIHC	52	MALE	ASIAN	Stage I	Hepatoce	G3	2013	-19073	Alive	TUMOR F	552						R0	0	552	0	552	0	552	0	552
TCGA-DD	TCGA-DD	LIHC	51	MALE	ASIAN	Stage I	Hepatoce	G3	2013	-18695	Alive	TUMOR F	638						R0	0	638	0	638	0	638	0	638
TCGA-DD	TCGA-DD	LIHC	50	MALE	ASIAN	Stage I	Hepatoce	G2	2013	-18271	Alive	TUMOR F	566						R0	0	566	0	566	0	566	0	566
TCGA-DD	TCGA-DD	LIHC	49	FEMALE	ASIAN	Stage I	Hepatoce	G1	2013	-18009	Alive	WITH TUN	608		Locoregic	Liver		111	R0	0	608	0	608	1	111	1	111
TCGA-DD	TCGA-DD	LIHC	59	FEMALE	ASIAN	Stage I	Hepatoce	G2	2012	-21764	Alive	WITH TUN	141		Intrahepa	Liver		105	R0	0	141	0	141	1	105	1	105
TCGA-DD	TCGA-DD	LIHC	72	MALE	ASIAN	Stage I	Hepatoce	G2	2012	-26568	Alive	TUMOR F	644						R0	0	644	0	644	0	644	0	644
TCGA-DD	TCGA-DD	LIHC	45	MALE	ASIAN	Stage I	Hepatoce	G3	2012	-16549	Alive	TUMOR F	664		Locoregic	Liver		28	R0	0	664	0	664	1	28	1	28
TCGA-DD	TCGA-DD	LIHC	69	MALE	ASIAN	Stage I	Hepatoce	G3	2012	-25451	Alive	TUMOR F	722						R0	0	722	0	722	0	722	0	722
TCGA-DD	TCGA-DD	LIHC	65	MALE	ASIAN	Stage I	Hepatoce	G3	2012	-24027	Alive	TUMOR F	575						R0	0	575	0	575	0	575	0	575
TCGA-DD	TCGA-DD	LIHC	60	MALE	ASIAN	Stage I	Hepatoce	G2	2012	-22121	Alive	TUMOR F	478						R0	0	478	0	478	0	478	0	478
TCGA-DD	TCGA-DD	LIHC	51	MALE	ASIAN	Stage I	Hepatoce	G3	2012	-18722	Alive	WITH TUN	763		Intrahepa	Liver		198	R0	0	763	0	763	1	198	1	198
TCGA-DD	TCGA-DD	LIHC	55	MALE	ASIAN	Stage I	Hepatoce	G4	2012	-20396	Alive	WITH TUN	810		Intrahepa	Liver		224	R0	0	810	0	810	1	224	1	224
TCGA-DD	TCGA-DD	LIHC	59	FEMALE	ASIAN	Stage I	Hepatoce	G3	2012	-21853	Alive	TUMOR F	719						R0	0	719	0	719	0	719	0	719
TCGA-DD	TCGA-DD	LIHC	73	MALE	ASIAN	Stage I	Hepatoce	G2	2012	-26894	Alive	TUMOR F	784		Extrahepa	Bone		644	R0	0	784	0	784	1	644	1	644
TCGA-DD	TCGA-DD	LIHC	72	MALE	ASIAN	Stage I	Hepatoce	G2	2010	-26465	Alive	WITH TUN	1531		Extrahepa	Bone		491	R0	0	1531	0	1531	1	491	1	491
TCGA-DD	TCGA-DD	LIHC	51	MALE	ASIAN	Stage II	Hepatoce	G3	2010	-18757	Alive	TUMOR F	1067						R0	0	1067	0	1067	0	1067	0	1067
TCGA-DD	TCGA-DD	LIHC	48	MALE	ASIAN	Stage I	Hepatoce	G1	2007	-17833	Alive	TUMOR F	2752		Locoregic	Liver		903	R0	0	2752	0	2752	1	903	1	903
TCGA-DD	TCGA-DD	LIHC	38	MALE	ASIAN	Stage I	Hepatoce	G2	2007	-13911	Alive	TUMOR F	2728		Locoregic	Liver		1509	R0	0	2728	0	2728	1	1509	1	1509
TCGA-DD	TCGA-DD	LIHC	44	MALE	ASIAN	Stage I	Hepatoce	G2	2007	-16157	Alive	TUMOR F	2513						R0	0	2513	0	2513	0	2513	0	2513
TCGA-DD	TCGA-DD	LIHC	56	MALE	ASIAN	Stage I	Hepatoce	G2	2007	-20586	Alive	TUMOR F	1823						R0	0	1823	0	1823	0	1823	0	1823
TCGA-DD	TCGA-DD	LIHC	46	MALE	ASIAN	Stage II	Hepatoce	G2	2008	-16889	Alive	TUMOR F	2202		Intrahepa	Liver		1630	R0	0	2202	0	2202	1	1630	1	1630
TCGA-DD	TCGA-DD	LIHC	56	MALE	ASIAN	Stage II	Hepatoce	G3	2007	-20751	Alive	TUMOR F	2455						R0	0	2455	0	2455	0	2455	0	2455
TCGA-DD	TCGA-DD	LIHC	35	MALE	ASIAN	Stage I	Hepatoce	G2	2008	-12986	Alive	TUMOR F	2317						R0	0	2317	0	2317	0	2317	0	2317
TCGA-DD	TCGA-DD	LIHC	38	MALE	ASIAN	Stage II	Hepatoce	G2	2008	-14016	Alive	WITH TUN	1718		Intrahepa	Liver		393	R0	0	1718	0	1718	1	393	1	393
TCGA-DD	TCGA-DD	LIHC	56	MALE	ASIAN	Stage IIIA	Hepatoce	G2	2009	-20709	Alive	TUMOR F	1970						R0	0	1970	0	1970	0	1970	0	1970
TCGA-DD	TCGA-DD	LIHC	38	MALE	ASIAN	Stage I	Hepatoce	G2	2009	-14005	Alive	TUMOR F	1900						R0	0	1900	0	1900	0	1900	0	1900
TCGA-DD	TCGA-DD	LIHC	54	MALE	ASIAN	Stage I	Hepatoce	G2	2009	-19981	Alive	TUMOR F	2015						R0	0	2015	0	2015	0	2015	0	2015
TCGA-DD	TCGA-DD	LIHC	55	MALE	ASIAN	Stage IIIA	Hepatoce	G2	2009	-20288	Alive	TUMOR F	1989						R0	0	1989	0	1989	0	1989	0	1989
TCGA-DD	TCGA-DD	LIHC	69	MALE	ASIAN	Stage I	Hepatoce	G2	2009	-25420	Alive	WITH TUN	1855		Intrahepa	Liver		391	R0	0	1855	0	1855	1	391	1	391
TCGA-DD	TCGA-DD	LIHC	69	MALE	ASIAN	Stage I	Hepatoce	G2	2009	-25254	Alive	TUMOR F	1633						R0	0	1633	0	1633	0	1633	0	1633
TCGA-ED	TCGA-ED	LIHC	47	MALE	ASIAN	Stage II	Hepatoce	G2	2012	-17402	Alive	TUMOR F	910						R0	0	910	0	910	0	910	0	910
TCGA-ED	TCGA-ED	LIHC	58	MALE	ASIAN	Stage II	Hepatoce	G3	2012	-21497	Alive	TUMOR F	819						R0	0	819	0	819	0	819	0	819
TCGA-ED	TCGA-ED	LIHC	60	FEMALE	ASIAN	Stage II	Hepatoce	G2	2012	-22258	Alive	WITH TUN	854		New Prim	Other, sp	Kidney	110	R0	0	854	0	854	0	854	1	110
TCGA-ED	TCGA-ED	LIHC	74	MALE	WHITE	Stage I	Hepatoce	G2	2012	-27303	Alive	TUMOR F	423						R0	0	423	0	423	0	423	0	423
TCGA-ED	TCGA-ED	LIHC	35	MALE	ASIAN	Stage IIIA	Hepatoce	G3	2013	-12947	Alive	TUMOR F	406		Intrahepa	Liver		124	R0	0	406	0	406	1	124	1	124
TCGA-ED	TCGA-ED	LIHC	51	FEMALE	ASIAN	Stage IIIA	Hepatoce	G3	2013	-18797	Dead	TUMOR FREE		296					R0	1	296	0	296	0	296	0	296
TCGA-ED	TCGA-ED	LIHC	48	FEMALE	ASIAN	Stage II	Hepatoce	G3	2013	-17638	Alive	TUMOR F	6						R0	0	6	0	6	0	6	0	6
TCGA-ED	TCGA-ED	LIHC	20	FEMALE	ASIAN	Stage II	Hepatoce	G3	2013	-7556	Alive	TUMOR F	390						R0	0	390	0	390	0	390	0	390
TCGA-ED	TCGA-ED	LIHC	61	MALE	ASIAN	Stage II	Hepatoce	G2	2013	-22601	Alive	TUMOR F	6						R0	0	6	0	6	0	6	0	6
TCGA-ED	TCGA-ED	LIHC	29	MALE	ASIAN	Stage IIIA	Hepatoce	G2	2013	-10828	Alive	WITH TUN	427		Locoregic	Liver		262	R0	0	427	0	427	1	262	1	262
TCGA-ED	TCGA-ED	LIHC	53	FEMALE	ASIAN	Stage II	Hepatoce	G3	2013	-19504	Alive	WITH TUN	400		Locoregic	Liver		187	R0	0	400	0	400	1	187	1	187
TCGA-ED	TCGA-ED	LIHC	60	FEMALE	ASIAN	Stage IIIA	Hepatoce	G2	2013	-22234	Alive	TUMOR F	408						R0	0	408	0	408	0	408	0	408
TCGA-ED	TCGA-ED	LIHC	59	FEMALE	ASIAN	Stage IIIA	Hepatoce	G3	2013	-21648	Alive	TUMOR F	406						R0	0	406	0	406	0	406	0	406
TCGA-ED	TCGA-ED	LIHC	50	FEMALE	ASIAN	Stage IIIA	Hepatoce	G3	2013	-18572	Dead	TUMOR FREE		56					R0	1	56	0	56	0	56	0	56
TCGA-ED	TCGA-ED	LIHC	54	MALE	ASIAN	Stage IIIA	Hepatoch	G2	2013	-20073	Alive	TUMOR F	6						R0	0	6	0	6	0	6	0	6
TCGA-EP	TCGA-EP	LIHC	62	MALE	BLACK OI	Stage I	Hepatoce	G1	2010	-22997	Alive	TUMOR F	570						R0	0	570	0	570	0	570	0	570
TCGA-EP	TCGA-EP	LIHC	62	MALE	BLACK OI	Stage I	Hepatoce	G1	2010	-22997	Alive	TUMOR F	570						R0	0	570	0	570	0	570	0	570
TCGA-EP	TCGA-EP	LIHC	70	MALE	WHITE	Stage I	Hepatoce	G2	2011	-25677	Alive	TUMOR F	608							0	608	0	608			0	608
TCGA-EP	TCGA-EP	LIHC	70	MALE	WHITE	Stage I	Hepatoce	G2	2011	-25677	Alive	TUMOR F	608							0	608	0	608			0	608
TCGA-EP	TCGA-EP	LIHC	52	FEMALE	WHITE	Stage IIIA	Hepatoce	G3	2011	-19214	Dead	WITH TUMOR		627	Extrahepa	Lung		293	R0	1	627	1	627	1	293	1	293
TCGA-EP	TCGA-EP	LIHC	46	FEMALE	WHITE	Stage I	Hepatoce	G2	2011	-16835	Dead	WITH TUMOR		596	Intrahepa	Liver		161	R0	1	596	1	596	1	161	1	161
TCGA-EP	TCGA-EP	LIHC	62	MALE	BLACK OI	Stage I	Hepatoce	G3	2011	-22677	Dead	TUMOR FREE		19					R0	1	19	0	19	0	19	0	19
TCGA-EP	TCGA-EP	LIHC	76	MALE	WHITE	Stage I	Hepatoce	G2	2011	-27886	Alive	TUMOR F	303							0	303	0	303			0	303
TCGA-EP	TCGA-EP	LIHC	73	MALE	WHITE	Stage IIIA	Hepatoce	G2	2012	-26985	Alive	WITH TUN	363		Locoregic	Liver		320		0	363	0	363			1	320
TCGA-EP	TCGA-EP	LIHC	73	MALE	WHITE	Stage IIIA	Hepatoce	G2	2012	-26985	Alive	WITH TUN	363		Locoregic	Liver		320		0	363	0	363			1	320
TCGA-ES	TCGA-ES	LIHC	80	MALE	WHITE	Stage I	Hepatoce	G2	2008	-29268	Dead	TUMOR FREE		688					R0	1	688	0	688	0	688	0	688
TCGA-ES	TCGA-ES	LIHC	80	MALE	WHITE	Stage I	Hepatoce	G2	2008	-29268	Dead	TUMOR FREE		688					R0	1	688	0	688	0	688	0	688
TCGA-ES	TCGA-ES	LIHC	54	MALE	BLACK OI	Stage I	Hepatoce	G2	2007	-20023	Dead	TUMOR FREE		438					R0	1	438	0	438	0	438	0	438
TCGA-ES	TCGA-ES	LIHC	54	MALE	BLACK OI	Stage I	Hepatoce	G2	2007	-20023	Dead	TUMOR FREE		438					R0	1	438	0	438	0	438	0	438
TCGA-FV	TCGA-FV	LIHC	70	FEMALE	WHITE	Stage II	Hepatocellular Carci		2005	-25788	Dead	WITH TUMOR		1852	Locoregic	Liver		1453	R0	1	1852	1	1852	1	1453	1	1453
TCGA-FV	TCGA-FV	LIHC	70	FEMALE	WHITE	Stage II	Hepatocellular Carci		2005	-25788	Dead	WITH TUMOR		1852	Locoregic	Liver		1453	R0	1	1852	1	1852	1	1453	1	1453
TCGA-FV	TCGA-FV	LIHC	80	MALE	WHITE	Stage I	Hepatoce	G2	2011	-29354	Alive	TUMOR F	729						R0	0	729	0	729	0	729	0	729
TCGA-FV	TCGA-FV	LIHC	75	MALE	WHITE	Stage I	Hepatoce	G1	2006		Dead	WITH TUMOR		581	Extrahepatic Recurrence			561	RX	1	581	1	581			1	561
TCGA-FV	TCGA-FV	LIHC	75	MALE	WHITE	Stage I	Hepatoce	G1	2006		Dead	WITH TUMOR		581	Extrahepatic Recurrence			561	RX	1	581	1	581			1	561
TCGA-FV	TCGA-FV	LIHC	76	FEMALE	WHITE	Stage II	Hepatoch	G2	2011	-28101	Alive	WITH TUN	848		Intrahepa	Liver		159	R0	0	848	0	848	1	159	1	159
TCGA-FV	TCGA-FV	LIHC	76	FEMALE	WHITE	Stage II	Hepatoch	G2	2011	-28101	Alive	WITH TUN	848		Intrahepa	Liver		159	R0	0	848	0	848	1	159	1	159
TCGA-FV	TCGA-FV	LIHC	81	FEMALE	WHITE	Stage II	Hepatoce	G2	2011		Dead	TUMOR FREE		247					R0	1	247	0	247	0	247	0	247
TCGA-FV	TCGA-FV	LIHC	81	FEMALE	WHITE	Stage II	Hepatoce	G2	2011		Dead	TUMOR FREE		247					R0	1	247	0	247	0	247	0	247
TCGA-FV	TCGA-FV	LIHC	75	MALE	WHITE	Stage I	Hepatocellular Carci		2005	-27530	Dead	TUMOR FREE		194					R0	1	194	0	194	0	194	0	194
TCGA-FV	TCGA-FV	LIHC	75	MALE	WHITE	Stage I	Hepatocellular Carci		2005	-27530	Dead	TUMOR FREE		194					R0	1	194	0	194	0	194	0	194
TCGA-FV	TCGA-FV	LIHC	38	FEMALE	WHITE	Stage I	Hepatoce	G2	2012	-14231	Dead	TUMOR FREE		366					R0	1	366	0	366	0	366	0	366
TCGA-FV	TCGA-FV	LIHC	51	FEMALE	WHITE	Stage II	Hepatoce	G2	2012	-18740	Alive	TUMOR F	1						R0	0	1	0	1	0	1	0	1
TCGA-FV	TCGA-FV	LIHC	84	FEMALE	WHITE	Stage I	Hepatoce	G2	2012	-30950	Alive	TUMOR F	10						R0	0	10	0	10	0	10	0	10
TCGA-FV	TCGA-FV	LIHC	78	MALE	WHITE	Stage IIIA	Hepatoce	G2	2005	-28771	Dead	TUMOR FREE		2486					R1	1	2486	0	2486			0	2486
TCGA-FV	TCGA-FV	LIHC	52	MALE	WHITE	Stage I	Hepatoce	G2	2012	-19090	Alive	TUMOR F	12						R0	0	12	0	12	0	12	0	12
TCGA-G3	TCGA-G3	LIHC	64	MALE	WHITE	Stage I	Hepatoce	G2	2009	-23571	Dead	WITH TUMOR		416	Extrahepa	Other, sp	Bone and	37	R1	1	416	1	416			1	37
TCGA-G3	TCGA-G3	LIHC	45	FEMALE	WHITE	Stage IIIA	Hepatoce	G2	2007	-16461	Alive	WITH TUN	1553		Intrahepa	Liver		452	R0	0	1553	0	1553	1	452	1	452
TCGA-G3	TCGA-G3	LIHC	63	FEMALE	ASIAN	Stage I	Hepatoce	G3	2007	-23207	Alive	TUMOR F	1636						R0	0	1636	0	1636	0	1636	0	1636
TCGA-G3	TCGA-G3	LIHC	68	MALE	WHITE	Stage I	Hepatoce	G2	2010	-25144	Alive	WITH TUN	860		Intrahepa	Liver		489	R0	0	860	0	860	1	489	1	489
TCGA-G3	TCGA-G3	LIHC	79	FEMALE	WHITE	Stage IIIB	Hepatoce	G2	2010	-29018	Alive	TUMOR F	935						R0	0	935	0	935	0	935	0	935
TCGA-G3	TCGA-G3	LIHC	79	FEMALE	WHITE	Stage IIIB	Hepatoce	G2	2010	-29018	Alive	TUMOR F	935						R0	0	935	0	935	0	935	0	935
TCGA-G3	TCGA-G3	LIHC	73	MALE	ASIAN	Stage II	Hepatoce	G3	2007	-26988	Alive	TUMOR F	1779						R0	0	1779	0	1779	0	1779	0	1779
TCGA-G3	TCGA-G3	LIHC	73	MALE	ASIAN	Stage II	Hepatoce	G3	2007	-26988	Alive	TUMOR F	1779						R0	0	1779	0	1779	0	1779	0	1779
TCGA-G3	TCGA-G3	LIHC	52	FEMALE	ASIAN	Stage I	Hepatoce	G3	2006	-19055	Dead	WITH TUMOR		452	Locoregic	Liver		355	R0	1	452	1	452	1	355	1	355
TCGA-G3	TCGA-G3	LIHC	58	MALE	ASIAN	Stage I	Hepatoce	G2	2010	-21509	Alive	TUMOR F	655						R0	0	655	0	655	0	655	0	655
TCGA-G3	TCGA-G3	LIHC	80	MALE	WHITE	Stage I	Hepatoce	G2	2011	-29432	Alive	WITH TUN	673		Intrahepa	Liver		329	R1	0	673	0	673			1	329
TCGA-G3	TCGA-G3	LIHC	53	MALE	ASIAN	Stage IIIA	Hepatoce	G2	2010	-19473	Alive	WITH TUN	780		Intrahepa	Liver		116	R0	0	780	0	780	1	116	1	116
TCGA-G3	TCGA-G3	LIHC	53	MALE	ASIAN	Stage IIIA	Hepatoce	G2	2010	-19473	Alive	WITH TUN	780		Intrahepa	Liver		116	R0	0	780	0	780	1	116	1	116
TCGA-G3	TCGA-G3	LIHC	71	MALE	WHITE	Stage I	Hepatoce	G2	2011	-26224	Alive	TUMOR F	180						R0	0	180	0	180	0	180	0	180
TCGA-G3	TCGA-G3	LIHC	52	MALE	AMERICA	Stage II	Hepatoce	G2	2011	-19305	Alive	WITH TUN	594		Extrahepa	Lung		296	R0	0	594	0	594	1	296	1	296
TCGA-G3	TCGA-G3	LIHC	61	MALE	ASIAN	Stage I	Hepatoce	G2	2011	-22462	Alive	TUMOR F	585						R0	0	585	0	585	0	585	0	585
TCGA-G3	TCGA-G3	LIHC	44	MALE	ASIAN	Stage II	Hepatoce	G2	2011	-16126	Dead	WITH TUMOR		768	Intrahepa	Liver		348	R0	1	768	1	768	1	348	1	348
TCGA-G3	TCGA-G3	LIHC	59	MALE	WHITE	Stage I	Hepatoce	G2	2012	-21848	Alive	TUMOR F	698		Intrahepa	Liver		308	R0	0	698	0	698	1	308	1	308
TCGA-G3	TCGA-G3	LIHC	58	MALE	WHITE	Stage I	Hepatoce	G1	2012	-21320	Alive	WITH TUN	744		Intrahepa	Liver		483	R0	0	744	0	744	1	483	1	483
TCGA-G3	TCGA-G3	LIHC	70	MALE	WHITE	Stage II	Hepatoce	G2	2012	-25671	Alive	TUMOR F	621						R0	0	621	0	621	0	621	0	621
TCGA-G3	TCGA-G3	LIHC	58	MALE	WHITE	Stage II	Hepatoce	G3	2012	-21236	Alive	TUMOR F	520		Intrahepa	Liver		252	R0	0	520	0	520	1	252	1	252
TCGA-G3	TCGA-G3	LIHC	65	MALE	WHITE	Stage IIIB	Hepatoce	G2	2012	-23793	Alive	WITH TUN	671		Intrahepa	Liver		639	R0	0	671	0	671	1	639	1	639
TCGA-G3	TCGA-G3	LIHC	76	MALE	ASIAN	Stage I	Hepatoce	G2	2013	-27963	Alive	TUMOR F	447						R0	0	447	0	447	0	447	0	447
TCGA-G3	TCGA-G3	LIHC	60	FEMALE	WHITE	Stage I	Hepatoce	G3	2013	-22205	Alive	WITH TUN	632		Locoregic	Liver		293	R0	0	632	0	632	1	293	1	293
TCGA-G3	TCGA-G3	LIHC	65	MALE	WHITE	Stage I	Hepatoce	G1	2013	-24002	Alive	TUMOR F	361						R0	0	361	0	361	0	361	0	361
TCGA-G3	TCGA-G3	LIHC	31	MALE	ASIAN	Stage I	Hepatoce	G1	2013	-11527	Alive	TUMOR F	430						RX	0	430	0	430			0	430
TCGA-G3	TCGA-G3	LIHC	70	MALE	WHITE	Stage IIIB	Hepatoce	G2	2013	-25588	Dead			56					R0	1	56		56	0	56	0	56
TCGA-G3	TCGA-G3	LIHC	48	MALE	[Unknowr	Stage I	Hepatoce	G2	2013	-17760	Alive	TUMOR F	480						R0	0	480	0	480	0	480	0	480
TCGA-G3	TCGA-G3	LIHC	58	MALE	ASIAN	Stage I	Hepatoce	G2	2013	-21249	Alive	TUMOR F	476						R0	0	476	0	476	0	476	0	476
TCGA-G3	TCGA-G3	LIHC	51	MALE	ASIAN	Stage IIIC	Hepatoce	G3	2013	-18973	Dead	WITH TUMOR		359	Locoregic	Liver		49	R0	1	359	1	359	1	49	1	49
TCGA-G3	TCGA-G3	LIHC	50	MALE	WHITE	Stage I	Hepatoce	G1	2013	-18363	Alive	TUMOR F	372						R0	0	372	0	372	0	372	0	372
TCGA-G3	TCGA-G3	LIHC	58	FEMALE	WHITE	Stage II	Hepatoce	G2	2013	-21449	Alive	TUMOR F	412						R0	0	412	0	412	0	412	0	412
TCGA-G3	TCGA-G3	LIHC	83	FEMALE	WHITE	Stage I	Hepatoce	G1	2012	-30466	Dead	TUMOR FREE		27					R0	1	27	0	27	0	27	0	27
TCGA-G3	TCGA-G3	LIHC	67	MALE	WHITE	Stage II	Hepatoce	G2	2013	-24655	Alive	WITH TUN	354		Locoregic	Liver		117	R0	0	354	0	354	1	117	1	117
TCGA-G3	TCGA-G3	LIHC	53	FEMALE	WHITE	Stage IIIA	Hepatoce	G3	2013	-19556	Dead	WITH TUMOR		65	Intrahepa	Liver		44	R0	1	65	1	65	1	44	1	44
TCGA-G3	TCGA-G3	LIHC	38	MALE	ASIAN	Stage II	Hepatoce	G2	2013	-14229	Alive	TUMOR F	361						R0	0	361	0	361	0	361	0	361
TCGA-GJ	TCGA-GJ	LIHC	59	MALE	WHITE	Stage I	Hepatoce	G2	2011	-21671	Alive	[Discrepar	879		Intrahepa	Liver		879	RX	0	879	0	879			1	879
TCGA-GJ	TCGA-GJ	LIHC	75	FEMALE	WHITE	Stage II	Hepatoce	G2	2012	-27626	Dead			31					RX	1	31		31			0	31
TCGA-GJ	TCGA-GJ	LIHC	68	MALE	WHITE	Stage II	Hepatoce	G2	2013	-25020	Dead			67					R0	1	67		67	0	67	0	67
TCGA-HP	TCGA-HP	LIHC	78	MALE	[Not Evalu	Stage I	Hepatoce	G2		-22702	Dead	TUMOR FREE		91					R0	1	91	0	91	0	91	0	91
TCGA-HP	TCGA-HP	LIHC	90	FEMALE	[Not Evaluated]		Hepatocellular Carcinoma			-32120	Dead	WITH TUMOR		1147	Locoregic	Liver		179	R0	1	1147	1	1147	1	179	1	179
TCGA-K7	TCGA-K7	LIHC	64	MALE	WHITE	Stage I	Hepatoce	G1	2012	-23557	Alive	TUMOR F	631		Intrahepa	Liver		205	R0	0	631	0	631	1	205	1	205
TCGA-K7	TCGA-K7	LIHC	66	MALE	BLACK OI	Stage I	Hepatoce	G1	2012	-24218	Alive	TUMOR F	519						R0	0	519	0	519	0	519	0	519
TCGA-K7	TCGA-K7	LIHC	66	MALE	WHITE	Stage I	Hepatoce	G2	2013	-24233	Alive	TUMOR F	512						RX	0	512	0	512			0	512
TCGA-K7	TCGA-K7	LIHC	61	MALE	WHITE	Stage II	Hepatoch	G2	2013	-22476	Alive	WITH TUN	359		Intrahepa	Liver		58	RX	0	359	0	359			1	58
TCGA-KR	TCGA-KR	LIHC	65	MALE	WHITE	Stage I	Hepatoce	G1	2013	-23974	Dead	TUMOR FREE		65					R0	1	65	0	65	0	65	0	65
TCGA-KR	TCGA-KR	LIHC	64	MALE	WHITE	Stage I	Hepatoch	G1	2013	-23699	Alive	TUMOR F	829						R0	0	829	0	829	0	829	0	829
TCGA-KR	TCGA-KR	LIHC	61	FEMALE	WHITE	Stage II	Hepatoce	G1	2012	-22587	Alive	WITH TUN	951		Extrahepa	Other, sp	Adrenal b	239	R0	0	951	0	951	1	239	1	239
TCGA-KR	TCGA-KR	LIHC	57	MALE	[Unknowr	Stage I	Hepatoce	G1	2012	-20914	Alive	TUMOR F	906						R0	0	906	0	906	0	906	0	906
TCGA-LG	TCGA-LG	LIHC	79	FEMALE	WHITE	Stage II	Hepatoce	G2	2013	-28871	Alive	TUMOR F	387						R0	0	387	0	387	0	387	0	387
TCGA-LG	TCGA-LG	LIHC	48	MALE	WHITE	Stage I	Hepatoce	G2	2013	-17842	Alive	TUMOR F	425						R0	0	425	0	425	0	425	0	425
TCGA-LG	TCGA-LG	LIHC	68	MALE	WHITE	Stage IIIA	Hepatoce	G2	2013	-24969	Alive	WITH TUN	366						R0	0	366	0	366	0	366	0	366
TCGA-MI	TCGA-MI	LIHC	64	MALE	WHITE	Stage I	Hepatoce	G3	2012	-23441	Alive	TUMOR F	291						R0	0	291	0	291	0	291	0	291
TCGA-MI	TCGA-MI	LIHC	61	MALE	WHITE	Stage IIIC	Hepatoce	G2	2013	-22457	Alive	TUMOR F	507						R0	0	507	0	507	0	507	0	507
TCGA-MI	TCGA-MI	LIHC	63	MALE	WHITE	Stage II	Hepatoce	G2	2012	-23284	Alive	TUMOR F	698						R0	0	698	0	698	0	698	0	698
TCGA-MI	TCGA-MI	LIHC	77	MALE	WHITE		Hepatocellular Carci		2012	-28371	Alive	WITH TUN	747		Locoregic	Liver		301	R0	0	747	0	747	1	301	1	301
TCGA-MI	TCGA-MI	LIHC	61	MALE	BLACK OR AFRICAN AMERICAN		Hepatoce	G1	2011	-22341	Alive		630			Liver		265	R0	0	630	0	630	1	265	1	265
TCGA-MF	TCGA-MF	LIHC	58	MALE	WHITE	Stage I	Hepatoce	G1	2012	-21398	Alive	TUMOR F	229							0	229	0	229			0	229
TCGA-MF	TCGA-MF	LIHC	34	MALE	WHITE	Stage I	Fibrolame	G3	2013	-12669	Alive	TUMOR F	330						RX	0	330	0	330			0	330
TCGA-NI	TCGA-NI	LIHC	71	MALE	WHITE	Stage IIIA	Hepatoce	G1	2005	-26011	Dead	WITH TUMOR		1791	Locoregic	Liver		106	R0	1	1791	1	1791	1	106	1	106
TCGA-NI	TCGA-NI	LIHC	74	MALE	WHITE	Stage I	Hepatoce	G3	2012	-27162	Alive	TUMOR F	799						R0	0	799	0	799	0	799	0	799
TCGA-O8	TCGA-O8	LIHC	54	MALE	[Unknowr	Stage I	Hepatoce	G2	2013	-19850	Alive	TUMOR F	538						R1	0	538	0	538			0	538
TCGA-PD	TCGA-PD	LIHC	58	FEMALE	WHITE	Stage IIIB	Hepatoce	G2	2007	-21414	Dead	WITH TUMOR		639						1	639	1	639			1	639
TCGA-OA	TCGA-OA	LIHC	48	MALE	BLACK OI	Stage II	Hepatoce	G2	2013	-17636	Alive	WITH TUN	94		Locoregic	Liver		88	RX	0	94	0	94			1	88
TCGA-RC	TCGA-RC	LIHC	24	MALE	AMERICA	Stage II	Hepatoce	G3	2009	-8899	Alive		0						R0	0	0	0	0	0	0	0	0
TCGA-RC	TCGA-RC	LIHC	74	FEMALE	WHITE	Stage IIIA	Hepatoce	G2	2010	-27327	Alive		22						R1	0	22	0	22			0	22
TCGA-RC	TCGA-RC	LIHC	20	FEMALE	WHITE	Stage IVA	Fibrolame	G2	2012	-7634	Alive		15						R0	0	15	0	15			0	15
TCGA-RC	TCGA-RC	LIHC	75	MALE	WHITE	Stage II	Hepatoce	G3	2011	-27649	Alive		9						R1	0	9	0	9			0	9
TCGA-RC	TCGA-RC	LIHC	47	FEMALE	ASIAN	Stage I	Hepatoce	G3	2012	-17403	Alive	TUMOR F	640						R0	0	640	0	640	0	640	0	640
TCGA-RC	TCGA-RC	LIHC	53	MALE	ASIAN	Stage II	Hepatoce	G2	2012	-19698	Alive	TUMOR F	588						R0	0	588	0	588	0	588	0	588
TCGA-RC	TCGA-RC	LIHC	66	MALE	ASIAN	Stage I	Hepatoce	G2	2012	-24432	Alive	WITH TUN	579		Locoregic	Liver		502	R0	0	579	0	579	1	502	1	502
TCGA-RC	TCGA-RC	LIHC	42	MALE	ASIAN	Stage II	Hepatoce	G3	2012	-15560	Alive	WITH TUN	468		Extrahepa	Lung		91	R0	0	468	0	468	1	91	1	91
TCGA-RC	TCGA-RC	LIHC	59	MALE	ASIAN	Stage I	Hepatoce	G3	2013	-21814	Alive	TUMOR F	472		Intrahepa	Liver		312	R0	0	472	0	472	1	312	1	312
TCGA-RG	TCGA-RG	LIHC	69	MALE	BLACK OI	Stage II	Hepatoce	G2	2012	-25302	Alive	TUMOR F	1098						R0	0	1098	0	1098	0	1098	0	1098
TCGA-T1	TCGA-T1	LIHC	68	MALE	WHITE		Hepatoce	G2	2013	-24853	Alive	TUMOR F	23						RX	0	23	0	23			0	23
TCGA-UB	TCGA-UB	LIHC	62	FEMALE	WHITE	Stage II	Hepatoch	G2	2013	-22843	Alive	WITH TUN	848		Intrahepa	Liver		221	R1	0	848	0	848			1	221
TCGA-UB	TCGA-UB	LIHC	24	MALE	WHITE	Stage II	Hepatoce	G3	2013	-8951	Alive	WITH TUN	601		Intrahepa	Liver		48	R0	0	601	0	601	1	48	1	48
TCGA-UB	TCGA-UB	LIHC	59	MALE	WHITE	Stage IIIA	Hepatoce	G3	2013	-21733	Alive	WITH TUN	500		Extrahepa	Other, sp	Adrenal g	359	R0	0	500	0	500	1	359	1	359
TCGA-UB	TCGA-UB	LIHC	67	MALE	BLACK OI	Stage I	Hepatoce	G3	2013	-24717	Dead	TUMOR FREE		52					R0	1	52	0	52	0	52	0	52
TCGA-UB	TCGA-UB	LIHC	51	MALE	ASIAN	Stage I	Hepatoce	G2	2013	-18846	Alive	TUMOR F	486						R0	0	486	0	486	0	486	0	486
TCGA-UB	TCGA-UB	LIHC	56	MALE	WHITE	Stage IIIA	Hepatoce	G2	2013	-20815	Dead	WITH TUMOR		214	Intrahepa	Liver		133	R0	1	214	1	214	1	133	1	133
TCGA-UB	TCGA-UB	LIHC	60	MALE	WHITE	Stage II	Hepatoce	G2	2013	-21985	Alive	TUMOR F	327		Intrahepa	Liver		106	R1	0	327	0	327			1	106
TCGA-UB	TCGA-UB	LIHC	69	FEMALE	WHITE	Stage I	Hepatoce	G1	2013	-25340	Alive	TUMOR F	314						R0	0	314	0	314	0	314	0	314
TCGA-WJ	TCGA-WJ	LIHC	68	FEMALE	WHITE	Stage I	Hepatoce	G2	2013	-25018	Alive	TUMOR F	345						R0	0	345	0	345	0	345	0	345
TCGA-WC	TCGA-WC	LIHC	71	FEMALE	WHITE		Hepatoce	G3	2013		Alive	TUMOR F	30						R0	0	30	0	30	0	30	0	30
TCGA-WC	TCGA-WC	LIHC	62	MALE	WHITE	Stage II	Hepatoce	G2	2013	-22769	Alive	WITH TUN	395		Intrahepatic Recurrence			273	R0	0	395	0	395	1	273	1	273
TCGA-WX	TCGA-WX	LIHC	64	FEMALE	WHITE	Stage I	Hepatoce	G3	2011	-23654	Alive	WITH TUN	615		Intrahepa	Liver		246	RX	0	615	0	615			1	246
TCGA-WX	TCGA-WX	LIHC	61	MALE	WHITE	Stage II	Hepatoce	G1	2012	-22639	Alive	TUMOR F	756						R0	0	756	0	756	0	756	0	756
TCGA-WX	TCGA-WX	LIHC	33	FEMALE	WHITE	Stage IIIA	Hepatoce	G2	2012	-12056	Dead	WITH TUMOR		556	Intrahepa	Liver		103	R0	1	556	1	556	1	103	1	103
TCGA-XR	TCGA-XR	LIHC	43	FEMALE	WHITE	Stage I	Hepatoce	G2	2011	-15817	Alive	WITH TUN	1339						R0	0	1339	0	1339	0	1339	0	1339
TCGA-XR	TCGA-XR	LIHC	49	FEMALE	WHITE	Stage IIIB	Hepatoce	G3	2012	-17908	Alive	TUMOR F	1030						R0	0	1030	0	1030	0	1030	0	1030
TCGA-XR	TCGA-XR	LIHC	16	MALE	WHITE	Stage IIIA	Hepatoce	G1	2012	-5862	Alive	WITH TUN	925		Intrahepa	Liver		210	R0	0	925	0	925	1	210	1	210
TCGA-XR	TCGA-XR	LIHC	74	MALE	WHITE	Stage I	Hepatoce	G1	2012	-27145	Dead	TUMOR FREE		693	Intrahepa	Liver		231	R0	1	693	0	693	1	231	1	231
TCGA-XR	TCGA-XR	LIHC	58	MALE	WHITE	Stage I	Hepatoce	G2	2012	-21544	Alive	TUMOR F	898		Intrahepa	Liver		350	R0	0	898	0	898	1	350	1	350
TCGA-YA	TCGA-YA	LIHC	68	MALE	WHITE	Stage IIIA	Hepatoce	G3	2012	-25188	Dead	WITH TUMOR		412	Extrahepa	Brain		398	R0	1	412	1	412	1	398	1	398
TCGA-ZP	TCGA-ZP	LIHC	59	MALE	WHITE		Hepatoce	G1	2011	-21633	Dead	WITH TUMOR		1088					R0	1	1088	1	1088			1	1088
TCGA-ZP	TCGA-ZP	LIHC	66	FEMALE	WHITE		Hepatoce	G1	2011	-24223	Alive	TUMOR F	782						R0	0	782	0	782	0	782	0	782

TCGA-ZP	TCGA-ZP	LIHC	72	MALE	ASIAN		Hepatoce	G1	2011	-26403	Alive	TUMOR F	706							R0	0	706	0	706	0	706	0	706
TCGA-ZP	TCGA-ZP	LIHC	67	FEMALE	BLACK OR AFRICAN AMERICAN		Hepatoce	G1	2011	-24525	Alive	TUMOR F	1091							R0	0	1091	0	1091	0	1091	0	1091
TCGA-ZP	TCGA-ZP	LIHC	56	FEMALE	WHITE		Hepatoce	G2	2012	-20584	Alive	TUMOR F	21							R0	0	21	0	21	0	21	0	21
TCGA-ZP	TCGA-ZP	LIHC	51	MALE	WHITE		Hepatoce	G2	2012	-18717	Dead	WITH TUMOR		765	Intrahepa	Liver		315		R0	1	765	1	765	1	315	1	315
TCGA-ZP	TCGA-ZP	LIHC	64	FEMALE	WHITE		Hepatoce	G1	2012	-23656	Alive	TUMOR F	395							R0	0	395	0	395	0	395	0	395
TCGA-ZS	TCGA-ZS	LIHC	73	MALE	WHITE	Stage II	Hepatoce	G2	2010	-26915	Dead			1386	Intrahepa	Liver		371		R0	1	1386		1386	1	371	1	371
TCGA-ZS	TCGA-ZS	LIHC	79	FEMALE	WHITE	Stage II	Hepatoce	G1	2011	-29051	Alive	TUMOR F	1241		New Prim	Other, sp	Right Terr	892		R0	0	1241	0	1241	0	1241	1	892
TCGA-ZS	TCGA-ZS	LIHC	64	MALE	WHITE	Stage II	Hepatoce	G2	2008	-23510	Alive	TUMOR F	2412		Intrahepa	Liver		636		R0	0	2412	0	2412	1	636	1	636
TCGA-ZS	TCGA-ZS	LIHC	64	MALE	WHITE	Stage II	Hepatoce	G2	2008	-23510	Alive	TUMOR F	2412		Intrahepa	Liver		636		R0	0	2412	0	2412	1	636	1	636
TCGA-ZS	TCGA-ZS	LIHC	55	MALE	WHITE	Stage II	Hepatoce	G2	2013	-20100	Alive	TUMOR F	341							R0	0	341	0	341	0	341	0	341
TCGA-05	TCGA-05	LUAD	70	MALE		Stage IV	Lung Adenocarcinom		2009	-25752	Alive	TUMOR F	0								0	0	0	0			0	0
TCGA-05	TCGA-05	LUAD	67	MALE		Stage IB	Lung Adenocarcinom		2007	-24532	Alive	TUMOR F	1523								0	1523	0	1523			0	1523
TCGA-05	TCGA-05	LUAD	79	FEMALE		Stage IIIA	Lung Adenocarcinom		2007	-29068	Dead			121							1	121		121			0	121
TCGA-05	TCGA-05	LUAD	68	MALE		Stage IB	Lung Adenocarcinom		2009	-24868	Alive	TUMOR F	607		Distant Metastasis			334	Complete Remission/Response		0	607	0	607	1	334	1	334
TCGA-05	TCGA-05	LUAD	66	MALE		Stage IIIA	Lung Adenocarcinom		2009	-24411	Alive	WITH TUM	426		Distant Metastasis			183	Progressive Disease		0	426	0	426			1	183
TCGA-05	TCGA-05	LUAD	70	MALE		Stage IA	Lung Adenocarcinom		2005	-25660	Alive	TUMOR F	1369								0	1369	0	1369			0	1369
TCGA-05	TCGA-05	LUAD	58	FEMALE		Stage IB	Lung Adenocarcinom		2005	-21430	Alive	TUMOR F	1126					395	Progressive Disease		0	1126	0	1126			1	395
TCGA-05	TCGA-05	LUAD	76	MALE		Stage IIIB	Lung Adenocarcinom		2006	-27971	Dead	TUMOR FREE		0							1	0	0	0			0	0
TCGA-05	TCGA-05	LUAD	76	MALE		Stage IIIB	Lung Adenocarcinom		2006	-28094	Dead			303							1	303		303			0	303
TCGA-05	TCGA-05	LUAD	65	MALE		Stage IIB	Lung Adenocarcinom		2006	-23833	Dead			731							1	731		731			0	731
TCGA-05	TCGA-05	LUAD	47	FEMALE		Stage IIIB	Lung Adenocarcinom		2006	-17471	Alive	TUMOR F	1431						Complete Remission/Response		0	1431	0	1431	0	1431	0	1431
TCGA-05	TCGA-05	LUAD	57	FEMALE		Stage IV	Lung Adenocarcinom		2007	-20819	Dead	TUMOR FREE		244					Complete Remission/Response		1	244	0	244			0	244
TCGA-05	TCGA-05	LUAD	76	MALE		Stage IB	Lung Adenocarcinom		2006	-27881	Alive		578								0	578	0	578			0	578
TCGA-05	TCGA-05	LUAD	74	FEMALE		Stage IB	Lung Adenocarcinom		2006	-27241	Alive	TUMOR F	610								0	610	0	610			0	610
TCGA-05	TCGA-05	LUAD	62	MALE		Stage IB	Lung Adenocarcinom		2007	-22888	Alive		0								0	0	0	0			0	0
TCGA-05	TCGA-05	LUAD	57	MALE		Stage IIIB	Lung Adenocarcinom		2008	-20880	Dead	WITH TUMOR		91				60	Progressive Disease		1	91	1	91			1	60
TCGA-05	TCGA-05	LUAD	51	FEMALE		Stage IB	Lung Adenocarcinom		2008	-18780	Alive	TUMOR F	455						Partial Remission/Response		0	455	0	455			0	455
TCGA-05	TCGA-05	LUAD	69	MALE		Stage IIIA	Lung Adenocarcinom		2008	-25417	Dead			274							1	274		274			0	274
TCGA-05	TCGA-05	LUAD	41	MALE		Stage IB	Lung Adenocarcinom		2008	-15159	Alive	TUMOR F	912						Complete Remission/Response		0	912	0	912	0	912	0	912
TCGA-05	TCGA-05	LUAD	68	MALE		Stage IB	Lung Adenocarcinom		2008	-24837	Alive		365								0	365	0	365			0	365
TCGA-05	TCGA-05	LUAD	70	MALE		Stage IIB	Lung Adenocarcinom		2008	-25689	Alive	WITH TUM	913		Distant Metastasis			153	Progressive Disease		0	913	0	913			1	153
TCGA-05	TCGA-05	LUAD	70	FEMALE		Stage IV	Lung Adenocarcinom		2008	-25902	Alive		669								0	669	0	669			0	669
TCGA-05	TCGA-05	LUAD	71	MALE		Stage IB	Lung Adenocarcinom		2008	-26084	Alive	TUMOR F	791					457	Complete Remission/Response		0	791	0	791	1	457	1	457
TCGA-05	TCGA-05	LUAD	65	FEMALE		Stage IIB	Lung Adenocarcinom		2008	-23893	Alive	TUMOR F	791						Complete Remission/Response		0	791	0	791	0	791	0	791
TCGA-05	TCGA-05	LUAD	59	FEMALE		Stage IB	Lung Adenocarcinom		2008	-21884	Alive	TUMOR F	761								0	761	0	761			0	761
TCGA-05	TCGA-05	LUAD	66	MALE		Stage IIB	Lung Adenocarcinom		2008	-24350	Alive	TUMOR F	761						Complete Remission/Response		0	761	0	761	0	761	0	761
TCGA-05	TCGA-05	LUAD	82	MALE		Stage IB	Lung Adenocarcinom		2008	-30194	Alive	TUMOR F	730								0	730	0	730			0	730
TCGA-05	TCGA-05	LUAD	67	FEMALE		Stage IV	Lung Adenocarcinom		2008	-24472	Dead			457							1	457		457			0	457
TCGA-05	TCGA-05	LUAD	67	MALE		Stage IIIA	Lung Adenocarcinom		2008	-24472	Alive	WITH TUM	457					245	Progressive Disease		0	457	0	457			1	245
TCGA-05	TCGA-05	LUAD	67	MALE		Stage IIIA	Lung Adenocarcinom		2008	-24472	Alive	WITH TUM	457					245	Progressive Disease		0	457	0	457			1	245
TCGA-05	TCGA-05	LUAD	65	MALE		Stage IIB	Lung Adenocarcinom		2006	-23863	Alive	TUMOR F	151								0	151	0	151			0	151
TCGA-05	TCGA-05	LUAD	68	MALE		Stage IIB	Lung Adenocarcinom		2009	-25020	Alive	WITH TUM	882					486			0	882	0	882			1	486
TCGA-05	TCGA-05	LUAD	57	MALE		Stage IIA	Lung Adenocarcinom		2010	-21061	Alive	TUMOR F	670						Complete Remission/Response		0	670	0	670	0	670	0	670
TCGA-05	TCGA-05	LUAD	60	MALE		Stage IIIA	Lung Adenocarcinom		2010	-22066	Dead	TUMOR FREE		275							1	275	0	275			0	275
TCGA-05	TCGA-05	LUAD	69	FEMALE		Stage IB	Lung Adenocarcinom		2010	-25383	Alive	TUMOR F	62								0	62	0	62			0	62
TCGA-35	TCGA-35	LUAD	57	MALE	WHITE	Stage IB	Lung Adenocarcinom		2007	-21076	Alive	TUMOR F	14								0	14	0	14			0	14
TCGA-35	TCGA-35	LUAD	69	MALE	WHITE	Stage IA	Lung Adenocarcinom		2010	-25491	Alive	TUMOR F	225						[Unknown]		0	225	0	225			0	225
TCGA-35	TCGA-35	LUAD	38	MALE	WHITE	Stage IA	Lung Adenocarcinom		2010	-14064	Alive	TUMOR F	182								0	182	0	182			0	182
TCGA-35	TCGA-35	LUAD	61	MALE	WHITE	Stage IIIA	Lung Adenocarcinom		2010	-22628	Alive	TUMOR F	264								0	264	0	264			0	264
TCGA-38	TCGA-38	LUAD	66	FEMALE	WHITE	Stage IB	Lung Adenocarcinom		2002	-24241	Alive		2973						Complete Remission/Response		0	2973	0	2973	0	2973	0	2973
TCGA-38	TCGA-38	LUAD	66	FEMALE	WHITE	Stage IB	Lung Adenocarcinom		2002	-24241	Alive		2973						Complete Remission/Response		0	2973	0	2973	0	2973	0	2973
TCGA-38	TCGA-38	LUAD	57	FEMALE	WHITE	[Discrepancy]	Lung Adenocarcinom		2003	-20903	Alive	TUMOR F	3674		New Primary Tumor			2518	Complete Remission/Response		0	3674	0	3674	0	3674	1	2518
TCGA-38	TCGA-38	LUAD	57	FEMALE	WHITE	[Discrepancy]	Lung Adenocarcinom		2003	-20903	Alive	TUMOR F	3674		New Primary Tumor			2518	Complete Remission/Response		0	3674	0	3674	0	3674	1	2518
TCGA-38	TCGA-38	LUAD	64	FEMALE	WHITE	Stage IIA	Lung Adenocarcinom		2003	-23510	Dead			1147					Complete Remission/Response		1	1147		1147	0	1147	0	1147
TCGA-38	TCGA-38	LUAD	64	FEMALE	WHITE	Stage IIA	Lung Adenocarcinom		2003	-23510	Dead			1147					Complete Remission/Response		1	1147		1147	0	1147	0	1147
TCGA-38	TCGA-38	LUAD	65	FEMALE	WHITE	Stage IIB	Lung Adenocarcinom		2002	-24057	Dead	WITH TUMOR		1492				1083	Complete Remission/Response		1	1492	1	1492	1	1083	1	1083
TCGA-38	TCGA-38	LUAD	65	FEMALE	WHITE	Stage IIB	Lung Adenocarcinom		2002	-24057	Dead	WITH TUMOR		1492				1083	Complete Remission/Response		1	1492	1	1492	1	1083	1	1083
TCGA-38	TCGA-38	LUAD	68	MALE	WHITE	Stage IIB	Lung Adenocarcinom		2003	-25104	Dead	WITH TUMOR		864				379	Complete Remission/Response		1	864	1	864	1	379	1	379
TCGA-38	TCGA-38	LUAD	68	MALE	WHITE	Stage IIB	Lung Adenocarcinom		2003	-25104	Dead	WITH TUMOR		864				379	Complete Remission/Response		1	864	1	864	1	379	1	379
TCGA-38	TCGA-38	LUAD	75	FEMALE	WHITE	Stage IB	Lung Adenocarcinom		2000	-27667	Dead	WITH TUMOR		1073				524	Complete Remission/Response		1	1073	1	1073	1	524	1	524
TCGA-38	TCGA-38	LUAD	75	FEMALE	WHITE	Stage IB	Lung Adenocarcinom		2000	-27667	Dead	WITH TUMOR		1073				524	Complete Remission/Response		1	1073	1	1073	1	524	1	524
TCGA-38	TCGA-38	LUAD	72	FEMALE	WHITE	Stage IB	Lung Adenocarcinom		1997	-26538	Dead	WITH TUMOR		354					Progressive Disease		1	354	1	354			1	354
TCGA-38	TCGA-38	LUAD	72	FEMALE	WHITE	Stage IB	Lung Adenocarcinom		1997	-26538	Dead	WITH TUMOR		354					Progressive Disease		1	354	1	354			1	354
TCGA-38	TCGA-38	LUAD	42	MALE	BLACK OR	Stage IV	Lung Adenocarcinom		1998	-15418	Dead	WITH TUMOR		1357				680	Complete Remission/Response		1	1357	1	1357			1	680
TCGA-38	TCGA-38	LUAD	42	MALE	BLACK OR	Stage IV	Lung Adenocarcinom		1998	-15418	Dead	WITH TUMOR		1357				680	Complete Remission/Response		1	1357	1	1357			1	680
TCGA-38	TCGA-38	LUAD	70	FEMALE	WHITE	Stage IIIA	Lung Adenocarcinom		2010	-25593	Alive	WITH TUM	448						Stable Disease		0	448	0	448			0	448
TCGA-38	TCGA-38	LUAD	72	FEMALE	WHITE	Stage IA	Lung Adenocarcinom		2006	-26440	Dead	WITH TUMOR		800				304	Complete Remission/Response		1	800	1	800	1	304	1	304
TCGA-38	TCGA-38	LUAD	80	MALE	WHITE	Stage IB	Lung Adenocarcinom		2012	-29534	Alive	TUMOR F	133						Complete Remission/Response		0	133	0	133	0	133	0	133
TCGA-44	TCGA-44	LUAD	65	FEMALE	WHITE	Stage IA	Lung Adenocarcinom		2009	-23854	Alive	TUMOR F	1324		New Primary Tumor			1009	Complete Remission/Response		0	1324	0	1324	0	1324	1	1009
TCGA-44	TCGA-44	LUAD	65	FEMALE	WHITE	Stage IA	Lung Adenocarcinom		2009	-23854	Alive	TUMOR F	1324		New Primary Tumor			1009	Complete Remission/Response		0	1324	0	1324	0	1324	1	1009
TCGA-44	TCGA-44	LUAD	59	MALE	WHITE	Stage IB	Lung Adenocarcinom		2009	-21766	Alive	TUMOR F	1429		New Primary Tumor			568	Complete Remission/Response		0	1429	0	1429	0	1429	1	568
TCGA-44	TCGA-44	LUAD	59	MALE	WHITE	Stage IB	Lung Adenocarcinom		2009	-21766	Alive	TUMOR F	1429		New Primary Tumor			568	Complete Remission/Response		0	1429	0	1429	0	1429	1	568
TCGA-44	TCGA-44	LUAD	74	FEMALE	WHITE	Stage IB	Lung Adenocarcinom		2009	-27298	Alive	TUMOR F	1351						Complete Remission/Response		0	1351	0	1351	0	1351	0	1351
TCGA-44	TCGA-44	LUAD	74	FEMALE	WHITE	Stage IB	Lung Adenocarcinom		2009	-27298	Alive	TUMOR F	1351						Complete Remission/Response		0	1351	0	1351	0	1351	0	1351
TCGA-44	TCGA-44	LUAD	65	FEMALE	WHITE	Stage IIB	Lung Adenocarcinom		2009	-23808	Alive	TUMOR F	1367		Locoregional Recurrence			1146	Complete Remission/Response		0	1367	0	1367	1	1146	1	1146
TCGA-44	TCGA-44	LUAD	65	FEMALE	WHITE	Stage IIB	Lung Adenocarcinom		2009	-23808	Alive	TUMOR F	1367		Locoregional Recurrence			1146	Complete Remission/Response		0	1367	0	1367	1	1146	1	1146
TCGA-44	TCGA-44	LUAD	69	FEMALE	WHITE	Stage IA	Lung Adenocarcinom		2009	-25313	Alive	TUMOR F	1159						Complete Remission/Response		0	1159	0	1159	0	1159	0	1159
TCGA-44	TCGA-44	LUAD	69	FEMALE	WHITE	Stage IA	Lung Adenocarcinom		2009	-25313	Alive	TUMOR F	1159						Complete Remission/Response		0	1159	0	1159	0	1159	0	1159
TCGA-44	TCGA-44	LUAD	65	MALE	WHITE	Stage IB	Lung Adenocarcinom		2009	-24014	Alive	TUMOR F	1280					245	Progressive Disease		0	1280	0	1280			1	245
TCGA-44	TCGA-44	LUAD	65	MALE	WHITE	Stage IB	Lung Adenocarcinom		2009	-24014	Alive	TUMOR F	1280					245	Progressive Disease		0	1280	0	1280			1	245
TCGA-44	TCGA-44	LUAD	55	FEMALE	WHITE	Stage IIB	Lung Adenocarcinom		2009	-20349	Alive	TUMOR F	1301						Stable Disease		0	1301	0	1301			0	1301
TCGA-44	TCGA-44	LUAD	55	FEMALE	WHITE	Stage IIB	Lung Adenocarcinom		2009	-20349	Alive	TUMOR F	1301						Stable Disease		0	1301	0	1301			0	1301
TCGA-44	TCGA-44	LUAD	43	MALE	WHITE	Stage IB	Lung Adenocarcinom		2009	-15970	Dead	WITH TUM	97	97							1	97	1	97			1	97
TCGA-44	TCGA-44	LUAD	51	MALE	WHITE	Stage IB	Lung Adenocarcinom		2009	-18856	Dead	WITH TUMOR		761				433	Complete Remission/Response		1	761	1	761	1	433	1	433
TCGA-44	TCGA-44	LUAD	51	MALE	WHITE	Stage IB	Lung Adenocarcinom		2009	-18856	Dead	WITH TUMOR		761				433	Complete Remission/Response		1	761	1	761	1	433	1	433
TCGA-44	TCGA-44	LUAD	74	FEMALE	WHITE	Stage IIIA	Lung Adenocarcinom		2009	-27073	Alive	TUMOR F	1130						Complete Remission/Response		0	1130	0	1130	0	1130	0	1130
TCGA-44	TCGA-44	LUAD	74	FEMALE	WHITE	Stage IIIA	Lung Adenocarcinom		2009	-27073	Alive	TUMOR F	1130						Complete Remission/Response		0	1130	0	1130	0	1130	0	1130
TCGA-44	TCGA-44	LUAD	77	FEMALE	WHITE	Stage IA	Lung Adenocarcinom		2009	-28392	Alive	TUMOR F	1163						Complete Remission/Response		0	1163	0	1163	0	1163	0	1163
TCGA-44	TCGA-44	LUAD	77	FEMALE	WHITE	Stage IA	Lung Adenocarcinom		2009	-28392	Alive	TUMOR F	1163						Complete Remission/Response		0	1163	0	1163	0	1163	0	1163
TCGA-44	TCGA-44	LUAD	33	FEMALE	WHITE	Stage IB	Lung Adenocarcinom		2009	-12179	Alive	TUMOR F	1183						Complete Remission/Response		0	1183	0	1183	0	1183	0	1183
TCGA-44	TCGA-44	LUAD	60	FEMALE	WHITE	Stage IA	Lung Adenocarcinom		2010	-22236	Alive	WITH TUM	1036					511	Complete Remission/Response		0	1036	0	1036	1	511	1	511
TCGA-44	TCGA-44	LUAD	60	FEMALE	WHITE	Stage IA	Lung Adenocarcinom		2010	-22236	Alive	WITH TUM	1036					511	Complete Remission/Response		0	1036	0	1036	1	511	1	511
TCGA-44	TCGA-44	LUAD	71	FEMALE	WHITE	Stage IA	Lung Adenocarcinom		2010	-26081	Dead	WITH TUMOR		1026	Locoregional Recurrence			921	Complete Remission/Response		1	1026	1	1026	1	921	1	921
TCGA-44	TCGA-44	LUAD	71	FEMALE	WHITE	Stage IA	Lung Adenocarcinom		2010	-26081	Dead	WITH TUMOR		1026	Locoregional Recurrence			921	Complete Remission/Response		1	1026	1	1026	1	921	1	921
TCGA-44	TCGA-44	LUAD	60	FEMALE	WHITE	Stage IB	Lung Adenocarcinom		2010	-22106	Dead	WITH TUMOR		808				619	Complete Remission/Response		1	808	1	808	1	619	1	619
TCGA-44	TCGA-44	LUAD	60	FEMALE	WHITE	Stage IB	Lung Adenocarcinom		2010	-22106	Dead	WITH TUMOR		808				619	Complete Remission/Response		1	808	1	808	1	619	1	619
TCGA-44	TCGA-44	LUAD	53	MALE	BLACK OR	Stage IIIA	Lung Adenocarcinom		2010	-19426	Alive	TUMOR F	1013								0	1013	0	1013			0	1013
TCGA-44	TCGA-44	LUAD	53	MALE	BLACK OR	Stage IIIA	Lung Adenocarcinom		2010	-19426	Alive	TUMOR F	1013								0	1013	0	1013			0	1013
TCGA-44	TCGA-44	LUAD	51	FEMALE	WHITE	Stage IB	Lung Adenocarcinom		2010	-18644	Alive	TUMOR F	863						Complete Remission/Response		0	863	0	863	0	863	0	863
TCGA-44	TCGA-44	LUAD	61	FEMALE	BLACK OR	Stage IA	Lung Adenocarcinom		2010	-22471	Alive	TUMOR F	852						Complete Remission/Response		0	852	0	852	0	852	0	852
TCGA-44	TCGA-44	LUAD	61	FEMALE	BLACK OR	Stage IA	Lung Adenocarcinom		2010	-22471	Alive	TUMOR F	852						Complete Remission/Response		0	852	0	852	0	852	0	852
TCGA-44	TCGA-44	LUAD	58	MALE	WHITE	Stage IA	Lung Adenocarcinom		2011	-21283	Alive	WITH TUM	723					399	Complete Remission/Response		0	723	0	723	1	399	1	399
TCGA-44	TCGA-44	LUAD	58	MALE	WHITE	Stage IA	Lung Adenocarcinom		2011	-21283	Alive	WITH TUM	723					399	Complete Remission/Response		0	723	0	723	1	399	1	399
TCGA-44	TCGA-44	LUAD	62	FEMALE	WHITE	Stage IA	Lung Adenocarcinom		2011	-22745	Alive	TUMOR F	595						Complete Remission/Response		0	595	0	595	0	595	0	595
TCGA-44	TCGA-44	LUAD	62	FEMALE	WHITE	Stage IA	Lung Adenocarcinom		2011	-22745	Alive	TUMOR F	595						Complete Remission/Response		0	595	0	595	0	595	0	595
TCGA-44	TCGA-44	LUAD	64	MALE	WHITE	Stage IIB	Lung Adenocarcinom		2010	-23378	Alive	TUMOR F	728		Locoregional Recurrence			631	Complete Remission/Response		0	728	0	728	1	631	1	631
TCGA-44	TCGA-44	LUAD	64	MALE	WHITE	Stage IIB	Lung Adenocarcinom		2010	-23378	Alive	TUMOR F	728		Locoregional Recurrence			631	Complete Remission/Response		0	728	0	728	1	631	1	631
TCGA-44	TCGA-44	LUAD	67	FEMALE	WHITE	Stage IA	Lung Adenocarcinom		2010	-24631	Alive	TUMOR F	845						Complete Remission/Response		0	845	0	845	0	845	0	845
TCGA-44	TCGA-44	LUAD	67	FEMALE	WHITE	Stage IA	Lung Adenocarcinom		2010	-24631	Alive	TUMOR F	845						Complete Remission/Response		0	845	0	845	0	845	0	845
TCGA-44	TCGA-44	LUAD	60	MALE	WHITE	Stage IA	Lung Adenocarcinom		2011	-22138	Alive	TUMOR F	704						Complete Remission/Response		0	704	0	704	0	704	0	704
TCGA-44	TCGA-44	LUAD	60	MALE	WHITE	Stage IA	Lung Adenocarcinom		2011	-22138	Alive	TUMOR F	704						Complete Remission/Response		0	704	0	704	0	704	0	704
TCGA-44	TCGA-44	LUAD	56	FEMALE	WHITE	Stage IIIA	Lung Adenocarcinom		2011	-20462	Alive	TUMOR F	658						Complete Remission/Response		0	658	0	658	0	658	0	658
TCGA-44	TCGA-44	LUAD	72	FEMALE	WHITE	Stage IB	Lung Adenocarcinom		2011	-26415	Alive	WITH TUM	705		Locoregional Recurrence			684	Complete Remission/Response		0	705	0	705	1	684	1	684
TCGA-44	TCGA-44	LUAD	60	FEMALE	WHITE	Stage IA	Lung Adenocarcinom		2005	-22150	Alive	TUMOR F	2616						Complete Remission/Response		0	2616	0	2616	0	2616	0	2616
TCGA-44	TCGA-44	LUAD	60	FEMALE	WHITE	Stage IA	Lung Adenocarcinom		2005	-22150	Alive	TUMOR F	2616						Complete Remission/Response		0	2616	0	2616	0	2616	0	2616
TCGA-44	TCGA-44	LUAD	85	FEMALE	WHITE	Stage IB	Lung Adenocarcinom		2006	-31234	Dead	TUMOR FREE		987					Complete Remission/Response		1	987	0	987	0	987	0	987
TCGA-44	TCGA-44	LUAD	85	FEMALE	WHITE	Stage IB	Lung Adenocarcinom		2006	-31234	Dead	TUMOR FREE		987					Complete Remission/Response		1	987	0	987	0	987	0	987
TCGA-44	TCGA-44	LUAD	59	MALE	BLACK OR	Stage IA	Lung Adenocarcinom		2007	-21725	Alive	TUMOR F	1864						Complete Remission/Response		0	1864	0	1864	0	1864	0	1864
TCGA-44	TCGA-44	LUAD	59	MALE	BLACK OR	Stage IA	Lung Adenocarcinom		2007	-21725	Alive	TUMOR F	1864						Complete Remission/Response		0	1864	0	1864	0	1864	0	1864
TCGA-44	TCGA-44	LUAD	50	FEMALE	WHITE	Stage IIB	Lung Adenocarcinom		2008	-18469	Dead	WITH TUMOR		500				232	Progressive Disease		1	500	1	500			1	232
TCGA-44	TCGA-44	LUAD	70	MALE	WHITE	Stage IA	Lung Adenocarcinom		2010	-25730	Alive	TUMOR F	691						Complete Remission/Response		0	691	0	691	0	691	0	691
TCGA-44	TCGA-44	LUAD	70	MALE	WHITE	Stage IA	Lung Adenocarcinom		2010	-25730	Alive	TUMOR F	691						Complete Remission/Response		0	691	0	691	0	691	0	691
TCGA-44	TCGA-44	LUAD	72	MALE	WHITE	Stage IB	Lung Adenocarcinom		2011	-26594	Alive	WITH TUM	592		Locoregional Recurrence			253	Progressive Disease		0	592	0	592			1	253
TCGA-44	TCGA-44	LUAD	72	MALE	WHITE	Stage IB	Lung Adenocarcinom		2011	-26594	Alive	WITH TUM	592		Locoregional Recurrence			253	Progressive Disease		0	592	0	592			1	253
TCGA-44	TCGA-44	LUAD	69	FEMALE	WHITE	Stage IB	Lung Adenocarcinom		2011	-25483	Dead	WITH TUMOR		557	Locoregional Recurrence			335	Complete Remission/Response		1	557	1	557	1	335	1	335
TCGA-44	TCGA-44	LUAD	69	FEMALE	WHITE	Stage IB	Lung Adenocarcinom		2011	-25483	Dead	WITH TUMOR		557	Locoregional Recurrence			335	Complete Remission/Response		1	557	1	557	1	335	1	335
TCGA-44	TCGA-44	LUAD	61	MALE	WHITE	Stage IB	Lung Adenocarcinom		2011	-22543	Alive	TUMOR F	218						Complete Remission/Response		0	218	0	218	0	218	0	218
TCGA-44	TCGA-44	LUAD	61	MALE	WHITE	Stage IB	Lung Adenocarcinom		2011	-22543	Alive	TUMOR F	218						Complete Remission/Response		0	218	0	218	0	218	0	218
TCGA-44	TCGA-44	LUAD	49	FEMALE	WHITE	Stage IIB	Lung Adenocarcinom		2009	-18062	Alive	TUMOR F	1097						Complete Remission/Response		0	1097	0	1097	0	1097	0	1097
TCGA-44	TCGA-44	LUAD	49	FEMALE	WHITE	Stage IIB	Lung Adenocarcinom		2009	-18062	Alive	TUMOR F	1097						Complete Remission/Response		0	1097	0	1097	0	1097	0	1097
TCGA-44	TCGA-44	LUAD	59	MALE	BLACK OR	Stage IIA	Lung Adenocarcinom		2010	-21893	Dead	WITH TUMOR		574				390	Complete Remission/Response		1	574	1	574	1	390	1	390
TCGA-44	TCGA-44	LUAD	59	MALE	BLACK OR	Stage IIA	Lung Adenocarcinom		2010	-21893	Dead	WITH TUMOR		574				390	Complete Remission/Response		1	574	1	574	1	390	1	390
TCGA-44	TCGA-44	LUAD	47	FEMALE	WHITE	Stage IIA	Lung Adenocarcinom		2010	-17392	Alive	TUMOR F	882						Complete Remission/Response		0	882	0	882	0	882	0	882
TCGA-44	TCGA-44	LUAD	47	FEMALE	WHITE	Stage IIA	Lung Adenocarcinom		2010	-17392	Alive	TUMOR F	882						Complete Remission/Response		0	882	0	882	0	882	0	882
TCGA-44	TCGA-44	LUAD	64	MALE	BLACK OR	Stage IB	Lung Adenocarcinom		2010	-23538	Alive	WITH TUM	889		Locoregional Recurrence			883	Complete Remission/Response		0	889	0	889	1	883	1	883
TCGA-44	TCGA-44	LUAD	64	MALE	BLACK OR	Stage IB	Lung Adenocarcinom		2010	-23538	Alive	WITH TUM	889		Locoregional Recurrence			883	Complete Remission/Response		0	889	0	889	1	883	1	883
TCGA-44	TCGA-44	LUAD	52	FEMALE	WHITE	Stage IA	Lung Adenocarcinom		2010	-19200	Alive	TUMOR F	719						Complete Remission/Response		0	719	0	719	0	719	0	719
TCGA-44	TCGA-44	LUAD	52	FEMALE	WHITE	Stage IA	Lung Adenocarcinom		2010	-19200	Alive	TUMOR F	719						Complete Remission/Response		0	719	0	719	0	719	0	719
TCGA-44	TCGA-44	LUAD	54	FEMALE	WHITE	Stage IB	Lung Adenocarcinom		2011	-19855	Alive	TUMOR F	385						Complete Remission/Response		0	385	0	385	0	385	0	385
TCGA-44	TCGA-44	LUAD	73	MALE	WHITE	Stage IIB	Lung Adenocarcinom		2012	-27003	Alive	TUMOR F	285						Complete Remission/Response		0	285	0	285	0	285	0	285
TCGA-44	TCGA-44	LUAD	58	MALE	BLACK OR	Stage IB	Lung Adenocarcinom		2011	-21188	Alive	TUMOR F	260						Complete Remission/Response		0	260	0	260	0	260	0	260
TCGA-44	TCGA-44	LUAD	73	FEMALE	BLACK OR	Stage IB	Lung Adenocarcinom		2012	-27024	Alive	TUMOR F	486		New Primary Tumor			437	Complete Remission/Response		0	486	0	486	0	486	1	437
TCGA-44	TCGA-44	LUAD	78	FEMALE	WHITE	Stage IB	Lung Adenocarcinom		2012	-28782	Alive	WITH TUM	466		Distant Metastasis			397	Complete Remission/Response		0	466	0	466	1	397	1	397
TCGA-44	TCGA-44	LUAD	79	MALE	WHITE	Stage IB	Lung Adenocarcinom		2012	-28869	Alive	TUMOR F	287						Complete Remission/Response		0	287	0	287	0	287	0	287
TCGA-44	TCGA-44	LUAD	74	MALE	WHITE	Stage IIB	Lung Adenocarcinom		2012	-27122	Alive	TUMOR F	337						Complete Remission/Response		0	337	0	337	0	337	0	337
TCGA-44	TCGA-44	LUAD	73	FEMALE	WHITE	Stage IA	Lung Adenocarcinom		2012	-26800	Alive	TUMOR F	351						Complete Remission/Response		0	351	0	351	0	351	0	351
TCGA-44	TCGA-44	LUAD	73	MALE	WHITE	Stage IA	Lung Adenocarcinom		2012	-26991	Alive	TUMOR F	415						Complete Remission/Response		0	415	0	415	0	415	0	415
TCGA-44	TCGA-44	LUAD	67	FEMALE	WHITE	Stage IA	Lung Adenocarcinom		2012	-24820	Dead	WITH TUMOR		409	Locoregional Recurrence			260	Complete Remission/Response		1	409	1	409	1	260	1	260
TCGA-49	TCGA-49	LUAD	72	MALE	WHITE	Stage IA	Lung Adenocarcinom		1992	-26415	Dead	WITH TUMOR		2318	Distant Metastasis			2045	Complete Remission/Response		1	2318	1	2318	1	2045	1	2045
TCGA-49	TCGA-49	LUAD	72	MALE	WHITE	Stage IA	Lung Adenocarcinom		1992	-26415	Dead	WITH TUMOR		2318	Distant Metastasis			2045	Complete Remission/Response		1	2318	1	2318	1	2045	1	2045
TCGA-49	TCGA-49	LUAD	72	FEMALE	WHITE	Stage IA	Lung Adenocarcinom		1992	-26583	Dead	WITH TUMOR		855				697	Complete Remission/Response		1	855	1	855	1	697	1	697
TCGA-49	TCGA-49	LUAD	74	FEMALE	WHITE	Stage IA	Lung Adenocarcinom		1992	-27332	Dead			869				634			1	869		869			1	634
TCGA-49	TCGA-49	LUAD	74	FEMALE	WHITE	Stage IA	Lung Adenocarcinom		1992	-27332	Dead			869				634			1	869		869			1	634
TCGA-49	TCGA-49	LUAD	45	FEMALE	WHITE	Stage IIIA	Lung Adenocarcinom		1993	-16634	Dead	WITH TUMOR		385							1	385	1	385			1	385
TCGA-49	TCGA-49	LUAD	45	FEMALE	WHITE	Stage IIIA	Lung Adenocarcinom		1993	-16634	Dead	WITH TUMOR		385							1	385	1	385			1	385
TCGA-49	TCGA-49	LUAD	77	MALE	WHITE	Stage IIIA	Lung Adenocarcinom		1995	-28387	Dead	WITH TUMOR		1081					Complete Remission/Response		1	1081	1	1081			1	1081
TCGA-49	TCGA-49	LUAD	77	MALE	WHITE	Stage IIIA	Lung Adenocarcinom		1995	-28387	Dead	WITH TUMOR		1081					Complete Remission/Response		1	1081	1	1081			1	1081
TCGA-49	TCGA-49	LUAD	67	FEMALE	WHITE	Stage IB	Lung Adenocarcinom		1996	-24817	Dead	WITH TUMOR		1421				545	Complete Remission/Response		1	1421	1	1421	1	545	1	545
TCGA-49	TCGA-49	LUAD	67	FEMALE	WHITE	Stage IB	Lung Adenocarcinom		1996	-24817	Dead	WITH TUMOR		1421				545	Complete Remission/Response		1	1421	1	1421	1	545	1	545
TCGA-49	TCGA-49	LUAD	61	FEMALE	WHITE	Stage IIB	Lung Adenocarcinom		1996	-22628	Dead	WITH TUMOR		428	Distant Metastasis|New Primary			417	Complete Remission/Response		1	428	1	428	1	417	1	417
TCGA-49	TCGA-49	LUAD	61	FEMALE	WHITE	Stage IIB	Lung Adenocarcinom		1996	-22628	Dead	WITH TUMOR		428	Distant Metastasis|New Primary			417	Complete Remission/Response		1	428	1	428	1	417	1	417
TCGA-49	TCGA-49	LUAD	68	FEMALE	WHITE	Stage IIB	Lung Adenocarcinom		1997	-25132	Dead	[Discrepancy]		999	Locoregional Recurrence			692	Complete Remission/Response		1	999		999	1	692	1	692
TCGA-49	TCGA-49	LUAD	68	FEMALE	WHITE	Stage IIB	Lung Adenocarcinom		1997	-25132	Dead	[Discrepancy]		999	Locoregional Recurrence			692	Complete Remission/Response		1	999		999	1	692	1	692
TCGA-49	TCGA-49	LUAD	73	FEMALE	WHITE	Stage IIIA	Lung Adenocarcinom		1997	-26851	Dead	WITH TUMOR		268				158	Progressive Disease		1	268	1	268			1	158
TCGA-49	TCGA-49	LUAD	73	FEMALE	WHITE	Stage IIIA	Lung Adenocarcinom		1997	-26851	Dead	WITH TUMOR		268				158	Progressive Disease		1	268	1	268			1	158
TCGA-49	TCGA-49	LUAD	51	FEMALE	BLACK OR	Stage IIB	Lung Adenocarcinom		2001	-18762	Dead	WITH TUMOR		896	Distant Metastasis			502	Stable Disease		1	896	1	896			1	502
TCGA-49	TCGA-49	LUAD	51	FEMALE	BLACK OR	Stage IIB	Lung Adenocarcinom		2001	-18762	Dead	WITH TUMOR		896	Distant Metastasis			502	Stable Disease		1	896	1	896			1	502
TCGA-49	TCGA-49	LUAD	69	FEMALE	WHITE	Stage IIIA	Lung Adenocarcinom		2007	-25502	Dead	WITH TUM	157	905					Progressive Disease		1	905	1	905			1	905
TCGA-49	TCGA-49	LUAD	69	FEMALE	WHITE	Stage IIIA	Lung Adenocarcinom		2007	-25502	Dead	WITH TUM	157	905					Progressive Disease		1	905	1	905			1	905
TCGA-49	TCGA-49	LUAD	79	FEMALE	WHITE	Stage IA	Lung Adenocarcinom		2007	-28908	Alive	TUMOR F	1700						Complete Remission/Response		0	1700	0	1700	0	1700	0	1700
TCGA-49	TCGA-49	LUAD	70	MALE	WHITE	Stage IIA	Lung Adenocarcinom		2010	-25839	Dead	WITH TUMOR		488				214	Progressive Disease		1	488	1	488			1	214
TCGA-49	TCGA-49	LUAD	70	MALE	WHITE	Stage IIA	Lung Adenocarcinom		2010	-25839	Dead	WITH TUMOR		488				214	Progressive Disease		1	488	1	488			1	214
TCGA-49	TCGA-49	LUAD	81	FEMALE	WHITE	Stage IIIA	Lung Adenocarcinom		2010	-29807	Alive	TUMOR F	1621						Complete Remission/Response		0	1621	0	1621	0	1621	0	1621
TCGA-49	TCGA-49	LUAD	81	FEMALE	WHITE	Stage IIIA	Lung Adenocarcinom		2010	-29807	Alive	TUMOR F	1621						Complete Remission/Response		0	1621	0	1621	0	1621	0	1621
TCGA-49	TCGA-49	LUAD	64	FEMALE	WHITE	Stage IIA	Lung Adenocarcinom		2010	-23484	Alive	TUMOR F	1683						Complete Remission/Response		0	1683	0	1683	0	1683	0	1683
TCGA-49	TCGA-49	LUAD	64	FEMALE	WHITE	Stage IIA	Lung Adenocarcinom		2010	-23484	Alive	TUMOR F	1683						Complete Remission/Response		0	1683	0	1683	0	1683	0	1683
TCGA-49	TCGA-49	LUAD	82	MALE	WHITE	Stage IIIA	Lung Adenocarcinom		2011	-30133	Alive	TUMOR F	522								0	522	0	522			0	522
TCGA-49	TCGA-49	LUAD	82	MALE	WHITE	Stage IIIA	Lung Adenocarcinom		2011	-30133	Alive	TUMOR F	522								0	522	0	522			0	522
TCGA-49	TCGA-49	LUAD	68	FEMALE	WHITE	Stage IIIA	Lung Adenocarcinom		2009	-24849	Alive	TUMOR F	354						Complete Remission/Response		0	354	0	354	0	354	0	354
TCGA-49	TCGA-49	LUAD	68	FEMALE	WHITE	Stage IIIA	Lung Adenocarcinom		2009	-24849	Alive	TUMOR F	354						Complete Remission/Response		0	354	0	354	0	354	0	354
TCGA-49	TCGA-49	LUAD	46	FEMALE	WHITE	Stage IIB	Lung Adenocarcinom		2010	-17108	Alive	TUMOR F	677						[Unknown]		0	677	0	677			0	677
TCGA-49	TCGA-49	LUAD	63	FEMALE	BLACK OR	Stage II	Lung Adenocarcinom		2012	-23370	Dead	TUMOR FREE		677	Distant Metastasis			509	Complete Remission/Response		1	677	0	677	1	509	1	509
TCGA-49	TCGA-49	LUAD	57	MALE	BLACK OR	Stage IA	Lung Adenocarcinom		2002	-20932	Alive	TUMOR F	4765						Complete Remission/Response		0	4765	0	4765	0	4765	0	4765
TCGA-49	TCGA-49	LUAD	64	MALE	BLACK OR	Stage IB	Lung Adenocarcinom		2009	-23479	Alive	TUMOR F	2224						Complete Remission/Response		0	2224	0	2224	0	2224	0	2224
TCGA-49	TCGA-49	LUAD	69	MALE	BLACK OR	Stage IIB	Lung Adenocarcinom		2008	-25469	Alive	[Discrepan	1893		Locoregional Recurrence			1893	Complete Remission/Response		0	1893	0	1893	1	1893	1	1893
TCGA-49	TCGA-49	LUAD	51	MALE	BLACK OR	Stage IIIA	Lung Adenocarcinom		2006	-18939	Dead	WITH TUMOR		879					Stable Disease		1	879	1	879			1	879
TCGA-49	TCGA-49	LUAD	61	MALE	BLACK OR	Stage IIB	Lung Adenocarcinom		1999	-22510	Dead	WITH TUMOR		260					Progressive Disease		1	260	1	260			1	260
TCGA-49	TCGA-49	LUAD	51	FEMALE	BLACK OR	Stage IA	Lung Adenocarcinom		1999	-18893	Dead	WITH TUMOR		1229	Locoregional Recurrence			388	Complete Remission/Response		1	1229	1	1229	1	388	1	388
TCGA-49	TCGA-49	LUAD	56	FEMALE	BLACK OR	Stage IA	Lung Adenocarcinom		2000	-20605	Dead			1135					Complete Remission/Response		1	1135		1135	0	1135	0	1135
TCGA-49	TCGA-49	LUAD	39	FEMALE	BLACK OR	Stage IA	Lung Adenocarcinom		2003	-14527	Alive	WITH TUM	3759		Locoregional Recurrence			1144	Complete Remission/Response		0	3759	0	3759	1	1144	1	1144
TCGA-49	TCGA-49	LUAD	41	FEMALE	BLACK OR	Stage I	Lung Adenocarcinom		1996	-15065	Alive	TUMOR F	6732						Complete Remission/Response		0	6732	0	6732	0	6732	0	6732
TCGA-49	TCGA-49	LUAD	68	MALE	BLACK OR	Stage IA	Lung Adenocarcinom		2001	-24942	Alive	WITH TUM	4992		New Primary Tumor			1099	Complete Remission/Response		0	4992	0	4992	1	4812	1	1099
TCGA-4B	TCGA-4B	LUAD	52	FEMALE	BLACK OR	Stage IA	Lung Adenocarcinom		2013	-19035	Dead	WITH TUMOR		300	Distant Metastasis			251	Progressive Disease		1	300	1	300			1	251
TCGA-50	TCGA-50	LUAD	72	FEMALE	WHITE	Stage IIIB	Lung Adenocarcinom		2002	-26567	Dead	WITH TUMOR		624	Distant Metastasis			483	Progressive Disease		1	624	1	624			1	483
TCGA-50	TCGA-50	LUAD	57	FEMALE	BLACK OR	[Discrepancy]	Lung Adenocarcinom		2004	-20961	Dead	WITH TUMOR		2174	Locoregional Recurrence			1433	Progressive Disease		1	2174	1	2174			1	1433
TCGA-50	TCGA-50	LUAD	70	MALE	WHITE	Stage IA	Lung Adenocarcinom		2006	-25577	Alive	WITH TUM	3094		Locoregional Recurrence			1568	Progressive Disease		0	3094	0	3094			1	1568
TCGA-50	TCGA-50	LUAD	42	FEMALE	WHITE	Stage IIIA	Lung Adenocarcinom		2007	-15585	Dead	WITH TUMOR		478	Distant Metastasis			184	Progressive Disease		1	478	1	478			1	184
TCGA-50	TCGA-50	LUAD	79	FEMALE	WHITE	Stage IIA	Lung Adenocarcinom		2008	-28988	Dead	WITH TUMOR		1830	Locoregional Recurrence|Distan			771	Stable Disease		1	1830	1	1830			1	771
TCGA-50	TCGA-50	LUAD	72	MALE	BLACK OR	Stage IB	Lung Adenocarcinom		2009	-26385	Alive	TUMOR F	1442		Distant Metastasis			477	Complete Remission/Response		0	1442	0	1442	1	477	1	477
TCGA-50	TCGA-50	LUAD	72	MALE	BLACK OR	Stage IB	Lung Adenocarcinom		2009	-26385	Alive	TUMOR F	1442		Distant Metastasis			477	Complete Remission/Response		0	1442	0	1442	1	477	1	477
TCGA-50	TCGA-50	LUAD	59	FEMALE	WHITE	Stage IIB	Lung Adenocarcinom		2003	-21728	Dead	WITH TUM	1499	1499	Distant Metastasis			686	Progressive Disease		1	1499	1	1499			1	686
TCGA-50	TCGA-50	LUAD	74	MALE	WHITE	Stage IIIA	Lung Adenocarcinom		2009	-27036	Dead	WITH TUMOR		250	Distant Metastasis			213	Progressive Disease		1	250	1	250			1	213
TCGA-50	TCGA-50	LUAD	47	MALE	WHITE	Stage IIIA	Lung Adenocarcinom		2001	-17238	Dead	WITH TUMOR		282	Distant Metastasis			177	Progressive Disease		1	282	1	282			1	177
TCGA-50	TCGA-50	LUAD	47	MALE	WHITE	Stage IIIA	Lung Adenocarcinom		2001	-17238	Dead	WITH TUMOR		282	Distant Metastasis			177	Progressive Disease		1	282	1	282			1	177
TCGA-50	TCGA-50	LUAD	75	FEMALE	WHITE	Stage IB	Lung Adenocarcinom		2001	-27733	Dead	WITH TUMOR		434	Distant Metastasis			425	Progressive Disease		1	434	1	434			1	425
TCGA-50	TCGA-50	LUAD	75	FEMALE	WHITE	Stage IB	Lung Adenocarcinom		2001	-27733	Dead	WITH TUMOR		434	Distant Metastasis			425	Progressive Disease		1	434	1	434			1	425
TCGA-50	TCGA-50	LUAD	75	MALE	WHITE	Stage IIB	Lung Adenocarcinom		2001	-27454	Dead	WITH TUM	1091	1235	Locoregional Recurrence			1090	Progressive Disease		1	1235	1	1235			1	1090
TCGA-50	TCGA-50	LUAD	75	MALE	WHITE	Stage IIB	Lung Adenocarcinom		2001	-27454	Dead	WITH TUM	1091	1235	Locoregional Recurrence			1090	Progressive Disease		1	1235	1	1235			1	1090
TCGA-50	TCGA-50	LUAD	72	MALE	WHITE	Stage IIIB	Lung Adenocarcinom		2001	-26377	Dead	TUMOR F	2370	2393					Complete Remission/Response		1	2393	0	2393	0	2393	0	2393
TCGA-50	TCGA-50	LUAD	72	MALE	WHITE	Stage IIIB	Lung Adenocarcinom		2001	-26377	Dead	TUMOR F	2370	2393					Complete Remission/Response		1	2393	0	2393	0	2393	0	2393
TCGA-50	TCGA-50	LUAD	86	FEMALE	WHITE	Stage IA	Lung Adenocarcinom		2006	-31610	Dead	TUMOR F	653	653					Complete Remission/Response		1	653	0	653	0	653	0	653
TCGA-50	TCGA-50	LUAD	86	FEMALE	WHITE	Stage IA	Lung Adenocarcinom		2006	-31610	Dead	TUMOR F	653	653					Complete Remission/Response		1	653	0	653	0	653	0	653
TCGA-50	TCGA-50	LUAD	58	MALE	WHITE	Stage IIIA	Lung Adenocarcinom		2007	-21238	Dead	WITH TUMOR		257				150	Progressive Disease		1	257	1	257			1	150
TCGA-50	TCGA-50	LUAD	58	MALE	WHITE	Stage IIIA	Lung Adenocarcinom		2007	-21238	Dead	WITH TUMOR		257				150	Progressive Disease		1	257	1	257			1	150
TCGA-50	TCGA-50	LUAD	85	MALE	WHITE	Stage IB	Lung Adenocarcinom		2008	-31236	Dead	TUMOR FREE		460					Complete Remission/Response		1	460	0	460	0	460	0	460
TCGA-50	TCGA-50	LUAD	85	MALE	WHITE	Stage IB	Lung Adenocarcinom		2008	-31236	Dead	TUMOR FREE		460					Complete Remission/Response		1	460	0	460	0	460	0	460
TCGA-50	TCGA-50	LUAD	55	FEMALE	WHITE	Stage IIIA	Lung Adenocarcinom		2010	-20350	Alive	TUMOR F	1474						Complete Remission/Response		0	1474	0	1474	0	1474	0	1474
TCGA-50	TCGA-50	LUAD	67	FEMALE	WHITE	Stage IA	Lung Adenocarcinom		2009	-24781	Alive	WITH TUM	1847		Locoregional Recurrence			1378	Stable Disease		0	1847	0	1847			1	1378
TCGA-50	TCGA-50	LUAD	69	FEMALE	WHITE	Stage IA	Lung Adenocarcinom		2010	-25440	Alive	TUMOR F	1750						Complete Remission/Response		0	1750	0	1750	0	1750	0	1750
TCGA-50	TCGA-50	LUAD	62	MALE	WHITE	Stage IA	Lung Adenocarcinom		2010	-22852	Alive	WITH TUM	1617		Distant Metastasis			221	Progressive Disease		0	1617	0	1617			1	221
TCGA-50	TCGA-50	LUAD	62	MALE	WHITE	Stage IA	Lung Adenocarcinom		2010	-22852	Alive	WITH TUM	1617		Distant Metastasis			221	Progressive Disease		0	1617	0	1617			1	221
TCGA-50	TCGA-50	LUAD	72	FEMALE	WHITE	Stage IB	Lung Adenocarcinom		2005	-26444	Dead	TUMOR FREE		1288					Complete Remission/Response		1	1288	0	1288	0	1288	0	1288
TCGA-50	TCGA-50	LUAD	63	FEMALE	WHITE	Stage IV	Lung Adenocarcinom		2007	-23067	Dead	WITH TUMOR		119					Progressive Disease		1	119	1	119			1	119
TCGA-50	TCGA-50	LUAD	63	FEMALE	WHITE	Stage IV	Lung Adenocarcinom		2007	-23067	Dead	WITH TUMOR		119					Progressive Disease		1	119	1	119			1	119
TCGA-50	TCGA-50	LUAD	71	FEMALE	WHITE	Stage IB	Lung Adenocarcinom		2007	-26191	Dead	WITH TUMOR		777					Progressive Disease		1	777	1	777			1	777
TCGA-50	TCGA-50	LUAD	71	FEMALE	WHITE	Stage IB	Lung Adenocarcinom		2007	-26191	Dead	WITH TUMOR		777					Progressive Disease		1	777	1	777			1	777
TCGA-50	TCGA-50	LUAD	49	FEMALE	WHITE	Stage IIIA	Lung Adenocarcinom		2008	-18152	Dead	WITH TUMOR		336	Distant Metastasis			266	Progressive Disease		1	336	1	336			1	266
TCGA-50	TCGA-50	LUAD	49	FEMALE	WHITE	Stage IIIA	Lung Adenocarcinom		2008	-18152	Dead	WITH TUMOR		336	Distant Metastasis			266	Progressive Disease		1	336	1	336			1	266
TCGA-50	TCGA-50	LUAD	79	FEMALE	BLACK OR	Stage IIIA	Lung Adenocarcinom		2009	-28924	Dead	WITH TUMOR		370	Distant Metastasis			285	Progressive Disease		1	370	1	370			1	285
TCGA-50	TCGA-50	LUAD	79	FEMALE	BLACK OR	Stage IIIA	Lung Adenocarcinom		2009	-28924	Dead	WITH TUMOR		370	Distant Metastasis			285	Progressive Disease		1	370	1	370			1	285
TCGA-50	TCGA-50	LUAD	74	FEMALE	WHITE	Stage IIIA	Lung Adenocarcinom		2009	-27197	Dead	WITH TUMOR		189	Distant Metastasis			182	Progressive Disease		1	189	1	189			1	182
TCGA-50	TCGA-50	LUAD	74	FEMALE	WHITE	Stage IIIA	Lung Adenocarcinom		2009	-27197	Dead	WITH TUMOR		189	Distant Metastasis			182	Progressive Disease		1	189	1	189			1	182
TCGA-50	TCGA-50	LUAD	79	FEMALE	WHITE	Stage IB	Lung Adenocarcinom		2007	-29195	Dead	TUMOR FREE		1268					Partial Remission/Response		1	1268	0	1268			0	1268
TCGA-50	TCGA-50	LUAD	84	FEMALE	WHITE	Stage I	Lung Adenocarcinom		2004	-30689	Dead			22					[Unknown]		1	22		22			0	22
TCGA-50	TCGA-50	LUAD	60	MALE	WHITE	Stage IA	Lung Adenocarcinom		2011	-21979	Dead	WITH TUMOR		308	Distant Metastasis			15	Progressive Disease		1	308	1	308			1	15
TCGA-50	TCGA-50	LUAD	60	MALE	WHITE	Stage IA	Lung Adenocarcinom		2011	-21979	Dead	WITH TUMOR		308	Distant Metastasis			15	Progressive Disease		1	308	1	308			1	15
TCGA-50	TCGA-50	LUAD	63	FEMALE	BLACK OR	Stage IA	Lung Adenocarcinom		2011	-23055	Alive	TUMOR F	1125						Complete Remission/Response		0	1125	0	1125	0	1125	0	1125
TCGA-50	TCGA-50	LUAD	68	MALE	WHITE	Stage IIB	Lung Adenocarcinom		2012	-24859	Alive	WITH TUM	1119		Locoregional Recurrence			432	Progressive Disease		0	1119	0	1119			1	432
TCGA-50	TCGA-50	LUAD	74	MALE	WHITE	Stage IA	Lung Adenocarcinom		2012	-27270	Alive	TUMOR F	829						Complete Remission/Response		0	829	0	829	0	829	0	829
TCGA-53	TCGA-53	LUAD	40	FEMALE	WHITE	Stage IV	Lung Adenocarcinom		2008	-14794	Dead	WITH TUMOR		1043	Distant Metastasis			400	Progressive Disease		1	1043	1	1043			1	400
TCGA-53	TCGA-53	LUAD	76	FEMALE	WHITE	Stage IIA	Lung Adenocarcinom		2008	-28024	Dead	WITH TUMOR		929	Locoregional Recurrence			865	Progressive Disease		1	929	1	929			1	865
TCGA-53	TCGA-53	LUAD	51	FEMALE	WHITE	Stage IIIB	Lung Adenocarcinom		2008	-18875	Alive	TUMOR F	424						Progressive Disease		0	424	0	424			0	424
TCGA-53	TCGA-53	LUAD	63	MALE	WHITE	Stage IIA	Lung Adenocarcinom		2012	-23201	Alive	TUMOR F	1071						Stable Disease		0	1071	0	1071			0	1071
TCGA-55	TCGA-55	LUAD	65	MALE	WHITE	Stage IA	Lung Adenocarcinom		2005		Dead	WITH TUMOR		701				452	Complete Remission/Response		1	701	1	701	1	452	1	452
TCGA-55	TCGA-55	LUAD	65	MALE	WHITE	Stage IA	Lung Adenocarcinom		2005		Dead	WITH TUMOR		701				452	Complete Remission/Response		1	701	1	701	1	452	1	452
TCGA-55	TCGA-55	LUAD	68	MALE	WHITE	Stage IIIA	Lung Adenocarcinom		2005	-25008	Alive	TUMOR F	1178								0	1178	0	1178			0	1178
TCGA-55	TCGA-55	LUAD	68	MALE	WHITE	Stage IIIA	Lung Adenocarcinom		2005	-25008	Alive	TUMOR F	1178								0	1178	0	1178			0	1178
TCGA-55	TCGA-55	LUAD	74	FEMALE	ASIAN	Stage IA	Lung Adenocarcinom		2004		Alive	TUMOR F	1479						Complete Remission/Response		0	1479	0	1479	0	1479	0	1479
TCGA-55	TCGA-55	LUAD	74	FEMALE	ASIAN	Stage IA	Lung Adenocarcinom		2004		Alive	TUMOR F	1479						Complete Remission/Response		0	1479	0	1479	0	1479	0	1479
TCGA-55	TCGA-55	LUAD	55	MALE	ASIAN	Stage IIB	Lung Adenocarcinom		2004	-20381	Alive	TUMOR F	2065						Complete Remission/Response		0	2065	0	2065	0	2065	0	2065
TCGA-55	TCGA-55	LUAD	55	MALE	ASIAN	Stage IIB	Lung Adenocarcinom		2004	-20381	Alive	TUMOR F	2065						Complete Remission/Response		0	2065	0	2065	0	2065	0	2065
TCGA-55	TCGA-55	LUAD	58	MALE	WHITE	[Discrepancy]	Lung Adenocarcinom		2010		Alive	TUMOR F	930						Complete Remission/Response		0	930	0	930	0	930	0	930
TCGA-55	TCGA-55	LUAD	60	FEMALE	WHITE	Stage IA	Lung Adenocarcinom		2010		Alive	TUMOR F	435						Complete Remission/Response		0	435	0	435	0	435	0	435
TCGA-55	TCGA-55	LUAD	63	MALE	WHITE	Stage IB	Lung Adenocarcinom		2006	-23107	Alive	TUMOR F	2449						Complete Remission/Response		0	2449	0	2449	0	2449	0	2449
TCGA-55	TCGA-55	LUAD	71	MALE	WHITE	Stage IIA	Lung Adenocarcinom		2010		Dead	WITH TUMOR		171					Progressive Disease		1	171	1	171			1	171
TCGA-55	TCGA-55	LUAD	61	MALE	WHITE	Stage IV	Lung Adenocarcinom		2004	-22344	Dead	WITH TUMOR		1293					Stable Disease		1	1293	1	1293			1	1293
TCGA-55	TCGA-55	LUAD	61	MALE	WHITE	Stage IV	Lung Adenocarcinom		2004	-22344	Dead	WITH TUMOR		1293					Stable Disease		1	1293	1	1293			1	1293
TCGA-55	TCGA-55	LUAD	52	MALE	WHITE	Stage IB	Lung Adenocarcinom		2004	-19270	Alive	TUMOR F	1239								0	1239	0	1239			0	1239
TCGA-55	TCGA-55	LUAD	52	MALE	WHITE	Stage IB	Lung Adenocarcinom		2004	-19270	Alive	TUMOR F	1239								0	1239	0	1239			0	1239
TCGA-55	TCGA-55	LUAD	67	FEMALE	WHITE	Stage IIIA	Lung Adenocarcinom		2004	-24773	Dead	WITH TUMOR		464	Locoregional Recurrence			457	Stable Disease		1	464	1	464			1	457
TCGA-55	TCGA-55	LUAD	67	FEMALE	WHITE	Stage IIIA	Lung Adenocarcinom		2004	-24773	Dead	WITH TUMOR		464	Locoregional Recurrence			457	Stable Disease		1	464	1	464			1	457
TCGA-55	TCGA-55	LUAD	59	FEMALE	WHITE	Stage IB	Lung Adenocarcinom		2010	-21734	Alive	TUMOR F	1400						Complete Remission/Response		0	1400	0	1400	0	1400	0	1400
TCGA-55	TCGA-55	LUAD	59	FEMALE	WHITE	Stage IB	Lung Adenocarcinom		2010	-21734	Alive	TUMOR F	1400						Complete Remission/Response		0	1400	0	1400	0	1400	0	1400
TCGA-55	TCGA-55	LUAD	72	MALE	WHITE	Stage IB	Lung Adenocarcinom		2005	-26625	Dead	TUMOR FREE		1632					Complete Remission/Response		1	1632	0	1632	0	1632	0	1632
TCGA-55	TCGA-55	LUAD	72	MALE	WHITE	Stage IB	Lung Adenocarcinom		2005	-26625	Dead	TUMOR FREE		1632					Complete Remission/Response		1	1632	0	1632	0	1632	0	1632
TCGA-55	TCGA-55	LUAD	61	MALE	WHITE	Stage IIB	Lung Adenocarcinom		2004		Dead	WITH TUMOR		118					Progressive Disease		1	118	1	118			1	118
TCGA-55	TCGA-55	LUAD	61	MALE	WHITE	Stage IIB	Lung Adenocarcinom		2004		Dead	WITH TUMOR		118					Progressive Disease		1	118	1	118			1	118
TCGA-55	TCGA-55	LUAD	81	MALE	WHITE	Stage IIA	Lung Adenocarcinom		2010		Dead	WITH TUMOR		176				42	Progressive Disease		1	176	1	176			1	42
TCGA-55	TCGA-55	LUAD	81	MALE	WHITE	Stage IIA	Lung Adenocarcinom		2010		Dead	WITH TUMOR		176				42	Progressive Disease		1	176	1	176			1	42
TCGA-55	TCGA-55	LUAD	59	FEMALE	WHITE	Stage IIB	Lung Adenocarcinom		2006	-21780	Dead	WITH TUMOR		237	Distant Metastasis			195	Progressive Disease		1	237	1	237			1	195
TCGA-55	TCGA-55	LUAD	59	FEMALE	WHITE	Stage IIB	Lung Adenocarcinom		2006	-21780	Dead	WITH TUMOR		237	Distant Metastasis			195	Progressive Disease		1	237	1	237			1	195
TCGA-55	TCGA-55	LUAD	56	MALE	WHITE	Stage IA	Lung Adenocarcinom		2006		Alive	TUMOR F	2109						Complete Remission/Response		0	2109	0	2109	0	2109	0	2109
TCGA-55	TCGA-55	LUAD	56	MALE	WHITE	Stage IA	Lung Adenocarcinom		2006		Alive	TUMOR F	2109						Complete Remission/Response		0	2109	0	2109	0	2109	0	2109
TCGA-55	TCGA-55	LUAD	53	FEMALE	WHITE	Stage IIIA	Lung Adenocarcinom		2005	-19497	Dead	TUMOR FREE		1379					Complete Remission/Response		1	1379	0	1379	0	1379	0	1379
TCGA-55	TCGA-55	LUAD	53	FEMALE	WHITE	Stage IIIA	Lung Adenocarcinom		2005	-19497	Dead	TUMOR FREE		1379					Complete Remission/Response		1	1379	0	1379	0	1379	0	1379
TCGA-55	TCGA-55	LUAD	79	FEMALE	WHITE	Stage IIB	Lung Adenocarcinom		2004		Dead	WITH TUMOR		995	Distant Metastasis			183	Progressive Disease		1	995	1	995			1	183
TCGA-55	TCGA-55	LUAD	79	FEMALE	WHITE	Stage IIB	Lung Adenocarcinom		2004		Dead	WITH TUMOR		995	Distant Metastasis			183	Progressive Disease		1	995	1	995			1	183
TCGA-55	TCGA-55	LUAD	81	MALE	WHITE	Stage IIB	Lung Adenocarcinom		2005		Alive	TUMOR F	2823						Complete Remission/Response		0	2823	0	2823	0	2823	0	2823
TCGA-55	TCGA-55	LUAD	81	MALE	WHITE	Stage IIB	Lung Adenocarcinom		2005		Alive	TUMOR F	2823						Complete Remission/Response		0	2823	0	2823	0	2823	0	2823
TCGA-55	TCGA-55	LUAD	71	FEMALE	WHITE	Stage IIB	Lung Adenocarcinom		2006		Dead	WITH TUMOR		760				724	Complete Remission/Response		1	760	1	760	1	724	1	724
TCGA-55	TCGA-55	LUAD	71	FEMALE	WHITE	Stage IIB	Lung Adenocarcinom		2006		Dead	WITH TUMOR		760				724	Complete Remission/Response		1	760	1	760	1	724	1	724
TCGA-55	TCGA-55	LUAD	58	FEMALE	WHITE	Stage IB	Lung Adenocarcinom		2004	-21381	Alive	TUMOR F	1233						Complete Remission/Response		0	1233	0	1233	0	1233	0	1233
TCGA-55	TCGA-55	LUAD	58	FEMALE	WHITE	Stage IB	Lung Adenocarcinom		2004	-21381	Alive	TUMOR F	1233						Complete Remission/Response		0	1233	0	1233	0	1233	0	1233
TCGA-55	TCGA-55	LUAD	74	FEMALE	WHITE	Stage IB	Lung Adenocarcinom		2004		Alive	TUMOR F	3261						Complete Remission/Response		0	3261	0	3261	0	3261	0	3261
TCGA-55	TCGA-55	LUAD	74	FEMALE	WHITE	Stage IB	Lung Adenocarcinom		2004		Alive	TUMOR F	3261						Complete Remission/Response		0	3261	0	3261	0	3261	0	3261
TCGA-55	TCGA-55	LUAD	77	MALE	WHITE	Stage IA	Lung Adenocarcinom		2006	-28382	Alive	TUMOR F	2137						Complete Remission/Response		0	2137	0	2137	0	2137	0	2137
TCGA-55	TCGA-55	LUAD	77	MALE	WHITE	Stage IIIA	Lung Adenocarcinom		2011	-28458	Dead	WITH TUMOR		952	Locoregional Recurrence			255	Complete Remission/Response		1	952	1	952	1	255	1	255
TCGA-55	TCGA-55	LUAD	70	FEMALE	WHITE	Stage IA	Lung Adenocarcinom		2011	-25870	Alive	WITH TUM	872		Distant Metastasis			339	Complete Remission/Response		0	872	0	872	1	339	1	339
TCGA-55	TCGA-55	LUAD	76	FEMALE	WHITE	Stage IIIA	Lung Adenocarcinom		2011	-27946	Alive	WITH TUM	609						Partial Remission/Response		0	609	0	609			0	609
TCGA-55	TCGA-55	LUAD	76	FEMALE	WHITE	Stage IIIA	Lung Adenocarcinom		2011	-27946	Alive	WITH TUM	609						Partial Remission/Response		0	609	0	609			0	609

TCGA-55-	TCGA-55-	LUAD	74	MALE	WHITE	Stage IIB	Lung Adenocarcinon	2011	-27087	Dead	WITH TUMOR		243	Distant Metastasis	231	Complete Remission/Response	1	243	1	243	1	231	1	231
TCGA-55-	TCGA-55-	LUAD	60	MALE	BLACK OI	Stage IA	Lung Adenocarcinon	2011	-22218	Alive	TUMOR F	824				Complete Remission/Response	0	824	0	824	0	824	0	824
TCGA-55-	TCGA-55-	LUAD	60	MALE	BLACK OI	Stage IA	Lung Adenocarcinon	2011	-22218	Alive	TUMOR F	824				Complete Remission/Response	0	824	0	824	0	824	0	824
TCGA-55-	TCGA-55-	LUAD	72	FEMALE	WHITE	Stage IA	Lung Adenocarcinon	2011	-26302	Alive	TUMOR F	487				Complete Remission/Response	0	487	0	487	0	487	0	487
TCGA-55-	TCGA-55-	LUAD	64	FEMALE	WHITE	Stage IB	Lung Adenocarcinon	2011	-23703	Dead	WITH TUMOR		995	Distant Metastasis	478	Complete Remission/Response	1	995	1	995	1	478	1	478
TCGA-55-	TCGA-55-	LUAD	64	FEMALE	WHITE	Stage IB	Lung Adenocarcinon	2011	-23703	Dead	WITH TUMOR		995	Distant Metastasis	478	Complete Remission/Response	1	995	1	995	1	478	1	478
TCGA-55-	TCGA-55-	LUAD	54	MALE	BLACK OI	Stage IB	Lung Adenocarcinon	2011	-19917	Alive	TUMOR F	670				Complete Remission/Response	0	670	0	670	0	670	0	670
TCGA-55-	TCGA-55-	LUAD	54	MALE	BLACK OI	Stage IB	Lung Adenocarcinon	2011	-19917	Alive	TUMOR F	670				Complete Remission/Response	0	670	0	670	0	670	0	670
TCGA-55-	TCGA-55-	LUAD	76	FEMALE	WHITE	Stage IB	Lung Adenocarcinon	2011	-27891	Alive	TUMOR F	705				Complete Remission/Response	0	705	0	705	0	705	0	705
TCGA-55-	TCGA-55-	LUAD	76	FEMALE	WHITE	Stage IB	Lung Adenocarcinon	2011	-27891	Alive	TUMOR F	705				Complete Remission/Response	0	705	0	705	0	705	0	705
TCGA-55-	TCGA-55-	LUAD	68	FEMALE	WHITE	Stage IA	Lung Adenocarcinon	2011	-24848	Alive	TUMOR F	442				Complete Remission/Response	0	442	0	442	0	442	0	442
TCGA-55-	TCGA-55-	LUAD	68	FEMALE	WHITE	Stage IA	Lung Adenocarcinon	2011	-24848	Alive	TUMOR F	442				Complete Remission/Response	0	442	0	442	0	442	0	442
TCGA-55-	TCGA-55-	LUAD	72	FEMALE	WHITE	Stage IA	Lung Adenocarcinon	2011	-26451	Alive	TUMOR F	652				Complete Remission/Response	0	652	0	652	0	652	0	652
TCGA-55-	TCGA-55-	LUAD	72	FEMALE	WHITE	Stage IA	Lung Adenocarcinon	2011	-26451	Alive	TUMOR F	652				Complete Remission/Response	0	652	0	652	0	652	0	652
TCGA-55-	TCGA-55-	LUAD	70	MALE	WHITE	Stage IIIA	Lung Adenocarcinon	2011	-25929	Alive	TUMOR F	119				Complete Remission/Response	0	119	0	119	0	119	0	119
TCGA-55-	TCGA-55-	LUAD	64	FEMALE	WHITE	Stage IB	Lung Adenocarcinon	2011	-23505	Alive	TUMOR F	704				Complete Remission/Response	0	704	0	704	0	704	0	704
TCGA-55-	TCGA-55-	LUAD	76	MALE		Stage IB	Lung Adenocarcinon	2011	-28119	Alive	WITH TUN	773		Locoregional Recurrence	466	Complete Remission/Response	0	773	0	773	1	466	1	466
TCGA-55-	TCGA-55-	LUAD	49	FEMALE	WHITE	Stage IV	Lung Adenocarcinon	2011	-18170	Dead	WITH TUMOR		468			Progressive Disease	1	468	1	468			1	468
TCGA-55-	TCGA-55-	LUAD	64	MALE	WHITE	Stage IA	Lung Adenocarcinon	2011	-23509	Alive	TUMOR F	567				Complete Remission/Response	0	567	0	567	0	567	0	567
TCGA-55-	TCGA-55-	LUAD	64	MALE	WHITE	Stage IA	Lung Adenocarcinon	2011	-23509	Alive	TUMOR F	567				Complete Remission/Response	0	567	0	567	0	567	0	567
TCGA-55-	TCGA-55-	LUAD	77	MALE	WHITE	Stage IIA	Lung Adenocarcinon	2011	-28201	Dead	WITH TUMOR		343	Distant Metastasis	294	Complete Remission/Response	1	343	1	343	1	294	1	294
TCGA-55-	TCGA-55-	LUAD	50	FEMALE	BLACK OI	Stage IIA	Lung Adenocarcinon	2011	-18289	Alive	WITH TUN	1040			1018	Complete Remission/Response	0	1040	0	1040	1	1018	1	1018
TCGA-55-	TCGA-55-	LUAD	50	FEMALE	BLACK OI	Stage IIA	Lung Adenocarcinon	2011	-18289	Alive	WITH TUN	1040			1018	Complete Remission/Response	0	1040	0	1040	1	1018	1	1018
TCGA-55-	TCGA-55-	LUAD	70	FEMALE	WHITE	Stage IA	Lung Adenocarcinon	2011	-25746	Alive	TUMOR F	537		New Primary Tumor	515	Complete Remission/Response	0	537	0	537	0	537	1	515
TCGA-55-	TCGA-55-	LUAD	70	FEMALE	WHITE	Stage IA	Lung Adenocarcinon	2011	-25746	Alive	TUMOR F	537		New Primary Tumor	515	Complete Remission/Response	0	537	0	537	0	537	1	515
TCGA-55-	TCGA-55-	LUAD	61	FEMALE	BLACK OI	Stage IA	Lung Adenocarcinon	2011	-22326	Dead	WITH TUMOR		561	Distant Metastasis	480	Complete Remission/Response	1	561	1	561	1	480	1	480
TCGA-55-	TCGA-55-	LUAD	71	FEMALE	WHITE	Stage IIA	Lung Adenocarcinon	2011	-26105	Dead	WITH TUMOR		187			Progressive Disease	1	187	1	187			1	187
TCGA-55-	TCGA-55-	LUAD	71	FEMALE	WHITE	Stage IIA	Lung Adenocarcinon	2011	-26105	Dead	WITH TUMOR		187			Progressive Disease	1	187	1	187			1	187
TCGA-55-	TCGA-55-	LUAD	81	MALE	WHITE	Stage IIB	Lung Adenocarcinon	2011	-29858	Alive	TUMOR F	603				Complete Remission/Response	0	603	0	603	0	603	0	603
TCGA-55-	TCGA-55-	LUAD	73	FEMALE	WHITE	Stage IA	Lung Adenocarcinon	2012	-26837	Alive	TUMOR F	889		Locoregional Recurrence	468	Complete Remission/Response	0	889	0	889	1	468	1	468
TCGA-55-	TCGA-55-	LUAD	64	MALE	WHITE	Stage IA	Lung Adenocarcinon	2011	-23596	Alive	TUMOR F	904				Complete Remission/Response	0	904	0	904	0	904	0	904
TCGA-55-	TCGA-55-	LUAD	59	FEMALE	WHITE	Stage IB	Lung Adenocarcinon	2011	-21655	Alive	TUMOR F	462				Complete Remission/Response	0	462	0	462	0	462	0	462
TCGA-55-	TCGA-55-	LUAD	56	MALE	WHITE	Stage IA	Lung Adenocarcinon	2011	-20576	Dead	TUMOR FREE		702			Complete Remission/Response	1	702	0	702	0	702	0	702
TCGA-55-	TCGA-55-	LUAD	80	MALE	WHITE	Stage IA	Lung Adenocarcinon	2011	-29235	Dead	WITH TUMOR		598	Distant Metastasis	548	Complete Remission/Response	1	598	1	598	1	548	1	548
TCGA-55-	TCGA-55-	LUAD	74	MALE	WHITE	Stage IB	Lung Adenocarcinon	2011	-27385	Alive	TUMOR F	600				Complete Remission/Response	0	600	0	600	0	600	0	600
TCGA-55-	TCGA-55-	LUAD	75	MALE	WHITE	Stage IIB	Lung Adenocarcinon	2012	-27597	Dead	WITH TUMOR		154	Locoregional Recurrence	127	Complete Remission/Response	1	154	1	154	1	127	1	127
TCGA-55-	TCGA-55-	LUAD	51	MALE	WHITE	Stage IV	Lung Adenocarcinon	2012	-18903	Alive	WITH TUN	541				Progressive Disease	0	541	0	541			0	541
TCGA-55-	TCGA-55-	LUAD	67	FEMALE	WHITE	Stage IB	Lung Adenocarcinon	2011	-24684	Dead	WITH TUMOR		719	Distant Metastasis	566	Complete Remission/Response	1	719	1	719	1	566	1	566
TCGA-55-	TCGA-55-	LUAD	60	FEMALE	WHITE	Stage IA	Lung Adenocarcinon	2012	-22260	Alive	TUMOR F	476				Complete Remission/Response	0	476	0	476	0	476	0	476
TCGA-55-	TCGA-55-	LUAD	69	FEMALE	WHITE	Stage IA	Lung Adenocarcinon	2011	-25477	Alive	TUMOR F	547				Complete Remission/Response	0	547	0	547	0	547	0	547
TCGA-55-	TCGA-55-	LUAD	87	FEMALE	WHITE	Stage IB	Lung Adenocarcinon	2011	-31818	Alive	TUMOR F	515				Complete Remission/Response	0	515	0	515	0	515	0	515
TCGA-55-	TCGA-55-	LUAD	76	FEMALE	WHITE	Stage IIA	Lung Adenocarcinon	2012	-28063	Alive	WITH TUN	599		Distant Metastasis	495	Progressive Disease	0	599	0	599			1	495
TCGA-55-	TCGA-55-	LUAD	56	MALE	WHITE	Stage IA	Lung Adenocarcinon	2012	-20690	Alive	TUMOR F	888				Complete Remission/Response	0	888	0	888	0	888	0	888
TCGA-55-	TCGA-55-	LUAD	73	MALE	WHITE	Stage IB	Lung Adenocarcinon	2011	-26897	Alive	TUMOR F	977				Complete Remission/Response	0	977	0	977	0	977	0	977
TCGA-55-	TCGA-55-	LUAD	73	FEMALE	WHITE	Stage IA	Lung Adenocarcinon	2012	-26716	Alive	WITH TUN	674		Distant Metastasis	511	Complete Remission/Response	0	674	0	674	1	511	1	511
TCGA-55-	TCGA-55-	LUAD	61	FEMALE	WHITE	Stage IA	Lung Adenocarcinon	2012	-22626	Dead	WITH TUMOR		469	Distant Metastasis	274	Complete Remission/Response	1	469	1	469	1	274	1	274
TCGA-55-	TCGA-55-	LUAD	58	MALE	WHITE	Stage IB	Lung Adenocarcinon	2012	-21535	Alive	WITH TUN	534		Locoregional Recurrence	238	Complete Remission/Response	0	534	0	534	1	238	1	238
TCGA-55-	TCGA-55-	LUAD	54	MALE	WHITE	Stage IB	Lung Adenocarcinon	2012	-20011	Alive	TUMOR F	478				Complete Remission/Response	0	478	0	478	0	478	0	478
TCGA-55-	TCGA-55-	LUAD	62	MALE	WHITE	Stage IIIA	Lung Adenocarcinon	2012	-22676	Alive	TUMOR F	440				Complete Remission/Response	0	440	0	440	0	440	0	440
TCGA-55-	TCGA-55-	LUAD	62	FEMALE	WHITE	Stage IIB	Lung Adenocarcinon	2012	-22850	Alive		11					0	11	0	11			0	11
TCGA-55-	TCGA-55-	LUAD	53	MALE	WHITE	Stage IA	Lung Adenocarcinon	2012	-19390	Alive	TUMOR F	418				Complete Remission/Response	0	418	0	418	0	418	0	418
TCGA-55-	TCGA-55-	LUAD	60	FEMALE	BLACK OI	Stage IIA	Lung Adenocarcinon	2012	-22159	Alive	TUMOR F	617				Complete Remission/Response	0	617	0	617	0	617	0	617
TCGA-55-	TCGA-55-	LUAD	55	FEMALE	WHITE	Stage IB	Lung Adenocarcinon	2012	-20410	Alive	TUMOR F	539				Complete Remission/Response	0	539	0	539	0	539	0	539
TCGA-55-	TCGA-55-	LUAD	73	FEMALE	WHITE	Stage IB	Lung Adenocarcinon	2012	-26986	Alive	WITH TUN	552			460	Complete Remission/Response	0	552	0	552	1	460	1	460
TCGA-55-	TCGA-55-	LUAD	41	MALE	WHITE	Stage IV	Lung Adenocarcinon	2012	-15266	Dead	WITH TUMOR		607			Progressive Disease	1	607	1	607			1	607
TCGA-55-	TCGA-55-	LUAD	77	FEMALE	WHITE	Stage IIB	Lung Adenocarcinon	2012	-28284	Alive	WITH TUN	791		Distant Metastasis	317	Complete Remission/Response	0	791	0	791	1	317	1	317
TCGA-55-	TCGA-55-	LUAD	70	FEMALE	BLACK OI	Stage IB	Lung Adenocarcinon	2012	-25608	Alive	TUMOR F	520				Complete Remission/Response	0	520	0	520	0	520	0	520
TCGA-55-	TCGA-55-	LUAD	76	MALE	WHITE	Stage IB	Lung Adenocarcinon	2011	-27924	Alive	TUMOR F	536				Complete Remission/Response	0	536	0	536	0	536	0	536
TCGA-55-	TCGA-55-	LUAD	67	MALE	WHITE	Stage IIIA	Lung Adenocarcinon	2012	-24786	Alive	WITH TUN	446		Distant Metastasis	224	Complete Remission/Response	0	446	0	446	1	224	1	224
TCGA-55-	TCGA-55-	LUAD	58	FEMALE	WHITE	Stage IB	Lung Adenocarcinon	2012	-21225	Alive	TUMOR F	48					0	48	0	48			0	48
TCGA-55-	TCGA-55-	LUAD	72	FEMALE	WHITE	Stage IIB	Lung Adenocarcinon	2012	-26616	Alive	TUMOR F	416				Complete Remission/Response	0	416	0	416	0	416	0	416
TCGA-55-	TCGA-55-	LUAD	60	MALE	WHITE	Stage IV	Lung Adenocarcinon	2012	-22091	Dead	WITH TUMOR		375			Progressive Disease	1	375	1	375			1	375
TCGA-55-	TCGA-55-	LUAD	75	FEMALE	WHITE	Stage IA	Lung Adenocarcinon	2012	-27485	Alive	TUMOR F	515				Complete Remission/Response	0	515	0	515	0	515	0	515
TCGA-55-	TCGA-55-	LUAD	63	FEMALE	WHITE	Stage IIA	Lung Adenocarcinon	2012	-23341	Alive	TUMOR F	689		New Primary Tumor	615	Complete Remission/Response	0	689	0	689	0	689	1	615
TCGA-55-	TCGA-55-	LUAD	69	MALE	WHITE	Stage IIA	Lung Adenocarcinon	2012	-25384	Alive	TUMOR F	630				Complete Remission/Response	0	630	0	630	0	630	0	630
TCGA-55-	TCGA-55-	LUAD	60	FEMALE	WHITE	Stage IIIB	Lung Adenocarcinon	2012	-22056	Alive	WITH TUN	651		Distant Metastasis	536	Stable Disease	0	651	0	651			1	536
TCGA-55-	TCGA-55-	LUAD	78	MALE	WHITE	Stage IIA	Lung Adenocarcinon	2012	-28728	Dead	TUMOR FREE		99				1	99	0	99			0	99
TCGA-55-	TCGA-55-	LUAD	81	FEMALE	WHITE	Stage IA	Lung Adenocarcinon	2012	-29593	Alive	TUMOR F	626				Complete Remission/Response	0	626	0	626	0	626	0	626
TCGA-55-	TCGA-55-	LUAD	70	FEMALE	WHITE	Stage IA	Lung Adenocarcinon	2012	-25719	Alive	TUMOR F	596				Complete Remission/Response	0	596	0	596	0	596	0	596
TCGA-55-	TCGA-55-	LUAD	54	FEMALE	WHITE	Stage IB	Lung Adenocarcinon	2012	-20027	Alive	TUMOR F	28				[Unknown]	0	28	0	28			0	28
TCGA-55-	TCGA-55-	LUAD	61	FEMALE	WHITE	Stage IB	Lung Adenocarcinon	2012	-22540	Alive	TUMOR F	481				Complete Remission/Response	0	481	0	481	0	481	0	481
TCGA-55-	TCGA-55-	LUAD	88	MALE	WHITE	Stage IA	Lung Adenocarcinon	2012	-32432	Dead	WITH TUMOR		614	Distant Metastasis	515	Complete Remission/Response	1	614	1	614	1	515	1	515
TCGA-55-	TCGA-55-	LUAD	71	MALE	WHITE	Stage IA	Lung Adenocarcinon	2012	-26044	Alive	TUMOR F	608				Complete Remission/Response	0	608	0	608	0	608	0	608
TCGA-55-	TCGA-55-	LUAD	80	FEMALE	BLACK OI	Stage IA	Lung Adenocarcinon	2012	-29452	Alive	TUMOR F	546				Complete Remission/Response	0	546	0	546	0	546	0	546
TCGA-62-	TCGA-62-	LUAD	65	FEMALE	WHITE	Stage IIIB	Lung Adenocarcinon	2008	-23758	Dead	TUMOR FREE		139			Complete Remission/Response	1	139	0	139	0	139	0	139
TCGA-62-	TCGA-62-	LUAD	80	FEMALE	WHITE	Stage IIB	Lung Adenocarcinon	2009	-29311	Alive	WITH TUN	1216		Locoregional Recurrence	395	Complete Remission/Response	0	1216	0	1216	1	395	1	395
TCGA-62-	TCGA-62-	LUAD	70	FEMALE	WHITE	Stage IIB	Lung Adenocarcinon	2008	-25728	Alive	TUMOR F	1289				Complete Remission/Response	0	1289	0	1289	0	1289	0	1289
TCGA-62-	TCGA-62-	LUAD	55	MALE	WHITE	Stage IIIA	Lung Adenocarcinon	2007	-20197	Dead	TUMOR FREE		444			Complete Remission/Response	1	444	0	444	0	444	0	444
TCGA-62-	TCGA-62-	LUAD	62	MALE	WHITE	Stage IIIA	Lung Adenocarcinon	2006	-22952	Alive	TUMOR F	2696				Complete Remission/Response	0	2696	0	2696	0	2696	0	2696
TCGA-62-	TCGA-62-	LUAD	73	FEMALE	WHITE	Stage IIIA	Lung Adenocarcinon	2008	-26934	Dead	WITH TUMOR		1498	Distant Metastasis	772	Complete Remission/Response	1	1498	1	1498	1	772	1	772
TCGA-62-	TCGA-62-	LUAD	65	FEMALE	WHITE	Stage IB	Lung Adenocarcinon	2006	-24050	Dead	WITH TUMOR		1454	Locoregional Recurrence|Distan	943	Complete Remission/Response	1	1454	1	1454	1	943	1	943
TCGA-62-	TCGA-62-	LUAD	65	MALE	WHITE	Stage IB	Lung Adenocarcinon	2006	-23993	Dead	TUMOR FREE		594	Distant Metastasis	267	Complete Remission/Response	1	594	0	594	1	267	1	267
TCGA-62-	TCGA-62-	LUAD	54	FEMALE	WHITE	Stage IB	Lung Adenocarcinon	2006	-20063	Dead	TUMOR FREE		1725			Complete Remission/Response	1	1725	0	1725	0	1725	0	1725
TCGA-62-	TCGA-62-	LUAD	73	MALE	WHITE	Stage IB	Lung Adenocarcinon	2007	-26825	Dead	WITH TUMOR		1653	Locoregional Recurrence|Distan	527	Complete Remission/Response	1	1653	1	1653	1	527	1	527
TCGA-62-	TCGA-62-	LUAD	71	FEMALE	WHITE	Stage IIB	Lung Adenocarcinon	2007	-25998	Alive	TUMOR F	2067				Complete Remission/Response	0	2067	0	2067	0	2067	0	2067
TCGA-62-	TCGA-62-	LUAD	78	FEMALE	WHITE	Stage IB	Lung Adenocarcinon	2007	-28495	Alive	TUMOR F	2199				Complete Remission/Response	0	2199	0	2199	0	2199	0	2199
TCGA-62-	TCGA-62-	LUAD	70	FEMALE	WHITE	Stage IIIA	Lung Adenocarcinon	2008	-25905	Dead	WITH TUMOR		414	Distant Metastasis	314	Complete Remission/Response	1	414	1	414	1	314	1	314
TCGA-62-	TCGA-62-	LUAD	84	MALE	WHITE	Stage IB	Lung Adenocarcinon	2009	-30689	Dead	WITH TUMOR		1194	Locoregional Recurrence	539	Complete Remission/Response	1	1194	1	1194	1	539	1	539
TCGA-62-	TCGA-62-	LUAD	64	MALE	WHITE	Stage IIB	Lung Adenocarcinon	2010	-23689	Alive	TUMOR F	1246				Complete Remission/Response	0	1246	0	1246	0	1246	0	1246
TCGA-62-	TCGA-62-	LUAD	70	MALE	WHITE	Stage IIB	Lung Adenocarcinon	2011	-25893	Alive	WITH TUN	910		Locoregional Recurrence	290	Complete Remission/Response	0	910	0	910	1	290	1	290
TCGA-64-	TCGA-64-	LUAD	58	MALE	WHITE	Stage IA	Lung Adenocarcinon	2005	-21428	Alive	TUMOR F	1728				Complete Remission/Response	0	1728	0	1728	0	1728	0	1728
TCGA-64-	TCGA-64-	LUAD	77	FEMALE	WHITE	Stage IIIA	Lung Adenocarcinon	2005	-28482	Dead			628		360		1	628		628			1	360
TCGA-64-	TCGA-64-	LUAD	70	FEMALE	WHITE	[Discrepancy]	Lung Adenocarcinon	2006	-25931	Alive	TUMOR F	1189				Complete Remission/Response	0	1189	0	1189	0	1189	0	1189
TCGA-64-	TCGA-64-	LUAD	58	FEMALE	WHITE	Stage IIIA	Lung Adenocarcinon	2007	-21310	Alive	TUMOR F	2488				Complete Remission/Response	0	2488	0	2488	0	2488	0	2488
TCGA-64-	TCGA-64-	LUAD	63	MALE	WHITE	Stage IV	Lung Adenocarcinon	2007	-23267	Alive	TUMOR F	1126				Complete Remission/Response	0	1126	0	1126			0	1126
TCGA-64-	TCGA-64-	LUAD	61	FEMALE	WHITE	Stage IA	Lung Adenocarcinon	2008	-22525	Dead			1167		439		1	1167		1167			1	439
TCGA-64-	TCGA-64-	LUAD	60	MALE	WHITE	Stage IB	Lung Adenocarcinon	2004	-22059	Alive	TUMOR F	2676			246	Complete Remission/Response	0	2676	0	2676	1	246	1	246
TCGA-64-	TCGA-64-	LUAD	71	MALE	WHITE	Stage IIIA	Lung Adenocarcinon	2005	-26058	Dead	WITH TUMOR		62			Progressive Disease	1	62	1	62			1	62
TCGA-64-	TCGA-64-	LUAD	60	MALE	WHITE	Stage IB	Lung Adenocarcinon	2008	-21927	Alive	WITH TUN	1305		Distant Metastasis	962	Complete Remission/Response	0	1305	0	1305	1	962	1	962
TCGA-64-	TCGA-64-	LUAD	61	MALE	WHITE	Stage IIIA	Lung Adenocarcinon	2009	-22305	Alive	TUMOR F	864		Distant Metastasis	795	Stable Disease	0	864	0	864			1	795
TCGA-64-	TCGA-64-	LUAD	55	FEMALE	WHITE	Stage IB	Lung Adenocarcinon	2007	-20219	Alive	TUMOR F	1559			96	Progressive Disease	0	1559	0	1559			1	96
TCGA-64-	TCGA-64-	LUAD	74	MALE	WHITE	Stage IIB	Lung Adenocarcinon	2009	-27280	Alive	TUMOR F	866				Complete Remission/Response	0	866	0	866	0	866	0	866
TCGA-67-	TCGA-67-	LUAD	70	FEMALE	WHITE	Stage IA	Lung Adenocarcinon	2009	-25658	Alive	TUMOR F	610					0	610	0	610			0	610
TCGA-67-	TCGA-67-	LUAD	77	FEMALE	BLACK OI	Stage IA	Lung Adenocarcinon	2009	-28406	Alive	TUMOR F	610					0	610	0	610			0	610
TCGA-67-	TCGA-67-	LUAD	82	FEMALE	WHITE	Stage IB	Lung Adenocarcinon	2009	-29989	Alive	TUMOR F	573					0	573	0	573			0	573
TCGA-67-	TCGA-67-	LUAD	84	FEMALE	WHITE	Stage IB	Lung Adenocarcinon	2009	-30706	Alive	TUMOR F	427					0	427	0	427			0	427
TCGA-67-	TCGA-67-	LUAD	73	FEMALE	WHITE	Stage IB	Lung Adenocarcinon	2009	-26704	Alive	TUMOR F	385					0	385	0	385			0	385
TCGA-67-	TCGA-67-	LUAD	69	MALE	WHITE	[Discrepancy]	Lung Adenocarcinon	2009	-25202	Alive	TUMOR F	448					0	448	0	448			0	448
TCGA-67-	TCGA-67-	LUAD	52	FEMALE	WHITE	Stage IB	Lung Adenocarcinon	2010	-19310	Alive	TUMOR F	174				Complete Remission/Response	0	174	0	174	0	174	0	174
TCGA-67-	TCGA-67-	LUAD	57	FEMALE	WHITE	Stage IA	Lung Adenocarcinon	2010	-20967	Alive	TUMOR F	141				Complete Remission/Response	0	141	0	141	0	141	0	141
TCGA-67-	TCGA-67-	LUAD	73	FEMALE	WHITE	Stage IIA	Lung Adenocarcinon	2010	-26920	Alive	WITH TUN	422			296	Progressive Disease	0	422	0	422			1	296
TCGA-69-	TCGA-69-	LUAD	73	MALE	WHITE	Stage IIB	Lung Adenocarcinon	2011	-26777	Alive	TUMOR F	202					0	202	0	202			0	202
TCGA-69-	TCGA-69-	LUAD	84	MALE	WHITE	Stage IB	Lung Adenocarcinon	2011	-31041	Alive	TUMOR F	186					0	186	0	186			0	186
TCGA-69-	TCGA-69-	LUAD	69	MALE	WHITE	Stage IA	Lung Adenocarcinon	2010	-25316	Alive		690					0	690	0	690			0	690
TCGA-69-	TCGA-69-	LUAD	75	MALE	WHITE	Stage IA	Lung Adenocarcinon	2010	-27693	Alive	TUMOR F	414					0	414	0	414			0	414
TCGA-69-	TCGA-69-	LUAD	56	MALE	BLACK OI	[Discrepancy]	Lung Adenocarcinon	2011	-20625	Alive		165					0	165	0	165			0	165
TCGA-69-	TCGA-69-	LUAD	42	FEMALE	WHITE	Stage IB	Lung Adenocarcinon	2011	-15355	Alive	TUMOR F	230				Complete Remission/Response	0	230	0	230	0	230	0	230
TCGA-69-	TCGA-69-	LUAD	54	FEMALE	WHITE	Stage IIIA	Lung Adenocarcinon	2011	-20021	Alive	WITH TUN	184				Progressive Disease	0	184	0	184			0	184
TCGA-69-	TCGA-69-	LUAD	59	MALE	WHITE	Stage IIB	Lung Adenocarcinon	2011	-21785	Alive	[Discrepar	134				[Unknown]	0	134	0	134			0	134
TCGA-69-	TCGA-69-	LUAD	71	FEMALE	WHITE	Stage IB	Lung Adenocarcinon	2011	-26180	Alive		408				Stable Disease	0	408	0	408			0	408
TCGA-69-	TCGA-69-	LUAD	70	FEMALE	WHITE	Stage I	Lung Adenocarcinon	2011	-25583	Alive	TUMOR F	411				Complete Remission/Response	0	411	0	411	0	411	0	411
TCGA-69-	TCGA-69-	LUAD	59	FEMALE	BLACK OI	Stage IIA	Lung Adenocarcinon	2012	-21771	Alive	TUMOR F	426				Complete Remission/Response	0	426	0	426	0	426	0	426
TCGA-69-	TCGA-69-	LUAD	85	MALE	WHITE		Lung Adenocarcinon	2012	-31134	Alive	TUMOR F	409				Complete Remission/Response	0	409	0	409	0	409	0	409
TCGA-69-	TCGA-69-	LUAD	71	MALE	WHITE	Stage IA	Lung Adenocarcinon	2011	-26289	Alive		129				[Unknown]	0	129	0	129			0	129
TCGA-69-	TCGA-69-	LUAD	77	MALE	WHITE	Stage IIB	Lung Adenocarcinon	2012	-28413	Alive	WITH TUN	813		Locoregional Recurrence	344	Stable Disease	0	813	0	813			1	344
TCGA-69-	TCGA-69-	LUAD	60	FEMALE	BLACK OI	Stage IIB	Lung Adenocarcinon	2012	-22093	Alive	TUMOR F	591				Stable Disease	0	591	0	591			0	591
TCGA-71-	TCGA-71-	LUAD	48	FEMALE	ASIAN	Stage IIB	Lung Adenocarcinon	2011	-17793	Alive	WITH TUN	256		Locoregional Recurrence	164	Progressive Disease	0	256	0	256			1	164
TCGA-71-	TCGA-71-	LUAD	60	FEMALE	ASIAN	Stage IB	Lung Adenocarcinon	2011	-21921	Dead	WITH TUMOR		210	Locoregional Recurrence	179	Complete Remission/Response	1	210	1	210	1	179	1	179
TCGA-73-	TCGA-73-	LUAD	80	FEMALE	WHITE	Stage IB	Lung Adenocarcinon	2004	-29508	Dead	TUMOR F	1600	1600			Complete Remission/Response	1	1600	0	1600	0	1600	0	1600
TCGA-73-	TCGA-73-	LUAD	80	FEMALE	WHITE	Stage IB	Lung Adenocarcinon	2004	-29508	Dead	TUMOR F	1600	1600			Complete Remission/Response	1	1600	0	1600	0	1600	0	1600
TCGA-73-	TCGA-73-	LUAD	66	MALE	WHITE	Stage IIIA	Lung Adenocarcinon	2006	-24253	Dead	WITH TUN	711	711		35	Progressive Disease	1	711	1	711			1	35
TCGA-73-	TCGA-73-	LUAD	66	MALE	WHITE	Stage IIIA	Lung Adenocarcinon	2006	-24253	Dead	WITH TUN	711	711		35	Progressive Disease	1	711	1	711			1	35
TCGA-73-	TCGA-73-	LUAD	65	FEMALE	WHITE	Stage IA	Lung Adenocarcinon	2007	-23762	Alive	TUMOR F	2515			21	Complete Remission/Response	0	2515	0	2515	1	21	1	21
TCGA-73-	TCGA-73-	LUAD	65	FEMALE	WHITE	Stage IA	Lung Adenocarcinon	2007	-23762	Alive	TUMOR F	2515			21	Complete Remission/Response	0	2515	0	2515	1	21	1	21
TCGA-73-	TCGA-73-	LUAD	52	FEMALE	WHITE	Stage IV	Lung Adenocarcinon	2009	-19074	Alive	TUMOR F	800				Complete Remission/Response	0	800	0	800			0	800
TCGA-73-	TCGA-73-	LUAD	52	FEMALE	WHITE	Stage IV	Lung Adenocarcinon	2009	-19074	Alive	TUMOR F	800				Complete Remission/Response	0	800	0	800			0	800
TCGA-73-	TCGA-73-	LUAD	66	FEMALE	AMERICA	Stage IIB	Lung Adenocarcinon	2009	-24255	Alive	WITH TUN	467			236	Complete Remission/Response	0	467	0	467	1	236	1	236
TCGA-73-	TCGA-73-	LUAD	66	FEMALE	AMERICA	Stage IIB	Lung Adenocarcinon	2009	-24255	Alive	WITH TUN	467			236	Complete Remission/Response	0	467	0	467	1	236	1	236
TCGA-73-	TCGA-73-	LUAD	69	FEMALE	WHITE	Stage IV	Lung Adenocarcinon	2010	-25526	Alive	WITH TUN	131				Stable Disease	0	131	0	131			0	131
TCGA-73-	TCGA-73-	LUAD	69	FEMALE	WHITE	Stage IV	Lung Adenocarcinon	2010	-25526	Alive	WITH TUN	131				Stable Disease	0	131	0	131			0	131
TCGA-73-	TCGA-73-	LUAD	59	MALE	WHITE	Stage IIIA	Lung Adenocarcinon	2010	-21614	Dead	TUMOR FREE		922	Locoregional Recurrence	361	Progressive Disease	1	922	0	922			1	361
TCGA-73-	TCGA-73-	LUAD	59	MALE	WHITE	Stage IIIA	Lung Adenocarcinon	2010	-21614	Dead	TUMOR FREE		922	Locoregional Recurrence	361	Progressive Disease	1	922	0	922			1	361
TCGA-73-	TCGA-73-	LUAD	45	MALE	WHITE	Stage IIA	Lung Adenocarcinon	2010	-16746	Dead	WITH TUMOR		281			[Unknown]	1	281	1	281			1	281
TCGA-73-	TCGA-73-	LUAD	45	MALE	WHITE	Stage IIA	Lung Adenocarcinon	2010	-16746	Dead	WITH TUMOR		281			[Unknown]	1	281	1	281			1	281
TCGA-73-	TCGA-73-	LUAD	74	MALE	WHITE	[Discrepancy]	Lung Adenocarcinon	2010	-27381	Dead	WITH TUMOR		38			Stable Disease	1	38	1	38			1	38
TCGA-73-	TCGA-73-	LUAD	74	MALE	WHITE	[Discrepancy]	Lung Adenocarcinon	2010	-27381	Dead	WITH TUMOR		38			Stable Disease	1	38	1	38			1	38
TCGA-73-	TCGA-73-	LUAD	58	FEMALE	WHITE	Stage IA	Lung Adenocarcinon	2010	-21527	Alive	TUMOR F	1189				Complete Remission/Response	0	1189	0	1189	0	1189	0	1189
TCGA-73-	TCGA-73-	LUAD	81	FEMALE	WHITE	Stage IB	Lung Adenocarcinon	2010	-29683	Dead	TUMOR FREE		1531	Distant Metastasis	1447	Complete Remission/Response	1	1531	0	1531	1	1447	1	1447
TCGA-73-	TCGA-73-	LUAD	41	MALE	BLACK OI	Stage IIB	Lung Adenocarcinon	2013	-15162	Dead	WITH TUMOR		340	Locoregional Recurrence	107	Progressive Disease	1	340	1	340			1	107
TCGA-75-	TCGA-75-	LUAD		MALE		Stage IB	Lung Adenocarcinon	2005		Dead	WITH TUMOR						Complete Remission/Response	1		1				1	
TCGA-75-	TCGA-75-	LUAD		MALE		Stage IIB	Lung Adenocarcinoma			Dead	WITH TUMOR		2027		1752	Complete Remission/Response	1	2027	1	2027	1	1752	1	1752
TCGA-75-	TCGA-75-	LUAD		FEMALE		Stage IIIA	Lung Adenocarcinon	2007		Alive	WITH TUMOR				Partial Remission/Response	0		0				0	
TCGA-75-	TCGA-75-	LUAD		MALE		Stage IB	Lung Adenocarcinoma			Alive	TUMOR F	2368		New Primary Tumor	1773	Complete Remission/Response	0	2368	0	2368	0	2368	1	1773
TCGA-75-	TCGA-75-	LUAD		FEMALE		Stage IB	Lung Adenocarcinoma			Alive	TUMOR F	1333				Complete Remission/Response	0	1333	0	1333	0	1333	0	1333
TCGA-75-	TCGA-75-	LUAD		FEMALE		Stage IIIA	Lung Adenocarcinon	2008		Alive	TUMOR FREE					Complete Remission/Response	0		0				0	
TCGA-75-	TCGA-75-	LUAD		FEMALE		Stage IB	Lung Adenocarcinon	2006		Dead	WITH TUMOR					Complete Remission/Response	1		1				1	
TCGA-75-	TCGA-75-	LUAD		MALE		Stage IB	Lung Adenocarcinoma			Alive	TUMOR F	2590				Complete Remission/Response	0	2590	0	2590	0	2590	0	2590
TCGA-75-	TCGA-75-	LUAD		MALE		Stage IIIA	Lung Adenocarcinon	2007		Dead						Complete Remission/Response	1				0		0	
TCGA-75-	TCGA-75-	LUAD		FEMALE		Stage IB	Lung Adenocarcinon	2009		Dead	WITH TUMOR					Complete Remission/Response	1		1				1	
TCGA-75-	TCGA-75-	LUAD		FEMALE		Stage IIB	Lung Adenocarcinoma			Dead	WITH TUMOR		1516	New Primary Tumor	1340	Complete Remission/Response	1	1516	1	1516	0	1516	1	1340
TCGA-75-	TCGA-75-	LUAD		FEMALE		Stage IIIA	Lung Adenocarcinoma			Dead	WITH TUMOR		1115	Distant Metastasis	419	Complete Remission/Response	1	1115	1	1115	1	419	1	419
TCGA-75-	TCGA-75-	LUAD		MALE		Stage IB	Lung Adenocarcinoma			Alive	TUMOR F	3305		Locoregional Recurrence|Distan	1481	Complete Remission/Response	0	3305	0	3305	1	1481	1	1481
TCGA-75-	TCGA-75-	LUAD		MALE		Stage IB	Lung Adenocarcinoma			Alive	TUMOR F	3059		Locoregional Recurrence	2949	Complete Remission/Response	0	3059	0	3059	1	2949	1	2949
TCGA-75-	TCGA-75-	LUAD		MALE		Stage IIB	Lung Adenocarcinon	2007		Alive	TUMOR FREE					Complete Remission/Response	0		0		0		0	
TCGA-75-	TCGA-75-	LUAD		FEMALE		Stage IB	Lung Adenocarcinon	2008		Alive	TUMOR FREE						0		0				0	
TCGA-78-	TCGA-78-	LUAD	62	FEMALE	WHITE	Stage IB	Lung Adenocarcinon	1992	-22673	Dead	[Discrepancy]		4961		1500		1	4961		4961			1	1500
TCGA-78-	TCGA-78-	LUAD	52	FEMALE	WHITE	Stage IV	Lung Adenocarcinon	2000	-19080	Dead	WITH TUMOR		826		422		1	826	1	826			1	422
TCGA-78-	TCGA-78-	LUAD	71	FEMALE	WHITE	Stage IIIA	Lung Adenocarcinon	1999	-26272	Dead			173				1	173		173			0	173
TCGA-78-	TCGA-78-	LUAD	67	FEMALE	WHITE	Stage IIB	Lung Adenocarcinon	2001	-24809	Dead	WITH TUMOR		586		578		1	586	1	586			1	578
TCGA-78-	TCGA-78-	LUAD	71	MALE	WHITE	Stage IIB	Lung Adenocarcinon	2001	-25990	Dead			626		182		1	626		626			1	182
TCGA-78-	TCGA-78-	LUAD	71	MALE	WHITE	Stage IIIB	Lung Adenocarcinon	2002	-26285	Alive		3940				Complete Remission/Response	0	3940	0	3940	0	3940	0	3940
TCGA-78-	TCGA-78-	LUAD	59	MALE	WHITE	Stage IIB	Lung Adenocarcinon	2002	-21891	Dead	WITH TUMOR		666		139		1	666	1	666			1	139
TCGA-78-	TCGA-78-	LUAD	65	MALE	WHITE	Stage IB	Lung Adenocarcinon	2003	-23782	Dead	WITH TUMOR		1215		1202	Complete Remission/Response	1	1215	1	1215	1	1202	1	1202
TCGA-78-	TCGA-78-	LUAD	65	FEMALE	WHITE	Stage IB	Lung Adenocarcinon	2003	-23922	Alive		3635				Complete Remission/Response	0	3635	0	3635	0	3635	0	3635
TCGA-78-	TCGA-78-	LUAD	72	MALE	WHITE	Stage IIIA	Lung Adenocarcinon	2003	-26481	Dead			593				1	593		593			0	593
TCGA-78-	TCGA-78-	LUAD	68	MALE	WHITE	Stage IB	Lung Adenocarcinon	2005	-24863	Dead			1171		209		1	1171		1171			1	209
TCGA-78-	TCGA-78-	LUAD	62	MALE	WHITE	Stage IV	Lung Adenocarcinon	2006	-22720	Dead			976				1	976		976			0	976
TCGA-78-	TCGA-78-	LUAD	59	FEMALE	WHITE	Stage IIIB	Lung Adenocarcinon	2007	-21742	Dead	WITH TUMOR		179		132	Progressive Disease	1	179	1	179			1	132
TCGA-78-	TCGA-78-	LUAD	60	FEMALE	WHITE	Stage IA	Lung Adenocarcinon	2008	-22261	Alive		1974				Complete Remission/Response	0	1974	0	1974	0	1974	0	1974
TCGA-78-	TCGA-78-	LUAD	61	MALE	WHITE	Stage IV	Lung Adenocarcinon	2008	-22385	Dead			697			Complete Remission/Response	1	697		697			0	697
TCGA-78-	TCGA-78-	LUAD	69	FEMALE	WHITE	Stage IIB	Lung Adenocarcinon	2011	-25294	Dead	WITH TUMOR		291		162	Progressive Disease	1	291	1	291			1	162
TCGA-78-	TCGA-78-	LUAD	75	MALE	WHITE	Stage IA	Lung Adenocarcinon	1991	-27593	Dead			3169		2218		1	3169		3169			1	2218
TCGA-78-	TCGA-78-	LUAD	60	MALE	WHITE	Stage IB	Lung Adenocarcinon	1992	-22002	Alive	TUMOR F	7248				Complete Remission/Response	0	7248	0	7248	0	7248	0	7248
TCGA-78-	TCGA-78-	LUAD	60	MALE	WHITE	Stage IB	Lung Adenocarcinon	1992	-22002	Alive	TUMOR F	7248				Complete Remission/Response	0	7248	0	7248	0	7248	0	7248
TCGA-78-	TCGA-78-	LUAD	84	MALE	WHITE	Stage IIB	Lung Adenocarcinon	2002	-30869	Dead			258				1	258		258			0	258
TCGA-78-	TCGA-78-	LUAD	77	MALE	WHITE	Stage IV	Lung Adenocarcinon	2002	-28295	Dead	WITH TUMOR		2681		726		1	2681	1	2681			1	726
TCGA-78-	TCGA-78-	LUAD	53	FEMALE	WHITE	Stage IIIA	Lung Adenocarcinon	2000	-19480	Dead	WITH TUMOR		807		531		1	807	1	807			1	531
TCGA-78-	TCGA-78-	LUAD	45	MALE	WHITE	Stage IB	Lung Adenocarcinon	2000	-16571	Dead	WITH TUMOR		949		809	Stable Disease	1	949	1	949			1	809
TCGA-78-	TCGA-78-	LUAD	69	MALE	WHITE	Stage IIIA	Lung Adenocarcinon	2001	-25558	Dead	WITH TUMOR		244		231	Progressive Disease	1	244	1	244			1	231
TCGA-78-	TCGA-78-	LUAD	72	MALE		Stage IB	Lung Adenocarcinon	2002	-26610	Dead			1622				1	1622		1622			0	1622
TCGA-78-	TCGA-78-	LUAD	75	FEMALE	WHITE	Stage IIA	Lung Adenocarcinon	2011	-27445	Alive		791		Distant Metastasis	688	Complete Remission/Response	0	791	0	791	1	688	1	688
TCGA-78-	TCGA-78-	LUAD	66	FEMALE	WHITE	Stage IB	Lung Adenocarcinon	1999	-24440	Dead			1197			Complete Remission/Response	1	1197		1197	0	1197	0	1197
TCGA-78-	TCGA-78-	LUAD	66	FEMALE	WHITE	Stage IB	Lung Adenocarcinon	1999	-24440	Dead			1197			Complete Remission/Response	1	1197		1197	0	1197	0	1197
TCGA-78-	TCGA-78-	LUAD	56	MALE	WHITE	Stage IB	Lung Adenocarcinon	1993	-20763	Dead			321				1	321		321			0	321
TCGA-78-	TCGA-78-	LUAD	67	MALE	WHITE	Stage IB	Lung Adenocarcinon	2009	-24713	Dead	WITH TUMOR		1528	Locoregional Recurrence	1450	Complete Remission/Response	1	1528	1	1528	1	1450	1	1450
TCGA-78-	TCGA-78-	LUAD	59	MALE	[Not Evalu	Stage IIA	Lung Adenocarcinon	1994	-21832	Alive	TUMOR F	7062				Complete Remission/Response	0	7062	0	7062	0	7062	0	7062
TCGA-78-	TCGA-78-	LUAD	58	FEMALE	[Not Evalu	Stage IIB	Lung Adenocarcinon	2002	-21443	Dead			1209	Locoregional Recurrence	720	[Unknown]	1	1209		1209			1	720
TCGA-78-	TCGA-78-	LUAD	77	FEMALE	[Not Evalu	Stage IA	Lung Adenocarcinon	2003	-28379	Alive		2360				Complete Remission/Response	0	2360	0	2360	0	2360	0	2360
TCGA-78-	TCGA-78-	LUAD	69	MALE	[Not Evalu	Stage IIB	Lung Adenocarcinon	2005	-25422	Dead	WITH TUMOR		321	Distant Metastasis	242	[Unknown]	1	321	1	321			1	242
TCGA-78-	TCGA-78-	LUAD	53	FEMALE	[Not Evalu	Stage IB	Lung Adenocarcinon	2000	-19563	Dead			3361	Locoregional Recurrence	3044	Complete Remission/Response	1	3361		3361	1	3044	1	3044
TCGA-80-	TCGA-80-	LUAD		FEMALE		Stage IIB	Lung Adenocarcinon	2007		Alive	WITH TUMOR						0		0				0	
TCGA-80-	TCGA-80-	LUAD		FEMALE		Stage IA	Lung Adenocarcinoma			Alive	TUMOR F	2832				Complete Remission/Response	0	2832	0	2832	0	2832	0	2832
TCGA-80-	TCGA-80-	LUAD		MALE		Stage IB	Lung Adenocarcinoma			Alive	TUMOR F	2595				Complete Remission/Response	0	2595	0	2595	0	2595	0	2595
TCGA-83-	TCGA-83-	LUAD	59	FEMALE	WHITE	Stage IA	Lung Adenocarcinon	2009	-21760	Alive	TUMOR F	824				Stable Disease	0	824	0	824			0	824
TCGA-86-	TCGA-86-	LUAD	52	MALE	WHITE	Stage IIA	Lung Adenocarcinon	2011	-19301	Dead	WITH TUMOR		376		274	Progressive Disease	1	376	1	376			1	274
TCGA-86-	TCGA-86-	LUAD	73	FEMALE	WHITE	Stage IIA	Lung Adenocarcinon	2011	-26808	Alive	TUMOR F	179				Complete Remission/Response	0	179	0	179	0	179	0	179
TCGA-86-	TCGA-86-	LUAD	66	MALE	WHITE	Stage IV	Lung Adenocarcinon	2011	-24209	Alive	WITH TUN	947		Distant Metastasis	424	Complete Remission/Response	0	947	0	947			1	424
TCGA-86-	TCGA-86-	LUAD	70	MALE	WHITE	Stage IIA	Lung Adenocarcinon	2011	-25719	Dead	WITH TUMOR		1046			Progressive Disease	1	1046	1	1046			1	1046
TCGA-86-	TCGA-86-	LUAD	70	MALE	WHITE	Stage IIA	Lung Adenocarcinon	2011	-25719	Dead	WITH TUMOR		1046			Progressive Disease	1	1046	1	1046			1	1046
TCGA-86-	TCGA-86-	LUAD	70	MALE	WHITE	Stage IIA	Lung Adenocarcinon	2011	-25791	Alive	TUMOR F	1157				Complete Remission/Response	0	1157	0	1157	0	1157	0	1157
TCGA-86-	TCGA-86-	LUAD	70	MALE	WHITE	Stage IIA	Lung Adenocarcinon	2011	-25791	Alive	TUMOR F	1157				Complete Remission/Response	0	1157	0	1157	0	1157	0	1157
TCGA-86-	TCGA-86-	LUAD	61	FEMALE	BLACK OI	Stage IIIA	Lung Adenocarcinon	2011	-22619	Dead	WITH TUMOR		625			Stable Disease	1	625	1	625			1	625
TCGA-86-	TCGA-86-	LUAD	69	FEMALE	WHITE	Stage IA	Lung Adenocarcinon	2011	-25315	Alive	TUMOR F	997				Complete Remission/Response	0	997	0	997	0	997	0	997
TCGA-86-	TCGA-86-	LUAD	68	FEMALE	WHITE	Stage IB	Lung Adenocarcinon	2011	-25062	Alive	TUMOR F	605				Complete Remission/Response	0	605	0	605	0	605	0	605
TCGA-86-	TCGA-86-	LUAD	62	MALE	WHITE	Stage IB	Lung Adenocarcinon	2011	-22772	Alive	TUMOR F	1072		Locoregional Recurrence	820	Complete Remission/Response	0	1072	0	1072	1	820	1	820
TCGA-86-	TCGA-86-	LUAD	61	MALE	WHITE	Stage IIB	Lung Adenocarcinon	2011	-22584	Alive	TUMOR F	1148				Complete Remission/Response	0	1148	0	1148	0	1148	0	1148
TCGA-86-	TCGA-86-	LUAD	79	MALE	WHITE	Stage IIA	Lung Adenocarcinon	2011	-29124	Dead	TUMOR FREE		124			Complete Remission/Response	1	124	0	124	0	124	0	124
TCGA-86-	TCGA-86-	LUAD	63	FEMALE	WHITE	Stage IIIA	Lung Adenocarcinon	2011	-23369	Alive	TUMOR F	139				Complete Remission/Response	0	139	0	139	0	139	0	139
TCGA-86-	TCGA-86-	LUAD	58	MALE	WHITE	Stage IB	Lung Adenocarcinon	2011	-21214	Alive	TUMOR F	740				Complete Remission/Response	0	740	0	740	0	740	0	740
TCGA-86-	TCGA-86-	LUAD	62	FEMALE	WHITE	Stage IIA	Lung Adenocarcinon	2011	-22862	Alive	TUMOR F	24					0	24	0	24			0	24
TCGA-86-	TCGA-86-	LUAD	66	FEMALE	WHITE	Stage IB	Lung Adenocarcinon	2011	-24402	Dead	TUMOR FREE		694	Locoregional Recurrence	199	Complete Remission/Response	1	694	0	694	1	199	1	199
TCGA-86-	TCGA-86-	LUAD	42	MALE	WHITE	Stage IA	Lung Adenocarcinon	2011	-15605	Alive	TUMOR F	993				Complete Remission/Response	0	993	0	993	0	993	0	993
TCGA-86-	TCGA-86-	LUAD	63	FEMALE	WHITE	Stage IIB	Lung Adenocarcinon	2011	-23117	Alive	WITH TUN	944			29	Complete Remission/Response	0	944	0	944	1	29	1	29
TCGA-86-	TCGA-86-	LUAD	46	MALE	WHITE	Stage IIA	Lung Adenocarcinon	2011	-17032	Alive	TUMOR F	949				Complete Remission/Response	0	949	0	949	0	949	0	949
TCGA-86-	TCGA-86-	LUAD	54	FEMALE	WHITE	Stage IIA	Lung Adenocarcinon	2011	-19977	Alive	TUMOR F	701				Complete Remission/Response	0	701	0	701	0	701	0	701
TCGA-86-	TCGA-86-	LUAD	75	MALE	WHITE	Stage IA	Lung Adenocarcinon	2011	-27409	Alive	TUMOR F	0				Complete Remission/Response	0	0	0	0	0	0	0	0
TCGA-86-	TCGA-86-	LUAD	44	MALE	WHITE	Stage IB	Lung Adenocarcinon	2012	-16409	Alive	TUMOR F	653				Complete Remission/Response	0	653	0	653	0	653	0	653
TCGA-86-	TCGA-86-	LUAD	52	MALE	WHITE	Stage IIIA	Lung Adenocarcinon	2012	-19088	Dead	TUMOR FREE		444			[Unknown]	1	444	0	444			0	444
TCGA-86-	TCGA-86-	LUAD	57	MALE	WHITE	Stage IB	Lung Adenocarcinon	2012	-20918	Alive	TUMOR F	353				Complete Remission/Response	0	353	0	353	0	353	0	353
TCGA-86-	TCGA-86-	LUAD	61	FEMALE	WHITE	Stage IA	Lung Adenocarcinon	2012	-22383	Alive	TUMOR F	423				Complete Remission/Response	0	423	0	423	0	423	0	423
TCGA-86-	TCGA-86-	LUAD	64	MALE	WHITE	Stage IA	Lung Adenocarcinon	2012	-23443	Alive	TUMOR F	938		Distant Metastasis	849	Complete Remission/Response	0	938	0	938	1	849	1	849
TCGA-86-	TCGA-86-	LUAD	72	FEMALE	WHITE	Stage IIB	Lung Adenocarcinon	2012	-26448	Alive	TUMOR F	839				Complete Remission/Response	0	839	0	839	0	839	0	839
TCGA-86-	TCGA-86-	LUAD	59	MALE	WHITE	Stage IIB	Lung Adenocarcinon	2012	-21682	Dead	TUMOR FREE		19			Complete Remission/Response	1	19	0	19	0	19	0	19
TCGA-86-	TCGA-86-	LUAD	61	MALE	WHITE	Stage IB	Lung Adenocarcinon	2011	-22571	Alive	TUMOR F	862		Locoregional Recurrence	636	Complete Remission/Response	0	862	0	862	1	636	1	636
TCGA-86-	TCGA-86-	LUAD	50	MALE	WHITE	Stage IIA	Lung Adenocarcinon	2012	-18479	Alive	TUMOR F	806		Locoregional Recurrence	334	Complete Remission/Response	0	806	0	806	1	334	1	334
TCGA-86-	TCGA-86-	LUAD	78	FEMALE	ASIAN	Stage IA	Lung Adenocarcinon	2012	-28533	Alive	TUMOR F	896				Complete Remission/Response	0	896	0	896	0	896	0	896
TCGA-86-	TCGA-86-	LUAD	48	MALE	WHITE	Stage IIA	Lung Adenocarcinon	2012	-17607	Dead	TUMOR FREE		116			Complete Remission/Response	1	116	0	116	0	116	0	116
TCGA-86-	TCGA-86-	LUAD	56	MALE	WHITE	Stage IIB	Lung Adenocarcinon	2012	-20491	Dead	WITH TUMOR		737	Distant Metastasis	436	Complete Remission/Response	1	737	1	737	1	436	1	436
TCGA-86-	TCGA-86-	LUAD	63	FEMALE	WHITE	Stage IB	Lung Adenocarcinon	2012	-23330	Alive	TUMOR F	415				Complete Remission/Response	0	415	0	415	0	415	0	415
TCGA-86-	TCGA-86-	LUAD	59	FEMALE	WHITE	Stage IIIA	Lung Adenocarcinon	2012	-21738	Alive	TUMOR F	805				Complete Remission/Response	0	805	0	805	0	805	0	805
TCGA-91-	TCGA-91-	LUAD	70	MALE	WHITE	Stage IA	Lung Adenocarcinon	2011	-25870	Alive	TUMOR F	323				Complete Remission/Response	0	323	0	323	0	323	0	323
TCGA-91-	TCGA-91-	LUAD	70	MALE	WHITE	Stage IA	Lung Adenocarcinon	2011	-25870	Alive	TUMOR F	323				Complete Remission/Response	0	323	0	323	0	323	0	323
TCGA-91-	TCGA-91-	LUAD	78	MALE	WHITE	Stage IB	Lung Adenocarcinon	2001	-28841	Dead	TUMOR FREE		1258			Progressive Disease	1	1258	0	1258			0	1258
TCGA-91-	TCGA-91-	LUAD	78	MALE	WHITE	Stage IB	Lung Adenocarcinon	2001	-28841	Dead	TUMOR FREE		1258			Progressive Disease	1	1258	0	1258			0	1258
TCGA-91-	TCGA-91-	LUAD	65	FEMALE	WHITE	Stage IIA	Lung Adenocarcinon	2002	-23922	Alive	WITH TUN	60		Distant Metastasis	18	[Unknown]	0	60	0	60			1	18
TCGA-91-	TCGA-91-	LUAD	66	MALE	WHITE	Stage IB	Lung Adenocarcinon	2001	-24436	Alive	TUMOR F	310				Complete Remission/Response	0	310	0	310	0	310	0	310
TCGA-91-	TCGA-91-	LUAD	66	MALE	WHITE	Stage IB	Lung Adenocarcinon	2001	-24436	Alive	TUMOR F	310				Complete Remission/Response	0	310	0	310	0	310	0	310
TCGA-91-	TCGA-91-	LUAD	81	FEMALE	WHITE	Stage IA	Lung Adenocarcinon	2010	-29887	Alive	TUMOR F	79				Complete Remission/Response	0	79	0	79	0	79	0	79
TCGA-91-	TCGA-91-	LUAD	81	FEMALE	WHITE	Stage IA	Lung Adenocarcinon	2010	-29887	Alive	TUMOR F	79				Complete Remission/Response	0	79	0	79	0	79	0	79
TCGA-91-	TCGA-91-	LUAD	52	FEMALE	WHITE	Stage IB	Lung Adenocarcinon	2010	-19216	Alive	TUMOR F	417				Complete Remission/Response	0	417	0	417	0	417	0	417
TCGA-91-	TCGA-91-	LUAD	52	FEMALE	WHITE	Stage IB	Lung Adenocarcinon	2010	-19216	Alive	TUMOR F	417				Complete Remission/Response	0	417	0	417	0	417	0	417
TCGA-91-	TCGA-91-	LUAD	59	FEMALE	WHITE	Stage IA	Lung Adenocarcinon	2011	-21854	Alive	TUMOR F	372				Complete Remission/Response	0	372	0	372	0	372	0	372
TCGA-91-	TCGA-91-	LUAD	62	FEMALE	WHITE	Stage IB	Lung Adenocarcinon	2002	-22862	Alive	WITH TUN	842		Distant Metastasis	772	Progressive Disease	0	842	0	842			1	772
TCGA-91-	TCGA-91-	LUAD	62	FEMALE	WHITE	Stage IB	Lung Adenocarcinon	2002	-22862	Alive	WITH TUN	842		Distant Metastasis	772	Progressive Disease	0	842	0	842			1	772
TCGA-91-	TCGA-91-	LUAD	59	MALE	WHITE	Stage IIIA	Lung Adenocarcinon	2009	-21829	Alive	TUMOR F	224				Complete Remission/Response	0	224	0	224	0	224	0	224
TCGA-91-	TCGA-91-	LUAD	75	FEMALE	BLACK OI	Stage IIIA	Lung Adenocarcinon	2009	-27676	Alive		35				[Unknown]	0	35	0	35			0	35
TCGA-91-	TCGA-91-	LUAD	75	FEMALE	BLACK OI	Stage IIIA	Lung Adenocarcinon	2009	-27676	Alive		35				[Unknown]	0	35	0	35			0	35
TCGA-91-	TCGA-91-	LUAD	62	MALE	WHITE	Stage IIB	Lung Adenocarcinon	2011	-22926	Alive	TUMOR F	492				Complete Remission/Response	0	492	0	492	0	492	0	492
TCGA-91-	TCGA-91-	LUAD	63	FEMALE	WHITE	Stage IB	Lung Adenocarcinon	2011	-23358	Alive	TUMOR F	505				Complete Remission/Response	0	505	0	505	0	505	0	505
TCGA-91-	TCGA-91-	LUAD	75	FEMALE	WHITE	Stage IA	Lung Adenocarcinon	2012	-27632	Dead	TUMOR FREE		434			Complete Remission/Response	1	434	0	434	0	434	0	434
TCGA-91-	TCGA-91-	LUAD	76	FEMALE	WHITE	Stage IA	Lung Adenocarcinon	2011	-27853	Alive		36				[Unknown]	0	36	0	36			0	36
TCGA-91-	TCGA-91-	LUAD	59	MALE	WHITE	Stage IIA	Lung Adenocarcinon	2012	-21565	Alive		44				[Unknown]	0	44	0	44			0	44
TCGA-91-	TCGA-91-	LUAD	78	MALE	WHITE	Stage IIA	Lung Adenocarcinon	2012	-28544	Alive	TUMOR F	603				Complete Remission/Response	0	603	0	603	0	603	0	603
TCGA-93-	TCGA-93-	LUAD	76	FEMALE	WHITE	Stage IA	Lung Adenocarcinon	2011	-27949	Alive	TUMOR F	683				Complete Remission/Response	0	683	0	683	0	683	0	683
TCGA-93-	TCGA-93-	LUAD	75	FEMALE	WHITE	Stage IA	Lung Adenocarcinon	2011	-27456	Alive	TUMOR F	531				Complete Remission/Response	0	531	0	531	0	531	0	531
TCGA-93-	TCGA-93-	LUAD	75	FEMALE	WHITE	Stage IA	Lung Adenocarcinon	2011	-27456	Alive	TUMOR F	531				Complete Remission/Response	0	531	0	531	0	531	0	531
TCGA-93-	TCGA-93-	LUAD	77	MALE	ASIAN	Stage IB	Lung Adenocarcinon	2011	-28453	Alive	TUMOR F	186				Complete Remission/Response	0	186	0	186	0	186	0	186
TCGA-93-	TCGA-93-	LUAD	71	MALE	WHITE	Stage IV	Lung Adenocarcinon	2012	-26273	Alive	WITH TUN	718				Stable Disease	0	718	0	718			0	718
TCGA-93-	TCGA-93-	LUAD	70	MALE	WHITE	Stage IA	Lung Adenocarcinon	2012	-25898	Dead	TUMOR FREE		33				1	33	0	33			0	33
TCGA-93-	TCGA-93-	LUAD	64	MALE	ASIAN	Stage IV	Lung Adenocarcinon	2012	-23620	Alive	WITH TUN	578		Distant Metastasis	497	Partial Remission/Response	0	578	0	578			1	497
TCGA-93-	TCGA-93-	LUAD	49	MALE	WHITE	Stage IA	Lung Adenocarcinon	2012	-17951	Alive	TUMOR F	526				Complete Remission/Response	0	526	0	526	0	526	0	526
TCGA-95-	TCGA-95-	LUAD	54	FEMALE	WHITE	Stage IIB	Lung Adenocarcinon	2011	-19981	Alive	WITH TUN	1272		Locoregional Recurrence	1258	Complete Remission/Response	0	1272	0	1272	1	1258	1	1258
TCGA-95-	TCGA-95-	LUAD	63	FEMALE	WHITE	Stage IA	Lung Adenocarcinon	2011	-23165	Dead	WITH TUMOR		503			Complete Remission/Response	1	503	1	503			1	503
TCGA-95-	TCGA-95-	LUAD	71	MALE	BLACK OI	Stage IIA	Lung Adenocarcinon	2011	-26000	Dead	TUMOR FREE		87			[Not Evaluated]	1	87	0	87			0	87
TCGA-95-	TCGA-95-	LUAD	61	MALE	WHITE	Stage IIB	Lung Adenocarcinon	2011	-22437	Alive	TUMOR F	568				Stable Disease	0	568	0	568			0	568
TCGA-95-	TCGA-95-	LUAD	71	MALE	WHITE	Stage IA	Lung Adenocarcinon	2011	-26003	Alive	TUMOR F	377				Stable Disease	0	377	0	377			0	377
TCGA-95-	TCGA-95-	LUAD	67	MALE	WHITE	Stage IA	Lung Adenocarcinon	2011	-24752	Alive	TUMOR F	477				Complete Remission/Response	0	477	0	477	0	477	0	477
TCGA-95-	TCGA-95-	LUAD	42	FEMALE	WHITE	Stage IB	Lung Adenocarcinon	2011	-15363	Alive	TUMOR F	476				Complete Remission/Response	0	476	0	476	0	476	0	476
TCGA-95-	TCGA-95-	LUAD	72	MALE	WHITE	Stage IA	Lung Adenocarcinon	2012	-26317	Alive	TUMOR F	830		Locoregional Recurrence	228	Complete Remission/Response	0	830	0	830	1	228	1	228
TCGA-95-	TCGA-95-	LUAD	67	MALE	WHITE	Stage IIA	Lung Adenocarcinon	2012	-24773	Alive		84				Complete Remission/Response	0	84	0	84	0	84	0	84
TCGA-95-	TCGA-95-	LUAD	74	FEMALE	WHITE	Stage IIIA	Lung Adenocarcinon	2012	-27040	Alive	WITH TUN	651		Distant Metastasis	496	Stable Disease	0	651	0	651			1	496
TCGA-95-	TCGA-95-	LUAD	62	FEMALE	WHITE	Stage IIA	Lung Adenocarcinon	2012	-22927	Alive	TUMOR F	553				Stable Disease	0	553	0	553			0	553
TCGA-95-	TCGA-95-	LUAD	66	FEMALE	WHITE	Stage IIIA	Lung Adenocarcinon	2012	-24130	Alive	WITH TUN	605		Distant Metastasis	216	Stable Disease	0	605	0	605			1	216
TCGA-97-	TCGA-97-	LUAD	76	FEMALE	WHITE	Stage IA	Lung Adenocarcinon	2008	-27913	Alive	WITH TUN	1285		New Primary Tumor	1255	Progressive Disease	0	1285	0	1285			1	1255
TCGA-97-	TCGA-97-	LUAD	67	FEMALE	WHITE	Stage IB	Lung Adenocarcinon	2007	-24737	Alive	WITH TUN	1965			775	Progressive Disease	0	1965	0	1965			1	775
TCGA-97-	TCGA-97-	LUAD	70	MALE	WHITE	Stage IB	Lung Adenocarcinon	2007	-25578	Alive	TUMOR F	1932			798	Stable Disease	0	1932	0	1932			1	798
TCGA-97-	TCGA-97-	LUAD	58	FEMALE	WHITE	Stage IA	Lung Adenocarcinon	2007	-21518	Alive	TUMOR F	1870				Complete Remission/Response	0	1870	0	1870	0	1870	0	1870
TCGA-97-	TCGA-97-	LUAD	83	FEMALE	WHITE	Stage IIIA	Lung Adenocarcinon	2010	-30327	Alive	TUMOR F	775				Complete Remission/Response	0	775	0	775	0	775	0	775
TCGA-97-	TCGA-97-	LUAD	65	MALE	WHITE	Stage IB	Lung Adenocarcinon	2011	-23919	Alive	TUMOR F	564				Stable Disease	0	564	0	564			0	564
TCGA-97-	TCGA-97-	LUAD	76	FEMALE	WHITE	Stage IA	Lung Adenocarcinon	2011	-27980	Dead	TUMOR FREE		18			Complete Remission/Response	1	18	0	18	0	18	0	18

TCGA-97-	TCGA-97-	LUAD	72	FEMALE	WHITE	Stage IA	Lung Adenocarcinon	2011	-26298	Alive	TUMOR F	484				Complete Remission/Response	0	484	0	484	0	484	0	484
TCGA-97-	TCGA-97-	LUAD	81	MALE	ASIAN	Stage IV	Lung Adenocarcinon	2011	-29627	Alive	WITH TUI	568		Distant Metastasis	441	Progressive Disease	0	568	0	568			1	441
TCGA-97-	TCGA-97-	LUAD	75	FEMALE	WHITE	Stage IB	Lung Adenocarcinon	2011	-27416	Alive	TUMOR F	545				Complete Remission/Response	0	545	0	545	0	545	0	545
TCGA-97-	TCGA-97-	LUAD	67	MALE	WHITE	Stage IIA	Lung Adenocarcinon	2011	-24725	Dead			164				1	164		164			0	164
TCGA-97-	TCGA-97-	LUAD	55	FEMALE	WHITE	Stage IB	Lung Adenocarcinon	2011	-20206	Alive	TUMOR F	551		Locoregional Recurrence	294	Complete Remission/Response	0	551	0	551	1	294	1	294
TCGA-97-	TCGA-97-	LUAD	63	MALE	WHITE	Stage IIIA	Lung Adenocarcinon	2012	-23170	Dead	TUMOR FREE		468	Distant Metastasis	39	Stable Disease	1	468	0	468			1	39
TCGA-97-	TCGA-97-	LUAD	59	FEMALE	WHITE	Stage IB	Lung Adenocarcinon	2012	-21648	Alive	TUMOR F	499				[Not Evaluated]	0	499	0	499			0	499
TCGA-97-	TCGA-97-	LUAD	72	MALE	WHITE	Stage IA	Lung Adenocarcinon	2012	-26305	Alive	TUMOR F	435				Complete Remission/Response	0	435	0	435	0	435	0	435
TCGA-97-	TCGA-97-	LUAD	78	FEMALE	[Not Evalu	Stage IIIA	Lung Adenocarcinon	2012	-28801	Alive	TUMOR F	657				Complete Remission/Response	0	657	0	657	0	657	0	657
TCGA-97-	TCGA-97-	LUAD	55	FEMALE	WHITE	Stage I	Lung Adenocarcinon	2012	-20331	Alive	TUMOR F	626				Complete Remission/Response	0	626	0	626	0	626	0	626
TCGA-97-	TCGA-97-	LUAD	81	MALE	WHITE	Stage IB	Lung Adenocarcinon	2012	-29915	Alive	TUMOR F	614				Complete Remission/Response	0	614	0	614	0	614	0	614
TCGA-97-	TCGA-97-	LUAD	60	FEMALE	WHITE	Stage IB	Lung Adenocarcinon	2012	-22206	Alive	TUMOR F	652				Complete Remission/Response	0	652	0	652	0	652	0	652
TCGA-97-	TCGA-97-	LUAD	52	FEMALE	WHITE	Stage IA	Lung Adenocarcinon	2012	-19272	Alive	TUMOR F	601				Complete Remission/Response	0	601	0	601	0	601	0	601
TCGA-97-	TCGA-97-	LUAD	66	MALE	WHITE	Stage IA	Lung Adenocarcinon	2012	-24313	Alive	TUMOR F	624				Complete Remission/Response	0	624	0	624	0	624	0	624
TCGA-97-	TCGA-97-	LUAD	69	FEMALE	WHITE	Stage IA	Lung Adenocarcinon	2012	-25384	Alive	TUMOR F	540		New Primary Tumor	54	Complete Remission/Response	0	540	0	540	0	540	1	54
TCGA-97-	TCGA-97-	LUAD	83	MALE	WHITE	Stage IA	Lung Adenocarcinon	2012	-30665	Alive	TUMOR F	634				Complete Remission/Response	0	634	0	634	0	634	0	634
TCGA-97-	TCGA-97-	LUAD	45	FEMALE	WHITE	Stage IA	Lung Adenocarcinon	2012	-16764	Alive	TUMOR F	568				Complete Remission/Response	0	568	0	568	0	568	0	568
TCGA-97-	TCGA-97-	LUAD	74	MALE	WHITE	Stage IA	Lung Adenocarcinon	2012	-27062	Alive	TUMOR F	629				Complete Remission/Response	0	629	0	629	0	629	0	629
TCGA-99-	TCGA-99-	LUAD	74	FEMALE	WHITE	Stage IIIA	Lung Adenocarcinon	2010	-27359	Alive	WITH TUI	747				Stable Disease	0	747	0	747			0	747
TCGA-99-	TCGA-99-	LUAD	72	FEMALE	BLACK OI	Stage IIIA	Lung Adenocarcinon	2010	-26634	Alive	TUMOR F	1060				Complete Remission/Response	0	1060	0	1060	0	1060	0	1060
TCGA-99-	TCGA-99-	LUAD	50	FEMALE	BLACK OI	Stage IA	Lung Adenocarcinon	2011	-18542	Alive	TUMOR F	1118				Complete Remission/Response	0	1118	0	1118	0	1118	0	1118
TCGA-99-	TCGA-99-	LUAD	61	MALE	WHITE	Stage IA	Lung Adenocarcinon	2011	-22635	Alive	TUMOR F	44				Complete Remission/Response	0	44	0	44	0	44	0	44
TCGA-99-	TCGA-99-	LUAD	74	FEMALE	WHITE	Stage IV	Lung Adenocarcinon	2011	-27342	Dead	WITH TUMOR		656			Progressive Disease	1	656	1	656			1	656
TCGA-99-	TCGA-99-	LUAD	70	FEMALE	BLACK OI	Stage IA	Lung Adenocarcinon	2013	-25778	Alive	TUMOR F	658				Complete Remission/Response	0	658	0	658	0	658	0	658
TCGA-J2-	TCGA-J2-	LUAD	65	FEMALE	WHITE	Stage IIA	Lung Adenocarcinon	2011	-23892	Alive	TUMOR F	739		Locoregional Recurrence	482	Progressive Disease	0	739	0	739			1	482
TCGA-J2-	TCGA-J2-	LUAD	69	FEMALE	WHITE	Stage IIB	Lung Adenocarcinon	2011	-25441	Alive	WITH TUI	724		Distant Metastasis	470	Progressive Disease	0	724	0	724			1	470
TCGA-J2-	TCGA-J2-	LUAD	61	FEMALE	WHITE	Stage IA	Lung Adenocarcinon	2012	-22414	Dead	WITH TUMOR		550	Locoregional Recurrence|Distan	524	[Unknown]	1	550	1	550			1	524
TCGA-J2-	TCGA-J2-	LUAD	77	FEMALE	WHITE	Stage IA	Lung Adenocarcinon	2012	-28163	Alive	TUMOR F	1079				Complete Remission/Response	0	1079	0	1079	0	1079	0	1079
TCGA-J2-	TCGA-J2-	LUAD	66	FEMALE	WHITE	Stage IA	Lung Adenocarcinon	2012	-24224	Alive	TUMOR F	988				Complete Remission/Response	0	988	0	988	0	988	0	988
TCGA-L4-	TCGA-L4-	LUAD	48	FEMALE	WHITE	Stage I	Lung Adenocarcinon	2012	-17680	Alive	TUMOR F	578				Complete Remission/Response	0	578	0	578	0	578	0	578
TCGA-L4-	TCGA-L4-	LUAD	67	MALE	WHITE	Stage IA	Lung Adenocarcinon	2012	-24631	Alive	TUMOR F	435				Complete Remission/Response	0	435	0	435	0	435	0	435
TCGA-L9-	TCGA-L9-	LUAD	63	FEMALE	WHITE	Stage IA	Lung Adenocarcinon	2012	-23062	Dead	TUMOR FREE		193			Complete Remission/Response	1	193	0	193	0	193	0	193
TCGA-L9-	TCGA-L9-	LUAD	60	FEMALE	WHITE	Stage IA	Lung Adenocarcinon	2012	-22202	Alive	TUMOR F	307				Complete Remission/Response	0	307	0	307	0	307	0	307
TCGA-L9-	TCGA-L9-	LUAD	75	MALE	BLACK OI	Stage IIA	Lung Adenocarcinon	2012	-27414	Dead	WITH TUMOR		442	Distant Metastasis	389	Complete Remission/Response	1	442	1	442	1	389	1	389
TCGA-L9-	TCGA-L9-	LUAD	40	FEMALE	BLACK OI	Stage IV	Lung Adenocarcinon	2012	-14681	Dead	WITH TUMOR		58	Locoregional Recurrence|Distan	49	Progressive Disease	1	58	1	58			1	49
TCGA-L9-	TCGA-L9-	LUAD	56	MALE	BLACK OI	Stage IIA	Lung Adenocarcinon	2013	-20780	Alive	TUMOR F	664				Complete Remission/Response	0	664	0	664	0	664	0	664
TCGA-L9-	TCGA-L9-	LUAD	69	MALE	BLACK OI	Stage IIA	Lung Adenocarcinon	2013	-25298	Alive	TUMOR F	565				Complete Remission/Response	0	565	0	565	0	565	0	565
TCGA-L9-	TCGA-L9-	LUAD	64	FEMALE	BLACK OI	Stage IB	Lung Adenocarcinon	2013	-23617	Alive	TUMOR F	476					0	476	0	476			0	476
TCGA-MN	TCGA-MN	LUAD	60	MALE	BLACK OI	Stage IIA	Lung Adenocarcinon	2011	-21939	Alive	TUMOR F	827				Complete Remission/Response	0	827	0	827	0	827	0	827
TCGA-MN	TCGA-MN	LUAD	57	MALE	WHITE	Stage IA	Lung Adenocarcinon	2010	-20888	Alive	TUMOR F	1175				Complete Remission/Response	0	1175	0	1175	0	1175	0	1175
TCGA-MN	TCGA-MN	LUAD	63	MALE	WHITE	Stage IA	Lung Adenocarcinon	2011	-23257	Alive	TUMOR F	84				Complete Remission/Response	0	84	0	84	0	84	0	84
TCGA-MP	TCGA-MP	LUAD	67	MALE	[Unknowr	Stage IB	Lung Adenocarcinon	2001	-24516	Dead			2620			Complete Remission/Response	1	2620		2620	0	2620	0	2620
TCGA-MP	TCGA-MP	LUAD	53	MALE	WHITE	Stage IIB	Lung Adenocarcinon	2002	-19640	Dead			1778			Complete Remission/Response	1	1778		1778	0	1778	0	1778
TCGA-MP	TCGA-MP	LUAD	61	MALE	WHITE	Stage IIB	Lung Adenocarcinon	2002	-22448	Dead	TUMOR FREE		1501	Locoregional Recurrence	489	Complete Remission/Response	1	1501	0	1501	1	489	1	489
TCGA-MP	TCGA-MP	LUAD	71	MALE	WHITE	Stage IA	Lung Adenocarcinon	2003	-26010	Dead	WITH TUMOR		1136	Distant Metastasis	575	Complete Remission/Response	1	1136	1	1136	1	575	1	575
TCGA-MP	TCGA-MP	LUAD	68	FEMALE	WHITE	Stage IIB	Lung Adenocarcinon	2004	-25052	Dead			2617			Complete Remission/Response	1	2617		2617	0	2617	0	2617
TCGA-MP	TCGA-MP	LUAD	76	FEMALE	WHITE	Stage IIIA	Lung Adenocarcinon	2005	-27993	Dead	TUMOR FREE		1790			Complete Remission/Response	1	1790	0	1790	0	1790	0	1790
TCGA-MP	TCGA-MP	LUAD	75	FEMALE	[Unknowr	Stage IV	Lung Adenocarcinon	2005	-27603	Dead			167			[Unknown]	1	167		167			0	167
TCGA-MP	TCGA-MP	LUAD	68	MALE	[Unknowr	Stage IIIA	Lung Adenocarcinon	2006	-25069	Dead			161			[Unknown]	1	161		161			0	161
TCGA-MP	TCGA-MP	LUAD	54	FEMALE	WHITE	Stage IIIA	Lung Adenocarcinon	2006	-19767	Dead	WITH TUMOR		1265	Locoregional Recurrence|Distan	341	Complete Remission/Response	1	1265	1	1265	1	341	1	341
TCGA-MP	TCGA-MP	LUAD	75	FEMALE	WHITE	Stage IA	Lung Adenocarcinon	2006	-27513	Dead	WITH TUMOR		950	Distant Metastasis	725	Complete Remission/Response	1	950	1	950	1	725	1	725
TCGA-MP	TCGA-MP	LUAD	77	MALE	WHITE	Stage IIIA	Lung Adenocarcinon	2008	-28471	Dead	TUMOR FREE		74			Complete Remission/Response	1	74	0	74	0	74	0	74
TCGA-MP	TCGA-MP	LUAD	71	MALE	WHITE	Stage IIIA	Lung Adenocarcinon	2008	-25983	Dead			307	Locoregional Recurrence	226	Complete Remission/Response	1	307		307	1	226	1	226
TCGA-MP	TCGA-MP	LUAD	56	MALE	WHITE	Stage IIA	Lung Adenocarcinon	2010	-20627	Dead	WITH TUMOR		896	Locoregional Recurrence	226	Complete Remission/Response	1	896	1	896	1	226	1	226
TCGA-MP	TCGA-MP	LUAD	58	FEMALE	WHITE	Stage IIA	Lung Adenocarcinon	2010	-21387	Dead	WITH TUMOR		336	Distant Metastasis	195	Partial Remission/Response	1	336	1	336			1	195
TCGA-MP	TCGA-MP	LUAD	70	FEMALE	WHITE	Stage IA	Lung Adenocarcinon	2012	-25759	Alive	TUMOR F	741				Complete Remission/Response	0	741	0	741	0	741	0	741
TCGA-MP	TCGA-MP	LUAD	72	MALE	WHITE	Stage IIA	Lung Adenocarcinon	2010	-26565	Dead	WITH TUMOR		429	Locoregional Recurrence|Distan	84	[Unknown]	1	429	1	429			1	84
TCGA-MP	TCGA-MP	LUAD	62	FEMALE	[Unknowr	Stage IA	Lung Adenocarcinon	2006	-22875	Dead			339			Complete Remission/Response	1	339		339	0	339	0	339
TCGA-MP	TCGA-MP	LUAD	56	FEMALE	[Unknowr	Stage IIB	Lung Adenocarcinon	2002	-20581	Dead	WITH TUMOR		582	Distant Metastasis	397	Complete Remission/Response	1	582	1	582	1	397	1	397
TCGA-MP	TCGA-MP	LUAD	76	FEMALE	WHITE	Stage IB	Lung Adenocarcinon	2008	-28015	Alive	TUMOR F	2248				Complete Remission/Response	0	2248	0	2248	0	2248	0	2248
TCGA-NJ	TCGA-NJ	LUAD	50	FEMALE	BLACK OI	Stage IA	Lung Adenocarcinon	2007	-18584	Alive	TUMOR F	2161				Stable Disease	0	2161	0	2161			0	2161
TCGA-NJ	TCGA-NJ	LUAD	65	MALE	WHITE	Stage IB	Lung Adenocarcinon	2008	-23812	Alive	TUMOR F	2261				Stable Disease	0	2261	0	2261			0	2261
TCGA-NJ	TCGA-NJ	LUAD	87	FEMALE	WHITE	Stage IIIA	Lung Adenocarcinon	2009	-31867	Dead			4			[Unknown]	1	4		4			0	4
TCGA-NJ	TCGA-NJ	LUAD	52	MALE	WHITE	Stage IB	Lung Adenocarcinon	2011	-19106	Alive	TUMOR F	50				Stable Disease	0	50	0	50			0	50
TCGA-NJ	TCGA-NJ	LUAD	69	FEMALE	WHITE	Stage IA	Lung Adenocarcinon	2011	-25427	Alive	TUMOR F	1432				Stable Disease	0	1432	0	1432			0	1432
TCGA-NJ	TCGA-NJ	LUAD	76	FEMALE	WHITE	Stage IB	Lung Adenocarcinon	2009	-28022	Alive	TUMOR F	15				Stable Disease	0	15	0	15			0	15
TCGA-NJ	TCGA-NJ	LUAD	56	FEMALE	WHITE	Stage IIA	Lung Adenocarcinon	2011	-20628	Alive	TUMOR F	13				Stable Disease	0	13	0	13			0	13
TCGA-NJ	TCGA-NJ	LUAD	67	MALE	WHITE	Stage IA	Lung Adenocarcinon	2011	-24738	Alive	TUMOR F	603				Stable Disease	0	603	0	603			0	603
TCGA-NJ	TCGA-NJ	LUAD	49	MALE	BLACK OI	Stage IIIA	Lung Adenocarcinon	2013	-18127	Alive	WITH TUI	617				Stable Disease	0	617	0	617			0	617
TCGA-O1	TCGA-O1	LUAD	74	FEMALE	WHITE	Stage IA	Lung Adenocarcinon	2007	-27223	Dead	WITH TUMOR		1798	Locoregional Recurrence	898	[Unknown]	1	1798	1	1798			1	898
TCGA-S2-	TCGA-S2-	LUAD	68	FEMALE	BLACK OI	Stage IA	Lung Adenocarcinon	2013	-24903	Alive	TUMOR F	513				Complete Remission/Response	0	513	0	513	0	513	0	513
TCGA-18-	TCGA-18-	LUSC	67	MALE	WHITE	Stage IA	Lung Squamous Cell	2003	-24477	Dead	WITH TUMOR		371	Locoregional Recurrence	357	Progressive Disease	1	371	1	371			1	357
TCGA-18-	TCGA-18-	LUSC	67	MALE	WHITE	Stage IA	Lung Squamous Cell	2003	-24477	Dead	WITH TUMOR		371	Locoregional Recurrence	357	Progressive Disease	1	371	1	371			1	357
TCGA-18-	TCGA-18-	LUSC	72	MALE	ASIAN	Stage IB	Lung Squamous Cell	2003	-26615	Dead	TUMOR FREE		136				1	136	0	136			0	136
TCGA-18-	TCGA-18-	LUSC	72	MALE	ASIAN	Stage IB	Lung Squamous Cell	2003	-26615	Dead	TUMOR FREE		136				1	136	0	136			0	136
TCGA-18-	TCGA-18-	LUSC	77	FEMALE	WHITE	Stage IB	Lung Squamous Cell	2004	-28171	Dead	WITH TUMOR		2304	Locoregional Recurrence	1793	[Unknown]	1	2304	1	2304			1	1793
TCGA-18-	TCGA-18-	LUSC	77	FEMALE	WHITE	Stage IB	Lung Squamous Cell	2004	-28171	Dead	WITH TUMOR		2304	Locoregional Recurrence	1793	[Unknown]	1	2304	1	2304			1	1793
TCGA-18-	TCGA-18-	LUSC	74	MALE	WHITE	Stage IA	Lung Squamous Cell	2004	-27154	Alive	TUMOR F	3747		New Primary Tumor	2291	Complete Remission/Response	0	3747	0	3747	0	3747	1	2291
TCGA-18-	TCGA-18-	LUSC	74	MALE	WHITE	Stage IA	Lung Squamous Cell	2004	-27154	Alive	TUMOR F	3747		New Primary Tumor	2291	Complete Remission/Response	0	3747	0	3747	0	3747	1	2291
TCGA-18-	TCGA-18-	LUSC	81	MALE		Stage IIB	Lung Squamous Cell	2004	-29827	Dead	TUMOR FREE		146				1	146	0	146			0	146
TCGA-18-	TCGA-18-	LUSC	81	MALE		Stage IIB	Lung Squamous Cell	2004	-29827	Dead	TUMOR FREE		146				1	146	0	146			0	146
TCGA-18-	TCGA-18-	LUSC	63	FEMALE		Stage IIIA	Lung Squamous Cell	2005	-23370	Alive	TUMOR F	3576				[Unknown]	0	3576	0	3576			0	3576
TCGA-18-	TCGA-18-	LUSC	63	FEMALE		Stage IIIA	Lung Squamous Cell	2005	-23370	Alive	TUMOR F	3576				[Unknown]	0	3576	0	3576			0	3576
TCGA-18-	TCGA-18-	LUSC	52	MALE	WHITE	Stage IB	Lung Squamous Cell	2005	-19025	Dead	WITH TUMOR		345	Distant Metastasis	273	Progressive Disease	1	345	1	345			1	273
TCGA-18-	TCGA-18-	LUSC	52	MALE	WHITE	Stage IB	Lung Squamous Cell	2005	-19025	Dead	WITH TUMOR		345	Distant Metastasis	273	Progressive Disease	1	345	1	345			1	273
TCGA-18-	TCGA-18-	LUSC	73	MALE	ASIAN	Stage IV	Lung Squamous Cell	2005	-26938	Dead	TUMOR FREE		716			Complete Remission/Response	1	716	0	716			0	716
TCGA-18-	TCGA-18-	LUSC	73	MALE	ASIAN	Stage IV	Lung Squamous Cell	2005	-26938	Dead	TUMOR FREE		716			Complete Remission/Response	1	716	0	716			0	716
TCGA-18-	TCGA-18-	LUSC	77	MALE		Stage IB	Lung Squamous Cell	2006	-28430	Dead	TUMOR FREE		2803			Complete Remission/Response	1	2803	0	2803	0	2803	0	2803
TCGA-18-	TCGA-18-	LUSC	77	MALE		Stage IB	Lung Squamous Cell	2006	-28430	Dead	TUMOR FREE		2803			Complete Remission/Response	1	2803	0	2803	0	2803	0	2803
TCGA-18-	TCGA-18-	LUSC	83	MALE		Stage IIB	Lung Squamous Cell	2006	-30435	Dead	WITH TUMOR		973	Distant Metastasis	940	Complete Remission/Response	1	973	1	973	1	940	1	940
TCGA-18-	TCGA-18-	LUSC	83	MALE		Stage IIB	Lung Squamous Cell	2006	-30435	Dead	WITH TUMOR		973	Distant Metastasis	940	Complete Remission/Response	1	973	1	973	1	940	1	940
TCGA-18-	TCGA-18-	LUSC	65	MALE		Stage IV	Lung Squamous Cell	2006	-24019	Dead	TUMOR FREE		1097			Complete Remission/Response	1	1097	0	1097			0	1097
TCGA-18-	TCGA-18-	LUSC	65	MALE		Stage IV	Lung Squamous Cell	2006	-24019	Dead	TUMOR FREE		1097			Complete Remission/Response	1	1097	0	1097			0	1097
TCGA-18-	TCGA-18-	LUSC	73	MALE		Stage IIB	Lung Squamous Cell	2007	-26813	Alive	TUMOR F	2811				Complete Remission/Response	0	2811	0	2811	0	2811	0	2811
TCGA-18-	TCGA-18-	LUSC	73	MALE		Stage IIB	Lung Squamous Cell	2007	-26813	Alive	TUMOR F	2811				Complete Remission/Response	0	2811	0	2811	0	2811	0	2811
TCGA-18-	TCGA-18-	LUSC	65	MALE	WHITE	Stage IB	Lung Squamous Cell	2007	-23972	Alive	TUMOR F	2645				Complete Remission/Response	0	2645	0	2645	0	2645	0	2645
TCGA-18-	TCGA-18-	LUSC	65	MALE	WHITE	Stage IB	Lung Squamous Cell	2007	-23972	Alive	TUMOR F	2645				Complete Remission/Response	0	2645	0	2645	0	2645	0	2645
TCGA-18-	TCGA-18-	LUSC	63	MALE	WHITE	Stage IIB	Lung Squamous Cell	2005	-23125	Dead	TUMOR FREE		188			[Unknown]	1	188	0	188			0	188
TCGA-18-	TCGA-18-	LUSC	64	MALE		Stage IB	Lung Squamous Cell	2009	-23731	Dead	TUMOR FREE		85				1	85	0	85			0	85
TCGA-18-	TCGA-18-	LUSC	74	MALE	WHITE	Stage IA	Lung Squamous Cell	2002	-27076	Alive	TUMOR F	4694				Complete Remission/Response	0	4694	0	4694	0	4694	0	4694
TCGA-18-	TCGA-18-	LUSC	74	MALE	WHITE	Stage IA	Lung Squamous Cell	2002	-27076	Alive	TUMOR F	4694				Complete Remission/Response	0	4694	0	4694	0	4694	0	4694
TCGA-18-	TCGA-18-	LUSC	57	MALE		Stage IIB	Lung Squamous Cell	2010	-21020	Alive	TUMOR F	1519				[Unknown]	0	1519	0	1519			0	1519
TCGA-18-	TCGA-18-	LUSC	57	MALE		Stage IIB	Lung Squamous Cell	2010	-21020	Alive	TUMOR F	1519				[Unknown]	0	1519	0	1519			0	1519
TCGA-18-	TCGA-18-	LUSC	50	MALE		Stage IB	Lung Squamous Cell	2008	-18611	Dead			827				1	827		827			0	827
TCGA-18-	TCGA-18-	LUSC	50	MALE		Stage IB	Lung Squamous Cell	2008	-18611	Dead			827				1	827		827			0	827
TCGA-21-	TCGA-21-	LUSC	60	FEMALE	BLACK OI	Stage IIIA	Lung Squamous Cell	2000	-22273	Alive	TUMOR F	3636					0	3636	0	3636			0	3636
TCGA-21-	TCGA-21-	LUSC	67	MALE	WHITE	Stage IB	Lung Squamous Cell	2001	-24515	Dead	WITH TUI	1426	1426		993		1	1426	1	1426			1	993
TCGA-21-	TCGA-21-	LUSC	75	MALE	WHITE	Stage IB	Lung Squamous Cell	2001	-27397	Alive	TUMOR F	3016					0	3016	0	3016			0	3016
TCGA-21-	TCGA-21-	LUSC	57	MALE	WHITE	Stage IIB	Lung Squamous Cell	2002	-21093	Alive	TUMOR F	2134					0	2134	0	2134			0	2134
TCGA-21-	TCGA-21-	LUSC	54	FEMALE	WHITE	Stage IB	Lung Squamous Cell	2005	-19863	Alive	TUMOR F	1852			1575		0	1852	0	1852			1	1575
TCGA-21-	TCGA-21-	LUSC	64	MALE	WHITE	Stage IIB	Lung Squamous Cell	2006	-23523	Dead	WITH TUI	1058	1058		644		1	1058	1	1058			1	644
TCGA-21-	TCGA-21-	LUSC	77	MALE	WHITE	Stage IB	Lung Squamous Cell	2006	-28125	Dead	WITH TUI	469	474		257		1	474	1	474			1	257
TCGA-21-	TCGA-21-	LUSC	71	MALE	WHITE	Stage IIIA	Lung Squamous Cell	1995	-26176	Dead	WITH TUI	965	965		317		1	965	1	965			1	317
TCGA-21-	TCGA-21-	LUSC	66	MALE	WHITE	Stage IB	Lung Squamous Cell	1996	-24253	Alive	TUMOR F	3724					0	3724	0	3724			0	3724
TCGA-21-	TCGA-21-	LUSC	69	MALE	WHITE	Stage IIB	Lung Squamous Cell	1996	-25440	Dead	WITH TUI	284	284				1	284	1	284			1	284
TCGA-21-	TCGA-21-	LUSC	61	MALE	WHITE	Stage IB	Lung Squamous Cell	1997	-22492	Alive	TUMOR F	3644					0	3644	0	3644			0	3644
TCGA-21-	TCGA-21-	LUSC	75	MALE	WHITE	Stage IA	Lung Squamous Cell	1997	-27708	Dead	TUMOR F	267	1315				1	1315	0	1315			0	1315
TCGA-21-	TCGA-21-	LUSC	68	FEMALE	WHITE	Stage IB	Lung Squamous Cell	2004	-24969	Dead		962	962			Complete Remission/Response	1	962		962	0	962	0	962
TCGA-21-	TCGA-21-	LUSC	76	MALE	WHITE	Stage IB	Lung Squamous Cell	2004	-27904	Dead			2680				1	2680		2680			0	2680
TCGA-21-	TCGA-21-	LUSC	80	FEMALE	WHITE	Stage IB	Lung Squamous Cell	2008	-29362	Alive	TUMOR F	1268				Complete Remission/Response	0	1268	0	1268	0	1268	0	1268
TCGA-21-	TCGA-21-	LUSC	64	MALE	WHITE	Stage IB	Lung Squamous Cell	2009	-23649	Alive	TUMOR F	1032		Locoregional Recurrence	817	Complete Remission/Response	0	1032	0	1032	1	817	1	817
TCGA-21-	TCGA-21-	LUSC	65	MALE	BLACK OI	Stage IIIA	Lung Squamous Cell	2009	-24071	Dead	WITH TUI	329	329		103	Progressive Disease	1	329	1	329			1	103
TCGA-21-	TCGA-21-	LUSC	77	MALE	WHITE	Stage IA	Lung Squamous Cell	2006	-28374	Alive	TUMOR F	979				Complete Remission/Response	0	979	0	979	0	979	0	979
TCGA-22-	TCGA-22-	LUSC	71	MALE	WHITE	Stage IIA	Lung Squamous Cell	2000	-26272	Dead	WITH TUI	608	669				1	669	1	669			1	669
TCGA-22-	TCGA-22-	LUSC	61	MALE	WHITE	Stage IB	Lung Squamous Cell	2000	-22329	Dead	TUMOR F	223	223				1	223	0	223			0	223
TCGA-22-	TCGA-22-	LUSC	76	MALE	WHITE	Stage IB	Lung Squamous Cell	2000	-27901	Dead	WITH TUMOR		454		263	Complete Remission/Response	1	454	1	454	1	263	1	263
TCGA-22-	TCGA-22-	LUSC	69	MALE	WHITE	Stage IA	Lung Squamous Cell	2001	-25359	Dead	TUMOR F	131	131				1	131	0	131			0	131
TCGA-22-	TCGA-22-	LUSC	63	MALE	WHITE	Stage IA	Lung Squamous Cell	2001	-23322	Dead	TUMOR F	1953	1953			Complete Remission/Response	1	1953	0	1953	0	1953	0	1953
TCGA-22-	TCGA-22-	LUSC	73	MALE	WHITE	Stage IB	Lung Squamous Cell	2001	-26732	Dead	TUMOR F	53	53				1	53	0	53			0	53
TCGA-22-	TCGA-22-	LUSC	80	FEMALE	WHITE	Stage IB	Lung Squamous Cell	2001	-29273	Dead	TUMOR FREE		429			Complete Remission/Response	1	429	0	429	0	429	0	429
TCGA-22-	TCGA-22-	LUSC	65	MALE	WHITE	Stage IB	Lung Squamous Cell	2001	-24078	Dead	TUMOR F	802	822			Complete Remission/Response	1	822	0	822	0	822	0	822
TCGA-22-	TCGA-22-	LUSC	62	MALE	WHITE	Stage IA	Lung Squamous Cell	1999	-22880	Dead	WITH TUMOR		1485	Locoregional Recurrence|Distan	904	Complete Remission/Response	1	1485	1	1485	1	904	1	904
TCGA-22-	TCGA-22-	LUSC	80	MALE	WHITE	Stage IIIA	Lung Squamous Cell	2001	-29250	Dead	WITH TUI	623	623		443	Partial Remission/Response	1	623	1	623			1	443
TCGA-22-	TCGA-22-	LUSC	77	MALE	WHITE	Stage IIA	Lung Squamous Cell	2002	-28385	Dead	TUMOR F	1067	1067				1	1067	0	1067			0	1067
TCGA-22-	TCGA-22-	LUSC	77	MALE	WHITE	Stage IIA	Lung Squamous Cell	2002	-28385	Dead	TUMOR F	1067	1067				1	1067	0	1067			0	1067
TCGA-22-	TCGA-22-	LUSC	60	FEMALE		Stage IIIA	Lung Squamous Cell	2002	-22115	Dead		1470	1470				1	1470		1470			0	1470
TCGA-22-	TCGA-22-	LUSC	57	MALE	WHITE	Stage IIIA	Lung Squamous Cell	2002	-21146	Dead	WITH TUI	734	734		250	Complete Remission/Response	1	734	1	734	1	250	1	250
TCGA-22-	TCGA-22-	LUSC	69	FEMALE	WHITE	Stage IB	Lung Squamous Cell	2002	-25448	Dead	TUMOR F	17	17				1	17	0	17			0	17
TCGA-22-	TCGA-22-	LUSC	73	FEMALE	WHITE	Stage IB	Lung Squamous Cell	2002	-26925	Dead		1161	1161				1	1161		1161			0	1161
TCGA-22-	TCGA-22-	LUSC	73	FEMALE	WHITE	Stage IB	Lung Squamous Cell	2002	-26925	Dead		1161	1161				1	1161		1161			0	1161
TCGA-22-	TCGA-22-	LUSC	73	FEMALE	WHITE	Stage IIIA	Lung Squamous Cell	2003	-26937	Dead	WITH TUI	1057	1057		525		1	1057	1	1057			1	525
TCGA-22-	TCGA-22-	LUSC	73	FEMALE	WHITE	Stage IIIA	Lung Squamous Cell	2003	-26937	Dead	WITH TUI	1057	1057		525		1	1057	1	1057			1	525
TCGA-22-	TCGA-22-	LUSC	73	MALE	WHITE	Stage IIA	Lung Squamous Cell	2003	-26952	Dead	WITH TUMOR		399		306		1	399	1	399			1	306
TCGA-22-	TCGA-22-	LUSC	78	FEMALE		Stage IB	Lung Squamous Cell	2003	-28623	Dead	TUMOR FREE		974			Complete Remission/Response	1	974	0	974	0	974	0	974
TCGA-22-	TCGA-22-	LUSC	75	MALE	WHITE	Stage IB	Lung Squamous Cell	2003	-27601	Dead	TUMOR F	587	587				1	587	0	587			0	587
TCGA-22-	TCGA-22-	LUSC	81	MALE	WHITE	Stage IA	Lung Squamous Cell	2003	-29844	Dead	TUMOR FREE		291			Complete Remission/Response	1	291	0	291	0	291	0	291
TCGA-22-	TCGA-22-	LUSC	81	MALE	WHITE	Stage IA	Lung Squamous Cell	2003	-29844	Dead	TUMOR FREE		291			Complete Remission/Response	1	291	0	291	0	291	0	291
TCGA-22-	TCGA-22-	LUSC	73	FEMALE	WHITE	Stage IA	Lung Squamous Cell	2004	-26722	Dead		358	358				1	358		358			0	358
TCGA-22-	TCGA-22-	LUSC	73	FEMALE	WHITE	Stage IA	Lung Squamous Cell	2004	-26722	Dead		358	358				1	358		358			0	358
TCGA-22-	TCGA-22-	LUSC	75	MALE	WHITE	Stage IB	Lung Squamous Cell	2005	-27749	Alive		1845			229	Complete Remission/Response	0	1845	0	1845	1	229	1	229
TCGA-22-	TCGA-22-	LUSC	75	MALE	WHITE	Stage IB	Lung Squamous Cell	2005	-27749	Alive		1845			229	Complete Remission/Response	0	1845	0	1845	1	229	1	229
TCGA-22-	TCGA-22-	LUSC	67	MALE	WHITE	Stage IB	Lung Squamous Cell	2005	-24482	Dead	TUMOR FREE		1975		746	Complete Remission/Response	1	1975	0	1975	1	746	1	746
TCGA-22-	TCGA-22-	LUSC	67	MALE	WHITE	Stage IB	Lung Squamous Cell	2005	-24482	Dead	TUMOR FREE		1975		746	Complete Remission/Response	1	1975	0	1975	1	746	1	746
TCGA-22-	TCGA-22-	LUSC	78	MALE	WHITE	[Discrepancy]	Lung Squamous Cell	2005	-28826	Dead	TUMOR FREE		1933		1655	Complete Remission/Response	1	1933	0	1933	1	1655	1	1655
TCGA-22-	TCGA-22-	LUSC	78	MALE	WHITE	[Discrepancy]	Lung Squamous Cell	2005	-28826	Dead	TUMOR FREE		1933		1655	Complete Remission/Response	1	1933	0	1933	1	1655	1	1655
TCGA-22-	TCGA-22-	LUSC	74	MALE	WHITE	Stage IB	Lung Squamous Cell	2005	-27271	Dead	TUMOR F	445	445			Complete Remission/Response	1	445	0	445	0	445	0	445
TCGA-22-	TCGA-22-	LUSC	74	MALE	WHITE	Stage IB	Lung Squamous Cell	2005	-27271	Dead	TUMOR F	445	445			Complete Remission/Response	1	445	0	445	0	445	0	445
TCGA-22-	TCGA-22-	LUSC	65	MALE	WHITE	Stage IA	Lung Squamous Cell	2004	-24055	Dead		1346	1346		511	Complete Remission/Response	1	1346		1346	1	511	1	511
TCGA-22-	TCGA-22-	LUSC	65	MALE	WHITE	Stage IA	Lung Squamous Cell	2004	-24055	Dead		1346	1346		511	Complete Remission/Response	1	1346		1346	1	511	1	511
TCGA-22-	TCGA-22-	LUSC	79	MALE		Stage IB	Lung Squamous Cell	2004	-28978	Dead	TUMOR F	24	24			Complete Remission/Response	1	24	0	24	0	24	0	24
TCGA-22-	TCGA-22-	LUSC	79	MALE		Stage IB	Lung Squamous Cell	2004	-28978	Dead	TUMOR F	24	24			Complete Remission/Response	1	24	0	24	0	24	0	24
TCGA-22-	TCGA-22-	LUSC	64	MALE	WHITE	Stage IB	Lung Squamous Cell	2004	-23563	Dead			2625			[Unknown]	1	2625		2625			0	2625
TCGA-22-	TCGA-22-	LUSC	66	FEMALE	WHITE	Stage IA	Lung Squamous Cell	2002	-24245	Dead	WITH TUI	2170	2170		1779	Complete Remission/Response	1	2170	1	2170	1	1779	1	1779
TCGA-22-	TCGA-22-	LUSC	66	FEMALE	WHITE	Stage IA	Lung Squamous Cell	2002	-24245	Dead	WITH TUI	2170	2170		1779	Complete Remission/Response	1	2170	1	2170	1	1779	1	1779
TCGA-22-	TCGA-22-	LUSC	72	FEMALE	WHITE	Stage IIB	Lung Squamous Cell	2002	-26419	Dead			2409		346	Complete Remission/Response	1	2409		2409	1	346	1	346
TCGA-22-	TCGA-22-	LUSC	72	FEMALE	WHITE	Stage IIB	Lung Squamous Cell	2002	-26419	Dead			2409		346	Complete Remission/Response	1	2409		2409	1	346	1	346
TCGA-22-	TCGA-22-	LUSC	81	MALE	WHITE	Stage IB	Lung Squamous Cell	2005	-29657	Dead	TUMOR F	357	357			Complete Remission/Response	1	357	0	357	0	357	0	357
TCGA-22-	TCGA-22-	LUSC	81	MALE	WHITE	Stage IB	Lung Squamous Cell	2005	-29657	Dead	TUMOR F	357	357			Complete Remission/Response	1	357	0	357	0	357	0	357
TCGA-22-	TCGA-22-	LUSC	74	MALE	WHITE	Stage IIA	Lung Squamous Cell	2005	-27109	Dead	WITH TUI	573	573		568	Complete Remission/Response	1	573	1	573	1	568	1	568
TCGA-22-	TCGA-22-	LUSC	74	MALE	WHITE	Stage IIA	Lung Squamous Cell	2005	-27109	Dead	WITH TUI	573	573		568	Complete Remission/Response	1	573	1	573	1	568	1	568
TCGA-22-	TCGA-22-	LUSC	58	FEMALE	WHITE	Stage IA	Lung Squamous Cell	2001	-21488	Dead	WITH TUI	916	916		840	Complete Remission/Response	1	916	1	916	1	840	1	840
TCGA-22-	TCGA-22-	LUSC	58	FEMALE	WHITE	Stage IA	Lung Squamous Cell	2001	-21488	Dead	WITH TUI	916	916		840	Complete Remission/Response	1	916	1	916	1	840	1	840
TCGA-22-	TCGA-22-	LUSC	64	MALE	WHITE	Stage IA	Lung Squamous Cell	2003	-23618	Dead	WITH TUI	1912	1912		428	Complete Remission/Response	1	1912	1	1912	1	428	1	428
TCGA-22-	TCGA-22-	LUSC	64	MALE	WHITE	Stage IA	Lung Squamous Cell	2003	-23618	Dead	WITH TUI	1912	1912		428	Complete Remission/Response	1	1912	1	1912	1	428	1	428
TCGA-22-	TCGA-22-	LUSC	74	MALE	WHITE	Stage IA	Lung Squamous Cell	2003	-27145	Dead		1713	1713				1	1713		1713			0	1713
TCGA-22-	TCGA-22-	LUSC	74	MALE	WHITE	Stage IA	Lung Squamous Cell	2003	-27145	Dead		1713	1713				1	1713		1713			0	1713
TCGA-22-	TCGA-22-	LUSC	73	FEMALE		Stage IIIA	Lung Squamous Cell	2003	-26838	Dead		506	506				1	506		506			0	506
TCGA-22-	TCGA-22-	LUSC	73	FEMALE		Stage IIIA	Lung Squamous Cell	2003	-26838	Dead		506	506				1	506		506			0	506
TCGA-22-	TCGA-22-	LUSC	70	MALE	WHITE	Stage IIA	Lung Squamous Cell	2012	-25629	Alive	TUMOR F	671				Complete Remission/Response	0	671	0	671	0	671	0	671
TCGA-33-	TCGA-33-	LUSC	68	MALE	WHITE	Stage IB	Lung Squamous Cell	2001	-25169	Dead	TUMOR FREE		3924			Complete Remission/Response	1	3924	0	3924	0	3924	0	3924
TCGA-33-	TCGA-33-	LUSC	76	FEMALE	BLACK OI	Stage IB	Lung Squamous Cell	2001	-28001	Alive	TUMOR F	4068				Complete Remission/Response	0	4068	0	4068	0	4068	0	4068
TCGA-33-	TCGA-33-	LUSC	66	MALE	WHITE	Stage IIIA	Lung Squamous Cell	2002	-24174	Dead			2979			Complete Remission/Response	1	2979		2979	0	2979	0	2979
TCGA-33-	TCGA-33-	LUSC	68	MALE	WHITE	Stage IB	Lung Squamous Cell	2007	-25056	Alive	TUMOR F	2419				Complete Remission/Response	0	2419	0	2419	0	2419	0	2419
TCGA-33-	TCGA-33-	LUSC	40	MALE	WHITE	Stage IB	Lung Squamous Cell	1996	-14643	Dead			5287			[Unknown]	1	5287		5287			0	5287
TCGA-33-	TCGA-33-	LUSC	40	MALE	WHITE	Stage IB	Lung Squamous Cell	1996	-14643	Dead			5287			[Unknown]	1	5287		5287			0	5287
TCGA-33-	TCGA-33-	LUSC	55	MALE	WHITE	Stage IA	Lung Squamous Cell	2000	-20402	Dead	WITH TUMOR		3149	Locoregional Recurrence	2923	Complete Remission/Response	1	3149	1	3149	1	2923	1	2923
TCGA-33-	TCGA-33-	LUSC	55	MALE	WHITE	Stage IA	Lung Squamous Cell	2000	-20402	Dead	WITH TUMOR		3149	Locoregional Recurrence	2923	Complete Remission/Response	1	3149	1	3149	1	2923	1	2923
TCGA-33-	TCGA-33-	LUSC	73	MALE	WHITE	Stage IA	Lung Squamous Cell	1999	-26799	Dead	TUMOR FREE		4601			Complete Remission/Response	1	4601	0	4601	0	4601	0	4601
TCGA-33-	TCGA-33-	LUSC	73	MALE	WHITE	Stage IA	Lung Squamous Cell	1999	-26799	Dead	TUMOR FREE		4601			Complete Remission/Response	1	4601	0	4601	0	4601	0	4601
TCGA-33-	TCGA-33-	LUSC	57	MALE	WHITE	Stage IIIA	Lung Squamous Cell	2007	-21148	Dead			428	Locoregional Recurrence	169	Complete Remission/Response	1	428		428	1	169	1	169
TCGA-33-	TCGA-33-	LUSC	57	MALE	WHITE	Stage IIIA	Lung Squamous Cell	2007	-21148	Dead			428	Locoregional Recurrence	169	Complete Remission/Response	1	428		428	1	169	1	169
TCGA-33-	TCGA-33-	LUSC	63	FEMALE	WHITE	Stage IB	Lung Squamous Cell	2003	-23062	Dead	WITH TUMOR		1656			Progressive Disease	1	1656	1	1656			1	1656
TCGA-33-	TCGA-33-	LUSC	63	FEMALE	WHITE	Stage IB	Lung Squamous Cell	2003	-23062	Dead	WITH TUMOR		1656			Progressive Disease	1	1656	1	1656			1	1656
TCGA-33-	TCGA-33-	LUSC	62	FEMALE	WHITE	Stage IIB	Lung Squamous Cell	1992	-22754	Dead	TUMOR FREE		47			Complete Remission/Response	1	47	0	47	0	47	0	47
TCGA-33-	TCGA-33-	LUSC	62	FEMALE	WHITE	Stage IIB	Lung Squamous Cell	1992	-22754	Dead	TUMOR FREE		47			Complete Remission/Response	1	47	0	47	0	47	0	47
TCGA-33-	TCGA-33-	LUSC	71	MALE	WHITE	Stage IIIA	Lung Squamous Cell	2008	-26033	Dead	WITH TUMOR		601			Progressive Disease	1	601	1	601			1	601
TCGA-33-	TCGA-33-	LUSC	71	MALE	WHITE	Stage IIIA	Lung Squamous Cell	2008	-26033	Dead	WITH TUMOR		601			Progressive Disease	1	601	1	601			1	601
TCGA-33-	TCGA-33-	LUSC	80	MALE	WHITE	Stage IIIA	Lung Squamous Cell	2009	-29368	Alive	TUMOR F	1927				Complete Remission/Response	0	1927	0	1927	0	1927	0	1927
TCGA-33-	TCGA-33-	LUSC	60	MALE	WHITE	Stage IB	Lung Squamous Cell	2011	-22268	Dead	TUMOR FREE		143			Complete Remission/Response	1	143	0	143	0	143	0	143
TCGA-33-	TCGA-33-	LUSC	67	MALE	WHITE	Stage IIA	Lung Squamous Cell	2012	-24586	Dead	TUMOR FREE		9			Complete Remission/Response	1	9	0	9	0	9	0	9
TCGA-33-	TCGA-33-	LUSC	59	FEMALE	BLACK OI	Stage IA	Lung Squamous Cell	1998	-21675	Dead	TUMOR FREE		1114			Complete Remission/Response	1	1114	0	1114	0	1114	0	1114
TCGA-33-	TCGA-33-	LUSC	66	MALE	BLACK OI	Stage IB	Lung Squamous Cell	2002	-24183	Dead	WITH TUMOR		211	Distant Metastasis	30	Progressive Disease	1	211	1	211			1	30
TCGA-33-	TCGA-33-	LUSC	83	MALE	BLACK OI	Stage IA	Lung Squamous Cell	2001	-30623	Dead	WITH TUMOR		3376	Locoregional Recurrence	1912	Progressive Disease	1	3376	1	3376			1	1912
TCGA-33-	TCGA-33-	LUSC	65	FEMALE	BLACK OI	Stage IIB	Lung Squamous Cell	2008	-23965	Dead	WITH TUMOR		1344			Stable Disease	1	1344	1	1344			1	1344
TCGA-33-	TCGA-33-	LUSC	60	MALE	BLACK OI	Stage IB	Lung Squamous Cell	1999	-22179	Dead	WITH TUMOR		3600	Locoregional Recurrence	1135	Complete Remission/Response	1	3600	1	3600	1	1135	1	1135
TCGA-33-	TCGA-33-	LUSC	57	FEMALE	BLACK OI	Stage IA	Lung Squamous Cell	2000	-20940	Dead	WITH TUMOR		826	Locoregional Recurrence	149	Progressive Disease	1	826	1	826			1	149
TCGA-34-	TCGA-34-	LUSC	70	MALE	WHITE	Stage IIB	Lung Squamous Cell	2004	-25574	Dead	TUMOR F	80	80			Complete Remission/Response	1	80	0	80	0	80	0	80
TCGA-34-	TCGA-34-	LUSC	76	FEMALE	WHITE	Stage IA	Lung Squamous Cell	2000	-28072	Dead	TUMOR F	1874	1874			Complete Remission/Response	1	1874	0	1874	0	1874	0	1874
TCGA-34-	TCGA-34-	LUSC	81	FEMALE	WHITE	Stage IB	Lung Squamous Cell	1999	-29827	Dead	TUMOR F	958	958			Complete Remission/Response	1	958	0	958	0	958	0	958
TCGA-34-	TCGA-34-	LUSC	76	MALE	WHITE	Stage IIIB	Lung Squamous Cell	2006	-27833	Dead	WITH TUI	881	881		394	Complete Remission/Response	1	881	1	881	1	394	1	394
TCGA-34-	TCGA-34-	LUSC	84	MALE	WHITE	Stage IB	Lung Squamous Cell	1999	-30958	Dead	TUMOR F	1000	1000			Complete Remission/Response	1	1000	0	1000	0	1000	0	1000
TCGA-34-	TCGA-34-	LUSC	81	MALE	BLACK OI	Stage IIB	Lung Squamous Cell	2004	-29871	Dead	WITH TUI	951	951		387	Complete Remission/Response	1	951	1	951	1	387	1	387
TCGA-34-	TCGA-34-	LUSC	72	MALE	WHITE	Stage IA	Lung Squamous Cell	2006	-26525	Dead	TUMOR FREE		1984			Stable Disease	1	1984	0	1984			0	1984
TCGA-34-	TCGA-34-	LUSC	75	FEMALE	BLACK OI	Stage IIA	Lung Squamous Cell	2006	-27633	Alive	TUMOR F	2471				Complete Remission/Response	0	2471	0	2471	0	2471	0	2471
TCGA-34-	TCGA-34-	LUSC	71	FEMALE	WHITE	Stage IA	Lung Squamous Cell	2007	-26072	Alive	TUMOR F	2271				Complete Remission/Response	0	2271	0	2271	0	2271	0	2271
TCGA-34-	TCGA-34-	LUSC	60	MALE	WHITE	Stage IIB	Lung Squamous Cell	2007	-21935	Dead	WITH TUMOR		276	Locoregional Recurrence	113	Progressive Disease	1	276	1	276			1	113
TCGA-34-	TCGA-34-	LUSC	75	MALE	WHITE	Stage IIIA	Lung Squamous Cell	2010	-27528	Alive	WITH TUI	1834		Distant Metastasis	1530	Complete Remission/Response	0	1834	0	1834	1	1530	1	1530
TCGA-34-	TCGA-34-	LUSC	73	FEMALE	WHITE	Stage IIB	Lung Squamous Cell	2009	-26735	Alive	TUMOR F	1541				Complete Remission/Response	0	1541	0	1541	0	1541	0	1541
TCGA-34-	TCGA-34-	LUSC	79	MALE	WHITE	Stage IB	Lung Squamous Cell	2009	-29098	Dead	TUMOR FREE		515			Stable Disease	1	515	0	515			0	515
TCGA-34-	TCGA-34-	LUSC	70	FEMALE	WHITE	Stage IA	Lung Squamous Cell	2009	-25857	Alive	TUMOR F	1361		Distant Metastasis	245	[Unknown]	0	1361	0	1361			1	245
TCGA-34-	TCGA-34-	LUSC	83	FEMALE	WHITE	Stage IIB	Lung Squamous Cell	2010	-30334	Alive	TUMOR F	1196				Complete Remission/Response	0	1196	0	1196	0	1196	0	1196
TCGA-34-	TCGA-34-	LUSC	78	FEMALE	WHITE	Stage IB	Lung Squamous Cell	2010	-28844	Dead	TUMOR FREE		151			Stable Disease	1	151	0	151			0	151
TCGA-34-	TCGA-34-	LUSC	78	FEMALE	WHITE	Stage IB	Lung Squamous Cell	2010	-28844	Dead	TUMOR FREE		151			Stable Disease	1	151	0	151			0	151
TCGA-34-	TCGA-34-	LUSC	70	MALE	WHITE	Stage II	Lung Squamous Cell	2011	-25677	Dead			34				1	34		34			0	34
TCGA-34-	TCGA-34-	LUSC	70	MALE	WHITE	Stage II	Lung Squamous Cell	2011	-25677	Dead			34				1	34		34			0	34
TCGA-34-	TCGA-34-	LUSC	62	FEMALE	WHITE	Stage IIIA	Lung Squamous Cell	2011	-22913	Alive	TUMOR F	1180				Complete Remission/Response	0	1180	0	1180	0	1180	0	1180
TCGA-34-	TCGA-34-	LUSC	62	FEMALE	WHITE	Stage IIIA	Lung Squamous Cell	2011	-22913	Alive	TUMOR F	1180				Complete Remission/Response	0	1180	0	1180	0	1180	0	1180
TCGA-34-	TCGA-34-	LUSC	67	MALE	WHITE	Stage IV	Lung Squamous Cell	2011	-24624	Dead	WITH TUMOR		123			Progressive Disease	1	123	1	123			1	123
TCGA-34-	TCGA-34-	LUSC	71	FEMALE	WHITE	Stage IIA	Lung Squamous Cell	2011	-26183	Alive	TUMOR F	804				Complete Remission/Response	0	804	0	804	0	804	0	804
TCGA-34-	TCGA-34-	LUSC	71	FEMALE	WHITE	Stage IIA	Lung Squamous Cell	2011	-26183	Alive	TUMOR F	804				Complete Remission/Response	0	804	0	804	0	804	0	804
TCGA-34-	TCGA-34-	LUSC	80	MALE	WHITE	Stage IIB	Lung Squamous Cell	2012	-29391	Alive	TUMOR F	1031				Complete Remission/Response	0	1031	0	1031	0	1031	0	1031
TCGA-37-	TCGA-37-	LUSC	51	MALE	WHITE	Stage IIIA	Lung Squamous Cell	2006	-18728	Alive	TUMOR F	122					0	122	0	122			0	122
TCGA-37-	TCGA-37-	LUSC	65	MALE	WHITE	Stage IB	Lung Squamous Cell	2006	-23782	Alive	TUMOR F	13					0	13	0	13			0	13
TCGA-37-	TCGA-37-	LUSC	69	MALE	WHITE	Stage IB	Lung Squamous Cell	2006	-25502	Alive	TUMOR F	12					0	12	0	12			0	12
TCGA-37-	TCGA-37-	LUSC	52	FEMALE	WHITE	Stage IA	Lung Squamous Cell	2010	-19154	Alive	TUMOR F	242				Complete Remission/Response	0	242	0	242	0	242	0	242
TCGA-37-	TCGA-37-	LUSC	56	MALE	WHITE	Stage IA	Lung Squamous Cell	2010	-20652	Alive	TUMOR F	247					0	247	0	247			0	247
TCGA-37-	TCGA-37-	LUSC	61	FEMALE	WHITE	Stage IV	Lung Squamous Cell	2010	-22520	Alive	TUMOR F	227				Complete Remission/Response	0	227	0	227			0	227
TCGA-37-	TCGA-37-	LUSC	63	MALE	WHITE	Stage IIIA	Lung Squamous Cell	2010	-23083	Alive	TUMOR F	238					0	238	0	238			0	238
TCGA-37-	TCGA-37-	LUSC	68	MALE	WHITE	Stage IB	Lung Squamous Cell	2010	-25041	Alive	TUMOR F	207					0	207	0	207			0	207
TCGA-37-	TCGA-37-	LUSC	65	FEMALE	WHITE	Stage IA	Lung Squamous Cell	2010	-24048	Alive	TUMOR F	12					0	12	0	12			0	12
TCGA-37-	TCGA-37-	LUSC	64	MALE	WHITE	Stage IIIA	Lung Squamous Cell	2010	-23726	Alive	TUMOR F	103					0	103	0	103			0	103
TCGA-37-	TCGA-37-	LUSC	53	MALE	WHITE	Stage IIB	Lung Squamous Cell	2011	-19371	Dead	WITH TUMOR		1143	Distant Metastasis	946	[Discrepancy]	1	1143	1	1143			1	946
TCGA-37-	TCGA-37-	LUSC	49	MALE	WHITE	Stage II	Lung Squamous Cell	2011	-18011	Alive	TUMOR F	867				Complete Remission/Response	0	867	0	867	0	867	0	867
TCGA-37-	TCGA-37-	LUSC	59	MALE	WHITE	Stage IIIB	Lung Squamous Cell	2011	-21600	Alive	TUMOR F	660				Complete Remission/Response	0	660	0	660	0	660	0	660
TCGA-39-	TCGA-39-	LUSC	70	FEMALE	WHITE	Stage IA	Lung Squamous Cell	2002	-25788	Alive	TUMOR F	4053			565	Complete Remission/Response	0	4053	0	4053	1	565	1	565
TCGA-39-	TCGA-39-	LUSC	70	FEMALE	WHITE	Stage IA	Lung Squamous Cell	2002	-25788	Alive	TUMOR F	4053			565	Complete Remission/Response	0	4053	0	4053	1	565	1	565
TCGA-39-	TCGA-39-	LUSC	44	MALE	WHITE	Stage IIA	Lung Squamous Cell	2003	-16306	Alive	TUMOR F	3850				Complete Remission/Response	0	3850	0	3850	0	3850	0	3850
TCGA-39-	TCGA-39-	LUSC	44	MALE	WHITE	Stage IIA	Lung Squamous Cell	2003	-16306	Alive	TUMOR F	3850				Complete Remission/Response	0	3850	0	3850	0	3850	0	3850
TCGA-39-	TCGA-39-	LUSC	70	MALE	WHITE	Stage IB	Lung Squamous Cell	2004	-25756	Alive	TUMOR F	3387			378	Complete Remission/Response	0	3387	0	3387	1	378	1	378
TCGA-39-	TCGA-39-	LUSC	70	MALE	WHITE	Stage IB	Lung Squamous Cell	2004	-25756	Alive	TUMOR F	3387			378	Complete Remission/Response	0	3387	0	3387	1	378	1	378
TCGA-39-	TCGA-39-	LUSC	70	MALE	WHITE	Stage IB	Lung Squamous Cell	2004	-25895	Dead	WITH TUI	2057	2086		1875	Complete Remission/Response	1	2086	1	2086	1	1875	1	1875
TCGA-39-	TCGA-39-	LUSC	70	MALE	WHITE	Stage IB	Lung Squamous Cell	2004	-25895	Dead	WITH TUI	2057	2086		1875	Complete Remission/Response	1	2086	1	2086	1	1875	1	1875
TCGA-39-	TCGA-39-	LUSC	76	MALE	WHITE	Stage IB	Lung Squamous Cell	2005	-27856	Dead	TUMOR FREE		1679		1448		1	1679	0	1679			1	1448
TCGA-39-	TCGA-39-	LUSC	65	FEMALE	WHITE	Stage IIIA	Lung Squamous Cell	2005	-23975	Alive	TUMOR F	2510				Complete Remission/Response	0	2510	0	2510	0	2510	0	2510
TCGA-39-	TCGA-39-	LUSC	73	MALE	WHITE	Stage IB	Lung Squamous Cell	2006	-26714	Alive	WITH TUI	3108		New Primary Tumor	3081	Complete Remission/Response	0	3108	0	3108	0	3108	1	3081
TCGA-39-	TCGA-39-	LUSC	75	MALE	WHITE	Stage IIIA	Lung Squamous Cell	2006	-27707	Dead		52	52				1	52		52			0	52
TCGA-39-	TCGA-39-	LUSC	75	MALE	WHITE	Stage IIIA	Lung Squamous Cell	2006	-27707	Dead		52	52				1	52		52			0	52
TCGA-39-	TCGA-39-	LUSC	67	MALE	WHITE	Stage IIIA	Lung Squamous Cell	2006	-24713	Dead	WITH TUMOR		740		491	Stable Disease	1	740	1	740			1	491
TCGA-39-	TCGA-39-	LUSC	67	MALE	WHITE	Stage IIIA	Lung Squamous Cell	2006	-24713	Dead	WITH TUMOR		740		491	Stable Disease	1	740	1	740			1	491
TCGA-39-	TCGA-39-	LUSC	81	FEMALE	ASIAN	Stage IIIA	Lung Squamous Cell	2006	-29893	Dead		59	59			Complete Remission/Response	1	59		59	0	59	0	59
TCGA-39-	TCGA-39-	LUSC	81	FEMALE	ASIAN	Stage IIIA	Lung Squamous Cell	2006	-29893	Dead		59	59			Complete Remission/Response	1	59		59	0	59	0	59
TCGA-39-	TCGA-39-	LUSC	76	FEMALE		Stage IA	Lung Squamous Cell	2007	-28106	Dead	TUMOR FREE		1841			Complete Remission/Response	1	1841	0	1841	0	1841	0	1841
TCGA-39-	TCGA-39-	LUSC	76	FEMALE		Stage IA	Lung Squamous Cell	2007	-28106	Dead	TUMOR FREE		1841			Complete Remission/Response	1	1841	0	1841	0	1841	0	1841
TCGA-39-	TCGA-39-	LUSC	73	FEMALE	WHITE	Stage IIB	Lung Squamous Cell	2007	-26682	Dead		1107	1107		352	Complete Remission/Response	1	1107		1107	1	352	1	352
TCGA-39-	TCGA-39-	LUSC	73	FEMALE	WHITE	Stage IIB	Lung Squamous Cell	2007	-26682	Dead		1107	1107		352	Complete Remission/Response	1	1107		1107	1	352	1	352
TCGA-39-	TCGA-39-	LUSC	72	FEMALE	WHITE	Stage IA	Lung Squamous Cell	2007	-26383	Alive	TUMOR F	2080				Complete Remission/Response	0	2080	0	2080	0	2080	0	2080
TCGA-39-	TCGA-39-	LUSC	72	FEMALE	WHITE	Stage IA	Lung Squamous Cell	2007	-26383	Alive	TUMOR F	2080				Complete Remission/Response	0	2080	0	2080	0	2080	0	2080
TCGA-39-	TCGA-39-	LUSC	73	MALE	WHITE	Stage IB	Lung Squamous Cell	2007	-26963	Alive	TUMOR F	2165				Complete Remission/Response	0	2165	0	2165	0	2165	0	2165
TCGA-39-	TCGA-39-	LUSC	73	MALE	WHITE	Stage IB	Lung Squamous Cell	2007	-26963	Alive	TUMOR F	2165				Complete Remission/Response	0	2165	0	2165	0	2165	0	2165
TCGA-39-	TCGA-39-	LUSC	65	MALE	WHITE	Stage IIA	Lung Squamous Cell	2007	-23868	Alive	TUMOR F	1690				Complete Remission/Response	0	1690	0	1690	0	1690	0	1690
TCGA-39-	TCGA-39-	LUSC	65	MALE	WHITE	Stage IIA	Lung Squamous Cell	2007	-23868	Alive	TUMOR F	1690				Complete Remission/Response	0	1690	0	1690	0	1690	0	1690
TCGA-39-	TCGA-39-	LUSC	76	MALE	WHITE	Stage IIA	Lung Squamous Cell	2008	-27781	Dead	TUMOR F	544	544			Complete Remission/Response	1	544	0	544	0	544	0	544
TCGA-39-	TCGA-39-	LUSC	76	MALE	WHITE	Stage IIA	Lung Squamous Cell	2008	-27781	Dead	TUMOR F	544	544			Complete Remission/Response	1	544	0	544	0	544	0	544
TCGA-39-	TCGA-39-	LUSC	59	MALE	WHITE	Stage IIIA	Lung Squamous Cell	2008	-21790	Dead	WITH TUMOR		519		442	Stable Disease	1	519	1	519			1	442
TCGA-39-	TCGA-39-	LUSC	59	MALE	WHITE	Stage IIIA	Lung Squamous Cell	2008	-21790	Dead	WITH TUMOR		519		442	Stable Disease	1	519	1	519			1	442
TCGA-43-	TCGA-43-	LUSC	62	FEMALE	WHITE	Stage IIIA	Lung Squamous Cell	2009	-22805	Alive	TUMOR F	1223				Complete Remission/Response	0	1223	0	1223	0	1223	0	1223
TCGA-43-	TCGA-43-	LUSC	59	FEMALE	WHITE	Stage IA	Lung Squamous Cell	2009	-21747	Dead	TUMOR FREE		684			Complete Remission/Response	1	684	0	684	0	684	0	684
TCGA-43-	TCGA-43-	LUSC	47	FEMALE	WHITE	Stage IIIA	Lung Squamous Cell	2009	-17447	Alive	TUMOR F	1176				Complete Remission/Response	0	1176	0	1176	0	1176	0	1176
TCGA-43-	TCGA-43-	LUSC	52	MALE	BLACK OI	Stage IB	Lung Squamous Cell	2009	-19310	Dead	WITH TUMOR		1190		552	Complete Remission/Response	1	1190	1	1190	1	552	1	552
TCGA-43-	TCGA-43-	LUSC	52	MALE	BLACK OI	Stage IB	Lung Squamous Cell	2009	-19310	Dead	WITH TUMOR		1190		552	Complete Remission/Response	1	1190	1	1190	1	552	1	552
TCGA-43-	TCGA-43-	LUSC	71	MALE	WHITE	Stage IB	Lung Squamous Cell	2009	-26059	Alive	TUMOR F	1007				Complete Remission/Response	0	1007	0	1007	0	1007	0	1007
TCGA-43-	TCGA-43-	LUSC	71	MALE	WHITE	Stage IB	Lung Squamous Cell	2009	-26059	Alive	TUMOR F	1007				Complete Remission/Response	0	1007	0	1007	0	1007	0	1007
TCGA-43-	TCGA-43-	LUSC	78	MALE	WHITE	Stage IIA	Lung Squamous Cell	2010	-28635	Dead	WITH TUMOR		559		470	Complete Remission/Response	1	559	1	559	1	470	1	470
TCGA-43-	TCGA-43-	LUSC	78	MALE	WHITE	Stage IIA	Lung Squamous Cell	2010	-28635	Dead	WITH TUMOR		559		470	Complete Remission/Response	1	559	1	559	1	470	1	470
TCGA-43-	TCGA-43-	LUSC	70	MALE	WHITE	Stage IIA	Lung Squamous Cell	2010	-25652	Alive	TUMOR F	849				Complete Remission/Response	0	849	0	849	0	849	0	849
TCGA-43-	TCGA-43-	LUSC	70	MALE	WHITE	Stage IIA	Lung Squamous Cell	2010	-25652	Alive	TUMOR F	849				Complete Remission/Response	0	849	0	849	0	849	0	849
TCGA-43-	TCGA-43-	LUSC	70	MALE	WHITE	Stage IB	Lung Squamous Cell	2010	-25820	Alive	TUMOR F	699				Complete Remission/Response	0	699	0	699	0	699	0	699
TCGA-43-	TCGA-43-	LUSC	70	MALE	WHITE	Stage IB	Lung Squamous Cell	2010	-25820	Alive	TUMOR F	699				Complete Remission/Response	0	699	0	699	0	699	0	699
TCGA-43-	TCGA-43-	LUSC	69	FEMALE	WHITE	Stage IIB	Lung Squamous Cell	2010	-25378	Alive	TUMOR F	757				Complete Remission/Response	0	757	0	757	0	757	0	757
TCGA-43-	TCGA-43-	LUSC	69	FEMALE	WHITE	Stage IIB	Lung Squamous Cell	2010	-25378	Alive	TUMOR F	757				Complete Remission/Response	0	757	0	757	0	757	0	757

TCGA-43-	TCGA-43-	LUSC	59	FEMALE	BLACK OI	Stage IB	Lung Squamous Cell	2011	-21683	Alive	TUMOR F	653				Complete Remission/Response	0	653	0	653	0	653	0	653
TCGA-43-	TCGA-43-	LUSC	85	MALE	WHITE	Stage IB	Lung Squamous Cell	2006	-31052	Dead	WITH TUMOR		166		154	Progressive Disease	1	166	1	166			1	154
TCGA-43-	TCGA-43-	LUSC	85	MALE	WHITE	Stage IB	Lung Squamous Cell	2006	-31052	Dead	WITH TUMOR		166		154	Progressive Disease	1	166	1	166			1	154
TCGA-43-	TCGA-43-	LUSC	76	MALE	WHITE	Stage IIB	Lung Squamous Cell	2008	-28036	Dead	TUMOR FREE		116				1	116	0	116			0	116
TCGA-43-	TCGA-43-	LUSC	76	MALE	WHITE	Stage IIB	Lung Squamous Cell	2008	-28036	Dead	TUMOR FREE		116				1	116	0	116			0	116
TCGA-43-	TCGA-43-	LUSC	71	MALE	WHITE	Stage IA	Lung Squamous Cell	2011	-25991	Alive	TUMOR F	596				Complete Remission/Response	0	596	0	596	0	596	0	596
TCGA-43-	TCGA-43-	LUSC	68	FEMALE	WHITE	Stage IA	Lung Squamous Cell	2011	-25042	Alive	TUMOR F	236				Complete Remission/Response	0	236	0	236	0	236	0	236
TCGA-43-	TCGA-43-	LUSC	68	FEMALE	WHITE	Stage IA	Lung Squamous Cell	2011	-25042	Alive	TUMOR F	236				Complete Remission/Response	0	236	0	236	0	236	0	236
TCGA-43-	TCGA-43-	LUSC	75	FEMALE	WHITE	Stage IA	Lung Squamous Cell	2006	-27734	Dead	WITH TUMOR		2378	Distant Metastasis|New Primary	2305	Complete Remission/Response	1	2378	1	2378	1	2305	1	2305
TCGA-43-	TCGA-43-	LUSC	75	FEMALE	WHITE	Stage IA	Lung Squamous Cell	2006	-27734	Dead	WITH TUMOR		2378	Distant Metastasis|New Primarv	2305	Complete Remission/Response	1	2378	1	2378	1	2305	1	2305
TCGA-43-	TCGA-43-	LUSC	72	FEMALE	WHITE	Stage IIA	Lung Squamous Cell	2011	-26406	Alive	TUMOR F	407				Stable Disease	0	407	0	407			0	407
TCGA-43-	TCGA-43-	LUSC	73	MALE	WHITE	Stage IA	Lung Squamous Cell	2011	-26790	Alive	TUMOR F	358				Complete Remission/Response	0	358	0	358	0	358	0	358
TCGA-43-	TCGA-43-	LUSC	55	FEMALE	WHITE	Stage IA	Lung Squamous Cell	2011	-20224	Dead			89			[Unknown]	1	89		89			0	89
TCGA-43-	TCGA-43-	LUSC	66	MALE	WHITE	Stage IIA	Lung Squamous Cell	2012	-24286	Alive	TUMOR F	353				Complete Remission/Response	0	353	0	353	0	353	0	353
TCGA-43-	TCGA-43-	LUSC	67	FEMALE	WHITE	Stage IIB	Lung Squamous Cell	2012	-24680	Alive	TUMOR F	296				Complete Remission/Response	0	296	0	296	0	296	0	296
TCGA-43-	TCGA-43-	LUSC	76	FEMALE	WHITE	Stage IA	Lung Squamous Cell	2012	-27847	Alive	TUMOR F	432				Complete Remission/Response	0	432	0	432	0	432	0	432
TCGA-43-	TCGA-43-	LUSC	61	MALE	BLACK OI	Stage IIIA	Lung Squamous Cell	2012	-22475	Alive	WITH TUN	366		Locoregional Recurrence|Distan	355	Complete Remission/Response	0	366	0	366	1	355	1	355
TCGA-46-	TCGA-46-	LUSC	59	FEMALE	WHITE	Stage IA	Lung Squamous Cell	2009	-21762	Alive	TUMOR F	405				Complete Remission/Response	0	405	0	405	0	405	0	405
TCGA-46-	TCGA-46-	LUSC	62	FEMALE	WHITE	Stage IA	Lung Squamous Cell	2009	-22707	Alive	TUMOR F	370				Complete Remission/Response	0	370	0	370	0	370	0	370
TCGA-46-	TCGA-46-	LUSC	76	MALE	WHITE	Stage IA	Lung Squamous Cell	2009	-27912	Alive	TUMOR F	396				Complete Remission/Response	0	396	0	396	0	396	0	396
TCGA-46-	TCGA-46-	LUSC	58	MALE	WHITE	Stage IIIA	Lung Squamous Cell	2010	-21307	Dead	TUMOR F	4	299			Complete Remission/Response	1	299	0	299	0	299	0	299
TCGA-46-	TCGA-46-	LUSC	57	MALE	WHITE	[Discrepancy]	Lung Squamous Cell	2009	-20941	Alive	TUMOR F	135				Complete Remission/Response	0	135	0	135	0	135	0	135
TCGA-46-	TCGA-46-	LUSC	71	MALE	WHITE	Stage IIB	Lung Squamous Cell	2010	-26168	Alive	TUMOR F	324				Complete Remission/Response	0	324	0	324	0	324	0	324
TCGA-46-	TCGA-46-	LUSC	81	MALE	WHITE	Stage IIB	Lung Squamous Cell	2010	-29890	Alive	TUMOR F	423				Complete Remission/Response	0	423	0	423	0	423	0	423
TCGA-51-	TCGA-51-	LUSC	73	FEMALE	BLACK OI	Stage IB	Lung Squamous Cell	2009	-26899	Dead			12				1	12		12			0	12
TCGA-51-	TCGA-51-	LUSC	73	FEMALE	BLACK OI	Stage IB	Lung Squamous Cell	2009	-26899	Dead			12				1	12		12			0	12
TCGA-51-	TCGA-51-	LUSC	65	MALE	BLACK OI	Stage IIIB	Lung Squamous Cell	2009	-24054	Dead	TUMOR FREE		12				1	12	0	12			0	12
TCGA-51-	TCGA-51-	LUSC	65	MALE	BLACK OI	Stage IIIB	Lung Squamous Cell	2009	-24054	Dead	TUMOR FREE		12				1	12	0	12			0	12
TCGA-51-	TCGA-51-	LUSC	55	MALE	WHITE	Stage IIB	Lung Squamous Cell	2009	-20261	Alive	TUMOR F	911					0	911	0	911			0	911
TCGA-51-	TCGA-51-	LUSC	55	MALE	WHITE	Stage IIB	Lung Squamous Cell	2009	-20261	Alive	TUMOR F	911					0	911	0	911			0	911
TCGA-51-	TCGA-51-	LUSC	72	FEMALE	WHITE	Stage I	Lung Squamous Cell	2003	-26523	Dead	WITH TUMOR		1856		788	Complete Remission/Response	1	1856	1	1856	1	788	1	788
TCGA-52-	TCGA-52-	LUSC	62	FEMALE	WHITE	Stage IA	Lung Squamous Cell	2010	-22807	Alive	TUMOR F	862				Complete Remission/Response	0	862	0	862	0	862	0	862
TCGA-52-	TCGA-52-	LUSC	74	MALE	WHITE	Stage IB	Lung Squamous Cell	2009	-27233	Dead	WITH TUMOR		166			Progressive Disease	1	166	1	166			1	166
TCGA-52-	TCGA-52-	LUSC	60	FEMALE	WHITE	Stage IIB	Lung Squamous Cell	2008	-22241	Alive	TUMOR F	923				Complete Remission/Response	0	923	0	923	0	923	0	923
TCGA-52-	TCGA-52-	LUSC	67	MALE	WHITE	Stage IB	Lung Squamous Cell	2009	-24782	Dead	WITH TUMOR		266	Distant Metastasis	153	Progressive Disease	1	266	1	266			1	153
TCGA-52-	TCGA-52-	LUSC	68	MALE	WHITE	[Discrepancy]	Lung Squamous Cell	2009	-24887	Dead	WITH TUMOR		835	Distant Metastasis	791	Progressive Disease	1	835	1	835			1	791
TCGA-56-	TCGA-56-	LUSC	58	MALE	WHITE	Stage IB	Lung Squamous Cell	2007	-21371	Dead	WITH TUMOR		881			Complete Remission/Response	1	881	1	881			1	881
TCGA-56-	TCGA-56-	LUSC	74	MALE	WHITE	Stage IA	Lung Squamous Cell	2010	-27118	Alive	TUMOR F	378				Complete Remission/Response	0	378	0	378	0	378	0	378
TCGA-56-	TCGA-56-	LUSC	69	MALE	WHITE	Stage IA	Lung Squamous Cell	2010	-25441	Alive	TUMOR F	555				Complete Remission/Response	0	555	0	555	0	555	0	555
TCGA-56-	TCGA-56-	LUSC	77	FEMALE	WHITE	Stage IB	Lung Squamous Cell	2010	-28400	Alive	TUMOR F	666				Complete Remission/Response	0	666	0	666	0	666	0	666
TCGA-56-	TCGA-56-	LUSC	67	MALE	WHITE	Stage IIA	Lung Squamous Cell	2011	-24594	Alive	TUMOR FREE						0		0				0	
TCGA-56-	TCGA-56-	LUSC	79	MALE	WHITE	Stage IB	Lung Squamous Cell	2011	-28873	Alive	TUMOR F	608				Complete Remission/Response	0	608	0	608	0	608	0	608
TCGA-56-	TCGA-56-	LUSC	60	MALE	WHITE	Stage IB	Lung Squamous Cell	2011	-22097	Dead	WITH TUMOR		562		427	Complete Remission/Response	1	562	1	562	1	427	1	427
TCGA-56-	TCGA-56-	LUSC	60	MALE	WHITE	Stage IB	Lung Squamous Cell	2011	-22097	Dead	WITH TUMOR		562		427	Complete Remission/Response	1	562	1	562	1	427	1	427
TCGA-56-	TCGA-56-	LUSC	66	MALE	WHITE	Stage IIIA	Lung Squamous Cell	2011	-24288	Dead	WITH TUMOR		442	Locoregional Recurrence	132	Progressive Disease	1	442	1	442			1	132
TCGA-56-	TCGA-56-	LUSC	61	MALE	WHITE	Stage IIIA	Lung Squamous Cell	2011	-22632	Dead	WITH TUMOR		951	Locoregional Recurrence	149	Progressive Disease	1	951	1	951			1	149
TCGA-56-	TCGA-56-	LUSC	61	MALE	WHITE	Stage IIIA	Lung Squamous Cell	2011	-22632	Dead	WITH TUMOR		951	Locoregional Recurrence	149	Progressive Disease	1	951	1	951			1	149
TCGA-56-	TCGA-56-	LUSC	84	MALE	WHITE	Stage IB	Lung Squamous Cell	2011	-30740	Alive	TUMOR F	925				Complete Remission/Response	0	925	0	925	0	925	0	925
TCGA-56-	TCGA-56-	LUSC	84	MALE	WHITE	Stage IB	Lung Squamous Cell	2011	-30740	Alive	TUMOR F	925				Complete Remission/Response	0	925	0	925	0	925	0	925
TCGA-56-	TCGA-56-	LUSC	83	MALE	WHITE	Stage IB	Lung Squamous Cell	2011	-30678	Alive	TUMOR F	601				Complete Remission/Response	0	601	0	601	0	601	0	601
TCGA-56-	TCGA-56-	LUSC	83	MALE	WHITE	Stage IB	Lung Squamous Cell	2011	-30678	Alive	TUMOR F	601				Complete Remission/Response	0	601	0	601	0	601	0	601
TCGA-56-	TCGA-56-	LUSC	73	MALE	WHITE	Stage IIA	Lung Squamous Cell	2011	-26699	Dead	WITH TUMOR		198			Progressive Disease	1	198	1	198			1	198
TCGA-56-	TCGA-56-	LUSC	73	MALE	WHITE	Stage IIA	Lung Squamous Cell	2011	-26699	Dead	WITH TUMOR		198			Progressive Disease	1	198	1	198			1	198
TCGA-56-	TCGA-56-	LUSC	66	FEMALE	WHITE	Stage IB	Lung Squamous Cell	2011	-24441	Dead	TUMOR FREE		3				1	3	0	3			0	3
TCGA-56-	TCGA-56-	LUSC	66	FEMALE	WHITE	Stage IB	Lung Squamous Cell	2011	-24441	Dead	TUMOR FREE		3				1	3	0	3			0	3
TCGA-56-	TCGA-56-	LUSC	75	MALE	BLACK OI	Stage IIB	Lung Squamous Cell	2011	-27755	Dead	WITH TUMOR		532		301	[Unknown]	1	532	1	532			1	301
TCGA-56-	TCGA-56-	LUSC	58	FEMALE	WHITE	Stage IIA	Lung Squamous Cell	2011	-21344	Alive	TUMOR F	1011				Complete Remission/Response	0	1011	0	1011	0	1011	0	1011
TCGA-56-	TCGA-56-	LUSC	58	FEMALE	WHITE	Stage IIA	Lung Squamous Cell	2011	-21344	Alive	TUMOR F	1011				Complete Remission/Response	0	1011	0	1011	0	1011	0	1011
TCGA-56-	TCGA-56-	LUSC	80	FEMALE	WHITE	Stage IIA	Lung Squamous Cell	2011	-29440	Alive	TUMOR F	455				Complete Remission/Response	0	455	0	455	0	455	0	455
TCGA-56-	TCGA-56-	LUSC	80	FEMALE	WHITE	Stage IIA	Lung Squamous Cell	2011	-29440	Alive	TUMOR F	455				Complete Remission/Response	0	455	0	455	0	455	0	455
TCGA-56-	TCGA-56-	LUSC	56	MALE	WHITE	Stage IB	Lung Squamous Cell	2011	-20696	Alive	TUMOR F	150				[Unknown]	0	150	0	150			0	150
TCGA-56-	TCGA-56-	LUSC	56	MALE	WHITE	Stage IB	Lung Squamous Cell	2011	-20696	Alive	TUMOR F	150				[Unknown]	0	150	0	150			0	150
TCGA-56-	TCGA-56-	LUSC	74	MALE	WHITE	Stage IIB	Lung Squamous Cell	2011	-27211	Dead	WITH TUMOR		397			[Unknown]	1	397	1	397			1	397
TCGA-56-	TCGA-56-	LUSC	74	MALE	WHITE	Stage IIB	Lung Squamous Cell	2011	-27211	Dead	WITH TUMOR		397			[Unknown]	1	397	1	397			1	397
TCGA-56-	TCGA-56-	LUSC	73	FEMALE	WHITE	Stage IA	Lung Squamous Cell	2011	-26803	Alive	TUMOR F	106				Complete Remission/Response	0	106	0	106	0	106	0	106
TCGA-56-	TCGA-56-	LUSC	72	MALE	WHITE	Stage IB	Lung Squamous Cell	2011	-26428	Alive	TUMOR F	105				Complete Remission/Response	0	105	0	105	0	105	0	105
TCGA-56-	TCGA-56-	LUSC	55	FEMALE	WHITE	Stage IIB	Lung Squamous Cell	2012	-20411	Alive	TUMOR F	818				Complete Remission/Response	0	818	0	818	0	818	0	818
TCGA-56-	TCGA-56-	LUSC	79	MALE	WHITE	Stage IIB	Lung Squamous Cell	2012	-29139	Alive	WITH TUN	517				Stable Disease	0	517	0	517			0	517
TCGA-56-	TCGA-56-	LUSC	66	MALE	WHITE	Stage IA	Lung Squamous Cell	2012	-24382	Alive	TUMOR F	428				Complete Remission/Response	0	428	0	428	0	428	0	428
TCGA-56-	TCGA-56-	LUSC	66	MALE	WHITE	Stage IA	Lung Squamous Cell	2012	-24382	Alive	TUMOR F	428				Complete Remission/Response	0	428	0	428	0	428	0	428
TCGA-56-	TCGA-56-	LUSC	76	FEMALE	WHITE	Stage IIB	Lung Squamous Cell	2012	-28095	Alive	TUMOR F	41					0	41	0	41			0	41
TCGA-56-	TCGA-56-	LUSC	74	MALE	WHITE	Stage IB	Lung Squamous Cell	2012	-27210	Alive	TUMOR F	510				Complete Remission/Response	0	510	0	510	0	510	0	510
TCGA-56-	TCGA-56-	LUSC	74	MALE	WHITE	Stage IB	Lung Squamous Cell	2012	-27210	Alive	TUMOR F	510				Complete Remission/Response	0	510	0	510	0	510	0	510
TCGA-56-	TCGA-56-	LUSC	68	MALE	WHITE	Stage IB	Lung Squamous Cell	2012	-24861	Alive	TUMOR F	55					0	55	0	55			0	55
TCGA-56-	TCGA-56-	LUSC	71	MALE	WHITE	Stage IB	Lung Squamous Cell	2012	-26231	Dead	TUMOR FREE		692	New Primary Tumor	645	Complete Remission/Response	1	692	0	692	0	692	1	645
TCGA-56-	TCGA-56-	LUSC	71	MALE	WHITE	Stage IB	Lung Squamous Cell	2012	-26231	Dead	TUMOR FREE		692	New Primary Tumor	645	Complete Remission/Response	1	692	0	692	0	692	1	645
TCGA-56-	TCGA-56-	LUSC	84	MALE	WHITE	Stage IIB	Lung Squamous Cell	2012	-30798	Alive	TUMOR F	420				Complete Remission/Response	0	420	0	420	0	420	0	420
TCGA-56-	TCGA-56-	LUSC	84	MALE	WHITE	Stage IIB	Lung Squamous Cell	2012	-30798	Alive	TUMOR F	420				Complete Remission/Response	0	420	0	420	0	420	0	420
TCGA-56-	TCGA-56-	LUSC	66	FEMALE	WHITE	Stage IIIA	Lung Squamous Cell	2012	-24188	Dead	WITH TUMOR		315	Distant Metastasis	272	Progressive Disease	1	315	1	315			1	272
TCGA-56-	TCGA-56-	LUSC	59	MALE	WHITE	Stage IA	Lung Squamous Cell	2012	-21908	Dead	TUMOR FREE		302			Complete Remission/Response	1	302	0	302	0	302	0	302
TCGA-56-	TCGA-56-	LUSC	78	MALE	WHITE	Stage IA	Lung Squamous Cell	2012	-28805	Alive	TUMOR F	616				Complete Remission/Response	0	616	0	616	0	616	0	616
TCGA-56-	TCGA-56-	LUSC	63	MALE	WHITE	Stage IIA	Lung Squamous Cell	2012	-23346	Alive	TUMOR F	481				Complete Remission/Response	0	481	0	481	0	481	0	481
TCGA-56-	TCGA-56-	LUSC	63	MALE	WHITE	Stage IIA	Lung Squamous Cell	2012	-23346	Alive	TUMOR F	481				Complete Remission/Response	0	481	0	481	0	481	0	481
TCGA-56-	TCGA-56-	LUSC	67	MALE	WHITE	Stage IIIA	Lung Squamous Cell	2012	-24745	Alive	TUMOR F	637				Complete Remission/Response	0	637	0	637	0	637	0	637
TCGA-56-	TCGA-56-	LUSC	55	MALE	WHITE	Stage IIA	Lung Squamous Cell	2012	-20301	Alive	TUMOR F	585				Complete Remission/Response	0	585	0	585	0	585	0	585
TCGA-56-	TCGA-56-	LUSC	70	MALE	WHITE	Stage IIA	Lung Squamous Cell	2012	-25745	Alive	TUMOR F	405				Complete Remission/Response	0	405	0	405	0	405	0	405
TCGA-56-	TCGA-56-	LUSC	66	MALE	WHITE	Stage IB	Lung Squamous Cell	2012	-24445	Dead	WITH TUMOR		543			Complete Remission/Response	1	543	1	543			1	543
TCGA-56-	TCGA-56-	LUSC	75	FEMALE	WHITE	Stage IA	Lung Squamous Cell	2012	-27516	Alive	TUMOR F	640				Complete Remission/Response	0	640	0	640	0	640	0	640
TCGA-56-	TCGA-56-	LUSC	76	FEMALE	WHITE	Stage IB	Lung Squamous Cell	2012	-28039	Alive	TUMOR F	570				Complete Remission/Response	0	570	0	570	0	570	0	570
TCGA-56-	TCGA-56-	LUSC	81	MALE	WHITE	Stage IA	Lung Squamous Cell	2012	-29828	Alive	TUMOR F	4				Complete Remission/Response	0	4	0	4	0	4	0	4
TCGA-56-	TCGA-56-	LUSC	72	FEMALE	WHITE	Stage IB	Lung Squamous Cell	2012	-26355	Alive	TUMOR F	8				Complete Remission/Response	0	8	0	8	0	8	0	8
TCGA-56-	TCGA-56-	LUSC	78	MALE	BLACK OI	Stage IIA	Lung Squamous Cell	2013	-28522	Alive	TUMOR F	440				Complete Remission/Response	0	440	0	440	0	440	0	440
TCGA-58-	TCGA-58-	LUSC	75	MALE	[Not Evalu	Stage IV	Lung Squamous Cell	2008	-27455	Dead	TUMOR FREE		1			Complete Remission/Response	1	1	0	1			0	1
TCGA-58-	TCGA-58-	LUSC	75	MALE	[Not Evalu	Stage IV	Lung Squamous Cell	2008	-27455	Dead	TUMOR FREE		1			Complete Remission/Response	1	1	0	1			0	1
TCGA-58-	TCGA-58-	LUSC	60	MALE	WHITE	Stage IIA	Lung Squamous Cell	2010	-21952	Dead	TUMOR FREE		403			Complete Remission/Response	1	403	0	403	0	403	0	403
TCGA-58-	TCGA-58-	LUSC	60	MALE	WHITE	Stage IB	Lung Squamous Cell	2010	-21949	Dead	TUMOR FREE		412			Complete Remission/Response	1	412	0	412	0	412	0	412
TCGA-58-	TCGA-58-	LUSC	70	MALE	WHITE	Stage IIA	Lung Squamous Cell	2010	-25827	Alive	TUMOR F	911				Complete Remission/Response	0	911	0	911	0	911	0	911
TCGA-58-	TCGA-58-	LUSC	57	FEMALE	WHITE	Stage IIIA	Lung Squamous Cell	2006	-21102	Alive	WITH TUN	2167		Distant Metastasis|New Primarv	2033	Complete Remission/Response	0	2167	0	2167	1	2033	1	2033
TCGA-58-	TCGA-58-	LUSC	70	MALE	WHITE	Stage IB	Lung Squamous Cell	2010	-25710	Dead	WITH TUMOR		501	Distant Metastasis	96	Complete Remission/Response	1	501	1	501	1	96	1	96
TCGA-58-	TCGA-58-	LUSC	68	FEMALE	WHITE	Stage IB	Lung Squamous Cell	2010	-25132	Alive	TUMOR F	1058				Complete Remission/Response	0	1058	0	1058	0	1058	0	1058
TCGA-58-	TCGA-58-	LUSC	68	FEMALE	WHITE	Stage IB	Lung Squamous Cell	2010	-25132	Alive	TUMOR F	1058				Complete Remission/Response	0	1058	0	1058	0	1058	0	1058
TCGA-58-	TCGA-58-	LUSC	64	MALE	WHITE	Stage IIB	Lung Squamous Cell	2006	-23653	Alive	TUMOR F	2589				Complete Remission/Response	0	2589	0	2589	0	2589	0	2589
TCGA-58-	TCGA-58-	LUSC	59	MALE	WHITE	Stage IIIA	Lung Squamous Cell	2008	-21867	Dead	WITH TUMOR		1045	Locoregional Recurrence|Distan	735	Complete Remission/Response	1	1045	1	1045	1	735	1	735
TCGA-58-	TCGA-58-	LUSC	73	MALE	WHITE	Stage IIIA	Lung Squamous Cell	2008	-26685	Alive	TUMOR F	1723				Complete Remission/Response	0	1723	0	1723	0	1723	0	1723
TCGA-58-	TCGA-58-	LUSC	61	MALE	WHITE	Stage IIB	Lung Squamous Cell	2010	-22592	Alive	TUMOR F	1072				Complete Remission/Response	0	1072	0	1072	0	1072	0	1072
TCGA-58-	TCGA-58-	LUSC	52	MALE	WHITE	Stage IB	Lung Squamous Cell	2011	-19050	Alive	WITH TUN	910		Locoregional Recurrence	630	Complete Remission/Response	0	910	0	910	1	630	1	630
TCGA-60-	TCGA-60-	LUSC	74	FEMALE	WHITE	Stage IB	Lung Squamous Cell	2009	-27211	Alive	TUMOR F	642				Complete Remission/Response	0	642	0	642	0	642	0	642
TCGA-60-	TCGA-60-	LUSC	76	FEMALE	BLACK OI	Stage IIA	Lung Squamous Cell	2009	-27991	Dead		109	109				1	109		109			0	109
TCGA-60-	TCGA-60-	LUSC	41	MALE	WHITE	Stage IIA	Lung Squamous Cell	1992	-14984	Dead	WITH TUMOR		372		200	Complete Remission/Response	1	372	1	372	1	200	1	200
TCGA-60-	TCGA-60-	LUSC	62	MALE	WHITE	Stage IIB	Lung Squamous Cell	1993	-22800	Dead	WITH TUMOR		311		114		1	311	1	311			1	114
TCGA-60-	TCGA-60-	LUSC	73	MALE	WHITE	Stage IIB	Lung Squamous Cell	2002	-27003	Dead			2945		1757	Complete Remission/Response	1	2945		2945	1	1757	1	1757
TCGA-60-	TCGA-60-	LUSC	73	MALE	WHITE	Stage IIB	Lung Squamous Cell	2003	-26927	Dead	WITH TUMOR		1154		532		1	1154	1	1154			1	532
TCGA-60-	TCGA-60-	LUSC	58	MALE	WHITE	Stage IA	Lung Squamous Cell	2003	-21477	Alive	TUMOR F	2820				Complete Remission/Response	0	2820	0	2820	0	2820	0	2820
TCGA-60-	TCGA-60-	LUSC	70	MALE		Stage IB	Lung Squamous Cell	2003	-25758	Dead	WITH TUMOR		667		579	Complete Remission/Response	1	667	1	667	1	579	1	579
TCGA-60-	TCGA-60-	LUSC	64	FEMALE	WHITE	Stage IIB	Lung Squamous Cell	2004	-23691	Alive	TUMOR F	2447				Complete Remission/Response	0	2447	0	2447	0	2447	0	2447
TCGA-60-	TCGA-60-	LUSC	69	MALE	BLACK OI	Stage IB	Lung Squamous Cell	2004	-25453	Alive	TUMOR F	1505				Complete Remission/Response	0	1505	0	1505	0	1505	0	1505
TCGA-60-	TCGA-60-	LUSC	69	MALE	BLACK OI	Stage IB	Lung Squamous Cell	2004	-25453	Alive	TUMOR F	1505				Complete Remission/Response	0	1505	0	1505	0	1505	0	1505
TCGA-60-	TCGA-60-	LUSC	67	FEMALE	WHITE	Stage IIA	Lung Squamous Cell	2005	-24669	Alive	TUMOR F	2024				Complete Remission/Response	0	2024	0	2024	0	2024	0	2024
TCGA-60-	TCGA-60-	LUSC	64	FEMALE	WHITE	Stage IB	Lung Squamous Cell	2007	-23651	Alive	TUMOR F	1260				Complete Remission/Response	0	1260	0	1260	0	1260	0	1260
TCGA-60-	TCGA-60-	LUSC	79	FEMALE	WHITE	Stage IIB	Lung Squamous Cell	2006	-29131	Dead		274	274			Complete Remission/Response	1	274		274	0	274	0	274
TCGA-60-	TCGA-60-	LUSC	64	MALE	WHITE	Stage IB	Lung Squamous Cell	2006	-23630	Alive		1731			1356	Complete Remission/Response	0	1731	0	1731	1	1356	1	1356
TCGA-60-	TCGA-60-	LUSC	66	FEMALE	WHITE	Stage IIB	Lung Squamous Cell	2006	-24169	Alive	TUMOR F	1531				Complete Remission/Response	0	1531	0	1531	0	1531	0	1531
TCGA-60-	TCGA-60-	LUSC	51	MALE	WHITE	Stage IA	Lung Squamous Cell	2008	-18990	Dead	WITH TUMOR		1075			Progressive Disease	1	1075	1	1075			1	1075
TCGA-60-	TCGA-60-	LUSC	39	MALE		Stage IIB	Lung Squamous Cell	2007	-14345	Alive	TUMOR F	1475				Complete Remission/Response	0	1475	0	1475	0	1475	0	1475
TCGA-60-	TCGA-60-	LUSC	83	FEMALE	WHITE	Stage IB	Lung Squamous Cell	2007	-30379	Alive	TUMOR F	1297				Complete Remission/Response	0	1297	0	1297	0	1297	0	1297
TCGA-60-	TCGA-60-	LUSC	60	FEMALE	WHITE	Stage IB	Lung Squamous Cell	2007	-22238	Alive	TUMOR F	97					0	97	0	97			0	97
TCGA-60-	TCGA-60-	LUSC	73	MALE	WHITE	Stage IB	Lung Squamous Cell	2008	-27007	Alive	TUMOR F	983				Complete Remission/Response	0	983	0	983	0	983	0	983
TCGA-60-	TCGA-60-	LUSC	66	MALE	WHITE	Stage IIB	Lung Squamous Cell	2008	-24267	Alive	TUMOR F	908			851	Complete Remission/Response	0	908	0	908	1	851	1	851
TCGA-60-	TCGA-60-	LUSC	74	FEMALE	WHITE	Stage IB	Lung Squamous Cell	2008	-27225	Alive	TUMOR F	1092				Complete Remission/Response	0	1092	0	1092	0	1092	0	1092
TCGA-60-	TCGA-60-	LUSC	47	MALE	WHITE	Stage IIIA	Lung Squamous Cell	2008	-17462	Alive	TUMOR F	717					0	717	0	717			0	717
TCGA-60-	TCGA-60-	LUSC	74	MALE	WHITE	Stage IB	Lung Squamous Cell	2009	-27073	Alive	TUMOR F	816				Complete Remission/Response	0	816	0	816	0	816	0	816
TCGA-60-	TCGA-60-	LUSC	56	MALE	WHITE	Stage IIA	Lung Squamous Cell	2009	-20618	Dead	TUMOR F	358	358			Complete Remission/Response	1	358	0	358	0	358	0	358
TCGA-63-	TCGA-63-	LUSC		MALE		Stage IB	Lung Squamous Cell	2007		Dead	WITH TUMOR					Complete Remission/Response	1		1				1	
TCGA-63-	TCGA-63-	LUSC		MALE		Stage IIB	Lung Squamous Cell	2008		Dead	TUMOR FREE					Complete Remission/Response	1		0		0		0	
TCGA-63-	TCGA-63-	LUSC		MALE		Stage IIA	Lung Squamous Cell	2008		Alive	TUMOR F	1602				Complete Remission/Response	0	1602	0	1602	0	1602	0	1602
TCGA-63-	TCGA-63-	LUSC		MALE		Stage IA	Lung Squamous Cell	2007		Alive	TUMOR F	2133				Complete Remission/Response	0	2133	0	2133	0	2133	0	2133
TCGA-63-	TCGA-63-	LUSC		MALE		Stage IA	Lung Squamous Cell	2007		Alive	TUMOR F	2142		New Primary Tumor	1503	Complete Remission/Response	0	2142	0	2142	0	2142	1	1503
TCGA-63-	TCGA-63-	LUSC		FEMALE		Stage IA	Lung Squamous Cell	2007		Alive	TUMOR F	2073				Complete Remission/Response	0	2073	0	2073	0	2073	0	2073
TCGA-63-	TCGA-63-	LUSC		MALE		Stage IIA	Lung Squamous Cell	2008		Alive	TUMOR F	1682				Complete Remission/Response	0	1682	0	1682	0	1682	0	1682
TCGA-63-	TCGA-63-	LUSC		FEMALE	[Not Evalu	Stage IIB	Lung Squamous Cell Carcinoma			Alive		0				[Unknown]	0	0	0	0			0	0
TCGA-63-	TCGA-63-	LUSC	62	MALE	[Not Evalu	Stage IB	Lung Squamous Cell Carcinoma		-22930	Alive	TUMOR F	3123				Complete Remission/Response	0	3123	0	3123	0	3123	0	3123
TCGA-63-	TCGA-63-	LUSC	68	MALE	[Not Evalu	Stage IB	Lung Squamous Cell Carcinoma		-24957	Alive	TUMOR F	2148				Complete Remission/Response	0	2148	0	2148	0	2148	0	2148
TCGA-63-	TCGA-63-	LUSC	68	MALE	[Not Evalu	Stage IA	Lung Squamous Cell Carcinoma		-25159	Alive	TUMOR F	2026				Complete Remission/Response	0	2026	0	2026	0	2026	0	2026
TCGA-63-	TCGA-63-	LUSC	65	MALE	[Not Evalu	Stage IIIA	Lung Squamous Cell Carcinoma		-24040	Alive	TUMOR F	1784				Complete Remission/Response	0	1784	0	1784	0	1784	0	1784
TCGA-63-	TCGA-63-	LUSC	54	MALE	[Not Evalu	Stage IIB	Lung Squamous Cell Carcinoma		-20018	Alive	TUMOR F	1824				Complete Remission/Response	0	1824	0	1824	0	1824	0	1824
TCGA-63-	TCGA-63-	LUSC	68	MALE	[Not Evalu	Stage IB	Lung Squamous Cell Carcinoma		-24947	Alive	TUMOR F	1386				Complete Remission/Response	0	1386	0	1386	0	1386	0	1386
TCGA-63-	TCGA-63-	LUSC	69	FEMALE	[Not Evalu	Stage IIB	Lung Squamous Cell Carcinoma		-25348	Dead	WITH TUMOR		456		218	Complete Remission/Response	1	456	1	456	1	218	1	218
TCGA-63-	TCGA-63-	LUSC	78	FEMALE	[Not Evalu	Stage IIB	Lung Squamous Cell Carcinoma		-28677	Dead	WITH TUMOR		345		320	Complete Remission/Response	1	345	1	345	1	320	1	320
TCGA-63-	TCGA-63-	LUSC	56	MALE	[Not Evalu	Stage IIB	Lung Squamous Cell Carcinoma		-20818	Alive	WITH TUN	769		Locoregional Recurrence	511	Complete Remission/Response	0	769	0	769	1	511	1	511
TCGA-63-	TCGA-63-	LUSC	70	FEMALE	[Not Evalu	Stage IB	Lung Squamous Cell Carcinoma		-25879	Alive	TUMOR F	2716				Complete Remission/Response	0	2716	0	2716	0	2716	0	2716
TCGA-63-	TCGA-63-	LUSC	78	MALE	[Not Evalu	Stage IB	Lung Squamous Cell Carcinoma		-28827	Alive	TUMOR F	2381				Complete Remission/Response	0	2381	0	2381	0	2381	0	2381
TCGA-63-	TCGA-63-	LUSC	74	MALE	[Not Evalu	Stage IIB	Lung Squamous Cell Carcinoma		-27263	Alive	WITH TUN	498		Distant Metastasis	427	Complete Remission/Response	0	498	0	498	1	427	1	427
TCGA-63-	TCGA-63-	LUSC	48	MALE	[Not Evalu	Stage IIB	Lung Squamous Cell Carcinoma		-17801	Dead	WITH TUMOR			Distant Metastasis	420	Complete Remission/Response	1		1		1	420	1	420
TCGA-63-	TCGA-63-	LUSC	69	MALE	[Not Evalu	Stage IIA	Lung Squamous Cell Carcinoma		-25505	Alive	TUMOR F	1100				Complete Remission/Response	0	1100	0	1100	0	1100	0	1100
TCGA-63-	TCGA-63-	LUSC	76	MALE	[Not Evalu	Stage IB	Lung Squamous Cell Carcinoma		-27892	Alive	TUMOR F	1639				Complete Remission/Response	0	1639	0	1639	0	1639	0	1639
TCGA-63-	TCGA-63-	LUSC	63	MALE	[Not Evalu	Stage IA	Lung Squamous Cell Carcinoma		-23276	Alive	TUMOR F	1052				Complete Remission/Response	0	1052	0	1052	0	1052	0	1052
TCGA-66-	TCGA-66-	LUSC	55	FEMALE		Stage IB	Lung Squamous Cell	2004	-20150	Dead			516				1	516		516			0	516
TCGA-66-	TCGA-66-	LUSC	55	FEMALE		Stage IB	Lung Squamous Cell	2004	-20150	Dead			516				1	516		516			0	516
TCGA-66-	TCGA-66-	LUSC	62	FEMALE		Stage IB	Lung Squamous Cell	2005	-22919	Alive	TUMOR F	1311					0	1311	0	1311			0	1311
TCGA-66-	TCGA-66-	LUSC	62	FEMALE		Stage IB	Lung Squamous Cell	2005	-22919	Alive	TUMOR F	1311					0	1311	0	1311			0	1311
TCGA-66-	TCGA-66-	LUSC	72	MALE		Stage IIB	Lung Squamous Cell	2005	-26390	Alive	TUMOR F	61					0	61	0	61			0	61
TCGA-66-	TCGA-66-	LUSC	72	MALE		Stage IIB	Lung Squamous Cell	2005	-26390	Alive	TUMOR F	61					0	61	0	61			0	61
TCGA-66-	TCGA-66-	LUSC	70	MALE		Stage IV	Lung Squamous Cell	2006	-25688	Alive	TUMOR F	641					0	641	0	641			0	641
TCGA-66-	TCGA-66-	LUSC	70	MALE		Stage IV	Lung Squamous Cell	2006	-25688	Alive	TUMOR F	641					0	641	0	641			0	641
TCGA-66-	TCGA-66-	LUSC	71	MALE		Stage IIB	Lung Squamous Cell	2006	-26084	Alive	TUMOR F	30					0	30	0	30			0	30
TCGA-66-	TCGA-66-	LUSC	71	MALE		Stage IIB	Lung Squamous Cell	2006	-26084	Alive	TUMOR F	30					0	30	0	30			0	30
TCGA-66-	TCGA-66-	LUSC	69	MALE		Stage IB	Lung Squamous Cell	2007	-25506	Alive	TUMOR F	31					0	31	0	31			0	31
TCGA-66-	TCGA-66-	LUSC	69	MALE		Stage IB	Lung Squamous Cell	2007	-25506	Alive	TUMOR F	31					0	31	0	31			0	31
TCGA-66-	TCGA-66-	LUSC	67	MALE		Stage IIIA	Lung Squamous Cell	2007	-24471	Alive	TUMOR F	61					0	61	0	61			0	61
TCGA-66-	TCGA-66-	LUSC	67	MALE		Stage IIIA	Lung Squamous Cell	2007	-24471	Alive	TUMOR F	61					0	61	0	61			0	61
TCGA-66-	TCGA-66-	LUSC	63	MALE		Stage IB	Lung Squamous Cell	2007	-23348	Alive	TUMOR F	28					0	28	0	28			0	28
TCGA-66-	TCGA-66-	LUSC	68	MALE		Stage IIIB	Lung Squamous Cell	2007	-24929	Alive	TUMOR F	30					0	30	0	30			0	30
TCGA-66-	TCGA-66-	LUSC	65	FEMALE		Stage IA	Lung Squamous Cell	2007	-24014	Dead	WITH TUMOR		1338		912		1	1338	1	1338			1	912
TCGA-66-	TCGA-66-	LUSC	71	MALE		Stage IB	Lung Squamous Cell	2007	-26209	Alive	TUMOR F	639				Complete Remission/Response	0	639	0	639	0	639	0	639
TCGA-66-	TCGA-66-	LUSC	66	MALE		Stage IIIA	Lung Squamous Cell	2007	-24287	Alive	TUMOR F	762				Complete Remission/Response	0	762	0	762	0	762	0	762
TCGA-66-	TCGA-66-	LUSC	63	FEMALE		Stage IB	Lung Squamous Cell	2007	-23315	Alive	TUMOR F	30					0	30	0	30			0	30
TCGA-66-	TCGA-66-	LUSC	64	MALE		Stage IB	Lung Squamous Cell	2007	-23465	Alive	TUMOR F	61					0	61	0	61			0	61
TCGA-66-	TCGA-66-	LUSC	54	MALE		Stage IIIA	Lung Squamous Cell	2007	-19814	Alive	TUMOR F	31					0	31	0	31			0	31
TCGA-66-	TCGA-66-	LUSC	62	MALE		Stage IIIB	Lung Squamous Cell	2007	-22888	Alive	TUMOR F	61					0	61	0	61			0	61
TCGA-66-	TCGA-66-	LUSC	57	MALE		Stage IIB	Lung Squamous Cell	2007	-20970	Alive	TUMOR F	61					0	61	0	61			0	61
TCGA-66-	TCGA-66-	LUSC	75	MALE		Stage IIIB	Lung Squamous Cell	2007	-27515	Dead			215				1	215		215			0	215
TCGA-66-	TCGA-66-	LUSC	79	MALE		Stage IB	Lung Squamous Cell	2007	-28945	Alive		700					0	700	0	700			0	700
TCGA-66-	TCGA-66-	LUSC	60	MALE		Stage IIB	Lung Squamous Cell	2007	-22219	Alive	TUMOR F	578				Complete Remission/Response	0	578	0	578	0	578	0	578
TCGA-66-	TCGA-66-	LUSC	60	MALE		Stage IIB	Lung Squamous Cell	2007	-22219	Alive	TUMOR F	578				Complete Remission/Response	0	578	0	578	0	578	0	578
TCGA-66-	TCGA-66-	LUSC	69	MALE		Stage IB	Lung Squamous Cell	2007	-25355	Dead	TUMOR FREE		92				1	92	0	92			0	92
TCGA-66-	TCGA-66-	LUSC	71	MALE		Stage IB	Lung Squamous Cell	2007	-26085	Alive	TUMOR F	61					0	61	0	61			0	61
TCGA-66-	TCGA-66-	LUSC	68	FEMALE		Stage IIIB	Lung Squamous Cell	2007	-25080	Alive	TUMOR F	578				Complete Remission/Response	0	578	0	578	0	578	0	578
TCGA-66-	TCGA-66-	LUSC	65	MALE		Stage IB	Lung Squamous Cell	2007	-23955	Dead			366				1	366		366			0	366
TCGA-66-	TCGA-66-	LUSC	67	MALE		Stage IB	Lung Squamous Cell	2007	-24471	Alive	TUMOR F	121					0	121	0	121			0	121
TCGA-66-	TCGA-66-	LUSC	71	MALE		Stage IIB	Lung Squamous Cell	2004	-26237	Dead	WITH TUMOR		365		304	Progressive Disease	1	365	1	365			1	304
TCGA-66-	TCGA-66-	LUSC	67	MALE		Stage IIIB	Lung Squamous Cell	2008	-24686	Alive	TUMOR F	759				Complete Remission/Response	0	759	0	759	0	759	0	759
TCGA-66-	TCGA-66-	LUSC	65	MALE		Stage IB	Lung Squamous Cell	2008	-23772	Alive	TUMOR F	60					0	60	0	60			0	60
TCGA-66-	TCGA-66-	LUSC	68	FEMALE		Stage IA	Lung Squamous Cell	2008	-25051	Alive	TUMOR F	790					0	790	0	790			0	790
TCGA-66-	TCGA-66-	LUSC	57	MALE		Stage IA	Lung Squamous Cell	2004	-21124	Alive	TUMOR F	1217					0	1217	0	1217			0	1217
TCGA-66-	TCGA-66-	LUSC	57	MALE		Stage IA	Lung Squamous Cell	2004	-21124	Alive	TUMOR F	1217					0	1217	0	1217			0	1217
TCGA-66-	TCGA-66-	LUSC	56	MALE		Stage IB	Lung Squamous Cell	2008	-20637	Alive	TUMOR F	699					0	699	0	699			0	699
TCGA-66-	TCGA-66-	LUSC	56	MALE		Stage IB	Lung Squamous Cell	2008	-20637	Alive	TUMOR F	699					0	699	0	699			0	699
TCGA-66-	TCGA-66-	LUSC	73	MALE		Stage IIIB	Lung Squamous Cell	2008	-26753	Dead	TUMOR FREE		123				1	123	0	123			0	123
TCGA-66-	TCGA-66-	LUSC	73	MALE		Stage IIIB	Lung Squamous Cell	2008	-26753	Dead	TUMOR FREE		123				1	123	0	123			0	123
TCGA-66-	TCGA-66-	LUSC	72	MALE		Stage IIB	Lung Squamous Cell	2008	-26420	Alive	WITH TUN	699			699	Progressive Disease	0	699	0	699			1	699
TCGA-66-	TCGA-66-	LUSC	72	MALE		Stage IIB	Lung Squamous Cell	2008	-26420	Alive	WITH TUN	699			699	Progressive Disease	0	699	0	699			1	699
TCGA-66-	TCGA-66-	LUSC	66	MALE		Stage IIIB	Lung Squamous Cell	2008	-24138	Dead			153				1	153		153			0	153
TCGA-66-	TCGA-66-	LUSC	66	MALE		Stage IIIB	Lung Squamous Cell	2008	-24138	Dead			153				1	153		153			0	153
TCGA-66-	TCGA-66-	LUSC	58	MALE		Stage IIB	Lung Squamous Cell	2008	-21428	Alive	TUMOR F	913				Complete Remission/Response	0	913	0	913	0	913	0	913
TCGA-66-	TCGA-66-	LUSC	58	MALE		Stage IIB	Lung Squamous Cell	2008	-21428	Alive	TUMOR F	913				Complete Remission/Response	0	913	0	913	0	913	0	913
TCGA-66-	TCGA-66-	LUSC	68	MALE		Stage IIIB	Lung Squamous Cell	2008	-25111	Dead	TUMOR FREE		306				1	306	0	306			0	306
TCGA-66-	TCGA-66-	LUSC	64	MALE		Stage IIIB	Lung Squamous Cell	2006	-23710	Alive	TUMOR F	1645				Complete Remission/Response	0	1645	0	1645	0	1645	0	1645
TCGA-66-	TCGA-66-	LUSC	68	MALE		Stage IIIB	Lung Squamous Cell	2008	-24897	Alive	TUMOR F	122					0	122	0	122			0	122
TCGA-66-	TCGA-66-	LUSC	68	MALE		Stage IIIB	Lung Squamous Cell	2008	-24897	Alive	TUMOR F	122					0	122	0	122			0	122
TCGA-66-	TCGA-66-	LUSC	70	MALE		Stage IIIB	Lung Squamous Cell	2004	-25810	Alive	TUMOR F	1492					0	1492	0	1492			0	1492
TCGA-68-	TCGA-68-	LUSC	60	FEMALE	WHITE	Stage IIA	Lung Squamous Cell	2011	-21936	Alive	TUMOR F	83					0	83	0	83			0	83
TCGA-68-	TCGA-68-	LUSC	84	MALE	WHITE	Stage IIIA	Lung Squamous Cell	2011	-30763	Alive		202					0	202	0	202			0	202
TCGA-68-	TCGA-68-	LUSC	74	MALE	WHITE	Stage IA	Lung Squamous Cell	2011	-27272	Alive		211					0	211	0	211			0	211
TCGA-68-	TCGA-68-	LUSC	66	MALE	BLACK OI	Stage IA	Lung Squamous Cell	2011	-24118	Alive	TUMOR F	244					0	244	0	244			0	244
TCGA-68-	TCGA-68-	LUSC	78	MALE	WHITE	Stage IB	Lung Squamous Cell	2012	-28739	Alive	TUMOR F	406				Stable Disease	0	406	0	406			0	406
TCGA-68-	TCGA-68-	LUSC	73	FEMALE	BLACK OI	Stage IIIA	Lung Squamous Cell	2012	-26839	Alive	WITH TUN	492		New Primary Tumor	159	Stable Disease	0	492	0	492			1	159
TCGA-68-	TCGA-68-	LUSC	74	FEMALE	WHITE	Stage IB	Lung Squamous Cell	2012	-27031	Alive	TUMOR F	448				Complete Remission/Response	0	448	0	448	0	448	0	448
TCGA-6A-	TCGA-6A-	LUSC	73	FEMALE	BLACK OI	Stage IB	Lung Squamous Cell	2009		Dead	TUMOR FREE					Progressive Disease	1		0				0	
TCGA-70-	TCGA-70-	LUSC	47	MALE	ASIAN	Stage IIIA	Lung Squamous Cell	2010	-17328	Alive	WITH TUN	367		Locoregional Recurrence	293	Progressive Disease	0	367	0	367			1	293
TCGA-70-	TCGA-70-	LUSC	65	MALE	ASIAN	Stage IIA	Lung Squamous Cell	2010	-24035	Alive	WITH TUN	375		Locoregional Recurrence	312	Progressive Disease	0	375	0	375			1	312
TCGA-77-	TCGA-77-	LUSC	79	MALE	WHITE	Stage IIB	Lung Squamous Cell	2000	-28861	Dead			899		201		1	899		899			1	201
TCGA-77-	TCGA-77-	LUSC	74	MALE	WHITE	Stage IIA	Lung Squamous Cell	2002	-27291	Dead			2224				1	2224		2224			0	2224
TCGA-77-	TCGA-77-	LUSC	74	MALE	WHITE	Stage IIIA	Lung Squamous Cell	2002	-27220	Dead			2284		1788	Complete Remission/Response	1	2284		2284	1	1788	1	1788
TCGA-77-	TCGA-77-	LUSC	69	MALE	WHITE	Stage IIB	Lung Squamous Cell	2003	-25321	Dead			708				1	708		708			0	708
TCGA-77-	TCGA-77-	LUSC	67	MALE		Stage IB	Lung Squamous Cell	1999	-24594	Dead			340				1	340		340			0	340
TCGA-77-	TCGA-77-	LUSC	67	MALE		Stage IB	Lung Squamous Cell	1999	-24594	Dead			340				1	340		340			0	340
TCGA-77-	TCGA-77-	LUSC	56	MALE	WHITE	Stage IIB	Lung Squamous Cell	2002	-20632	Alive		4261				Complete Remission/Response	0	4261	0	4261	0	4261	0	4261
TCGA-77-	TCGA-77-	LUSC	69	FEMALE	WHITE	Stage IIB	Lung Squamous Cell	2002	-25303	Dead			632		250		1	632		632			1	250
TCGA-77-	TCGA-77-	LUSC	64	MALE	WHITE	Stage IB	Lung Squamous Cell	2003	-23584	Alive		15					0	15	0	15			0	15
TCGA-77-	TCGA-77-	LUSC	59	FEMALE	WHITE	Stage IB	Lung Squamous Cell	2007	-21796	Alive	TUMOR F	2227					0	2227	0	2227			0	2227
TCGA-77-	TCGA-77-	LUSC	59	FEMALE	WHITE	Stage IB	Lung Squamous Cell	2007	-21796	Alive	TUMOR F	2227					0	2227	0	2227			0	2227
TCGA-77-	TCGA-77-	LUSC	62	FEMALE	WHITE	Stage IIIB	Lung Squamous Cell	1996	-22863	Dead			2133				1	2133		2133			0	2133
TCGA-77-	TCGA-77-	LUSC	62	FEMALE	WHITE	Stage IIIB	Lung Squamous Cell	1996	-22863	Dead			2133				1	2133		2133			0	2133
TCGA-77-	TCGA-77-	LUSC	65	MALE	WHITE	Stage IIB	Lung Squamous Cell	2000	-24065	Dead			3253				1	3253		3253			0	3253
TCGA-77-	TCGA-77-	LUSC	65	MALE	WHITE	Stage IIB	Lung Squamous Cell	2000	-24065	Dead			3253				1	3253		3253			0	3253
TCGA-77-	TCGA-77-	LUSC	64	MALE	WHITE	Stage IB	Lung Squamous Cell	1995	-23480	Dead			5				1	5		5			0	5
TCGA-77-	TCGA-77-	LUSC	64	MALE	WHITE	Stage IB	Lung Squamous Cell	1995	-23480	Dead			5				1	5		5			0	5
TCGA-77-	TCGA-77-	LUSC	75	MALE	WHITE	Stage IB	Lung Squamous Cell	2001	-27520	Dead			1423				1	1423		1423			0	1423
TCGA-77-	TCGA-77-	LUSC	58	MALE	WHITE	Stage IIA	Lung Squamous Cell	2010	-21548	Alive		990				Complete Remission/Response	0	990	0	990	0	990	0	990
TCGA-77-	TCGA-77-	LUSC	68	MALE		Stage IIB	Lung Squamous Cell	2001	-25102	Dead			198		139		1	198		198			1	139
TCGA-77-	TCGA-77-	LUSC	68	MALE		Stage IIB	Lung Squamous Cell	2001	-25102	Dead			198		139		1	198		198			1	139
TCGA-77-	TCGA-77-	LUSC	68	MALE		Stage IB	Lung Squamous Cell	2001	-24948	Dead			2639				1	2639		2639			0	2639
TCGA-77-	TCGA-77-	LUSC	68	MALE		Stage IB	Lung Squamous Cell	2001	-24948	Dead			2639				1	2639		2639			0	2639
TCGA-77-	TCGA-77-	LUSC	68	MALE		Stage IIB	Lung Squamous Cell	2007	-25037	Dead			1695		607	Complete Remission/Response	1	1695		1695	1	607	1	607
TCGA-77-	TCGA-77-	LUSC	60	MALE	[Not Evalu	Stage IIIA	Lung Squamous Cell	1999	-21986	Dead			1150				1	1150		1150			0	1150
TCGA-77-	TCGA-77-	LUSC	69	MALE	[Not Evalu	Stage IIB	Lung Squamous Cell	2000	-25260	Alive	TUMOR F	4765		New Primary Tumor	4471	Complete Remission/Response	0	4765	0	4765	0	4765	1	4471
TCGA-77-	TCGA-77-	LUSC	72	MALE	[Not Evalu	Stage IB	Lung Squamous Cell	2000	-26611	Dead			383			[Unknown]	1	383		383			0	383
TCGA-77-	TCGA-77-	LUSC	74	MALE	[Not Evalu	Stage IIA	Lung Squamous Cell	2001	-27139	Dead			1640				1	1640		1640			0	1640
TCGA-77-	TCGA-77-	LUSC	74	FEMALE	[Not Evalu	Stage IIB	Lung Squamous Cell	2002	-27193	Dead			1189	Distant Metastasis	301		1	1189		1189			1	301
TCGA-77-	TCGA-77-	LUSC	74	MALE	[Not Evalu	Stage IB	Lung Squamous Cell	2003	-27225	Dead	WITH TUMOR		539	Locoregional Recurrence	289		1	539	1	539			1	289
TCGA-77-	TCGA-77-	LUSC	72	MALE	[Not Evalu	Stage IIB	Lung Squamous Cell	2003	-26464	Alive	TUMOR F	3166				Complete Remission/Response	0	3166	0	3166	0	3166	0	3166
TCGA-77-	TCGA-77-	LUSC	66	FEMALE	[Not Evalu	Stage IIB	Lung Squamous Cell	2003	-24415	Dead			351				1	351		351			0	351
TCGA-77-	TCGA-77-	LUSC	76	MALE	[Not Evalu	Stage IIIA	Lung Squamous Cell	2004	-27772	Dead	WITH TUMOR		803			[Unknown]	1	803	1	803			1	803
TCGA-77-	TCGA-77-	LUSC	70	MALE	[Not Evalu	Stage IB	Lung Squamous Cell	2004	-25796	Alive		833				Complete Remission/Response	0	833	0	833	0	833	0	833
TCGA-77-	TCGA-77-	LUSC	73	MALE	[Not Evalu	Stage IIIB	Lung Squamous Cell	2000	-26707	Dead			212	Distant Metastasis	136	[Unknown]	1	212		212			1	136
TCGA-77-	TCGA-77-	LUSC	72	MALE	[Not Evalu	Stage IA	Lung Squamous Cell	2005	-26533	Alive		3189				Complete Remission/Response	0	3189	0	3189	0	3189	0	3189
TCGA-77-	TCGA-77-	LUSC	68	MALE	[Not Evalu	Stage IIIA	Lung Squamous Cell	2006	-24918	Alive		2023				Complete Remission/Response	0	2023	0	2023	0	2023	0	2023
TCGA-77-	TCGA-77-	LUSC	64	MALE	[Not Evalu	Stage IIIA	Lung Squamous Cell	2007	-23474	Dead	WITH TUMOR		1655	Locoregional Recurrence	1278		1	1655	1	1655			1	1278
TCGA-77-	TCGA-77-	LUSC	77	FEMALE	[Not Evalu	Stage IB	Lung Squamous Cell	2008	-28253	Alive	TUMOR F	1992				Complete Remission/Response	0	1992	0	1992	0	1992	0	1992
TCGA-77-	TCGA-77-	LUSC	77	FEMALE	[Not Evalu	Stage IB	Lung Squamous Cell	2008	-28253	Alive	TUMOR F	1992				Complete Remission/Response	0	1992	0	1992	0	1992	0	1992
TCGA-77-	TCGA-77-	LUSC	67	MALE	[Not Evalu	Stage IA	Lung Squamous Cell	2008	-24640	Alive	TUMOR F	1841				Complete Remission/Response	0	1841	0	1841	0	1841	0	1841
TCGA-77-	TCGA-77-	LUSC	60	MALE	[Not Evalu	Stage IB	Lung Squamous Cell	2010	-22198	Alive		1106				Complete Remission/Response	0	1106	0	1106	0	1106	0	1106
TCGA-77-	TCGA-77-	LUSC	64	MALE	[Not Evalu	Stage IIIB	Lung Squamous Cell	2001	-23436	Dead			3838			Complete Remission/Response	1	3838		3838	0	3838	0	3838
TCGA-77-	TCGA-77-	LUSC	75	MALE	[Not Evalu	Stage IIIA	Lung Squamous Cell	2002	-27555	Alive		4026		New Primary Tumor	2197	Complete Remission/Response	0	4026	0	4026	0	4026	1	2197
TCGA-77-	TCGA-77-	LUSC	63	MALE	[Not Evalu	Stage IIB	Lung Squamous Cell	2002	-23171	Alive	TUMOR F	4570				Complete Remission/Response	0	4570	0	4570	0	4570	0	4570
TCGA-77-	TCGA-77-	LUSC	66	MALE	[Not Evalu	Stage IIIA	Lung Squamous Cell	2009	-24215	Dead	WITH TUMOR		678	Locoregional Recurrence	392	Complete Remission/Response	1	678	1	678	1	392	1	392
TCGA-77-	TCGA-77-	LUSC	63	MALE	[Not Evalu	Stage IA	Lung Squamous Cell	2009	-23090	Dead			180			Complete Remission/Response	1	180		180	0	180	0	180
TCGA-77-	TCGA-77-	LUSC	70	MALE	[Not Evalu	Stage IIB	Lung Squamous Cell	2009	-25921	Alive		1884				[Unknown]	0	1884	0	1884			0	1884
TCGA-77-	TCGA-77-	LUSC	76	MALE	[Not Evalu	Stage IB	Lung Squamous Cell	2010	-27762	Alive		1280				Complete Remission/Response	0	1280	0	1280	0	1280	0	1280
TCGA-77-	TCGA-77-	LUSC	90	MALE	[Not Evalu	Stage IB	Lung Squamous Cell	2010	-32872	Dead			937	New Primary Tumor	229	Complete Remission/Response	1	937		937	0	937	1	229
TCGA-77-	TCGA-77-	LUSC	70	MALE	[Not Evalu	Stage IIA	Lung Squamous Cell	2011	-25654	Dead	WITH TUMOR		840	Locoregional Recurrence	722	Complete Remission/Response	1	840	1	840	1	722	1	722
TCGA-77-	TCGA-77-	LUSC	81	MALE	[Not Evalu	Stage IB	Lung Squamous Cell	2011	-29843	Alive		1182				Complete Remission/Response	0	1182	0	1182	0	1182	0	1182
TCGA-79-	TCGA-79-	LUSC		MALE		Stage IIIA	Lung Squamous Cell	2006		Alive	TUMOR FREE						0		0				0	
TCGA-85-	TCGA-85-	LUSC	63	FEMALE	WHITE	Stage IIB	Lung Squamous Cell	2010	-23228	Dead	WITH TUMOR		294			Stable Disease	1	294	1	294			1	294
TCGA-85-	TCGA-85-	LUSC	59	MALE	WHITE	Stage IIA	Lung Squamous Cell	2011	-21768	Alive	TUMOR F	1259				Complete Remission/Response	0	1259	0	1259	0	1259	0	1259
TCGA-85-	TCGA-85-	LUSC	66	MALE	WHITE	Stage IB	Lung Squamous Cell	2011	-24234	Alive	TUMOR F	1224				Complete Remission/Response	0	1224	0	1224	0	1224	0	1224
TCGA-85-	TCGA-85-	LUSC	57	MALE	WHITE	Stage IIIA	Lung Squamous Cell	2011	-20837	Dead	WITH TUMOR		195	Distant Metastasis	180	Complete Remission/Response	1	195	1	195	1	180	1	180
TCGA-85-	TCGA-85-	LUSC	64	MALE	WHITE	Stage IA	Lung Squamous Cell	2011	-23413	Alive	TUMOR F	1111				Complete Remission/Response	0	1111	0	1111	0	1111	0	1111
TCGA-85-	TCGA-85-	LUSC	49	MALE	WHITE	Stage IIB	Lung Squamous Cell	2011	-17921	Alive	TUMOR F	1063				Complete Remission/Response	0	1063	0	1063	0	1063	0	1063
TCGA-85-	TCGA-85-	LUSC	48	MALE	WHITE	Stage IA	Lung Squamous Cell	2011	-17651	Alive	TUMOR F	952		Distant Metastasis	650	Complete Remission/Response	0	952	0	952	1	650	1	650
TCGA-85-	TCGA-85-	LUSC	73	MALE	WHITE	Stage IIIA	Lung Squamous Cell	2011	-26965	Dead	WITH TUMOR		1001	Distant Metastasis	760	Complete Remission/Response	1	1001	1	1001	1	760	1	760
TCGA-85-	TCGA-85-	LUSC	59	FEMALE	WHITE	Stage IA	Lung Squamous Cell	2011	-21790	Alive	TUMOR F	42				Complete Remission/Response	0	42	0	42	0	42	0	42
TCGA-85-	TCGA-85-	LUSC	59	FEMALE	WHITE	Stage IA	Lung Squamous Cell	2011	-21790	Alive	TUMOR F	42				Complete Remission/Response	0	42	0	42	0	42	0	42
TCGA-85-	TCGA-85-	LUSC	50	MALE	WHITE	Stage IIA	Lung Squamous Cell	2011	-18353	Alive		35					0	35	0	35			0	35
TCGA-85-	TCGA-85-	LUSC	71	MALE	WHITE	Stage IB	Lung Squamous Cell	2011	-26141	Alive	TUMOR F	911				Complete Remission/Response	0	911	0	911	0	911	0	911
TCGA-85-	TCGA-85-	LUSC	46	MALE	WHITE	Stage IB	Lung Squamous Cell	2011	-16975	Alive	TUMOR F	576				Complete Remission/Response	0	576	0	576	0	576	0	576
TCGA-85-	TCGA-85-	LUSC	62	MALE	WHITE	Stage IA	Lung Squamous Cell	2011	-22907	Alive	TUMOR F	765				Complete Remission/Response	0	765	0	765	0	765	0	765
TCGA-85-	TCGA-85-	LUSC	57	MALE	WHITE	Stage IB	Lung Squamous Cell	2011	-20995	Alive	TUMOR F	579				Complete Remission/Response	0	579	0	579	0	579	0	579
TCGA-85-	TCGA-85-	LUSC	53	MALE	WHITE	Stage IIB	Lung Squamous Cell	2011	-19671	Alive	TUMOR F	734				Complete Remission/Response	0	734	0	734	0	734	0	734
TCGA-85-	TCGA-85-	LUSC	71	MALE	WHITE	Stage IB	Lung Squamous Cell	2011	-26183	Alive	TUMOR F	960				Complete Remission/Response	0	960	0	960	0	960	0	960

TCGA-85-	TCGA-85-	LUSC	52	MALE	WHITE	Stage IIA	Lung Squamous Cell		2011	-19158	Alive	TUMOR F	815					Complete Remission/Response		0	815	0	815	0	815	0	815
TCGA-85-	TCGA-85-	LUSC	60	MALE	WHITE	Stage IA	Lung Squamous Cell		2011	-22069	Alive	TUMOR F	932					Complete Remission/Response		0	932	0	932	0	932	0	932
TCGA-85-	TCGA-85-	LUSC	62	MALE	WHITE	Stage IIA	Lung Squamous Cell		2011	-22707	Alive	WITH TUN	1050		Distant Metastasis		1048	Complete Remission/Response		0	1050	0	1050	1	1048	1	1048
TCGA-85-	TCGA-85-	LUSC	70	MALE	WHITE	Stage IIIA	Lung Squamous Cell		2011	-25793	Dead	TUMOR FREE		307				Complete Remission/Response		1	307	0	307	0	307	0	307
TCGA-85-	TCGA-85-	LUSC	72	MALE	WHITE	Stage IA	Lung Squamous Cell		2011	-26401	Dead	TUMOR FREE		23				Complete Remission/Response		1	23	0	23	0	23	0	23
TCGA-85-	TCGA-85-	LUSC	70	MALE	WHITE	Stage IIA	Lung Squamous Cell		2011	-25629	Dead	TUMOR FREE		402				Complete Remission/Response		1	402	0	402	0	402	0	402
TCGA-85-	TCGA-85-	LUSC	61	MALE	WHITE	Stage IA	Lung Squamous Cell		2011	-22526	Alive	TUMOR F	683					Complete Remission/Response		0	683	0	683	0	683	0	683
TCGA-85-	TCGA-85-	LUSC	72	MALE	WHITE	Stage IIA	Lung Squamous Cell		2011	-26437	Alive	TUMOR F	510					Complete Remission/Response		0	510	0	510	0	510	0	510
TCGA-85-	TCGA-85-	LUSC	67	MALE	WHITE	Stage IIIA	Lung Squamous Cell		2011	-24788	Dead	WITH TUMOR		161	Distant Metastasis		128	Complete Remission/Response		1	161	1	161	1	128	1	128
TCGA-85-	TCGA-85-	LUSC	72	MALE	WHITE	Stage IIIA	Lung Squamous Cell		2011	-26549	Dead	WITH TUMOR		94				Stable Disease		1	94	1	94			1	94
TCGA-85-	TCGA-85-	LUSC	53	MALE	WHITE	Stage IB	Lung Squamous Cell		2011	-19692	Alive	TUMOR F	995					Complete Remission/Response		0	995	0	995	0	995	0	995
TCGA-85-	TCGA-85-	LUSC	63	MALE	WHITE	Stage IA	Lung Squamous Cell		2011	-23227	Alive	TUMOR F	61					Complete Remission/Response		0	61	0	61	0	61	0	61
TCGA-85-	TCGA-85-	LUSC	66	MALE	WHITE	Stage IA	Lung Squamous Cell		2012	-24350	Alive	TUMOR F	468					Complete Remission/Response		0	468	0	468	0	468	0	468
TCGA-85-	TCGA-85-	LUSC	70	MALE	WHITE	Stage IIB	Lung Squamous Cell		2012	-25782	Dead	TUMOR FREE		236				Partial Remission/Response		1	236	0	236			0	236
TCGA-85-	TCGA-85-	LUSC	52	FEMALE	WHITE	Stage IB	Lung Squamous Cell		2012	-19301	Alive	TUMOR F	1113					Complete Remission/Response		0	1113	0	1113	0	1113	0	1113
TCGA-85-	TCGA-85-	LUSC	49	MALE	WHITE	Stage IA	Lung Squamous Cell		2012	-18168	Alive	TUMOR F	1160					Complete Remission/Response		0	1160	0	1160	0	1160	0	1160
TCGA-85-	TCGA-85-	LUSC	71	MALE	WHITE	Stage IIA	Lung Squamous Cell		2012	-26081	Dead	TUMOR FREE		383				Complete Remission/Response		1	383	0	383	0	383	0	383
TCGA-85-	TCGA-85-	LUSC	73	MALE	WHITE	Stage IIB	Lung Squamous Cell		2011	-26954	Dead	WITH TUMOR		550	Distant Metastasis		434	Complete Remission/Response		1	550	1	550	1	434	1	434
TCGA-85-	TCGA-85-	LUSC	65	MALE	WHITE	Stage IB	Lung Squamous Cell		2011	-23906	Dead	TUMOR FREE		687				Complete Remission/Response		1	687	0	687	0	687	0	687
TCGA-85-	TCGA-85-	LUSC	65	MALE	WHITE	Stage IA	Lung Squamous Cell		2012	-24005	Dead	TUMOR FREE		921				Complete Remission/Response		1	921	0	921	0	921	0	921
TCGA-85-	TCGA-85-	LUSC	56	FEMALE	WHITE	Stage IIB	Lung Squamous Cell		2012	-20490	Alive	TUMOR F	1038					Complete Remission/Response		0	1038	0	1038	0	1038	0	1038
TCGA-85-	TCGA-85-	LUSC	74	MALE	WHITE	Stage IIB	Lung Squamous Cell		2012	-27315	Alive	TUMOR F	942					Complete Remission/Response		0	942	0	942	0	942	0	942
TCGA-85-	TCGA-85-	LUSC	84	MALE	WHITE	Stage IIA	Lung Squamous Cell		2012	-30942	Dead	WITH TUMOR		604	Locoregional Recurrence		548	Complete Remission/Response		1	604	1	604	1	548	1	548
TCGA-85-	TCGA-85-	LUSC	61	MALE	WHITE	Stage IB	Lung Squamous Cell		2012	-22614	Alive	TUMOR F	741					Complete Remission/Response		0	741	0	741	0	741	0	741
TCGA-85-	TCGA-85-	LUSC	68	MALE	WHITE	Stage IB	Lung Squamous Cell		2012	-25065	Dead	TUMOR FREE		927				Complete Remission/Response		1	927	0	927	0	927	0	927
TCGA-85-	TCGA-85-	LUSC	67	MALE	WHITE	Stage IB	Lung Squamous Cell		2012	-24756	Alive	TUMOR F	600					Complete Remission/Response		0	600	0	600	0	600	0	600
TCGA-85-	TCGA-85-	LUSC	47	MALE	ASIAN	Stage IIA	Lung Squamous Cell		2012	-17429	Dead	TUMOR FREE		826				Complete Remission/Response		1	826	0	826	0	826	0	826
TCGA-85-	TCGA-85-	LUSC	57	MALE	WHITE	Stage IIA	Lung Squamous Cell		2012	-20941	Alive	TUMOR F	493		New Primary Tumor		462	Complete Remission/Response		0	493	0	493	0	493	1	462
TCGA-85-	TCGA-85-	LUSC	74	FEMALE	WHITE	Stage IIB	Lung Squamous Cell		2012	-27067	Alive	TUMOR F	482		Distant Metastasis		363	Complete Remission/Response		0	482	0	482	1	363	1	363
TCGA-85-	TCGA-85-	LUSC	62	MALE	WHITE	Stage IIB	Lung Squamous Cell		2012	-22759	Dead	WITH TUMOR		455	Distant Metastasis		390	Complete Remission/Response		1	455	1	455	1	390	1	390
TCGA-85-	TCGA-85-	LUSC	46	MALE	WHITE	Stage IIA	Lung Squamous Cell		2012	-16811	Alive	TUMOR F	465					Complete Remission/Response		0	465	0	465	0	465	0	465
TCGA-85-	TCGA-85-	LUSC	60	FEMALE	ASIAN	Stage IA	Lung Squamous Cell		2012	-21967	Alive	TUMOR F	910					Complete Remission/Response		0	910	0	910	0	910	0	910
TCGA-85-	TCGA-85-	LUSC	63	MALE	ASIAN	Stage IIA	Lung Squamous Cell		2012	-23065	Alive	TUMOR F	377					Complete Remission/Response		0	377	0	377	0	377	0	377
TCGA-85-	TCGA-85-	LUSC	58	MALE	WHITE	Stage IA	Lung Squamous Cell		2012	-21450	Alive	TUMOR F	111					Complete Remission/Response		0	111	0	111	0	111	0	111
TCGA-90-	TCGA-90-	LUSC	64	MALE	WHITE	Stage IIB	Lung Squamous Cell		2011	-23511	Alive	TUMOR F	758					Complete Remission/Response		0	758	0	758	0	758	0	758
TCGA-90-	TCGA-90-	LUSC	64	MALE	WHITE	Stage IIB	Lung Squamous Cell		2011	-23511	Alive	TUMOR F	758					Complete Remission/Response		0	758	0	758	0	758	0	758
TCGA-90-	TCGA-90-	LUSC	66	FEMALE	WHITE	Stage IA	Lung Squamous Cell		2011	-24354	Alive	WITH TUN	289		Locoregional Recurrence		289	Progressive Disease		0	289	0	289			1	289
TCGA-90-	TCGA-90-	LUSC	56	MALE	WHITE	Stage IIB	Lung Squamous Cell		2011	-20534	Alive	TUMOR F	89					[Unknown]		0	89	0	89			0	89
TCGA-90-	TCGA-90-	LUSC	56	MALE	WHITE	Stage IIB	Lung Squamous Cell		2011	-20534	Alive	TUMOR F	89					[Unknown]		0	89	0	89			0	89
TCGA-90-	TCGA-90-	LUSC	55	MALE	WHITE	Stage IIB	Lung Squamous Cell		2011	-20276	Alive	TUMOR F	358					Complete Remission/Response		0	358	0	358	0	358	0	358
TCGA-90-	TCGA-90-	LUSC	70	MALE	WHITE	Stage IB	Lung Squamous Cell		2011	-25646	Alive	TUMOR F	428					Complete Remission/Response		0	428	0	428	0	428	0	428
TCGA-90-	TCGA-90-	LUSC	69	MALE	WHITE	Stage IB	Lung Squamous Cell		2012	-25453	Alive	TUMOR F	615					Complete Remission/Response		0	615	0	615	0	615	0	615
TCGA-90-	TCGA-90-	LUSC	53	MALE	WHITE	Stage IIA	Lung Squamous Cell		2012	-19676	Alive	TUMOR F	688					Complete Remission/Response		0	688	0	688	0	688	0	688
TCGA-90-	TCGA-90-	LUSC	61	FEMALE	WHITE	Stage IIA	Lung Squamous Cell		2012	-22420	Dead	WITH TUMOR		322	Distant Metastasis		274	Complete Remission/Response		1	322	1	322	1	274	1	274
TCGA-92-	TCGA-92-	LUSC	45	FEMALE	WHITE	Stage IIA	Lung Squamous Cell		2011	-16447	Alive	WITH TUN	82					Stable Disease		0	82	0	82			0	82
TCGA-92-	TCGA-92-	LUSC	45	FEMALE	WHITE	Stage IIA	Lung Squamous Cell		2011	-16447	Alive	WITH TUN	82					Stable Disease		0	82	0	82			0	82
TCGA-92-	TCGA-92-	LUSC	71	MALE	WHITE	Stage IB	Lung Squamous Cell		2011	-26134	Alive	WITH TUN	106					Partial Remission/Response		0	106	0	106			0	106
TCGA-92-	TCGA-92-	LUSC	71	MALE	WHITE	Stage IB	Lung Squamous Cell		2011	-26134	Alive	WITH TUN	106					Partial Remission/Response		0	106	0	106			0	106
TCGA-92-	TCGA-92-	LUSC	52	MALE	WHITE	Stage IIIA	Lung Squamous Cell		2011	-19193	Alive	TUMOR F	122					Complete Remission/Response		0	122	0	122	0	122	0	122
TCGA-92-	TCGA-92-	LUSC	58	MALE	WHITE	[Discrepancy]	Lung Squamous Cell		2011	-21417	Alive	TUMOR F	160					Complete Remission/Response		0	160	0	160	0	160	0	160
TCGA-92-	TCGA-92-	LUSC	74	FEMALE	WHITE	Stage IIB	Lung Squamous Cell		2012	-27049	Alive	TUMOR F	70					Complete Remission/Response		0	70	0	70	0	70	0	70
TCGA-94-	TCGA-94-	LUSC	73	MALE	WHITE	Stage IB	Lung Squamous Cell		2011	-26948	Alive	TUMOR F	640					Complete Remission/Response		0	640	0	640	0	640	0	640
TCGA-94-	TCGA-94-	LUSC	73	MALE	BLACK OI	Stage IB	Lung Squamous Cell		2011	-26802	Dead	TUMOR FREE		5				Complete Remission/Response		1	5	0	5	0	5	0	5
TCGA-94-	TCGA-94-	LUSC	80	MALE	WHITE	Stage IA	Lung Squamous Cell		2011	-29282	Alive	WITH TUN	559		Locoregional Recurrence		556	Complete Remission/Response		0	559	0	559	1	556	1	556
TCGA-94-	TCGA-94-	LUSC	64	MALE	WHITE	Stage IIB	Lung Squamous Cell		2011	-23399	Alive	TUMOR F	122					Complete Remission/Response		0	122	0	122	0	122	0	122
TCGA-94-	TCGA-94-	LUSC	70	MALE	WHITE	Stage IIB	Lung Squamous Cell		2011	-25588	Alive	TUMOR F	153							0	153	0	153			0	153
TCGA-94-	TCGA-94-	LUSC	73	MALE	WHITE	Stage IIA	Lung Squamous Cell		2012	-26698	Alive	TUMOR F	810		Distant Metastasis		641	Progressive Disease		0	810	0	810			1	641
TCGA-94-	TCGA-94-	LUSC	71	FEMALE	WHITE	Stage IA	Lung Squamous Cell		2012	-25991	Alive	TUMOR F	430					Complete Remission/Response		0	430	0	430	0	430	0	430
TCGA-94-	TCGA-94-	LUSC	61	MALE	WHITE	Stage IIA	Lung Squamous Cell		2012	-22299	Alive	WITH TUN	491		Distant Metastasis		314	Complete Remission/Response		0	491	0	491	1	314	1	314
TCGA-94-	TCGA-94-	LUSC	62	MALE	WHITE	Stage IIB	Lung Squamous Cell		2012	-22949	Alive	WITH TUN	538		Distant Metastasis		481	Progressive Disease		0	538	0	538			1	481
TCGA-96-	TCGA-96-	LUSC	83	MALE	WHITE	Stage IIB	Lung Squamous Cell		2008	-30548	Dead	TUMOR FREE		2160				Complete Remission/Response		1	2160	0	2160	0	2160	0	2160
TCGA-96-	TCGA-96-	LUSC	73	MALE	WHITE	Stage IA	Lung Squamous Cell		2008	-26729	Dead			1736	Locoregional Recurrence		1693	Progressive Disease		1	1736		1736			1	1693
TCGA-96-	TCGA-96-	LUSC	67	FEMALE	WHITE	Stage IA	Lung Squamous Cell		2011	-24674	Alive	TUMOR F	557					Complete Remission/Response		0	557	0	557	0	557	0	557
TCGA-96-	TCGA-96-	LUSC	75	FEMALE	WHITE	Stage IIA	Lung Squamous Cell		2011	-27558	Alive	TUMOR F	531					Complete Remission/Response		0	531	0	531	0	531	0	531
TCGA-96-	TCGA-96-	LUSC	65	MALE	WHITE	Stage IIA	Lung Squamous Cell		2012	-23974	Alive	TUMOR F	589					Complete Remission/Response		0	589	0	589	0	589	0	589
TCGA-96-	TCGA-96-	LUSC	78	FEMALE	ASIAN	Stage IIA	Lung Squamous Cell		2012	-28686	Alive	TUMOR F	842					Complete Remission/Response		0	842	0	842	0	842	0	842
TCGA-98-	TCGA-98-	LUSC	73	MALE	WHITE	Stage IB	Lung Squamous Cell		2011	-26827	Alive	TUMOR F	652					Complete Remission/Response		0	652	0	652	0	652	0	652
TCGA-98-	TCGA-98-	LUSC	56	FEMALE	BLACK OI	Stage IIIA	Lung Squamous Cell		2011	-20509	Dead	WITH TUMOR		84	Distant Metastasis		11	[Unknown]		1	84	1	84			1	11
TCGA-98-	TCGA-98-	LUSC	75	FEMALE	WHITE	Stage IA	Lung Squamous Cell		2011	-27738	Alive	TUMOR F	937		Locoregional Recurrence		918	Complete Remission/Response		0	937	0	937	1	918	1	918
TCGA-98-	TCGA-98-	LUSC	61	MALE	WHITE	Stage IA	Lung Squamous Cell		2011	-22487	Dead	TUMOR FREE		933				Complete Remission/Response		1	933	0	933	0	933	0	933
TCGA-98-	TCGA-98-	LUSC	70	MALE	WHITE	Stage IIIA	Lung Squamous Cell		2011	-25732	Alive	TUMOR F	649					Complete Remission/Response		0	649	0	649	0	649	0	649
TCGA-98-	TCGA-98-	LUSC	67	MALE	WHITE	Stage IIB	Lung Squamous Cell		2012	-24698	Alive	TUMOR F	826					Complete Remission/Response		0	826	0	826	0	826	0	826
TCGA-98-	TCGA-98-	LUSC	63	MALE	WHITE	Stage IIB	Lung Squamous Cell		2011	-23098	Alive	WITH TUN	173		Locoregional Recurrence		173			0	173	0	173			1	173
TCGA-98-	TCGA-98-	LUSC	70	MALE	BLACK OI	Stage IB	Lung Squamous Cell		2012	-25662	Dead			552				Complete Remission/Response		1	552		552	0	552	0	552
TCGA-98-	TCGA-98-	LUSC	69	MALE	WHITE	Stage IB	Lung Squamous Cell		2012	-25390	Dead	TUMOR FREE		61				Complete Remission/Response		1	61	0	61	0	61	0	61
TCGA-98-	TCGA-98-	LUSC	77	FEMALE	WHITE	Stage IA	Lung Squamous Cell		2012	-28146	Alive	TUMOR F	822					Complete Remission/Response		0	822	0	822	0	822	0	822
TCGA-98-	TCGA-98-	LUSC	68	MALE	WHITE	Stage IIB	Lung Squamous Cell		2012	-24891	Dead	WITH TUMOR		645	Locoregional Recurrence		413	Complete Remission/Response		1	645	1	645	1	413	1	413
TCGA-98-	TCGA-98-	LUSC	76	FEMALE	WHITE	Stage IA	Lung Squamous Cell		2012	-27928	Alive		618		New Primary Tumor		427	Complete Remission/Response		0	618	0	618	0	618	1	427
TCGA-98-	TCGA-98-	LUSC	64	MALE	WHITE	Stage IIA	Lung Squamous Cell		2012	-23502	Alive	TUMOR F	565					Complete Remission/Response		0	565	0	565	0	565	0	565
TCGA-98-	TCGA-98-	LUSC	77	MALE	WHITE	Stage IB	Lung Squamous Cell		2012	-28485	Alive	TUMOR F	630					Complete Remission/Response		0	630	0	630	0	630	0	630
TCGA-J1-	TCGA-J1-	LUSC	70	MALE	WHITE	Stage IIA	Lung Squamous Cell		2012	-25893	Alive	TUMOR F	581					Complete Remission/Response		0	581	0	581	0	581	0	581
TCGA-L3-	TCGA-L3-	LUSC	71	MALE	WHITE	Stage IB	Lung Squamous Cell		2012	-26232	Alive	TUMOR F	392					Complete Remission/Response		0	392	0	392	0	392	0	392
TCGA-L3-	TCGA-L3-	LUSC	45	FEMALE	WHITE	Stage IIB	Lung Squamous Cell		2011	-16765	Dead	TUMOR FREE		490				Complete Remission/Response		1	490	0	490	0	490	0	490
TCGA-LA	TCGA-LA	LUSC	68	MALE	WHITE	Stage IA	Lung Squamous Cell		2012	-25199	Alive	TUMOR F	401					Complete Remission/Response		0	401	0	401	0	401	0	401
TCGA-LA	TCGA-LA	LUSC	71	MALE	BLACK OI	Stage IIIA	Lung Squamous Cell		2013	-26116	Dead	WITH TUMOR		408	Locoregional Recurrence		235			1	408	1	408			1	235
TCGA-MF	TCGA-MF	LUSC	54	MALE	WHITE	Stage IB	Lung Squamous Cell		2012	-20051	Dead	WITH TUMOR		360	Distant Metastasis		173	Partial Remission/Response		1	360	1	360			1	173
TCGA-NC	TCGA-NC	LUSC	79	MALE	WHITE	Stage IIB	Lung Squamous Cell		2008	-28916	Dead	TUMOR FREE		2						1	2	0	2			0	2
TCGA-NC	TCGA-NC	LUSC	60	MALE	WHITE	Stage IIB	Lung Squamous Cell		2008	-22179	Alive	TUMOR F	2336					Complete Remission/Response		0	2336	0	2336	0	2336	0	2336
TCGA-NC	TCGA-NC	LUSC	74	MALE	WHITE	Stage IIIB	Lung Squamous Cell		2008	-27038	Dead	WITH TUMOR		138	Distant Metastasis		132	Progressive Disease		1	138	1	138			1	132
TCGA-NC	TCGA-NC	LUSC	59	MALE	WHITE	Stage IIIA	Lung Squamous Cell		2009	-21608	Alive	TUMOR F	1963					Complete Remission/Response		0	1963	0	1963	0	1963	0	1963
TCGA-NC	TCGA-NC	LUSC	53	MALE	WHITE	Stage IA	Lung Squamous Cell		2009	-19485	Alive	TUMOR F	37					Complete Remission/Response		0	37	0	37	0	37	0	37
TCGA-NC	TCGA-NC	LUSC	68	FEMALE	WHITE	Stage IB	Lung Squamous Cell		2009	-24981	Alive	TUMOR F	1743		New Primary Tumor		70	Complete Remission/Response		0	1743	0	1743	0	1743	1	70
TCGA-NC	TCGA-NC	LUSC	59	MALE	WHITE	Stage IIB	Lung Squamous Cell		2009	-21730	Dead	WITH TUMOR		418	New Primary Tumor		328	Complete Remission/Response		1	418	1	418	0	418	1	328
TCGA-NC	TCGA-NC	LUSC	58	FEMALE	WHITE	Stage IIB	Lung Squamous Cell		2010	-21531	Alive	TUMOR F	128					Complete Remission/Response		0	128	0	128	0	128	0	128
TCGA-NC	TCGA-NC	LUSC	73	MALE	WHITE	Stage IIA	Lung Squamous Cell		2010	-26977	Dead	TUMOR FREE		88						1	88	0	88			0	88
TCGA-NC	TCGA-NC	LUSC	76	MALE	WHITE	Stage IB	Lung Squamous Cell		2010	-27938	Alive	TUMOR F	1212					Complete Remission/Response		0	1212	0	1212	0	1212	0	1212
TCGA-NC	TCGA-NC	LUSC	77	MALE	WHITE	Stage IIA	Lung Squamous Cell		2010	-28151	Alive	TUMOR F	1499					Complete Remission/Response		0	1499	0	1499	0	1499	0	1499
TCGA-NC	TCGA-NC	LUSC	70	FEMALE	WHITE	Stage IIIA	Lung Squamous Cell		2011	-25694	Alive	TUMOR F	1336					Complete Remission/Response		0	1336	0	1336	0	1336	0	1336
TCGA-NC	TCGA-NC	LUSC	69	MALE	WHITE	Stage IV	Lung Squamous Cell		2011	-25286	Dead	WITH TUMOR		770	Distant Metastasis		317	Progressive Disease		1	770	1	770			1	317
TCGA-NC	TCGA-NC	LUSC	70	MALE	WHITE	Stage IIIA	Lung Squamous Cell		2011	-25781	Dead			448				Complete Remission/Response		1	448		448	0	448	0	448
TCGA-NC	TCGA-NC	LUSC	75	FEMALE	WHITE	Stage IIA	Lung Squamous Cell		2011	-27629	Alive	TUMOR F	1244					Complete Remission/Response		0	1244	0	1244	0	1244	0	1244
TCGA-NC	TCGA-NC	LUSC	69	MALE	WHITE	Stage IIIA	Lung Squamous Cell		2012	-25466	Alive	WITH TUN	804		Locoregional Recurrence		203	Complete Remission/Response		0	804	0	804	1	203	1	203
TCGA-NK	TCGA-NK	LUSC	77	MALE	[Unknowr	Stage IB	Lung Squamous Cell		2007	-28126	Alive	TUMOR F	2542					Stable Disease		0	2542	0	2542			0	2542
TCGA-NK	TCGA-NK	LUSC	70	MALE	WHITE	Stage IA	Lung Squamous Cell		2008	-25726	Alive	WITH TUN	1997		Locoregional Recurrence		1990	Stable Disease		0	1997	0	1997			1	1990
TCGA-NK	TCGA-NK	LUSC	73	MALE	WHITE	Stage IIA	Lung Squamous Cell		2011	-26879	Alive	TUMOR F	111					Stable Disease		0	111	0	111			0	111
TCGA-NK	TCGA-NK	LUSC	57	MALE	WHITE	Stage IIA	Lung Squamous Cell		2011	-20909	Alive	WITH TUN	511		Distant Metastasis		151	Progressive Disease		0	511	0	511			1	151
TCGA-NK	TCGA-NK	LUSC	66	MALE	BLACK OI	Stage IIIB	Lung Squamous Cell		2013	-24319	Alive	TUMOR F	13					Stable Disease		0	13	0	13			0	13
TCGA-O2	TCGA-O2	LUSC	78	MALE	WHITE	Stage I	Lung Squamous Cell		2005	-28520	Dead	TUMOR FREE		1006				[Unknown]		1	1006	0	1006			0	1006
TCGA-O2	TCGA-O2	LUSC	44	FEMALE	WHITE	Stage III	Lung Squamous Cell		2005	-16429	Dead	WITH TUMOR		113	Distant Metastasis		87	[Unknown]		1	113	1	113			1	87
TCGA-O2	TCGA-O2	LUSC	57	FEMALE	WHITE	Stage III	Lung Squamous Cell		2006	-21182	Dead	WITH TUMOR		387	Distant Metastasis		246	[Unknown]		1	387	1	387			1	246
TCGA-O2	TCGA-O2	LUSC	75	FEMALE	BLACK OI	Stage II	Lung Squamous Cell		2006	-27449	Dead	TUMOR FREE		1335	Distant Metastasis		690	[Unknown]		1	1335	0	1335			1	690
TCGA-O2	TCGA-O2	LUSC	63	MALE	BLACK OI	Stage I	Lung Squamous Cell		2006	-23156	Dead	TUMOR FREE		261						1	261	0	261			0	261
TCGA-O2	TCGA-O2	LUSC	71	FEMALE	WHITE	Stage III	Lung Squamous Cell		2005	-26211	Dead	WITH TUMOR		340	Distant Metastasis		253	[Unknown]		1	340	1	340			1	253
TCGA-XC	TCGA-XC	LUSC	77	FEMALE	BLACK OI	Stage IA	Lung Squamous Cell		2013	-28211	Dead	TUMOR FREE		6						1	6	0	6			0	6
TCGA-3H	TCGA-3H	MESO	64	MALE	WHITE	Stage II	Diffuse malignant m		2008	-23591	Dead	WITH TUMOR		1715	Distant M	Contralateral Pleura	841			1	1715	1	1715			1	841
TCGA-3H	TCGA-3H	MESO	60	MALE	WHITE	Stage III	Epithelioid mesothel		2008	-21972	Dead	WITH TUMOR		361	Intrapleur	Ipsilateral Pleura	222			1	361	1	361			1	222
TCGA-3H	TCGA-3H	MESO	53	FEMALE	WHITE	Stage III	Epithelioid mesothel		2008	-19503	Dead	WITH TUMOR		885	Distant M	Lung	107			1	885	1	885			1	107
TCGA-3H	TCGA-3H	MESO	58	MALE	WHITE	Stage III	Epithelioid mesothel		2008	-21423	Dead	WITH TUMOR		1302	Intrapleur	Ipsilateral Pleura	627			1	1302	1	1302			1	627
TCGA-3H	TCGA-3H	MESO	69	MALE	WHITE	Stage II	Epithelioid mesothel		2009	-25471	Dead	WITH TUMOR		385	Locoregic	Other, sp Ipsilateral	186		[Unknowr	1	385	1	385			1	186
TCGA-3H	TCGA-3H	MESO	79	MALE	WHITE	Stage I	Biphasic mesothelior		2009	-28925	Dead	WITH TUMOR		414	Intrapleur	Other, sp Ipsilateral	312			1	414	1	414			1	312
TCGA-3H	TCGA-3H	MESO	71	MALE	WHITE	Stage III	Epithelioid mesothel		2009	-26220	Dead	WITH TUMOR		826	Intrapleur	Ipsilateral Pleura	108		[Unknowr	1	826	1	826			1	108
TCGA-3H	TCGA-3H	MESO	62	MALE	WHITE	Stage III	Epithelioid mesothel		2010	-22702	Alive	TUMOR F	1490						[Unknowr	0	1490	0	1490			0	1490
TCGA-3U	TCGA-3U	MESO	80	MALE	WHITE	Stage III	Biphasic mesothelior		2012	-29500	Dead			142	Locoregic	Ipsilateral Chest Wall	125			1	142		142			1	125
TCGA-3U	TCGA-3U	MESO	71	FEMALE	WHITE	Stage IV	Epithelioid mesothel		2010	-26264	Alive	WITH TUN	1916		Intrapleural Progression		1257			0	1916	0	1916			1	1257
TCGA-3U	TCGA-3U	MESO	73	MALE	WHITE	Stage III	Biphasic mesothelior		2012	-26926	Dead	WITH TUMOR		536	Intrapleural Progression		365			1	536	1	536			1	365
TCGA-3U	TCGA-3U	MESO	71	MALE	WHITE	Stage IV	Biphasic mesothelior		2012	-26185	Dead	WITH TUMOR		630	Intrapleural Progression		497		R0	1	630	1	630			1	497
TCGA-3U	TCGA-3U	MESO	57	MALE	WHITE	Stage IV	Biphasic mesothelior		2010	-21034	Dead	WITH TUMOR		1156	Intrapleural Progression		798			1	1156	1	1156			1	798
TCGA-3U	TCGA-3U	MESO	68	MALE	WHITE	Stage IV	Biphasic mesothelior		2013	-25108	Dead	WITH TUMOR		860	Intrapleural Progression		404			1	860	1	860			1	404
TCGA-3U	TCGA-3U	MESO	55	MALE	WHITE	Stage III	Epithelioid mesothel		2012	-20338	Dead	WITH TUMOR		741						1	741	1	741			1	741
TCGA-LK-	TCGA-LK-	MESO	60	MALE	WHITE	Stage IV	Diffuse malignant m		2007	-21922	Dead	WITH TUMOR		546					RX	1	546	1	546			1	546
TCGA-LK-	TCGA-LK-	MESO	66	FEMALE	WHITE	Stage III	Biphasic mesothelior		2009	-24391	Dead	TUMOR FREE		168					R0	1	168	0	168	0	168	0	168
TCGA-LK-	TCGA-LK-	MESO	75	MALE	WHITE	Stage II	Epithelioid mesothel		2009	-27720	Dead	WITH TUMOR		244					R2	1	244	1	244			1	244
TCGA-LK-	TCGA-LK-	MESO	81	MALE	WHITE	Stage IA	Sarcomatoid mesoth		2009	-29798	Dead	TUMOR FREE		185					R0	1	185	0	185	0	185	0	185
TCGA-LK-	TCGA-LK-	MESO	56	FEMALE	WHITE	Stage III	Epithelioid mesothel		2011	-20757	Dead	WITH TUMOR		57					R2	1	57	1	57			1	57
TCGA-LK-	TCGA-LK-	MESO	69	MALE	WHITE	Stage III	Epithelioid mesothel		2008	-25298	Dead	TUMOR FREE		949					R0	1	949	0	949	0	949	0	949
TCGA-LK-	TCGA-LK-	MESO	68	FEMALE	WHITE	Stage III	Epithelioid mesothel		2011	-25000	Dead	WITH TUMOR		387					R1	1	387	1	387			1	387
TCGA-LK-	TCGA-LK-	MESO	66	FEMALE	WHITE	Stage III	Biphasic mesothelior		2012	-24449	Dead	WITH TUMOR		448					RX	1	448	1	448			1	448
TCGA-LK-	TCGA-LK-	MESO	64	MALE	WHITE	Stage III	Epithelioid mesothel		2012	-23606	Dead	WITH TUMOR		350					R0	1	350	1	350			1	350
TCGA-MC	TCGA-MC	MESO	55	MALE	WHITE	Stage I	Epithelioid mesothel		2002	-20336	Dead			2459	Distant M	Other, sp IPSILATER	471		R0	1	2459		2459	1	471	1	471
TCGA-MC	TCGA-MC	MESO	54	MALE	WHITE	Stage II	Epithelioid mesothel		2001	-19866	Dead	TUMOR FREE		2790	Locoregic	Ipsilateral Chest Cavi	1908		R0	1	2790	0	2790	1	1908	1	1908
TCGA-MC	TCGA-MC	MESO	60	FEMALE	WHITE	Stage II	Epithelioid mesothel		1999	-21951	Dead	WITH TUMOR		572	Distant M	Other, sp Chest	416		R1	1	572	1	572			1	416
TCGA-MC	TCGA-MC	MESO	53	MALE	WHITE	Stage III	Epithelioid mesothel		1999	-19437	Dead			709	Intrapleur	Other, sp Ipsilateral	409		[Unknowr	1	709		709			1	409
TCGA-MC	TCGA-MC	MESO	67	MALE	WHITE	Stage III	Biphasic mesothelior		2002	-24704	Dead			158	Intrapleur	Other, sp CONTRAL	113		[Unknowr	1	158		158			1	113
TCGA-MC	TCGA-MC	MESO	67	MALE	WHITE	Stage III	Biphasic mesothelior		2000	-24652	Dead			527					[Unknowr	1	527		527			0	527
TCGA-MC	TCGA-MC	MESO	63	MALE	WHITE	Stage III	Biphasic mesothelior		2002	-23110	Dead	TUMOR FREE		107	Intrapleur	Ipsilateral Pleura	99		R0	1	107	0	107	1	99	1	99
TCGA-MC	TCGA-MC	MESO	78	MALE	WHITE	Stage II	Biphasic mesothelior		2007	-28590	Dead			333	Distant M	Other, sp Bone (Sca	186		[Unknowr	1	333		333			1	186
TCGA-MC	TCGA-MC	MESO	73	FEMALE	WHITE	Stage III	Epithelioid mesothel		2007	-26790	Dead	TUMOR FREE		757	Distant M	Other, sp Chest Wa	485		R0	1	757	0	757	1	485	1	485
TCGA-MC	TCGA-MC	MESO	58	MALE	WHITE	Stage II	Epithelioid mesothel		2009	-21236	Alive	WITH TUN	2359						R1	0	2359	0	2359			0	2359
TCGA-MC	TCGA-MC	MESO	55	FEMALE	WHITE	Stage III	Epithelioid mesothel		2008	-20134	Alive	WITH TUN	2090		Distant M	Other, sp Chest Wa	665		RX	0	2090	0	2090			1	665
TCGA-MC	TCGA-MC	MESO	55	MALE	WHITE	Stage II	Epithelioid mesothel		2012	-20384	Dead	TUMOR FREE		608					R0	1	608	0	608	0	608	0	608
TCGA-NC	TCGA-NC	MESO	71	MALE	WHITE	Stage IB	Biphasic mesothelior		2012	-26212	Dead	WITH TUMOR		20					R2	1	20	1	20			1	20
TCGA-NC	TCGA-NC	MESO	66	MALE	WHITE	Stage II	Epithelioid mesothel		2012	-24410	Alive	TUMOR F	-8						R0	0	0	0	0	0	0	0	0
TCGA-SC	TCGA-SC	MESO	64	FEMALE	WHITE	Stage IV	Epithelioid mesothel		2010	-23400	Dead	WITH TUMOR		881	Locoregic	Other, sp Ipsilateral	363		R2	1	881	1	881			1	363
TCGA-SC	TCGA-SC	MESO	76	MALE	ASIAN	Stage III	Biphasic mesothelior		2012	-27917	Dead	TUMOR FREE		414					R0	1	414	0	414	0	414	0	414
TCGA-SC	TCGA-SC	MESO	58	MALE	WHITE	Stage II	Biphasic mesothelior		2012	-21411	Dead	WITH TUMOR		78					RX	1	78	1	78			1	78
TCGA-SC	TCGA-SC	MESO	60	FEMALE	WHITE	Stage III	Epithelioid mesothel		2012	-22014	Dead	WITH TUMOR		297	Distant M	Abdomen	238		R0	1	297	1	297	1	238	1	238
TCGA-SC	TCGA-SC	MESO	73	MALE	WHITE	Stage III	Epithelioid mesothel		2012	-26773	Alive	TUMOR F	1168						R2	0	1168	0	1168			0	1168
TCGA-SC	TCGA-SC	MESO	54	MALE	WHITE	Stage IV	Epithelioid mesothel		2013	-19746	Alive	WITH TUN	664						R2	0	664	0	664			0	664
TCGA-SH	TCGA-SH	MESO	66	MALE	WHITE	Stage III	Epithelioid mesothel		2011	-24459	Dead	WITH TUMOR		727	Locoregic	Other, sp Ipsilateral	507		R2	1	727	1	727			1	507
TCGA-SH	TCGA-SH	MESO	68	MALE	WHITE	Stage IV	Epithelioid mesothel		2012	-25019	Dead	WITH TUMOR		459	Locoregic	Other, sp Ipsilateral	200		R2	1	459	1	459			1	200
TCGA-SH	TCGA-SH	MESO	65	FEMALE	WHITE	Stage III	Biphasic mesothelior		2012	-23850	Dead	WITH TUMOR		243	Locoregic	Other, sp Ipsilateral	125		R2	1	243	1	243			1	125
TCGA-SH	TCGA-SH	MESO	67	MALE	WHITE	Stage II	Epithelioid mesothel		2013	-24528	Dead	WITH TUMOR		449						1	449	1	449			1	449
TCGA-SH	TCGA-SH	MESO	60	MALE	WHITE	Stage IV	Epithelioid mesothel		2013	-22135	Dead	WITH TUMOR		732					RX	1	732	1	732			1	732
TCGA-TS-	TCGA-TS-	MESO	56	MALE	WHITE	Stage IV	Epithelioid mesothel		2007	-20747	Dead			1675						1	1675		1675			0	1675
TCGA-TS-	TCGA-TS-	MESO	62	MALE	WHITE	Stage IV	Epithelioid mesothel		2009	-22720	Dead			255					[Unknowr	1	255		255			0	255
TCGA-TS-	TCGA-TS-	MESO	67	MALE	WHITE	Stage III	Epithelioid mesothel		2010	-24745	Dead			844						1	844		844			0	844
TCGA-TS-	TCGA-TS-	MESO	42	MALE	WHITE	Stage III	Epithelioid mesothel		2010	-15656	Dead			1261	Locoregic	Ipsilateral Chest Cavi	277		[Unknowr	1	1261		1261			1	277
TCGA-TS-	TCGA-TS-	MESO	52	MALE	WHITE	Stage I	Epithelioid mesothel		2010	-19191	Dead			563			494		[Unknowr	1	563		563			1	494
TCGA-TS-	TCGA-TS-	MESO	57	MALE	WHITE	Stage III	Epithelioid mesothel		2011	-20964	Dead			689					[Unknowr	1	689		689			0	689
TCGA-TS-	TCGA-TS-	MESO	61	MALE	WHITE	Stage III	Epithelioid mesothel		2011	-22338	Dead			283					[Unknowr	1	283		283			0	283
TCGA-TS-	TCGA-TS-	MESO	62	MALE	WHITE	Stage III	Epithelioid mesothel		2012	-22670	Dead			591						1	591		591			0	591
TCGA-TS-	TCGA-TS-	MESO	80	MALE	WHITE	Stage III	Epithelioid mesothel		2012	-29464	Dead			457		Other, sp Ipsilateral	301		[Unknowr	1	457		457			1	301
TCGA-TS-	TCGA-TS-	MESO	62	MALE	WHITE	Stage III	Biphasic mesothelior		2011	-22822	Dead			982					[Unknowr	1	982		982			0	982
TCGA-TS-	TCGA-TS-	MESO	69	MALE	WHITE	Stage III	Epithelioid mesothel		2007	-25486	Dead			358			325		[Unknowr	1	358		358			1	325
TCGA-TS-	TCGA-TS-	MESO	58	MALE	WHITE	Stage IV	Epithelioid mesothel		2009	-21323	Dead			328					[Unknowr	1	328		328			0	328
TCGA-TS-	TCGA-TS-	MESO	77	MALE	WHITE	Stage IA	Epithelioid mesothel		2011	-28273	Dead			795					[Unknowr	1	795		795			0	795
TCGA-TS-	TCGA-TS-	MESO	55	MALE	WHITE	Stage III	Sarcomatoid mesoth		2011	-20437	Dead								[Unknowr	1						0	
TCGA-TS-	TCGA-TS-	MESO	48	FEMALE	BLACK OI	Stage III	Epithelioid mesothel		2013	-17894	Dead			469					[Unknowr	1	469		469			0	469
TCGA-UD	TCGA-UD	MESO	50	MALE	WHITE	Stage III	Diffuse malignant m		1999	-18315	Dead	WITH TUMOR		843	Intrapleur	Ipsilateral Pleura	47		RX	1	843	1	843			1	47
TCGA-UD	TCGA-UD	MESO	46	MALE	WHITE	Stage II	Epithelioid mesothel		2004	-16913	Dead	WITH TUMOR		197					RX	1	197	1	197			1	197
TCGA-UD	TCGA-UD	MESO	71	FEMALE	WHITE	Stage III	Epithelioid mesothel		2005	-26057	Dead	WITH TUMOR		789	Intrapleur	Ipsilateral Pleura	85		R2	1	789	1	789			1	85
TCGA-UD	TCGA-UD	MESO	65	MALE	WHITE	Stage III	Diffuse malignant m		2004	-23949	Dead	WITH TUMOR		719	Intrapleur	Ipsilateral Pleura	267		R2	1	719	1	719			1	267
TCGA-UD	TCGA-UD	MESO	66	MALE	WHITE	Stage I	Epithelioid mesothel		2005	-24314	Dead	WITH TUMOR		253	Distant M	Abdomen	230		RX	1	253	1	253			1	230
TCGA-UD	TCGA-UD	MESO	67	MALE	WHITE	Stage II	Biphasic mesothelior		2006	-24571	Dead	WITH TUMOR		456	Intrapleur	Ipsilateral Pleura	126		R2	1	456	1	456			1	126
TCGA-UD	TCGA-UD	MESO	47	MALE	WHITE	Stage II	Epithelioid mesothel		2008	-17299	Dead	WITH TUMOR		406	Intrapleur	Ipsilateral Pleura	265		R2	1	406	1	406			1	265
TCGA-UT	TCGA-UT	MESO	62	MALE	WHITE	Stage III	Epithelioid mesothel		2012	-23002	Dead	WITH TUMOR		863	Intrapleur	Other, sp Mediastin	479		R0	1	863	1	863	1	479	1	479
TCGA-UT	TCGA-UT	MESO	66	MALE	WHITE	Stage II	Epithelioid mesothel		2012	-24300	Dead	TUMOR FREE		823					R0	1	823	0	823	0	823	0	823
TCGA-UT	TCGA-UT	MESO	74	MALE	WHITE	Stage I	Epithelioid mesothel		2012	-27347	Dead	WITH TUMOR		434	Intrapleur	Other, sp chest wall	121		RX	1	434	1	434			1	121
TCGA-UT	TCGA-UT	MESO	73	MALE	WHITE	Stage III	Biphasic mesothelior		2012	-26704	Dead	WITH TUMOR		76	Intrapleur	Other, sp Right Hen	44		RX	1	76	1	76			1	44
TCGA-UT	TCGA-UT	MESO	81	FEMALE	WHITE	Stage I	Epithelioid mesothel		2011	-29905	Dead	WITH TUMOR		144					RX	1	144	1	144			1	144
TCGA-XT	TCGA-XT	MESO	60	MALE	WHITE	Stage III	Biphasic mesothelior		2005	-22021	Dead	WITH TUMOR		142	Locoregic	Ipsilateral Chest Cavi	133		R0	1	142	1	142	1	133	1	133
TCGA-YS	TCGA-YS	MESO	28	FEMALE	WHITE	Stage IV	Epithelioid mesothel		2009	-10510	Dead	WITH TUMOR		39					R2	1	39	1	39			1	39
TCGA-YS	TCGA-YS	MESO	54	MALE	WHITE	Stage III	Epithelioid mesothel		2013	-19818	Dead	TUMOR FREE		133					R0	1	133	0	133	0	133	0	133
TCGA-YS	TCGA-YS	MESO	66	MALE	WHITE	Stage IV	Diffuse malignant m		2013	-24176	Dead	WITH TUMOR		84	Distant M	Bone	-131		R2	1	84	1	84			1	
TCGA-ZN	TCGA-ZN	MESO	36	MALE	WHITE	Stage IV	Epithelioid mesothel		2011	-13243	Alive	TUMOR F	261							0	261	0	261			0	261
TCGA-ZN	TCGA-ZN	MESO	51	MALE	WHITE	Stage III	Epithelioid mesothel		2012	-18697	Alive	TUMOR F	228							0	228	0	228			0	228
TCGA-ZN	TCGA-ZN	MESO	61	MALE	WHITE	Stage I	Biphasic mesothelior		2012	-22443	Alive	WITH TUN	499		Intrapleur	Other, sp Right che:	313			0	499	0	499			1	313
TCGA-ZN	TCGA-ZN	MESO	67	MALE	WHITE	Stage III	Epithelioid mesothel		2011	-24490	Alive	WITH TUN	1228		Intrapleur	Ipsilateral Pleura	1025			0	1228	0	1228			1	1025
TCGA-ZN	TCGA-ZN	MESO	70	MALE	WHITE	Stage II	Epithelioid mesothel		2012	-25714	Alive	TUMOR F	977		Intrapleur	Other, sp left chest	585			0	977	0	977			1	585
TCGA-ZN	TCGA-ZN	MESO	68	MALE	WHITE	Stage III	Biphasic mesothelior		2010	-25096	Alive	WITH TUN	1309		Intrapleur	Ipsilateral Pleura	676			0	1309	0	1309			1	676
TCGA-ZN	TCGA-ZN	MESO	61	MALE	WHITE	Stage IV	Biphasic mesothelior		2010	-22588	Dead	WITH TUMOR		84	Intrapleur	Other, sp right ches	-30			1	84	1	84			1	
TCGA-04-	TCGA-04-	OV	78	FEMALE	WHITE	Stage IIIC	Serous Cy	G3	2004	-28848	Dead	WITH TUN	1224	1336	Recurrence		459	Complete Remission/Response		1	1336	1	1336	1	459	1	459
TCGA-04-	TCGA-04-	OV	70	FEMALE	WHITE	Stage IIIC	Serous Cy	G3	2002	-25786	Dead	WITH TUN	1247	1247	Recurrence		393			1	1247	1	1247			1	393
TCGA-04-	TCGA-04-	OV	60	FEMALE	BLACK OR AFRICAN	Stage IB	Serous Cy	G2	2005	-21963	Dead	WITH TUMOR		55						1	55	1	55			1	55
TCGA-04-	TCGA-04-	OV	55	FEMALE	WHITE	Stage IIIB	Serous Cy	G3	2005	-20271	Alive	TUMOR F	1495					Complete Remission/Response		0	1495	0	1495	0	1495	0	1495
TCGA-04-	TCGA-04-	OV	78	FEMALE	WHITE	Stage IIIC	Serous Cy	G2	2005	-28626	Dead	WITH TUMOR		61						1	61	1	61			1	61
TCGA-04-	TCGA-04-	OV	78	FEMALE	WHITE	Stage IIIC	Serous Cy	G3	2005	-28789	Alive	WITH TUN	1418		Recurrence		380			0	1418	0	1418			1	380
TCGA-04-	TCGA-04-	OV	85	FEMALE	WHITE		Serous Cy	G3	2006	-31215	Alive		33					Complete Remission/Response		0	33	0	33	0	33	0	33
TCGA-04-	TCGA-04-	OV	80	FEMALE	WHITE	Stage IV	Serous Cy	G2	2006	-29501	Dead	WITH TUMOR		563				Progressive Disease		1	563	1	563			1	563
TCGA-04-	TCGA-04-	OV	72	FEMALE	WHITE	Stage IV	Serous Cy	G3	2002	-26428	Dead	WITH TUMOR		361				Progressive Disease		1	361	1	361			1	361
TCGA-04-	TCGA-04-	OV	73	FEMALE	WHITE	Stage IIIC	Serous Cy	G2	2003	-26865	Alive	TUMOR F	1993					Complete Remission/Response		0	1993	0	1993	0	1993	0	1993
TCGA-04-	TCGA-04-	OV	81	FEMALE	WHITE	Stage IV	Serous Cy	G3	2004	-29695	Alive	TUMOR F	1919					Complete Remission/Response		0	1919	0	1919			0	1919
TCGA-04-	TCGA-04-	OV	44	FEMALE	WHITE	Stage IIIB	Serous Cy	G3	2004	-16236	Dead	WITH TUMOR		1483	Recurrence		575	Complete Remission/Response		1	1483	1	1483	1	575	1	575
TCGA-04-	TCGA-04-	OV	69	FEMALE	WHITE	Stage IV	Serous Cy	G3	2004	-25213	Dead	WITH TUMOR		656	Recurrence		428	Complete Remission/Response		1	656	1	656			1	428
TCGA-04-	TCGA-04-	OV	46	FEMALE	WHITE	Stage IIIC	Serous Cy	G3	2003	-16942	Dead	WITH TUMOR		1946	Recurrence		111	Stable Disease		1	1946	1	1946			1	111
TCGA-04-	TCGA-04-	OV	76	FEMALE	WHITE	Stage IIIC	Serous Cy	G2	2004	-28045	Alive	TUMOR F	1991		Recurrence		1658	Complete Remission/Response		0	1991	0	1991	1	1658	1	1658
TCGA-04-	TCGA-04-	OV	64	FEMALE	ASIAN	Stage IIIC	Serous Cystadenocai		2003	-23611	Alive		16							0	16	0	16			0	16
TCGA-04-	TCGA-04-	OV	64	FEMALE	ASIAN	Stage IIIC	Serous Cystadenocai		2003	-23611	Alive		16							0	16	0	16			0	16
TCGA-04-	TCGA-04-	OV	62	FEMALE	WHITE	Stage IIC	Serous Cy	G3	2004	-22855	Dead	WITH TUMOR		1499	Recurrence		156	Progressive Disease		1	1499	1	1499			1	156
TCGA-04-	TCGA-04-	OV	52	FEMALE		Stage IIIB	Serous Cy	G3	2005	-18999	Alive									0		0				0	
TCGA-04-	TCGA-04-	OV	65	FEMALE		Stage IA	Serous Cy	G3	2005	-23993	Alive									0		0				0	
TCGA-04-	TCGA-04-	OV	57	FEMALE	WHITE	Stage IIIB	Serous Cy	G3	2005	-20860	Alive		989		Recurrence		927	Stable Disease		0	989	0	989			1	927
TCGA-04-	TCGA-04-	OV	59	FEMALE	WHITE	Stage IIC	Serous Cy	G3	2002	-21745	Dead	WITH TUMOR		1348	Recurrence		223			1	1348	1	1348			1	223
TCGA-04-	TCGA-04-	OV	61	FEMALE	WHITE	Stage IIIC	Serous Cy	G3	2003	-22294	Dead	WITH TUMOR		1024	Recurrence		288	Progressive Disease		1	1024	1	1024			1	288
TCGA-04-	TCGA-04-	OV	87	FEMALE	WHITE	Stage IIIB	Serous Cy	G3	2003	-31925	Alive	WITH TUN	2329		Recurrence		1328	Complete Remission/Response		0	2329	0	2329	1	1328	1	1328
TCGA-04-	TCGA-04-	OV	50	FEMALE	WHITE	Stage IIIC	Serous Cy	G3	2003	-18577	Alive	WITH TUN	3145		Progression of Disease		2997	Complete Remission/Response		0	3145	0	3145	1	2997	1	2997
TCGA-04-	TCGA-04-	OV	53	FEMALE	WHITE	Stage IIIC	Serous Cy	G2	2004	-19571	Dead	WITH TUMOR		642	Progression of Disease		315			1	642	1	642			1	315
TCGA-04-	TCGA-04-	OV	53	FEMALE	WHITE	Stage IIIC	Serous Cy	G2	2004	-19571	Dead	WITH TUMOR		642	Progression of Disease		315			1	642	1	642			1	315
TCGA-04-	TCGA-04-	OV	58	FEMALE	WHITE	Stage IIIC	Serous Cy	G3	2001	-21243	Alive	TUMOR F	2506							0	2506	0	2506			0	2506
TCGA-04-	TCGA-04-	OV	45	FEMALE	WHITE	Stage IIIA	Serous Cy	G2	2004	-16725	Dead	TUMOR FREE		1720	Recurrence		1221	Complete Remission/Response		1	1720	0	1720	1	1221	1	1221
TCGA-04-	TCGA-04-	OV	48	FEMALE	WHITE	Stage IA	Serous Cy	G3	2006	-17768	Alive	TUMOR F	1265					Complete Remission/Response		0	1265	0	1265	0	1265	0	1265
TCGA-04-	TCGA-04-	OV	48	FEMALE	WHITE	Stage IA	Serous Cy	G3	2006	-17768	Alive	TUMOR F	1265					Complete Remission/Response		0	1265	0	1265	0	1265	0	1265
TCGA-04-	TCGA-04-	OV	79	FEMALE	WHITE	Stage IIIC	Serous Cy	G3	2006	-29045	Dead	WITH TUMOR		608	Recurrence		349	Complete Remission/Response		1	608	1	608	1	349	1	349
TCGA-04-	TCGA-04-	OV	48	FEMALE		Stage IIIC	Serous Cy	G3	2007	-17671	Alive		24					[Unknown]		0	24	0	24			0	24
TCGA-04-	TCGA-04-	OV	47	FEMALE	WHITE	Stage IIIC	Serous Cy	G3	2005	-17469	Dead	WITH TUN	1167	1167	Recurrence		213	Complete Remission/Response		1	1167	1	1167	1	213	1	213
TCGA-04-	TCGA-04-	OV	68	FEMALE	WHITE	Stage IIIC	Serous Cy	G3	1995	-25013	Dead	WITH TUMOR		3622	Recurrence		350	Complete Remission/Response		1	3622	1	3622	1	350	1	350
TCGA-04-	TCGA-04-	OV	60	FEMALE	BLACK OR AFRICAN	Stage IV	Serous Cy	G3	2001	-21939	Dead	WITH TUMOR		885	Recurrence		498			1	885	1	885			1	498
TCGA-04-	TCGA-04-	OV	52	FEMALE	WHITE	Stage IIIB	Serous Cy	G2	2001	-19263	Dead	WITH TUMOR		2561	Recurrence		1020	Complete Remission/Response		1	2561	1	2561	1	1020	1	1020
TCGA-04-	TCGA-04-	OV	57	FEMALE	BLACK OR AFRICAN	Stage IV	Serous Cy	G3	2002	-20987	Dead	TUMOR FREE		1686	Recurrence		298			1	1686	0	1686			1	298
TCGA-04-	TCGA-04-	OV	48	FEMALE	WHITE	Stage IIIC	Serous Cy	G3	2002	-17725	Dead	WITH TUMOR		1487	Progression of Disease		634	Partial Remission/Response		1	1487	1	1487			1	634
TCGA-04-	TCGA-04-	OV	48	FEMALE	WHITE	Stage IIIC	Serous Cy	G3	2002	-17725	Dead	WITH TUMOR		1487	Progression of Disease		634	Partial Remission/Response		1	1487	1	1487			1	634
TCGA-04-	TCGA-04-	OV	60	FEMALE	WHITE	Stage IIIC	Serous Cy	G3	2003	-22207	Dead	WITH TUMOR		848	Recurrence		508			1	848	1	848			1	508
TCGA-04-	TCGA-04-	OV	57	FEMALE	WHITE	Stage IIIC	Serous Cy	G2	2003	-21113	Dead			871	Recurrence		412			1	871		871			1	412
TCGA-04-	TCGA-04-	OV	74	FEMALE	WHITE	Stage IIIC	Serous Cy	G3	2003	-27165	Alive		1966		Recurrence		1672			0	1966	0	1966			1	1672
TCGA-04-	TCGA-04-	OV	53	FEMALE	WHITE	Stage IIIC	Serous Cy	G3	2004	-19628	Dead			1102	Recurrence		990			1	1102		1102			1	990
TCGA-04-	TCGA-04-	OV	76	FEMALE	WHITE	Stage IIIC	Serous Cy	G2	2004	-28101	Dead	WITH TUMOR		969	Recurrence		880	Complete Remission/Response		1	969	1	969	1	880	1	880
TCGA-04-	TCGA-04-	OV	69	FEMALE	WHITE	Stage IIIC	Serous Cy	G2	1999	-25387	Dead			1451	Recurrence		808			1	1451		1451			1	808
TCGA-04-	TCGA-04-	OV	49	FEMALE	WHITE	Stage IIIB	Serous Cy	G2	1999	-17988	Dead			1380	Recurrence		819			1	1380		1380			1	819
TCGA-09-	TCGA-09-	OV	80	FEMALE	WHITE	Stage IIC	Serous Cy	G3	1998	-29262	Dead	WITH TUMOR		887	Recurrence		395	Complete Remission/Response		1	887	1	887	1	395	1	395
TCGA-09-	TCGA-09-	OV	70	FEMALE	WHITE	Stage IIIC	Serous Cy	G3	1999	-25703	Dead	WITH TUMOR		288	Recurrence		251	Complete Remission/Response		1	288	1	288	1	251	1	251
TCGA-09-	TCGA-09-	OV	55	FEMALE	WHITE	Stage IIIC	Serous Cy	G3	1999	-20440	Dead	WITH TUMOR		1757	Recurrence		190	Stable Disease		1	1757	1	1757			1	190
TCGA-09-	TCGA-09-	OV	67	FEMALE	NATIVE HAWAIIAN (	Stage IIIC	Serous Cy	G3	2001	-24473	Dead	WITH TUMOR		547				Progressive Disease		1	547	1	547			1	547
TCGA-09-	TCGA-09-	OV	56	FEMALE	WHITE	Stage IIIC	Serous Cy	G3	2003	-20705	Dead	WITH TUMOR		1082	Recurrence		274	Complete Remission/Response		1	1082	1	1082	1	274	1	274
TCGA-09-	TCGA-09-	OV	51	FEMALE	WHITE	Stage IIIC	Serous Cy	G3	1997	-18733	Dead	WITH TUMOR		304				Stable Disease		1	304	1	304			1	304
TCGA-09-	TCGA-09-	OV	75	FEMALE	WHITE	Stage IIIC	Serous Cy	G3	2000	-27486	Dead	WITH TUMOR		1169				Stable Disease		1	1169	1	1169			1	1169
TCGA-09-	TCGA-09-	OV	58	FEMALE	WHITE	Stage IV	Serous Cy	G3	2001	-21246	Dead	WITH TUMOR		2717	Recurrence		1046	Complete Remission/Response		1	2717	1	2717			1	1046
TCGA-09-	TCGA-09-	OV	37	FEMALE	WHITE	Stage IIIC	Serous Cy	G1	2002	-13739	Dead			2279				Complete Remission/Response		1	2279		2279	0	2279	0	2279
TCGA-09-	TCGA-09-	OV	73	FEMALE	WHITE	Stage IIIC	Serous Cy	G2	2002	-27010	Dead	WITH TUMOR		1266	Recurrence		468	Complete Remission/Response		1	1266	1	1266	1	468	1	468
TCGA-09-	TCGA-09-	OV	57	FEMALE	WHITE	Stage IIIC	Serous Cy	G3	2003	-21066	Alive	WITH TUN	1752		Recurrence		480	Complete Remission/Response		0	1752	0	1752	1	480	1	480
TCGA-09-	TCGA-09-	OV	61	FEMALE	WHITE	Stage IIC	Serous Cy	G2	2004	-22306	Alive	WITH TUN	1882		Recurrence		460	Complete Remission/Response		0	1882	0	1882	1	460	1	460
TCGA-09-	TCGA-09-	OV	57	FEMALE	WHITE	Stage IIIC	Serous Cy	G3	2004	-20972	Alive	WITH TUN	1684					Complete Remission/Response		0	1684	0	1684	0	1684	0	1684
TCGA-09-	TCGA-09-	OV	54	FEMALE	WHITE	Stage IIIA	Serous Cy	G3	2004	-20052	Alive	WITH TUN	928					Complete Remission/Response		0	928	0	928	0	928	0	928
TCGA-09-	TCGA-09-	OV	57	FEMALE	WHITE	Stage IIIA	Serous Cy	G3	2005	-21147	Alive	WITH TUN	547		Recurrence		547	Complete Remission/Response		0	547	0	547	1	547	1	547
TCGA-09-	TCGA-09-	OV	78	FEMALE	WHITE	Stage IIIC	Serous Cy	G1	2008	-28556	Alive	TUMOR F	178							0	178	0	178			0	178
TCGA-09-	TCGA-09-	OV	50	FEMALE	WHITE	Stage IV	Serous Cy	G3	2008	-18599	Alive	WITH TUN	92							0	92	0	92			0	92
TCGA-09-	TCGA-09-	OV	79	FEMALE	WHITE	Stage IIIC	Serous Cy	G3	2008	-28878	Alive	WITH TUN	260					Complete Remission/Response		0	260	0	260	0	260	0	260
TCGA-09-	TCGA-09-	OV	50	FEMALE		Stage IC	Serous Cy	G2	2001	-18596	Alive	TUMOR F	2967					Complete Remission/Response		0	2967	0	2967	0	2967	0	2967
TCGA-09-	TCGA-09-	OV	69	FEMALE	WHITE	Stage IC	Serous Cy	G3	2009	-25480	Alive	TUMOR F	188					Complete Remission/Response		0	188	0	188	0	188	0	188
TCGA-09-	TCGA-09-	OV	77	FEMALE	ASIAN	Stage IIB	Serous Cy	G3	2009	-28231	Alive	TUMOR F	186					Complete Remission/Response		0	186	0	186	0	186	0	186
TCGA-09-	TCGA-09-	OV	50	FEMALE	ASIAN	Stage IV	Serous Cy	G2	1996	-18285	Dead	WITH TUMOR		1069	Recurrence		631	Stable Disease		1	1069	1	1069			1	631
TCGA-09-	TCGA-09-	OV	63	FEMALE	WHITE	Stage IIIC	Serous Cy	G3	1999	-23043	Dead	WITH TUMOR		138				Stable Disease		1	138	1	138			1	138
TCGA-09-	TCGA-09-	OV	64	FEMALE	WHITE	Stage IIIC	Serous Cy	G3	1999	-23410	Alive	WITH TUN	3419		Recurrence		953	Complete Remission/Response		0	3419	0	3419	1	953	1	953
TCGA-09-	TCGA-09-	OV	65	FEMALE	WHITE	Stage IIA	Serous Cy	G2	2003	-23987	Alive	TUMOR F	2163					Complete Remission/Response		0	2163	0	2163	0	2163	0	2163
TCGA-09-	TCGA-09-	OV	42	FEMALE	WHITE	Stage IIIC	Serous Cy	G3	2004	-15650	Alive	TUMOR F	1919		Recurrence		583	Complete Remission/Response		0	1919	0	1919	1	583	1	583
TCGA-09-	TCGA-09-	OV	72	FEMALE	WHITE	Stage IIIC	Serous Cy	G3	2005	-26433	Alive	TUMOR F	1209					Complete Remission/Response		0	1209	0	1209	0	1209	0	1209
TCGA-09-	TCGA-09-	OV	58	FEMALE	BLACK OR AFRICAN	Stage IIIC	Serous Cy	G3	2005	-21285	Dead	WITH TUN	637	637				Progressive Disease		1	637	1	637			1	637
TCGA-09-	TCGA-09-	OV	48	FEMALE	WHITE	Stage IC	Serous Cy	G3	2007	-17545	Alive	TUMOR F	819					Complete Remission/Response		0	819	0	819	0	819	0	819
TCGA-09-	TCGA-09-	OV	62	FEMALE	WHITE	Stage IIIC	Serous Cy	G3	2008	-22864	Alive	TUMOR F	379					Complete Remission/Response		0	379	0	379	0	379	0	379
TCGA-10-	TCGA-10-	OV	58	FEMALE	WHITE	Stage IIIB	Serous Cy	G1	2000	-21498	Dead	WITH TUN	1652	1652	Recurrence		558	Partial Remission/Response		1	1652	1	1652			1	558
TCGA-10-	TCGA-10-	OV	63	FEMALE	WHITE	Stage IIIC	Serous Cy	G3	2000	-23307	Dead	WITH TUN	788	788	Recurrence		287	Complete Remission/Response		1	788	1	788	1	287	1	287
TCGA-10-	TCGA-10-	OV	65	FEMALE		Stage IIIC	Serous Cy	G2	2000	-23982	Dead	WITH TUN	2478	2490	Recurrence		1001	Complete Remission/Response		1	2490	1	2490	1	1001	1	1001
TCGA-10-	TCGA-10-	OV	71	FEMALE	WHITE	Stage IIIC	Serous Cy	G3	2000	-26066	Dead	WITH TUN	563	563						1	563	1	563			1	563
TCGA-10-	TCGA-10-	OV	70	FEMALE	WHITE	Stage IIIC	Serous Cy	G3	2002	-25737	Dead	WITH TUN	1011	1040	Recurrence		680	Complete Remission/Response		1	1040	1	1040	1	680	1	680
TCGA-10-	TCGA-10-	OV	44	FEMALE	WHITE	Stage IIIC	Serous Cy	G3	2002	-16262	Dead	WITH TUN	982	1000	Recurrence		300	Complete Remission/Response		1	1000	1	1000	1	300	1	300
TCGA-10-	TCGA-10-	OV	77	FEMALE	WHITE	Stage IIIC	Serous Cy	G3	2003	-28433	Dead	WITH TUN	356	446	Recurrence		356	Complete Remission/Response		1	446	1	446	1	356	1	356
TCGA-10-	TCGA-10-	OV	50	FEMALE	WHITE	Stage IIIC	Serous Cy	G3	2003	-18602	Dead	WITH TUN	119	204	Recurrence		111	Progressive Disease		1	204	1	204			1	111
TCGA-10-	TCGA-10-	OV	68	FEMALE	WHITE	Stage IIIC	Serous Cy	G3	2003	-25098	Dead	WITH TUN	1078	1078						1	1078	1	1078			1	1078
TCGA-10-	TCGA-10-	OV	69	FEMALE	BLACK OR AFRICAN	Stage IIIC	Serous Cy	G3	2005	-25454	Dead	WITH TUN	1089	1123				Partial Remission/Response		1	1123	1	1123			1	1123
TCGA-10-	TCGA-10-	OV	44	FEMALE	WHITE	Stage IIIC	Serous Cy	G3	2005	-16222	Dead	WITH TUN	460	608	Recurrence		235	Complete Remission/Response		1	608	1	608	1	235	1	235
TCGA-10-	TCGA-10-	OV	80	FEMALE	WHITE	Stage IIIC	Serous Cy	G3	2002	-29558	Dead	WITH TUN	604	636	Progression of Disease		362	Progressive Disease		1	636	1	636			1	362

TCGA-13-	TCGA-13-	OV	55	FEMALE	WHITE	Stage IV	Serous Cy	G3	2000	-20109	Dead	WITH TUM	189	189	Progression of Disease	110	Progressive Disease	1	189	1	189			1	110
TCGA-13-	TCGA-13-	OV	54	FEMALE	WHITE	Stage IIIC	Serous Cy	G3	2000	-20042	Dead	WITH TUM	748	748	Recurrence	294	Complete Remission/Response	1	748	1	748	1	294	1	294
TCGA-13-	TCGA-13-	OV	48	FEMALE	WHITE	Stage IIIC	Serous Cy	G3	2001	-17799	Dead	WITH TUM	1355	1355	Recurrence	292	Complete Remission/Response	1	1355	1	1355	1	292	1	292
TCGA-13-	TCGA-13-	OV	63	FEMALE	WHITE	Stage IIIC	Serous Cy	G3	2002	-23094	Dead	WITH TUM	1204	1204	Progression of Disease	210	Progressive Disease	1	1204	1	1204			1	210
TCGA-13-	TCGA-13-	OV	72	FEMALE	WHITE	Stage IV	Serous Cy	G3	2002	-26342	Dead	WITH TUM	83	83	Progression of Disease	83	Progressive Disease	1	83	1	83			1	83
TCGA-13-	TCGA-13-	OV	44	FEMALE	WHITE	Stage IIIC	Serous Cy	G3	2002	-16086	Dead	WITH TUM	377	377	Recurrence	190	Complete Remission/Response	1	377	1	377	1	190	1	190
TCGA-13-	TCGA-13-	OV	55	FEMALE	WHITE	Stage IIIC	Serous Cy	GX	2002	-20134	Dead	WITH TUM	946	949	Recurrence	392	Complete Remission/Response	1	949	1	949	1	392	1	392
TCGA-13-	TCGA-13-	OV	71	FEMALE	WHITE	Stage IIIC	Serous Cy	G3	2003	-26185	Dead	WITH TUM	462	462	Recurrence	253	Complete Remission/Response	1	462	1	462	1	253	1	253
TCGA-13-	TCGA-13-	OV	71	FEMALE	WHITE	Stage IIIC	Serous Cy	G3	2003	-26086	Dead			542			[Unknown]	1	542		542			0	542
TCGA-13-	TCGA-13-	OV	44	FEMALE	WHITE	Stage IIIC	Serous Cy	GX	1999	-16304	Dead	WITH TUM	1678	1678	Recurrence	653	Complete Remission/Response	1	1678	1	1678	1	653	1	653
TCGA-13-	TCGA-13-	OV	75	FEMALE	WHITE	Stage IV	Serous Cy	G3	2003	-27438	Dead	WITH TUM	76	76	Progression of Disease	34	Progressive Disease	1	76	1	76			1	34
TCGA-13-	TCGA-13-	OV	71	FEMALE	WHITE	Stage IIIC	Serous Cy	G3	2005	-25999	Dead	WITH TUM	340	340	Recurrence	293	Complete Remission/Response	1	340	1	340	1	293	1	293
TCGA-13-	TCGA-13-	OV	60	FEMALE	WHITE	Stage IV	Serous Cy	G3	2005	-22088	Dead	WITH TUM	346	346	Progression of Disease	212	Partial Remission/Response	1	346	1	346			1	212
TCGA-13-	TCGA-13-	OV	63	FEMALE	WHITE	Stage IV	Serous Cy	G3	2006	-23036	Dead		351	351				1	351		351			0	351
TCGA-13-	TCGA-13-	OV	51	FEMALE	WHITE	Stage IV	Serous Cy	G3	2006	-18697	Dead	WITH TUMOR		1156	Recurrence	424	Complete Remission/Response	1	1156	1	1156			1	424
TCGA-13-	TCGA-13-	OV	65	FEMALE	WHITE	Stage IIIC	Serous Cy	G3	2006	-23960	Alive	WITH TUM	3400			1003	Complete Remission/Response	0	3400	0	3400	1	1003	1	1003
TCGA-13-	TCGA-13-	OV	62	FEMALE	WHITE	Stage IV	Serous Cy	G3	2006	-22965	Dead	WITH TUMOR		1881	Recurrence	272	Partial Remission/Response	1	1881	1	1881			1	272
TCGA-13-	TCGA-13-	OV	50	FEMALE	WHITE	Stage IIIC	Serous Cy	G3	2006	-18574	Dead	WITH TUMOR		1389	Recurrence	668	Complete Remission/Response	1	1389	1	1389	1	668	1	668
TCGA-13-	TCGA-13-	OV	42	FEMALE	WHITE	Stage IIIC	Serous Cy	G3	2006	-15571	Dead	WITH TUMOR		1725	Recurrence	628	Complete Remission/Response	1	1725	1	1725	1	628	1	628
TCGA-13-	TCGA-13-	OV	73	FEMALE	WHITE	Stage IIIC	Serous Cy	G3	2008	-26999	Dead	WITH TUMOR		1784		398	Complete Remission/Response	1	1784	1	1784	1	398	1	398
TCGA-13-	TCGA-13-	OV	58	FEMALE	WHITE	Stage IIIC	Serous Cy	G3	2005	-21380	Dead	WITH TUMOR		1224	Recurrence	281	Complete Remission/Response	1	1224	1	1224	1	281	1	281
TCGA-13-	TCGA-13-	OV	58	FEMALE	WHITE	Stage IIIC	Serous Cy	G3	2005	-21380	Dead	WITH TUMOR		1224	Recurrence	281	Complete Remission/Response	1	1224	1	1224	1	281	1	281
TCGA-13-	TCGA-13-	OV	40	FEMALE	WHITE	Stage IIIC	Serous Cy	G3	2005	-14729	Alive	WITH TUM	3602		Recurrence	816	Complete Remission/Response	0	3602	0	3602	1	816	1	816
TCGA-13-	TCGA-13-	OV	40	FEMALE	WHITE	Stage IV	Serous Cy	G3	2006	-14708	Alive	WITH TUM	3263		Recurrence	351	Complete Remission/Response	0	3263	0	3263			1	351
TCGA-13-	TCGA-13-	OV	60	FEMALE	WHITE	Stage IIIC	Serous Cy	G3	2006	-21945	Dead	WITH TUMOR		1919	Recurrence	845	Complete Remission/Response	1	1919	1	1919	1	845	1	845
TCGA-13-	TCGA-13-	OV	66	FEMALE	WHITE	Stage IIIC	Serous Cy	G3	2007	-24403	Dead	WITH TUMOR		619	Recurrence	325	Complete Remission/Response	1	619	1	619	1	325	1	325
TCGA-13-	TCGA-13-	OV	49	FEMALE	WHITE	Stage IIIC	Serous Cy	G3	2008	-18051	Alive	WITH TUM	2121			525	Complete Remission/Response	0	2121	0	2121	1	525	1	525
TCGA-13-	TCGA-13-	OV	44	FEMALE	ASIAN	Stage IIIC	Serous Cy	G3	2008	-16171	Alive	TUMOR F	2535			1262	Complete Remission/Response	0	2535	0	2535	1	1262	1	1262
TCGA-13-	TCGA-13-	OV	52	FEMALE	WHITE	Stage IIIC	Serous Cy	G3	2008	-19087	Alive	TUMOR F	2661			1396	Complete Remission/Response	0	2661	0	2661	1	1396	1	1396
TCGA-13-	TCGA-13-	OV	46	FEMALE	BLACK OR AFRICAN	Stage IIIC	Serous Cy	G3	2008	-16812	Dead	WITH TUMOR		1399		345	Complete Remission/Response	1	1399	1	1399	1	345	1	345
TCGA-13-	TCGA-13-	OV	79	FEMALE	ASIAN	Stage IIIC	Serous Cy	G3	2008	-29075	Dead	WITH TUMOR		1552		510	Complete Remission/Response	1	1552	1	1552	1	510	1	510
TCGA-13-	TCGA-13-	OV	81	FEMALE	WHITE	Stage IIIC	Serous Cy	G3	2001	-29685	Dead	WITH TUM	1334	1334	Recurrence	337	Complete Remission/Response	1	1334	1	1334	1	337	1	337
TCGA-13-	TCGA-13-	OV	73	FEMALE	WHITE	Stage IIIC	Serous Cy	G3	2003	-27021	Dead	WITH TUM	1073	1073	Recurrence	329	Complete Remission/Response	1	1073	1	1073	1	329	1	329
TCGA-13-	TCGA-13-	OV	57	FEMALE	WHITE	Stage IIIC	Serous Cy	G3	2005	-21014	Dead	WITH TUM	1073	1074	Progression of Disease	177	Progressive Disease	1	1074	1	1074			1	177
TCGA-13-	TCGA-13-	OV	54	FEMALE	WHITE	Stage IIIC	Serous Cy	G3	2005	-19890	Dead		414	414				1	414		414			0	414
TCGA-13-	TCGA-13-	OV	61	FEMALE	WHITE	Stage IIIC	Serous Cy	G3	2002	-22395	Dead	WITH TUM	2097	2097	Recurrence	819	Complete Remission/Response	1	2097	1	2097	1	819	1	819
TCGA-13-	TCGA-13-	OV	39	FEMALE	WHITE	Stage IIIC	Serous Cy	G3	1999	-14538	Dead	WITH TUM	3260	3260	Recurrence	1251	Complete Remission/Response	1	3260	1	3260	1	1251	1	1251
TCGA-13-	TCGA-13-	OV	70	FEMALE	WHITE	Stage IIIC	Serous Cy	G3	2000	-25763	Alive		3388				Complete Remission/Response	0	3388	0	3388	0	3388	0	3388
TCGA-13-	TCGA-13-	OV	67	FEMALE	WHITE	Stage IIIC	Serous Cy	G3	2002	-24724	Alive	TUMOR F	4665				Complete Remission/Response	0	4665	0	4665	0	4665	0	4665
TCGA-13-	TCGA-13-	OV	42	FEMALE	WHITE	Stage IIIC	Serous Cy	G3	2002	-15669	Dead	WITH TUM	2028	2028	Recurrence	514	Complete Remission/Response	1	2028	1	2028	1	514	1	514
TCGA-13-	TCGA-13-	OV	78	FEMALE	WHITE	Stage IIIC	Serous Cy	G3	2003	-28498	Dead	TUMOR FREE		2811			[Discrepancy]	1	2811	0	2811			0	2811
TCGA-13-	TCGA-13-	OV	75	FEMALE	WHITE	Stage IV	Serous Cy	GX	2003	-27674	Dead	TUMOR FREE		2894			Complete Remission/Response	1	2894	0	2894			0	2894
TCGA-13-	TCGA-13-	OV	56	FEMALE	WHITE	Stage IIIC	Serous Cy	G3	2003	-20770	Alive	TUMOR F	4424				Complete Remission/Response	0	4424	0	4424	0	4424	0	4424
TCGA-13-	TCGA-13-	OV	73	FEMALE	WHITE	Stage IV	Serous Cy	G3	2003	-26886	Dead	WITH TUMOR		3128	Recurrence	762	Complete Remission/Response	1	3128	1	3128			1	762
TCGA-13-	TCGA-13-	OV	48	FEMALE	BLACK OR AFRICAN	Stage IIIC	Serous Cy	G3	2003	-17573	Dead	WITH TUM	1319	1319	Recurrence	461	Partial Remission/Response	1	1319	1	1319			1	461
TCGA-13-	TCGA-13-	OV	53	FEMALE	WHITE	Stage IIIC	Serous Cy	G3	2003	-19466	Dead	WITH TUM	1509	1509	Recurrence	565	Complete Remission/Response	1	1509	1	1509	1	565	1	565
TCGA-13-	TCGA-13-	OV	54	FEMALE	WHITE	Stage IIIC	Serous Cy	G3	2004	-19894	Dead	WITH TUMOR		2182	Recurrence	554	Complete Remission/Response	1	2182	1	2182	1	554	1	554
TCGA-13-	TCGA-13-	OV	60	FEMALE	WHITE	Stage IIIC	Serous Cy	G3	2004	-22031	Dead	WITH TUMOR		2012	Recurrence	547	Complete Remission/Response	1	2012	1	2012	1	547	1	547
TCGA-13-	TCGA-13-	OV	59	FEMALE	WHITE	Stage IIIC	Serous Cy	G3	2004	-21698	Alive	TUMOR F	4077				Complete Remission/Response	0	4077	0	4077	0	4077	0	4077
TCGA-13-	TCGA-13-	OV	41	FEMALE	ASIAN	Stage IIIC	Serous Cy	G3	2004	-15333	Alive	WITH TUM	589		Recurrence	390	Complete Remission/Response	0	589	0	589	1	390	1	390
TCGA-13-	TCGA-13-	OV	42	FEMALE	WHITE	Stage IV	Serous Cy	G3	2004	-15705	Alive	TUMOR F	3887				Complete Remission/Response	0	3887	0	3887			0	3887
TCGA-13-	TCGA-13-	OV	63	FEMALE	WHITE	Stage IIIC	Serous Cy	G3	2004	-23278	Dead	WITH TUMOR		1532	Progression of Disease	330	Partial Remission/Response	1	1532	1	1532			1	330
TCGA-13-	TCGA-13-	OV	51	FEMALE	WHITE	Stage IIIC	Serous Cy	G3	2005	-18757	Alive	WITH TUM	3746			1535	Complete Remission/Response	0	3746	0	3746	1	1535	1	1535
TCGA-13-	TCGA-13-	OV	50	FEMALE	WHITE	Stage IIIC	Serous Cy	G3	2005	-18383	Alive	TUMOR F	3619				Complete Remission/Response	0	3619	0	3619	0	3619	0	3619
TCGA-13-	TCGA-13-	OV	58	FEMALE	WHITE	Stage IV	Serous Cy	G3	2005	-21422	Dead	WITH TUMOR		1843	Recurrence	552	Complete Remission/Response	1	1843	1	1843			1	552
TCGA-13-	TCGA-13-	OV	58	FEMALE	WHITE	Stage IIIC	Serous Cy	G3	2005	-21473	Alive	WITH TUM	3548			1212	Complete Remission/Response	0	3548	0	3548	1	1212	1	1212
TCGA-13-	TCGA-13-	OV	55	FEMALE	WHITE	Stage IV	Serous Cy	G3	2005	-20276	Dead	WITH TUMOR		1355	Progression of Disease	279	Partial Remission/Response	1	1355	1	1355			1	279
TCGA-13-	TCGA-13-	OV	58	FEMALE	WHITE	Stage IIIC	Serous Cy	G3	2006	-21427	Dead	WITH TUMOR		1145	Recurrence	469	Complete Remission/Response	1	1145	1	1145	1	469	1	469
TCGA-13-	TCGA-13-	OV	53	FEMALE	WHITE	Stage IIIC	Serous Cy	G3	2006	-19573	Alive	WITH TUM	3325		Recurrence	871	Complete Remission/Response	0	3325	0	3325	1	871	1	871
TCGA-13-	TCGA-13-	OV	53	FEMALE	WHITE	Stage IIIC	Serous Cy	G3	2006	-19573	Alive	WITH TUM	3325		Recurrence	871	Complete Remission/Response	0	3325	0	3325	1	871	1	871
TCGA-13-	TCGA-13-	OV	49	FEMALE	WHITE	Stage IIIC	Serous Cy	G3	2006	-18189	Alive	TUMOR F	1785				Complete Remission/Response	0	1785	0	1785	0	1785	0	1785
TCGA-13-	TCGA-13-	OV	52	FEMALE	WHITE	Stage IIIC	Serous Cy	G3	2007	-19337	Alive	TUMOR F	2571				Complete Remission/Response	0	2571	0	2571	0	2571	0	2571
TCGA-13-	TCGA-13-	OV	65	FEMALE	WHITE	Stage IIIC	Serous Cy	G3	2007	-24064	Dead	WITH TUMOR		1484	Recurrence	297	Complete Remission/Response	1	1484	1	1484	1	297	1	297
TCGA-13-	TCGA-13-	OV	72	FEMALE	WHITE	Stage IIIC	Serous Cy	G3	2008	-26606	Dead	TUMOR FREE		1483			Complete Remission/Response	1	1483	0	1483	0	1483	0	1483
TCGA-13-	TCGA-13-	OV	74	FEMALE	WHITE	Stage IIIC	Serous Cy	G3	2008	-27081	Alive	TUMOR F	2547			1719	Complete Remission/Response	0	2547	0	2547	1	1719	1	1719
TCGA-13-	TCGA-13-	OV	45	FEMALE	WHITE	Stage IV	Serous Cy	G3	2008	-16670	Alive	WITH TUM	2614			962	Complete Remission/Response	0	2614	0	2614			1	962
TCGA-13-	TCGA-13-	OV	48	FEMALE	WHITE	Stage IIIC	Serous Cy	G3	2008	-17697	Dead	WITH TUMOR		2345		418	Complete Remission/Response	1	2345	1	2345	1	418	1	418
TCGA-13-	TCGA-13-	OV	48	FEMALE	WHITE	Stage IIIC	Serous Cy	G3	2008	-17784	Alive	WITH TUM	2469			1581	Complete Remission/Response	0	2469	0	2469	1	1581	1	1581
TCGA-13-	TCGA-13-	OV	49	FEMALE	WHITE	Stage IV	Serous Cy	G3	2008	-18218	Dead	WITH TUMOR		868		228	Complete Remission/Response	1	868	1	868			1	228
TCGA-13-	TCGA-13-	OV	51	FEMALE	WHITE	Stage IIIC	Serous Cy	G3	2008	-18896	Alive	WITH TUM	2534			1333	Complete Remission/Response	0	2534	0	2534	1	1333	1	1333
TCGA-13-	TCGA-13-	OV	59	FEMALE	WHITE	Stage IIIC	Serous Cy	G3	2008	-21635	Dead	WITH TUMOR		1680		765	Partial Remission/Response	1	1680	1	1680			1	765
TCGA-13-	TCGA-13-	OV	73	FEMALE	WHITE	Stage IIIC	Serous Cy	G3	2008	-26836	Dead	WITH TUMOR		1742		690		1	1742	1	1742			1	690
TCGA-13-	TCGA-13-	OV	57	FEMALE	WHITE	Stage IV	Serous Cy	GX	2008	-20950	Alive	WITH TUM	2464			319	Complete Remission/Response	0	2464	0	2464			1	319
TCGA-13-	TCGA-13-	OV	81	FEMALE	WHITE	Stage IIIC	Serous Cy	G3	2008	-29874	Dead	WITH TUMOR		531		238	Partial Remission/Response	1	531	1	531			1	238
TCGA-13-	TCGA-13-	OV	41	FEMALE	WHITE	Stage IV	Serous Cy	G3	2008	-15119	Dead	WITH TUMOR		984		409	Complete Remission/Response	1	984	1	984			1	409
TCGA-13-	TCGA-13-	OV	49	FEMALE	WHITE	Stage IV	Serous Cy	G2	2000	-18111	Dead	WITH TUM	1662	1662	Progression of Disease	235	Progressive Disease	1	1662	1	1662			1	235
TCGA-13-	TCGA-13-	OV	76	FEMALE	WHITE	Stage IIIC	Serous Cy	G2	2000	-27898	Dead	TUMOR F	2648	2648			Complete Remission/Response	1	2648	0	2648	0	2648	0	2648
TCGA-13-	TCGA-13-	OV	52	FEMALE	WHITE	Stage IV	Serous Cy	G2	2000	-19178	Dead	WITH TUM	1877	1877	Recurrence	602	Partial Remission/Response	1	1877	1	1877			1	602
TCGA-13-	TCGA-13-	OV	61	FEMALE	WHITE	Stage IIIC	Serous Cy	G3	2000	-22342	Dead	WITH TUM	895	895	Recurrence	297	Partial Remission/Response	1	895	1	895			1	297
TCGA-13-	TCGA-13-	OV	62	FEMALE	WHITE	Stage IIIC	Serous Cy	G3	2000	-22698	Dead	WITH TUMOR		3785	Recurrence	497	Complete Remission/Response	1	3785	1	3785	1	497	1	497
TCGA-13-	TCGA-13-	OV	48	FEMALE	WHITE	Stage IV	Serous Cy	G2	2001	-17642	Dead	WITH TUM	629	629	Progression of Disease	170	Partial Remission/Response	1	629	1	629			1	170
TCGA-13-	TCGA-13-	OV	74	FEMALE	WHITE	Stage IV	Serous Cy	GX	2002	-27176	Dead	WITH TUM	681	681	Recurrence	412	Complete Remission/Response	1	681	1	681			1	412
TCGA-13-	TCGA-13-	OV	59	FEMALE	WHITE	Stage IV	Serous Cy	G3	2002	-21726	Dead	WITH TUM	2154	2154	Recurrence	351	Complete Remission/Response	1	2154	1	2154			1	351
TCGA-13-	TCGA-13-	OV	70	FEMALE	WHITE	Stage IIIC	Serous Cy	G2	2002	-25768	Dead	TUMOR FREE		2553	Recurrence	806	Complete Remission/Response	1	2553	0	2553	1	806	1	806
TCGA-13-	TCGA-13-	OV	70	FEMALE	WHITE	Stage IIIC	Serous Cy	G2	2002	-25768	Dead	TUMOR FREE		2553	Recurrence	806	Complete Remission/Response	1	2553	0	2553	1	806	1	806
TCGA-13-	TCGA-13-	OV	55	FEMALE	ASIAN	Stage IIIC	Serous Cy	G3	2002	-20262	Dead	WITH TUM	1595	1595	Recurrence	508	Partial Remission/Response	1	1595	1	1595			1	508
TCGA-13-	TCGA-13-	OV	66	FEMALE	WHITE	Stage IIIC	Serous Cy	G3	2002	-24332	Dead	TUMOR FREE		3819			Complete Remission/Response	1	3819	0	3819	0	3819	0	3819
TCGA-13-	TCGA-13-	OV	43	FEMALE	WHITE	Stage IV	Serous Cy	G3	2004	-15993	Dead	WITH TUMOR		2856	Recurrence	693	Complete Remission/Response	1	2856	1	2856			1	693
TCGA-13-	TCGA-13-	OV	60	FEMALE	WHITE	Stage IIIC	Serous Cy	G2	2004	-22046	Dead	WITH TUMOR		2749	Recurrence	977	Complete Remission/Response	1	2749	1	2749	1	977	1	977
TCGA-13-	TCGA-13-	OV	65	FEMALE	WHITE	Stage IIIC	Serous Cy	G3	2004	-23993	Dead		129	129				1	129		129			0	129
TCGA-13-	TCGA-13-	OV	47	FEMALE	WHITE	Stage IIIC	Serous Cy	G3	2004	-17528	Alive	TUMOR F	3871		Recurrence	813	Complete Remission/Response	0	3871	0	3871	1	813	1	813
TCGA-13-	TCGA-13-	OV	73	FEMALE	WHITE	Stage IIIC	Serous Cy	G3	2006	-26820	Dead	WITH TUMOR		2012	Progression of Disease	534	Partial Remission/Response	1	2012	1	2012			1	534
TCGA-13-	TCGA-13-	OV	56	FEMALE	WHITE	Stage IIIC	Serous Cy	G3	2005	-20668	Alive	WITH TUM	3500		Recurrence	583	Complete Remission/Response	0	3500	0	3500	1	583	1	583
TCGA-13-	TCGA-13-	OV	71	FEMALE	WHITE	Stage IIIC	Serous Cy	G3	2006	-26172	Dead	WITH TUM	425	425	Recurrence	253	Partial Remission/Response	1	425	1	425			1	253
TCGA-13-	TCGA-13-	OV	50	FEMALE	WHITE	Stage IV	Serous Cy	G3	2006	-18393	Dead	WITH TUMOR		1314	Progression of Disease	392	Partial Remission/Response	1	1314	1	1314			1	392
TCGA-13-	TCGA-13-	OV	68	FEMALE	WHITE	Stage IIIC	Serous Cy	G3	2007	-25008	Dead	WITH TUMOR		1059		548	Complete Remission/Response	1	1059	1	1059	1	548	1	548
TCGA-13-	TCGA-13-	OV	63	FEMALE	WHITE	Stage IIIC	Serous Cy	G3	2008	-23238	Alive	WITH TUM	1998			1561	Complete Remission/Response	0	1998	0	1998	1	1561	1	1561
TCGA-13-	TCGA-13-	OV	45	FEMALE	WHITE	Stage IIIC	Serous Cy	G3	2008	-16734	Dead	WITH TUMOR		1039		666	Complete Remission/Response	1	1039	1	1039	1	666	1	666
TCGA-13-	TCGA-13-	OV	77	FEMALE	WHITE	Stage IIIC	Serous Cy	G3	2008	-28445	Dead	WITH TUMOR		1993		324	Complete Remission/Response	1	1993	1	1993	1	324	1	324
TCGA-13-	TCGA-13-	OV	64	FEMALE	WHITE	Stage IV	Serous Cy	G3	2008	-23481	Alive	WITH TUM	2438			578	Complete Remission/Response	0	2438	0	2438			1	578
TCGA-13-	TCGA-13-	OV	62	FEMALE	WHITE	Stage IIIC	Serous Cy	G3	2008	-22708	Dead	WITH TUMOR		1359		1189		1	1359	1	1359			1	1189
TCGA-13-	TCGA-13-	OV	52	FEMALE	ASIAN	Stage IV	Serous Cy	G3	2008	-19258	Dead	WITH TUMOR		1650		459	Partial Remission/Response	1	1650	1	1650			1	459
TCGA-13-	TCGA-13-	OV	49	FEMALE	WHITE	Stage IIIC	Serous Cy	G3	2008	-18134	Alive	WITH TUM	442			427		0	442	0	442			1	427
TCGA-13-	TCGA-13-	OV	56	FEMALE	WHITE	Stage IIIC	Serous Cy	G3	2001	-20771	Dead	WITH TUM	1024	1024	Recurrence	541	Partial Remission/Response	1	1024	1	1024			1	541
TCGA-13-	TCGA-13-	OV	56	FEMALE	WHITE	Stage IIIC	Serous Cy	G3	2001	-20771	Dead	WITH TUM	1024	1024	Recurrence	541	Partial Remission/Response	1	1024	1	1024			1	541
TCGA-13-	TCGA-13-	OV	59	FEMALE	WHITE	Stage IIC	Serous Cy	G3	2004	-21635	Dead	WITH TUMOR		2025	Recurrence	429	Complete Remission/Response	1	2025	1	2025	1	429	1	429
TCGA-13-	TCGA-13-	OV	59	FEMALE	WHITE	Stage IIC	Serous Cy	G3	2004	-21635	Dead	WITH TUMOR		2025	Recurrence	429	Complete Remission/Response	1	2025	1	2025	1	429	1	429
TCGA-13-	TCGA-13-	OV	59	FEMALE	WHITE	Stage IIIC	Serous Cy	G3	2008	-21554	Alive	WITH TUM	2434			897	Complete Remission/Response	0	2434	0	2434	1	897	1	897
TCGA-13-	TCGA-13-	OV	50	FEMALE	WHITE	Stage IIIC	Serous Cy	G3	2009	-18449	Alive	WITH TUM	2387			1005	Complete Remission/Response	0	2387	0	2387	1	1005	1	1005
TCGA-13-	TCGA-13-	OV	51	FEMALE	WHITE	Stage IV	Serous Cy	G3	2009	-18635	Alive	WITH TUM	2369			1475	Complete Remission/Response	0	2369	0	2369			1	1475
TCGA-13-	TCGA-13-	OV	58	FEMALE	WHITE	Stage IIIC	Serous Cy	G3	2009	-21543	Alive	TUMOR F	2339				Complete Remission/Response	0	2339	0	2339	0	2339	0	2339
TCGA-13-	TCGA-13-	OV	71	FEMALE	BLACK OR AFRICAN	Stage IIIC	Serous Cy	G3	2009	-25973	Alive	WITH TUM	1993			946	Complete Remission/Response	0	1993	0	1993	1	946	1	946
TCGA-13-	TCGA-13-	OV	49	FEMALE	WHITE	Stage IV	Serous Cy	G3	2009	-18170	Dead	WITH TUMOR		2133		1028	Complete Remission/Response	1	2133	1	2133			1	1028
TCGA-13-	TCGA-13-	OV	63	FEMALE	WHITE	Stage IV	Serous Cy	G3	2009	-23367	Dead	WITH TUMOR		773		322	Complete Remission/Response	1	773	1	773			1	322
TCGA-13-	TCGA-13-	OV	67	FEMALE	BLACK OR AFRICAN	Stage IIIC	Serous Cy	G3	2009	-24743	Alive	TUMOR F	2143				Complete Remission/Response	0	2143	0	2143	0	2143	0	2143
TCGA-13-	TCGA-13-	OV	60	FEMALE	BLACK OR AFRICAN	Stage IV	Serous Cy	G3	2009	-22256	Dead	WITH TUMOR		256			Partial Remission/Response	1	256	1	256			1	256
TCGA-20-	TCGA-20-	OV	61	FEMALE	WHITE	Stage IIIC	Serous Cy	G3	2004	-22287	Dead	WITH TUMOR		701	Recurrence	442		1	701	1	701			1	442
TCGA-20-	TCGA-20-	OV	74	FEMALE	WHITE	Stage IIIC	Serous Cy	G3	2005	-27170	Alive	WITH TUM	789		Recurrence	871		0	789	0	789			1	871
TCGA-20-	TCGA-20-	OV	78	FEMALE	WHITE	Stage IIB	Serous Cy	G3	2006	-28694	Alive	TUMOR F	797				Complete Remission/Response	0	797	0	797	0	797	0	797
TCGA-20-	TCGA-20-	OV	59	FEMALE	WHITE	Stage IIIC	Serous Cy	G2	2001	-21572	Alive	WITH TUM	2196					0	2196	0	2196			0	2196
TCGA-20-	TCGA-20-	OV	56	FEMALE	WHITE	Stage IIIC	Serous Cy	GX	2007	-20583	Alive	TUMOR F	837				Complete Remission/Response	0	837	0	837	0	837	0	837
TCGA-20-	TCGA-20-	OV	65	FEMALE	WHITE	Stage IIIC	Serous Cy	G3	2007	-24006	Alive	TUMOR F	772		Recurrence	618	Stable Disease	0	772	0	772			1	618
TCGA-20-	TCGA-20-	OV	51	FEMALE	WHITE	Stage IIIC	Serous Cy	G3	2007	-18755	Alive	TUMOR F	581				Complete Remission/Response	0	581	0	581	0	581	0	581
TCGA-20-	TCGA-20-	OV	45	FEMALE	WHITE	Stage IIIC	Serous Cy	G3	2008	-16745	Alive	TUMOR F	508				Complete Remission/Response	0	508	0	508	0	508	0	508
TCGA-20-	TCGA-20-	OV	75	FEMALE	WHITE	Stage IIIC	Serous Cy	G3	2008	-27588	Alive	TUMOR F	89				Complete Remission/Response	0	89	0	89	0	89	0	89
TCGA-20-	TCGA-20-	OV	46	FEMALE	WHITE	Stage IV	Serous Cy	G3	2008	-16961	Alive	TUMOR F	81				Complete Remission/Response	0	81	0	81			0	81
TCGA-23-	TCGA-23-	OV	45	FEMALE	WHITE	Stage IV	Serous Cy	G3	2004	-16484	Dead	WITH TUM	1446	1446			Partial Remission/Response	1	1446	1	1446			1	1446
TCGA-23-	TCGA-23-	OV	67	FEMALE	WHITE	Stage IIIC	Serous Cy	G3	2003	-24798	Dead	WITH TUMOR		1511	Recurrence	450	Complete Remission/Response	1	1511	1	1511	1	450	1	450
TCGA-23-	TCGA-23-	OV	65	FEMALE	WHITE	Stage IIIC	Serous Cy	G3	2005	-23826	Alive	WITH TUM	1233		Recurrence	490	Complete Remission/Response	0	1233	0	1233	1	490	1	490
TCGA-23-	TCGA-23-	OV	52	FEMALE	WHITE	Stage IV	Serous Cy	G3	2007	-19052	Alive	TUMOR F	468				Complete Remission/Response	0	468	0	468			0	468
TCGA-23-	TCGA-23-	OV	45	FEMALE	WHITE	Stage IIIC	Serous Cy	G3	2004	-16442	Alive	WITH TUM	816		Recurrence	797	Complete Remission/Response	0	816	0	816	1	797	1	797
TCGA-23-	TCGA-23-	OV	48	FEMALE	WHITE	Stage IIIC	Serous Cy	G3	2005	-17588	Dead		966	976	Recurrence	114	Progressive Disease	1	976		976			1	114
TCGA-23-	TCGA-23-	OV	43	FEMALE	WHITE	Stage IIIC	Serous Cy	G3	2004	-16018	Alive	WITH TUM	1503		Recurrence	133	Complete Remission/Response	0	1503	0	1503	1	133	1	133
TCGA-23-	TCGA-23-	OV	46	FEMALE	WHITE	Stage IIIC	Serous Cy	G3	2007	-16889	Alive	TUMOR F	268				Complete Remission/Response	0	268	0	268	0	268	0	268
TCGA-23-	TCGA-23-	OV	64	FEMALE	WHITE	Stage IIIC	Serous Cy	G3	2006	-23416	Alive	TUMOR F	886		Recurrence	544	Complete Remission/Response	0	886	0	886	1	544	1	544
TCGA-23-	TCGA-23-	OV	60	FEMALE	WHITE	Stage IV	Serous Cy	G3	2003	-22225	Dead	WITH TUM	575	575			Partial Remission/Response	1	575	1	575			1	575
TCGA-23-	TCGA-23-	OV	73	FEMALE	BLACK OR AFRICAN	Stage IV	Serous Cy	G3	2002	-26715	Dead	WITH TUM	68	84				1	84	1	84			1	84
TCGA-23-	TCGA-23-	OV	59	FEMALE	WHITE	Stage IV	Serous Cy	G3	1999	-21561	Dead	WITH TUM	9	9				1	9	1	9			1	9
TCGA-23-	TCGA-23-	OV	62	FEMALE	WHITE	Stage IIIC	Serous Cy	G3	1999	-23002	Dead	WITH TUM	1562	1562	Recurrence	987	Partial Remission/Response	1	1562	1	1562			1	987
TCGA-23-	TCGA-23-	OV	42	FEMALE	WHITE	Stage IIIC	Serous Cy	G3	2003	-15383	Alive	TUMOR F	1658		Recurrence	326	Complete Remission/Response	0	1658	0	1658	1	326	1	326
TCGA-23-	TCGA-23-	OV	63	FEMALE	WHITE	Stage IIIC	Serous Cy	G3	2008	-23171	Alive		98					0	98	0	98			0	98
TCGA-23-	TCGA-23-	OV	48	FEMALE	WHITE	Stage IV	Serous Cy	G3	2002	-17791	Dead	WITH TUMOR		949			Partial Remission/Response	1	949	1	949			1	949
TCGA-23-	TCGA-23-	OV	55	FEMALE	WHITE	Stage IIIC	Serous Cy	G3	2000	-20409	Dead	WITH TUM	2089	2089	Recurrence	634	Complete Remission/Response	1	2089	1	2089	1	634	1	634
TCGA-23-	TCGA-23-	OV	83	FEMALE	WHITE	Stage IIIC	Serous Cy	G3	2008	-30464	Dead	WITH TUMOR		592	Recurrence	286	Complete Remission/Response	1	592	1	592	1	286	1	286
TCGA-23-	TCGA-23-	OV	42	FEMALE	WHITE	Stage IIIC	Serous Cy	G3	1998	-15690	Dead	WITH TUMOR		1013	Recurrence	418	Complete Remission/Response	1	1013	1	1013	1	418	1	418
TCGA-23-	TCGA-23-	OV	45	FEMALE	WHITE	Stage IIIC	Serous Cy	G3	2001	-16471	Alive	TUMOR F	2616				Complete Remission/Response	0	2616	0	2616	0	2616	0	2616
TCGA-23-	TCGA-23-	OV	64	FEMALE	WHITE	Stage IIIC	Serous Cy	G3	1997	-23686	Alive	TUMOR F	3953		Recurrence	3378	Complete Remission/Response	0	3953	0	3953	1	3378	1	3378
TCGA-23-	TCGA-23-	OV	60	FEMALE	WHITE	Stage IIIC	Serous Cy	G3	2008	-22148	Alive		130				Complete Remission/Response	0	130	0	130	0	130	0	130
TCGA-23-	TCGA-23-	OV	51	FEMALE		Stage IIIC	Serous Cy	G3	2008	-18983	Alive		194					0	194	0	194			0	194
TCGA-23-	TCGA-23-	OV	53	FEMALE	WHITE	Stage IIIC	Serous Cy	G3	1998	-19537	Dead	WITH TUMOR		1189	Recurrence	447	Complete Remission/Response	1	1189	1	1189	1	447	1	447
TCGA-23-	TCGA-23-	OV	59	FEMALE	WHITE	Stage IIIC	Serous Cy	G3	1998	-21552	Dead	WITH TUMOR		1018			Progressive Disease	1	1018	1	1018			1	1018
TCGA-23-	TCGA-23-	OV	62	FEMALE	WHITE	Stage IIIC	Serous Cy	G3	1999	-23005	Dead	WITH TUMOR		1768	Recurrence	641	Complete Remission/Response	1	1768	1	1768	1	641	1	641
TCGA-23-	TCGA-23-	OV	63	FEMALE	WHITE	Stage IIC	Serous Cy	G3	2009	-23111	Alive		16					0	16	0	16			0	16
TCGA-23-	TCGA-23-	OV	58	FEMALE	WHITE	Stage IV	Serous Cy	G3	2000	-21523	Dead	WITH TUMOR		759	Recurrence	476	Complete Remission/Response	1	759	1	759			1	476
TCGA-23-	TCGA-23-	OV	45	FEMALE	WHITE	Stage IIIC	Serous Cy	G3	1999	-16552	Alive	TUMOR F	3525		Recurrence	1232	Complete Remission/Response	0	3525	0	3525	1	1232	1	1232
TCGA-23-	TCGA-23-	OV	66	FEMALE	WHITE	Stage IIIC	Serous Cy	G3	2001	-24223	Alive	TUMOR F	2661				Complete Remission/Response	0	2661	0	2661	0	2661	0	2661
TCGA-23-	TCGA-23-	OV	46	FEMALE	WHITE	Stage IIIC	Serous Cy	G3	2001	-17122	Alive	TUMOR F	2788		Recurrence	283	Complete Remission/Response	0	2788	0	2788	1	283	1	283
TCGA-23-	TCGA-23-	OV	49	FEMALE	WHITE	Stage IV	Serous Cy	G3	2000	-18162	Dead	WITH TUMOR		2342			Progressive Disease	1	2342	1	2342			1	2342
TCGA-23-	TCGA-23-	OV	45	FEMALE	WHITE	Stage IV	Serous Cy	G3	2000	-16493	Dead	WITH TUMOR		1516	Recurrence	619	Complete Remission/Response	1	1516	1	1516			1	619
TCGA-23-	TCGA-23-	OV	85	FEMALE	WHITE	Stage IIIC	Serous Cy	G3	2009	-31232	Alive		84					0	84	0	84			0	84
TCGA-23-	TCGA-23-	OV	74	FEMALE	WHITE	Stage IIIC	Serous Cy	G3	2009	-27093	Alive	TUMOR F	143				Complete Remission/Response	0	143	0	143	0	143	0	143
TCGA-23-	TCGA-23-	OV	54	FEMALE	ASIAN	Stage IIIC	Serous Cy	G3	2009	-19758	Alive		137					0	137	0	137			0	137
TCGA-23-	TCGA-23-	OV	49	FEMALE	ASIAN	Stage IIIC	Serous Cy	G3	2009	-18205	Alive		135					0	135	0	135			0	135
TCGA-23-	TCGA-23-	OV	60	FEMALE	WHITE	Stage IIIC	Serous Cy	G3	2009	-22210	Alive		116					0	116	0	116			0	116
TCGA-24-	TCGA-24-	OV	78	FEMALE	BLACK OR AFRICAN	Stage IIIC	Serous Cy	G3	2008	-28529	Alive	WITH TUM	232				Stable Disease	0	232	0	232			0	232
TCGA-24-	TCGA-24-	OV	59	FEMALE	WHITE	Stage IIIC	Serous Cy	G3	2005	-21664	Dead	WITH TUM	598				Partial Remission/Response	1		1				1	
TCGA-24-	TCGA-24-	OV	63	FEMALE	WHITE	Stage IIIC	Serous Cy	G3	2005	-23276	Dead	WITH TUM	354	354	Recurrence	280	Partial Remission/Response	1	354	1	354			1	280
TCGA-24-	TCGA-24-	OV	58	FEMALE	WHITE	Stage IIIC	Serous Cy	G3	2004	-21417	Dead	WITH TUM	663	663			Partial Remission/Response	1	663	1	663			1	663
TCGA-24-	TCGA-24-	OV	53	FEMALE	WHITE	Stage IV	Serous Cy	G3	2003	-19595	Dead	WITH TUM	1264	1264	Recurrence	428	Complete Remission/Response	1	1264	1	1264			1	428
TCGA-24-	TCGA-24-	OV	53	FEMALE	WHITE	Stage IIIC	Serous Cy	G3	2003	-19579	Dead	WITH TUM	233	233	Recurrence	145	Progressive Disease	1	233	1	233			1	145
TCGA-24-	TCGA-24-	OV	77	FEMALE	WHITE	Stage IIIC	Serous Cy	G3	2003	-28201	Dead	WITH TUM	679	679	Recurrence	153	Complete Remission/Response	1	679	1	679	1	153	1	153
TCGA-24-	TCGA-24-	OV	50	FEMALE	BLACK OR AFRICAN	Stage IIIC	Serous Cy	G3	2001	-18583	Dead	WITH TUM	1646	1646	Recurrence	678	Complete Remission/Response	1	1646	1	1646	1	678	1	678
TCGA-24-	TCGA-24-	OV	56	FEMALE	WHITE	Stage IV	Serous Cy	G3	2001	-20527	Dead	WITH TUM	1933	1933	Recurrence	959	Complete Remission/Response	1	1933	1	1933			1	959
TCGA-24-	TCGA-24-	OV	36	FEMALE	WHITE	Stage IIIC	Serous Cy	G3	2001	-13187	Dead	WITH TUM	1442	1442	Recurrence	470	Complete Remission/Response	1	1442	1	1442	1	470	1	470
TCGA-24-	TCGA-24-	OV	51	FEMALE	WHITE	Stage IIIC	Serous Cy	G3	2008	-18846	Alive	TUMOR F	192				Complete Remission/Response	0	192	0	192	0	192	0	192
TCGA-24-	TCGA-24-	OV	34	FEMALE	WHITE	Stage IV	Serous Cy	G3	2008	-12772	Alive	TUMOR F	194				Complete Remission/Response	0	194	0	194			0	194
TCGA-24-	TCGA-24-	OV	54	FEMALE	WHITE	Stage IV	Serous Cy	G3	2008	-19775	Alive	TUMOR F	238				Complete Remission/Response	0	238	0	238			0	238
TCGA-24-	TCGA-24-	OV	68	FEMALE	WHITE	Stage IIIC	Serous Cy	G3	2008	-24842	Alive	TUMOR F	243				Complete Remission/Response	0	243	0	243	0	243	0	243
TCGA-24-	TCGA-24-	OV	62	FEMALE	WHITE	Stage IIIC	Serous Cy	G3	2008	-22659	Alive	TUMOR F	239				Complete Remission/Response	0	239	0	239	0	239	0	239
TCGA-24-	TCGA-24-	OV	82	FEMALE	BLACK OR AFRICAN	Stage IIIC	Serous Cy	G3	2008	-30234	Dead		23	23				1	23		23			0	23
TCGA-24-	TCGA-24-	OV	61	FEMALE	WHITE	Stage IIIC	Serous Cy	G3	2008	-22281	Alive	TUMOR F	190				Complete Remission/Response	0	190	0	190	0	190	0	190
TCGA-24-	TCGA-24-	OV	67	FEMALE	WHITE	Stage IIIC	Serous Cy	G3	2008	-24545	Alive	TUMOR F	183				Complete Remission/Response	0	183	0	183	0	183	0	183
TCGA-24-	TCGA-24-	OV	45	FEMALE	WHITE	Stage IIIC	Serous Cy	G3	2008	-16764	Alive	TUMOR F	181				Complete Remission/Response	0	181	0	181	0	181	0	181
TCGA-24-	TCGA-24-	OV	43	FEMALE	WHITE	Stage IIIC	Serous Cy	G3	2008	-15949	Alive	TUMOR F	163				Complete Remission/Response	0	163	0	163	0	163	0	163
TCGA-24-	TCGA-24-	OV	58	FEMALE		Stage IIIC	Serous Cy	G3	2008	-21423	Alive	WITH TUM	147				Partial Remission/Response	0	147	0	147			0	147
TCGA-24-	TCGA-24-	OV	50	FEMALE	WHITE	Stage IIIC	Serous Cy	G3	2008	-18306	Alive	WITH TUM	529		Recurrence	448	Complete Remission/Response	0	529	0	529	1	448	1	448
TCGA-24-	TCGA-24-	OV	68	FEMALE	WHITE	Stage IIIC	Serous Cy	G3	2000	-24939	Dead	WITH TUM	863	863	Recurrence	449	Complete Remission/Response	1	863	1	863	1	449	1	449
TCGA-24-	TCGA-24-	OV	67	FEMALE	WHITE	Stage IIIC	Serous Cy	G3	1999	-24758	Dead	WITH TUM	583	567	Recurrence	199		1	567	1	567			1	199
TCGA-24-	TCGA-24-	OV	59	FEMALE	WHITE	Stage IIIC	Serous Cy	G3	1999	-21810	Dead	WITH TUM	568	568	Recurrence	337	Partial Remission/Response	1	568	1	568			1	337
TCGA-24-	TCGA-24-	OV	57	FEMALE	WHITE	Stage IIIC	Serous Cy	G3	1999	-21107	Dead	WITH TUM	1324	1324	Recurrence	505	Complete Remission/Response	1	1324	1	1324	1	505	1	505
TCGA-24-	TCGA-24-	OV	57	FEMALE	WHITE	Stage IIIC	Serous Cy	G3	1999	-20881	Dead	WITH TUM	260	260			Partial Remission/Response	1	260	1	260			1	260
TCGA-24-	TCGA-24-	OV	70	FEMALE	WHITE	Stage IIIC	Serous Cy	G3	2000	-25734	Dead	WITH TUM	2218	2218	Recurrence	971	Complete Remission/Response	1	2218	1	2218	1	971	1	971
TCGA-24-	TCGA-24-	OV	70	FEMALE	WHITE	Stage IIIC	Serous Cy	G3	1998	-25874	Dead	WITH TUM	379	379	Recurrence	323		1	379	1	379			1	323
TCGA-24-	TCGA-24-	OV	74	FEMALE	WHITE	Stage IIIC	Serous Cy	G3	1998	-27307	Dead	WITH TUM	1373	1373	Recurrence	491	Complete Remission/Response	1	1373	1	1373	1	491	1	491
TCGA-24-	TCGA-24-	OV	51	FEMALE	WHITE	Stage IIIC	Serous Cy	G3	1998	-18652	Dead	WITH TUM	3224	3224	Recurrence	1253	Complete Remission/Response	1	3224	1	3224	1	1253	1	1253
TCGA-24-	TCGA-24-	OV	71	FEMALE	WHITE	Stage IIIC	Serous Cy	G3	2008	-25937	Alive	TUMOR F	277				Complete Remission/Response	0	277	0	277	0	277	0	277
TCGA-24-	TCGA-24-	OV	54	FEMALE	WHITE	Stage IIIB	Serous Cy	G3	2009	-20007	Alive	TUMOR F	105				Complete Remission/Response	0	105	0	105	0	105	0	105
TCGA-24-	TCGA-24-	OV	60	FEMALE	WHITE	Stage IIIC	Serous Cy	G3	2009	-22081	Alive		36					0	36	0	36			0	36
TCGA-24-	TCGA-24-	OV	57	FEMALE	BLACK OR AFRICAN	Stage IIIC	Serous Cy	G3	2000	-20902	Dead	WITH TUM	676	676	Recurrence	332	Partial Remission/Response	1	676	1	676			1	332
TCGA-24-	TCGA-24-	OV	71	FEMALE	BLACK OR AFRICAN	Stage IIIC	Serous Cy	G3	2000	-26141	Dead	WITH TUM	820	820	Progression of Disease	643	Partial Remission/Response	1	820	1	820			1	643
TCGA-24-	TCGA-24-	OV	69	FEMALE	WHITE	Stage IIIC	Serous Cy	G3	1997	-25460	Dead	WITH TUM	1746	1746	Recurrence	861	Complete Remission/Response	1	1746	1	1746	1	861	1	861
TCGA-24-	TCGA-24-	OV	46	FEMALE	WHITE	Stage IIIC	Serous Cy	G3	1997	-17130	Dead		1955	1955				1	1955		1955			0	1955
TCGA-24-	TCGA-24-	OV	57	FEMALE	WHITE	Stage IIIC	Serous Cy	G3	1997	-20945	Dead	WITH TUM	493	493	Recurrence	207	Complete Remission/Response	1	493	1	493	1	207	1	207
TCGA-24-	TCGA-24-	OV	58	FEMALE	WHITE	Stage IIIB	Serous Cy	G3	1997	-21432	Dead	WITH TUM	1721	1721	Recurrence	1042	Complete Remission/Response	1	1721	1	1721	1	1042	1	1042
TCGA-24-	TCGA-24-	OV	49	FEMALE	WHITE	Stage IIIC	Serous Cy	G3	1997	-18049	Dead	WITH TUM	1249	1249	Recurrence	892	Complete Remission/Response	1	1249	1	1249	1	892	1	892
TCGA-24-	TCGA-24-	OV	53	FEMALE	WHITE	Stage IIIC	Serous Cy	G3	1997	-19661	Dead	WITH TUM	1579	1579	Recurrence	764	Complete Remission/Response	1	1579	1	1579	1	764	1	764
TCGA-24-	TCGA-24-	OV	77	FEMALE	WHITE	Stage IIIC	Serous Cy	G3	1997	-28129	Dead	WITH TUM	1259	1259	Recurrence	403	Complete Remission/Response	1	1259	1	1259	1	403	1	403
TCGA-24-	TCGA-24-	OV	53	FEMALE	WHITE	Stage IIIB	Serous Cy	G3	1997	-19393	Dead	WITH TUM	1767	1767	Recurrence	553	Complete Remission/Response	1	1767	1	1767	1	553	1	553
TCGA-24-	TCGA-24-	OV	50	FEMALE	WHITE	Stage IIIC	Serous Cy	G3	1997	-18404	Dead	WITH TUM	2692	2692	Recurrence	1577	Complete Remission/Response	1	2692	1	2692	1	1577	1	1577
TCGA-24-	TCGA-24-	OV	50	FEMALE	WHITE	Stage IIB	Serous Cy	G3	2000	-18517	Dead	WITH TUM	2148	2148	Recurrence	539	Complete Remission/Response	1	2148	1	2148	1	539	1	539
TCGA-24-	TCGA-24-	OV	49	FEMALE	WHITE	Stage IIIC	Serous Cy	G3	2000	-17943	Dead	WITH TUM	1213	1213	Progression of Disease	447	Partial Remission/Response	1	1213	1	1213			1	447
TCGA-24-	TCGA-24-	OV	73	FEMALE	WHITE	Stage IIIC	Serous Cy	G3	2000	-27004	Dead	WITH TUM	594	594	Recurrence	273	Partial Remission/Response	1	594	1	594			1	273
TCGA-24-	TCGA-24-	OV	51	FEMALE	WHITE	Stage IIIC	Serous Cy	G4	1999	-18671	Dead	WITH TUM	1341	1341	Recurrence	143	Complete Remission/Response	1	1341	1	1341	1	143	1	143
TCGA-24-	TCGA-24-	OV	67	FEMALE	WHITE	Stage IIIC	Serous Cy	G3	1999	-24747	Dead	WITH TUM	1384	1384	Recurrence	229	Complete Remission/Response	1	1384	1	1384	1	229	1	229
TCGA-24-	TCGA-24-	OV	66	FEMALE	BLACK OR AFRICAN	Stage IIIC	Serous Cy	G3	1999	-24274	Dead	WITH TUM	1451	1451	Recurrence	396	Complete Remission/Response	1	1451	1	1451	1	396	1	396
TCGA-24-	TCGA-24-	OV	67	FEMALE	WHITE	Stage IIIC	Serous Cy	G3	1997	-24668	Dead	WITH TUM	787	787	Recurrence	221	Complete Remission/Response	1	787	1	787	1	221	1	221
TCGA-24-	TCGA-24-	OV	74	FEMALE	WHITE	Stage IIIC	Serous Cy	G3	1997	-27048	Dead	WITH TUM	312	312	Recurrence	145	Partial Remission/Response	1	312	1	312			1	145
TCGA-24-	TCGA-24-	OV	54	FEMALE	WHITE	Stage IIIB	Serous Cy	G3	1999	-19843	Dead	WITH TUM	524	524	Recurrence	319		1	524	1	524			1	319
TCGA-24-	TCGA-24-	OV	53	FEMALE	WHITE	Stage IIIB	Serous Cy	G3	1996	-19681	Dead	WITH TUM	2742	2742	Recurrence	992	Complete Remission/Response	1	2742	1	2742	1	992	1	992
TCGA-24-	TCGA-24-	OV	66	FEMALE	WHITE	Stage IIIC	Serous Cy	G3	1996	-24471	Dead	WITH TUM	2688	2688			Complete Remission/Response	1	2688	1	2688			1	2688
TCGA-24-	TCGA-24-	OV	57	FEMALE	WHITE	Stage IIIC	Serous Cy	G3	2001	-20993	Dead	WITH TUM	1470	1470	Recurrence	644	Complete Remission/Response	1	1470	1	1470	1	644	1	644
TCGA-24-	TCGA-24-	OV	56	FEMALE	WHITE	Stage IIIC	Serous Cy	G3	2001	-20785	Dead	WITH TUM	1163	1163	Recurrence	405	Complete Remission/Response	1	1163	1	1163	1	405	1	405
TCGA-24-	TCGA-24-	OV	49	FEMALE	WHITE	Stage IIIC	Serous Cy	G3	2009	-18227	Alive	TUMOR F	253				Complete Remission/Response	0	253	0	253	0	253	0	253
TCGA-24-	TCGA-24-	OV	66	FEMALE	WHITE	Stage IIIC	Serous Cy	G3	2009	-24469	Alive		106				Complete Remission/Response	0	106	0	106	0	106	0	106
TCGA-24-	TCGA-24-	OV	64	FEMALE	WHITE	Stage IIIC	Serous Cy	G3	2009	-23542	Alive		113					0	113	0	113			0	113
TCGA-24-	TCGA-24-	OV	42	FEMALE	WHITE	Stage IIIC	Serous Cy	G3	2009	-15599	Alive		116					0	116	0	116			0	116
TCGA-24-	TCGA-24-	OV	45	FEMALE		Stage IIIC	Serous Cy	G3	2009	-16534	Alive		133					0	133	0	133			0	133
TCGA-24-	TCGA-24-	OV	45	FEMALE	WHITE	Stage IV	Serous Cy	G3	2009	-16529	Alive	TUMOR F	343				Complete Remission/Response	0	343	0	343			0	343
TCGA-24-	TCGA-24-	OV	80	FEMALE	WHITE	Stage IIIC	Serous Cy	G3	2009	-29555	Alive	TUMOR F	176				Complete Remission/Response	0	176	0	176	0	176	0	176
TCGA-24-	TCGA-24-	OV	72	FEMALE	WHITE	Stage IIIC	Serous Cy	G3	2009	-26369	Alive	TUMOR F	168				Complete Remission/Response	0	168	0	168	0	168	0	168
TCGA-24-	TCGA-24-	OV	55	FEMALE	WHITE	Stage IIIC	Serous Cy	G3	1997	-20390	Dead	WITH TUM	2201	2201	Recurrence	1009	Complete Remission/Response	1	2201	1	2201	1	1009	1	1009
TCGA-24-	TCGA-24-	OV	55	FEMALE	WHITE	Stage IIIC	Serous Cy	G3	1997	-20390	Dead	WITH TUM	2201	2201	Recurrence	1009	Complete Remission/Response	1	2201	1	2201	1	1009	1	1009
TCGA-24-	TCGA-24-	OV	74	FEMALE	WHITE	Stage IIIC	Serous Cy	G3	2009	-27042	Alive	TUMOR F	298				Complete Remission/Response	0	298	0	298	0	298	0	298
TCGA-24-	TCGA-24-	OV	51	FEMALE	WHITE	Stage IIIC	Serous Cy	G3	2001	-18628	Dead	WITH TUM	690	690	Recurrence	343	Complete Remission/Response	1	690	1	690	1	343	1	343
TCGA-24-	TCGA-24-	OV	65	FEMALE	WHITE	Stage IIIC	Serous Cy	G3	2001	-23801	Dead	WITH TUM	919	919	Recurrence	320	Complete Remission/Response	1	919	1	919	1	320	1	320
TCGA-24-	TCGA-24-	OV	59	FEMALE	WHITE	Stage IIIC	Serous Cy	G3	1999	-21719	Dead	WITH TUM	500	500	Recurrence	290	Complete Remission/Response	1	500	1	500	1	290	1	290
TCGA-24-	TCGA-24-	OV	77	FEMALE	WHITE	Stage IIIC	Serous Cy	G3	1998	-28126	Dead	WITH TUM	336	336	Recurrence	70	Progressive Disease	1	336	1	336			1	70
TCGA-24-	TCGA-24-	OV	53	FEMALE	WHITE	Stage IIIC	Serous Cy	G3	1998	-19387	Dead	WITH TUM	2467	2467	Recurrence	544	Complete Remission/Response	1	2467	1	2467	1	544	1	544
TCGA-24-	TCGA-24-	OV	46	FEMALE	WHITE	Stage IIIC	Serous Cy	G3	2009	-17009	Alive		148					0	148	0	148			0	148
TCGA-24-	TCGA-24-	OV	67	FEMALE	WHITE	Stage IIIC	Serous Cy	G3	1994	-24682	Dead	WITH TUM	4624	4624	Recurrence	723	Complete Remission/Response	1	4624	1	4624	1	723	1	723
TCGA-24-	TCGA-24-	OV	54	FEMALE	WHITE	Stage IIIA	Serous Cy	G3	1994	-19901	Dead	WITH TUM	1364	1364	Recurrence	1069	Complete Remission/Response	1	1364	1	1364	1	1069	1	1069
TCGA-24-	TCGA-24-	OV	72	FEMALE	BLACK OR AFRICAN	Stage IIIC	Serous Cy	G3	1994	-26476	Dead	WITH TUM	1769	1769	Recurrence	421	Complete Remission/Response	1	1769	1	1769	1	421	1	421
TCGA-24-	TCGA-24-	OV	79	FEMALE	WHITE	Stage IIIC	Serous Cy	G3	1995	-29050	Dead		1059	1059				1	1059		1059			0	1059
TCGA-24-	TCGA-24-	OV	51	FEMALE	WHITE	Stage IV	Serous Cy	G3	1995	-18835	Dead	WITH TUM	3337	3337	Recurrence	617	Complete Remission/Response	1	3337	1	3337			1	617
TCGA-24-	TCGA-24-	OV	75	FEMALE	WHITE	Stage IIIC	Serous Cy	G3	1995	-27527	Dead	WITH TUM	439	439	Recurrence	372	Complete Remission/Response	1	439	1	439	1	372	1	372
TCGA-24-	TCGA-24-	OV	87	FEMALE	WHITE	Stage IIIC	Serous Cy	G3	1995	-31900	Dead	WITH TUM	1641	1701	Recurrence	646	Complete Remission/Response	1	1701	1	1701	1	646	1	646
TCGA-24-	TCGA-24-	OV	87	FEMALE	WHITE	Stage IIIC	Serous Cy	G3	1995	-31977	Dead	WITH TUM	562	562	Recurrence	351	Partial Remission/Response	1	562	1	562			1	351
TCGA-24-	TCGA-24-	OV	65	FEMALE	WHITE	Stage IIIC	Serous Cy	G3	1996	-23855	Dead	WITH TUM	857	857	Recurrence	287	Partial Remission/Response	1	857	1	857			1	287
TCGA-24-	TCGA-24-	OV	50	FEMALE	WHITE	Stage IIIA	Serous Cy	G3	1996	-18582	Dead	WITH TUM	1947	1947	Recurrence	914	Complete Remission/Response	1	1947	1	1947	1	914	1	914
TCGA-24-	TCGA-24-	OV	68	FEMALE	WHITE	Stage IIIA	Serous Cy	GB	2000	-24957	Dead	WITH TUM	1354	1354	Recurrence	1169	Complete Remission/Response	1	1354	1	1354	1	1169	1	1169
TCGA-24-	TCGA-24-	OV	66	FEMALE	WHITE	Stage IIIC	Serous Cy	G3	2003	-24407	Dead	WITH TUM	1736	1736	Recurrence	606	Complete Remission/Response	1	1736	1	1736	1	606	1	606
TCGA-24-	TCGA-24-	OV	74	FEMALE	WHITE	Stage IIIB	Serous Cy	G3	1992	-27112	Dead	WITH TUM	1100	1100	Recurrence	667	Complete Remission/Response	1	1100	1	1100	1	667	1	667
TCGA-24-	TCGA-24-	OV	76	FEMALE	WHITE	Stage IIIC	Serous Cy	G3	1992	-27897	Dead	WITH TUM	24	24				1	24	1	24			1	24
TCGA-24-	TCGA-24-	OV	57	FEMALE	WHITE	Stage IIIC	Serous Cy	G3	1992	-20861	Dead	WITH TUM	11	11				1	11	1	11			1	11
TCGA-24-	TCGA-24-	OV	58	FEMALE	WHITE	Stage IIB	Serous Cy	G3	1993	-21293	Dead	WITH TUM	1446	1446	Recurrence	512	Complete Remission/Response	1	1446	1	1446	1	512	1	512
TCGA-24-	TCGA-24-	OV	55	FEMALE	ASIAN	Stage IIIC	Serous Cy	G3	1999	-20399	Dead	WITH TUM	962	962	Recurrence	827		1	962	1	962			1	827
TCGA-24-	TCGA-24-	OV	74	FEMALE	WHITE	Stage IIIC	Serous Cy	G3	2004	-27356	Alive	WITH TUM	2143		Recurrence	535	Complete Remission/Response	0	2143	0	2143	1	535	1	535
TCGA-24-	TCGA-24-	OV	68	FEMALE	WHITE	Stage IIA	Serous Cy	G3	2006	-25019	Alive	TUMOR F	1357				Complete Remission/Response	0	1357	0	1357	0	1357	0	1357
TCGA-24-	TCGA-24-	OV	70	FEMALE	WHITE	Stage IIIC	Serous Cy	G3	1999	-25831	Dead	WITH TUM	25	25				1	25	1	25			1	25
TCGA-24-	TCGA-24-	OV	68	FEMALE	WHITE	Stage IV	Serous Cy	G3	1997	-25191	Dead	WITH TUM	2049	2049	Recurrence	568	Complete Remission/Response	1	2049	1	2049			1	568
TCGA-24-	TCGA-24-	OV	56	FEMALE	WHITE	Stage IIIC	Serous Cy	G3	1997	-20530	Dead	WITH TUM	1102	1102	Recurrence	324	Complete Remission/Response	1	1102	1	1102	1	324	1	324
TCGA-24-	TCGA-24-	OV	47	FEMALE	WHITE		Serous Cy	G3	1995	-17235	Dead		506	506			Partial Remission/Response	1	506		506			0	506
TCGA-24-	TCGA-24-	OV	69	FEMALE	WHITE	Stage IIIC	Serous Cy	G3	1995	-25416	Dead	WITH TUM	1007	1007	Recurrence	281	Complete Remission/Response	1	1007	1	1007	1	281	1	281
TCGA-24-	TCGA-24-	OV	56	FEMALE	WHITE	Stage IIIC	Serous Cy	G3	1993	-20487	Dead		1699	1699			Complete Remission/Response	1	1699		1699	0	1699	0	1699
TCGA-24-	TCGA-24-	OV	55	FEMALE	WHITE	Stage IIIC	Serous Cy	G3	1993	-20453	Dead	WITH TUM	1620	1620	Recurrence	469	Complete Remission/Response	1	1620	1	1620	1	469	1	469
TCGA-25-	TCGA-25-	OV	69	FEMALE	WHITE	Stage IV	Serous Cy	G3	2001	-25537	Dead		31	31				1	31		31			0	31
TCGA-25-	TCGA-25-	OV	62	FEMALE	WHITE	Stage IV	Serous Cy	G3	2000	-22982	Dead	WITH TUM	820	820	Recurrence	394	Complete Remission/Response	1	820	1	820			1	394
TCGA-25-	TCGA-25-	OV	42	FEMALE	WHITE	Stage IV	Serous Cy	G3	2000	-15462	Dead	WITH TUM	974	1004	Progression of Disease	274	Partial Remission/Response	1	1004	1	1004			1	274
TCGA-25-	TCGA-25-	OV	50	FEMALE	WHITE	Stage IIIC	Serous Cy	G3	2000	-18263	Dead	WITH TUM	1553	1583	Progression of Disease	153	Partial Remission/Response	1	1583	1	1583			1	153
TCGA-25-	TCGA-25-	OV	55	FEMALE	WHITE	Stage IIIC	Serous Cy	G3	2000	-20331	Dead	WITH TUM	1126	1279	Progression of Disease	276	Progressive Disease	1	1279	1	1279			1	276
TCGA-25-	TCGA-25-	OV	66	FEMALE	WHITE	Stage IIIC	Serous Cy	G3	2000	-24199	Dead			61				1	61		61			0	61
TCGA-25-	TCGA-25-	OV	54	FEMALE	WHITE	Stage IIIC	Serous Cy	G3	2000	-19755	Dead	WITH TUM	1064	1064	Recurrence	185	Partial Remission/Response	1	1064	1	1064			1	185
TCGA-25-	TCGA-25-	OV	73	FEMALE	WHITE	Stage IIIC	Serous Cy	G3	2000	-26786	Dead		1977	1977	Recurrence	730		1	1977		1977			1	730
TCGA-25-	TCGA-25-	OV	65	FEMALE	WHITE	Stage IIIC	Serous Cy	G3	2001	-24018	Dead	WITH TUM	1124	1155	Recurrence	424	Progressive Disease	1	1155	1	1155			1	424
TCGA-25-	TCGA-25-	OV	65	FEMALE	WHITE	Stage IIIC	Serous Cy	G3	2001	-23987	Dead	WITH TUM	1033	1033	Recurrence	454	Complete Remission/Response	1	1033	1	1033	1	454	1	454
TCGA-25-	TCGA-25-	OV	62	FEMALE	WHITE	Stage IV	Serous Cy	G3	2001	-22646	Dead	WITH TUM	91	91				1	91	1	91			1	91
TCGA-25-	TCGA-25-	OV	72	FEMALE	WHITE	Stage IIIC	Serous Cy	G3	2001	-26602	Dead	WITH TUM	365	395	Progression of Disease	365	Complete Remission/Response	1	395	1	395	1	365	1	365
TCGA-25-	TCGA-25-	OV	74	FEMALE	WHITE	Stage IIIC	Serous Cy	G3	2001	-27090	Dead		1035	1035				1	1035		1035			0	1035
TCGA-25-	TCGA-25-	OV	77	FEMALE	WHITE	Stage IV	Serous Cy	G3	2002	-28183	Dead		976	976				1	976		976			0	976
TCGA-25-	TCGA-25-	OV	61	FEMALE	WHITE	Stage IIIC	Serous Cy	G3	2002	-22339	Dead	WITH TUM	945	1249	Recurrence	396	Partial Remission/Response	1	1249	1	1249			1	396
TCGA-25-	TCGA-25-	OV	38	FEMALE	WHITE	Stage IIIC	Serous Cy	G3	2001	-13911	Dead	WITH TUM	1979	2009	Recurrence	244	Partial Remission/Response	1	2009	1	2009			1	244
TCGA-25-	TCGA-25-	OV	76	FEMALE	WHITE	Stage IIIC	Serous Cy	G3	2002	-28093	Dead		90	457				1	457		457			0	457
TCGA-25-	TCGA-25-	OV	71	FEMALE	WHITE	Stage IV	Serous Cy	G3	2006	-26060	Dead	WITH TUM	565	565	Recurrence	342	Complete Remission/Response	1	565	1	565			1	342
TCGA-25-	TCGA-25-	OV	66	FEMALE	WHITE	Stage IIIC	Serous Cy	G3	2006	-24298	Dead	WITH TUM	548	840	Recurrence	526	Stable Disease	1	840	1	840			1	526
TCGA-25-	TCGA-25-	OV	65	FEMALE	WHITE	Stage IIIC	Serous Cy	G3	2006	-23932	Dead	WITH TUM	481	518	Recurrence	318	Partial Remission/Response	1	518	1	518			1	318
TCGA-25-	TCGA-25-	OV	73	FEMALE	WHITE	Stage IIIC	Serous Cy	G3	2003	-26784	Dead		11	394				1	394		394			0	394
TCGA-25-	TCGA-25-	OV	67	FEMALE	WHITE	Stage IIIC	Serous Cy	G3	2002	-24488	Dead	WITH TUM	627	627	Recurrence	287	Stable Disease	1	627	1	627			1	287
TCGA-25-	TCGA-25-	OV	73	FEMALE	WHITE	Stage IIIC	Serous Cy	G3	2002	-27006	Dead		239	1162			Complete Remission/Response	1	1162		1162	0	1162	0	1162
TCGA-25-	TCGA-25-	OV	73	FEMALE	WHITE	Stage IIIC	Serous Cy	G3	2002	-26687	Dead	TUMOR F	9	9				1	9	0	9			0	9
TCGA-25-	TCGA-25-	OV	68	FEMALE	WHITE	Stage IV	Serous Cy	G3	2002	-24993	Dead	WITH TUM	1732	1799	Recurrence	537	Complete Remission/Response	1	1799	1	1799			1	537

TCGA-25-	TCGA-25-	OV	64	FEMALE	WHITE		Stage IIIC	Serous Cy	G3	2002	-23407	Dead	WITH TUM	1843	1891		Recurrence			616	Complete Remission/Response	1	1891	1	1891	1	616	1	616
TCGA-25-	TCGA-25-	OV	75	FEMALE	WHITE		Stage IIIC	Serous Cy	G3	2002	-27749	Dead	WITH TUM	1081	1091		Recurrence			380	Complete Remission/Response	1	1091	1	1091	1	380	1	380
TCGA-25-	TCGA-25-	OV	71	FEMALE	WHITE		Stage IIIC	Serous Cy	G3	2002	-26200	Dead	WITH TUM	1558	1583		Recurrence			614	Complete Remission/Response	1	1583	1	1583	1	614	1	614
TCGA-25-	TCGA-25-	OV	59	FEMALE	WHITE		Stage IIIC	Serous Cy	G3	1994	-21550	Dead	WITH TUM	455	455		Recurrence			243	Partial Remission/Response	1	455	1	455			1	243
TCGA-25-	TCGA-25-	OV	70	FEMALE	WHITE		Stage IIIC	Serous Cy	G3	1993	-25627	Dead	WITH TUM	760	760		Recurrence			230	Progressive Disease	1	760	1	760			1	230
TCGA-25-	TCGA-25-	OV	81	FEMALE	WHITE		Stage IIIC	Serous Cy	G3	1993	-29615	Dead	WITH TUM	549	730		Recurrence			549	Complete Remission/Response	1	730	1	730	1	549	1	549
TCGA-25-	TCGA-25-	OV	60	FEMALE	WHITE		Stage IIIC	Serous Cy	G3	1994	-22035	Dead	WITH TUM	2587	2587		Recurrence			971	Complete Remission/Response	1	2587	1	2587	1	971	1	971
TCGA-25-	TCGA-25-	OV	60	FEMALE	AMERICAN INDIAN (		Stage IIIC	Serous Cy	G3	2006	-22127	Dead	WITH TUM	396	396						Progressive Disease	1	396	1	396			1	396
TCGA-25-	TCGA-25-	OV	80	FEMALE				Serous Cystadenocar		2003	-29257																	0	
TCGA-25-	TCGA-25-	OV	57	FEMALE	WHITE		Stage IIIC	Serous Cy	G3	2003	-20939	Dead	TUMOR F	1492	1492						Complete Remission/Response	1	1492	0	1492	0	1492	0	1492
TCGA-25-	TCGA-25-	OV	75	FEMALE	WHITE		Stage IV	Serous Cy	G3	2003	-27667	Dead		31	31							1	31		31			0	31
TCGA-25-	TCGA-25-	OV	81	FEMALE	WHITE		Stage IIIC	Serous Cy	G3	2003	-29585	Dead	WITH TUM	1157	1157		Recurrence			305	Partial Remission/Response	1	1157	1	1157			1	305
TCGA-25-	TCGA-25-	OV	71	FEMALE	WHITE		Stage IIIC	Serous Cy	G3	2004	-26145	Dead		92	92							1	92		92			0	92
TCGA-25-	TCGA-25-	OV	59	FEMALE	WHITE		Stage IV	Serous Cy	G3	2004	-21581	Dead		365	365							1	365		365			0	365
TCGA-25-	TCGA-25-	OV	71	FEMALE	WHITE		Stage IIIC	Serous Cy	G3	2004	-26267	Dead	WITH TUM	1369	1369		Recurrence			577	Complete Remission/Response	1	1369	1	1369	1	577	1	577
TCGA-25-	TCGA-25-	OV	80	FEMALE	WHITE		Stage IIIC	Serous Cy	G3	2004	-29373	Dead	WITH TUM	608	608		Recurrence			243	Partial Remission/Response	1	608	1	608			1	243
TCGA-25-	TCGA-25-	OV	76	FEMALE	WHITE		Stage IIIC	Serous Cy	G3	2004	-27789	Dead	WITH TUM	1278	1278		Recurrence			577	Complete Remission/Response	1	1278	1	1278	1	577	1	577
TCGA-25-	TCGA-25-	OV	64	FEMALE	WHITE		Stage IIIC	Serous Cy	G3	2004	-23711	Dead		90	90							1	90		90			0	90
TCGA-25-	TCGA-25-	OV	38	FEMALE	AMERICAN INDIAN (		Stage IIIC	Serous Cy	G3	2005	-14184	Dead	WITH TUM	883	883		Recurrence			426	Complete Remission/Response	1	883	1	883	1	426	1	426
TCGA-25-	TCGA-25-	OV	37	FEMALE	WHITE		Stage IV	Serous Cy	G2	2005	-13606	Dead	WITH TUM	943	943		Recurrence			334	Partial Remission/Response	1	943	1	943			1	334
TCGA-25-	TCGA-25-	OV	71	FEMALE	WHITE		Stage IV	Serous Cy	G3	2005	-26267	Dead	WITH TUM	821	821		Recurrence			304	Complete Remission/Response	1	821	1	821			1	304
TCGA-29-	TCGA-29-	OV	39	FEMALE	WHITE		Stage IIIC	Serous Cy	G2	1992	-14574	Dead	WITH TUM	2400	2400		Recurrence			184	Stable Disease	1	2400	1	2400			1	184
TCGA-29-	TCGA-29-	OV	66	FEMALE	WHITE		Stage IIIC	Serous Cy	G2	1993	-24319	Dead	WITH TUM	1360	1448							1	1448	1	1448			1	1448
TCGA-29-	TCGA-29-	OV	51	FEMALE	WHITE		Stage IIIC	Serous Cy	G2	1995	-18664	Dead	WITH TUM	1435	1470		Recurrence			613	Complete Remission/Response	1	1470	1	1470	1	613	1	613
TCGA-29-	TCGA-29-	OV	58	FEMALE	WHITE		Stage IIIC	Serous Cy	G2	1997	-21193	Dead	WITH TUM	2872	2872		Recurrence			544	Complete Remission/Response	1	2872	1	2872	1	544	1	544
TCGA-29-	TCGA-29-	OV	58	FEMALE	WHITE		Stage IIIC	Serous Cy	G2	1997	-21193	Dead	WITH TUM	2872	2872		Recurrence			544	Complete Remission/Response	1	2872	1	2872	1	544	1	544
TCGA-29-	TCGA-29-	OV	72	FEMALE	WHITE		Stage IIIC	Serous Cy	G3	2001	-26300	Alive	WITH TUM	3096			Recurrence			694	Complete Remission/Response	0	3096	0	3096	1	694	1	694
TCGA-29-	TCGA-29-	OV	45	FEMALE	WHITE		Stage IIIC	Serous Cy	GX	2002	-16523	Dead	WITH TUMOR		1187						Stable Disease	1	1187	1	1187			1	1187
TCGA-29-	TCGA-29-	OV	62	FEMALE	WHITE		Stage IIIC	Serous Cy	G2	2003	-22895	Dead		24	1229							1	1229		1229			0	1229
TCGA-29-	TCGA-29-	OV	43	FEMALE	WHITE		Stage IIIC	Serous Cy	G2	2003	-15818	Dead	WITH TUM	1025	1032		Recurrence			342	Partial Remission/Response	1	1032	1	1032			1	342
TCGA-29-	TCGA-29-	OV	62	FEMALE	WHITE		Stage IIIC	Serous Cy	G3	2003	-22933	Dead	WITH TUM	949	949		Recurrence			500	Complete Remission/Response	1	949	1	949	1	500	1	500
TCGA-29-	TCGA-29-	OV	53	FEMALE	BLACK OR AFRICAN		Stage IIIC	Serous Cy	G3	2004	-19611	Alive	WITH TUM	2078			Recurrence			181	Complete Remission/Response	0	2078	0	2078	1	181	1	181
TCGA-29-	TCGA-29-	OV	57	FEMALE	WHITE		Stage IIIC	Serous Cy	G3	2004	-21130	Dead		16	1106							1	1106		1106			0	1106
TCGA-29-	TCGA-29-	OV	56	FEMALE	WHITE		Stage IIIC	Serous Cy	G3	2004	-20737	Dead	WITH TUM	514	515		Recurrence			298	Partial Remission/Response	1	515	1	515			1	298
TCGA-29-	TCGA-29-	OV	84	FEMALE	WHITE		Stage IIIC	Serous Cy	G3	2005	-30910	Dead	WITH TUM	568	728		Recurrence			286	Complete Remission/Response	1	728	1	728	1	286	1	286
TCGA-29-	TCGA-29-	OV	56	FEMALE	BLACK OR AFRICAN		Stage IIIC	Serous Cy	G2	2005	-20622	Dead	WITH TUM	1815	1815		Recurrence			324	Complete Remission/Response	1	1815	1	1815	1	324	1	324
TCGA-29-	TCGA-29-	OV	61	FEMALE	WHITE		Stage IIIC	Serous Cy	G3	2005	-22511	Alive	WITH TUM	1587			Recurrence			568	Complete Remission/Response	0	1587	0	1587	1	568	1	568
TCGA-29-	TCGA-29-	OV	61	FEMALE	WHITE		Stage IIIC	Serous Cy	G3	2005	-22511	Alive	WITH TUM	1587			Recurrence			568	Complete Remission/Response	0	1587	0	1587	1	568	1	568
TCGA-29-	TCGA-29-	OV	47	FEMALE	WHITE		Stage IIIC	Serous Cy	G2	2005	-17530	Dead	WITH TUM	555	555		Recurrence			283	Complete Remission/Response	1	555	1	555	1	283	1	283
TCGA-29-	TCGA-29-	OV	47	FEMALE	WHITE		Stage IIIC	Serous Cy	G2	2005	-17530	Dead	WITH TUM	555	555		Recurrence			283	Complete Remission/Response	1	555	1	555	1	283	1	283
TCGA-29-	TCGA-29-	OV	41	FEMALE	WHITE		Stage IIC	Serous Cy	G3	2006	-15017	Alive		1277			Recurrence			1201	Complete Remission/Response	0	1277	0	1277	1	1201	1	1201
TCGA-29-	TCGA-29-	OV	41	FEMALE	WHITE		Stage IIC	Serous Cy	G3	2006	-15017	Alive		1277			Recurrence			1201	Complete Remission/Response	0	1277	0	1277	1	1201	1	1201
TCGA-29-	TCGA-29-	OV	54	FEMALE	WHITE		Stage IIIC	Serous Cy	G2	2006	-19969	Dead	WITH TUM	889	951		Recurrence			315	Partial Remission/Response	1	951	1	951			1	315
TCGA-29-	TCGA-29-	OV	54	FEMALE	WHITE		Stage IIIC	Serous Cy	G2	2006	-19969	Dead	WITH TUM	889	951		Recurrence			315	Partial Remission/Response	1	951	1	951			1	315
TCGA-29-	TCGA-29-	OV	45	FEMALE	BLACK OR AFRICAN		Stage IIIC	Serous Cy	G2	2006	-16485	Alive	TUMOR F	1053							Complete Remission/Response	0	1053	0	1053	0	1053	0	1053
TCGA-29-	TCGA-29-	OV	80	FEMALE	ASIAN		Stage IIIC	Serous Cy	G3	1998	-29337	Dead	WITH TUM	517	528						Stable Disease	1	528	1	528			1	528
TCGA-29-	TCGA-29-	OV	59	FEMALE	WHITE		Stage IV	Serous Cy	G2	1998	-21879	Dead	WITH TUM	2606	2634		Recurrence			1375	Complete Remission/Response	1	2634	1	2634			1	1375
TCGA-29-	TCGA-29-	OV	43	FEMALE	BLACK OR AFRICAN		Stage IIC	Serous Cy	G2	2004	-16021	Alive	WITH TUM	2032			Recurrence			428	Complete Remission/Response	0	2032	0	2032	1	428	1	428
TCGA-29-	TCGA-29-	OV	49	FEMALE	WHITE		Stage IIIC	Serous Cy	G2	2004	-18012	Alive	WITH TUM	1914			Recurrence			792	Complete Remission/Response	0	1914	0	1914	1	792	1	792
TCGA-29-	TCGA-29-	OV	74	FEMALE	WHITE		Stage IIIC	Serous Cy	G2	2006	-27359	Dead	WITH TUM	1190	1199		Recurrence			672	Progressive Disease	1	1199	1	1199			1	672
TCGA-29-	TCGA-29-	OV	50	FEMALE	WHITE		Stage IV	Serous Cy	G3	2006	-18396	Dead	WITH TUM	950	952		Recurrence			547	Stable Disease	1	952	1	952			1	547
TCGA-29-	TCGA-29-	OV	40	FEMALE	WHITE		Stage IIIC	Serous Cy	G3	2007	-14754	Alive	TUMOR F	699							Complete Remission/Response	0	699	0	699	0	699	0	699
TCGA-29-	TCGA-29-	OV	54	FEMALE	WHITE		Stage IIIC	Serous Cy	G2	2007	-20015	Alive	WITH TUM	741			Recurrence			377	Complete Remission/Response	0	741	0	741	1	377	1	377
TCGA-29-	TCGA-29-	OV	54	FEMALE	WHITE		Stage IIIC	Serous Cy	G2	2007	-20015	Alive	WITH TUM	741			Recurrence			377	Complete Remission/Response	0	741	0	741	1	377	1	377
TCGA-29-	TCGA-29-	OV	76	FEMALE	WHITE		Stage IIIC	Serous Cy	G2	2007	-28093	Alive	WITH TUM	686			Recurrence			595	Complete Remission/Response	0	686	0	686	1	595	1	595
TCGA-29-	TCGA-29-	OV	82	FEMALE	WHITE		Stage IIIC	Serous Cy	G3	2008	-29991	Alive	WITH TUM	527			Recurrence			234	Progressive Disease	0	527	0	527			1	234
TCGA-29-	TCGA-29-	OV	51	FEMALE	WHITE		Stage IIIC	Serous Cy	G2	2008	-18943	Alive	WITH TUM	376			Recurrence			341	Complete Remission/Response	0	376	0	376	1	341	1	341
TCGA-29-	TCGA-29-	OV	63	FEMALE	BLACK OR AFRICAN		Stage IIIC	Serous Cy	G3	2008	-23016	Alive	WITH TUM	360							Stable Disease	0	360	0	360			0	360
TCGA-29-	TCGA-29-	OV	47	FEMALE	WHITE		Stage IIIC	Serous Cy	G2	2008	-17356	Alive	WITH TUM	374							Progressive Disease	0	374	0	374			0	374
TCGA-29-	TCGA-29-	OV	77	FEMALE	WHITE		Stage IIIC	Serous Cy	G3	2008	-28147	Alive	TUMOR F	454							Complete Remission/Response	0	454	0	454	0	454	0	454
TCGA-29-	TCGA-29-	OV	69	FEMALE	WHITE		Stage IIIC	Serous Cy	G3	2009	-25404	Alive	TUMOR F	255							Complete Remission/Response	0	255	0	255	0	255	0	255
TCGA-29-	TCGA-29-	OV	58	FEMALE	WHITE		Stage IIIC	Serous Cy	G3	2009	-21284	Alive	TUMOR F	220								0	220	0	220			0	220
TCGA-29-	TCGA-29-	OV	55	FEMALE	WHITE		Stage IIIC	Serous Cy	G3	2009	-20210	Alive	TUMOR F	163							Complete Remission/Response	0	163	0	163	0	163	0	163
TCGA-29-	TCGA-29-	OV	55	FEMALE	WHITE		Stage IIIC	Serous Cy	G3	2006	-20417	Dead		1053	1104		Recurrence			473	Complete Remission/Response	1	1104		1104	1	473	1	473
TCGA-29-	TCGA-29-	OV	75	FEMALE	WHITE		Stage IIIC	Serous Cy	G2	1998	-27417	Dead	WITH TUM	2621	2621		Recurrence			552	Complete Remission/Response	1	2621	1	2621	1	552	1	552
TCGA-29-	TCGA-29-	OV	75	FEMALE	WHITE		Stage IIIC	Serous Cy	G2	1998	-27417	Dead	WITH TUM	2621	2621		Recurrence			552	Complete Remission/Response	1	2621	1	2621	1	552	1	552
TCGA-29-	TCGA-29-	OV	60	FEMALE	WHITE		Stage IIIC	Serous Cy	G2	2004	-22089	Alive	TUMOR F	1977			Progression of Disease			1577	Complete Remission/Response	0	1977	0	1977	1	1577	1	1577
TCGA-29-	TCGA-29-	OV	60	FEMALE	WHITE		Stage IIIC	Serous Cy	G3	2004	-21940	Alive	WITH TUM	1900			Recurrence			1582	Complete Remission/Response	0	1900	0	1900	1	1582	1	1582
TCGA-29-	TCGA-29-	OV	58	FEMALE	WHITE		Stage IIIC	Serous Cy	G3	2005	-21392	Alive	TUMOR F	1372							Complete Remission/Response	0	1372	0	1372	0	1372	0	1372
TCGA-29-	TCGA-29-	OV	79	FEMALE	WHITE		Stage IIIC	Serous Cy	G3	2005	-29156	Dead	WITH TUMOR		421						[Unknown]	1	421	1	421			1	421
TCGA-29-	TCGA-29-	OV	59	FEMALE	WHITE		Stage IIIC	Serous Cy	G2	2006	-21833	Alive	WITH TUM	1285			Recurrence			960	Complete Remission/Response	0	1285	0	1285	1	960	1	960
TCGA-29-	TCGA-29-	OV	61	FEMALE	WHITE		Stage IIIC	Serous Cy	G3	2008	-22605	Dead	WITH TUM	360	653		Recurrence			174	Progressive Disease	1	653	1	653			1	174
TCGA-29-	TCGA-29-	OV	52	FEMALE	WHITE		Stage IIIC	Serous Cy	G3	2009	-18997	Alive	TUMOR F	349							Complete Remission/Response	0	349	0	349	0	349	0	349
TCGA-29-	TCGA-29-	OV	36	FEMALE	WHITE		Stage IIIC	Serous Cy	G2	2009	-13346	Alive	TUMOR F	297							Complete Remission/Response	0	297	0	297	0	297	0	297
TCGA-29-	TCGA-29-	OV	66	FEMALE	BLACK OR AFRICAN		Stage IIIC	Serous Cy	G3	2011	-24161	Dead	WITH TUMOR		1088		Recurrence			358	Complete Remission/Response	1	1088	1	1088	1	358	1	358
TCGA-30-	TCGA-30-	OV	68	FEMALE	WHITE		Stage IV	Serous Cy	G3	2001	-25032	Dead	WITH TUM	1158	1158		Recurrence			241	Complete Remission/Response	1	1158	1	1158			1	241
TCGA-30-	TCGA-30-	OV	44	FEMALE	WHITE		Stage IIIC	Serous Cy	G3	2002	-16400	Dead	WITH TUM	1579	1579		Recurrence			896		1	1579	1	1579			1	896
TCGA-30-	TCGA-30-	OV	58	FEMALE	WHITE		Stage IIIC	Serous Cy	G3	2000	-21404	Dead	WITH TUM	1103	1103		Recurrence			634	Complete Remission/Response	1	1103	1	1103	1	634	1	634
TCGA-30-	TCGA-30-	OV	61	FEMALE	WHITE		Stage IIIC	Serous Cy	G3	2001	-22394	Dead	WITH TUM	75	75							1	75	1	75			1	75
TCGA-30-	TCGA-30-	OV	56	FEMALE	WHITE		Stage IIIC	Serous Cy	G3	2001	-20626	Dead	WITH TUM	477	477		Recurrence			220	Partial Remission/Response	1	477	1	477			1	220
TCGA-30-	TCGA-30-	OV	64	FEMALE	WHITE		Stage IV	Serous Cy	G3	2001	-23689	Dead	WITH TUM	8	8							1	8	1	8			1	8
TCGA-30-	TCGA-30-	OV	56	FEMALE	WHITE			Serous Cy	G3	2002	-20498	Dead	WITH TUM	1463	1463						Partial Remission/Response	1	1463	1	1463			1	1463
TCGA-30-	TCGA-30-	OV	58	FEMALE	WHITE		Stage IIIC	Serous Cy	G3	2002	-21406	Dead	WITH TUM	1366	1366		Recurrence			397	Complete Remission/Response	1	1366	1	1366	1	397	1	397
TCGA-30-	TCGA-30-	OV	74	FEMALE	WHITE		Stage IIIC	Serous Cy	G3	2002	-27103	Dead		1058	1058							1	1058		1058			0	1058
TCGA-30-	TCGA-30-	OV	65	FEMALE	WHITE		Stage IV	Serous Cy	G2	2003	-23895	Dead	WITH TUM	186	186							1	186	1	186			1	186
TCGA-30-	TCGA-30-	OV	61	FEMALE	WHITE		Stage IV	Serous Cy	G2	2003	-22562	Dead	WITH TUM	1114	1114		Recurrence			285	Complete Remission/Response	1	1114	1	1114			1	285
TCGA-30-	TCGA-30-	OV	46	FEMALE			Stage IV	Serous Cy	GX	2004	-17159	Dead	WITH TUM	37	37							1	37	1	37			1	37
TCGA-30-	TCGA-30-	OV	50	FEMALE	WHITE		Stage IV	Serous Cy	G3	2004	-18398	Dead	WITH TUM	1563	1563		Recurrence			767	Complete Remission/Response	1	1563	1	1563			1	767
TCGA-30-	TCGA-30-	OV	56	FEMALE	WHITE		Stage IIIC	Serous Cy	G3	2001	-20525	Dead	WITH TUM	1751	1751		Recurrence			348	Partial Remission/Response	1	1751	1	1751			1	348
TCGA-30-	TCGA-30-	OV	67	FEMALE	WHITE		Stage IIIC	Serous Cy	G2	2005	-24495	Dead	WITH TUM	738	738						Partial Remission/Response	1	738	1	738			1	738
TCGA-30-	TCGA-30-	OV	61	FEMALE	WHITE		Stage IIIC	Serous Cy	G2	2004	-22344	Dead	WITH TUM	914	914		Recurrence			181	Partial Remission/Response	1	914	1	914			1	181
TCGA-30-	TCGA-30-	OV	52	FEMALE	WHITE		Stage IIIC	Serous Cy	G3	2004	-19256	Dead	WITH TUM	1484	1484		Recurrence			253	Complete Remission/Response	1	1484	1	1484	1	253	1	253
TCGA-31-	TCGA-31-	OV	47	FEMALE	WHITE		Stage IIIC	Serous Cy	G3	2005	-17207	Alive	WITH TUM	1386			Recurrence			678	Complete Remission/Response	0	1386	0	1386	1	678	1	678
TCGA-31-	TCGA-31-	OV	30	FEMALE			Stage IIIC	Serous Cy	G3	2006	-11146	Alive	WITH TUM	918			Recurrence			664		0	918	0	918			1	664
TCGA-31-	TCGA-31-	OV	76	FEMALE	WHITE		Stage IIIC	Serous Cy	G2	2007	-28100	Alive	WITH TUM	571			Recurrence			349	Partial Remission/Response	0	571	0	571			1	349
TCGA-31-	TCGA-31-	OV	58	FEMALE	WHITE		Stage IIIC	Serous Cy	G3	2007	-21352	Alive	WITH TUM	684			Recurrence			659		0	684	0	684			1	659
TCGA-31-	TCGA-31-	OV	52	FEMALE	ASIAN		Stage IIIC	Serous Cy	G3	2008	-19064	Alive	WITH TUM	204			Recurrence			70	Progressive Disease	0	204	0	204			1	70
TCGA-31-	TCGA-31-	OV	43	FEMALE	WHITE		Stage IIIC	Serous Cy	G3	2005	-15926	Alive	WITH TUM	1293			Recurrence			918		0	1293	0	1293			1	918
TCGA-31-	TCGA-31-	OV	60	FEMALE	WHITE		Stage IIIB	Serous Cy	G3	2005	-22100	Alive	WITH TUM	1342								0	1342	0	1342			0	1342
TCGA-31-	TCGA-31-	OV	49	FEMALE	WHITE		Stage IV	Serous Cy	G2	2009	-18247	Alive	WITH TUM	67								0	67	0	67			0	67
TCGA-36-	TCGA-36-	OV	52	FEMALE			Stage IIIC	Serous Cy	G3	2007	-19311	Alive	WITH TUM	875			Recurrence			548	Partial Remission/Response	0	875	0	875			1	548
TCGA-36-	TCGA-36-	OV	52	FEMALE	WHITE		Stage IIIC	Serous Cy	G3	2007	-19151	Alive		885							Partial Remission/Response	0	885	0	885			0	885
TCGA-36-	TCGA-36-	OV	49	FEMALE	WHITE		Stage IIIC	Serous Cy	G3	2007	-17956	Alive	WITH TUM	655			Recurrence			375	Partial Remission/Response	0	655	0	655			1	375
TCGA-36-	TCGA-36-	OV	53	FEMALE	WHITE		Stage IIIB	Serous Cy	G3	2007	-19608	Dead	WITH TUM	695	695		Recurrence			375	Complete Remission/Response	1	695	1	695	1	375	1	375
TCGA-36-	TCGA-36-	OV	48	FEMALE	ASIAN		Stage IIIC	Serous Cy	G3	2007	-17847	Alive	WITH TUM	686			Recurrence			648	Complete Remission/Response	0	686	0	686	1	648	1	648
TCGA-36-	TCGA-36-	OV	83	FEMALE			Stage IIIB	Serous Cy	G3	2006	-30592	Alive	TUMOR F	260								0	260	0	260			0	260
TCGA-36-	TCGA-36-	OV	76	FEMALE			Stage IIIC	Serous Cy	G3	2006	-27778	Alive	WITH TUM	915			Recurrence			817	Complete Remission/Response	0	915	0	915	1	817	1	817
TCGA-36-	TCGA-36-	OV	43	FEMALE	ASIAN		Stage IIC	Serous Cy	G2	2007	-15804	Alive	TUMOR F	783							Complete Remission/Response	0	783	0	783	0	783	0	783
TCGA-36-	TCGA-36-	OV	63	FEMALE	ASIAN		Stage IV	Serous Cy	G3	2007	-23329	Alive	WITH TUM	847			Recurrence			310		0	847	0	847			1	310
TCGA-36-	TCGA-36-	OV	82	FEMALE			Stage IIIC	Serous Cy	G3	2007	-29992	Dead	WITH TUM	737	737		Recurrence			440		1	737	1	737			1	440
TCGA-36-	TCGA-36-	OV	63	FEMALE	WHITE		Stage IIC	Serous Cy	G3	2007	-23272	Alive	WITH TUM	751			Recurrence			702	Complete Remission/Response	0	751	0	751	1	702	1	702
TCGA-36-	TCGA-36-	OV	71	FEMALE			Stage IIIC	Serous Cy	G3	2007	-26092	Alive	WITH TUM	1066			Recurrence			567	Complete Remission/Response	0	1066	0	1066	1	567	1	567
TCGA-36-	TCGA-36-	OV	38	FEMALE	ASIAN		Stage IIIC	Serous Cy	G3	2007	-13885	Alive	WITH TUM	1004			Recurrence			630	Complete Remission/Response	0	1004	0	1004	1	630	1	630
TCGA-36-	TCGA-36-	OV	61	FEMALE			Stage IIIC	Serous Cy	G3	2007	-22333	Alive	WITH TUM	897								0	897	0	897			0	897
TCGA-36-	TCGA-36-	OV	55	FEMALE			Stage IIIC	Serous Cy	G3	2007	-20187			0														0	
TCGA-36-	TCGA-36-	OV	72	FEMALE	WHITE		Stage IIIC	Serous Cy	G3	2007	-26621	Alive	WITH TUM	907			Progression of Disease			383	Partial Remission/Response	0	907	0	907			1	383
TCGA-36-	TCGA-36-	OV	39	FEMALE			Stage IIIC	Serous Cy	G3	2008	-14595	Alive	WITH TUM	788			Progression of Disease			431	Partial Remission/Response	0	788	0	788			1	431
TCGA-36-	TCGA-36-	OV	37	FEMALE			Stage IIIB	Serous Cy	G3	2008	-13681	Alive	WITH TUM	763			Recurrence			523	Complete Remission/Response	0	763	0	763	1	523	1	523
TCGA-36-	TCGA-36-	OV	26	FEMALE	WHITE		Stage IIIC	Serous Cy	G1	2008	-9760	Alive	WITH TUM	724			Recurrence			665	Complete Remission/Response	0	724	0	724	1	665	1	665
TCGA-36-	TCGA-36-	OV	59	FEMALE	ASIAN		Stage IIIB	Serous Cy	G3	2008	-21902	Alive	TUMOR F	657							Complete Remission/Response	0	657	0	657	0	657	0	657
TCGA-36-	TCGA-36-	OV	85	FEMALE			Stage IIIC	Serous Cy	G3	2008	-31211	Dead	WITH TUM	68	68		Progression of Disease			27	Progressive Disease	1	68	1	68			1	27
TCGA-36-	TCGA-36-	OV	58	FEMALE			Stage IIIC	Serous Cy	G3	2008	-21384	Alive	WITH TUM	638			Progression of Disease			325	Partial Remission/Response	0	638	0	638			1	325
TCGA-36-	TCGA-36-	OV	42	FEMALE			Stage IIIA	Serous Cy	G3	2008	-15444	Alive	TUMOR F	557								0	557	0	557			0	557
TCGA-36-	TCGA-36-	OV	64	FEMALE			Stage IIIC	Serous Cy	G3	2008	-23706	Alive		624								0	624	0	624			0	624
TCGA-36-	TCGA-36-	OV	57	FEMALE			Stage IIIC	Serous Cy	G3	2008	-20897	Alive	WITH TUM	431			Recurrence			395		0	431	0	431			1	395
TCGA-36-	TCGA-36-	OV	54	FEMALE			Stage IIC	Serous Cy	G3	2008	-20076	Alive	TUMOR F	184								0	184	0	184			0	184
TCGA-36-	TCGA-36-	OV	57	FEMALE	WHITE		Stage IIIC	Serous Cy	G3	2009	-21045	Alive	WITH TUM	492			Recurrence			338	Complete Remission/Response	0	492	0	492	1	338	1	338
TCGA-36-	TCGA-36-	OV	64	FEMALE			Stage IIC	Serous Cy	G3	2009	-23379	Alive	TUMOR F	349								0	349	0	349			0	349
TCGA-3P-	TCGA-3P-	OV	55	FEMALE	BLACK OR AFRICAN		Stage IIB	Serous Cy	G3	2013	-20211	Alive	TUMOR F	420							Complete Remission/Response	0	420	0	420	0	420	0	420
TCGA-42-	TCGA-42-	OV	49	FEMALE	WHITE		Stage IIIC	Serous Cy	G3	2009	-17961	Dead	WITH TUMOR		1447		Recurrence			616	Stable Disease	1	1447	1	1447			1	616
TCGA-42-	TCGA-42-	OV	75	FEMALE	WHITE		Stage IIIC	Serous Cy	G3	2009	-27510	Alive	TUMOR F	722							Complete Remission/Response	0	722	0	722	0	722	0	722
TCGA-42-	TCGA-42-	OV	61	FEMALE	WHITE		Stage IV	Serous Cy	G3	2009	-22377	Dead	WITH TUMOR		880		Progression of Disease			112	Progressive Disease	1	880	1	880			1	112
TCGA-42-	TCGA-42-	OV	54	FEMALE	WHITE		Stage IIIC	Serous Cy	G3	2009	-19852	Alive	WITH TUM	1278			Recurrence			364	Complete Remission/Response	0	1278	0	1278	1	364	1	364
TCGA-42-	TCGA-42-	OV	57	FEMALE	WHITE		Stage IV	Serous Cy	G2	2009	-20965	Dead	WITH TUMOR		317		Progression of Disease			226	Progressive Disease	1	317	1	317			1	226
TCGA-42-	TCGA-42-	OV	51	FEMALE	WHITE		Stage IIIC	Serous Cy	GX	2009	-18654	Alive	TUMOR F	1240							Complete Remission/Response	0	1240	0	1240	0	1240	0	1240
TCGA-42-	TCGA-42-	OV	67	FEMALE	AMERICAN INDIAN (		Stage IIIC	Serous Cy	G3	2009	-24823	Dead	WITH TUMOR		45							1	45	1	45			1	45
TCGA-57-	TCGA-57-	OV	50	FEMALE	WHITE		Stage IIIC	Serous Cy	G3	2006		Dead	WITH TUM	731	731						Progressive Disease	1	731	1	731			1	731
TCGA-57-	TCGA-57-	OV	57	FEMALE	BLACK OR AFRICAN		Stage IIIC	Serous Cy	G3	2006	-21167	Dead	WITH TUM	346	346							1	346	1	346			1	346
TCGA-57-	TCGA-57-	OV	47	FEMALE	WHITE		Stage IIIC	Serous Cy	G3	2007	-17263	Alive	WITH TUM	643			Recurrence			530		0	643	0	643			1	530
TCGA-57-	TCGA-57-	OV	57	FEMALE	WHITE		Stage IIIC	Serous Cy	G3	2007	-20862	Dead	WITH TUM	53	53						Progressive Disease	1	53	1	53			1	53
TCGA-57-	TCGA-57-	OV	66	FEMALE	WHITE		Stage IIIC	Serous Cy	G3	2007	-24304	Alive	WITH TUM	679			Recurrence			259		0	679	0	679			1	259
TCGA-57-	TCGA-57-	OV	62	FEMALE			Stage IC	Serous Cy	G3	2006		Alive	TUMOR F	882							Complete Remission/Response	0	882	0	882	0	882	0	882
TCGA-57-	TCGA-57-	OV	56	FEMALE	WHITE		Stage IIIC	Serous Cy	G3	2007	-20498	Alive	TUMOR F	763								0	763	0	763			0	763
TCGA-57-	TCGA-57-	OV	63	FEMALE	WHITE			Serous Cystadenocar		2004	-23062	Alive		761								0	761	0	761			0	761
TCGA-59-	TCGA-59-	OV	59	FEMALE	WHITE		Stage IIIC	Serous Cy	G3	1994		Alive	TUMOR F	5481							Complete Remission/Response	0	5481	0	5481	0	5481	0	5481
TCGA-59-	TCGA-59-	OV	71	FEMALE			Stage IA	Serous Cy	G3	1994		Dead			2437						Complete Remission/Response	1	2437		2437	0	2437	0	2437
TCGA-59-	TCGA-59-	OV	44	FEMALE			Stage IV	Serous Cy	G3	1995		Dead	WITH TUMOR		679		Progression of Disease			211	Partial Remission/Response	1	679	1	679			1	211
TCGA-59-	TCGA-59-	OV	51	FEMALE	WHITE		Stage IIIC	Serous Cy	G3	1999		Alive	TUMOR F	3532			Progression of Disease			2139	Complete Remission/Response	0	3532	0	3532	1	2139	1	2139
TCGA-59-	TCGA-59-	OV	78	FEMALE	WHITE		Stage IIIC	Serous Cy	G3	2002		Dead	WITH TUMOR		286							1	286	1	286			1	286
TCGA-59-	TCGA-59-	OV	63	FEMALE	WHITE		Stage IIIC	Serous Cy	G3	2005		Dead	WITH TUMOR		1046		Progression of Disease			462	Stable Disease	1	1046	1	1046			1	462
TCGA-59-	TCGA-59-	OV	58	FEMALE	WHITE		Stage IV	Serous Cy	G3	2006		Dead	WITH TUMOR		65							1	65	1	65			1	65
TCGA-59-	TCGA-59-	OV	40	FEMALE	ASIAN		Stage IIIA	Serous Cy	G3	2007		Alive	TUMOR F	165							Complete Remission/Response	0	165	0	165	0	165	0	165
TCGA-59-	TCGA-59-	OV	73	FEMALE	WHITE		Stage IIIC	Serous Cy	G2	2005		Dead			1363							1	1363		1363			0	1363
TCGA-59-	TCGA-59-	OV	55	FEMALE	BLACK OR AFRICAN		Stage IC	Serous Cy	G3	2011	-20263	Dead	WITH TUMOR		624		Recurrence			145	Complete Remission/Response	1	624	1	624	1	145	1	145
TCGA-5X-	TCGA-5X-	OV	61	FEMALE	BLACK OR AFRICAN		Stage IIC	Serous Cy	GX	2013	-22438	Alive	TUMOR F	361							Complete Remission/Response	0	361	0	361	0	361	0	361
TCGA-61-	TCGA-61-	OV	38	FEMALE	WHITE		Stage IV	Serous Cy	G1	2008	-13894	Alive	TUMOR F	338							Complete Remission/Response	0	338	0	338			0	338
TCGA-61-	TCGA-61-	OV	89	FEMALE	WHITE		Stage IB	Serous Cy	G3	2008	-32853	Alive	WITH TUM	246							Stable Disease	0	246	0	246			0	246
TCGA-61-	TCGA-61-	OV	47	FEMALE	WHITE		Stage IIIC	Serous Cy	G3	2007	-17168	Dead	WITH TUM	637	637						Progressive Disease	1	637	1	637			1	637
TCGA-61-	TCGA-61-	OV	40	FEMALE	WHITE		Stage IIIC	Serous Cy	G3	2007	-14782	Alive	WITH TUM	956			Recurrence			844	Stable Disease	0	956	0	956			1	844
TCGA-61-	TCGA-61-	OV	74	FEMALE	WHITE		Stage IC	Serous Cy	G3	2007	-27203	Alive	TUMOR F	564							Complete Remission/Response	0	564	0	564	0	564	0	564
TCGA-61-	TCGA-61-	OV	59	FEMALE	WHITE		Stage IV	Serous Cy	G3	2007	-21582	Alive	TUMOR F	848			Recurrence			533	Complete Remission/Response	0	848	0	848			1	533
TCGA-61-	TCGA-61-	OV	78	FEMALE	WHITE		Stage IC	Serous Cy	G3	2007	-28508	Dead	WITH TUM	132	132							1	132	1	132			1	132
TCGA-61-	TCGA-61-	OV	71	FEMALE	WHITE		Stage IIIC	Serous Cy	G3	2006	-26180	Alive	WITH TUM	967			Recurrence			175	Stable Disease	0	967	0	967			1	175
TCGA-61-	TCGA-61-	OV	52	FEMALE	WHITE		Stage IC	Serous Cy	G3	2006	-19026	Alive	TUMOR F	866							Complete Remission/Response	0	866	0	866	0	866	0	866
TCGA-61-	TCGA-61-	OV	45	FEMALE	WHITE		Stage IIIC	Serous Cy	G3	2005	-16613	Dead	WITH TUM	1484	1484		Recurrence			764	Complete Remission/Response	1	1484	1	1484	1	764	1	764
TCGA-61-	TCGA-61-	OV	42	FEMALE	WHITE		Stage IV	Serous Cy	G3	2005	-15515	Alive	TUMOR F	1364							Complete Remission/Response	0	1364	0	1364			0	1364
TCGA-61-	TCGA-61-	OV	60	FEMALE	BLACK OR AFRICAN		Stage IIIC	Serous Cy	G3	2005	-21965	Dead	WITH TUM	1089	1089		Recurrence			276	Progressive Disease	1	1089	1	1089			1	276
TCGA-61-	TCGA-61-	OV	71	FEMALE	WHITE		Stage IIIC	Serous Cy	G3	2005	-26169	Dead	WITH TUM	74	74						Progressive Disease	1	74	1	74			1	74
TCGA-61-	TCGA-61-	OV	76	FEMALE	WHITE		Stage IIIB	Serous Cy	G3	2004	-27762	Dead	WITH TUM	1024	1024		Recurrence			488	Complete Remission/Response	1	1024	1	1024	1	488	1	488
TCGA-61-	TCGA-61-	OV	53	FEMALE	WHITE		Stage IIC	Serous Cy	G2	2004	-19365	Dead	WITH TUM	1329	1329		Recurrence			809	Complete Remission/Response	1	1329	1	1329	1	809	1	809
TCGA-61-	TCGA-61-	OV	52	FEMALE	WHITE		Stage IIIB	Serous Cy	G3	2009	-19060	Alive	TUMOR F	44							Stable Disease	0	44	0	44			0	44
TCGA-61-	TCGA-61-	OV	81	FEMALE	WHITE		Stage IIIC	Serous Cy	G3	2008	-29692	Alive	TUMOR F	256							Complete Remission/Response	0	256	0	256	0	256	0	256
TCGA-61-	TCGA-61-	OV	51	FEMALE	BLACK OR AFRICAN		Stage IIIB	Serous Cy	G3	2008	-18963	Alive	TUMOR F	176							Stable Disease	0	176	0	176			0	176
TCGA-61-	TCGA-61-	OV	65	FEMALE	WHITE		Stage IV	Serous Cy	G3	2008	-24042	Dead	WITH TUM	347	347		Progression of Disease			329	Stable Disease	1	347	1	347			1	329
TCGA-61-	TCGA-61-	OV	55	FEMALE	WHITE		Stage IB	Serous Cy	G3	2007	-20381	Alive	TUMOR F	396							Complete Remission/Response	0	396	0	396	0	396	0	396
TCGA-61-	TCGA-61-	OV	60	FEMALE	WHITE		Stage IIIC	Serous Cy	G3	2007	-22009	Alive	TUMOR F	276							Complete Remission/Response	0	276	0	276	0	276	0	276
TCGA-61-	TCGA-61-	OV	55	FEMALE	WHITE		Stage IIIC	Serous Cy	G3	2006	-20378	Dead	WITH TUM	1038	1038		Recurrence			236	Complete Remission/Response	1	1038	1	1038	1	236	1	236
TCGA-61-	TCGA-61-	OV	63	FEMALE	WHITE		Stage IIIC	Serous Cy	G3	2006	-23204	Alive	WITH TUM	952			Recurrence			743	Stable Disease	0	952	0	952			1	743
TCGA-61-	TCGA-61-	OV	56	FEMALE	WHITE		Stage IIC	Serous Cy	G3	2006	-20779	Alive	TUMOR F	1127							Complete Remission/Response	0	1127	0	1127	0	1127	0	1127
TCGA-61-	TCGA-61-	OV	55	FEMALE	WHITE		Stage IIA	Serous Cy	G3	2005	-20144	Alive	WITH TUM	1293			Recurrence			844	Complete Remission/Response	0	1293	0	1293	1	844	1	844
TCGA-61-	TCGA-61-	OV	48	FEMALE	WHITE		Stage IIIB	Serous Cy	G3	2005	-17575	Alive	TUMOR F	1488							Complete Remission/Response	0	1488	0	1488	0	1488	0	1488
TCGA-61-	TCGA-61-	OV	65	FEMALE			Stage IIIC	Serous Cy	G3	2003	-23850	Alive	WITH TUM	1722			Recurrence			1674	Complete Remission/Response	0	1722	0	1722	1	1674	1	1674
TCGA-61-	TCGA-61-	OV	50	FEMALE	WHITE		Stage IIC	Serous Cy	G3	2003	-18424	Alive	TUMOR F	2061							Complete Remission/Response	0	2061	0	2061	0	2061	0	2061
TCGA-61-	TCGA-61-	OV	53	FEMALE	WHITE		Stage IIIC	Serous Cy	G2	2002	-19463	Dead		475	475		Recurrence			326		1	475		475			1	326
TCGA-61-	TCGA-61-	OV	53	FEMALE	WHITE		Stage IIIC	Serous Cy	G2	2002	-19463	Dead		475	475		Recurrence			326		1	475		475			1	326
TCGA-61-	TCGA-61-	OV	60	FEMALE	WHITE		Stage IIIB	Serous Cy	G3	2002	-21972	Dead		1321	1321						Complete Remission/Response	1	1321		1321	0	1321	0	1321
TCGA-61-	TCGA-61-	OV	45	FEMALE	WHITE		Stage IV	Serous Cy	G3	2002	-16689	Dead	WITH TUM	479	479		Recurrence			413	Complete Remission/Response	1	479	1	479			1	413
TCGA-61-	TCGA-61-	OV	58	FEMALE	WHITE		Stage IIIC	Serous Cy	G2	2001	-21199	Dead		1161	1161		Recurrence			439	Complete Remission/Response	1	1161		1161	1	439	1	439
TCGA-61-	TCGA-61-	OV	43	FEMALE	WHITE		Stage IIIC	Serous Cy	G3	2009	-16016	Alive	WITH TUM	61							Stable Disease	0	61	0	61			0	61
TCGA-61-	TCGA-61-	OV	48	FEMALE	WHITE		Stage IIIC	Serous Cy	G3	2009	-17855	Alive	WITH TUM	168								0	168	0	168			0	168
TCGA-61-	TCGA-61-	OV	67	FEMALE	WHITE		Stage IIIC	Serous Cy	G3	2008	-24723	Alive	WITH TUM	441			Recurrence			339	Stable Disease	0	441	0	441			1	339
TCGA-61-	TCGA-61-	OV	46	FEMALE	WHITE		Stage IIIC	Serous Cy	G3	2008	-17115	Alive	WITH TUM	547							Progressive Disease	0	547	0	547			0	547
TCGA-61-	TCGA-61-	OV	53	FEMALE	WHITE		Stage IIIC	Serous Cy	G2	2008	-19605	Alive	WITH TUM	122							Progressive Disease	0	122	0	122			0	122
TCGA-61-	TCGA-61-	OV	40	FEMALE	ASIAN		Stage IIC	Serous Cy	G2	2007	-14791	Alive	WITH TUM	932			Recurrence			818	Complete Remission/Response	0	932	0	932	1	818	1	818
TCGA-61-	TCGA-61-	OV	40	FEMALE	ASIAN		Stage IIC	Serous Cy	G2	2007	-14791	Alive	WITH TUM	932			Recurrence			818	Complete Remission/Response	0	932	0	932	1	818	1	818
TCGA-61-	TCGA-61-	OV	65	FEMALE	WHITE		Stage IIIC	Serous Cy	G3	2006	-23772	Alive	WITH TUM	1212			Recurrence			101	Complete Remission/Response	0	1212	0	1212	1	101	1	101
TCGA-61-	TCGA-61-	OV	81	FEMALE	WHITE		Stage IIC	Serous Cy	G2	2006	-29830	Alive		932								0	932	0	932			0	932
TCGA-61-	TCGA-61-	OV	51	FEMALE	WHITE		Stage IIIC	Serous Cy	G3	2006	-18788	Dead	WITH TUMOR		36						Progressive Disease	1	36	1	36			1	36
TCGA-61-	TCGA-61-	OV	64	FEMALE	WHITE		Stage IIIC	Serous Cy	G1	2005	-23509	Alive	WITH TUM	1398			Recurrence			1006	Complete Remission/Response	0	1398	0	1398	1	1006	1	1006
TCGA-61-	TCGA-61-	OV	62	FEMALE	WHITE		Stage IC	Serous Cy	G3	2005	-22929	Alive	TUMOR F	1381							Complete Remission/Response	0	1381	0	1381	0	1381	0	1381
TCGA-61-	TCGA-61-	OV	49	FEMALE	WHITE		Stage IC	Serous Cy	G3	2009	-18182	Alive	TUMOR F	146							Complete Remission/Response	0	146	0	146	0	146	0	146
TCGA-61-	TCGA-61-	OV	51	FEMALE	WHITE		Stage IIIC	Serous Cy	G3	2009	-18770	Alive	TUMOR F	145								0	145	0	145			0	145
TCGA-61-	TCGA-61-	OV	57	FEMALE	WHITE		Stage IIIC	Serous Cy	G3	2004	-21072	Alive	TUMOR F	1573							Complete Remission/Response	0	1573	0	1573	0	1573	0	1573
TCGA-61-	TCGA-61-	OV	63	FEMALE	WHITE		Stage IIIC	Serous Cy	G3	2003	-23273	Alive	TUMOR F	2182							Complete Remission/Response	0	2182	0	2182	0	2182	0	2182
TCGA-61-	TCGA-61-	OV	54	FEMALE	WHITE		Stage IIIC	Serous Cy	G2	2003	-19797	Dead	WITH TUMOR		1875		Recurrence			445	Complete Remission/Response	1	1875	1	1875	1	445	1	445
TCGA-61-	TCGA-61-	OV	54	FEMALE	WHITE		Stage IIIC	Serous Cy	G2	2003	-19797	Dead	WITH TUMOR		1875		Recurrence			445	Complete Remission/Response	1	1875	1	1875	1	445	1	445
TCGA-61-	TCGA-61-	OV	56	FEMALE	WHITE		Stage IC	Serous Cy	G2	2003	-20663	Alive	TUMOR F	1776			Recurrence			421	Progressive Disease	0	1776	0	1776			1	421
TCGA-61-	TCGA-61-	OV	71	FEMALE	WHITE		Stage IIC	Serous Cy	G2	2003	-25961	Alive	TUMOR F	1844							Complete Remission/Response	0	1844	0	1844	0	1844	0	1844
TCGA-61-	TCGA-61-	OV	62	FEMALE	WHITE		Stage IIIC	Serous Cy	G2	2003	-22876	Alive	TUMOR F	1993							Complete Remission/Response	0	1993	0	1993	0	1993	0	1993
TCGA-61-	TCGA-61-	OV	55	FEMALE	WHITE		Stage IIIC	Serous Cy	G2	1999	-20287	Dead	WITH TUMOR		1688						Partial Remission/Response	1	1688	1	1688			1	1688
TCGA-61-	TCGA-61-	OV	74	FEMALE	WHITE		Stage IIIC	Serous Cy	G3	1999	-27256	Dead	WITH TUM	197	197							1	197	1	197			1	197
TCGA-61-	TCGA-61-	OV	53	FEMALE	WHITE		Stage IIC	Serous Cy	G2	2003	-19432	Alive		2338			Recurrence			1680	Complete Remission/Response	0	2338	0	2338	1	1680	1	1680
TCGA-61-	TCGA-61-	OV	40	FEMALE	WHITE		Stage IIIC	Serous Cy	G3	1999	-14900	Dead	WITH TUM	629	629		Recurrence			465		1	629	1	629			1	465
TCGA-61-	TCGA-61-	OV	56	FEMALE	WHITE		Stage IIIC	Serous Cystadenocar		1998	-20718	Dead	WITH TUM	1354	1354		Recurrence			213	Progressive Disease	1	1354	1	1354			1	213
TCGA-61-	TCGA-61-	OV	61	FEMALE	WHITE		Stage IV	Serous Cy	G3	1998	-22517	Alive	WITH TUM	3825			Recurrence			2205	Complete Remission/Response	0	3825	0	3825			1	2205
TCGA-61-	TCGA-61-	OV	53	FEMALE	WHITE		Stage IIC	Serous Cy	G3	1999	-19722	Dead		676	676		Recurrence			291		1	676		676			1	291
TCGA-61-	TCGA-61-	OV	61	FEMALE	WHITE		Stage IIIC	Serous Cy	G3	1998	-22481	Dead	WITH TUM	1579	1579		Recurrence			524	Stable Disease	1	1579	1	1579			1	524
TCGA-61-	TCGA-61-	OV	40	FEMALE	WHITE		Stage IIIC	Serous Cy	G3	2008	-14782	Dead	WITH TUM	483	483		Recurrence			383	Complete Remission/Response	1	483	1	483	1	383	1	383
TCGA-61-	TCGA-61-	OV	63	FEMALE	WHITE		Stage IIIC	Serous Cy	G3	2002	-23074	Dead	WITH TUM	193	193		Recurrence			179	Stable Disease	1	193	1	193			1	179
TCGA-61-	TCGA-61-	OV	73	FEMALE	WHITE		Stage IIIC	Serous Cy	G3	1998	-26779	Dead	WITH TUM	945	945		Recurrence			358	Stable Disease	1	945	1	945			1	358
TCGA-61-	TCGA-61-	OV	71	FEMALE	WHITE		Stage IIIC	Serous Cy	G3	2007	-26142	Dead	WITH TUM	262	262		Recurrence			38	Stable Disease	1	262	1	262			1	38
TCGA-OY	TCGA-OY	OV	48	FEMALE	BLACK OR AFRICAN		Stage IIIB	Serous Cy	G3	2010	-17580	Alive	WITH TUM	1207			Recurrence			794	Complete Remission/Response	0	1207	0	1207	1	794	1	794
TCGA-OY	TCGA-OY	OV	78	FEMALE	BLACK OR AFRICAN		Stage IIA	Serous Cy	G3	2012	-28623	Alive	TUMOR F	576							Complete Remission/Response	0	576	0	576	0	576	0	576
TCGA-VG	TCGA-VG	OV	55	FEMALE	BLACK OR AFRICAN		Stage IV	Serous Cy	GB	2013	-20274	Dead	WITH TUMOR		24							1	24	1	24			1	24
TCGA-WF	TCGA-WF	OV	72	FEMALE	BLACK OR AFRICAN		Stage IIIC	Serous Cy	G3	2013	-26418	Dead	WITH TUMOR		304		Progression of Disease			245	Progressive Disease	1	304	1	304			1	245
TCGA-2J-	TCGA-2J-	PAAD	65	MALE	WHITE	Stage IIB		Pancreas-	G3	2012	-23962	Dead	TUMOR FREE		66	Pancreatic Cancer					Complete Remission/Response	1	66	1	66	0	66	0	66
TCGA-2J-	TCGA-2J-	PAAD	48	MALE	WHITE	Stage IIB		Pancreas-	G2	2012	-17794	Alive	TUMOR F	729							Complete Remission/Response	0	729	0	729	0	729	0	729
TCGA-2J-	TCGA-2J-	PAAD	75	MALE	WHITE	Stage IIA		Pancreas-	G2	2012	-27600	Dead	WITH TUMOR		293	Pancreati	Distant M	Liver		169	Progressive Disease	1	293	1	293			1	169
TCGA-2J-	TCGA-2J-	PAAD	71	MALE	WHITE	Stage IIB		Pancreas-	G3	2012	-26028	Alive	TUMOR F	80							[Unknown]	0	80	0	80			0	80
TCGA-2J-	TCGA-2J-	PAAD	70	FEMALE	WHITE	Stage IIB		Pancreas-	G1	2012	-25920	Dead	WITH TUMOR		627	Pancreati	Distant M	Liver		521	Complete Remission/Response	1	627	1	627	1	521	1	521
TCGA-2J-	TCGA-2J-	PAAD	55	MALE	WHITE	Stage IIB		Pancreas-	G2	2012	-20133	Dead	WITH TUMOR		607	Pancreati	Distant M	Other, sp	subcut nc	160	Progressive Disease	1	607	1	607			1	160
TCGA-2J-	TCGA-2J-	PAAD	73	MALE	WHITE	Stage IIA		Pancreas-	G2	2012	-26943	Alive	TUMOR F	676							Complete Remission/Response	0	676	0	676	0	676	0	676
TCGA-2J-	TCGA-2J-	PAAD	73	MALE	WHITE	Stage IIB		Pancreas-	G3	2012	-26936	Dead	WITH TUMOR		691	Pancreati	Distant M	Other, sp	Liver/lunc	443	Progressive Disease	1	691	1	691			1	443
TCGA-2J-	TCGA-2J-	PAAD	61	MALE	WHITE	Stage IIA		Pancreas-	G3	2011	-22425	Alive	WITH TUM	1287			Distant M	Other, sp	perigastri	1287	Complete Remission/Response	0	1287	0	1287	1	1287	1	1287
TCGA-2J-	TCGA-2J-	PAAD	55	FEMALE	WHITE	Stage IIA		Pancreas-	G3	2011	-20316	Alive	WITH TUM	969			Distant M	Liver		728	Progressive Disease	0	969	0	969			1	728
TCGA-2J-	TCGA-2J-	PAAD	71	MALE	WHITE	Stage IIB		Pancreas-	G2	2012	-26188	Alive	TUMOR F	484							Complete Remission/Response	0	484	0	484	0	484	0	484
TCGA-2J-	TCGA-2J-	PAAD	43	MALE	WHITE	Stage IIB		Pancreas-	G2	2013	-15869	Alive	WITH TUM	440			Distant M	Liver		439	Progressive Disease	0	440	0	440			1	439
TCGA-2J-	TCGA-2J-	PAAD	58	FEMALE	WHITE	Stage IIB		Pancreas-	G4	2013	-21484	Alive	TUMOR F	463							Complete Remission/Response	0	463	0	463	0	463	0	463
TCGA-2J-	TCGA-2J-	PAAD	60	FEMALE	WHITE	Stage IIA		Pancreas-	G3	2013	-22131	Alive	WITH TUM	438			Distant M	Liver		327	Progressive Disease	0	438	0	438			1	327
TCGA-2J-	TCGA-2J-	PAAD	72	FEMALE	WHITE	Stage IIB		Pancreas-	G2	2012	-26379	Alive		319							[Unknown]	0	319	0	319			0	319
TCGA-2J-	TCGA-2J-	PAAD	56	MALE	WHITE	Stage IIB		Pancreas-	G3	2012	-20641	Dead	WITH TUMOR		277	Pancreati	Distant M	Liver		154	Progressive Disease	1	277	1	277			1	154
TCGA-2J-	TCGA-2J-	PAAD	74	MALE	WHITE	Stage IIB		Pancreas-	G4	2012	-27082	Dead	WITH TUMOR		652	Pancreati	Distant M	Liver		126	Progressive Disease	1	652	1	652			1	126
TCGA-2L-	TCGA-2L-	PAAD	76	MALE	WHITE	Stage IIB		Pancreas-	G2	2010	-28074	Dead	WITH TUMOR		143	Pancreatic Cancer					Progressive Disease	1	143	1	143			1	143
TCGA-2L-	TCGA-2L-	PAAD	56	MALE	WHITE	Stage IIB		Pancreas-	G2	2011	-20618	Dead	WITH TUMOR		684	Pancreatic Cancer					Progressive Disease	1	684	1	684			1	684
TCGA-2L-	TCGA-2L-	PAAD	66	MALE	WHITE	Stage IIB		Pancreas-	G3	2011	-24381	Dead	WITH TUMOR		103	Pancreatic Cancer					Progressive Disease	1	103	1	103			1	103
TCGA-2L-	TCGA-2L-	PAAD	49	FEMALE	WHITE	Stage III		Pancreas-	G2	2010	-18103	Dead	WITH TUMOR		394	Pancreatic Cancer					Progressive Disease	1	394	1	394			1	394
TCGA-2L-	TCGA-2L-	PAAD	82	MALE	WHITE	Stage IIB		Pancreas-	G3	2011	-30092	Dead	WITH TUMOR		292	Pancreatic Cancer					Progressive Disease	1	292	1	292			1	292
TCGA-2L-	TCGA-2L-	PAAD	52	MALE	WHITE	Stage IIB		Pancreas-	G1	2010	-19315	Alive	TUMOR F	1383							Stable Disease	0	1383	0	1383			0	1383
TCGA-3A	TCGA-3A	PAAD	57	MALE	WHITE	[Discrepancy]		Pancreas-	G1	2009	-21175	Alive	TUMOR F	1794							Complete Remission/Response	0	1794	0	1794	0	1794	0	1794
TCGA-3A	TCGA-3A	PAAD	45	MALE	BLACK OI	Stage IIB		Pancreas-	G2	2010	-16766	Alive	WITH TUM	1323			Locoregic	Peritoneal Surfaces		1309	[Unknown]	0	1323	0	1323			1	1309
TCGA-3A	TCGA-3A	PAAD	67	MALE	WHITE	Stage IIA		Pancreas-	G2	2011	-24731	Dead	WITH TUMOR		634	Pancreati	Locoregic	Liver		513	Stable Disease	1	634	1	634			1	513
TCGA-3A	TCGA-3A	PAAD	69	FEMALE	WHITE	Stage IIB		Pancreas-	G3	2011	-25312	Dead			224						[Unknown]	1	224		224			0	224
TCGA-3A	TCGA-3A	PAAD	61	FEMALE	WHITE	Stage IIB		Pancreas-	G2	2011	-22400	Dead	WITH TUMOR		738	Pancreatic Cancer					Stable Disease	1	738	1	738			1	738
TCGA-3A	TCGA-3A	PAAD	66	FEMALE	WHITE	Stage IA		Pancreas-	G2	2012	-24174	Alive	WITH TUM	1021			Distant M	Lung		763	Stable Disease	0	1021	0	1021			1	763
TCGA-3A	TCGA-3A	PAAD	65	MALE	WHITE	Stage IB		Pancreas-	G1	2009	-23791	Alive	TUMOR F	1854							Complete Remission/Response	0	1854	0	1854	0	1854	0	1854
TCGA-3A	TCGA-3A	PAAD	39	FEMALE	WHITE	Stage I		Pancreas-	G1	2007	-14599	Alive	TUMOR F	2741							[Unknown]	0	2741	0	2741			0	2741
TCGA-3A	TCGA-3A	PAAD	62	FEMALE	WHITE	Stage IB		Pancreas-	G2	2008	-22799	Alive	TUMOR F	2084							Complete Remission/Response	0	2084	0	2084	0	2084	0	2084
TCGA-3A	TCGA-3A	PAAD	55	MALE	WHITE			Pancreas-	G1	2009	-20451	Alive	TUMOR F	1942							[Unknown]	0	1942	0	1942			0	1942
TCGA-3A	TCGA-3A	PAAD	64	FEMALE	WHITE	Stage IB		Pancreas-	G1	2010	-23406	Alive	TUMOR F	1542							Complete Remission/Response	0	1542	0	1542	0	1542	0	1542
TCGA-3A	TCGA-3A	PAAD	67	MALE	WHITE	Stage IB		Pancreas-	G1	2010	-24621	Alive	TUMOR F	998							Complete Remission/Response	0	998	0	998	0	998	0	998
TCGA-3A	TCGA-3A	PAAD	65	MALE	WHITE	Stage IIB		Pancreas-	G3	2011	-23998	Dead	WITH TUMOR		458	Pancreati	Distant M	Liver		371	[Unknown]	1	458	1	458			1	371
TCGA-3A	TCGA-3A	PAAD	59	FEMALE	WHITE	Stage IB		Pancreas-	G1	2011	-21732	Alive	TUMOR F	1103							Complete Remission/Response	0	1103	0	1103	0	1103	0	1103
TCGA-3A	TCGA-3A	PAAD	40	MALE	WHITE	Stage IA		Pancreas-	G2	2012	-14729	Alive	TUMOR F	1037							Complete Remission/Response	0	1037	0	1037	0	1037	0	1037
TCGA-3A	TCGA-3A	PAAD	47	MALE	WHITE	Stage IIB		Pancreas-	G2	2012	-17294	Dead	WITH TUMOR		308	Pancreati	Locoregic	Tumor Bed		109	Progressive Disease	1	308	1	308			1	109
TCGA-3A	TCGA-3A	PAAD	75	MALE	WHITE	Stage IIB		Pancreas-	G2	2012	-27453	Alive	WITH TUM	743			Distant M	Lung		363	Progressive Disease	0	743	0	743			1	363
TCGA-3E-	TCGA-3E-	PAAD	67	MALE	WHITE	Stage IIB		Pancreas-	G3	2008	-24760	Alive	[Discrepar	2285			Distant M	Lung		987	Complete Remission/Response	0	2285	0	2285	1	987	1	987
TCGA-3E-	TCGA-3E-	PAAD	71	MALE	WHITE	Stage IIA		Pancreas-	G2	2008	-26234	Dead	WITH TUMOR		2182	Other, sp	New Prim	Lung		1600	[Unknown]	1	2182	0	2182			1	1600
TCGA-F2-	TCGA-F2-	PAAD	57	MALE	WHITE	Stage IIB		Pancreas-	G2	2009	-20984	Dead	WITH TUMOR		334		Distant Metastasis			183	Complete Remission/Response	1	334	1	334	1	183	1	183
TCGA-F2-	TCGA-F2-	PAAD	70	MALE	WHITE	Stage IIB		Pancreas-	G1	2009	-25809	Alive	TUMOR F	295							Complete Remission/Response	0	295	0	295	0	295	0	295
TCGA-F2-	TCGA-F2-	PAAD	54	MALE	BLACK OI	Stage IIB		Pancreas-	G3	2011	-20071	Dead	WITH TUMOR		592	Pancreati	Distant M	Liver		256	Progressive Disease	1	592	1	592			1	256
TCGA-F2-	TCGA-F2-	PAAD	64	MALE	WHITE	Stage IIB		Pancreas-	G1	2011	-23713	Dead	WITH TUMOR		216	Pancreatic Cancer					Progressive Disease	1	216	1	216			1	216
TCGA-F2-	TCGA-F2-	PAAD	79	FEMALE	WHITE	Stage IIB		Pancreas-	G2	2012	-29150	Dead	WITH TUMOR		233	Pancreati	Locoregic	Other, sp	malignant	214	Complete Remission/Response	1	233	1	233	1	214	1	214
TCGA-F2-	TCGA-F2-	PAAD	65	MALE	WHITE	Stage IIA		Pancreas-	G2	2012	-24007	Alive	TUMOR F	586							Complete Remission/Response	0	586	0	586	0	586	0	586
TCGA-F2-	TCGA-F2-	PAAD	77	MALE	WHITE	Stage IIB		Pancreas-	G3	2013	-28239	Dead	WITH TUMOR		95	Pancreatic Cancer					Progressive Disease	1	95	1	95			1	95
TCGA-F2-	TCGA-F2-	PAAD	76	MALE	WHITE	Stage IIA		Pancreas-	G2	2013	-28047	Alive	WITH TUM	517							Progressive Disease	0	517	0	517			0	517
TCGA-FB-	TCGA-FB-	PAAD	69	FEMALE	WHITE	Stage IIB		Pancreas-	G2	2012	-25412	Dead	WITH TUMOR		179						[Unknown]	1	179	1	179			1	179
TCGA-FB-	TCGA-FB-	PAAD	54	MALE	WHITE	Stage IIB		Pancreas-	G1	2012	-19904	Alive	TUMOR F	767			Locoregional Recurrence			620	Complete Remission/Response	0	767	0	767	1	620	1	620
TCGA-FB-	TCGA-FB-	PAAD	72	FEMALE	WHITE	Stage IIB		Pancreas-	G2	2012	-26635	Dead	TUMOR FREE		732	Pancreati	Distant M	Liver		378	Complete Remission/Response	1	732	1	732	1	378	1	378
TCGA-FB-	TCGA-FB-	PAAD	74	MALE	WHITE	Stage IB		Pancreas-	G3	2012	-27362	Dead	WITH TUMOR		498	Pancreati	Distant M	Liver		177	Progressive Disease	1	498	1	498			1	177
TCGA-FB-	TCGA-FB-	PAAD	71	FEMALE	WHITE	Stage IIB		Pancreas-	G2	2013	-26179	Dead			375	Pancreatic Cancer					Complete Remission/Response	1	375	1	375	0	375	0	375
TCGA-FB-	TCGA-FB-	PAAD	48	MALE	ASIAN	Stage IIA		Pancreas-	G2	2012	-17649	Dead	TUMOR FREE		353	Pancreatic Cancer					Complete Remission/Response	1	353	1	353	0	353	0	353
TCGA-FB-	TCGA-FB-	PAAD	71	MALE	WHITE	Stage IIB		Pancreas-	G3	2009	-26239	Dead	WITH TUMOR		485	Pancreati	Distant M	Lung		150	Progressive Disease	1	485	1	485			1	150
TCGA-FB-	TCGA-FB-	PAAD	65	MALE	WHITE	Stage IIB		Pancreas-	G2	2008	-24078	Dead			1130						Progressive Disease	1	1130		1130			0	1130
TCGA-FB-	TCGA-FB-	PAAD	62	FEMALE	WHITE	Stage IIB		Pancreas-	G2	2008	-22981	Alive		228							[Unknown]	0	228	0	228			0	228
TCGA-FB-	TCGA-FB-	PAAD	41	FEMALE	ASIAN	Stage IIA		Pancreas-	G2	2008	-15129	Dead	WITH TUMOR		381	Pancreatic Cancer					Progressive Disease	1	381	1	381			1	381
TCGA-FB-	TCGA-FB-	PAAD	71	MALE	WHITE	Stage IIB		Pancreas-	G2	2007	-26085	Dead	TUMOR FREE		1059						Complete Remission/Response	1	1059	0	1059	0	1059	0	1059
TCGA-FB-	TCGA-FB-	PAAD	54	MALE	WHITE	Stage IIB		Pancreas-	G3	2008	-19847	Alive	TUMOR F	716			Distant M	Other, sp	Adrenal a	716	Complete Remission/Response	0	716	0	716	1	716	1	716
TCGA-FB-	TCGA-FB-	PAAD	68	MALE	WHITE	Stage IIA		Pancreas-	G3	2011	-24988	Dead	TUMOR FREE		473						Complete Remission/Response	1	473	0	473	0	473	0	473
TCGA-FB-	TCGA-FB-	PAAD	49	MALE	WHITE	Stage IIB		Pancreas-	G2	2011	-18172	Dead	WITH TUMOR		123						Stable Disease	1	123	1	123			1	123
TCGA-FB-	TCGA-FB-	PAAD	81	FEMALE	WHITE	Stage IIB		Pancreas-	G3	2011	-29585	Dead	WITH TUMOR		153		Distant M	Liver		92	Partial Remission/Response	1	153	1	153			1	92

TCGA-FB	TCGA-FB	PAAD	65	FEMALE	WHITE	Stage IIB	Pancreas-	G2	2010	-23953	Dead	TUMOR FREE		31	Surgical Complications					Complete Remission/Response	1	31	0	31	0	31	0	31
TCGA-FB	TCGA-FB	PAAD	85	MALE	WHITE	Stage IA	Pancreas-	G2	2011	-31319	Dead	WITH TUMOR		244	Pancreati	Locoregic	Peritoneal Surfaces		213	Progressive Disease	1	244	1	244			1	213
TCGA-FZ	TCGA-FZ	PAAD	59	FEMALE	WHITE	Stage IIB	Pancreas-	G3	2007	-21611	Dead	WITH TUMOR		741	Pancreati	Distant M	Liver		55	Progressive Disease	1	741	1	741			1	55
TCGA-FZ	TCGA-FZ	PAAD	59	FEMALE	WHITE	Stage IIB	Pancreas-	G3	2007	-21611	Dead	WITH TUMOR		741	Pancreati	Distant M	Liver		55	Progressive Disease	1	741	1	741			1	55
TCGA-FZ	TCGA-FZ	PAAD	52	MALE	BLACK OI	Stage IIB	Pancreas-	G3	2007	-19287	Dead			61						[Unknown]	1	61		61			0	61
TCGA-FZ	TCGA-FZ	PAAD	52	MALE	BLACK OI	Stage IIB	Pancreas-	G3	2007	-19287	Dead			61						[Unknown]	1	61		61			0	61
TCGA-FZ	TCGA-FZ	PAAD	85	FEMALE	[Unknowr	Stage IIB	Pancreas-	GX	2007	-31074	Dead	WITH TUMOR		155		Locoregic	Peritoneal Surfaces		103	Progressive Disease	1	155	1	155			1	103
TCGA-FZ	TCGA-FZ	PAAD	81	MALE	WHITE	Stage IIA	Pancreas-	G2	2007	-29633	Dead	WITH TUMOR		1101	Pancreati	Distant M	Peritoneal Surfaces		740	Progressive Disease	1	1101	1	1101			1	740
TCGA-FZ	TCGA-FZ	PAAD	81	MALE	WHITE	Stage IIA	Pancreas-	G2	2007	-29633	Dead	WITH TUMOR		1101	Pancreati	Distant M	Peritoneal Surfaces		740	Progressive Disease	1	1101	1	1101			1	740
TCGA-FZ	TCGA-FZ	PAAD	71	MALE	WHITE	Stage IV	Pancreas-	G1	2008	-26216	Dead	WITH TUMOR		619						Progressive Disease	1	619	1	619			1	619
TCGA-FZ	TCGA-FZ	PAAD	71	MALE	WHITE	Stage IV	Pancreas-	G1	2008	-26216	Dead	WITH TUMOR		619						Progressive Disease	1	619	1	619			1	619
TCGA-FZ	TCGA-FZ	PAAD	83	MALE	WHITE	Stage IIA	Pancreas-	G3	2008	-30571	Dead	WITH TUMOR		480						Progressive Disease	1	480	1	480			1	480
TCGA-FZ	TCGA-FZ	PAAD	83	MALE	WHITE	Stage IIA	Pancreas-	G3	2008	-30571	Dead	WITH TUMOR		480						Progressive Disease	1	480	1	480			1	480
TCGA-FZ	TCGA-FZ	PAAD	73	FEMALE	WHITE	Stage III	Pancreas-	G2	2001	-26881	Dead	WITH TUMOR		541						Progressive Disease	1	541	1	541			1	541
TCGA-FZ	TCGA-FZ	PAAD	73	FEMALE	WHITE	Stage III	Pancreas-	G2	2001	-26881	Dead	WITH TUMOR		541						Progressive Disease	1	541	1	541			1	541
TCGA-H6	TCGA-H6	PAAD	56	FEMALE	WHITE	Stage IIB	Pancreas-	G3	2011	-20741	Alive	WITH TUI	392			Distant M	Liver		259	Complete Remission/Response	0	392	0	392	1	259	1	259
TCGA-H6	TCGA-H6	PAAD	56	FEMALE	WHITE	Stage IIB	Pancreas-	G3	2011	-20741	Alive	WITH TUI	392			Distant M	Liver		259	Complete Remission/Response	0	392	0	392	1	259	1	259
TCGA-H6	TCGA-H6	PAAD	88	FEMALE	WHITE	Stage IIB	Pancreas-	G3	2012	-32475	Dead	WITH TUMOR		421	Pancreati	Locoregic	Tumor Bed		363	Progressive Disease	1	421	1	421			1	363
TCGA-H6	TCGA-H6	PAAD	88	FEMALE	WHITE	Stage IIB	Pancreas-	G3	2012	-32475	Dead	WITH TUMOR		421	Pancreati	Locoregic	Tumor Bed		363	Progressive Disease	1	421	1	421			1	363
TCGA-H8	TCGA-H8	PAAD	53	MALE	WHITE	Stage IIA	Pancreas-	G2	2012	-19677	Alive	WITH TUI	671			Distant M	Liver		455	[Discrepancy]	0	671	0	671			1	455
TCGA-HV	TCGA-HV	PAAD	50	MALE	ASIAN	Stage IIA	Pancreas-	G2	2012	-18475	Dead	TUMOR FREE		128						Complete Remission/Response	1	128	0	128	0	128	0	128
TCGA-HV	TCGA-HV	PAAD	50	MALE	ASIAN	Stage IIA	Pancreas-	G2	2012	-18475	Dead	TUMOR FREE		128						Complete Remission/Response	1	128	0	128	0	128	0	128
TCGA-HV	TCGA-HV	PAAD	72	FEMALE	ASIAN	Stage IIA	Pancreas-	G2	2012	-26311	Alive		232							Complete Remission/Response	0	232	0	232	0	232	0	232
TCGA-HV	TCGA-HV	PAAD	61	FEMALE	ASIAN	Stage IIB	Pancreas-	G2	2012	-22329	Alive		289							Complete Remission/Response	0	289	0	289	0	289	0	289
TCGA-HV	TCGA-HV	PAAD	65	FEMALE	ASIAN	Stage IIB	Pancreas-	G1	2001	-24025	Dead	WITH TUMOR		2036	Other ma	Distant M	Lung		1511	Complete Remission/Response	1	2036	0	2036	1	1511	1	1511
TCGA-HV	TCGA-HV	PAAD	70	MALE	ASIAN	Stage IIA	Pancreas-	G2	2012	-25768	Alive		252			Distant M	Non-regional / Dista		123	Stable Disease	0	252	0	252			1	123
TCGA-HV	TCGA-HV	PAAD	72	MALE	ASIAN	Stage IIB	Pancreas-	GX	2012	-26322	Alive		978			Distant M	Liver		342	[Discrepancy]	0	978	0	978			1	342
TCGA-HV	TCGA-HV	PAAD	50	MALE	ASIAN	Stage IIB	Pancreas-	G3	2012	-18357	Alive		920			Distant M	Other, sp	bone	526	[Discrepancy]	0	920	0	920			1	526
TCGA-HV	TCGA-HV	PAAD	75	FEMALE	ASIAN	Stage IIB	Pancreas-	G2	2013	-27662	Dead	WITH TUMOR		532		Distant M	Other, sp	Omentur	375	[Discrepancy]	1	532	1	532			1	375
TCGA-HZ	TCGA-HZ	PAAD	77	MALE	WHITE	Stage IIB	Pancreas-	G1	2010	-28174	Dead	WITH TUMOR		661	Pancreati	Distant M	Liver		228	[Unknown]	1	661	1	661			1	228
TCGA-HZ	TCGA-HZ	PAAD	72	MALE	WHITE	Stage IIB	Pancreas-	G3	2011	-26573	Alive	WITH TUI	969			Distant M	Liver		542	Complete Remission/Response	0	969	0	969	1	542	1	542
TCGA-HZ	TCGA-HZ	PAAD	52	FEMALE	WHITE	Stage IIB	Pancreas-	G2	2011	-19064	Dead	WITH TUMOR		593	Pancreatic Cancer					[Unknown]	1	593	1	593			1	593
TCGA-HZ	TCGA-HZ	PAAD	71	MALE	WHITE	Stage IB	Pancreas-	G2	2011	-26167	Dead	TUMOR FREE		236	Other, non-malignant disease					Complete Remission/Response	1	236	0	236	0	236	0	236
TCGA-HZ	TCGA-HZ	PAAD	61	FEMALE	WHITE	Stage IIB	Pancreas-	G1	2011	-22413	Alive		4								0	4	0	4			0	4
TCGA-HZ	TCGA-HZ	PAAD	65	MALE	WHITE	Stage IIA	Pancreas-	G2	2011	-23760	Alive	TUMOR F	314							Complete Remission/Response	0	314	0	314	0	314	0	314
TCGA-HZ	TCGA-HZ	PAAD	60	FEMALE	WHITE	Stage IIA	Pancreas-	G2	2011	-22090	Alive	WITH TUI	840			Distant Metastasis	Liver		831	Complete Remission/Response	0	840	0	840	1	831	1	831
TCGA-HZ	TCGA-HZ	PAAD	66	MALE	WHITE	Stage IIB	Pancreas-	G2	2011	-24150	Dead	WITH TUMOR		614	Pancreati	Locoregic	Other, sp	Regional l	364	Complete Remission/Response	1	614	1	614	1	364	1	364
TCGA-HZ	TCGA-HZ	PAAD	57	MALE	BLACK OI	Stage IIB	Pancreas-	G1	2011	-21024	Dead	WITH TUMOR		518	Pancreati	Distant M	Lung		340	Progressive Disease	1	518	1	518			1	340
TCGA-HZ	TCGA-HZ	PAAD	69	MALE	WHITE	Stage III	Pancreas-	G2	2011	-25227	Alive	TUMOR F	706							Complete Remission/Response	0	706	0	706	0	706	0	706
TCGA-HZ	TCGA-HZ	PAAD	61	MALE	WHITE	Stage IIB	Pancreas-	G2	2011	-22433	Dead	WITH TUMOR		366						[Unknown]	1	366	1	366			1	366
TCGA-HZ	TCGA-HZ	PAAD	65	FEMALE	WHITE	Stage IIB	Pancreas-	G2	2011	-23868	Dead	WITH TUMOR		596	Pancreatic Cancer					Complete Remission/Response	1	596	1	596			1	596
TCGA-HZ	TCGA-HZ	PAAD	81	MALE	WHITE	Stage IIB	Pancreas-	G3	2012	-29674	Dead	WITH TUMOR		120	Pancreatic Cancer					Progressive Disease	1	120	1	120			1	120
TCGA-HZ	TCGA-HZ	PAAD	54	FEMALE	WHITE	Stage IIA	Pancreas-	G2	2012	-19839	Dead	WITH TUMOR		299	Pancreatic Cancer					[Unknown]	1	299	1	299			1	299
TCGA-HZ	TCGA-HZ	PAAD	69	FEMALE	WHITE	Stage IIB	Pancreas-	G1	2012	-25214	Dead	WITH TUMOR		378	Pancreatic Cancer					Progressive Disease	1	378	1	378			1	378
TCGA-HZ	TCGA-HZ	PAAD	73	MALE	WHITE	Stage IA	Pancreas-	G3	2012	-26947	Alive	TUMOR F	454							Complete Remission/Response	0	454	0	454	0	454	0	454
TCGA-HZ	TCGA-HZ	PAAD	58	FEMALE	WHITE	Stage IV	Pancreas-	G3	2012	-21501	Dead	WITH TUMOR		545	Pancreatic Cancer					Progressive Disease	1	545	1	545			1	545
TCGA-HZ	TCGA-HZ	PAAD	76	FEMALE	WHITE	Stage IIB	Pancreas-	G3	2012	-27929	Dead	TUMOR FREE		517	Other, non-malignant disease					Complete Remission/Response	1	517	0	517	0	517	0	517
TCGA-HZ	TCGA-HZ	PAAD	84	FEMALE	WHITE	Stage IA	Pancreas-	GX	2012	-30821	Dead	TUMOR FREE		151	Surgical Complications					Complete Remission/Response	1	151	0	151	0	151	0	151
TCGA-HZ	TCGA-HZ	PAAD	58	FEMALE	WHITE	Stage IIB	Pancreas-	G2	2012	-21491	Alive	WITH TUI	660							Progressive Disease	0	660	0	660			0	660
TCGA-HZ	TCGA-HZ	PAAD	68	FEMALE	WHITE	Stage IIB	Pancreas-	G2	2012	-24891	Alive	TUMOR F	491							Complete Remission/Response	0	491	0	491	0	491	0	491
TCGA-HZ	TCGA-HZ	PAAD	77	MALE	WHITE	Stage IIB	Pancreas-	G2	2012	-28454	Dead	WITH TUMOR		308	Pancreatic Cancer					Progressive Disease	1	308	1	308			1	308
TCGA-HZ	TCGA-HZ	PAAD	75	MALE	WHITE	Stage IIB	Pancreas-	G3	2011	-27637	Alive	WITH TUI	194							[Unknown]	0	194	0	194			0	194
TCGA-HZ	TCGA-HZ	PAAD	72	MALE	WHITE	Stage IIB	Pancreas-	G3	2012	-26503	Alive	WITH TUI	657							Progressive Disease	0	657	0	657			0	657
TCGA-HZ	TCGA-HZ	PAAD	77	FEMALE	WHITE	Stage IIB	Pancreas-	G2	2013	-28434	Dead	WITH TUMOR		160	Pancreati	Locoregic	Peritoneal Surfaces		98	Complete Remission/Response	1	160	1	160	1	98	1	98
TCGA-HZ	TCGA-HZ	PAAD	77	MALE	WHITE	Stage IIB	Pancreas-	G1	2013	-28403	Alive	TUMOR F	330							Complete Remission/Response	0	330	0	330	0	330	0	330
TCGA-HZ	TCGA-HZ	PAAD	55	FEMALE	WHITE	Stage IIB	Pancreas-	G2	2013	-20366	Alive	TUMOR F	33							[Unknown]	0	33	0	33			0	33
TCGA-HZ	TCGA-HZ	PAAD	76	MALE	WHITE	Stage IIB	Pancreas-	G1	2013	-27850	Alive	TUMOR F	0							[Unknown]	0	0	0	0			0	0
TCGA-HZ	TCGA-HZ	PAAD	81	MALE	WHITE	Stage IB	Pancreas-	G1	2013	-29631	Alive	TUMOR F	7							[Unknown]	0	7	0	7			0	7
TCGA-HZ	TCGA-HZ	PAAD	70	MALE	WHITE	Stage IV	Pancreas-	G2	2013	-25849	Alive	WITH TUI	603			Distant M	Peritoneal Surfaces		555	[Unknown]	0	603	0	603			1	555
TCGA-HZ	TCGA-HZ	PAAD	70	MALE	WHITE	Stage IV	Pancreas-	G2	2013	-25849	Alive	WITH TUI	603			Distant M	Peritoneal Surfaces		555	[Unknown]	0	603	0	603			1	555
TCGA-IB-	TCGA-IB-	PAAD	65	FEMALE	WHITE	Stage IV	Pancreas-	G2	2011	-24043	Dead	WITH TUMOR		394	Pancreati	Distant M	Other, sp	Omentur	306	Progressive Disease	1	394	1	394			1	306
TCGA-IB-	TCGA-IB-	PAAD	44	FEMALE	[Unknowr	Stage IIB	Pancreas-	G1	2007	-16126	Dead	WITH TUMOR		1502	Pancreati	Distant M	Liver		984	Progressive Disease	1	1502	1	1502			1	984
TCGA-IB-	TCGA-IB-	PAAD	60	MALE	WHITE	Stage IIB	Pancreas-	G2	2007	-22148	Dead	WITH TUMOR		145	Pancreati	Locoregic	Liver		132	Progressive Disease	1	145	1	145			1	132
TCGA-IB-	TCGA-IB-	PAAD	41	MALE	WHITE	Stage IIB	Pancreas-	G2	2008	-15188	Dead	WITH TUMOR		666	Pancreati	Distant M	Liver		390	Progressive Disease	1	666	1	666			1	390
TCGA-IB-	TCGA-IB-	PAAD	73	FEMALE	[Unknowr	Stage IIB	Pancreas-	G2	2007	-26773	Dead			467		Distant M	Liver		282	Partial Remission/Response	1	467		467			1	282
TCGA-IB-	TCGA-IB-	PAAD	64	FEMALE	WHITE	Stage IIB	Pancreas-	G2	2009	-23580	Dead	WITH TUMOR		603	Pancreati	Locoregional Recurrence			492	Complete Remission/Response	1	603	1	603	1	492	1	492
TCGA-IB-	TCGA-IB-	PAAD	49	FEMALE	ASIAN	Stage IIB	Pancreas-	G2	2011	-18129	Alive	TUMOR F	1116							Complete Remission/Response	0	1116	0	1116	0	1116	0	1116
TCGA-IB-	TCGA-IB-	PAAD	80	MALE	WHITE	Stage IIB	Pancreas-	G2	2008	-29292	Dead	WITH TUMOR		476	Other malignancy					Progressive Disease	1	476	0	476			1	476
TCGA-IB-	TCGA-IB-	PAAD	78	FEMALE	WHITE	Stage IIB	Pancreas-	G2	2010	-28554	Alive	TUMOR F	1257							Complete Remission/Response	0	1257	0	1257	0	1257	0	1257
TCGA-IB-	TCGA-IB-	PAAD	80	MALE	WHITE	Stage IIB	Pancreas-	G3	2010	-29319	Dead	TUMOR FREE		123	Other, non-malignant disease					Complete Remission/Response	1	123	0	123	0	123	0	123
TCGA-IB-	TCGA-IB-	PAAD	62	FEMALE	WHITE	Stage IIB	Pancreas-	G2	2009	-22990	Dead			110	Pancreatic Cancer					[Unknown]	1	110	1	110			0	110
TCGA-IB-	TCGA-IB-	PAAD	66	FEMALE	BLACK OI	Stage IIA	Pancreas-	G2	2008	-24117	Dead	WITH TUMOR		1332	Pancreati	Distant M	Other, sp	Retroperit	427	Stable Disease	1	1332	1	1332			1	427
TCGA-IB-	TCGA-IB-	PAAD	85	FEMALE	WHITE	Stage IIB	Pancreas-	G1	2011	-31393	Dead	WITH TUMOR		481	Pancreati	Locoregional Recurrence			352	Progressive Disease	1	481	1	481			1	352
TCGA-IB-	TCGA-IB-	PAAD	73	MALE	[Unknowr	Stage IB	Pancreas-	G3	2008	-26747	Dead	WITH TUMOR		598	Pancreati	Distant M	Liver		519	Progressive Disease	1	598	1	598			1	519
TCGA-IB-	TCGA-IB-	PAAD	49	FEMALE	WHITE	Stage IIB	Pancreas-	G1	2011	-18202	Dead	WITH TUMOR		913	Pancreati	Distant M	Other, sp	Lung and	525	Complete Remission/Response	1	913	1	913	1	525	1	525
TCGA-IB-	TCGA-IB-	PAAD	64	MALE	WHITE	Stage IIA	Pancreas-	G3	2009	-23703	Dead	WITH TUMOR		117	Pancreati	Distant M	Peritoneal Surfaces		80	Progressive Disease	1	117	1	117			1	80
TCGA-IB-	TCGA-IB-	PAAD	53	FEMALE	WHITE	Stage IIB	Pancreas-	G2	2008	-19718	Dead	WITH TUMOR		486	Pancreati	Locoregic	Liver		397	Progressive Disease	1	486	1	486			1	397
TCGA-IB-	TCGA-IB-	PAAD	79	FEMALE	WHITE	Stage III	Pancreas-	G1	2011	-29213	Alive	WITH TUI	462			Distant M	Lung		83	Partial Remission/Response	0	462	0	462			1	83
TCGA-IB-	TCGA-IB-	PAAD	59	MALE	WHITE	Stage IIB	Pancreas-	G2	2011	-21676	Alive	WITH TUI	522			Locoregic	Other, sp	Tumor Be	486	Complete Remission/Response	0	522	0	522	1	486	1	486
TCGA-IB-	TCGA-IB-	PAAD	71	MALE	WHITE	Stage IIB	Pancreas-	G2	2012	-26129	Dead	WITH TUMOR		365	Pancreati	Locoregic	Peritoneal Surfaces		276	Progressive Disease	1	365	1	365			1	276
TCGA-IB-	TCGA-IB-	PAAD	77	MALE	WHITE	Stage IIA	Pancreas-	G2	2011	-28317	Alive	WITH TUI	482			Locoregic	Other, sp	Superior I	300	[Unknown]	0	482	0	482			1	300
TCGA-IB-	TCGA-IB-	PAAD	56	FEMALE	WHITE	Stage IB	Pancreas-	G2	2012	-20760	Dead	WITH TUMOR		219		Distant M	Lung		197	Complete Remission/Response	1	219	1	219	1	197	1	197
TCGA-IB-	TCGA-IB-	PAAD	64	FEMALE	WHITE	Stage IIB	Pancreas-	G3	2011	-23431	Dead	WITH TUMOR		460	Pancreati	Distant M	Liver		66	Complete Remission/Response	1	460	1	460	1	66	1	66
TCGA-IB-	TCGA-IB-	PAAD	64	FEMALE	WHITE	Stage IIB	Pancreas-	G2	2012	-23672	Alive	TUMOR F	635							Complete Remission/Response	0	635	0	635	0	635	0	635
TCGA-IB-	TCGA-IB-	PAAD	63	MALE	WHITE	Stage IIB	Pancreas-	G2	2012	-23152	Alive	WITH TUI	666			Distant M	Liver		593	Partial Remission/Response	0	666	0	666			1	593
TCGA-IB-	TCGA-IB-	PAAD	65	MALE	WHITE	Stage IIB	Pancreas-	G3	2012	-23751	Dead	WITH TUMOR		41	Other, non-malignant disease					Partial Remission/Response	1	41	0	41			1	41
TCGA-IB-	TCGA-IB-	PAAD	57	MALE	WHITE	Stage IIB	Pancreas-	G2	2012	-20898	Dead	WITH TUMOR		250	Pancreati	Distant M	Liver		7	Progressive Disease	1	250	1	250			1	7
TCGA-IB-	TCGA-IB-	PAAD	81	MALE	WHITE	Stage IIB	Pancreas-	G3	2013	-29792	Alive	TUMOR F	483							Complete Remission/Response	0	483	0	483	0	483	0	483
TCGA-IB-	TCGA-IB-	PAAD	76	MALE	WHITE	Stage IIB	Pancreas-	G3	2013	-28013	Alive	WITH TUI	8							Partial Remission/Response	0	8	0	8			0	8
TCGA-IB-	TCGA-IB-	PAAD	74	FEMALE	WHITE	Stage IB	Pancreas-	G2	2013	-27128	Dead			144		Locoregic	Other, specify		131	Complete Remission/Response	1	144		144	1	131	1	131
TCGA-IB-	TCGA-IB-	PAAD	64	FEMALE	WHITE	Stage IIB	Pancreas-	G3	2013	-23622	Dead	WITH TUMOR		239		Distant M	Liver		74	Partial Remission/Response	1	239	1	239			1	74
TCGA-IB-	TCGA-IB-	PAAD	68	MALE	WHITE	Stage IIB	Pancreas-	G2	2013	-24864	Alive	TUMOR F	431							Complete Remission/Response	0	431	0	431	0	431	0	431
TCGA-IB-	TCGA-IB-	PAAD	50	MALE	WHITE	Stage IIB	Pancreas-	G2	2013	-18315	Dead	WITH TUMOR		183	Pancreati	Distant M	Non-regional / Dista		107	Partial Remission/Response	1	183	1	183			1	107
TCGA-IB-	TCGA-IB-	PAAD	67	MALE	WHITE	Stage IIB	Pancreas-	G1	2013	-24576	Alive	WITH TUI	338			Locoregic	Tumor Bed		318	Partial Remission/Response	0	338	0	338			1	318
TCGA-IB-	TCGA-IB-	PAAD	84	FEMALE	WHITE	Stage IIB	Pancreas-	G2	2013	-30684	Alive	WITH TUI	225								0	225	0	225			0	225
TCGA-IB-	TCGA-IB-	PAAD	65	MALE	WHITE	Stage IIB	Pancreas-	G1	2013	-23801	Alive	TUMOR F	287							Complete Remission/Response	0	287	0	287	0	287	0	287
TCGA-IB-	TCGA-IB-	PAAD	35	MALE	WHITE	Stage IIB	Pancreas-	G3	2013	-13127	Alive	TUMOR F	245							Complete Remission/Response	0	245	0	245	0	245	0	245
TCGA-IB-	TCGA-IB-	PAAD	49	MALE	WHITE	Stage IIB	Pancreas-	G2	2013	-18059	Alive	TUMOR F	404							Complete Remission/Response	0	404	0	404	0	404	0	404
TCGA-IB-	TCGA-IB-	PAAD	63	FEMALE	WHITE	Stage IIB	Pancreas-	G3	2013	-23306	Dead	WITH TUMOR		230		Locoregic	Tumor Bed		185	Complete Remission/Response	1	230	1	230	1	185	1	185
TCGA-L1-	TCGA-L1-	PAAD	48	FEMALE	WHITE	Stage IIB	Pancreas-	G3	2013	-17821	Dead	WITH TUMOR		278	Pancreati	Distant M	Liver		129	Progressive Disease	1	278	1	278			1	129
TCGA-LB	TCGA-LB	PAAD	74	FEMALE	BLACK OI	Stage IIB	Pancreas-	G2	2013	-27295	Dead			393	Other, non-malignant disease					Stable Disease	1	393	0	393			0	393
TCGA-LB	TCGA-LB	PAAD	64	FEMALE	BLACK OI	Stage IIA	Pancreas-	G1	2013	-23624	Alive	TUMOR F	379							[Unknown]	0	379	0	379			0	379
TCGA-LB	TCGA-LB	PAAD	63	FEMALE	WHITE	Stage IIB	Pancreas-	G3	2013	-23218	Dead	WITH TUMOR		313	Pancreati	Locoregic	Other, sp	omentum	290	[Unknown]	1	313	1	313			1	290
TCGA-M8	TCGA-M8	PAAD	48	FEMALE	[Not Evalı	Stage IIA	Pancreas-	G2		-17628	Alive	WITH TUI	584			Locoregic	Other, sp	retroperit	416	Partial Remission/Response	0	584	0	584			1	416
TCGA-OE	TCGA-OE	PAAD	75	MALE	WHITE	Stage IIA	Pancreas-	G1	2013	-27684	Dead			267		Distant M	Liver		146	Progressive Disease	1	267		267			1	146
TCGA-PZ	TCGA-PZ	PAAD	44	FEMALE	WHITE	Stage IIB	Pancreas-	G3	2012	-16255	Dead	WITH TUMOR		470	Pancreati	Distant M	Liver		393	[Unknown]	1	470	1	470			1	393
TCGA-O3	TCGA-O3	PAAD	58	MALE	WHITE	Stage IIB	Pancreas-	G2	2012	-21277	Alive		416							[Discrepancy]	0	416	0	416			0	416
TCGA-Q3	TCGA-Q3	PAAD	64	FEMALE	WHITE	Stage IB	Pancreas-	G1	2013	-23685	Alive	TUMOR F	95							[Unknown]	0	95	0	95			0	95
TCGA-RB	TCGA-RB	PAAD	81	FEMALE	WHITE	Stage IIB	Pancreas-	G2	2013	-29585	Dead	TUMOR FREE		466	Pancreati	Locoregional Recurrence			449	Complete Remission/Response	1	466	1	466	1	449	1	449
TCGA-RB	TCGA-RB	PAAD	43	MALE	WHITE	Stage IIB	Pancreas-	G3	2013	-15807	Alive	WITH TUI	286			Distant M	Liver		258	Progressive Disease	0	286	0	286			1	258
TCGA-RL	TCGA-RL	PAAD	60	FEMALE	WHITE	Stage IB	Pancreas-	G2	2013	-22126	Alive	TUMOR F	9							Complete Remission/Response	0	9	0	9	0	9	0	9
TCGA-S4	TCGA-S4	PAAD	67	MALE	WHITE	Stage IIB	Pancreas-	G3	2013	-24589	Alive	TUMOR F	737							Complete Remission/Response	0	737	0	737	0	737	0	737
TCGA-S4	TCGA-S4	PAAD	75	FEMALE	WHITE	Stage IIB	Pancreas-	G2	2013	-27645	Alive	WITH TUI	525			Distant M	Lung		291	Complete Remission/Response	0	525	0	525	1	291	1	291
TCGA-S4	TCGA-S4	PAAD	77	FEMALE	WHITE	Stage IIB	Pancreas-	G3	2010	-28353	Dead			702						[Unknown]	1	702		702			0	702
TCGA-US	TCGA-US	PAAD	76	FEMALE	WHITE	Stage IIB	Pancreas-	G3	2011	-27957	Dead	WITH TUMOR		695	Pancreati	Distant M	Liver		366	Progressive Disease	1	695	1	695			1	366
TCGA-US	TCGA-US	PAAD	61	MALE	WHITE	Stage IIA	Pancreas-	G2	2011	-22283	Alive	TUMOR F	1216							Complete Remission/Response	0	1216	0	1216	0	1216	0	1216
TCGA-US	TCGA-US	PAAD	54	FEMALE	WHITE	Stage IIB	Pancreas-	G1	2011	-20051	Dead	WITH TUMOR		511	Pancreatic Cancer						1	511	1	511			1	511
TCGA-US	TCGA-US	PAAD	73	MALE	WHITE	Stage IIB	Pancreas-	G3	2012	-26745	Dead	WITH TUMOR		430	Pancreati	Distant M	Liver		166		1	430	1	430			1	166
TCGA-US	TCGA-US	PAAD	64	MALE	WHITE	Stage IIB	Pancreas-	G2	2010	-23443	Dead	TUMOR FREE		12	Surgical Complications						1	12	0	12			0	12
TCGA-US	TCGA-US	PAAD	81	FEMALE	WHITE	Stage IIB	Pancreas-	G2	2010	-29904	Dead	WITH TUMOR		568	Pancreatic Cancer						1	568	1	568			1	568
TCGA-XD	TCGA-XD	PAAD	66	FEMALE	WHITE	Stage IV	Pancreas-	G2	2013	-24291	Alive	TUMOR F	420							[Discrepancy]	0	420	0	420			0	420
TCGA-XD	TCGA-XD	PAAD	57	FEMALE	WHITE	Stage IIB	Pancreas-	G2	2013	-20858	Alive		395			New Prim	Other, sp	Left shoul	87	[Unknown]	0	395	0	395			1	87
TCGA-XD	TCGA-XD	PAAD	50	FEMALE	WHITE	Stage IIB	Pancreas-	G2	2013	-18607	Dead	WITH TUMOR		366	Pancreati	Distant M	Liver		175	Progressive Disease	1	366	1	366			1	175
TCGA-XD	TCGA-XD	PAAD	56	MALE	WHITE	Stage IIA	Pancreas-	G2	2013	-20607	Alive	TUMOR F	498							Complete Remission/Response	0	498	0	498	0	498	0	498
TCGA-XN	TCGA-XN	PAAD	67	MALE	WHITE	Stage IB	Pancreas-	G2	2012	-24810	Alive	WITH TUI	951							Progressive Disease	0	951	0	951			0	951
TCGA-XN	TCGA-XN	PAAD	53	FEMALE	WHITE	Stage IB	Pancreas-	G2	2012	-19707	Alive	WITH TUI	720			Distant M	Liver		242	Progressive Disease	0	720	0	720			1	242
TCGA-YB	TCGA-YB	PAAD	59	MALE	WHITE	Stage IIB	Pancreas-	G2	2013	-21792	Alive	TUMOR F	350							Complete Remission/Response	0	350	0	350	0	350	0	350
TCGA-YB	TCGA-YB	PAAD	59	MALE	WHITE	Stage IIB	Pancreas-	G2	2013	-21792	Alive	TUMOR F	350							Complete Remission/Response	0	350	0	350	0	350	0	350
TCGA-YH	TCGA-YH	PAAD	73	FEMALE	WHITE	[Discrepancy]	[Discrepar	G2	2011	-26859	Alive		388							[Unknown]	0	388	0	388			0	388
TCGA-YY	TCGA-YY	PAAD	61	FEMALE	WHITE	Stage IIB	Pancreas-	G3	2009	-22376	Alive	TUMOR F	2016							Complete Remission/Response	0	2016	0	2016	0	2016	0	2016
TCGA-Z5	TCGA-Z5	PAAD	74	FEMALE	WHITE	Stage IIA	Pancreas-	G1	2013	-27152	Alive	TUMOR F	467							Complete Remission/Response	0	467	0	467	0	467	0	467
TCGA-P7	TCGA-P7	PCPG	78	FEMALE	WHITE		Pheochromocytoma		2012	-28497	Alive	TUMOR F	818							Complete Remission/Response	0	818	0	818	0	818	0	818
TCGA-P7	TCGA-P7	PCPG	21	FEMALE	WHITE		Pheochromocytoma		2011	-7834	Alive	TUMOR F	957							Complete Remission/Response	0	957	0	957	0	957	0	957
TCGA-P7	TCGA-P7	PCPG	21	FEMALE	WHITE		Pheochromocytoma		2011	-7834	Alive	TUMOR F	957							Complete Remission/Response	0	957	0	957	0	957	0	957
TCGA-P8	TCGA-P8	PCPG	48	MALE	WHITE		Pheochromocytoma		2011	-17790	Alive	TUMOR F	883							Complete Remission/Response	0	883	0	883	0	883	0	883
TCGA-P8	TCGA-P8	PCPG	48	MALE	WHITE		Pheochromocytoma		2011	-17790	Alive	TUMOR F	883							Complete Remission/Response	0	883	0	883	0	883	0	883
TCGA-P8	TCGA-P8	PCPG	35	FEMALE	WHITE		Pheochromocytoma		2012	-12954	Alive	TUMOR F	123							[Unknown]	0	123	0	123			0	123
TCGA-P8	TCGA-P8	PCPG	35	FEMALE	WHITE		Pheochromocytoma		2012	-12954	Alive	TUMOR F	123							[Unknown]	0	123	0	123			0	123
TCGA-P8	TCGA-P8	PCPG	65	MALE	WHITE		Paraganglioma		2013	-23825	Alive	TUMOR F	540							[Unknown]	0	540	0	540			0	540
TCGA-P8	TCGA-P8	PCPG	55	MALE	WHITE		Pheochromocytoma		2012	-20109	Alive	TUMOR F	179								0	179	0	179			0	179
TCGA-PR	TCGA-PR	PCPG	62	FEMALE	WHITE		Pheochromocytoma		2011	-22987	Alive	TUMOR F	546							Complete Remission/Response	0	546	0	546	0	546	0	546
TCGA-PR	TCGA-PR	PCPG	42	FEMALE	WHITE		Pheochromocytoma		2007	-15577	Alive	WITH TUI	2485			Distant M	Bone		2278	Complete Remission/Response	0	2485	0	2485	1	2278	1	2278
TCGA-PR	TCGA-PR	PCPG	47	MALE	WHITE		Pheochromocytoma		2007	-17347	Alive	TUMOR F	854							Complete Remission/Response	0	854	0	854	0	854	0	854
TCGA-QR	TCGA-QR	PCPG	43	MALE	WHITE		Pheochromocytoma		2009	-15799	Alive	TUMOR F	1065							Complete Remission/Response	0	1065	0	1065	0	1065	0	1065
TCGA-QR	TCGA-QR	PCPG	30	FEMALE	WHITE		Pheochromocytoma		2009	-11089	Alive	TUMOR F	703							Complete Remission/Response	0	703	0	703	0	703	0	703
TCGA-QR	TCGA-QR	PCPG	51	FEMALE	WHITE		Pheochromocytoma		2007	-18798	Alive	TUMOR F	38							Complete Remission/Response	0	38	0	38	0	38	0	38
TCGA-QR	TCGA-QR	PCPG	63	FEMALE	WHITE		Pheochromocytoma		2002	-23208	Alive	WITH TUI	4185			Distant M	Liver		1476	Progressive Disease	0	4185	0	4185			1	1476
TCGA-QR	TCGA-QR	PCPG	31	MALE	WHITE		Pheochromocytoma		2008	-11592	Alive	TUMOR F	1791							Complete Remission/Response	0	1791	0	1791		1791	0	1791
TCGA-QR	TCGA-QR	PCPG	53	FEMALE	WHITE		Pheochromocytoma		2009	-19639	Alive	TUMOR F	491							Complete Remission/Response	0	491	0	491	0	491	0	491
TCGA-QR	TCGA-QR	PCPG	28	FEMALE	BLACK OR AFRICAN AMERICAN		Pheochromocytoma		2011	-10548	Alive	TUMOR F	579							Complete Remission/Response	0	579	0	579	0	579	0	579
TCGA-QR	TCGA-QR	PCPG	37	FEMALE	WHITE		Pheochromocytoma		2006	-13612	Alive	TUMOR F	32							Complete Remission/Response	0	32	0	32	0	32	0	32
TCGA-QR	TCGA-QR	PCPG	31	MALE	WHITE		Pheochromocytoma		2009	-11573	Alive	TUMOR F	928							Complete Remission/Response	0	928	0	928	0	928	0	928
TCGA-QR	TCGA-QR	PCPG	31	MALE	WHITE		Pheochromocytoma		2009	-11573	Alive	TUMOR F	928							Complete Remission/Response	0	928	0	928	0	928	0	928
TCGA-QR	TCGA-QR	PCPG	53	MALE	WHITE		Paraganglioma; Extra		2012	-19372	Alive	TUMOR F	280							Complete Remission/Response	0	280	0	280	0	280	0	280
TCGA-QR	TCGA-QR	PCPG	55	MALE	WHITE		Pheochromocytoma		2009	-20295	Alive	TUMOR F	431							Complete Remission/Response	0	431	0	431	0	431	0	431
TCGA-QR	TCGA-QR	PCPG	33	MALE	ASIAN		Pheochromocytoma		2003	-12281	Alive	TUMOR F	302							Complete Remission/Response	0	302	0	302	0	302	0	302
TCGA-QR	TCGA-QR	PCPG	41	MALE	WHITE		Paraganglioma		2013	-15106	Alive	TUMOR F	434							Complete Remission/Response	0	434	0	434	0	434	0	434
TCGA-QR	TCGA-QR	PCPG	56	FEMALE	WHITE		Pheochromocytoma		2007	-20492	Alive	TUMOR F	2661							Complete Remission/Response	0	2661	0	2661	0	2661	0	2661
TCGA-QR	TCGA-QR	PCPG	30	FEMALE	BLACK OR AFRICAN AMERICAN		Pheochromocytoma		2008	-10987	Alive	TUMOR F	959							Complete Remission/Response	0	959	0	959	0	959	0	959
TCGA-QR	TCGA-QR	PCPG	27	MALE	WHITE		Paraganglioma		2012	-10180	Alive	TUMOR F	474							Complete Remission/Response	0	474	0	474	0	474	0	474
TCGA-QR	TCGA-QR	PCPG	30	FEMALE	WHITE		Pheochromocytoma		2013	-11075	Alive	TUMOR F	48							Complete Remission/Response	0	48	0	48	0	48	0	48
TCGA-QR	TCGA-QR	PCPG	67	MALE	WHITE		Pheochromocytoma		2011	-24734	Alive	WITH TUI	978			Distant M	Lung		606	Progressive Disease	0	978	0	978			1	606
TCGA-QR	TCGA-QR	PCPG	75	FEMALE	WHITE		Paraganglioma		2010	-27395	Alive	TUMOR F	39							Complete Remission/Response	0	39	0	39	0	39	0	39
TCGA-QR	TCGA-QR	PCPG	23	MALE	WHITE		Pheochromocytoma		2008	-8421	Alive	TUMOR F	1167			New Prim	Other, sp	Left Adrer	0	Complete Remission/Response	0	1167	0	1167	0	1167	1	0
TCGA-QR	TCGA-QR	PCPG	63	FEMALE	WHITE		Paraganglioma; Extra		2010	-23086	Alive	TUMOR F	317							Complete Remission/Response	0	317	0	317	0	317	0	317
TCGA-QR	TCGA-QR	PCPG	50	FEMALE	WHITE		Paraganglioma		2012	-18585	Alive	TUMOR F	125							Complete Remission/Response	0	125	0	125	0	125	0	125
TCGA-QR	TCGA-QR	PCPG	43	FEMALE	WHITE		Pheochromocytoma		2010	-15870	Alive	TUMOR F	398							Complete Remission/Response	0	398	0	398	0	398	0	398
TCGA-QR	TCGA-QR	PCPG	44	FEMALE	WHITE		Pheochromocytoma		2011	-16170	Alive	TUMOR F	35							Complete Remission/Response	0	35	0	35	0	35	0	35
TCGA-QR	TCGA-QR	PCPG	54	FEMALE	WHITE		Pheochromocytoma		2006	-19755	Alive	TUMOR F	2119							Complete Remission/Response	0	2119	0	2119	0	2119	0	2119
TCGA-QR	TCGA-QR	PCPG	51	FEMALE	BLACK OR AFRICAN AMERICAN		Pheochromocytoma		2004	-18628	Alive	TUMOR F	3534							Complete Remission/Response	0	3534	0	3534	0	3534	0	3534
TCGA-QR	TCGA-QR	PCPG	39	FEMALE	BLACK OR AFRICAN AMERICAN		Paraganglioma		2012	-14412	Alive	TUMOR F	7							Complete Remission/Response	0	7	0	7	0	7	0	7
TCGA-QR	TCGA-QR	PCPG	31	FEMALE	WHITE		Pheochromocytoma		2011	-11400	Alive	TUMOR F	192							Complete Remission/Response	0	192	0	192	0	192	0	192
TCGA-QR	TCGA-QR	PCPG	54	MALE	BLACK OR AFRICAN AMERICAN		Pheochromocytoma		2011	-19914	Alive	TUMOR F	71							Complete Remission/Response	0	71	0	71	0	71	0	71
TCGA-QR	TCGA-QR	PCPG	36	MALE	WHITE		Pheochromocytoma		2007	-13314	Alive	TUMOR F	816							Complete Remission/Response	0	816	0	816	0	816	0	816
TCGA-QR	TCGA-QR	PCPG	24	MALE	BLACK OR AFRICAN AMERICAN		Paraganglioma		2010	-9070	Alive	TUMOR F	910							Complete Remission/Response	0	910	0	910	0	910	0	910
TCGA-QR	TCGA-QR	PCPG	42	FEMALE	WHITE		Pheochromocytoma		2011	-15420	Alive	TUMOR F	399							Complete Remission/Response	0	399	0	399	0	399	0	399
TCGA-QR	TCGA-QR	PCPG	61	MALE	WHITE		Pheochromocytoma		2012	-22519	Alive	TUMOR F	274							Complete Remission/Response	0	274	0	274	0	274	0	274
TCGA-QR	TCGA-QR	PCPG	38	MALE	WHITE		Pheochromocytoma		2010	-14091	Alive	TUMOR F	1034							Complete Remission/Response	0	1034	0	1034	0	1034	0	1034
TCGA-QR	TCGA-QR	PCPG	32	MALE	BLACK OR AFRICAN AMERICAN		Pheochromocytoma		2008	-11781	Alive	TUMOR F	43							Complete Remission/Response	0	43	0	43	0	43	0	43
TCGA-QR	TCGA-QR	PCPG	35	FEMALE	WHITE		Pheochromocytoma		2013	-13076	Alive	TUMOR F	111							Complete Remission/Response	0	111	0	111	0	111	0	111
TCGA-QR	TCGA-QR	PCPG	45	FEMALE	WHITE		Paraganglioma		2004	-16655	Dead	WITH TUMOR		3563		Locoregic	Other, sp	L jugular	648	Progressive Disease	1	3563	1	3563			1	648
TCGA-QR	TCGA-QR	PCPG	33	FEMALE	WHITE		Paraganglioma; Extra		2012	-12394	Alive	TUMOR F	140							Complete Remission/Response	0	140	0	140	0	140	0	140
TCGA-QR	TCGA-QR	PCPG	43	MALE	WHITE		Pheochromocytoma		2012	-15917	Alive	TUMOR F	432							Complete Remission/Response	0	432	0	432	0	432	0	432
TCGA-QR	TCGA-QR	PCPG	40	FEMALE	BLACK OR AFRICAN AMERICAN		Paraganglioma		2011	-14780	Alive	TUMOR F	267							Complete Remission/Response	0	267	0	267	0	267	0	267
TCGA-QR	TCGA-QR	PCPG	46	FEMALE	WHITE		Pheochromocytoma		2009	-17062	Alive	TUMOR F	1473							Complete Remission/Response	0	1473	0	1473	0	1473	0	1473
TCGA-QR	TCGA-QR	PCPG	66	FEMALE	[Unknown]		Pheochromocytoma		2012	-24437	Alive	TUMOR F	2							Complete Remission/Response	0	2	0	2	0	2	0	2
TCGA-QR	TCGA-QR	PCPG	40	MALE	WHITE		Pheochromocytoma		2009	-14613	Alive	TUMOR F	1271							Complete Remission/Response	0	1271	0	1271	0	1271	0	1271
TCGA-QR	TCGA-QR	PCPG	46	FEMALE	BLACK OR AFRICAN AMERICAN		Pheochromocytoma		2012	-16828	Alive	TUMOR F	183							Complete Remission/Response	0	183	0	183	0	183	0	183
TCGA-QR	TCGA-QR	PCPG	52	FEMALE	BLACK OR AFRICAN AMERICAN		Pheochromocytoma		2011	-19237	Alive	TUMOR F	377							Complete Remission/Response	0	377	0	377	0	377	0	377
TCGA-QR	TCGA-QR	PCPG	30	MALE	WHITE		Paraganglioma		2012	-11051	Alive	TUMOR F	546							Complete Remission/Response	0	546	0	546	0	546	0	546
TCGA-QT	TCGA-QT	PCPG	42	FEMALE	BLACK OR AFRICAN AMERICAN		Pheochromocytoma		2011	-15535	Alive	WITH TUI	1102			Distant M	Other, sp	Bilateral E	77	Progressive Disease	0	1102	0	1102			1	77
TCGA-QT	TCGA-QT	PCPG	56	FEMALE	BLACK OR AFRICAN AMERICAN		Pheochromocytoma		2011	-20724	Alive	TUMOR F	211							Complete Remission/Response	0	211	0	211	0	211	0	211
TCGA-QT	TCGA-QT	PCPG	27	MALE	WHITE		Pheochromocytoma		2010	-10028	Alive	TUMOR F	55							Complete Remission/Response	0	55	0	55	0	55	0	55
TCGA-QT	TCGA-QT	PCPG	43	MALE	WHITE		Pheochromocytoma		2012	-15915	Alive	TUMOR F	22							Complete Remission/Response	0	22	0	22	0	22	0	22
TCGA-QT	TCGA-QT	PCPG	51	FEMALE	BLACK OR AFRICAN AMERICAN		Pheochromocytoma		2010	-18812	Alive	TUMOR F	766							Complete Remission/Response	0	766	0	766	0	766	0	766
TCGA-QT	TCGA-QT	PCPG	63	FEMALE	BLACK OR AFRICAN AMERICAN		Pheochromocytoma		2012	-23154	Alive	TUMOR F	70							Complete Remission/Response	0	70	0	70	0	70	0	70
TCGA-QT	TCGA-QT	PCPG	57	FEMALE	BLACK OR AFRICAN AMERICAN		Pheochromocytoma		2012	-21072	Alive	TUMOR F	134							Complete Remission/Response	0	134	0	134	0	134	0	134
TCGA-QT	TCGA-QT	PCPG	40	MALE	WHITE		Pheochromocytoma		2012	-14866	Alive	TUMOR F	24							Complete Remission/Response	0	24	0	24	0	24	0	24
TCGA-QT	TCGA-QT	PCPG	56	MALE	WHITE		Paraganglioma; Extra		2013	-20538	Alive	TUMOR F	398							Complete Remission/Response	0	398	0	398	0	398	0	398
TCGA-RM	TCGA-RM	PCPG	46	MALE	BLACK OR AFRICAN AMERICAN		Paraganglioma		2007	-17065	Alive	TUMOR F	2372							Complete Remission/Response	0	2372	0	2372	0	2372	0	2372
TCGA-RM	TCGA-RM	PCPG	41	FEMALE	WHITE		Paraganglioma; Extra		2010	-15273	Alive	TUMOR F	28							Complete Remission/Response	0	28	0	28	0	28	0	28
TCGA-RT	TCGA-RT	PCPG	50	FEMALE	WHITE		Pheochromocytoma		2005	-18594	Alive	TUMOR F	3293			New Prim	Other, sp	left thigh	3282	Complete Remission/Response	0	3293	0	3293	0	3293	1	3282
TCGA-RT	TCGA-RT	PCPG	71	FEMALE	WHITE		Pheochromocytoma		2009	-26095	Alive	TUMOR F	1540							Complete Remission/Response	0	1540	0	1540	0	1540	0	1540
TCGA-RT	TCGA-RT	PCPG	31	MALE	WHITE		Pheochromocytoma		2013	-11572	Alive	TUMOR F	246							Complete Remission/Response	0	246	0	246	0	246	0	246
TCGA-RW	TCGA-RW	PCPG	44	FEMALE	WHITE		Pheochromocytoma		2004	-16139	Alive	TUMOR F	2643			Locoregic	Other, sp	Left adrer	551	Progressive Disease	0	2643	0	2643			1	551
TCGA-RW	TCGA-RW	PCPG	46	FEMALE	WHITE		Pheochromocytoma		2010	-16983	Alive	TUMOR F	604							Complete Remission/Response	0	604	0	604	0	604	0	604
TCGA-RW	TCGA-RW	PCPG	47	MALE	WHITE		Pheochromocytoma		2003	-17307	Alive	TUMOR F	89							Complete Remission/Response	0	89	0	89	0	89	0	89
TCGA-RW	TCGA-RW	PCPG	43	FEMALE	WHITE		Pheochromocytoma		2011	-15729	Alive	TUMOR F	216							Complete Remission/Response	0	216	0	216	0	216	0	216
TCGA-RW	TCGA-RW	PCPG	57	FEMALE	WHITE		Paraganglioma; Extra		2011	-21010	Alive	TUMOR F	1449							Complete Remission/Response	0	1449	0	1449	0	1449	0	1449
TCGA-RW	TCGA-RW	PCPG	56	MALE	WHITE		Pheochromocytoma		2011	-20812	Alive	TUMOR F	1141							Complete Remission/Response	0	1141	0	1141	0	1141	0	1141
TCGA-RW	TCGA-RW	PCPG	64	FEMALE	WHITE		Pheochromocytoma		2011	-23440	Alive	TUMOR F	32							Complete Remission/Response	0	32	0	32	0	32	0	32
TCGA-RW	TCGA-RW	PCPG	70	FEMALE	WHITE		Pheochromocytoma		2012	-25790	Alive	TUMOR F	26							Complete Remission/Response	0	26	0	26	0	26	0	26
TCGA-RW	TCGA-RW	PCPG	62	MALE	WHITE		Pheochromocytoma		2012	-22718	Alive	TUMOR F	587							Complete Remission/Response	0	587	0	587	0	587	0	587
TCGA-RW	TCGA-RW	PCPG	62	MALE	WHITE		Pheochromocytoma		2012	-22718	Alive	TUMOR F	587							Complete Remission/Response	0	587	0	587	0	587	0	587
TCGA-RW	TCGA-RW	PCPG	69	FEMALE	WHITE		Pheochromocytoma		2013	-25216	Alive	TUMOR F	37			New Prim	Lung		27	Complete Remission/Response	0	37	0	37	0	37	1	27
TCGA-RW	TCGA-RW	PCPG	58	MALE	WHITE		Pheochromocytoma		2012	-21402	Alive	TUMOR F	828							Complete Remission/Response	0	828	0	828	0	828	0	828
TCGA-RW	TCGA-RW	PCPG	19	FEMALE	WHITE		Pheochromocytoma		2012	-7248	Alive	TUMOR F	762							Complete Remission/Response	0	762	0	762	0	762	0	762
TCGA-RW	TCGA-RW	PCPG	31	MALE	[Not Evaluated]		Pheochromocytoma		2013	-11557	Alive	TUMOR F	727							Complete Remission/Response	0	727	0	727	0	727	0	727
TCGA-RW	TCGA-RW	PCPG	43	MALE	WHITE		Pheochromocytoma		2013	-15962	Alive	TUMOR F	191							Complete Remission/Response	0	191	0	191	0	191	0	191
TCGA-RW	TCGA-RW	PCPG	31	FEMALE	ASIAN		Pheochromocytoma		2006	-11438	Alive	TUMOR F	2214							Complete Remission/Response	0	2214	0	2214	0	2214	0	2214
TCGA-RW	TCGA-RW	PCPG	51	FEMALE	WHITE		Pheochromocytoma		2010	-18974	Alive	TUMOR F	1354							Complete Remission/Response	0	1354	0	1354	0	1354	0	1354
TCGA-RW	TCGA-RW	PCPG	49	MALE	WHITE		Pheochromocytoma		2011	-17959	Alive	TUMOR F	1274							Complete Remission/Response	0	1274	0	1274	0	1274	0	1274
TCGA-RW	TCGA-RW	PCPG	72	FEMALE	WHITE		Paraganglioma; Extra		2013	-26657	Alive	TUMOR F	422							Complete Remission/Response	0	422	0	422	0	422	0	422
TCGA-RW	TCGA-RW	PCPG	47	FEMALE	WHITE		Pheochromocytoma		2013	-17422	Alive	TUMOR F	352							Complete Remission/Response	0	352	0	352	0	352	0	352
TCGA-RW	TCGA-RW	PCPG	66	MALE	WHITE		Pheochromocytoma		2013	-24268	Dead	TUMOR FREE		88						[Unknown]	1	88	0	88			0	88
TCGA-RX	TCGA-RX	PCPG	44	MALE	WHITE		Paraganglioma		2013	-16182	Alive	TUMOR F	611							Complete Remission/Response	0	611	0	611	0	611	0	611
TCGA-S7	TCGA-S7	PCPG	59	MALE	WHITE		Pheochromocytoma		2012	-21643	Alive	TUMOR F	961							Complete Remission/Response	0	961	0	961	0	961	0	961
TCGA-S7	TCGA-S7	PCPG	76	MALE	WHITE		Pheochromocytoma		2011	-27984	Alive	TUMOR F	1112							Complete Remission/Response	0	1112	0	1112	0	1112	0	1112
TCGA-S7	TCGA-S7	PCPG	40	MALE	WHITE		Pheochromocytoma		2011	-14611	Alive	TUMOR F	1133							Complete Remission/Response	0	1133	0	1133	0	1133	0	1133
TCGA-S7	TCGA-S7	PCPG	76	FEMALE	WHITE		Pheochromocytoma		2011	-28114	Alive	TUMOR F	1080							Complete Remission/Response	0	1080	0	1080	0	1080	0	1080
TCGA-S7	TCGA-S7	PCPG	25	FEMALE	WHITE		Pheochromocytoma		2012	-9241	Alive	TUMOR F	944							Complete Remission/Response	0	944	0	944	0	944	0	944
TCGA-S7	TCGA-S7	PCPG	19	MALE	WHITE		Pheochromocytoma		2012	-7269	Alive	TUMOR F	820							Complete Remission/Response	0	820	0	820	0	820	0	820
TCGA-S7	TCGA-S7	PCPG	32	FEMALE	WHITE		Pheochromocytoma		2011	-12011	Alive	TUMOR F	1238							Complete Remission/Response	0	1238	0	1238	0	1238	0	1238
TCGA-S7	TCGA-S7	PCPG	74	MALE	WHITE		Pheochromocytoma		2011	-27311	Alive	TUMOR F	1301							Complete Remission/Response	0	1301	0	1301	0	1301	0	1301
TCGA-S7	TCGA-S7	PCPG	63	FEMALE	WHITE		Pheochromocytoma		2012	-23187	Alive	TUMOR F	928							Complete Remission/Response	0	928	0	928	0	928	0	928
TCGA-S7	TCGA-S7	PCPG	35	FEMALE	WHITE		Pheochromocytoma		2012	-12893	Alive	TUMOR F	849							Complete Remission/Response	0	849	0	849	0	849	0	849
TCGA-S7	TCGA-S7	PCPG	34	MALE	WHITE		Pheochromocytoma		2013	-12729	Alive	TUMOR F	706							Complete Remission/Response	0	706	0	706	0	706	0	706
TCGA-S7	TCGA-S7	PCPG	52	MALE	WHITE		Pheochromocytoma		2012	-19190	Alive	TUMOR F	770							Complete Remission/Response	0	770	0	770	0	770	0	770
TCGA-S7	TCGA-S7	PCPG	75	FEMALE	WHITE		Paraganglioma; Extra		2013	-27729	Alive	TUMOR F	546							Complete Remission/Response	0	546	0	546	0	546	0	546
TCGA-S7	TCGA-S7	PCPG	23	MALE	WHITE		Pheochromocytoma		2013	-8426	Alive	TUMOR F	576							Complete Remission/Response	0	576	0	576	0	576	0	576
TCGA-S7	TCGA-S7	PCPG	57	FEMALE	WHITE		Pheochromocytoma		2012	-21054	Alive	TUMOR F	736							Complete Remission/Response	0	736	0	736	0	736	0	736
TCGA-SA	TCGA-SA	PCPG	46	FEMALE	BLACK OR AFRICAN AMERICAN		Paraganglioma; Extra		2012	-16805	Alive	TUMOR F	296							Complete Remission/Response	0	296	0	296	0	296	0	296
TCGA-SP	TCGA-SP	PCPG	46	MALE	WHITE		Pheochromocytoma		2004	-16891	Alive	TUMOR F	3491							Complete Remission/Response	0	3491	0	3491	0	3491	0	3491
TCGA-SP	TCGA-SP	PCPG	26	MALE	[Not Evaluated]		Pheochromocytoma		2004	-9829	Alive	TUMOR F	3323							Complete Remission/Response	0	3323	0	3323	0	3323	0	3323
TCGA-SP	TCGA-SP	PCPG	48	MALE	ASIAN		Pheochromocytoma		2007	-17795	Alive	TUMOR F	2513			New Prim	Other, sp	Thyroid	588	Complete Remission/Response	0	2513	0	2513	0	2513	1	588
TCGA-SP	TCGA-SP	PCPG	35	FEMALE	WHITE		Paraganglioma		2008	-12874	Alive	TUMOR F	2417			New Prim	Other, sp	Breast, lef	626	Complete Remission/Response	0	2417	0	2417	0	2417	1	626
TCGA-SP	TCGA-SP	PCPG	35	FEMALE	ASIAN		Pheochromocytoma		2008	-12876	Alive	TUMOR F	2203							Complete Remission/Response	0	2203	0	2203	0	2203	0	2203
TCGA-SP	TCGA-SP	PCPG	47	MALE	WHITE		Pheochromocytoma		2005	-17167	Alive	TUMOR F	3347							Complete Remission/Response	0	3347	0	3347	0	3347	0	3347
TCGA-SP	TCGA-SP	PCPG	64	FEMALE	WHITE		Pheochromocytoma		2012	-23406	Alive	TUMOR F	472							Complete Remission/Response	0	472	0	472	0	472	0	472
TCGA-SP	TCGA-SP	PCPG	46	FEMALE	ASIAN		Pheochromocytoma		2005	-16923	Alive	TUMOR F	3384							Complete Remission/Response	0	3384	0	3384	0	3384	0	3384
TCGA-SQ	TCGA-SQ	PCPG	53	MALE	WHITE		Pheochromocytoma		2012	-19399	Alive	TUMOR F	43							[Unknown]	0	43	0	43			0	43
TCGA-SQ	TCGA-SQ	PCPG	53	MALE	WHITE		Pheochromocytoma		2012	-19399	Alive	TUMOR F	43							[Unknown]	0	43	0	43			0	43
TCGA-SQ	TCGA-SQ	PCPG	39	MALE	WHITE		Pheochromocytoma		2012	-14451	Alive	TUMOR F	3							[Unknown]	0	3	0	3			0	3
TCGA-SR	TCGA-SR	PCPG	30	FEMALE	WHITE		Pheochromocytoma		2009	-11050	Alive	TUMOR F	1558							Complete Remission/Response	0	1558	0	1558	0	1558	0	1558
TCGA-SR	TCGA-SR	PCPG	61	FEMALE	ASIAN		Paraganglioma		2008	-22621	Alive	TUMOR F	2087							Complete Remission/Response	0	2087	0	2087	0	2087	0	2087
TCGA-SR	TCGA-SR	PCPG	41	MALE	WHITE		Paraganglioma		2007	-14991	Alive	WITH TUI	2409			New Prim	Retroperitoneum		928	Stable Disease	0	2409	0	2409			1	928
TCGA-SR	TCGA-SR	PCPG	35	FEMALE	WHITE		Pheochromocytoma		2007	-12979	Alive	TUMOR F	2492							Complete Remission/Response	0	2492	0	2492	0	2492	0	2492
TCGA-SR	TCGA-SR	PCPG	57	FEMALE	WHITE		Pheochromocytoma		2004	-20944	Alive	TUMOR F	3247							Complete Remission/Response	0	3247	0	3247	0	3247	0	3247
TCGA-SR	TCGA-SR	PCPG	49	FEMALE	WHITE		Pheochromocytoma		2004	-18073	Alive	TUMOR F	3362							Complete Remission/Response	0	3362	0	3362	0	3362	0	3362
TCGA-SR	TCGA-SR	PCPG	35	FEMALE	WHITE		Paraganglioma		2004	-13074	Alive	WITH TUI	3682							Partial Remission/Response	0	3682	0	3682			0	3682
TCGA-SR	TCGA-SR	PCPG	55	MALE	WHITE		Paraganglioma		2013	-20186	Dead	WITH TUMOR		95						Progressive Disease	1	95	1	95			1	95
TCGA-SR	TCGA-SR	PCPG	55	MALE	WHITE		Paraganglioma		2013	-20186	Dead	WITH TUMOR		95						Progressive Disease	1	95	1	95			1	95
TCGA-SR	TCGA-SR	PCPG	55	MALE	WHITE		Paraganglioma		2013	-20186	Dead	WITH TUMOR		95						Progressive Disease	1	95	1	95			1	95
TCGA-SR	TCGA-SR	PCPG	54	FEMALE	WHITE		Pheochromocytoma		2012	-19892	Alive	TUMOR F	750							Complete Remission/Response	0	750	0	750	0	750	0	750
TCGA-SR	TCGA-SR	PCPG	46	FEMALE	WHITE		Pheochromocytoma		2012	-16802	Alive	TUMOR F	797							Complete Remission/Response	0	797	0	797	0	797	0	797
TCGA-SR	TCGA-SR	PCPG	45	FEMALE	WHITE		Pheochromocytoma		2010	-16489	Alive	TUMOR F	1509							Complete Remission/Response	0	1509	0	1509	0	1509	0	1509
TCGA-TT	TCGA-TT	PCPG	45	FEMALE	[Unknown]		Pheochromocytoma		2011	-16475	Alive	TUMOR F	132							Complete Remission/Response	0	132	0	132	0	132	0	132
TCGA-TT	TCGA-TT	PCPG	61	MALE	WHITE		Paraganglioma; Extra		2011	-22500	Alive	TUMOR F	1136							Complete Remission/Response	0	1136	0	1136	0	1136	0	1136
TCGA-TT	TCGA-TT	PCPG	37	FEMALE	WHITE		Pheochromocytoma		2012	-13605	Alive	TUMOR F	606							Complete Remission/Response	0	606	0	606	0	606	0	606
TCGA-TT	TCGA-TT	PCPG	64	FEMALE	BLACK OR AFRICAN AMERICAN		Pheochromocytoma		2013	-23554	Alive	TUMOR F	405			Biochemical evidence of disease			377	Complete Remission/Response	0	405	0	405	1	377	1	377
TCGA-TT	TCGA-TT	PCPG	19	FEMALE	WHITE		Pheochromocytoma		2011	-7240	Alive	TUMOR F	1292							Complete Remission/Response	0	1292	0	1292	0	1292	0	1292
TCGA-W2	TCGA-W2	PCPG	25	FEMALE	WHITE		Pheochromocytoma		2013	-9363	Alive	TUMOR F	611							Complete Remission/Response	0	611	0	611	0	611	0	611
TCGA-W2	TCGA-W2	PCPG	59	MALE	WHITE		Pheochromocytoma		2012	-21711	Alive	TUMOR F	522							Complete Remission/Response	0	522	0	522	0	522	0	522
TCGA-W2	TCGA-W2	PCPG	63	FEMALE	AMERICAN INDIAN OR ALASKA		Pheochromocytoma		2011	-23059	Alive	TUMOR F	36							Complete Remission/Response	0	36	0	36	0	36	0	36
TCGA-W2	TCGA-W2	PCPG	24	MALE	WHITE		Pheochromocytoma		2012	-8979	Alive	TUMOR F	604							Complete Remission/Response	0	604	0	604	0	604	0	604
TCGA-W2	TCGA-W2	PCPG	67	MALE	WHITE		Pheochromocytoma		2013	-24834	Alive	TUMOR F	439							Complete Remission/Response	0	439	0	439	0	439	0	439
TCGA-W2	TCGA-W2	PCPG	76	FEMALE	WHITE		Pheochromocytoma		2013	-27962	Alive	TUMOR F	561							Complete Remission/Response	0	561	0	561	0	561	0	561
TCGA-W2	TCGA-W2	PCPG	80	MALE	WHITE		Pheochromocytoma		2013	-29241	Alive	TUMOR F	505							Complete Remission/Response	0	505	0	505	0	505	0	505
TCGA-W2	TCGA-W2	PCPG	28	FEMALE	BLACK OR AFRICAN AMERICAN		Pheochromocytoma		2013	-10436	Alive	TUMOR F	538							Complete Remission/Response	0	538	0	538	0	538	0	538
TCGA-W2	TCGA-W2	PCPG	69	FEMALE	WHITE		Paraganglioma		2012	-25291	Alive	TUMOR F	779							Complete Remission/Response	0	779	0	779	0	779	0	779
TCGA-W2	TCGA-W2	PCPG	44	FEMALE	WHITE		Pheochromocytoma		2013	-16335	Alive	TUMOR F	450							[Unknown]	0	450	0	450			0	450
TCGA-WB	TCGA-WB	PCPG	49	MALE	WHITE		Pheochromocytoma		2011	-18033	Alive	TUMOR F	1255			Distant M	Lymph Node(s)		772	Complete Remission/Response	0	1255	0	1255	1	772	1	772
TCGA-WB	TCGA-WB	PCPG	30	FEMALE	WHITE		Pheochromocytoma		2012	-10980	Alive	TUMOR F	1042							Complete Remission/Response	0	1042	0	1042	0	1042	0	1042
TCGA-WB	TCGA-WB	PCPG	63	MALE	WHITE		Pheochromocytoma		2012	-23163	Alive	TUMOR F	136							Complete Remission/Response	0	136	0	136	0	136	0	136
TCGA-WB	TCGA-WB	PCPG	54	MALE	WHITE		Pheochromocytoma		2012	-19980	Alive	TUMOR F	144							Complete Remission/Response	0	144	0	144	0	144	0	144
TCGA-WB	TCGA-WB	PCPG	83	FEMALE	WHITE		Pheochromocytoma		2013	-30555	Alive	TUMOR F	776							Complete Remission/Response	0	776	0	776	0	776	0	776
TCGA-WB	TCGA-WB	PCPG	20	MALE	WHITE		Paraganglioma; Extra		1988	-7608	Alive	WITH TUI	9634			Distant M	Bone		6866	Progressive Disease	0	9634	0	9634			1	6866
TCGA-WB	TCGA-WB	PCPG	64	FEMALE	WHITE		Pheochromocytoma		2003	-23653	Alive	TUMOR F	3880							Complete Remission/Response	0	3880	0	3880		3880	0	3880
TCGA-WB	TCGA-WB	PCPG	63	FEMALE	WHITE		Pheochromocytoma		2011	-23254	Alive	TUMOR F	887							Complete Remission/Response	0	887	0	887	0	887	0	887
TCGA-WB	TCGA-WB	PCPG	59	FEMALE	WHITE		Pheochromocytoma		2011	-21902	Dead	TUMOR FREE		596						Complete Remission/Response	1	596	0	596	0	596	0	596

TCGA-WE	TCGA-WE	PCPG	35	FEMALE	BLACK OR AFRICAN AMERICAN	Paraganglioma; Extra	2008	-12936	Alive	TUMOR F	2252							Complete Remission/Response	0	2252	0	2252	0	2252	0	2252
TCGA-WE	TCGA-WE	PCPG	68	FEMALE	WHITE	Pheochromocytoma	2010	-24859	Alive	TUMOR F	1630							Complete Remission/Response	0	1630	0	1630	0	1630	0	1630
TCGA-WE	TCGA-WE	PCPG	42	FEMALE	WHITE	Pheochromocytoma	2010	-15400	Alive	TUMOR F	1254							Complete Remission/Response	0	1254	0	1254	0	1254	0	1254
TCGA-WE	TCGA-WE	PCPG	27	MALE	WHITE	Pheochromocytoma	2011	-9981	Alive	TUMOR F	1310							Complete Remission/Response	0	1310	0	1310	0	1310	0	1310
TCGA-WE	TCGA-WE	PCPG	37	MALE	WHITE	Pheochromocytoma	2011	-13596	Alive	TUMOR F	1288							Complete Remission/Response	0	1288	0	1288	0	1288	0	1288
TCGA-WE	TCGA-WE	PCPG	36	MALE	WHITE	Pheochromocytoma	2011	-13233	Alive	TUMOR F	1031							Complete Remission/Response	0	1031	0	1031	0	1031	0	1031
TCGA-WE	TCGA-WE	PCPG	65	FEMALE	WHITE	Pheochromocytoma	2012	-23966	Alive	TUMOR F	735							Complete Remission/Response	0	735	0	735	0	735	0	735
TCGA-WE	TCGA-WE	PCPG	82	FEMALE	WHITE	Pheochromocytoma	2011	-30101	Alive	TUMOR F	1090							Complete Remission/Response	0	1090	0	1090	0	1090	0	1090
TCGA-WE	TCGA-WE	PCPG	47	FEMALE	WHITE	Paraganglioma; Extra	2011	-17407	Alive	WITH TUM	1476			Locoregic	Lymph Node(s)		887	Complete Remission/Response	0	1476	0	1476	1	887	1	887
TCGA-WE	TCGA-WE	PCPG	40	MALE	WHITE	Pheochromocytoma	2009	-14933	Alive	TUMOR F	1737							Complete Remission/Response	0	1737	0	1737	0	1737	0	1737
TCGA-WE	TCGA-WE	PCPG	25	MALE	WHITE	Pheochromocytoma	2011	-9248	Alive	TUMOR F	1179							Complete Remission/Response	0	1179	0	1179	0	1179	0	1179
TCGA-WE	TCGA-WE	PCPG	44	FEMALE	WHITE	Pheochromocytoma	2009	-16425	Alive	TUMOR F	1946							Complete Remission/Response	0	1946	0	1946	0	1946	0	1946
TCGA-WE	TCGA-WE	PCPG	64	MALE	WHITE	Pheochromocytoma	2012	-23566	Alive	TUMOR F	679							Complete Remission/Response	0	679	0	679	0	679	0	679
TCGA-WE	TCGA-WE	PCPG	40	MALE	WHITE	Pheochromocytoma	2012	-14665	Alive	TUMOR F	741							Complete Remission/Response	0	741	0	741	0	741	0	741
TCGA-WE	TCGA-WE	PCPG	45	MALE	WHITE	Pheochromocytoma	2010	-16483	Alive	TUMOR F	1218							Complete Remission/Response	0	1218	0	1218	0	1218	0	1218
TCGA-WE	TCGA-WE	PCPG	42	FEMALE	WHITE	Pheochromocytoma	2010	-15505	Alive	TUMOR F	1394							Complete Remission/Response	0	1394	0	1394	0	1394	0	1394
TCGA-WE	TCGA-WE	PCPG	28	FEMALE	WHITE	Pheochromocytoma	2010	-10283	Alive	TUMOR F	1887							Complete Remission/Response	0	1887	0	1887	0	1887	0	1887
TCGA-WE	TCGA-WE	PCPG	73	FEMALE	WHITE	Pheochromocytoma	2011	-26867	Alive	TUMOR F	395							Complete Remission/Response	0	395	0	395	0	395	0	395
TCGA-WE	TCGA-WE	PCPG	44	MALE	WHITE	Pheochromocytoma	1997	-16422	Dead	WITH TUMOR		755						Progressive Disease	1	755	1	755			1	755
TCGA-WE	TCGA-WE	PCPG	55	FEMALE	WHITE	Pheochromocytoma	2012	-20438	Alive	TUMOR F	49							Complete Remission/Response	0	49	0	49	0	49	0	49
TCGA-WE	TCGA-WE	PCPG	59	FEMALE	WHITE	Pheochromocytoma	2012	-21587	Alive	TUMOR F	723							Complete Remission/Response	0	723	0	723	0	723	0	723
TCGA-WE	TCGA-WE	PCPG	45	MALE	WHITE	Pheochromocytoma	2013	-16506	Alive	TUMOR F	776							Complete Remission/Response	0	776	0	776	0	776	0	776
TCGA-WE	TCGA-WE	PCPG	57	MALE	WHITE	Pheochromocytoma	2006	-21106	Alive	TUMOR F	2161							Complete Remission/Response	0	2161	0	2161	0	2161	0	2161
TCGA-WE	TCGA-WE	PCPG	41	MALE	WHITE	Pheochromocytoma	2007	-15001	Alive	TUMOR F	2379							Complete Remission/Response	0	2379	0	2379	0	2379	0	2379
TCGA-WE	TCGA-WE	PCPG	40	MALE	WHITE	Pheochromocytoma	2004	-14949	Dead	WITH TUMOR		309						Progressive Disease	1	309	1	309			1	309
TCGA-WE	TCGA-WE	PCPG	73	FEMALE	WHITE	Pheochromocytoma	2006	-26805	Alive	TUMOR F	2832							Complete Remission/Response	0	2832	0	2832	0	2832	0	2832
TCGA-WE	TCGA-WE	PCPG	23	MALE	WHITE	Pheochromocytoma	2006	-8708	Alive	WITH TUM	3138			Distant M	Bone		69	Progressive Disease	0	3138	0	3138			1	69
TCGA-XG	TCGA-XG	PCPG	25	FEMALE	WHITE	Pheochromocytoma	2013	-9149	Alive	TUMOR F	614			New Prim	Other, sp	Carotid B	190	Complete Remission/Response	0	614	0	614	0	614	1	190
TCGA-2A	TCGA-2A	PRAD	51	MALE		Prostate Adenocarci	2010	-18658	Alive	TUMOR F	621								0	621	0	621			0	621
TCGA-2A	TCGA-2A	PRAD	57	MALE		Prostate Adenocarci	2010	-20958	Alive	TUMOR F	1701								0	1701	0	1701			0	1701
TCGA-2A	TCGA-2A	PRAD	47	MALE		Prostate Adenocarci	2011	-17365	Alive	[Discrepar	1373							[Discrepancy]	0	1373	0	1373			0	1373
TCGA-2A	TCGA-2A	PRAD	52	MALE		Prostate Adenocarci	2010	-19065	Alive	TUMOR F	671								0	671	0	671			0	671
TCGA-2A	TCGA-2A	PRAD	70	MALE		Prostate Adenocarci	2011	-25904	Alive	TUMOR F	1378								0	1378	0	1378			0	1378
TCGA-2A	TCGA-2A	PRAD	54	MALE		Prostate Adenocarci	2011	-19964	Alive		112								0	112	0	112			0	112
TCGA-2A	TCGA-2A	PRAD	69	MALE		Prostate Adenocarci	2011	-25557	Alive	[Discrepar	863			Biochemical evidence of disease			198		0	863	0	863			1	198
TCGA-2A	TCGA-2A	PRAD	57	MALE		Prostate Adenocarci	2011	-20866	Alive	TUMOR F	1364							Complete Remission/Response	0	1364	0	1364	0	1364	0	1364
TCGA-2A	TCGA-2A	PRAD	57	MALE		Prostate Adenocarci	2011	-21183	Alive	TUMOR F	1272							Complete Remission/Response	0	1272	0	1272	0	1272	0	1272
TCGA-2A	TCGA-2A	PRAD	56	MALE		Prostate Adenocarci	2012	-20582	Alive	TUMOR F	615							[Discrepancy]	0	615	0	615			0	615
TCGA-4L-	TCGA-4L-	PRAD	64	MALE		Prostate Adenocarci	2013	-23665	Alive	TUMOR F	350							Complete Remission/Response	0	350	0	350	0	350	0	350
TCGA-CH	TCGA-CH	PRAD	73	MALE	WHITE	Prostate Adenocarci	2007	-26753	Alive	TUMOR F	91								0	91	0	91			0	91
TCGA-CH	TCGA-CH	PRAD	72	MALE	WHITE	Prostate Adenocarci	2006	-26329	Alive	TUMOR F	212							Complete Remission/Response	0	212	0	212	0	212	0	212
TCGA-CH	TCGA-CH	PRAD	65	MALE	WHITE	Prostate Adenocarci	2006	-23953	Alive	TUMOR F	671							Complete Remission/Response	0	671	0	671	0	671	0	671
TCGA-CH	TCGA-CH	PRAD	57	MALE	WHITE	Prostate Adenocarci	2009	-20880	Alive	TUMOR F	31								0	31	0	31			0	31
TCGA-CH	TCGA-CH	PRAD	56	MALE	WHITE	Prostate Adenocarci	2008	-20759	Alive	TUMOR F	395							Complete Remission/Response	0	395	0	395	0	395	0	395
TCGA-CH	TCGA-CH	PRAD	66	MALE	WHITE	Prostate Adenocarci	2008	-24321	Alive	TUMOR F	425			New Primary Tumor			425	Complete Remission/Response	0	425	0	425	0	425	1	425
TCGA-CH	TCGA-CH	PRAD	64	MALE	WHITE	Prostate Adenocarci	2008	-23621	Alive	TUMOR F	60								0	60	0	60			0	60
TCGA-CH	TCGA-CH	PRAD	68	MALE	WHITE	Prostate Adenocarci	2008	-24837	Alive	TUMOR F	91								0	91	0	91			0	91
TCGA-CH	TCGA-CH	PRAD	57	MALE	WHITE	Prostate Adenocarci	2007	-20941	Alive	TUMOR F	731							Complete Remission/Response	0	731	0	731	0	731	0	731
TCGA-CH	TCGA-CH	PRAD	64	MALE	WHITE	Prostate Adenocarci	2009	-23649	Alive	WITH TUM	31								0	31	0	31			0	31
TCGA-CH	TCGA-CH	PRAD	72	MALE	WHITE	Prostate Adenocarci	2008	-26389	Alive	TUMOR F	396							Complete Remission/Response	0	396	0	396	0	396	0	396
TCGA-CH	TCGA-CH	PRAD	68	MALE	WHITE	Prostate Adenocarci	2006	-24957	Alive	WITH TUM	1065			Distant M	Bone		365	Complete Remission/Response	0	1065	0	1065	1	365	1	365
TCGA-CH	TCGA-CH	PRAD	66	MALE	WHITE	Prostate Adenocarci	2006	-24290	Alive	TUMOR F	943							Complete Remission/Response	0	943	0	943	0	943	0	943
TCGA-CH	TCGA-CH	PRAD	70	MALE	WHITE	Prostate Adenocarci	2008	-25780	Alive	TUMOR F	31								0	31	0	31			0	31
TCGA-CH	TCGA-CH	PRAD	65	MALE	WHITE	Prostate Adenocarci	2008	-23803	Alive	WITH TUM	62								0	62	0	62			0	62
TCGA-CH	TCGA-CH	PRAD	61	MALE	WHITE	Prostate Adenocarci	2007	-22433	Alive	TUMOR F	28								0	28	0	28			0	28
TCGA-CH	TCGA-CH	PRAD	61	MALE	WHITE	Prostate Adenocarci	2007	-22433	Alive	TUMOR F	28								0	28	0	28			0	28
TCGA-CH	TCGA-CH	PRAD	60	MALE	WHITE	Prostate Adenocarci	2005	-22068	Alive	[Discrepar	1339							Complete Remission/Response	0	1339	0	1339	0	1339	0	1339
TCGA-CH	TCGA-CH	PRAD	60	MALE	WHITE	Prostate Adenocarci	2005	-22068	Alive	[Discrepar	1339							Complete Remission/Response	0	1339	0	1339	0	1339	0	1339
TCGA-CH	TCGA-CH	PRAD	66	MALE	WHITE	Prostate Adenocarci	2008	-24380	Alive	TUMOR F	365							Complete Remission/Response	0	365	0	365	0	365	0	365
TCGA-CH	TCGA-CH	PRAD	66	MALE	WHITE	Prostate Adenocarci	2008	-24380	Alive	TUMOR F	365							Complete Remission/Response	0	365	0	365	0	365	0	365
TCGA-CH	TCGA-CH	PRAD	66	MALE	WHITE	Prostate Adenocarci	2008	-24257	Alive	TUMOR F	31								0	31	0	31			0	31
TCGA-CH	TCGA-CH	PRAD	66	MALE	WHITE	Prostate Adenocarci	2008	-24257	Alive	TUMOR F	31								0	31	0	31			0	31
TCGA-CH	TCGA-CH	PRAD	55	MALE	WHITE	Prostate Adenocarci	2007	-20364	Alive	TUMOR F	700							Complete Remission/Response	0	700	0	700	0	700	0	700
TCGA-CH	TCGA-CH	PRAD	55	MALE	WHITE	Prostate Adenocarci	2007	-20364	Alive	TUMOR F	700							Complete Remission/Response	0	700	0	700	0	700	0	700
TCGA-CH	TCGA-CH	PRAD	55	MALE	WHITE	Prostate Adenocarci	2007	-20392	Alive	TUMOR F	31								0	31	0	31			0	31
TCGA-CH	TCGA-CH	PRAD	55	MALE	WHITE	Prostate Adenocarci	2007	-20392	Alive	TUMOR F	31								0	31	0	31			0	31
TCGA-CH	TCGA-CH	PRAD	66	MALE	WHITE	Prostate Adenocarci	2007	-24287	Alive	TUMOR F	458							Complete Remission/Response	0	458	0	458	0	458	0	458
TCGA-CH	TCGA-CH	PRAD	66	MALE	WHITE	Prostate Adenocarci	2007	-24287	Alive	TUMOR F	458							Complete Remission/Response	0	458	0	458	0	458	0	458
TCGA-CH	TCGA-CH	PRAD	72	MALE	WHITE	Prostate Adenocarci	2007	-26480	Alive	TUMOR F	731							Complete Remission/Response	0	731	0	731	0	731	0	731
TCGA-CH	TCGA-CH	PRAD	72	MALE	WHITE	Prostate Adenocarci	2007	-26480	Alive	TUMOR F	731							Complete Remission/Response	0	731	0	731	0	731	0	731
TCGA-CH	TCGA-CH	PRAD	48	MALE	WHITE	Prostate Adenocarci	2007	-17807	Alive	WITH TUM	62								0	62	0	62			0	62
TCGA-CH	TCGA-CH	PRAD	48	MALE	WHITE	Prostate Adenocarci	2007	-17807	Alive	WITH TUM	62								0	62	0	62			0	62
TCGA-CH	TCGA-CH	PRAD	63	MALE	WHITE	Prostate Adenocarci	2008	-23101	Alive	TUMOR F	396							Complete Remission/Response	0	396	0	396	0	396	0	396
TCGA-CH	TCGA-CH	PRAD	63	MALE	WHITE	Prostate Adenocarci	2008	-23101	Alive	TUMOR F	396							Complete Remission/Response	0	396	0	396	0	396	0	396
TCGA-CH	TCGA-CH	PRAD	63	MALE	WHITE	Prostate Adenocarci	2008	-23194	Alive	TUMOR F	486							Complete Remission/Response	0	486	0	486	0	486	0	486
TCGA-CH	TCGA-CH	PRAD	63	MALE	WHITE	Prostate Adenocarci	2008	-23194	Alive	TUMOR F	486							Complete Remission/Response	0	486	0	486	0	486	0	486
TCGA-CH	TCGA-CH	PRAD	69	MALE	WHITE	Prostate Adenocarci	2007	-25233	Alive	WITH TUM	821							Progressive Disease	0	821	0	821			0	821
TCGA-CH	TCGA-CH	PRAD	61	MALE	WHITE	Prostate Adenocarci	2006	-22494	Alive	TUMOR F	304							Complete Remission/Response	0	304	0	304	0	304	0	304
TCGA-CH	TCGA-CH	PRAD	64	MALE	WHITE	Prostate Adenocarci	2006	-23529	Alive	TUMOR F	974							Complete Remission/Response	0	974	0	974	0	974	0	974
TCGA-CH	TCGA-CH	PRAD	72	MALE	WHITE	Prostate Adenocarci	2006	-26390	Alive		1004			New Primary Tumor			396	Complete Remission/Response	0	1004	0	1004	0	1004	1	396
TCGA-CH	TCGA-CH	PRAD	57	MALE	WHITE	Prostate Adenocarci	2009	-21002	Alive	WITH TUM	91								0	91	0	91			0	91
TCGA-CH	TCGA-CH	PRAD	65	MALE	WHITE	Prostate Adenocarci	2006	-23924	Alive	TUMOR F	882							Complete Remission/Response	0	882	0	882	0	882	0	882
TCGA-EJ-	TCGA-EJ-	PRAD	50	MALE	WHITE	Prostate Adenocarci	2009	-18596	Alive	TUMOR F	1477							Complete Remission/Response	0	1477	0	1477	0	1477	0	1477
TCGA-EJ-	TCGA-EJ-	PRAD	68	MALE	WHITE	Prostate Adenocarci	2009	-24904	Alive	TUMOR F	1760							Complete Remission/Response	0	1760	0	1760	0	1760	0	1760
TCGA-EJ-	TCGA-EJ-	PRAD	59	MALE	WHITE	Prostate Adenocarci	2009	-21897	Alive	TUMOR F	595							Complete Remission/Response	0	595	0	595	0	595	0	595
TCGA-EJ-	TCGA-EJ-	PRAD	47	MALE	WHITE	Prostate Adenocarci	2009	-17523	Alive	TUMOR F	405							Complete Remission/Response	0	405	0	405	0	405	0	405
TCGA-EJ-	TCGA-EJ-	PRAD	56	MALE	WHITE	Prostate Adenocarci	2010	-20472	Alive	TUMOR F	1720							Complete Remission/Response	0	1720	0	1720	0	1720	0	1720
TCGA-EJ-	TCGA-EJ-	PRAD	61	MALE	ASIAN	Prostate Adenocarci	2010	-22286	Alive	TUMOR F	1415							Complete Remission/Response	0	1415	0	1415	0	1415	0	1415
TCGA-EJ-	TCGA-EJ-	PRAD	55	MALE	WHITE	Prostate Adenocarci	2010	-20409	Alive	TUMOR F	1365							Complete Remission/Response	0	1365	0	1365	0	1365	0	1365
TCGA-EJ-	TCGA-EJ-	PRAD	50	MALE		Prostate Adenocarci	2010	-18447	Alive	TUMOR F	1171							Complete Remission/Response	0	1171	0	1171	0	1171	0	1171
TCGA-EJ-	TCGA-EJ-	PRAD	50	MALE	WHITE	Prostate Adenocarci	2010	-18395	Alive	TUMOR F	1391							Complete Remission/Response	0	1391	0	1391	0	1391	0	1391
TCGA-EJ-	TCGA-EJ-	PRAD	65	MALE	WHITE	Prostate Adenocarci	2010	-24072	Alive	WITH TUM	1427							Progressive Disease	0	1427	0	1427			0	1427
TCGA-EJ-	TCGA-EJ-	PRAD	57	MALE	WHITE	Prostate Adenocarci	2010	-21164	Alive	TUMOR F	922							Complete Remission/Response	0	922	0	922	0	922	0	922
TCGA-EJ-	TCGA-EJ-	PRAD	67	MALE	WHITE	Prostate Adenocarci	2010	-24617	Alive	TUMOR F	1381							Complete Remission/Response	0	1381	0	1381	0	1381	0	1381
TCGA-EJ-	TCGA-EJ-	PRAD	54	MALE	WHITE	Prostate Adenocarci	2010	-19759	Alive	TUMOR F	1457							Complete Remission/Response	0	1457	0	1457	0	1457	0	1457
TCGA-EJ-	TCGA-EJ-	PRAD	65	MALE	WHITE	Prostate Adenocarci	2008	-23758	Alive	TUMOR F	1965							Complete Remission/Response	0	1965	0	1965	0	1965	0	1965
TCGA-EJ-	TCGA-EJ-	PRAD	63	MALE	WHITE	Prostate Adenocarci	2009	-23368	Alive	TUMOR F	1464							Complete Remission/Response	0	1464	0	1464	0	1464	0	1464
TCGA-EJ-	TCGA-EJ-	PRAD	48	MALE	WHITE	Prostate Adenocarci	2009	-17895	Alive	TUMOR F	1887							Complete Remission/Response	0	1887	0	1887	0	1887	0	1887
TCGA-EJ-	TCGA-EJ-	PRAD	55	MALE	BLACK OR AFRICAN AMERICAN	Prostate Adenocarci	2009	-20409	Alive	TUMOR F	1476							Complete Remission/Response	0	1476	0	1476	0	1476	0	1476
TCGA-EJ-	TCGA-EJ-	PRAD	46	MALE	WHITE	Prostate Adenocarci	2009	-17127	Alive	TUMOR F	1733							Complete Remission/Response	0	1733	0	1733	0	1733	0	1733
TCGA-EJ-	TCGA-EJ-	PRAD	66	MALE	WHITE	Prostate Adenocarci	2009	-24165	Alive	TUMOR F	1829							Complete Remission/Response	0	1829	0	1829	0	1829	0	1829
TCGA-EJ-	TCGA-EJ-	PRAD	60	MALE	WHITE	Prostate Adenocarci	2009	-22097	Alive	TUMOR F	1832							Complete Remission/Response	0	1832	0	1832	0	1832	0	1832
TCGA-EJ-	TCGA-EJ-	PRAD	49	MALE	WHITE	Prostate Adenocarci	2009	-17980	Alive	TUMOR F	1889							Complete Remission/Response	0	1889	0	1889	0	1889	0	1889
TCGA-EJ-	TCGA-EJ-	PRAD	55	MALE	WHITE	Prostate Adenocarci	2008	-20422	Alive	TUMOR F	1889							Complete Remission/Response	0	1889	0	1889	0	1889	0	1889
TCGA-EJ-	TCGA-EJ-	PRAD	66	MALE	WHITE	Prostate Adenocarci	2008	-24313	Alive	WITH TUM	2118			Distant M	Bone		2104	Progressive Disease	0	2118	0	2118			1	2104
TCGA-EJ-	TCGA-EJ-	PRAD	64	MALE	WHITE	Prostate Adenocarci	2008	-23669	Alive	TUMOR F	1962							Complete Remission/Response	0	1962	0	1962	0	1962	0	1962
TCGA-EJ-	TCGA-EJ-	PRAD	63	MALE	WHITE	Prostate Adenocarci	2008	-23304	Alive	TUMOR F	2279							Complete Remission/Response	0	2279	0	2279	0	2279	0	2279
TCGA-EJ-	TCGA-EJ-	PRAD	51	MALE	WHITE	Prostate Adenocarci	2008	-18802	Alive	TUMOR F	2079							Complete Remission/Response	0	2079	0	2079	0	2079	0	2079
TCGA-EJ-	TCGA-EJ-	PRAD	57	MALE	WHITE	Prostate Adenocarci	2009	-20854	Alive	WITH TUM	1882			Locoregional Recurrence			265	Progressive Disease	0	1882	0	1882			1	265
TCGA-EJ-	TCGA-EJ-	PRAD	67	MALE	WHITE	Prostate Adenocarci	2009	-24535	Alive	WITH TUM	1115			Distant M	Bone		546	Progressive Disease	0	1115	0	1115			1	546
TCGA-EJ-	TCGA-EJ-	PRAD	56	MALE	WHITE	Prostate Adenocarci	2009	-20558	Alive	WITH TUM	1909			Locoregional Recurrence			537	Progressive Disease	0	1909	0	1909			1	537
TCGA-EJ-	TCGA-EJ-	PRAD	69	MALE	WHITE	Prostate Adenocarci	2009	-25234	Alive	TUMOR F	1778							Complete Remission/Response	0	1778	0	1778	0	1778	0	1778
TCGA-EJ-	TCGA-EJ-	PRAD	61	MALE	WHITE	Prostate Adenocarci	2008	-22316	Alive	TUMOR F	1832							Complete Remission/Response	0	1832	0	1832	0	1832	0	1832
TCGA-EJ-	TCGA-EJ-	PRAD	62	MALE	WHITE	Prostate Adenocarci	2009	-22760	Alive	TUMOR F	1273							Complete Remission/Response	0	1273	0	1273	0	1273	0	1273
TCGA-EJ-	TCGA-EJ-	PRAD	57	MALE	WHITE	Prostate Adenocarci	2009	-21110	Alive	TUMOR F	1828							Complete Remission/Response	0	1828	0	1828	0	1828	0	1828
TCGA-EJ-	TCGA-EJ-	PRAD	60	MALE	WHITE	Prostate Adenocarci	2010	-21942	Alive	TUMOR F	1513							Complete Remission/Response	0	1513	0	1513	0	1513	0	1513
TCGA-EJ-	TCGA-EJ-	PRAD	65	MALE	WHITE	Prostate Adenocarci	2006	-23986	Alive	TUMOR F	2687							Complete Remission/Response	0	2687	0	2687	0	2687	0	2687
TCGA-EJ-	TCGA-EJ-	PRAD	65	MALE	WHITE	Prostate Adenocarci	2006	-23986	Alive	TUMOR F	2687							Complete Remission/Response	0	2687	0	2687	0	2687	0	2687
TCGA-EJ-	TCGA-EJ-	PRAD	59	MALE	WHITE	Prostate Adenocarci	2007	-21741	Alive	TUMOR F	2572							Complete Remission/Response	0	2572	0	2572	0	2572	0	2572
TCGA-EJ-	TCGA-EJ-	PRAD	59	MALE	WHITE	Prostate Adenocarci	2007	-21741	Alive	TUMOR F	2572							Complete Remission/Response	0	2572	0	2572	0	2572	0	2572
TCGA-EJ-	TCGA-EJ-	PRAD	44	MALE	WHITE	Prostate Adenocarci	2005	-16425	Alive	TUMOR F	2850							Complete Remission/Response	0	2850	0	2850	0	2850	0	2850
TCGA-EJ-	TCGA-EJ-	PRAD	44	MALE	WHITE	Prostate Adenocarci	2005	-16425	Alive	TUMOR F	2850							Complete Remission/Response	0	2850	0	2850	0	2850	0	2850
TCGA-EJ-	TCGA-EJ-	PRAD	71	MALE		Prostate Adenocarci	2006	-26181	Alive	TUMOR F	2542							Complete Remission/Response	0	2542	0	2542	0	2542	0	2542
TCGA-EJ-	TCGA-EJ-	PRAD	58	MALE		Prostate Adenocarci	2011	-21292	Alive	TUMOR F	1099							Complete Remission/Response	0	1099	0	1099	0	1099	0	1099
TCGA-EJ-	TCGA-EJ-	PRAD	62	MALE	WHITE	Prostate Adenocarci	2011	-22766	Alive	TUMOR F	1150							Complete Remission/Response	0	1150	0	1150	0	1150	0	1150
TCGA-EJ-	TCGA-EJ-	PRAD	62	MALE	WHITE	Prostate Adenocarci	2011	-22766	Alive	TUMOR F	1150							Complete Remission/Response	0	1150	0	1150	0	1150	0	1150
TCGA-EJ-	TCGA-EJ-	PRAD	68	MALE	WHITE	Prostate Adenocarci	2011	-25154	Alive	TUMOR F	1008							Complete Remission/Response	0	1008	0	1008	0	1008	0	1008
TCGA-EJ-	TCGA-EJ-	PRAD	68	MALE	WHITE	Prostate Adenocarci	2011	-25154	Alive	TUMOR F	1008							Complete Remission/Response	0	1008	0	1008	0	1008	0	1008
TCGA-EJ-	TCGA-EJ-	PRAD	71	MALE	WHITE	Prostate Adenocarci	2011	-26064	Alive	TUMOR F	771							Complete Remission/Response	0	771	0	771	0	771	0	771
TCGA-EJ-	TCGA-EJ-	PRAD	71	MALE	WHITE	Prostate Adenocarci	2011	-26064	Alive	TUMOR F	771							Complete Remission/Response	0	771	0	771	0	771	0	771
TCGA-EJ-	TCGA-EJ-	PRAD	51	MALE		Prostate Adenocarci	2011	-18686	Alive	TUMOR F	1029			Locoregional Recurrence			380	Partial Remission/Response	0	1029	0	1029			1	380
TCGA-EJ-	TCGA-EJ-	PRAD	57	MALE	BLACK OR AFRICAN AMERICAN	Prostate Adenocarci	2011	-21108	Alive	TUMOR F	824							Complete Remission/Response	0	824	0	824	0	824	0	824
TCGA-EJ-	TCGA-EJ-	PRAD	57	MALE	BLACK OR AFRICAN AMERICAN	Prostate Adenocarci	2011	-21108	Alive	TUMOR F	824							Complete Remission/Response	0	824	0	824	0	824	0	824
TCGA-EJ-	TCGA-EJ-	PRAD	63	MALE		Prostate Adenocarci	2011	-23029	Alive	TUMOR F	1070							Complete Remission/Response	0	1070	0	1070	0	1070	0	1070
TCGA-EJ-	TCGA-EJ-	PRAD	61	MALE	WHITE	Prostate Adenocarci	2011	-22475	Alive	TUMOR F	923							Complete Remission/Response	0	923	0	923	0	923	0	923
TCGA-EJ-	TCGA-EJ-	PRAD	61	MALE	WHITE	Prostate Adenocarci	2011	-22475	Alive	TUMOR F	923							Complete Remission/Response	0	923	0	923	0	923	0	923
TCGA-EJ-	TCGA-EJ-	PRAD	70	MALE	WHITE	Prostate Adenocarci	2011	-25756	Alive	TUMOR F	842							Complete Remission/Response	0	842	0	842	0	842	0	842
TCGA-EJ-	TCGA-EJ-	PRAD	70	MALE	WHITE	Prostate Adenocarci	2011	-25756	Alive	TUMOR F	842							Complete Remission/Response	0	842	0	842	0	842	0	842
TCGA-EJ-	TCGA-EJ-	PRAD	68	MALE	WHITE	Prostate Adenocarci	2011	-25050	Alive	TUMOR F	191								0	191	0	191			0	191
TCGA-EJ-	TCGA-EJ-	PRAD	68	MALE	WHITE	Prostate Adenocarci	2011	-25050	Alive	TUMOR F	191								0	191	0	191			0	191
TCGA-EJ-	TCGA-EJ-	PRAD	64	MALE	WHITE	Prostate Adenocarci	2011	-23458	Alive	TUMOR F	742							Complete Remission/Response	0	742	0	742	0	742	0	742
TCGA-EJ-	TCGA-EJ-	PRAD	64	MALE	WHITE	Prostate Adenocarci	2011	-23458	Alive	TUMOR F	742							Complete Remission/Response	0	742	0	742	0	742	0	742
TCGA-EJ-	TCGA-EJ-	PRAD	65	MALE	WHITE	Prostate Adenocarci	2011	-23937	Alive	TUMOR F	1073							Complete Remission/Response	0	1073	0	1073	0	1073	0	1073
TCGA-EJ-	TCGA-EJ-	PRAD	65	MALE	WHITE	Prostate Adenocarci	2011	-23937	Alive	TUMOR F	1073							Complete Remission/Response	0	1073	0	1073	0	1073	0	1073
TCGA-EJ-	TCGA-EJ-	PRAD	71	MALE	BLACK OR AFRICAN AMERICAN	Prostate Adenocarci	2010	-25965	Alive	TUMOR F	1167							Complete Remission/Response	0	1167	0	1167	0	1167	0	1167
TCGA-EJ-	TCGA-EJ-	PRAD	71	MALE	BLACK OR AFRICAN AMERICAN	Prostate Adenocarci	2010	-25965	Alive	TUMOR F	1167							Complete Remission/Response	0	1167	0	1167	0	1167	0	1167
TCGA-EJ-	TCGA-EJ-	PRAD	70	MALE	WHITE	Prostate Adenocarci	2011	-25600	Alive	TUMOR F	787							Complete Remission/Response	0	787	0	787	0	787	0	787
TCGA-EJ-	TCGA-EJ-	PRAD	70	MALE	WHITE	Prostate Adenocarci	2011	-25600	Alive	TUMOR F	787							Complete Remission/Response	0	787	0	787	0	787	0	787
TCGA-EJ-	TCGA-EJ-	PRAD	63	MALE	WHITE	Prostate Adenocarci	2011	-23183	Alive	TUMOR F	781							Complete Remission/Response	0	781	0	781	0	781	0	781
TCGA-EJ-	TCGA-EJ-	PRAD	63	MALE	WHITE	Prostate Adenocarci	2011	-23183	Alive	TUMOR F	781							Complete Remission/Response	0	781	0	781	0	781	0	781
TCGA-EJ-	TCGA-EJ-	PRAD	54	MALE	WHITE	Prostate Adenocarci	2011	-19939	Alive	TUMOR F	1177							Complete Remission/Response	0	1177	0	1177	0	1177	0	1177
TCGA-EJ-	TCGA-EJ-	PRAD	54	MALE	WHITE	Prostate Adenocarci	2011	-19939	Alive	TUMOR F	1177							Complete Remission/Response	0	1177	0	1177	0	1177	0	1177
TCGA-EJ-	TCGA-EJ-	PRAD	62	MALE	WHITE	Prostate Adenocarci	2011	-22793	Alive	TUMOR F	1096							Complete Remission/Response	0	1096	0	1096	0	1096	0	1096
TCGA-EJ-	TCGA-EJ-	PRAD	62	MALE	WHITE	Prostate Adenocarci	2011	-22793	Alive	TUMOR F	1096							Complete Remission/Response	0	1096	0	1096	0	1096	0	1096
TCGA-EJ-	TCGA-EJ-	PRAD	53	MALE	WHITE	Prostate Adenocarci	2011	-19623	Alive	TUMOR F	859							Complete Remission/Response	0	859	0	859	0	859	0	859
TCGA-EJ-	TCGA-EJ-	PRAD	66	MALE	BLACK OR AFRICAN AMERICAN	Prostate Adenocarci	2011	-24107	Alive	TUMOR F	1105							Complete Remission/Response	0	1105	0	1105	0	1105	0	1105
TCGA-EJ-	TCGA-EJ-	PRAD	66	MALE	BLACK OR AFRICAN AMERICAN	Prostate Adenocarci	2011	-24107	Alive	TUMOR F	1105							Complete Remission/Response	0	1105	0	1105	0	1105	0	1105
TCGA-EJ-	TCGA-EJ-	PRAD	67	MALE	WHITE	Prostate Adenocarci	2011	-24621	Alive	TUMOR F	1118							Complete Remission/Response	0	1118	0	1118	0	1118	0	1118
TCGA-EJ-	TCGA-EJ-	PRAD	53	MALE	WHITE	Prostate Adenocarci	2010	-19454	Alive	TUMOR F	1396							Complete Remission/Response	0	1396	0	1396	0	1396	0	1396
TCGA-EJ-	TCGA-EJ-	PRAD	53	MALE	WHITE	Prostate Adenocarci	2010	-19454	Alive	TUMOR F	1396							Complete Remission/Response	0	1396	0	1396	0	1396	0	1396
TCGA-EJ-	TCGA-EJ-	PRAD	49	MALE	WHITE	Prostate Adenocarci	2011	-17982	Alive	TUMOR F	114							Complete Remission/Response	0	114	0	114	0	114	0	114
TCGA-EJ-	TCGA-EJ-	PRAD	49	MALE	WHITE	Prostate Adenocarci	2011	-17982	Alive	TUMOR F	114							Complete Remission/Response	0	114	0	114	0	114	0	114
TCGA-EJ-	TCGA-EJ-	PRAD	67	MALE	WHITE	Prostate Adenocarci	2010	-24478	Alive	TUMOR F	1306							Complete Remission/Response	0	1306	0	1306	0	1306	0	1306
TCGA-EJ-	TCGA-EJ-	PRAD	67	MALE	WHITE	Prostate Adenocarci	2010	-24478	Alive	TUMOR F	1306							Complete Remission/Response	0	1306	0	1306	0	1306	0	1306
TCGA-EJ-	TCGA-EJ-	PRAD	53	MALE	WHITE	Prostate Adenocarci	2011	-19616	Alive	TUMOR F	983							Complete Remission/Response	0	983	0	983	0	983	0	983
TCGA-EJ-	TCGA-EJ-	PRAD	53	MALE	WHITE	Prostate Adenocarci	2011	-19616	Alive	TUMOR F	983							Complete Remission/Response	0	983	0	983	0	983	0	983
TCGA-EJ-	TCGA-EJ-	PRAD	63	MALE		Prostate Adenocarci	2007	-23371	Alive	TUMOR F	2347							Complete Remission/Response	0	2347	0	2347	0	2347	0	2347
TCGA-EJ-	TCGA-EJ-	PRAD	46	MALE		Prostate Adenocarci	2007	-17014	Alive	WITH TUM	2324			Locoregional Recurrence			1925	Partial Remission/Response	0	2324	0	2324			1	1925
TCGA-EJ-	TCGA-EJ-	PRAD	58	MALE		Prostate Adenocarci	2010	-21209	Alive	TUMOR F	1159							Complete Remission/Response	0	1159	0	1159	0	1159	0	1159
TCGA-EJ-	TCGA-EJ-	PRAD	63	MALE		Prostate Adenocarci	2011	-23104	Alive	TUMOR F	756			Biochemical evidence of disease			196	Partial Remission/Response	0	756	0	756			1	196
TCGA-EJ-	TCGA-EJ-	PRAD	69	MALE		Prostate Adenocarci	2011	-25561	Alive	TUMOR F	766							Complete Remission/Response	0	766	0	766	0	766	0	766
TCGA-EJ-	TCGA-EJ-	PRAD	66	MALE		Prostate Adenocarci	2012	-24305	Alive	TUMOR F	657							Complete Remission/Response	0	657	0	657	0	657	0	657
TCGA-EJ-	TCGA-EJ-	PRAD	53	MALE		Prostate Adenocarci	2012	-19466	Alive	TUMOR F	626							Complete Remission/Response	0	626	0	626	0	626	0	626
TCGA-EJ-	TCGA-EJ-	PRAD	57	MALE		Prostate Adenocarci	2011	-21054	Alive	TUMOR F	524							Complete Remission/Response	0	524	0	524	0	524	0	524
TCGA-EJ-	TCGA-EJ-	PRAD	57	MALE		Prostate Adenocarci	2011	-21160	Alive	TUMOR F	783			Biochemical evidence of disease			215	Partial Remission/Response	0	783	0	783			1	215
TCGA-EJ-	TCGA-EJ-	PRAD	71	MALE		Prostate Adenocarci	2012	-26219	Alive	TUMOR F	668							Complete Remission/Response	0	668	0	668	0	668	0	668
TCGA-EJ-	TCGA-EJ-	PRAD	60	MALE		Prostate Adenocarci	2012	-22059	Alive	TUMOR F	724							Complete Remission/Response	0	724	0	724	0	724	0	724
TCGA-EJ-	TCGA-EJ-	PRAD	57	MALE		Prostate Adenocarci	2011	-21046	Alive	TUMOR F	668							Complete Remission/Response	0	668	0	668	0	668	0	668
TCGA-EJ-	TCGA-EJ-	PRAD	55	MALE		Prostate Adenocarci	2012	-20204	Alive	TUMOR F	710							Complete Remission/Response	0	710	0	710	0	710	0	710
TCGA-EJ-	TCGA-EJ-	PRAD	66	MALE		Prostate Adenocarci	2012	-24297	Alive	TUMOR F	393							Complete Remission/Response	0	393	0	393	0	393	0	393
TCGA-EJ-	TCGA-EJ-	PRAD	67	MALE		Prostate Adenocarci	2012	-24558	Alive	TUMOR F	417							Complete Remission/Response	0	417	0	417	0	417	0	417
TCGA-EJ-	TCGA-EJ-	PRAD	59	MALE		Prostate Adenocarci	2012	-21681	Alive	[Discrepar	560			Biochemical evidence of disease			75	Progressive Disease	0	560	0	560			1	75
TCGA-EJ-	TCGA-EJ-	PRAD	57	MALE		Prostate Adenocarci	2012	-20821	Alive	TUMOR F	666							Complete Remission/Response	0	666	0	666	0	666	0	666
TCGA-EJ-	TCGA-EJ-	PRAD	62	MALE		Prostate Adenocarci	2012	-22904	Alive	TUMOR F	451							Complete Remission/Response	0	451	0	451	0	451	0	451
TCGA-EJ-	TCGA-EJ-	PRAD	65	MALE		Prostate Adenocarci	2013	-23767	Alive	TUMOR F	230							Complete Remission/Response	0	230	0	230	0	230	0	230
TCGA-EJ-	TCGA-EJ-	PRAD	70	MALE		Prostate Adenocarci	2012	-25679	Alive	TUMOR F	353			Locoregional Recurrence			353	Partial Remission/Response	0	353	0	353			1	353
TCGA-EJ-	TCGA-EJ-	PRAD	64	MALE		Prostate Adenocarci	2011	-23598	Alive	TUMOR F	873							Complete Remission/Response	0	873	0	873	0	873	0	873
TCGA-EJ-	TCGA-EJ-	PRAD	56	MALE		Prostate Adenocarci	2013	-20587	Alive	TUMOR F	250							Complete Remission/Response	0	250	0	250	0	250	0	250
TCGA-EJ-	TCGA-EJ-	PRAD	64	MALE		Prostate Adenocarci	2013	-23458	Alive	TUMOR F	432							Complete Remission/Response	0	432	0	432	0	432	0	432
TCGA-EJ-	TCGA-EJ-	PRAD	56	MALE		Prostate Adenocarci	2013	-20734	Alive	TUMOR F	474							Complete Remission/Response	0	474	0	474	0	474	0	474
TCGA-EJ-	TCGA-EJ-	PRAD	59	MALE		Prostate Adenocarci	2013	-21748	Alive	TUMOR F	197							Complete Remission/Response	0	197	0	197	0	197	0	197
TCGA-EJ-	TCGA-EJ-	PRAD	58	MALE		Prostate Adenocarci	2012	-21530	Alive	TUMOR F	476							Complete Remission/Response	0	476	0	476	0	476	0	476
TCGA-EJ-	TCGA-EJ-	PRAD	64	MALE		Prostate Adenocarci	2013	-23603	Alive	TUMOR F	128							Complete Remission/Response	0	128	0	128	0	128	0	128
TCGA-EJ-	TCGA-EJ-	PRAD	61	MALE		Prostate Adenocarci	2013	-22499	Alive	TUMOR F	197			Biochemical evidence of disease			197	Complete Remission/Response	0	197	0	197	1	197	1	197
TCGA-EJ-	TCGA-EJ-	PRAD	58	MALE		Prostate Adenocarci	2013	-21506	Alive	TUMOR F	329							Complete Remission/Response	0	329	0	329	0	329	0	329
TCGA-EJ-	TCGA-EJ-	PRAD	51	MALE		Prostate Adenocarci	2013	-18875	Alive	TUMOR F	282							Complete Remission/Response	0	282	0	282	0	282	0	282
TCGA-EJ-	TCGA-EJ-	PRAD	51	MALE		Prostate Adenocarci	2013	-18875	Alive	TUMOR F	282							Complete Remission/Response	0	282	0	282	0	282	0	282
TCGA-EJ-	TCGA-EJ-	PRAD	59	MALE		Prostate Adenocarci	2013	-21769	Alive	TUMOR F	299			Biochemical evidence of disease			117	Complete Remission/Response	0	299	0	299	1	117	1	117
TCGA-EJ-	TCGA-EJ-	PRAD	66	MALE		Prostate Adenocarci	2013	-24380	Alive	TUMOR F	266			Biochemical evidence of disease			216	Complete Remission/Response	0	266	0	266	1	216	1	216
TCGA-EJ-	TCGA-EJ-	PRAD	64	MALE		Prostate Adenocarci	2013	-23472	Alive	TUMOR F	138							Complete Remission/Response	0	138	0	138	0	138	0	138
TCGA-EJ-	TCGA-EJ-	PRAD	61	MALE		Prostate Adenocarci	2013	-22626	Alive	TUMOR F	131							Complete Remission/Response	0	131	0	131	0	131	0	131
TCGA-EJ-	TCGA-EJ-	PRAD	51	MALE		Prostate Adenocarci	2013	-18648	Alive	TUMOR F	145							Complete Remission/Response	0	145	0	145	0	145	0	145
TCGA-FC	TCGA-FC	PRAD	52	MALE	ASIAN	Prostate Adenocarci	2011	-19144	Alive	TUMOR F	864							Complete Remission/Response	0	864	0	864	0	864	0	864
TCGA-FC	TCGA-FC	PRAD	62	MALE	WHITE	Prostate Adenocarci	2011	-22888	Alive	TUMOR F	469							Complete Remission/Response	0	469	0	469	0	469	0	469
TCGA-FC	TCGA-FC	PRAD	70	MALE		Prostate Adenocarci	2012	-25931	Alive	TUMOR F	877							Complete Remission/Response	0	877	0	877	0	877	0	877
TCGA-FC	TCGA-FC	PRAD	53	MALE		Prostate Adenocarci	2012	-19413	Alive	WITH TUM	680							[Unknown]	0	680	0	680			0	680
TCGA-FC	TCGA-FC	PRAD	74	MALE		Prostate Adenocarci	2012	-27282	Alive	TUMOR F	524							Complete Remission/Response	0	524	0	524	0	524	0	524
TCGA-FC	TCGA-FC	PRAD	77	MALE		Prostate Adenocarci	2013	-28197	Alive	TUMOR F	789							Complete Remission/Response	0	789	0	789	0	789	0	789
TCGA-FC	TCGA-FC	PRAD	68	MALE		Prostate Adenocarci	2013	-25115	Alive	TUMOR F	616							Complete Remission/Response	0	616	0	616	0	616	0	616
TCGA-G9	TCGA-G9	PRAD	67	MALE	WHITE	Prostate Adenocarci	2009	-24797	Alive	TUMOR F	1266							Complete Remission/Response	0	1266	0	1266	0	1266	0	1266
TCGA-G9	TCGA-G9	PRAD	55	MALE	WHITE	Prostate Adenocarci	2005	-20166	Alive	WITH TUM	2657			Biochemical evidence of disease			1180	Complete Remission/Response	0	2657	0	2657	1	1180	1	1180
TCGA-G9	TCGA-G9	PRAD	55	MALE	WHITE	Prostate Adenocarci	2005	-20166	Alive	WITH TUM	2657			Biochemical evidence of disease			1180	Complete Remission/Response	0	2657	0	2657	1	1180	1	1180
TCGA-G9	TCGA-G9	PRAD	66	MALE	WHITE	Prostate Adenocarci	2006	-24249	Alive	TUMOR F	2465							Complete Remission/Response	0	2465	0	2465	0	2465	0	2465
TCGA-G9	TCGA-G9	PRAD	66	MALE	WHITE	Prostate Adenocarci	2006	-24249	Alive	TUMOR F	2465							Complete Remission/Response	0	2465	0	2465	0	2465	0	2465
TCGA-G9	TCGA-G9	PRAD	57	MALE	WHITE	Prostate Adenocarci	2007	-20851	Alive	TUMOR F	2068							Complete Remission/Response	0	2068	0	2068	0	2068	0	2068
TCGA-G9	TCGA-G9	PRAD	61	MALE	WHITE	Prostate Adenocarci	2007	-22360	Alive	TUMOR F	2028							Complete Remission/Response	0	2028	0	2028	0	2028	0	2028
TCGA-G9	TCGA-G9	PRAD	58	MALE		Prostate Adenocarci	2007	-21305	Alive	TUMOR F	2551			Biochemical evidence of disease			1634	Complete Remission/Response	0	2551	0	2551	1	1634	1	1634
TCGA-G9	TCGA-G9	PRAD	61	MALE	WHITE	Prostate Adenocarci	2008	-22472	Alive	TUMOR F	1696							Complete Remission/Response	0	1696	0	1696	0	1696	0	1696
TCGA-G9	TCGA-G9	PRAD	61	MALE	WHITE	Prostate Adenocarci	2008	-22472	Alive	TUMOR F	1696							Complete Remission/Response	0	1696	0	1696	0	1696	0	1696
TCGA-G9	TCGA-G9	PRAD	62	MALE	WHITE	Prostate Adenocarci	2008	-22843	Alive	TUMOR F	2450							Complete Remission/Response	0	2450	0	2450	0	2450	0	2450
TCGA-G9	TCGA-G9	PRAD	59	MALE		Prostate Adenocarci	2008	-21721	Alive	TUMOR F	2089							Complete Remission/Response	0	2089	0	2089	0	2089	0	2089
TCGA-G9	TCGA-G9	PRAD	68	MALE	WHITE	Prostate Adenocarci	2008	-24936	Alive	TUMOR F	1515							Complete Remission/Response	0	1515	0	1515	0	1515	0	1515
TCGA-G9	TCGA-G9	PRAD	68	MALE	WHITE	Prostate Adenocarci	2008	-24936	Alive	TUMOR F	1515							Complete Remission/Response	0	1515	0	1515	0	1515	0	1515
TCGA-G9	TCGA-G9	PRAD	51	MALE	WHITE	Prostate Adenocarci	2008	-18723	Alive	TUMOR F	2048							Complete Remission/Response	0	2048	0	2048	0	2048	0	2048
TCGA-G9	TCGA-G9	PRAD	51	MALE	WHITE	Prostate Adenocarci	2008	-18723	Alive	TUMOR F	2048							Complete Remission/Response	0	2048	0	2048	0	2048	0	2048
TCGA-G9	TCGA-G9	PRAD	58	MALE	WHITE	Prostate Adenocarci	2009	-21221	Alive	TUMOR F	1542							Complete Remission/Response	0	1542	0	1542	0	1542	0	1542
TCGA-G9	TCGA-G9	PRAD	58	MALE	WHITE	Prostate Adenocarci	2009	-21221	Alive	TUMOR F	1542							Complete Remission/Response	0	1542	0	1542	0	1542	0	1542
TCGA-G9	TCGA-G9	PRAD	55	MALE		Prostate Adenocarci	2009	-20403	Alive	TUMOR F	2066							Complete Remission/Response	0	2066	0	2066	0	2066	0	2066
TCGA-G9	TCGA-G9	PRAD	60	MALE	WHITE	Prostate Adenocarci	2009	-22010	Alive	TUMOR F	1434							Complete Remission/Response	0	1434	0	1434	0	1434	0	1434
TCGA-G9	TCGA-G9	PRAD	60	MALE	WHITE	Prostate Adenocarci	2009	-22010	Alive	TUMOR F	1434							Complete Remission/Response	0	1434	0	1434	0	1434	0	1434
TCGA-G9	TCGA-G9	PRAD	61	MALE	WHITE	Prostate Adenocarci	2009	-22565	Alive	TUMOR F	1415							Complete Remission/Response	0	1415	0	1415	0	1415	0	1415
TCGA-G9	TCGA-G9	PRAD	57	MALE	WHITE	Prostate Adenocarci	2009	-21149	Alive	WITH TUM	1443							Progressive Disease	0	1443	0	1443			0	1443
TCGA-G9	TCGA-G9	PRAD	57	MALE	WHITE	Prostate Adenocarci	2009	-21149	Alive	WITH TUM	1443							Progressive Disease	0	1443	0	1443			0	1443
TCGA-G9	TCGA-G9	PRAD	64	MALE	WHITE	Prostate Adenocarci	2009	-23480	Alive	TUMOR F	1378							Complete Remission/Response	0	1378	0	1378	0	1378	0	1378
TCGA-G9	TCGA-G9	PRAD	64	MALE	WHITE	Prostate Adenocarci	2009	-23480	Alive	TUMOR F	1378							Complete Remission/Response	0	1378	0	1378	0	1378	0	1378
TCGA-G9	TCGA-G9	PRAD	72	MALE	WHITE	Prostate Adenocarci	2009	-26603	Alive	TUMOR F	1198							Complete Remission/Response	0	1198	0	1198	0	1198	0	1198
TCGA-G9	TCGA-G9	PRAD	72	MALE	WHITE	Prostate Adenocarci	2009	-26603	Alive	TUMOR F	1198							Complete Remission/Response	0	1198	0	1198	0	1198	0	1198
TCGA-G9	TCGA-G9	PRAD	71	MALE	WHITE	Prostate Adenocarci	2009	-26207	Alive	TUMOR F	1363							Complete Remission/Response	0	1363	0	1363	0	1363	0	1363
TCGA-G9	TCGA-G9	PRAD	71	MALE	WHITE	Prostate Adenocarci	2009	-26207	Alive	TUMOR F	1363							Complete Remission/Response	0	1363	0	1363	0	1363	0	1363
TCGA-G9	TCGA-G9	PRAD	61	MALE	WHITE	Prostate Adenocarci	2009	-22428	Alive		1947							Complete Remission/Response	0	1947	0	1947	0	1947	0	1947
TCGA-G9	TCGA-G9	PRAD	60	MALE	WHITE	Prostate Adenocarci	2009	-22176	Alive	WITH TUM	1222							Progressive Disease	0	1222	0	1222			0	1222
TCGA-G9	TCGA-G9	PRAD	60	MALE	WHITE	Prostate Adenocarci	2009	-22176	Alive	WITH TUM	1222							Progressive Disease	0	1222	0	1222			0	1222
TCGA-G9	TCGA-G9	PRAD	55	MALE	WHITE	Prostate Adenocarci	2009	-20238	Alive	TUMOR F	1215							Complete Remission/Response	0	1215	0	1215	0	1215	0	1215
TCGA-G9	TCGA-G9	PRAD	52	MALE	WHITE	Prostate Adenocarci	2009	-19149	Alive	TUMOR F	1156							Complete Remission/Response	0	1156	0	1156	0	1156	0	1156
TCGA-G9	TCGA-G9	PRAD	58	MALE	WHITE	Prostate Adenocarci	2009	-21365	Alive	TUMOR F	1226							Complete Remission/Response	0	1226	0	1226	0	1226	0	1226
TCGA-G9	TCGA-G9	PRAD	68	MALE	WHITE	Prostate Adenocarci	2009	-24887	Alive	TUMOR F	811							Complete Remission/Response	0	811	0	811	0	811	0	811
TCGA-G9	TCGA-G9	PRAD	68	MALE	WHITE	Prostate Adenocarci	2009	-24887	Alive	TUMOR F	811							Complete Remission/Response	0	811	0	811	0	811	0	811
TCGA-G9	TCGA-G9	PRAD	61	MALE	WHITE	Prostate Adenocarci	2009	-22419	Alive	TUMOR F	958							Complete Remission/Response	0	958	0	958	0	958	0	958
TCGA-G9	TCGA-G9	PRAD	54	MALE	BLACK OR AFRICAN AMERICAN	Prostate Adenocarci	2009	-19879	Alive	TUMOR F	1155							Complete Remission/Response	0	1155	0	1155	0	1155	0	1155
TCGA-G9	TCGA-G9	PRAD	68	MALE		Prostate Adenocarci	2009	-25126	Alive	TUMOR F	1778							Complete Remission/Response	0	1778	0	1778	0	1778	0	1778
TCGA-G9	TCGA-G9	PRAD	53	MALE	WHITE	Prostate Adenocarci	2010	-19620	Alive	TUMOR F	765							Complete Remission/Response	0	765	0	765	0	765	0	765
TCGA-G9	TCGA-G9	PRAD	53	MALE	WHITE	Prostate Adenocarci	2010	-19620	Alive	TUMOR F	765							Complete Remission/Response	0	765	0	765	0	765	0	765
TCGA-G9	TCGA-G9	PRAD	66	MALE	WHITE	Prostate Adenocarci	2010	-24399	Alive	TUMOR F	830							Complete Remission/Response	0	830	0	830	0	830	0	830
TCGA-G9	TCGA-G9	PRAD	66	MALE	WHITE	Prostate Adenocarci	2010	-24399	Alive	TUMOR F	830							Complete Remission/Response	0	830	0	830	0	830	0	830
TCGA-G9	TCGA-G9	PRAD	66	MALE	WHITE	Prostate Adenocarci	2008	-24381	Alive	WITH TUM	1771							Progressive Disease	0	1771	0	1771			0	1771
TCGA-G9	TCGA-G9	PRAD	66	MALE	WHITE	Prostate Adenocarci	2008	-24381	Alive	WITH TUM	1771							Progressive Disease	0	1771	0	1771			0	1771
TCGA-G9	TCGA-G9	PRAD	61	MALE	WHITE	Prostate Adenocarci	2008	-22602	Alive	TUMOR F	1726							Complete Remission/Response	0	1726	0	1726	0	1726	0	1726
TCGA-G9	TCGA-G9	PRAD	61	MALE	WHITE	Prostate Adenocarci	2008	-22602	Alive	TUMOR F	1726							Complete Remission/Response	0	1726	0	1726	0	1726	0	1726
TCGA-G9	TCGA-G9	PRAD	53	MALE		Prostate Adenocarci	2008	-19568	Alive	TUMOR F	1952			Biochemical evidence of disease			1342	Complete Remission/Response	0	1952	0	1952	1	1342	1	1342
TCGA-G9	TCGA-G9	PRAD	66	MALE	WHITE	Prostate Adenocarci	2008	-24388	Alive	TUMOR F	1543							Complete Remission/Response	0	1543	0	1543	0	1543	0	1543
TCGA-G9	TCGA-G9	PRAD	66	MALE	WHITE	Prostate Adenocarci	2008	-24388	Alive	TUMOR F	1543							Complete Remission/Response	0	1543	0	1543	0	1543	0	1543
TCGA-G9	TCGA-G9	PRAD	48	MALE		Prostate Adenocarci	2009	-17538	Alive	TUMOR F	1610							Complete Remission/Response	0	1610	0	1610	0	1610	0	1610
TCGA-G9	TCGA-G9	PRAD	66	MALE		Prostate Adenocarci	2010	-24187	Alive	TUMOR F	1185							Complete Remission/Response	0	1185	0	1185	0	1185	0	1185
TCGA-G9	TCGA-G9	PRAD	58	MALE		Prostate Adenocarci	2010	-21276	Alive	TUMOR F	849							Complete Remission/Response	0	849	0	849	0	849	0	849
TCGA-G9	TCGA-G9	PRAD	52	MALE		Prostate Adenocarci	2010	-19216	Alive	[Discrepar	942							[Discrepancy]	0	942	0	942			0	942
TCGA-G9	TCGA-G9	PRAD	49	MALE		Prostate Adenocarci	2011	-18033	Alive	TUMOR F	1078							Complete Remission/Response	0	1078	0	1078	0	1078	0	1078
TCGA-G9	TCGA-G9	PRAD	44	MALE		Prostate Adenocarci	2011	-16269	Alive	TUMOR F	857							Complete Remission/Response	0	857	0	857	0	857	0	857
TCGA-G9	TCGA-G9	PRAD	64	MALE		Prostate Adenocarci	2010	-23481	Alive	TUMOR F	994							Complete Remission/Response	0	994	0	994	0	994	0	994
TCGA-G9	TCGA-G9	PRAD	53	MALE		Prostate Adenocarci	2011	-19530	Dead	WITH TUMOR		791	Prostate (	Distant M	Bone		423	Progressive Disease	1	791	1	791			1	423
TCGA-G9	TCGA-G9	PRAD	62	MALE		Prostate Adenocarci	2012	-22684	Alive	TUMOR F	906							Complete Remission/Response	0	906	0	906	0	906	0	906
TCGA-G9	TCGA-G9	PRAD	55	MALE		Prostate Adenocarci	2012	-20213	Alive	WITH TUM	728							Partial Remission/Response	0	728	0	728			0	728
TCGA-H9	TCGA-H9	PRAD	68	MALE	WHITE	Prostate Adenocarci	2011	-24898	Alive	TUMOR F	185							Complete Remission/Response	0	185	0	185	0	185	0	185
TCGA-H9	TCGA-H9	PRAD	60	MALE		Prostate Adenocarci	2012	-22039	Alive	TUMOR F	941							Complete Remission/Response	0	941	0	941	0	941	0	941
TCGA-H9	TCGA-H9	PRAD	63	MALE		Prostate Adenocarci	2012	-23297	Alive		112							[Unknown]	0	112	0	112			0	112
TCGA-HC	TCGA-HC	PRAD	63	MALE	WHITE	Prostate Adenocarci	2010		Alive	TUMOR F	601							Complete Remission/Response	0	601	0	601	0	601	0	601
TCGA-HC	TCGA-HC	PRAD	64	MALE	BLACK OR AFRICAN AMERICAN	Prostate Adenocarci	2006	-23636	Alive	TUMOR F	2115							Complete Remission/Response	0	2115	0	2115	0	2115	0	2115
TCGA-HC	TCGA-HC	PRAD	69	MALE	WHITE	Prostate Adenocarci	2007	-25402	Alive	TUMOR F	1985							Complete Remission/Response	0	1985	0	1985	0	1985	0	1985
TCGA-HC	TCGA-HC	PRAD	51	MALE	WHITE	Prostate Adenocarci	2010	-18734	Alive	WITH TUM	1184			Biochemical evidence of disease			380	Partial Remission/Response	0	1184	0	1184			1	380
TCGA-HC	TCGA-HC	PRAD	66	MALE	WHITE	Prostate Adenocarci	2010	-24243	Alive	TUMOR F	1106			New Primary Tumor			894	Complete Remission/Response	0	1106	0	1106	0	1106	1	894
TCGA-HC	TCGA-HC	PRAD	62	MALE	WHITE	Prostate Adenocarci	2010		Alive	WITH TUM	1136							Partial Remission/Response	0	1136	0	1136			0	1136
TCGA-HC	TCGA-HC	PRAD	60	MALE	WHITE	Prostate Adenocarci	2011	-22129	Alive	TUMOR F	440							Complete Remission/Response	0	440	0	440	0	440	0	440
TCGA-HC	TCGA-HC	PRAD	74	MALE	WHITE	Prostate Adenocarci	2011	-27343	Alive	TUMOR F	868							Complete Remission/Response	0	868	0	868	0	868	0	868
TCGA-HC	TCGA-HC	PRAD	62	MALE	WHITE	Prostate Adenocarci	2010	-22768	Alive	TUMOR F	1221							Complete Remission/Response	0	1221	0	1221	0	1221	0	1221
TCGA-HC	TCGA-HC	PRAD	62	MALE	WHITE	Prostate Adenocarci	2010	-22768	Alive	TUMOR F	1221							Complete Remission/Response	0	1221	0	1221	0	1221	0	1221
TCGA-HC	TCGA-HC	PRAD	56	MALE	WHITE	Prostate Adenocarci	2011	-20576	Alive	TUMOR F	870							Complete Remission/Response	0	870	0	870	0	870	0	870
TCGA-HC	TCGA-HC	PRAD	53	MALE	WHITE	Prostate Adenocarci	2010	-19699	Alive	TUMOR F	1219			Biochemical evidence of disease			170	Partial Remission/Response	0	1219	0	1219			1	170
TCGA-HC	TCGA-HC	PRAD	50	MALE	WHITE	Prostate Adenocarci	2011	-18577	Alive	TUMOR F	1001							Complete Remission/Response	0	1001	0	1001	0	1001	0	1001
TCGA-HC	TCGA-HC	PRAD	66	MALE	WHITE	Prostate Adenocarci	2011	-24278	Alive	TUMOR F	939							Complete Remission/Response	0	939	0	939	0	939	0	939
TCGA-HC	TCGA-HC	PRAD	66	MALE	WHITE	Prostate Adenocarci	2011	-24324	Alive	WITH TUM	1161			Biochemical evidence of disease			766	Complete Remission/Response	0	1161	0	1161	1	766	1	766
TCGA-HC	TCGA-HC	PRAD	73	MALE	WHITE	Prostate Adenocarci	2010		Alive	TUMOR F	1355							Complete Remission/Response	0	1355	0	1355	0	1355	0	1355

TCGA-HC	TCGA-HC	PRAD	59	MALE	WHITE	Prostate Adenocarci	2010	-21703	Alive	TUMOR F	1103							Complete Remission/Response	0	1103	0	1103	0	1103	0	1103
TCGA-HC	TCGA-HC	PRAD	55	MALE	BLACK OR AFRICAN AMERICAN	Prostate Adenocarci	2010	-20122	Alive	TUMOR F	1090							Complete Remission/Response	0	1090	0	1090	0	1090	0	1090
TCGA-HC	TCGA-HC	PRAD	55	MALE	BLACK OR AFRICAN AMERICAN	Prostate Adenocarci	2010	-20122	Alive	TUMOR F	1090							Complete Remission/Response	0	1090	0	1090	0	1090	0	1090
TCGA-HC	TCGA-HC	PRAD	58	MALE	WHITE	Prostate Adenocarci	2011	-21195	Alive	WITH TUN	964			Biochemi	Other, sp	biochemi	420	Partial Remission/Response	0	964	0	964			1	420
TCGA-HC	TCGA-HC	PRAD	58	MALE	WHITE	Prostate Adenocarci	2011	-21195	Alive	WITH TUN	964			Biochemi	Other, sp	biochemi	420	Partial Remission/Response	0	964	0	964			1	420
TCGA-HC	TCGA-HC	PRAD	59	MALE	WHITE	Prostate Adenocarci	2011	-21883	Alive	TUMOR F	717							Complete Remission/Response	0	717	0	717	0	717	0	717
TCGA-HC	TCGA-HC	PRAD	59	MALE	WHITE	Prostate Adenocarci	2011	-21883	Alive	TUMOR F	717							Complete Remission/Response	0	717	0	717	0	717	0	717
TCGA-HC	TCGA-HC	PRAD	58	MALE	WHITE	Prostate Adenocarci	2011	-21323	Alive	WITH TUN	938							Partial Remission/Response	0	938	0	938			0	938
TCGA-HC	TCGA-HC	PRAD	58	MALE	WHITE	Prostate Adenocarci	2011	-21323	Alive	WITH TUN	938							Partial Remission/Response	0	938	0	938			0	938
TCGA-HC	TCGA-HC	PRAD	46	MALE	WHITE	Prostate Adenocarci	2011	-17065	Alive	TUMOR F	531							Complete Remission/Response	0	531	0	531	0	531	0	531
TCGA-HC	TCGA-HC	PRAD	65	MALE	WHITE	Prostate Adenocarci	2011	-24078	Alive	TUMOR F	721							Complete Remission/Response	0	721	0	721	0	721	0	721
TCGA-HC	TCGA-HC	PRAD	65	MALE	WHITE	Prostate Adenocarci	2011	-24078	Alive	TUMOR F	721							Complete Remission/Response	0	721	0	721	0	721	0	721
TCGA-HC	TCGA-HC	PRAD	52	MALE	WHITE	Prostate Adenocarci	2011	-19188	Alive	TUMOR F	748							Complete Remission/Response	0	748	0	748	0	748	0	748
TCGA-HC	TCGA-HC	PRAD	52	MALE	WHITE	Prostate Adenocarci	2011	-19188	Alive	TUMOR F	748							Complete Remission/Response	0	748	0	748	0	748	0	748
TCGA-HC	TCGA-HC	PRAD	60	MALE	WHITE	Prostate Adenocarci	2011	-22072	Alive	TUMOR F	792							Complete Remission/Response	0	792	0	792	0	792	0	792
TCGA-HC	TCGA-HC	PRAD	70	MALE	WHITE	Prostate Adenocarci	2011	-25688	Alive	TUMOR F	724							Complete Remission/Response	0	724	0	724	0	724	0	724
TCGA-HC	TCGA-HC	PRAD	50	MALE	WHITE	Prostate Adenocarci	2011	-18580	Alive	TUMOR F	522							Complete Remission/Response	0	522	0	522	0	522	0	522
TCGA-HC	TCGA-HC	PRAD	61	MALE	WHITE	Prostate Adenocarci	2011	-22366	Alive	TUMOR F	782							Complete Remission/Response	0	782	0	782	0	782	0	782
TCGA-HC	TCGA-HC	PRAD	61	MALE	WHITE	Prostate Adenocarci	2011	-22366	Alive	TUMOR F	782							Complete Remission/Response	0	782	0	782	0	782	0	782
TCGA-HC	TCGA-HC	PRAD	52	MALE		Prostate Adenocarci	2011	-19207	Alive	TUMOR F	928							Complete Remission/Response	0	928	0	928	0	928	0	928
TCGA-HC	TCGA-HC	PRAD	66	MALE	WHITE	Prostate Adenocarci	2011	-24391	Alive	TUMOR F	938							Complete Remission/Response	0	938	0	938	0	938	0	938
TCGA-HC	TCGA-HC	PRAD	53	MALE	WHITE	Prostate Adenocarci	2011	-19663	Alive	TUMOR F	543							Complete Remission/Response	0	543	0	543	0	543	0	543
TCGA-HC	TCGA-HC	PRAD	53	MALE	WHITE	Prostate Adenocarci	2011	-19663	Alive	TUMOR F	543							Complete Remission/Response	0	543	0	543	0	543	0	543
TCGA-HC	TCGA-HC	PRAD	72	MALE	WHITE	Prostate Adenocarci	2011	-26576	Alive	TUMOR F	747							Complete Remission/Response	0	747	0	747	0	747	0	747
TCGA-HC	TCGA-HC	PRAD	72	MALE	WHITE	Prostate Adenocarci	2011	-26576	Alive	TUMOR F	747							Complete Remission/Response	0	747	0	747	0	747	0	747
TCGA-HC	TCGA-HC	PRAD	54	MALE	WHITE	Prostate Adenocarci	2011	-19894	Alive	WITH TUN	956							Complete Remission/Response	0	956	0	956	0	956	0	956
TCGA-HC	TCGA-HC	PRAD	48	MALE		Prostate Adenocarci	2011	-17673	Alive	TUMOR F	589							Complete Remission/Response	0	589	0	589	0	589	0	589
TCGA-HC	TCGA-HC	PRAD	58	MALE		Prostate Adenocarci	2011	-21428	Alive	TUMOR F	682							Complete Remission/Response	0	682	0	682	0	682	0	682
TCGA-HC	TCGA-HC	PRAD	53	MALE		Prostate Adenocarci	2011	-19598	Alive	TUMOR F	860							Complete Remission/Response	0	860	0	860	0	860	0	860
TCGA-HC	TCGA-HC	PRAD	70	MALE		Prostate Adenocarci	2011	-25629	Alive	TUMOR F	726							Complete Remission/Response	0	726	0	726	0	726	0	726
TCGA-HC	TCGA-HC	PRAD	56	MALE		Prostate Adenocarci	2011	-20703	Alive	TUMOR F	847							Complete Remission/Response	0	847	0	847	0	847	0	847
TCGA-HC	TCGA-HC	PRAD	56	MALE		Prostate Adenocarci	2011	-20703	Alive	TUMOR F	847							Complete Remission/Response	0	847	0	847	0	847	0	847
TCGA-HC	TCGA-HC	PRAD	50	MALE		Prostate Adenocarci	2011	-18309	Alive	TUMOR F	924							Complete Remission/Response	0	924	0	924	0	924	0	924
TCGA-HC	TCGA-HC	PRAD	50	MALE		Prostate Adenocarci	2011	-18309	Alive	TUMOR F	924							Complete Remission/Response	0	924	0	924	0	924	0	924
TCGA-HC	TCGA-HC	PRAD	43	MALE		Prostate Adenocarci	2011	-15884	Alive	TUMOR F	686							Complete Remission/Response	0	686	0	686	0	686	0	686
TCGA-HC	TCGA-HC	PRAD	43	MALE		Prostate Adenocarci	2011	-15884	Alive	TUMOR F	686							Complete Remission/Response	0	686	0	686	0	686	0	686
TCGA-HC	TCGA-HC	PRAD	48	MALE		Prostate Adenocarci	2011	-17874	Alive	TUMOR F	546							Complete Remission/Response	0	546	0	546	0	546	0	546
TCGA-HC	TCGA-HC	PRAD	57	MALE		Prostate Adenocarci	2011	-20946	Alive	TUMOR F	679							Complete Remission/Response	0	679	0	679	0	679	0	679
TCGA-HC	TCGA-HC	PRAD	57	MALE		Prostate Adenocarci	2011	-20946	Alive	TUMOR F	679							Complete Remission/Response	0	679	0	679	0	679	0	679
TCGA-HC	TCGA-HC	PRAD	60	MALE		Prostate Adenocarci	2011	-22177	Alive	WITH TUN	48								0	48	0	48			0	48
TCGA-HC	TCGA-HC	PRAD	66	MALE		Prostate Adenocarci	2011	-24382	Alive	TUMOR F	483							Complete Remission/Response	0	483	0	483	0	483	0	483
TCGA-HC	TCGA-HC	PRAD	55	MALE		Prostate Adenocarci	2012	-20280	Alive	WITH TUN	473							[Unknown]	0	473	0	473			0	473
TCGA-HC	TCGA-HC	PRAD	47	MALE		Prostate Adenocarci	2012	-17434	Alive	TUMOR F	680							Complete Remission/Response	0	680	0	680	0	680	0	680
TCGA-HC	TCGA-HC	PRAD	56	MALE		Prostate Adenocarci	2012	-20634	Alive	WITH TUN	23							[Unknown]	0	23	0	23			0	23
TCGA-HC	TCGA-HC	PRAD	67	MALE		Prostate Adenocarci	2012	-24749	Alive	WITH TUN	54								0	54	0	54			0	54
TCGA-HC	TCGA-HC	PRAD	68	MALE		Prostate Adenocarci	2013	-24916	Alive	TUMOR F	61							[Unknown]	0	61	0	61			0	61
TCGA-HC	TCGA-HC	PRAD	64	MALE		Prostate Adenocarci	2012	-23618	Alive	TUMOR F	67							[Unknown]	0	67	0	67			0	67
TCGA-HC	TCGA-HC	PRAD	67	MALE		Prostate Adenocarci	2012	-24697	Alive	WITH TUN	49								0	49	0	49			0	49
TCGA-HC	TCGA-HC	PRAD	52	MALE		Prostate Adenocarci	2012	-19298	Alive	TUMOR F	530							Complete Remission/Response	0	530	0	530	0	530	0	530
TCGA-HC	TCGA-HC	PRAD	61	MALE		Prostate Adenocarci	2012	-22318	Alive		71							[Unknown]	0	71	0	71			0	71
TCGA-HC	TCGA-HC	PRAD	60	MALE		Prostate Adenocarci	2012	-22208	Alive	TUMOR F	106							[Unknown]	0	106	0	106			0	106
TCGA-HC	TCGA-HC	PRAD	70	MALE		Prostate Adenocarci	2012	-25661	Alive	TUMOR F	44							[Unknown]	0	44	0	44			0	44
TCGA-HC	TCGA-HC	PRAD	55	MALE		Prostate Adenocarci	2013	-20263	Alive	TUMOR F	38							[Unknown]	0	38	0	38			0	38
TCGA-HC	TCGA-HC	PRAD	51	MALE		Prostate Adenocarci	2012	-18779	Alive	TUMOR F	134							[Unknown]	0	134	0	134			0	134
TCGA-HC	TCGA-HC	PRAD	73	MALE		Prostate Adenocarci	2012	-26755	Alive	WITH TUN	213							[Unknown]	0	213	0	213			0	213
TCGA-HC	TCGA-HC	PRAD	62	MALE		Prostate Adenocarci	2013	-22756	Alive	TUMOR F	150							[Unknown]	0	150	0	150			0	150
TCGA-HC	TCGA-HC	PRAD	63	MALE		Prostate Adenocarci	2013	-23154	Alive	WITH TUN	290							[Unknown]	0	290	0	290			0	290
TCGA-HC	TCGA-HC	PRAD	61	MALE		Prostate Adenocarci	2012	-22465	Alive	TUMOR F	775							Complete Remission/Response	0	775	0	775	0	775	0	775
TCGA-HC	TCGA-HC	PRAD	68	MALE		Prostate Adenocarci	2012	-25137	Alive	TUMOR F	573							Complete Remission/Response	0	573	0	573	0	573	0	573
TCGA-HC	TCGA-HC	PRAD	64	MALE		Prostate Adenocarci	2013	-23543	Alive	WITH TUN	587			Biochemi	Other, sp	Biochemi	216	Partial Remission/Response	0	587	0	587			1	216
TCGA-HC	TCGA-HC	PRAD	58	MALE		Prostate Adenocarci	2013	-21460	Alive	WITH TUN	844			Biochemi	Other, sp	Biochemi	351	Partial Remission/Response	0	844	0	844			1	351
TCGA-HI-	TCGA-HI-	PRAD	62	MALE	WHITE	Prostate Adenocarci	2005	-22881	Alive	WITH TUN	3323			Biochemi	Bone		2505	Stable Disease	0	3323	0	3323			1	2505
TCGA-HI-	TCGA-HI-	PRAD	55	MALE	WHITE	Prostate Adenocarci	2006	-20133	Alive	TUMOR F	2684							Complete Remission/Response	0	2684	0	2684	0	2684	0	2684
TCGA-HI-	TCGA-HI-	PRAD	58	MALE	WHITE	Prostate Adenocarci	2006	-21199	Alive	TUMOR F	2522							Complete Remission/Response	0	2522	0	2522	0	2522	0	2522
TCGA-HI-	TCGA-HI-	PRAD	56	MALE	WHITE	Prostate Adenocarci	2008	-20776	Dead	WITH TUMOR		1329	[Unknown]					Stable Disease	1	1329	1	1329			1	1329
TCGA-J4-	TCGA-J4-	PRAD	49	MALE		Prostate Adenocarci	2011	-17935	Alive	TUMOR F	614							Complete Remission/Response	0	614	0	614	0	614	0	614
TCGA-J4-	TCGA-J4-	PRAD	47	MALE		Prostate Adenocarci	2011	-17167	Alive	TUMOR F	1218							Complete Remission/Response	0	1218	0	1218	0	1218	0	1218
TCGA-J4-	TCGA-J4-	PRAD	68	MALE		Prostate Adenocarci	2011	-24908	Alive	TUMOR F	1004							Complete Remission/Response	0	1004	0	1004	0	1004	0	1004
TCGA-J4-	TCGA-J4-	PRAD	54	MALE		Prostate Adenocarci	2011	-20034	Alive	TUMOR F	770							Complete Remission/Response	0	770	0	770	0	770	0	770
TCGA-J4-	TCGA-J4-	PRAD	55	MALE		Prostate Adenocarci	2011	-20211	Alive	TUMOR F	941							Complete Remission/Response	0	941	0	941	0	941	0	941
TCGA-J4-	TCGA-J4-	PRAD	61	MALE		Prostate Adenocarci	2011	-22420	Alive	WITH TUN	937			Biochemical evidence of disease			442	Partial Remission/Response	0	937	0	937			1	442
TCGA-J4-	TCGA-J4-	PRAD	59	MALE		Prostate Adenocarci	2012	-21714	Alive	TUMOR F	839							Complete Remission/Response	0	839	0	839	0	839	0	839
TCGA-J4-	TCGA-J4-	PRAD	77	MALE		Prostate Adenocarci	2011	-28447	Alive	TUMOR F	975							Complete Remission/Response	0	975	0	975	0	975	0	975
TCGA-J4-	TCGA-J4-	PRAD	65	MALE		Prostate Adenocarci	2012	-23807	Alive	TUMOR F	910							Complete Remission/Response	0	910	0	910	0	910	0	910
TCGA-J4-	TCGA-J4-	PRAD	63	MALE		Prostate Adenocarci	2012	-23332	Alive	WITH TUN	833			Biochemical evidence of disease			708	Complete Remission/Response	0	833	0	833	1	708	1	708
TCGA-J4-	TCGA-J4-	PRAD	63	MALE		Prostate Adenocarci	2012	-23211	Alive	TUMOR F	183							Complete Remission/Response	0	183	0	183	0	183	0	183
TCGA-J4-	TCGA-J4-	PRAD	68	MALE		Prostate Adenocarci	2012	-24945	Alive	TUMOR F	769							Complete Remission/Response	0	769	0	769	0	769	0	769
TCGA-J4-	TCGA-J4-	PRAD	57	MALE		Prostate Adenocarci	2012	-20836	Alive	WITH TUN	849			Biochemical evidence of disease			618	Partial Remission/Response	0	849	0	849			1	618
TCGA-J4-	TCGA-J4-	PRAD	53	MALE		Prostate Adenocarci	2012	-19640	Alive	TUMOR F	512							Complete Remission/Response	0	512	0	512	0	512	0	512
TCGA-J4-	TCGA-J4-	PRAD	63	MALE		Prostate Adenocarci	2013	-23240	Alive	TUMOR F	685							Complete Remission/Response	0	685	0	685	0	685	0	685
TCGA-J4-	TCGA-J4-	PRAD	68	MALE		Prostate Adenocarci	2013	-24960	Alive	TUMOR F	696							Complete Remission/Response	0	696	0	696	0	696	0	696
TCGA-J4-	TCGA-J4-	PRAD	68	MALE		Prostate Adenocarci	2013	-24960	Alive	TUMOR F	696							Complete Remission/Response	0	696	0	696	0	696	0	696
TCGA-J4-	TCGA-J4-	PRAD	52	MALE		Prostate Adenocarci	2013	-19299	Alive	TUMOR F	328							Complete Remission/Response	0	328	0	328	0	328	0	328
TCGA-J4-	TCGA-J4-	PRAD	61	MALE		Prostate Adenocarci	2013	-22338	Alive	TUMOR F	720							Complete Remission/Response	0	720	0	720	0	720	0	720
TCGA-J4-	TCGA-J4-	PRAD	64	MALE		Prostate Adenocarci	2012	-23624	Alive		543			Biochemical evidence of disease			521	Complete Remission/Response	0	543	0	543	1	521	1	521
TCGA-J4-	TCGA-J4-	PRAD	57	MALE		Prostate Adenocarci	2012	-20841	Alive	TUMOR F	992			New Primary Tumor			433	Complete Remission/Response	0	992	0	992	0	992	1	433
TCGA-J4-	TCGA-J4-	PRAD	71	MALE		Prostate Adenocarci	2013	-25943	Alive	WITH TUN	553							[Discrepancy]	0	553	0	553			0	553
TCGA-J4-	TCGA-J4-	PRAD	66	MALE		Prostate Adenocarci	2013	-24226	Alive	WITH TUN	412			Biochemical evidence of disease			79	Complete Remission/Response	0	412	0	412	1	79	1	79
TCGA-J4-	TCGA-J4-	PRAD	59	MALE		Prostate Adenocarci	2012	-21656	Alive	TUMOR F	825							Complete Remission/Response	0	825	0	825	0	825	0	825
TCGA-J9-	TCGA-J9-	PRAD	62	MALE		Prostate Adenocarci	2012	-22849	Alive		422			Biochemical evidence of disease			57	Complete Remission/Response	0	422	0	422	1	57	1	57
TCGA-J9-	TCGA-J9-	PRAD	57	MALE		Prostate Adenocarci	2012	-20966	Alive		178								0	178	0	178			0	178
TCGA-J9-	TCGA-J9-	PRAD	71	MALE		Prostate Adenocarci	2012	-26023	Alive	TUMOR F	212								0	212	0	212			0	212
TCGA-J9-	TCGA-J9-	PRAD	65	MALE		Prostate Adenocarci	2012	-23741	Alive	TUMOR F	323							Partial Remission/Response	0	323	0	323			0	323
TCGA-J9-	TCGA-J9-	PRAD	66	MALE		Prostate Adenocarci	2012	-24285	Alive	TUMOR F	321								0	321	0	321			0	321
TCGA-J9-	TCGA-J9-	PRAD	66	MALE		Prostate Adenocarci	2013	-24145	Alive	WITH TUN	405			Biochemical evidence of disease			132	[Unknown]	0	405	0	405			1	132
TCGA-J9-	TCGA-J9-	PRAD	66	MALE		Prostate Adenocarci	2013	-24118	Alive	WITH TUN	463			Biochemical evidence of disease			344	Partial Remission/Response	0	463	0	463			1	344
TCGA-J9-	TCGA-J9-	PRAD	53	MALE		Prostate Adenocarci	2011	-19662	Alive	TUMOR F	1237							Complete Remission/Response	0	1237	0	1237	0	1237	0	1237
TCGA-J9-	TCGA-J9-	PRAD	66	MALE		Prostate Adenocarci	2013	-24299	Alive	TUMOR F	386							Complete Remission/Response	0	386	0	386	0	386	0	386
TCGA-KC	TCGA-KC	PRAD	65	MALE		Prostate Adenocarci	2006	-24081	Alive	WITH TUN	934			Biochemical evidence of disease			193	Partial Remission/Response	0	934	0	934			1	193
TCGA-KC	TCGA-KC	PRAD	55	MALE		Prostate Adenocarci	2009	-20392	Alive	TUMOR F	1815							Complete Remission/Response	0	1815	0	1815	0	1815	0	1815
TCGA-KC	TCGA-KC	PRAD	55	MALE		Prostate Adenocarci	2010	-20368	Alive	TUMOR F	607							Complete Remission/Response	0	607	0	607	0	607	0	607
TCGA-KC	TCGA-KC	PRAD	75	MALE		Prostate Adenocarci	2009	-27741	Alive	WITH TUN	1349			Biochemical evidence of disease			1022	Complete Remission/Response	0	1349	0	1349	1	1022	1	1022
TCGA-KC	TCGA-KC	PRAD	66	MALE		Prostate Adenocarci	2010	-24138	Alive	WITH TUN	1328			Biochemical evidence of disease			1328	Complete Remission/Response	0	1328	0	1328	1	1328	1	1328
TCGA-KC	TCGA-KC	PRAD	67	MALE		Prostate Adenocarci	2013	-24676	Alive	TUMOR F	665							Complete Remission/Response	0	665	0	665	0	665	0	665
TCGA-KC	TCGA-KC	PRAD	54	MALE		Prostate Adenocarci	2013	-20015	Alive	TUMOR F	91							Complete Remission/Response	0	91	0	91	0	91	0	91
TCGA-KC	TCGA-KC	PRAD	63	MALE		Prostate Adenocarci	2013	-23367	Alive	TUMOR F	263							Complete Remission/Response	0	263	0	263	0	263	0	263
TCGA-KC	TCGA-KC	PRAD	63	MALE		Prostate Adenocarci	2013	-23208	Alive	TUMOR F	598							Complete Remission/Response	0	598	0	598	0	598	0	598
TCGA-KC	TCGA-KC	PRAD	62	MALE		Prostate Adenocarci	2013	-22745	Alive	TUMOR F	270							Complete Remission/Response	0	270	0	270	0	270	0	270
TCGA-KC	TCGA-KC	PRAD	62	MALE		Prostate Adenocarci	2013	-22718	Alive	TUMOR F	352							Complete Remission/Response	0	352	0	352	0	352	0	352
TCGA-KK	TCGA-KK	PRAD	64	MALE		Prostate Adenocarci	2005	-23483	Alive	TUMOR F	3440							Complete Remission/Response	0	3440	0	3440	0	3440	0	3440
TCGA-KK	TCGA-KK	PRAD	55	MALE		Prostate Adenocarci	2008	-20416	Alive	WITH TUN	2535			Locoregic	Other, sp	Bladder	2233	[Unknown]	0	2535	0	2535			1	2233
TCGA-KK	TCGA-KK	PRAD	56	MALE		Prostate Adenocarci	2009	-20768	Alive	TUMOR F	1968							Complete Remission/Response	0	1968	0	1968	0	1968	0	1968
TCGA-KK	TCGA-KK	PRAD	66	MALE		Prostate Adenocarci	2007	-24306	Alive	TUMOR F	2628								0	2628	0	2628			0	2628
TCGA-KK	TCGA-KK	PRAD	72	MALE		Prostate Adenocarci	2003	-26415	Dead			2469		Locoregional Recurrence			203	Partial Remission/Response	1	2469		2469			1	203
TCGA-KK	TCGA-KK	PRAD	50	MALE		Prostate Adenocarci	2002	-18420	Alive	WITH TUN	4295								0	4295	0	4295			0	4295
TCGA-KK	TCGA-KK	PRAD	59	MALE		Prostate Adenocarci	2011	-21700	Alive	TUMOR F	1483			Biochemical evidence of disease			941	Complete Remission/Response	0	1483	0	1483	1	941	1	941
TCGA-KK	TCGA-KK	PRAD	57	MALE		Prostate Adenocarci	2008	-21063	Alive	TUMOR F	2198							Complete Remission/Response	0	2198	0	2198	0	2198	0	2198
TCGA-KK	TCGA-KK	PRAD	54	MALE		Prostate Adenocarci	2000	-19974	Alive	TUMOR F	5024							Complete Remission/Response	0	5024	0	5024	0	5024	0	5024
TCGA-KK	TCGA-KK	PRAD	56	MALE		Prostate Adenocarci	2004	-20547	Alive	TUMOR F	2056								0	2056	0	2056			0	2056
TCGA-KK	TCGA-KK	PRAD	69	MALE		Prostate Adenocarci	2004	-25338	Dead	TUMOR FREE		3502						Complete Remission/Response	1	3502	0	3502	0	3502	0	3502
TCGA-KK	TCGA-KK	PRAD	63	MALE		Prostate Adenocarci	2009	-23144	Alive	TUMOR F	2073							Complete Remission/Response	0	2073	0	2073	0	2073	0	2073
TCGA-KK	TCGA-KK	PRAD	69	MALE		Prostate Adenocarci	2002	-25320	Alive	TUMOR F	3447								0	3447	0	3447			0	3447
TCGA-KK	TCGA-KK	PRAD	46	MALE		Prostate Adenocarci	2007	-17074	Alive	WITH TUN	2760			Biochemical evidence of disease			925	Partial Remission/Response	0	2760	0	2760			1	925
TCGA-KK	TCGA-KK	PRAD	68	MALE		Prostate Adenocarci	2007	-25026	Alive	TUMOR F	1770								0	1770	0	1770			0	1770
TCGA-KK	TCGA-KK	PRAD	55	MALE		Prostate Adenocarci	2010	-20403	Alive		196							[Unknown]	0	196	0	196			0	196
TCGA-KK	TCGA-KK	PRAD	62	MALE		Prostate Adenocarci	2010	-22731	Alive	TUMOR F	1610			Biochemical evidence of disease			1217	Complete Remission/Response	0	1610	0	1610	1	1217	1	1217
TCGA-KK	TCGA-KK	PRAD	63	MALE		Prostate Adenocarci	2010	-23118	Alive	TUMOR F	1775			Biochemical evidence of disease			207	Progressive Disease	0	1775	0	1775			1	207
TCGA-KK	TCGA-KK	PRAD	58	MALE		Prostate Adenocarci	2012	-21192	Alive	TUMOR F	822							Complete Remission/Response	0	822	0	822	0	822	0	822
TCGA-KK	TCGA-KK	PRAD	57	MALE		Prostate Adenocarci	2010	-20869	Alive	TUMOR F	1054							[Unknown]	0	1054	0	1054			0	1054
TCGA-KK	TCGA-KK	PRAD	60	MALE		Prostate Adenocarci	2010	-22220	Alive	TUMOR F	1754			Biochemical evidence of disease			1124	Complete Remission/Response	0	1754	0	1754	1	1124	1	1124
TCGA-KK	TCGA-KK	PRAD	56	MALE		Prostate Adenocarci	2010	-20507	Alive		1519							[Unknown]	0	1519	0	1519			0	1519
TCGA-KK	TCGA-KK	PRAD	67	MALE		Prostate Adenocarci	2011	-24756	Alive	TUMOR F	1329			Biochemical evidence of disease			606	Stable Disease	0	1329	0	1329			1	606
TCGA-KK	TCGA-KK	PRAD	65	MALE		Prostate Adenocarci	2011	-23803	Alive		1063							Complete Remission/Response	0	1063	0	1063	0	1063	0	1063
TCGA-KK	TCGA-KK	PRAD	68	MALE		Prostate Adenocarci	2012	-24845	Alive	WITH TUN	1099			Biochemical evidence of disease			692	Stable Disease	0	1099	0	1099			1	692
TCGA-KK	TCGA-KK	PRAD	62	MALE		Prostate Adenocarci	2012	-22830	Alive	TUMOR F	899			Biochemical evidence of disease			294	Complete Remission/Response	0	899	0	899	1	294	1	294
TCGA-KK	TCGA-KK	PRAD	64	MALE		Prostate Adenocarci	2012	-23644	Alive	WITH TUN	987			Biochemical evidence of disease			637	Stable Disease	0	987	0	987			1	637
TCGA-KK	TCGA-KK	PRAD	64	MALE		Prostate Adenocarci	2008	-23706	Alive	WITH TUN	2259			Distant M	Bone		1162	Progressive Disease	0	2259	0	2259			1	1162
TCGA-KK	TCGA-KK	PRAD	55	MALE		Prostate Adenocarci	2006	-20337	Alive	TUMOR F	3096							Complete Remission/Response	0	3096	0	3096	0	3096	0	3096
TCGA-KK	TCGA-KK	PRAD	58	MALE		Prostate Adenocarci	2011	-21499	Alive	TUMOR F	668							Complete Remission/Response	0	668	0	668	0	668	0	668
TCGA-KK	TCGA-KK	PRAD	55	MALE		Prostate Adenocarci	2009	-20256	Alive	TUMOR F	2002			Biochemical evidence of disease			1088	Progressive Disease	0	2002	0	2002			1	1088
TCGA-KK	TCGA-KK	PRAD	69	MALE		Prostate Adenocarci	2012	-25546	Alive	TUMOR F	967							Complete Remission/Response	0	967	0	967	0	967	0	967
TCGA-KK	TCGA-KK	PRAD	61	MALE		Prostate Adenocarci	2012	-22295	Alive	WITH TUN	1001			Biochemical evidence of disease			940	Complete Remission/Response	0	1001	0	1001	1	940	1	940
TCGA-KK	TCGA-KK	PRAD	69	MALE		Prostate Adenocarci	2009	-25284	Alive		1931							[Unknown]	0	1931	0	1931			0	1931
TCGA-KK	TCGA-KK	PRAD	65	MALE		Prostate Adenocarci	2012	-24041	Alive		83							[Unknown]	0	83	0	83			0	83
TCGA-KK	TCGA-KK	PRAD	54	MALE		Prostate Adenocarci	2008	-19902	Alive	WITH TUN	2051			Biochemical evidence of disease			1060	Progressive Disease	0	2051	0	2051			1	1060
TCGA-KK	TCGA-KK	PRAD	70	MALE		Prostate Adenocarci	2009	-25851	Alive	TUMOR F	2107							Complete Remission/Response	0	2107	0	2107	0	2107	0	2107
TCGA-KK	TCGA-KK	PRAD	57	MALE		Prostate Adenocarci	2007	-20888	Alive	WITH TUN	804			Biochemical evidence of disease			648	[Unknown]	0	804	0	804			1	648
TCGA-KK	TCGA-KK	PRAD	55	MALE		Prostate Adenocarci	2008	-20266	Alive	TUMOR F	2505							Complete Remission/Response	0	2505	0	2505	0	2505	0	2505
TCGA-KK	TCGA-KK	PRAD	50	MALE		Prostate Adenocarci	2009	-18560	Alive	TUMOR F	2013							Complete Remission/Response	0	2013	0	2013	0	2013	0	2013
TCGA-KK	TCGA-KK	PRAD	61	MALE		Prostate Adenocarci	2004	-22460	Dead	WITH TUMOR		3467	[Unknowr	Biochemical evidence of disease			626	Progressive Disease	1	3467	1	3467			1	626
TCGA-KK	TCGA-KK	PRAD	59	MALE		Prostate Adenocarci	2009	-21793	Alive		1559			New Prim	Other, sp	Melanom	307	[Unknown]	0	1559	0	1559			1	307
TCGA-KK	TCGA-KK	PRAD	56	MALE		Prostate Adenocarci	2008	-20594	Alive	TUMOR F	2052							Complete Remission/Response	0	2052	0	2052	0	2052	0	2052
TCGA-KK	TCGA-KK	PRAD	65	MALE		Prostate Adenocarci	2009	-23849	Dead			628	[Unknown]					[Unknown]	1	628		628			0	628
TCGA-KK	TCGA-KK	PRAD	55	MALE		Prostate Adenocarci	2009	-20425	Alive	TUMOR F	1915							Complete Remission/Response	0	1915	0	1915	0	1915	0	1915
TCGA-M7	TCGA-M7	PRAD	55	MALE		Prostate Adenocarci	2013	-20357	Alive	TUMOR F	493							Complete Remission/Response	0	493	0	493	0	493	0	493
TCGA-M7	TCGA-M7	PRAD	62	MALE		Prostate Adenocarci	2013	-22785	Alive	TUMOR F	643							Complete Remission/Response	0	643	0	643	0	643	0	643
TCGA-M7	TCGA-M7	PRAD	53	MALE		Prostate Adenocarci	2013	-19642	Alive	TUMOR F	384							Complete Remission/Response	0	384	0	384	0	384	0	384
TCGA-M7	TCGA-M7	PRAD	70	MALE		Prostate Adenocarci	2012	-25585	Alive	TUMOR F	400							Complete Remission/Response	0	400	0	400	0	400	0	400
TCGA-M7	TCGA-M7	PRAD	65	MALE		Prostate Adenocarci	2011	-23880	Alive	TUMOR F	1060			Biochemical evidence of disease			559	Partial Remission/Response	0	1060	0	1060			1	559
TCGA-M7	TCGA-M7	PRAD	54	MALE		Prostate Adenocarci	2012	-20064	Alive	TUMOR F	763							Complete Remission/Response	0	763	0	763	0	763	0	763
TCGA-M7	TCGA-M7	PRAD	64	MALE		Prostate Adenocarci	2012	-23595	Alive	WITH TUN	889							Progressive Disease	0	889	0	889			0	889
TCGA-M7	TCGA-M7	PRAD	56	MALE		Prostate Adenocarci	2012	-20474	Alive	TUMOR F	573							Complete Remission/Response	0	573	0	573	0	573	0	573
TCGA-MO	TCGA-MO	PRAD	59	MALE		Prostate Adenocarci	2013	-21846	Alive	TUMOR F	173							Complete Remission/Response	0	173	0	173	0	173	0	173
TCGA-OU	TCGA-OU	PRAD	64	MALE		Prostate Adenocarci	2002	-23710	Alive	TUMOR F	97							Complete Remission/Response	0	97	0	97	0	97	0	97
TCGA-OU	TCGA-OU	PRAD	59	MALE		Prostate Adenocarci	2003	-21792	Alive	TUMOR F	1247							Complete Remission/Response	0	1247	0	1247	0	1247	0	1247
TCGA-OU	TCGA-OU	PRAD	61	MALE		Prostate Adenocarci	2003	-22286	Alive	TUMOR F	4264							Complete Remission/Response	0	4264	0	4264	0	4264	0	4264
TCGA-OU	TCGA-OU	PRAD	53	MALE		Prostate Adenocarci	2003	-19564	Alive	TUMOR F	3716							Complete Remission/Response	0	3716	0	3716	0	3716	0	3716
TCGA-OU	TCGA-OU	PRAD	66	MALE		Prostate Adenocarci	2004	-24212	Alive	TUMOR F	2620							Complete Remission/Response	0	2620	0	2620	0	2620	0	2620
TCGA-SU	TCGA-SU	PRAD	60	MALE		Prostate Adenocarci	2013	-22185	Alive	TUMOR F	551							Complete Remission/Response	0	551	0	551	0	551	0	551
TCGA-TK	TCGA-TK	PRAD	73	MALE		Prostate Adenocarci	2013	-26976	Alive	TUMOR F	27							[Unknown]	0	27	0	27			0	27
TCGA-TP	TCGA-TP	PRAD	64	MALE		Prostate Adenocarci	2013	-23476	Alive		526							Partial Remission/Response	0	526	0	526			0	526
TCGA-TP	TCGA-TP	PRAD	62	MALE		Prostate Adenocarci	2013	-22872	Alive	TUMOR F	610							Complete Remission/Response	0	610	0	610	0	610	0	610
TCGA-V1	TCGA-V1	PRAD	65	MALE		Prostate Adenocarcinoma Acinar Type		-24090	Alive		1449							Complete Remission/Response	0	1449	0	1449	0	1449	0	1449
TCGA-V1	TCGA-V1	PRAD	53	MALE		Prostate Adenocarcinoma Acinar Type		-19710	Alive		857							Complete Remission/Response	0	857	0	857	0	857	0	857
TCGA-V1	TCGA-V1	PRAD	58	MALE		Prostate Adenocarcinoma Acinar Type		-21535	Alive		1791							Progressive Disease	0	1791	0	1791			0	1791
TCGA-V1	TCGA-V1	PRAD	41	MALE		Prostate Adenocarcinoma Acinar Type		-15330	Alive		508							Complete Remission/Response	0	508	0	508	0	508	0	508
TCGA-V1	TCGA-V1	PRAD	63	MALE		Prostate Adenocarcinoma Acinar Type		-23360	Alive		448							Complete Remission/Response	0	448	0	448	0	448	0	448
TCGA-V1	TCGA-V1	PRAD	60	MALE		Prostate Adenocarcinoma Acinar Type		-22265	Alive		1195			Biochemical evidence of disease			990	Complete Remission/Response	0	1195	0	1195	1	990	1	990
TCGA-V1	TCGA-V1	PRAD	56	MALE		Prostate Adenocarcinoma Acinar Type		-20805	Alive		1876							Progressive Disease	0	1876	0	1876			0	1876
TCGA-V1	TCGA-V1	PRAD	64	MALE		Prostate Adenocarcinoma Acinar Type		-23725	Alive		1948							Complete Remission/Response	0	1948	0	1948	0	1948	0	1948
TCGA-V1	TCGA-V1	PRAD	47	MALE		Prostate Adenocarcinoma Acinar Type		-17520	Alive		924							Complete Remission/Response	0	924	0	924	0	924	0	924
TCGA-V1	TCGA-V1	PRAD	56	MALE		Prostate Adenocarcinoma Acinar Type		-20805	Alive		474							Progressive Disease	0	474	0	474			0	474
TCGA-V1	TCGA-V1	PRAD	52	MALE		Prostate Adenocarcinoma Acinar Type		-19345	Alive		686							Complete Remission/Response	0	686	0	686	0	686	0	686
TCGA-V1	TCGA-V1	PRAD	59	MALE		Prostate Adenocarcinoma Acinar Type		-21900	Alive		1078							Complete Remission/Response	0	1078	0	1078	0	1078	0	1078
TCGA-V1	TCGA-V1	PRAD	51	MALE		Prostate Adenocarcinoma Acinar Type		-18980	Alive		202							Complete Remission/Response	0	202	0	202	0	202	0	202
TCGA-V1	TCGA-V1	PRAD	64	MALE		Prostate Adenocarcinoma Acinar Type		-23725	Alive		1932			Biochemical evidence of disease			124	Complete Remission/Response	0	1932	0	1932	1	124	1	124
TCGA-V1	TCGA-V1	PRAD	64	MALE		Prostate Adenocarcinoma Acinar Type		-23725	Alive		1932			Biochemical evidence of disease			124	Complete Remission/Response	0	1932	0	1932	1	124	1	124
TCGA-V1	TCGA-V1	PRAD	60	MALE		Prostate Adenocarcinoma Acinar Type		-22265	Alive		2499			Biochemical evidence of disease			819	Progressive Disease	0	2499	0	2499			1	819
TCGA-V1	TCGA-V1	PRAD	56	MALE		Prostate Adenocarcinoma Acinar Type		-20805	Alive		2870							Complete Remission/Response	0	2870	0	2870	0	2870	0	2870
TCGA-V1	TCGA-V1	PRAD	61	MALE		Prostate Adenocarcinoma Acinar Type		-22630	Alive		636							Complete Remission/Response	0	636	0	636	0	636	0	636
TCGA-V1	TCGA-V1	PRAD	49	MALE		Prostate Adenocarcinoma Acinar Type		-18250	Alive		1184							Complete Remission/Response	0	1184	0	1184	0	1184	0	1184
TCGA-V1	TCGA-V1	PRAD	63	MALE		Prostate Adenocarcinoma Acinar Type		-23360	Alive		2403							Complete Remission/Response	0	2403	0	2403	0	2403	0	2403
TCGA-V1	TCGA-V1	PRAD	65	MALE		Prostate Adenocarcinoma Acinar Type		-24090	Alive		1913			Biochemical evidence of disease			105	Complete Remission/Response	0	1913	0	1913	1	105	1	105
TCGA-V1	TCGA-V1	PRAD	67	MALE		Prostate Adenocarcinoma Acinar Type		-24820	Alive		371							Complete Remission/Response	0	371	0	371	0	371	0	371
TCGA-V1	TCGA-V1	PRAD	61	MALE		Prostate Adenocarcinoma Acinar Type		-22630	Alive		476			Biochemical evidence of disease			292	Progressive Disease	0	476	0	476			1	292
TCGA-V1	TCGA-V1	PRAD	56	MALE		Prostate Adenocarcinoma Acinar Type		-20805	Alive		1134							Partial Remission/Response	0	1134	0	1134			0	1134
TCGA-V1	TCGA-V1	PRAD	57	MALE		Prostate Adenocarcinoma Acinar Type		-21170	Alive		1067							Complete Remission/Response	0	1067	0	1067	0	1067	0	1067
TCGA-V1	TCGA-V1	PRAD	54	MALE		Prostate Adenocarcinoma Acinar Type		-20075	Alive		874							Complete Remission/Response	0	874	0	874	0	874	0	874
TCGA-V1	TCGA-V1	PRAD	59	MALE		Prostate Adenocarcinoma Acinar Type		-21900	Alive	TUMOR F	827							Complete Remission/Response	0	827	0	827	0	827	0	827
TCGA-V1	TCGA-V1	PRAD	63	MALE		Prostate Adenocarcinoma Acinar Type		-23360	Alive	TUMOR F	540							Complete Remission/Response	0	540	0	540	0	540	0	540
TCGA-V1	TCGA-V1	PRAD	64	MALE		Prostate Adenocarcinoma Acinar Type		-23725	Alive	TUMOR F	1517							Complete Remission/Response	0	1517	0	1517	0	1517	0	1517
TCGA-V1	TCGA-V1	PRAD	68	MALE		Prostate Adenocarcinoma Acinar Type		-25185	Alive		1640							Progressive Disease	0	1640	0	1640			0	1640
TCGA-V1	TCGA-V1	PRAD	68	MALE		Prostate Adenocarcinoma Acinar Type		-25185	Alive	TUMOR F	1383							Complete Remission/Response	0	1383	0	1383	0	1383	0	1383
TCGA-V1	TCGA-V1	PRAD	68	MALE		Prostate Adenocarcinoma Acinar Type		-25185	Alive	TUMOR F	1361							Complete Remission/Response	0	1361	0	1361	0	1361	0	1361
TCGA-VN	TCGA-VN	PRAD	59	MALE		Prostate Adenocarci	2011	-21576	Alive	WITH TUN	269							Partial Remission/Response	0	269	0	269			0	269
TCGA-VN	TCGA-VN	PRAD	58	MALE		Prostate Adenocarci	2011	-21524	Alive		776							Complete Remission/Response	0	776	0	776	0	776	0	776
TCGA-VN	TCGA-VN	PRAD	54	MALE		Prostate Adenocarci	2012	-20026	Alive	TUMOR F	746							Complete Remission/Response	0	746	0	746	0	746	0	746
TCGA-VN	TCGA-VN	PRAD	55	MALE		Prostate Adenocarci	2013	-20157	Alive	TUMOR F	189							Complete Remission/Response	0	189	0	189	0	189	0	189
TCGA-VN	TCGA-VN	PRAD	62	MALE		Prostate Adenocarci	2013	-22695	Alive	TUMOR F	766							Complete Remission/Response	0	766	0	766	0	766	0	766
TCGA-VN	TCGA-VN	PRAD	49	MALE		Prostate Adenocarci	2012	-17945	Alive	TUMOR F	481							Complete Remission/Response	0	481	0	481	0	481	0	481
TCGA-VN	TCGA-VN	PRAD	60	MALE		Prostate Adenocarci	2011	-21916	Alive	TUMOR F	1260							Complete Remission/Response	0	1260	0	1260	0	1260	0	1260
TCGA-VN	TCGA-VN	PRAD	60	MALE		Prostate Adenocarci	2010	-22087	Alive	TUMOR F	1366							Complete Remission/Response	0	1366	0	1366	0	1366	0	1366
TCGA-VN	TCGA-VN	PRAD	53	MALE		Prostate Adenocarci	2012	-19513	Alive		784			Biochemical evidence of disease			512	Partial Remission/Response	0	784	0	784			1	512
TCGA-VN	TCGA-VN	PRAD	71	MALE		Prostate Adenocarci	2013	-26162	Alive	TUMOR F	496							Complete Remission/Response	0	496	0	496	0	496	0	496
TCGA-VP	TCGA-VP	PRAD	60	MALE		Prostate Adenocarci	2005	-22098	Alive	TUMOR F	3631							Complete Remission/Response	0	3631	0	3631	0	3631	0	3631
TCGA-VP	TCGA-VP	PRAD	67	MALE		Prostate Adenocarci	2005	-24742	Alive	TUMOR F	2309							Complete Remission/Response	0	2309	0	2309	0	2309	0	2309
TCGA-VP	TCGA-VP	PRAD	46	MALE		Prostate Adenocarci	2005	-16934	Alive	TUMOR F	3333							Complete Remission/Response	0	3333	0	3333	0	3333	0	3333
TCGA-VP	TCGA-VP	PRAD	58	MALE		Prostate Adenocarci	2006	-21402	Alive	WITH TUN	3130			Biochemical evidence of disease			98	Partial Remission/Response	0	3130	0	3130			1	98
TCGA-VP	TCGA-VP	PRAD	70	MALE		Prostate Adenocarci	2006	-25799	Dead	TUMOR FREE		728	[Unknown]					Complete Remission/Response	1	728	0	728	0	728	0	728
TCGA-VP	TCGA-VP	PRAD	63	MALE		Prostate Adenocarci	2007	-23271	Alive	WITH TUN	2722			New Prim	Lung		2473	Complete Remission/Response	0	2722	0	2722	0	2722	1	2473
TCGA-VP	TCGA-VP	PRAD	67	MALE		Prostate Adenocarci	2007	-24608	Alive	TUMOR F	1690							Complete Remission/Response	0	1690	0	1690	0	1690	0	1690
TCGA-VP	TCGA-VP	PRAD	54	MALE		Prostate Adenocarci	2007	-19818	Alive	TUMOR F	1853			Biochemical evidence of disease			1194	Complete Remission/Response	0	1853	0	1853	1	1194	1	1194
TCGA-VP	TCGA-VP	PRAD	59	MALE		Prostate Adenocarci	2008	-21811	Alive	TUMOR F	2037							Complete Remission/Response	0	2037	0	2037	0	2037	0	2037
TCGA-VP	TCGA-VP	PRAD	76	MALE		Prostate Adenocarci	2009	-28060	Alive	TUMOR F	663							Complete Remission/Response	0	663	0	663	0	663	0	663
TCGA-VP	TCGA-VP	PRAD	56	MALE		Prostate Adenocarci	2010	-20792	Alive	TUMOR F	1774							Complete Remission/Response	0	1774	0	1774	0	1774	0	1774
TCGA-VP	TCGA-VP	PRAD	63	MALE		Prostate Adenocarci	2010	-23030	Alive	TUMOR F	1306			Biochemical evidence of disease			533	Complete Remission/Response	0	1306	0	1306	1	533	1	533
TCGA-VP	TCGA-VP	PRAD	70	MALE		Prostate Adenocarci	2008	-25781	Alive	TUMOR F	1365							Complete Remission/Response	0	1365	0	1365	0	1365	0	1365
TCGA-WW	TCGA-WW	PRAD	70	MALE		Prostate Adenocarci	2013	-25593	Alive	TUMOR F	449							Complete Remission/Response	0	449	0	449	0	449	0	449
TCGA-X4	TCGA-X4	PRAD	65	MALE		Prostate Adenocarci	2010	-23999	Alive	WITH TUN	1383							Progressive Disease	0	1383	0	1383			0	1383
TCGA-X4	TCGA-X4	PRAD	61	MALE		Prostate Adenocarci	2011	-22571	Alive	TUMOR F	979							Stable Disease	0	979	0	979			0	979
TCGA-XA	TCGA-XA	PRAD	68	MALE		Prostate Adenocarci	2013	-25090	Alive	TUMOR F	376							Complete Remission/Response	0	376	0	376	0	376	0	376
TCGA-XJ-	TCGA-XJ-	PRAD	67	MALE		Prostate Adenocarci	2011		Alive	TUMOR F	1127							Complete Remission/Response	0	1127	0	1127	0	1127	0	1127
TCGA-XJ-	TCGA-XJ-	PRAD	51	MALE		Prostate Adenocarci	2010		Alive	TUMOR F	1461							Complete Remission/Response	0	1461	0	1461	0	1461	0	1461
TCGA-XJ-	TCGA-XJ-	PRAD	57	MALE		Prostate Adenocarci	2007		Alive		1280							Partial Remission/Response	0	1280	0	1280			0	1280
TCGA-XJ-	TCGA-XJ-	PRAD	62	MALE		Prostate Adenocarci	2009		Alive	TUMOR F	1461							Complete Remission/Response	0	1461	0	1461	0	1461	0	1461
TCGA-XJ-	TCGA-XJ-	PRAD	63	MALE		Prostate Adenocarci	2007		Alive	TUMOR F	275							Complete Remission/Response	0	275	0	275	0	275	0	275
TCGA-XJ-	TCGA-XJ-	PRAD	48	MALE		Prostate Adenocarci	2007		Alive		91							[Unknown]	0	91	0	91			0	91
TCGA-XJ-	TCGA-XJ-	PRAD	51	MALE		Prostate Adenocarci	2012		Alive	TUMOR F	973							Partial Remission/Response	0	973	0	973			0	973
TCGA-XK	TCGA-XK	PRAD	75	MALE		Prostate Adenocarci	2011	-27703	Alive	TUMOR F	972							Complete Remission/Response	0	972	0	972	0	972	0	972
TCGA-XK	TCGA-XK	PRAD	63	MALE		Prostate Adenocarci	2011	-23325	Alive	TUMOR F	1133							Complete Remission/Response	0	1133	0	1133	0	1133	0	1133
TCGA-XK	TCGA-XK	PRAD	78	MALE		Prostate Adenocarci	2011	-28721	Alive	TUMOR F	1218			New Primary Tumor			427	Complete Remission/Response	0	1218	0	1218	0	1218	1	427
TCGA-XK	TCGA-XK	PRAD	56	MALE		Prostate Adenocarci	2010	-20604	Alive	TUMOR F	1262							Complete Remission/Response	0	1262	0	1262	0	1262	0	1262
TCGA-XK	TCGA-XK	PRAD	62	MALE		Prostate Adenocarci	2010	-22771	Alive		930							Complete Remission/Response	0	930	0	930	0	930	0	930
TCGA-XK	TCGA-XK	PRAD	66	MALE		Prostate Adenocarci	2011	-24320	Alive	TUMOR F	1259							Complete Remission/Response	0	1259	0	1259	0	1259	0	1259
TCGA-XK	TCGA-XK	PRAD	61	MALE		Prostate Adenocarci	2011	-22643	Alive	TUMOR F	1124			Biochemical evidence of disease			131	Complete Remission/Response	0	1124	0	1124	1	131	1	131
TCGA-XK	TCGA-XK	PRAD	75	MALE		Prostate Adenocarci	2010	-27428	Alive	TUMOR F	1434							Partial Remission/Response	0	1434	0	1434			0	1434
TCGA-XK	TCGA-XK	PRAD	65	MALE		Prostate Adenocarci	2010	-23958	Alive	TUMOR F	1576							Complete Remission/Response	0	1576	0	1576	0	1576	0	1576
TCGA-XK	TCGA-XK	PRAD	62	MALE		Prostate Adenocarci	2012	-22965	Alive	TUMOR F	979							Complete Remission/Response	0	979	0	979	0	979	0	979
TCGA-XO	TCGA-XO	PRAD	59	MALE		Prostate Adenocarci	2012	-21889	Dead	WITH TUMOR		146	Prostate Cancer					Progressive Disease	1	146	1	146			1	146
TCGA-XO	TCGA-XO	PRAD	67	MALE		Prostate Adenocarci	2012	-24623	Alive	TUMOR F	770							Complete Remission/Response	0	770	0	770	0	770	0	770
TCGA-Y6	TCGA-Y6	PRAD	65	MALE		Prostate Adenocarci	2012	-23776	Alive	TUMOR F	992								0	992	0	992			0	992
TCGA-Y6	TCGA-Y6	PRAD	72	MALE		Prostate Adenocarci	2012	-26471	Alive	TUMOR F	526							Complete Remission/Response	0	526	0	526	0	526	0	526
TCGA-YJ-	TCGA-YJ-	PRAD	67	MALE		Prostate Adenocarci	2008		Alive		148							[Unknown]	0	148	0	148			0	148
TCGA-YL-	TCGA-YL-	PRAD	58	MALE		Prostate Adenocarci	2009	-21416	Alive	TUMOR F	1611							Complete Remission/Response	0	1611	0	1611	0	1611	0	1611
TCGA-YL-	TCGA-YL-	PRAD	59	MALE		Prostate Adenocarci	2009	-21850	Alive	WITH TUN	1491			Biochemical evidence of disease			1376	Complete Remission/Response	0	1491	0	1491	1	1376	1	1376
TCGA-YL-	TCGA-YL-	PRAD	58	MALE		Prostate Adenocarci	2010	-21249	Alive		1565							Stable Disease	0	1565	0	1565			0	1565
TCGA-YL-	TCGA-YL-	PRAD	66	MALE		Prostate Adenocarci	2010	-24121	Alive	WITH TUN	1482			Biochemical evidence of disease			1473	Complete Remission/Response	0	1482	0	1482	1	1473	1	1473
TCGA-YL-	TCGA-YL-	PRAD	67	MALE		Prostate Adenocarci	2008	-24509	Alive	WITH TUN	2304			Biochemical evidence of disease			1068	Stable Disease	0	2304	0	2304			1	1068
TCGA-YL-	TCGA-YL-	PRAD	68	MALE		Prostate Adenocarci	2008	-25119	Alive	WITH TUN	1890			Biochemical evidence of disease			679	Stable Disease	0	1890	0	1890			1	679
TCGA-YL-	TCGA-YL-	PRAD	63	MALE		Prostate Adenocarci	2008	-23204	Alive		1842			Locoregional Recurrence			1006	Stable Disease	0	1842	0	1842			1	1006
TCGA-YL-	TCGA-YL-	PRAD	69	MALE		Prostate Adenocarci	2009	-25340	Alive		512							Stable Disease	0	512	0	512			0	512
TCGA-YL-	TCGA-YL-	PRAD	62	MALE		Prostate Adenocarci	2010	-22991	Alive	WITH TUN	1719			Biochemical evidence of disease			1384	Stable Disease	0	1719	0	1719			1	1384
TCGA-YL-	TCGA-YL-	PRAD	66	MALE		Prostate Adenocarci	2010	-24206	Alive	WITH TUN	1261			Biochemical evidence of disease			152	Partial Remission/Response	0	1261	0	1261			1	152
TCGA-YL-	TCGA-YL-	PRAD	62	MALE		Prostate Adenocarci	2010	-22844	Alive		1373							Stable Disease	0	1373	0	1373			0	1373
TCGA-YL-	TCGA-YL-	PRAD	69	MALE		Prostate Adenocarci	2009	-25395	Dead	TUMOR FREE		875	Other, non-malignant disease					Complete Remission/Response	1	875	0	875	0	875	0	875
TCGA-YL-	TCGA-YL-	PRAD	69	MALE		Prostate Adenocarci	2010	-25292	Alive	TUMOR F	1614			Biochemical evidence of disease			1423	Complete Remission/Response	0	1614	0	1614	1	1423	1	1423
TCGA-YL-	TCGA-YL-	PRAD	60	MALE		Prostate Adenocarci	2010	-21916	Alive	WITH TUN	1395			Biochemical evidence of disease			752	Complete Remission/Response	0	1395	0	1395	1	752	1	752
TCGA-YL-	TCGA-YL-	PRAD	67	MALE		Prostate Adenocarci	2010	-24759	Alive		1408							Stable Disease	0	1408	0	1408			0	1408
TCGA-YL-	TCGA-YL-	PRAD	74	MALE		Prostate Adenocarci	2011	-27033	Alive	TUMOR F	987							Complete Remission/Response	0	987	0	987	0	987	0	987
TCGA-YL-	TCGA-YL-	PRAD	64	MALE		Prostate Adenocarci	2004	-23584	Alive		3479							Stable Disease	0	3479	0	3479			0	3479
TCGA-YL-	TCGA-YL-	PRAD	58	MALE		Prostate Adenocarci	2008	-21455	Alive	WITH TUN	2366			Biochemical evidence of disease			2036	Stable Disease	0	2366	0	2366			1	2036
TCGA-YL-	TCGA-YL-	PRAD	61	MALE		Prostate Adenocarci	2008	-22482	Alive	WITH TUN	2083			Biochemical evidence of disease			329	Stable Disease	0	2083	0	2083			1	329
TCGA-YL-	TCGA-YL-	PRAD	64	MALE		Prostate Adenocarci	2011	-23488	Alive		273							Stable Disease	0	273	0	273			0	273
TCGA-YL-	TCGA-YL-	PRAD	67	MALE		Prostate Adenocarci	2011	-24755	Alive		427							Stable Disease	0	427	0	427			0	427
TCGA-YL-	TCGA-YL-	PRAD	63	MALE		Prostate Adenocarci	2010	-23184	Alive	TUMOR F	1623							Stable Disease	0	1623	0	1623			0	1623
TCGA-YL-	TCGA-YL-	PRAD	47	MALE		Prostate Adenocarci	2008	-17322	Dead	WITH TUMOR		1855	Prostate (	Distant M	Other, sp	Neck	1476	Progressive Disease	1	1855	1	1855			1	1476
TCGA-YL-	TCGA-YL-	PRAD	63	MALE		Prostate Adenocarci	2008	-23272	Alive	WITH TUN	2136			Biochemical evidence of disease			1009	Stable Disease	0	2136	0	2136			1	1009
TCGA-YL-	TCGA-YL-	PRAD	59	MALE		Prostate Adenocarci	2011	-21626	Alive	WITH TUN	740			Biochemical evidence of disease			740	Complete Remission/Response	0	740	0	740	1	740	1	740
TCGA-YL-	TCGA-YL-	PRAD	68	MALE		Prostate Adenocarci	2009	-25008	Alive	WITH TUN	1518			Biochemical evidence of disease			1506	Stable Disease	0	1518	0	1518			1	1506
TCGA-YL-	TCGA-YL-	PRAD	57	MALE		Prostate Adenocarci	2011	-21164	Alive	WITH TUN	1098			Biochemical evidence of disease			765	Stable Disease	0	1098	0	1098			1	765
TCGA-ZG	TCGA-ZG	PRAD	72	MALE		Prostate Adenocarci	2013	-26632	Alive	TUMOR F	94							Progressive Disease	0	94	0	94			0	94
TCGA-ZG	TCGA-ZG	PRAD	56	MALE		Prostate Adenocarci	2013	-20683	Alive	TUMOR F	442							Stable Disease	0	442	0	442			0	442
TCGA-ZG	TCGA-ZG	PRAD	67	MALE		Prostate Adenocarci	2013	-24731	Alive	WITH TUN	404							Stable Disease	0	404	0	404			0	404
TCGA-ZG	TCGA-ZG	PRAD	65	MALE		Prostate Adenocarci	2013	-23980	Alive	TUMOR F	308							Stable Disease	0	308	0	308			0	308
TCGA-ZG	TCGA-ZG	PRAD	73	MALE		Prostate Adenocarci	2013	-27000	Alive		130							Stable Disease	0	130	0	130			0	130
TCGA-ZG	TCGA-ZG	PRAD	71	MALE		Prostate Adenocarci	2013	-26055	Alive	TUMOR F	355							Stable Disease	0	355	0	355			0	355
TCGA-ZG	TCGA-ZG	PRAD	66	MALE		Prostate Adenocarci	2010	-24214	Alive	WITH TUN	1302							Partial Remission/Response	0	1302	0	1302			0	1302
TCGA-ZG	TCGA-ZG	PRAD	70	MALE		Prostate Adenocarci	2011	-25573	Alive	WITH TUN	1154			Biochemical evidence of disease			180	Stable Disease	0	1154	0	1154			1	180
TCGA-ZG	TCGA-ZG	PRAD	61	MALE		Prostate Adenocarci	2011	-22388	Alive		1108							Stable Disease	0	1108	0	1108			0	1108
TCGA-ZG	TCGA-ZG	PRAD	58	MALE		Prostate Adenocarci	2011	-21293	Alive	WITH TUN	908							Progressive Disease	0	908	0	908			0	908

TCGA-ZG	TCGA-ZG	PRAD	64	MALE			Prostate Adenocarci	2011	-23681	Alive	WITH TUN	687		Biochemical evidence of disease			664	Partial Remission/Response		0	687	0	687			1	664
TCGA-ZG	TCGA-ZG	PRAD	60	MALE			Prostate Adenocarci	2012	-22108	Alive	WITH TUN	617		Biochemical evidence of disease			51	Progressive Disease		0	617	0	617			1	51
TCGA-ZG	TCGA-ZG	PRAD	72	MALE			Prostate Adenocarci	2012	-26447	Alive	WITH TUN	583						Partial Remission/Response		0	583	0	583			0	583
TCGA-ZG	TCGA-ZG	PRAD	72	MALE			Prostate Adenocarci	2013	-26377	Alive	TUMOR F	378						Complete Remission/Response		0	378	0	378	0	378	0	378
TCGA-ZG	TCGA-ZG	PRAD	57	MALE			Prostate Adenocarci	2013	-21050	Alive	WITH TUN	115						Partial Remission/Response		0	115	0	115			0	115
TCGA-ZG	TCGA-ZG	PRAD	64	MALE			Prostate Adenocarci	2013	-23540	Alive	TUMOR F	516						Complete Remission/Response		0	516	0	516	0	516	0	516
TCGA-ZG	TCGA-ZG	PRAD	67	MALE			Prostate Adenocarci	2012	-24742	Alive	WITH TUN	579						Partial Remission/Response		0	579	0	579			0	579
TCGA-ZG	TCGA-ZG	PRAD	60	MALE			Prostate Adenocarci	2012	-21942	Alive	TUMOR F	370						Complete Remission/Response		0	370	0	370	0	370	0	370
TCGA-ZG	TCGA-ZG	PRAD	66	MALE			Prostate Adenocarci	2011	-24132	Alive	WITH TUN	692						Partial Remission/Response		0	692	0	692			0	692
TCGA-ZG	TCGA-ZG	PRAD	65	MALE			Prostate Adenocarci	2012	-24030	Alive	WITH TUN	547						Partial Remission/Response		0	547	0	547			0	547
TCGA-ZG	TCGA-ZG	PRAD	69	MALE			Prostate Adenocarci	2012	-25477	Alive	WITH TUN	455						Partial Remission/Response		0	455	0	455			0	455
TCGA-ZG	TCGA-ZG	PRAD	73	MALE			Prostate Adenocarci	2013	-26902	Alive	WITH TUN	349						Partial Remission/Response		0	349	0	349			0	349
TCGA-ZG	TCGA-ZG	PRAD	55	MALE			Prostate Adenocarci	2013	-20428	Alive	TUMOR F	410						Complete Remission/Response		0	410	0	410	0	410	0	410
TCGA-ZG	TCGA-ZG	PRAD	73	MALE			Prostate Adenocarci	2013	-26697	Alive	TUMOR F	132						Complete Remission/Response		0	132	0	132	0	132	0	132
TCGA-AF	TCGA-AF	READ	57	MALE	WHITE	Stage IIIC	Rectal Adenocarcino	2009	-21098	Alive	TUMOR F	1427					1267	Complete Remission/Response		0	1427	0	1427	1	1267	1	1267
TCGA-AF	TCGA-AF	READ	41	FEMALE	WHITE	Stage IV	Rectal Adenocarcino	2009	-15082	Dead	WITH TUMOR		1201				301			1	1201	1	1201			1	301
TCGA-AF	TCGA-AF	READ	41	FEMALE	WHITE	Stage IV	Rectal Adenocarcino	2009	-15082	Dead	WITH TUMOR		1201				301			1	1201	1	1201			1	301
TCGA-AF	TCGA-AF	READ	76	FEMALE	WHITE	Stage IIIC	Rectal Adenocarcino	2009	-28119	Dead	TUMOR FREE		524							1	524	0	524			0	524
TCGA-AF	TCGA-AF	READ	48	FEMALE	WHITE	Stage I	Rectal Adenocarcino	2009	-17759	Alive	TUMOR F	1309						Complete Remission/Response		0	1309	0	1309	0	1309	0	1309
TCGA-AF	TCGA-AF	READ	48	FEMALE	WHITE	Stage I	Rectal Adenocarcino	2009	-17759	Alive	TUMOR F	1309						Complete Remission/Response		0	1309	0	1309	0	1309	0	1309
TCGA-AF	TCGA-AF	READ	54	FEMALE	WHITE	Stage IIA		2009	-19830	Alive	WITH TUN	412		Metastatic			327			0	412	0	412			1	327
TCGA-AF	TCGA-AF	READ	54	FEMALE	WHITE	Stage IIA		2009	-19830	Alive	WITH TUN	412		Metastatic			327			0	412	0	412			1	327
TCGA-AF	TCGA-AF	READ	75	MALE	WHITE	Stage I		2009	-27443	Alive	TUMOR F	1155							0	1155	0	1155			0	1155
TCGA-AF	TCGA-AF	READ	54	MALE	WHITE	Stage IIA	Rectal Mucinous Ade	2009	-19826	Alive	TUMOR F	1049							0	1049	0	1049			0	1049
TCGA-AF	TCGA-AF	READ	54	MALE	WHITE	Stage IIA	Rectal Mucinous Ade	2009	-19826	Alive	TUMOR F	1049							0	1049	0	1049			0	1049
TCGA-AF	TCGA-AF	READ	48	MALE	WHITE	Stage IIIC	Rectal Adenocarcino	2009	-17609	Alive	WITH TUN	1148					695	Complete Remission/Response		0	1148	0	1148	1	695	1	695
TCGA-AF	TCGA-AF	READ	60	MALE	BLACK OI	Stage IV	Rectal Adenocarcino	2009	-21970	Dead	WITH TUMOR		316							1	316	1	316			1	316
TCGA-AF	TCGA-AF	READ	60	MALE	BLACK OI	Stage IV	Rectal Adenocarcino	2009	-21970	Dead	WITH TUMOR		316							1	316	1	316			1	316
TCGA-AF	TCGA-AF	READ	77	MALE	WHITE	Stage IVA		2010	-28289	Alive	TUMOR F	912						Complete Remission/Response		0	912	0	912			0	912
TCGA-AF	TCGA-AF	READ	73	FEMALE	WHITE	Stage I	Rectal Adenocarcino	2010	-26738	Dead	TUMOR FREE		512							1	512	0	512			0	512
TCGA-AF	TCGA-AF	READ	73	FEMALE	WHITE	Stage I	Rectal Adenocarcino	2010	-26738	Dead	TUMOR FREE		512							1	512	0	512			0	512
TCGA-AF	TCGA-AF	READ	72	FEMALE	WHITE	Stage IIIB	Rectal Adenocarcino	2011	-26490	Alive	WITH TUN	741					199	Progressive Disease		0	741	0	741			1	199
TCGA-AF	TCGA-AF	READ	66	MALE	WHITE	Stage IIIA	Rectal Adenocarcino	2010	-24417	Alive	TUMOR F	609						Complete Remission/Response		0	609	0	609	0	609	0	609
TCGA-AF	TCGA-AF	READ	43	MALE	WHITE	Stage IV	Rectal Adenocarcino	2011	-15965	Alive	WITH TUN	748		Metastatic			279	Progressive Disease		0	748	0	748			1	279
TCGA-AF	TCGA-AF	READ	56	MALE	BLACK OI	Stage IIA	Rectal Adenocarcino	2006	-20527	Alive	TUMOR F	2635		Locoregional Disease			1217	Complete Remission/Response		0	2635	0	2635	1	1217	1	1217
TCGA-AF	TCGA-AF	READ	48	FEMALE	BLACK OI	Stage IIIC	Rectal Adenocarcino	2008	-17734	Alive	TUMOR F	2007						Complete Remission/Response		0	2007	0	2007	0	2007	0	2007
TCGA-AF	TCGA-AF	READ	47	FEMALE	BLACK OI	Stage IIA	Rectal Adenocarcino	2012	-17480	Alive	TUMOR F	360						Complete Remission/Response		0	360	0	360	0	360	0	360
TCGA-AG	TCGA-AG	READ	89	FEMALE		Stage II	Rectal Adenocarcino	2005	-32781	Dead	WITH TUMOR		1096							1	1096	1	1096			1	1096
TCGA-AG	TCGA-AG	READ	51	MALE		Stage II	Rectal Adenocarcino	2006	-18932	Alive	TUMOR F	365							0	365	0	365			0	365
TCGA-AG	TCGA-AG	READ	76	FEMALE		Stage IIA	Rectal Mucinous Ade	2007	-27851	Alive	TUMOR F	974							0	974	0	974			0	974
TCGA-AG	TCGA-AG	READ	67	MALE		Stage I	Rectal Adenocarcino	2007	-24775	Alive	TUMOR F	244							0	244	0	244			0	244
TCGA-AG	TCGA-AG	READ	63	MALE		Stage I	Rectal Adenocarcino	2007	-23345	Alive	TUMOR F	215							0	215	0	215			0	215
TCGA-AG	TCGA-AG	READ	75	MALE		Stage IV	Rectal Adenocarcino	2007	-27636	Dead	WITH TUMOR		1096							1	1096	1	1096			1	1096
TCGA-AG	TCGA-AG	READ	66	MALE		Stage IV	Rectal Mucinous Ade	2008	-24107	Dead	WITH TUMOR		610							1	610	1	610			1	610
TCGA-AG	TCGA-AG	READ	60	MALE		Stage IV	Rectal Adenocarcino	2008	-22189	Dead	WITH TUMOR		730						Progressive Disease		1	730	1	730			1	730
TCGA-AG	TCGA-AG	READ	72	FEMALE		Stage III		2006	-26542	Alive	WITH TUN	31							0	31	0	31			0	31
TCGA-AG	TCGA-AG	READ	65	MALE		Stage I	Rectal Adenocarcino	2007	-24075	Alive	TUMOR F	1400					1216	[Unknown]		0	1400	0	1400			1	1216
TCGA-AG	TCGA-AG	READ	66	FEMALE		Stage IIA	Rectal Adenocarcino	2007	-24167	Alive	TUMOR F	1035						Complete Remission/Response		0	1035	0	1035	0	1035	0	1035
TCGA-AG	TCGA-AG	READ	68	MALE		Stage IIA	Rectal Adenocarcino	2007	-25141	Alive	TUMOR F	1035							0	1035	0	1035			0	1035
TCGA-AG	TCGA-AG	READ	72	FEMALE		Stage IIA	Rectal Adenocarcino	2007	-26480	Alive	TUMOR F	1035						Complete Remission/Response		0	1035	0	1035	0	1035	0	1035
TCGA-AG	TCGA-AG	READ	77	MALE		Stage IIA	Rectal Mucinous Ade	2007	-28277	Dead	WITH TUMOR		61							1	61	1	61			1	61
TCGA-AG	TCGA-AG	READ	69	MALE		Stage IIA	Rectal Adenocarcino	2007	-25324	Alive	TUMOR F	1522							0	1522	0	1522			0	1522
TCGA-AG	TCGA-AG	READ	70	MALE		Stage I		2007	-25749	Alive	TUMOR F	366							0	366	0	366			0	366
TCGA-AG	TCGA-AG	READ	62	MALE		Stage IIIC	Rectal Adenocarcino	2007	-22767	Alive	TUMOR F	184						Complete Remission/Response		0	184	0	184	0	184	0	184
TCGA-AG	TCGA-AG	READ	68	MALE		Stage IIIB	Rectal Adenocarcino	2007	-24837	Alive	WITH TUN	0							0	0	0	0			0	0
TCGA-AG	TCGA-AG	READ	66	FEMALE		Stage IIIC	Rectal Adenocarcino	2007	-24350	Alive	WITH TUN	0							0	0	0	0			0	0
TCGA-AG	TCGA-AG	READ	85	FEMALE		Stage IV	Rectal Adenocarcino	2008	-31078	Alive	WITH TUN	30							0	30	0	30			0	30
TCGA-AG	TCGA-AG	READ	79	FEMALE		Stage IIA	Rectal Adenocarcino	2008	-29008	Alive	TUMOR F	485							0	485	0	485			0	485
TCGA-AG	TCGA-AG	READ	65	FEMALE		Stage IIIC	Rectal Adenocarcino	2008	-23925	Alive	TUMOR F	608						Complete Remission/Response		0	608	0	608	0	608	0	608
TCGA-AG	TCGA-AG	READ	72	MALE		Stage IIA	Rectal Adenocarcino	2009	-26420	Alive	TUMOR F	424						Complete Remission/Response		0	424	0	424	0	424	0	424
TCGA-AG	TCGA-AG	READ	55	FEMALE		Stage IIIB	Rectal Adenocarcino	2009	-20242	Alive	TUMOR F	608						Complete Remission/Response		0	608	0	608	0	608	0	608
TCGA-AG	TCGA-AG	READ	90	FEMALE		Stage III	Rectal Adenocarcino	2005	-32872	Alive	TUMOR F	638							0	638	0	638			0	638
TCGA-AG	TCGA-AG	READ	90	FEMALE		Stage III	Rectal Adenocarcino	2005	-32872	Alive	TUMOR F	638							0	638	0	638			0	638
TCGA-AG	TCGA-AG	READ	63	FEMALE		Stage I	Rectal Adenocarcino	2006	-23345	Alive	TUMOR F	243							0	243	0	243			0	243
TCGA-AG	TCGA-AG	READ	78	FEMALE		Stage III	Rectal Adenocarcino	2006	-28641	Alive	WITH TUN	30							0	30	0	30			0	30
TCGA-AG	TCGA-AG	READ	73	MALE		Stage IIIB	Rectal Adenocarcino	2006	-26906	Alive	WITH TUN	912						Complete Remission/Response		0	912	0	912	0	912	0	912
TCGA-AG	TCGA-AG	READ	65	MALE		Stage IV	Rectal Adenocarcino	2004	-23923	Alive	TUMOR F	1126						Complete Remission/Response		0	1126	0	1126			0	1126
TCGA-AG	TCGA-AG	READ	65	MALE		Stage IV	Rectal Adenocarcino	2004	-23923	Alive	TUMOR F	1126						Complete Remission/Response		0	1126	0	1126			0	1126
TCGA-AG	TCGA-AG	READ	78	FEMALE		Stage I	Rectal Adenocarcino	2005	-28521	Alive	TUMOR F	1003						Complete Remission/Response		0	1003	0	1003	0	1003	0	1003
TCGA-AG	TCGA-AG	READ	78	FEMALE		Stage I	Rectal Adenocarcino	2005	-28521	Alive	TUMOR F	1003						Complete Remission/Response		0	1003	0	1003	0	1003	0	1003
TCGA-AG	TCGA-AG	READ	85	FEMALE		Stage I	Rectal Adenocarcino	2004	-31078	Alive	WITH TUN	30							0	30	0	30			0	30
TCGA-AG	TCGA-AG	READ	85	FEMALE		Stage I	Rectal Adenocarcino	2004	-31078	Alive	WITH TUN	30							0	30	0	30			0	30
TCGA-AG	TCGA-AG	READ	64	MALE		Stage I	Rectal Adenocarcino	2007	-23527	Alive	WITH TUN	30							0	30	0	30			0	30
TCGA-AG	TCGA-AG	READ	83	FEMALE		Stage IIA		2008	-30467	Alive	TUMOR F	579							0	579	0	579			0	579
TCGA-AG	TCGA-AG	READ	66	FEMALE		Stage I	Rectal Adenocarcino	2008	-24198	Alive	TUMOR F	608							0	608	0	608			0	608
TCGA-AG	TCGA-AG	READ	69	MALE		Stage I	Rectal Adenocarcino	2008	-25415	Alive	WITH TUN	31							0	31	0	31			0	31
TCGA-AG	TCGA-AG	READ	71	FEMALE		Stage IIIB	Rectal Adenocarcino	2008	-26145	Alive	TUMOR F	546						Complete Remission/Response		0	546	0	546	0	546	0	546
TCGA-AG	TCGA-AG	READ	68	MALE		Stage IIA	Rectal Mucinous Ade	2009	-24960	Alive	WITH TUN	1124					1064			0	1124	0	1124			1	1064
TCGA-AG	TCGA-AG	READ	62	MALE		Stage I	Rectal Adenocarcino	2009	-22950	Alive	TUMOR F	518							0	518	0	518			0	518
TCGA-AG	TCGA-AG	READ	57	FEMALE		Stage I	Rectal Adenocarcino	2009	-20880	Alive	TUMOR F	396						Complete Remission/Response		0	396	0	396	0	396	0	396
TCGA-AG	TCGA-AG	READ	74	MALE		Stage IIIB	Rectal Adenocarcino	2009	-27272	Alive	TUMOR F	1065						Complete Remission/Response		0	1065	0	1065	0	1065	0	1065
TCGA-AG	TCGA-AG	READ	65	MALE		Stage IIA	Rectal Adenocarcino	2009	-23954	Alive	TUMOR F	426						Complete Remission/Response		0	426	0	426	0	426	0	426
TCGA-AG	TCGA-AG	READ	85	FEMALE		Stage I	Rectal Adenocarcino	2009	-31046	Alive	WITH TUN	31							0	31	0	31			0	31
TCGA-AG	TCGA-AG	READ	61	MALE		Stage IIA	Rectal Adenocarcino	2006	-22492	Alive	WITH TUN	1461							0	1461	0	1461			0	1461
TCGA-AG	TCGA-AG	READ	67	FEMALE		Stage IIIB	Rectal Mucinous Ade	2007	-24502	Alive	WITH TUN	761							0	761	0	761			0	761
TCGA-AG	TCGA-AG	READ	61	MALE		Stage IIA	Rectal Adenocarcino	2007	-22492	Alive	TUMOR F	974							0	974	0	974			0	974
TCGA-AG	TCGA-AG	READ	69	FEMALE		Stage IIIB	Rectal Adenocarcino	2008	-25537	Alive	TUMOR F	608						Complete Remission/Response		0	608	0	608	0	608	0	608
TCGA-AG	TCGA-AG	READ	61	FEMALE		Stage IIIC	Rectal Adenocarcino	2008	-22311	Alive	TUMOR F	853					791	Progressive Disease		0	853	0	853			1	791
TCGA-AG	TCGA-AG	READ	74	FEMALE		Stage IIA	Rectal Adenocarcino	2009	-27302	Alive	TUMOR F	1096							0	1096	0	1096			0	1096
TCGA-AG	TCGA-AG	READ	64	MALE		Stage IV	Rectal Adenocarcino	2007	-23407	Alive	TUMOR F	427						Complete Remission/Response		0	427	0	427			0	427
TCGA-AG	TCGA-AG	READ	87	MALE		Stage IV	Rectal Adenocarcino	2007	-31776	Alive	WITH TUN	31							0	31	0	31			0	31
TCGA-AG	TCGA-AG	READ	63	MALE		Stage IIA	Rectal Adenocarcino	2008	-23192	Alive	TUMOR F	518						Complete Remission/Response		0	518	0	518	0	518	0	518
TCGA-AG	TCGA-AG	READ	85	FEMALE		Stage IIA	Rectal Adenocarcino	2009	-31291	Alive	WITH TUN	0							0	0	0	0			0	0
TCGA-AG	TCGA-AG	READ	84	FEMALE		Stage IV	Rectal Adenocarcino	2005	-31015	Dead	WITH TUMOR		121				0			1	121	1	121			1	0
TCGA-AG	TCGA-AG	READ	59	FEMALE		Stage II	Rectal Adenocarcino	2006	-21823	Alive	TUMOR F	1400						Complete Remission/Response		0	1400	0	1400	0	1400	0	1400
TCGA-AG	TCGA-AG	READ	35	MALE		Stage I	Rectal Adenocarcino	2009	-13090	Alive	TUMOR F	638						Complete Remission/Response		0	638	0	638	0	638	0	638
TCGA-AG	TCGA-AG	READ	50	FEMALE		Stage I	Rectal Mucinous Ade	2009	-18416	Alive	TUMOR F	424						Complete Remission/Response		0	424	0	424	0	424	0	424
TCGA-AG	TCGA-AG	READ	49	FEMALE		Stage IIIB	Rectal Adenocarcino	2007	-18201	Alive	TUMOR F	183						Complete Remission/Response		0	183	0	183	0	183	0	183
TCGA-AG	TCGA-AG	READ	75	MALE		Stage IIA	Rectal Adenocarcino	2008	-27485	Alive	WITH TUN	790					790	Progressive Disease		0	790	0	790			1	790
TCGA-AG	TCGA-AG	READ	68	MALE		Stage IIA	Rectal Adenocarcino	2008	-24837	Alive	TUMOR F	700					123	Complete Remission/Response		0	700	0	700	1	123	1	123
TCGA-AG	TCGA-AG	READ	80	MALE		Stage IIA	Rectal Adenocarcino	2009	-29463	Alive	TUMOR F	1126							0	1126	0	1126			0	1126
TCGA-AG	TCGA-AG	READ	86	MALE		Stage I	Rectal Adenocarcino	2009	-31718	Alive	WITH TUN	485							0	485	0	485			0	485
TCGA-AG	TCGA-AG	READ	64	FEMALE		Stage I	Rectal Adenocarcino	2009	-23496	Alive	TUMOR F	1096					518			0	1096	0	1096			1	518
TCGA-AG	TCGA-AG	READ	55	MALE		Stage IV	Rectal Adenocarcino	2009	-20178	Alive	WITH TUN	276						Progressive Disease		0	276	0	276			0	276
TCGA-AG	TCGA-AG	READ	59	FEMALE		Stage IIA	Rectal Adenocarcino	2009	-21581	Alive	WITH TUN	31							0	31	0	31			0	31
TCGA-AG	TCGA-AG	READ	58	MALE		Stage IIIB	Rectal Adenocarcino	2009	-21369	Alive	WITH TUN	0							0	0	0	0			0	0
TCGA-AG	TCGA-AG	READ	68	FEMALE		Stage IV	Rectal Adenocarcino	2009	-25049	Alive	WITH TUN	943						Partial Remission/Response		0	943	0	943			0	943
TCGA-AG	TCGA-AG	READ	67	FEMALE		Stage II	Rectal Adenocarcino	2004	-24745	Alive	WITH TUN	0							0	0	0	0			0	0
TCGA-AG	TCGA-AG	READ	67	FEMALE		Stage II	Rectal Adenocarcino	2004	-24745	Alive	WITH TUN	0							0	0	0	0			0	0
TCGA-AG	TCGA-AG	READ	49	FEMALE		Stage II	Rectal Adenocarcino	2004	-18112	Alive	WITH TUN	0							0	0	0	0			0	0
TCGA-AG	TCGA-AG	READ	49	FEMALE		Stage II	Rectal Adenocarcino	2004	-18112	Alive	WITH TUN	0							0	0	0	0			0	0
TCGA-AG	TCGA-AG	READ	57	FEMALE		Stage III	Rectal Mucinous Ade	2005	-20851	Alive	WITH TUN	31							0	31	0	31			0	31
TCGA-AG	TCGA-AG	READ	57	FEMALE		Stage III	Rectal Mucinous Ade	2005	-20851	Alive	WITH TUN	31							0	31	0	31			0	31
TCGA-AG	TCGA-AG	READ	62	FEMALE		Stage IV	Rectal Adenocarcino	2005	-22830	Dead	WITH TUMOR		1581				822			1	1581	1	1581			1	822
TCGA-AG	TCGA-AG	READ	62	FEMALE		Stage I	Rectal Adenocarcino	2006	-22952	Alive	WITH TUN	1520							0	1520	0	1520			0	1520
TCGA-AG	TCGA-AG	READ	66	MALE		Stage II	Rectal Adenocarcino	2006	-24413	Dead	WITH TUMOR		59							1	59	1	59			1	59
TCGA-AG	TCGA-AG	READ	66	MALE		Stage IV	Rectal Adenocarcino	2007	-24259	Dead	WITH TUMOR		1185				578	Partial Remission/Response		1	1185	1	1185			1	578
TCGA-AG	TCGA-AG	READ	67	MALE		Stage II	Rectal Adenocarcino	2005	-24687	Alive	WITH TUN	1885							0	1885	0	1885			0	1885
TCGA-AG	TCGA-AG	READ	67	MALE		Stage II	Rectal Adenocarcino	2005	-24687	Alive	WITH TUN	1885							0	1885	0	1885			0	1885
TCGA-AG	TCGA-AG	READ	77	MALE		Stage I	Rectal Adenocarcino	2007	-28399	Alive	WITH TUN	1247							0	1247	0	1247			0	1247
TCGA-AG	TCGA-AG	READ	68	MALE		Stage IIIB	Rectal Adenocarcino	2007	-24865	Alive	WITH TUN	1157					792	Partial Remission/Response		0	1157	0	1157			1	792
TCGA-AG	TCGA-AG	READ	71	MALE		Stage III	Rectal Adenocarcino	2002	-26206	Alive	TUMOR F	3562						Complete Remission/Response		0	3562	0	3562	0	3562	0	3562
TCGA-AG	TCGA-AG	READ	71	MALE		Stage III	Rectal Adenocarcino	2002	-26206	Alive	TUMOR F	3562						Complete Remission/Response		0	3562	0	3562	0	3562	0	3562
TCGA-AH	TCGA-AH	READ	60	MALE	WHITE		Rectal Adenocarcino	2010	-21977	Alive	WITH TUN	1173						Stable Disease		0	1173	0	1173			0	1173
TCGA-AH	TCGA-AH	READ	79	FEMALE	WHITE		Rectal Adenocarcino	2010	-29030	Dead	TUMOR FREE		76					Complete Remission/Response		1	76	0	76	0	76	0	76
TCGA-AH	TCGA-AH	READ	66	MALE	WHITE		Rectal Adenocarcino	2010	-24337	Alive	TUMOR F	532						Complete Remission/Response		0	532	0	532	0	532	0	532
TCGA-AH	TCGA-AH	READ	50	MALE	WHITE	Stage IIIC	Rectal Adenocarcino	2010	-18273	Dead	WITH TUMOR		1314				1026	Complete Remission/Response		1	1314	1	1314	1	1026	1	1026
TCGA-AH	TCGA-AH	READ	50	MALE	WHITE	Stage IIIC	Rectal Adenocarcino	2010	-18273	Dead	WITH TUMOR		1314				1026	Complete Remission/Response		1	1314	1	1314	1	1026	1	1026
TCGA-AH	TCGA-AH	READ	73	MALE	WHITE		Rectal Adenocarcino	2010	-26892	Alive	TUMOR F	838						Complete Remission/Response		0	838	0	838	0	838	0	838
TCGA-AH	TCGA-AH	READ	48	MALE	WHITE	Stage I	Rectal Adenocarcino	2010	-17536	Alive	TUMOR F	804						Complete Remission/Response		0	804	0	804	0	804	0	804
TCGA-AH	TCGA-AH	READ	46	MALE	WHITE	Stage IIIB	Rectal Mucinous Ade	2010	-17030	Alive	TUMOR F	592						Complete Remission/Response		0	592	0	592	0	592	0	592
TCGA-BM	TCGA-BM	READ	73	MALE	WHITE	Stage IIIB	Rectal Adenocarcino	2010	-26837	Alive	TUMOR F	646						Complete Remission/Response		0	646	0	646	0	646	0	646
TCGA-CI-	TCGA-CI-	READ	41	MALE	WHITE	Stage IV	Rectal Adenocarcino	2010	-15322	Alive		184							0	184	0	184			0	184
TCGA-CI-	TCGA-CI-	READ	41	FEMALE	WHITE	Stage IVA	Rectal Adenocarcino	2010	-15322	Alive	WITH TUN	1009					90	Progressive Disease		0	1009	0	1009			1	90
TCGA-CI-	TCGA-CI-	READ	63	MALE	WHITE	Stage IIIB	Rectal Adenocarcino	2010	-23240	Alive		419						[Unknown]		0	419	0	419			0	419
TCGA-CI-	TCGA-CI-	READ	74	MALE	WHITE	Stage IIB	Rectal Adenocarcino	2010	-27336	Alive	TUMOR F	1362						[Unknown]		0	1362	0	1362			0	1362
TCGA-CI-	TCGA-CI-	READ	44	MALE	WHITE	Stage I	Rectal Adenocarcino	2011	-16177	Alive	TUMOR F	1443						[Unknown]		0	1443	0	1443			0	1443
TCGA-CI-	TCGA-CI-	READ	53	FEMALE	WHITE	Stage I	Rectal Adenocarcino	2011	-19419	Alive		1466						[Unknown]		0	1466	0	1466			0	1466
TCGA-CL	TCGA-CL	READ	79	FEMALE		[Discrepancy]	Rectal Adenocarcino	2008	-29081	Alive		425						Complete Remission/Response		0	425	0	425	0	425	0	425
TCGA-CL	TCGA-CL	READ	71	FEMALE	WHITE	Stage IIIC	Rectal Adenocarcino	2006	-26084	Alive	TUMOR F	2376						Complete Remission/Response		0	2376	0	2376	0	2376	0	2376
TCGA-CL	TCGA-CL	READ	90	FEMALE	WHITE	Stage IIA	Rectal Adenocarcino	2008	-32873	Alive	TUMOR F	218						Complete Remission/Response		0	218	0	218	0	218	0	218
TCGA-DC	TCGA-DC	READ	49	FEMALE	WHITE	Stage IIIB	Rectal Adenocarcino	2010	-17958	Alive	TUMOR F	639						Complete Remission/Response		0	639	0	639	0	639	0	639
TCGA-DC	TCGA-DC	READ	57	MALE	WHITE	Stage I	Rectal Adenocarcino	2010	-21092	Alive	TUMOR F	762						Complete Remission/Response		0	762	0	762	0	762	0	762
TCGA-DC	TCGA-DC	READ	69	MALE	WHITE	Stage I	Rectal Adenocarcino	2010	-25202	Alive	TUMOR F	792						[Unknown]		0	792	0	792			0	792
TCGA-DC	TCGA-DC	READ	62	FEMALE	BLACK OI	Stage IIIB	Rectal Adenocarcino	2010	-22676	Alive	TUMOR F	943							0	943	0	943			0	943
TCGA-DC	TCGA-DC	READ	57	FEMALE	WHITE	Stage IVA	Rectal Adenocarcino	2010	-21093	Alive	TUMOR F	365							0	365	0	365			0	365
TCGA-DC	TCGA-DC	READ	31	FEMALE	WHITE	Stage IIIA	Rectal Adenocarcino	2010	-11381	Alive	TUMOR F	425							0	425	0	425			0	425
TCGA-DC	TCGA-DC	READ	62	MALE	WHITE	Stage IVA	Rectal Adenocarcino	2010	-22706	Alive	WITH TUN	943					30			0	943	0	943			1	30
TCGA-DC	TCGA-DC	READ	48	MALE	BLACK OI	Stage I	Rectal Adenocarcino	2009	-17807	Alive	TUMOR F	1581							0	1581	0	1581			0	1581
TCGA-DC	TCGA-DC	READ	70	MALE	WHITE	Stage I	Rectal Adenocarcino	2010	-25842	Dead	TUMOR FREE		334							1	334	0	334			0	334
TCGA-DC	TCGA-DC	READ	68	MALE	WHITE	Stage I	Rectal Adenocarcino	2009	-24990	Alive	TUMOR F	1339							0	1339	0	1339			0	1339
TCGA-DC	TCGA-DC	READ	70	FEMALE	WHITE	Stage IVA	Rectal Adenocarcino	2011	-25659	Alive	TUMOR F	790							0	790	0	790			0	790
TCGA-DC	TCGA-DC	READ	57	MALE	WHITE	Stage IIA	Rectal Adenocarcino	2011	-21062	Alive	TUMOR F	762							0	762	0	762			0	762
TCGA-DC	TCGA-DC	READ	43	MALE	WHITE	Stage IIIB	Rectal Adenocarcino	2011	-15918	Alive	TUMOR F	762							0	762	0	762			0	762
TCGA-DT	TCGA-DT	READ	51	MALE	ASIAN	Stage II	Rectal Mucinous Ade	2010	-18669	Alive	TUMOR F	384						Complete Remission/Response		0	384	0	384	0	384	0	384
TCGA-DY	TCGA-DY	READ	57	FEMALE	WHITE	Stage IIA	Rectal Adenocarcino	2001	-21115	Alive		3846							0	3846	0	3846			0	3846
TCGA-DY	TCGA-DY	READ	72	FEMALE	WHITE	Stage IIA	Rectal Adenocarcino	2000	-26436	Dead			1258	Locoregional Disease			630			1	1258		1258			1	630
TCGA-DY	TCGA-DY	READ	77	FEMALE	WHITE	Stage IIIB	Rectal Adenocarcino	1999	-28159	Dead		1741	1741							1	1741		1741			0	1741
TCGA-DY	TCGA-DY	READ	56	FEMALE	WHITE	Stage IIA	Rectal Adenocarcino	2001	-20504	Alive		3932					3316			0	3932	0	3932			1	3316
TCGA-DY	TCGA-DY	READ	73	FEMALE	WHITE	Stage IIIC	Rectal Adenocarcino	2001	-26763	Dead		734	734							1	734		734			0	734
TCGA-DY	TCGA-DY	READ	75	MALE	WHITE	Stage IVA	Rectal Adenocarcino	2000	-27396	Dead			1566				592			1	1566		1566			1	592
TCGA-DY	TCGA-DY	READ	77	FEMALE	WHITE	Stage IIIA	Rectal Adenocarcino	2000	-28459	Dead		992	992							1	992		992			0	992
TCGA-EF-	TCGA-EF-	READ	54	MALE	WHITE	Stage IIB	Rectal Adenocarcino	2010	-19802	Alive	TUMOR F	106							0	106	0	106			0	106
TCGA-EF-	TCGA-EF-	READ	72	MALE	WHITE	Stage IIA	Rectal Adenocarcino	2010	-26331	Alive	TUMOR F	127							0	127	0	127			0	127
TCGA-EI-	TCGA-EI-	READ	78	FEMALE	WHITE	Stage IIA	Rectal Adenocarcino	2010	-28587	Alive	TUMOR F	625							0	625	0	625			0	625
TCGA-EI-	TCGA-EI-	READ	60	MALE	WHITE	Stage IIA	Rectal Adenocarcino	2010	-22176	Alive	TUMOR F	607							0	607	0	607			0	607
TCGA-EI-	TCGA-EI-	READ	48	FEMALE	WHITE	Stage IIIB	Rectal Adenocarcino	2010	-17827	Alive	TUMOR F	636							0	636	0	636			0	636
TCGA-EI-	TCGA-EI-	READ	53	MALE	WHITE	Stage IIIC	Rectal Adenocarcino	2010	-19666	Alive	WITH TUN	517		Metastatic			90			0	517	0	517			1	90
TCGA-EI-	TCGA-EI-	READ	77	FEMALE	WHITE	[Discrepancy]	Rectal Adenocarcino	2010	-28328	Alive	TUMOR F	556							0	556	0	556			0	556
TCGA-EI-	TCGA-EI-	READ	52	MALE	WHITE	Stage IIIB	Rectal Adenocarcino	2010	-19237	Alive	TUMOR F	482							0	482	0	482			0	482
TCGA-EI-	TCGA-EI-	READ	64	FEMALE	WHITE	Stage IIIB	Rectal Adenocarcino	2010	-23537	Alive	TUMOR F	538							0	538	0	538			0	538
TCGA-EI-	TCGA-EI-	READ	59	MALE	WHITE	Stage IIIB	Rectal Adenocarcino	2010	-21776	Alive	TUMOR F	497							0	497	0	497			0	497
TCGA-EI-	TCGA-EI-	READ	59	FEMALE	WHITE	Stage IIA	Rectal Adenocarcino	2010	-21618	Alive	TUMOR F	496							0	496	0	496			0	496
TCGA-EI-	TCGA-EI-	READ	60	MALE	WHITE	Stage IIIA	Rectal Adenocarcino	2010	-22057	Alive	TUMOR F	499							0	499	0	499			0	499
TCGA-EI-	TCGA-EI-	READ	59	MALE	WHITE	Stage IIA	Rectal Mucinous Ade	2011	-21682	Alive	TUMOR F	262							0	262	0	262			0	262
TCGA-EI-	TCGA-EI-	READ	63	MALE	WHITE	Stage IIC	Rectal Adenocarcino	2011	-23268	Alive	TUMOR F	350							0	350	0	350			0	350
TCGA-EI-	TCGA-EI-	READ	71	MALE	WHITE	Stage IIIA	Rectal Adenocarcino	2011	-25995	Alive	TUMOR F	328							0	328	0	328			0	328
TCGA-EI-	TCGA-EI-	READ	57	FEMALE	WHITE	Stage IV	Rectal Adenocarcino	2011	-21140	Alive	WITH TUN	415		Locoregional Disease			295			0	415	0	415			1	295
TCGA-EI-	TCGA-EI-	READ	33	MALE	WHITE	Stage IIIA	Rectal Adenocarcino	2010	-12113	Alive	TUMOR F	531							0	531	0	531			0	531
TCGA-EI-	TCGA-EI-	READ	58	MALE	WHITE	Stage IV	Rectal Adenocarcino	2011	-21192	Alive	TUMOR F	364							0	364	0	364			0	364
TCGA-EI-	TCGA-EI-	READ	37	FEMALE	WHITE	[Discrepancy]	Rectal Mucinous Ade	2011	-13566	Alive	TUMOR F	257							0	257	0	257			0	257
TCGA-F5·	TCGA-F5·	READ	77	FEMALE	WHITE	Stage IIIC	Rectal Adenocarcino	2011	-28168	Dead			303							1	303		303			0	303
TCGA-F5·	TCGA-F5·	READ	64	FEMALE	WHITE	Stage IIA	Rectal Adenocarcino	2010	-23432	Alive	TUMOR F	1506						Complete Remission/Response		0	1506	0	1506	0	1506	0	1506
TCGA-F5·	TCGA-F5·	READ	62	FEMALE	WHITE	Stage IIA	Rectal Adenocarcino	2011	-22666	Alive	WITH TUN	1288		Metastatic			122	Complete Remission/Response		0	1288	0	1288	1	122	1	122
TCGA-F5·	TCGA-F5·	READ	71	MALE	WHITE	Stage IVA	Rectal Adenocarcino	2010	-26123	Alive	WITH TUN	452					368	Progressive Disease		0	452	0	452			1	368
TCGA-F5·	TCGA-F5·	READ	72	FEMALE	WHITE	Stage IIA	Rectal Adenocarcino	2011	-26575	Alive	TUMOR F	979						Complete Remission/Response		0	979	0	979	0	979	0	979
TCGA-F5·	TCGA-F5·	READ	67	MALE	WHITE	Stage IIA	Rectal Adenocarcino	2011	-24490	Alive	TUMOR F	1110						Complete Remission/Response		0	1110	0	1110	0	1110	0	1110
TCGA-F5·	TCGA-F5·	READ	70	FEMALE	WHITE	Stage IIIC	Rectal Adenocarcino	2011	-25651	Dead	WITH TUMOR		598				65	[Unknown]		1	598	1	598			1	65
TCGA-F5·	TCGA-F5·	READ	57	MALE	WHITE	Stage IIA	Rectal Adenocarcino	2011	-20828	Alive	TUMOR F	1131						Complete Remission/Response		0	1131	0	1131	0	1131	0	1131
TCGA-F5·	TCGA-F5·	READ	60	FEMALE	WHITE	Stage IIA	Rectal Adenocarcino	2011	-21936	Alive	TUMOR F	1160						Complete Remission/Response		0	1160	0	1160	0	1160	0	1160
TCGA-F5·	TCGA-F5·	READ	71	FEMALE	WHITE	Stage IIIB	Rectal Adenocarcino	2011	-25936	Dead	WITH TUMOR		361				191	[Unknown]		1	361	1	361			1	191
TCGA-F5·	TCGA-F5·	READ	74	FEMALE	WHITE	Stage IIIB	Rectal Adenocarcino	2011	-27058	Alive	TUMOR F	379						Complete Remission/Response		0	379	0	379	0	379	0	379
TCGA-G5	TCGA-G5	READ	74	MALE	WHITE	[Discrepancy]	Rectal Adenocarcino	2009	-27190	Dead	WITH TUMOR		556				92			1	556	1	556			1	92
TCGA-G5	TCGA-G5	READ	72	MALE	WHITE	Stage IIIB	Rectal Adenocarcino	2007	-26348	Alive	TUMOR F	1696					1042			0	1696	0	1696			1	1042
TCGA-G5	TCGA-G5	READ	56	MALE	WHITE		Rectal Adenocarcino	2004	-20475	Dead	TUMOR FREE		1432							1	1432	0	1432			0	1432
TCGA-G5	TCGA-G5	READ	56	MALE	WHITE		Rectal Adenocarcino	2004	-20475	Dead	TUMOR FREE		1432							1	1432	0	1432			0	1432
TCGA-G5	TCGA-G5	READ	67	MALE	WHITE	Stage IIIA	Rectal Mucinous Ade	2009	-24486	Alive	TUMOR F	804							0	804	0	804			0	804
TCGA-3B·	TCGA-3B·	SARC	68	MALE	WHITE		Dedifferentiated lipo	2009	-24984	Alive	WITH TUN	1521		Locoregional Recurrence			606	Positive	R1	0	1521	0	1521			1	606
TCGA-3B·	TCGA-3B·	SARC	68	MALE	WHITE		Dedifferentiated lipo	2011	-24962	Alive	TUMOR F	1104						[Unknowr	RX	0	1104	0	1104			0	1104
TCGA-3B·	TCGA-3B·	SARC	67	MALE	WHITE		Dedifferentiated lipo	2011	-24628	Dead	TUMOR FREE		599	Locoregic	Other, sp	Retroperit	229	Positive	R1	1	599	0	599			1	229
TCGA-3B·	TCGA-3B·	SARC	75	MALE	WHITE		Dedifferentiated lipo	2012	-27664	Alive	TUMOR F	959		New Prim	Other, sp	Abdomina	595	Positive	R1	0	959	0	959			1	595
TCGA-3B·	TCGA-3B·	SARC	57	FEMALE	WHITE		Leiomyosarcoma (LM	2007	-21094	Dead	WITH TUMOR		1627	Distant M	Lung		517	Positive	R1	1	1627	1	1627			1	517
TCGA-3B·	TCGA-3B·	SARC	66	FEMALE	WHITE		Leiomyosarcoma (LM	2007	-24386	Alive		2085						Negative	R0	0	2085	0	2085	0	2085	0	2085
TCGA-3B·	TCGA-3B·	SARC	38	FEMALE	BLACK OR AFRICAN AMERICAN		Leiomyosarcoma (LM	2007	-14041	Dead	WITH TUMOR		2694	Distant M	Lung		496	Negative	R0	1	2694	1	2694	1	496	1	496
TCGA-3B·	TCGA-3B·	SARC	60	MALE	WHITE		Leiomyosarcoma (LM	2008	-21946	Dead	WITH TUMOR		1366	Locoregional Recurrence			139	Negative	R0	1	1366	1	1366	1	139	1	139
TCGA-3B·	TCGA-3B·	SARC	53	MALE	WHITE		Leiomyosarcoma (LM	2010	-19637	Alive	TUMOR F	1665		New Prim	Liver		670	Negative	R0	0	1665	0	1665	0	1665	1	670
TCGA-3B·	TCGA-3B·	SARC	53	FEMALE	BLACK OR AFRICAN AMERICAN		Leiomyosarcoma (LM	2010	-19669	Alive	WITH TUN	768		Distant M	Lung		631	Negative	R0	0	768	0	768	1	631	1	631
TCGA-3B·	TCGA-3B·	SARC	48	FEMALE	WHITE		Leiomyosarcoma (LM	2010	-17638	Dead	WITH TUMOR		437	Distant M	Lung		41	Negative	RX	1	437	1	437			1	41
TCGA-3B·	TCGA-3B·	SARC	70	FEMALE	WHITE		Leiomyosarcoma (LM	2011	-25627	Alive	TUMOR F	1303						Negative	R0	0	1303	0	1303	0	1303	0	1303
TCGA-3B·	TCGA-3B·	SARC	49	MALE	WHITE		Leiomyosarcoma (LM	2011	-18109	Alive	TUMOR F	314						Negative	R0	0	314	0	314	0	314	0	314
TCGA-3B·	TCGA-3B·	SARC	66	MALE	WHITE		Leiomyosarcoma (LM	2011	-24433	Alive	WITH TUN	1170		Distant M	Lung		309	Positive	R1	0	1170	0	1170			1	309
TCGA-3B·	TCGA-3B·	SARC	33	MALE	WHITE		Leiomyosarcoma (LM	2011	-12148	Alive	WITH TUN	1096		Distant M	Liver		503	Negative	R0	0	1096	0	1096	1	503	1	503
TCGA-3B·	TCGA-3B·	SARC	56	FEMALE	BLACK OR AFRICAN AMERICAN		Leiomyosarcoma (LM	2012	-20629	Dead	WITH TUMOR		567	Distant M	Other, sp	Pelvis	192	Negative	RX	1	567	1	567			1	192
TCGA-3B·	TCGA-3B·	SARC	62	MALE	WHITE		Leiomyosarcoma (LM	2012	-22985	Alive	TUMOR F	808		New Prim	Other, sp	Axillary Ly	87	Positive	R1	0	808	0	808			1	87
TCGA-3R·	TCGA-3R·	SARC	66	MALE	[Not Evaluated]		Dedifferentiated lipo	2013	-24412	Dead	WITH TUMOR		17					Positive	R2	1	17	1	17			1	17
TCGA-DX	TCGA-DX	SARC	82	MALE	WHITE		Dedifferentiated lipo	2010	-30054	Dead	WITH TUMOR		427	Distant M	Other, sp	Abdomen	94		R2	1	427	1	427			1	94
TCGA-DX	TCGA-DX	SARC	67	MALE	WHITE		Dedifferentiated lipo	2009	-24647	Dead			995							R1	1	995		995			0	995
TCGA-DX	TCGA-DX	SARC	67	FEMALE	WHITE		Dedifferentiated lipo	2009	-24784	Alive	TUMOR F	2087							R0	0	2087	0	2087	0	2087	0	2087
TCGA-DX	TCGA-DX	SARC	66	MALE	WHITE		Dedifferentiated lipo	2009	-24281	Dead	WITH TUMOR		391	Distant Metastasis			205		R1	1	391	1	391			1	205
TCGA-DX	TCGA-DX	SARC	66	MALE	WHITE		Dedifferentiated lipo	2009	-24362	Alive	WITH TUN	1372		Locoregic	Other, specify		1294		R1	0	1372	0	1372			1	1294
TCGA-DX	TCGA-DX	SARC	58	MALE	WHITE		Dedifferentiated lipo	2008	-21434	Dead	WITH TUMOR		564	Locoregional Recurrence			103		R1	1	564	1	564			1	103
TCGA-DX	TCGA-DX	SARC	60	MALE	WHITE		Dedifferentiated lipo	2008	-22218	Dead	WITH TUMOR		1649	Locoregional Recurrence			349		R0	1	1649	1	1649	1	349	1	349
TCGA-DX	TCGA-DX	SARC	78	MALE	WHITE		Dedifferentiated lipo	2008	-28692	Alive	WITH TUN	2586		Locoregional Recurrence			1480		R0	0	2586	0	2586	1	1480	1	1480
TCGA-DX	TCGA-DX	SARC	60	FEMALE	WHITE		Dedifferentiated lipo	2009	-22240	Dead	WITH TUMOR		1020	Locoregional Recurrence			437		R1	1	1020	1	1020			1	437
TCGA-DX	TCGA-DX	SARC	60	MALE	WHITE		Dedifferentiated lipo	2009	-22195	Alive	WITH TUN	2036		Locoregional Recurrence			340		R0	0	2036	0	2036	1	340	1	340
TCGA-DX	TCGA-DX	SARC	55	MALE	WHITE		Dedifferentiated lipo	2002	-20442	Alive	TUMOR F	2271							R1	0	2271	0	2271			0	2271
TCGA-DX	TCGA-DX	SARC	40	FEMALE	WHITE		Dedifferentiated lipo	2006	-14892	Dead	WITH TUMOR		160	Locoregional Recurrence			104		R1	1	160	1	160			1	104
TCGA-DX	TCGA-DX	SARC	81	MALE	WHITE		Dedifferentiated lipo	2003	-29905	Dead	WITH TUMOR		2324	Locoregional Recurrence			873		R1	1	2324	1	2324			1	873
TCGA-DX	TCGA-DX	SARC	57	FEMALE	WHITE		Dedifferentiated lipo	2003	-21155	Dead	WITH TUMOR		2034	Locoregional Recurrence			378		R1	1	2034	1	2034			1	378
TCGA-DX	TCGA-DX	SARC	80	MALE	WHITE		Dedifferentiated lipo	2006	-29272	Dead	WITH TUMOR		191	Locoregional Recurrence			172		R1	1	191	1	191			1	172
TCGA-DX	TCGA-DX	SARC	74	MALE	WHITE		Dedifferentiated lipo	2006	-27357	Dead	WITH TUMOR		846	Locoregional Recurrence			751		R1	1	846	1	846			1	751
TCGA-DX	TCGA-DX	SARC	51	MALE	WHITE		Dedifferentiated lipo	2006	-18721	Alive	TUMOR F	1834							R0	0	1834	0	1834	0	1834	0	1834
TCGA-DX	TCGA-DX	SARC	60	MALE	WHITE		Dedifferentiated lipo	2007	-22245	Alive	TUMOR F	261						Negative	R0	0	261	0	261	0	261	0	261
TCGA-DX	TCGA-DX	SARC	61	MALE	WHITE		Dedifferentiated lipo	2005	-22369	Dead	WITH TUMOR		295	Locoregional Recurrence			213		R1	1	295	1	295			1	213
TCGA-DX	TCGA-DX	SARC	53	MALE	WHITE		Dedifferentiated lipo	2007	-19707	Alive	TUMOR F	2901						Positive	R1	0	2901	0	2901			0	2901
TCGA-DX	TCGA-DX	SARC	60	MALE	WHITE		Dedifferentiated lipo	2008	-22131	Alive	TUMOR F	2408							R0	0	2408	0	2408	0	2408	0	2408
TCGA-DX	TCGA-DX	SARC	78	FEMALE	WHITE		Dedifferentiated lipo	2011	-28649	Dead	WITH TUMOR		275	Distant M	Lung		114		R1	1	275	1	275			1	114
TCGA-DX	TCGA-DX	SARC	62	MALE	WHITE		Dedifferentiated lipo	2011	-22837	Alive	WITH TUN	1492		Locoregional Recurrence			568	Negative	R0	0	1492	0	1492	1	568	1	568
TCGA-DX	TCGA-DX	SARC	60	MALE	WHITE		Dedifferentiated lipo	2007	-21915	Alive	TUMOR F	2641		Locoregic	Other, sp	Retroperit	290	Positive	R1	0	2641	0	2641			1	290
TCGA-DX	TCGA-DX	SARC	43	MALE	WHITE		Dedifferentiated lipo	2004	-15969	Alive	WITH TUN	4150		Locoregional Recurrence			1125	Positive	R1	0	4150	0	4150			1	1125
TCGA-DX	TCGA-DX	SARC	66	MALE	WHITE		Dedifferentiated lipo	2011	-24166	Alive	TUMOR F	1017						Negative	R0	0	1017	0	1017	0	1017	0	1017
TCGA-DX	TCGA-DX	SARC	79	MALE	WHITE		Dedifferentiated lipo	2010	-28932	Alive	WITH TUN	1359		Locoregic	Other, sp	Retroperit	1212	Positive	R2	0	1359	0	1359			1	1212
TCGA-DX	TCGA-DX	SARC	59	MALE	WHITE		Dedifferentiated lipo	2011	-21689	Alive	WITH TUN	1055						Positive	R1	0	1055	0	1055			0	1055
TCGA-DX	TCGA-DX	SARC	52	MALE	WHITE		Dedifferentiated lipo	2011	-19088	Alive	TUMOR F	969						Positive	R1	0	969	0	969			0	969
TCGA-DX	TCGA-DX	SARC	63	FEMALE	WHITE		Dedifferentiated lipo	2011	-23193	Dead	WITH TUMOR		1088	Locoregional Recurrence			160	Negative	R0	1	1088	1	1088	1	160	1	160
TCGA-DX	TCGA-DX	SARC	67	MALE	WHITE		Leiomyosarcoma (LM	2009	-24723	Dead	WITH TUMOR		1552	Distant M	Lung		349	Negative	R0	1	1552	1	1552	1	349	1	349
TCGA-DX	TCGA-DX	SARC	43	MALE	[Unknown]		Leiomyosarcoma (LM	2010	-15751	Dead	WITH TUMOR		138	Distant M	Lung		128		R0	1	138	1	138	1	128	1	128
TCGA-DX	TCGA-DX	SARC	84	MALE	WHITE		Leiomyosarcoma (LM	2005	-31010	Dead	WITH TUMOR		456	Locoregional Recurrence			203		R1	1	456	1	456			1	203
TCGA-DX	TCGA-DX	SARC	47	FEMALE	WHITE		Leiomyosarcoma (LM	2009	-17342	Dead	[Discrepancy]		325							R0	1	325		325	0	325	0	325
TCGA-DX	TCGA-DX	SARC	54	MALE	WHITE		Leiomyosarcoma (LM	2009	-19862	Alive	TUMOR F	2247						Negative	R0	0	2247	0	2247	0	2247	0	2247
TCGA-DX	TCGA-DX	SARC	37	FEMALE	WHITE		Leiomyosarcoma (LM	2009	-13745	Alive	WITH TUN	2085		Distant M	Lung		176	Negative	R0	0	2085	0	2085	1	176	1	176
TCGA-DX	TCGA-DX	SARC	56	MALE	WHITE		Leiomyosarcoma (LM	2010	-20703	Alive	WITH TUN	1534		Locoregional Recurrence			603	Negative	R0	0	1534	0	1534	1	603	1	603
TCGA-DX	TCGA-DX	SARC	66	FEMALE	WHITE		Leiomyosarcoma (LM	2007	-24388	Dead	WITH TUMOR		1061	Distant Metastasis			224		R0	1	1061	1	1061	1	224	1	224
TCGA-DX	TCGA-DX	SARC	65	FEMALE	WHITE		Leiomyosarcoma (LM	2007	-23993	Dead	WITH TUMOR		2599	Locoregional Recurrence			964	Positive	R1	1	2599	1	2599			1	964
TCGA-DX	TCGA-DX	SARC	44	MALE	BLACK OR AFRICAN AMERICAN		Leiomyosarcoma (LM	2003	-16279	Dead	WITH TUMOR		296	Locoregional Recurrence			97		R0	1	296	1	296	1	97	1	97
TCGA-DX	TCGA-DX	SARC	65	MALE	WHITE		Leiomyosarcoma (LM	2011	-23756	Alive	TUMOR F	1399						Negative	R0	0	1399	0	1399	0	1399	0	1399
TCGA-DX	TCGA-DX	SARC	49	FEMALE	WHITE		Leiomyosarcoma (LM	2010	-18113	Dead	WITH TUMOR		738	Distant M	Lung		316		R0	1	738	1	738	1	316	1	316
TCGA-DX	TCGA-DX	SARC	47	MALE	WHITE		Dedifferentiated lipo	2012	-17364	Alive	TUMOR F	1073						Positive	R1	0	1073	0	1073			0	1073
TCGA-DX	TCGA-DX	SARC	63	FEMALE	WHITE		Leiomyosarcoma (LM	2010	-23364	Alive	TUMOR F	1605						Negative	R0	0	1605	0	1605	0	1605	0	1605
TCGA-DX	TCGA-DX	SARC	54	FEMALE	WHITE		Leiomyosarcoma (LM	2011	-19794	Alive	TUMOR F	1400						Negative	R0	0	1400	0	1400	0	1400	0	1400
TCGA-DX	TCGA-DX	SARC	41	MALE	WHITE		Leiomyosarcoma (LM	2002	-15107	Alive	WITH TUN	805		Locoregional Recurrence			315	Positive	R1	0	805	0	805			1	315
TCGA-DX	TCGA-DX	SARC	57	MALE	WHITE		Leiomyosarcoma (LM	2005	-20853	Alive	TUMOR F	3310						Negative	R0	0	3310	0	3310	0	3310	0	3310
TCGA-DX	TCGA-DX	SARC	78	MALE	WHITE		Leiomyosarcoma (LM	2010	-28754	Alive	TUMOR F	1820		Distant M	Other, sp	Flank	1407	Negative	R0	0	1820	0	1820	1	1407	1	1407
TCGA-DX	TCGA-DX	SARC	55	MALE	WHITE		Leiomyosarcoma (LM	2012	-20365	Alive	TUMOR F	1143						Negative	R0	0	1143	0	1143	0	1143	0	1143
TCGA-DX	TCGA-DX	SARC	80	MALE	WHITE		Leiomyosarcoma (LM	2002	-29513	Dead	WITH TUMOR		485	Distant M	Lung		42	Positive	R0	1	485	1	485	1	42	1	42
TCGA-DX	TCGA-DX	SARC	45	FEMALE	BLACK OR AFRICAN AMERICAN		Leiomyosarcoma (LM	2012	-16561	Alive	TUMOR F	1138						Negative	R0	0	1138	0	1138	0	1138	0	1138
TCGA-DX	TCGA-DX	SARC	53	FEMALE	WHITE		Leiomyosarcoma (LM	2008	-19455	Alive	WITH TUN	2515		Locoregional Recurrence			408	Negative	R0	0	2515	0	2515	1	408	1	408
TCGA-DX	TCGA-DX	SARC	74	MALE	WHITE		Leiomyosarcoma (LM	2012	-27150	Alive	WITH TUN	1105						Positive	R1	0	1105	0	1105			0	1105
TCGA-DX	TCGA-DX	SARC	77	MALE	WHITE		Dedifferentiated lipo	2011	-28368	Alive	TUMOR F	881						Negative	R0	0	881	0	881	0	881	0	881
TCGA-DX	TCGA-DX	SARC	82	FEMALE	WHITE		Dedifferentiated lipo	2012	-30071	Alive	WITH TUN	972		Locoregic	Other, specify		947	Positive	R1	0	972	0	972			1	947
TCGA-DX	TCGA-DX	SARC	51	MALE	WHITE		Dedifferentiated lipo	2012	-18682	Alive	WITH TUN	1121		Locoregional Recurrence			149	Negative	R0	0	1121	0	1121	1	149	1	149
TCGA-DX	TCGA-DX	SARC	34	MALE	WHITE		Dedifferentiated lipo	2012	-12742	Alive	WITH TUN	821		Locoregional Recurrence			232	Positive	R1	0	821	0	821			1	232
TCGA-DX	TCGA-DX	SARC	85	FEMALE	WHITE		Dedifferentiated lipo	2009	-31159	Dead	TUMOR FREE		492					Positive	R1	1	492	0	492			0	492
TCGA-DX	TCGA-DX	SARC	86	FEMALE	[Unknown]		Myxofibrosarcoma	2011	-31421	Dead	TUMOR FREE		93					Negative	R0	1	93	0	93	0	93	0	93
TCGA-DX	TCGA-DX	SARC	75	MALE	WHITE		Myxofibrosarcoma	2011	-27434	Alive	TUMOR F	1247						Negative	R0	0	1247	0	1247	0	1247	0	1247
TCGA-DX	TCGA-DX	SARC	61	MALE	WHITE		Myxofibrosarcoma	2008	-22310	Alive	TUMOR F	2381						Negative	R0	0	2381	0	2381	0	2381	0	2381
TCGA-DX	TCGA-DX	SARC	31	FEMALE	WHITE		Myxofibrosarcoma	2008	-11633	Alive	TUMOR F	2044						Negative	R0	0	2044	0	2044	0	2044	0	2044
TCGA-DX	TCGA-DX	SARC	50	FEMALE	WHITE		Myxofibrosarcoma	2012	-18351	Alive	TUMOR F	1045						Negative	R0	0	1045	0	1045	0	1045	0	1045
TCGA-DX	TCGA-DX	SARC	74	MALE	WHITE		Myxofibrosarcoma	2008	-27053	Dead	WITH TUMOR		1536	Distant M	Other, sp	Groin - lv	516	Negative	R0	1	1536	1	1536	1	516	1	516
TCGA-DX	TCGA-DX	SARC	68	FEMALE	[Unknown]		Myxofibrosarcoma	2005	-24871	Dead	WITH TUMOR		513	Distant M	Lung		200	Negative	R0	1	513	1	513	1	200	1	200
TCGA-DX	TCGA-DX	SARC	59	MALE	WHITE		Myxofibrosarcoma	2007	-21886	Alive	TUMOR F	2625						Negative	R0	0	2625	0	2625	0	2625	0	2625
TCGA-DX	TCGA-DX	SARC	78	FEMALE	WHITE		Myxofibrosarcoma	2006	-28796	Dead	TUMOR FREE		1953					Positive	R1	1	1953	0	1953			0	1953
TCGA-DX	TCGA-DX	SARC	82	FEMALE	WHITE		Leiomyosarcoma (LM	2007	-30042	Alive	WITH TUN	3079		Distant M	Lung		2659	Positive	R0	0	3079	0	3079	1	2659	1	2659
TCGA-DX	TCGA-DX	SARC	88	FEMALE	WHITE		Pleomorphic 'MFH' /	2005	-32302	Dead	WITH TUMOR		648	Distant M	Lung		294	Positive	R0	1	648	1	648	1	294	1	294
TCGA-DX	TCGA-DX	SARC	78	FEMALE	WHITE		Dedifferentiated lipo	2003	-28610	Dead	WITH TUMOR		1049	Locoregic	Other, sp	Retroperit	828	Positive	R1	1	1049	1	1049			1	828
TCGA-DX	TCGA-DX	SARC	56	FEMALE	WHITE		Leiomyosarcoma (LM	2007	-20767	Alive	TUMOR F	2291		Distant M	Lung		2249	Positive	R0	0	2291	0	2291	1	2249	1	2249
TCGA-DX	TCGA-DX	SARC	61	MALE	WHITE		Leiomyosarcoma (LM	2010	-22531	Alive	TUMOR F	1892						Positive	R0	0	1892	0	1892	0	1892	0	1892
TCGA-DX	TCGA-DX	SARC	53	FEMALE	WHITE		Leiomyosarcoma (LM	2012	-19588	Alive	WITH TUN	923		Distant M	Lung		352	Positive	R0	0	923	0	923	1	352	1	352
TCGA-DX	TCGA-DX	SARC	20	FEMALE	WHITE		Synovial Sarcoma - I	2002	-7604	Alive	TUMOR F	4627						Negative	R0	0	4627	0	4627	0	4627	0	4627
TCGA-DX	TCGA-DX	SARC	72	MALE	WHITE		Synovial Sarcoma - I	2006	-26503	Dead	WITH TUMOR		897	Distant M	Lung		251	Negative	R0	1	897	1	897	1	251	1	251

TCGA-DX	TCGA-DX	SARC	69	MALE	WHITE		Synovial Sarcoma - I	2008	-25363	Dead	TUMOR FREE		1466					Negative	R0	1	1466	0	1466	0	1466	0	1466
TCGA-DX	TCGA-DX	SARC	50	FEMALE	WHITE		Dedifferentiated lipo	2013	-18528	Alive	TUMOR F	610						Positive	R0	0	610	0	610	0	610	0	610
TCGA-DX	TCGA-DX	SARC	71	MALE	BLACK OR AFRICAN AMERICAN		Pleomorphic 'MFH' /	2013	-26193	Alive	TUMOR F	463						Negative	R0	0	463	0	463	0	463	0	463
TCGA-DX	TCGA-DX	SARC	79	MALE	WHITE		Myxofibrosarcoma	2013	-29211	Alive	TUMOR F	721						Negative	R0	0	721	0	721	0	721	0	721
TCGA-DX	TCGA-DX	SARC	83	FEMALE	WHITE		Myxofibrosarcoma	2013	-30333	Alive	TUMOR F	546		Distant M	Lung		330	Negative	R0	0	546	0	546	1	330	1	330
TCGA-DX	TCGA-DX	SARC	86	MALE	WHITE		Pleomorphic 'MFH' /	2013	-31770	Dead	WITH TUMOR		439	Distant M	Other, sp	Pleura	272	Positive	R0	1	439	1	439	1	272	1	272
TCGA-DX	TCGA-DX	SARC	76	MALE	WHITE		Pleomorphic 'MFH' /	2011	-28091	Dead	TUMOR FREE		284					Negative	R0	1	284	0	284	0	284	0	284
TCGA-DX	TCGA-DX	SARC	61	FEMALE	WHITE		Pleomorphic 'MFH' /	2009	-22407	Alive	TUMOR F	1885						Negative	R0	0	1885	0	1885	0	1885	0	1885
TCGA-DX	TCGA-DX	SARC	59	MALE	WHITE		Pleomorphic 'MFH' /	2011	-21728	Alive	TUMOR F	1373						Negative	R0	0	1373	0	1373	0	1373	0	1373
TCGA-DX	TCGA-DX	SARC	60	MALE	WHITE		Pleomorphic 'MFH' /	2011	-22152	Alive	TUMOR F	1456						Negative	R0	0	1456	0	1456	0	1456	0	1456
TCGA-DX	TCGA-DX	SARC	78	FEMALE	WHITE		Pleomorphic 'MFH' /	2011	-28760	Alive	TUMOR F	1161						Positive	R1	0	1161	0	1161			0	1161
TCGA-DX	TCGA-DX	SARC	67	FEMALE	WHITE		Pleomorphic 'MFH' /	2011	-24513	Alive	TUMOR F	1240		Locoregic	Other, sp	Calf	333	Positive	R1	0	1240	0	1240			1	333
TCGA-DX	TCGA-DX	SARC	85	MALE	WHITE		Pleomorphic 'MFH' /	2009	-31382	Alive	WITH TUI	428		Distant M	Lung		216	Positive	R1	0	428	0	428			1	216
TCGA-DX	TCGA-DX	SARC	63	MALE	WHITE		Pleomorphic 'MFH' /	2010	-23027	Alive	TUMOR F	1579						Negative	R0	0	1579	0	1579	0	1579	0	1579
TCGA-DX	TCGA-DX	SARC	63	FEMALE	WHITE		Pleomorphic 'MFH' /	2002	-23302	Alive	TUMOR F	4573						Negative	R0	0	4573	0	4573	0	4573	0	4573
TCGA-DX	TCGA-DX	SARC	68	FEMALE	BLACK OR AFRICAN AMERICAN		Pleomorphic 'MFH' /	2006	-25023	Dead	TUMOR FREE		850					Positive	R0	1	850	0	850	0	850	0	850
TCGA-DX	TCGA-DX	SARC	63	FEMALE	WHITE		Pleomorphic 'MFH' /	2011	-23204	Alive	TUMOR F	1230		Locoregic	Other, sp	Axilla	668	Positive	R1	0	1230	0	1230			1	668
TCGA-DX	TCGA-DX	SARC	58	MALE	WHITE		Pleomorphic 'MFH' /	2006	-21470	Dead	WITH TUMOR		1116	Distant M	Lung		107	Positive	R1	1	1116	1	1116			1	107
TCGA-DX	TCGA-DX	SARC	57	MALE	WHITE		Myxofibrosarcoma	2004	-21005	Dead	WITH TUMOR		1845	Locoregic	Other, sp	Retroperit	357	Negative	R0	1	1845	1	1845	1	357	1	357
TCGA-DX	TCGA-DX	SARC	52	MALE	WHITE		Pleomorphic 'MFH' /	2006	-19339	Dead	WITH TUMOR		591					Negative	R0	1	591	1	591			1	591
TCGA-DX	TCGA-DX	SARC	78	FEMALE	BLACK OR AFRICAN AMERICAN		Leiomyosarcoma (LM	2013	-28853	Dead	WITH TUMOR		200	Distant M	Lung		90	Positive	R0	1	200	1	200	1	90	1	90
TCGA-DX	TCGA-DX	SARC	72	MALE	WHITE		Pleomorphic 'MFH' /	2008	-26624	Dead	TUMOR FREE		35					Positive	R1	1	35	0	35			0	35
TCGA-DX	TCGA-DX	SARC	60	MALE	WHITE		Myxofibrosarcoma	2013	-22117	Alive	WITH TUI	541		Distant M	Lung		132	Positive	R0	0	541	0	541	1	132	1	132
TCGA-DX	TCGA-DX	SARC	76	FEMALE	WHITE		Dedifferentiated lipo	2013	-27840	Alive	TUMOR F	365						Positive	R0	0	365	0	365	0	365	0	365
TCGA-DX	TCGA-DX	SARC	36	MALE	WHITE		Dedifferentiated lipo	2013	-13239	Alive	TUMOR F	478						Negative	R0	0	478	0	478	0	478	0	478
TCGA-DX	TCGA-DX	SARC	80	FEMALE	WHITE		Dedifferentiated lipo	2013	-29569	Alive	TUMOR F	482						Negative	R0	0	482	0	482	0	482	0	482
TCGA-DX	TCGA-DX	SARC	71	FEMALE	WHITE		Pleomorphic 'MFH' /	2006	-26154	Dead	WITH TUMOR		1235					Positive	R1	1	1235	1	1235			1	1235
TCGA-DX	TCGA-DX	SARC	35	MALE	WHITE		Myxofibrosarcoma	2009	-12974	Alive	TUMOR F	2007						Positive	R1	0	2007	0	2007			0	2007
TCGA-DX	TCGA-DX	SARC	78	FEMALE	WHITE		Pleomorphic 'MFH' /	2007	-28757	Alive	TUMOR F	2619						Negative	R0	0	2619	0	2619	0	2619	0	2619
TCGA-DX	TCGA-DX	SARC	79	MALE	WHITE		Pleomorphic 'MFH' /	2005	-29136	Dead	WITH TUMOR		367	Locoregional Recurrence			161	Negative	R0	1	367	1	367	1	161	1	161
TCGA-DX	TCGA-DX	SARC	65	FEMALE	WHITE		Pleomorphic 'MFH' /	2009	-23748	Alive	TUMOR F	2030		Distant M	Other, sp	inguinal l\	490	Negative	R0	0	2030	0	2030	1	490	1	490
TCGA-DX	TCGA-DX	SARC	53	FEMALE	WHITE		Myxofibrosarcoma	2005	-19708	Alive	TUMOR F	280						Negative	R0	0	280	0	280	0	280	0	280
TCGA-DX	TCGA-DX	SARC	54	FEMALE	BLACK OR AFRICAN AMERICAN		Myxofibrosarcoma	2008	-19749	Alive	TUMOR F	2369		Distant M	Lung		784	Negative	R0	0	2369	0	2369	1	784	1	784
TCGA-DX	TCGA-DX	SARC	81	MALE	WHITE		Myxofibrosarcoma	2006	-29716	Alive	TUMOR F	3241						Negative	R0	0	3241	0	3241	0	3241	0	3241
TCGA-DX	TCGA-DX	SARC	62	FEMALE	WHITE		Pleomorphic 'MFH' /	2006	-22951	Alive	TUMOR F	3238						Negative	R0	0	3238	0	3238	0	3238	0	3238
TCGA-DX	TCGA-DX	SARC	73	FEMALE	WHITE		Pleomorphic 'MFH' /	2010	-26736	Dead	WITH TUMOR		404	Locoregional Recurrence			111	Negative	R0	1	404	1	404	1	111	1	111
TCGA-DX	TCGA-DX	SARC	87	FEMALE	WHITE		Pleomorphic 'MFH' /	2010	-31780	Alive	TUMOR F	1533						Negative	R0	0	1533	0	1533	0	1533	0	1533
TCGA-DX	TCGA-DX	SARC	53	FEMALE	WHITE		Myxofibrosarcoma	2004	-19698	Dead	WITH TUMOR		695	Distant M	Lung		504	Negative	R0	1	695	1	695	1	504	1	504
TCGA-DX	TCGA-DX	SARC	51	MALE	WHITE		Myxofibrosarcoma	2006	-18631	Alive	TUMOR F	3102						Negative	R0	0	3102	0	3102	0	3102	0	3102
TCGA-DX	TCGA-DX	SARC	65	MALE	WHITE		Dedifferentiated lipo	2012	-24026	Alive	WITH TUI	928		Locoregic	Other, sp	Retroperit	284	Negative	R0	0	928	0	928	1	284	1	284
TCGA-DX	TCGA-DX	SARC	86	MALE	WHITE		Dedifferentiated lipo	2008	-31704	Dead			1825					Positive	R1	1	1825		1825			0	1825
TCGA-DX	TCGA-DX	SARC	73	FEMALE	WHITE		Dedifferentiated lipo	2003	-26696	Dead			2575					Negative	R0	1	2575		2575	0	2575	0	2575
TCGA-DX	TCGA-DX	SARC	80	MALE	WHITE		Dedifferentiated lipo	2009	-29230	Dead	WITH TUMOR		978	Locoregional Recurrence			533	Negative	R0	1	978	1	978	1	533	1	533
TCGA-DX	TCGA-DX	SARC	28	FEMALE	WHITE		Synovial Sarcoma - I	2009	-10337	Alive	TUMOR F	1548						Negative	R0	0	1548	0	1548	0	1548	0	1548
TCGA-DX	TCGA-DX	SARC	27	MALE	WHITE		Synovial Sarcoma - I	2008	-10034	Alive	WITH TUI	2169		Distant M	Lung		567	Positive	R0	0	2169	0	2169	1	567	1	567
TCGA-FX	TCGA-FX	SARC	61	FEMALE	WHITE		Dedifferentiated lipo	2011	-22422	Alive	TUMOR F	1495						Negative	R0	0	1495	0	1495	0	1495	0	1495
TCGA-FX	TCGA-FX	SARC	61	FEMALE	WHITE		Dedifferentiated lipo	2011	-22422	Alive	TUMOR F	1495						Negative	R0	0	1495	0	1495	0	1495	0	1495
TCGA-FX	TCGA-FX	SARC	54	MALE	WHITE		Leiomyosarcoma (LM	2011	-19903	Alive	TUMOR F	947						Negative	R0	0	947	0	947	0	947	0	947
TCGA-FX	TCGA-FX	SARC	53	MALE	WHITE		Dedifferentiated lipo	2011	-19533	Alive	TUMOR F	505						Negative	R0	0	505	0	505	0	505	0	505
TCGA-FX	TCGA-FX	SARC	65	FEMALE	WHITE		Leiomyosarcoma (LM	2011	-23757	Alive	TUMOR F	660						[Unknowr	RX	0	660	0	660			0	660
TCGA-FX	TCGA-FX	SARC	87	MALE	WHITE		Undifferentiated Plec	2012	-31868	Alive	TUMOR F	618						Positive	R1	0	618	0	618			0	618
TCGA-FX	TCGA-FX	SARC	56	FEMALE	WHITE		Leiomyosarcoma (LM	2011	-20756	Dead	WITH TUMOR		605	Distant M	Lung		168	[Unknowr	RX	1	605	1	605			1	168
TCGA-FX	TCGA-FX	SARC	56	FEMALE	WHITE		Leiomyosarcoma (LM	2011	-20756	Dead	WITH TUMOR		605	Distant M	Lung		168	[Unknowr	RX	1	605	1	605			1	168
TCGA-FX	TCGA-FX	SARC	49	FEMALE	WHITE		Leiomyosarcoma (LM	2013	-18181	Alive	TUMOR F	471						Negative	R0	0	471	0	471	0	471	0	471
TCGA-FX	TCGA-FX	SARC	31	FEMALE	WHITE		Sarcoma; synovial; p	2013	-11376	Dead	TUMOR FREE		245					Negative	R0	1	245	0	245	0	245	0	245
TCGA-HB	TCGA-HB	SARC	78	FEMALE	BLACK OR AFRICAN AMERICAN		Undifferentiated Plec	2009	-28632	Alive	TUMOR F	2057						Negative	R0	0	2057	0	2057	0	2057	0	2057
TCGA-HB	TCGA-HB	SARC	46	FEMALE	WHITE		Leiomyosarcoma (LM	2009	-17044	Dead	WITH TUMOR		1061	Distant M	Lung		326	Negative	R0	1	1061	1	1061	1	326	1	326
TCGA-HB	TCGA-HB	SARC	75	MALE	WHITE		Undifferentiated Plec	2012	-27583	Dead	WITH TUMOR		139					Positive	R1	1	139	1	139			1	139
TCGA-HB	TCGA-HB	SARC	58	FEMALE	WHITE		Leiomyosarcoma (LM	2012	-21233	Alive	WITH TUI	1126		Distant M	Lung		894	Negative	R0	0	1126	0	1126	1	894	1	894
TCGA-HB	TCGA-HB	SARC	58	FEMALE	WHITE		Leiomyosarcoma (LM	2010	-21416	Dead	WITH TUMOR		384					Negative	R0	1	384	1	384			1	384
TCGA-HS	TCGA-HS	SARC	67	FEMALE	[Not Evaluated]		Leiomyosarcoma (LMS)		-24775	Alive	TUMOR F	1013						Negative	R0	0	1013	0	1013	0	1013	0	1013
TCGA-HS	TCGA-HS	SARC	69	FEMALE	[Not Evaluated]		Leiomyosarcoma (LMS)		-25549	Dead	WITH TUMOR		22					[Unknowr	R2	1	22	1	22			1	22
TCGA-HS	TCGA-HS	SARC	58	FEMALE	[Not Evaluated]		Leiomyosarcoma (LMS)		-21463	Alive	TUMOR F	1585						Negative	R0	0	1585	0	1585	0	1585	0	1585
TCGA-HS	TCGA-HS	SARC	65	FEMALE	[Not Evaluated]		Leiomyosarcoma (LMS)		-24025	Alive	WITH TUI	298						[Unknowr	RX	0	298	0	298			0	298
TCGA-IE-	TCGA-IE-	SARC	42	MALE	WHITE		Leiomyosarcoma (LM	2004	-15607	Dead	WITH TUMOR		2448	Distant M	Lung		490	Negative	R0	1	2448	1	2448	1	490	1	490
TCGA-IE-	TCGA-IE-	SARC	35	FEMALE	WHITE		Leiomyosarcoma (LM	2011	-12871	Alive	TUMOR F	819						Negative	R0	0	819	0	819	0	819	0	819
TCGA-IE-	TCGA-IE-	SARC	67	FEMALE	WHITE		Leiomyosarcoma (LM	2012	-24663	Alive	TUMOR F	594						Negative	R0	0	594	0	594	0	594	0	594
TCGA-IE-	TCGA-IE-	SARC	84	FEMALE	WHITE		Undifferentiated Plec	2012	-30858	Alive	TUMOR F	98						Positive	R1	0	98	0	98			0	98
TCGA-IE-	TCGA-IE-	SARC	54	MALE	WHITE		Leiomyosarcoma (LM	2012	-19815	Alive		42						Positive	R1	0	42	0	42			0	42
TCGA-IE-	TCGA-IE-	SARC	65	FEMALE	WHITE		Undifferentiated Plec	2012	-23756	Alive	TUMOR F	605						Negative	R0	0	605	0	605	0	605	0	605
TCGA-IF-	TCGA-IF-	SARC	70	FEMALE	WHITE		Dedifferentiated lipo	1998	-25680	Dead	WITH TUMOR		1129	Locoregic	Other, sp	retroperit	441	Negative	R0	1	1129	1	1129	1	441	1	441
TCGA-IF-	TCGA-IF-	SARC	80	MALE	WHITE		Leiomyosarcoma (LM	1995	-29469	Dead	WITH TUMOR		767	Distant M	Lung		560	Negative	R0	1	767	1	767	1	560	1	560
TCGA-IF-	TCGA-IF-	SARC	80	MALE	WHITE		Leiomyosarcoma (LM	1995	-29469	Dead	WITH TUMOR		767	Distant M	Lung		560	Negative	R0	1	767	1	767	1	560	1	560
TCGA-IF-	TCGA-IF-	SARC	82	FEMALE	WHITE		Leiomyosarcoma (LM	2010	-30141	Alive	TUMOR F	1590						Negative	R0	0	1590	0	1590	0	1590	0	1590
TCGA-IS-	TCGA-IS-	SARC	55	FEMALE	WHITE		Leiomyosarcoma (LM	2000	-20205	Dead	WITH TUMOR		858						R2	1	858	1	858			1	858
TCGA-IS-	TCGA-IS-	SARC	63	FEMALE	WHITE		Leiomyosarcoma (LM	2000	-23022	Alive	TUMOR F	5204		Distant M	Lung		119		R0	0	5204	0	5204	1	119	1	119
TCGA-IS-	TCGA-IS-	SARC	44	FEMALE	WHITE		Leiomyosarcoma (LM	2002	-16265	Alive	TUMOR F	4845							R0	0	4845	0	4845	0	4845	0	4845
TCGA-IS-	TCGA-IS-	SARC	73	FEMALE	WHITE		Leiomyosarcoma (LM	2004	-26775	Dead	WITH TUMOR		413	Distant M	Lung		76		R0	1	413	1	413	1	76	1	76
TCGA-IW	TCGA-IW	SARC	48	FEMALE	WHITE		Leiomyosarcoma (LM	2005	-17721	Alive	TUMOR F	2615		Locoregional Disease			2207		R0	0	2615	0	2615	1	2207	1	2207
TCGA-IW	TCGA-IW	SARC	76	FEMALE	WHITE		Leiomyosarcoma (LM	2010	-27869	Dead	WITH TUMOR		180	Distant M	Lung		9	Negative	R0	1	180	1	180	1	9	1	9
TCGA-IW	TCGA-IW	SARC	59	FEMALE	ASIAN		Leiomyosarcoma (LM	2011	-21674	Alive	TUMOR F	688							R0	0	688	0	688	0	688	0	688
TCGA-JV-	TCGA-JV-	SARC	60	FEMALE	BLACK OR AFRICAN AMERICAN		Leiomyosarcoma (LM	2012	-21933	Alive	TUMOR F	499						Negative	R0	0	499	0	499	0	499	0	499
TCGA-JV-	TCGA-JV-	SARC	69	FEMALE	BLACK OR AFRICAN AMERICAN		Leiomyosarcoma (LM	2012	-25505	Dead	WITH TUMOR		32					Positive	R2	1	32	1	32			1	32
TCGA-JV-	TCGA-JV-	SARC	43	FEMALE	WHITE		Leiomyosarcoma (LM	2013	-16001	Dead	WITH TUMOR		119					Positive	R2	1	119	1	119			1	119
TCGA-K1	TCGA-K1	SARC	64	FEMALE	WHITE		Leiomyosarcoma (LM	2006	-23631	Alive	TUMOR F	3080					1033	Negative	R0	0	3080	0	3080	1	1033	1	1033
TCGA-K1	TCGA-K1	SARC	64	FEMALE	WHITE		Leiomyosarcoma (LM	2006	-23631	Alive	TUMOR F	3080					1033	Negative	R0	0	3080	0	3080	1	1033	1	1033
TCGA-K1	TCGA-K1	SARC	42	MALE	WHITE		Leiomyosarcoma (LM	2010	-15494	Alive	TUMOR F	1622						Negative	R0	0	1622	0	1622	0	1622	0	1622
TCGA-K1	TCGA-K1	SARC	42	MALE	WHITE		Leiomyosarcoma (LM	2010	-15494	Alive	TUMOR F	1622						Negative	R0	0	1622	0	1622	0	1622	0	1622
TCGA-K1	TCGA-K1	SARC	65	FEMALE	WHITE		Leiomyosarcoma (LM	2009	-23910	Alive	WITH TUI	1891		Distant M	Liver		826	Negative	R0	0	1891	0	1891	1	826	1	826
TCGA-K1	TCGA-K1	SARC	62	FEMALE	WHITE		Leiomyosarcoma (LM	2003	-22680	Alive	WITH TUI	3765		Locoregic	Other, sp	adjeacent	2036	Negative	R0	0	3765	0	3765	1	2036	1	2036
TCGA-K1	TCGA-K1	SARC	62	FEMALE	WHITE		Leiomyosarcoma (LM	2003	-22680	Alive	WITH TUI	3765		Locoregic	Other, sp	adjeacent	2036	Negative	R0	0	3765	0	3765	1	2036	1	2036
TCGA-K1	TCGA-K1	SARC	62	FEMALE	WHITE		Leiomyosarcoma (LM	2003	-22680	Alive	WITH TUI	3765		Locoregic	Other, sp	adjeacent	2036	Negative	R0	0	3765	0	3765	1	2036	1	2036
TCGA-K1	TCGA-K1	SARC	48	MALE	WHITE		Leiomyosarcoma (LM	2012	-17739	Alive	WITH TUI	528		Distant M	Lung		129	Negative	R0	0	528	0	528	1	129	1	129
TCGA-K1	TCGA-K1	SARC	66	FEMALE	WHITE		Myxofibrosarcoma	2013	-24356	Dead	WITH TUMOR		711	Distant M	Bone		142	Positive	R1	1	711	1	711			1	142
TCGA-K1	TCGA-K1	SARC	67	MALE	WHITE		Leiomyosarcoma (LM	2013	-24701	Alive	WITH TUI	158						Positive	R1	0	158	0	158			0	158
TCGA-KD	TCGA-KD	SARC	55	FEMALE	WHITE		Leiomyosarcoma (LM	2009	-20410	Dead	WITH TUMOR		1941	Distant M	Bone		351	[Unknowr	RX	1	1941	1	1941			1	351
TCGA-KD	TCGA-KD	SARC	69	FEMALE	[Not Evaluated]		Leiomyosarcoma (LM	2010	-25249	Alive	TUMOR F	499						Negative	R0	0	499	0	499	0	499	0	499
TCGA-KD	TCGA-KD	SARC	41	FEMALE	WHITE		Leiomyosarcoma (LM	2010	-15018	Dead	WITH TUMOR		1073	Distant M	Lung		1060	Negative	R0	1	1073	1	1073	1	1060	1	1060
TCGA-KF	TCGA-KF	SARC	71	FEMALE	WHITE		Leiomyosarcoma (LM	2012	-25969	Alive	TUMOR F	356						Negative	RX	0	356	0	356			0	356
TCGA-KF	TCGA-KF	SARC	71	FEMALE	WHITE		Leiomyosarcoma (LM	2012	-25969	Alive	TUMOR F	356						Negative	RX	0	356	0	356			0	356
TCGA-LI-	TCGA-LI-	SARC	75	FEMALE	WHITE		Myxofibrosarcoma	2013	-27671	Alive	TUMOR F	922		Distant M	Lung		574	Positive	RX	0	922	0	922			1	574
TCGA-LI-	TCGA-LI-	SARC	72	FEMALE	WHITE		Pleomorphic 'MFH' /	2013	-26468	Dead	TUMOR FREE		540	Distant M	Lung		426	Negative	R2	1	540	0	540			1	426
TCGA-ME	TCGA-ME	SARC	60	FEMALE	WHITE		Undifferentiated Plec	2012	-22115	Alive	TUMOR F	773						Negative	R0	0	773	0	773	0	773	0	773
TCGA-ME	TCGA-ME	SARC	90	MALE	WHITE		Undifferentiated Plec	2012	-32873	Alive	WITH TUI	252						Positive	R1	0	252	0	252	0	252	0	252
TCGA-ME	TCGA-ME	SARC	63	MALE	WHITE		Leiomyosarcoma (LM	2012	-23115	Alive	TUMOR F	15						Negative	R0	0	15	0	15	0	15	0	15
TCGA-ME	TCGA-ME	SARC	49	MALE	WHITE		Pleomorphic 'MFH' /	2013	-18177	Alive	TUMOR F	661						Negative	RX	0	661	0	661			0	661
TCGA-ME	TCGA-ME	SARC	53	FEMALE	WHITE		Leiomyosarcoma (LM	2013	-19510	Alive	TUMOR F	600						Negative	R0	0	600	0	600	0	600	0	600
TCGA-MJ	TCGA-MJ	SARC	57	FEMALE	BLACK OR AFRICAN AMERICAN		Leiomyosarcoma (LM	2013	-21105	Alive	TUMOR F	456						Negative	R0	0	456	0	456	0	456	0	456
TCGA-MJ	TCGA-MJ	SARC	55	FEMALE	BLACK OR AFRICAN AMERICAN		Myxofibrosarcoma	2012	-20100	Alive	WITH TUI	547		New Prim	Other, sp	Foot	268	Positive	R1	0	547	0	547			1	268
TCGA-MJ	TCGA-MJ	SARC	28	MALE	WHITE		Synovial Sarcoma - I	2013	-10451	Alive	TUMOR F	681							RX	0	681	0	681			0	681
TCGA-MC	TCGA-MC	SARC	37	FEMALE	ASIAN		Dedifferentiated lipo	2012	-13765	Alive	TUMOR F	352							R0	0	352	0	352	0	352	0	352
TCGA-MC	TCGA-MC	SARC	75	FEMALE	ASIAN		Leiomyosarcoma (LM	2010	-27731	Dead	WITH TUMOR		318	Distant M	Brain		259		R0	1	318	1	318	1	259	1	259
TCGA-N1	TCGA-N1	SARC	41	FEMALE	BLACK OR AFRICAN AMERICAN		Leiomyosarcoma (LM	2011	-15112	Dead	WITH TUMOR		224	Distant M	Lung		116	[Unknowr	RX	1	224	1	224			1	116
TCGA-PC	TCGA-PC	SARC	63	MALE	WHITE		Leiomyosarcoma (LM	2004	-23019	Alive	TUMOR F	3740						Negative	R0	0	3740	0	3740	0	3740	0	3740
TCGA-PC	TCGA-PC	SARC	67	MALE	WHITE		Leiomyosarcoma (LM	2004	-24761	Dead			1142						RX	1	1142		1142			0	1142
TCGA-PC	TCGA-PC	SARC	73	MALE	WHITE		Leiomyosarcoma (LM	2005	-26699	Dead			193					Positive	R1	1	193		193			0	193
TCGA-PC	TCGA-PC	SARC	77	FEMALE	WHITE		Leiomyosarcoma (LM	2005	-28421	Dead	TUMOR FREE		1175						R1	1	1175	0	1175			0	1175
TCGA-PC	TCGA-PC	SARC	59	FEMALE	WHITE		Leiomyosarcoma (LM	2006	-21606	Dead	WITH TUMOR		2464					Negative	R1	1	2464	1	2464			1	2464
TCGA-PC	TCGA-PC	SARC	72	FEMALE	WHITE		Leiomyosarcoma (LM	2007	-26414	Dead	WITH TUMOR		550						R1	1	550	1	550			1	550
TCGA-PT	TCGA-PT	SARC	57	FEMALE	WHITE		Leiomyosarcoma (LM	2013	-20985	Alive	TUMOR F	813						Positive	R1	0	813	0	813			0	813
TCGA-OC	TCGA-OC	SARC	68	MALE	WHITE		Pleomorphic 'MFH' /	2013	-25113	Alive	TUMOR F	637						Negative	R0	0	637	0	637	0	637	0	637
TCGA-OC	TCGA-OC	SARC	75	MALE	WHITE		Pleomorphic 'MFH' /	2013	-27556	Alive	TUMOR F	398		New Prim	Other, sp	skin	259	Positive	RX	0	398	0	398			1	259
TCGA-OC	TCGA-OC	SARC	53	FEMALE	BLACK OR AFRICAN AMERICAN		Undifferentiated Plec	2013	-19395	Alive	TUMOR F	537						Negative	RX	0	537	0	537			0	537
TCGA-OC	TCGA-OC	SARC	42	MALE	WHITE		Giant cell 'MFH' / Ur	2006	-15420	Alive		56						[Unknowr	R1	0	56	0	56			0	56
TCGA-OC	TCGA-OC	SARC	76	MALE	WHITE		Undifferentiated Plec	1997	-27977	Dead	WITH TUMOR		1424	Distant M	Lung		405	Negative	R0	1	1424	1	1424	1	405	1	405
TCGA-QC	TCGA-QC	SARC	60	FEMALE	WHITE		Dedifferentiated lipo	1996	-22231	Dead	WITH TUMOR		550					Negative	RX	1	550	1	550			1	550
TCGA-OC	TCGA-OC	SARC	53	FEMALE	WHITE		Leiomyosarcoma (LM	1994	-19654	Dead	WITH TUMOR		1478	Locoregic	Other, sp	Left Pelvis	111	Positive	R1	1	1478	1	1478			1	111
TCGA-OC	TCGA-OC	SARC	63	FEMALE	WHITE		Leiomyosarcoma (LM	2011	-23357	Alive	TUMOR F	1092						Negative	R0	0	1092	0	1092	0	1092	0	1092
TCGA-OC	TCGA-OC	SARC	52	MALE	WHITE		Leiomyosarcoma (LM	2011	-19004	Alive	TUMOR F	1129						Negative	R0	0	1129	0	1129	0	1129	0	1129
TCGA-OC	TCGA-OC	SARC	68	FEMALE	WHITE		Malignant Periphera	1998	-24916	Alive	TUMOR F	5723		New Prim	Other, sp	gastrointe	2682	Negative	RX	0	5723	0	5723			1	2682
TCGA-OC	TCGA-OC	SARC	56	MALE	WHITE		Desmoid Tumor	2003	-20729	Alive	WITH TUI	3964		Locoregic	Other, sp	left abdor	499	Positive	RX	0	3964	0	3964			1	499
TCGA-OC	TCGA-OC	SARC	70	MALE	WHITE		Leiomyosarcoma (LM	2005	-25682	Alive	TUMOR F	3406						Negative	RX	0	3406	0	3406			0	3406
TCGA-QC	TCGA-QC	SARC	52	MALE	WHITE		Malignant Periphera	2009	-19304	Dead	WITH TUMOR		1970	New Prim	Liver		1852	Negative	RX	1	1970	1	1970			1	1852
TCGA-OC	TCGA-OC	SARC	31	FEMALE	WHITE		Malignant Periphera	2010	-11599	Dead	WITH TUMOR		320	Locoregic	Other, sp	Spine T6 t	197	Positive	RX	1	320	1	320			1	197
TCGA-RN	TCGA-RN	SARC	62	FEMALE	WHITE		Malignant Periphera	2010	-22981	Alive	TUMOR F	22						[Unknowr	RX	0	22	0	22			0	22
TCGA-RN	TCGA-RN	SARC	52	MALE	WHITE		Malignant Periphera	2013	-19123	Alive	WITH TUI	510		Locoregic	Other, sp	Left Shoul	307	[Unknown]		0	510	0	510			1	307
TCGA-SG	TCGA-SG	SARC	47	MALE	WHITE		Pleomorphic 'MFH' /	2013	-17368	Alive	WITH TUI	576		Distant M	Lung		105	Negative	R1	0	576	0	576			1	105
TCGA-SG	TCGA-SG	SARC	44	FEMALE	WHITE		Dedifferentiated lipo	2004	-16188	Dead	TUMOR FREE		118					[Unknowr	RX	1	118	0	118			0	118
TCGA-SG	TCGA-SG	SARC	78	MALE	WHITE		Undifferentiated Plec	2013	-28498	Alive	TUMOR F	533						Positive	R1	0	533	0	533			0	533
TCGA-SI-	TCGA-SI-	SARC	29	MALE	WHITE		Malignant Periphera	2013	-10710	Dead	WITH TUMOR		694	Distant M	Lung		264	Positive	RX	1	694	1	694			1	264
TCGA-SI-	TCGA-SI-	SARC	29	MALE	WHITE		Malignant Periphera	2013	-10710	Dead	WITH TUMOR		694	Distant M	Lung		264	Positive	RX	1	694	1	694			1	264
TCGA-SI-	TCGA-SI-	SARC	50	MALE	WHITE		Malignant Periphera	2012	-18367	Alive	TUMOR F	771						[Unknowr	RX	0	771	0	771			0	771
TCGA-SI-	TCGA-SI-	SARC	34	FEMALE	BLACK OR AFRICAN AMERICAN		Malignant Periphera	2012	-12559	Alive	TUMOR F	996						Negative	R0	0	996	0	996	0	996	0	996
TCGA-SI-	TCGA-SI-	SARC	80	FEMALE	WHITE		Undifferentiated Plec	2013	-29315	Dead	WITH TUMOR		262					Positive	R1	1	262	1	262			1	262
TCGA-SI-	TCGA-SI-	SARC	20	FEMALE	WHITE		Malignant Periphera	2013	-7530	Alive	WITH TUI	597		Distant M	Lung		273	Positive	R1	0	597	0	597			1	273
TCGA-UE	TCGA-UE	SARC	50	FEMALE	ASIAN		Undifferentiated Plec	2013	-18516	Alive	TUMOR F	402						Negative	R0	0	402	0	402	0	402	0	402
TCGA-UE	TCGA-UE	SARC	90	FEMALE	ASIAN		Undifferentiated Plec	2013	-32872	Alive	TUMOR F	408						Negative	R0	0	408	0	408	0	408	0	408
TCGA-VT	TCGA-VT	SARC	66	MALE	WHITE		Undifferentiated Plec	2013	-24128	Alive	WITH TUI	326		Distant M	Lung		26	Negative	R0	0	326	0	326	1	26	1	26
TCGA-VT	TCGA-VT	SARC	49	FEMALE	WHITE		Undifferentiated Plec	2012	-17925	Alive	TUMOR F	796		Distant M	Other, sp	inguinal l\	355	Negative	R0	0	796	0	796	1	355	1	355
TCGA-VT	TCGA-VT	SARC	49	FEMALE	WHITE		Undifferentiated Plec	2012	-17925	Alive	TUMOR F	796		Distant M	Other, sp	inguinal l\	355	Negative	R0	0	796	0	796	1	355	1	355
TCGA-VT	TCGA-VT	SARC	71	MALE	WHITE		Undifferentiated Plec	2013	-26100	Alive	TUMOR F	379						Negative	R0	0	379	0	379	0	379	0	379
TCGA-WK	TCGA-WK	SARC	66	FEMALE	WHITE		Dedifferentiated lipo	2010	-24201	Dead	WITH TUMOR		363	Locoregic	Other, sp	Abdomina	105	Negative	R0	1	363	1	363	1	105	1	105
TCGA-WK	TCGA-WK	SARC	57	MALE	WHITE		Dedifferentiated lipo	2009	-20891	Dead	WITH TUMOR		146	Locoregic	Other, sp	Abdomina	27	Positive	R2	1	146	1	146			1	27
TCGA-WK	TCGA-WK	SARC	58	FEMALE	WHITE		Leiomyosarcoma (LM	2008	-21521	Alive	TUMOR F	2579						Negative	R0	0	2579	0	2579	0	2579	0	2579
TCGA-WK	TCGA-WK	SARC	40	FEMALE	WHITE		Synovial Sarcoma - I	2008	-14958	Dead	WITH TUMOR		1262	Locoregic	Other, sp	Flank	599	Negative	R0	1	1262	1	1262	1	599	1	599
TCGA-WK	TCGA-WK	SARC	70	MALE	WHITE		Leiomyosarcoma (LM	2007	-25701	Dead	WITH TUMOR		1164	Locoregic	Other, sp	Retroperit	111	Negative	R0	1	1164	1	1164	1	111	1	111
TCGA-WK	TCGA-WK	SARC	53	FEMALE	WHITE		Leiomyosarcoma (LM	2007	-19597	Dead	WITH TUMOR		1991	Locoregic	Other, sp	Intraabdo	103	Positive	R1	1	1991	1	1991			1	103
TCGA-WK	TCGA-WK	SARC	56	FEMALE	WHITE		Leiomyosarcoma (LM	2007	-20460	Dead	WITH TUMOR		1722	Distant M	Other, sp	Scalp	89	Negative	R0	1	1722	1	1722	1	89	1	89
TCGA-WK	TCGA-WK	SARC	49	FEMALE	WHITE		Undifferentiated Plec	2010	-18233	Dead	WITH TUMOR		688	Distant M	Lung		111	Negative	R0	1	688	1	688	1	111	1	111
TCGA-WF	TCGA-WF	SARC	59	FEMALE	WHITE		Leiomyosarcoma (LM	2012		Dead	WITH TUMOR		261	Distant M	Lung		214	Negative	RX	1	261	1	261			1	214
TCGA-X2	TCGA-X2	SARC	49	FEMALE	WHITE		Leiomyosarcoma (LM	2009	-18079	Alive	TUMOR F	1927						Negative	R0	0	1927	0	1927	0	1927	0	1927
TCGA-X6	TCGA-X6	SARC	89	MALE	WHITE		Myxofibrosarcoma	2011	-32848	Dead	WITH TUMOR		804	Distant M	Lung		146	Positive	R1	1	804	1	804			1	146
TCGA-X6	TCGA-X6	SARC	90	FEMALE	WHITE		Leiomyosarcoma (LM	2012	-32872	Alive	TUMOR F	1092						Positive	R1	0	1092	0	1092			0	1092
TCGA-X6	TCGA-X6	SARC	58	FEMALE	WHITE		Leiomyosarcoma (LM	2011	-21467	Dead	WITH TUMOR		550	Distant M	Lung		104	Positive	R1	1	550	1	550			1	104
TCGA-X6	TCGA-X6	SARC	74	MALE	WHITE		Leiomyosarcoma (LM	2011	-27252	Alive	TUMOR F	1379		Distant M	Lung		301	Negative	R0	0	1379	0	1379	1	301	1	301
TCGA-X6	TCGA-X6	SARC	63	FEMALE	WHITE		Leiomyosarcoma (LM	2013	-23341	Alive	WITH TUI	938		Distant M	Lung		558	Negative	R0	0	938	0	938	1	558	1	558
TCGA-X6	TCGA-X6	SARC	56	MALE	WHITE		Undifferentiated Plec	2012	-20507	Alive	WITH TUI	1070		New Prim	Lung		7	Positive	R1	0	1070	0	1070			1	7
TCGA-X6	TCGA-X6	SARC	59	FEMALE	ASIAN		Myxofibrosarcoma	2013	-21751	Alive	TUMOR F	583						Positive	R1	0	583	0	583			0	583
TCGA-X6	TCGA-X6	SARC	70	FEMALE	WHITE		Undifferentiated Plec	2013	-25896	Dead	WITH TUMOR		628	Distant M	Lung		71	Positive	R1	1	628	1	628			1	71
TCGA-X6	TCGA-X6	SARC	63	FEMALE	BLACK OR AFRICAN AMERICAN		Leiomyosarcoma (LM	2011	-23350	Alive	TUMOR F	1547						Negative	R0	0	1547	0	1547	0	1547	0	1547
TCGA-X6	TCGA-X6	SARC	55	MALE	WHITE		Myxofibrosarcoma	2011	-20090	Dead	WITH TUMOR		1067	Distant M	Bone	& leg, opp	408	Positive	R1	1	1067	1	1067			1	408
TCGA-X6	TCGA-X6	SARC	24	FEMALE	WHITE		Synovial Sarcoma - E	2013	-9104	Alive	TUMOR F	896						Negative	R0	0	896	0	896	0	896	0	896
TCGA-X9	TCGA-X9	SARC	52	FEMALE	WHITE		Leiomyosarcoma (LM	2013	-19207	Alive	WITH TUI	831		Distant M	Lung		224	Negative	R0	0	831	0	831	1	224	1	224
TCGA-X9	TCGA-X9	SARC	55	MALE	WHITE		Undifferentiated Plec	2011	-20146	Alive	WITH TUI	1108		Distant M	Lung		119	Negative	R0	0	1108	0	1108	1	119	1	119
TCGA-Z4	TCGA-Z4	SARC	24	FEMALE	WHITE		Desmoid Tumor	2013	-9123	Alive	TUMOR F	133						Negative	R0	0	133	0	133	0	133	0	133
TCGA-Z4	TCGA-Z4	SARC	37	MALE	WHITE		Dedifferentiated lipo	2013	-13576	Alive	TUMOR F	407						Negative	R0	0	407	0	407	0	407	0	407
TCGA-Z4	TCGA-Z4	SARC	35	FEMALE	WHITE		Sarcoma; synovial; p	2013	-12959	Alive	TUMOR F	485						Negative	R0	0	485	0	485	0	485	0	485
TCGA-Z4	TCGA-Z4	SARC	64	FEMALE	WHITE		Undifferentiated Plec	2013	-23390	Alive	TUMOR F	486						Negative	R0	0	486	0	486	0	486	0	486
TCGA-3N	TCGA-3N	SKCM	71	MALE	WHITE	Stage IA		2012	-26176	Dead	WITH TUMOR		518	Locoregic	Other, Sp	Axillary	426			1	518	1	518			1	426
TCGA-3N	TCGA-3N	SKCM	82	MALE	WHITE	Stage IIA		2009	-30286	Alive	WITH TUI	2022		Distant M	Lung		1705			0	2022	0	2022			1	1705
TCGA-3N	TCGA-3N	SKCM	82	MALE	WHITE	Stage IIIA		2013	-30163	Dead	WITH TUMOR		395	Distant M	Brain		306			1	395	1	395			1	306
TCGA-BF	TCGA-BF	SKCM	46	FEMALE	WHITE	Stage IIC		2010	-17025	Alive	TUMOR F	387		Distant M	Lung		484			0	387	0	387			1	484
TCGA-BF	TCGA-BF	SKCM	74	FEMALE	WHITE	Stage IIC		2010	-27124	Alive	TUMOR F	14								0	14	0	14			0	14
TCGA-BF	TCGA-BF	SKCM	56	MALE	WHITE	Stage IIIB		2010	-20626	Dead	TUMOR FREE		282							1	282	0	282			0	282
TCGA-BF	TCGA-BF	SKCM	71	FEMALE	WHITE	Stage IIB		2010	-26240	Alive	TUMOR F	853								0	853	0	853			0	853
TCGA-BF	TCGA-BF	SKCM	80	MALE	WHITE	Stage IIC		2010	-29380	Alive	TUMOR F	831								0	831	0	831			0	831
TCGA-BF	TCGA-BF	SKCM	36	FEMALE	WHITE	Stage IIIB		2011	-13332	Alive	TUMOR F	464		Distant M	Liver		217			0	464	0	464			1	217
TCGA-BF	TCGA-BF	SKCM	84	FEMALE	WHITE	Stage IIIB		2011	-30805	Alive	TUMOR F	769								0	769	0	769			0	769
TCGA-BF	TCGA-BF	SKCM	63	MALE	WHITE	Stage IIA		2011	-23076	Alive	TUMOR F	601								0	601	0	601			0	601
TCGA-BF	TCGA-BF	SKCM	81	FEMALE	WHITE	Stage IIIC		2011	-29801	Alive	TUMOR F	717								0	717	0	717			0	717
TCGA-BF	TCGA-BF	SKCM	65	MALE	WHITE	Stage IIC		2012	-23940	Alive	TUMOR F	703								0	703	0	703			0	703
TCGA-BF	TCGA-BF	SKCM	75	FEMALE	WHITE	Stage IIIC		2012	-27622	Alive	TUMOR F	335								0	335	0	335			0	335
TCGA-BF	TCGA-BF	SKCM	63	MALE	WHITE	Stage IIC		2012	-23353	Alive	TUMOR F	323								0	323	0	323			0	323
TCGA-BF	TCGA-BF	SKCM	63	MALE	WHITE	Stage IIC		2012	-23199	Alive	TUMOR F	327								0	327	0	327			0	327
TCGA-BF	TCGA-BF	SKCM	76	FEMALE	WHITE	Stage IIC		2012	-28092	Alive	TUMOR F	490								0	490	0	490			0	490
TCGA-BF	TCGA-BF	SKCM	77	MALE	WHITE	Stage IIC		2013	-28312	Alive	TUMOR F	440								0	440	0	440			0	440
TCGA-BF	TCGA-BF	SKCM	73	FEMALE	WHITE	Stage IIC		2013	-26750	Alive	TUMOR F	476								0	476	0	476			0	476
TCGA-BF	TCGA-BF	SKCM	83	MALE	WHITE	Stage IIC		2013	-30318	Alive	TUMOR F	444								0	444	0	444			0	444
TCGA-BF	TCGA-BF	SKCM	40	FEMALE	WHITE	Stage IV		2013	-14773	Alive	TUMOR F	454								0	454	0	454			0	454
TCGA-BF	TCGA-BF	SKCM	86	MALE	WHITE	Stage IIC		2013	-31666	Alive	TUMOR F	409								0	409	0	409			0	409
TCGA-BF	TCGA-BF	SKCM	62	MALE	WHITE	Stage IIB		2013	-22734	Alive	TUMOR F	405								0	405	0	405			0	405
TCGA-BF	TCGA-BF	SKCM	61	MALE	WHITE	Stage IIC		2013	-22303	Alive	TUMOR F	335								0	335	0	335			0	335
TCGA-BF	TCGA-BF	SKCM	55	MALE	WHITE	Stage III		2013	-20384	Alive	TUMOR F	325								0	325	0	325			0	325
TCGA-BF	TCGA-BF	SKCM	76	FEMALE	WHITE	Stage IIC		2013	-28073	Alive	TUMOR F	318								0	318	0	318			0	318
TCGA-BF	TCGA-BF	SKCM	58	MALE	WHITE	Stage IIC		2013	-21414	Alive	TUMOR F	447								0	447	0	447			0	447
TCGA-D3	TCGA-D3	SKCM	79	FEMALE	WHITE	Stage IIIC		2004	-29212	Dead	WITH TUMOR		504	Locoregional Recurrence			58			1	504	1	504			1	58
TCGA-D3	TCGA-D3	SKCM	64	MALE	WHITE	Stage IIC		2001	-23604	Dead	WITH TUMOR		507							1	507	1	507			1	507
TCGA-D3	TCGA-D3	SKCM	53	FEMALE	WHITE	Stage IIIC		2003	-19687	Alive	TUMOR F	3408								0	3408	0	3408			0	3408
TCGA-D3	TCGA-D3	SKCM	60	MALE	WHITE	I/II NOS		1996	-22200	Dead			3424	Locoregional Recurrence			2452			1	3424		3424			1	2452
TCGA-D3	TCGA-D3	SKCM	55	MALE	WHITE	Stage III		2003	-20230	Dead	TUMOR FREE		2184	Regional lymph node			48			1	2184	0	2184			1	48
TCGA-D3	TCGA-D3	SKCM	42	FEMALE	WHITE	Stage IB		2002	-15531	Alive	TUMOR F	4053		Distant M	Other, Sp	soft tissue	921			0	4053	0	4053			1	921
TCGA-D3	TCGA-D3	SKCM	33	MALE	WHITE	Stage IV		2004	-12330	Dead	WITH TUMOR		854	Distant M	Lung		147			1	854	1	854			1	147
TCGA-D3	TCGA-D3	SKCM	72	MALE	WHITE	Stage IIIB		2005	-26471	Dead	WITH TUMOR		961	Regional lymph node			44			1	961	1	961			1	44
TCGA-D3	TCGA-D3	SKCM	55	MALE	WHITE	Stage IB		2005	-20223	Alive	WITH TUI	2765		Distant M	Lung		1393			0	2765	0	2765			1	1393
TCGA-D3	TCGA-D3	SKCM	55	MALE	WHITE	Stage IB		2005	-20223	Alive	WITH TUI	2765		Distant M	Lung		1393			0	2765	0	2765			1	1393
TCGA-D3	TCGA-D3	SKCM	75	FEMALE	WHITE	Stage III		2005	-27566	Alive	TUMOR F	2912		Locoregional Recurrence			0			0	2912	0	2912			1	0
TCGA-D3	TCGA-D3	SKCM	65	MALE	WHITE	Stage IIB		2002	-24066	Dead	WITH TUMOR		1321	New primary melanoma			448			1	1321	1	1321			1	448
TCGA-D3	TCGA-D3	SKCM	67	MALE	WHITE	Stage IIIC		2003	-24692	Dead	WITH TUMOR		3136	Regional lymph node			13			1	3136	1	3136			1	13
TCGA-D3	TCGA-D3	SKCM	48	MALE	WHITE	Stage IB		2003	-17626	Dead	WITH TUMOR		1992	Locoregional Recurrence			1737			1	1992	1	1992			1	1737
TCGA-D3	TCGA-D3	SKCM	75	MALE	WHITE	Stage IIIC		2003	-27428	Dead	WITH TUMOR		723	Distant M	Lung		231			1	723	1	723			1	231
TCGA-D3	TCGA-D3	SKCM	68	MALE	WHITE	Stage IIIA		2003	-25085	Alive	TUMOR F	3514		Regional lymph node			40			0	3514	0	3514			1	40
TCGA-D3	TCGA-D3	SKCM	70	FEMALE	BLACK OI	Stage 0		1997	-25784	Dead	TUMOR FREE		5110	Locoregional Recurrence			3035										
TCGA-D3	TCGA-D3	SKCM	53	FEMALE	WHITE	Stage III		2006	-19644	Alive	TUMOR F	2639								0	2639	0	2639			0	2639
TCGA-D3	TCGA-D3	SKCM	58	MALE	WHITE	Stage IIIC		2007	-21440	Dead	WITH TUMOR		361	Distant M	Liver		85			1	361	1	361			1	85
TCGA-D3	TCGA-D3	SKCM	75	FEMALE	WHITE	Stage IIIC		2009	-27604	Dead	WITH TUMOR		841				149			1	841	1	841			1	149
TCGA-D3	TCGA-D3	SKCM	74	MALE	WHITE	Stage IA		2008	-27255	Alive	TUMOR F	1888		New primary melanoma			25			0	1888	0	1888			1	25
TCGA-D3	TCGA-D3	SKCM	30	FEMALE	WHITE	Stage IIIA		2001	-11105	Dead	WITH TUMOR		3453	Distant M	Other, Sp	Left ischia	3279			1	3453	1	3453			1	3279
TCGA-D3	TCGA-D3	SKCM	68	MALE	WHITE	Stage IB		2010	-24882	Alive	TUMOR F	1280		Distant M	Brain		569			0	1280	0	1280			1	569
TCGA-D3	TCGA-D3	SKCM	24	MALE	WHITE	Stage IIIC		2005	-8952	Dead	WITH TUMOR		368	Distant M	Lung		261			1	368	1	368			1	261
TCGA-D3	TCGA-D3	SKCM	43	FEMALE	WHITE	I/II NOS		1999	-15710	Alive	TUMOR F	5219		Distant M	Lung		2638			0	5219	0	5219			1	2638
TCGA-D3	TCGA-D3	SKCM	46	FEMALE	WHITE	Stage III		2007	-17128	Dead	TUMOR FREE		2022							1	2022	0	2022			0	2022
TCGA-D3	TCGA-D3	SKCM	50	FEMALE	WHITE	Stage IIIC		2008	-18577	Alive	WITH TUI	2010		Distant M	Other, Sp	Chest wal	50			0	2010	0	2010			1	50
TCGA-D3	TCGA-D3	SKCM	37	MALE	WHITE	Stage IIIC		2008	-13552	Alive	WITH TUI	1812		Distant M	Liver		227			0	1812	0	1812			1	227
TCGA-D3	TCGA-D3	SKCM	63	MALE	WHITE	Stage IIB		2002	-23286	Alive	TUMOR F	3976					2340			0	3976	0	3976			1	2340
TCGA-D3	TCGA-D3	SKCM		MALE	WHITE	I/II NOS		1998		Alive	WITH TUMOR									0		0				0	
TCGA-D3	TCGA-D3	SKCM		FEMALE	WHITE	I/II NOS		1985		Alive	WITH TUMOR									0		0				0	
TCGA-D3	TCGA-D3	SKCM	54	FEMALE	WHITE	Stage IB		2006	-19858	Dead	WITH TUMOR		1766	Distant M	Lung		1441			1	1766	1	1766			1	1441
TCGA-D3	TCGA-D3	SKCM	57	FEMALE	WHITE	Stage III		2009	-20979	Alive	TUMOR F	1429								0	1429	0	1429			0	1429
TCGA-D3	TCGA-D3	SKCM	58	FEMALE	WHITE	Stage IIIC		2009	-21281	Alive	WITH TUI	1409		Distant M	Other, Sp	Lymph nc	55			0	1409	0	1409			1	55
TCGA-D3	TCGA-D3	SKCM	39	MALE	WHITE	I/II NOS		1999	-14563	Alive	TUMOR F	5065								0	5065	0	5065			0	5065
TCGA-D3	TCGA-D3	SKCM	69	FEMALE	WHITE	Stage IIC		2005	-25396	Alive	WITH TUI	2644		Locoregional Recurrence			96			0	2644	0	2644			1	96
TCGA-D3	TCGA-D3	SKCM	74	FEMALE	WHITE	Stage III		2001	-27355	Dead	WITH TUMOR		1832	Regional lymph node			0			1	1832	1	1832			1	0
TCGA-D3	TCGA-D3	SKCM	61	FEMALE	WHITE	Stage IIIC		2009	-22409	Dead	WITH TUMOR		746	Distant M	Bone		129			1	746	1	746			1	129
TCGA-D3	TCGA-D3	SKCM	70	MALE	WHITE	Stage IIIA		2003	-25643	Dead	WITH TUMOR		422	Regional lymph node			32			1	422	1	422			1	32
TCGA-D3	TCGA-D3	SKCM	47	MALE	WHITE	Stage III		2009	-17220	Dead	WITH TUMOR		284	Distant M	Lung		47			1	284	1	284			1	47
TCGA-D3	TCGA-D3	SKCM	42	MALE	WHITE	Stage III		2004	-15695	Alive	TUMOR F	3151					3108			0	3151	0	3151			1	3108
TCGA-D3	TCGA-D3	SKCM	53	MALE	WHITE	Stage IIIA		2010	-19423	Alive	WITH TUI	1209		Distant M	Brain		750			0	1209	0	1209			1	750
TCGA-D3	TCGA-D3	SKCM	38	FEMALE	WHITE	Stage IIIB		2010	-14236	Alive	TUMOR F	1378		Regional lymph node			16			0	1378	0	1378			1	16
TCGA-D3	TCGA-D3	SKCM	39	FEMALE	WHITE	I/II NOS		1999	-14305	Alive	TUMOR F	5318								0	5318	0	5318			0	5318
TCGA-D3	TCGA-D3	SKCM	51	MALE	WHITE	Stage IIIC		2009	-18776	Alive	TUMOR F	1695								0	1695	0	1695			0	1695
TCGA-D3	TCGA-D3	SKCM		MALE	WHITE	Stage 0		1990		Alive	TUMOR FREE																
TCGA-D3	TCGA-D3	SKCM	60	MALE	WHITE	Stage IIIC		2009	-22119	Alive	TUMOR F	1714		Regional lymph node			838			0	1714	0	1714			1	838
TCGA-D3	TCGA-D3	SKCM	19	MALE	WHITE	Stage III		2001	-7194	Alive	TUMOR F	4414		Regional lymph node			2008			0	4414	0	4414			1	2008
TCGA-D3	TCGA-D3	SKCM	51	MALE	WHITE	Stage IIIB		2011	-18971	Alive	TUMOR F	1002								0	1002	0	1002			0	1002
TCGA-D3	TCGA-D3	SKCM	56	FEMALE	WHITE	Stage IV		2012	-20559	Alive	WITH TUI	688		Distant M	Other, Sp	Adrenal G	18			0	688	0	688			1	18
TCGA-D3	TCGA-D3	SKCM	60	MALE	WHITE	Stage IIA		2008	-22040	Alive	WITH TUI	1941		Regional lymph node			1016			0	1941	0	1941			1	1016
TCGA-D3	TCGA-D3	SKCM	59	FEMALE	WHITE	Stage IIIC		2011	-21823	Alive	TUMOR F	818		New primary melanoma			210			0	818	0	818			1	210
TCGA-D3	TCGA-D3	SKCM	74	MALE	WHITE	Stage IB		2003	-27095	Alive	TUMOR F	3826		Distant M	Other, Sp	Large Inte	1092			0	3826	0	3826			1	1092
TCGA-D3	TCGA-D3	SKCM	15	FEMALE	WHITE	Stage I		2001	-5684	Alive	TUMOR F	4129		New primary melanoma			1279			0	4129	0	4129			1	1279
TCGA-D3	TCGA-D3	SKCM	61	MALE	WHITE	Stage II		2001	-22287	Alive	TUMOR F	4195					757			0	4195	0	4195			1	757
TCGA-D3	TCGA-D3	SKCM	23	FEMALE	WHITE	Stage III		1998	-8637	Alive	WITH TUI	5424		Regional lymph node			0			0	5424	0	5424			1	0
TCGA-D3	TCGA-D3	SKCM	58	MALE	WHITE	Stage IV		2012	-21379	Alive	WITH TUI	553		Distant M	Bone		44			0	553	0	553			1	44
TCGA-D3	TCGA-D3	SKCM	43	MALE	WHITE	Stage IIIC		2012	-15935	Alive	WITH TUI	487		Distant M	Lung|Live	[Not Appl	291			0	487	0	487			1	291
TCGA-D3	TCGA-D3	SKCM	36	MALE	WHITE	Stage IB		2002	-13296	Alive	WITH TUI	3808		Distant M	Bone		3074			0	3808	0	3808			1	3074
TCGA-D3	TCGA-D3	SKCM	48	MALE	WHITE	Stage IIIB		2003	-17871	Dead	WITH TUMOR		938	Distant M	Other, Sp	Soft tissue	347			1	938	1	938			1	347
TCGA-D3	TCGA-D3	SKCM	48	MALE	WHITE	Stage IIIC		2000	-17668	Dead	WITH TUMOR		2421	Distant M	Other, Sp	Supraclav	1859			1	2421	1	2421			1	1859
TCGA-D3	TCGA-D3	SKCM	63	FEMALE	WHITE	Stage IIIC		2012	-23109	Alive	TUMOR F	718		Distant M	Other, Sp	Retroperit	147			0	718	0	718			1	147
TCGA-D3	TCGA-D3	SKCM	26	MALE	WHITE	Stage IV		2012	-9517	Alive	WITH TUI	804		Distant M	Lung		59			0	804	0	804			1	59
TCGA-D3	TCGA-D3	SKCM	68	MALE	WHITE	Stage IA		2008	-25069	Dead	WITH TUMOR		1780	New primary melanoma			50			1	1780	1	1780			1	50
TCGA-D3	TCGA-D3	SKCM	18	MALE	WHITE	Stage II		1994	-6684	Alive	TUMOR F	7342		Distant M	Brain		1443			0	7342	0	7342			1	1443
TCGA-D3	TCGA-D3	SKCM	45	MALE	WHITE	Stage IIA		2000	-16569	Alive	TUMOR F	5177		Locoregional Recurrence			1555			0	5177	0	5177			1	1555
TCGA-D3	TCGA-D3	SKCM	43	MALE	WHITE	Stage IIIB		1996	-15947	Dead	WITH TUMOR		2711	Regional lymph node			1333			1	2711	1	2711			1	1333
TCGA-D3	TCGA-D3	SKCM	73	MALE	WHITE	Stage IIB		2005	-26782	Dead	TUMOR FREE		3259							1	3259	0	3259			0	3259
TCGA-D3	TCGA-D3	SKCM	27	FEMALE	WHITE	I/II NOS		2000	-10185	Alive	TUMOR F	4897		Distant M	Lung		787			0	4897	0	4897			1	787
TCGA-D3	TCGA-D3	SKCM		FEMALE	WHITE	I/II NOS		1996		Dead	WITH TUMOR									1		1				1	
TCGA-D3	TCGA-D3	SKCM	77	MALE	WHITE	Stage III		2002	-28234	Alive	TUMOR F	4646		Locoregional Recurrence			535			0	4646	0	4646			1	535
TCGA-D3	TCGA-D3	SKCM	66	MALE	WHITE	Stage II		2004	-24348	Dead	WITH TUMOR		884	Distant M	Liver		578			1	884	1	884			1	578
TCGA-D3	TCGA-D3	SKCM	54	FEMALE	WHITE	Stage 0		1999	-19973	Dead	WITH TUMOR		3943	Locoregional Recurrence			1720										
TCGA-D3	TCGA-D3	SKCM	52	MALE	WHITE	Stage I		1997	-19127	Dead	WITH TUMOR		3564	Regional lymph node			2641			1	3564	1	3564			1	2641
TCGA-D3	TCGA-D3	SKCM	25	MALE	WHITE	I/II NOS		1993	-9344	Dead	WITH TUMOR		5101	Regional lymph node			3896			1	5101	1	5101			1	3896
TCGA-D9	TCGA-D9	SKCM	40	MALE	WHITE			1999	-14805	Alive	WITH TUI	4609		Distant Metastasis			4287			0	4609	0	4609			1	4287
TCGA-D9	TCGA-D9	SKCM	65	FEMALE	WHITE			2007	-23779	Alive	TUMOR F	1663		New prim	Other, Specify		1290			0	1663	0	1663			1	1290
TCGA-D9	TCGA-D9	SKCM	82	MALE	WHITE			2010	-30142	Alive	TUMOR F	111		Distant M	Other, Specify		10			0	111	0	111			1	10
TCGA-D9	TCGA-D9	SKCM	80	FEMALE	WHITE			2010	-29323	Dead	WITH TUMOR		216	Distant M	Other, Sp	iliac nymp	195			1	216	1	216			1	195
TCGA-D9	TCGA-D9	SKCM	63	MALE	WHITE			2010	-23171	Alive	WITH TUI	551								0	551	0	551			0	551
TCGA-D9	TCGA-D9	SKCM	63	MALE	WHITE			2010	-23171	Alive	WITH TUI	551								0	551	0	551			0	551
TCGA-D9	TCGA-D9	SKCM	66	MALE	WHITE	Stage IIIC		2011	-24192	Dead	WITH TUMOR		468	Locoregic	Lung		335			1	468	1	468			1	335
TCGA-D9	TCGA-D9	SKCM	39	FEMALE	WHITE	Stage IIIB		2012	-14489	Alive	TUMOR F	678								0	678	0	678			0	678
TCGA-D9	TCGA-D9	SKCM	54	MALE	WHITE	Stage IIIC		2012	-19749	Dead	WITH TUMOR		519	Regional I	Other, Sp	lymph no	192			1	519	1	519			1	192
TCGA-D9	TCGA-D9	SKCM	50	MALE	WHITE	Stage IIIC		2012	-18462	Dead	WITH TUMOR		190	Distant M	Lung		80			1	190	1	190			1	80

TCGA-D9	TCGA-D9	SKCM	73	FEMALE	WHITE	Stage IIIC	2012	-26812	Alive	TUMOR F	505		Locoregional Recurrence	100	0	505	0	505	1	100
TCGA-D9	TCGA-D9	SKCM	68	MALE	WHITE	Stage IIB	2012	-24979	Alive	TUMOR F	218				0	218	0	218	0	218
TCGA-D9	TCGA-D9	SKCM	54	MALE	WHITE	Stage IIIC	2012	-20072	Dead	WITH TUMOR		561	Distant M Brain	213	1	561	1	561	1	213
TCGA-D9	TCGA-D9	SKCM	75	FEMALE	WHITE	Stage IIIA	2013	-27537	Alive	TUMOR F	301				0	301	0	301	0	301
TCGA-D9	TCGA-D9	SKCM	70	MALE	WHITE	Stage IIIC	2012	-25582	Alive	TUMOR F	766				0	766	0	766	0	766
TCGA-D9	TCGA-D9	SKCM	56	MALE	WHITE	Stage IIIA	2008	-20493	Alive	TUMOR F	2359				0	2359	0	2359	0	2359
TCGA-D9	TCGA-D9	SKCM	56	MALE	WHITE	Stage IIIA	2012	-20570	Dead			698	Distant M Bone	398	1	698		698	1	398
TCGA-DA	TCGA-DA	SKCM	75	FEMALE	WHITE	Stage IIIB	2008	-27553	Alive	TUMOR F	2329		Distant M Other, Sp mediastin	866	0	2329	0	2329	1	866
TCGA-DA	TCGA-DA	SKCM	37	FEMALE	WHITE	Stage IIIB	2009	-13585	Alive	TUMOR F	820			852	0	820	0	820	1	852
TCGA-DA	TCGA-DA	SKCM	42	MALE	WHITE	Stage III	2002	-15357	Alive	TUMOR F	4407				0	4407	0	4407	0	4407
TCGA-DA	TCGA-DA	SKCM	63	MALE	WHITE	Stage IV	2008	-23157	Dead	WITH TUMOR		620	Distant M Brain	599	1	620	1	620	1	599
TCGA-DA	TCGA-DA	SKCM	55	MALE	WHITE	Stage III	1996	-20399	Alive	TUMOR F	6768		Distant M Other, Sp Jejunum	5591	0	6768	0	6768	1	5591
TCGA-DA	TCGA-DA	SKCM	45	MALE	WHITE	Stage III	1996	-16701	Dead	WITH TUMOR		5370	Regional lymph node	4679	1	5370	1	5370	1	4679
TCGA-DA	TCGA-DA	SKCM	51	MALE	WHITE	Stage IIIC	2006	-18961	Dead	WITH TUMOR		1093	Distant M Brain	889	1	1093	1	1093	1	889
TCGA-DA	TCGA-DA	SKCM	27	FEMALE	WHITE	Stage IV	2003	-10017	Alive	TUMOR F	4107				0	4107	0	4107	0	4107
TCGA-DA	TCGA-DA	SKCM	62	MALE	WHITE	Stage IIIB	2007	-22940	Alive	WITH TUI	2703		Distant M Other, Sp skin scrot	755	0	2703	0	2703	1	755
TCGA-DA	TCGA-DA	SKCM	63	FEMALE	WHITE	Stage IIC	2005	-23286	Dead	WITH TUMOR		1640	Regional lymph node	961	1	1640	1	1640	1	961
TCGA-DA	TCGA-DA	SKCM	32	FEMALE	WHITE	Stage IIIB	2005	-11744	Dead	WITH TUMOR		2005			1	2005	1	2005	1	2005
TCGA-DA	TCGA-DA	SKCM	69	FEMALE	WHITE	Stage IIIC	2010	-25219	Alive	WITH TUI	825		Distant M Brain	243	0	825	0	825	1	243
TCGA-DA	TCGA-DA	SKCM	81	MALE	WHITE	Stage IIIB	2005	-29859	Dead	WITH TUMOR		2071	Distant M Lung	1930	1	2071	1	2071	1	1930
TCGA-DA	TCGA-DA	SKCM	55	MALE	WHITE	Stage IIIB	2010	-20365	Dead	WITH TUMOR		1032	Distant M Other, Sp peritonea	430	1	1032	1	1032	1	430
TCGA-DA	TCGA-DA	SKCM	52	MALE	WHITE	Stage IIIB	2010	-19330	Dead	WITH TUMOR		319			1	319	1	319	1	319
TCGA-DA	TCGA-DA	SKCM	45	MALE	WHITE	Stage I	1994	-16701	Dead	WITH TUMOR		6873	Distant M Lung	6841	1	6873	1	6873	1	6841
TCGA-DA	TCGA-DA	SKCM	39	MALE	WHITE	Stage IIIB	2011	-14327	Alive	TUMOR F	1319				0	1319	0	1319	0	1319
TCGA-DA	TCGA-DA	SKCM	83	FEMALE	WHITE	Stage IIC	2009	-30623	Alive	TUMOR F	2193				0	2193	0	2193	0	2193
TCGA-DA	TCGA-DA	SKCM	52	MALE	WHITE	Stage IIIB	2011	-19105	Alive	TUMOR F	1136				0	1136	0	1136	0	1136
TCGA-DA	TCGA-DA	SKCM	62	MALE	WHITE	Stage IB	2009	-22718	Alive	TUMOR F	2249				0	2249	0	2249	0	2249
TCGA-DA	TCGA-DA	SKCM	68	MALE	WHITE	Stage IIC	2012	-25083	Dead	WITH TUMOR		430	Distant M Lung	338	1	430	1	430	1	338
TCGA-DA	TCGA-DA	SKCM	87	MALE	WHITE	Stage IV	2013	-31837	Alive	TUMOR F	396				0	396	0	396	0	396
TCGA-DA	TCGA-DA	SKCM	73	MALE	WHITE	Stage IIB	2012	-26708	Alive	TUMOR F	804				0	804	0	804	0	804
TCGA-EB	TCGA-EB	SKCM	48	MALE	WHITE	Stage IIC	2010	-17569	Alive	TUMOR F	1039				0	1039	0	1039	0	1039
TCGA-EB	TCGA-EB	SKCM	56	MALE	WHITE		2011	-20539	Alive	TUMOR F	632		Regional lymph node	465	0	632	0	632	1	465
TCGA-EB	TCGA-EB	SKCM	72	MALE	WHITE	Stage IIIB	2011	-26439	Alive	TUMOR F	645		Regional lymph node	500	0	645	0	645	1	500
TCGA-EB	TCGA-EB	SKCM	63	MALE	WHITE	Stage IIA	2011	-23322	Alive	TUMOR F	378				0	378	0	378	0	378
TCGA-EB	TCGA-EB	SKCM	37	MALE	WHITE	Stage IIC	2011	-13712	Alive	WITH TUI	39				0	39	0	39	0	39
TCGA-EB	TCGA-EB	SKCM	63	MALE	WHITE	Stage II	2011	-23091	Alive	TUMOR F	796				0	796	0	796	0	796
TCGA-EB	TCGA-EB	SKCM	74	MALE	WHITE	Stage IIC	2012	-27124	Alive	TUMOR F	650		Distant M Other, Sp Chest wal	74	0	650	0	650	1	74
TCGA-EB	TCGA-EB	SKCM	53	FEMALE	WHITE	Stage IIC	2011	-19431	Alive	TUMOR F	1160				0	1160	0	1160	0	1160
TCGA-EB	TCGA-EB	SKCM	77	FEMALE	WHITE	Stage IIA	2012	-28148	Alive	TUMOR F	180				0	180	0	180	0	180
TCGA-EB	TCGA-EB	SKCM	57	MALE	WHITE	Stage IIC	2012	-20910	Alive	TUMOR F	278				0	278	0	278	0	278
TCGA-EB	TCGA-EB	SKCM	56	FEMALE	WHITE	Stage IIC	2012	-20605	Alive	TUMOR F	126				0	126	0	126	0	126
TCGA-EB	TCGA-EB	SKCM	86	FEMALE	WHITE	Stage IIIB	2012	-31622	Dead	TUMOR FREE		326			1	326	0	326	0	326
TCGA-EB	TCGA-EB	SKCM	90	MALE	WHITE	Stage IIC	2012	-32872	Alive	TUMOR F	262				0	262	0	262	0	262
TCGA-EB	TCGA-EB	SKCM	76	FEMALE	WHITE	Stage IIC	2012	-27998	Alive	TUMOR F	291				0	291	0	291	0	291
TCGA-EB	TCGA-EB	SKCM	73	FEMALE	ASIAN	Stage IIC	2012	-26712	Dead	WITH TUMOR		721	Regional lymph node	721	1	721	1	721	1	721
TCGA-EB	TCGA-EB	SKCM	49	MALE	ASIAN	Stage IIIC	2012	-18157	Alive	TUMOR F	441				0	441	0	441	0	441
TCGA-EB	TCGA-EB	SKCM	83	MALE	WHITE	Stage IIC	2012	-30344	Alive	TUMOR F	-2				0	0	0	0	0	0
TCGA-EB	TCGA-EB	SKCM	34	MALE	WHITE	Stage IIC	2012	-12484	Alive	TUMOR F	568				0	568	0	568	0	568
TCGA-EB	TCGA-EB	SKCM	59	MALE	WHITE	Stage IIC	2012	-21623	Dead	WITH TUMOR		205	Regional [Not Avai [Not Appl	115	1	205	1	205	1	115
TCGA-EB	TCGA-EB	SKCM	69	MALE	WHITE	Stage IIB	2012	-25537	Alive	WITH TUI	81		Regional lymph node	60	0	81	0	81	1	60
TCGA-EB	TCGA-EB	SKCM	58	FEMALE	WHITE	Stage IIC	2012	-21519	Alive	TUMOR F	741				0	741	0	741	0	741
TCGA-EB	TCGA-EB	SKCM	51	FEMALE	ASIAN	Stage IIIC	2012	-18910	Alive	TUMOR F	422				0	422	0	422	0	422
TCGA-EB	TCGA-EB	SKCM	52	MALE	ASIAN	Stage IIIB	2011	-19298	Dead	TUMOR FREE		315			1	315	0	315	0	315
TCGA-EB	TCGA-EB	SKCM	42	FEMALE	ASIAN	Stage IIIB	2012	-15516	Dead	TUMOR FREE		636	Locoregic [Not Avai [Not Appl	337	1	636	0	636	1	337
TCGA-EB	TCGA-EB	SKCM	77	MALE	WHITE	Stage IIB	2012	-28315	Alive	TUMOR F	774				0	774	0	774	0	774
TCGA-EB	TCGA-EB	SKCM	65	FEMALE	WHITE	Stage IIIB	2012	-23773	Alive	TUMOR F	977				0	977	0	977	0	977
TCGA-EB	TCGA-EB	SKCM	41	FEMALE	WHITE	Stage IIIC	2012	-15337	Alive	TUMOR F	620				0	620	0	620	0	620
TCGA-EB	TCGA-EB	SKCM	82	MALE	WHITE	Stage IIC	2012	-30225	Dead	TUMOR FREE		326			1	326	0	326	0	326
TCGA-EB	TCGA-EB	SKCM	56	FEMALE	WHITE	Stage IIC	2012	-20486	Alive	TUMOR F	777				0	777	0	777	0	777
TCGA-EB	TCGA-EB	SKCM	53	MALE	WHITE	Stage IIC	2012	-19645	Alive	TUMOR F	931				0	931	0	931	0	931
TCGA-EB	TCGA-EB	SKCM	75	FEMALE	WHITE	Stage IIC	2012	-27556	Dead	TUMOR FREE		264			1	264	0	264	0	264
TCGA-EB	TCGA-EB	SKCM	78	FEMALE	WHITE	Stage IIIC	2012	-28671	Alive	TUMOR F	590				0	590	0	590	0	590
TCGA-EB	TCGA-EB	SKCM	62	MALE	WHITE	Stage IIC	2012	-22972	Alive	TUMOR F	226				0	226	0	226	0	226
TCGA-EB	TCGA-EB	SKCM	56	MALE	ASIAN	Stage IIIB	2012	-20698	Dead	TUMOR FREE		472			1	472	0	472	0	472
TCGA-EB	TCGA-EB	SKCM	65	FEMALE	WHITE	Stage IV	2012	-23833	Dead	WITH TUMOR		454	Regional [Not Avai [Not Appl	24	1	454	1	454	1	24
TCGA-EB	TCGA-EB	SKCM	55	MALE	ASIAN	Stage III	2011	-20424	Dead	TUMOR FREE		619			1	619	0	619	0	619
TCGA-EB	TCGA-EB	SKCM	73	MALE	WHITE	Stage IIB	2012	-26965	Dead	WITH TUMOR		401	Distant M Brain	367	1	401	1	401	1	367
TCGA-EB	TCGA-EB	SKCM	78	FEMALE	WHITE	Stage IIC	2012	-28772	Dead	TUMOR FREE		369			1	369	0	369	0	369
TCGA-EB	TCGA-EB	SKCM	57	FEMALE	WHITE		2008	-20978	Alive	TUMOR F	2076		Locoregional Recurrence	1372	0	2076	0	2076	1	1372
TCGA-EB	TCGA-EB	SKCM	60	FEMALE	WHITE	Stage III	2009	-21920	Alive	TUMOR F	1643		Locoregional Recurrence	1065	0	1643	0	1643	1	1065
TCGA-EB	TCGA-EB	SKCM	71	MALE	WHITE	Stage III	2012	-26058	Alive	TUMOR F	891				0	891	0	891	0	891
TCGA-EB	TCGA-EB	SKCM	48	FEMALE	WHITE	Stage IIC	2012	-17867	Alive	TUMOR F	779				0	779	0	779	0	779
TCGA-EB	TCGA-EB	SKCM	49	MALE	WHITE	Stage IIC	2009	-18226	Alive		1792		Distant M Brain	1689	0	1792	0	1792	1	1689
TCGA-EB	TCGA-EB	SKCM	56	MALE	ASIAN	Stage IIIB	2012	-20610	Dead	WITH TUMOR		321	Locoregional Recurrence	217	1	321	1	321	1	217
TCGA-EB	TCGA-EB	SKCM	74	FEMALE	WHITE	Stage IIIB	2012	-27364	Alive	TUMOR F	214				0	214	0	214	0	214
TCGA-EB	TCGA-EB	SKCM	55	MALE	ASIAN	Stage IIIC	2012	-20172	Alive	TUMOR F	1109				0	1109	0	1109	0	1109
TCGA-EB	TCGA-EB	SKCM	71	MALE	WHITE	Stage IIC	2013	-26216	Alive	TUMOR F	382		Regional lymph node	36	0	382	0	382	1	36
TCGA-EB	TCGA-EB	SKCM	76	FEMALE	WHITE	Stage IIA	2013	-28078	Dead	WITH TUMOR		352	Regional [Not Avai [Not Appl	129	1	352	1	352	1	129
TCGA-EB	TCGA-EB	SKCM	58	FEMALE	WHITE	Stage IIC	2012	-21367	Dead	TUMOR FREE		608			1	608	0	608	0	608
TCGA-EB	TCGA-EB	SKCM	58	FEMALE	ASIAN	Stage III	2013	-21239	Alive	TUMOR F	390				0	390	0	390	0	390
TCGA-EB	TCGA-EB	SKCM	70	FEMALE	ASIAN	Stage IIC	2013	-25850	Alive	TUMOR F	17				0	17	0	17	0	17
TCGA-EB	TCGA-EB	SKCM	66	MALE	WHITE	Stage IIC	2013	-24408	Alive	TUMOR F	362				0	362	0	362	0	362
TCGA-EB	TCGA-EB	SKCM	66	FEMALE	WHITE	Stage IIB	2013	-24263	Alive	TUMOR F	360		Regional lymph node	138	0	360	0	360	1	138
TCGA-EB	TCGA-EB	SKCM	66	MALE	WHITE	Stage IIC	2013	-24228	Alive	TUMOR F	414				0	414	0	414	0	414
TCGA-EE	TCGA-EE	SKCM	54	MALE	WHITE	Stage IA	2006	-20021	Dead	WITH TUMOR		907	Locoregional Recurrence	531	1	907	1	907	1	531
TCGA-EE	TCGA-EE	SKCM	69	MALE	ASIAN	Stage IIIB	2007	-25281	Dead	WITH TUMOR		828	Regional lymph node	317	1	828	1	828	1	317
TCGA-EE	TCGA-EE	SKCM	57	MALE	WHITE	Stage IIB	2008	-20970	Dead	WITH TUMOR		263	Regional lymph node	138	1	263	1	263	1	138
TCGA-EE	TCGA-EE	SKCM	69	MALE	WHITE	Stage III	2001	-25523	Dead	WITH TUMOR		2889	New primary melanoma	850	1	2889	1	2889	1	850
TCGA-EE	TCGA-EE	SKCM	82	FEMALE	WHITE	Stage II	2005	-30003	Dead	TUMOR FREE		1026	Locoregional Recurrence	788	1	1026	0	1026	1	788
TCGA-EE	TCGA-EE	SKCM	84	FEMALE	WHITE	Stage IIIC	2008	-30718	Dead	WITH TUMOR		447	Locoregional Recurrence	270	1	447	1	447	1	270
TCGA-EE	TCGA-EE	SKCM	48	MALE	WHITE	Stage 0	2007	-17788	Dead	WITH TUMOR		818	Locoregional Recurrence	450						
TCGA-EE	TCGA-EE	SKCM	72	MALE	WHITE	Stage IB	2003	-26447	Dead			2073	Distant M Other, Sp Right low	1309	1	2073		2073	1	1309
TCGA-EE	TCGA-EE	SKCM	55	FEMALE	WHITE	Stage IIIC	2008	-20235	Dead	WITH TUMOR		151	Regional lymph node	85	1	151	1	151	1	85
TCGA-EE	TCGA-EE	SKCM	66	FEMALE	WHITE	Stage II	2002	-24445	Alive	TUMOR F	4070				0	4070	0	4070	0	4070
TCGA-EE	TCGA-EE	SKCM	59	MALE	WHITE	Stage 0	1997	-21660	Dead	WITH TUMOR		4601	New primary melanoma	142						
TCGA-EE	TCGA-EE	SKCM	53	MALE	WHITE	Stage I	2002	-19372	Alive	TUMOR F	2785				0	2785	0	2785	0	2785
TCGA-EE	TCGA-EE	SKCM	56	MALE	WHITE	Stage I	1995	-20476	Dead	WITH TUMOR		5118	Regional lymph node	4667	1	5118	1	5118	1	4667
TCGA-EE	TCGA-EE	SKCM	79	MALE	WHITE	Stage IV	2008	-28883	Dead	WITH TUMOR		412	Distant M Brain	53	1	412	1	412	1	53
TCGA-EE	TCGA-EE	SKCM	68	MALE	WHITE	Stage IIIA	2003	-25095	Dead	WITH TUMOR		1927	Locoregional Recurrence	867	1	1927	1	1927	1	867
TCGA-EE	TCGA-EE	SKCM	67	MALE	WHITE	Stage IIB	2004	-24618	Dead	WITH TUMOR		2588	New primary melanoma	490	1	2588	1	2588	1	490
TCGA-EE	TCGA-EE	SKCM	20	MALE	WHITE	Stage IB	2007	-7510	Dead	WITH TUMOR		2402	Regional lymph node	204	1	2402	1	2402	1	204
TCGA-EE	TCGA-EE	SKCM	87	MALE	WHITE	Stage IIIC	2008	-31790	Dead			425			1	425		425	0	425
TCGA-EE	TCGA-EE	SKCM	54	MALE	WHITE	Stage IIIB	2008	-19961	Alive	TUMOR F	1940				0	1940	0	1940	0	1940
TCGA-EE	TCGA-EE	SKCM	53	MALE	WHITE	Stage IIIA	2003	-19664	Dead	WITH TUMOR		2192	Locoregional Recurrence	726	1	2192	1	2192	1	726
TCGA-EE	TCGA-EE	SKCM	59	FEMALE	WHITE	Stage IA	2008	-21552	Alive	TUMOR F	1966		Distant M Other, Sp Perihilar l	1231	0	1966	0	1966	1	1231
TCGA-EE	TCGA-EE	SKCM	78	MALE	WHITE	Stage IIIC	2009	-28824	Dead			79	Distant M Brain	55	1	79		79	1	55
TCGA-EE	TCGA-EE	SKCM	33	FEMALE	WHITE	Stage IB	2007	-12350	Alive	TUMOR F	1729				0	1729	0	1729	0	1729
TCGA-EE	TCGA-EE	SKCM	78	MALE	WHITE	I/II NOS	2009	-28805	Dead	WITH TUMOR		566	New primary melanoma	478	1	566	1	566	1	478
TCGA-EE	TCGA-EE	SKCM	73	FEMALE	WHITE	Stage IIC	2006	-26899	Alive	WITH TUI	1716		Distant M Lung	1668	0	1716	0	1716	1	1668
TCGA-EE	TCGA-EE	SKCM	70	FEMALE	WHITE	Stage IIB	2007	-25847	Dead	WITH TUMOR		2030	Regional [Not Avai [Not Appl	1256	1	2030	1	2030	1	1256
TCGA-EE	TCGA-EE	SKCM	48	FEMALE	WHITE	Stage IIIC	2010	-17857	Alive	WITH TUI	440		Distant M Lung	426	0	440	0	440	1	426
TCGA-EE	TCGA-EE	SKCM	79	MALE	WHITE	Stage IIA	2006	-29087	Dead	WITH TUMOR		1864	Locoregional Recurrence	1418	1	1864	1	1864	1	1418
TCGA-EE	TCGA-EE	SKCM	51	FEMALE	WHITE		1982	-18779	Alive	TUMOR F	11252		Regional lymph node	7483	0	11252	0	11252	1	7483
TCGA-EE	TCGA-EE	SKCM	85	MALE	WHITE	Stage IIIC	2008	-31092	Dead	WITH TUMOR		787	Locoregional Recurrence	96	1	787	1	787	1	96
TCGA-EE	TCGA-EE	SKCM	42	MALE	WHITE	Stage 0	1997	-15396	Alive	TUMOR F	5932		New primary melanoma	4408						
TCGA-EE	TCGA-EE	SKCM	58	FEMALE	WHITE	Stage IB	2009	-21363	Dead	WITH TUMOR		545	Regional lymph node	391	1	545	1	545	1	391
TCGA-EE	TCGA-EE	SKCM	77	FEMALE	WHITE	Stage IIA	2006	-28319	Dead	WITH TUMOR		1424	Distant M Other, Sp Left Inner	1247	1	1424	1	1424	1	1247
TCGA-EE	TCGA-EE	SKCM	46	MALE	WHITE	Stage IB	2004	-17109	Alive	TUMOR F	3527		Regional lymph node	3488	0	3527	0	3527	1	3488
TCGA-EE	TCGA-EE	SKCM	71	MALE	WHITE	Stage IIIC	2009	-26048	Alive	TUMOR F	1814				0	1814	0	1814	0	1814
TCGA-EE	TCGA-EE	SKCM	43	MALE	WHITE	Stage IB	2007	-16041	Dead	WITH TUMOR		1195	Regional lymph node	1028	1	1195	1	1195	1	1028
TCGA-EE	TCGA-EE	SKCM	43	MALE	WHITE	Stage IA	2006	-15953	Alive	TUMOR F	2620		New primary melanoma	1505	0	2620	0	2620	1	1505
TCGA-EE	TCGA-EE	SKCM	51	MALE	WHITE	Stage IIIB	2007	-18876	Alive	TUMOR F	1803				0	1803	0	1803	0	1803
TCGA-EE	TCGA-EE	SKCM	82	MALE	WHITE	Stage IIB	2008	-30084	Alive	TUMOR F	2051				0	2051	0	2051	0	2051
TCGA-EE	TCGA-EE	SKCM	58	FEMALE	WHITE	Stage IIB	1984	-21526	Dead	WITH TUMOR		10346	Locoregional Recurrence	3689	1	10346	1	10346	1	3689
TCGA-EE	TCGA-EE	SKCM	44	MALE	WHITE	Stage I	1999	-16353	Alive	TUMOR F	5286		[Not Avai [Not Avai [Not Appl	5276	0	5286	0	5286	1	5276
TCGA-EE	TCGA-EE	SKCM	34	MALE	WHITE	Stage I	1996	-12727	Alive	TUMOR F	6699				0	6699	0	6699	0	6699
TCGA-EE	TCGA-EE	SKCM	39	MALE	WHITE	Stage IA	2008	-14498	Alive	TUMOR F	1482				0	1482	0	1482	0	1482
TCGA-EE	TCGA-EE	SKCM	83	MALE	WHITE	Stage IA	2004	-30602	Dead	WITH TUMOR		3266	New primary melanoma	733	1	3266	1	3266	1	733
TCGA-EE	TCGA-EE	SKCM	46	FEMALE	WHITE	Stage I	2007	-16879	Alive	TUMOR F	1665			1498	0	1665	0	1665	1	1498
TCGA-EE	TCGA-EE	SKCM	40	FEMALE	WHITE	Stage IIA	2006	-14701	Alive	WITH TUI	2423		Distant M Lung|Oth [Not Appl	2329	0	2423	0	2423	1	2329
TCGA-EE	TCGA-EE	SKCM	70	FEMALE	WHITE	Stage IIC	2004	-25711	Alive	TUMOR F	2296		Locoregional Recurrence	1218	0	2296	0	2296	1	1218
TCGA-EE	TCGA-EE	SKCM	67	MALE	WHITE	Stage IIA	2003	-24683	Dead	WITH TUMOR		3106	Distant M Other, Sp adrenal gl	2422	1	3106	1	3106	1	2422
TCGA-EE	TCGA-EE	SKCM	66	FEMALE	WHITE	Stage II	2002	-24159	Alive	TUMOR F	3857		Regional lymph node	2216	0	3857	0	3857	1	2216
TCGA-EE	TCGA-EE	SKCM	80	MALE	WHITE	Stage IIIB	2009	-29501	Dead	WITH TUMOR		423	Distant M Lung	251	1	423	1	423	1	251
TCGA-EE	TCGA-EE	SKCM	78	MALE	WHITE	Stage II	2009	-28510	Dead	WITH TUMOR		1301			1	1301	1	1301	1	1301
TCGA-EE	TCGA-EE	SKCM	28	FEMALE	WHITE	Stage IB	2006	-10421	Dead	WITH TUMOR		2470	Distant M Other, Sp right butt	1565	1	2470	1	2470	1	1565
TCGA-EE	TCGA-EE	SKCM	77	MALE	WHITE	Stage IIA	2009	-28364	Alive	WITH TUI	1365		Locoregional Recurrence	281	0	1365	0	1365	1	281
TCGA-EE	TCGA-EE	SKCM	65	FEMALE	WHITE	Stage IA	2005	-23784	Alive	TUMOR F	2884				0	2884	0	2884	0	2884
TCGA-EE	TCGA-EE	SKCM	49	MALE	WHITE	Stage I	1999	-17950	Dead	WITH TUMOR		659	Locoregional Recurrence	537	1	659	1	659	1	537
TCGA-EE	TCGA-EE	SKCM	61	MALE	WHITE	Stage I	1997	-22564	Alive	TUMOR F	3932				0	3932	0	3932	0	3932
TCGA-EE	TCGA-EE	SKCM	66	MALE	WHITE	Stage II	1999	-24371	Dead	WITH TUMOR		877	Distant M Other, Sp mediastin	799	1	877	1	877	1	799
TCGA-EE	TCGA-EE	SKCM	54	FEMALE	WHITE	Stage III	2000	-19808	Dead	WITH TUMOR		601	Distant M Brain	498	1	601	1	601	1	498
TCGA-EE	TCGA-EE	SKCM	73	MALE	WHITE	Stage I	2000	-26872	Dead	TUMOR FREE		1871	Distant M Other, Sp right neck	896	1	1871	0	1871	1	896
TCGA-EE	TCGA-EE	SKCM	52	MALE	WHITE	Stage II	1998	-19333	Dead	WITH TUMOR		1438	Regional lymph node	1344	1	1438	1	1438	1	1344
TCGA-EE	TCGA-EE	SKCM	51	MALE	WHITE	Stage I	1995	-18841	Dead	WITH TUMOR		3141	Distant M Other, Sp retroperit	2703	1	3141	1	3141	1	2703
TCGA-EE	TCGA-EE	SKCM	39	FEMALE	WHITE	Stage I	1979	-14312	Dead	WITH TUMOR		8174	New primary melanoma	8071	1	8174	1	8174	1	8071
TCGA-EE	TCGA-EE	SKCM	23	MALE	WHITE	Stage I	1997	-8405	Dead	WITH TUMOR		3139	Distant M Lung	2585	1	3139	1	3139	1	2585
TCGA-EE	TCGA-EE	SKCM	66	MALE	WHITE	Stage III	2001	-24125	Dead	WITH TUMOR		516	Distant M Bone	440	1	516	1	516	1	440
TCGA-EE	TCGA-EE	SKCM	43	MALE	WHITE	Stage IIB	1986	-15880	Dead	WITH TUMOR		6225	New primary melanoma	2673	1	6225	1	6225	1	2673
TCGA-EE	TCGA-EE	SKCM	60	MALE	WHITE	Stage III	1999	-22050	Dead	TUMOR FREE		2927	Regional lymph node	396	1	2927	0	2927	1	396
TCGA-EE	TCGA-EE	SKCM	18	FEMALE	WHITE	Stage III	1999	-6831	Alive	TUMOR F	5487				0	5487	0	5487	0	5487
TCGA-EE	TCGA-EE	SKCM	35	MALE	WHITE	Stage II	1994	-13140	Dead	WITH TUMOR		6590	Distant M Bone	4983	1	6590	1	6590	1	4983
TCGA-EE	TCGA-EE	SKCM	63	FEMALE	WHITE	Stage I	1993	-23132	Dead	WITH TUMOR		5107	New primary melanoma	1357	1	5107	1	5107	1	1357
TCGA-EE	TCGA-EE	SKCM	58	MALE	WHITE	Stage I	2000	-21202	Dead	WITH TUMOR		1446	Distant M Brain	969	1	1446	1	1446	1	969
TCGA-EE	TCGA-EE	SKCM	34	FEMALE	WHITE	Stage I	1991	-12474	Alive	WITH TUI	7563		Locoregional Recurrence	365	0	7563	0	7563	1	365
TCGA-EE	TCGA-EE	SKCM	70	FEMALE	WHITE	Stage IIIA	2006	-25926	Dead	WITH TUMOR		1315	Regional lymph node	986	1	1315	1	1315	1	986
TCGA-EE	TCGA-EE	SKCM	61	MALE	WHITE	Stage I	2002	-22535	Alive	TUMOR F	4088			3136	0	4088	0	4088	1	3136
TCGA-EE	TCGA-EE	SKCM	72	MALE	WHITE	Stage II	1999	-26466	Alive	TUMOR F	4942		New primary melanoma	1883	0	4942	0	4942	1	1883
TCGA-EE	TCGA-EE	SKCM	45	MALE	WHITE	Stage IB	2007	-16768	Alive	TUMOR F	2166				0	2166	0	2166	0	2166
TCGA-EE	TCGA-EE	SKCM	71	MALE	WHITE	Stage IA	2005	-26237	Alive	TUMOR F	1620				0	1620	0	1620	0	1620
TCGA-EE	TCGA-EE	SKCM	47	MALE	WHITE	Stage III	2001	-17196	Alive	TUMOR F	3781				0	3781	0	3781	0	3781
TCGA-EE	TCGA-EE	SKCM	30	MALE	WHITE	Stage III	2001	-11054	Alive	TUMOR F	3733				0	3733	0	3733	0	3733
TCGA-EE	TCGA-EE	SKCM	47	MALE	WHITE	Stage III	2009	-17319	Alive	TUMOR F	1948				0	1948	0	1948	0	1948
TCGA-EE	TCGA-EE	SKCM	50	MALE	WHITE	Stage III	2009	-18491	Dead	WITH TUMOR		875	Distant M Other, Sp spinal cor	554	1	875	1	875	1	554
TCGA-EE	TCGA-EE	SKCM	52	FEMALE	WHITE	Stage IA	2009	-19216	Alive	TUMOR F	1658				0	1658	0	1658	0	1658
TCGA-EE	TCGA-EE	SKCM	48	FEMALE	WHITE	Stage IIIC	2009	-17731	Dead	WITH TUMOR		420	Locoregional Recurrence	201	1	420	1	420	1	201
TCGA-EE	TCGA-EE	SKCM	25	MALE	WHITE	Stage III	2009	-9161	Dead	WITH TUMOR		1265	Regional lymph node	229	1	1265	1	1265	1	229
TCGA-EE	TCGA-EE	SKCM	30	MALE	WHITE	Stage II	1998	-10988	Dead	WITH TUMOR		4222	Locoregional Recurrence	1981	1	4222	1	4222	1	1981
TCGA-EE	TCGA-EE	SKCM	42	MALE	WHITE	Stage IB	1986	-15446	Dead	WITH TUMOR		5237	Regional lymph node	4523	1	5237	1	5237	1	4523
TCGA-EE	TCGA-EE	SKCM	72	MALE	WHITE	Stage II	1991	-26540	Dead	WITH TUMOR		3869	Locoregional Recurrence	1429	1	3869	1	3869	1	1429
TCGA-EE	TCGA-EE	SKCM	71	MALE	WHITE	Stage III	2000	-26226	Dead	WITH TUMOR		1124	Locoregional Recurrence	467	1	1124	1	1124	1	467
TCGA-EE	TCGA-EE	SKCM	43	MALE	WHITE	Stage I	1998	-15896	Alive	TUMOR F	1949		Regional lymph node	1270	0	1949	0	1949	1	1270
TCGA-EE	TCGA-EE	SKCM	59	MALE	WHITE	Stage IIIA	2006	-21688	Dead	TUMOR FREE		1044		1035	1	1044	0	1044	1	1035
TCGA-EE	TCGA-EE	SKCM	44	MALE	WHITE	Stage IB	2006	-16430	Dead	WITH TUMOR		1618	Regional lymph node	539	1	1618	1	1618	1	539
TCGA-EE	TCGA-EE	SKCM	60	FEMALE	WHITE	Stage III	1992	-22150	Alive	WITH TUI	6138		Locoregional Recurrence	1434	0	6138	0	6138	1	1434
TCGA-EE	TCGA-EE	SKCM	70	MALE	WHITE	Stage III	2009	-25930	Dead	WITH TUMOR		832	Distant M Lung	364	1	832	1	832	1	364
TCGA-EE	TCGA-EE	SKCM	75	MALE	WHITE	Stage IIIB	2009	-27463	Alive	TUMOR F	1562		Regional lymph node	194	0	1562	0	1562	1	194
TCGA-EE	TCGA-EE	SKCM	54	MALE	WHITE	Stage IB	2003	-19852	Alive	TUMOR F	4086		Regional lymph node	537	0	4086	0	4086	1	537
TCGA-EE	TCGA-EE	SKCM	48	MALE	WHITE	Stage I	1999	-17835	Dead	WITH TUMOR		4648	New primary melanoma	725	1	4648	1	4648	1	725
TCGA-ER	TCGA-ER	SKCM	62	MALE	WHITE	Stage IIB	2006	-22702	Dead	WITH TUMOR		955	Distant M Brain	806	1	955	1	955	1	806
TCGA-ER	TCGA-ER	SKCM	77	MALE	WHITE		2005	-28175	Dead	WITH TUMOR		1354			1	1354	1	1354	1	1354
TCGA-ER	TCGA-ER	SKCM	46	MALE	WHITE		2005	-16948	Dead	WITH TUMOR		1078			1	1078	1	1078	1	1078
TCGA-ER	TCGA-ER	SKCM	64	FEMALE	WHITE	Stage IIC	2007	-23533	Alive	TUMOR F	1785				0	1785	0	1785	0	1785
TCGA-ER	TCGA-ER	SKCM	83	FEMALE	WHITE	Stage IIIB	2006	-30454	Dead	WITH TUMOR		424	Distant M Lung	110	1	424	1	424	1	110
TCGA-ER	TCGA-ER	SKCM	45	MALE	WHITE		2003	-16540	Dead	WITH TUMOR		1544		0	1	1544	1	1544	1	0
TCGA-ER	TCGA-ER	SKCM	86	FEMALE	WHITE	Stage IIIC	2007	-31661	Dead	WITH TUMOR		279		119	1	279	1	279	1	119
TCGA-ER	TCGA-ER	SKCM	79	MALE	WHITE	Stage IV	2006	-29167	Alive	TUMOR F	2365			1617	0	2365	0	2365	1	1617
TCGA-ER	TCGA-ER	SKCM	42	MALE	WHITE		2000	-15437	Dead	WITH TUMOR		2993	Regional lymph node	829	1	2993	1	2993	1	829
TCGA-ER	TCGA-ER	SKCM	77	MALE	WHITE	Stage I	1998	-28383	Dead	WITH TUMOR		1487			1	1487	1	1487	1	1487
TCGA-ER	TCGA-ER	SKCM	46	FEMALE	WHITE	Stage IB	2004	-16968	Dead	WITH TUMOR		383	Distant M Lung	357	1	383	1	383	1	357
TCGA-ER	TCGA-ER	SKCM	36	FEMALE	WHITE	Stage IB	2008	-13297	Dead	WITH TUMOR		396	Regional Other, Sp Cervical l	64	1	396	1	396	1	64
TCGA-ER	TCGA-ER	SKCM	82	MALE	WHITE		2003	-30176	Dead	TUMOR FREE		802			1	802	0	802	0	802
TCGA-ER	TCGA-ER	SKCM	48	FEMALE	WHITE		1987	-17820	Alive	TUMOR F	9188				0	9188	0	9188	0	9188
TCGA-ER	TCGA-ER	SKCM	40	MALE	WHITE		1995	-14935	Dead	TUMOR FREE		4634			1	4634	0	4634	0	4634
TCGA-ER	TCGA-ER	SKCM	54	MALE	WHITE	Stage IV	2004	-19733	Dead	WITH TUI	196	196	Distant M Lung	98	1	196	1	196	1	98
TCGA-ER	TCGA-ER	SKCM	79	FEMALE	WHITE	Stage IIC	2006	-28916	Dead	WITH TUMOR		469	Regional Other, Sp Neck- cer	211	1	469	1	469	1	211
TCGA-ER	TCGA-ER	SKCM	35	MALE	WHITE		1994	-13061	Dead	WITH TUMOR		4000	Distant M Lung	3689	1	4000	1	4000	1	3689
TCGA-ER	TCGA-ER	SKCM	36	MALE	WHITE	Stage IB	2006	-13300	Dead	WITH TUMOR		1857	Locoregic Other, Sp lt.Brachial	1152	1	1857	1	1857	1	1152
TCGA-ER	TCGA-ER	SKCM	47	MALE	WHITE		2008	-17443	Dead	TUMOR FREE		1341		0	1	1341	0	1341	1	0
TCGA-ER	TCGA-ER	SKCM	56	MALE	WHITE	Stage IIIB	2006	-20740	Dead	WITH TUMOR			Distant M Other, Sp Rt. chest v	308	1		1		1	308
TCGA-ER	TCGA-ER	SKCM	47	FEMALE	WHITE		1996	-17511	Dead	WITH TUMOR		4930	Distant M Brain	4352	1	4930	1	4930	1	4352
TCGA-ER	TCGA-ER	SKCM	37	FEMALE	WHITE		2001	-13648	Dead	WITH TUMOR		1548	Distant M Bone	1259	1	1548	1	1548	1	1259
TCGA-ER	TCGA-ER	SKCM	81	FEMALE	WHITE		2008	-29603	Alive	TUMOR F	1505				0	1505	0	1505	0	1505
TCGA-ER	TCGA-ER	SKCM	51	MALE	WHITE	Stage IV	2006	-18732	Dead	WITH TUMOR		270	Distant M Brain	118	1	270	1	270	1	118
TCGA-ER	TCGA-ER	SKCM	51	MALE	WHITE	Stage IV	2006	-18732	Dead	WITH TUMOR		270	Distant M Brain	118	1	270	1	270	1	118
TCGA-ER	TCGA-ER	SKCM	48	FEMALE	WHITE		1994	-17830	Dead	WITH TUMOR		4507	Distant M Brain	4126	1	4507	1	4507	1	4126
TCGA-ER	TCGA-ER	SKCM	58	MALE	WHITE	Stage IIIC	2005	-21209	Alive	TUMOR F	3196			1971	0	3196	0	3196	1	1971
TCGA-ER	TCGA-ER	SKCM	57	MALE	WHITE	Stage IIIB	2010	-20938	Dead	WITH TUMOR		857		461	1	857	1	857	1	461
TCGA-ER	TCGA-ER	SKCM	50	MALE	WHITE	Stage IB	2006	-18582	Dead	WITH TUMOR		1333	Distant M Bone	1157	1	1333	1	1333	1	1157
TCGA-ER	TCGA-ER	SKCM	57	FEMALE	WHITE	Stage IIIC	2004	-20867	Dead	WITH TUMOR		710			1	710	1	710	1	710
TCGA-ER	TCGA-ER	SKCM	39	MALE	WHITE	Stage 0	2007	-14259	Dead	WITH TUMOR		613								
TCGA-ER	TCGA-ER	SKCM	53	MALE	WHITE	Stage IIIB	2010	-19499	Dead	WITH TUMOR		877		230	1	877	1	877	1	230
TCGA-ER	TCGA-ER	SKCM	53	MALE	WHITE	Stage IIIB	2010	-19499	Dead	WITH TUMOR		877		230	1	877	1	877	1	230
TCGA-ER	TCGA-ER	SKCM	43	FEMALE	WHITE	Stage IIIC	2009	-15903	Dead	WITH TUMOR		1490	Distant M Other, Sp Right Delt	766	1	1490	1	1490	1	766
TCGA-ER	TCGA-ER	SKCM	49	MALE	WHITE	Stage IIIC	2009	-18220	Alive	WITH TUI	1264				0	1264	0	1264	0	1264
TCGA-ER	TCGA-ER	SKCM	25	MALE	WHITE		1987	-9366	Dead	WITH TUMOR		7514			1	7514	1	7514	1	7514
TCGA-ER	TCGA-ER	SKCM	64	FEMALE	WHITE	Stage IIIA	2005	-23650	Dead	WITH TUMOR		2829	Distant M Lung	2435	1	2829	1	2829	1	2435
TCGA-ER	TCGA-ER	SKCM	55	MALE	WHITE	Stage III	2001	-20354	Dead	WITH TUI	1429	1429		728	1	1429	1	1429	1	728
TCGA-ER	TCGA-ER	SKCM	30	MALE	WHITE	Stage IV	2009	-11032	Alive	WITH TUI	1010			945	0	1010	0	1010	1	945
TCGA-ER	TCGA-ER	SKCM	76	MALE	WHITE		2010	-28110	Dead	WITH TUMOR		426		259	1	426	1	426	1	259
TCGA-ER	TCGA-ER	SKCM	40	FEMALE	WHITE	Stage IIIC	2012	-14628	Dead	WITH TUMOR		394		206	1	394	1	394	1	206
TCGA-ER	TCGA-ER	SKCM	49	MALE	WHITE	Stage II	2001	-17984	Alive	TUMOR F	4533			3812	0	4533	0	4533	1	3812
TCGA-FR	TCGA-FR	SKCM	49	FEMALE	WHITE	Stage IIC	2008	-18122	Dead	WITH TUMOR		368	Locoregional Recurrence	283	1	368	1	368	1	283
TCGA-FR	TCGA-FR	SKCM	69	MALE	WHITE	Stage IIC	2011	-25271	Alive	WITH TUI	685		Locoregional Recurrence	678	0	685	0	685	1	678
TCGA-FR	TCGA-FR	SKCM	44	MALE	WHITE	Stage IB	2005	-16278	Alive	TUMOR F	2828				0	2828	0	2828	0	2828
TCGA-FR	TCGA-FR	SKCM		FEMALE	WHITE	I/II NOS	2007		Alive	WITH TUMOR					0		0		0	
TCGA-FR	TCGA-FR	SKCM	29	FEMALE	WHITE	Stage II	1999	-10707	Alive	TUMOR F	5299				0	5299	0	5299	0	5299
TCGA-FR	TCGA-FR	SKCM	34	FEMALE	WHITE	Stage IIIC	2013	-12679	Alive	TUMOR F	478		Regional lymph node	274	0	478	0	478	1	274
TCGA-FR	TCGA-FR	SKCM	90	MALE	WHITE	Stage IIC	2013	-32873	Dead	WITH TUMOR		305	Locoregional Recurrence	273	1	305	1	305	1	273
TCGA-FR	TCGA-FR	SKCM	54	FEMALE	WHITE	Stage IIIB	2013	-19936	Alive	TUMOR F	583		Locoregional Recurrence	64	0	583	0	583	1	64
TCGA-FR	TCGA-FR	SKCM	38	FEMALE	WHITE	Stage I	1996	-14043	Alive	WITH TUI	6716		Distant M Other, Sp spleen	6299	0	6716	0	6716	1	6299
TCGA-FR	TCGA-FR	SKCM	50	MALE	WHITE	Stage IIIC	2012	-18346	Alive	WITH TUI	847		Distant M Brain	567	0	847	0	847	1	567
TCGA-FR	TCGA-FR	SKCM	63	FEMALE	WHITE	Stage IIIC	2013	-23245	Alive	TUMOR F	571		Locoregional Recurrence	95	0	571	0	571	1	95
TCGA-FR	TCGA-FR	SKCM	65	FEMALE	WHITE	Stage IB	2011	-24032	Alive	TUMOR F	1164				0	1164	0	1164	0	1164
TCGA-FR	TCGA-FR	SKCM	78	MALE	WHITE	Stage IIB	2011	-28680	Dead	WITH TUMOR		1059			1	1059	1	1059	1	1059
TCGA-FR	TCGA-FR	SKCM	56	FEMALE	WHITE	Stage IIC	2011	-20782	Dead	WITH TUMOR		1103			1	1103	1	1103	1	1103
TCGA-FR	TCGA-FR	SKCM	41	MALE	WHITE	Stage IA	2006	-15159	Alive	TUMOR F	3176				0	3176	0	3176	0	3176
TCGA-FS	TCGA-FS	SKCM	52	MALE	WHITE	Stage IB	1987	-19078	Dead	WITH TUMOR		6598			1	6598	1	6598	1	6598
TCGA-FS	TCGA-FS	SKCM	39	FEMALE	WHITE	Stage I	2001	-14387	Dead	WITH TUMOR		1478			1	1478	1	1478	1	1478
TCGA-FS	TCGA-FS	SKCM	55	FEMALE	WHITE	Stage IIA	1987	-20396	Dead	WITH TUMOR		6953		6777	1	6953	1	6953	1	6777
TCGA-FS	TCGA-FS	SKCM	32	FEMALE	WHITE	Stage IA	1990	-11815	Dead	WITH TUMOR		6164	Distant M Lung	5503	1	6164	1	6164	1	5503
TCGA-FS	TCGA-FS	SKCM	72	FEMALE	WHITE	Stage IV	2002	-26435	Dead	WITH TUMOR		636		276	1	636	1	636	1	276
TCGA-FS	TCGA-FS	SKCM	62	MALE	WHITE	Stage I	2001	-22932	Dead	WITH TUMOR		854			1	854	1	854	1	854
TCGA-FS	TCGA-FS	SKCM	19	MALE	WHITE	Stage IIIC	2005	-7293	Dead	WITH TUMOR		237	Distant M Lung	136	1	237	1	237	1	136
TCGA-FS	TCGA-FS	SKCM	45	FEMALE	WHITE	Stage IIIB	2003	-16705	Dead	WITH TUMOR		843		663	1	843	1	843	1	663
TCGA-FS	TCGA-FS	SKCM	57	MALE	WHITE	Stage II	2002	-20963	Dead	WITH TUMOR		1486		1461	1	1486	1	1486	1	1461
TCGA-FS	TCGA-FS	SKCM	51	MALE	WHITE	I/II NOS	1978	-18843	Dead	WITH TUMOR		10870		10334	1	10870	1	10870	1	10334
TCGA-FS	TCGA-FS	SKCM	63	MALE	WHITE	Stage IIA	2001	-23344	Dead	WITH TUMOR		1628		1497	1	1628	1	1628	1	1497
TCGA-FS	TCGA-FS	SKCM	40	MALE	WHITE	Stage IIC	2008	-14618	Dead	WITH TUMOR		1413		761	1	1413	1	1413	1	761
TCGA-FS	TCGA-FS	SKCM	78	FEMALE	WHITE	Stage IIC	2007	-28491	Dead	WITH TUMOR		470			1	470	1	470	1	470
TCGA-FS	TCGA-FS	SKCM	60	FEMALE	WHITE	Stage IIIC	2007	-22252	Dead	WITH TUMOR		295		140	1	295	1	295	1	140
TCGA-FS	TCGA-FS	SKCM	71	FEMALE	WHITE	Stage IV	2003	-26232	Dead	WITH TUMOR		996		577	1	996	1	996	1	577
TCGA-FS	TCGA-FS	SKCM	75	FEMALE	WHITE	Stage I	2001	-27476	Dead	WITH TUMOR		1441		1225	1	1441	1	1441	1	1225
TCGA-FS	TCGA-FS	SKCM	68	MALE	WHITE	Stage II	2005	-24839	Dead	WITH TUMOR		728		367	1	728	1	728	1	367
TCGA-FS	TCGA-FS	SKCM	74	MALE	WHITE	Stage III	2001	-27210	Alive	TUMOR F	3080				0	3080	0	3080	0	3080
TCGA-FS	TCGA-FS	SKCM	43	MALE	WHITE	Stage IIIA	2002	-15890	Dead	WITH TUMOR		730		361	1	730	1	730	1	361
TCGA-FS	TCGA-FS	SKCM	52	MALE	WHITE	Stage II	1997	-19175	Dead	WITH TUMOR		2273			1	2273	1	2273	1	2273
TCGA-FS	TCGA-FS	SKCM	31	MALE	WHITE	I/II NOS	1997	-11599	Dead	WITH TUMOR		4062		3940	1	4062	1	4062	1	3940
TCGA-FS	TCGA-FS	SKCM	36	MALE	WHITE	Stage II	1995	-13325	Dead	WITH TUMOR		347		152	1	347	1	347	1	152
TCGA-FS	TCGA-FS	SKCM	54	MALE	WHITE	Stage I	1998	-19813	Alive	WITH TUI	4526		Regional [Not Avai [Not Appl	2861	0	4526	0	4526	1	2861
TCGA-FS	TCGA-FS	SKCM	55	MALE	WHITE	Stage III	2005	-20284	Alive	TUMOR F	1617				0	1617	0	1617	0	1617
TCGA-FS	TCGA-FS	SKCM	70	FEMALE	WHITE	Stage IIC	2002	-25856	Dead	WITH TUMOR		808		679	1	808	1	808	1	679
TCGA-FS	TCGA-FS	SKCM	65	MALE	WHITE	Stage IIIB	2005	-24087	Alive	TUMOR F	1505				0	1505	0	1505	0	1505
TCGA-FS	TCGA-FS	SKCM	71	MALE	WHITE	Stage IIB	2006	-26197	Dead	WITH TUMOR		824			1	824	1	824	1	824
TCGA-FS	TCGA-FS	SKCM	54	FEMALE	WHITE	Stage IIB	2006	-19733	Dead	WITH TUMOR		822			1	822	1	822	1	822
TCGA-FS	TCGA-FS	SKCM	67	FEMALE	WHITE	Stage IIB	2003	-24717	Alive	TUMOR F	2367		Locoregic [Not Avai [Not Appl	872	0	2367	0	2367	1	872
TCGA-FS	TCGA-FS	SKCM	46	FEMALE	WHITE	Stage IIC	2003	-16944	Dead	WITH TUMOR		1525			1	1525	1	1525	1	1525
TCGA-FS	TCGA-FS	SKCM	64	MALE	WHITE	Stage II	2001	-23663	Dead	WITH TUMOR		2028			1	2028	1	2028	1	2028
TCGA-FS	TCGA-FS	SKCM	77	FEMALE	WHITE	Stage IB	2005	-28412	Dead	WITH TUMOR		874			1	874	1	874	1	874
TCGA-FS	TCGA-FS	SKCM	52	MALE	WHITE	Stage I	1997	-19137	Dead	WITH TUMOR		5318			1	5318	1	5318	1	5318
TCGA-FS	TCGA-FS	SKCM	80	MALE	WHITE	Stage IIIC	2011	-29324	Alive	WITH TUI	1035		Locoregic [Not Avai [Not Appl	376	0	1035	0	1035	1	376
TCGA-FS	TCGA-FS	SKCM	46	FEMALE	WHITE	Stage III	2009	-16896	Dead	WITH TUMOR		813			1	813	1	813	1	813
TCGA-FS	TCGA-FS	SKCM	75	FEMALE	WHITE	Stage IIA	2007	-27482	Dead	WITH TUMOR		1655			1	1655	1	1655	1	1655
TCGA-FS	TCGA-FS	SKCM	39	MALE	WHITE	Stage IIIC	2004	-14426	Dead	WITH TUMOR		2454	Distant M Lung|Lun [Not Appl	2010	1	2454	1	2454	1	2010
TCGA-FW	TCGA-FW	SKCM	59	FEMALE	WHITE	Stage IV	2011	-21731	Alive	TUMOR F	531				0	531	0	531	0	531
TCGA-FW	TCGA-FW	SKCM	68	MALE	WHITE	Stage III	2010	-24868	Alive	TUMOR F	1124				0	1124	0	1124	0	1124
TCGA-FW	TCGA-FW	SKCM	68	MALE	WHITE	Stage III	2010	-24868	Alive	TUMOR F	1124				0	1124	0	1124	0	1124
TCGA-FW	TCGA-FW	SKCM	72	FEMALE	WHITE		2007	-26589	Dead	WITH TUMOR		1691			1	1691	1	1691	1	1691
TCGA-FW	TCGA-FW	SKCM	57	FEMALE	WHITE	Stage IIIB	2012	-20972	Alive	WITH TUI	411		Locoregional Recurrence	139	0	411	0	411	1	139
TCGA-FW	TCGA-FW	SKCM	71	MALE	WHITE	Stage IIIC	2012	-26056	Alive	TUMOR F	640				0	640	0	640	0	640
TCGA-FW	TCGA-FW	SKCM	48	FEMALE	WHITE	Stage III	2012	-17730	Alive	TUMOR F	587				0	587	0	587	0	587
TCGA-GF	TCGA-GF	SKCM	48	MALE	WHITE	Stage IIC	2011	-17545	Alive	TUMOR F	21				0	21	0	21	0	21
TCGA-GF	TCGA-GF	SKCM	58	FEMALE	WHITE	Stage IIIC	2011	-21359	Alive	WITH TUI	301		Distant M Lung	248	0	301	0	301	1	248
TCGA-GF	TCGA-GF	SKCM	74	FEMALE	WHITE	Stage IIIC	2012	-27114	Alive	TUMOR F	591				0	591	0	591	0	591
TCGA-GF	TCGA-GF	SKCM	62	FEMALE	WHITE	Stage IIB	2012	-22731	Alive		62				0	62	0	62	0	62
TCGA-GF	TCGA-GF	SKCM	78	MALE	WHITE	Stage IIIB	2012	-28774	Alive	TUMOR F	480				0	480	0	480	0	480
TCGA-GF	TCGA-GF	SKCM	39	MALE	WHITE	Stage IIC	2010	-14304	Dead	WITH TUMOR		1070			1	1070	1	1070	1	1070
TCGA-GN	TCGA-GN	SKCM	47	FEMALE	WHITE		2001	-17239	Alive	WITH TUI	4255		Locoregional Recurrence	2908	0	4255	0	4255	1	2908
TCGA-GN	TCGA-GN	SKCM	24	MALE	WHITE	Stage IV	2004	-8952	Dead	WITH TUMOR		467	Distant M Liver	39	1	467	1	467	1	39
TCGA-GN	TCGA-GN	SKCM	60	MALE	WHITE		1997	-22254	Dead	WITH TUMOR		3587	Distant M Lung	3221	1	3587	1	3587	1	3221

TCGA-GN	TCGA-GN	SKCM	53	MALE	WHITE				2004	-19712	Alive	TUMOR F	2948			Regional lymph node			99		0	2948	0	2948			1	99
TCGA-GN	TCGA-GN	SKCM	45	MALE	WHITE				2004	-16456	Dead	WITH TUMOR		308		Distant M	Brain		109		1	308	1	308			1	109
TCGA-GN	TCGA-GN	SKCM	38	MALE	WHITE	Stage IIIA			2005	-14055	Dead	WITH TUMOR		1960		Locoregional Recurrence			456		1	1960	1	1960			1	456
TCGA-GN	TCGA-GN	SKCM	83	FEMALE	WHITE	Stage IIB			2005	-30430	Dead	WITH TUMOR		1910		Locoregional Recurrence			908		1	1910	1	1910			1	908
TCGA-GN	TCGA-GN	SKCM	70	MALE	WHITE	Stage IIIC			2006	-25583	Dead	WITH TUMOR		170							1	170	1	170			1	170
TCGA-GN	TCGA-GN	SKCM	63	FEMALE	WHITE	Stage IIIA			2006	-23288	Dead	WITH TUMOR		988		Distant M	Lung		750		1	988	1	988			1	750
TCGA-GN	TCGA-GN	SKCM	77	MALE	WHITE	Stage IIIC			2006	-28336	Dead	WITH TUMOR		821		Distant M	Lung		593		1	821	1	821			1	593
TCGA-GN	TCGA-GN	SKCM	72	FEMALE	WHITE	Stage IIC			2008	-26494	Dead	WITH TUMOR		1460		Locoregional Recurrence			622		1	1460	1	1460			1	622
TCGA-GN	TCGA-GN	SKCM	30	MALE	WHITE	Stage III			2004	-11294	Alive	TUMOR F	3708			Distant M	Lung|Brai	[Not Appl	2274		0	3708	0	3708			1	2274
TCGA-GN	TCGA-GN	SKCM	73	MALE	WHITE	Stage IIA			2011	-26761	Alive	TUMOR F	1197			Distant M	Liver		217		0	1197	0	1197			1	217
TCGA-GN	TCGA-GN	SKCM	61	FEMALE	WHITE	Stage IB			2011	-22499	Alive	TUMOR F	1156								0	1156	0	1156			0	1156
TCGA-GN	TCGA-GN	SKCM	56	FEMALE	WHITE	Stage IIIC			2011	-20458	Dead	WITH TUMOR		317		Distant M	Lung		183		1	317	1	317			1	183
TCGA-GN	TCGA-GN	SKCM	51	MALE	WHITE				2009	-18953	Alive	TUMOR F	1487								0	1487	0	1487			0	1487
TCGA-GN	TCGA-GN	SKCM	51	MALE	WHITE				2009	-18953	Alive	TUMOR F	1487								0	1487	0	1487			0	1487
TCGA-GN	TCGA-GN	SKCM	71	MALE	WHITE	Stage IIIC			2012	-26118	Dead	WITH TUMOR		673		Locoregional Recurrence			151		1	673	1	673			1	151
TCGA-GN	TCGA-GN	SKCM	70	MALE	WHITE	Stage IB			2009	-25725	Dead	WITH TUMOR		1524		Locoregional Recurrence			436		1	1524	1	1524			1	436
TCGA-GN	TCGA-GN	SKCM	68	FEMALE	WHITE	Stage IIC			2012	-25000	Dead	WITH TUMOR		650							1	650	1	650			1	650
TCGA-GN	TCGA-GN	SKCM	68	MALE	WHITE	Stage IIC			2013	-24872	Alive	WITH TUI	772			New primary melanoma			186		0	772	0	772			1	186
TCGA-GN	TCGA-GN	SKCM	59	FEMALE	WHITE	Stage IA			2009	-21851	Dead	WITH TUMOR		1807		Locoregional Recurrence			1137		1	1807	1	1807			1	1137
TCGA-HR	TCGA-HR	SKCM	50	FEMALE	[Not Evaluated]					-21767	Alive	TUMOR F	7								0	7	0	7			0	7
TCGA-HR	TCGA-HR	SKCM	50	FEMALE	[Not Evaluated]					-21767	Alive	TUMOR F	7								0	7	0	7			0	7
TCGA-HR	TCGA-HR	SKCM	46	FEMALE	[Not Evalu	Stage IIIB				-16910	Dead	WITH TUMOR		2004							1	2004	1	2004			1	2004
TCGA-HR	TCGA-HR	SKCM	46	FEMALE	[Not Evalu	Stage IIIB				-16910	Dead	WITH TUMOR		2004							1	2004	1	2004			1	2004
TCGA-HR	TCGA-HR	SKCM	90	FEMALE	[Not Evaluated]					-32871	Alive	TUMOR F	1000			Locoregional Recurrence			154		0	1000	0	1000			1	154
TCGA-IH-	TCGA-IH-	SKCM	61	MALE	WHITE	Stage IIC			2008	-22577	Alive	WITH TUI	524			Distant M	Brain		314		0	524	0	524			1	314
TCGA-LH	TCGA-LH	SKCM	24	FEMALE	WHITE				1984	-9058	Alive	TUMOR F	11217								0	11217	0	11217			0	11217
TCGA-OD	TCGA-OD	SKCM	49	MALE	WHITE				1989	-17910	Dead	WITH TUMOR		9061		Distant M	Brain|Oth	[Not Appl	8987		1	9061	1	9061			1	8987
TCGA-OB	TCGA-OB	SKCM	49	MALE	WHITE	Stage IIIC			2013	-18134	Alive	WITH TUI	220			Distant M	Other, Sp	SKIN, MID	63		0	220	0	220			1	63
TCGA-OB	TCGA-OB	SKCM	73	MALE	WHITE	Stage IIIC			2012	-27009	Dead	WITH TUMOR		549							1	549	1	549			1	549
TCGA-RP	TCGA-RP	SKCM	66	FEMALE	WHITE				2005	-24450	Alive	TUMOR F	6								0	6	0	6			0	6
TCGA-RP	TCGA-RP	SKCM	77	MALE	WHITE	Stage IV			2008	-28394	Alive	TUMOR F	10								0	10	0	10			0	10
TCGA-RP	TCGA-RP	SKCM	71	MALE	WHITE	Stage IV			2009	-26044	Alive	TUMOR F	21								0	21	0	21			0	21
TCGA-RP	TCGA-RP	SKCM		MALE	WHITE	Stage IV			1999		Alive	TUMOR FREE									0		0				0	
TCGA-RP	TCGA-RP	SKCM		FEMALE	WHITE				2001		Alive										0		0				0	
TCGA-W3	TCGA-W3	SKCM	63	MALE	WHITE	Stage I			1995	-23131	Alive	WITH TUI	6940			Distant M	Other, Sp	Spleen	2406		0	6940	0	6940			1	2406
TCGA-W3	TCGA-W3	SKCM	60	FEMALE	WHITE	Stage II			1998	-22097	Dead	WITH TUMOR		1917							1	1917	1	1917			1	1917
TCGA-W3	TCGA-W3	SKCM	66	MALE	WHITE	Stage II			1997	-24258	Dead			3683							1	3683		3683			0	3683
TCGA-W3	TCGA-W3	SKCM	85	MALE	WHITE	Stage III			1997	-31351	Dead	WITH TUMOR		122							1	122	1	122			1	122
TCGA-W3	TCGA-W3	SKCM	57	MALE	WHITE	Stage III			1994	-21033	Dead	WITH TUMOR		2101		Distant M	Other, Sp	Thigh	1066		1	2101	1	2101			1	1066
TCGA-W3	TCGA-W3	SKCM	71	MALE	WHITE	Stage II			1995	-26145	Dead	WITH TUMOR		3379		Distant M	Lung		1037		1	3379	1	3379			1	1037
TCGA-W3	TCGA-W3	SKCM	63	MALE	WHITE	Stage II			1994	-23314	Dead	WITH TUMOR		1280		Distant M	Liver		1219		1	1280	1	1280			1	1219
TCGA-W3	TCGA-W3	SKCM	64	MALE	WHITE	Stage II			1996	-23711	Alive	TUMOR F	6666			Regional lymph node			335		0	6666	0	6666			1	335
TCGA-W3	TCGA-W3	SKCM	26	MALE	WHITE	Stage I			1994	-9527	Dead	WITH TUMOR		3195		Distant M	Other, Sp	Chest	2952		1	3195	1	3195			1	2952
TCGA-WE	TCGA-WE	SKCM	70	MALE	[Not Evalu	Stage IIIB			2012	-25884	Alive	TUMOR F	731								0	731	0	731			0	731
TCGA-WE	TCGA-WE	SKCM	74	MALE	WHITE	Stage IIIC			2010	-27191	Alive	TUMOR F	1492								0	1492	0	1492			0	1492
TCGA-WE	TCGA-WE	SKCM	85	MALE	WHITE	Stage IIB			2013	-31308	Alive	TUMOR F	614								0	614	0	614			0	614
TCGA-WE	TCGA-WE	SKCM	65	MALE	WHITE	Stage IV			2009	-23837	Dead	WITH TUMOR		1860		Distant M	Brain		1498		1	1860	1	1860			1	1498
TCGA-WE	TCGA-WE	SKCM	79	MALE	WHITE	Stage IIIB			2013	-29216	Alive	WITH TUI	546			Distant M	Liver		256		0	546	0	546			1	256
TCGA-WE	TCGA-WE	SKCM	70	MALE	WHITE	Stage IIIB			2006	-25869	Alive	WITH TUI	3082			Locoregional Recurrence			646		0	3082	0	3082			1	646
TCGA-WE	TCGA-WE	SKCM	57	MALE	[Not Evalu	Stage IIB			2009	-20942	Alive	WITH TUI	1794						1449		0	1794	0	1794			1	1449
TCGA-WE	TCGA-WE	SKCM	73	FEMALE	[Not Evalu	Stage IIIB			2009	-26946	Alive	TUMOR F	2145			Regional lymph node			1371		0	2145	0	2145			1	1371
TCGA-WE	TCGA-WE	SKCM	48	MALE	[Not Evalu	Stage IIA			2009	-17824	Alive	WITH TUI	1923			Distant M	Lung|Oth	[Not Appl	1757		0	1923	0	1923			1	1757
TCGA-WE	TCGA-WE	SKCM	49	MALE	WHITE	Stage IIIC			2013	-18051	Dead	WITH TUMOR		274		Distant M	Liver|Lunc	[Not Appl	126		1	274	1	274			1	126
TCGA-WE	TCGA-WE	SKCM	25	FEMALE	WHITE	Stage IV			2013	-9379	Alive	TUMOR F	359			Locoregional Recurrence			69		0	359	0	359			1	69
TCGA-WE	TCGA-WE	SKCM	45	MALE	WHITE	Stage IIIB			2012	-16736	Alive	TUMOR F	1089			Locoregional Recurrence			326		0	1089	0	1089			1	326
TCGA-WE	TCGA-WE	SKCM	62	MALE	WHITE	Stage IIA			2010	-22676	Dead	WITH TUMOR		1506		Distant M	Liver|[Not	[Not Appl	1389		1	1506	1	1506			1	1389
TCGA-WE	TCGA-WE	SKCM	37	MALE	WHITE	Stage IIIC			2013	-13850	Alive	TUMOR F	370								0	370	0	370			0	370
TCGA-WE	TCGA-WE	SKCM	47	MALE	WHITE	Stage IA			2011	-17261	Alive	TUMOR F	1229			Distant M	Other, Sp	Spleen|[N	650		0	1229	0	1229			1	650
TCGA-WE	TCGA-WE	SKCM	84	FEMALE	WHITE	Stage IIIC			2013	-30843	Alive	TUMOR F	651								0	651	0	651			0	651
TCGA-WE	TCGA-WE	SKCM	56	FEMALE	WHITE	Stage IIIC			2012	-20708	Alive	WITH TUI	760			Distant M	Brain|[No	[Not Appl	523		0	760	0	760			1	523
TCGA-XV	TCGA-XV	SKCM	90	FEMALE	WHITE	Stage II			2013	-32873	Alive	TUMOR F	11								0	11	0	11			0	11
TCGA-XV	TCGA-XV	SKCM	81	MALE	WHITE	Stage I			2013	-29836	Alive	TUMOR F	417								0	417	0	417			0	417
TCGA-XV	TCGA-XV	SKCM	51	MALE	WHITE	I/II NOS			2013	-18739	Alive	WITH TUI	392								0	392	0	392			0	392
TCGA-XV	TCGA-XV	SKCM	56	FEMALE	WHITE	Stage II			2013	-20545	Alive	TUMOR F	412								0	412	0	412			0	412
TCGA-XV	TCGA-XV	SKCM	62	FEMALE	WHITE	Stage II			2013	-22778	Dead	WITH TUMOR		393							1	393	1	393			1	393
TCGA-XV	TCGA-XV	SKCM	76	FEMALE	WHITE	Stage IIIC			2013	-27938	Alive	TUMOR F	405								0	405	0	405			0	405
TCGA-XV	TCGA-XV	SKCM	54	FEMALE	WHITE	Stage II			2013	-20058	Alive	WITH TUI	403								0	403	0	403			0	403
TCGA-XV	TCGA-XV	SKCM	54	FEMALE	WHITE	Stage II			2013	-20058	Alive	WITH TUI	403								0	403	0	403			0	403
TCGA-YD	TCGA-YD	SKCM	43	FEMALE	WHITE	Stage IA			2013	-15804	Alive	TUMOR F	210								0	210	0	210			0	210
TCGA-YD	TCGA-YD	SKCM	75	MALE	WHITE				2010	-27498	Alive	TUMOR F	1496								0	1496	0	1496			0	1496
TCGA-YD	TCGA-YD	SKCM		FEMALE	WHITE				1987		Alive	WITH TUMOR									0		0				0	
TCGA-YG	TCGA-YG	SKCM	67	MALE	[Unknowr	Stage IIC			2013	-24525	Alive	TUMOR F	306								0	306	0	306			0	306
TCGA-YG	TCGA-YG	SKCM	62	MALE	[Unknown]				2011	-22999	Dead	WITH TUMOR		1154		Regional lymph node			999		1	1154	1	1154			1	999
TCGA-YG	TCGA-YG	SKCM	63	FEMALE	[Unknowr	Stage IIB			2013	-23219	Alive	WITH TUI	439								0	439	0	439			0	439
TCGA-Z2	TCGA-Z2	SKCM	42	FEMALE	WHITE	Stage IIB			2012	-15342	Alive	TUMOR F	839								0	839	0	839			0	839
TCGA-Z2	TCGA-Z2	SKCM	58	MALE	WHITE	Stage IA			2006	-21318	Alive	WITH TUI	2950			Distant M	Lung		2727		0	2950	0	2950			1	2727
TCGA-Z2	TCGA-Z2	SKCM	57	FEMALE	WHITE	Stage IA			2013	-20937	Alive	TUMOR F	486								0	486	0	486			0	486
TCGA-3M	TCGA-3M	STAD	70	MALE	WHITE	Stage IB	Stomach,	G2	2009		Alive	TUMOR F	1765							Complete Remission/Response	0	1765	0	1765	0	1765	0	1765
TCGA-3M	TCGA-3M	STAD	51	MALE	WHITE	Stage IIIB	Stomach,	G3	2008		Dead	WITH TUMOR			Stomach	Locoregional Recurrence			395	Stable Disease	1		1				1	395
TCGA-B7	TCGA-B7	STAD	51	FEMALE	WHITE	Stage IIB	Stomach,	G3	2010	-18698	Alive	TUMOR F	812							Complete Remission/Response	0	812	0	812	0	812	0	812
TCGA-B7	TCGA-B7	STAD	62	MALE	WHITE	Stage IB	Stomach,	G3	2010	-22792	Alive	TUMOR F	356							[Unknown]	0	356	0	356			0	356
TCGA-B7	TCGA-B7	STAD	52	MALE	WHITE	Stage IIIC	Stomach,	G3	2012	-19014	Alive	TUMOR F	595							Complete Remission/Response	0	595	0	595	0	595	0	595
TCGA-B7	TCGA-B7	STAD	74	MALE	WHITE	Stage IIB	Stomach,	G1	2012	-27057	Alive	TUMOR F	335							Complete Remission/Response	0	335	0	335	0	335	0	335
TCGA-B7	TCGA-B7	STAD	51	MALE	WHITE	Stage IIIA	Stomach,	G3	2012	-18750	Alive	TUMOR F	288							Complete Remission/Response	0	288	0	288	0	288	0	288
TCGA-B7	TCGA-B7	STAD	60	MALE	WHITE	Stage IIB	Stomach,	G2	2012	-21962	Alive	TUMOR F	287							Complete Remission/Response	0	287	0	287	0	287	0	287
TCGA-BR	TCGA-BR	STAD	55	FEMALE	WHITE	Stage IIIA	Stomach,	G3	2007	-20420	Dead			201							1	201		201			0	201
TCGA-BR	TCGA-BR	STAD	55	FEMALE	WHITE	Stage IIIA	Stomach,	G3	2007	-20420	Dead			201							1	201		201			0	201
TCGA-BR	TCGA-BR	STAD	70	MALE	WHITE	Stage IIIA	Stomach,	G2	2007	-25816	Dead			212							1	212		212			0	212
TCGA-BR	TCGA-BR	STAD	70	MALE	WHITE	Stage IIIA	Stomach,	G2	2007	-25816	Dead			212							1	212		212			0	212
TCGA-BR	TCGA-BR	STAD	56	MALE	WHITE		Stomach,	G3	2008	-20635	Dead			141							1	141		141			0	141
TCGA-BR	TCGA-BR	STAD	56	MALE	WHITE		Stomach,	G3	2008	-20635	Dead			141							1	141		141			0	141
TCGA-BR	TCGA-BR	STAD	53	FEMALE	WHITE		Stomach,	G3	2008	-19585	Dead			226							1	226		226			0	226
TCGA-BR	TCGA-BR	STAD	53	FEMALE	WHITE		Stomach,	G3	2008	-19585	Dead			226							1	226		226			0	226
TCGA-BR	TCGA-BR	STAD	72	MALE	WHITE		Stomach,	G2	2008	-26562	Dead			558							1	558		558			0	558
TCGA-BR	TCGA-BR	STAD	72	MALE	WHITE		Stomach,	G2	2008	-26562	Dead			558							1	558		558			0	558
TCGA-BR	TCGA-BR	STAD	66	FEMALE	WHITE		Stomach,	G1	2008	-24354	Dead			940						Stable Disease	1	940		940			0	940
TCGA-BR	TCGA-BR	STAD	66	FEMALE	WHITE		Stomach,	G1	2008	-24354	Dead			940						Stable Disease	1	940		940			0	940
TCGA-BR	TCGA-BR	STAD	80	FEMALE	WHITE	Stage IIIA	Stomach,	G3	2008	-29397	Dead			124							1	124		124			0	124
TCGA-BR	TCGA-BR	STAD	80	FEMALE	WHITE	Stage IIIA	Stomach,	G3	2008	-29397	Dead			124							1	124		124			0	124
TCGA-BR	TCGA-BR	STAD	76	FEMALE	WHITE	Stage IIIA	Stomach,	G3	2008	-27893	Dead			124							1	124		124			0	124
TCGA-BR	TCGA-BR	STAD	76	FEMALE	WHITE	Stage IIIA	Stomach,	G3	2008	-27893	Dead			124							1	124		124			0	124
TCGA-BR	TCGA-BR	STAD	80	MALE	WHITE		Stomach,	G2	2008	-29441	Dead			284							1	284		284			0	284
TCGA-BR	TCGA-BR	STAD	80	MALE	WHITE		Stomach,	G2	2008	-29441	Dead			284							1	284		284			0	284
TCGA-BR	TCGA-BR	STAD	79	FEMALE	WHITE		Stomach,	G2	2008	-29014	Dead			294							1	294		294			0	294
TCGA-BR	TCGA-BR	STAD	79	FEMALE	WHITE		Stomach,	G2	2008	-29014	Dead			294							1	294		294			0	294
TCGA-BR	TCGA-BR	STAD	51	MALE	WHITE	Stage IB	Stomach,	G2	2008	-18699	Dead			188							1	188		188			0	188
TCGA-BR	TCGA-BR	STAD	51	MALE	WHITE	Stage IB	Stomach,	G2	2008	-18699	Dead			188							1	188		188			0	188
TCGA-BR	TCGA-BR	STAD	43	MALE	WHITE	Stage II	Stomach,	G3	2009	-16065	Dead			291							1	291		291			0	291
TCGA-BR	TCGA-BR	STAD	43	MALE	WHITE	Stage II	Stomach,	G3	2009	-16065	Dead			291							1	291		291			0	291
TCGA-BR	TCGA-BR	STAD	78	FEMALE	WHITE	Stage III	Stomach,	G2	2009	-28515	Dead			201							1	201		201			0	201
TCGA-BR	TCGA-BR	STAD	78	FEMALE	WHITE	Stage III	Stomach,	G2	2009	-28515	Dead			201							1	201		201			0	201
TCGA-BR	TCGA-BR	STAD	73	FEMALE	WHITE		Stomach,	G1	2009	-26670	Alive		0								0	0	0	0			0	0
TCGA-BR	TCGA-BR	STAD	73	FEMALE	WHITE		Stomach,	G1	2009	-26670	Alive		0								0	0	0	0			0	0
TCGA-BR	TCGA-BR	STAD	65	MALE	WHITE		Stomach,	G2	2009	-23758	Alive		0								0	0	0	0			0	0
TCGA-BR	TCGA-BR	STAD	65	MALE	WHITE		Stomach,	G2	2009	-23758	Alive		0								0	0	0	0			0	0
TCGA-BR	TCGA-BR	STAD	58	MALE	WHITE		Stomach,	G2	2008	-21295	Alive		0								0	0	0	0			0	0
TCGA-BR	TCGA-BR	STAD	58	MALE	WHITE		Stomach,	G2	2008	-21295	Alive		0								0	0	0	0			0	0
TCGA-BR	TCGA-BR	STAD	66	FEMALE	WHITE	Stage IIIA	Stomach,	G3	2008	-24260	Alive		0								0	0	0	0			0	0
TCGA-BR	TCGA-BR	STAD	66	FEMALE	WHITE	Stage IIIA	Stomach,	G3	2008	-24260	Alive		0								0	0	0	0			0	0
TCGA-BR	TCGA-BR	STAD	74	FEMALE	WHITE		Stomach,	G3	2008	-27179	Alive		0								0	0	0	0			0	0
TCGA-BR	TCGA-BR	STAD	74	FEMALE	WHITE		Stomach,	G3	2008	-27179	Alive		0								0	0	0	0			0	0
TCGA-BR	TCGA-BR	STAD	60	FEMALE	WHITE		Stomach,	G3	2008	-22196	Alive		0								0	0	0	0			0	0
TCGA-BR	TCGA-BR	STAD	60	FEMALE	WHITE		Stomach,	G3	2008	-22196	Alive		0								0	0	0	0			0	0
TCGA-BR	TCGA-BR	STAD	87	MALE	WHITE		Stomach,	G2	2008	-32024	Alive		0								0	0	0	0			0	0
TCGA-BR	TCGA-BR	STAD	87	MALE	WHITE		Stomach,	G2	2008	-32024	Alive		0								0	0	0	0			0	0
TCGA-BR	TCGA-BR	STAD	78	MALE	WHITE		Stomach,	G3	2008	-28817	Alive		0								0	0	0	0			0	0
TCGA-BR	TCGA-BR	STAD	78	MALE	WHITE		Stomach,	G3	2008	-28817	Alive		0								0	0	0	0			0	0
TCGA-BR	TCGA-BR	STAD	78	FEMALE	WHITE	Stage IV	Stomach,	G3	2008	-28770	Alive		0								0	0	0	0			0	0
TCGA-BR	TCGA-BR	STAD	78	FEMALE	WHITE	Stage IV	Stomach,	G3	2008	-28770	Alive		0								0	0	0	0			0	0
TCGA-BR	TCGA-BR	STAD	75	MALE	WHITE		Stomach,	G3	2008	-27509	Alive		0								0	0	0	0			0	0
TCGA-BR	TCGA-BR	STAD	75	MALE	WHITE		Stomach,	G3	2008	-27509	Alive		0								0	0	0	0			0	0
TCGA-BR	TCGA-BR	STAD	74	FEMALE	WHITE		Stomach,	G2	2008	-27344	Alive		0								0	0	0	0			0	0
TCGA-BR	TCGA-BR	STAD	74	FEMALE	WHITE		Stomach,	G2	2008	-27344	Alive		0								0	0	0	0			0	0
TCGA-BR	TCGA-BR	STAD	71	FEMALE	WHITE		Stomach,	G1	2008	-26290	Alive		0								0	0	0	0			0	0
TCGA-BR	TCGA-BR	STAD	71	FEMALE	WHITE		Stomach,	G1	2008	-26290	Alive		0								0	0	0	0			0	0
TCGA-BR	TCGA-BR	STAD	78	FEMALE	WHITE	Stage IIA	Stomach,	G3	2011	-28827	Alive	TUMOR F	1055							Complete Remission/Response	0	1055	0	1055	0	1055	0	1055
TCGA-BR	TCGA-BR	STAD	78	FEMALE	WHITE	Stage IIA	Stomach,	G3	2011	-28827	Alive	TUMOR F	1055							Complete Remission/Response	0	1055	0	1055	0	1055	0	1055
TCGA-BR	TCGA-BR	STAD	54	MALE	WHITE	Stage IIA	Stomach,	G3	2011	-20061	Alive	TUMOR F	485							Complete Remission/Response	0	485	0	485	0	485	0	485
TCGA-BR	TCGA-BR	STAD	54	MALE	WHITE	Stage IIA	Stomach,	G3	2011	-20061	Alive	TUMOR F	485							Complete Remission/Response	0	485	0	485	0	485	0	485
TCGA-BR	TCGA-BR	STAD	58	MALE	WHITE	Stage IIA	Stomach,	G3	2011	-21501	Alive		0								0	0	0	0			0	0
TCGA-BR	TCGA-BR	STAD	58	MALE	WHITE	Stage IIA	Stomach,	G3	2011	-21501	Alive		0								0	0	0	0			0	0
TCGA-BR	TCGA-BR	STAD	59	MALE	WHITE	Stage IIB	Stomach,	G3	2011	-21865	Dead	WITH TUMOR		422		Distant M	Liver		385	Stable Disease	1	422	1	422			1	385
TCGA-BR	TCGA-BR	STAD	74	FEMALE	WHITE	Stage IIB	Stomach,	G2	2011	-27288	Dead	TUMOR FREE		526						Complete Remission/Response	1	526	0	526	0	526	0	526
TCGA-BR	TCGA-BR	STAD	69	MALE	WHITE	Stage IIA	Stomach,	G3	2011	-25396	Alive	TUMOR F	416							Complete Remission/Response	0	416	0	416	0	416	0	416
TCGA-BR	TCGA-BR	STAD	69	MALE	WHITE	Stage IIA	Stomach,	G3	2011	-25396	Alive	TUMOR F	416							Complete Remission/Response	0	416	0	416	0	416	0	416
TCGA-BR	TCGA-BR	STAD	57	FEMALE	WHITE	Stage IIB	Stomach,	G3	2011	-21069	Dead	WITH TUMOR		588		Distant M	Liver		574	Complete Remission/Response	1	588	1	588	1	574	1	574
TCGA-BR	TCGA-BR	STAD	57	FEMALE	WHITE	Stage IIB	Stomach,	G3	2011	-21069	Dead	WITH TUMOR		588		Distant M	Liver		574	Complete Remission/Response	1	588	1	588	1	574	1	574
TCGA-BR	TCGA-BR	STAD	60	MALE	WHITE	Stage IIB	Stomach,	G3	2011	-21936	Alive	TUMOR F	1190			New Primary Tumor			148	Complete Remission/Response	0	1190	0	1190	0	1190	1	148
TCGA-BR	TCGA-BR	STAD	46	FEMALE	WHITE	Stage IIIA	Stomach,	G3	2011	-17129	Dead	TUMOR FREE		794						Complete Remission/Response	1	794	0	794	0	794	0	794
TCGA-BR	TCGA-BR	STAD	46	FEMALE	WHITE	Stage IIIA	Stomach,	G3	2011	-17129	Dead	TUMOR FREE		794						Complete Remission/Response	1	794	0	794	0	794	0	794
TCGA-BR	TCGA-BR	STAD	67	MALE	WHITE	Stage IIB	Stomach,	G2	2011	-24495	Dead	WITH TUMOR		279						Stable Disease	1	279	1	279			1	279
TCGA-BR	TCGA-BR	STAD	64	FEMALE	WHITE	Stage IIA	Stomach,	G3	2011	-23404	Alive	TUMOR F	997							Complete Remission/Response	0	997	0	997	0	997	0	997
TCGA-BR	TCGA-BR	STAD	64	FEMALE	WHITE	Stage IIA	Stomach,	G3	2011	-23404	Alive	TUMOR F	997							Complete Remission/Response	0	997	0	997	0	997	0	997
TCGA-BR	TCGA-BR	STAD	68	FEMALE	WHITE	Stage IIIB	Stomach,	G3	2011	-25073	Dead	WITH TUMOR		779						Complete Remission/Response	1	779	1	779			1	779
TCGA-BR	TCGA-BR	STAD	63	FEMALE	WHITE	Stage IIIA	Stomach,	G3	2010	-23053	Dead	WITH TUMOR		549		Locoregional Recurrence			402	Complete Remission/Response	1	549	1	549	1	402	1	402
TCGA-BR	TCGA-BR	STAD	75	MALE	WHITE	Stage IIA	Stomach,	G3	2011	-27529	Alive	WITH TUI	491			Locoregional Recurrence			426	Complete Remission/Response	0	491	0	491	1	426	1	426
TCGA-BR	TCGA-BR	STAD	57	FEMALE	WHITE	Stage IIIB	Stomach,	G3	2011	-20935	Dead	WITH TUMOR		370						Partial Remission/Response	1	370	1	370			1	370
TCGA-BR	TCGA-BR	STAD	41	MALE	WHITE	Stage IB	Stomach,	G2	2011	-15316	Alive	TUMOR F	273							Complete Remission/Response	0	273	0	273	0	273	0	273
TCGA-BR	TCGA-BR	STAD	70	MALE	WHITE	Stage IIA	Stomach,	G2	2011	-25637	Alive	TUMOR F	1223							Complete Remission/Response	0	1223	0	1223	0	1223	0	1223
TCGA-BR	TCGA-BR	STAD	65	MALE	WHITE	Stage IIIA	Stomach,	G3	2011	-23915	Alive	TUMOR F	940			Distant M	Non-regional / Dista		807	Complete Remission/Response	0	940	0	940	1	807	1	807
TCGA-BR	TCGA-BR	STAD	65	MALE	WHITE	Stage IIIA	Stomach,	G3	2011	-23915	Alive	TUMOR F	940			Distant M	Non-regional / Dista		807	Complete Remission/Response	0	940	0	940	1	807	1	807
TCGA-BR	TCGA-BR	STAD	54	FEMALE	WHITE	Stage IIA	Stomach,	G3	2011	-19751	Alive	TUMOR F	949							Complete Remission/Response	0	949	0	949	0	949	0	949
TCGA-BR	TCGA-BR	STAD	64	FEMALE	WHITE	Stage IIA	Stomach,	G3	2011	-23469	Alive	TUMOR F	1367							Complete Remission/Response	0	1367	0	1367	0	1367	0	1367
TCGA-BR	TCGA-BR	STAD	64	FEMALE	WHITE	Stage IIA	Stomach,	G3	2011	-23469	Alive	TUMOR F	1367							Complete Remission/Response	0	1367	0	1367	0	1367	0	1367
TCGA-BR	TCGA-BR	STAD	64	MALE	WHITE	Stage IV	Stomach,	G3	2011	-23611	Alive	WITH TUI	666			Distant M	Lung		348	Complete Remission/Response	0	666	0	666			1	348
TCGA-BR	TCGA-BR	STAD	69	MALE	WHITE	Stage II	Stomach,	GX	2011	-25445	Alive	TUMOR F	280							Complete Remission/Response	0	280	0	280	0	280	0	280
TCGA-BR	TCGA-BR	STAD	81	MALE	WHITE	Stage IA	Stomach,	G2	2011	-29711	Alive	TUMOR F	1100							Complete Remission/Response	0	1100	0	1100	0	1100	0	1100
TCGA-BR	TCGA-BR	STAD	81	MALE	WHITE	Stage IA	Stomach,	G2	2011	-29711	Alive	TUMOR F	1100							Complete Remission/Response	0	1100	0	1100	0	1100	0	1100
TCGA-BR	TCGA-BR	STAD	69	FEMALE	WHITE	Stage II	Stomach,	G3	2011	-25300	Alive	TUMOR F	1072							Complete Remission/Response	0	1072	0	1072	0	1072	0	1072
TCGA-BR	TCGA-BR	STAD	69	FEMALE	WHITE	Stage II	Stomach,	G3	2011	-25300	Alive	TUMOR F	1072							Complete Remission/Response	0	1072	0	1072	0	1072	0	1072
TCGA-BR	TCGA-BR	STAD	69	FEMALE	WHITE	Stage IB	Stomach,	G3	2011	-25325	Alive	TUMOR F	1090							Complete Remission/Response	0	1090	0	1090	0	1090	0	1090
TCGA-BR	TCGA-BR	STAD	65	MALE	WHITE	Stage IIA	Stomach,	G2	2011	-23861	Alive	TUMOR F	1023							Complete Remission/Response	0	1023	0	1023	0	1023	0	1023
TCGA-BR	TCGA-BR	STAD	65	MALE	WHITE	Stage IIA	Stomach,	G2	2011	-23861	Alive	TUMOR F	1023							Complete Remission/Response	0	1023	0	1023	0	1023	0	1023
TCGA-BR	TCGA-BR	STAD	62	FEMALE	WHITE	Stage IIB	Stomach,	G2	2011	-22829	Alive	TUMOR F	1210							Complete Remission/Response	0	1210	0	1210	0	1210	0	1210
TCGA-BR	TCGA-BR	STAD	62	FEMALE	WHITE	Stage IIB	Stomach,	G2	2011	-22829	Alive	TUMOR F	1210							Complete Remission/Response	0	1210	0	1210	0	1210	0	1210
TCGA-BR	TCGA-BR	STAD	63	MALE	WHITE	Stage IV	Stomach,	G2	2011	-23102	Dead	TUMOR FREE		552						Complete Remission/Response	1	552	0	552			0	552
TCGA-BR	TCGA-BR	STAD	63	MALE	WHITE	Stage IV	Stomach,	G2	2011	-23102	Dead	TUMOR FREE		552						Complete Remission/Response	1	552	0	552			0	552
TCGA-BR	TCGA-BR	STAD	62	MALE	WHITE	Stage IIB	Stomach,	G3	2011	-22701	Dead	TUMOR FREE		466						Complete Remission/Response	1	466	0	466	0	466	0	466
TCGA-BR	TCGA-BR	STAD	59	MALE	WHITE	Stage IIIB	Stomach,	G3	2011	-21839	Dead	WITH TUMOR		874		Distant M	Liver		559	Complete Remission/Response	1	874	1	874	1	559	1	559
TCGA-BR	TCGA-BR	STAD	74	MALE	WHITE	Stage IIB	Stomach,	G3	2011	-27091	Dead	TUMOR F	574							Complete Remission/Response	1		0		0		0	
TCGA-BR	TCGA-BR	STAD	74	MALE	WHITE	Stage IIB	Stomach,	G3	2011	-27091	Dead	TUMOR F	574							Complete Remission/Response	1		0		0		0	
TCGA-BR	TCGA-BR	STAD	74	MALE	WHITE	Stage IIB	Stomach,	G2	2011	-27031	Dead	TUMOR FREE		105		Locoregional Recurrence			91	Complete Remission/Response	1	105	0	105	1	91	1	91
TCGA-BR	TCGA-BR	STAD	50	FEMALE	WHITE	Stage IV	Stomach,	GX	2011	-18517	Dead	TUMOR FREE		276						Complete Remission/Response	1	276	0	276			0	276
TCGA-BR	TCGA-BR	STAD	60	MALE	WHITE	Stage IIIB	Stomach,	G3	2011	-22204	Alive	TUMOR F	899							Complete Remission/Response	0	899	0	899	0	899	0	899
TCGA-BR	TCGA-BR	STAD	59	MALE	WHITE	Stage IIIA	Stomach,	G3	2011	-21730	Alive	TUMOR F	1010							Complete Remission/Response	0	1010	0	1010	0	1010	0	1010
TCGA-BR	TCGA-BR	STAD	53	FEMALE	WHITE	Stage IIIB	Stomach,	G3	2011	-19658	Alive	TUMOR F	1133			New Prim	Other, sp	Rectum	89	Complete Remission/Response	0	1133	0	1133	0	1133	1	89
TCGA-BR	TCGA-BR	STAD	72	MALE	WHITE	Stage III	Stomach,	G3	2011	-26474	Dead	TUMOR FREE		439						Complete Remission/Response	1	439	0	439	0	439	0	439
TCGA-BR	TCGA-BR	STAD	75	FEMALE	WHITE	Stage IIB	Stomach,	G3	2011	-27561	Dead	TUMOR FREE		348						Complete Remission/Response	1	348	0	348	0	348	0	348
TCGA-BR	TCGA-BR	STAD	75	FEMALE	WHITE	Stage IIB	Stomach,	G3	2011	-27561	Dead	TUMOR FREE		348						Complete Remission/Response	1	348	0	348	0	348	0	348
TCGA-BR	TCGA-BR	STAD	58	FEMALE	WHITE	Stage IIIB	Stomach,	G2	2011	-21413	Alive	TUMOR F	21							Complete Remission/Response	0	21	0	21	0	21	0	21
TCGA-BR	TCGA-BR	STAD	70	FEMALE	WHITE	Stage IIB	Stomach,	G3	2011	-25861	Alive	TUMOR F	900							Complete Remission/Response	0	900	0	900	0	900	0	900
TCGA-BR	TCGA-BR	STAD	72	FEMALE	WHITE	Stage IIIC	Stomach,	G3	2011	-26546	Dead	TUMOR FREE		292						Complete Remission/Response	1	292	0	292	0	292	0	292
TCGA-BR	TCGA-BR	STAD	71	FEMALE	WHITE	Stage IIB	Stomach,	G2	2011	-26095	Alive	TUMOR F	981							Complete Remission/Response	0	981	0	981	0	981	0	981
TCGA-BR	TCGA-BR	STAD	72	FEMALE	WHITE	Stage IIIC	Stomach,	G3	2011	-26526	Dead	TUMOR FREE		245						Complete Remission/Response	1	245	0	245	0	245	0	245
TCGA-BR	TCGA-BR	STAD	57	FEMALE	WHITE	Stage IIIC	Stomach,	G3	2011	-20849	Dead	TUMOR FREE		17						Complete Remission/Response	1	17	0	17	0	17	0	17
TCGA-BR	TCGA-BR	STAD	49	MALE	WHITE	Stage II	Stomach,	G3	2011	-17966	Alive	TUMOR F	895							Complete Remission/Response	0	895	0	895	0	895	0	895
TCGA-BR	TCGA-BR	STAD	57	MALE	WHITE	Stage IV	Stomach,	G3	2011	-20849	Dead	TUMOR FREE		81						Complete Remission/Response	1	81	0	81			0	81
TCGA-BR	TCGA-BR	STAD	61	MALE	WHITE	Stage IIB	Stomach,	GX	2011	-22552	Dead	TUMOR FREE		607						Complete Remission/Response	1	607	0	607	0	607	0	607
TCGA-BR	TCGA-BR	STAD	60	FEMALE	WHITE	Stage IIB	Stomach,	G3	2011	-22256	Dead	WITH TUMOR		67						Progressive Disease	1	67	1	67			1	67
TCGA-BR	TCGA-BR	STAD	58	FEMALE	WHITE	Stage IIIC	Stomach,	G3	2011	-21527	Dead	WITH TUMOR		474		Distant M	Liver		390	Stable Disease	1	474	1	474			1	390
TCGA-BR	TCGA-BR	STAD	58	FEMALE	WHITE	Stage IIIC	Stomach,	GX	2012	-21224	Alive	WITH TUI	225							Progressive Disease	0	225	0	225			0	225
TCGA-BR	TCGA-BR	STAD	66	MALE	WHITE	Stage IIA	Stomach,	G2	2011	-24394	Alive	TUMOR F	188							Complete Remission/Response	0	188	0	188	0	188	0	188
TCGA-BR	TCGA-BR	STAD	71	FEMALE	WHITE	Stage IIIC	Stomach,	G3	2012	-25999	Alive	TUMOR F	946							Complete Remission/Response	0	946	0	946	0	946	0	946
TCGA-BR	TCGA-BR	STAD	65	MALE	WHITE	Stage IIIC	Stomach,	G3	2012	-23797	Dead	WITH TUMOR		398		Distant M	Non-regional / Dista		356	Complete Remission/Response	1	398	1	398	1	356	1	356
TCGA-BR	TCGA-BR	STAD	73	FEMALE	WHITE	Stage IB	Stomach,	G3	2012	-26976	Dead	TUMOR FREE		8	Other, non-malignant disease					Complete Remission/Response	1	8	0	8	0	8	0	8
TCGA-BR	TCGA-BR	STAD	42	FEMALE	WHITE	Stage IIIC	Stomach,	G3	2011	-15389	Alive	TUMOR F	675							Complete Remission/Response	0	675	0	675	0	675	0	675
TCGA-BR	TCGA-BR	STAD	70	FEMALE	WHITE	Stage IIA	Stomach,	G3	2011	-25725	Dead	WITH TUMOR		533						Stable Disease	1	533	1	533			1	533
TCGA-BR	TCGA-BR	STAD	80	FEMALE	WHITE	Stage IIA	Stomach,	G3	2011	-29394	Alive	TUMOR F	29							Complete Remission/Response	0	29	0	29	0	29	0	29
TCGA-BR	TCGA-BR	STAD	55	MALE	WHITE	Stage IIIB	Stomach,	G3	2011	-20441	Dead	WITH TUMOR		801		Distant M	Other, sp	Adrenal g	580	Complete Remission/Response	1	801	1	801	1	580	1	580
TCGA-BR	TCGA-BR	STAD	84	FEMALE	WHITE	Stage IB	Stomach,	G3	2011	-30874	Alive	TUMOR F	131							Complete Remission/Response	0	131	0	131	0	131	0	131
TCGA-BR	TCGA-BR	STAD	76	FEMALE	WHITE	Stage IIIB	Stomach,	G3	2011	-28051	Alive	WITH TUI	427			Distant M	Other, sp	Bone	419	Complete Remission/Response	0	427	0	427	1	419	1	419
TCGA-BR	TCGA-BR	STAD	64	MALE	WHITE	Stage IIIB	Stomach,	G3	2011	-23636	Alive	TUMOR F	101							Complete Remission/Response	0	101	0	101	0	101	0	101
TCGA-BR	TCGA-BR	STAD	62	MALE	WHITE	Stage IIIB	Stomach,	G3	2011	-22883	Dead	WITH TUMOR		359		Distant M	Other, sp	Brain	357	Progressive Disease	1	359	1	359			1	357
TCGA-BR	TCGA-BR	STAD	63	MALE	WHITE	Stage IIIC	Stomach,	G3	2012	-23346	Alive	TUMOR F	951							Complete Remission/Response	0	951	0	951	0	951	0	951
TCGA-BR	TCGA-BR	STAD	65	FEMALE	WHITE	Stage IIIA	Stomach,	G2	2011	-23744	Alive	TUMOR F	450							Complete Remission/Response	0	450	0	450	0	450	0	450
TCGA-BR	TCGA-BR	STAD	55	MALE	WHITE	Stage IIIC	Stomach,	G3	2012	-20157	Alive	WITH TUI	21			Locoregional Recurrence			467	Complete Remission/Response	0	21	0	21	1	467	1	467
TCGA-BR	TCGA-BR	STAD	51	MALE	WHITE	Stage IIB	Stomach,	G3	2012	-18843	Alive	TUMOR F	224							Complete Remission/Response	0	224	0	224	0	224	0	224
TCGA-BR	TCGA-BR	STAD	67	FEMALE	WHITE	Stage IIIC	Stomach,	G3	2011	-24737	Dead	TUMOR FREE		762						Complete Remission/Response	1	762	0	762	0	762	0	762
TCGA-BR	TCGA-BR	STAD	69	MALE	WHITE	Stage IIIC	Stomach,	G3	2011	-25281	Alive	TUMOR F	113							Complete Remission/Response	0	113	0	113	0	113	0	113
TCGA-BR	TCGA-BR	STAD	59	MALE	WHITE	Stage IIIA	Stomach,	G2	2012	-21731	Alive	TUMOR F	164							[Unknown]	0	164	0	164			0	164
TCGA-BR	TCGA-BR	STAD	61	MALE	WHITE	Stage IIIA	Stomach,	G2	2012	-22583	Dead	TUMOR FREE		766						Complete Remission/Response	1	766	0	766	0	766	0	766
TCGA-BR	TCGA-BR	STAD	68	FEMALE	WHITE	Stage IIIC	Stomach,	G3	2012	-24845	Alive	TUMOR F	280							Complete Remission/Response	0	280	0	280	0	280	0	280
TCGA-BR	TCGA-BR	STAD	90	FEMALE	WHITE	Stage IA	Stomach,	G2	2012	-32872	Alive	TUMOR F	615							Complete Remission/Response	0	615	0	615	0	615	0	615
TCGA-BR	TCGA-BR	STAD	64	FEMALE	WHITE	Stage IIA	Stomach,	G3	2012	-23400	Alive	TUMOR F	34							Complete Remission/Response	0	34	0	34	0	34	0	34
TCGA-BR	TCGA-BR	STAD	55	FEMALE	WHITE	Stage IIB	Stomach,	G3	2012	-20177	Alive	TUMOR F	389							Complete Remission/Response	0	389	0	389	0	389	0	389
TCGA-BR	TCGA-BR	STAD	56	MALE	WHITE	Stage IIIB	Stomach,	G3	2011	-20727	Alive	TUMOR F	825							Complete Remission/Response	0	825	0	825	0	825	0	825
TCGA-BR	TCGA-BR	STAD	62	MALE	WHITE	Stage IIIC	Stomach,	G3	2011	-22937	Dead	WITH TUMOR		284		Distant M	Lung		223	Complete Remission/Response	1	284	1	284	1	223	1	223
TCGA-BR	TCGA-BR	STAD	79	MALE	WHITE	Stage IIIC	Stomach,	G3	2012	-29054	Alive	TUMOR F	856							Complete Remission/Response	0	856	0	856	0	856	0	856
TCGA-BR	TCGA-BR	STAD	63	FEMALE	WHITE	Stage IIIB	Stomach,	G3	2012	-23133	Dead	TUMOR FREE		191						Complete Remission/Response	1	191	0	191	0	191	0	191
TCGA-BR	TCGA-BR	STAD	59	MALE	WHITE	Stage IIIB	Stomach,	G3	2012	-21619	Alive	TUMOR F	229							Complete Remission/Response	0	229	0	229	0	229	0	229
TCGA-BR	TCGA-BR	STAD	74	FEMALE	WHITE	Stage IIIB	Stomach,	G3	2012	-27223	Alive	TUMOR F	813							Complete Remission/Response	0	813	0	813	0	813	0	813
TCGA-BR	TCGA-BR	STAD	76	MALE	WHITE	Stage IB	Stomach,	G2	2012	-28037	Alive	TUMOR F	754							Complete Remission/Response	0	754	0	754	0	754	0	754
TCGA-BR	TCGA-BR	STAD	63	FEMALE	WHITE	Stage IB	Stomach,	G2	2012	-23084	Alive	TUMOR F	0							Complete Remission/Response	0	0	0	0	0	0	0	0
TCGA-BR	TCGA-BR	STAD	45	MALE	ASIAN	Stage IV	Stomach,	G2	2012	-16637	Alive	TUMOR F	972							Complete Remission/Response	0	972	0	972			0	972
TCGA-BR	TCGA-BR	STAD	52	MALE	ASIAN	Stage IIB	Stomach,	G3	2012	-19165	Alive	TUMOR F	991							Complete Remission/Response	0	991	0	991	0	991	0	991
TCGA-BR	TCGA-BR	STAD	75	MALE	ASIAN	Stage IIIB	Stomach,	G3	2012	-27549	Dead	TUMOR FREE		300						Complete Remission/Response	1	300	0	300	0	300	0	300
TCGA-BR	TCGA-BR	STAD	69	MALE	ASIAN	Stage IIIB	Stomach,	G3	2012	-25372	Dead	TUMOR FREE		635						Complete Remission/Response	1	635	0	635	0	635	0	635
TCGA-BR	TCGA-BR	STAD	67	FEMALE	WHITE	Stage IIIC	Stomach,	G3	2012	-24472	Dead	WITH TUMOR		250						Stable Disease	1	250	1	250			1	250
TCGA-BR	TCGA-BR	STAD	54	FEMALE	WHITE	Stage IIIB	Stomach,	G2	2012	-19855	Alive	TUMOR F	325							Complete Remission/Response	0	325	0	325	0	325	0	325
TCGA-BR	TCGA-BR	STAD	53	FEMALE	WHITE	Stage IIA	Stomach,	G3	2012	-19407	Alive	WITH TUI	1038			Locoregional Recurrence			682	Complete Remission/Response	0	1038	0	1038	1	682	1	682
TCGA-BR	TCGA-BR	STAD	70	MALE	WHITE	Stage IIIB	Stomach,	G3	2012	-25577	Dead	TUMOR FREE		422						Complete Remission/Response	1	422	0	422	0	422	0	422
TCGA-BR	TCGA-BR	STAD	57	MALE	ASIAN	Stage IIIA	Stomach,	G3	2012	-21107	Dead	TUMOR FREE		229						Complete Remission/Response	1	229	0	229	0	229	0	229
TCGA-BR	TCGA-BR	STAD	51	MALE	ASIAN	Stage IV	Stomach,	G3	2012	-18988	Dead	TUMOR FREE		185						Complete Remission/Response	1	185	0	185			0	185
TCGA-BR	TCGA-BR	STAD	58	MALE	WHITE	Stage IIIA	Stomach,	G3	2012	-21207	Alive	WITH TUI	1064			Distant Metastasis			962	Complete Remission/Response	0	1064	0	1064	1	962	1	962
TCGA-BR	TCGA-BR	STAD	70	FEMALE	WHITE	Stage IIIC	Stomach,	G3	2012	-25669	Alive	TUMOR F	0							Complete Remission/Response	0	0	0	0	0	0	0	0
TCGA-BR	TCGA-BR	STAD	77	MALE	WHITE	Stage IIIC	Stomach,	G3	2012	-28211	Dead	TUMOR FREE		45						Complete Remission/Response	1	45	0	45	0	45	0	45
TCGA-BR	TCGA-BR	STAD	34	FEMALE	ASIAN	Stage IIIA	Stomach,	G3	2012	-12601	Alive	TUMOR F	838							Complete Remission/Response	0	838	0	838	0	838	0	838
TCGA-BR	TCGA-BR	STAD	47	MALE	ASIAN	Stage IIIB	Stomach,	G3	2012	-17515	Dead	WITH TUMOR		869		Locoregional Recurrence			737	Complete Remission/Response	1	869	1	869	1	737	1	737
TCGA-BR	TCGA-BR	STAD	58	MALE	ASIAN	Stage IIB	Stomach,	G3	2012	-21314	Alive	TUMOR F	392							Complete Remission/Response	0	392	0	392	0	392	0	392
TCGA-BR	TCGA-BR	STAD	45	FEMALE	ASIAN	Stage IIIB	Stomach,	G3	2012	-16695	Dead	TUMOR FREE		273						Progressive Disease	1	273	0	273			0	273
TCGA-BR	TCGA-BR	STAD	63	MALE	ASIAN	Stage IIIA	Stomach,	G3	2012	-23266	Dead			22	[Unknown]					[Unknown]	1	22		22			0	22
TCGA-BR	TCGA-BR	STAD	70	MALE	ASIAN	Stage IIB	Stomach,	G3	2012	-25891	Alive	TUMOR F	431							Complete Remission/Response	0	431	0	431	0	431	0	431
TCGA-BR	TCGA-BR	STAD	39	MALE	ASIAN	Stage IIIB	Stomach,	G3	2012	-14459	Alive	TUMOR F	16								0	16	0	16			0	16
TCGA-BR	TCGA-BR	STAD	56	MALE	ASIAN	Stage IIIA	Stomach,	G3	2012	-20714	Alive	TUMOR F	862							Complete Remission/Response	0	862	0	862	0	862	0	862
TCGA-BR	TCGA-BR	STAD	69	FEMALE	WHITE	Stage IIA	Stomach,	G2	2012	-25279	Alive	TUMOR F	20							Complete Remission/Response	0	20	0	20	0	20	0	20
TCGA-BR	TCGA-BR	STAD	49	MALE	ASIAN	Stage IIB	Stomach,	G3	2012	-17932	Alive	TUMOR F	989							Complete Remission/Response	0	989	0	989	0	989	0	989
TCGA-BR	TCGA-BR	STAD	71	FEMALE	WHITE	Stage IIIB	Stomach,	G3	2012	-26048	Alive	TUMOR F	411							Complete Remission/Response	0	411	0	411	0	411	0	411
TCGA-BR	TCGA-BR	STAD	55	MALE	WHITE	Stage IIA	Stomach,	G3	2012	-20093	Alive	TUMOR F	14							Complete Remission/Response	0	14	0	14	0	14	0	14
TCGA-BR	TCGA-BR	STAD	72	FEMALE	WHITE	Stage IIB	Stomach,	G3	2012	-26334	Alive	TUMOR F	628							Complete Remission/Response	0	628	0	628	0	628	0	628
TCGA-BR	TCGA-BR	STAD	68	FEMALE	WHITE	Stage IB	Stomach,	G2	2012	-24986	Alive	TUMOR F	621							Complete Remission/Response	0	621	0	621	0	621	0	621
TCGA-BR	TCGA-BR	STAD	72	MALE	WHITE	Stage IIIB	Stomach,	G3	2012	-26412	Alive	TUMOR F	35							Complete Remission/Response	0	35	0	35	0	35	0	35
TCGA-BR	TCGA-BR	STAD	70	FEMALE	WHITE	Stage IIA	Stomach,	G3	2012	-25928	Dead	WITH TUMOR		652		Distant M	Other, sp	Pancreas	383	Complete Remission/Response	1	652	1	652	1	383	1	383
TCGA-BR	TCGA-BR	STAD	75	FEMALE	WHITE	Stage IIIB	Stomach,	G2	2012	-27668	Dead	TUMOR FREE		491						Complete Remission/Response	1	491	0	491	0	491	0	491
TCGA-BR	TCGA-BR	STAD	65	MALE	WHITE	Stage IIIA	Stomach,	G3	2012	-23999	Alive	WITH TUI	156			Locoregional Recurrence			127	Complete Remission/Response	0	156	0	156	1	127	1	127
TCGA-CD	TCGA-CD	STAD	82	MALE	ASIAN	Stage II	Stomach,	G2	2010	-30310	Alive	TUMOR F	408							Complete Remission/Response	0	408	0	408	0	408	0	408
TCGA-CD	TCGA-CD	STAD	45	MALE	ASIAN	Stage II	Stomach,	G3	2010	-16533	Alive	TUMOR F	396							Complete Remission/Response	0	396	0	396	0	396	0	396
TCGA-CD	TCGA-CD	STAD	51	FEMALE	ASIAN	Stage II	Stomach,	G1	2010		Alive	TUMOR F	400							Complete Remission/Response	0	400	0	400	0	400	0	400
TCGA-CD	TCGA-CD	STAD	69	MALE	ASIAN	Stage IIIA	Stomach,	G3	2010		Dead	WITH TUMOR		401						Progressive Disease	1	401	1	401			1	401
TCGA-CD	TCGA-CD	STAD	58	MALE	ASIAN	Stage II	Stomach,	G2	2010	-21331	Alive	TUMOR F	406							Complete Remission/Response	0	406	0	406	0	406	0	406
TCGA-CD	TCGA-CD	STAD	78	FEMALE	ASIAN	Stage II	Stomach,	G3	2010	-28608	Dead	WITH TUMOR		341		Distant M	Liver		300	Progressive Disease	1	341	1	341			1	300
TCGA-CD	TCGA-CD	STAD	90	MALE	ASIAN	[Discrepancy]	Stomach,	G2	2010	-32872	Alive	TUMOR F	368							Complete Remission/Response	0	368	0	368	0	368	0	368
TCGA-CD	TCGA-CD	STAD	60	MALE	ASIAN	Stage II	Stomach,	G3	2010	-21995	Dead	WITH TUMOR		377		Distant M	Liver		315	Complete Remission/Response	1	377	1	377	1	315	1	315
TCGA-CD	TCGA-CD	STAD	61	FEMALE	ASIAN	Stage II	Stomach,	G3	2010	-22445	Alive	WITH TUI	388			Locoregional Recurrence			69	Complete Remission/Response	0	388	0	388	1	69	1	69
TCGA-CD	TCGA-CD	STAD	82	FEMALE	ASIAN	Stage IIIA	Stomach,	G3	2010	-30004	Alive	TUMOR F	383							Complete Remission/Response	0	383	0	383	0	383	0	383
TCGA-CD	TCGA-CD	STAD	73	FEMALE	ASIAN	Stage IIIA	Stomach,	G3	2010	-26840	Alive	TUMOR F	381							Complete Remission/Response	0	381	0	381	0	381	0	381
TCGA-CD	TCGA-CD	STAD	72	FEMALE	ASIAN	Stage II	Stomach,	G2	2010	-26503	Dead	WITH TUMOR		218		Distant M	Liver		159	Complete Remission/Response	1	218	1	218	1	159	1	159
TCGA-CD	TCGA-CD	STAD	43	FEMALE	ASIAN	Stage IIIA	Stomach,	G3	2010	-15986	Alive	TUMOR F	375							Complete Remission/Response	0	375	0	375	0	375	0	375
TCGA-CD	TCGA-CD	STAD	65	MALE	ASIAN	Stage IV	Stomach,	G2	2011	-23979	Alive	TUMOR F	374							Complete Remission/Response	0	374	0	374			0	374
TCGA-CD	TCGA-CD	STAD	51	MALE	ASIAN	Stage II	Stomach,	G3	2011	-18992	Alive	TUMOR F	377							Complete Remission/Response	0	377	0	377	0	377	0	377
TCGA-CD	TCGA-CD	STAD	66	FEMALE	ASIAN	Stage IIIA	Stomach,	G3	2011	-24453	Alive	TUMOR F	383							Complete Remission/Response	0	383	0	383	0	383	0	383
TCGA-CD	TCGA-CD	STAD	52	MALE	ASIAN	Stage II	Stomach,	G2	2011	-19284	Dead	WITH TUMOR		354		Locoregional Recurrence			282	Complete Remission/Response	1	354	1	354	1	282	1	282
TCGA-CD	TCGA-CD	STAD	48	MALE	ASIAN	Stage II	Stomach,	G3	2011	-17821	Alive	WITH TUI	468			Distant M	Other, sp	liver and l	226	Complete Remission/Response	0	468	0	468	1	226	1	226
TCGA-CD	TCGA-CD	STAD	41	MALE	ASIAN	Stage II	Stomach,	G3	2011	-15227	Alive	WITH TUI	367			Distant M	Other, sp	Pancreas.	201	Progressive Disease	0	367	0	367			1	201
TCGA-CD	TCGA-CD	STAD	59	MALE	ASIAN	Stage IIIA	Stomach,	G3	2011	-21559	Alive	TUMOR F	390							Complete Remission/Response	0	390	0	390	0	390	0	390
TCGA-CD	TCGA-CD	STAD	74	MALE	ASIAN	Stage II	Stomach,	G3	2011	-27328	Alive	TUMOR F	378							Complete Remission/Response	0	378	0	378	0	378	0	378
TCGA-CD	TCGA-CD	STAD	68	MALE	ASIAN	Stage IIA	Stomach,	G2	2011	-24879	Dead	WITH TUMOR		192		Distant M	Liver	liver and l	79	Complete Remission/Response	1	192	1	192	1	79	1	79
TCGA-CD	TCGA-CD	STAD	51	MALE	ASIAN	Stage IIB	Stomach,	G3	2011	-18694	Alive	TUMOR F	374							Complete Remission/Response	0	374	0	374	0	374	0	374
TCGA-CD	TCGA-CD	STAD	58	MALE	ASIAN	Stage IIA	Stomach,	G3	2011	-21517	Dead	WITH TUMOR		344		Locoregional Recurrence			277	Complete Remission/Response	1	344	1	344	1	277	1	277
TCGA-CD	TCGA-CD	STAD	57	MALE	ASIAN	Stage IIA	Stomach,	G2	2011	-20839	Alive	TUMOR F	378							Complete Remission/Response	0	378	0	378	0	378	0	378
TCGA-CD	TCGA-CD	STAD	79	FEMALE	ASIAN	Stage IIB	Stomach,	G3	2011	-29203	Dead	WITH TUMOR		353		Locoregional Recurrence			230	Complete Remission/Response	1	353	1	353	1	230	1	230

TCGA-CD	TCGA-CD	STAD	76	MALE	ASIAN	Stage IIA	Stomach.	G3	2011	-28019	Dead	WITH TUMOR		200		Locoregional Recurrence			74	Complete Remission/Response	1	200	1	200	1	74	1	74
TCGA-CD	TCGA-CD	STAD	86	FEMALE	ASIAN	Stage IIA	Stomach.	G2	2011	-31683	Alive	TUMOR F	371							Complete Remission/Response	0	371	0	371	0	371	0	371
TCGA-CD	TCGA-CD	STAD	62	MALE	ASIAN	Stage IIIA	Stomach.	G1	2011	-22721	Dead	WITH TUMOR		358		Locoregional Recurrence			271	Complete Remission/Response	1	358	1	358	1	271	1	271
TCGA-CD	TCGA-CD	STAD	60	MALE	ASIAN	Stage IB	Stomach.	G2	2011	-21932	Alive	WITH TUI	384			Locoregional Recurrence			69	Complete Remission/Response	0	384	0	384	1	69	1	69
TCGA-CG	TCGA-CG	STAD	79	MALE		Stage IIIB	Stomach.	G2	2006	-29128	Dead			609							1	609		609			0	609
TCGA-CG	TCGA-CG	STAD	75	FEMALE		Stage IV	Stomach.	G3	2006	-27516	Alive	WITH TUI	92								0	92	0	92			0	92
TCGA-CG	TCGA-CG	STAD	84	MALE		Stage IB	Stomach.	G2	2008	-31017	Alive										0		0				0	
TCGA-CG	TCGA-CG	STAD	69	MALE		Stage II	Stomach.	G3	2008	-25295	Alive	TUMOR F	485						457	[Unknown]	0	485	0	485			1	457
TCGA-CG	TCGA-CG	STAD	90	MALE		Stage IV	Stomach.	G3	2006	-32871	Dead			31							1	31		31			0	31
TCGA-CG	TCGA-CG	STAD	57	MALE		Stage IB	Stomach.	G2	2005	-20941	Alive	TUMOR F	243							Complete Remission/Response	0	243	0	243	0	243	0	243
TCGA-CG	TCGA-CG	STAD	83	MALE		Stage II	Stomach.	G2	2006	-30467	Alive	TUMOR F	245							Complete Remission/Response	0	245	0	245	0	245	0	245
TCGA-CG	TCGA-CG	STAD	56	MALE		Stage IV	Stomach.	G3	2006	-20574	Alive		1645								0	1645	0	1645			0	1645
TCGA-CG	TCGA-CG	STAD	68	FEMALE		Stage IV	Stomach.	G3	2006	-25171	Dead			122							1	122		122			0	122
TCGA-CG	TCGA-CG	STAD	83	MALE		Stage IIIA	Stomach.	G2	2006	-30528	Dead			426							1	426		426			0	426
TCGA-CG	TCGA-CG	STAD	85	MALE		Stage IB	Stomach.	G3	2006	-31350	Alive	TUMOR F	0								0	0	0	0			0	0
TCGA-CG	TCGA-CG	STAD	68	MALE		Stage IA	Stomach.	G2	2006	-24990	Alive		912								0	912	0	912			0	912
TCGA-CG	TCGA-CG	STAD	76	MALE		Stage IIIA	Stomach.	G3	2006	-27912	Alive	TUMOR F	1431							Complete Remission/Response	0	1431	0	1431	0	1431	0	1431
TCGA-CG	TCGA-CG	STAD	81	MALE		Stage II	Stomach.	G2	2007	-29705	Alive	WITH TUI	580						336	Partial Remission/Response	0	580	0	580			1	336
TCGA-CG	TCGA-CG	STAD	72	MALE		Stage II	Stomach.	G3	2008	-26421	Alive	TUMOR F	366							Complete Remission/Response	0	366	0	366	0	366	0	366
TCGA-CG	TCGA-CG	STAD	66	FEMALE		Stage IV	Stomach.	G2	2008	-24229	Dead	WITH TUMOR		669					487	Partial Remission/Response	1	669	1	669			1	487
TCGA-CG	TCGA-CG	STAD	72	FEMALE		Stage IV	Stomach.	G3	2008	-26511	Dead	WITH TUMOR		0							1	0	1	0			1	0
TCGA-CG	TCGA-CG	STAD	69	FEMALE		Stage IV	Stomach.	G3	2008	-25233	Dead			274							1	274		274			0	274
TCGA-CG	TCGA-CG	STAD	81	FEMALE		Stage IB	Stomach.	G2	2009	-29739	Alive	TUMOR F	577							Complete Remission/Response	0	577	0	577	0	577	0	577
TCGA-CG	TCGA-CG	STAD	70	MALE		Stage IV	Stomach.	G3	2009	-25598	Dead			215							1	215		215			0	215
TCGA-CG	TCGA-CG	STAD	49	MALE		Stage IV		G3	2009	-18110	Alive	WITH TUI	365						122	Partial Remission/Response	0	365	0	365			1	122
TCGA-CG	TCGA-CG	STAD	67	FEMALE		Stage IV	Stomach.	G3	2010	-24686	Alive	WITH TUI	0								0	0	0	0			0	0
TCGA-CG	TCGA-CG	STAD	76	MALE		Stage IIB		G3	2010	-28065	Alive	TUMOR F	699							Partial Remission/Response	0	699	0	699			0	699
TCGA-CG	TCGA-CG	STAD	69	MALE		Stage IIIC	Stomach.	G3	2010	-25506	Alive	TUMOR F	0								0	0	0	0			0	0
TCGA-CG	TCGA-CG	STAD	58	FEMALE		Stage IB	Stomach.	G3	2008	-21427	Alive	WITH TUI	942						365	Progressive Disease	0	942	0	942			1	365
TCGA-CG	TCGA-CG	STAD	86	MALE		Stage IV	Stomach.	G2	2010	-31411	Alive	WITH TUI	0								0	0	0	0			0	0
TCGA-CG	TCGA-CG	STAD	58	MALE		Stage II	Stomach.	G3	2006	-21458	Dead	WITH TUMOR		212					120	Progressive Disease	1	212	1	212			1	120
TCGA-CG	TCGA-CG	STAD	78	FEMALE		Stage II	Stomach.	G2	2008	-28763	Dead	TUMOR FREE		1095						[Unknown]	1	1095	0	1095			0	1095
TCGA-CG	TCGA-CG	STAD	54	FEMALE		Stage IV	Stomach.	G1	2010	-19966	Alive	TUMOR F	31								0	31	0	31			0	31
TCGA-CG	TCGA-CG	STAD	71	MALE		Stage IB	Stomach.	G3	2003	-26176	Dead	TUMOR FREE		30							1	30	0	30			0	30
TCGA-CG	TCGA-CG	STAD	71	MALE		Stage IB	Stomach.	G3	2003	-26176	Dead	TUMOR FREE		30							1	30	0	30			0	30
TCGA-CG	TCGA-CG	STAD	58	MALE		Stage IV	Stomach.	G3	2005	-21244	Alive	TUMOR F	183							Complete Remission/Response	0	183	0	183			0	183
TCGA-CG	TCGA-CG	STAD	58	MALE		Stage IV	Stomach.	G3	2005	-21244	Alive	TUMOR F	183							Complete Remission/Response	0	183	0	183			0	183
TCGA-CG	TCGA-CG	STAD	67	FEMALE		Stage IV	Stomach.	G3	2005	-24472	Alive	WITH TUI	30								0	30	0	30			0	30
TCGA-CG	TCGA-CG	STAD	67	FEMALE		Stage IV	Stomach.	G3	2005	-24472	Alive	WITH TUI	30								0	30	0	30			0	30
TCGA-CG	TCGA-CG	STAD	83	MALE		Stage II	Stomach.	G3	2003	-30376	Alive		3196							[Unknown]	0	3196	0	3196			0	3196
TCGA-CG	TCGA-CG	STAD	59	MALE		Stage IV	Stomach.	G3	2003	-21762	Dead	WITH TUMOR		366					122	Progressive Disease	1	366	1	366			1	122
TCGA-CG	TCGA-CG	STAD	72	MALE		Stage IB	Stomach.	G3	2005	-26329	Dead	WITH TUMOR		457					153	Progressive Disease	1	457	1	457			1	153
TCGA-CG	TCGA-CG	STAD	72	MALE		Stage IB	Stomach.	G3	2005	-26329	Dead	WITH TUMOR		457					153	Progressive Disease	1	457	1	457			1	153
TCGA-CG	TCGA-CG	STAD	73	MALE		Stage IA	Stomach.	G2	2004	-26998	Dead	TUMOR FREE		881							1	881	0	881			0	881
TCGA-CG	TCGA-CG	STAD	66	MALE		Stage II	Stomach.	G3	2005	-24289	Alive	TUMOR F	2405							Complete Remission/Response	0	2405	0	2405	0	2405	0	2405
TCGA-CG	TCGA-CG	STAD	88	FEMALE		Stage IB	Stomach.	G3	2003	-32293	Alive	WITH TUI	1980								0	1980	0	1980			0	1980
TCGA-CG	TCGA-CG	STAD	88	FEMALE		Stage IB	Stomach.	G3	2003	-32293	Alive	WITH TUI	1980								0	1980	0	1980			0	1980
TCGA-CG	TCGA-CG	STAD	80	FEMALE		Stage IV	Stomach.	G2	2005	-29432	Alive	TUMOR F	122								0	122	0	122			0	122
TCGA-CG	TCGA-CG	STAD	80	FEMALE		Stage IV	Stomach.	G2	2005	-29432	Alive	TUMOR F	122								0	122	0	122			0	122
TCGA-CG	TCGA-CG	STAD	66	MALE		Stage IV	Stomach.	G2	2003	-24379	Dead	WITH TUMOR		2100					1949	[Discrepancy]	1	2100	1	2100			1	1949
TCGA-CG	TCGA-CG	STAD	83	FEMALE		Stage IIIA	Stomach.	G3	2003	-30650	Dead	WITH TUMOR		641					427	Progressive Disease	1	641	1	641			1	427
TCGA-CG	TCGA-CG	STAD	83	FEMALE		Stage IIIA	Stomach.	G3	2003	-30650	Dead	WITH TUMOR		641					427	Progressive Disease	1	641	1	641			1	427
TCGA-CG	TCGA-CG	STAD	68	MALE		Stage IIIA	Stomach.	G3	2005	-25079	Dead			243							1	243		243			0	243
TCGA-CG	TCGA-CG	STAD	68	MALE		Stage IIIA	Stomach.	G3	2005	-25079	Dead			243							1	243		243			0	243
TCGA-D7	TCGA-D7	STAD	53	FEMALE	WHITE	Stage IIIA	Stomach.	G2	2010	-19668	Dead	WITH TUMOR		782		Distant M	Other, sp	liver, peri	376	Progressive Disease	1	782	1	782			1	376
TCGA-D7	TCGA-D7	STAD	80	MALE	WHITE	Stage IIIA	Stomach.	G3	2010	-29390	Alive	TUMOR F	385								0	385	0	385			0	385
TCGA-D7	TCGA-D7	STAD	74	MALE	WHITE	Stage IIIA	Stomach.	G3	2010	-27175	Alive	TUMOR F	636							Complete Remission/Response	0	636	0	636	0	636	0	636
TCGA-D7	TCGA-D7	STAD	75	MALE	WHITE	Stage IIIA	Stomach.	G3	2010	-27594	Alive	TUMOR F	582							Complete Remission/Response	0	582	0	582	0	582	0	582
TCGA-D7	TCGA-D7	STAD	64	FEMALE	WHITE	[Discrepancy]	Stomach.	G3	2010	-23413	Alive	WITH TUI	625			Distant M	Liver	cervix	429	Progressive Disease	0	625	0	625			1	429
TCGA-D7	TCGA-D7	STAD	53	MALE	WHITE	Stage IIIA	Stomach.	G3	2010	-19467	Alive	WITH TUI	573			Distant M	Liver		46	Progressive Disease	0	573	0	573			1	46
TCGA-D7	TCGA-D7	STAD	65	MALE	WHITE	[Discrepancy]	Stomach.	G3	2010	-23776	Alive	WITH TUI	564			Locoregic	Other, specify		192	Progressive Disease	0	564	0	564			1	192
TCGA-D7	TCGA-D7	STAD	58	MALE	WHITE	Stage IB	Stomach.	G3	2010	-21465	Alive	TUMOR F	566							Complete Remission/Response	0	566	0	566	0	566	0	566
TCGA-D7	TCGA-D7	STAD	53	MALE	WHITE	Stage II	Stomach.	G3	2010	-19532	Alive	WITH TUI	543			Locoregic	Non-regional / Dista		543	Progressive Disease	0	543	0	543			1	543
TCGA-D7	TCGA-D7	STAD	58	MALE	WHITE	Stage IIIA	Stomach.	G3	2010	-21223	Dead	WITH TUMOR		406		Distant Metastasis			171	Progressive Disease	1	406	1	406			1	171
TCGA-D7	TCGA-D7	STAD	67	FEMALE	WHITE	Stage IIIA	Stomach.	G2	2010	-24477	Alive	TUMOR F	523							Complete Remission/Response	0	523	0	523	0	523	0	523
TCGA-D7	TCGA-D7	STAD	62	MALE	WHITE	Stage II	Stomach.	G2	2010	-22793	Dead	WITH TUMOR		312		Distant M	Liver		75	Progressive Disease	1	312	1	312			1	75
TCGA-D7	TCGA-D7	STAD	70	FEMALE	WHITE	Stage IB	Stomach.	G2	2010	-25815	Alive	TUMOR F	463							Complete Remission/Response	0	463	0	463	0	463	0	463
TCGA-D7	TCGA-D7	STAD	70	FEMALE	WHITE	Stage IIB	Stomach.	G2	2010	-25811	Alive	TUMOR F	486							Complete Remission/Response	0	486	0	486	0	486	0	486
TCGA-D7	TCGA-D7	STAD	63	MALE	WHITE	Stage IIIA	Stomach.	G3	2011	-23142	Alive	TUMOR F	389								0	389	0	389			0	389
TCGA-D7	TCGA-D7	STAD	53	MALE	WHITE	Stage IIIA	Stomach.	G3	2011	-19391	Dead	WITH TUMOR		376		Locoregional Recurrence			247	Progressive Disease	1	376	1	376			1	247
TCGA-D7	TCGA-D7	STAD	64	MALE	WHITE	Stage IIB	Stomach.	G2	2011	-23486	Alive	TUMOR F	344							Complete Remission/Response	0	344	0	344	0	344	0	344
TCGA-D7	TCGA-D7	STAD	77	MALE	WHITE	Stage IB	Stomach.	G2	2011	-28214	Alive	TUMOR F	375							Complete Remission/Response	0	375	0	375	0	375	0	375
TCGA-D7	TCGA-D7	STAD	44	MALE	WHITE	Stage IIIB	Stomach.	G3	2011	-16150	Alive	WITH TUI	752							Complete Remission/Response	0	752	0	752	0	752	0	752
TCGA-D7	TCGA-D7	STAD	57	MALE	WHITE	Stage IIB	Stomach.	G2	2012	-20870	Alive	WITH TUI	511							Complete Remission/Response	0	511	0	511	0	511	0	511
TCGA-D7	TCGA-D7	STAD	57	MALE	WHITE	Stage IIA	Stomach.	G3	2012	-20963	Alive	WITH TUI	593							Complete Remission/Response	0	593	0	593	0	593	0	593
TCGA-D7	TCGA-D7	STAD	72	MALE	WHITE	Stage IIIA	Stomach.	G3	2012	-26586	Alive	TUMOR F	523							Complete Remission/Response	0	523	0	523	0	523	0	523
TCGA-D7	TCGA-D7	STAD	75	MALE	WHITE	Stage IIIA	Stomach.	G3	2012	-27712	Dead	WITH TUMOR		554		Distant M	Peritoneal Surfaces		427	Complete Remission/Response	1	554	1	554	1	427	1	427
TCGA-D7	TCGA-D7	STAD	54	FEMALE	WHITE	Stage IIIB	Stomach.	G3	2012	-20014	Dead	WITH TUMOR		446		Locoregic	Lung		204	Complete Remission/Response	1	446	1	446	1	204	1	204
TCGA-D7	TCGA-D7	STAD	72	MALE	WHITE	Stage IB	Stomach.	G2	2012	-26620	Alive	WITH TUI	643			Locoregic	Lung		451	Complete Remission/Response	0	643	0	643	1	451	1	451
TCGA-D7	TCGA-D7	STAD	66	FEMALE	WHITE	Stage IIB	Stomach.	G2	2012	-24120	Alive	TUMOR F	636							Complete Remission/Response	0	636	0	636	0	636	0	636
TCGA-D7	TCGA-D7	STAD	56	MALE	WHITE	Stage IIIA	Stomach.	G2	2012	-20742	Alive	TUMOR F	434							Complete Remission/Response	0	434	0	434	0	434	0	434
TCGA-D7	TCGA-D7	STAD	73	MALE	WHITE	Stage IIIB	Stomach.	G3	2012	-26781	Alive	WITH TUI	500							Stable Disease	0	500	0	500			0	500
TCGA-D7	TCGA-D7	STAD	69	FEMALE	WHITE	Stage IIB	Stomach.	G3	2012	-25421	Dead	TUMOR FREE		180						Complete Remission/Response	1	180	0	180	0	180	0	180
TCGA-D7	TCGA-D7	STAD	63	MALE	WHITE	Stage IIB	Stomach.	G3	2012	-23249	Alive	TUMOR F	1108							Complete Remission/Response	0	1108	0	1108	0	1108	0	1108
TCGA-D7	TCGA-D7	STAD	61	MALE	WHITE	Stage IIIB	Stomach.	G3	2012	-22287	Alive	TUMOR F	419							Stable Disease	0	419	0	419			0	419
TCGA-D7	TCGA-D7	STAD	60	FEMALE	WHITE	Stage IIB	Stomach.	G3	2012	-22129	Alive	TUMOR F	449							Complete Remission/Response	0	449	0	449	0	449	0	449
TCGA-D7	TCGA-D7	STAD	76	MALE	WHITE	Stage IIIB	Stomach.	G2	2012	-27928	Alive	WITH TUI	804			Locoregional Recurrence			777	Complete Remission/Response	0	804	0	804	1	777	1	777
TCGA-D7	TCGA-D7	STAD	71	FEMALE	WHITE	Stage IIB	Stomach.	G3	2013	-26226	Alive	TUMOR F	342							Complete Remission/Response	0	342	0	342	0	342	0	342
TCGA-D7	TCGA-D7	STAD	72	FEMALE	WHITE	Stage IIIA	Stomach.	G3	2013	-26525	Alive	TUMOR F	344							Complete Remission/Response	0	344	0	344	0	344	0	344
TCGA-D7	TCGA-D7	STAD	72	FEMALE	WHITE	Stage IIIB	Stomach.	G3	2013	-26526	Dead	WITH TUMOR		348	Stomach Cancer					Progressive Disease	1	348	1	348			1	348
TCGA-D7	TCGA-D7	STAD	66	MALE	WHITE	Stage IIIA	Stomach.	G3	2013	-24425	Dead	WITH TUMOR		618						Complete Remission/Response	1	618	1	618			1	618
TCGA-D7	TCGA-D7	STAD	79	FEMALE	WHITE	Stage IB	Stomach.	G3	2013	-28923	Alive	TUMOR F	678							Complete Remission/Response	0	678	0	678	0	678	0	678
TCGA-D7	TCGA-D7	STAD	62	MALE	WHITE	Stage IB	Stomach.	G3	2012	-22801	Alive	TUMOR F	476							Complete Remission/Response	0	476	0	476	0	476	0	476
TCGA-D7	TCGA-D7	STAD	57	MALE	WHITE	Stage IIB	Stomach.	G3	2013	-20923	Dead	WITH TUMOR		255	Stomach Cancer					Progressive Disease	1	255	1	255			1	255
TCGA-D7	TCGA-D7	STAD	41	FEMALE	WHITE	Stage IV	Stomach.	G3	2013	-15190	Dead	WITH TUMOR		132	Stomach	Locoregional RecurrencelDistan			99	Progressive Disease	1	132	1	132			1	99
TCGA-D7	TCGA-D7	STAD	61	FEMALE	WHITE	Stage IIIA	Stomach.	G2	2013	-22435	Alive	TUMOR F	607							Complete Remission/Response	0	607	0	607	0	607	0	607
TCGA-D7	TCGA-D7	STAD	52	FEMALE	WHITE	Stage IIIB	Stomach.	G3	2013	-19066	Dead	WITH TUMOR		217	Stomach	Locoregional Recurrence			174	Progressive Disease	1	217	1	217			1	174
TCGA-EO	TCGA-EO	STAD	86	FEMALE	WHITE	Stage IV	Stomach.	G2	2010	-31431	Dead	WITH TUMOR		19							1	19	1	19			1	19
TCGA-EQ	TCGA-EQ	STAD	71	FEMALE	WHITE	Stage IIB	Stomach.	G3	2011	-26289	Dead			243							1	243		243			0	243
TCGA-EO	TCGA-EO	STAD	70	MALE	WHITE	Stage IIIB	[Discrepar	G2	2012	-25897	Alive	TUMOR F	494							Complete Remission/Response	0	494	0	494	0	494	0	494
TCGA-F1-	TCGA-F1-	STAD	90	MALE	BLACK OI	Stage I	Stomach.	G2	2009	-32873	Alive	TUMOR F	200							Complete Remission/Response	0	200	0	200	0	200	0	200
TCGA-F1-	TCGA-F1-	STAD	79	MALE	WHITE	Stage IB	Stomach.	G3	2011	-29083	Alive	TUMOR F	440							Complete Remission/Response	0	440	0	440	0	440	0	440
TCGA-F1-	TCGA-F1-	STAD	79	MALE	BLACK OI	Stage IA	Stomach.	G2	1996	-29209	Dead	TUMOR FREE		2197						Complete Remission/Response	1	2197	0	2197	0	2197	0	2197
TCGA-F1-	TCGA-F1-	STAD	70	MALE	WHITE	Stage IIIB	Stomach.	G3	2012	-25846	Alive	TUMOR F	647							Complete Remission/Response	0	647	0	647	0	647	0	647
TCGA-F1-	TCGA-F1-	STAD	68	MALE	ASIAN	Stage IIA	Stomach.	G2	2013	-25104	Alive	WITH TUI	346			Distant M	Liver		343	Complete Remission/Response	0	346	0	346	1	343	1	343
TCGA-FP-	TCGA-FP-	STAD	77	MALE	BLACK OI	Stage IB	Stomach.	G2	2011	-28232	Dead	TUMOR FREE		106						Complete Remission/Response	1	106	0	106	0	106	0	106
TCGA-FP-	TCGA-FP-	STAD	77	MALE	BLACK OI	Stage IB	Stomach.	G2	2011	-28232	Dead	TUMOR FREE		106						Complete Remission/Response	1	106	0	106	0	106	0	106
TCGA-FP-	TCGA-FP-	STAD	69	MALE	WHITE	Stage IIB	Stomach.	G2	2011	-25534	Alive	TUMOR F	594							Complete Remission/Response	0	594	0	594	0	594	0	594
TCGA-FP-	TCGA-FP-	STAD	69	MALE	WHITE	Stage IIB	Stomach.	G2	2011	-25534	Alive	TUMOR F	594							Complete Remission/Response	0	594	0	594	0	594	0	594
TCGA-FP-	TCGA-FP-	STAD	77	MALE	ASIAN	Stage IIIC	Stomach.	G3	2011	-28475	Dead	WITH TUMOR		428						Progressive Disease	1	428	1	428			1	428
TCGA-FP-	TCGA-FP-	STAD	77	MALE	WHITE	Stage IIIC	Stomach.	G3	2011	-28302	Alive	TUMOR F	678							Complete Remission/Response	0	678	0	678	0	678	0	678
TCGA-FP-	TCGA-FP-	STAD	79	MALE	WHITE	Stage IIA	Stomach.	G2	2011	-29129	Alive	TUMOR F	519							Complete Remission/Response	0	519	0	519	0	519	0	519
[truncated: 355,014 more chars]
